# Mouse model validity for studying the impact of tobacco smoke on the human gut microbiota assessed via in silico and experimental approaches

**DOI:** 10.18332/tid/208251

**Published:** 2025-09-17

**Authors:** Irene Victoria Bermúdez-Pérez, Juliana Meißner, Corinna Bang, Jan N. Hartmann, John F. Baines, Susanne Krauss-Etschmann, Robert Häsler

**Affiliations:** 1 Department of Dermatology and Allergy, University Hospital Schleswig-Holstein, Kiel, Germany; 2 Division of Early Life Origins of Chronic Lung Diseases, Research Center Borstel, Airway Research Center North, German Center for Lung Research, Borstel, Germany; 3 Institute of Clinical Molecular Biology, Christian-Albrechts-University of Kiel, Kiel, Germany; 4 Section of Evolutionary Medicine, Max Planck Institute for Evolutionary Biology, Plön, Germany; 5 Section of Evolutionary Medicine, Institute of Experimental Medicine, Christian-Albrechts-University of Kiel, Kiel, Germany; 6 Institute of Experimental Medicine, ChristianAlbrechts-University of Kiel, Kiel, Germany; 7 Laboratory for Experimental Microbiome Research, Research Center Borstel, Airway Research Center North, German Center for Lung Research, Borstel, Germany

**Keywords:** smoking, mouse model, microbiota, transcriptome, sequencing

## Abstract

**INTRODUCTION:**

The contribution of cigarettes to disease initiation, manifestation and progression is well-established for complex disorders, such as inflammatory bowel disease. However, studying its impact on disease pathophysiology in a controlled setting is challenging in humans, resulting in the application of various model systems, amongst them tobacco smoke-exposed mice. While frequently employed, it is unclear to what extent this model reflects human responses to tobacco smoke.

**METHODS:**

Employing a mouse study of experimental nature, we assessed established parameters for monitoring responses to tobacco smoke, paralleled by 16S rRNA gene-based profiling of the murine gut microbiome in n=32 suitable animals. This was supplemented by a case-control study design, based on n=3 publicly available transcriptome datasets, from human oral mucosa, human large airway epithelium and murine lung tissues, where we assessed which components of the response to tobacco smoke observed in mice are functionally comparable to responses seen in humans.

**RESULTS:**

We observed several physiological responses in mice that paralleled human scenarios (weight loss, serum cotinine and Cyp1a1 mRNA expression), serving as a proof of principle. We identified shared microbiome-associated processes: stress related functions were enriched in mice and humans, while other processes, such as inflammatory functions, were discordant. The mouse microbiota showed significant changes in response to tobacco smoke, which mimicked patterns seen in human datasets, such as changes for *Lachnospiraceae* and *Prevotellaceae*. In contrast, some families that show significant responses to tobacco smoke in humans, such as *Bacteroidaceae*, could not be observed in mice.

**CONCLUSIONS:**

Considering the high inter-individual variation in humans and the well-controlled conditions in mice, our results suggest that mice, despite the identified limitations, most likely represent a suitable model for studying specific processes, such as stress responses, in the context of tobacco smoke exposure and its impact on the microbiota.

## INTRODUCTION

Inflammatory bowel diseases (IBDs) have become a global health concern, affecting 6–8 million people worldwide, and therefore place a significant burden on the economy and healthcare. While genetic factors play a crucial role in IBD development, the contribution of environmental factors, such as smoking^1^, has gained increasing attention in recent years^1^. Active tobacco smoking has already been associated with a multitude of adverse health effects, including an increased risk of developing various complex disorders^2^. However, the exact mechanisms through which smoking contributes to the pathogenesis of these diseases are not yet completely understood. Such interactions between the host and environmental factors are discussed to be mediated by the human gut microbiota, which is a diverse ecosystem of microorganisms inhabiting the gastrointestinal tract^3^. It is known that dysbiosis and alterations in the gut’s microbial composition promote the development and progression of numerous chronic inflammatory diseases, including IBD^3^. Interestingly, smoking has been shown to disrupt gut microbiota composition in both human and animal studies^4^. This leads to the hypothesis that this alteration may mediate the association between smoking and IBD development^4^.

Mouse models have been instrumental in advancing our understanding of the complex interplay between smoking, the gut microbiota and IBDs^5^. Due to their genetic similarity to humans and ability to replicate human pathophysiology to a certain extent, mice are commonly used as experimental models^4,5^. Contrary to humans^3^, the effects of smoking on the gut microbiota^4^ can be investigated under controlled conditions in murine models^4-6^, which thus provide valuable insights into potential mechanisms that could be at play in human subjects^4-6^. Nevertheless, it is still essential to critically assess their suitability as models because of their inherent differences in human physiology, including variations in gut microbial composition, immune system, and metabolic pathways^6^.

This raises the key question of whether mouse systems truly reflect the complexities of the human gut microbiota’s response to tobacco smoke exposure. To assess the validity, we conducted a comparison between mice and humans to assess the impact of tobacco smoke on microbiome-associated processes by analyzing n=3 publicly available transcriptome datasets. Subsequently, we performed experimental validation on the impact of tobacco smoke’s exposure on the murine gut microbiota.

## METHODS

The presented approach employs an experimental animal study, where various physiological parameters and the gut microbiome were examined in smoke exposed mice, compared to air exposed mice. This was supported by a second analysis, where publicly available transcriptome datasets were subjected to a case-control setup comparing functional responses to smoke exposure in human and mice.

### Public datasets

Public transcriptome datasets were obtained from the Gene Expression Omnibus repository (GEO, https://www.ncbi.nlm.nih.gov/geo/). The dataset GDS3709 was included as it reflects the effect of cigarette smoke on human oral mucosa. It consists of a total of 80 samples, classified as cigarette smokers (n=40) and never smokers (n=40) which were part of a previous study^7^. This was supplemented by the dataset GDS2489 (Large airway epithelium response to cigarette smoking - HuGeneFL), containing 44 human samples, classified as control (n=18) and cigarette smoking (n=26). To serve as a corresponding mouse dataset, GDS5438 (Lung from cigarette smoke-related chronic obstructive pulmonary disease model: time course) was selected. This dataset includes a total of 12 samples, classified as control (n=6) and cigarette smoke (n=6). Unequal sample sizes of datasets were addressed by employing nonparametric methods for the transcriptome analysis (see below). Details on the datasets are summarized in Table 1.

**Table 1:** Description of the selected datasets obtained from the National Center for Biotechnology Information. The table has the identified code for the dataset, the title, the organism used, the total number of samples, the number of samples exposed to smoke and air and the transcriptome method used

*Organism*	*Samples*	*Samples exposed to smoke*	*Samples exposed to air*	*Transcriptome method*
Dataset: **GDS3709**				
Title: Cigarette smoke effect on the oral mucosa				
Details: https://www.ncbi.nlm.nih.gov/sites/GDSbrowser?acc=GDS3709				
Homo Sapiens	80	40	40	Affymetrix Human Genome U133 Plus 2.0 Array
Dataset: **GDS2489**				
Title: Large airway epithelium response to cigarette smoking - HuGeneFL				
Details: https://www.ncbi.nlm.nih.gov/sites/GDSbrowser?acc=GDS2489				
Homo Sapiens	44	26	18	Affymetrix Human Genome U133A Array
Dataset: **GDS5438**				
Title: Lung from cigarette smoke-related COPD model: time course				
Details: https://www.ncbi.nlm.nih.gov/sites/GDSbrowser?acc=GDS5438				
Mus Musculus	12	6	6	ILLUMINA RNA-Seq

### Transcriptome analysis

The GDS3709 transcriptome dataset was normalized using the z-score method. Hierarchical clustering was employed to illustrate differences in transcriptome levels between smokers and non-smokers in the oral mucosa, using Spearman correlation as a distance measure and the Unweighted Pair Group Method with Arithmetic Mean (UPGMA) for clustering. The datasets GDS2489 and GDS5438 had already undergone normalization using global scaling and quantile normalization, respectively. All data pre-processing methods included a log-transformation. They were subjected to a Gene Ontology analysis as previously published^8^, employing a Fisher test (two-tailed) with a threshold of p≤0.05, corrected for multiple testing using a Benjamini-Hochberg correction, to assess whether a biological process was enriched or depleted. To minimize dataset specific bias and batch effects, this was calculated for each dataset independently and then compared on a functional level. Concordance and discordance of functional responses were defined as signed fold changes of enrichments pointing in the same or opposite direction, respectively.

To functionally compare the transcriptome datasets, specific microbiome-associated terms were selected: bacterial-associated processes, defense processes, inflammatory processes and stress-associated processes, supplemented by vascularization-associated processes, and nitrogen compound metabolic processes. The median expression values of the genes associated with these terms were used to calculate each dataset’s signed fold change between cigarette smoking samples versus control samples. Fold change enrichment was calculated as the signed observed/expected ratio. If the observed value was less than the expected value, the formula -1/(observed/expected) was used to reflect the negative enrichment. The p-value was obtained by applying the Mann-Whitney U test. Finally, the results of the human and mouse datasets were plotted on a graph to visualize the fold change of enrichment in each selected term.

### Mouse model

All mouse experiments conducted as part of this study were approved by the local ethics committee (reference number V244 - 52645/2022(54-7/22), Research Center Borstel, Borstel, Germany). To analyze the gut microbiota changes upon cigarette exposure, male and female WT C57BL6/J mice (Charles River) were whole-body exposed to research cigarettes (University of Kentucky, USA, 3R4F) using a smoking robot (in Expose System, Scireq; flexiWare Version 6.1). In total, 120 mice were part of the experiment. As described earlier^9^, six-week-old mice were exposed to 7 cigarettes (1 puff/min) for one hour daily over three consecutive days to support adaptation to smoke-exposed. From the 4th to the 24th day, the exposure was increased to 21 cigarettes/day (3 puffs/min).

Stool pellets were collected from all the individual animals right before the smoke exposure, starting before the first exposure and then every third day, for 16S rRNA gene sequencing analysis of microbial composition. Body weight was measured every third day. To collect stool samples, mice were separated into containers, and disinfected forceps were used to collect pellets individually. From each cage, stool samples from one mouse were chosen and subjected to DNA extraction and subsequent 16S rRNA gene sequencing as previously described^10^, no co-housed animals were subjected to the analysis. A total of 282 samples were sequenced. As a stable metabolite of nicotine, cotinine concentration in smoke-exposed and air-exposed mice, was quantified (Cotinine Direct ELISA Kit; Abcam) on the last day of exposure. To further confirm the exposure, the post-caval lobe of the lung was used to quantify Cyp1a1 expression by qPCR (Table 2); 44 animals were suitable for the measurement of Cyp1a1, as the remainder had been exposed to FITC Dextran, which interferes with the Cybr Green dye used in qPCR.

**Table 2:** Primers for Cyp1a1 expression

Cyp1a1 fwd 913	CGTTACCTGCCTAACTCTTC
Cyp1a1 rev 1056	ATGCTCAATGAGGCTGTCTG

### Downstream analysis for the microbiota

The microbiota analysis was conducted as previously published^11^ and as deposited at the corresponding GitHub repository: https://github.com/mruehlemann/ikmb_amplicon_processing. The analysis was performed using R (version 4.3.1), with the packages *phyloseq* (version 1.44), *microbiome* (version 1.22) and *vegan* (version 2.6-8). Alpha diversity was assessed using the Shannon index, while beta diversity was evaluated using Bray-Curtis dissimilarity. A constrained analysis of principal coordinates was conducted to evaluate beta diversity measures, and the statistical significance of the models was evaluated with 1000 permutations (PERMANOVA, two-tailed). Differential abundance of the amplicon sequence variants (ASV) was determined using compositional transformation, identifying the common families from the differential abundances. Overlap between families was assessed by identifying the top 10 families for each condition (air-exposed and smoke-exposed) in male and female samples. Seven families were common across genders in both conditions.

Functional characteristics associated with these families under air-exposed and smoke-exposed conditions were analyzed using PICRUSt2 (version 2.3.5) in Python (version 3.6.8). These results were subsequently analyzed for pathway differential abundance analysis (DAA) using the DESeq2 method, which includes a log2 transformation. The annotations were based on the Kyoto Encyclopedia of Genes and Genomes (KEGG) pathway descriptions in R, utilizing the *ggpicrust2* package (version 1.7.3), which included the resulting p-values being adjusted via a Benjamini-Hochberg correction.

## RESULTS

### Physiological parameters in mice exposed to cigarette smoke

**Figure 1:**
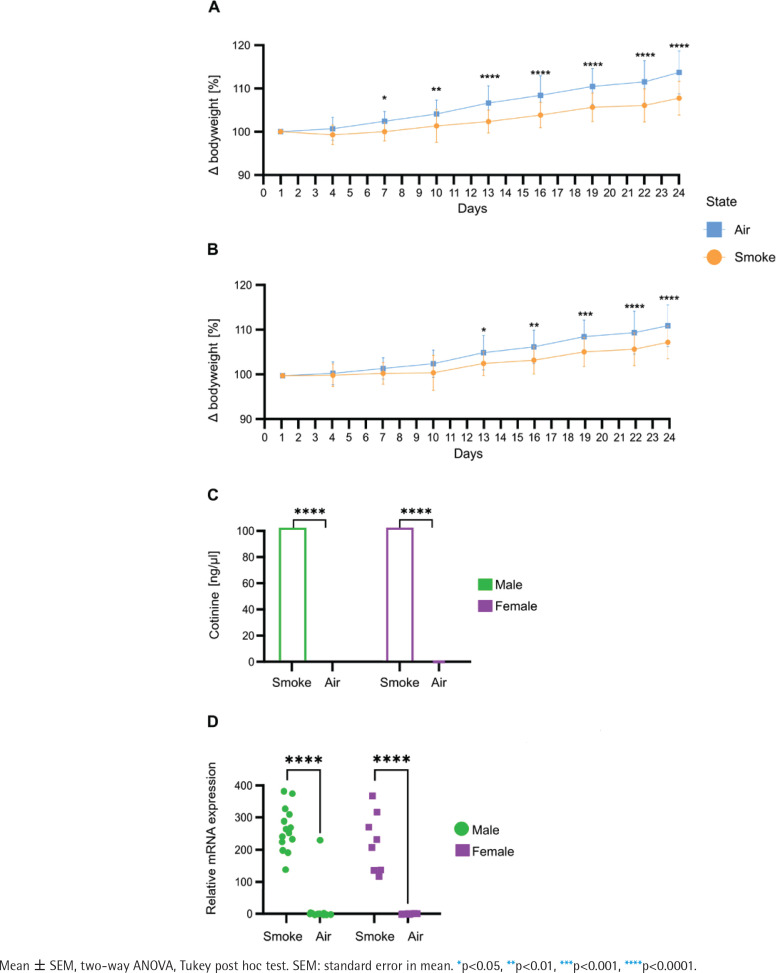
Comparative analysis of physiological and molecular responses to smoking in male and female: A) Bodyweight development in male mice (n=16); B) Bodyweight development in female mice (n=16), data are normalized to the weight before the first exposure; C) Cotinine concentration in serum in male and female (n=44); D) mRNA expression levels of Cyp1a1 quantified via qPCR in post caval lobe in male and female (n=44). Air-exposed group (blue) and smoke-exposed group (orange). Male (green) and female (purple)

Bodyweight measurement every third day over 24 days revealed significant differences between the groups as body weight gain was significantly lower in male (Figure 1A) and female (Figure 1B) mice exposed to smoking compared to their air-exposed mice. Serum cotinine concentration (Figure 1C) was significantly higher in smoke conditions in both male and female animals. Cyp1a1 mRNA expression levels (Figure 1D) in the post caval lobe (lung) were significantly increased in smoke-exposed males and females (males: 76.8-fold increase, p=1.5x10^-12^; females 94.8-fold increase, p=3.4x10^-8^).

### Tobacco smoke-induced transcriptomic changes as a proxy for microbiota-associated processes

**Figure 2:**
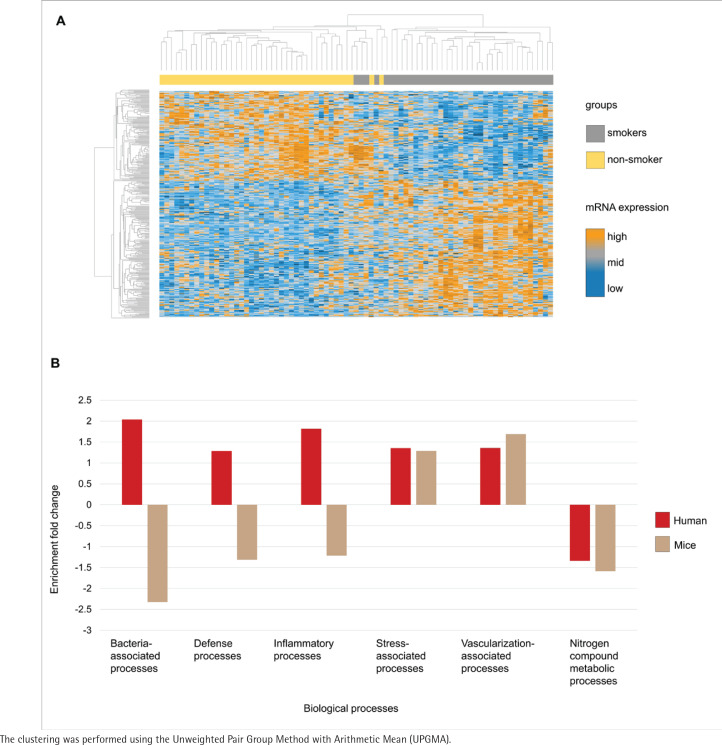
Comparative transcriptome analysis and gene ontology in smokers and non-smokers: A) Heatmap of the oral mucosa human transcriptome, displayed as z-score normalized expression values; B) Gene ontology analysis of the common biological processes of humans and mice from the public transcriptome datasets. Transcripts are organized in rows (upregulated genes in orange, not regulated in grey, and downregulated in dark blue) and groups in columns (smokers in grey and non-smokers in yellow). Humans (red) and mice (brown)

The transcriptome analysis of the oral mucosa in humans revealed patterns differentiating between smokers and non-smokers (Figure 2A). Gene Ontology analysis comparing the mice and human transcriptomes showed distinct patterns of enrichment in different biological processes (Figure 2B). Both groups exhibited positive enrichment fold change in stress-associated and vascularization-associated processes. In contrast, nitrogen compound metabolic processes such as converting amino acids to energy had a negative enrichment fold change in the two species transcriptome datasets. Additionally, processes directly linked to host-microbiota interactions, including bacteria-associated processes defense processes and inflammatory processes, demonstrated a higher enrichment fold change in humans while in mice these processes had a decreased enrichment fold change.

### Murine gut microbiota after experimental smoke exposure

**Figure 3:**
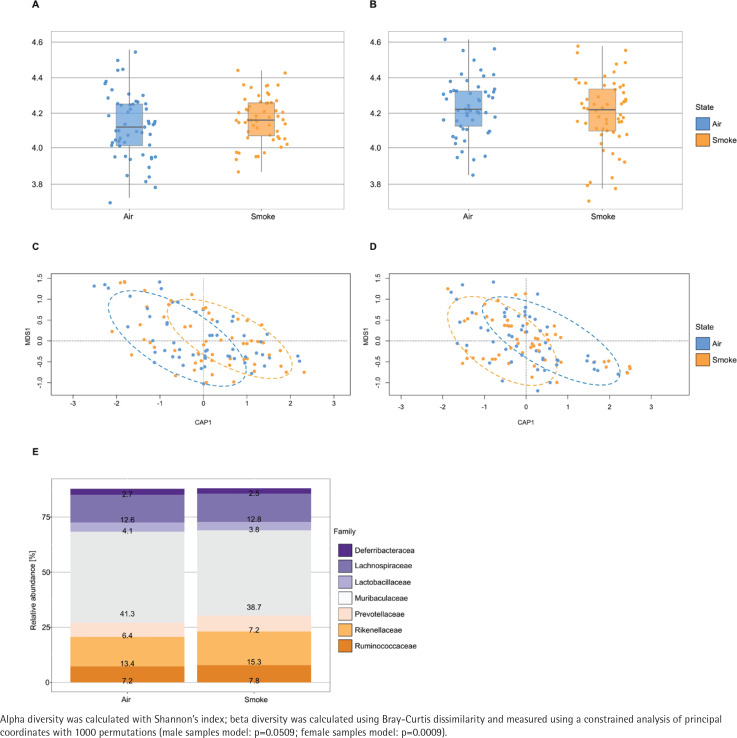
Alpha diversity, beta diversity and relative abundance: A) Alpha diversity in male samples (n=110); B) Alpha diversity in female samples (n=109); C) Beta diversity in male samples (n=110); D) Beta diversity in female samples (n=109); E) relative abundance of common families between air and smoke-exposed animals (n=219). Air-exposed group (blue) and smoke-exposed group (orange)

**Figure 4:**
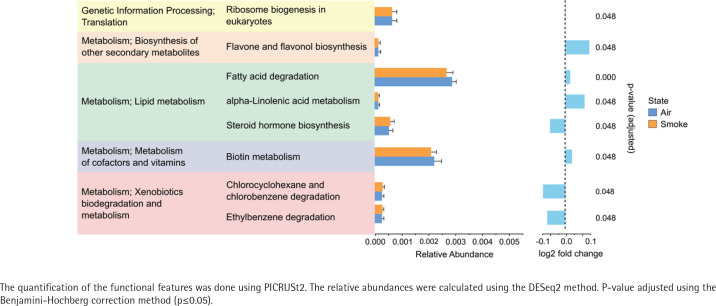
Functional features of the most abundant bacterial families in female and male subjects. Air-exposed group (blue) and smoke-exposed group (orange). Error bars represent the corresponding standard deviation. Relative log2 fold change values are provided adjacent to the relative abundance, indicating the direction and magnitude of changes in each condition

The alpha diversity (Shannon diversity index) revealed that the median alpha diversity in air-exposed samples shows a non-significant difference for either male (Figure 3A) or female samples (Figure 3B) compared to smoke-exposed samples. The beta diversity analysis using the Bray-Curtis index showed distinct clusters for air-exposed and smoke-exposed groups, more pronounced in males (n=110, Figure 3C) and significant in females (n=109, Figure 3D) from day 4 to day 24. The compositional analysis of the common families between male and female samples (Figure 3E) revealed distinct patterns in the relative abundance between air-exposed and smoke-exposed. The families enhanced by smoke exposure were *Lachnospiraceae*, *Prevotellaceae*, *Rikenellaceae*, and *Ruminococcaceae*. In contrast, the families *Deferribacteraceae*, *Lactobacillaceae,* and *Muribaculaceae* decreased in smoke conditions. The analysis of functional characteristics related to common families revealed significant differences between air- and smoke-exposure conditions. Eight pathways were identified with an adjusted p<0.05 (Figure 4). The relative abundance of eukaryotic ribosome biogenesis, steroid hormone biosynthesis, chlorocyclohexane and chlorobenzene degradation, and ethylbenzene degradation was higher under smoke conditions. In contrast, flavone and flavonol biosynthesis, fatty acid degradation, alpha-linolenic acid metabolism, and biotin metabolism showed lower relative abundance in smoke-exposed animals.

## DISCUSSION

While mouse models are being regularly employed to study the impact of cigarette smoking on human health, little is known about the validity of this experimental setting. Murine systems offer unique opportunities to study the intestinal microbiome under controlled conditions, which is of specific relevance to complex human diseases like IBD, where the intestinal microbiome plays a key role^12^. Linking this to the adverse health effects of smoking, these animal models represent a powerful tool to show how human smoking habits, microbiome and intestinal diseases like IBD are interconnected, assuming that the model is able to mimic pathophysiological mechanisms of human diseases^5^. To assess where mice and humans are concordant or discordant in their microbiome’s response to smoking, we: 1) compared microbiome-associated responses on the transcriptome level; and 2) supplemented these findings with our microbiome data from an experimental smoking model in mice. As a primary result, we observed that mice exhibit specific responses to smoke exposure that mimic human smoking. At the same time, we found that several biological processes in response to cigarette smoke exposure are discordant between mice and humans.

Comparing publicly available transcriptome datasets from smoke-exposed mice and humans, while focusing on biological functions relevant to the microbiome, we detected almost identical patterns in stress-associated processes, nitrogen compound metabolic processes and vascularization-associated processes. This is of high pathophysiological relevance since stress-associated processes include mechanisms such as oxidative stress, which occur when the antioxidant system is overwhelmed by reactive oxygen species^2^ and is often observed in close interconnection with the microbiome and its modulation. Similarly, tobacco smoke increases oxidative stress due to reactive nitrogen species, damaging macromolecular components like proteins and lipids, promoting oxidative damage and enhancing inflammation, increasing the vulnerability to bacterial pathogens and potentially leading to disease or cancer initiation^13^. Nitrogen compound metabolic processes are closely linked to the gut microbiota, as microbial communities play a key role in the transformation and utilization of nitrogen compounds, including proteins and amino acids. These microbial functions can have beneficial effects on the host, such as the generation of short-chain fatty acids, but also detrimental effects, such as the production of ammonia and hydrogen sulfide, which, at high concentrations, are harmful^14^. In addition, exposure to tobacco and its various chemical compounds can induce gut microbiota dysbiosis, altering metabolic pathways, including those related to the metabolism of nitrogenous compounds, which may lead to the accumulation of harmful nitrogenous metabolites such as ammonia and nitrosamines^4^. Vascularization, the third process observed to increase in both species, has been demonstrated to rise due to smoking in organs such as the diaphragm muscle^15^, while also being a key element of inflammatory processes which are linked to the microbiome. Taken together, these observations cover microbiome-associated processes that are highly relevant for disease susceptibility, manifestation and progression.

At the same time, we observed differences between mice and humans in bacteria-associated processes, defense processes and inflammatory processes. These processes behaved oppositely in each species, indicating that mice may not be suitable models for all biological processes, presenting a limitation when studying smoking effects in humans and mice. Further subdivisions of these processes might reveal a different picture; however, such approaches might require a different study setup.

To further validate mice as a suitable model organism for smoke effects, bodyweight gain, cotinine levels in serum and Cyp1a1 mRNA expression levels are used as indicators of successful smoke exposure. Bodyweight gain is often reduced in human smokers caused by systemic stress, inflammation and oxidative stress in tissues imposed by toxic components in cigarette smoke. This may lead to appetite regulation or disrupt normal metabolic processes^16^. We observed decreased bodyweight gain in mice which covers the physiological aspects of smoke exposure in them as a model organism, which has been described in humans consuming tobacco smoke^17,18^. To address more of the complex response of a body to cigarette smoke, cotinine levels in blood serum add a pharmacokinetic dimension in verifying physiologically relevant smoke exposure in our model. Cotinine is a primary metabolite of nicotine but has a longer half-life time making it a more stable biomarker for recent smoke exposure. Nicotine inhalation leads to the presence of cotinine in the blood serum of smoke-exposed mice and confirms that nicotine was metabolized in the body. In humans, cotinine is widely used as a biomarker for smoke exposure and can be detected in blood, urine^19^ or breastmilk.

Lastly, to validate mice as a suitable model for experimental research with cigarette smoke exposure, biochemical aspects of the exposure are considered by quantifying Cyp1a1 mRNA expression levels. The Cyp1a1 (cytochrome P450 1A1) enzyme in mice shares 80% genetic homology to the human CYP1A1. Both are activated upon exposure to bioactivated polycyclic aromatic hydrocarbons (PAHs) abundant in cigarette smoke. As we quantified increased expression levels of the body’s detoxification response in this study, we provide an indirect yet reliable indicator that mice have been exposed to cigarette smoke and thus reflect the human reaction to those substances.

Disturbances in the gut microbiota are strongly associated with the progression of IBD, with significant differences being found between patients with Crohn’s disease and healthy patients^3^. Mice are commonly used as model organisms in the study of changes in gut microbiota due to their similarities to humans and the possibility of controlling environmental factors in studies^20^. The gut microbiota is affected by various environmental factors, including tobacco smoke exposure^4^. Our alpha diversity analysis showed a trend where females exhibited higher microbiota diversity than males under air exposure, consistent with previous findings in mice^21^. Tobacco exposure has been reported to have a greater impact on males compared to females. In concordance with that, our observations indicate that alpha diversity in females under smoke exposure displays minimal differences compared to air exposure, while males show a tendency towards higher alpha diversity under smoke exposure. The trends observed on alpha diversity are further supported by our beta diversity analysis, which revealed distinct clustering patterns between air- and tobacco smoke-exposed groups, with a more pronounced separation in males and a statistically significant effect in females. Larsen and Claassen^22^ showed that higher diversity in systems such as gut microbiota leads to more efficient and redundant systems.

Taken together, our findings suggest that smoke exposure affects diversity in both males and females, although with differences in the degree and nature of these effects. These findings align with previous research indicating that the composition of gut microbiota in mice varies depending on strain, gender, and other factors, such as diet^6^. Moreover, studies in humans have shown that gut microbiota composition differs between males and females due to hormonal, genetic, and environmental influences^23^. This suggests that the gender differences observed in mice could reflect similar differences in human scenarios, highlighting the relevance of using mice as a model system to study the effects of environmental factors on the gut microbiota, while also being able to monitor a proportion of gender specific effects. In this context it is important to note that the study presented here was not designed to capture gender-specific effects not gender differences, therefore these observations should be considered as trends that require further validation.

The composition of the murine and human intestinal microbiota exhibits large overlaps, with the two phyla Firmicutes and Bacteroidota being the most common taxa found in both^24^. While our study focuses on the impact of tobacco smoke on mouse microbiota, similar observations have been noted in human microbiota studies. For instance, Lee et al.^25^ described an increase in Bacteroidota and a decrease in Firmicutes and Proteobacteria in smokers’ microbiota. Similarly, in our study, we found that most of the variations identified in families from male and female samples belong to these phyla. Specifically, families such as *Lachnospiraceae*, *Prevotellaceae*, *Rikenellaceae*, and *Ruminococcaceae* increased in relative abundance upon exposure to tobacco smoke in mice. These findings align with human studies, where an increased presence of *Lachnospiraceae* ^26^, *Prevotella* ^3^, *Rikenellaceae*, and *Ruminococcaceae* ^27^ has been reported in smokers compared to non-smokers. While a previous study reported diverging results in mice on the level of *Lactobacillus* ^28^, a recent study confirmed our findings by showing a significant increase in the *Rikenellaceae* family after exposure to tobacco smoke^29^.

Despite sharing similar molecular pathways, metabolic rates differ significantly, paralleled by structural and cellular differences between human and murine lungs. For example, the mouse lung is built from a single large left lobe and four right lobes, whereas the human lung has two left and three right lobes. Similarly, submucosal glands are only present in the upper part of the mouse trachea but extend to the bronchioles in humans. Mice further metabolize certain compounds differently^30^. This can be improved by adapting dose and inhalation protocols to better mimic the human smoking pattern. Although this study included both male and female mice, it did not include hormonal effects across genders and life stages as in humans such as puberty and menopause which could also influence how smoking affects endocrine functions and reproductive health^2^. To capture this, further studies could monitor hormone levels alongside biomarkers like Cyp1a1 to provide insights into hormonal variation and its impact on smoke-induced health effects.

Bioinformatically assessing the functions provided by the microbiota, our results showed that the most significantly upregulated pathways under smoke exposure conditions are related to xenobiotics biodegradation and metabolism. Yang et al.^31^ observed similar increases in mice exposed to smoke, as did Qu et al.^32^ who reported a positive upregulation of the ethylbenzene degradation in A/J mice exposed to tobacco smoke carcinogens (NNK-BaP). These studies reinforce our findings and demonstrate consistency across different experiments. Moreover, in humans, previous studies have reported an upregulation of pathways related to xenobiotic metabolism in smokers gut microbiota^33^. This supports the hypothesis that mice may be suitable for human-like xenobiotic biodegradation and metabolism upon smoke exposure.

Our results also showed that biotin metabolism was compromised in mice exposed to tobacco smoke, while Qu et al.^32^ found the opposite effect, with increased biotin metabolism in their NNK-BaP-exposed mice. This difference could be due to the type of mice used in experiments. Metabolic pathways related to lipid metabolism have been reported to be affected by cigarette smoke exposure in mice^29^. Our results showed a decrease in the relative abundance of fatty acid degradation and alpha-linolenic acid metabolism in smoke-exposed samples, while steroid hormone biosynthesis increased. At the same time, we found no significant differences between their smoke- and air-exposed samples for the metabolic pathway of fatty acid degradation, while for the other outcomes, there is no supporting evidence. However, knowing that these processes are part of lipid metabolism and are affected by tobacco smoke, our results suggest a complex interaction between cigarette smoke and lipid metabolic pathways. Further research is needed to clarify how cigarette smoke influences these metabolic processes.

Finally, ribosome biogenesis in eukaryotes is a pathway enhanced by tobacco exposure. In previous studies, the ribosome pathway has been found to be enhanced by tobacco exposure in the lower respiratory tract microbiota of mice^34^. This suggests that tobacco might alter the composition of bacterial communities. However, in humans, there are still no conclusive observations on tobacco smoke consumption affecting this pathway.

### Limitations

While the approach of employing microbiome profiling and whole transcriptome analysis for a functional comparison between mouse and human allows for an unbiased picture, this type of exploration cannot capture all aspects relevant to responses to tobacco smoke exposure. This is exemplified by our observation that the microbiome mediated biosynthesis of flavones and flavonols in mice is slightly enhanced under air exposure conditions, yet there are no previous studies about this specific pathway. Although previous studies have reported that those compounds in the human gut microbiota are related to anti-inflammatory and antioxidant processes^35^, our study encountered challenges in validating this finding in humans and mice. This discrepancy underscores an inherent limitation in our experimental design. Therefore, further experiments, designed to specifically target selected processes are required to assess the validity of mice as a model for this process in humans. In addition, our study could not assess dose-response effects, as this would require a specific experimental setup. Several other confounding factors, such as diet, lifestyle, environmental conditions and comorbidities could not be addressed here. However, mouse models represent a powerful tool for creating scenarios aiming to control those confounding factors that cannot be captured in human settings.

## CONCLUSIONS

Our study demonstrates a strong concordance between murine and human gut microbiota responses to cigarette smoke, while also revealing important species-specific differences. Keeping those differences, and the resulting limitations in mind, such models offer a unique tool to close the gap between challenges when studying the impact of smoking behavior in humans and the urgent need to better address the interaction between smoking and complex diseases.

## Supplementary Material



## Data Availability

The data supporting this research can be found in the Supplementary file. The microbiota data generated as part of this study are provided as Supplementary file Tables 1–3. Supplementary file Table 1 contains the ASV-Taxa association for ASV #1 to ASV #3353; Supplementary file Table 2 contains the quantitative data from the 16S analysis, relative abundances, normalized via sub-sampling; Supplementary file Table 3 contains characteristics of individual samples. All other datasets are publicly available via the reference numbers listed in the materials section.
