# Supplementary material for: Mouse model validity for studying the impact of tobacco smoke on the human gut microbiota assessed via in silico and experimental approaches
Source: Tob Induc Dis. 2025 Sep 17;23:10.18332/tid/208251. doi: 10.18332/tid/208251 (PMC12442884; doi:10.18332/tid/208251)
Supplement: Supplementary file 1 [file TID-23-132-s1.pdf]

Supplemental Table 1

Format: tab delmited text

ASV_#	Kingdom	Phylum	Class	Order	Family	Genus	Species
ASV_1	Bacteria	Bacteroidetes	Bacteroidia	Bacteroidales	Muribaculaceae	Unknown	
ASV_2	Bacteria	Bacteroidetes	Bacteroidia	Bacteroidales	Rikenellaceae	Alistipes	
ASV_3	Bacteria	Bacteroidetes	Bacteroidia	Bacteroidales	Prevotellaceae	Prevotellaceae_NK3B31_group	
ASV_4	Bacteria	Bacteroidetes	Bacteroidia	Bacteroidales	Rikenellaceae	Alistipes	
ASV_5	Bacteria	Deferribacteres	Deferribacteres	Deferribacterales	Deferribacteraceae	Mucispirillum	Mucispirillum_schaedleri
ASV_6	Bacteria	Bacteroidetes	Bacteroidia	Bacteroidales	Muribaculaceae	Unknown	
ASV_7	Bacteria	Bacteroidetes	Bacteroidia	Bacteroidales	Muribaculaceae	Unknown	
ASV_8	Bacteria	Bacteroidetes	Bacteroidia	Bacteroidales	Muribaculaceae	Unknown	
ASV_9	Bacteria	Firmicutes	Bacilli	Lactobacillales	Lactobacillaceae	Lactobacillus	
ASV_10	Bacteria	Bacteroidetes	Bacteroidia	Bacteroidales	Prevotellaceae	Prevotellaceae_UCG-001	
ASV_11	Bacteria	Bacteroidetes	Bacteroidia	Bacteroidales	Muribaculaceae	Unknown	
ASV_12	Bacteria	Proteobacteria	Gammaproteobacteria	Betaproteobacteriales	Burkholderiaceae	Parasutterella	Parasutterella_excrementihominis
ASV_13	Bacteria	Bacteroidetes	Bacteroidia	Bacteroidales	Prevotellaceae	Prevotellaceae_UCG-001	
ASV_14	Bacteria	Bacteroidetes	Bacteroidia	Bacteroidales	Muribaculaceae	Unknown	
ASV_15	Bacteria	Firmicutes	Clostridia	Clostridiales	Lachnospiraceae	Lachnospiraceae_NK4A136_group	Lachnospiraceae_NK4A136_group_bacterium
ASV_16	Bacteria	Bacteroidetes	Bacteroidia	Bacteroidales	Muribaculaceae	Unknown	
ASV_17	Bacteria	Bacteroidetes	Bacteroidia	Bacteroidales	Muribaculaceae	Unknown	
ASV_18	Bacteria	Bacteroidetes	Bacteroidia	Bacteroidales	Muribaculaceae	Muribaculum	
ASV_19	Bacteria	Bacteroidetes	Bacteroidia	Bacteroidales	Muribaculaceae	Unknown	
ASV_20	Bacteria	Tenericutes	Mollicutes	Mollicutes_RF39	Unknown		
ASV_21	Bacteria	Bacteroidetes	Bacteroidia	Bacteroidales	Rikenellaceae	Alistipes	
ASV_22	Bacteria	Firmicutes	Clostridia	Clostridiales	Lachnospiraceae	Lachnospiraceae_NK4A136_group	
ASV_23	Bacteria	Bacteroidetes	Bacteroidia	Bacteroidales	Bacteroidaceae	Bacteroides	
ASV_24	Bacteria	Bacteroidetes	Bacteroidia	Bacteroidales	Rikenellaceae	Alistipes	
ASV_25	Bacteria	Bacteroidetes	Bacteroidia	Bacteroidales	Muribaculaceae	Unknown	
ASV_26	Bacteria	Bacteroidetes	Bacteroidia	Bacteroidales	Muribaculaceae	Unknown	
ASV_27	Bacteria	Bacteroidetes	Bacteroidia	Bacteroidales	Muribaculaceae	Unknown	

ASV_28	Bacteria	Bacteroidetes	Bacteroidia	Bacteroidales	Rikenellaceae	Alistipes	
ASV_29	Bacteria	Bacteroidetes	Bacteroidia	Bacteroidales	Muribaculaceae	Unknown	
ASV_30	Bacteria	Firmicutes	Clostridia	Clostridiales	Lachnospiraceae	Lachnospiraceae_NK4A136_group	
ASV_31	Bacteria	Bacteroidetes	Bacteroidia	Bacteroidales	Muribaculaceae	Unknown	
ASV_32	Bacteria	Bacteroidetes	Bacteroidia	Bacteroidales	Muribaculaceae	Unknown	
ASV_33	Bacteria	Bacteroidetes	Bacteroidia	Bacteroidales	Prevotellaceae	Prevotellaceae_UCG-001	
ASV_34	Bacteria	Bacteroidetes	Bacteroidia	Bacteroidales	Muribaculaceae	Unknown	
ASV_35	Bacteria	Bacteroidetes	Bacteroidia	Bacteroidales	Muribaculaceae	Unknown	
ASV_36	Bacteria	Proteobacteria	Alphaproteobacteria	Rhodospirillales	Unknown		
ASV_37	Bacteria	Firmicutes	Clostridia	Clostridiales	Lachnospiraceae	Lachnospiraceae_NK4A136_group	
ASV_38	Bacteria	Bacteroidetes	Bacteroidia	Bacteroidales	Rikenellaceae	Alistipes	
ASV_39	Bacteria	Firmicutes	Clostridia	Clostridiales	Lachnospiraceae	Lachnospiraceae_NK4A136_group	
ASV_40	Bacteria	Bacteroidetes	Bacteroidia	Bacteroidales	Muribaculaceae	Unknown	
ASV_41	Bacteria	Firmicutes	Bacilli	Lactobacillales	Lactobacillaceae	Lactobacillus	
ASV_42	Bacteria	Bacteroidetes	Bacteroidia	Bacteroidales	Muribaculaceae	Unknown	
ASV_43	Bacteria	Bacteroidetes	Bacteroidia	Bacteroidales	Muribaculaceae	Unknown	
ASV_44	Bacteria	Bacteroidetes	Bacteroidia	Bacteroidales	Muribaculaceae	Unknown	
ASV_45	Bacteria	Verrucomicrobia	Verrucomicrobiae	Verrucomicrobiales	Akkermansiaceae	Akkermansia	
ASV_46	Bacteria	Bacteroidetes	Bacteroidia	Bacteroidales	Muribaculaceae	Unknown	
ASV_47	Bacteria	Bacteroidetes	Bacteroidia	Bacteroidales	Muribaculaceae	Unknown	
ASV_48	Bacteria	Bacteroidetes	Bacteroidia	Bacteroidales	Muribaculaceae	Unknown	
ASV_49	Bacteria	Bacteroidetes	Bacteroidia	Bacteroidales	Muribaculaceae	Unknown	
ASV_50	Bacteria	Firmicutes	Clostridia	Clostridiales	Lachnospiraceae	Lachnospiraceae_NK4A136_group	
ASV_51	Bacteria	Bacteroidetes	Bacteroidia	Bacteroidales	Muribaculaceae	Unknown	
ASV_52	Bacteria	Firmicutes	Bacilli	Lactobacillales	Lactobacillaceae	Lactobacillus	
ASV_53	Bacteria	Bacteroidetes	Bacteroidia	Bacteroidales	Muribaculaceae	Unknown	
ASV_54	Bacteria	Firmicutes	Erysipelotrichia	Erysipelotrichales	Erysipelotrichaceae	Faecalibaculum	Faecalibaculum_rodentium
ASV_55	Bacteria	Bacteroidetes	Bacteroidia	Bacteroidales	Muribaculaceae	Unknown	
ASV_56	Bacteria	Firmicutes	Clostridia	Clostridiales	Lachnospiraceae	Lachnospiraceae_NK4A136_group	
ASV_57	Bacteria	Firmicutes	Clostridia	Clostridiales	Lachnospiraceae	Lachnospiraceae_NK4A136_group	
ASV_58	Bacteria	Firmicutes	Clostridia	Clostridiales	Ruminococcaceae	Ruminiclostridium_9	
ASV_59	Bacteria	Bacteroidetes	Bacteroidia	Bacteroidales	Muribaculaceae	Unknown	
ASV_60	Bacteria	Proteobacteria	Deltaproteobacteria	Desulfovibrionales	Desulfovibrionaceae	Bilophila	
ASV_61	Bacteria	Bacteroidetes	Bacteroidia	Bacteroidales	Muribaculaceae	Unknown	
ASV_62	Bacteria	Firmicutes	Clostridia	Clostridiales	Ruminococcaceae	Ruminiclostridium	
ASV_63	Bacteria	Bacteroidetes	Bacteroidia	Bacteroidales	Bacteroidaceae	Bacteroides	Bacteroides_caecimuris
ASV_64	Bacteria	Bacteroidetes	Bacteroidia	Bacteroidales	Tannerellaceae	Parabacteroides	Parabacteroides_goldsteinii

ASV_65	Bacteria	Bacteroidetes	Bacteroidia	Bacteroidales	Muribaculaceae	Unknown	
ASV_66	Bacteria	Cyanobacteria	Melainabacteria	Gastranaerophilales		Unknown	
ASV_67	Bacteria	Firmicutes	Clostridia	Clostridiales	Clostridiales_vadinBB60_group	Unknown	
ASV_68	Bacteria	Bacteroidetes	Bacteroidia	Bacteroidales	Muribaculaceae	Unknown	
ASV_69	Bacteria	Firmicutes	Clostridia	Clostridiales	Lachnospiraceae	Unknown	
ASV_70	Bacteria	Bacteroidetes	Bacteroidia	Bacteroidales	Bacteroidaceae	Bacteroides	Bacteroides_caecimuris
ASV_71	Bacteria	Tenericutes	Mollicutes	Anaeroplasmatales	Anaeroplasmataceae	Anaeroplasma	
ASV_72	Bacteria	Firmicutes	Clostridia	Clostridiales	Clostridiales_vadinBB60_group	Unknown	
ASV_73	Bacteria	Firmicutes	Clostridia	Clostridiales	Ruminococcaceae	Ruminiclostridium	
ASV_74	Bacteria	Firmicutes	Clostridia	Clostridiales	Ruminococcaceae	Ruminiclostridium_9	
ASV_75	Bacteria	Firmicutes	Clostridia	Clostridiales	Clostridiales_vadinBB60_group	Unknown	
ASV_76	Bacteria	Bacteroidetes	Bacteroidia	Bacteroidales	Muribaculaceae	Unknown	
ASV_77	Bacteria	Bacteroidetes	Bacteroidia	Bacteroidales	Muribaculaceae	Muribaculum	
ASV_78	Bacteria	Firmicutes	Bacilli	Lactobacillales	Lactobacillaceae	Lactobacillus	Lactobacillus_reuteri
ASV_79	Bacteria	Firmicutes	Clostridia	Clostridiales	Ruminococcaceae	Ruminiclostridium_9	
ASV_80	Bacteria	Firmicutes	Clostridia	Clostridiales	Lachnospiraceae	Lachnospiraceae_NK4A136_group	
ASV_81	Bacteria	Bacteroidetes	Bacteroidia	Bacteroidales	Muribaculaceae	Unknown	
ASV_82	Bacteria	Proteobacteria	Gammaproteobacteria	Betaproteobacteriales	Burkholderiaceae	Parasutterella	
ASV_83	Bacteria	Firmicutes	Bacilli	Lactobacillales	Lactobacillaceae	Lactobacillus	
ASV_84	Bacteria	Firmicutes	Clostridia	Clostridiales	Lachnospiraceae	Lachnospiraceae_NK4A136_group	Lachnospiraceae_NK4A136_group_bacterium
ASV_85	Bacteria	Bacteroidetes	Bacteroidia	Bacteroidales	Muribaculaceae	Unknown	
ASV_86	Bacteria	Firmicutes	Bacilli	Lactobacillales	Lactobacillaceae	Lactobacillus	
ASV_87	Bacteria	Bacteroidetes	Bacteroidia	Bacteroidales	Muribaculaceae	Unknown	
ASV_88	Bacteria	Firmicutes	Clostridia	Clostridiales	Ruminococcaceae	Oscillibacter	
ASV_89	Bacteria	Firmicutes	Clostridia	Clostridiales	Ruminococcaceae	Ruminococcaceae_UCG-014	
ASV_90	Bacteria	Firmicutes	Clostridia	Clostridiales	Ruminococcaceae	Ruminiclostridium_9	
ASV_91	Bacteria	Firmicutes	Erysipelotrichia	Erysipelotrichales	Erysipelotrichaceae	Faecalibaculum	Faecalibaculum_rodentium
ASV_92	Bacteria	Tenericutes	Mollicutes	Mollicutes_RF39	Unknown		
ASV_93	Bacteria	Firmicutes	Clostridia	Clostridiales	Lachnospiraceae	Unknown	
ASV_94	Bacteria	Bacteroidetes	Bacteroidia	Bacteroidales	Muribaculaceae	Unknown	
ASV_95	Bacteria	Bacteroidetes	Bacteroidia	Bacteroidales	Muribaculaceae	Unknown	
ASV_96	Bacteria	Firmicutes	Clostridia	Clostridiales	Ruminococcaceae	Ruminococcaceae_UCG-014	
ASV_97	Bacteria	Bacteroidetes	Bacteroidia	Bacteroidales	Muribaculaceae	Unknown	
ASV_98	Bacteria	Firmicutes	Clostridia	Clostridiales	Lachnospiraceae	Lachnospiraceae_NK4A136_group	
ASV_99	Bacteria	Bacteroidetes	Bacteroidia	Bacteroidales	Muribaculaceae	Unknown	
ASV_100	Bacteria	Firmicutes	Clostridia	Clostridiales	Ruminococcaceae	Ruminiclostridium	

ASV_101	Bacteria	Bacteroidetes	Bacteroidia	Bacteroidales	Muribaculaceae	Unknown	
ASV_102	Bacteria	Firmicutes	Clostridia	Clostridiales	Lachnospiraceae	Unknown	
ASV_103	Bacteria	Firmicutes	Clostridia	Clostridiales	Lachnospiraceae	Unknown	
ASV_104	Bacteria	Firmicutes	Clostridia	Clostridiales	Ruminococcaceae	Ruminococcaceae_UCG-014	
ASV_105	Bacteria	Firmicutes	Clostridia	Clostridiales	Ruminococcaceae	Ruminiclostridium	
ASV_106	Bacteria	Firmicutes	Clostridia	Clostridiales	Lachnospiraceae	Lachnospiraceae_NK4A136_group	
ASV_107	Bacteria	Firmicutes	Clostridia	Clostridiales	Lachnospiraceae	Lachnospiraceae_NK4A136_group	
ASV_108	Bacteria	Firmicutes	Clostridia	Clostridiales	Ruminococcaceae	Unknown	
ASV_109	Bacteria	Bacteroidetes	Bacteroidia	Bacteroidales	Muribaculaceae	Unknown	
ASV_110	Bacteria	Firmicutes	Clostridia	Clostridiales	Ruminococcaceae	Unknown	
ASV_111	Bacteria	Firmicutes	Clostridia	Clostridiales	Ruminococcaceae	Oscillibacter	
ASV_112	Bacteria	Firmicutes	Clostridia	Clostridiales	Lachnospiraceae	Lachnospiraceae_NK4A136_group	
ASV_113	Bacteria	Firmicutes	Clostridia	Clostridiales	Lachnospiraceae	Unknown	
ASV_114	Bacteria	Firmicutes	Bacilli	Lactobacillales	Lactobacillaceae	Lactobacillus	
ASV_115	Bacteria	Firmicutes	Clostridia	Clostridiales	Lachnospiraceae	Lachnoclostridium_10	
ASV_116	Bacteria	Firmicutes	Clostridia	Clostridiales	Lachnospiraceae	Lachnospiraceae_NK4A136_group	
ASV_117	Bacteria	Firmicutes	Clostridia	Clostridiales	Ruminococcaceae	Unknown	
ASV_118	Bacteria	Firmicutes	Clostridia	Clostridiales	Peptococcaceae	Unknown	
ASV_119	Bacteria	Firmicutes	Clostridia	Clostridiales	Ruminococcaceae	Oscillibacter	
ASV_120	Bacteria	Firmicutes	Bacilli	Lactobacillales	Enterococcaceae	Enterococcus	
ASV_121	Bacteria	Bacteroidetes	Bacteroidia	Bacteroidales	Bacteroidaceae	Bacteroides	Bacteroides_dorei
ASV_122	Bacteria	Bacteroidetes	Bacteroidia	Bacteroidales	Bacteroidaceae	Bacteroides	Bacteroides_uniformis
ASV_123	Bacteria	Firmicutes	Bacilli	Lactobacillales	Lactobacillaceae	Lactobacillus	
ASV_124	Bacteria	Firmicutes	Clostridia	Clostridiales	Lachnospiraceae	Unknown	
ASV_125	Bacteria	Firmicutes	Clostridia	Clostridiales	Ruminococcaceae	Oscillibacter	
ASV_126	Bacteria	Firmicutes	Clostridia	Clostridiales	Ruminococcaceae	Ruminiclostridium	
ASV_127	Bacteria	Firmicutes	Clostridia	Clostridiales	Lachnospiraceae	Lachnospiraceae_NK4A136_group	
ASV_128	Bacteria	Firmicutes	Clostridia	Clostridiales	Lachnospiraceae	Lachnospiraceae_NK4A136_group	
ASV_129	Bacteria	Firmicutes	Clostridia	Clostridiales	Lachnospiraceae	A2	
ASV_130	Bacteria	Firmicutes	Clostridia	Clostridiales	Lachnospiraceae	Unknown	
ASV_131	Bacteria	Firmicutes	Clostridia	Clostridiales	Lachnospiraceae	Lachnospiraceae_NK4A136_group	
ASV_132	Bacteria	Firmicutes	Clostridia	Clostridiales	Lachnospiraceae	Lachnospiraceae_NK4A136_group	Lachnospiraceae_NK4A136_group_bacterium
ASV_133	Bacteria	Firmicutes	Bacilli	Lactobacillales	Lactobacillaceae	Lactobacillus	
ASV_134	Bacteria	Firmicutes	Clostridia	Clostridiales	Lachnospiraceae	Unknown	
ASV_135	Bacteria	Bacteroidetes	Bacteroidia	Bacteroidales	Muribaculaceae	Unknown	
ASV_136	Bacteria	Firmicutes	Clostridia	Clostridiales	Lachnospiraceae	Lachnospiraceae_NK4A136_group	

ASV_137	Bacteria	Firmicutes	Clostridia	Clostridiales	Ruminococcaceae	Unknown
ASV_138	Bacteria	Bacteroidetes	Bacteroidia	Bacteroidales	Muribaculaceae	Unknown
ASV_139	Bacteria	Firmicutes	Clostridia	Clostridiales	Ruminococcaceae	Oscillibacter
ASV_140	Bacteria	Firmicutes	Clostridia	Clostridiales	Lachnospiraceae	Lachnospiraceae_NK4A136_group
ASV_141	Bacteria	Firmicutes	Clostridia	Clostridiales	Lachnospiraceae	Unknown
ASV_142	Bacteria	Tenericutes	Mollicutes	Mollicutes_RF39	Unknown	
ASV_143	Bacteria	Firmicutes	Clostridia	Clostridiales	Clostridiales_vadinBB60_group	Unknown
ASV_144	Bacteria	Proteobacteria	Gammaproteobacteria	Enterobacteriales	Enterobacteriaceae	Salmonella
ASV_145	Bacteria	Firmicutes	Clostridia	Clostridiales	Clostridiales_vadinBB60_group	Unknown
ASV_146	Bacteria	Bacteroidetes	Bacteroidia	Bacteroidales	Muribaculaceae	Unknown
ASV_147	Bacteria	Firmicutes	Clostridia	Clostridiales	Ruminococcaceae	Ruminiclostridium_9
ASV_148	Bacteria	Firmicutes	Clostridia	Clostridiales	Clostridiales_vadinBB60_group	Unknown
ASV_149	Bacteria	Proteobacteria	Gammaproteobacteria	Pseudomonadales	Pseudomonadaceae	Pseudomonas
ASV_150	Bacteria	Firmicutes	Clostridia	Clostridiales	Ruminococcaceae	Ruminococcaceae_UCG-014
ASV_151	Bacteria	Firmicutes	Bacilli	Bacillales	Staphylococcaceae	Staphylococcus
ASV_152	Bacteria	Firmicutes	Erysipelotrichia	Erysipelotrichales	Erysipelotrichaceae	Turicibacter
ASV_153	Bacteria	Firmicutes	Clostridia	Clostridiales	Lachnospiraceae	Lachnoclostridium
ASV_154	Bacteria	Firmicutes	Clostridia	Clostridiales	Ruminococcaceae	Ruminococcaceae_UCG-003
ASV_155	Bacteria	Firmicutes	Clostridia	Clostridiales	Clostridiales_vadinBB60_group	Unknown
ASV_156	Bacteria	Firmicutes	Bacilli	Lactobacillales	Lactobacillaceae	Lactobacillus
ASV_157	Bacteria	Firmicutes	Clostridia	Clostridiales	Unknown	
ASV_158	Bacteria	Firmicutes	Erysipelotrichia	Erysipelotrichales	Erysipelotrichaceae	Faecalibaculum
ASV_159	Bacteria	Firmicutes	Clostridia	Clostridiales	Lachnospiraceae	Lachnospiraceae_NK4A136_group
ASV_160	Bacteria	Firmicutes	Clostridia	Clostridiales	Lachnospiraceae	Lachnospiraceae_NK4A136_group
ASV_161	Bacteria	Firmicutes	Clostridia	Clostridiales	Lachnospiraceae	Lachnospiraceae_NK4A136_group
ASV_162	Bacteria	Firmicutes	Clostridia	Clostridiales	Ruminococcaceae	Ruminiclostridium
ASV_163	Bacteria	Firmicutes	Bacilli	Bacillales	Bacillaceae	Bacillus
ASV_164	Bacteria	Firmicutes	Clostridia	Clostridiales	Ruminococcaceae	Ruminococcaceae_UCG-014
ASV_165	Bacteria	Firmicutes	Clostridia	Clostridiales	Ruminococcaceae	Ruminococcaceae_UCG-014
ASV_166	Bacteria	Firmicutes	Clostridia	Clostridiales	Ruminococcaceae	Oscillibacter
ASV_167	Bacteria	Firmicutes	Clostridia	Clostridiales	Lachnospiraceae	Lachnospiraceae_NK4A136_group
ASV_168	Bacteria	Firmicutes	Clostridia	Clostridiales	Lachnospiraceae	Lachnospiraceae_NK4A136_group
ASV_169	Bacteria	Firmicutes	Clostridia	Clostridiales	Ruminococcaceae	Unknown
ASV_170	Bacteria	Firmicutes	Clostridia	Clostridiales	Ruminococcaceae	Oscillibacter
ASV_171	Bacteria	Bacteroidetes	Bacteroidia	Bacteroidales	Muribaculaceae	Unknown
ASV_172	Bacteria	Firmicutes	Erysipelotrichia	Erysipelotrichales	Erysipelotrichaceae	Faecalibaculum
ASV_173	Bacteria	Firmicutes	Clostridia	Clostridiales	Ruminococcaceae	Ruminiclostridium_9

ASV_174	Bacteria	Firmicutes	Clostridia	Clostridiales	Lachnospiraceae	Lachnospiraceae_NK4A136_group
ASV_175	Bacteria	Firmicutes	Clostridia	Clostridiales	Ruminococcaceae	Ruminococcaceae_UCG-014
ASV_176	Bacteria	Firmicutes	Clostridia	Clostridiales	Lachnospiraceae	Unknown
ASV_177	Bacteria	Firmicutes	Clostridia	Clostridiales	Lachnospiraceae	Lachnospiraceae_NK4A136_group
ASV_178	Bacteria	Firmicutes	Clostridia	Clostridiales	Ruminococcaceae	Oscillibacter
ASV_179	Bacteria	Firmicutes	Clostridia	Clostridiales	Ruminococcaceae	Ruminococcaceae_UCG-014
ASV_180	Bacteria	Firmicutes	Clostridia	Clostridiales	Ruminococcaceae	Ruminococcaceae_UCG-014
ASV_181	Bacteria	Firmicutes	Clostridia	Clostridiales	Ruminococcaceae	Unknown
ASV_182	Bacteria	Bacteroidetes	Bacteroidia	Bacteroidales	Muribaculaceae	Unknown
ASV_183	Bacteria	Firmicutes	Clostridia	Clostridiales	Clostridiales_vadinBB60_group	Unknown
ASV_184	Bacteria	Firmicutes	Clostridia	Clostridiales	Lachnospiraceae	Unknown
ASV_185	Bacteria	Firmicutes	Clostridia	Clostridiales	Lachnospiraceae	Unknown
ASV_186	Bacteria	Firmicutes	Clostridia	Clostridiales	Peptococcaceae	Unknown
ASV_187	Bacteria	Firmicutes	Clostridia	Clostridiales	Ruminococcaceae	UBA1819
ASV_188	Bacteria	Firmicutes	Clostridia	Clostridiales	Ruminococcaceae	Ruminococcaceae_UCG-014
ASV_189	Bacteria	Firmicutes	Clostridia	Clostridiales	Peptococcaceae	Unknown
ASV_190	Bacteria	Firmicutes	Clostridia	Clostridiales	Ruminococcaceae	Unknown
ASV_191	Bacteria	Firmicutes	Clostridia	Clostridiales	Lachnospiraceae	Lachnospiraceae_NK4A136_group
ASV_192	Bacteria	Firmicutes	Clostridia	Clostridiales	Ruminococcaceae	Ruminiclostridium
ASV_193	Bacteria	Firmicutes	Clostridia	Clostridiales	Lachnospiraceae	Lachnospiraceae_NK4A136_group
ASV_194	Bacteria	Firmicutes	Clostridia	Clostridiales	Lachnospiraceae	Lachnospiraceae_NK4A136_group
ASV_195	Bacteria	Firmicutes	Clostridia	Clostridiales	Lachnospiraceae	Unknown
ASV_196	Bacteria	Firmicutes	Erysipelotrichia	Erysipelotrichales	Erysipelotrichaceae	Faecalibaculum
ASV_197	Bacteria	Firmicutes	Clostridia	Clostridiales	Ruminococcaceae	Oscillibacter
ASV_198	Bacteria	Firmicutes	Clostridia	Clostridiales	Ruminococcaceae	Ruminiclostridium_9
ASV_199	Bacteria	Firmicutes	Clostridia	Clostridiales	Ruminococcaceae	Anaerotruncus
ASV_200	Bacteria	Firmicutes	Clostridia	Clostridiales	Lachnospiraceae	Unknown
ASV_201	Bacteria	Firmicutes	Clostridia	Clostridiales	Lachnospiraceae	Lachnospiraceae_NK4A136_group
ASV_202	Bacteria	Firmicutes	Clostridia	Clostridiales	Ruminococcaceae	Ruminiclostridium_9
ASV_203	Bacteria	Bacteroidetes	Bacteroidia	Bacteroidales	Rikenellaceae	Alistipes
ASV_204	Bacteria	Firmicutes	Clostridia	Clostridiales	Lachnospiraceae	Roseburia
ASV_205	Bacteria	Firmicutes	Clostridia	Clostridiales	Lachnospiraceae	Lachnospiraceae_NK4A136_group
ASV_206	Bacteria	Cyanobacteria	Melainabacteria	Gastranaerophilales		Unknown
ASV_207	Bacteria	Firmicutes	Clostridia	Clostridiales	Lachnospiraceae	A2
ASV_208	Bacteria	Firmicutes	Clostridia	Clostridiales	Ruminococcaceae	Ruminococcaceae_UCG-014
ASV_209	Bacteria	Firmicutes	Clostridia	Clostridiales	Lachnospiraceae	ASF356
ASV_210	Bacteria	Firmicutes	Clostridia	Clostridiales	Ruminococcaceae	Oscillibacter

ASV_211	Bacteria	Firmicutes	Clostridia	Clostridiales	Lachnospiraceae	Unknown	
ASV_212	Bacteria	Firmicutes	Bacilli	Bacillales	Listeriaceae	Listeria	Listeria_monocytogenes
ASV_213	Bacteria	Firmicutes	Clostridia	Clostridiales	Ruminococcaceae	Ruminiclostridium	
ASV_214	Bacteria	Firmicutes	Clostridia	Clostridiales	Ruminococcaceae	Oscillibacter	
ASV_215	Bacteria	Firmicutes	Clostridia	Clostridiales	Ruminococcaceae	Ruminiclostridium	
ASV_216	Bacteria	Firmicutes	Bacilli	Bacillales	Family_XI	Gemella	Gemella_haemolysans
ASV_217	Bacteria	Actinobacteria	Actinobacteria	Propionibacteriales	Propionibacteriaceae	Cutibacterium	
ASV_218	Bacteria	Firmicutes	Clostridia	Clostridiales	Ruminococcaceae	Oscillibacter	
ASV_219	Bacteria	Firmicutes	Clostridia	Clostridiales	Lachnospiraceae	Lachnospiraceae_NK4A136_group	
ASV_220	Bacteria	Firmicutes	Clostridia	Clostridiales	Lachnospiraceae	A2	
ASV_221	Bacteria	Firmicutes	Clostridia	Clostridiales	Lachnospiraceae	A2	
ASV_222	Bacteria	Firmicutes	Clostridia	Clostridiales	Ruminococcaceae	Ruminococcaceae_UCG-014	
ASV_223	Bacteria	Firmicutes	Bacilli	Bacillales	Bacillaceae	Bacillus	
ASV_224	Bacteria	Firmicutes	Clostridia	Clostridiales	Ruminococcaceae	Ruminococcaceae_UCG-014	
ASV_225	Bacteria	Firmicutes	Clostridia	Clostridiales	Lachnospiraceae	Lachnospiraceae_UCG-001	
ASV_226	Bacteria	Tenericutes	Mollicutes	Mollicutes_RF39	Unknown		
ASV_227	Bacteria	Firmicutes	Clostridia	Clostridiales	Ruminococcaceae	Ruminiclostridium	
ASV_228	Bacteria	Firmicutes	Erysipelotrichia	Erysipelotrichales	Erysipelotrichaceae	Faecalibaculum	Faecalibaculum_rodentium
ASV_229	Bacteria	Firmicutes	Clostridia	Clostridiales	Clostridiaceae_1	Clostridium_sensu_stricto_1	
ASV_230	Bacteria	Firmicutes	Clostridia	Clostridiales	Ruminococcaceae	Ruminiclostridium_9	
ASV_231	Bacteria	Bacteroidetes	Bacteroidia	Bacteroidales	Muribaculaceae	Unknown	
ASV_232	Bacteria	Bacteroidetes	Bacteroidia	Bacteroidales	Barnesiellaceae	Barnesiella	
ASV_233	Bacteria	Firmicutes	Clostridia	Clostridiales	Lachnospiraceae	Unknown	
ASV_234	Bacteria	Proteobacteria	Gammaproteobacteria	Betaproteobacteriales	Burkholderiaceae	Parasutterella	
ASV_235	Bacteria	Firmicutes	Clostridia	Clostridiales	Lachnospiraceae	Lachnospiraceae_NK4A136_group	
ASV_236	Bacteria			Unknown			
ASV_237	Bacteria	Firmicutes	Clostridia	Clostridiales	Lachnospiraceae	Unknown	
ASV_238	Bacteria	Firmicutes	Clostridia	Clostridiales	Ruminococcaceae	Ruminococcus_2	
ASV_239	Bacteria	Firmicutes	Clostridia	Clostridiales	Ruminococcaceae	Ruminococcaceae_UCG-014	
ASV_240	Bacteria	Firmicutes	Clostridia	Clostridiales	Ruminococcaceae	Ruminococcaceae_NK4A214_group	
ASV_241	Bacteria	Firmicutes	Erysipelotrichia	Erysipelotrichales	Erysipelotrichaceae	Unknown	
ASV_242	Bacteria	Firmicutes	Clostridia	Clostridiales	Ruminococcaceae	Ruminococcaceae_UCG-014	
ASV_243	Bacteria	Firmicutes	Clostridia	Clostridiales	Ruminococcaceae	Ruminiclostridium_9	
ASV_244	Bacteria	Firmicutes	Clostridia	Clostridiales	Ruminococcaceae	Unknown	
ASV_245	Bacteria	Firmicutes	Clostridia	Clostridiales	Lachnospiraceae	Lachnospiraceae_UCG-001	
ASV_246	Bacteria	Firmicutes	Clostridia	Clostridiales	Ruminococcaceae	Unknown	
ASV_247	Bacteria	Firmicutes	Clostridia	Clostridiales	Ruminococcaceae	Ruminiclostridium	

ASV_248	Bacteria	Firmicutes	Bacilli	Bacillales	Listeriaceae	Listeria	Listeria_monocytogenes
ASV_249	Bacteria	Firmicutes	Negativicutes	Selenomonadales	Veillonellaceae	Dialister	Dialister_invisus
ASV_250	Bacteria	Firmicutes	Clostridia	Clostridiales		Unknown	
ASV_251	Bacteria	Firmicutes	Clostridia	Clostridiales	Ruminococcaceae	Ruminiclostridium	
ASV_252	Bacteria	Firmicutes	Bacilli	Lactobacillales	Streptococcaceae	Streptococcus	Streptococcus_mitis
ASV_253	Bacteria	Bacteroidetes	Bacteroidia	Bacteroidales	Rikenellaceae	Alistipes	Alistipes_putredinis
ASV_254	Bacteria	Firmicutes	Clostridia	Clostridiales	Lachnospiraceae	A2	
ASV_255	Bacteria	Proteobacteria	Gammaproteobacteria	Pasteurellales	Pasteurellaceae	Haemophilus	
ASV_256	Bacteria	Firmicutes	Clostridia	Clostridiales	Ruminococcaceae	Ruminococcaceae_NK4A214_group	
ASV_257	Bacteria	Firmicutes	Erysipelotrichia	Erysipelotrichales	Erysipelotrichaceae	Faecalibaculum	
ASV_258	Bacteria	Firmicutes	Clostridia	Clostridiales	Ruminococcaceae	Ruminococcaceae_UCG-014	
ASV_259	Bacteria	Tenericutes	Mollicutes	Mollicutes_RF39	Unknown		
ASV_260	Bacteria	Firmicutes	Clostridia	Clostridiales	Lachnospiraceae	Lachnospiraceae_NK4A136_group	
ASV_261	Bacteria	Firmicutes	Clostridia	Clostridiales	Clostridiales_vadinBB60_group	Unknown	
ASV_262	Bacteria	Firmicutes	Clostridia	Clostridiales		Unknown	
ASV_263	Bacteria	Firmicutes	Clostridia	Clostridiales	Ruminococcaceae	Ruminiclostridium_9	
ASV_264	Bacteria	Firmicutes	Clostridia	Clostridiales	Lachnospiraceae	Unknown	
ASV_265	Bacteria	Firmicutes	Clostridia	Clostridiales	Ruminococcaceae	Ruminiclostridium	
ASV_266	Bacteria	Firmicutes	Clostridia	Clostridiales	Ruminococcaceae	Ruminococcaceae_UCG-014	
ASV_267	Bacteria	Firmicutes	Clostridia	Clostridiales	Lachnospiraceae	Unknown	
ASV_268	Bacteria	Firmicutes	Bacilli	Lactobacillales	Lactobacillaceae	Lactobacillus	
ASV_269	Bacteria	Firmicutes	Clostridia	Clostridiales	Lachnospiraceae	Lachnospiraceae_NK4A136_group	
ASV_270	Bacteria	Firmicutes	Clostridia	Clostridiales	Ruminococcaceae	Oscillibacter	
ASV_271	Bacteria	Firmicutes	Clostridia	Clostridiales	Lachnospiraceae	Unknown	
ASV_272	Bacteria	Firmicutes	Clostridia	Clostridiales	Clostridiales_vadinBB60_group	Unknown	
ASV_273	Bacteria	Firmicutes	Bacilli	Lactobacillales	Lactobacillaceae	Lactobacillus	
ASV_274	Bacteria	Firmicutes	Clostridia	Clostridiales	Lachnospiraceae	Unknown	
ASV_275	Bacteria	Firmicutes	Clostridia	Clostridiales	Lachnospiraceae	Unknown	
ASV_276	Bacteria	Firmicutes	Clostridia	Clostridiales	Lachnospiraceae	Unknown	
ASV_277	Bacteria	Firmicutes	Clostridia	Clostridiales	Clostridiaceae_1	Candidatus_Arthromitus	
ASV_278	Bacteria	Firmicutes	Clostridia	Clostridiales	Lachnospiraceae	Lachnospiraceae_NK4A136_group	
ASV_279	Bacteria	Firmicutes	Clostridia	Clostridiales	Ruminococcaceae	Ruminiclostridium_9	
ASV_280	Bacteria	Firmicutes	Clostridia	Clostridiales	Lachnospiraceae	Unknown	
ASV_281	Bacteria	Firmicutes	Clostridia	Clostridiales	Ruminococcaceae	Ruminococcaceae_NK4A214_group	
ASV_282	Bacteria	Firmicutes	Clostridia	Clostridiales	Lachnospiraceae	Lachnospiraceae_UCG-006	
ASV_283	Bacteria	Firmicutes	Erysipelotrichia	Erysipelotrichales	Erysipelotrichaceae	Faecalibaculum	
ASV_284	Bacteria	Firmicutes	Clostridia	Clostridiales	Ruminococcaceae	Ruminococcus_2	

ASV_285	Bacteria	Firmicutes	Clostridia	Clostridiales	Ruminococcaceae	Ruminococcaceae_UCG-014	
ASV_286	Bacteria	Firmicutes	Clostridia	Clostridiales	Ruminococcaceae	Ruminiclostridium_9	
ASV_287	Bacteria	Firmicutes	Clostridia	Clostridiales	Lachnospiraceae	Unknown	
ASV_288	Bacteria	Firmicutes	Clostridia	Clostridiales	Lachnospiraceae	Lachnoclostridium_10	
ASV_289	Bacteria	Bacteroidetes	Bacteroidia	Bacteroidales	Tannerellaceae	Parabacteroides	Parabacteroides_distasonis
ASV_290	Bacteria	Firmicutes	Clostridia	Clostridiales	Ruminococcaceae	Subdoligranulum	
ASV_291	Bacteria	Proteobacteria	Gammaproteobacteria	Enterobacteriales	Enterobacteriaceae	Escherichia/Shigella	Escherichia/Shigella_coli
ASV_292	Bacteria	Firmicutes	Clostridia	Clostridiales	Lachnospiraceae	Roseburia	
ASV_293	Bacteria	Firmicutes	Clostridia	Clostridiales		Unknown	
ASV_294	Bacteria	Firmicutes	Erysipelotrichia	Erysipelotrichales	Erysipelotrichaceae	Faecalibaculum	
ASV_295	Bacteria	Firmicutes	Clostridia	Clostridiales	Ruminococcaceae	Ruminiclostridium_6	
ASV_296	Bacteria	Firmicutes	Clostridia	Clostridiales	Ruminococcaceae	Ruminiclostridium_9	
ASV_297	Bacteria	Firmicutes	Clostridia	Clostridiales	Lachnospiraceae	Unknown	
ASV_298	Bacteria	Bacteroidetes	Bacteroidia	Bacteroidales	Muribaculaceae	Unknown	
ASV_299	Bacteria	Firmicutes	Clostridia	Clostridiales	Ruminococcaceae	Ruminococcaceae_UCG-014	
ASV_300	Bacteria	Firmicutes	Clostridia	Clostridiales	Ruminococcaceae	Ruminococcaceae_UCG-009	
ASV_301	Bacteria	Firmicutes	Clostridia	Clostridiales	Lachnospiraceae	Lachnospiraceae_NK4A136_group	
ASV_302	Bacteria	Firmicutes	Clostridia	Clostridiales	Ruminococcaceae	Unknown	
ASV_303	Bacteria	Firmicutes	Clostridia	Clostridiales	Ruminococcaceae	Ruminococcaceae_UCG-014	
ASV_304	Bacteria	Proteobacteria	Gammaproteobacteria	Enterobacteriales	Enterobacteriaceae	Escherichia/Shigella	
ASV_305	Bacteria	Firmicutes	Clostridia	Clostridiales	Ruminococcaceae	Anaerotruncus	
ASV_306	Bacteria	Firmicutes	Clostridia	Clostridiales	Ruminococcaceae	Ruminococcaceae_UCG-014	
ASV_307	Bacteria	Firmicutes	Clostridia	Clostridiales	Ruminococcaceae	Oscillibacter	
ASV_308	Bacteria	Firmicutes	Bacilli	Lactobacillales	Lactobacillaceae	Lactobacillus	Lactobacillus_fermentum
ASV_309	Bacteria	Firmicutes	Clostridia	Clostridiales	Lachnospiraceae	Unknown	
ASV_310	Bacteria	Proteobacteria	Alphaproteobacteria	Rhodospirillales	Unknown		
ASV_311	Bacteria	Firmicutes	Clostridia	Clostridiales	Ruminococcaceae	Ruminococcus_1	
ASV_312	Bacteria	Firmicutes	Bacilli	Bacillales	Family_XI	Gemella	
ASV_313	Bacteria	Firmicutes	Clostridia	Clostridiales	Ruminococcaceae	Oscillibacter	
ASV_314	Bacteria	Firmicutes	Clostridia	Clostridiales	Ruminococcaceae	Ruminococcaceae_UCG-014	
ASV_315	Bacteria	Firmicutes	Clostridia	Clostridiales	Lachnospiraceae	Lachnospiraceae_NK4A136_group	
ASV_316	Bacteria	Firmicutes	Clostridia	Clostridiales	Lachnospiraceae	Unknown	
ASV_317	Bacteria	Firmicutes	Clostridia	Clostridiales	Ruminococcaceae	Butyricicoccus	
ASV_318	Bacteria	Firmicutes	Clostridia	Clostridiales	Clostridiales_vadinBB60_group	Unknown	
ASV_319	Bacteria	Tenericutes	Mollicutes	Mollicutes_RF39	Unknown		
ASV_320	Bacteria	Firmicutes	Clostridia	Clostridiales	Lachnospiraceae	Lachnospiraceae_UCG-001	

ASV_321	Bacteria	Tenericutes	Mollicutes	Mollicutes_RF39	Unknown		
ASV_322	Bacteria	Firmicutes	Clostridia	Clostridiales	Family_XIII	Unknown	
ASV_323	Bacteria	Firmicutes	Clostridia	Clostridiales	Ruminococcaceae	Ruminiclostridium_9	
ASV_324	Bacteria	Firmicutes	Clostridia	Clostridiales	Ruminococcaceae	Ruminococcaceae_UCG-014	
ASV_325	Bacteria	Firmicutes	Clostridia	Clostridiales	Unknown		
ASV_326	Bacteria	Firmicutes	Clostridia	Clostridiales	Ruminococcaceae	Ruminococcaceae_UCG-014	
ASV_327	Bacteria	Firmicutes	Clostridia	Clostridiales	Lachnospiraceae	Lachnospiraceae_NK4A136_group	
ASV_328	Bacteria	Bacteroidetes	Bacteroidia	Bacteroidales	Tannerellaceae	Parabacteroides	Parabacteroides_goldsteinii
ASV_329	Bacteria	Firmicutes	Clostridia	Clostridiales	Lachnospiraceae	A2	
ASV_330	Bacteria	Firmicutes	Clostridia	Clostridiales	Ruminococcaceae	Ruminococcaceae_UCG-010	
ASV_331	Bacteria	Firmicutes	Erysipelotrichia	Erysipelotrichales	Erysipelotrichaceae	Unknown	
ASV_332	Bacteria	Firmicutes	Clostridia	Clostridiales	Lachnospiraceae	Unknown	
ASV_333	Bacteria	Firmicutes	Bacilli	Lactobacillales	Carnobacteriaceae	Granulicatella	
ASV_334	Bacteria	Firmicutes	Clostridia	Clostridiales	Ruminococcaceae	Ruminococcaceae_UCG-014	
ASV_335	Bacteria	Firmicutes	Clostridia	Clostridiales	Ruminococcaceae	Unknown	
ASV_336	Bacteria	Firmicutes	Clostridia	Clostridiales	Ruminococcaceae	Unknown	
ASV_337	Bacteria	Firmicutes	Clostridia	Clostridiales	Lachnospiraceae	Lachnospiraceae_UCG-006	
ASV_338	Bacteria	Actinobacteria	Coriobacteriia	Coriobacteriales	Eggerthellaceae	Unknown	
ASV_339	Bacteria	Firmicutes	Clostridia	Clostridiales	Ruminococcaceae	Ruminiclostridium_9	
ASV_340	Bacteria	Firmicutes	Clostridia	Clostridiales	Lachnospiraceae	Unknown	
ASV_341	Bacteria	Firmicutes	Clostridia	Clostridiales	Lachnospiraceae	Lachnospiraceae_UCG-006	
ASV_342	Bacteria	Actinobacteria	Coriobacteriia	Coriobacteriales	Eggerthellaceae	Enterorhabdus	Enterorhabdus_caecimuris
ASV_343	Bacteria	Firmicutes	Erysipelotrichia	Erysipelotrichales	Erysipelotrichaceae	Candidatus_Stoquefichus	
ASV_344	Bacteria	Firmicutes	Clostridia	Clostridiales	Ruminococcaceae	Ruminococcaceae_UCG-014	
ASV_345	Bacteria	Firmicutes	Clostridia	Clostridiales	Ruminococcaceae	Oscillibacter	
ASV_346	Bacteria	Bacteroidetes	Bacteroidia	Bacteroidales	Bacteroidaceae	Bacteroides	Bacteroides_thetaiotaomicron
ASV_347	Bacteria	Firmicutes	Clostridia	Clostridiales	Lachnospiraceae	Lachnospiraceae_NK4A136_group	
ASV_348	Bacteria	Bacteroidetes	Bacteroidia	Bacteroidales	Bacteroidaceae	Bacteroides	Bacteroides_stercoris
ASV_349	Bacteria	Firmicutes	Clostridia	Clostridiales	Ruminococcaceae	Ruminiclostridium_9	
ASV_350	Bacteria	Firmicutes	Clostridia	Clostridiales	Lachnospiraceae	Unknown	
ASV_351	Bacteria	Firmicutes	Clostridia	Clostridiales	Unknown		
ASV_352	Bacteria	Firmicutes	Clostridia	Clostridiales	Ruminococcaceae	Ruminiclostridium_6	
ASV_353	Bacteria	Proteobacteria	Gammaproteobacteria	Enterobacteriales	Enterobacteriaceae	Escherichia/Shigella	
ASV_354	Bacteria	Firmicutes	Clostridia	Clostridiales	Ruminococcaceae	Ruminococcaceae_UCG-014	
ASV_355	Bacteria	Firmicutes	Clostridia	Clostridiales	Ruminococcaceae	Unknown	
ASV_356	Bacteria	Firmicutes	Clostridia	Clostridiales	Lachnospiraceae	Lachnospiraceae_NK4A136_group	
ASV_357	Bacteria	Firmicutes	Clostridia	Clostridiales	Ruminococcaceae	Ruminiclostridium_9	

ASV_358	Bacteria	Firmicutes	Clostridia	Clostridiales	Lachnospiraceae	Unknown	
ASV_359	Bacteria	Bacteroidetes	Bacteroidia	Bacteroidales	Bacteroidaceae	Bacteroides	Bacteroides_fragilis
ASV_360	Bacteria	Firmicutes	Clostridia	Clostridiales	Lachnospiraceae	Unknown	
ASV_361	Bacteria	Firmicutes	Clostridia	Clostridiales	Lachnospiraceae	Lachnospiraceae_NK4A136_group	
ASV_362	Bacteria	Firmicutes	Clostridia	Clostridiales	Lachnospiraceae	Unknown	
ASV_363	Bacteria	Firmicutes	Clostridia	Clostridiales	Lachnospiraceae	Unknown	
ASV_364	Bacteria	Proteobacteria	Gammaproteobacteria	Pasteurellales	Pasteurellaceae	Haemophilus	Haemophilus_parainfluenzae
ASV_365	Bacteria	Firmicutes	Clostridia	Clostridiales	Clostridiales_vadinBB60_group	Unknown	
ASV_366	Bacteria	Firmicutes	Clostridia	Clostridiales	Lachnospiraceae	Unknown	
ASV_367	Bacteria	Firmicutes	Clostridia	Clostridiales	Peptococcaceae	Unknown	
ASV_368	Bacteria	Tenericutes	Mollicutes	Mollicutes_RF39	Unknown		
ASV_369	Bacteria	Proteobacteria	Gammaproteobacteria	Enterobacteriales	Enterobacteriaceae	Escherichia/Shigella	
ASV_370	Bacteria	Firmicutes	Clostridia	Clostridiales	Lachnospiraceae	Lachnoclostridium	
ASV_371	Bacteria	Firmicutes	Clostridia	Clostridiales	Lachnospiraceae	Unknown	
ASV_372	Bacteria	Bacteroidetes	Bacteroidia	Bacteroidales	Muribaculaceae	Unknown	
ASV_373	Bacteria	Firmicutes	Clostridia	Clostridiales	Lachnospiraceae	Lachnospiraceae_NK4A136_group	
ASV_374	Bacteria	Firmicutes	Clostridia	Clostridiales	Lachnospiraceae	Lachnospiraceae_UCG-001	
ASV_375	Bacteria	Firmicutes	Clostridia	Clostridiales	Lachnospiraceae	ASF356	
ASV_376	Bacteria	Firmicutes	Clostridia	Clostridiales	Ruminococcaceae	Ruminiclostridium_9	
ASV_377	Bacteria	Firmicutes	Erysipelotrichia	Erysipelotrichales	Erysipelotrichaceae	Faecalibaculum	
ASV_378	Bacteria	Proteobacteria	Gammaproteobacteria	Enterobacteriales	Enterobacteriaceae	Salmonella	
ASV_379	Bacteria	Firmicutes	Clostridia	Clostridiales	Lachnospiraceae	Unknown	
ASV_380	Bacteria	Firmicutes	Clostridia	Clostridiales	Ruminococcaceae	Oscillibacter	
ASV_381	Bacteria	Firmicutes	Clostridia	Clostridiales	Ruminococcaceae	Oscillibacter	
ASV_382	Bacteria	Bacteroidetes	Bacteroidia	Bacteroidales	Bacteroidaceae	Bacteroides	Bacteroides_vulgatus
ASV_383	Bacteria	Firmicutes	Clostridia	Clostridiales	Lachnospiraceae	Lachnospiraceae_UCG-001	
ASV_384	Bacteria	Firmicutes	Clostridia	Clostridiales	Christensenellaceae	Christensenellaceae_R-7_group	
ASV_385	Bacteria	Firmicutes	Clostridia	Clostridiales	Lachnospiraceae	ASF356	
ASV_386	Bacteria	Firmicutes	Clostridia	Clostridiales	Lachnospiraceae	Unknown	
ASV_387	Bacteria	Firmicutes	Clostridia	Clostridiales	Lachnospiraceae	Lachnospiraceae_NK4A136_group	
ASV_388	Bacteria	Firmicutes	Clostridia	Clostridiales	Ruminococcaceae	Butyricicoccus	
ASV_389	Bacteria	Firmicutes	Negativicutes	Selenomonadales	Veillonellaceae	Veillonella	
ASV_390	Bacteria	Firmicutes	Clostridia	Clostridiales	Ruminococcaceae	Ruminococcaceae_UCG-014	
ASV_391	Bacteria	Firmicutes	Clostridia	Clostridiales	Ruminococcaceae	Oscillibacter	
ASV_392	Bacteria	Firmicutes	Clostridia	Clostridiales	Ruminococcaceae	Oscillibacter	
ASV_393	Bacteria	Bacteroidetes	Bacteroidia	Bacteroidales	Bacteroidaceae	Bacteroides	Bacteroides_caccae
ASV_394	Bacteria	Firmicutes	Clostridia	Clostridiales	Ruminococcaceae	Ruminiclostridium	

ASV_395	Bacteria	Firmicutes	Clostridia	Clostridiales	Ruminococcaceae	Unknown	
ASV_396	Bacteria	Firmicutes	Clostridia	Clostridiales	Lachnospiraceae	Unknown	
ASV_397	Bacteria	Firmicutes	Clostridia	Clostridiales	Ruminococcaceae	Unknown	
ASV_398	Bacteria	Firmicutes	Bacilli	Lactobacillales	Streptococcaceae	Streptococcus	
ASV_399	Bacteria	Proteobacteria	Deltaproteobacteria	Desulfovibrionales	Desulfovibrionaceae	Desulfovibrio	
ASV_400	Bacteria	Firmicutes	Clostridia	Clostridiales	Lachnospiraceae	Lachnospiraceae_NK4A136_group	
ASV_401	Bacteria	Firmicutes	Clostridia	Clostridiales	Lachnospiraceae	Unknown	
ASV_402	Bacteria	Firmicutes	Clostridia	Clostridiales	Lachnospiraceae	Unknown	
ASV_403	Bacteria	Firmicutes	Clostridia	Clostridiales	Lachnospiraceae	Unknown	
ASV_404	Bacteria	Firmicutes	Clostridia	Clostridiales	Lachnospiraceae	Unknown	
ASV_405	Bacteria	Firmicutes	Erysipelotrichia	Erysipelotrichales	Erysipelotrichaceae	Erysipelatoclostridium	
ASV_406	Bacteria	Firmicutes	Clostridia	Clostridiales	Clostridiales_vadinBB60_group	Unknown	
ASV_407	Bacteria	Firmicutes	Clostridia	Clostridiales	Lachnospiraceae	Unknown	
ASV_408	Bacteria	Firmicutes	Clostridia	Clostridiales	Lachnospiraceae	Lachnospiraceae_NK4A136_group	
ASV_409	Bacteria	Firmicutes	Clostridia	Clostridiales	Ruminococcaceae	Unknown	
ASV_410	Bacteria	Firmicutes	Clostridia	Clostridiales	Ruminococcaceae	Ruminiclostridium_9	
ASV_411	Bacteria	Firmicutes	Clostridia	Clostridiales	Lachnospiraceae	Unknown	
ASV_412	Bacteria	Firmicutes	Clostridia	Clostridiales	Ruminococcaceae	Ruminococcaceae_UCG-013	
ASV_413	Bacteria	Firmicutes	Clostridia	Clostridiales	Ruminococcaceae	Ruminiclostridium_9	
ASV_414	Bacteria	Firmicutes	Clostridia	Clostridiales	Lachnospiraceae	Unknown	
ASV_415	Bacteria	Firmicutes	Clostridia	Clostridiales	Lachnospiraceae	Lachnospiraceae_NC2004_group	
ASV_416	Bacteria	Firmicutes	Clostridia	Clostridiales	Lachnospiraceae	Unknown	
ASV_417	Bacteria	Firmicutes	Bacilli	Lactobacillales	Streptococcaceae	Streptococcus	Streptococcus_mitis
ASV_418	Bacteria	Proteobacteria	Gammaproteobacteria	Enterobacteriales	Enterobacteriaceae	Yokenella	
ASV_419	Bacteria	Firmicutes	Clostridia	Clostridiales	Lachnospiraceae	Lachnospiraceae_UCG-001	
ASV_420	Bacteria	Firmicutes	Clostridia	Clostridiales	Ruminococcaceae	Oscillibacter	
ASV_421	Bacteria	Firmicutes	Clostridia	Clostridiales	Lachnospiraceae	Lachnospiraceae_NK4A136_group	
ASV_422	Bacteria	Proteobacteria	Gammaproteobacteria	Pasteurellales	Pasteurellaceae	Actinobacillus	
ASV_423	Bacteria	Firmicutes	Clostridia	Clostridiales	Ruminococcaceae	Unknown	
ASV_424	Bacteria	Firmicutes	Clostridia	Clostridiales	Ruminococcaceae	Oscillibacter	
ASV_425	Bacteria	Firmicutes	Clostridia	Clostridiales	Lachnospiraceae	Lachnospiraceae_NK4A136_group	
ASV_426	Bacteria	Actinobacteria	Coriobacteriia	Coriobacteriales	Atopobiaceae	Unknown	
ASV_427	Bacteria	Firmicutes	Clostridia	Clostridiales	Ruminococcaceae	Ruminococcaceae_UCG-014	
ASV_428	Bacteria	Firmicutes	Clostridia	Clostridiales	Lachnospiraceae	Unknown	
ASV_429	Bacteria	Tenericutes	Mollicutes	Mollicutes_RF39	Unknown		
ASV_430	Bacteria	Tenericutes	Mollicutes	Mollicutes_RF39	Unknown		
ASV_431	Bacteria	Proteobacteria	Gammaproteobacteria	Enterobacteriales	Enterobacteriaceae	Unknown	

ASV_432	Bacteria	Firmicutes	Clostridia	Clostridiales	Lachnospiraceae	Unknown	
ASV_433	Bacteria	Proteobacteria	Gammaproteobacteria	Betaproteobacteriales	Burkholderiaceae	Diaphorobacter	Diaphorobacter_nitroreducens
ASV_434	Bacteria	Firmicutes	Erysipelotrichia	Erysipelotrichales	Erysipelotrichaceae	Unknown	
ASV_435	Bacteria	Firmicutes	Bacilli	Lactobacillales	Lactobacillaceae	Lactobacillus	Lactobacillus_fermentum
ASV_436	Bacteria	Firmicutes	Bacilli	Bacillales	Listeriaceae	Listeria	Listeria_monocytogenes
ASV_437	Bacteria	Firmicutes	Bacilli	Bacillales	Staphylococcaceae	Staphylococcus	
ASV_438	Bacteria	Tenericutes	Mollicutes	Mollicutes_RF39	Unknown		
ASV_439	Bacteria	Firmicutes	Clostridia	Clostridiales	Ruminococcaceae	Ruminococcaceae_UCG-014	
ASV_440	Bacteria	Firmicutes	Clostridia	Clostridiales	Lachnospiraceae	Roseburia	
ASV_441	Bacteria	Firmicutes	Clostridia	Clostridiales	Ruminococcaceae	Ruminococcaceae_UCG-014	
ASV_442	Bacteria	Firmicutes	Clostridia	Clostridiales	Ruminococcaceae	Ruminococcaceae_UCG-014	
ASV_443	Bacteria	Firmicutes	Clostridia	Clostridiales	Lachnospiraceae	ASF356	
ASV_444	Bacteria	Firmicutes	Clostridia	Clostridiales	Ruminococcaceae	Ruminiclostridium_9	
ASV_445	Bacteria	Firmicutes	Clostridia	Clostridiales	Clostridiaceae_1	Clostridium_sensu_stricto_1	
ASV_446	Bacteria	Firmicutes	Clostridia	Clostridiales	Lachnospiraceae	Lachnospiraceae_NK4A136_group	
ASV_447	Bacteria	Firmicutes	Clostridia	Clostridiales	Lachnospiraceae	Roseburia	
ASV_448	Bacteria	Firmicutes	Bacilli	Lactobacillales	Lactobacillaceae	Lactobacillus	
ASV_449	Bacteria	Firmicutes	Clostridia	Clostridiales	Ruminococcaceae	Unknown	
ASV_450	Bacteria	Firmicutes	Clostridia	Clostridiales	Ruminococcaceae	Ruminococcaceae_UCG-014	
ASV_451	Bacteria	Firmicutes	Clostridia	Clostridiales	Ruminococcaceae	Ruminococcaceae_UCG-014	
ASV_452	Bacteria	Firmicutes	Clostridia	Clostridiales	Lachnospiraceae	Lachnospiraceae_NK4A136_group	
ASV_453	Bacteria	Firmicutes	Clostridia	Clostridiales	Lachnospiraceae	Unknown	
ASV_454	Bacteria	Firmicutes	Clostridia	Clostridiales	Ruminococcaceae	Ruminiclostridium_9	
ASV_455	Bacteria	Firmicutes	Clostridia	Clostridiales	Ruminococcaceae	Ruminococcaceae_UCG-014	
ASV_456	Bacteria	Firmicutes	Clostridia	Clostridiales	Lachnospiraceae	Unknown	
ASV_457	Bacteria	Firmicutes	Clostridia	Clostridiales	Clostridiales_vadinBB60_group	Unknown	
ASV_458	Bacteria	Firmicutes	Clostridia	Clostridiales	Ruminococcaceae	Ruminiclostridium_9	
ASV_459	Bacteria	Firmicutes	Clostridia	Clostridiales	Lachnospiraceae	Unknown	
ASV_460	Bacteria	Firmicutes	Clostridia	Clostridiales	Ruminococcaceae	Unknown	
ASV_461	Bacteria	Firmicutes	Bacilli	Bacillales	Staphylococcaceae	Staphylococcus	
ASV_462	Bacteria	Proteobacteria	Gammaproteobacteria	Enterobacteriales	Enterobacteriaceae	Escherichia/Shigella	Escherichia/Shigella_coli
ASV_463	Bacteria	Firmicutes	Clostridia	Clostridiales	Ruminococcaceae	Oscillibacter	
ASV_464	Bacteria	Firmicutes	Bacilli	Lactobacillales	Lactobacillaceae	Lactobacillus	
ASV_465	Bacteria	Firmicutes	Bacilli	Lactobacillales	Lactobacillaceae	Lactobacillus	
ASV_466	Bacteria	Firmicutes	Clostridia	Clostridiales	Ruminococcaceae	Unknown	

ASV_467	Bacteria	Firmicutes	Clostridia	Clostridiales	Lachnospiraceae	Unknown	
ASV_468	Bacteria	Bacteroidetes	Bacteroidia	Bacteroidales	Bacteroidaceae	Bacteroides	Bacteroides_dorei
ASV_469	Bacteria	Firmicutes	Clostridia	Clostridiales	Lachnospiraceae	Lachnospiraceae_UCG-001	
ASV_470	Bacteria	Firmicutes	Bacilli	Bacillales	Staphylococcaceae	Staphylococcus	
ASV_471	Bacteria	Firmicutes	Clostridia	Clostridiales	Lachnospiraceae	A2	
ASV_472	Bacteria	Firmicutes	Clostridia	Clostridiales	Lachnospiraceae	Lachnospiraceae_NK4A136_group	
ASV_473	Bacteria	Bacteroidetes	Bacteroidia	Bacteroidales	Muribaculaceae	Unknown	
ASV_474	Bacteria	Firmicutes	Clostridia	Clostridiales	Lachnospiraceae	Lachnospiraceae_NK4A136_group	
ASV_475	Bacteria	Bacteroidetes	Bacteroidia	Bacteroidales	Muribaculaceae	Unknown	
ASV_476	Bacteria	Firmicutes	Clostridia	Clostridiales	Lachnospiraceae	Lachnospiraceae_NK4A136_group	
ASV_477	Bacteria	Firmicutes	Clostridia	Clostridiales	Lachnospiraceae	Unknown	
ASV_478	Bacteria	Actinobacteria	Coriobacteriia	Coriobacteriales	Eggerthellaceae	Unknown	
ASV_479	Bacteria	Actinobacteria	Coriobacteriia	Coriobacteriales	Eggerthellaceae	Unknown	
ASV_480	Bacteria	Firmicutes	Clostridia	Clostridiales	Ruminococcaceae	Ruminococcaceae_UCG-014	
ASV_481	Bacteria	Firmicutes	Clostridia	Clostridiales	Clostridiales_vadinBB60_group	Unknown	
ASV_482	Bacteria	Firmicutes	Clostridia	Clostridiales	Ruminococcaceae	Ruminiclostridium_9	
ASV_483	Bacteria	Firmicutes	Clostridia	Clostridiales	Lachnospiraceae	Unknown	
ASV_484	Bacteria	Firmicutes	Clostridia	Clostridiales	Ruminococcaceae	Unknown	
ASV_485	Bacteria	Proteobacteria	Gammaproteobacteria	Betaproteobacteriales	Burkholderiaceae	Sutterella	Sutterella_wadsworthensis
ASV_486	Bacteria	Firmicutes	Clostridia	Clostridiales	Ruminococcaceae	Ruminococcaceae_UCG-014	
ASV_487	Bacteria	Firmicutes	Clostridia	Clostridiales	Ruminococcaceae	Ruminococcaceae_UCG-014	
ASV_488	Bacteria	Firmicutes	Clostridia	Clostridiales	Ruminococcaceae	Ruminococcaceae_NK4A214_group	
ASV_489	Bacteria	Firmicutes	Clostridia	Clostridiales	Lachnospiraceae	Unknown	
ASV_490	Bacteria	Firmicutes	Clostridia	Clostridiales	Ruminococcaceae	Ruminococcaceae_UCG-010	
ASV_491	Bacteria	Firmicutes	Clostridia	Clostridiales	Lachnospiraceae	Lachnospiraceae_NK4A136_group	
ASV_492	Bacteria	Firmicutes	Clostridia	Clostridiales	Lachnospiraceae	Lachnospiraceae_UCG-001	
ASV_493	Bacteria	Firmicutes	Clostridia	Clostridiales	Ruminococcaceae	Ruminococcaceae_UCG-013	
ASV_494	Bacteria	Firmicutes	Clostridia	Clostridiales	Ruminococcaceae	Unknown	
ASV_495	Bacteria	Firmicutes	Clostridia	Clostridiales	Ruminococcaceae	Faecalibacterium	
ASV_496	Bacteria	Firmicutes	Clostridia	Clostridiales	Lachnospiraceae	Unknown	
ASV_497	Bacteria	Firmicutes	Clostridia	Clostridiales	Ruminococcaceae	Ruminiclostridium	
ASV_498	Bacteria	Firmicutes	Clostridia	Clostridiales	Lachnospiraceae	Lachnospiraceae_NK4A136_group	
ASV_499	Bacteria	Firmicutes	Clostridia	Clostridiales	Lachnospiraceae	Unknown	
ASV_500	Bacteria	Firmicutes	Clostridia	Clostridiales	Ruminococcaceae	Oscillibacter	
ASV_501	Bacteria	Firmicutes	Clostridia	Clostridiales	Lachnospiraceae	Unknown	
ASV_502	Bacteria	Firmicutes	Clostridia	Clostridiales	Ruminococcaceae	Unknown	

ASV_503	Bacteria	Firmicutes	Clostridia	Clostridiales	Ruminococcaceae	Ruminococcaceae_UCG-014	
ASV_504	Bacteria	Bacteroidetes	Bacteroidia	Bacteroidales	Muribaculaceae	Unknown	
ASV_505	Bacteria	Firmicutes	Clostridia	Clostridiales	Lachnospiraceae	Lachnospiraceae_NK4A136_group	
ASV_506	Bacteria	Tenericutes	Mollicutes	Mollicutes_RF39	Unknown		
ASV_507	Bacteria	Firmicutes	Clostridia	Clostridiales	Ruminococcaceae	Faecalibacterium	
ASV_508	Bacteria	Firmicutes	Clostridia	Clostridiales	Ruminococcaceae	Ruminococcaceae_UCG-010	
ASV_509	Bacteria	Bacteroidetes	Bacteroidia	Bacteroidales	Prevotellaceae	Prevotella_9	
ASV_510	Bacteria	Firmicutes	Clostridia	Clostridiales	Ruminococcaceae	Ruminiclostridium_9	
ASV_511	Bacteria	Firmicutes	Clostridia	Clostridiales	Family_XIII	Family_XIII_AD3011_group	
ASV_512	Bacteria	Firmicutes	Clostridia	Clostridiales	Lachnospiraceae	Unknown	
ASV_513	Bacteria	Firmicutes	Clostridia	Clostridiales	Lachnospiraceae	Lachnospiraceae_NK4A136_group	
ASV_514	Bacteria	Firmicutes	Clostridia	Clostridiales	Ruminococcaceae	Ruminococcaceae_UCG-014	
ASV_515	Bacteria	Firmicutes	Clostridia	Clostridiales	Ruminococcaceae	Ruminococcaceae_UCG-014	
ASV_516	Bacteria	Firmicutes	Clostridia	Clostridiales	Ruminococcaceae	Ruminococcus_1	
ASV_517	Bacteria	Firmicutes	Clostridia	Clostridiales	Lachnospiraceae	Lachnospiraceae_NK4A136_group	
ASV_518	Bacteria	Firmicutes	Clostridia	Clostridiales	Ruminococcaceae	Ruminiclostridium_5	
ASV_519	Bacteria	Firmicutes	Clostridia	Clostridiales	Lachnospiraceae	Unknown	
ASV_520	Bacteria	Firmicutes	Clostridia	Clostridiales	Ruminococcaceae	Unknown	
ASV_521	Bacteria	Firmicutes	Clostridia	Clostridiales	Lachnospiraceae	Lachnospiraceae_NK4A136_group	
ASV_522	Bacteria	Firmicutes	Clostridia	Clostridiales	Lachnospiraceae	Unknown	
ASV_523	Bacteria	Firmicutes	Clostridia	Clostridiales	Lachnospiraceae	Unknown	
ASV_524	Bacteria	Firmicutes	Clostridia	Clostridiales	Lachnospiraceae	Lachnospiraceae_NK4A136_group	
ASV_525	Bacteria	Bacteroidetes	Bacteroidia	Bacteroidales	Bacteroidaceae	Bacteroides	
ASV_526	Bacteria	Proteobacteria	Gammaproteobacteria	Pasteurellales	Pasteurellaceae	Unknown	
ASV_527	Bacteria	Firmicutes	Bacilli	Lactobacillales	Carnobacteriaceae	Granulicatella	
ASV_528	Bacteria	Firmicutes	Clostridia	Clostridiales	Lachnospiraceae	Roseburia	
ASV_529	Bacteria	Firmicutes	Clostridia	Clostridiales	Lachnospiraceae	Unknown	
ASV_530	Bacteria	Firmicutes	Clostridia	Clostridiales	Clostridiales_vadinBB60_group	Unknown	
ASV_531	Bacteria	Firmicutes	Clostridia	Clostridiales	Ruminococcaceae	Ruminococcaceae_UCG-014	
ASV_532	Bacteria	Firmicutes	Clostridia	Clostridiales	Lachnospiraceae	ASF356	
ASV_533	Bacteria	Bacteroidetes	Bacteroidia	Bacteroidales	Muribaculaceae	Unknown	
ASV_534	Bacteria	Actinobacteria	Actinobacteria	Micrococcales	Microbacteriaceae	Pseudoclavibacter	Pseudoclavibacter_helvolus
ASV_535	Bacteria	Proteobacteria	Gammaproteobacteria	Pasteurellales	Pasteurellaceae	Haemophilus	
ASV_536	Bacteria	Firmicutes	Clostridia	Clostridiales	Lachnospiraceae	Unknown	
ASV_537	Bacteria	Firmicutes	Clostridia	Clostridiales	Clostridiales_vadinBB60_group	Unknown	
ASV_538	Bacteria	Firmicutes	Erysipelotrichia	Erysipelotrichales	Erysipelotrichaceae	Unknown	
ASV_539	Bacteria	Firmicutes	Clostridia	Clostridiales	Lachnospiraceae	Lachnospiraceae_UCG-006	

ASV_540	Bacteria	Firmicutes	Clostridia	Clostridiales	Ruminococcaceae	Ruminococcaceae_UCG-014	
ASV_541	Bacteria	Firmicutes	Clostridia	Clostridiales	Lachnospiraceae	Unknown	
ASV_542	Bacteria	Firmicutes	Erysipelotrichia	Erysipelotrichales	Erysipelotrichaceae	Unknown	
ASV_543	Bacteria	Firmicutes	Clostridia	Clostridiales	Lachnospiraceae	Lachnospiraceae_UCG-006	
ASV_544	Bacteria	Firmicutes	Clostridia	Clostridiales	Lachnospiraceae	Roseburia	
ASV_545	Bacteria	Bacteroidetes	Bacteroidia	Bacteroidales	Tannerellaceae	Parabacteroides	Parabacteroides_distasonis
ASV_546	Bacteria	Firmicutes	Clostridia	Clostridiales	Lachnospiraceae	Unknown	
ASV_547	Bacteria	Firmicutes	Clostridia	Clostridiales	Ruminococcaceae	Ruminiclostridium	
ASV_548	Bacteria	Firmicutes	Clostridia	Clostridiales	Ruminococcaceae	Ruminococcaceae_UCG-014	
ASV_549	Bacteria	Firmicutes	Bacilli	Lactobacillales	Aerococcaceae	Aerococcus	
ASV_550	Bacteria	Firmicutes	Clostridia	Clostridiales	Lachnospiraceae	Unknown	
ASV_551	Bacteria	Firmicutes	Clostridia	Clostridiales	Lachnospiraceae	Unknown	
ASV_552	Bacteria	Firmicutes	Clostridia	Clostridiales	Clostridiales_vadinBB60_group	Unknown	
ASV_553	Bacteria	Firmicutes	Clostridia	Clostridiales	Ruminococcaceae	Oscillibacter	
ASV_554	Bacteria	Firmicutes	Clostridia	Clostridiales	Lachnospiraceae	Unknown	
ASV_555	Bacteria	Firmicutes	Clostridia	Clostridiales	Lachnospiraceae	Lachnoclostridium	
ASV_556	Bacteria	Firmicutes	Clostridia	Clostridiales	Lachnospiraceae	Anaerostipes	Anaerostipes_hadrus
ASV_557	Bacteria	Firmicutes	Clostridia	Clostridiales	Ruminococcaceae	Unknown	
ASV_558	Bacteria	Firmicutes	Clostridia	Clostridiales	Lachnospiraceae	A2	
ASV_559	Bacteria	Firmicutes	Clostridia	Clostridiales	Lachnospiraceae	Lachnospiraceae_NK4A136_group	
ASV_560	Bacteria	Firmicutes	Clostridia	Clostridiales	Lachnospiraceae	Lachnospiraceae_UCG-001	
ASV_561	Bacteria	Firmicutes	Clostridia	Clostridiales	Lachnospiraceae	Lachnospiraceae_NK4A136_group	
ASV_562	Bacteria	Firmicutes	Clostridia	Clostridiales	Ruminococcaceae	Ruminococcaceae_UCG-014	
ASV_563	Bacteria	Firmicutes	Clostridia	Clostridiales	Ruminococcaceae	Ruminiclostridium_9	
ASV_564	Bacteria	Proteobacteria	Gammaproteobacteria	Pasteurellales	Pasteurellaceae	Unknown	
ASV_565	Bacteria	Firmicutes	Clostridia	Clostridiales	Ruminococcaceae	Subdoligranulum	
ASV_566	Bacteria	Firmicutes	Clostridia		Unknown		
ASV_567	Bacteria	Firmicutes	Clostridia	Clostridiales	Lachnospiraceae	Unknown	
ASV_568	Bacteria	Tenericutes	Mollicutes	Mollicutes_RF39	Unknown		
ASV_569	Bacteria	Firmicutes	Clostridia	Clostridiales	Lachnospiraceae	Lachnospiraceae_NK4A136_group	
ASV_570	Bacteria	Bacteroidetes	Bacteroidia	Bacteroidales	Muribaculaceae	Unknown	
ASV_571	Bacteria	Firmicutes	Clostridia	Clostridiales	Clostridiales_vadinBB60_group	Unknown	
ASV_572	Bacteria	Firmicutes	Clostridia	Clostridiales	Ruminococcaceae	Ruminiclostridium_5	
ASV_573	Bacteria	Firmicutes	Clostridia	Clostridiales	Ruminococcaceae	Ruminococcaceae_UCG-005	
ASV_574	Bacteria	Firmicutes	Erysipelotrichia	Erysipelotrichales	Erysipelotrichaceae	Faecalibaculum	
ASV_575	Bacteria	Firmicutes	Clostridia	Clostridiales	Ruminococcaceae	Unknown	
ASV_576	Bacteria	Firmicutes	Clostridia	Clostridiales	Lachnospiraceae	Lachnospiraceae_UCG-006	

ASV_577	Bacteria	Firmicutes	Clostridia	Clostridiales	Ruminococcaceae	Ruminiclostridium	
ASV_578	Bacteria	Firmicutes	Clostridia	Clostridiales	Lachnospiraceae	Lachnoclostridium	
ASV_579	Bacteria	Firmicutes	Clostridia	Clostridiales	Lachnospiraceae	Lachnospiraceae_NK4A136_group	
ASV_580	Bacteria	Bacteroidetes	Bacteroidia	Bacteroidales	Muribaculaceae	Unknown	
ASV_581	Bacteria	Firmicutes	Clostridia	Clostridiales	Lachnospiraceae	Roseburia	
ASV_582	Bacteria	Firmicutes	Clostridia	Clostridiales	Ruminococcaceae	Ruminococcaceae_UCG-014	
ASV_583	Bacteria	Firmicutes		Unknown			
ASV_584	Bacteria	Firmicutes	Clostridia	Clostridiales	Ruminococcaceae	Unknown	
ASV_585	Bacteria	Firmicutes	Clostridia	Clostridiales	Lachnospiraceae	Lachnospiraceae_NK4A136_group	
ASV_586	Bacteria	Bacteroidetes	Bacteroidia	Bacteroidales	Muribaculaceae	Unknown	
ASV_587	Bacteria	Firmicutes	Clostridia	Clostridiales	Ruminococcaceae	Ruminococcaceae_UCG-014	
ASV_588	Bacteria	Actinobacteria	Coriobacteriia	Coriobacteriales	Eggerthellaceae	Adlercreutzia	
ASV_589	Bacteria	Tenericutes	Mollicutes	Mollicutes_RF39	Unknown		
ASV_590	Bacteria	Firmicutes	Clostridia	Clostridiales	Lachnospiraceae	Unknown	
ASV_591	Bacteria	Firmicutes	Clostridia	Clostridiales	Clostridiales_vadinBB60_group	Unknown	
ASV_592	Bacteria	Proteobacteria	Alphaproteobacteria	Rhodospirillales	Unknown		
ASV_593	Bacteria	Firmicutes	Clostridia	Clostridiales	Lachnospiraceae	Blautia	
ASV_594	Bacteria	Firmicutes	Clostridia	Clostridiales	Lachnospiraceae	Lachnospiraceae_NK4A136_group	
ASV_595	Bacteria	Firmicutes	Clostridia	Clostridiales	Lachnospiraceae	Unknown	
ASV_596	Bacteria	Firmicutes	Clostridia	Clostridiales	Ruminococcaceae	Unknown	
ASV_597	Bacteria	Firmicutes	Clostridia	Clostridiales	Lachnospiraceae	Lachnospiraceae_UCG-001	
ASV_598	Bacteria	Firmicutes	Clostridia	Clostridiales	Clostridiales_vadinBB60_group	Unknown	
ASV_599	Bacteria	Firmicutes	Clostridia	Clostridiales	Ruminococcaceae	Butyricicoccus	
ASV_600	Bacteria	Firmicutes	Clostridia	Clostridiales	Ruminococcaceae	Ruminococcaceae_UCG-014	
ASV_601	Bacteria	Firmicutes	Clostridia	Clostridiales	Lachnospiraceae	Unknown	
ASV_602	Bacteria	Firmicutes	Clostridia	Clostridiales	Ruminococcaceae	Ruminococcaceae_UCG-009	
ASV_603	Bacteria	Firmicutes	Clostridia	Clostridiales	Lachnospiraceae	Unknown	
ASV_604	Bacteria	Firmicutes	Erysipelotrichia	Erysipelotrichales	Erysipelotrichaceae	Erysipelatoclostridium	
ASV_605	Bacteria	Bacteroidetes	Bacteroidia	Bacteroidales	Bacteroidaceae	Bacteroides	Bacteroides_massiliensis
ASV_606	Bacteria	Proteobacteria	Gammaproteobacteria	Pasteurellales	Pasteurellaceae	Aggregatibacter	Aggregatibacter_segnis
ASV_607	Bacteria	Fusobacteria	Fusobacteriia	Fusobacteriales	Leptotrichiaceae	Leptotrichia	
ASV_608	Bacteria	Firmicutes	Clostridia	Clostridiales	Ruminococcaceae	Ruminococcaceae_UCG-005	
ASV_609	Bacteria	Firmicutes	Erysipelotrichia	Erysipelotrichales	Erysipelotrichaceae	Unknown	
ASV_610	Bacteria	Firmicutes	Clostridia	Clostridiales	Ruminococcaceae	Ruminococcaceae_UCG-014	
ASV_611	Bacteria	Firmicutes	Clostridia	Clostridiales	Ruminococcaceae	Ruminococcaceae_UCG-014	
ASV_612	Bacteria	Firmicutes	Clostridia	Clostridiales	Lachnospiraceae	A2	
ASV_613	Bacteria	Firmicutes	Clostridia	Clostridiales	Lachnospiraceae	Unknown	

ASV_614	Bacteria	Firmicutes	Clostridia	Clostridiales	Lachnospiraceae	Unknown	
ASV_615	Bacteria	Firmicutes	Clostridia	Clostridiales	Lachnospiraceae	Lachnospiraceae_NK4A136_group	
ASV_616	Bacteria	Firmicutes	Clostridia	Clostridiales	Ruminococcaceae	Unknown	
ASV_617	Bacteria	Firmicutes	Clostridia	Clostridiales	Ruminococcaceae	Butyricicoccus	
ASV_618	Bacteria	Firmicutes	Clostridia	Clostridiales	Ruminococcaceae	Ruminiclostridium	
ASV_619	Bacteria	Firmicutes	Clostridia	Clostridiales	Lachnospiraceae	Unknown	
ASV_620	Bacteria	Firmicutes	Clostridia	Clostridiales	Ruminococcaceae	Ruminococcaceae_UCG-014	
ASV_621	Bacteria	Firmicutes	Clostridia	Clostridiales	Ruminococcaceae	Ruminiclostridium_9	
ASV_622	Bacteria	Bacteroidetes	Bacteroidia	Bacteroidales	Prevotellaceae	Prevotella_7	
ASV_623	Bacteria	Firmicutes	Clostridia	Clostridiales	Ruminococcaceae	Unknown	
ASV_624	Bacteria	Bacteroidetes	Bacteroidia	Bacteroidales	Muribaculaceae	Unknown	
ASV_625	Bacteria	Firmicutes	Clostridia	Clostridiales	Lachnospiraceae	Unknown	
ASV_626	Bacteria	Firmicutes	Clostridia	Clostridiales	Lachnospiraceae	Unknown	
ASV_627	Bacteria	Tenericutes	Mollicutes	Mollicutes_RF39	Unknown		
ASV_628	Bacteria	Firmicutes	Clostridia	Clostridiales	Ruminococcaceae	Ruminiclostridium_9	
ASV_629	Bacteria	Tenericutes	Mollicutes	Izimaplasmatales	Unknown		
ASV_630	Bacteria	Firmicutes	Clostridia	Clostridiales	Lachnospiraceae	Unknown	
ASV_631	Bacteria	Firmicutes	Clostridia	Clostridiales	Ruminococcaceae	Ruminococcaceae_UCG-014	
ASV_632	Bacteria	Firmicutes	Clostridia	Clostridiales	Ruminococcaceae	Ruminiclostridium_9	
ASV_633	Bacteria	Firmicutes	Clostridia	Clostridiales	Lachnospiraceae	Unknown	
ASV_634	Bacteria	Firmicutes	Bacilli	Bacillales	Staphylococcaceae	Staphylococcus	
ASV_635	Bacteria	Firmicutes	Clostridia	Clostridiales	Ruminococcaceae	Ruminiclostridium_9	
ASV_636	Bacteria	Firmicutes	Clostridia	Clostridiales	Lachnospiraceae	Lachnospiraceae_NK4A136_group	
ASV_637	Bacteria	Firmicutes	Clostridia	Clostridiales	Lachnospiraceae	Unknown	
ASV_638	Bacteria	Verrucomicrobia	Verrucomicrobiae	Verrucomicrobiales	Akkermansiaceae	Akkermansia	Akkermansia_muciniphila
ASV_639	Bacteria	Firmicutes	Clostridia	Clostridiales	Christensenellaceae	Christensenellaceae_R-7_group	
ASV_640	Bacteria	Firmicutes	Clostridia	Clostridiales	Lachnospiraceae	Lachnospiraceae_UCG-006	
ASV_641	Bacteria	Firmicutes	Clostridia	Clostridiales	Lachnospiraceae	Unknown	
ASV_642	Bacteria	Firmicutes	Clostridia	Clostridiales	Christensenellaceae	Christensenellaceae_R-7_group	
ASV_643	Bacteria	Tenericutes	Mollicutes	Mollicutes_RF39	Unknown		
ASV_644	Bacteria	Firmicutes	Clostridia	Clostridiales	Lachnospiraceae	Lachnospiraceae_NK4A136_group	
ASV_645	Bacteria	Firmicutes	Clostridia	Clostridiales	Lachnospiraceae	Unknown	
ASV_646	Bacteria	Firmicutes	Clostridia	Clostridiales	Ruminococcaceae	Ruminococcaceae_UCG-014	
ASV_647	Bacteria	Firmicutes	Clostridia	Clostridiales	Lachnospiraceae	Unknown	
ASV_648	Bacteria	Firmicutes	Clostridia	Clostridiales	Ruminococcaceae	Ruminococcus_1	
ASV_649	Bacteria	Firmicutes	Clostridia	Clostridiales	Ruminococcaceae	Ruminococcaceae_UCG-014	

ASV_650	Bacteria	Firmicutes	Clostridia	Clostridiales	Lachnospiraceae	Unknown
ASV_651	Bacteria	Firmicutes	Clostridia	Clostridiales	Lachnospiraceae	Roseburia
ASV_652	Bacteria	Firmicutes	Clostridia	Clostridiales	Ruminococcaceae	Ruminococcaceae_UCG-014
ASV_653	Bacteria	Firmicutes	Clostridia	Clostridiales	Lachnospiraceae	Unknown
ASV_654	Bacteria	Firmicutes	Clostridia	Clostridiales	Lachnospiraceae	Unknown
ASV_655	Bacteria	Firmicutes	Clostridia	Clostridiales	Lachnospiraceae	Unknown
ASV_656	Bacteria	Firmicutes	Clostridia	Clostridiales	Ruminococcaceae	Unknown
ASV_657	Bacteria	Firmicutes	Clostridia	Clostridiales	Ruminococcaceae	Unknown
ASV_658	Bacteria	Firmicutes	Clostridia	Clostridiales	Lachnospiraceae	Unknown
ASV_659	Bacteria	Firmicutes	Clostridia	Clostridiales	Lachnospiraceae	Unknown
ASV_660	Bacteria	Firmicutes	Clostridia	Clostridiales	Lachnospiraceae	Unknown
ASV_661	Bacteria	Firmicutes	Clostridia	Clostridiales	Lachnospiraceae	Unknown
ASV_662	Bacteria	Firmicutes	Clostridia	Clostridiales	Lachnospiraceae	Unknown
ASV_663	Bacteria	Firmicutes	Clostridia	Clostridiales	Ruminococcaceae	Ruminococcaceae_UCG-005
ASV_664	Bacteria	Firmicutes	Clostridia	Clostridiales	Lachnospiraceae	Unknown
ASV_665	Bacteria	Firmicutes	Clostridia	Clostridiales	Ruminococcaceae	Ruminococcaceae_UCG-014
ASV_666	Bacteria	Firmicutes	Clostridia	Clostridiales	Lachnospiraceae	Roseburia
ASV_667	Bacteria	Firmicutes	Clostridia	Clostridiales	Lachnospiraceae	Roseburia
ASV_668	Bacteria	Bacteroidetes	Bacteroidia	Bacteroidales	Muribaculaceae	Unknown
ASV_669	Bacteria	Actinobacteria	Actinobacteria	Corynebacteriales	Corynebacteriaceae	Corynebacterium_1
ASV_670	Bacteria	Firmicutes	Clostridia	Clostridiales	Lachnospiraceae	Lachnospiraceae_NK4A136_group
ASV_671	Bacteria	Firmicutes	Clostridia	Clostridiales	Ruminococcaceae	Ruminococcaceae_UCG-014
ASV_672	Bacteria	Firmicutes	Clostridia	Clostridiales	Lachnospiraceae	Lachnospiraceae_NK4A136_group
ASV_673	Bacteria	Firmicutes	Clostridia	Clostridiales	Lachnospiraceae	Unknown
ASV_674	Bacteria	Firmicutes	Clostridia	Clostridiales	Lachnospiraceae	Unknown
ASV_675	Bacteria	Firmicutes	Clostridia	Clostridiales	Ruminococcaceae	Oscillospira
ASV_676	Bacteria	Firmicutes	Clostridia	Clostridiales	Lachnospiraceae	Agathobacter
ASV_677	Bacteria	Bacteroidetes	Bacteroidia	Bacteroidales	Muribaculaceae	Unknown
ASV_678	Bacteria	Bacteroidetes	Bacteroidia	Bacteroidales	Muribaculaceae	Unknown
ASV_679	Bacteria	Firmicutes	Clostridia	Clostridiales	Lachnospiraceae	Unknown
ASV_680	Bacteria	Firmicutes	Clostridia	Clostridiales	Ruminococcaceae	Ruminococcaceae_UCG-014
ASV_681	Bacteria	Firmicutes	Clostridia	Clostridiales	Ruminococcaceae	Ruminococcaceae_UCG-014
ASV_682	Bacteria	Firmicutes	Clostridia	Clostridiales	Lachnospiraceae	Unknown
ASV_683	Bacteria	Firmicutes	Clostridia	Clostridiales	Lachnospiraceae	Unknown
ASV_684	Bacteria	Firmicutes	Clostridia	Clostridiales	Lachnospiraceae	Unknown
ASV_685	Bacteria	Firmicutes	Clostridia	Clostridiales	Ruminococcaceae	Ruminiclostridium
ASV_686	Bacteria	Firmicutes	Clostridia	Clostridiales	Lachnospiraceae	Lachnoclostridium

ASV_687	Bacteria	Bacteroidetes	Bacteroidia	Bacteroidales	Prevotellaceae	Prevotella_9		
ASV_688	Bacteria	Firmicutes	Clostridia	Clostridiales	Ruminococcaceae	Unknown		
ASV_689	Bacteria	Firmicutes	Clostridia	Clostridiales	Ruminococcaceae	Ruminococcaceae_UCG-014		
ASV_690	Bacteria	Firmicutes	Clostridia	Clostridiales	Lachnospiraceae	Unknown		
ASV_691	Bacteria	Firmicutes	Clostridia	Clostridiales	Lachnospiraceae	Lachnospiraceae_NK4A136_group		
ASV_692	Bacteria	Firmicutes	Clostridia	Clostridiales	Ruminococcaceae	Ruminococcaceae_UCG-014		
ASV_693	Bacteria	Firmicutes	Clostridia	Clostridiales	Ruminococcaceae	Unknown		
ASV_694	Bacteria	Firmicutes	Clostridia	Clostridiales	Ruminococcaceae	Ruminiclostridium_5		
ASV_695	Bacteria	Patescibacteria	Saccharimonadia	Saccharimonadales	Saccharimonadaceae	Unknown		
ASV_696	Bacteria	Tenericutes	Mollicutes	Mollicutes_RF39	Unknown			
ASV_697	Bacteria	Firmicutes	Clostridia	Clostridiales	Ruminococcaceae	Oscillibacter		
ASV_698	Bacteria	Firmicutes	Clostridia	Clostridiales	Ruminococcaceae	Ruminococcaceae_UCG-014		
ASV_699	Bacteria	Firmicutes	Clostridia	Clostridiales	Lachnospiraceae	Unknown		
ASV_700	Bacteria	Firmicutes	Clostridia	Clostridiales	Lachnospiraceae	Lachnospiraceae_NK4A136_group		
ASV_701	Bacteria	Firmicutes	Clostridia	Clostridiales	Lachnospiraceae	Unknown		
ASV_702	Bacteria	Firmicutes	Clostridia	Clostridiales	Lachnospiraceae	Unknown		
ASV_703	Bacteria	Firmicutes	Clostridia	Clostridiales	Lachnospiraceae	Unknown		
ASV_704	Bacteria	Tenericutes	Mollicutes	Mollicutes_RF39	Unknown			
ASV_705	Bacteria	Firmicutes	Clostridia	Clostridiales	Lachnospiraceae	Unknown		
ASV_706	Bacteria	Firmicutes	Clostridia	Clostridiales	Lachnospiraceae	Roseburia		
ASV_707	Bacteria	Firmicutes	Clostridia	Clostridiales	Lachnospiraceae	Lachnoclostridium		
ASV_708	Bacteria	Firmicutes	Clostridia	Clostridiales	Lachnospiraceae	Lachnospiraceae_UCG-001		
ASV_709	Bacteria	Bacteroidetes	Bacteroidia	Bacteroidales	Bacteroidaceae	Bacteroides		
ASV_710	Bacteria	Firmicutes	Clostridia	Clostridiales	Lachnospiraceae	Unknown		
ASV_711	Bacteria	Firmicutes	Clostridia	Clostridiales	Ruminococcaceae	Ruminiclostridium_9		
ASV_712	Bacteria	Firmicutes	Clostridia	Clostridiales	Ruminococcaceae	Oscillibacter		
ASV_713	Bacteria	Firmicutes	Clostridia	Clostridiales	Ruminococcaceae	Ruminiclostridium_9		
ASV_714	Bacteria	Proteobacteria	Deltaproteobacteria	Desulfovibrionales	Desulfovibrionaceae	Bilophila	Bilophila_wadsworthia	
ASV_715	Bacteria	Firmicutes	Clostridia	Clostridiales	Ruminococcaceae	Ruminococcus_1		
ASV_716	Bacteria	Firmicutes	Clostridia	Clostridiales	Ruminococcaceae	Oscillibacter		
ASV_717	Bacteria	Proteobacteria	Gammaproteobacteria	Enterobacteriales	Enterobacteriaceae	Escherichia/Shigella	Escherichia/Shigella_flexneri	
ASV_718	Bacteria	Firmicutes	Clostridia	Clostridiales	Lachnospiraceae	Unknown		
ASV_719	Bacteria	Firmicutes	Clostridia	Clostridiales	Ruminococcaceae	Ruminiclostridium_9		
ASV_720	Bacteria	Firmicutes	Clostridia	Clostridiales	Ruminococcaceae	Ruminiclostridium_9		
ASV_721	Bacteria	Firmicutes	Clostridia	Clostridiales	Lachnospiraceae	ASF356		
ASV_722	Bacteria	Firmicutes	Clostridia	Clostridiales	Ruminococcaceae	Ruminiclostridium_5		

ASV_723	Bacteria	Tenericutes	Mollicutes	Mollicutes_RF39	Unknown		
ASV_724	Bacteria	Bacteroidetes	Bacteroidia	Bacteroidales	Prevotellaceae	Prevotella_9	
ASV_725	Bacteria	Firmicutes	Clostridia	Clostridiales	Lachnospiraceae	Lachnospiraceae_UCG-001	
ASV_726	Bacteria	Firmicutes	Clostridia	Clostridiales	Lachnospiraceae	Unknown	
ASV_727	Bacteria	Bacteroidetes	Bacteroidia	Bacteroidales	Prevotellaceae	Prevotella_6	
ASV_728	Bacteria	Firmicutes	Clostridia	Clostridiales	Ruminococcaceae	Oscillibacter	
ASV_729	Bacteria	Firmicutes	Clostridia	Clostridiales	Lachnospiraceae	Unknown	
ASV_730	Bacteria	Firmicutes	Clostridia	Clostridiales	Lachnospiraceae	Lachnospiraceae_NK4A136_group	
ASV_731	Bacteria	Firmicutes	Clostridia	Clostridiales	Ruminococcaceae	Subdoligranulum	
ASV_732	Bacteria	Firmicutes	Clostridia	Clostridiales	Lachnospiraceae	Lachnospiraceae_NK4A136_group	
ASV_733	Bacteria	Firmicutes	Clostridia	Clostridiales	Ruminococcaceae	Unknown	
ASV_734	Bacteria	Firmicutes	Erysipelotrichia	Erysipelotrichales	Erysipelotrichaceae	Unknown	
ASV_735	Bacteria	Firmicutes	Clostridia	Clostridiales	Lachnospiraceae	Lachnospiraceae_UCG-001	
ASV_736	Bacteria	Firmicutes	Clostridia	Clostridiales	Family_XIII	Family_XIII_UCG-001	
ASV_737	Bacteria	Firmicutes	Clostridia	Clostridiales	Ruminococcaceae	Butyricicoccus	
ASV_738	Bacteria	Bacteroidetes	Bacteroidia	Bacteroidales	Rikenellaceae	Alistipes	Alistipes_finegoldii
ASV_739	Bacteria	Bacteroidetes	Bacteroidia	Bacteroidales	Bacteroidaceae	Bacteroides	Bacteroides_uniformis
ASV_740	Bacteria	Firmicutes	Clostridia	Clostridiales	Lachnospiraceae	Unknown	
ASV_741	Bacteria	Firmicutes	Clostridia	Clostridiales	Ruminococcaceae	Ruminococcaceae_UCG-005	
ASV_742	Bacteria	Firmicutes	Clostridia	Clostridiales	Ruminococcaceae	Ruminococcaceae_UCG-014	
ASV_743	Bacteria	Firmicutes	Clostridia	Clostridiales	Ruminococcaceae	Ruminiclostridium_9	
ASV_744	Bacteria	Firmicutes	Clostridia	Clostridiales	Ruminococcaceae	Oscillibacter	
ASV_745	Bacteria	Firmicutes	Clostridia	Clostridiales	Ruminococcaceae	Oscillibacter	
ASV_746	Bacteria	Tenericutes	Mollicutes	Mollicutes_RF39	Unknown		
ASV_747	Bacteria	Actinobacteria	Coriobacteriia	Coriobacteriales	Eggerthellaceae	Enterorhabdus	
ASV_748	Bacteria	Firmicutes	Clostridia	Clostridiales	Lachnospiraceae	ASF356	
ASV_749	Bacteria	Bacteroidetes	Bacteroidia	Bacteroidales	Prevotellaceae	Prevotella_9	
ASV_750	Bacteria	Firmicutes	Clostridia	Clostridiales	Lachnospiraceae	Unknown	
ASV_751	Bacteria	Firmicutes	Clostridia	Clostridiales	Lachnospiraceae	Unknown	
ASV_752	Bacteria	Firmicutes	Clostridia	Clostridiales	Ruminococcaceae	Ruminiclostridium	
ASV_753	Bacteria	Firmicutes	Clostridia	Clostridiales	Ruminococcaceae	Ruminococcaceae_UCG-014	
ASV_754	Bacteria	Firmicutes	Clostridia	Clostridiales	Ruminococcaceae	Ruminococcus_2	
ASV_755	Bacteria	Firmicutes	Clostridia	Clostridiales	Lachnospiraceae	Lachnospiraceae_NK4A136_group	
ASV_756	Bacteria	Firmicutes	Clostridia	Clostridiales	Lachnospiraceae	Unknown	
ASV_757	Bacteria	Firmicutes	Clostridia	Clostridiales	Ruminococcaceae	Ruminococcaceae_UCG-014	
ASV_758	Bacteria	Proteobacteria	Gammaproteobacteria	Betaproteobacteriales	Burkholderiaceae	Pelomonas	
ASV_759	Bacteria	Proteobacteria	Alphaproteobacteria	Rhodobacterales	Rhodobacteraceae	Unknown	

ASV_760	Bacteria	Firmicutes	Clostridia	Clostridiales	Ruminococcaceae	Ruminococcaceae_UCG-014
ASV_761	Bacteria	Firmicutes	Clostridia	Clostridiales	Ruminococcaceae	Ruminococcaceae_UCG-014
ASV_762	Bacteria	Firmicutes	Clostridia	Clostridiales	Ruminococcaceae	Ruminococcaceae_UCG-014
ASV_763	Bacteria	Firmicutes	Clostridia	Clostridiales	Ruminococcaceae	Ruminococcaceae_UCG-014
ASV_764	Bacteria	Tenericutes	Mollicutes	Mollicutes_RF39	Unknown	
ASV_765	Bacteria	Firmicutes	Clostridia	Clostridiales	Lachnospiraceae	Lachnoclostridium
ASV_766	Bacteria	Firmicutes	Clostridia	Clostridiales	Lachnospiraceae	Unknown
ASV_767	Bacteria	Firmicutes	Clostridia	Clostridiales	Lachnospiraceae	Acetatifactor
ASV_768	Bacteria	Firmicutes	Clostridia	Clostridiales	Ruminococcaceae	Butyricicoccus
ASV_769	Bacteria	Firmicutes	Clostridia	Clostridiales	Lachnospiraceae	Unknown
ASV_770	Bacteria	Firmicutes	Clostridia	Clostridiales	Lachnospiraceae	Unknown
ASV_771	Bacteria	Firmicutes	Clostridia	Clostridiales	Lachnospiraceae	Unknown
ASV_772	Bacteria	Bacteroidetes	Bacteroidia	Bacteroidales	Bacteroidaceae	Bacteroides
ASV_773	Bacteria	Firmicutes	Clostridia	Clostridiales	Ruminococcaceae	Ruminiclostridium
ASV_774	Bacteria	Firmicutes	Clostridia	Clostridiales	Clostridiales_vadinBB60_group	Unknown
ASV_775	Bacteria	Firmicutes	Clostridia	Clostridiales	Ruminococcaceae	Ruminococcaceae_UCG-014
ASV_776	Bacteria	Firmicutes	Clostridia	Clostridiales	Ruminococcaceae	Ruminococcaceae_UCG-014
ASV_777	Bacteria	Firmicutes	Clostridia	Clostridiales	Ruminococcaceae	Ruminiclostridium_5
ASV_778	Bacteria	Firmicutes	Clostridia	Clostridiales	Ruminococcaceae	Ruminococcaceae_UCG-014
ASV_779	Bacteria	Firmicutes	Clostridia	Clostridiales	Lachnospiraceae	Unknown
ASV_780	Bacteria	Firmicutes	Clostridia	Clostridiales	Lachnospiraceae	Unknown
ASV_781	Bacteria	Firmicutes	Clostridia	Clostridiales	Ruminococcaceae	Ruminococcaceae_UCG-014
ASV_782	Bacteria	Firmicutes	Clostridia	Clostridiales	Ruminococcaceae	Ruminococcaceae_UCG-014
ASV_783	Bacteria	Firmicutes	Clostridia	Clostridiales	Lachnospiraceae	Lachnospiraceae_NK4A136_group
ASV_784	Bacteria	Firmicutes	Clostridia	Clostridiales	Lachnospiraceae	Lachnospiraceae_NK4A136_group
ASV_785	Bacteria	Firmicutes	Clostridia	Clostridiales	Ruminococcaceae	Oscillibacter
ASV_786	Bacteria	Firmicutes	Clostridia	Clostridiales	Ruminococcaceae	Ruminococcaceae_UCG-013
ASV_787	Bacteria	Firmicutes	Clostridia	Clostridiales	Ruminococcaceae	Ruminococcaceae_UCG-014
ASV_788	Bacteria	Firmicutes	Clostridia	Clostridiales	Lachnospiraceae	Lachnoclostridium
ASV_789	Bacteria	Tenericutes	Mollicutes	Mollicutes_RF39	Unknown	
ASV_790	Bacteria	Firmicutes	Clostridia	Clostridiales	Ruminococcaceae	Ruminococcaceae_UCG-014
ASV_791	Bacteria	Firmicutes	Clostridia	Clostridiales	Lachnospiraceae	Unknown
ASV_792	Bacteria	Firmicutes	Clostridia	Clostridiales	Ruminococcaceae	Ruminiclostridium_5
ASV_793	Bacteria	Firmicutes	Clostridia	Clostridiales	Lachnospiraceae	Unknown
ASV_794	Bacteria	Firmicutes	Clostridia	Clostridiales	Ruminococcaceae	Ruminiclostridium
ASV_795	Bacteria	Firmicutes	Clostridia	Clostridiales	Lachnospiraceae	Lachnospiraceae_UCG-001
ASV_796	Bacteria	Proteobacteria	Gammaproteobacteria	Betaproteobacteriales	Burkholderiaceae	Sutterella

ASV_797	Bacteria	Firmicutes	Clostridia	Clostridiales	Christensenellaceae	Unknown	
ASV_798	Bacteria	Firmicutes	Negativicutes	Selenomonadales	Acidaminococcaceae	Phascolarctobacterium	
ASV_799	Bacteria	Firmicutes	Clostridia	Clostridiales	Lachnospiraceae	Unknown	
ASV_800	Bacteria	Firmicutes	Clostridia	Clostridiales	Ruminococcaceae	Faecalibacterium	
ASV_801	Bacteria	Firmicutes	Clostridia	Clostridiales	Lachnospiraceae	GCA-900066575	
ASV_802	Bacteria	Firmicutes	Clostridia	Clostridiales	Ruminococcaceae	Ruminiclostridium_9	
ASV_803	Bacteria	Firmicutes	Clostridia	Clostridiales	Lachnospiraceae	Lachnospiraceae_NK4A136_group	
ASV_804	Bacteria	Firmicutes	Clostridia	Clostridiales	Lachnospiraceae	Lachnospiraceae_UCG-001	
ASV_805	Bacteria	Firmicutes	Clostridia	Clostridiales	Ruminococcaceae	Unknown	
ASV_806	Bacteria	Firmicutes	Clostridia	Clostridiales	Lachnospiraceae	Lachnospiraceae_UCG-006	
ASV_807	Bacteria	Firmicutes	Clostridia	Clostridiales	Lachnospiraceae	Unknown	
ASV_808	Bacteria	Tenericutes	Mollicutes	Mollicutes_RF39	Unknown		
ASV_809	Bacteria	Bacteroidetes	Bacteroidia	Bacteroidales	Muribaculaceae	Unknown	
ASV_810	Bacteria	Firmicutes	Clostridia	Clostridiales	Lachnospiraceae	Unknown	
ASV_811	Bacteria	Firmicutes	Clostridia	Clostridiales	Ruminococcaceae	Ruminiclostridium_5	
ASV_812	Bacteria	Firmicutes	Clostridia	Clostridiales	Ruminococcaceae	Ruminococcaceae_UCG-014	
ASV_813	Bacteria	Firmicutes	Clostridia	Clostridiales	Ruminococcaceae	Oscillibacter	
ASV_814	Bacteria	Firmicutes	Clostridia	Clostridiales	Ruminococcaceae	Ruminococcaceae_UCG-014	
ASV_815	Bacteria	Firmicutes	Clostridia	Clostridiales	Lachnospiraceae	Unknown	
ASV_816	Bacteria	Firmicutes	Clostridia	Clostridiales	Ruminococcaceae	Ruminococcaceae_UCG-014	
ASV_817	Bacteria	Firmicutes	Clostridia	Clostridiales	Lachnospiraceae	Unknown	
ASV_818	Bacteria	Firmicutes	Clostridia	Clostridiales	Lachnospiraceae	Unknown	
ASV_819	Bacteria	Firmicutes	Clostridia	Clostridiales	Lachnospiraceae	Unknown	
ASV_820	Bacteria	Firmicutes	Clostridia	Clostridiales	Lachnospiraceae	Unknown	
ASV_821	Bacteria	Firmicutes	Clostridia	Clostridiales	Ruminococcaceae	Ruminiclostridium_9	
ASV_822	Bacteria	Firmicutes	Clostridia	Clostridiales	Lachnospiraceae	Unknown	
ASV_823	Bacteria	Firmicutes	Clostridia	Clostridiales	Ruminococcaceae	Anaerotruncus	
ASV_824	Bacteria	Firmicutes	Clostridia	Clostridiales	Lachnospiraceae	Lachnospiraceae_NK4A136_group	
ASV_825	Bacteria	Firmicutes	Clostridia	Clostridiales	Ruminococcaceae	Ruminococcaceae_UCG-014	
ASV_826	Bacteria	Firmicutes	Clostridia	Clostridiales	Lachnospiraceae	Lachnospiraceae_UCG-001	
ASV_827	Bacteria	Firmicutes	Negativicutes	Selenomonadales	Acidaminococcaceae	Phascolarctobacterium	Phascolarctobacterium_faecium
ASV_828	Bacteria	Firmicutes	Clostridia	Clostridiales	Ruminococcaceae	Ruminococcaceae_UCG-014	
ASV_829	Bacteria	Firmicutes	Clostridia	Clostridiales	Ruminococcaceae	Oscillibacter	
ASV_830	Bacteria	Firmicutes	Clostridia	Clostridiales	Lachnospiraceae	Tyzzerella	
ASV_831	Bacteria	Firmicutes	Clostridia	Clostridiales	Ruminococcaceae	Ruminococcaceae_UCG-014	
ASV_832	Bacteria	Firmicutes	Clostridia	Clostridiales	Lachnospiraceae	Lachnoclostridium	

ASV_833	Bacteria	Firmicutes	Bacilli	Lactobacillales	Lactobacillaceae	Lactobacillus	
ASV_834	Bacteria	Firmicutes	Clostridia	Clostridiales	Ruminococcaceae	Ruminococcaceae_UCG-014	
ASV_835	Bacteria	Firmicutes	Clostridia	Clostridiales	Lachnospiraceae	Unknown	
ASV_836	Bacteria	Firmicutes	Clostridia	Clostridiales	Ruminococcaceae	Ruminococcus_1	
ASV_837	Bacteria	Firmicutes	Clostridia	Clostridiales	Ruminococcaceae	Ruminococcus_1	
ASV_838	Bacteria	Bacteroidetes	Bacteroidia	Bacteroidales	Muribaculaceae	CAG-873	
ASV_839	Bacteria	Firmicutes	Clostridia	Clostridiales	Ruminococcaceae	Acetanaerobacterium	
ASV_840	Bacteria	Firmicutes	Clostridia	Clostridiales	Lachnospiraceae	Unknown	
ASV_841	Bacteria	Firmicutes	Erysipelotrichia	Erysipelotrichales	Erysipelotrichaceae	Holdemanella	
ASV_842	Bacteria	Firmicutes	Clostridia	Clostridiales	Lachnospiraceae	Unknown	
ASV_843	Bacteria	Firmicutes	Clostridia	Clostridiales	Ruminococcaceae	Ruminiclostridium	
ASV_844	Bacteria	Firmicutes	Clostridia	Clostridiales	Lachnospiraceae	Unknown	
ASV_845	Bacteria	Actinobacteria	Actinobacteria	Micrococcales	Micrococcaceae	Rothia	
ASV_846	Bacteria	Firmicutes	Clostridia	Clostridiales	Lachnospiraceae	Lachnospiraceae_UCG-001	
ASV_847	Bacteria	Firmicutes	Clostridia	Clostridiales	Clostridiales_vadinBB60_group	Unknown	
ASV_848	Bacteria	Firmicutes	Clostridia	Clostridiales	Ruminococcaceae	Ruminiclostridium	
ASV_849	Bacteria	Firmicutes	Bacilli	Lactobacillales	Lactobacillaceae	Lactobacillus	
ASV_850	Bacteria	Proteobacteria	Alphaproteobacteria	Rhodospirillales	Unknown		
ASV_851	Bacteria	Firmicutes	Clostridia	Clostridiales	Ruminococcaceae	Faecalibacterium	
ASV_852	Bacteria	Firmicutes	Clostridia	Clostridiales	Ruminococcaceae	Ruminococcaceae_UCG-014	
ASV_853	Bacteria	Firmicutes	Clostridia	Clostridiales	Ruminococcaceae	Ruminococcaceae_UCG-014	
ASV_854	Bacteria	Firmicutes	Clostridia	Clostridiales	Lachnospiraceae	Lachnospiraceae_UCG-001	
ASV_855	Bacteria	Bacteroidetes	Bacteroidia	Bacteroidales	Tannerellaceae	Parabacteroides	Parabacteroides_merdae
ASV_856	Bacteria	Firmicutes	Clostridia	Clostridiales	Lachnospiraceae	Unknown	
ASV_857	Bacteria	Bacteroidetes	Bacteroidia	Bacteroidales	Prevotellaceae	Unknown	
ASV_858	Bacteria	Firmicutes	Negativicutes	Selenomonadales	Veillonellaceae	Dialister	Dialister_invisus
ASV_859	Bacteria	Firmicutes	Clostridia	Clostridiales	Lachnospiraceae	Lachnoclostridium	
ASV_860	Bacteria	Firmicutes	Clostridia	Clostridiales	Lachnospiraceae	Unknown	
ASV_861	Bacteria	Firmicutes	Clostridia	Clostridiales	Lachnospiraceae	Unknown	
ASV_862	Bacteria	Firmicutes	Clostridia	Clostridiales	Ruminococcaceae	Ruminococcaceae_UCG-014	
ASV_863	Bacteria	Firmicutes	Clostridia	Clostridiales	Lachnospiraceae	Lachnospiraceae_UCG-010	
ASV_864	Bacteria	Firmicutes	Erysipelotrichia	Erysipelotrichales	Erysipelotrichaceae	Unknown	
ASV_865	Bacteria	Firmicutes	Clostridia	Clostridiales	Lachnospiraceae	Unknown	
ASV_866	Bacteria	Firmicutes	Clostridia	Clostridiales	Lachnospiraceae	Unknown	
ASV_867	Bacteria	Firmicutes	Clostridia	Clostridiales	Lachnospiraceae	ASF356	
ASV_868	Bacteria	Firmicutes	Clostridia	Clostridiales	Ruminococcaceae	Ruminococcaceae_UCG-014	
ASV_869	Bacteria	Firmicutes	Clostridia	Clostridiales	Ruminococcaceae	Ruminiclostridium_5	

ASV_870	Bacteria	Firmicutes	Clostridia	Clostridiales	Ruminococcaceae	Harryflintia	
ASV_871	Bacteria	Firmicutes	Clostridia	Clostridiales	Lachnospiraceae	ASF356	
ASV_872	Bacteria	Actinobacteria	Coriobacteriia	Coriobacteriales	Coriobacteriaceae	Collinsella	Collinsella_aerofaciens
ASV_873	Bacteria	Firmicutes	Clostridia	Clostridiales	Ruminococcaceae	Anaerotruncus	
ASV_874	Bacteria	Firmicutes	Clostridia	Clostridiales	Ruminococcaceae	Ruminococcaceae_UCG-014	
ASV_875	Bacteria	Firmicutes	Clostridia	Clostridiales	Lachnospiraceae	Unknown	
ASV_876	Bacteria	Firmicutes	Clostridia	Clostridiales	Lachnospiraceae	Unknown	
ASV_877	Bacteria	Firmicutes	Clostridia	Clostridiales	Lachnospiraceae	Unknown	
ASV_878	Bacteria	Firmicutes	Clostridia	Clostridiales	Lachnospiraceae	Unknown	
ASV_879	Bacteria	Bacteroidetes	Bacteroidia	Bacteroidales	Prevotellaceae	Prevotella_9	
ASV_880	Bacteria	Firmicutes	Clostridia	Clostridiales	Ruminococcaceae	Unknown	
ASV_881	Bacteria	Firmicutes	Erysipelotrichia	Erysipelotrichales	Erysipelotrichaceae	Erysipelotrichaceae_UCG-003	
ASV_882	Bacteria	Firmicutes	Clostridia	Clostridiales	Ruminococcaceae	Ruminiclostridium	
ASV_883	Bacteria	Firmicutes	Clostridia	Clostridiales	Clostridiales_vadinBB60_group	Unknown	
ASV_884	Bacteria	Firmicutes	Clostridia	Clostridiales	Ruminococcaceae	Oscillibacter	
ASV_885	Bacteria	Firmicutes	Clostridia	Clostridiales	Ruminococcaceae	Ruminococcaceae_UCG-014	
ASV_886	Bacteria	Firmicutes	Clostridia	Clostridiales	Lachnospiraceae	Lachnospiraceae_NK4A136_group	
ASV_887	Bacteria	Firmicutes	Clostridia	Clostridiales	Ruminococcaceae	Ruminococcaceae_UCG-014	
ASV_888	Bacteria	Firmicutes	Erysipelotrichia	Erysipelotrichales	Erysipelotrichaceae	Erysipelatoclostridium	
ASV_889	Bacteria	Bacteroidetes	Bacteroidia	Bacteroidales	Prevotellaceae	Prevotella_9	
ASV_890	Bacteria	Firmicutes	Clostridia	Clostridiales	Lachnospiraceae	Unknown	
ASV_891	Bacteria	Firmicutes	Clostridia	Clostridiales	Lachnospiraceae	GCA-900066575	
ASV_892	Bacteria	Bacteroidetes	Bacteroidia	Bacteroidales	Barnesiellaceae	Barnesiella	
ASV_893	Bacteria	Firmicutes	Clostridia	Clostridiales	Clostridiales_vadinBB60_group	Unknown	
ASV_894	Bacteria	Firmicutes	Clostridia	Clostridiales	Lachnospiraceae	Unknown	
ASV_895	Bacteria	Firmicutes	Clostridia	Clostridiales	Ruminococcaceae	Faecalibacterium	
ASV_896	Bacteria	Firmicutes	Clostridia	Clostridiales	Lachnospiraceae	Lachnoclostridium	
ASV_897	Bacteria	Firmicutes	Clostridia	Clostridiales	Lachnospiraceae	Unknown	
ASV_898	Bacteria	Bacteroidetes	Bacteroidia	Bacteroidales	Barnesiellaceae	Barnesiella	
ASV_899	Bacteria	Bacteroidetes	Bacteroidia	Bacteroidales	Prevotellaceae	Prevotella_9	
ASV_900	Bacteria	Firmicutes	Clostridia	Clostridiales	Ruminococcaceae	Unknown	
ASV_901	Bacteria	Bacteroidetes	Bacteroidia	Bacteroidales	Bacteroidaceae	Bacteroides	Bacteroides_uniformis
ASV_902	Bacteria	Firmicutes	Clostridia	Clostridiales	Clostridiales_vadinBB60_group	Unknown	
ASV_903	Bacteria	Firmicutes	Clostridia	Clostridiales	Ruminococcaceae	Ruminococcaceae_UCG-014	
ASV_904	Bacteria	Bacteroidetes	Bacteroidia	Bacteroidales	Muribaculaceae	Unknown	
ASV_905	Bacteria	Firmicutes	Clostridia	Clostridiales	Ruminococcaceae	Ruminiclostridium	
ASV_906	Bacteria	Firmicutes	Clostridia	Clostridiales	Lachnospiraceae	Lachnoclostridium	

ASV_907	Bacteria	Firmicutes	Clostridia	Clostridiales	Lachnospiraceae	Unknown	
ASV_908	Bacteria	Proteobacteria	Gammaproteobacteria	Pseudomonadales	Moraxellaceae	Enhydrobacter	
ASV_909	Bacteria	Firmicutes	Clostridia	Clostridiales	Ruminococcaceae	Ruminiclostridium_9	
ASV_910	Bacteria	Firmicutes	Clostridia	Clostridiales	Clostridiaceae_1	Clostridium_sensu_stricto_1	
ASV_911	Bacteria	Firmicutes	Clostridia	Clostridiales	Lachnospiraceae	Unknown	
ASV_912	Bacteria	Bacteroidetes	Bacteroidia	Bacteroidales	Rikenellaceae	Alistipes	
ASV_913	Bacteria	Firmicutes	Clostridia	Clostridiales	Lachnospiraceae	Unknown	
ASV_914	Bacteria	Firmicutes	Clostridia	Clostridiales	Lachnospiraceae	Unknown	
ASV_915	Bacteria	Firmicutes	Clostridia	Clostridiales	Lachnospiraceae	Lachnoclostridium	
ASV_916	Bacteria	Firmicutes	Clostridia	Clostridiales	Ruminococcaceae	Ruminiclostridium	
ASV_917	Bacteria	Firmicutes	Clostridia	Clostridiales	Lachnospiraceae	Unknown	
ASV_918	Bacteria	Firmicutes	Clostridia	Clostridiales	Ruminococcaceae	Ruminococcus_2	
ASV_919	Bacteria	Firmicutes	Clostridia	Clostridiales	Ruminococcaceae	Ruminococcaceae_UCG-014	
ASV_920	Bacteria	Firmicutes	Erysipelotrichia	Erysipelotrichales	Erysipelotrichaceae	Unknown	
ASV_921	Bacteria	Firmicutes	Clostridia	Clostridiales	Lachnospiraceae	Unknown	
ASV_922	Bacteria	Firmicutes	Clostridia	Clostridiales	Lachnospiraceae	Unknown	
ASV_923	Bacteria	Bacteroidetes	Bacteroidia	Bacteroidales	Rikenellaceae	Alistipes	Alistipes_onderdonkii
ASV_924	Bacteria	Firmicutes	Clostridia	Clostridiales	Ruminococcaceae	Ruminiclostridium_9	
ASV_925	Bacteria	Firmicutes	Clostridia	Clostridiales	Clostridiales_vadinBB60_group	Unknown	
ASV_926	Bacteria	Firmicutes	Erysipelotrichia	Erysipelotrichales	Erysipelotrichaceae	Erysipelotrichaceae_UCG-003	
ASV_927	Bacteria	Firmicutes	Clostridia	Clostridiales	Lachnospiraceae	Unknown	
ASV_928	Bacteria	Firmicutes	Clostridia	Clostridiales	Ruminococcaceae	Unknown	
ASV_929	Bacteria	Bacteroidetes	Bacteroidia	Bacteroidales	Rikenellaceae	Alistipes	
ASV_930	Bacteria	Firmicutes	Clostridia	Clostridiales	Lachnospiraceae	Lachnospiraceae_NK4A136_group	
ASV_931	Bacteria	Bacteroidetes	Bacteroidia	Bacteroidales	Bacteroidaceae	Bacteroides	
ASV_932	Bacteria	Firmicutes	Clostridia	Clostridiales	Ruminococcaceae	Ruminiclostridium_9	
ASV_933	Bacteria	Firmicutes	Clostridia	Clostridiales	Lachnospiraceae	Unknown	
ASV_934	Bacteria	Firmicutes	Clostridia	Clostridiales	Ruminococcaceae	Ruminococcaceae_UCG-014	
ASV_935	Bacteria	Firmicutes	Clostridia	Clostridiales	Lachnospiraceae	Lachnoclostridium	
ASV_936	Bacteria	Firmicutes	Clostridia	Clostridiales	Lachnospiraceae	Roseburia	
ASV_937	Bacteria	Firmicutes	Clostridia	Clostridiales	Lachnospiraceae	Lachnospiraceae_NK4A136_group	
ASV_938	Bacteria	Firmicutes	Clostridia	Clostridiales	Ruminococcaceae	Ruminiclostridium_5	
ASV_939	Bacteria	Bacteroidetes	Bacteroidia	Bacteroidales	Bacteroidaceae	Bacteroides	
ASV_940	Bacteria	Bacteroidetes	Bacteroidia	Bacteroidales	Rikenellaceae	Alistipes	
ASV_941	Bacteria	Firmicutes	Clostridia	Clostridiales	Lachnospiraceae	Unknown	
ASV_942	Bacteria	Firmicutes	Clostridia	Clostridiales	Ruminococcaceae	Ruminococcaceae_UCG-014	
ASV_943	Bacteria	Firmicutes	Clostridia	Clostridiales	Ruminococcaceae	Ruminiclostridium_6	

ASV_944	Bacteria	Firmicutes	Clostridia	Clostridiales	Lachnospiraceae	Lachnospiraceae_UCG-006	
ASV_945	Bacteria	Firmicutes	Clostridia	Clostridiales	Lachnospiraceae	Unknown	
ASV_946	Bacteria	Firmicutes	Clostridia	Clostridiales	Ruminococcaceae	Ruminiclostridium_5	
ASV_947	Bacteria	Firmicutes	Clostridia	Clostridiales	Lachnospiraceae	Unknown	
ASV_948	Bacteria	Firmicutes	Clostridia	Clostridiales	Ruminococcaceae	Ruminiclostridium_9	
ASV_949	Bacteria	Firmicutes	Clostridia	Clostridiales	Ruminococcaceae	Oscillibacter	
ASV_950	Bacteria	Firmicutes	Clostridia	Clostridiales	Lachnospiraceae	Coprococcus_2	Coprococcus_2_eutactus
ASV_951	Bacteria	Firmicutes	Clostridia	Clostridiales	Christensenellaceae	Christensenellaceae_R-7_group	
ASV_952	Bacteria	Firmicutes	Clostridia	Clostridiales	Lachnospiraceae	Unknown	
ASV_953	Bacteria	Bacteroidetes	Bacteroidia	Bacteroidales	Rikenellaceae	Alistipes	
ASV_954	Bacteria	Bacteroidetes	Bacteroidia	Bacteroidales	Prevotellaceae	Unknown	
ASV_955	Bacteria	Firmicutes	Clostridia	Clostridiales	Ruminococcaceae	Ruminococcaceae_UCG-014	
ASV_956	Bacteria	Firmicutes	Clostridia	Clostridiales	Ruminococcaceae	Unknown	
ASV_957	Bacteria	Firmicutes	Clostridia	Clostridiales	Ruminococcaceae	Unknown	
ASV_958	Bacteria	Firmicutes	Clostridia	Clostridiales	Lachnospiraceae	Unknown	
ASV_959	Bacteria	Firmicutes	Clostridia	Clostridiales	Ruminococcaceae	Ruminiclostridium_9	
ASV_960	Bacteria	Firmicutes	Clostridia	Clostridiales	Lachnospiraceae	Lachnospiraceae_NK4A136_group	
ASV_961	Bacteria	Firmicutes	Bacilli	Lactobacillales	Streptococcaceae	Streptococcus	
ASV_962	Bacteria	Firmicutes	Clostridia	Clostridiales	Lachnospiraceae	Unknown	
ASV_963	Bacteria	Firmicutes	Clostridia	Clostridiales	Lachnospiraceae	Unknown	
ASV_964	Bacteria	Firmicutes	Clostridia	Clostridiales	Lachnospiraceae	Unknown	
ASV_965	Bacteria	Firmicutes	Clostridia	Clostridiales	Clostridiales_vadinBB60_group	Unknown	
ASV_966	Bacteria	Bacteroidetes	Bacteroidia	Bacteroidales	Rikenellaceae	Alistipes	
ASV_967	Bacteria	Firmicutes	Clostridia	Clostridiales	Ruminococcaceae	Unknown	
ASV_968	Bacteria	Bacteroidetes	Bacteroidia	Bacteroidales	Prevotellaceae	Prevotellaceae_UCG-001	
ASV_969	Bacteria	Firmicutes	Clostridia	Clostridiales	Lachnospiraceae	Lachnospiraceae_UCG-006	
ASV_970	Bacteria	Firmicutes	Clostridia	Clostridiales	Ruminococcaceae	Ruminiclostridium_9	
ASV_971	Bacteria	Firmicutes	Clostridia	Clostridiales	Lachnospiraceae	Lachnospiraceae_NK4A136_group	
ASV_972	Bacteria	Firmicutes	Clostridia	Clostridiales	Ruminococcaceae	Unknown	
ASV_973	Bacteria	Firmicutes	Clostridia	Clostridiales	Lachnospiraceae	Lachnospiraceae_NK4A136_group	
ASV_974	Bacteria	Firmicutes	Clostridia	Clostridiales	Lachnospiraceae	Unknown	
ASV_975	Bacteria	Firmicutes	Clostridia	Clostridiales	Ruminococcaceae	Ruminococcaceae_UCG-014	
ASV_976	Bacteria	Firmicutes	Clostridia	Clostridiales	Ruminococcaceae	Ruminiclostridium_5	
ASV_977	Bacteria	Firmicutes	Clostridia	Clostridiales	Lachnospiraceae	Lachnospira	Lachnospira_pectinoschiza
ASV_978	Bacteria	Firmicutes	Clostridia	Clostridiales	Christensenellaceae	Christensenellaceae_R-7_group	
ASV_979	Bacteria	Firmicutes	Clostridia	Clostridiales	Lachnospiraceae	A2	
ASV_980	Bacteria	Firmicutes	Clostridia	Clostridiales	Ruminococcaceae	Oscillibacter	

ASV_981	Bacteria	Firmicutes	Clostridia	Clostridiales	Lachnospiraceae	Lachnospiraceae_NK4A136_group	
ASV_982	Bacteria	Firmicutes	Clostridia	Clostridiales	Lachnospiraceae	Unknown	
ASV_983	Bacteria	Firmicutes	Erysipelotrichia	Erysipelotrichales	Erysipelotrichaceae	Holdemanella	
ASV_984	Bacteria	Firmicutes	Clostridia	Clostridiales	Lachnospiraceae	Roseburia	
ASV_985	Bacteria	Firmicutes	Clostridia	Clostridiales	Lachnospiraceae	Unknown	
ASV_986	Bacteria	Firmicutes	Clostridia	Clostridiales	Ruminococcaceae	Ruminococcaceae_UCG-014	
ASV_987	Bacteria	Bacteroidetes	Bacteroidia	Bacteroidales	Prevotellaceae	Prevotellaceae_UCG-001	
ASV_988	Bacteria	Firmicutes	Clostridia	Clostridiales	Lachnospiraceae	Lachnospiraceae_UCG-006	
ASV_989	Bacteria	Firmicutes	Clostridia	Clostridiales	Lachnospiraceae	Roseburia	
ASV_990	Bacteria	Firmicutes	Clostridia	Clostridiales	Lachnospiraceae	Butyrivibrio	Butyrivibrio_crossotus
ASV_991	Bacteria	Firmicutes	Clostridia	Clostridiales	Lachnospiraceae	Tyzzerella_3	
ASV_992	Bacteria	Firmicutes	Clostridia	Clostridiales	Ruminococcaceae	Unknown	
ASV_993	Bacteria	Firmicutes	Clostridia	Clostridiales	Lachnospiraceae	Lachnospiraceae_UCG-006	
ASV_994	Bacteria	Firmicutes	Clostridia	Clostridiales	Lachnospiraceae	Lachnospiraceae_UCG-001	
ASV_995	Bacteria	Firmicutes	Clostridia	Clostridiales	Lachnospiraceae	Unknown	
ASV_996	Bacteria	Firmicutes	Clostridia	Clostridiales	Lachnospiraceae	Lachnoclostridium	
ASV_997	Bacteria	Proteobacteria	Gammaproteobacteria	Enterobacteriales	Enterobacteriaceae	Escherichia/Shigella	
ASV_998	Bacteria	Firmicutes	Clostridia	Clostridiales	Ruminococcaceae	Faecalibacterium	
ASV_999	Bacteria	Firmicutes	Clostridia	Clostridiales	Lachnospiraceae	Unknown	
ASV_1000	Bacteria	Firmicutes	Clostridia	Clostridiales	Lachnospiraceae	Unknown	
ASV_1001	Bacteria	Firmicutes	Clostridia	Clostridiales	Lachnospiraceae	A2	
ASV_1002	Bacteria	Firmicutes	Clostridia	Clostridiales	Ruminococcaceae	Unknown	
ASV_1003	Bacteria	Firmicutes	Clostridia	Clostridiales	Lachnospiraceae	Lachnospiraceae_NK4A136_group	
ASV_1004	Bacteria	Firmicutes	Negativicutes	Selenomonadales	Acidaminococcaceae	Acidaminococcus	
ASV_1005	Bacteria	Firmicutes	Erysipelotrichia	Erysipelotrichales	Erysipelotrichaceae	Holdemanella	
ASV_1006	Bacteria	Firmicutes	Clostridia	Clostridiales	Lachnospiraceae	Unknown	
ASV_1007	Bacteria	Firmicutes	Clostridia	Clostridiales	Ruminococcaceae	Faecalibacterium	
ASV_1008	Bacteria	Firmicutes	Clostridia	Clostridiales	Lachnospiraceae	A2	
ASV_1009	Bacteria	Firmicutes	Clostridia	Clostridiales	Lachnospiraceae	Unknown	
ASV_1010	Bacteria	Tenericutes	Mollicutes	Mollicutes_RF39	Unknown		
ASV_1011	Bacteria	Firmicutes	Clostridia	Clostridiales	Ruminococcaceae	Ruminococcaceae_UCG-014	
ASV_1012	Bacteria	Firmicutes	Clostridia	Clostridiales	Clostridiaceae_1	Clostridium_sensu_stricto_1	
ASV_1013	Bacteria	Firmicutes	Clostridia	Clostridiales	Lachnospiraceae	Unknown	
ASV_1014	Bacteria	Bacteroidetes	Bacteroidia	Bacteroidales	Muribaculaceae	Unknown	
ASV_1015	Bacteria	Bacteroidetes	Bacteroidia	Bacteroidales	Rikenellaceae	Alistipes	
ASV_1016	Bacteria	Firmicutes	Clostridia	Clostridiales	Ruminococcaceae	Ruminiclostridium_9	
ASV_1017	Bacteria	Firmicutes	Clostridia	Clostridiales	Ruminococcaceae	UBA1819	

ASV_1018	Bacteria	Firmicutes	Clostridia	Clostridiales	Ruminococcaceae	Unknown	
ASV_1019	Bacteria	Firmicutes	Clostridia	Clostridiales	Ruminococcaceae	Ruminiclostridium_9	
ASV_1020	Bacteria	Firmicutes	Clostridia	Clostridiales	Lachnospiraceae	Lachnospiraceae_FCS020_group	
ASV_1021	Bacteria	Firmicutes	Clostridia	Clostridiales	Ruminococcaceae	Ruminococcaceae_UCG-014	
ASV_1022	Bacteria	Bacteroidetes	Bacteroidia	Bacteroidales	Bacteroidaceae	Bacteroides	Bacteroides_vulgatus
ASV_1023	Bacteria	Tenericutes	Mollicutes	Mollicutes_RF39	Unknown		
ASV_1024	Bacteria	Firmicutes	Clostridia	Clostridiales	Lachnospiraceae	Lachnoclostridium	
ASV_1025	Bacteria	Firmicutes	Clostridia	Clostridiales	Ruminococcaceae	Intestinimonas	
ASV_1026	Bacteria	Firmicutes	Clostridia	Clostridiales	Lachnospiraceae	Blautia	
ASV_1027	Bacteria	Firmicutes	Clostridia	Clostridiales	Ruminococcaceae	Oscillibacter	
ASV_1028	Bacteria	Firmicutes	Clostridia	Clostridiales	Ruminococcaceae	Oscillibacter	
ASV_1029	Bacteria	Firmicutes	Clostridia	Clostridiales	Lachnospiraceae	Lachnoclostridium	
ASV_1030	Bacteria	Firmicutes	Clostridia	Clostridiales	Lachnospiraceae	Unknown	
ASV_1031	Bacteria	Firmicutes	Clostridia	Clostridiales	Lachnospiraceae	Roseburia	
ASV_1032	Bacteria	Tenericutes	Mollicutes	Mollicutes_RF39	Unknown		
ASV_1033	Bacteria	Firmicutes	Clostridia	Clostridiales	Lachnospiraceae	Lachnospiraceae_NK4A136_group	
ASV_1034	Bacteria	Firmicutes	Clostridia	Clostridiales	Ruminococcaceae	Ruminococcaceae_UCG-005	
ASV_1035	Bacteria	Firmicutes	Clostridia	Clostridiales	Lachnospiraceae	Unknown	
ASV_1036	Bacteria			Unknown			
ASV_1037	Bacteria	Firmicutes	Clostridia	Clostridiales	Lachnospiraceae	Unknown	
ASV_1038	Bacteria	Firmicutes	Clostridia	Clostridiales	Lachnospiraceae	Lachnospiraceae_NK4A136_group	
ASV_1039	Bacteria	Bacteroidetes	Bacteroidia	Bacteroidales	Muribaculaceae	Unknown	
ASV_1040	Bacteria	Firmicutes	Clostridia	Clostridiales	Lachnospiraceae	Lachnoclostridium	
ASV_1041	Bacteria	Bacteroidetes	Bacteroidia	Bacteroidales	Bacteroidaceae	Bacteroides	
ASV_1042	Bacteria	Firmicutes	Clostridia	Clostridiales	Ruminococcaceae	Ruminiclostridium_9	
ASV_1043	Bacteria	Firmicutes	Clostridia	Clostridiales	Ruminococcaceae	Ruminococcaceae_UCG-013	
ASV_1044	Bacteria	Actinobacteria	Coriobacteriia	Coriobacteriales	Coriobacteriaceae	Collinsella	Collinsella_aerofaciens
ASV_1045	Bacteria	Firmicutes	Clostridia	Clostridiales	Ruminococcaceae	Ruminiclostridium	
ASV_1046	Bacteria	Firmicutes	Clostridia	Clostridiales	Ruminococcaceae	Ruminococcus_1	
ASV_1047	Bacteria	Firmicutes	Clostridia	Clostridiales	Lachnospiraceae	Unknown	
ASV_1048	Bacteria	Firmicutes	Clostridia	Clostridiales	Ruminococcaceae	Ruminiclostridium_9	
ASV_1049	Bacteria	Firmicutes	Clostridia	Clostridiales	Lachnospiraceae	Unknown	
ASV_1050	Bacteria	Firmicutes	Clostridia	Clostridiales	Lachnospiraceae	Lachnospiraceae_UCG-001	
ASV_1051	Bacteria	Tenericutes	Mollicutes	Mollicutes_RF39	Unknown		
ASV_1052	Bacteria	Firmicutes	Clostridia	Clostridiales	Ruminococcaceae	Oscillibacter	
ASV_1053	Bacteria	Firmicutes	Clostridia	Clostridiales	Ruminococcaceae	Unknown	
ASV_1054	Bacteria	Firmicutes	Clostridia	Clostridiales	Ruminococcaceae	Ruminococcaceae_UCG-014	

ASV_1055	Bacteria	Firmicutes	Clostridia	Clostridiales	Lachnospiraceae	Unknown	
ASV_1056	Bacteria	Firmicutes	Clostridia	Clostridiales	Lachnospiraceae	Lachnoclostridium	
ASV_1057	Bacteria	Bacteroidetes	Bacteroidia	Bacteroidales	Prevotellaceae	Prevotellaceae_NK3B31_group	
ASV_1058	Bacteria	Firmicutes	Clostridia	Clostridiales	Ruminococcaceae	Ruminiclostridium	
ASV_1059	Bacteria	Bacteroidetes	Bacteroidia	Bacteroidales	Prevotellaceae	Prevotella_9	
ASV_1060	Bacteria	Firmicutes	Clostridia	Clostridiales	Family_XIII	Family_XIII_UCG-001	
ASV_1061	Bacteria	Firmicutes	Clostridia	Clostridiales	Lachnospiraceae	Unknown	
ASV_1062	Bacteria	Bacteroidetes	Bacteroidia	Bacteroidales	Prevotellaceae	Prevotella_7	
ASV_1063	Bacteria	Bacteroidetes	Bacteroidia	Bacteroidales	Prevotellaceae	Prevotella_2	
ASV_1064	Bacteria	Firmicutes	Clostridia	Clostridiales	Lachnospiraceae	Unknown	
ASV_1065	Bacteria	Bacteroidetes	Bacteroidia	Bacteroidales	Barnesiellaceae	Barnesiella	
ASV_1066	Bacteria	Firmicutes	Clostridia	Clostridiales	Lachnospiraceae	A2	
ASV_1067	Bacteria	Firmicutes	Clostridia	Clostridiales	Lachnospiraceae	Unknown	
ASV_1068	Bacteria	Firmicutes	Clostridia	Clostridiales	Ruminococcaceae	Faecalibacterium	
ASV_1069	Bacteria	Firmicutes	Clostridia	Clostridiales		Unknown	
ASV_1070	Bacteria	Firmicutes	Clostridia	Clostridiales	Ruminococcaceae	Ruminococcaceae_UCG-002	
ASV_1071	Bacteria	Firmicutes	Clostridia	Clostridiales	Ruminococcaceae	Unknown	
ASV_1072	Bacteria	Bacteroidetes	Bacteroidia	Bacteroidales	Bacteroidaceae	Bacteroides	Bacteroides_eggerthii
ASV_1073	Bacteria	Firmicutes	Clostridia	Clostridiales	Lachnospiraceae	Unknown	
ASV_1074	Bacteria	Firmicutes	Clostridia	Clostridiales	Ruminococcaceae	Ruminiclostridium_6	
ASV_1075	Bacteria	Bacteroidetes	Bacteroidia	Bacteroidales	Bacteroidaceae	Bacteroides	Bacteroides_eggerthii
ASV_1076	Bacteria	Firmicutes	Clostridia	Clostridiales	Christensenellaceae	Unknown	
ASV_1077	Bacteria	Firmicutes	Clostridia	Clostridiales	Ruminococcaceae	Ruminococcaceae_UCG-014	
ASV_1078	Bacteria	Firmicutes	Clostridia	Clostridiales	Lachnospiraceae	Lachnospiraceae_UCG-006	
ASV_1079	Bacteria	Firmicutes	Clostridia	Clostridiales	Lachnospiraceae	Lachnospiraceae_NK4A136_group	
ASV_1080	Bacteria	Firmicutes	Clostridia	Clostridiales	Ruminococcaceae	Faecalibacterium	
ASV_1081	Bacteria	Firmicutes	Clostridia	Clostridiales	Lachnospiraceae	Unknown	
ASV_1082	Bacteria	Firmicutes	Bacilli	Lactobacillales	Lactobacillaceae	Lactobacillus	
ASV_1083	Bacteria	Firmicutes	Clostridia	Clostridiales	Lachnospiraceae	Agathobacter	
ASV_1084	Bacteria	Firmicutes	Clostridia	Clostridiales	Lachnospiraceae	Unknown	
ASV_1085	Bacteria	Firmicutes	Clostridia	Clostridiales	Christensenellaceae	Christensenellaceae_R-7_group	
ASV_1086	Bacteria	Firmicutes	Clostridia	Clostridiales	Ruminococcaceae	Negativibacillus	
ASV_1087	Bacteria	Firmicutes	Clostridia	Clostridiales	Lachnospiraceae	A2	
ASV_1088	Bacteria	Firmicutes	Clostridia	Clostridiales	Ruminococcaceae	Ruminococcaceae_UCG-014	
ASV_1089	Bacteria	Firmicutes	Clostridia	Clostridiales	Lachnospiraceae	Unknown	
ASV_1090	Bacteria	Firmicutes	Clostridia	Clostridiales	Ruminococcaceae	Ruminiclostridium	
ASV_1091	Bacteria	Firmicutes	Clostridia	Clostridiales	Ruminococcaceae	Ruminiclostridium_9	

ASV_1092	Bacteria	Firmicutes	Clostridia	Clostridiales	Ruminococcaceae	Ruminococcaceae_UCG-014	
ASV_1093	Bacteria	Firmicutes	Clostridia	Clostridiales	Lachnospiraceae	Lachnospiraceae_UCG-006	
ASV_1094	Bacteria	Firmicutes	Clostridia	Clostridiales	Ruminococcaceae	Ruminococcaceae_UCG-014	
ASV_1095	Bacteria	Bacteroidetes	Bacteroidia	Bacteroidales	Tannerellaceae	Parabacteroides	Parabacteroides_merdae
ASV_1096	Bacteria	Firmicutes	Clostridia	Clostridiales	Lachnospiraceae	Lachnospiraceae_NK4A136_group	
ASV_1097	Bacteria	Firmicutes	Clostridia	Clostridiales	Lachnospiraceae	Lachnoclostridium	
ASV_1098	Bacteria	Bacteroidetes	Bacteroidia	Bacteroidales	Rikenellaceae	Rikenellaceae_RC9_gut_group	
ASV_1099	Bacteria			Unknown			
ASV_1100	Bacteria	Firmicutes	Clostridia	Clostridiales	Ruminococcaceae	Ruminiclostridium_5	
ASV_1101	Bacteria	Firmicutes	Clostridia	Clostridiales	Ruminococcaceae	Ruminiclostridium_9	
ASV_1102	Bacteria	Bacteroidetes	Bacteroidia	Bacteroidales	Muribaculaceae	Unknown	
ASV_1103	Bacteria	Firmicutes	Clostridia	Clostridiales	Lachnospiraceae	Lachnospiraceae_UCG-006	
ASV_1104	Bacteria	Firmicutes	Clostridia	Clostridiales	Lachnospiraceae	Lachnospiraceae_UCG-006	
ASV_1105	Bacteria	Firmicutes	Clostridia	Clostridiales	Lachnospiraceae	Unknown	
ASV_1106	Bacteria	Firmicutes	Clostridia	Clostridiales	Lachnospiraceae	GCA-900066575	
ASV_1107	Bacteria	Firmicutes	Clostridia	Clostridiales	Lachnospiraceae	GCA-900066575	
ASV_1108	Bacteria	Actinobacteria	Coriobacteriia	Coriobacteriales	Eggerthellaceae	Denitrobacterium	
ASV_1109	Bacteria	Spirochaetes	Spirochaetia	Spirochaetales	Spirochaetaceae	Treponema_2	
ASV_1110	Bacteria	Firmicutes	Clostridia	Clostridiales	Lachnospiraceae	Unknown	
ASV_1111	Bacteria	Firmicutes	Clostridia	Clostridiales	Lachnospiraceae	Unknown	
ASV_1112	Bacteria	Firmicutes	Clostridia	Clostridiales	Ruminococcaceae	Faecalibacterium	
ASV_1113	Bacteria	Firmicutes	Clostridia	Clostridiales	Ruminococcaceae	Ruminococcaceae_UCG-014	
ASV_1114	Bacteria	Firmicutes	Clostridia	Clostridiales	Lachnospiraceae	Lachnoclostridium	
ASV_1115	Bacteria	Firmicutes	Clostridia	Clostridiales	Lachnospiraceae	Lachnospiraceae_NK4A136_group	
ASV_1116	Bacteria	Firmicutes	Clostridia	Clostridiales	Clostridiales_vadinBB60_group	Unknown	
ASV_1117	Bacteria	Firmicutes	Clostridia	Clostridiales	Lachnospiraceae	Unknown	
ASV_1118	Bacteria			Unknown			
ASV_1119	Bacteria	Proteobacteria	Gammaproteobacteria	Pseudomonadales	Moraxellaceae	Acinetobacter	Acinetobacter_johnsonii
ASV_1120	Bacteria	Bacteroidetes	Bacteroidia	Bacteroidales	Bacteroidaceae	Bacteroides	
ASV_1121	Bacteria	Firmicutes	Clostridia	Clostridiales	Lachnospiraceae	Unknown	
ASV_1122	Bacteria	Bacteroidetes	Bacteroidia	Bacteroidales	Bacteroidaceae	Bacteroides	
ASV_1123	Bacteria	Bacteroidetes	Bacteroidia	Bacteroidales	Bacteroidaceae	Bacteroides	
ASV_1124	Bacteria	Firmicutes	Clostridia	Clostridiales	Ruminococcaceae	Unknown	
ASV_1125	Bacteria	Firmicutes	Clostridia	Clostridiales	Lachnospiraceae	Lachnospiraceae_NK4A136_group	
ASV_1126	Bacteria	Firmicutes	Clostridia	Clostridiales	Lachnospiraceae	Lachnospiraceae_NK4A136_group	
ASV_1127	Bacteria	Firmicutes	Clostridia	Clostridiales	Lachnospiraceae	Unknown	
ASV_1128	Bacteria	Firmicutes	Clostridia	Clostridiales	Lachnospiraceae	Unknown	

ASV_1129	Bacteria	Firmicutes	Clostridia	Clostridiales	Lachnospiraceae	Unknown	
ASV_1130	Bacteria	Bacteroidetes	Bacteroidia	Bacteroidales	Prevotellaceae	Prevotellaceae_UCG-001	
ASV_1131	Bacteria	Firmicutes	Clostridia	Clostridiales	Ruminococcaceae	Ruminococcus_2	
ASV_1132	Bacteria	Deferribacteres	Deferribacteres	Deferribacterales	Deferribacteraceae	Mucispirillum	
ASV_1133	Bacteria	Firmicutes	Bacilli	Lactobacillales	Streptococcaceae	Streptococcus	
ASV_1134	Bacteria	Firmicutes	Clostridia	Clostridiales	Lachnospiraceae	Unknown	
ASV_1135	Bacteria	Bacteroidetes	Bacteroidia	Bacteroidales	Muribaculaceae	Unknown	
ASV_1136	Bacteria	Firmicutes	Clostridia	Clostridiales	Lachnospiraceae	Marvinbryantia	
ASV_1137	Bacteria	Bacteroidetes	Bacteroidia	Bacteroidales	Rikenellaceae	Alistipes	
ASV_1138	Bacteria	Firmicutes	Negativicutes	Selenomonadales	Veillonellaceae	Dialister	
ASV_1139	Bacteria	Firmicutes	Clostridia	Clostridiales	Clostridiales_vadinBB60_group	Unknown	
ASV_1140	Bacteria	Firmicutes	Clostridia	Clostridiales	Lachnospiraceae	Lachnospiraceae_UCG-001	
ASV_1141	Bacteria	Bacteroidetes	Bacteroidia	Bacteroidales	Muribaculaceae	Unknown	
ASV_1142	Bacteria	Firmicutes	Erysipelotrichia	Erysipelotrichales	Erysipelotrichaceae	Erysipelatoclostridium	
ASV_1143	Bacteria	Firmicutes				Unknown	
ASV_1144	Bacteria	Deferribacteres	Deferribacteres	Deferribacterales	Deferribacteraceae	Mucispirillum	
ASV_1145	Bacteria	Actinobacteria	Coriobacteriia	Coriobacteriales	Eggerthellaceae	Enterorhabdus	
ASV_1146	Bacteria	Firmicutes	Clostridia	Clostridiales	Lachnospiraceae	Unknown	
ASV_1147	Bacteria	Bacteroidetes	Bacteroidia	Bacteroidales	Muribaculaceae	Unknown	
ASV_1148	Bacteria	Firmicutes	Clostridia	Clostridiales	Lachnospiraceae	Lachnospiraceae_NK4A136_group	
ASV_1149	Bacteria	Firmicutes	Negativicutes	Selenomonadales	Acidaminococcaceae	Phascolarctobacterium	
ASV_1150	Bacteria	Firmicutes	Bacilli	Lactobacillales	Lactobacillaceae	Lactobacillus	
ASV_1151	Bacteria	Firmicutes	Clostridia	Clostridiales	Clostridiales_vadinBB60_group	Unknown	
ASV_1152	Bacteria	Firmicutes	Clostridia	Clostridiales	Lachnospiraceae	Roseburia	
ASV_1153	Bacteria	Firmicutes	Negativicutes	Selenomonadales	Acidaminococcaceae	Phascolarctobacterium	
ASV_1154	Bacteria	Firmicutes	Clostridia	Clostridiales	Ruminococcaceae	Ruminococcaceae_UCG-014	
ASV_1155	Bacteria	Firmicutes	Clostridia	Clostridiales	Lachnospiraceae	Lachnoclostridium	
ASV_1156	Bacteria	Firmicutes	Clostridia	Clostridiales	Ruminococcaceae	Ruminococcus_2	
ASV_1157	Bacteria	Firmicutes	Clostridia	Clostridiales	Lachnospiraceae	Unknown	
ASV_1158	Bacteria	Proteobacteria	Alphaproteobacteria	Caulobacterales	Caulobacteraceae	Brevundimonas	Brevundimonas_vesicularis
ASV_1159	Bacteria	Bacteroidetes	Bacteroidia	Bacteroidales	Bacteroidaceae	Bacteroides	
ASV_1160	Bacteria	Firmicutes	Clostridia	Clostridiales	Ruminococcaceae	Ruminococcaceae_UCG-014	
ASV_1161	Bacteria	Firmicutes	Clostridia	Clostridiales	Lachnospiraceae	Unknown	
ASV_1162	Bacteria	Firmicutes	Clostridia	Clostridiales	Lachnospiraceae	Unknown	
ASV_1163	Bacteria	Firmicutes	Clostridia	Clostridiales	Lachnospiraceae	Agathobacter	
ASV_1164	Bacteria	Firmicutes	Clostridia	Clostridiales	Lachnospiraceae	Unknown	

ASV_1165	Bacteria	Firmicutes	Clostridia	Clostridiales	Lachnospiraceae	Lachnospiraceae_UCG-006	
ASV_1166	Bacteria	Bacteroidetes	Bacteroidia	Bacteroidales	Bacteroidaceae	Bacteroides	
ASV_1167	Bacteria	Firmicutes	Clostridia	Clostridiales	Family_XIII	Family_XIII_AD3011_group	
ASV_1168	Bacteria	Bacteroidetes	Bacteroidia	Bacteroidales	Rikenellaceae	Alistipes	
ASV_1169	Bacteria	Bacteroidetes	Bacteroidia	Bacteroidales	Bacteroidaceae	Bacteroides	
ASV_1170	Bacteria	Firmicutes	Clostridia	Clostridiales	Lachnospiraceae	Unknown	
ASV_1171	Bacteria	Firmicutes	Clostridia	Clostridiales	Lachnospiraceae	Unknown	
ASV_1172	Bacteria	Firmicutes	Clostridia	Clostridiales	Ruminococcaceae	Ruminiclostridium_9	
ASV_1173	Bacteria	Firmicutes	Clostridia	Clostridiales	Lachnospiraceae	Lachnospiraceae_NK4A136_group	
ASV_1174	Bacteria	Firmicutes	Clostridia	Clostridiales	Lachnospiraceae	Unknown	
ASV_1175	Bacteria	Actinobacteria	Coriobacteriia	Coriobacteriales	Coriobacteriales_Incertae_Sedis	Unknown	
ASV_1176	Bacteria	Firmicutes	Clostridia	Clostridiales	Ruminococcaceae	Ruminococcus_2	
ASV_1177	Bacteria	Firmicutes	Clostridia	Clostridiales	Ruminococcaceae	Butyricicoccus	
ASV_1178	Bacteria	Firmicutes	Clostridia	Clostridiales	Ruminococcaceae	Unknown	
ASV_1179	Bacteria	Bacteroidetes	Bacteroidia	Bacteroidales	Rikenellaceae	Alistipes	
ASV_1180	Bacteria	Bacteroidetes	Bacteroidia	Bacteroidales	Bacteroidaceae	Bacteroides	Bacteroides_cellulosilyticus
ASV_1181	Bacteria	Bacteroidetes	Bacteroidia	Bacteroidales	Rikenellaceae	Alistipes	
ASV_1182	Bacteria	Bacteroidetes	Bacteroidia	Bacteroidales	Bacteroidaceae	Bacteroides	
ASV_1183	Bacteria	Firmicutes	Clostridia	Clostridiales	Lachnospiraceae	Unknown	
ASV_1184	Bacteria	Bacteroidetes	Bacteroidia	Bacteroidales	Muribaculaceae	Unknown	
ASV_1185	Bacteria	Firmicutes	Clostridia	Clostridiales	Lachnospiraceae	Unknown	
ASV_1186	Bacteria	Firmicutes	Clostridia	Clostridiales	Lachnospiraceae	Lachnospiraceae_NK4A136_group	
ASV_1187	Bacteria	Firmicutes	Clostridia	Clostridiales	Lachnospiraceae	Unknown	
ASV_1188	Bacteria	Deferribacteres	Deferribacteres	Deferribacterales	Deferribacteraceae	Mucispirillum	
ASV_1189	Bacteria	Firmicutes	Clostridia	Clostridiales	Ruminococcaceae	Ruminiclostridium_9	
ASV_1190	Bacteria	Firmicutes	Clostridia	Clostridiales	Lachnospiraceae	Lachnospiraceae_NK4A136_group	
ASV_1191	Bacteria	Bacteroidetes	Bacteroidia	Bacteroidales	Rikenellaceae	Alistipes	
ASV_1192	Bacteria	Firmicutes	Clostridia	Clostridiales	Lachnospiraceae	Lachnoclostridium	
ASV_1193	Bacteria	Bacteroidetes	Bacteroidia	Bacteroidales	Barnesiellaceae	Barnesiella	
ASV_1194	Bacteria	Firmicutes	Clostridia	Clostridiales	Ruminococcaceae	Subdoligranulum	
ASV_1195	Bacteria	Bacteroidetes	Bacteroidia	Bacteroidales	Bacteroidaceae	Bacteroides	
ASV_1196	Bacteria	Firmicutes	Clostridia	Clostridiales	Lachnospiraceae	Unknown	
ASV_1197	Bacteria	Firmicutes	Clostridia	Clostridiales	Ruminococcaceae	Unknown	
ASV_1198	Bacteria	Firmicutes	Clostridia	Clostridiales	Clostridiaceae_1	Clostridium_sensu_stricto_1	
ASV_1199	Bacteria	Firmicutes	Clostridia	Clostridiales	Lachnospiraceae	Unknown	
ASV_1200	Bacteria	Bacteroidetes	Bacteroidia	Bacteroidales	Bacteroidaceae	Bacteroides	Bacteroides_fragilis
ASV_1201	Bacteria	Firmicutes	Clostridia	Clostridiales	Lachnospiraceae	Lachnospiraceae_UCG-006	

ASV_1202	Bacteria	Firmicutes	Clostridia	Clostridiales	Lachnospiraceae	Roseburia	
ASV_1203	Bacteria	Firmicutes	Clostridia	Clostridiales	Ruminococcaceae	Butyricicoccus	
ASV_1204	Bacteria	Firmicutes	Clostridia	Clostridiales	Clostridiales_vadinBB60_group	Unknown	
ASV_1205	Bacteria	Firmicutes	Clostridia	Clostridiales	Lachnospiraceae	Unknown	
ASV_1206	Bacteria	Firmicutes	Clostridia	Clostridiales	Ruminococcaceae	Ruminiclostridium	
ASV_1207	Bacteria	Firmicutes	Clostridia	Clostridiales	Ruminococcaceae	Ruminococcaceae_UCG-014	
ASV_1208	Bacteria	Firmicutes	Clostridia	Clostridiales	Lachnospiraceae	Unknown	
ASV_1209	Bacteria	Firmicutes	Clostridia	Clostridiales	Ruminococcaceae	Unknown	
ASV_1210	Bacteria	Proteobacteria	Gammaproteobacteria	Enterobacteriales	Enterobacteriaceae	Escherichia/Shigella	Escherichia/Shigella_coli
ASV_1211	Bacteria	Bacteroidetes	Bacteroidia	Bacteroidales	Muribaculaceae	Unknown	
ASV_1212	Bacteria	Firmicutes	Clostridia	Clostridiales	Lachnospiraceae	GCA-900066575	
ASV_1213	Bacteria	Firmicutes	Clostridia	Clostridiales	Ruminococcaceae	Ruminococcaceae_UCG-014	
ASV_1214	Bacteria	Firmicutes	Clostridia	Clostridiales	Lachnospiraceae	Unknown	
ASV_1215	Bacteria	Bacteroidetes	Bacteroidia	Bacteroidales	Bacteroidaceae	Bacteroides	Bacteroides_thetaiotaomicron
ASV_1216	Bacteria	Firmicutes	Clostridia	Clostridiales	Ruminococcaceae	Ruminococcaceae_NK4A214_group	
ASV_1217	Bacteria	Firmicutes	Clostridia	Clostridiales	Lachnospiraceae	Lachnospiraceae_NK4A136_group	
ASV_1218	Bacteria	Bacteroidetes	Bacteroidia	Bacteroidales	Bacteroidaceae	Bacteroides	
ASV_1219	Bacteria	Bacteroidetes	Bacteroidia	Bacteroidales	Rikenellaceae	Alistipes	
ASV_1220	Bacteria	Firmicutes	Clostridia	Clostridiales	Ruminococcaceae	Ruminococcaceae_UCG-014	
ASV_1221	Bacteria	Firmicutes	Clostridia	Clostridiales	Ruminococcaceae	Ruminococcaceae_UCG-014	
ASV_1222	Bacteria	Firmicutes	Clostridia	Clostridiales	Lachnospiraceae	Lachnospiraceae_UCG-001	
ASV_1223	Bacteria	Bacteroidetes	Bacteroidia	Bacteroidales	Bacteroidaceae	Bacteroides	
ASV_1224	Bacteria	Actinobacteria	Coriobacteriia	Coriobacteriales	Eggerthellaceae	Adlercreutzia	
ASV_1225	Bacteria	Firmicutes	Clostridia	Clostridiales	Ruminococcaceae	Ruminococcaceae_UCG-014	
ASV_1226	Bacteria	Tenericutes	Mollicutes	Mollicutes_RF39	Unknown		
ASV_1227	Bacteria	Firmicutes	Erysipelotrichia	Erysipelotrichales	Erysipelotrichaceae	Faecalibaculum	
ASV_1228	Bacteria	Proteobacteria	Deltaproteobacteria	Desulfovibrionales	Desulfovibrionaceae	Desulfovibrio	Desulfovibrio_piger
ASV_1229	Bacteria	Firmicutes	Bacilli	Lactobacillales	Lactobacillaceae	Lactobacillus	
ASV_1230	Bacteria	Firmicutes	Negativicutes	Selenomonadales	Acidaminococcaceae	Phascolarctobacterium	
ASV_1231	Bacteria	Firmicutes	Clostridia	Clostridiales	Unknown		
ASV_1232	Bacteria	Firmicutes	Clostridia	Clostridiales	Lachnospiraceae	Lachnoclostridium	
ASV_1233	Bacteria	Firmicutes	Clostridia	Clostridiales	Lachnospiraceae	Unknown	
ASV_1234	Bacteria	Firmicutes	Clostridia	Clostridiales	Ruminococcaceae	Ruminiclostridium_9	
ASV_1235	Bacteria	Firmicutes	Clostridia	Clostridiales	Ruminococcaceae	Anaerotruncus	
ASV_1236	Bacteria	Firmicutes	Clostridia	Clostridiales	Family_XIII	Family_XIII_AD3011_group	
ASV_1237	Bacteria	Bacteroidetes	Bacteroidia	Bacteroidales	Rikenellaceae	Alistipes	

ASV_1238	Bacteria	Bacteroidetes	Bacteroidia	Bacteroidales	Bacteroidaceae	Bacteroides	
ASV_1239	Bacteria	Bacteroidetes	Bacteroidia	Bacteroidales	Bacteroidaceae	Bacteroides	
ASV_1240	Bacteria	Firmicutes	Clostridia	Clostridiales	Ruminococcaceae	Ruminococcaceae_UCG-014	
ASV_1241	Bacteria	Firmicutes	Clostridia	Clostridiales	Lachnospiraceae	Unknown	
ASV_1242	Bacteria	Firmicutes	Clostridia	Clostridiales	Unknown		
ASV_1243	Bacteria	Bacteroidetes	Bacteroidia	Bacteroidales	Rikenellaceae	Rikenellaceae_RC9_gut_group	
ASV_1244	Bacteria	Firmicutes	Clostridia	Clostridiales	Lachnospiraceae	Unknown	
ASV_1245	Bacteria	Firmicutes	Clostridia	Clostridiales	Ruminococcaceae	Ruminococcus_1	
ASV_1246	Bacteria	Firmicutes	Clostridia	Clostridiales	Lachnospiraceae	Unknown	
ASV_1247	Bacteria	Firmicutes	Clostridia	Clostridiales	Lachnospiraceae	Lachnoclostridium	
ASV_1248	Bacteria	Firmicutes	Clostridia	Clostridiales	Ruminococcaceae	Ruminiclostridium_9	
ASV_1249	Bacteria	Proteobacteria	Gammaproteobacteria	Betaproteobacteriales	Burkholderiaceae	Sutterella	
ASV_1250	Bacteria	Firmicutes	Clostridia	Clostridiales	Lachnospiraceae	Unknown	
ASV_1251	Bacteria	Firmicutes	Clostridia	Clostridiales	Ruminococcaceae	Harryflintia	
ASV_1252	Bacteria	Firmicutes	Clostridia	Clostridiales	Peptococcaceae	Unknown	
ASV_1253	Bacteria	Firmicutes	Clostridia	Clostridiales	Unknown		
ASV_1254	Bacteria	Firmicutes	Clostridia	Clostridiales	Lachnospiraceae	Lachnoclostridium	
ASV_1255	Bacteria	Firmicutes	Clostridia	Clostridiales	Lachnospiraceae	Unknown	
ASV_1256	Bacteria	Firmicutes	Clostridia	Clostridiales	Ruminococcaceae	Subdoligranulum	
ASV_1257	Bacteria	Firmicutes	Clostridia	Clostridiales	Ruminococcaceae	Unknown	
ASV_1258	Bacteria	Proteobacteria	Gammaproteobacteria	Enterobacteriales	Enterobacteriaceae	Escherichia/Shigella	Escherichia/Shigella_coli
ASV_1259	Bacteria	Firmicutes	Clostridia	Clostridiales	Ruminococcaceae	Ruminiclostridium_9	
ASV_1260	Bacteria	Firmicutes	Clostridia	Clostridiales	Lachnospiraceae	Unknown	
ASV_1261	Bacteria	Bacteroidetes	Bacteroidia	Bacteroidales	Bacteroidaceae	Bacteroides	
ASV_1262	Bacteria	Bacteroidetes	Bacteroidia	Bacteroidales	Prevotellaceae	Paraprevotella	
ASV_1263	Bacteria	Firmicutes	Clostridia	Clostridiales	Ruminococcaceae	Oscillibacter	
ASV_1264	Bacteria	Firmicutes	Clostridia	Clostridiales	Lachnospiraceae	Blautia	Blautia_obeum
ASV_1265	Bacteria	Firmicutes	Clostridia	Clostridiales	Ruminococcaceae	Ruminiclostridium_9	
ASV_1266	Bacteria	Firmicutes	Clostridia	Clostridiales	Lachnospiraceae	Lachnospiraceae_NK4A136_group	
ASV_1267	Bacteria	Firmicutes	Clostridia	Clostridiales	Ruminococcaceae	Ruminococcaceae_UCG-014	
ASV_1268	Bacteria	Firmicutes	Clostridia	Clostridiales	Ruminococcaceae	Ruminococcaceae_UCG-010	
ASV_1269	Bacteria	Firmicutes	Clostridia	Clostridiales	Ruminococcaceae	Butyricicoccus	
ASV_1270	Bacteria	Firmicutes	Clostridia	Clostridiales	Lachnospiraceae	Lachnospiraceae_NK4A136_group	
ASV_1271	Bacteria	Firmicutes	Negativicutes	Selenomonadales	Acidaminococcaceae	Phascolarctobacterium	
ASV_1272	Bacteria	Firmicutes	Clostridia	Clostridiales	Lachnospiraceae	Fusicatenibacter	
ASV_1273	Bacteria	Firmicutes	Clostridia	Clostridiales	Lachnospiraceae	Roseburia	

ASV_1274	Bacteria	Firmicutes	Clostridia	Clostridiales	Ruminococcaceae	Unknown	
ASV_1275	Bacteria	Deferribacteres	Deferribacteres	Deferribacterales	Deferribacteraceae	Mucispirillum	
ASV_1276	Bacteria	Bacteroidetes	Bacteroidia	Bacteroidales	Tannerellaceae	Parabacteroides	
ASV_1277	Bacteria	Bacteroidetes	Bacteroidia	Bacteroidales	Rikenellaceae	Alistipes	Alistipes_shahii
ASV_1278	Bacteria	Bacteroidetes	Bacteroidia	Bacteroidales	Muribaculaceae	Unknown	
ASV_1279	Bacteria	Firmicutes	Negativicutes	Selenomonadales	Veillonellaceae	Dialister	
ASV_1280	Bacteria	Firmicutes	Clostridia	Clostridiales	Lachnospiraceae	Unknown	
ASV_1281	Bacteria	Firmicutes	Clostridia	Clostridiales	Lachnospiraceae	Unknown	
ASV_1282	Bacteria	Firmicutes	Clostridia	Clostridiales	Ruminococcaceae	Ruminococcaceae_UCG-014	
ASV_1283	Bacteria	Firmicutes	Clostridia	Clostridiales	Lachnospiraceae	Lachnospiraceae_NK4A136_group	
ASV_1284	Bacteria	Firmicutes	Clostridia	Clostridiales	Ruminococcaceae	Ruminiclostridium_9	
ASV_1285	Bacteria	Bacteroidetes	Bacteroidia	Bacteroidales	Rikenellaceae	Alistipes	
ASV_1286	Bacteria	Firmicutes	Clostridia	Clostridiales	Lachnospiraceae	Unknown	
ASV_1287	Bacteria	Firmicutes	Clostridia	Clostridiales	Lachnospiraceae	Lachnoclostridium	
ASV_1288	Bacteria	Proteobacteria	Alphaproteobacteria	Rhodospirillales	Unknown		
ASV_1289	Bacteria	Bacteroidetes	Bacteroidia	Bacteroidales	Barnesiellaceae	Barnesiella	
ASV_1290	Bacteria	Firmicutes	Clostridia	Clostridiales	Family_XIII	Unknown	
ASV_1291	Bacteria	Firmicutes	Clostridia	Clostridiales	Eubacteriaceae	Anaerofustis	
ASV_1292	Bacteria	Bacteroidetes	Bacteroidia	Bacteroidales	Barnesiellaceae	Barnesiella	
ASV_1293	Bacteria	Bacteroidetes	Bacteroidia	Bacteroidales	Bacteroidaceae	Bacteroides	
ASV_1294	Bacteria	Firmicutes	Clostridia	Clostridiales	Lachnospiraceae	A2	
ASV_1295	Bacteria	Firmicutes	Clostridia	Clostridiales	Lachnospiraceae	Coprococcus_3	Coprococcus_3_comes
ASV_1296	Bacteria	Firmicutes	Clostridia	Clostridiales	Lachnospiraceae	Lachnoclostridium	
ASV_1297	Bacteria	Firmicutes	Clostridia	Clostridiales	Lachnospiraceae	Unknown	
ASV_1298	Bacteria			Unknown			
ASV_1299	Bacteria	Firmicutes	Negativicutes	Selenomonadales	Veillonellaceae	Megamonas	
ASV_1300	Bacteria	Firmicutes	Clostridia	Clostridiales	Ruminococcaceae	Ruminococcaceae_UCG-014	
ASV_1301	Bacteria	Bacteroidetes	Bacteroidia	Bacteroidales	Prevotellaceae	Prevotella_9	
ASV_1302	Bacteria	Firmicutes	Clostridia	Clostridiales	Clostridiales_vadinBB60_group	Unknown	
ASV_1303	Bacteria	Firmicutes	Clostridia	Clostridiales	Lachnospiraceae	Unknown	
ASV_1304	Bacteria	Firmicutes	Clostridia	Clostridiales	Clostridiales_vadinBB60_group	Unknown	
ASV_1305	Bacteria	Bacteroidetes	Bacteroidia	Bacteroidales	Muribaculaceae	Muribaculum	
ASV_1306	Bacteria	Bacteroidetes	Bacteroidia	Bacteroidales	Muribaculaceae	Unknown	
ASV_1307	Bacteria	Firmicutes	Clostridia	Clostridiales	Lachnospiraceae	Lachnospiraceae_UCG-006	
ASV_1308	Bacteria	Bacteroidetes	Bacteroidia	Bacteroidales	Muribaculaceae	Unknown	
ASV_1309	Bacteria	Firmicutes	Clostridia	Clostridiales	Lachnospiraceae	Unknown	
ASV_1310	Bacteria	Tenericutes	Mollicutes	Mollicutes_RF39	Unknown		

ASV_1311	Bacteria	Bacteroidetes	Bacteroidia	Bacteroidales	Muribaculaceae	Unknown	
ASV_1312	Bacteria	Firmicutes	Clostridia	Clostridiales	Lachnospiraceae	Unknown	
ASV_1313	Bacteria	Firmicutes	Negativicutes	Selenomonadales	Acidaminococcaceae	Phascolarctobacterium	
ASV_1314	Bacteria	Bacteroidetes	Bacteroidia	Bacteroidales	Bacteroidaceae	Bacteroides	
ASV_1315	Bacteria	Firmicutes	Clostridia	Clostridiales	Lachnospiraceae	Unknown	
ASV_1316	Bacteria	Firmicutes	Clostridia	Clostridiales	Ruminococcaceae	Ruminococcaceae_UCG-013	
ASV_1317	Bacteria	Firmicutes	Clostridia	Clostridiales	Ruminococcaceae	Ruminococcaceae_UCG-014	
ASV_1318	Bacteria	Bacteroidetes	Bacteroidia	Bacteroidales	Bacteroidaceae	Bacteroides	Bacteroides_coprophilus
ASV_1319	Bacteria	Firmicutes	Clostridia	Clostridiales	Ruminococcaceae	Ruminiclostridium	
ASV_1320	Bacteria	Bacteroidetes	Bacteroidia	Bacteroidales	Rikenellaceae	Alistipes	
ASV_1321	Bacteria	Firmicutes	Clostridia	Clostridiales	Ruminococcaceae	Ruminococcaceae_UCG-014	
ASV_1322	Bacteria	Firmicutes	Clostridia	Clostridiales	Ruminococcaceae	Unknown	
ASV_1323	Bacteria	Actinobacteria	Coriobacteriia	Coriobacteriales	Eggerthellaceae	Enterorhabdus	Enterorhabdus_mucosicola
ASV_1324	Bacteria	Firmicutes	Clostridia	Clostridiales	Ruminococcaceae	Ruminococcaceae_UCG-014	
ASV_1325	Bacteria	Firmicutes	Clostridia	Clostridiales	Ruminococcaceae	Ruminococcaceae_UCG-014	
ASV_1326	Bacteria	Firmicutes	Clostridia	Clostridiales	Lachnospiraceae	Unknown	
ASV_1327	Bacteria	Firmicutes	Clostridia	Clostridiales	Ruminococcaceae	Unknown	
ASV_1328	Bacteria	Firmicutes	Clostridia	Clostridiales	Lachnospiraceae	Unknown	
ASV_1329	Bacteria	Firmicutes	Clostridia	Clostridiales	Ruminococcaceae	Ruminiclostridium_9	
ASV_1330	Bacteria	Firmicutes	Clostridia	Clostridiales	Ruminococcaceae	Butyricicoccus	
ASV_1331	Bacteria	Firmicutes	Clostridia	Clostridiales	Ruminococcaceae	Unknown	
ASV_1332	Bacteria	Deferribacteres	Deferribacteres	Deferribacterales	Deferribacteraceae	Mucispirillum	
ASV_1333	Bacteria	Tenericutes	Mollicutes	Mollicutes_RF39	Unknown		
ASV_1334	Bacteria	Firmicutes	Clostridia	Clostridiales	Lachnospiraceae	Unknown	
ASV_1335	Bacteria	Firmicutes	Clostridia	Clostridiales	Clostridiales_vadinBB60_group	Unknown	
ASV_1336	Bacteria	Firmicutes	Negativicutes	Selenomonadales	Veillonellaceae	Dialister	
ASV_1337	Bacteria	Bacteroidetes	Bacteroidia	Bacteroidales	Bacteroidaceae	Bacteroides	
ASV_1338	Bacteria	Firmicutes	Clostridia	Clostridiales	Ruminococcaceae	Ruminococcaceae_NK4A214_group	
ASV_1339	Bacteria	Firmicutes	Clostridia	Clostridiales	Lachnospiraceae	Lachnospiraceae_NK4A136_group	
ASV_1340	Bacteria	Tenericutes	Mollicutes	Mollicutes_RF39	Unknown		
ASV_1341	Bacteria	Firmicutes	Clostridia	Clostridiales	Ruminococcaceae	Ruminococcaceae_UCG-013	
ASV_1342	Bacteria	Firmicutes	Clostridia	Clostridiales	Lachnospiraceae	Unknown	
ASV_1343	Bacteria	Firmicutes	Clostridia	Clostridiales	Ruminococcaceae	Ruminiclostridium_6	
ASV_1344	Bacteria	Bacteroidetes	Bacteroidia	Bacteroidales	Prevotellaceae	Prevotella_7	Prevotella_7_buccae
ASV_1345	Bacteria	Bacteroidetes	Bacteroidia	Bacteroidales	Muribaculaceae	Unknown	
ASV_1346	Bacteria	Bacteroidetes	Bacteroidia	Bacteroidales	Prevotellaceae	Prevotellaceae_NK3B31_group	
ASV_1347	Bacteria	Bacteroidetes	Bacteroidia	Bacteroidales	Bacteroidaceae	Bacteroides	

ASV_1348	Bacteria	Firmicutes	Clostridia	Clostridiales	Lachnospiraceae	Unknown	
ASV_1349	Bacteria	Firmicutes	Clostridia	Clostridiales	Ruminococcaceae	Unknown	
ASV_1350	Bacteria	Firmicutes	Bacilli	Bacillales	Staphylococcaceae	Staphylococcus	Staphylococcus_lentus
ASV_1351	Bacteria	Firmicutes	Clostridia	Clostridiales	Ruminococcaceae	Ruminococcaceae_UCG-013	
ASV_1352	Bacteria	Bacteroidetes	Bacteroidia	Bacteroidales	Muribaculaceae	Unknown	
ASV_1353	Bacteria	Bacteroidetes	Bacteroidia	Bacteroidales	Muribaculaceae	Unknown	
ASV_1354	Bacteria	Firmicutes	Erysipelotrichia	Erysipelotrichales	Erysipelotrichaceae	Catenibacterium	
ASV_1355	Bacteria	Firmicutes	Clostridia	Clostridiales	Lachnospiraceae	Lachnospiraceae_NK4A136_group	
ASV_1356	Bacteria	Proteobacteria	Deltaproteobacteria	Desulfovibrionales	Desulfovibrionaceae	Bilophila	Bilophila_wadsworthia
ASV_1357	Bacteria	Firmicutes	Clostridia	Clostridiales	Ruminococcaceae	Ruminococcus_2	
ASV_1358	Bacteria	Firmicutes	Clostridia	Clostridiales	Lachnospiraceae	Lachnospiraceae_NK4A136_group	
ASV_1359	Bacteria	Bacteroidetes	Bacteroidia	Bacteroidales	Bacteroidaceae	Bacteroides	
ASV_1360	Bacteria	Bacteroidetes	Bacteroidia	Bacteroidales	Muribaculaceae	Unknown	
ASV_1361	Bacteria	Bacteroidetes	Bacteroidia	Flavobacteriales	Weeksellaceae	Cloacibacterium	
ASV_1362	Bacteria	Bacteroidetes	Bacteroidia	Bacteroidales	Rikenellaceae	Alistipes	
ASV_1363	Bacteria	Firmicutes	Clostridia	Clostridiales	Lachnospiraceae	Lachnospiraceae_UCG-006	
ASV_1364	Bacteria	Firmicutes	Clostridia	Clostridiales	Lachnospiraceae	Unknown	
ASV_1365	Bacteria	Firmicutes	Bacilli	Lactobacillales	Lactobacillaceae	Lactobacillus	
ASV_1366	Bacteria	Firmicutes	Clostridia	Clostridiales	Ruminococcaceae	Ruminococcaceae_UCG-005	
ASV_1367	Bacteria	Firmicutes	Clostridia	Clostridiales	Ruminococcaceae	Ruminococcaceae_UCG-014	
ASV_1368	Bacteria	Firmicutes	Clostridia	Clostridiales	Ruminococcaceae	Ruminiclostridium	
ASV_1369	Bacteria	Firmicutes	Clostridia	Clostridiales	Lachnospiraceae	Lachnospiraceae_UCG-001	
ASV_1370	Bacteria	Firmicutes	Clostridia	Clostridiales	Clostridiales_vadinBB60_group	Unknown	
ASV_1371	Bacteria	Bacteroidetes	Bacteroidia	Bacteroidales	Bacteroidaceae	Bacteroides	
ASV_1372	Bacteria	Firmicutes	Erysipelotrichia	Erysipelotrichales	Erysipelotrichaceae	Erysipelotrichaceae_UCG-003	
ASV_1373	Bacteria	Bacteroidetes	Bacteroidia	Bacteroidales	Bacteroidaceae	Bacteroides	
ASV_1374	Bacteria	Firmicutes	Clostridia	Clostridiales	Ruminococcaceae	Ruminococcaceae_UCG-005	
ASV_1375	Bacteria	Firmicutes	Clostridia	Clostridiales	Ruminococcaceae	Ruminococcaceae_NK4A214_group	
ASV_1376	Bacteria	Bacteroidetes	Bacteroidia	Bacteroidales	Bacteroidaceae	Bacteroides	
ASV_1377	Bacteria	Firmicutes	Clostridia	Clostridiales	Lachnospiraceae	Lachnospira	
ASV_1378	Bacteria	Bacteroidetes	Bacteroidia	Bacteroidales	Prevotellaceae	Prevotellaceae_UCG-001	
ASV_1379	Bacteria	Firmicutes	Clostridia	Clostridiales	Ruminococcaceae	Ruminiclostridium_9	
ASV_1380	Bacteria	Firmicutes	Clostridia	Clostridiales	Ruminococcaceae	Ruminococcaceae_NK4A214_group	
ASV_1381	Bacteria	Bacteroidetes	Bacteroidia	Bacteroidales	Rikenellaceae	Alistipes	
ASV_1382	Bacteria	Tenericutes	Mollicutes	Mollicutes_RF39	Unknown		
ASV_1383	Bacteria	Tenericutes	Mollicutes	Mollicutes_RF39	Unknown		
ASV_1384	Bacteria	Tenericutes	Mollicutes	Mollicutes_RF39	Unknown		

ASV_1385	Bacteria	Firmicutes	Clostridia	Clostridiales	Lachnospiraceae	Lachnospiraceae_NK4A136_group
ASV_1386	Bacteria	Tenericutes	Mollicutes	Mollicutes_RF39	Unknown	
ASV_1387	Bacteria	Firmicutes	Clostridia	Clostridiales	Lachnospiraceae	Unknown
ASV_1388	Bacteria	Firmicutes	Bacilli	Lactobacillales	Lactobacillaceae	Lactobacillus
ASV_1389	Bacteria	Firmicutes	Clostridia	Clostridiales	Ruminococcaceae	Anaerotruncus
ASV_1390	Bacteria	Firmicutes	Clostridia	Clostridiales	Ruminococcaceae	Unknown
ASV_1391	Bacteria	Firmicutes	Clostridia	Clostridiales	Lachnospiraceae	Unknown
ASV_1392	Bacteria	Firmicutes	Clostridia	Clostridiales	Ruminococcaceae	Oscillibacter
ASV_1393	Bacteria	Tenericutes	Mollicutes	Izimaplasmatales	Unknown	
ASV_1394	Bacteria	Firmicutes	Clostridia	Clostridiales	Ruminococcaceae	Subdoligranulum
ASV_1395	Bacteria	Firmicutes	Clostridia	Clostridiales	Defluviitaleaceae	Defluviitaleaceae_UCG-011
ASV_1396	Bacteria	Firmicutes	Clostridia	Clostridiales	Ruminococcaceae	Ruminiclostridium_9
ASV_1397	Bacteria	Bacteroidetes	Bacteroidia	Bacteroidales	Muribaculaceae	Unknown
ASV_1398	Bacteria	Firmicutes	Clostridia	Clostridiales	Lachnospiraceae	Lachnospiraceae_NK4A136_group
ASV_1399	Bacteria	Bacteroidetes	Bacteroidia	Bacteroidales	Muribaculaceae	Unknown
ASV_1400	Bacteria	Bacteroidetes	Bacteroidia	Bacteroidales	Muribaculaceae	Unknown
ASV_1401	Bacteria	Firmicutes	Clostridia	Clostridiales	Lachnospiraceae	Unknown
ASV_1402	Bacteria	Firmicutes	Clostridia	Clostridiales	Lachnospiraceae	Lachnospiraceae_NK4A136_group
ASV_1403	Bacteria	Firmicutes	Bacilli	Lactobacillales	Lactobacillaceae	Lactobacillus
ASV_1404	Bacteria	Bacteroidetes	Bacteroidia	Bacteroidales	Rikenellaceae	Alistipes
ASV_1405	Bacteria	Firmicutes	Clostridia	Clostridiales	Lachnospiraceae	Unknown
ASV_1406	Bacteria	Firmicutes	Erysipelotrichia	Erysipelotrichales	Erysipelotrichaceae	Unknown
ASV_1407	Bacteria	Firmicutes	Clostridia	Clostridiales	Lachnospiraceae	Unknown
ASV_1408	Bacteria	Deferribacteres	Deferribacteres	Deferribacterales	Deferribacteraceae	Mucispirillum
ASV_1409	Bacteria	Firmicutes	Clostridia	Clostridiales	Lachnospiraceae	Unknown
ASV_1410	Bacteria	Bacteroidetes	Bacteroidia	Bacteroidales	Rikenellaceae	Alistipes
ASV_1411	Bacteria	Bacteroidetes	Bacteroidia	Bacteroidales	Prevotellaceae	Prevotellaceae_UCG-001
ASV_1412	Bacteria	Verrucomicrobia	Verrucomicrobiae	Verrucomicrobiales	Akkermansiaceae	Akkermansia
ASV_1413	Bacteria	Bacteroidetes	Bacteroidia	Bacteroidales	Bacteroidaceae	Bacteroides
ASV_1414	Bacteria	Firmicutes	Clostridia	Clostridiales	Lachnospiraceae	Unknown
ASV_1415	Bacteria	Firmicutes	Clostridia	Clostridiales	Lachnospiraceae	Unknown
ASV_1416	Bacteria	Firmicutes	Clostridia	Clostridiales	Lachnospiraceae	Unknown
ASV_1417	Bacteria	Bacteroidetes	Bacteroidia	Bacteroidales	Unknown	
ASV_1418	Bacteria	Firmicutes	Bacilli	Lactobacillales	Lactobacillaceae	Lactobacillus
ASV_1419	Bacteria	Firmicutes	Clostridia	Clostridiales	Ruminococcaceae	Ruminococcaceae_UCG-014
ASV_1420	Bacteria	Firmicutes	Clostridia	Clostridiales	Clostridiales_vadinBB60_group	Unknown
ASV_1421	Bacteria	Firmicutes	Clostridia	Clostridiales	Lachnospiraceae	Unknown

ASV_1422	Bacteria	Firmicutes	Clostridia	Clostridiales	Ruminococcaceae	Ruminococcaceae_UCG-010
ASV_1423	Bacteria	Firmicutes	Clostridia	Clostridiales	Christensenellaceae	Christensenellaceae_R-7_group
ASV_1424	Bacteria	Firmicutes	Clostridia	Clostridiales	Lachnospiraceae	Lachnoclostridium
ASV_1425	Bacteria	Firmicutes	Erysipelotrichia	Erysipelotrichales	Erysipelotrichaceae	Holdemanella
ASV_1426	Bacteria	Firmicutes	Clostridia	Clostridiales	Lachnospiraceae	Lachnoclostridium
ASV_1427	Bacteria	Firmicutes	Clostridia	Clostridiales	Lachnospiraceae	A2
ASV_1428	Bacteria	Firmicutes	Clostridia	Clostridiales	Clostridiales_vadinBB60_group	Unknown
ASV_1429	Bacteria	Firmicutes	Clostridia	Clostridiales	Ruminococcaceae	GCA-900066225
ASV_1430	Bacteria	Firmicutes	Clostridia	Clostridiales	Ruminococcaceae	UBA1819
ASV_1431	Bacteria	Firmicutes	Clostridia	Clostridiales	Lachnospiraceae	Lachnospiraceae_NK4A136_group
ASV_1432	Bacteria	Proteobacteria	Alphaproteobacteria	Rhodospirillales	Unknown	
ASV_1433	Bacteria	Firmicutes	Clostridia	Clostridiales	Ruminococcaceae	Ruminococcaceae_UCG-014
ASV_1434	Bacteria	Firmicutes	Clostridia	Clostridiales	Lachnospiraceae	Lachnospiraceae_FCS020_group
ASV_1435	Bacteria	Bacteroidetes	Bacteroidia	Bacteroidales	Muribaculaceae	Unknown
ASV_1436	Bacteria	Firmicutes	Clostridia	Clostridiales	Ruminococcaceae	Ruminococcaceae_NK4A214_group
ASV_1437	Bacteria	Firmicutes	Clostridia	Clostridiales	Ruminococcaceae	Ruminococcaceae_UCG-002
ASV_1438	Bacteria	Firmicutes	Clostridia	Clostridiales	Lachnospiraceae	Unknown
ASV_1439	Bacteria	Bacteroidetes	Bacteroidia	Bacteroidales	Muribaculaceae	Unknown
ASV_1440	Bacteria	Bacteroidetes	Bacteroidia	Bacteroidales	Muribaculaceae	Unknown
ASV_1441	Bacteria	Firmicutes	Bacilli	Lactobacillales	Lactobacillaceae	Lactobacillus
ASV_1442	Bacteria	Firmicutes	Clostridia	Clostridiales	Lachnospiraceae	GCA-900066575
ASV_1443	Bacteria	Firmicutes	Clostridia	Clostridiales	Lachnospiraceae	A2
ASV_1444	Bacteria	Firmicutes	Clostridia	Clostridiales	Ruminococcaceae	Ruminococcaceae_UCG-014
ASV_1445	Bacteria	Firmicutes	Clostridia	Clostridiales	Ruminococcaceae	Ruminiclostridium_5
ASV_1446	Bacteria	Firmicutes	Unknown			
ASV_1447	Bacteria	Firmicutes	Clostridia	Clostridiales	Ruminococcaceae	Oscillibacter
ASV_1448	Bacteria	Firmicutes	Clostridia	Clostridiales	Family_XI	Anaerococcus
ASV_1449	Bacteria	Firmicutes	Clostridia	Clostridiales	Ruminococcaceae	Ruminococcaceae_UCG-014
ASV_1450	Bacteria	Firmicutes	Clostridia	Clostridiales	Ruminococcaceae	Ruminococcaceae_UCG-014
ASV_1451	Bacteria	Bacteroidetes	Bacteroidia	Bacteroidales	Bacteroidaceae	Bacteroides
ASV_1452	Bacteria	Firmicutes	Clostridia	Clostridiales	Lachnospiraceae	Lachnospira
ASV_1453	Bacteria	Firmicutes	Clostridia	Clostridiales	Lachnospiraceae	Lachnoclostridium
ASV_1454	Bacteria	Bacteroidetes	Bacteroidia	Bacteroidales	Bacteroidaceae	Bacteroides
ASV_1455	Bacteria	Firmicutes	Clostridia	Clostridiales	Ruminococcaceae	UBA1819
ASV_1456	Bacteria	Firmicutes	Clostridia	Clostridiales	Lachnospiraceae	Unknown
ASV_1457	Bacteria	Bacteroidetes	Bacteroidia	Bacteroidales	Muribaculaceae	Unknown
ASV_1458	Bacteria	Firmicutes	Clostridia	Clostridiales	Lachnospiraceae	Unknown

ASV_1459	Bacteria	Firmicutes	Bacilli	Lactobacillales	Lactobacillaceae	Lactobacillus	
ASV_1460	Bacteria	Verrucomicrobia	Verrucomicrobiae	Verrucomicrobiales	Akkermansiaceae	Akkermansia	
ASV_1461	Bacteria	Firmicutes	Clostridia	Clostridiales	Lachnospiraceae	Lachnoclostridium	
ASV_1462	Bacteria	Firmicutes	Clostridia	Clostridiales	Lachnospiraceae	Lachnospiraceae_NK4A136_group	
ASV_1463	Bacteria	Bacteroidetes	Bacteroidia	Bacteroidales	Muribaculaceae	Muribaculum	
ASV_1464	Bacteria	Bacteroidetes	Bacteroidia	Bacteroidales	Muribaculaceae	Muribaculum	
ASV_1465	Bacteria	Firmicutes	Clostridia	Clostridiales	Lachnospiraceae	ASF356	
ASV_1466	Bacteria	Firmicutes	Clostridia	Clostridiales	Lachnospiraceae	Unknown	
ASV_1467	Bacteria	Firmicutes	Bacilli	Lactobacillales	Lactobacillaceae	Lactobacillus	
ASV_1468	Bacteria	Firmicutes	Clostridia	Clostridiales	Lachnospiraceae	Unknown	
ASV_1469	Bacteria	Firmicutes	Clostridia	Clostridiales	Lachnospiraceae	Lachnospira	
ASV_1470	Bacteria	Firmicutes	Clostridia	Clostridiales	Ruminococcaceae	Ruminococcaceae_UCG-014	
ASV_1471	Bacteria	Firmicutes	Clostridia	Clostridiales	Lachnospiraceae	Unknown	
ASV_1472	Bacteria	Firmicutes	Clostridia	Clostridiales	Ruminococcaceae	Ruminococcaceae_UCG-014	
ASV_1473	Bacteria	Firmicutes	Clostridia	Clostridiales	Lachnospiraceae	Unknown	
ASV_1474	Bacteria	Firmicutes	Clostridia	Clostridiales	Lachnospiraceae	Lachnospiraceae_UCG-006	
ASV_1475	Bacteria	Firmicutes	Clostridia	Clostridiales	Ruminococcaceae	Ruminiclostridium_6	
ASV_1476	Bacteria			Unknown			
ASV_1477	Bacteria	Bacteroidetes	Bacteroidia	Bacteroidales	Rikenellaceae	Alistipes	Alistipes_inops
ASV_1478	Bacteria	Firmicutes	Bacilli	Lactobacillales	Lactobacillaceae	Lactobacillus	
ASV_1479	Bacteria	Firmicutes	Clostridia	Clostridiales	Lachnospiraceae	Unknown	
ASV_1480	Bacteria	Firmicutes	Clostridia	Clostridiales	Ruminococcaceae	Unknown	
ASV_1481	Bacteria	Firmicutes	Clostridia	Clostridiales	Lachnospiraceae	Unknown	
ASV_1482	Bacteria	Firmicutes	Clostridia	Clostridiales	Ruminococcaceae	Oscillibacter	
ASV_1483	Bacteria	Firmicutes	Clostridia	Clostridiales	Lachnospiraceae	Unknown	
ASV_1484	Bacteria	Tenericutes	Mollicutes	Mollicutes_RF39	Unknown		
ASV_1485	Bacteria	Firmicutes	Clostridia	Clostridiales	Lachnospiraceae	Lachnospiraceae_NK4A136_group	
ASV_1486	Bacteria	Firmicutes	Clostridia	Clostridiales	Ruminococcaceae	Ruminiclostridium_9	
ASV_1487	Bacteria	Firmicutes	Bacilli	Lactobacillales	Lactobacillaceae	Lactobacillus	
ASV_1488	Bacteria	Firmicutes	Clostridia	Clostridiales	Lachnospiraceae	Unknown	
ASV_1489	Bacteria	Firmicutes	Clostridia	Clostridiales	Lachnospiraceae	Lachnospiraceae_NK4A136_group	
ASV_1490	Bacteria	Firmicutes	Clostridia	Clostridiales	Lachnospiraceae	Unknown	
ASV_1491	Bacteria	Firmicutes	Clostridia	Clostridiales	Lachnospiraceae	Unknown	
ASV_1492	Bacteria	Firmicutes	Clostridia	Clostridiales	Lachnospiraceae	Unknown	
ASV_1493	Bacteria	Firmicutes	Clostridia	Clostridiales	Ruminococcaceae	Unknown	
ASV_1494	Bacteria	Firmicutes	Clostridia	Clostridiales	Christensenellaceae	Christensenellaceae_R-7_group	
ASV_1495	Bacteria	Bacteroidetes	Bacteroidia	Bacteroidales	Prevotellaceae	Prevotellaceae_NK3B31_group	

ASV_1496	Bacteria	Firmicutes	Clostridia	Clostridiales	Ruminococcaceae	Ruminococcaceae_UCG-014
ASV_1497	Bacteria	Firmicutes	Clostridia	Clostridiales	Lachnospiraceae	Lachnospiraceae_FCS020_group
ASV_1498	Bacteria	Bacteroidetes	Bacteroidia	Bacteroidales	Muribaculaceae	Unknown
ASV_1499	Bacteria	Firmicutes	Clostridia	Clostridiales	Lachnospiraceae	Unknown
ASV_1500	Bacteria	Deferribacteres	Deferribacteres	Deferribacterales	Deferribacteraceae	Mucispirillum
ASV_1501	Bacteria	Firmicutes	Clostridia	Clostridiales	Ruminococcaceae	Ruminiclostridium
ASV_1502	Bacteria	Firmicutes	Clostridia	Clostridiales	Lachnospiraceae	Marvinbryantia
ASV_1503	Bacteria	Firmicutes	Clostridia	Clostridiales	Ruminococcaceae	Ruminiclostridium_5
ASV_1504	Bacteria	Bacteroidetes	Bacteroidia	Bacteroidales	Bacteroidaceae	Bacteroides
ASV_1505	Bacteria			Unknown		
ASV_1506	Bacteria	Firmicutes	Erysipelotrichia	Erysipelotrichales	Erysipelotrichaceae	Erysipelotrichaceae_UCG-003
ASV_1507	Bacteria	Firmicutes	Clostridia	Clostridiales	Ruminococcaceae	Unknown
ASV_1508	Bacteria	Firmicutes	Clostridia	Clostridiales	Lachnospiraceae	Unknown
ASV_1509	Bacteria	Firmicutes	Clostridia	Clostridiales	Lachnospiraceae	Lachnospiraceae_UCG-010
ASV_1510	Bacteria	Firmicutes	Clostridia	Clostridiales	Ruminococcaceae	Ruminococcaceae_UCG-014
ASV_1511	Bacteria	Firmicutes	Clostridia	Clostridiales	Ruminococcaceae	Ruminococcaceae_UCG-014
ASV_1512	Bacteria	Deferribacteres	Deferribacteres	Deferribacterales	Deferribacteraceae	Mucispirillum
ASV_1513	Bacteria	Firmicutes	Clostridia	Clostridiales	Ruminococcaceae	Ruminococcaceae_UCG-014
ASV_1514	Bacteria	Tenericutes	Mollicutes	Mollicutes_RF39	Unknown	
ASV_1515	Bacteria	Firmicutes	Clostridia	Clostridiales	Lachnospiraceae	Unknown
ASV_1516	Bacteria	Firmicutes	Clostridia	Clostridiales	Ruminococcaceae	Butyricicoccus
ASV_1517	Bacteria	Tenericutes	Mollicutes	Mollicutes_RF39	Unknown	
ASV_1518	Bacteria	Bacteroidetes	Bacteroidia	Bacteroidales	Rikenellaceae	Alistipes
ASV_1519	Bacteria	Firmicutes	Clostridia	Clostridiales	Ruminococcaceae	Ruminococcaceae_UCG-014
ASV_1520	Bacteria	Firmicutes	Clostridia	Clostridiales	Lachnospiraceae	GCA-900066575
ASV_1521	Bacteria	Firmicutes	Clostridia	Clostridiales	Lachnospiraceae	GCA-900066575
ASV_1522	Bacteria	Bacteroidetes	Bacteroidia	Bacteroidales	Rikenellaceae	Alistipes
ASV_1523	Bacteria	Bacteroidetes	Bacteroidia	Bacteroidales	Rikenellaceae	Alistipes
ASV_1524	Bacteria	Firmicutes	Bacilli	Lactobacillales	Unknown	
ASV_1525	Bacteria	Bacteroidetes	Bacteroidia	Bacteroidales	Muribaculaceae	Unknown
ASV_1526	Bacteria	Bacteroidetes	Bacteroidia	Bacteroidales	Muribaculaceae	Unknown
ASV_1527	Bacteria	Tenericutes	Mollicutes	Mollicutes_RF39	Unknown	
ASV_1528	Bacteria	Firmicutes	Clostridia	Clostridiales	Lachnospiraceae	Lachnospiraceae_NK4A136_group
ASV_1529	Bacteria	Bacteroidetes	Bacteroidia	Bacteroidales	Tannerellaceae	Parabacteroides
ASV_1530	Bacteria	Firmicutes	Clostridia	Clostridiales	Peptostreptococcaceae	Intestinibacter
ASV_1531	Bacteria	Firmicutes	Clostridia	Clostridiales	Lachnospiraceae	Coprococcus_2
ASV_1532	Bacteria	Bacteroidetes	Bacteroidia	Bacteroidales	Muribaculaceae	Muribaculum

ASV_1533	Bacteria	Bacteroidetes	Bacteroidia	Bacteroidales	Muribaculaceae	Unknown
ASV_1534	Bacteria	Firmicutes	Clostridia	Clostridiales	Ruminococcaceae	Unknown
ASV_1535	Bacteria	Firmicutes	Bacilli	Lactobacillales	Lactobacillaceae	Lactobacillus
ASV_1536	Bacteria	Firmicutes	Clostridia	Clostridiales	Lachnospiraceae	Unknown
ASV_1537	Bacteria	Firmicutes	Bacilli	Lactobacillales	Lactobacillaceae	Lactobacillus
ASV_1538	Bacteria	Bacteroidetes	Bacteroidia	Bacteroidales	Rikenellaceae	Alistipes
ASV_1539	Bacteria	Firmicutes	Clostridia	Clostridiales	Ruminococcaceae	Ruminiclostridium_9
ASV_1540	Bacteria	Firmicutes	Clostridia	Clostridiales	Ruminococcaceae	Unknown
ASV_1541	Bacteria	Verrucomicrobia	Verrucomicrobiae	Verrucomicrobiales	Akkermansiaceae	Akkermansia
ASV_1542	Bacteria	Bacteroidetes	Bacteroidia	Bacteroidales	Unknown	
ASV_1543	Bacteria	Bacteroidetes	Bacteroidia	Bacteroidales	Prevotellaceae	Prevotellaceae_UCG-001
ASV_1544	Bacteria	Firmicutes	Clostridia	Clostridiales	Lachnospiraceae	Lachnospiraceae_NK4A136_group
ASV_1545	Bacteria			Unknown		
ASV_1546	Bacteria	Firmicutes	Clostridia	Clostridiales	Clostridiales_vadinBB60_group	Unknown
ASV_1547	Bacteria	Bacteroidetes	Bacteroidia	Bacteroidales	Prevotellaceae	Prevotellaceae_UCG-001
ASV_1548	Bacteria	Firmicutes	Bacilli	Lactobacillales	Lactobacillaceae	Lactobacillus
ASV_1549	Bacteria	Firmicutes	Clostridia	Clostridiales	Lachnospiraceae	A2
ASV_1550	Bacteria	Bacteroidetes	Bacteroidia	Bacteroidales	Muribaculaceae	Unknown
ASV_1551	Bacteria	Bacteroidetes	Bacteroidia	Bacteroidales	Bacteroidaceae	Bacteroides
ASV_1552	Bacteria	Firmicutes	Clostridia	Clostridiales	Lachnospiraceae	Lachnoclostridium
ASV_1553	Bacteria	Firmicutes	Clostridia	Clostridiales	Ruminococcaceae	Butyricicoccus
ASV_1554	Bacteria	Firmicutes	Clostridia	Clostridiales	Lachnospiraceae	Lachnospiraceae_NK4A136_group
ASV_1555	Bacteria	Deferribacteres	Deferribacteres	Deferribacterales	Deferribacteraceae	Mucispirillum
ASV_1556	Bacteria	Firmicutes	Clostridia	Clostridiales	Ruminococcaceae	Ruminococcaceae_UCG-014
ASV_1557	Bacteria	Firmicutes	Clostridia	Clostridiales	Ruminococcaceae	Ruminiclostridium_9
ASV_1558	Bacteria	Bacteroidetes	Bacteroidia	Bacteroidales	Rikenellaceae	Alistipes
ASV_1559	Bacteria	Firmicutes	Clostridia	Clostridiales	Lachnospiraceae	Lachnoclostridium
ASV_1560	Bacteria	Bacteroidetes	Bacteroidia	Bacteroidales	Prevotellaceae	Prevotellaceae_NK3B31_group
ASV_1561	Bacteria	Bacteroidetes	Bacteroidia	Bacteroidales	Muribaculaceae	Unknown
ASV_1562	Bacteria	Firmicutes	Bacilli	Lactobacillales	Lactobacillaceae	Lactobacillus
ASV_1563	Bacteria	Firmicutes	Clostridia	Clostridiales	Ruminococcaceae	Ruminiclostridium
ASV_1564	Bacteria	Firmicutes	Clostridia	Clostridiales	Lachnospiraceae	Unknown
ASV_1565	Bacteria	Firmicutes	Clostridia	Clostridiales	Lachnospiraceae	Unknown
ASV_1566	Bacteria	Firmicutes	Clostridia	Clostridiales	Lachnospiraceae	Unknown
ASV_1567	Bacteria	Firmicutes	Clostridia	Clostridiales	Ruminococcaceae	Unknown
ASV_1568	Bacteria	Firmicutes	Clostridia	Clostridiales	Lachnospiraceae	Lachnospiraceae_NK4A136_group
ASV_1569	Bacteria	Bacteroidetes	Bacteroidia	Bacteroidales	Muribaculaceae	Unknown

ASV_1570	Bacteria	Firmicutes	Clostridia	Clostridiales	Ruminococcaceae	Unknown	
ASV_1571	Bacteria	Proteobacteria	Alphaproteobacteria	Unknown			
ASV_1572	Bacteria	Firmicutes	Bacilli	Lactobacillales	Lactobacillaceae	Lactobacillus	
ASV_1573	Bacteria	Firmicutes	Clostridia	Clostridiales	Ruminococcaceae	Anaerotruncus	
ASV_1574	Bacteria	Firmicutes	Clostridia	Clostridiales	Lachnospiraceae	Fusicatenibacter	Fusicatenibacter_saccharivorans
ASV_1575	Bacteria	Firmicutes	Clostridia	Clostridiales	Lachnospiraceae	Unknown	
ASV_1576	Bacteria	Actinobacteria	Actinobacteria	Micrococcales	Microbacteriaceae	Curtobacterium	
ASV_1577	Bacteria	Firmicutes	Clostridia	Clostridiales	Ruminococcaceae	Ruminococcaceae_UCG-010	
ASV_1578	Bacteria	Firmicutes	Clostridia	Clostridiales	Lachnospiraceae	Unknown	
ASV_1579	Bacteria	Firmicutes	Clostridia	Clostridiales	Ruminococcaceae	Ruminococcaceae_UCG-014	
ASV_1580	Bacteria	Actinobacteria	Coriobacteriia	Coriobacteriales	Eggerthellaceae	Unknown	
ASV_1581	Bacteria	Tenericutes	Mollicutes	Izimaplasmatales	Unknown		
ASV_1582	Bacteria	Firmicutes	Clostridia	Clostridiales	Lachnospiraceae	Unknown	
ASV_1583	Bacteria	Firmicutes	Clostridia	Clostridiales	Lachnospiraceae	Unknown	
ASV_1584	Bacteria	Deferribacteres	Deferribacteres	Deferribacterales	Deferribacteraceae	Mucispirillum	
ASV_1585	Bacteria	Firmicutes	Clostridia	Clostridiales	Lachnospiraceae	Lachnospiraceae_FCS020_group	
ASV_1586	Bacteria	Firmicutes	Clostridia	Clostridiales	Clostridiales_vadinBB60_group	Unknown	
ASV_1587	Bacteria	Firmicutes	Clostridia	Clostridiales	Clostridiales_vadinBB60_group	Unknown	
ASV_1588	Bacteria	Bacteroidetes	Bacteroidia	Bacteroidales	Muribaculaceae	Unknown	
ASV_1589	Bacteria	Firmicutes	Clostridia	Clostridiales	Lachnospiraceae	Lachnospiraceae_NK4A136_group	
ASV_1590	Bacteria	Firmicutes	Clostridia	Clostridiales	Ruminococcaceae	Ruminococcaceae_UCG-014	
ASV_1591	Bacteria	Firmicutes	Clostridia	Clostridiales	Lachnospiraceae	Unknown	
ASV_1592	Bacteria	Firmicutes	Bacilli	Lactobacillales	Lactobacillaceae	Lactobacillus	
ASV_1593	Bacteria	Firmicutes	Clostridia	Clostridiales	Unknown		
ASV_1594	Bacteria	Firmicutes	Clostridia	Clostridiales	Lachnospiraceae	Unknown	
ASV_1595	Bacteria	Firmicutes	Clostridia	Clostridiales	Lachnospiraceae	Tyzzerella_3	
ASV_1596	Bacteria	Firmicutes	Clostridia	Clostridiales	Ruminococcaceae	Ruminococcaceae_UCG-014	
ASV_1597	Bacteria	Firmicutes	Clostridia	Clostridiales	Lachnospiraceae	Lachnospiraceae_UCG-001	Lachnospiraceae_UCG-001_bacterium
ASV_1598	Bacteria	Firmicutes	Bacilli	Lactobacillales	Lactobacillaceae	Lactobacillus	
ASV_1599	Bacteria	Firmicutes	Clostridia	Clostridiales	Lachnospiraceae	A2	
ASV_1600	Bacteria	Firmicutes	Clostridia	Clostridiales	Lachnospiraceae	Unknown	
ASV_1601	Bacteria	Firmicutes	Clostridia	Clostridiales	Lachnospiraceae	Unknown	
ASV_1602	Bacteria	Firmicutes	Clostridia	Clostridiales	Lachnospiraceae	Unknown	
ASV_1603	Bacteria	Firmicutes	Bacilli	Lactobacillales	Lactobacillaceae	Lactobacillus	
ASV_1604	Bacteria	Bacteroidetes	Bacteroidia	Bacteroidales	Muribaculaceae	Unknown	

ASV_1605	Bacteria	Firmicutes	Clostridia	Clostridiales	Ruminococcaceae	Ruminiclostridium_9
ASV_1606	Bacteria	Firmicutes	Clostridia	Clostridiales	Lachnospiraceae	Unknown
ASV_1607	Bacteria	Firmicutes	Erysipelotrichia	Erysipelotrichales	Erysipelotrichaceae	Unknown
ASV_1608	Bacteria	Firmicutes	Clostridia	Clostridiales	Ruminococcaceae	Ruminococcaceae_UCG-014
ASV_1609	Bacteria	Firmicutes	Clostridia	Clostridiales	Lachnospiraceae	Lachnospiraceae_NK4A136_group
ASV_1610	Bacteria	Bacteroidetes	Bacteroidia	Bacteroidales	Rikenellaceae	Alistipes
ASV_1611	Bacteria	Bacteroidetes	Bacteroidia	Bacteroidales	Muribaculaceae	Unknown
ASV_1612	Bacteria	Firmicutes	Clostridia	Clostridiales	Lachnospiraceae	Lachnoclostridium
ASV_1613	Bacteria	Firmicutes	Clostridia	Clostridiales	Ruminococcaceae	Ruminiclostridium_9
ASV_1614	Bacteria	Firmicutes	Clostridia	Clostridiales	Ruminococcaceae	Ruminococcaceae_UCG-014
ASV_1615	Bacteria	Firmicutes	Clostridia	Clostridiales	Family_XIII	Anaerovorax
ASV_1616	Bacteria	Firmicutes	Clostridia	Clostridiales	Lachnospiraceae	Unknown
ASV_1617	Bacteria	Firmicutes	Clostridia	Clostridiales		Unknown
ASV_1618	Bacteria	Firmicutes	Clostridia	Clostridiales	Lachnospiraceae	Unknown
ASV_1619	Bacteria	Firmicutes	Clostridia	Clostridiales	Lachnospiraceae	Lachnospiraceae_NK4A136_group
ASV_1620	Bacteria	Firmicutes	Clostridia	Clostridiales	Ruminococcaceae	Unknown
ASV_1621	Bacteria	Deferribacteres	Deferribacteres	Deferribacterales	Deferribacteraceae	Mucispirillum
ASV_1622	Bacteria	Bacteroidetes	Bacteroidia	Bacteroidales	Muribaculaceae	Unknown
ASV_1623	Bacteria	Firmicutes	Clostridia	Clostridiales	Ruminococcaceae	Ruminococcaceae_UCG-013
ASV_1624	Bacteria	Bacteroidetes	Bacteroidia	Bacteroidales	Muribaculaceae	Unknown
ASV_1625	Bacteria	Bacteroidetes	Bacteroidia	Bacteroidales	Muribaculaceae	Unknown
ASV_1626	Bacteria	Bacteroidetes	Bacteroidia	Bacteroidales	Muribaculaceae	Muribaculum
ASV_1627	Bacteria	Firmicutes	Clostridia	Clostridiales	Clostridiales_vadinBB60_group	Unknown
ASV_1628	Bacteria	Firmicutes	Clostridia	Clostridiales	Lachnospiraceae	Unknown
ASV_1629	Bacteria	Firmicutes	Clostridia	Clostridiales	Ruminococcaceae	Anaerotruncus
ASV_1630	Bacteria	Firmicutes	Clostridia	Clostridiales		Unknown
ASV_1631	Bacteria	Firmicutes	Clostridia	Clostridiales	Ruminococcaceae	Ruminococcaceae_UCG-005
ASV_1632	Bacteria	Bacteroidetes	Bacteroidia	Bacteroidales	Muribaculaceae	Unknown
ASV_1633	Bacteria	Bacteroidetes	Bacteroidia	Bacteroidales	Muribaculaceae	Unknown
ASV_1634	Bacteria	Firmicutes	Clostridia	Clostridiales	Lachnospiraceae	Lachnospiraceae_NK4A136_group
ASV_1635	Bacteria	Firmicutes	Clostridia	Clostridiales	Ruminococcaceae	Butyricicoccus
ASV_1636	Bacteria	Firmicutes	Clostridia	Clostridiales	Christensenellaceae	Christensenellaceae_R-7_group
ASV_1637	Bacteria	Firmicutes	Clostridia	Clostridiales	Ruminococcaceae	Butyricicoccus
ASV_1638	Bacteria	Firmicutes	Clostridia	Clostridiales	Ruminococcaceae	Ruminiclostridium_9
ASV_1639	Bacteria	Bacteroidetes	Bacteroidia	Bacteroidales	Rikenellaceae	Alistipes
ASV_1640	Bacteria	Firmicutes	Clostridia	Clostridiales	Lachnospiraceae	Lachnospiraceae_UCG-001
ASV_1641	Bacteria	Firmicutes	Clostridia	Clostridiales	Ruminococcaceae	Oscillospira

ASV_1642	Bacteria	Firmicutes	Clostridia	Clostridiales	Clostridiales_vadinBB60_group	Unknown	
ASV_1643	Bacteria	Firmicutes	Clostridia	Clostridiales	Ruminococcaceae	Ruminiclostridium_9	
ASV_1644	Bacteria	Firmicutes	Erysipelotrichia	Erysipelotrichales	Erysipelotrichaceae	Unknown	
ASV_1645	Bacteria	Firmicutes	Clostridia	Clostridiales	Lachnospiraceae	Unknown	
ASV_1646	Bacteria	Bacteroidetes	Bacteroidia	Bacteroidales	Rikenellaceae	Alistipes	
ASV_1647	Bacteria	Firmicutes	Bacilli	Lactobacillales	Lactobacillaceae	Lactobacillus	
ASV_1648	Bacteria	Firmicutes	Clostridia	Clostridiales	Lachnospiraceae	Lachnospiraceae_NK4A136_group	
ASV_1649	Bacteria	Firmicutes	Clostridia	Clostridiales	Ruminococcaceae	Oscillibacter	
ASV_1650	Bacteria	Firmicutes	Clostridia	Clostridiales	Lachnospiraceae	Unknown	
ASV_1651	Bacteria	Bacteroidetes	Bacteroidia	Bacteroidales	Muribaculaceae	Unknown	
ASV_1652	Bacteria	Firmicutes	Clostridia	Clostridiales	Lachnospiraceae	Unknown	
ASV_1653	Bacteria	Actinobacteria	Coriobacteriia	Coriobacteriales	Coriobacteriaceae	Collinsella	
ASV_1654	Bacteria	Firmicutes	Clostridia	Clostridiales	Clostridiaceae_1	Clostridium_sensu_stricto_1	
ASV_1655	Bacteria	Firmicutes	Clostridia	Clostridiales	Ruminococcaceae	Unknown	
ASV_1656	Bacteria	Firmicutes	Bacilli	Lactobacillales	Lactobacillaceae	Lactobacillus	
ASV_1657	Bacteria	Deferribacteres	Deferribacteres	Deferribacterales	Deferribacteraceae	Mucispirillum	
ASV_1658	Bacteria	Firmicutes	Clostridia	Clostridiales	Lachnospiraceae	Lachnospiraceae_NK4A136_group	
ASV_1659	Bacteria	Firmicutes	Clostridia	Clostridiales	Lachnospiraceae	Unknown	
ASV_1660	Bacteria	Firmicutes	Clostridia	Clostridiales	Ruminococcaceae	Ruminococcaceae_NK4A214_group	
ASV_1661	Bacteria	Firmicutes	Clostridia	Clostridiales	Clostridiales_vadinBB60_group	Unknown	
ASV_1662	Bacteria	Actinobacteria	Coriobacteriia	Coriobacteriales	Coriobacteriaceae	Collinsella	Collinsella_aerofaciens
ASV_1663	Bacteria	Firmicutes	Clostridia	Clostridiales	Ruminococcaceae	Ruminiclostridium_6	
ASV_1664	Bacteria	Bacteroidetes	Bacteroidia	Bacteroidales	Prevotellaceae	Prevotellaceae_UCG-001	
ASV_1665	Bacteria	Firmicutes	Clostridia	Clostridiales	Ruminococcaceae	Unknown	
ASV_1666	Bacteria	Tenericutes	Mollicutes	Anaeroplasmatales	Anaeroplasmataceae	Anaeroplasma	
ASV_1667	Bacteria	Firmicutes	Clostridia	Clostridiales	Lachnospiraceae	Unknown	
ASV_1668	Bacteria	Firmicutes	Erysipelotrichia	Erysipelotrichales	Erysipelotrichaceae	Asteroleplasma	
ASV_1669	Bacteria	Firmicutes	Clostridia	Clostridiales	Lachnospiraceae	Unknown	
ASV_1670	Bacteria	Firmicutes	Clostridia	Clostridiales	Lachnospiraceae	Unknown	
ASV_1671	Bacteria	Firmicutes	Bacilli	Lactobacillales	Lactobacillaceae	Lactobacillus	
ASV_1672	Bacteria	Bacteroidetes	Bacteroidia	Bacteroidales	Muribaculaceae	Unknown	
ASV_1673	Bacteria	Firmicutes	Clostridia	Clostridiales	Ruminococcaceae	Ruminococcaceae_UCG-013	
ASV_1674	Bacteria	Bacteroidetes	Bacteroidia	Bacteroidales	Rikenellaceae	Alistipes	
ASV_1675	Bacteria	Actinobacteria	Coriobacteriia	Coriobacteriales	Eggerthellaceae	Denitrobacterium	
ASV_1676	Bacteria	Cyanobacteria	Melainabacteria	Gastranaerophilales	Unknown		
ASV_1677	Bacteria	Firmicutes	Clostridia	Clostridiales	Ruminococcaceae	Anaerotruncus	
ASV_1678	Bacteria	Firmicutes	Clostridia	Clostridiales	Lachnospiraceae	Unknown	

ASV_1679	Bacteria	Bacteroidetes	Bacteroidia	Bacteroidales	Bacteroidaceae	Bacteroides
ASV_1680	Bacteria	Firmicutes	Clostridia	Clostridiales	Ruminococcaceae	Faecalibacterium
ASV_1681	Bacteria	Bacteroidetes	Bacteroidia	Bacteroidales	Muribaculaceae	Unknown
ASV_1682	Bacteria	Firmicutes	Clostridia	Clostridiales	Lachnospiraceae	Unknown
ASV_1683	Bacteria	Firmicutes	Bacilli	Lactobacillales	Lactobacillaceae	Lactobacillus
ASV_1684	Bacteria	Bacteroidetes	Bacteroidia	Bacteroidales	Muribaculaceae	Unknown
ASV_1685	Bacteria	Firmicutes	Clostridia	Clostridiales	Lachnospiraceae	GCA-900066575
ASV_1686	Bacteria	Firmicutes	Clostridia	Clostridiales	Lachnospiraceae	Lachnospiraceae_NK4A136_group
ASV_1687	Bacteria	Firmicutes	Clostridia	Clostridiales	Ruminococcaceae	Ruminiclostridium_9
ASV_1688	Bacteria	Firmicutes	Clostridia	Clostridiales	Ruminococcaceae	Ruminococcus_2
ASV_1689	Bacteria	Bacteroidetes	Bacteroidia	Bacteroidales	Rikenellaceae	Alistipes
ASV_1690	Bacteria	Bacteroidetes	Bacteroidia	Bacteroidales	Rikenellaceae	Rikenellaceae_RC9_gut_group
ASV_1691	Bacteria	Firmicutes	Erysipelotrichia	Erysipelotrichales	Erysipelotrichaceae	Unknown
ASV_1692	Bacteria	Firmicutes	Clostridia	Clostridiales		Unknown
ASV_1693	Bacteria	Firmicutes	Erysipelotrichia	Erysipelotrichales	Erysipelotrichaceae	Erysipelotrichaceae_UCG-004
ASV_1694	Bacteria	Firmicutes	Clostridia	Clostridiales	Ruminococcaceae	Unknown
ASV_1695	Bacteria	Firmicutes	Bacilli	Lactobacillales	Lactobacillaceae	Lactobacillus
ASV_1696	Bacteria	Firmicutes	Clostridia	Clostridiales	Clostridiales_vadinBB60_group	Unknown
ASV_1697	Bacteria	Firmicutes	Clostridia	Clostridiales	Ruminococcaceae	Anaerotruncus
ASV_1698	Bacteria	Firmicutes	Clostridia	Clostridiales	Ruminococcaceae	Unknown
ASV_1699	Bacteria	Firmicutes	Clostridia	Clostridiales	Lachnospiraceae	ASF356
ASV_1700	Bacteria	Firmicutes	Clostridia	Clostridiales	Lachnospiraceae	Lachnospiraceae_NK4A136_group
ASV_1701	Bacteria	Cyanobacteria	Oxyphotobacteria	Chloroplast		Unknown
ASV_1702	Bacteria	Bacteroidetes	Bacteroidia	Bacteroidales	Rikenellaceae	Alistipes
ASV_1703	Bacteria	Firmicutes	Clostridia	Clostridiales	Lachnospiraceae	Roseburia
ASV_1704	Bacteria	Firmicutes	Clostridia	Clostridiales	Lachnospiraceae	Lachnospiraceae_NK4A136_group
ASV_1705	Bacteria	Firmicutes	Clostridia	Clostridiales	Ruminococcaceae	Ruminococcaceae_UCG-014
ASV_1706	Bacteria	Bacteroidetes	Bacteroidia	Bacteroidales	Rikenellaceae	Alistipes
ASV_1707	Bacteria	Firmicutes	Clostridia	Clostridiales	Lachnospiraceae	Lachnospiraceae_NK4A136_group
ASV_1708	Bacteria	Firmicutes	Clostridia	Clostridiales	Ruminococcaceae	Unknown
ASV_1709	Bacteria	Firmicutes	Clostridia	Clostridiales	Lachnospiraceae	Unknown
ASV_1710	Bacteria	Firmicutes	Clostridia	Clostridiales	Clostridiales_vadinBB60_group	Unknown
ASV_1711	Bacteria	Proteobacteria	Gammaproteobacteria	Betaproteobacteriales	Burkholderiaceae	Parasutterella
ASV_1712	Bacteria	Firmicutes	Bacilli	Lactobacillales	Lactobacillaceae	Lactobacillus
ASV_1713	Bacteria	Firmicutes	Clostridia	Clostridiales	Ruminococcaceae	Ruminococcaceae_UCG-014
ASV_1714	Bacteria	Firmicutes	Clostridia	Clostridiales	Ruminococcaceae	Ruminiclostridium
ASV_1715	Bacteria	Firmicutes	Clostridia	Clostridiales	Ruminococcaceae	Unknown

ASV_1716	Bacteria	Firmicutes	Clostridia	Clostridiales	Lachnospiraceae	Lachnoclostridium
ASV_1717	Bacteria	Firmicutes	Clostridia	Clostridiales	Lachnospiraceae	Unknown
ASV_1718	Bacteria	Proteobacteria	Deltaproteobacteria	Desulfovibrionales	Desulfovibrionaceae	Bilophila
ASV_1719	Bacteria	Firmicutes	Clostridia	Clostridiales	Lachnospiraceae	Unknown
ASV_1720	Bacteria	Firmicutes	Clostridia	Clostridiales	Lachnospiraceae	Unknown
ASV_1721	Bacteria	Bacteroidetes	Bacteroidia	Bacteroidales	Muribaculaceae	Unknown
ASV_1722	Bacteria	Firmicutes	Clostridia	Clostridiales	Ruminococcaceae	Unknown
ASV_1723	Bacteria	Firmicutes	Bacilli	Lactobacillales	Lactobacillaceae	Lactobacillus
ASV_1724	Bacteria	Firmicutes	Clostridia	Clostridiales	Lachnospiraceae	Lachnospiraceae_NK4A136_group
ASV_1725	Bacteria	Firmicutes	Clostridia	Clostridiales	Lachnospiraceae	Unknown
ASV_1726	Bacteria	Tenericutes	Mollicutes	Mollicutes_RF39	Unknown	
ASV_1727	Bacteria	Firmicutes	Clostridia	Clostridiales	Ruminococcaceae	Unknown
ASV_1728	Bacteria	Firmicutes	Clostridia	Clostridiales	Ruminococcaceae	Unknown
ASV_1729	Bacteria	Firmicutes	Clostridia	Clostridiales	Lachnospiraceae	Unknown
ASV_1730	Bacteria	Firmicutes	Clostridia	Clostridiales	Ruminococcaceae	Oscillibacter
ASV_1731	Bacteria	Tenericutes	Mollicutes	Mollicutes_RF39	Unknown	
ASV_1732	Bacteria	Firmicutes	Clostridia	Clostridiales	Ruminococcaceae	Ruminococcaceae_UCG-014
ASV_1733	Bacteria	Firmicutes	Bacilli	Lactobacillales	Lactobacillaceae	Lactobacillus
ASV_1734	Bacteria	Bacteroidetes	Bacteroidia	Bacteroidales	Muribaculaceae	Unknown
ASV_1735	Bacteria	Bacteroidetes	Bacteroidia	Bacteroidales	Muribaculaceae	Unknown
ASV_1736	Bacteria	Firmicutes	Clostridia	Clostridiales	Ruminococcaceae	Butyricicoccus
ASV_1737	Bacteria	Firmicutes	Clostridia	Clostridiales	Ruminococcaceae	Ruminococcaceae_UCG-010
ASV_1738	Bacteria	Firmicutes	Clostridia	Clostridiales	Lachnospiraceae	Lachnospiraceae_NK4A136_group
ASV_1739	Bacteria	Tenericutes	Mollicutes	Mollicutes_RF39	Unknown	
ASV_1740	Bacteria	Firmicutes	Clostridia	Clostridiales	Lachnospiraceae	Marvinbryantia
ASV_1741	Bacteria	Firmicutes	Clostridia	Clostridiales	Lachnospiraceae	Unknown
ASV_1742	Bacteria	Firmicutes	Clostridia	Clostridiales	Ruminococcaceae	Ruminococcus_1
ASV_1743	Bacteria	Bacteroidetes	Bacteroidia	Bacteroidales	Muribaculaceae	Unknown
ASV_1744	Bacteria	Bacteroidetes	Bacteroidia	Bacteroidales	Muribaculaceae	Unknown
ASV_1745	Bacteria	Proteobacteria	Gammaproteobacteria	Betaproteobacteriales	Burkholderiaceae	Sutterella
ASV_1746	Bacteria	Bacteroidetes	Bacteroidia	Bacteroidales	Rikenellaceae	Alistipes
ASV_1747	Bacteria	Firmicutes	Clostridia	Clostridiales	Ruminococcaceae	Ruminiclostridium_9
ASV_1748	Bacteria	Firmicutes	Clostridia	Clostridiales	Lachnospiraceae	Unknown
ASV_1749	Bacteria	Firmicutes	Clostridia	Clostridiales	Lachnospiraceae	Fusicatenibacter
ASV_1750	Bacteria	Firmicutes	Clostridia	Clostridiales	Lachnospiraceae	Lachnospiraceae_NK4A136_group
ASV_1751	Bacteria	Firmicutes	Clostridia	Clostridiales	Lachnospiraceae	Unknown
ASV_1752	Bacteria	Bacteroidetes	Bacteroidia	Bacteroidales	Prevotellaceae	Prevotellaceae_UCG-001

ASV_1753	Bacteria	Firmicutes	Clostridia	Clostridiales	Lachnospiraceae	Unknown
ASV_1754	Bacteria	Bacteroidetes	Bacteroidia	Bacteroidales	Prevotellaceae	Prevotellaceae_UCG-001
ASV_1755	Bacteria	Bacteroidetes	Bacteroidia	Bacteroidales	Muribaculaceae	Unknown
ASV_1756	Bacteria	Firmicutes	Clostridia	Clostridiales	Lachnospiraceae	Unknown
ASV_1757	Bacteria			Unknown		
ASV_1758	Bacteria	Firmicutes	Clostridia	Clostridiales	Unknown	
ASV_1759	Bacteria	Actinobacteria	Coriobacteriia	Coriobacteriales	Eggerthellaceae	Denitrobacterium
ASV_1760	Bacteria	Firmicutes	Clostridia	Clostridiales	Lachnospiraceae	Lachnospiraceae_UCG-010
ASV_1761	Bacteria	Actinobacteria	Coriobacteriia	Coriobacteriales	Eggerthellaceae	Unknown
ASV_1762	Bacteria	Firmicutes	Clostridia	Clostridiales	Ruminococcaceae	Unknown
ASV_1763	Bacteria	Bacteroidetes	Bacteroidia	Bacteroidales	Muribaculaceae	Unknown
ASV_1764	Bacteria	Firmicutes	Clostridia	Clostridiales	Family_XIII	Unknown
ASV_1765	Bacteria	Firmicutes	Clostridia	Clostridiales	Lachnospiraceae	Unknown
ASV_1766	Bacteria	Bacteroidetes	Bacteroidia	Bacteroidales	Muribaculaceae	Unknown
ASV_1767	Bacteria	Firmicutes	Clostridia	Clostridiales	Ruminococcaceae	Unknown
ASV_1768	Bacteria	Bacteroidetes	Bacteroidia	Bacteroidales	Muribaculaceae	Unknown
ASV_1769	Bacteria	Firmicutes	Clostridia	Clostridiales	Lachnospiraceae	Tyzzerella
ASV_1770	Bacteria	Firmicutes	Clostridia	Clostridiales	Lachnospiraceae	Unknown
ASV_1771	Bacteria	Firmicutes	Clostridia	Clostridiales	Lachnospiraceae	Unknown
ASV_1772	Bacteria	Bacteroidetes	Bacteroidia	Bacteroidales	Muribaculaceae	Unknown
ASV_1773	Bacteria	Bacteroidetes	Bacteroidia	Bacteroidales	Muribaculaceae	Unknown
ASV_1774	Bacteria	Firmicutes	Clostridia	Clostridiales	Lachnospiraceae	Unknown
ASV_1775	Bacteria	Firmicutes	Clostridia	Clostridiales	Lachnospiraceae	ASF356
ASV_1776	Bacteria	Firmicutes	Clostridia	Clostridiales	Ruminococcaceae	Ruminiclostridium
ASV_1777	Bacteria	Firmicutes	Clostridia	Clostridiales	Lachnospiraceae	Roseburia
ASV_1778	Bacteria	Firmicutes	Clostridia	Clostridiales	Unknown	
ASV_1779	Bacteria	Firmicutes	Clostridia	Clostridiales	Family_XIII	Family_XIII_UCG-001
ASV_1780	Bacteria	Bacteroidetes	Bacteroidia	Bacteroidales	Muribaculaceae	Unknown
ASV_1781	Bacteria	Firmicutes	Clostridia	Clostridiales	Ruminococcaceae	Unknown
ASV_1782	Bacteria	Firmicutes	Clostridia	Clostridiales	Lachnospiraceae	Unknown
ASV_1783	Bacteria	Firmicutes	Clostridia	Clostridiales	Lachnospiraceae	Unknown
ASV_1784	Bacteria	Bacteroidetes	Bacteroidia	Bacteroidales	Barnesiellaceae	Barnesiella
ASV_1785	Bacteria			Unknown		
ASV_1786	Bacteria	Bacteroidetes	Bacteroidia	Bacteroidales	Rikenellaceae	Alistipes
ASV_1787	Bacteria	Firmicutes	Clostridia	Clostridiales	Ruminococcaceae	Oscillibacter
ASV_1788	Bacteria	Bacteroidetes	Bacteroidia	Bacteroidales	Rikenellaceae	Alistipes
ASV_1789	Bacteria	Firmicutes	Bacilli	Lactobacillales	Lactobacillaceae	Lactobacillus

ASV_1790	Bacteria	Firmicutes	Bacilli	Lactobacillales	Lactobacillaceae	Lactobacillus	
ASV_1791	Bacteria	Firmicutes	Clostridia	Clostridiales	Lachnospiraceae	Lachnospiraceae_NK4A136_group	
ASV_1792	Bacteria	Bacteroidetes	Bacteroidia	Bacteroidales	Muribaculaceae	Unknown	
ASV_1793	Bacteria	Bacteroidetes	Bacteroidia	Bacteroidales	Rikenellaceae	Alistipes	
ASV_1794	Bacteria	Firmicutes	Clostridia	Clostridiales	Ruminococcaceae	Subdoligranulum	
ASV_1795	Bacteria	Firmicutes	Clostridia	Clostridiales	Lachnospiraceae	Lachnospiraceae_UCG-006	
ASV_1796	Bacteria	Firmicutes	Clostridia	Clostridiales	Ruminococcaceae	Anaerotruncus	
ASV_1797	Bacteria	Firmicutes	Clostridia	Clostridiales	Ruminococcaceae	Subdoligranulum	
ASV_1798	Bacteria	Bacteroidetes	Bacteroidia	Bacteroidales	Bacteroidaceae	Bacteroides	
ASV_1799	Bacteria	Firmicutes	Erysipelotrichia	Erysipelotrichales	Erysipelotrichaceae	Unknown	
ASV_1800	Bacteria	Firmicutes	Clostridia	Clostridiales	Lachnospiraceae	Unknown	
ASV_1801	Bacteria	Firmicutes	Negativicutes	Selenomonadales	Veillonellaceae	Dialister	Dialister_invisus
ASV_1802	Bacteria	Firmicutes	Bacilli	Bacillales	Staphylococcaceae	Staphylococcus	
ASV_1803	Bacteria	Proteobacteria	Gammaproteobacteria	Betaproteobacteriales	Burkholderiaceae	Tepidimonas	Tepidimonas_fonticaldi
ASV_1804	Bacteria	Bacteroidetes	Bacteroidia	Bacteroidales	Prevotellaceae	Prevotella_2	
ASV_1805	Bacteria	Firmicutes	Clostridia	Clostridiales	Ruminococcaceae	Ruminococcaceae_UCG-014	
ASV_1806	Bacteria	Bacteroidetes	Bacteroidia	Bacteroidales	Muribaculaceae	Unknown	
ASV_1807	Bacteria	Firmicutes	Clostridia	Clostridiales	Lachnospiraceae	GCA-900066575	
ASV_1808	Bacteria	Firmicutes	Clostridia	Clostridiales	Lachnospiraceae	Lachnospiraceae_UCG-006	
ASV_1809	Bacteria	Firmicutes	Clostridia	Clostridiales	Lachnospiraceae	Lachnospiraceae_NK4A136_group	
ASV_1810	Bacteria	Firmicutes	Bacilli	Lactobacillales		Unknown	
ASV_1811	Bacteria	Bacteroidetes	Bacteroidia	Bacteroidales	Muribaculaceae	Unknown	
ASV_1812	Bacteria	Firmicutes	Clostridia	Clostridiales	Clostridiales_vadinBB60_group	Unknown	
ASV_1813	Bacteria	Bacteroidetes	Bacteroidia	Bacteroidales	Muribaculaceae	Unknown	
ASV_1814	Bacteria					Unknown	
ASV_1815	Bacteria	Firmicutes	Clostridia	Clostridiales	Lachnospiraceae	Unknown	
ASV_1816	Bacteria	Actinobacteria	Coriobacteriia	Coriobacteriales	Eggerthellaceae	Adlercreutzia	
ASV_1817	Bacteria	Firmicutes	Clostridia	Clostridiales	Ruminococcaceae	Butyricicoccus	
ASV_1818	Bacteria	Firmicutes	Clostridia	Clostridiales	Lachnospiraceae	Lachnospiraceae_FCS020_group	
ASV_1819	Bacteria	Firmicutes	Clostridia	Clostridiales		Unknown	
ASV_1820	Bacteria	Firmicutes	Bacilli	Lactobacillales	Lactobacillaceae	Lactobacillus	
ASV_1821	Bacteria	Firmicutes	Clostridia	Clostridiales	Lachnospiraceae	Lachnospiraceae_UCG-001	
ASV_1822	Bacteria	Firmicutes	Clostridia	Clostridiales	Lachnospiraceae	Unknown	
ASV_1823	Bacteria	Firmicutes	Clostridia	Clostridiales	Clostridiales_vadinBB60_group	Unknown	
ASV_1824	Bacteria	Firmicutes	Clostridia	Clostridiales	Ruminococcaceae	Ruminococcaceae_UCG-014	
ASV_1825	Bacteria	Firmicutes	Clostridia	Clostridiales	Lachnospiraceae	Lachnospiraceae_NK4A136_group	
ASV_1826	Bacteria	Firmicutes	Clostridia	Clostridiales	Lachnospiraceae	Lachnospiraceae_UCG-001	

ASV_1827	Bacteria	Bacteroidetes	Bacteroidia	Bacteroidales	Muribaculaceae	Unknown
ASV_1828	Bacteria	Firmicutes	Erysipelotrichia	Erysipelotrichales	Erysipelotrichaceae	Turicibacter
ASV_1829	Bacteria	Bacteroidetes	Bacteroidia	Bacteroidales	Prevotellaceae	Prevotellaceae_UCG-001
ASV_1830	Bacteria	Firmicutes	Clostridia	Clostridiales	Peptococcaceae	Unknown
ASV_1831	Bacteria	Firmicutes	Clostridia	Clostridiales	Lachnospiraceae	Unknown
ASV_1832	Bacteria	Bacteroidetes	Bacteroidia	Bacteroidales	Prevotellaceae	Prevotellaceae_UCG-001
ASV_1833	Bacteria	Firmicutes	Clostridia	Clostridiales	Lachnospiraceae	Unknown
ASV_1834	Bacteria	Bacteroidetes	Bacteroidia	Bacteroidales	Unknown	
ASV_1835	Bacteria	Firmicutes	Clostridia	Clostridiales	Lachnospiraceae	ASF356
ASV_1836	Bacteria	Firmicutes	Clostridia	Clostridiales	Clostridiales_vadinBB60_group	Unknown
ASV_1837	Bacteria	Firmicutes	Clostridia	Clostridiales	Ruminococcaceae	Oscillibacter
ASV_1838	Bacteria	Firmicutes	Clostridia	Clostridiales	Clostridiaceae_1	Clostridium_sensu_stricto_1
ASV_1839	Bacteria	Proteobacteria	Gammaproteobacteria	Betaproteobacteriales	Burkholderiaceae	Sutterella
ASV_1840	Bacteria	Firmicutes	Clostridia	Clostridiales	Ruminococcaceae	Ruminococcaceae_UCG-014
ASV_1841	Bacteria	Tenericutes	Mollicutes	Izimaplasmatales	Unknown	
ASV_1842	Bacteria	Firmicutes	Clostridia	Clostridiales	Clostridiales_vadinBB60_group	Unknown
ASV_1843	Bacteria	Bacteroidetes	Bacteroidia	Bacteroidales	Prevotellaceae	Prevotellaceae_UCG-001
ASV_1844	Bacteria	Verrucomicrobia	Verrucomicrobiae	Verrucomicrobiales	Akkermansiaceae	Akkermansia
ASV_1845	Bacteria	Bacteroidetes	Bacteroidia	Bacteroidales	Prevotellaceae	Prevotellaceae_UCG-001
ASV_1846	Bacteria	Firmicutes	Clostridia	Clostridiales	Lachnospiraceae	Unknown
ASV_1847	Bacteria	Bacteroidetes	Bacteroidia	Bacteroidales	Muribaculaceae	Unknown
ASV_1848	Bacteria	Firmicutes	Clostridia	Clostridiales	Lachnospiraceae	Lachnoclostridium
ASV_1849	Bacteria	Firmicutes	Clostridia	Clostridiales	Lachnospiraceae	A2
ASV_1850	Bacteria	Firmicutes	Clostridia	Clostridiales	Unknown	
ASV_1851	Bacteria	Firmicutes	Clostridia	Clostridiales	Ruminococcaceae	Ruminococcaceae_UCG-014
ASV_1852	Bacteria	Proteobacteria	Deltaproteobacteria	Desulfovibrionales	Desulfovibrionaceae	Bilophila
ASV_1853	Bacteria	Firmicutes	Clostridia	Clostridiales	Lachnospiraceae	Lachnoclostridium
ASV_1854	Bacteria	Firmicutes	Bacilli	Lactobacillales	Lactobacillaceae	Lactobacillus
ASV_1855	Bacteria	Bacteroidetes	Bacteroidia	Bacteroidales	Prevotellaceae	Unknown
ASV_1856	Bacteria	Bacteroidetes	Bacteroidia	Bacteroidales	Muribaculaceae	Unknown
ASV_1857	Bacteria	Firmicutes	Erysipelotrichia	Erysipelotrichales	Erysipelotrichaceae	Unknown
ASV_1858	Bacteria	Firmicutes	Clostridia	Clostridiales	Ruminococcaceae	Ruminiclostridium_5
ASV_1859	Bacteria	Proteobacteria	Alphaproteobacteria	Rhodospirillales	Unknown	
ASV_1860	Bacteria	Firmicutes	Clostridia	Clostridiales	Lachnospiraceae	Unknown
ASV_1861	Bacteria	Firmicutes	Clostridia	Clostridiales	Lachnospiraceae	Unknown
ASV_1862	Bacteria	Firmicutes	Clostridia	Clostridiales	Lachnospiraceae	Unknown
ASV_1863	Bacteria	Bacteroidetes	Bacteroidia	Bacteroidales	Muribaculaceae	Unknown

ASV_1864	Bacteria	Firmicutes	Clostridia	Clostridiales	Lachnospiraceae	Unknown	
ASV_1865	Bacteria	Firmicutes	Clostridia	Clostridiales	Ruminococcaceae	Ruminiclostridium_9	
ASV_1866	Bacteria	Proteobacteria	Deltaproteobacteria	Desulfovibrionales	Desulfovibrionaceae	Desulfovibrio	Desulfovibrio_piger
ASV_1867	Bacteria	Firmicutes	Clostridia	Clostridiales	Lachnospiraceae	Lachnospiraceae_UCG-006	
ASV_1868	Bacteria	Firmicutes	Clostridia	Clostridiales	Ruminococcaceae	Ruminococcaceae_UCG-014	
ASV_1869	Bacteria	Bacteroidetes	Bacteroidia	Bacteroidales	Muribaculaceae	Unknown	
ASV_1870	Bacteria	Firmicutes	Clostridia	Clostridiales	Lachnospiraceae	Unknown	
ASV_1871	Bacteria	Firmicutes	Clostridia	Clostridiales	Lachnospiraceae	Lachnospiraceae_NK4A136_group	
ASV_1872	Bacteria	Firmicutes	Clostridia	Clostridiales	Lachnospiraceae	Tyzzerella	
ASV_1873	Bacteria	Bacteroidetes	Bacteroidia	Bacteroidales	Muribaculaceae	Unknown	
ASV_1874	Bacteria	Proteobacteria	Gammaproteobacteria	Enterobacteriales	Enterobacteriaceae	Escherichia/Shigella	
ASV_1875	Bacteria	Firmicutes	Clostridia	Clostridiales	Ruminococcaceae	Ruminococcaceae_UCG-013	
ASV_1876	Bacteria	Firmicutes	Clostridia	Clostridiales	Lachnospiraceae	Unknown	
ASV_1877	Bacteria	Firmicutes	Clostridia	Clostridiales	Lachnospiraceae	Unknown	
ASV_1878	Bacteria	Bacteroidetes	Bacteroidia	Bacteroidales	Bacteroidaceae	Bacteroides	
ASV_1879	Bacteria	Firmicutes	Clostridia	Clostridiales	Lachnospiraceae	Lachnospiraceae_NK4A136_group	
ASV_1880	Bacteria	Firmicutes	Clostridia	Clostridiales	Ruminococcaceae	Unknown	
ASV_1881	Bacteria	Firmicutes	Clostridia	Clostridiales	Lachnospiraceae	Lachnospiraceae_UCG-006	
ASV_1882	Bacteria	Firmicutes	Clostridia	Clostridiales	Clostridiales_vadinBB60_group	Unknown	
ASV_1883	Bacteria	Firmicutes	Clostridia	Clostridiales	Lachnospiraceae	Lachnospiraceae_NK4A136_group	
ASV_1884	Bacteria	Firmicutes	Clostridia	Clostridiales	Clostridiales_vadinBB60_group	Unknown	
ASV_1885	Bacteria	Firmicutes	Clostridia	Clostridiales	Clostridiales_vadinBB60_group	Unknown	
ASV_1886	Bacteria	Firmicutes	Clostridia	Clostridiales	Ruminococcaceae	Ruminococcaceae_UCG-014	
ASV_1887	Bacteria	Tenericutes	Mollicutes	Mollicutes_RF39	Unknown		
ASV_1888	Bacteria	Bacteroidetes	Bacteroidia	Bacteroidales	Muribaculaceae	Unknown	
ASV_1889	Bacteria	Firmicutes	Clostridia	Clostridiales	Ruminococcaceae	Unknown	
ASV_1890	Bacteria	Bacteroidetes	Bacteroidia	Bacteroidales	Muribaculaceae	Unknown	
ASV_1891	Bacteria	Firmicutes	Clostridia	Clostridiales	Lachnospiraceae	Lachnospiraceae_NK4A136_group	
ASV_1892	Bacteria	Firmicutes	Clostridia	Clostridiales	Lachnospiraceae	Lachnospiraceae_NK4A136_group	
ASV_1893	Bacteria	Bacteroidetes	Bacteroidia	Bacteroidales	Muribaculaceae	Unknown	
ASV_1894	Bacteria	Firmicutes	Clostridia	Clostridiales	Ruminococcaceae	Ruminococcaceae_UCG-014	
ASV_1895	Bacteria	Firmicutes	Clostridia	Clostridiales	Lachnospiraceae	Lachnospiraceae_NK4A136_group	
ASV_1896	Bacteria	Tenericutes	Mollicutes	Mollicutes_RF39	Unknown		
ASV_1897	Bacteria	Firmicutes	Clostridia	Clostridiales	Lachnospiraceae	Unknown	
ASV_1898	Bacteria	Firmicutes	Clostridia	Clostridiales	Ruminococcaceae	Ruminiclostridium_5	
ASV_1899	Bacteria	Firmicutes	Clostridia	Clostridiales	Ruminococcaceae	Unknown	
ASV_1900	Bacteria	Firmicutes	Clostridia	Clostridiales	Lachnospiraceae	Unknown	

ASV_1901	Bacteria	Firmicutes	Clostridia	Clostridiales	Ruminococcaceae	Unknown
ASV_1902	Bacteria	Firmicutes	Clostridia	Clostridiales	Ruminococcaceae	Unknown
ASV_1903	Bacteria	Firmicutes	Clostridia	Clostridiales	Lachnospiraceae	Unknown
ASV_1904	Bacteria	Firmicutes	Clostridia	Clostridiales	Unknown	
ASV_1905	Bacteria			Unknown		
ASV_1906	Bacteria	Bacteroidetes	Bacteroidia	Bacteroidales	Muribaculaceae	Unknown
ASV_1907	Bacteria	Firmicutes	Clostridia	Clostridiales	Family_XIII	Family_XIII_AD3011_group
ASV_1908	Bacteria	Bacteroidetes	Bacteroidia		Unknown	
ASV_1909	Bacteria	Bacteroidetes	Bacteroidia	Bacteroidales	Muribaculaceae	Unknown
ASV_1910	Bacteria	Bacteroidetes	Bacteroidia	Bacteroidales	Muribaculaceae	Unknown
ASV_1911	Bacteria	Bacteroidetes	Bacteroidia	Bacteroidales	Muribaculaceae	Muribaculum
ASV_1912	Bacteria	Firmicutes	Clostridia	Clostridiales	Lachnospiraceae	Unknown
ASV_1913	Bacteria	Firmicutes	Clostridia	Clostridiales	Ruminococcaceae	Unknown
ASV_1914	Bacteria	Firmicutes	Clostridia	Clostridiales	Ruminococcaceae	Ruminiclostridium_9
ASV_1915	Bacteria	Firmicutes	Clostridia	Clostridiales	Lachnospiraceae	Unknown
ASV_1916	Bacteria	Firmicutes	Clostridia	Clostridiales	Lachnospiraceae	Lachnospiraceae_NK4A136_group
ASV_1917	Bacteria	Firmicutes	Clostridia	Clostridiales	Ruminococcaceae	Ruminiclostridium_9
ASV_1918	Bacteria	Firmicutes	Clostridia	Clostridiales	Ruminococcaceae	Ruminococcaceae_UCG-014
ASV_1919	Bacteria	Firmicutes	Clostridia	Clostridiales	Ruminococcaceae	Ruminococcaceae_UCG-014
ASV_1920	Bacteria	Firmicutes	Clostridia	Clostridiales	Lachnospiraceae	Lachnospiraceae_NK4A136_group
ASV_1921	Bacteria	Actinobacteria	Coriobacteriia	Coriobacteriales	Eggerthellaceae	Denitrobacterium
ASV_1922	Bacteria	Firmicutes	Bacilli	Unknown		
ASV_1923	Bacteria	Firmicutes	Clostridia	Clostridiales	Clostridiales_vadinBB60_group	Unknown
ASV_1924	Bacteria	Firmicutes	Clostridia	Clostridiales	Ruminococcaceae	Phocea
ASV_1925	Bacteria	Firmicutes	Clostridia	Clostridiales	Ruminococcaceae	Ruminiclostridium_9
ASV_1926	Bacteria	Firmicutes	Clostridia	Clostridiales	Lachnospiraceae	Unknown
ASV_1927	Bacteria	Firmicutes	Clostridia	Clostridiales	Clostridiales_vadinBB60_group	Unknown
ASV_1928	Bacteria	Tenericutes	Mollicutes	Mollicutes_RF39	Unknown	
ASV_1929	Bacteria	Firmicutes	Clostridia	Clostridiales	Clostridiales_vadinBB60_group	Unknown
ASV_1930	Bacteria	Firmicutes	Clostridia	Clostridiales	Lachnospiraceae	Unknown
ASV_1931	Bacteria	Firmicutes	Clostridia	Clostridiales	Ruminococcaceae	Ruminococcaceae_UCG-014
ASV_1932	Bacteria	Firmicutes	Clostridia	Clostridiales	Ruminococcaceae	Faecalibacterium
ASV_1933	Bacteria	Firmicutes	Clostridia	Clostridiales	Ruminococcaceae	Unknown
ASV_1934	Bacteria	Bacteroidetes	Bacteroidia	Bacteroidales	Muribaculaceae	Unknown
ASV_1935	Bacteria	Firmicutes	Clostridia	Clostridiales	Lachnospiraceae	Unknown
ASV_1936	Bacteria	Proteobacteria	Gammaproteobacteria	Betaproteobacteriales	Burkholderiaceae	Parasutterella
ASV_1937	Bacteria	Tenericutes	Mollicutes	Mollicutes_RF39	Unknown	

ASV_1938	Bacteria	Firmicutes	Clostridia	Clostridiales	Ruminococcaceae	Oscillibacter
ASV_1939	Bacteria	Firmicutes	Clostridia	Clostridiales	Ruminococcaceae	Ruminiclostridium_9
ASV_1940	Bacteria	Firmicutes	Clostridia	Clostridiales		Unknown
ASV_1941	Bacteria	Bacteroidetes	Bacteroidia	Bacteroidales	Muribaculaceae	Unknown
ASV_1942	Bacteria	Firmicutes	Bacilli	Lactobacillales	Lactobacillaceae	Lactobacillus
ASV_1943	Bacteria	Bacteroidetes	Bacteroidia	Bacteroidales	Muribaculaceae	Unknown
ASV_1944	Bacteria	Firmicutes	Clostridia	Clostridiales	Lachnospiraceae	Unknown
ASV_1945	Bacteria	Tenericutes	Mollicutes	Mollicutes_RF39		Unknown
ASV_1946	Bacteria	Firmicutes	Clostridia	Clostridiales	Ruminococcaceae	Unknown
ASV_1947	Bacteria	Firmicutes	Clostridia	Clostridiales	Ruminococcaceae	Unknown
ASV_1948	Bacteria					Unknown
ASV_1949	Bacteria	Bacteroidetes	Bacteroidia	Bacteroidales	Muribaculaceae	Muribaculum
ASV_1950	Bacteria	Deferribacteres	Deferribacteres	Deferribacterales	Deferribacteraceae	Mucispirillum
ASV_1951	Bacteria	Bacteroidetes	Bacteroidia	Bacteroidales	Prevotellaceae	Prevotellaceae_NK3B31_group
ASV_1952	Bacteria	Firmicutes	Clostridia	Clostridiales	Lachnospiraceae	Unknown
ASV_1953	Bacteria	Firmicutes	Clostridia	Clostridiales	Ruminococcaceae	Ruminiclostridium_9
ASV_1954	Bacteria	Firmicutes	Clostridia	Clostridiales	Lachnospiraceae	Lachnospiraceae_UCG-006
ASV_1955	Bacteria	Firmicutes	Clostridia	Clostridiales	Ruminococcaceae	Unknown
ASV_1956	Bacteria	Firmicutes	Clostridia	Clostridiales	Clostridiales_vadinBB60_group	Unknown
ASV_1957	Bacteria	Bacteroidetes	Bacteroidia	Bacteroidales	Muribaculaceae	Unknown
ASV_1958	Bacteria	Bacteroidetes	Bacteroidia	Bacteroidales	Rikenellaceae	Alistipes
ASV_1959	Bacteria	Firmicutes	Clostridia	Clostridiales	Ruminococcaceae	Ruminococcaceae_UCG-014
ASV_1960	Bacteria	Tenericutes	Mollicutes	Mollicutes_RF39		Unknown
ASV_1961	Bacteria	Bacteroidetes	Bacteroidia	Bacteroidales	Muribaculaceae	Unknown
ASV_1962	Bacteria	Firmicutes	Clostridia	Clostridiales	Lachnospiraceae	Unknown
ASV_1963	Bacteria	Firmicutes	Clostridia	Clostridiales		Unknown
ASV_1964	Bacteria	Firmicutes	Clostridia	Clostridiales	Lachnospiraceae	Unknown
ASV_1965	Bacteria	Firmicutes	Clostridia	Clostridiales	Lachnospiraceae	Unknown
ASV_1966	Bacteria	Firmicutes	Clostridia	Clostridiales	Ruminococcaceae	Ruminiclostridium_9
ASV_1967	Bacteria	Firmicutes	Clostridia	Clostridiales	Clostridiales_vadinBB60_group	Unknown
ASV_1968	Bacteria	Bacteroidetes	Bacteroidia	Bacteroidales	Tannerellaceae	Parabacteroides
ASV_1969	Bacteria	Firmicutes	Clostridia	Clostridiales	Lachnospiraceae	Marvinbryantia
ASV_1970	Bacteria					Unknown
ASV_1971	Bacteria	Bacteroidetes	Bacteroidia	Bacteroidales	Rikenellaceae	Alistipes
ASV_1972	Bacteria	Bacteroidetes	Bacteroidia	Bacteroidales	Muribaculaceae	Unknown
ASV_1973	Bacteria	Firmicutes	Clostridia	Clostridiales	Lachnospiraceae	Unknown
ASV_1974	Bacteria	Firmicutes	Clostridia	Clostridiales	Lachnospiraceae	Unknown

ASV_1975	Bacteria	Proteobacteria	Gammaproteobacteria	Pseudomonadales	Moraxellaceae	Acinetobacter	Acinetobacter_johnsonii
ASV_1976	Bacteria	Actinobacteria	Thermoleophilia	Solirubrobacterales	Solirubrobacteraceae	Parviterribacter	
ASV_1977	Bacteria	Bacteroidetes	Bacteroidia	Bacteroidales	Rikenellaceae	Alistipes	
ASV_1978	Bacteria	Bacteroidetes	Bacteroidia	Bacteroidales	Muribaculaceae	Unknown	
ASV_1979	Bacteria	Firmicutes	Clostridia	Clostridiales		Unknown	
ASV_1980	Bacteria	Firmicutes	Clostridia	Clostridiales	Ruminococcaceae	Negativibacillus	Negativibacillus_massiliensis
ASV_1981	Bacteria	Bacteroidetes	Bacteroidia	Bacteroidales	Rikenellaceae	Alistipes	
ASV_1982	Bacteria	Firmicutes	Clostridia	Clostridiales	Ruminococcaceae	Ruminococcaceae_NK4A214_group	
ASV_1983	Bacteria	Firmicutes	Clostridia	Clostridiales	Family_XIII	Family_XIII_UCG-001	
ASV_1984	Bacteria	Firmicutes	Clostridia	Clostridiales	Clostridiales_vadinBB60_group	Unknown	
ASV_1985	Bacteria	Firmicutes	Clostridia	Clostridiales	Clostridiales_vadinBB60_group	Unknown	
ASV_1986	Bacteria	Bacteroidetes	Bacteroidia	Bacteroidales	Bacteroidaceae	Bacteroides	
ASV_1987	Bacteria	Firmicutes	Clostridia	Clostridiales	Lachnospiraceae	Unknown	
ASV_1988	Bacteria	Firmicutes	Clostridia	Clostridiales	Lachnospiraceae	Unknown	
ASV_1989	Bacteria	Firmicutes	Clostridia	Clostridiales	Ruminococcaceae	Ruminiclostridium_5	
ASV_1990	Bacteria	Bacteroidetes	Bacteroidia	Bacteroidales	Muribaculaceae	Unknown	
ASV_1991	Bacteria	Firmicutes	Clostridia	Clostridiales	Lachnospiraceae	Lachnoclostridium	
ASV_1992	Bacteria	Firmicutes	Clostridia	Clostridiales	Clostridiales_vadinBB60_group	Unknown	
ASV_1993	Bacteria	Firmicutes	Clostridia	Clostridiales	Ruminococcaceae	Unknown	
ASV_1994	Bacteria	Firmicutes	Clostridia	Clostridiales	Ruminococcaceae	Ruminiclostridium_9	
ASV_1995	Bacteria	Firmicutes	Clostridia	Clostridiales	Ruminococcaceae	Ruminiclostridium_6	
ASV_1996	Bacteria	Firmicutes	Clostridia	Clostridiales	Lachnospiraceae	Unknown	
ASV_1997	Bacteria	Firmicutes	Clostridia	Clostridiales	Lachnospiraceae	Unknown	
ASV_1998	Bacteria	Tenericutes	Mollicutes	Anaeroplasmatales	Anaeroplasmataceae	Anaeroplasma	
ASV_1999	Bacteria	Firmicutes	Clostridia	Clostridiales	Lachnospiraceae	A2	
ASV_2000	Bacteria	Bacteroidetes	Bacteroidia	Bacteroidales	Muribaculaceae	Unknown	
ASV_2001	Bacteria	Firmicutes	Clostridia	Clostridiales	Ruminococcaceae	Unknown	
ASV_2002	Bacteria	Firmicutes	Clostridia	Clostridiales	Ruminococcaceae	Unknown	
ASV_2003	Bacteria	Firmicutes	Clostridia	Clostridiales	Ruminococcaceae	Faecalibacterium	
ASV_2004	Bacteria	Firmicutes	Clostridia	Clostridiales	Ruminococcaceae	Faecalibacterium	
ASV_2005	Bacteria	Firmicutes	Bacilli	Lactobacillales		Unknown	
ASV_2006	Bacteria	Bacteroidetes	Bacteroidia	Bacteroidales	Prevotellaceae	Prevotellaceae_UCG-001	
ASV_2007	Bacteria	Firmicutes	Clostridia	Clostridiales	Lachnospiraceae	Unknown	
ASV_2008	Bacteria	Firmicutes	Clostridia	Clostridiales	Family_XIII	Unknown	
ASV_2009	Bacteria	Bacteroidetes	Bacteroidia	Bacteroidales	Muribaculaceae	Unknown	
ASV_2010	Bacteria	Bacteroidetes	Bacteroidia			Unknown	
ASV_2011	Bacteria	Firmicutes	Clostridia	Clostridiales	Ruminococcaceae	Butyricicoccus	

ASV_2012	Bacteria	Actinobacteria	Actinobacteria	Micrococcales	Micrococcaceae	Rothia
ASV_2013	Bacteria	Firmicutes	Clostridia	Clostridiales	Family_XIII	Family_XIII_AD3011_group
ASV_2014	Bacteria	Firmicutes	Clostridia	Clostridiales	Ruminococcaceae	Ruminiclostridium
ASV_2015	Bacteria	Firmicutes	Erysipelotrichia	Erysipelotrichales	Erysipelotrichaceae	Faecalitalea
ASV_2016	Bacteria	Bacteroidetes	Bacteroidia	Bacteroidales	Muribaculaceae	Unknown
ASV_2017	Bacteria	Firmicutes	Clostridia	Clostridiales	Ruminococcaceae	Butyricicoccus
ASV_2018	Bacteria	Firmicutes	Clostridia	Clostridiales	Lachnospiraceae	Unknown
ASV_2019	Bacteria	Firmicutes	Erysipelotrichia	Erysipelotrichales	Erysipelotrichaceae	Turicibacter
ASV_2020	Bacteria	Firmicutes	Clostridia	Clostridiales	Ruminococcaceae	Ruminococcaceae_UCG-005
ASV_2021	Bacteria			Unknown		
ASV_2022	Bacteria	Firmicutes	Clostridia	Clostridiales	Ruminococcaceae	Oscillibacter
ASV_2023	Bacteria	Firmicutes	Clostridia	Clostridiales	Ruminococcaceae	Unknown
ASV_2024	Bacteria	Firmicutes	Erysipelotrichia	Erysipelotrichales	Erysipelotrichaceae	Faecalibaculum
ASV_2025	Bacteria	Firmicutes	Clostridia	Clostridiales	Unknown	
ASV_2026	Bacteria	Firmicutes	Clostridia	Clostridiales	Ruminococcaceae	Ruminococcaceae_UCG-014
ASV_2027	Bacteria	Firmicutes	Clostridia	Clostridiales	Lachnospiraceae	Unknown
ASV_2028	Bacteria	Firmicutes	Clostridia	Clostridiales	Lachnospiraceae	Lachnospiraceae_NK4A136_group
ASV_2029	Bacteria	Firmicutes	Erysipelotrichia	Erysipelotrichales	Erysipelotrichaceae	Unknown
ASV_2030	Bacteria	Firmicutes	Clostridia	Clostridiales	Lachnospiraceae	Lachnospiraceae_NK4A136_group
ASV_2031	Bacteria	Firmicutes	Clostridia	Clostridiales	Lachnospiraceae	Unknown
ASV_2032	Bacteria	Firmicutes	Clostridia	Clostridiales	Ruminococcaceae	Unknown
ASV_2033	Bacteria	Firmicutes	Clostridia	Clostridiales	Ruminococcaceae	Ruminiclostridium_9
ASV_2034	Bacteria	Firmicutes	Bacilli	Lactobacillales	Lactobacillaceae	Lactobacillus
ASV_2035	Bacteria	Bacteroidetes	Bacteroidia	Bacteroidales	Muribaculaceae	Unknown
ASV_2036	Bacteria	Firmicutes	Clostridia	Clostridiales	Lachnospiraceae	Lachnospiraceae_NK4A136_group
ASV_2037	Bacteria	Bacteroidetes	Bacteroidia	Bacteroidales	Muribaculaceae	Unknown
ASV_2038	Bacteria	Firmicutes	Clostridia	Clostridiales	Ruminococcaceae	Unknown
ASV_2039	Bacteria	Firmicutes	Clostridia	Clostridiales	Lachnospiraceae	Unknown
ASV_2040	Bacteria	Firmicutes	Clostridia	Clostridiales	Ruminococcaceae	Ruminococcaceae_UCG-003
ASV_2041	Bacteria	Firmicutes	Clostridia	Clostridiales	Lachnospiraceae	Unknown
ASV_2042	Bacteria	Bacteroidetes	Bacteroidia	Bacteroidales	Muribaculaceae	Unknown
ASV_2043	Bacteria	Firmicutes	Clostridia	Clostridiales	Lachnospiraceae	Unknown
ASV_2044	Bacteria	Firmicutes	Clostridia	Clostridiales	Ruminococcaceae	Ruminiclostridium_9
ASV_2045	Bacteria	Bacteroidetes	Bacteroidia	Bacteroidales	Prevotellaceae	Prevotellaceae_NK3B31_group
ASV_2046	Bacteria	Tenericutes	Mollicutes	Mollicutes_RF39	Unknown	
ASV_2047	Bacteria	Firmicutes	Clostridia	Clostridiales	Lachnospiraceae	Lachnoclostridium
ASV_2048	Bacteria	Firmicutes	Clostridia	Clostridiales	Ruminococcaceae	Ruminococcaceae_UCG-014

ASV_2049	Bacteria	Firmicutes	Unknown			
ASV_2050	Bacteria	Actinobacteria	Coriobacteriia	Coriobacteriales	Eggerthellaceae	Denitrobacterium
ASV_2051	Bacteria	Firmicutes	Clostridia	Clostridiales	Clostridiales_vadinBB60_group	Unknown
ASV_2052	Bacteria	Firmicutes	Clostridia	Clostridiales	Ruminococcaceae	Ruminiclostridium_9
ASV_2053	Bacteria	Firmicutes	Clostridia	Clostridiales	Lachnospiraceae	Unknown
ASV_2054	Bacteria	Bacteroidetes	Bacteroidia	Bacteroidales	Prevotellaceae	Prevotellaceae_UCG-001
ASV_2055	Bacteria	Firmicutes	Clostridia	Clostridiales	Peptococcaceae	Unknown
ASV_2056	Bacteria	Firmicutes	Clostridia	Clostridiales	Clostridiales_vadinBB60_group	Unknown
ASV_2057	Bacteria	Firmicutes	Clostridia	Clostridiales	Unknown	
ASV_2058	Bacteria	Bacteroidetes	Bacteroidia	Bacteroidales	Rikenellaceae	Alistipes
ASV_2059	Bacteria	Bacteroidetes	Bacteroidia	Bacteroidales	Muribaculaceae	Unknown
ASV_2060	Bacteria	Bacteroidetes	Bacteroidia	Unknown		
ASV_2061	Bacteria	Firmicutes	Clostridia	Clostridiales	Lachnospiraceae	Unknown
ASV_2062	Bacteria	Firmicutes	Clostridia	Clostridiales	Lachnospiraceae	Unknown
ASV_2063	Bacteria	Firmicutes	Clostridia	Clostridiales	Lachnospiraceae	Lachnospiraceae_NK4A136_group
ASV_2064	Bacteria	Bacteroidetes	Bacteroidia	Bacteroidales	Marinifilaceae	Odoribacter
ASV_2065	Bacteria	Firmicutes	Bacilli	Bacillales	Listeriaceae	Brochothrix
ASV_2066	Bacteria	Bacteroidetes	Bacteroidia	Bacteroidales	Muribaculaceae	Unknown
ASV_2067	Bacteria	Firmicutes	Clostridia	Clostridiales	Unknown	
ASV_2068	Bacteria	Bacteroidetes	Bacteroidia	Bacteroidales	Prevotellaceae	Prevotellaceae_UCG-001
ASV_2069	Bacteria	Firmicutes	Clostridia	Clostridiales	Lachnospiraceae	Unknown
ASV_2070	Bacteria	Bacteroidetes	Bacteroidia	Bacteroidales	Muribaculaceae	Unknown
ASV_2071	Bacteria	Firmicutes	Clostridia	Clostridiales	Lachnospiraceae	GCA-900066575
ASV_2072	Bacteria	Firmicutes	Clostridia	Clostridiales	Lachnospiraceae	Lachnospiraceae_NK4A136_group
ASV_2073	Bacteria	Bacteroidetes	Bacteroidia	Bacteroidales	Muribaculaceae	Unknown
ASV_2074	Bacteria	Firmicutes	Clostridia	Clostridiales	Unknown	
ASV_2075	Bacteria	Bacteroidetes	Bacteroidia	Bacteroidales	Muribaculaceae	CAG-873
ASV_2076	Bacteria	Bacteroidetes	Bacteroidia	Bacteroidales	Muribaculaceae	Unknown
ASV_2077	Bacteria	Bacteroidetes	Bacteroidia	Bacteroidales	Muribaculaceae	Unknown
ASV_2078	Bacteria	Firmicutes	Bacilli	Lactobacillales	Lactobacillaceae	Lactobacillus
ASV_2079	Bacteria	Firmicutes	Clostridia	Clostridiales	Lachnospiraceae	Unknown
ASV_2080	Bacteria	Bacteroidetes	Bacteroidia	Bacteroidales	Muribaculaceae	Unknown
ASV_2081	Bacteria	Bacteroidetes	Bacteroidia	Bacteroidales	Bacteroidaceae	Bacteroides
ASV_2082	Bacteria	Firmicutes	Clostridia	Clostridiales	Lachnospiraceae	Unknown
ASV_2083	Bacteria	Bacteroidetes	Bacteroidia	Bacteroidales	Prevotellaceae	Prevotellaceae_UCG-001
ASV_2084	Bacteria	Firmicutes	Clostridia	Clostridiales	Lachnospiraceae	Lachnospiraceae_NK4A136_group
ASV_2085	Bacteria	Bacteroidetes	Bacteroidia	Bacteroidales	Prevotellaceae	Prevotellaceae_UCG-001

ASV_2086	Bacteria	Firmicutes	Clostridia	Clostridiales	Ruminococcaceae	Anaerotruncus
ASV_2087	Bacteria	Firmicutes	Clostridia	Clostridiales	Lachnospiraceae	Lachnospiraceae_NK4A136_group
ASV_2088	Bacteria	Firmicutes	Clostridia	Clostridiales	Christensenellaceae	Christensenellaceae_R-7_group
ASV_2089	Bacteria	Firmicutes	Clostridia	Clostridiales	Lachnospiraceae	Lachnospiraceae_NK4A136_group
ASV_2090	Bacteria	Firmicutes	Clostridia	Clostridiales	Peptostreptococcaceae	Romboutsia
ASV_2091	Bacteria	Firmicutes	Clostridia	Clostridiales	Ruminococcaceae	Ruminococcaceae_UCG-014
ASV_2092	Bacteria	Firmicutes	Clostridia	Clostridiales	Lachnospiraceae	Unknown
ASV_2093	Bacteria	Firmicutes	Clostridia	Clostridiales	Ruminococcaceae	Ruminiclostridium_6
ASV_2094	Bacteria	Firmicutes	Clostridia	Clostridiales	Unknown	
ASV_2095	Bacteria	Firmicutes	Bacilli	Lactobacillales	Lactobacillaceae	Lactobacillus
ASV_2096	Bacteria	Firmicutes	Bacilli	Lactobacillales	Lactobacillaceae	Lactobacillus
ASV_2097	Bacteria	Firmicutes	Bacilli	Lactobacillales	Lactobacillaceae	Lactobacillus
ASV_2098	Bacteria	Tenericutes	Mollicutes	Mollicutes_RF39	Unknown	
ASV_2099	Bacteria	Bacteroidetes	Bacteroidia	Bacteroidales	Rikenellaceae	Alistipes
ASV_2100	Bacteria	Firmicutes	Clostridia	Clostridiales	Ruminococcaceae	Unknown
ASV_2101	Bacteria	Firmicutes	Clostridia	Clostridiales	Ruminococcaceae	Oscillibacter
ASV_2102	Bacteria	Bacteroidetes	Bacteroidia	Bacteroidales	Prevotellaceae	Prevotellaceae_NK3B31_group
ASV_2103	Bacteria	Bacteroidetes	Bacteroidia	Bacteroidales	Prevotellaceae	Prevotellaceae_NK3B31_group
ASV_2104	Bacteria	Firmicutes	Clostridia	Clostridiales	Lachnospiraceae	Unknown
ASV_2105	Bacteria	Firmicutes	Clostridia	Clostridiales	Ruminococcaceae	Ruminiclostridium
ASV_2106	Bacteria	Firmicutes	Clostridia	Clostridiales	Lachnospiraceae	Unknown
ASV_2107	Bacteria	Bacteroidetes	Bacteroidia	Bacteroidales	Muribaculaceae	CAG-873
ASV_2108	Bacteria	Firmicutes	Clostridia	Clostridiales	Clostridiales_vadinBB60_group	Unknown
ASV_2109	Bacteria	Firmicutes	Clostridia	Clostridiales	Ruminococcaceae	Unknown
ASV_2110	Bacteria	Firmicutes	Bacilli	Lactobacillales	Lactobacillaceae	Lactobacillus
ASV_2111	Bacteria	Bacteroidetes	Bacteroidia	Bacteroidales	Rikenellaceae	Alistipes
ASV_2112	Bacteria	Bacteroidetes	Bacteroidia	Bacteroidales	Muribaculaceae	Unknown
ASV_2113	Bacteria	Firmicutes	Clostridia	Clostridiales	Lachnospiraceae	Unknown
ASV_2114	Bacteria	Firmicutes	Bacilli	Lactobacillales	Lactobacillaceae	Lactobacillus
ASV_2115	Bacteria	Firmicutes	Clostridia	Clostridiales	Lachnospiraceae	Unknown
ASV_2116	Bacteria	Bacteroidetes	Bacteroidia	Bacteroidales	Muribaculaceae	Unknown
ASV_2117	Bacteria	Bacteroidetes	Bacteroidia	Bacteroidales	Muribaculaceae	Unknown
ASV_2118	Bacteria	Firmicutes	Clostridia	Clostridiales	Ruminococcaceae	Ruminiclostridium
ASV_2119	Bacteria	Firmicutes	Clostridia	Clostridiales	Ruminococcaceae	Ruminococcaceae_UCG-014
ASV_2120	Bacteria	Firmicutes	Erysipelotrichia	Erysipelotrichales	Erysipelotrichaceae	Unknown
ASV_2121	Bacteria	Firmicutes	Clostridia	Clostridiales	Ruminococcaceae	Ruminococcaceae_UCG-014
ASV_2122	Bacteria	Firmicutes	Clostridia	Clostridiales	Ruminococcaceae	Anaerotruncus

ASV_2123	Bacteria	Firmicutes	Clostridia	Clostridiales	Ruminococcaceae	Ruminococcaceae_UCG-014
ASV_2124	Bacteria	Firmicutes	Erysipelotrichia	Erysipelotrichales	Erysipelotrichaceae	Candidatus_Stoquefichus
ASV_2125	Bacteria	Bacteroidetes	Bacteroidia	Bacteroidales	Rikenellaceae	Alistipes
ASV_2126	Bacteria	Firmicutes	Clostridia	Clostridiales	Family_XIII	Family_XIII_UCG-001
ASV_2127	Bacteria	Bacteroidetes	Bacteroidia	Bacteroidales	Prevotellaceae	Prevotellaceae_NK3B31_group
ASV_2128	Bacteria	Firmicutes	Clostridia	Clostridiales	Clostridiales_vadinBB60_group	Unknown
ASV_2129	Bacteria	Firmicutes	Clostridia	Clostridiales	Lachnospiraceae	Lachnospiraceae_NK4A136_group
ASV_2130	Bacteria	Firmicutes	Clostridia	Clostridiales	Ruminococcaceae	Unknown
ASV_2131	Bacteria	Firmicutes	Clostridia	Clostridiales	Lachnospiraceae	Unknown
ASV_2132	Bacteria	Bacteroidetes	Bacteroidia	Bacteroidales	Rikenellaceae	Alistipes
ASV_2133	Bacteria	Bacteroidetes	Bacteroidia	Bacteroidales	Bacteroidaceae	Bacteroides
ASV_2134	Bacteria	Firmicutes	Clostridia	Clostridiales	Lachnospiraceae	Unknown
ASV_2135	Bacteria	Firmicutes	Clostridia	Clostridiales	Lachnospiraceae	Unknown
ASV_2136	Bacteria	Firmicutes	Clostridia	Clostridiales		Unknown
ASV_2137	Bacteria	Firmicutes	Clostridia	Clostridiales	Lachnospiraceae	Unknown
ASV_2138	Bacteria	Firmicutes	Erysipelotrichia	Erysipelotrichales	Erysipelotrichaceae	Turicibacter
ASV_2139	Bacteria	Firmicutes	Clostridia	Clostridiales	Lachnospiraceae	Lachnospiraceae_NK4A136_group
ASV_2140	Bacteria	Firmicutes	Clostridia	Clostridiales	Lachnospiraceae	CAG-56
ASV_2141	Bacteria	Firmicutes	Clostridia	Clostridiales	Lachnospiraceae	Unknown
ASV_2142	Bacteria	Cyanobacteria	Melainabacteria	Gastranaerophilales		Unknown
ASV_2143	Bacteria					Unknown
ASV_2144	Bacteria	Firmicutes	Clostridia	Clostridiales	Ruminococcaceae	Ruminococcaceae_NK4A214_group
ASV_2145	Bacteria	Firmicutes	Clostridia	Clostridiales	Ruminococcaceae	Ruminococcus_1
ASV_2146	Bacteria	Firmicutes	Clostridia	Clostridiales	Ruminococcaceae	Unknown
ASV_2147	Bacteria	Firmicutes	Clostridia	Clostridiales	Lachnospiraceae	Lachnospiraceae_NK4A136_group
ASV_2148	Bacteria	Bacteroidetes	Bacteroidia	Bacteroidales	Rikenellaceae	Unknown
ASV_2149	Bacteria	Firmicutes	Clostridia	Clostridiales	Lachnospiraceae	Lachnospiraceae_NK4A136_group
ASV_2150	Bacteria	Firmicutes	Clostridia	Clostridiales	Clostridiaceae_1	Candidatus_Arthromitus
ASV_2151	Bacteria	Firmicutes	Bacilli	Lactobacillales	Lactobacillaceae	Lactobacillus
ASV_2152	Bacteria	Firmicutes	Clostridia	Clostridiales	Lachnospiraceae	Unknown
ASV_2153	Bacteria	Deferribacteres	Deferribacteres	Deferribacterales	Deferribacteraceae	Mucispirillum
ASV_2154	Bacteria	Firmicutes	Clostridia	Clostridiales	Ruminococcaceae	Faecalibacterium
ASV_2155	Bacteria	Firmicutes	Bacilli	Bacillales	Thermoactinomycetaceae	Laceyella
ASV_2156	Bacteria	Bacteroidetes	Bacteroidia	Bacteroidales	Muribaculaceae	Unknown
ASV_2157	Bacteria	Firmicutes	Clostridia	Clostridiales	Clostridiales_vadinBB60_group	Unknown
ASV_2158	Bacteria	Firmicutes	Clostridia	Clostridiales	Lachnospiraceae	Lachnospiraceae_UCG-001
ASV_2159	Bacteria	Bacteroidetes	Bacteroidia	Bacteroidales		Unknown

ASV_2160	Bacteria	Firmicutes	Clostridia	Clostridiales	Christensenellaceae	Christensenella
ASV_2161	Bacteria	Firmicutes	Clostridia	Clostridiales	Clostridiales_vadinBB60_group	Unknown
ASV_2162	Bacteria	Firmicutes	Clostridia	Clostridiales	Ruminococcaceae	Unknown
ASV_2163	Bacteria	Firmicutes	Clostridia	Clostridiales	Ruminococcaceae	Anaerotruncus
ASV_2164	Bacteria	Firmicutes	Clostridia	Clostridiales	Lachnospiraceae	Lachnospiraceae_NK4A136_group
ASV_2165	Bacteria	Firmicutes	Clostridia	Clostridiales	Lachnospiraceae	Unknown
ASV_2166	Bacteria	Firmicutes	Clostridia	Clostridiales	Lachnospiraceae	Lachnospiraceae_NK4A136_group
ASV_2167	Bacteria	Bacteroidetes	Bacteroidia			Unknown
ASV_2168	Bacteria	Bacteroidetes	Bacteroidia	Bacteroidales	Muribaculaceae	Unknown
ASV_2169	Bacteria	Bacteroidetes	Bacteroidia	Bacteroidales	Muribaculaceae	Unknown
ASV_2170	Bacteria	Firmicutes	Clostridia	Clostridiales	Ruminococcaceae	Ruminiclostridium_9
ASV_2171	Bacteria	Bacteroidetes	Bacteroidia	Bacteroidales	Muribaculaceae	Unknown
ASV_2172	Bacteria	Firmicutes	Bacilli	Lactobacillales	Lactobacillaceae	Lactobacillus
ASV_2173	Bacteria	Firmicutes	Clostridia	Clostridiales	Lachnospiraceae	Unknown
ASV_2174	Bacteria	Proteobacteria	Alphaproteobacteria	Rhodospirillales		Unknown
ASV_2175	Bacteria	Firmicutes	Clostridia	Clostridiales		Unknown
ASV_2176	Bacteria	Bacteroidetes	Bacteroidia	Bacteroidales	Rikenellaceae	Alistipes
ASV_2177	Bacteria	Firmicutes	Erysipelotrichia	Erysipelotrichales	Erysipelotrichaceae	Candidatus_Stoquefichus
ASV_2178	Bacteria	Firmicutes	Clostridia	Clostridiales	Lachnospiraceae	Unknown
ASV_2179	Bacteria	Firmicutes	Clostridia	Clostridiales	Ruminococcaceae	Ruminiclostridium
ASV_2180	Bacteria	Firmicutes	Clostridia	Clostridiales	Lachnospiraceae	Lachnospiraceae_NK4A136_group
ASV_2181	Bacteria	Firmicutes	Clostridia	Clostridiales	Lachnospiraceae	Lachnospiraceae_NK4A136_group
ASV_2182	Bacteria	Bacteroidetes	Bacteroidia	Bacteroidales	Prevotellaceae	Prevotellaceae_UCG-001
ASV_2183	Bacteria	Firmicutes	Clostridia	Clostridiales	Christensenellaceae	Unknown
ASV_2184	Bacteria	Firmicutes	Bacilli	Lactobacillales	Aerococcaceae	Aerococcus
ASV_2185	Bacteria	Firmicutes	Clostridia	Clostridiales	Lachnospiraceae	Tyzzerella
ASV_2186	Bacteria	Firmicutes	Clostridia	Clostridiales	Lachnospiraceae	A2
ASV_2187	Bacteria	Bacteroidetes	Bacteroidia	Bacteroidales	Muribaculaceae	Unknown
ASV_2188	Bacteria	Proteobacteria	Alphaproteobacteria			Unknown
ASV_2189	Bacteria	Bacteroidetes	Bacteroidia	Bacteroidales	Muribaculaceae	Unknown
ASV_2190	Bacteria	Bacteroidetes	Bacteroidia	Bacteroidales	Muribaculaceae	Unknown
ASV_2191	Bacteria	Bacteroidetes	Bacteroidia	Bacteroidales	Muribaculaceae	Unknown
ASV_2192	Bacteria	Firmicutes	Clostridia	Clostridiales	Ruminococcaceae	Unknown
ASV_2193	Bacteria	Firmicutes	Clostridia	Clostridiales	Ruminococcaceae	Ruminococcaceae_UCG-014
ASV_2194	Bacteria	Tenericutes	Mollicutes	Mollicutes_RF39		Unknown
ASV_2195	Bacteria	Bacteroidetes	Bacteroidia	Bacteroidales	Muribaculaceae	CAG-873
ASV_2196	Bacteria	Bacteroidetes	Bacteroidia	Bacteroidales	Prevotellaceae	Prevotellaceae_NK3B31_group

ASV_2197	Bacteria	Firmicutes	Clostridia	Clostridiales	Lachnospiraceae	ASF356
ASV_2198	Bacteria	Bacteroidetes	Bacteroidia	Bacteroidales	Unknown	
ASV_2199	Bacteria	Firmicutes	Clostridia	Clostridiales	Christensenellaceae	Christensenellaceae_R-7_group
ASV_2200	Bacteria	Firmicutes	Clostridia	Clostridiales	Lachnospiraceae	Unknown
ASV_2201	Bacteria	Firmicutes	Clostridia	Clostridiales	Lachnospiraceae	Lachnospiraceae_NK4A136_group
ASV_2202	Bacteria	Bacteroidetes	Bacteroidia	Bacteroidales	Rikenellaceae	Alistipes
ASV_2203	Bacteria	Firmicutes	Clostridia	Clostridiales	Lachnospiraceae	Unknown
ASV_2204	Bacteria	Bacteroidetes	Bacteroidia	Bacteroidales	Muribaculaceae	CAG-873
ASV_2205	Bacteria	Bacteroidetes	Bacteroidia	Bacteroidales	Muribaculaceae	Unknown
ASV_2206	Bacteria	Firmicutes	Clostridia	Clostridiales	Ruminococcaceae	Ruminiclostridium_9
ASV_2207	Bacteria	Firmicutes	Clostridia	Clostridiales	Christensenellaceae	Unknown
ASV_2208	Bacteria	Firmicutes	Clostridia	Clostridiales	Lachnospiraceae	Unknown
ASV_2209	Bacteria	Firmicutes	Bacilli	Lactobacillales	Lactobacillaceae	Lactobacillus
ASV_2210	Bacteria	Firmicutes	Clostridia	Clostridiales	Ruminococcaceae	Ruminococcaceae_UCG-013
ASV_2211	Bacteria	Tenericutes	Mollicutes	Mollicutes_RF39	Unknown	
ASV_2212	Bacteria	Bacteroidetes	Bacteroidia	Bacteroidales	Muribaculaceae	Unknown
ASV_2213	Bacteria	Bacteroidetes	Bacteroidia	Bacteroidales	Muribaculaceae	Unknown
ASV_2214	Bacteria	Tenericutes	Mollicutes	Mollicutes_RF39	Unknown	
ASV_2215	Bacteria	Bacteroidetes	Bacteroidia	Bacteroidales	Muribaculaceae	Unknown
ASV_2216	Bacteria	Actinobacteria	Coriobacteriia	Coriobacteriales	Eggerthellaceae	Denitrobacterium
ASV_2217	Bacteria	Firmicutes	Clostridia	Clostridiales	Lachnospiraceae	Unknown
ASV_2218	Bacteria	Bacteroidetes	Bacteroidia	Bacteroidales	Rikenellaceae	Alistipes
ASV_2219	Bacteria	Cyanobacteria	Oxyphotobacteria	Chloroplast	Unknown	
ASV_2220	Bacteria	Firmicutes	Erysipelotrichia	Erysipelotrichales	Erysipelotrichaceae	Faecalibaculum
ASV_2221	Bacteria	Firmicutes	Bacilli	Lactobacillales	Lactobacillaceae	Lactobacillus
ASV_2222	Bacteria	Bacteroidetes	Bacteroidia	Bacteroidales	Rikenellaceae	Alistipes
ASV_2223	Bacteria	Firmicutes	Clostridia	Clostridiales	Lachnospiraceae	ASF356
ASV_2224	Bacteria	Firmicutes	Clostridia	Clostridiales	Lachnospiraceae	Lachnospiraceae_NK4A136_group
ASV_2225	Bacteria	Bacteroidetes	Bacteroidia	Bacteroidales	Muribaculaceae	Unknown
ASV_2226	Bacteria	Firmicutes	Clostridia	Clostridiales	Lachnospiraceae	Unknown
ASV_2227	Bacteria	Bacteroidetes	Bacteroidia	Bacteroidales	Muribaculaceae	Unknown
ASV_2228	Bacteria	Firmicutes	Erysipelotrichia	Erysipelotrichales	Erysipelotrichaceae	Unknown
ASV_2229	Bacteria	Bacteroidetes	Bacteroidia	Bacteroidales	Rikenellaceae	Alistipes
ASV_2230	Bacteria	Firmicutes	Clostridia	Clostridiales	Lachnospiraceae	Lachnospiraceae_NK4A136_group
ASV_2231	Bacteria	Firmicutes	Clostridia	Clostridiales	Ruminococcaceae	Ruminococcaceae_UCG-013
ASV_2232	Bacteria	Firmicutes	Clostridia	Clostridiales	Ruminococcaceae	Unknown
ASV_2233	Bacteria	Firmicutes	Clostridia	Clostridiales	Ruminococcaceae	Ruminiclostridium_9

ASV_2234	Bacteria	Firmicutes	Clostridia	Clostridiales	Lachnospiraceae	Unknown	
ASV_2235	Bacteria	Firmicutes	Clostridia	Clostridiales	Lachnospiraceae	Unknown	
ASV_2236	Bacteria	Firmicutes	Clostridia	Clostridiales	Clostridiales_vadinBB60_group	Unknown	
ASV_2237	Bacteria	Firmicutes	Clostridia	Clostridiales	Lachnospiraceae	Marvinbryantia	
ASV_2238	Bacteria	Firmicutes	Clostridia	Clostridiales	Lachnospiraceae	Unknown	
ASV_2239	Bacteria	Firmicutes	Clostridia	Clostridiales	Ruminococcaceae	Unknown	
ASV_2240	Bacteria	Firmicutes	Clostridia	Clostridiales	Lachnospiraceae	Unknown	
ASV_2241	Bacteria	Firmicutes	Erysipelotrichia	Erysipelotrichales	Erysipelotrichaceae	Unknown	
ASV_2242	Bacteria	Firmicutes	Clostridia	Clostridiales	Lachnospiraceae	Lachnospiraceae_NK4A136_group	
ASV_2243	Bacteria	Firmicutes	Clostridia	Clostridiales	Ruminococcaceae	Unknown	
ASV_2244	Bacteria	Bacteroidetes	Bacteroidia	Bacteroidales	Muribaculaceae	CAG-873	
ASV_2245	Bacteria	Firmicutes	Clostridia	Clostridiales	Ruminococcaceae	Ruminococcaceae_UCG-014	
ASV_2246	Bacteria	Bacteroidetes	Bacteroidia	Bacteroidales	Rikenellaceae	Unknown	
ASV_2247	Bacteria	Firmicutes	Clostridia	Clostridiales	Lachnospiraceae	Lachnospiraceae_UCG-001	
ASV_2248	Bacteria	Firmicutes	Clostridia	Clostridiales	Lachnospiraceae	Lachnospiraceae_NK4A136_group	
ASV_2249	Bacteria	Bacteroidetes	Bacteroidia	Bacteroidales	Muribaculaceae	Unknown	
ASV_2250	Bacteria	Cyanobacteria	Melainabacteria	Gastranaerophilales		Unknown	
ASV_2251	Bacteria	Bacteroidetes	Bacteroidia	Bacteroidales	Muribaculaceae	Unknown	
ASV_2252	Bacteria	Firmicutes	Clostridia	Clostridiales	Ruminococcaceae	Ruminococcaceae_UCG-014	
ASV_2253	Bacteria	Cyanobacteria	Melainabacteria	Gastranaerophilales		Unknown	
ASV_2254	Bacteria	Actinobacteria	Coriobacteriia	Coriobacteriales	Eggerthellaceae	Eggerthella	Eggerthella_lenta
ASV_2255	Bacteria	Bacteroidetes	Bacteroidia	Bacteroidales	Muribaculaceae	Unknown	
ASV_2256	Bacteria	Firmicutes	Clostridia	Clostridiales	Lachnospiraceae	Lachnospiraceae_NK4A136_group	
ASV_2257	Bacteria	Firmicutes	Clostridia	Clostridiales	Lachnospiraceae	Lachnospiraceae_FCS020_group	
ASV_2258	Bacteria	Bacteroidetes	Bacteroidia	Bacteroidales	Muribaculaceae	Unknown	
ASV_2259	Bacteria	Firmicutes	Clostridia	Clostridiales	Lachnospiraceae	Unknown	
ASV_2260	Bacteria	Firmicutes	Clostridia	Clostridiales	Lachnospiraceae	Unknown	
ASV_2261	Bacteria	Bacteroidetes	Bacteroidia	Bacteroidales	Prevotellaceae	Prevotellaceae_NK3B31_group	
ASV_2262	Bacteria	Firmicutes	Clostridia	Clostridiales	Lachnospiraceae	Unknown	
ASV_2263	Bacteria	Bacteroidetes	Bacteroidia	Bacteroidales	Muribaculaceae	Muribaculum	
ASV_2264	Bacteria	Bacteroidetes	Bacteroidia	Bacteroidales		Unknown	
ASV_2265	Bacteria	Firmicutes	Clostridia	Clostridiales	Lachnospiraceae	Lachnospiraceae_NK4A136_group	
ASV_2266	Bacteria	Firmicutes	Clostridia	Clostridiales	Ruminococcaceae	UBA1819	
ASV_2267	Bacteria	Deferribacteres	Deferribacteres	Deferribacterales	Deferribacteraceae	Mucispirillum	
ASV_2268	Bacteria	Firmicutes	Clostridia	Clostridiales		Unknown	
ASV_2269	Bacteria	Bacteroidetes	Bacteroidia	Bacteroidales	Muribaculaceae	Unknown	
ASV_2270	Bacteria	Bacteroidetes	Bacteroidia	Bacteroidales	Muribaculaceae	Unknown	

ASV_2271	Bacteria	Bacteroidetes	Bacteroidia	Bacteroidales	Prevotellaceae	Prevotellaceae_UCG-001
ASV_2272	Bacteria	Firmicutes	Clostridia	Clostridiales	Lachnospiraceae	A2
ASV_2273	Bacteria	Firmicutes	Clostridia	Clostridiales	Ruminococcaceae	Unknown
ASV_2274	Bacteria			Unknown		
ASV_2275	Bacteria	Firmicutes	Clostridia	Clostridiales	Lachnospiraceae	Unknown
ASV_2276	Bacteria	Firmicutes	Clostridia	Clostridiales	Lachnospiraceae	Unknown
ASV_2277	Bacteria	Tenericutes	Mollicutes	Mollicutes_RF39	Unknown	
ASV_2278	Bacteria	Tenericutes	Mollicutes	Mollicutes_RF39	Unknown	
ASV_2279	Bacteria	Firmicutes	Bacilli	Lactobacillales	Streptococcaceae	Streptococcus
ASV_2280	Bacteria	Firmicutes	Clostridia	Clostridiales	Clostridiales_vadinBB60_group	Unknown
ASV_2281	Bacteria	Bacteroidetes	Bacteroidia	Bacteroidales	Muribaculaceae	Unknown
ASV_2282	Bacteria	Firmicutes	Bacilli	Lactobacillales	Lactobacillaceae	Lactobacillus
ASV_2283	Bacteria	Firmicutes	Clostridia	Clostridiales	Lachnospiraceae	Unknown
ASV_2284	Bacteria	Firmicutes	Clostridia	Clostridiales	Lachnospiraceae	Unknown
ASV_2285	Bacteria	Bacteroidetes	Bacteroidia	Bacteroidales	Muribaculaceae	Unknown
ASV_2286	Bacteria	Firmicutes	Clostridia	Clostridiales	Clostridiaceae_1	Clostridium_sensu_stricto_1
ASV_2287	Bacteria	Firmicutes	Clostridia	Clostridiales	Ruminococcaceae	Ruminococcaceae_UCG-014
ASV_2288	Bacteria	Firmicutes	Clostridia	Clostridiales	Lachnospiraceae	Lachnospiraceae_NK4A136_group
ASV_2289	Bacteria	Bacteroidetes	Bacteroidia	Bacteroidales	Muribaculaceae	Unknown
ASV_2290	Bacteria	Firmicutes	Clostridia	Clostridiales	Ruminococcaceae	Ruminiclostridium_9
ASV_2291	Bacteria	Firmicutes	Clostridia	Clostridiales	Lachnospiraceae	Lachnospiraceae_NK4A136_group
ASV_2292	Bacteria	Firmicutes	Clostridia	Clostridiales	Christensenellaceae	Christensenellaceae_R-7_group
ASV_2293	Bacteria	Tenericutes	Mollicutes	Mollicutes_RF39	Unknown	
ASV_2294	Bacteria	Bacteroidetes	Bacteroidia	Bacteroidales	Muribaculaceae	Unknown
ASV_2295	Bacteria	Bacteroidetes	Bacteroidia	Bacteroidales	Muribaculaceae	CAG-873
ASV_2296	Bacteria	Firmicutes	Clostridia	Clostridiales	Ruminococcaceae	Ruminiclostridium_9
ASV_2297	Bacteria	Bacteroidetes	Bacteroidia	Bacteroidales	Muribaculaceae	Muribaculum
ASV_2298	Bacteria	Bacteroidetes	Bacteroidia	Bacteroidales	Bacteroidaceae	Bacteroides
ASV_2299	Bacteria	Firmicutes	Clostridia	Clostridiales	Lachnospiraceae	Lachnospiraceae_NK4A136_group
ASV_2300	Bacteria	Bacteroidetes	Bacteroidia	Bacteroidales	Muribaculaceae	Unknown
ASV_2301	Bacteria	Firmicutes	Clostridia	Clostridiales	Lachnospiraceae	Unknown
ASV_2302	Bacteria	Firmicutes	Clostridia	Clostridiales	Ruminococcaceae	Ruminococcaceae_UCG-014
ASV_2303	Bacteria	Firmicutes	Clostridia	Clostridiales	Ruminococcaceae	Unknown
ASV_2304	Bacteria	Bacteroidetes	Bacteroidia	Bacteroidales	Prevotellaceae	Prevotellaceae_UCG-001
ASV_2305	Bacteria	Bacteroidetes	Bacteroidia	Bacteroidales	Prevotellaceae	Prevotellaceae_UCG-001
ASV_2306	Bacteria	Bacteroidetes	Bacteroidia	Bacteroidales	Muribaculaceae	Unknown
ASV_2307	Bacteria	Bacteroidetes	Bacteroidia	Bacteroidales	Prevotellaceae	Prevotellaceae_UCG-001

ASV_2308	Bacteria	Firmicutes	Clostridia	Clostridiales	Lachnospiraceae	Unknown
ASV_2309	Bacteria	Firmicutes	Clostridia	Clostridiales	Ruminococcaceae	Ruminococcaceae_UCG-014
ASV_2310	Bacteria	Firmicutes	Clostridia	Clostridiales	Lachnospiraceae	Unknown
ASV_2311	Bacteria	Bacteroidetes	Bacteroidia	Bacteroidales	Prevotellaceae	Prevotellaceae_UCG-001
ASV_2312	Bacteria	Firmicutes	Clostridia	Clostridiales	Ruminococcaceae	Unknown
ASV_2313	Bacteria	Cyanobacteria	Melainabacteria	Gastranaerophilales	Unknown	
ASV_2314	Bacteria	Bacteroidetes	Bacteroidia		Unknown	
ASV_2315	Bacteria	Firmicutes	Clostridia	Clostridiales	Clostridiales_vadinBB60_group	Unknown
ASV_2316	Bacteria	Firmicutes	Clostridia	Clostridiales	Ruminococcaceae	Ruminococcaceae_UCG-014
ASV_2317	Bacteria	Firmicutes	Clostridia	Clostridiales	Ruminococcaceae	Anaerotruncus
ASV_2318	Bacteria	Tenericutes	Mollicutes	Mollicutes_RF39	Unknown	
ASV_2319	Bacteria	Firmicutes	Clostridia	Clostridiales	Ruminococcaceae	Ruminococcaceae_UCG-014
ASV_2320	Bacteria	Firmicutes	Clostridia	Clostridiales	Unknown	
ASV_2321	Bacteria	Firmicutes	Clostridia	Clostridiales	Ruminococcaceae	Unknown
ASV_2322	Bacteria	Firmicutes	Clostridia	Clostridiales	Lachnospiraceae	Unknown
ASV_2323	Bacteria	Tenericutes	Mollicutes	Mollicutes_RF39	Unknown	
ASV_2324	Bacteria	Proteobacteria	Gammaproteobacteria	Betaproteobacteriales	Burkholderiaceae	Sutterella
ASV_2325	Bacteria	Bacteroidetes	Bacteroidia	Bacteroidales	Muribaculaceae	Unknown
ASV_2326	Bacteria	Cyanobacteria	Melainabacteria	Gastranaerophilales	Unknown	
ASV_2327	Bacteria	Firmicutes	Clostridia	Clostridiales	Ruminococcaceae	Oscillibacter
ASV_2328	Bacteria	Bacteroidetes	Bacteroidia	Bacteroidales	Muribaculaceae	Unknown
ASV_2329	Bacteria	Firmicutes	Clostridia	Clostridiales	Ruminococcaceae	Ruminococcaceae_UCG-014
ASV_2330	Bacteria	Firmicutes	Bacilli	Unknown		
ASV_2331	Bacteria	Firmicutes	Clostridia	Clostridiales	Ruminococcaceae	Ruminococcaceae_UCG-009
ASV_2332	Bacteria	Firmicutes	Clostridia	Clostridiales	Lachnospiraceae	Lachnospiraceae_NK4A136_group
ASV_2333	Bacteria	Firmicutes	Clostridia	Clostridiales	Ruminococcaceae	Unknown
ASV_2334	Bacteria	Bacteroidetes	Bacteroidia	Bacteroidales	Rikenellaceae	Alistipes
ASV_2335	Bacteria	Firmicutes	Clostridia	Clostridiales	Clostridiales_vadinBB60_group	Unknown
ASV_2336	Bacteria	Firmicutes	Clostridia	Clostridiales	Ruminococcaceae	Oscillibacter
ASV_2337	Bacteria	Firmicutes	Clostridia	Clostridiales	Unknown	
ASV_2338	Bacteria	Firmicutes	Clostridia	Clostridiales	Lachnospiraceae	Lachnospiraceae_NK4A136_group
ASV_2339	Bacteria	Bacteroidetes	Bacteroidia	Bacteroidales	Muribaculaceae	Unknown
ASV_2340	Bacteria	Firmicutes	Clostridia	Clostridiales	Ruminococcaceae	Ruminiclostridium
ASV_2341	Bacteria	Firmicutes	Clostridia	Clostridiales	Unknown	
ASV_2342	Bacteria	Bacteroidetes	Bacteroidia	Bacteroidales	Muribaculaceae	Unknown
ASV_2343	Bacteria	Firmicutes	Clostridia	Clostridiales	Lachnospiraceae	Unknown
ASV_2344	Bacteria	Bacteroidetes	Bacteroidia	Bacteroidales	Muribaculaceae	Unknown

ASV_2345	Bacteria	Firmicutes	Clostridia	Clostridiales	Lachnospiraceae	Unknown
ASV_2346	Bacteria	Firmicutes	Clostridia	Clostridiales	Lachnospiraceae	Lachnospiraceae_NK4A136_group
ASV_2347	Bacteria	Firmicutes	Clostridia	Clostridiales	Ruminococcaceae	Ruminococcaceae_UCG-014
ASV_2348	Bacteria	Firmicutes	Clostridia	Clostridiales	Lachnospiraceae	Unknown
ASV_2349	Bacteria	Bacteroidetes	Bacteroidia	Bacteroidales	Muribaculaceae	Unknown
ASV_2350	Bacteria	Bacteroidetes	Bacteroidia	Bacteroidales	Muribaculaceae	Unknown
ASV_2351	Bacteria	Firmicutes	Clostridia	Clostridiales	Unknown	
ASV_2352	Bacteria	Bacteroidetes	Bacteroidia	Bacteroidales	Rikenellaceae	Alistipes
ASV_2353	Bacteria	Bacteroidetes	Bacteroidia	Bacteroidales	Muribaculaceae	Unknown
ASV_2354	Bacteria	Firmicutes	Clostridia	Clostridiales	Lachnospiraceae	Lachnospiraceae_NK4A136_group
ASV_2355	Bacteria	Firmicutes	Clostridia	Clostridiales	Ruminococcaceae	Ruminiclostridium
ASV_2356	Bacteria	Bacteroidetes	Bacteroidia	Bacteroidales	Muribaculaceae	Unknown
ASV_2357	Bacteria	Bacteroidetes	Bacteroidia	Bacteroidales	Muribaculaceae	Unknown
ASV_2358	Bacteria	Tenericutes	Mollicutes	Mollicutes_RF39	Unknown	
ASV_2359	Bacteria	Bacteroidetes	Bacteroidia	Bacteroidales	Muribaculaceae	Unknown
ASV_2360	Bacteria	Actinobacteria	Coriobacteriia	Coriobacteriales	Eggerthellaceae	Denitrobacterium
ASV_2361	Bacteria	Firmicutes	Clostridia	Clostridiales	Ruminococcaceae	Ruminococcaceae_UCG-014
ASV_2362	Bacteria	Bacteroidetes	Bacteroidia	Bacteroidales	Muribaculaceae	Unknown
ASV_2363	Bacteria	Firmicutes	Clostridia	Clostridiales	Lachnospiraceae	Unknown
ASV_2364	Bacteria	Tenericutes	Mollicutes	Mollicutes_RF39	Unknown	
ASV_2365	Bacteria	Firmicutes	Clostridia	Clostridiales	Lachnospiraceae	Lachnospiraceae_UCG-001
ASV_2366	Bacteria	Firmicutes	Bacilli	Lactobacillales	Lactobacillaceae	Lactobacillus
ASV_2367	Bacteria			Unknown		
ASV_2368	Bacteria	Firmicutes	Clostridia	Clostridiales	Ruminococcaceae	Phocea
ASV_2369	Bacteria	Firmicutes	Clostridia	Clostridiales	Ruminococcaceae	Unknown
ASV_2370	Bacteria	Firmicutes	Clostridia	Clostridiales	Ruminococcaceae	Unknown
ASV_2371	Bacteria	Firmicutes	Clostridia	Clostridiales	Ruminococcaceae	Ruminiclostridium_9
ASV_2372	Bacteria	Firmicutes	Clostridia	Clostridiales	Lachnospiraceae	Unknown
ASV_2373	Bacteria	Firmicutes	Clostridia	Clostridiales	Lachnospiraceae	Unknown
ASV_2374	Bacteria	Firmicutes	Clostridia	Clostridiales	Lachnospiraceae	Unknown
ASV_2375	Bacteria	Firmicutes	Bacilli	Lactobacillales	Lactobacillaceae	Lactobacillus
ASV_2376	Bacteria	Firmicutes	Clostridia	Clostridiales	Ruminococcaceae	Oscillibacter
ASV_2377	Bacteria	Firmicutes	Bacilli	Lactobacillales	Lactobacillaceae	Lactobacillus
ASV_2378	Bacteria	Firmicutes	Clostridia	Clostridiales	Lachnospiraceae	Lachnospiraceae_NK4A136_group
ASV_2379	Bacteria	Firmicutes	Clostridia	Clostridiales	Ruminococcaceae	Unknown
ASV_2380	Bacteria	Firmicutes	Clostridia	Clostridiales	Lachnospiraceae	Unknown
ASV_2381	Bacteria	Firmicutes	Clostridia	Clostridiales	Unknown	

ASV_2382	Bacteria	Bacteroidetes	Bacteroidia	Bacteroidales	Muribaculaceae	CAG-873
ASV_2383	Bacteria	Firmicutes	Clostridia	Clostridiales	Ruminococcaceae	Ruminococcaceae_UCG-014
ASV_2384	Bacteria	Firmicutes	Clostridia	Clostridiales	Lachnospiraceae	Unknown
ASV_2385	Bacteria	Bacteroidetes	Bacteroidia		Unknown	
ASV_2386	Bacteria	Firmicutes	Clostridia	Clostridiales	Lachnospiraceae	Unknown
ASV_2387	Bacteria	Bacteroidetes	Bacteroidia	Bacteroidales	Muribaculaceae	Unknown
ASV_2388	Bacteria	Firmicutes	Bacilli	Lactobacillales	Lactobacillaceae	Lactobacillus
ASV_2389	Bacteria	Firmicutes	Bacilli	Lactobacillales	Lactobacillaceae	Lactobacillus
ASV_2390	Bacteria	Bacteroidetes	Bacteroidia		Unknown	
ASV_2391	Bacteria	Firmicutes	Clostridia	Clostridiales	Lachnospiraceae	Lachnospiraceae_NK4A136_group
ASV_2392	Bacteria	Deferribacteres	Deferribacteres	Deferribacterales	Deferribacteraceae	Mucispirillum
ASV_2393	Bacteria	Bacteroidetes	Bacteroidia	Bacteroidales	Muribaculaceae	Unknown
ASV_2394	Bacteria	Bacteroidetes	Bacteroidia	Bacteroidales	Muribaculaceae	Unknown
ASV_2395	Bacteria	Bacteroidetes	Bacteroidia	Bacteroidales	Muribaculaceae	Unknown
ASV_2396	Bacteria	Firmicutes	Clostridia	Clostridiales	Unknown	
ASV_2397	Bacteria	Firmicutes	Clostridia	Clostridiales	Unknown	
ASV_2398	Bacteria	Proteobacteria	Gammaproteobacteria	Betaproteobacteriales	Burkholderiaceae	Parasutterella
ASV_2399	Bacteria	Bacteroidetes	Bacteroidia	Bacteroidales	Muribaculaceae	Unknown
ASV_2400	Bacteria	Bacteroidetes	Bacteroidia		Unknown	
ASV_2401	Bacteria	Bacteroidetes	Bacteroidia	Bacteroidales	Muribaculaceae	Unknown
ASV_2402	Bacteria	Bacteroidetes	Bacteroidia	Bacteroidales	Rikenellaceae	Alistipes
ASV_2403	Bacteria	Bacteroidetes	Bacteroidia	Bacteroidales	Unknown	
ASV_2404	Bacteria	Firmicutes	Clostridia	Clostridiales	Ruminococcaceae	Ruminococcaceae_NK4A214_group
ASV_2405	Bacteria	Firmicutes	Clostridia	Clostridiales	Lachnospiraceae	Lachnoclostridium
ASV_2406	Bacteria	Firmicutes	Clostridia	Clostridiales	Ruminococcaceae	Ruminococcaceae_UCG-014
ASV_2407	Bacteria	Firmicutes	Clostridia	Clostridiales	Ruminococcaceae	Unknown
ASV_2408	Bacteria	Firmicutes	Clostridia	Clostridiales	Ruminococcaceae	Ruminiclostridium_9
ASV_2409	Bacteria	Firmicutes	Clostridia	Clostridiales	Lachnospiraceae	Unknown
ASV_2410	Bacteria	Firmicutes	Clostridia	Clostridiales	Ruminococcaceae	Ruminococcaceae_UCG-014
ASV_2411	Bacteria	Bacteroidetes	Bacteroidia		Unknown	
ASV_2412	Bacteria	Firmicutes	Clostridia	Clostridiales	Clostridiales_vadinBB60_group	Unknown
ASV_2413	Bacteria	Firmicutes	Clostridia	Clostridiales	Ruminococcaceae	Ruminiclostridium_9
ASV_2414	Bacteria	Firmicutes	Clostridia	Clostridiales	Lachnospiraceae	Unknown
ASV_2415	Bacteria	Firmicutes	Clostridia	Clostridiales	Lachnospiraceae	possible_genus_Sk018
ASV_2416	Bacteria	Bacteroidetes	Bacteroidia	Bacteroidales	Muribaculaceae	Unknown
ASV_2417	Bacteria	Bacteroidetes	Bacteroidia	Bacteroidales	Muribaculaceae	Unknown
ASV_2418	Bacteria	Firmicutes	Clostridia	Clostridiales	Lachnospiraceae	Unknown

ASV_2419	Bacteria	Firmicutes	Clostridia	Clostridiales	Ruminococcaceae	Unknown
ASV_2420	Bacteria	Firmicutes	Clostridia	Clostridiales	Clostridiales_vadinBB60_group	Unknown
ASV_2421	Bacteria	Firmicutes	Clostridia	Clostridiales	Ruminococcaceae	Ruminococcaceae_UCG-014
ASV_2422	Bacteria	Firmicutes	Bacilli	Lactobacillales	Lactobacillaceae	Lactobacillus
ASV_2423	Bacteria	Firmicutes	Clostridia	Clostridiales	Lachnospiraceae	Unknown
ASV_2424	Bacteria	Firmicutes	Clostridia	Clostridiales	Unknown	
ASV_2425	Bacteria	Firmicutes	Clostridia	Clostridiales	Ruminococcaceae	Ruminiclostridium_9
ASV_2426	Bacteria	Firmicutes	Clostridia	Clostridiales	Lachnospiraceae	Unknown
ASV_2427	Bacteria	Firmicutes	Clostridia	Clostridiales	Ruminococcaceae	Unknown
ASV_2428	Bacteria	Firmicutes	Clostridia	Clostridiales	Unknown	
ASV_2429	Bacteria	Firmicutes	Clostridia	Clostridiales	Ruminococcaceae	Anaerotruncus
ASV_2430	Bacteria	Bacteroidetes	Bacteroidia	Bacteroidales	Rikenellaceae	Alistipes
ASV_2431	Bacteria	Unknown				
ASV_2432	Bacteria	Unknown				
ASV_2433	Bacteria	Firmicutes	Clostridia	Clostridiales	Family_XIII	Family_XIII_AD3011_group
ASV_2434	Bacteria	Firmicutes	Clostridia	Clostridiales	Ruminococcaceae	UBA1819
ASV_2435	Bacteria	Firmicutes	Clostridia	Clostridiales	Ruminococcaceae	Ruminiclostridium_9
ASV_2436	Bacteria	Firmicutes	Clostridia	Clostridiales	Lachnospiraceae	Lachnospiraceae_NK4A136_group
ASV_2437	Bacteria	Bacteroidetes	Bacteroidia	Bacteroidales	Rikenellaceae	Alistipes
ASV_2438	Bacteria	Firmicutes	Clostridia	Clostridiales	Ruminococcaceae	Ruminococcus_1
ASV_2439	Bacteria	Firmicutes	Clostridia	Clostridiales	Lachnospiraceae	Unknown
ASV_2440	Bacteria	Firmicutes	Clostridia	Clostridiales	Lachnospiraceae	Lachnospiraceae_NK4A136_group
ASV_2441	Bacteria	Bacteroidetes	Bacteroidia	Bacteroidales	Bacteroidaceae	Bacteroides
ASV_2442	Bacteria	Bacteroidetes	Bacteroidia	Bacteroidales	Muribaculaceae	Unknown
ASV_2443	Bacteria	Firmicutes	Clostridia	Clostridiales	Ruminococcaceae	Oscillibacter
ASV_2444	Bacteria	Firmicutes	Bacilli	Lactobacillales	Lactobacillaceae	Lactobacillus
ASV_2445	Bacteria	Firmicutes	Clostridia	Clostridiales	Ruminococcaceae	Anaerotruncus
ASV_2446	Bacteria	Tenericutes	Mollicutes	Mollicutes_RF39	Unknown	
ASV_2447	Bacteria	Firmicutes	Clostridia	Clostridiales	Lachnospiraceae	Unknown
ASV_2448	Bacteria	Proteobacteria	Alphaproteobacteria	Unknown		
ASV_2449	Bacteria	Firmicutes	Erysipelotrichia	Erysipelotrichales	Erysipelotrichaceae	Unknown
ASV_2450	Bacteria	Firmicutes	Clostridia	Clostridiales	Lachnospiraceae	Unknown
ASV_2451	Bacteria	Firmicutes	Clostridia	Clostridiales	Clostridiales_vadinBB60_group	Unknown
ASV_2452	Bacteria	Firmicutes	Erysipelotrichia	Erysipelotrichales	Erysipelotrichaceae	Unknown
ASV_2453	Bacteria	Bacteroidetes	Bacteroidia	Bacteroidales	Rikenellaceae	Alistipes
ASV_2454	Bacteria	Firmicutes	Clostridia	Clostridiales	Clostridiales_vadinBB60_group	Unknown
ASV_2455	Bacteria	Firmicutes	Clostridia	Clostridiales	Lachnospiraceae	Unknown

ASV_2456	Bacteria	Bacteroidetes	Bacteroidia	Bacteroidales		Unknown
ASV_2457	Bacteria	Firmicutes	Clostridia	Clostridiales	Lachnospiraceae	Unknown
ASV_2458	Bacteria	Firmicutes	Clostridia	Clostridiales	Ruminococcaceae	Oscillibacter
ASV_2459	Bacteria	Bacteroidetes	Bacteroidia	Bacteroidales	Rikenellaceae	Alistipes
ASV_2460	Bacteria	Firmicutes	Clostridia	Clostridiales	Lachnospiraceae	Unknown
ASV_2461	Bacteria	Firmicutes	Clostridia			Unknown
ASV_2462	Bacteria	Firmicutes	Clostridia	Clostridiales	Lachnospiraceae	Lachnospiraceae_NK4A136_group
ASV_2463	Bacteria					Unknown
ASV_2464	Bacteria	Bacteroidetes	Bacteroidia	Bacteroidales		Unknown
ASV_2465	Bacteria	Bacteroidetes	Bacteroidia	Bacteroidales		Unknown
ASV_2466	Bacteria	Tenericutes	Mollicutes	Mollicutes_RF39		Unknown
ASV_2467	Bacteria	Proteobacteria	Deltaproteobacteria	Desulfovibrionales	Desulfovibrionaceae	Desulfovibrio
ASV_2468	Bacteria	Firmicutes	Clostridia	Clostridiales	Lachnospiraceae	Lachnoclostridium
ASV_2469	Bacteria	Firmicutes	Clostridia	Clostridiales	Lachnospiraceae	Unknown
ASV_2470	Bacteria	Firmicutes	Clostridia	Clostridiales	Ruminococcaceae	Unknown
ASV_2471	Bacteria	Tenericutes	Mollicutes	Mollicutes_RF39		Unknown
ASV_2472	Bacteria	Firmicutes				Unknown
ASV_2473	Bacteria	Firmicutes	Clostridia	Clostridiales	Lachnospiraceae	Unknown
ASV_2474	Bacteria	Firmicutes	Clostridia	Clostridiales	Ruminococcaceae	Ruminococcaceae_NK4A214_group
ASV_2475	Bacteria	Bacteroidetes	Bacteroidia	Bacteroidales	Muribaculaceae	Unknown
ASV_2476	Bacteria	Proteobacteria	Gammaproteobacteria	Betaproteobacteriales	Burkholderiaceae	Massilia
ASV_2477	Bacteria	Bacteroidetes	Bacteroidia	Bacteroidales	Muribaculaceae	Unknown
ASV_2478	Bacteria	Firmicutes	Clostridia	Clostridiales		Unknown
ASV_2479	Bacteria	Firmicutes	Clostridia	Clostridiales	Clostridiaceae_1	Candidatus_Arthromitus
ASV_2480	Bacteria	Bacteroidetes	Bacteroidia	Bacteroidales	Rikenellaceae	Alistipes
ASV_2481	Bacteria	Firmicutes	Clostridia	Clostridiales	Lachnospiraceae	Lachnospiraceae_NK4A136_group
ASV_2482	Bacteria	Bacteroidetes	Bacteroidia	Bacteroidales	Muribaculaceae	Unknown
ASV_2483	Bacteria	Firmicutes	Clostridia	Clostridiales	Lachnospiraceae	Lachnospiraceae_NK4A136_group
ASV_2484	Bacteria	Bacteroidetes	Bacteroidia	Bacteroidales	Rikenellaceae	Alistipes
ASV_2485	Bacteria	Firmicutes	Clostridia	Clostridiales	Lachnospiraceae	Lachnospiraceae_NK4A136_group
ASV_2486	Bacteria	Firmicutes	Clostridia	Clostridiales	Lachnospiraceae	Lachnospiraceae_NK4A136_group
ASV_2487	Bacteria	Proteobacteria	Deltaproteobacteria	Desulfovibrionales	Desulfovibrionaceae	Bilophila
ASV_2488	Bacteria	Actinobacteria	Coriobacteriia	Coriobacteriales	Eggerthellaceae	Unknown
ASV_2489	Bacteria	Firmicutes	Erysipelotrichia	Erysipelotrichales	Erysipelotrichaceae	Erysipelatoclostridium
ASV_2490	Bacteria	Bacteroidetes	Bacteroidia	Bacteroidales	Prevotellaceae	Prevotellaceae_NK3B31_group
ASV_2491	Bacteria	Firmicutes	Clostridia	Clostridiales	Ruminococcaceae	Unknown
ASV_2492	Bacteria	Actinobacteria	Coriobacteriia	Coriobacteriales	Eggerthellaceae	Senegalimassilia

ASV_2493	Bacteria	Bacteroidetes	Bacteroidia	Bacteroidales	Muribaculaceae	Unknown
ASV_2494	Bacteria	Firmicutes	Clostridia	Clostridiales	Lachnospiraceae	Unknown
ASV_2495	Bacteria	Firmicutes	Clostridia	Clostridiales	Ruminococcaceae	Unknown
ASV_2496	Bacteria	Actinobacteria	Coriobacteriia	Coriobacteriales	Eggerthellaceae	Denitrobacterium
ASV_2497	Bacteria	Bacteroidetes	Bacteroidia	Bacteroidales	Muribaculaceae	Unknown
ASV_2498	Bacteria					Unknown
ASV_2499	Bacteria	Bacteroidetes	Bacteroidia			Unknown
ASV_2500	Bacteria	Bacteroidetes	Bacteroidia	Bacteroidales	Bacteroidaceae	Bacteroides
ASV_2501	Bacteria	Bacteroidetes	Bacteroidia	Bacteroidales	Prevotellaceae	Prevotellaceae_UCG-001
ASV_2502	Bacteria	Firmicutes	Clostridia	Clostridiales	Ruminococcaceae	Ruminiclostridium
ASV_2503	Bacteria	Bacteroidetes	Bacteroidia	Bacteroidales	Muribaculaceae	CAG-873
ASV_2504	Bacteria	Firmicutes	Clostridia	Clostridiales	Lachnospiraceae	Lachnospiraceae_NK4A136_group
ASV_2505	Bacteria	Firmicutes	Clostridia	Clostridiales	Lachnospiraceae	Unknown
ASV_2506	Bacteria	Firmicutes	Erysipelotrichia	Erysipelotrichales	Erysipelotrichaceae	Faecalibaculum
ASV_2507	Bacteria	Firmicutes	Bacilli	Lactobacillales	Lactobacillaceae	Lactobacillus
ASV_2508	Bacteria	Firmicutes	Clostridia	Clostridiales		Unknown
ASV_2509	Bacteria	Firmicutes	Clostridia	Clostridiales	Lachnospiraceae	Lachnospiraceae_NK4A136_group
ASV_2510	Bacteria	Bacteroidetes	Bacteroidia	Bacteroidales	Muribaculaceae	Unknown
ASV_2511	Bacteria	Bacteroidetes	Bacteroidia	Bacteroidales	Muribaculaceae	Unknown
ASV_2512	Bacteria	Firmicutes	Clostridia	Clostridiales	Lachnospiraceae	Unknown
ASV_2513	Bacteria	Firmicutes	Clostridia	Clostridiales	Ruminococcaceae	Unknown
ASV_2514	Bacteria	Bacteroidetes	Bacteroidia	Bacteroidales		Unknown
ASV_2515	Bacteria	Bacteroidetes	Bacteroidia	Bacteroidales	Muribaculaceae	Muribaculum
ASV_2516	Bacteria	Tenericutes	Mollicutes	Mollicutes_RF39		Unknown
ASV_2517	Bacteria	Firmicutes	Clostridia	Clostridiales	Lachnospiraceae	Unknown
ASV_2518	Bacteria	Bacteroidetes	Bacteroidia	Bacteroidales	Prevotellaceae	Prevotellaceae_NK3B31_group
ASV_2519	Bacteria	Firmicutes	Bacilli	Lactobacillales	Lactobacillaceae	Lactobacillus
ASV_2520	Bacteria	Bacteroidetes	Bacteroidia	Bacteroidales	Muribaculaceae	Unknown
ASV_2521	Bacteria	Firmicutes	Clostridia	Clostridiales	Lachnospiraceae	ASF356
ASV_2522	Bacteria	Firmicutes	Clostridia	Clostridiales	Ruminococcaceae	Ruminiclostridium_9
ASV_2523	Bacteria	Bacteroidetes	Bacteroidia	Bacteroidales		Unknown
ASV_2524	Bacteria	Firmicutes	Clostridia	Clostridiales	Ruminococcaceae	Phocea
ASV_2525	Bacteria	Firmicutes	Clostridia	Clostridiales	Lachnospiraceae	Unknown
ASV_2526	Bacteria	Firmicutes	Clostridia	Clostridiales		Unknown
ASV_2527	Bacteria	Firmicutes	Clostridia	Clostridiales	Lachnospiraceae	Unknown
ASV_2528	Bacteria	Bacteroidetes	Bacteroidia	Bacteroidales	Rikenellaceae	Alistipes
ASV_2529	Bacteria	Bacteroidetes	Bacteroidia	Bacteroidales	Muribaculaceae	Unknown

ASV_2530	Bacteria	Bacteroidetes	Bacteroidia	Bacteroidales	Muribaculaceae	Unknown	
ASV_2531	Bacteria	Firmicutes	Clostridia	Clostridiales	Ruminococcaceae	Ruminococcaceae_UCG-014	
ASV_2532	Bacteria	Firmicutes	Clostridia	Clostridiales	Lachnospiraceae	Unknown	
ASV_2533	Bacteria	Bacteroidetes	Bacteroidia	Bacteroidales	Rikenellaceae	Alistipes	
ASV_2534	Bacteria	Firmicutes	Clostridia	Clostridiales	Lachnospiraceae	Lachnospiraceae_NK4A136_group	
ASV_2535	Bacteria	Firmicutes	Clostridia	Clostridiales	Lachnospiraceae	UC5-1-2E3	
ASV_2536	Bacteria	Bacteroidetes	Bacteroidia	Bacteroidales	Muribaculaceae	Unknown	
ASV_2537	Bacteria	Firmicutes	Bacilli	Lactobacillales	Lactobacillaceae	Lactobacillus	
ASV_2538	Bacteria	Firmicutes	Clostridia	Clostridiales	Lachnospiraceae	Lachnospiraceae_NK4A136_group	
ASV_2539	Bacteria	Firmicutes	Bacilli	Lactobacillales	Enterococcaceae	Enterococcus	Enterococcus_faecalis
ASV_2540	Bacteria	Firmicutes	Clostridia	Clostridiales	Ruminococcaceae	Unknown	
ASV_2541	Bacteria	Bacteroidetes	Bacteroidia	Bacteroidales	Rikenellaceae	Alistipes	
ASV_2542	Bacteria	Firmicutes	Clostridia	Clostridiales	Ruminococcaceae	Oscillibacter	
ASV_2543	Bacteria	Firmicutes	Clostridia	Clostridiales	Lachnospiraceae	Lachnospiraceae_NK4A136_group	
ASV_2544	Bacteria	Firmicutes	Clostridia	Clostridiales	Lachnospiraceae	Roseburia	
ASV_2545	Bacteria	Bacteroidetes	Bacteroidia	Bacteroidales	Muribaculaceae	Unknown	
ASV_2546	Bacteria	Firmicutes	Clostridia	Clostridiales	Ruminococcaceae	Unknown	
ASV_2547	Bacteria	Firmicutes	Bacilli	Lactobacillales	Lactobacillaceae	Lactobacillus	
ASV_2548	Bacteria	Firmicutes	Clostridia	Clostridiales	Lachnospiraceae	Unknown	
ASV_2549	Bacteria	Firmicutes	Clostridia	Clostridiales	Ruminococcaceae	Ruminococcaceae_UCG-002	
ASV_2550	Bacteria	Bacteroidetes	Bacteroidia	Bacteroidales	Muribaculaceae	Unknown	
ASV_2551	Bacteria	Bacteroidetes	Bacteroidia	Bacteroidales	Muribaculaceae	Unknown	
ASV_2552	Bacteria	Firmicutes	Clostridia	Clostridiales	Lachnospiraceae	Lachnospiraceae_NK4A136_group	
ASV_2553	Bacteria	Firmicutes	Clostridia	Clostridiales	Ruminococcaceae	Ruminiclostridium	
ASV_2554	Bacteria	Bacteroidetes	Bacteroidia	Bacteroidales	Bacteroidaceae	Bacteroides	
ASV_2555	Bacteria	Firmicutes	Clostridia	Clostridiales	Lachnospiraceae	A2	
ASV_2556	Bacteria	Firmicutes	Clostridia	Clostridiales	Family_XIII	Unknown	
ASV_2557	Bacteria	Bacteroidetes	Bacteroidia	Bacteroidales		Unknown	
ASV_2558	Bacteria	Firmicutes	Clostridia	Clostridiales	Lachnospiraceae	UC5-1-2E3	
ASV_2559	Bacteria	Firmicutes	Clostridia	Clostridiales	Lachnospiraceae	Lachnospiraceae_NK4A136_group	
ASV_2560	Bacteria	Firmicutes	Clostridia	Clostridiales	Lachnospiraceae	Unknown	
ASV_2561	Bacteria	Firmicutes	Clostridia	Clostridiales	Lachnospiraceae	Unknown	
ASV_2562	Bacteria	Bacteroidetes	Bacteroidia	Bacteroidales	Muribaculaceae	Unknown	
ASV_2563	Bacteria	Firmicutes	Clostridia	Clostridiales	Ruminococcaceae	Unknown	
ASV_2564	Bacteria	Firmicutes	Clostridia	Clostridiales	Ruminococcaceae	Oscillibacter	
ASV_2565	Bacteria	Proteobacteria	Gammaproteobacteria	Pasteurellales	Pasteurellaceae	Haemophilus	
ASV_2566	Bacteria	Firmicutes	Clostridia	Clostridiales	Lachnospiraceae	Unknown	

ASV_2567	Bacteria	Firmicutes	Clostridia	Clostridiales	Unknown	
ASV_2568	Bacteria	Unknown				
ASV_2569	Bacteria	Bacteroidetes	Bacteroidia	Bacteroidales	Unknown	
ASV_2570	Bacteria	Firmicutes	Clostridia	Clostridiales	Ruminococcaceae	Ruminococcaceae_UCG-014
ASV_2571	Bacteria	Firmicutes	Clostridia	Clostridiales	Ruminococcaceae	Unknown
ASV_2572	Bacteria	Firmicutes	Clostridia	Clostridiales	Ruminococcaceae	Ruminococcaceae_UCG-014
ASV_2573	Bacteria	Bacteroidetes	Bacteroidia	Bacteroidales	Muribaculaceae	Unknown
ASV_2574	Bacteria	Firmicutes	Clostridia	Clostridiales	Lachnospiraceae	UC5-1-2E3
ASV_2575	Bacteria	Firmicutes	Clostridia	Clostridiales	Unknown	
ASV_2576	Bacteria	Firmicutes	Clostridia	Clostridiales	Lachnospiraceae	Unknown
ASV_2577	Bacteria	Firmicutes	Clostridia	Clostridiales	Lachnospiraceae	Unknown
ASV_2578	Bacteria	Firmicutes	Clostridia	Clostridiales	Ruminococcaceae	GCA-900066225
ASV_2579	Bacteria	Bacteroidetes	Bacteroidia	Bacteroidales	Muribaculaceae	Unknown
ASV_2580	Bacteria	Firmicutes	Clostridia	Clostridiales	Ruminococcaceae	Ruminococcaceae_UCG-014
ASV_2581	Bacteria	Firmicutes	Clostridia	Clostridiales	Lachnospiraceae	Unknown
ASV_2582	Bacteria	Firmicutes	Clostridia	Clostridiales	Lachnospiraceae	Lachnospiraceae_NK4A136_group
ASV_2583	Bacteria	Bacteroidetes	Bacteroidia	Bacteroidales	Muribaculaceae	Unknown
ASV_2584	Bacteria	Firmicutes	Clostridia	Clostridiales	Ruminococcaceae	Oscillibacter
ASV_2585	Bacteria	Firmicutes	Bacilli	Lactobacillales	Lactobacillaceae	Lactobacillus
ASV_2586	Bacteria	Firmicutes	Clostridia	Clostridiales	Family_XIII	Unknown
ASV_2587	Bacteria	Firmicutes	Clostridia	Clostridiales	Ruminococcaceae	Ruminococcaceae_UCG-014
ASV_2588	Bacteria	Firmicutes	Erysipelotrichia	Erysipelotrichales	Erysipelotrichaceae	Unknown
ASV_2589	Bacteria	Firmicutes	Clostridia	Clostridiales	Ruminococcaceae	Ruminococcus_1
ASV_2590	Bacteria	Bacteroidetes	Bacteroidia	Bacteroidales	Muribaculaceae	Unknown
ASV_2591	Bacteria	Unknown				
ASV_2592	Bacteria	Firmicutes	Clostridia	Clostridiales	Lachnospiraceae	Unknown
ASV_2593	Bacteria	Firmicutes	Clostridia	Clostridiales	Ruminococcaceae	Unknown
ASV_2594	Bacteria	Bacteroidetes	Bacteroidia	Bacteroidales	Muribaculaceae	Unknown
ASV_2595	Bacteria	Deferribacteres	Deferribacteres	Deferribacterales	Deferribacteraceae	Mucispirillum
ASV_2596	Bacteria	Bacteroidetes	Bacteroidia	Bacteroidales	Muribaculaceae	Unknown
ASV_2597	Bacteria	Firmicutes	Clostridia	Clostridiales	Ruminococcaceae	Ruminiclostridium
ASV_2598	Bacteria	Firmicutes	Clostridia	Clostridiales	Ruminococcaceae	Unknown
ASV_2599	Bacteria	Firmicutes	Clostridia	Clostridiales	Ruminococcaceae	Anaerotruncus
ASV_2600	Bacteria	Bacteroidetes	Bacteroidia	Bacteroidales	Muribaculaceae	Unknown
ASV_2601	Bacteria	Actinobacteria	Actinobacteria	Micrococcales	Micrococcaceae	Rothia
ASV_2602	Bacteria	Firmicutes	Clostridia	Clostridiales	Lachnospiraceae	Unknown
ASV_2603	Bacteria	Bacteroidetes	Bacteroidia	Bacteroidales	Muribaculaceae	Unknown

ASV_2604	Bacteria	Firmicutes	Clostridia	Clostridiales	Lachnospiraceae	Lactonifactor
ASV_2605	Bacteria	Firmicutes	Bacilli	Lactobacillales	Lactobacillaceae	Lactobacillus
ASV_2606	Bacteria	Firmicutes	Clostridia	Clostridiales	Lachnospiraceae	Unknown
ASV_2607	Bacteria	Firmicutes	Clostridia	Clostridiales	Ruminococcaceae	Ruminococcaceae_UCG-014
ASV_2608	Bacteria	Firmicutes	Clostridia	Clostridiales	Lachnospiraceae	Marvinbryantia
ASV_2609	Bacteria	Firmicutes	Clostridia	Clostridiales	Lachnospiraceae	Lachnospiraceae_NK4A136_group
ASV_2610	Bacteria	Firmicutes	Clostridia	Clostridiales	Lachnospiraceae	Unknown
ASV_2611	Bacteria	Bacteroidetes	Bacteroidia	Bacteroidales	Muribaculaceae	Unknown
ASV_2612	Bacteria	Firmicutes	Clostridia	Clostridiales	Lachnospiraceae	Lachnospiraceae_ND3007_group
ASV_2613	Bacteria	Firmicutes	Clostridia	Clostridiales	Ruminococcaceae	Ruminococcaceae_UCG-014
ASV_2614	Bacteria	Proteobacteria	Gammaproteobacteria	Betaproteobacteriales	Burkholderiaceae	Parasutterella
ASV_2615	Bacteria	Bacteroidetes	Bacteroidia	Bacteroidales	Prevotellaceae	Prevotellaceae_UCG-001
ASV_2616	Bacteria	Firmicutes	Clostridia	Clostridiales	Ruminococcaceae	Subdoligranulum
ASV_2617	Bacteria	Bacteroidetes	Bacteroidia	Bacteroidales	Prevotellaceae	Prevotellaceae_UCG-001
ASV_2618	Bacteria	Bacteroidetes	Bacteroidia	Bacteroidales	Muribaculaceae	Unknown
ASV_2619	Bacteria	Firmicutes	Clostridia	Clostridiales	Ruminococcaceae	Ruminococcus_2
ASV_2620	Bacteria	Firmicutes	Clostridia	Clostridiales	Ruminococcaceae	Ruminococcaceae_UCG-014
ASV_2621	Bacteria	Firmicutes	Erysipelotrichia	Erysipelotrichales	Erysipelotrichaceae	Faecalibaculum
ASV_2622	Bacteria	Firmicutes	Clostridia	Clostridiales	Lachnospiraceae	GCA-900066575
ASV_2623	Bacteria	Firmicutes	Clostridia	Clostridiales	Lachnospiraceae	A2
ASV_2624	Bacteria	Bacteroidetes	Bacteroidia	Bacteroidales	Muribaculaceae	Muribaculum
ASV_2625	Bacteria	Firmicutes	Clostridia	Clostridiales	Lachnospiraceae	Unknown
ASV_2626	Bacteria	Bacteroidetes	Bacteroidia	Bacteroidales		Unknown
ASV_2627	Bacteria	Firmicutes	Bacilli	Lactobacillales	Unknown	
ASV_2628	Bacteria	Bacteroidetes	Bacteroidia	Bacteroidales	Muribaculaceae	Unknown
ASV_2629	Bacteria	Firmicutes	Clostridia	Clostridiales	Lachnospiraceae	Unknown
ASV_2630	Bacteria	Bacteroidetes	Bacteroidia	Bacteroidales	Muribaculaceae	Unknown
ASV_2631	Bacteria	Firmicutes	Clostridia	Clostridiales		Unknown
ASV_2632	Bacteria	Firmicutes	Clostridia	Clostridiales	Ruminococcaceae	Ruminococcaceae_UCG-014
ASV_2633	Bacteria	Bacteroidetes	Bacteroidia	Bacteroidales	Muribaculaceae	Unknown
ASV_2634	Bacteria	Bacteroidetes	Bacteroidia	Bacteroidales	Muribaculaceae	Unknown
ASV_2635	Bacteria	Firmicutes	Clostridia	Clostridiales		Unknown
ASV_2636	Bacteria	Firmicutes	Erysipelotrichia	Erysipelotrichales	Erysipelotrichaceae	Unknown
ASV_2637	Bacteria	Cyanobacteria	Melainabacteria	Gastranaerophilales		Unknown
ASV_2638	Bacteria	Bacteroidetes	Bacteroidia	Bacteroidales	Muribaculaceae	Unknown
ASV_2639	Bacteria	Firmicutes	Clostridia	Clostridiales	Ruminococcaceae	Ruminococcaceae_UCG-014
ASV_2640	Bacteria	Firmicutes	Clostridia	Clostridiales	Lachnospiraceae	Lachnospiraceae_NK4A136_group

ASV_2641	Bacteria	Firmicutes	Clostridia	Clostridiales	Lachnospiraceae	Lachnoclostridium
ASV_2642	Bacteria	Firmicutes	Clostridia	Clostridiales	Ruminococcaceae	Ruminococcaceae_UCG-014
ASV_2643	Bacteria	Bacteroidetes	Bacteroidia	Bacteroidales	Muribaculaceae	Unknown
ASV_2644	Bacteria	Firmicutes	Clostridia	Clostridiales	Lachnospiraceae	Lachnospiraceae_NK4A136_group
ASV_2645	Bacteria	Firmicutes	Clostridia	Clostridiales	Lachnospiraceae	Unknown
ASV_2646	Bacteria	Bacteroidetes	Bacteroidia	Bacteroidales	Muribaculaceae	Unknown
ASV_2647	Bacteria	Bacteroidetes	Bacteroidia	Bacteroidales	Muribaculaceae	Muribaculum
ASV_2648	Bacteria	Bacteroidetes	Bacteroidia		Unknown	
ASV_2649	Bacteria	Firmicutes	Clostridia	Clostridiales	Ruminococcaceae	Ruminococcaceae_UCG-014
ASV_2650	Bacteria	Firmicutes	Clostridia	Clostridiales	Ruminococcaceae	Unknown
ASV_2651	Bacteria	Proteobacteria	Gammaproteobacteria	Pseudomonadales	Moraxellaceae	Acinetobacter
ASV_2652	Bacteria	Bacteroidetes	Bacteroidia	Bacteroidales	Rikenellaceae	Alistipes
ASV_2653	Bacteria	Firmicutes	Clostridia	Clostridiales	Ruminococcaceae	Ruminococcaceae_UCG-010
ASV_2654	Bacteria	Bacteroidetes	Bacteroidia	Bacteroidales	Bacteroidaceae	Bacteroides
ASV_2655	Bacteria	Bacteroidetes	Bacteroidia	Bacteroidales	Muribaculaceae	Unknown
ASV_2656	Bacteria	Firmicutes	Erysipelotrichia	Erysipelotrichales	Erysipelotrichaceae	Erysipelatoclostridium
ASV_2657	Bacteria	Bacteroidetes	Bacteroidia	Bacteroidales	Rikenellaceae	Alistipes
ASV_2658	Bacteria	Firmicutes	Clostridia	Clostridiales	Ruminococcaceae	Unknown
ASV_2659	Bacteria	Firmicutes	Clostridia	Clostridiales	Ruminococcaceae	Ruminococcaceae_UCG-014
ASV_2660	Bacteria	Firmicutes	Clostridia	Clostridiales	Lachnospiraceae	Lachnospiraceae_NK4A136_group
ASV_2661	Bacteria	Firmicutes	Clostridia	Clostridiales	Ruminococcaceae	Ruminiclostridium_9
ASV_2662	Bacteria	Firmicutes	Clostridia	Clostridiales	Ruminococcaceae	Unknown
ASV_2663	Bacteria	Firmicutes	Clostridia	Clostridiales	Lachnospiraceae	Lachnospiraceae_NK4A136_group
ASV_2664	Bacteria	Bacteroidetes	Bacteroidia	Bacteroidales	Muribaculaceae	Unknown
ASV_2665	Bacteria	Firmicutes	Clostridia	Clostridiales	Ruminococcaceae	Unknown
ASV_2666	Bacteria	Firmicutes	Clostridia	Clostridiales	Ruminococcaceae	Ruminococcaceae_UCG-014
ASV_2667	Bacteria	Firmicutes	Clostridia	Clostridiales	Ruminococcaceae	Ruminiclostridium_5
ASV_2668	Bacteria	Firmicutes	Clostridia	Clostridiales	Ruminococcaceae	Unknown
ASV_2669	Bacteria	Bacteroidetes	Bacteroidia	Bacteroidales	Muribaculaceae	Unknown
ASV_2670	Bacteria	Bacteroidetes	Bacteroidia	Bacteroidales	Muribaculaceae	Unknown
ASV_2671	Bacteria	Firmicutes	Clostridia	Clostridiales	Ruminococcaceae	Ruminococcaceae_UCG-014
ASV_2672	Bacteria	Firmicutes	Clostridia	Clostridiales	Lachnospiraceae	Lachnospiraceae_NK4A136_group
ASV_2673	Bacteria	Firmicutes	Clostridia	Clostridiales	Lachnospiraceae	Unknown
ASV_2674	Bacteria			Unknown		
ASV_2675	Bacteria	Verrucomicrobia	Verrucomicrobiae	Verrucomicrobiales	Akkermansiaceae	Akkermansia
ASV_2676	Bacteria	Bacteroidetes	Bacteroidia	Bacteroidales	Muribaculaceae	Unknown
ASV_2677	Bacteria	Firmicutes	Clostridia	Clostridiales	Ruminococcaceae	Unknown

ASV_2678	Bacteria	Firmicutes	Clostridia	Clostridiales	Ruminococcaceae	Ruminococcaceae_UCG-014
ASV_2679	Bacteria	Firmicutes	Clostridia	Clostridiales	Ruminococcaceae	Harryflintia
ASV_2680	Bacteria	Firmicutes	Bacilli	Lactobacillales	Lactobacillaceae	Lactobacillus
ASV_2681	Bacteria	Firmicutes	Clostridia	Clostridiales	Lachnospiraceae	Unknown
ASV_2682	Bacteria	Bacteroidetes	Bacteroidia	Bacteroidales	Rikenellaceae	Alistipes
ASV_2683	Bacteria	Bacteroidetes	Bacteroidia	Bacteroidales		Unknown
ASV_2684	Bacteria	Bacteroidetes	Bacteroidia	Bacteroidales	Muribaculaceae	Unknown
ASV_2685	Bacteria	Firmicutes	Clostridia	Clostridiales	Lachnospiraceae	Unknown
ASV_2686	Bacteria	Bacteroidetes	Bacteroidia	Bacteroidales	Prevotellaceae	Prevotellaceae_UCG-001
ASV_2687	Bacteria	Bacteroidetes	Bacteroidia	Bacteroidales	Prevotellaceae	Prevotellaceae_UCG-001
ASV_2688	Bacteria	Bacteroidetes	Bacteroidia	Bacteroidales	Muribaculaceae	Unknown
ASV_2689	Bacteria	Firmicutes	Clostridia	Clostridiales	Peptococcaceae	Unknown
ASV_2690	Bacteria	Bacteroidetes	Bacteroidia	Bacteroidales	Muribaculaceae	Unknown
ASV_2691	Bacteria	Firmicutes	Clostridia	Clostridiales	Clostridiales_vadinBB60_group	Unknown
ASV_2692	Bacteria	Firmicutes	Clostridia	Clostridiales	Clostridiales_vadinBB60_group	Unknown
ASV_2693	Bacteria	Firmicutes	Clostridia	Clostridiales	Ruminococcaceae	Ruminiclostridium_9
ASV_2694	Bacteria	Firmicutes	Clostridia	Clostridiales	Lachnospiraceae	Unknown
ASV_2695	Bacteria	Bacteroidetes	Bacteroidia	Bacteroidales	Muribaculaceae	Unknown
ASV_2696	Bacteria	Firmicutes	Clostridia	Clostridiales	Ruminococcaceae	Ruminococcaceae_UCG-014
ASV_2697	Bacteria	Firmicutes	Clostridia	Clostridiales	Lachnospiraceae	Unknown
ASV_2698	Bacteria	Firmicutes	Clostridia	Clostridiales	Ruminococcaceae	Ruminiclostridium
ASV_2699	Bacteria	Bacteroidetes	Bacteroidia	Bacteroidales		Unknown
ASV_2700	Bacteria	Firmicutes	Clostridia	Clostridiales	Clostridiales_vadinBB60_group	Unknown
ASV_2701	Bacteria	Firmicutes	Clostridia	Clostridiales	Clostridiales_vadinBB60_group	Unknown
ASV_2702	Bacteria	Bacteroidetes	Bacteroidia	Bacteroidales	Prevotellaceae	Prevotellaceae_NK3B31_group
ASV_2703	Bacteria	Firmicutes	Clostridia	Clostridiales	Lachnospiraceae	Lachnospiraceae_NK4A136_group
ASV_2704	Bacteria	Firmicutes	Clostridia	Clostridiales	Ruminococcaceae	Ruminococcaceae_UCG-014
ASV_2705	Bacteria	Firmicutes	Clostridia	Clostridiales	Ruminococcaceae	Ruminococcaceae_UCG-014
ASV_2706	Bacteria	Firmicutes	Clostridia	Clostridiales	Lachnospiraceae	Unknown
ASV_2707	Bacteria	Firmicutes	Clostridia	Clostridiales	Peptococcaceae	Unknown
ASV_2708	Bacteria	Firmicutes	Clostridia	Clostridiales	Ruminococcaceae	Ruminiclostridium_9
ASV_2709	Bacteria	Firmicutes	Bacilli	Lactobacillales	Lactobacillaceae	Lactobacillus
ASV_2710	Bacteria	Firmicutes	Bacilli	Lactobacillales	Lactobacillaceae	Lactobacillus
ASV_2711	Bacteria	Bacteroidetes	Bacteroidia	Bacteroidales	Rikenellaceae	Alistipes
ASV_2712	Bacteria	Firmicutes	Clostridia	Clostridiales	Lachnospiraceae	Unknown
ASV_2713	Bacteria	Bacteroidetes	Bacteroidia	Bacteroidales	Muribaculaceae	Unknown
ASV_2714	Bacteria	Firmicutes	Clostridia	Clostridiales	Lachnospiraceae	Lachnospiraceae_UCG-006

ASV_2715	Bacteria	Firmicutes	Clostridia	Clostridiales	Ruminococcaceae	Unknown
ASV_2716	Bacteria	Firmicutes	Clostridia	Clostridiales	Lachnospiraceae	Tyzzerella
ASV_2717	Bacteria	Bacteroidetes	Bacteroidia	Bacteroidales	Muribaculaceae	Unknown
ASV_2718	Bacteria	Unknown				
ASV_2719	Bacteria	Fusobacteria	Fusobacteriia	Fusobacteriales	Fusobacteriaceae	Fusobacterium
ASV_2720	Bacteria	Firmicutes	Clostridia	Clostridiales	Ruminococcaceae	Unknown
ASV_2721	Bacteria	Firmicutes	Clostridia	Clostridiales	Ruminococcaceae	Ruminiclostridium_9
ASV_2722	Bacteria	Bacteroidetes	Bacteroidia	Bacteroidales	Rikenellaceae	Alistipes
ASV_2723	Bacteria	Firmicutes	Clostridia	Clostridiales	Lachnospiraceae	Unknown
ASV_2724	Bacteria	Firmicutes	Clostridia	Clostridiales	Ruminococcaceae	Unknown
ASV_2725	Bacteria	Bacteroidetes	Bacteroidia	Bacteroidales	Muribaculaceae	Muribaculum
ASV_2726	Bacteria	Proteobacteria	Alphaproteobacteria	Unknown		
ASV_2727	Bacteria	Firmicutes	Bacilli	Lactobacillales	Lactobacillaceae	Lactobacillus
ASV_2728	Bacteria	Bacteroidetes	Bacteroidia	Bacteroidales	Bacteroidaceae	Bacteroides
ASV_2729	Bacteria	Firmicutes	Clostridia	Clostridiales	Lachnospiraceae	Marvinbryantia
ASV_2730	Bacteria	Bacteroidetes	Bacteroidia	Bacteroidales	Muribaculaceae	CAG-873
ASV_2731	Bacteria	Bacteroidetes	Bacteroidia	Bacteroidales	Unknown	
ASV_2732	Bacteria	Firmicutes	Clostridia	Clostridiales	Lachnospiraceae	Unknown
ASV_2733	Bacteria	Bacteroidetes	Bacteroidia	Bacteroidales	Muribaculaceae	Unknown
ASV_2734	Bacteria	Firmicutes	Unknown			
ASV_2735	Bacteria	Firmicutes	Clostridia	Clostridiales	Ruminococcaceae	Oscillibacter
ASV_2736	Bacteria	Unknown				
ASV_2737	Bacteria	Firmicutes	Clostridia	Clostridiales	Ruminococcaceae	Ruminococcaceae_NK4A214_group
ASV_2738	Bacteria	Firmicutes	Clostridia	Clostridiales	Ruminococcaceae	Ruminococcaceae_UCG-003
ASV_2739	Bacteria	Firmicutes	Clostridia	Clostridiales	Ruminococcaceae	Subdoligranulum
ASV_2740	Bacteria	Bacteroidetes	Bacteroidia	Bacteroidales	Muribaculaceae	Unknown
ASV_2741	Bacteria	Firmicutes	Clostridia	Clostridiales	Lachnospiraceae	Lachnospiraceae_NK4A136_group
ASV_2742	Bacteria	Firmicutes	Clostridia	Clostridiales	Lachnospiraceae	Lachnospiraceae_NK4A136_group
ASV_2743	Bacteria	Firmicutes	Clostridia	Clostridiales	Clostridiales_vadinBB60_group	Unknown
ASV_2744	Bacteria	Firmicutes	Clostridia	Clostridiales	Clostridiaceae_1	Unknown
ASV_2745	Bacteria	Firmicutes	Clostridia	Clostridiales	Unknown	
ASV_2746	Bacteria	Bacteroidetes	Bacteroidia	Bacteroidales	Rikenellaceae	Alistipes
ASV_2747	Bacteria	Verrucomicrobia	Verrucomicrobiae	Verrucomicrobiales	Akkermansiaceae	Akkermansia
ASV_2748	Bacteria	Bacteroidetes	Bacteroidia	Bacteroidales	Prevotellaceae	Prevotellaceae_UCG-001
ASV_2749	Bacteria	Firmicutes	Clostridia	Clostridiales	Lachnospiraceae	Lachnoclostridium
ASV_2750	Bacteria	Firmicutes	Clostridia	Clostridiales	Unknown	
ASV_2751	Bacteria	Unknown				

ASV_2752	Bacteria	Bacteroidetes	Bacteroidia	Bacteroidales	Prevotellaceae	Prevotellaceae_UCG-001
ASV_2753	Bacteria	Verrucomicrobia	Verrucomicrobiae	Verrucomicrobiales	Akkermansiaceae	Akkermansia
ASV_2754	Bacteria	Bacteroidetes	Bacteroidia	Bacteroidales	Muribaculaceae	Unknown
ASV_2755	Bacteria	Bacteroidetes	Bacteroidia		Unknown	
ASV_2756	Bacteria	Firmicutes	Clostridia	Clostridiales	Lachnospiraceae	Unknown
ASV_2757	Bacteria	Firmicutes	Clostridia	Clostridiales	Lachnospiraceae	Lachnospiraceae_NK4A136_group
ASV_2758	Bacteria	Bacteroidetes	Bacteroidia	Bacteroidales	Unknown	
ASV_2759	Bacteria	Bacteroidetes	Bacteroidia	Bacteroidales	Rikenellaceae	Alistipes
ASV_2760	Bacteria	Firmicutes	Clostridia	Clostridiales	Lachnospiraceae	Unknown
ASV_2761	Bacteria	Bacteroidetes	Bacteroidia	Bacteroidales	Rikenellaceae	Alistipes
ASV_2762	Bacteria	Bacteroidetes	Bacteroidia	Bacteroidales	Prevotellaceae	Prevotellaceae_NK3B31_group
ASV_2763	Bacteria	Firmicutes	Erysipelotrichia	Erysipelotrichales	Erysipelotrichaceae	Erysipelatoclostridium
ASV_2764	Bacteria	Firmicutes	Clostridia	Clostridiales	Lachnospiraceae	Unknown
ASV_2765	Bacteria	Bacteroidetes	Bacteroidia	Bacteroidales	Prevotellaceae	Prevotellaceae_UCG-001
ASV_2766	Bacteria	Bacteroidetes	Bacteroidia	Bacteroidales	Unknown	
ASV_2767	Bacteria	Bacteroidetes	Bacteroidia	Bacteroidales	Unknown	
ASV_2768	Bacteria	Firmicutes	Clostridia	Clostridiales	Ruminococcaceae	Unknown
ASV_2769	Bacteria	Firmicutes	Clostridia	Clostridiales	Unknown	
ASV_2770	Bacteria	Bacteroidetes	Bacteroidia	Bacteroidales	Rikenellaceae	Alistipes
ASV_2771	Bacteria	Bacteroidetes	Bacteroidia	Bacteroidales	Prevotellaceae	Prevotellaceae_NK3B31_group
ASV_2772	Bacteria	Firmicutes	Clostridia	Clostridiales	Lachnospiraceae	Unknown
ASV_2773	Bacteria	Tenericutes	Mollicutes	Mollicutes_RF39	Unknown	
ASV_2774	Bacteria	Firmicutes	Clostridia	Clostridiales	Lachnospiraceae	Unknown
ASV_2775	Bacteria	Firmicutes	Clostridia	Clostridiales	Ruminococcaceae	Ruminococcaceae_UCG-014
ASV_2776	Bacteria	Bacteroidetes	Bacteroidia	Bacteroidales	Prevotellaceae	Prevotellaceae_NK3B31_group
ASV_2777	Bacteria	Bacteroidetes	Bacteroidia	Bacteroidales	Prevotellaceae	Prevotellaceae_NK3B31_group
ASV_2778	Bacteria	Firmicutes	Clostridia	Clostridiales	Lachnospiraceae	Unknown
ASV_2779	Bacteria	Bacteroidetes	Bacteroidia	Bacteroidales	Rikenellaceae	Alistipes
ASV_2780	Bacteria	Firmicutes	Clostridia	Clostridiales	Lachnospiraceae	Unknown
ASV_2781	Bacteria	Firmicutes	Clostridia	Clostridiales	Lachnospiraceae	Unknown
ASV_2782	Bacteria	Bacteroidetes	Bacteroidia	Bacteroidales	Muribaculaceae	Unknown
ASV_2783	Bacteria	Firmicutes	Clostridia	Clostridiales	Lachnospiraceae	Unknown
ASV_2784	Bacteria	Firmicutes	Clostridia	Clostridiales	Lachnospiraceae	Lachnospiraceae_NK4A136_group
ASV_2785	Bacteria	Firmicutes	Clostridia	Clostridiales	Lachnospiraceae	Unknown
ASV_2786	Bacteria	Firmicutes	Clostridia	Clostridiales	Lachnospiraceae	Lachnospiraceae_NK4A136_group
ASV_2787	Bacteria	Firmicutes	Clostridia	Clostridiales	Clostridiales_vadinBB60_group	Unknown
ASV_2788	Bacteria	Bacteroidetes	Bacteroidia	Bacteroidales	Muribaculaceae	Unknown

ASV_2789	Bacteria	Firmicutes	Clostridia	Clostridiales	Ruminococcaceae	Ruminococcaceae_UCG-014	
ASV_2790	Bacteria	Firmicutes	Clostridia	Clostridiales	Lachnospiraceae	Unknown	
ASV_2791	Bacteria	Firmicutes	Clostridia	Clostridiales	Ruminococcaceae	Oscillibacter	
ASV_2792	Bacteria	Bacteroidetes	Bacteroidia	Bacteroidales	Rikenellaceae	Alistipes	
ASV_2793	Bacteria	Firmicutes	Bacilli	Lactobacillales	Lactobacillaceae	Lactobacillus	
ASV_2794	Bacteria	Firmicutes	Clostridia	Clostridiales	Ruminococcaceae	Oscillibacter	
ASV_2795	Bacteria	Firmicutes	Clostridia	Clostridiales	Lachnospiraceae	Unknown	
ASV_2796	Bacteria	Firmicutes	Clostridia	Clostridiales	Ruminococcaceae	Ruminococcaceae_UCG-014	
ASV_2797	Bacteria	Firmicutes	Clostridia	Clostridiales	Lachnospiraceae	Unknown	
ASV_2798	Bacteria	Bacteroidetes	Bacteroidia	Bacteroidales	Unknown		
ASV_2799	Bacteria	Bacteroidetes	Bacteroidia	Bacteroidales	Unknown		
ASV_2800	Bacteria	Firmicutes	Clostridia	Clostridiales	Ruminococcaceae	Unknown	
ASV_2801	Bacteria	Firmicutes	Clostridia	Clostridiales	Ruminococcaceae	Ruminococcaceae_NK4A214_group	
ASV_2802	Bacteria	Firmicutes	Bacilli	Lactobacillales	Unknown		
ASV_2803	Bacteria	Bacteroidetes	Bacteroidia	Bacteroidales	Muribaculaceae	Unknown	
ASV_2804	Bacteria	Firmicutes	Clostridia	Clostridiales	Lachnospiraceae	Unknown	
ASV_2805	Bacteria	Firmicutes	Clostridia	Clostridiales	Ruminococcaceae	Ruminococcaceae_UCG-014	
ASV_2806	Bacteria	Bacteroidetes	Bacteroidia	Bacteroidales	Unknown		
ASV_2807	Bacteria	Bacteroidetes	Bacteroidia	Bacteroidales	Muribaculaceae	Unknown	
ASV_2808	Bacteria	Firmicutes	Clostridia	Clostridiales	Lachnospiraceae	Unknown	
ASV_2809	Bacteria	Bacteroidetes	Bacteroidia	Bacteroidales	Muribaculaceae	Unknown	
ASV_2810	Bacteria	Firmicutes	Clostridia	Clostridiales	Ruminococcaceae	Ruminococcaceae_UCG-014	
ASV_2811	Bacteria	Firmicutes	Clostridia	Clostridiales	Lachnospiraceae	Unknown	
ASV_2812	Bacteria	Firmicutes	Clostridia	Clostridiales	Lachnospiraceae	Unknown	
ASV_2813	Bacteria	Firmicutes	Clostridia	Clostridiales	Lachnospiraceae	Unknown	
ASV_2814	Bacteria	Firmicutes	Clostridia	Clostridiales	Lachnospiraceae	Unknown	
ASV_2815	Bacteria	Firmicutes	Clostridia	Clostridiales	Unknown		
ASV_2816	Bacteria	Firmicutes	Clostridia	Clostridiales	Clostridiales_vadinBB60_group	Unknown	
ASV_2817	Bacteria	Actinobacteria	Actinobacteria	Micrococcales	Micrococcaceae	Rothia	Rothia_dentocariosa
ASV_2818	Bacteria	Firmicutes	Clostridia	Clostridiales	Lachnospiraceae	Lachnospiraceae_NK4A136_group	
ASV_2819	Bacteria	Bacteroidetes	Bacteroidia	Bacteroidales	Muribaculaceae	Unknown	
ASV_2820	Bacteria	Firmicutes	Clostridia	Clostridiales	Ruminococcaceae	Ruminococcaceae_UCG-014	
ASV_2821	Bacteria	Firmicutes	Bacilli	Lactobacillales	Carnobacteriaceae	Granulicatella	
ASV_2822	Bacteria	Firmicutes	Clostridia	Clostridiales	Unknown		
ASV_2823	Bacteria	Firmicutes	Clostridia	Clostridiales	Ruminococcaceae	Phocea	
ASV_2824	Bacteria	Firmicutes	Clostridia	Clostridiales	Ruminococcaceae	Unknown	
ASV_2825	Bacteria	Firmicutes	Clostridia	Clostridiales	Lachnospiraceae	UC5-1-2E3	

ASV_2826	Bacteria	Firmicutes	Clostridia	Clostridiales	Ruminococcaceae	Ruminiclostridium_9
ASV_2827	Bacteria	Firmicutes	Clostridia	Clostridiales	Ruminococcaceae	Unknown
ASV_2828	Bacteria	Firmicutes	Erysipelotrichia	Erysipelotrichales	Erysipelotrichaceae	Unknown
ASV_2829	Bacteria	Firmicutes	Clostridia	Clostridiales	Lachnospiraceae	Unknown
ASV_2830	Bacteria	Firmicutes	Clostridia	Clostridiales	Lachnospiraceae	Unknown
ASV_2831	Bacteria	Firmicutes	Clostridia	Clostridiales	Ruminococcaceae	Unknown
ASV_2832	Bacteria	Firmicutes	Clostridia	Clostridiales	Lachnospiraceae	Unknown
ASV_2833	Bacteria	Firmicutes	Clostridia	Clostridiales	Clostridiales_vadinBB60_group	Unknown
ASV_2834	Bacteria	Firmicutes	Clostridia	Clostridiales	Ruminococcaceae	Oscillibacter
ASV_2835	Bacteria	Firmicutes	Clostridia	Clostridiales	Ruminococcaceae	Ruminococcaceae_UCG-014
ASV_2836	Bacteria	Tenericutes	Mollicutes	Anaeroplasmatales	Anaeroplasmataceae	Anaeroplasma
ASV_2837	Bacteria	Firmicutes	Clostridia	Clostridiales	Lachnospiraceae	Unknown
ASV_2838	Bacteria	Firmicutes	Clostridia	Clostridiales	Lachnospiraceae	Unknown
ASV_2839	Bacteria	Bacteroidetes	Bacteroidia	Bacteroidales	Prevotellaceae	Prevotellaceae_UCG-001
ASV_2840	Bacteria	Bacteroidetes	Bacteroidia	Bacteroidales	Muribaculaceae	Unknown
ASV_2841	Bacteria	Firmicutes	Clostridia	Clostridiales	Ruminococcaceae	Unknown
ASV_2842	Bacteria	Firmicutes	Clostridia	Clostridiales	Ruminococcaceae	UBA1819
ASV_2843	Bacteria	Firmicutes	Clostridia	Clostridiales	Ruminococcaceae	Oscillibacter
ASV_2844	Bacteria	Firmicutes	Clostridia	Clostridiales	Lachnospiraceae	Unknown
ASV_2845	Bacteria	Bacteroidetes	Bacteroidia	Bacteroidales	Prevotellaceae	Prevotellaceae_UCG-001
ASV_2846	Bacteria	Firmicutes	Bacilli	Unknown		
ASV_2847	Bacteria	Firmicutes	Clostridia	Clostridiales	Lachnospiraceae	Unknown
ASV_2848	Bacteria	Firmicutes	Clostridia	Clostridiales	Lachnospiraceae	Lachnospiraceae_NK4A136_group
ASV_2849	Bacteria	Firmicutes	Clostridia	Clostridiales	Lachnospiraceae	Lachnospiraceae_UCG-006
ASV_2850	Bacteria	Bacteroidetes	Bacteroidia	Bacteroidales	Rikenellaceae	Alistipes
ASV_2851	Bacteria	Bacteroidetes	Bacteroidia	Bacteroidales	Muribaculaceae	Unknown
ASV_2852	Bacteria	Firmicutes	Clostridia	Clostridiales	Lachnospiraceae	Lachnospiraceae_NK4A136_group
ASV_2853	Bacteria			Unknown		
ASV_2854	Bacteria	Firmicutes	Clostridia	Clostridiales	Lachnospiraceae	Unknown
ASV_2855	Bacteria	Proteobacteria	Deltaproteobacteria	Desulfovibrionales	Desulfovibrionaceae	Bilophila
ASV_2856	Bacteria	Bacteroidetes	Bacteroidia	Bacteroidales	Muribaculaceae	Unknown
ASV_2857	Bacteria	Bacteroidetes	Bacteroidia	Bacteroidales	Rikenellaceae	Alistipes
ASV_2858	Bacteria	Firmicutes	Clostridia	Clostridiales	Lachnospiraceae	Lachnospiraceae_NK4A136_group
ASV_2859	Bacteria	Bacteroidetes	Bacteroidia	Bacteroidales	Muribaculaceae	Unknown
ASV_2860	Bacteria	Bacteroidetes	Bacteroidia	Bacteroidales	Muribaculaceae	Unknown
ASV_2861	Bacteria	Bacteroidetes	Bacteroidia	Bacteroidales	Muribaculaceae	Unknown
ASV_2862	Bacteria	Firmicutes	Clostridia	Clostridiales	Unknown	

ASV_2863	Bacteria	Firmicutes	Clostridia	Clostridiales	Lachnospiraceae	Lachnospiraceae_UCG-010
ASV_2864	Bacteria	Firmicutes	Clostridia	Clostridiales	Lachnospiraceae	Unknown
ASV_2865	Bacteria	Firmicutes	Clostridia	Clostridiales	Ruminococcaceae	Ruminococcus_1
ASV_2866	Bacteria	Firmicutes	Clostridia	Clostridiales	Lachnospiraceae	Unknown
ASV_2867	Bacteria	Bacteroidetes	Bacteroidia			Unknown
ASV_2868	Bacteria	Firmicutes	Clostridia	Clostridiales	Lachnospiraceae	Unknown
ASV_2869	Bacteria	Firmicutes	Clostridia	Clostridiales	Ruminococcaceae	Unknown
ASV_2870	Bacteria	Firmicutes	Bacilli	Bacillales	Paenibacillaceae	Paenibacillus
ASV_2871	Bacteria	Firmicutes	Bacilli	Lactobacillales	Lactobacillaceae	Lactobacillus
ASV_2872	Bacteria	Bacteroidetes	Bacteroidia	Bacteroidales	Muribaculaceae	Unknown
ASV_2873	Bacteria	Firmicutes	Clostridia	Clostridiales	Ruminococcaceae	Anaerotruncus
ASV_2874	Bacteria	Firmicutes	Clostridia	Clostridiales	Ruminococcaceae	Ruminiclostridium
ASV_2875	Bacteria	Bacteroidetes	Bacteroidia	Bacteroidales	Muribaculaceae	Unknown
ASV_2876	Bacteria	Firmicutes	Erysipelotrichia	Erysipelotrichales	Erysipelotrichaceae	Faecalibaculum
ASV_2877	Bacteria	Bacteroidetes	Bacteroidia	Bacteroidales	Muribaculaceae	Unknown
ASV_2878	Bacteria	Bacteroidetes	Bacteroidia	Bacteroidales	Rikenellaceae	Alistipes
ASV_2879	Bacteria	Firmicutes	Clostridia	Clostridiales	Lachnospiraceae	Unknown
ASV_2880	Bacteria	Bacteroidetes	Bacteroidia	Bacteroidales	Muribaculaceae	Unknown
ASV_2881	Bacteria	Bacteroidetes	Bacteroidia	Bacteroidales	Muribaculaceae	Unknown
ASV_2882	Bacteria	Bacteroidetes	Bacteroidia	Bacteroidales	Muribaculaceae	Unknown
ASV_2883	Bacteria	Bacteroidetes	Bacteroidia	Bacteroidales	Rikenellaceae	Alistipes
ASV_2884	Bacteria	Firmicutes	Bacilli	Lactobacillales	Lactobacillaceae	Lactobacillus
ASV_2885	Bacteria	Bacteroidetes	Bacteroidia	Bacteroidales	Prevotellaceae	Prevotellaceae_UCG-001
ASV_2886	Bacteria	Firmicutes	Clostridia	Clostridiales	Lachnospiraceae	Unknown
ASV_2887	Bacteria	Firmicutes	Clostridia	Clostridiales	Ruminococcaceae	Unknown
ASV_2888	Bacteria	Firmicutes	Clostridia	Clostridiales	Lachnospiraceae	Unknown
ASV_2889	Bacteria	Firmicutes	Clostridia	Clostridiales	Lachnospiraceae	Unknown
ASV_2890	Bacteria	Firmicutes	Bacilli	Bacillales	Staphylococcaceae	Staphylococcus
ASV_2891	Bacteria	Firmicutes	Clostridia	Clostridiales		Unknown
ASV_2892	Bacteria	Bacteroidetes	Bacteroidia	Bacteroidales	Muribaculaceae	Unknown
ASV_2893	Bacteria	Firmicutes	Clostridia	Clostridiales	Ruminococcaceae	Unknown
ASV_2894	Bacteria	Firmicutes	Clostridia	Clostridiales	Lachnospiraceae	Unknown
ASV_2895	Bacteria	Firmicutes	Erysipelotrichia	Erysipelotrichales	Erysipelotrichaceae	Faecalibaculum
ASV_2896	Bacteria	Bacteroidetes	Bacteroidia	Bacteroidales	Muribaculaceae	Unknown
ASV_2897	Bacteria	Firmicutes	Clostridia	Clostridiales	Ruminococcaceae	Ruminiclostridium_6
ASV_2898	Bacteria	Firmicutes	Clostridia	Clostridiales	Ruminococcaceae	Ruminococcaceae_UCG-014
ASV_2899	Bacteria	Firmicutes	Clostridia	Clostridiales	Lachnospiraceae	Lachnospiraceae_NK4A136_group

ASV_2900	Bacteria	Firmicutes	Clostridia	Clostridiales	Lachnospiraceae	Unknown	
ASV_2901	Bacteria	Bacteroidetes	Bacteroidia	Bacteroidales	Prevotellaceae	Prevotellaceae_NK3B31_group	
ASV_2902	Bacteria	Bacteroidetes	Bacteroidia	Bacteroidales	Muribaculaceae	Unknown	
ASV_2903	Bacteria	Firmicutes	Bacilli	Lactobacillales	Lactobacillaceae	Lactobacillus	
ASV_2904	Bacteria	Firmicutes	Bacilli	Lactobacillales		Unknown	
ASV_2905	Bacteria	Firmicutes	Clostridia	Clostridiales	Lachnospiraceae	Unknown	
ASV_2906	Bacteria	Firmicutes	Clostridia	Clostridiales	Lachnospiraceae	Unknown	
ASV_2907	Bacteria	Bacteroidetes	Bacteroidia	Bacteroidales	Muribaculaceae	Unknown	
ASV_2908	Bacteria	Bacteroidetes	Bacteroidia	Bacteroidales	Muribaculaceae	Unknown	
ASV_2909	Bacteria	Tenericutes	Mollicutes	Mollicutes_RF39		Unknown	
ASV_2910	Bacteria	Firmicutes	Clostridia	Clostridiales	Lachnospiraceae	Unknown	
ASV_2911	Bacteria	Firmicutes				Unknown	
ASV_2912	Bacteria	Firmicutes	Bacilli	Lactobacillales	Lactobacillaceae	Lactobacillus	
ASV_2913	Bacteria	Bacteroidetes	Bacteroidia	Bacteroidales	Muribaculaceae	Unknown	
ASV_2914	Bacteria	Firmicutes	Clostridia	Clostridiales	Lachnospiraceae	Lachnospiraceae_UCG-001	
ASV_2915	Bacteria	Bacteroidetes	Bacteroidia	Bacteroidales	Rikenellaceae	Alistipes	
ASV_2916	Bacteria	Firmicutes	Clostridia	Clostridiales	Ruminococcaceae	Ruminococcaceae_UCG-014	
ASV_2917	Bacteria	Firmicutes	Clostridia	Clostridiales	Lachnospiraceae	Unknown	
ASV_2918	Bacteria	Bacteroidetes	Bacteroidia	Bacteroidales	Rikenellaceae	Alistipes	
ASV_2919	Bacteria	Firmicutes	Clostridia	Clostridiales	Ruminococcaceae	Unknown	
ASV_2920	Bacteria	Firmicutes	Clostridia	Clostridiales	Ruminococcaceae	Unknown	
ASV_2921	Bacteria	Bacteroidetes	Bacteroidia	Bacteroidales	Muribaculaceae	Unknown	
ASV_2922	Bacteria	Firmicutes	Clostridia	Clostridiales		Unknown	
ASV_2923	Bacteria	Firmicutes	Clostridia	Clostridiales	Ruminococcaceae	Anaerotruncus	
ASV_2924	Bacteria	Firmicutes	Clostridia	Clostridiales	Lachnospiraceae	Unknown	
ASV_2925	Bacteria	Firmicutes	Clostridia	Clostridiales	Lachnospiraceae	A2	
ASV_2926	Bacteria	Actinobacteria	Coriobacteriia	Coriobacteriales	Eggerthellaceae	Enterorhabdus	
ASV_2927	Bacteria	Bacteroidetes	Bacteroidia	Bacteroidales	Rikenellaceae	Alistipes	
ASV_2928	Bacteria	Bacteroidetes	Bacteroidia	Flavobacteriales	Weeksellaceae	Cloacibacterium	Cloacibacterium_normanense
ASV_2929	Bacteria					Unknown	
ASV_2930	Bacteria	Firmicutes	Clostridia	Clostridiales	Lachnospiraceae	Acetatifactor	
ASV_2931	Bacteria	Firmicutes	Clostridia	Clostridiales	Ruminococcaceae	Unknown	
ASV_2932	Bacteria	Firmicutes	Clostridia	Clostridiales	Ruminococcaceae	Ruminiclostridium_9	
ASV_2933	Bacteria	Bacteroidetes	Bacteroidia	Bacteroidales		Unknown	
ASV_2934	Bacteria	Firmicutes	Clostridia	Clostridiales	Lachnospiraceae	A2	
ASV_2935	Bacteria	Bacteroidetes	Bacteroidia	Bacteroidales	Muribaculaceae	Unknown	
ASV_2936	Bacteria	Bacteroidetes	Bacteroidia	Bacteroidales	Prevotellaceae	Prevotellaceae_UCG-001	

ASV_2937	Bacteria	Bacteroidetes	Bacteroidia	Bacteroidales	Prevotellaceae	Prevotellaceae_UCG-001
ASV_2938	Bacteria	Bacteroidetes	Bacteroidia	Bacteroidales	Muribaculaceae	Unknown
ASV_2939	Bacteria	Proteobacteria	Deltaproteobacteria	Desulfovibrionales	Desulfovibrionaceae	Bilophila
ASV_2940	Bacteria	Bacteroidetes	Bacteroidia	Bacteroidales	Muribaculaceae	Unknown
ASV_2941	Bacteria	Firmicutes	Clostridia	Clostridiales	Lachnospiraceae	Unknown
ASV_2942	Bacteria	Bacteroidetes	Bacteroidia	Bacteroidales	Muribaculaceae	Unknown
ASV_2943	Bacteria	Tenericutes	Mollicutes	Mollicutes_RF39	Unknown	
ASV_2944	Bacteria	Firmicutes	Clostridia	Clostridiales	Ruminococcaceae	Ruminococcaceae_UCG-013
ASV_2945	Bacteria	Bacteroidetes	Bacteroidia	Bacteroidales	Prevotellaceae	Prevotellaceae_NK3B31_group
ASV_2946	Bacteria	Firmicutes	Clostridia	Clostridiales	Ruminococcaceae	Unknown
ASV_2947	Bacteria	Firmicutes	Clostridia	Clostridiales	Christensenellaceae	Unknown
ASV_2948	Bacteria	Bacteroidetes	Bacteroidia	Bacteroidales	Prevotellaceae	Prevotellaceae_NK3B31_group
ASV_2949	Bacteria	Firmicutes	Clostridia	Clostridiales	Lachnospiraceae	Unknown
ASV_2950	Bacteria	Bacteroidetes	Bacteroidia	Bacteroidales	Unknown	
ASV_2951	Bacteria	Firmicutes	Clostridia	Clostridiales	Lachnospiraceae	Lachnospiraceae_UCG-006
ASV_2952	Bacteria	Firmicutes	Clostridia	Clostridiales	Lachnospiraceae	Unknown
ASV_2953	Bacteria	Actinobacteria	Coriobacteriia	Coriobacteriales	Eggerthellaceae	Enterorhabdus
ASV_2954	Bacteria	Deferribacteres	Deferribacteres	Deferribacterales	Deferribacteraceae	Mucispirillum
ASV_2955	Bacteria	Firmicutes	Clostridia	Clostridiales	Lachnospiraceae	Lachnospiraceae_UCG-001
ASV_2956	Bacteria	Bacteroidetes	Bacteroidia	Bacteroidales	Unknown	
ASV_2957	Bacteria	Bacteroidetes	Bacteroidia	Bacteroidales	Unknown	
ASV_2958	Bacteria	Firmicutes	Clostridia	Clostridiales	Lachnospiraceae	Unknown
ASV_2959	Bacteria	Firmicutes	Clostridia	Clostridiales	Unknown	
ASV_2960	Bacteria	Bacteroidetes	Bacteroidia	Bacteroidales	Rikenellaceae	Alistipes
ASV_2961	Bacteria	Bacteroidetes	Bacteroidia		Unknown	
ASV_2962	Bacteria	Firmicutes	Clostridia	Clostridiales	Lachnospiraceae	Lachnospiraceae_NK4A136_group
ASV_2963	Bacteria	Firmicutes	Clostridia	Clostridiales	Ruminococcaceae	Unknown
ASV_2964	Bacteria	Firmicutes	Clostridia	Clostridiales	Ruminococcaceae	Unknown
ASV_2965	Bacteria	Firmicutes	Clostridia	Clostridiales	Ruminococcaceae	Ruminiclostridium_9
ASV_2966	Bacteria	Firmicutes	Clostridia	Clostridiales	Ruminococcaceae	Ruminiclostridium_9
ASV_2967	Bacteria	Firmicutes	Clostridia	Clostridiales	Lachnospiraceae	Lachnospiraceae_NK4A136_group
ASV_2968	Bacteria	Firmicutes	Clostridia	Clostridiales	Ruminococcaceae	Unknown
ASV_2969	Bacteria	Firmicutes	Clostridia	Clostridiales	Ruminococcaceae	Ruminiclostridium_9
ASV_2970	Bacteria	Firmicutes	Clostridia	Clostridiales	Lachnospiraceae	Unknown
ASV_2971	Bacteria	Firmicutes	Clostridia	Clostridiales	Unknown	
ASV_2972	Bacteria	Firmicutes	Clostridia	Clostridiales	Clostridiales_vadinBB60_group	Unknown
ASV_2973	Bacteria	Firmicutes	Clostridia	Clostridiales	Ruminococcaceae	Ruminococcaceae_UCG-014

ASV_2974	Bacteria	Firmicutes	Clostridia	Clostridiales	Lachnospiraceae	Unknown
ASV_2975	Bacteria	Bacteroidetes	Bacteroidia	Bacteroidales	Muribaculaceae	Unknown
ASV_2976	Bacteria	Firmicutes	Clostridia	Clostridiales	Lachnospiraceae	Unknown
ASV_2977	Bacteria	Firmicutes	Clostridia	Clostridiales	Lachnospiraceae	ASF356
ASV_2978	Bacteria	Firmicutes	Bacilli	Lactobacillales	Lactobacillaceae	Lactobacillus
ASV_2979	Bacteria	Firmicutes	Clostridia	Clostridiales	Ruminococcaceae	Unknown
ASV_2980	Bacteria	Bacteroidetes	Bacteroidia	Bacteroidales	Muribaculaceae	Unknown
ASV_2981	Bacteria	Firmicutes	Clostridia	Clostridiales	Lachnospiraceae	Unknown
ASV_2982	Bacteria	Bacteroidetes	Bacteroidia	Bacteroidales	Muribaculaceae	Unknown
ASV_2983	Bacteria	Firmicutes	Bacilli	Lactobacillales	Streptococcaceae	Streptococcus
ASV_2984	Bacteria	Firmicutes	Clostridia	Clostridiales	Clostridiales_vadinBB60_group	Unknown
ASV_2985	Bacteria	Firmicutes	Clostridia	Clostridiales	Ruminococcaceae	Unknown
ASV_2986	Bacteria	Firmicutes	Clostridia	Clostridiales		Unknown
ASV_2987	Bacteria	Firmicutes	Erysipelotrichia	Erysipelotrichales	Erysipelotrichaceae	Erysipelatoclostridium
ASV_2988	Bacteria	Bacteroidetes	Bacteroidia	Bacteroidales	Muribaculaceae	Unknown
ASV_2989	Bacteria	Firmicutes	Clostridia	Clostridiales	Lachnospiraceae	Lachnospiraceae_NK4A136_group
ASV_2990	Bacteria	Firmicutes	Clostridia	Clostridiales	Ruminococcaceae	Unknown
ASV_2991	Bacteria	Bacteroidetes	Bacteroidia	Bacteroidales	Marinifilaceae	Odoribacter
ASV_2992	Bacteria	Firmicutes	Clostridia	Clostridiales	Lachnospiraceae	Unknown
ASV_2993	Bacteria	Firmicutes	Clostridia	Clostridiales	Lachnospiraceae	Unknown
ASV_2994	Bacteria	Firmicutes	Bacilli	Lactobacillales	Lactobacillaceae	Lactobacillus
ASV_2995	Bacteria	Firmicutes	Clostridia	Clostridiales	Ruminococcaceae	Oscillibacter
ASV_2996	Bacteria	Firmicutes	Erysipelotrichia	Erysipelotrichales	Erysipelotrichaceae	Candidatus_Stoquefichus
ASV_2997	Bacteria	Tenericutes	Mollicutes	Mollicutes_RF39		Unknown
ASV_2998	Bacteria	Firmicutes	Clostridia	Clostridiales	Ruminococcaceae	Unknown
ASV_2999	Bacteria	Firmicutes	Clostridia	Clostridiales	Lachnospiraceae	A2
ASV_3000	Bacteria	Tenericutes	Mollicutes	Mollicutes_RF39		Unknown
ASV_3001	Bacteria	Firmicutes	Clostridia	Clostridiales	Ruminococcaceae	Ruminococcaceae_UCG-014
ASV_3002	Bacteria	Firmicutes	Clostridia	Clostridiales	Lachnospiraceae	Unknown
ASV_3003	Bacteria					Unknown
ASV_3004	Bacteria	Bacteroidetes	Bacteroidia	Bacteroidales	Prevotellaceae	Prevotellaceae_UCG-001
ASV_3005	Bacteria	Firmicutes	Clostridia	Clostridiales	Clostridiales_vadinBB60_group	Unknown
ASV_3006	Bacteria	Firmicutes	Clostridia	Clostridiales	Lachnospiraceae	Unknown
ASV_3007	Bacteria	Bacteroidetes	Bacteroidia			Unknown
ASV_3008	Bacteria	Firmicutes	Bacilli	Lactobacillales	Lactobacillaceae	Lactobacillus
ASV_3009	Bacteria	Firmicutes	Clostridia	Clostridiales	Clostridiales_vadinBB60_group	Unknown
ASV_3010	Bacteria	Firmicutes	Clostridia	Clostridiales	Clostridiaceae_1	Unknown

ASV_3011	Bacteria	Firmicutes	Clostridia	Clostridiales	Ruminococcaceae	Unknown
ASV_3012	Bacteria	Bacteroidetes	Bacteroidia	Bacteroidales	Muribaculaceae	Unknown
ASV_3013	Bacteria	Firmicutes	Clostridia	Clostridiales	Lachnospiraceae	Unknown
ASV_3014	Bacteria	Firmicutes	Clostridia	Clostridiales	Ruminococcaceae	Unknown
ASV_3015	Bacteria	Firmicutes	Bacilli	Lactobacillales	Lactobacillaceae	Lactobacillus
ASV_3016	Bacteria	Bacteroidetes	Bacteroidia	Bacteroidales	Muribaculaceae	Unknown
ASV_3017	Bacteria	Actinobacteria	Coriobacteriia	Coriobacteriales	Eggerthellaceae	Unknown
ASV_3018	Bacteria	Firmicutes	Clostridia	Clostridiales	Ruminococcaceae	Ruminococcaceae_UCG-014
ASV_3019	Bacteria	Firmicutes	Clostridia	Clostridiales	Lachnospiraceae	Unknown
ASV_3020	Bacteria	Firmicutes	Erysipelotrichia	Erysipelotrichales	Erysipelotrichaceae	Unknown
ASV_3021	Bacteria	Verrucomicrobia	Verrucomicrobiae	Verrucomicrobiales	Akkermansiaceae	Akkermansia
ASV_3022	Bacteria	Bacteroidetes	Bacteroidia	Bacteroidales	Rikenellaceae	Alistipes
ASV_3023	Bacteria	Firmicutes	Clostridia	Clostridiales	Ruminococcaceae	Ruminiclostridium
ASV_3024	Bacteria	Firmicutes	Clostridia	Clostridiales	Ruminococcaceae	Ruminiclostridium_9
ASV_3025	Bacteria	Bacteroidetes	Bacteroidia	Bacteroidales	Unknown	
ASV_3026	Bacteria	Firmicutes	Clostridia	Clostridiales	Ruminococcaceae	UBA1819
ASV_3027	Bacteria	Bacteroidetes	Bacteroidia	Bacteroidales	Muribaculaceae	Unknown
ASV_3028	Bacteria	Firmicutes	Clostridia	Clostridiales	Unknown	
ASV_3029	Bacteria	Firmicutes	Clostridia	Clostridiales	Ruminococcaceae	Anaerotruncus
ASV_3030	Bacteria	Bacteroidetes	Bacteroidia	Bacteroidales	Muribaculaceae	Unknown
ASV_3031	Bacteria	Bacteroidetes	Bacteroidia	Bacteroidales	Tannerellaceae	Parabacteroides
ASV_3032	Bacteria	Firmicutes	Unknown			
ASV_3033	Bacteria	Bacteroidetes	Bacteroidia	Bacteroidales	Muribaculaceae	CAG-873
ASV_3034	Bacteria	Firmicutes	Bacilli	Lactobacillales	Lactobacillaceae	Lactobacillus
ASV_3035	Bacteria	Firmicutes	Clostridia	Clostridiales	Ruminococcaceae	UBA1819
ASV_3036	Bacteria	Firmicutes	Clostridia	Clostridiales	Ruminococcaceae	GCA-900066225
ASV_3037	Bacteria	Bacteroidetes	Bacteroidia	Bacteroidales	Muribaculaceae	CAG-873
ASV_3038	Bacteria	Unknown				
ASV_3039	Bacteria	Bacteroidetes	Bacteroidia	Bacteroidales	Muribaculaceae	Unknown
ASV_3040	Bacteria	Unknown				
ASV_3041	Bacteria	Firmicutes	Clostridia	Clostridiales	Lachnospiraceae	Unknown
ASV_3042	Bacteria	Bacteroidetes	Bacteroidia	Bacteroidales	Muribaculaceae	Unknown
ASV_3043	Bacteria	Bacteroidetes	Bacteroidia	Bacteroidales	Muribaculaceae	Unknown
ASV_3044	Bacteria	Firmicutes	Clostridia	Clostridiales	Ruminococcaceae	Unknown
ASV_3045	Bacteria	Firmicutes	Clostridia	Clostridiales	Lachnospiraceae	Unknown
ASV_3046	Bacteria	Firmicutes	Clostridia	Clostridiales	Lachnospiraceae	Lachnospiraceae_NK4A136_group
ASV_3047	Bacteria	Bacteroidetes	Bacteroidia	Bacteroidales	Rikenellaceae	Alistipes

ASV_3048	Bacteria	Firmicutes	Clostridia	Clostridiales	Unknown	
ASV_3049	Bacteria	Bacteroidetes	Bacteroidia	Bacteroidales	Muribaculaceae	CAG-873
ASV_3050	Bacteria	Firmicutes	Clostridia	Clostridiales	Ruminococcaceae	Unknown
ASV_3051	Bacteria	Bacteroidetes	Bacteroidia	Bacteroidales	Muribaculaceae	Unknown
ASV_3052	Bacteria			Unknown		
ASV_3053	Bacteria	Firmicutes	Clostridia	Clostridiales	Lachnospiraceae	Unknown
ASV_3054	Bacteria	Firmicutes		Unknown		
ASV_3055	Bacteria	Bacteroidetes	Bacteroidia	Bacteroidales	Muribaculaceae	Unknown
ASV_3056	Bacteria	Bacteroidetes	Bacteroidia	Bacteroidales	Rikenellaceae	Alistipes
ASV_3057	Bacteria	Firmicutes	Clostridia	Clostridiales	Unknown	
ASV_3058	Bacteria	Firmicutes	Clostridia	Clostridiales	Ruminococcaceae	Unknown
ASV_3059	Bacteria	Bacteroidetes	Bacteroidia	Bacteroidales	Muribaculaceae	CAG-873
ASV_3060	Bacteria	Bacteroidetes	Bacteroidia		Unknown	
ASV_3061	Bacteria	Firmicutes	Clostridia	Clostridiales	Ruminococcaceae	Unknown
ASV_3062	Bacteria	Firmicutes	Clostridia	Clostridiales	Ruminococcaceae	Unknown
ASV_3063	Bacteria	Firmicutes	Clostridia	Clostridiales	Lachnospiraceae	Unknown
ASV_3064	Bacteria	Firmicutes	Clostridia	Clostridiales	Ruminococcaceae	Ruminiclostridium_9
ASV_3065	Bacteria	Firmicutes	Clostridia	Clostridiales	Ruminococcaceae	Unknown
ASV_3066	Bacteria	Bacteroidetes	Bacteroidia	Bacteroidales	Muribaculaceae	Unknown
ASV_3067	Bacteria	Firmicutes	Clostridia	Clostridiales	Unknown	
ASV_3068	Bacteria	Firmicutes	Clostridia	Clostridiales	Ruminococcaceae	Ruminiclostridium
ASV_3069	Bacteria	Firmicutes	Clostridia	Clostridiales	Ruminococcaceae	Unknown
ASV_3070	Bacteria	Bacteroidetes	Bacteroidia	Bacteroidales	Muribaculaceae	Unknown
ASV_3071	Bacteria	Bacteroidetes	Bacteroidia	Bacteroidales	Rikenellaceae	Alistipes
ASV_3072	Bacteria	Firmicutes	Clostridia	Clostridiales	Ruminococcaceae	Unknown
ASV_3073	Bacteria	Bacteroidetes	Bacteroidia	Bacteroidales	Prevotellaceae	Prevotellaceae_NK3B31_group
ASV_3074	Bacteria	Firmicutes	Clostridia	Clostridiales	Ruminococcaceae	Ruminococcaceae_UCG-014
ASV_3075	Bacteria	Firmicutes	Clostridia	Clostridiales	Ruminococcaceae	Ruminococcaceae_UCG-014
ASV_3076	Bacteria			Unknown		
ASV_3077	Bacteria	Firmicutes	Clostridia	Clostridiales	Clostridiales_vadinBB60_group	Unknown
ASV_3078	Bacteria	Firmicutes	Clostridia	Clostridiales	Lachnospiraceae	Lachnospiraceae_NK4A136_group
ASV_3079	Bacteria	Cyanobacteria	Melainabacteria	Gastranaerophilales		Unknown
ASV_3080	Bacteria	Firmicutes	Clostridia	Clostridiales	Lachnospiraceae	Unknown
ASV_3081	Bacteria	Firmicutes	Clostridia	Clostridiales	Ruminococcaceae	Unknown
ASV_3082	Bacteria	Bacteroidetes	Bacteroidia		Unknown	
ASV_3083	Bacteria	Firmicutes	Clostridia	Clostridiales	Ruminococcaceae	Ruminiclostridium
ASV_3084	Bacteria	Bacteroidetes	Bacteroidia	Bacteroidales	Muribaculaceae	Unknown

ASV_3085	Bacteria	Firmicutes	Clostridia	Clostridiales		Unknown
ASV_3086	Bacteria	Firmicutes	Clostridia	Clostridiales	Lachnospiraceae	Unknown
ASV_3087	Bacteria	Firmicutes	Clostridia	Clostridiales	Lachnospiraceae	Unknown
ASV_3088	Bacteria	Bacteroidetes	Bacteroidia	Bacteroidales	Rikenellaceae	Alistipes
ASV_3089	Bacteria	Firmicutes	Clostridia	Clostridiales	Ruminococcaceae	Unknown
ASV_3090	Bacteria	Bacteroidetes	Bacteroidia	Bacteroidales	Muribaculaceae	Unknown
ASV_3091	Bacteria	Firmicutes	Clostridia	Clostridiales	Lachnospiraceae	Lachnospiraceae_NK4A136_group
ASV_3092	Bacteria	Firmicutes	Clostridia	Clostridiales	Lachnospiraceae	Unknown
ASV_3093	Bacteria	Firmicutes	Clostridia	Clostridiales	Ruminococcaceae	Unknown
ASV_3094	Bacteria	Firmicutes	Clostridia	Clostridiales	Ruminococcaceae	Phocea
ASV_3095	Bacteria	Firmicutes	Clostridia	Clostridiales	Lachnospiraceae	Lachnospiraceae_NK4A136_group
ASV_3096	Bacteria	Firmicutes	Clostridia	Clostridiales	Lachnospiraceae	Unknown
ASV_3097	Bacteria	Tenericutes	Mollicutes	Mollicutes_RF39		Unknown
ASV_3098	Bacteria	Firmicutes	Bacilli			Unknown
ASV_3099	Bacteria	Firmicutes	Erysipelotrichia	Erysipelotrichales	Erysipelotrichaceae	Faecalibaculum
ASV_3100	Bacteria	Firmicutes	Clostridia	Clostridiales	Lachnospiraceae	A2
ASV_3101	Bacteria	Firmicutes	Clostridia	Clostridiales	Ruminococcaceae	Oscillibacter
ASV_3102	Bacteria	Firmicutes	Clostridia	Clostridiales	Lachnospiraceae	Unknown
ASV_3103	Bacteria	Firmicutes	Clostridia	Clostridiales	Ruminococcaceae	Ruminiclostridium_9
ASV_3104	Bacteria	Firmicutes	Clostridia	Clostridiales	Lachnospiraceae	Unknown
ASV_3105	Bacteria	Firmicutes	Clostridia	Clostridiales		Unknown
ASV_3106	Bacteria	Firmicutes	Bacilli	Lactobacillales	Lactobacillaceae	Lactobacillus
ASV_3107	Bacteria	Firmicutes	Clostridia	Clostridiales	Peptococcaceae	Unknown
ASV_3108	Bacteria	Firmicutes	Negativicutes	Selenomonadales	Veillonellaceae	Dialister
ASV_3109	Bacteria	Firmicutes	Clostridia	Clostridiales	Clostridiales_vadinBB60_group	Unknown
ASV_3110	Bacteria	Firmicutes	Clostridia	Clostridiales	Ruminococcaceae	Ruminiclostridium_5
ASV_3111	Bacteria	Bacteroidetes	Bacteroidia	Bacteroidales		Unknown
ASV_3112	Bacteria	Bacteroidetes	Bacteroidia	Bacteroidales	Muribaculaceae	Unknown
ASV_3113	Bacteria	Firmicutes	Bacilli	Lactobacillales	Lactobacillaceae	Lactobacillus
ASV_3114	Bacteria	Firmicutes	Clostridia	Clostridiales	Lachnospiraceae	ASF356
ASV_3115	Bacteria	Firmicutes	Clostridia	Clostridiales	Ruminococcaceae	Ruminiclostridium_5
ASV_3116	Bacteria	Bacteroidetes	Bacteroidia	Bacteroidales	Muribaculaceae	Unknown
ASV_3117	Bacteria	Bacteroidetes	Bacteroidia	Bacteroidales	Muribaculaceae	CAG-873
ASV_3118	Bacteria					Unknown
ASV_3119	Bacteria	Bacteroidetes	Bacteroidia	Bacteroidales	Muribaculaceae	Unknown
ASV_3120	Bacteria	Firmicutes	Clostridia	Clostridiales	Lachnospiraceae	Unknown
ASV_3121	Bacteria	Firmicutes	Clostridia	Clostridiales	Ruminococcaceae	Ruminococcaceae_UCG-014

ASV_3122	Bacteria	Firmicutes	Clostridia	Clostridiales	Lachnospiraceae	ASF356
ASV_3123	Bacteria	Proteobacteria	Gammaproteobacteria	Betaproteobacteriales	Burkholderiaceae	Parasutterella
ASV_3124	Bacteria	Firmicutes	Clostridia	Clostridiales	Lachnospiraceae	Unknown
ASV_3125	Bacteria	Firmicutes	Clostridia	Clostridiales	Ruminococcaceae	Ruminococcaceae_UCG-014
ASV_3126	Bacteria	Firmicutes	Clostridia	Clostridiales	Lachnospiraceae	Unknown
ASV_3127	Bacteria	Firmicutes	Clostridia	Clostridiales		Unknown
ASV_3128	Bacteria	Bacteroidetes	Bacteroidia	Bacteroidales		Unknown
ASV_3129	Bacteria	Bacteroidetes	Bacteroidia	Bacteroidales		Unknown
ASV_3130	Bacteria	Firmicutes	Clostridia	Clostridiales	Ruminococcaceae	Ruminiclostridium
ASV_3131	Bacteria	Firmicutes	Clostridia	Clostridiales	Lachnospiraceae	Unknown
ASV_3132	Bacteria	Bacteroidetes	Bacteroidia	Bacteroidales	Muribaculaceae	Unknown
ASV_3133	Bacteria	Firmicutes	Clostridia	Clostridiales	Lachnospiraceae	Unknown
ASV_3134	Bacteria	Bacteroidetes	Bacteroidia	Bacteroidales	Muribaculaceae	Unknown
ASV_3135	Bacteria	Bacteroidetes	Bacteroidia	Bacteroidales	Muribaculaceae	Unknown
ASV_3136	Bacteria	Bacteroidetes	Bacteroidia	Bacteroidales	Muribaculaceae	Unknown
ASV_3137	Bacteria	Firmicutes	Erysipelotrichia	Erysipelotrichales	Erysipelotrichaceae	Unknown
ASV_3138	Bacteria	Firmicutes	Clostridia	Clostridiales	Ruminococcaceae	Unknown
ASV_3139	Bacteria	Bacteroidetes	Bacteroidia	Bacteroidales	Tannerellaceae	Parabacteroides
ASV_3140	Bacteria	Firmicutes	Clostridia	Clostridiales	Lachnospiraceae	Lachnospiraceae_NK4A136_group
ASV_3141	Bacteria	Firmicutes	Clostridia	Clostridiales	Lachnospiraceae	Unknown
ASV_3142	Bacteria	Proteobacteria	Deltaproteobacteria	Desulfovibrionales	Desulfovibrionaceae	Bilophila
ASV_3143	Bacteria	Firmicutes	Clostridia	Clostridiales	Lachnospiraceae	Lachnospiraceae_NK4A136_group
ASV_3144	Bacteria	Firmicutes				Unknown
ASV_3145	Bacteria	Firmicutes	Erysipelotrichia	Erysipelotrichales	Erysipelotrichaceae	Faecalibaculum
ASV_3146	Bacteria	Firmicutes	Clostridia	Clostridiales	Ruminococcaceae	Ruminococcaceae_UCG-014
ASV_3147	Bacteria	Firmicutes	Clostridia	Clostridiales	Lachnospiraceae	Lachnospiraceae_NK4A136_group
ASV_3148	Bacteria	Firmicutes	Clostridia	Clostridiales	Ruminococcaceae	Ruminococcaceae_UCG-014
ASV_3149	Bacteria	Firmicutes	Clostridia	Clostridiales	Lachnospiraceae	Lachnospiraceae_NK4A136_group
ASV_3150	Bacteria	Bacteroidetes	Bacteroidia	Bacteroidales	Rikenellaceae	Alistipes
ASV_3151	Bacteria	Firmicutes	Clostridia	Clostridiales	Ruminococcaceae	Ruminococcaceae_UCG-014
ASV_3152	Bacteria	Firmicutes	Clostridia	Clostridiales	Lachnospiraceae	Unknown
ASV_3153	Bacteria	Firmicutes	Clostridia	Clostridiales	Lachnospiraceae	Lachnospiraceae_NK4A136_group
ASV_3154	Bacteria	Firmicutes	Clostridia	Clostridiales	Ruminococcaceae	Butyricicoccus
ASV_3155	Bacteria	Bacteroidetes	Bacteroidia	Bacteroidales	Rikenellaceae	Alistipes
ASV_3156	Bacteria	Bacteroidetes	Bacteroidia	Bacteroidales	Rikenellaceae	Alistipes
ASV_3157	Bacteria					Unknown
ASV_3158	Bacteria	Firmicutes	Clostridia	Clostridiales	Ruminococcaceae	Harryflintia

ASV_3159	Bacteria	Firmicutes	Unknown			
ASV_3160	Bacteria	Firmicutes	Clostridia	Clostridiales	Lachnospiraceae	Unknown
ASV_3161	Bacteria	Firmicutes	Clostridia	Clostridiales	Unknown	
ASV_3162	Bacteria	Firmicutes	Clostridia	Clostridiales	Ruminococcaceae	Ruminococcaceae_UCG-014
ASV_3163	Bacteria	Firmicutes	Clostridia	Clostridiales	Lachnospiraceae	Lachnospiraceae_NK4A136_group
ASV_3164	Bacteria	Firmicutes	Clostridia	Clostridiales	Peptococcaceae	Unknown
ASV_3165	Bacteria	Firmicutes	Clostridia	Clostridiales	Ruminococcaceae	Unknown
ASV_3166	Bacteria	Firmicutes	Clostridia	Clostridiales	Lachnospiraceae	A2
ASV_3167	Bacteria	Firmicutes	Clostridia	Clostridiales	Lachnospiraceae	Lachnospiraceae_NK4A136_group
ASV_3168	Bacteria	Firmicutes	Erysipelotrichia	Erysipelotrichales	Erysipelotrichaceae	Faecalibaculum
ASV_3169	Bacteria	Firmicutes	Clostridia	Clostridiales	Lachnospiraceae	Unknown
ASV_3170	Bacteria	Firmicutes	Bacilli	Lactobacillales	Lactobacillaceae	Lactobacillus
ASV_3171	Bacteria	Firmicutes	Clostridia	Clostridiales	Lachnospiraceae	Lachnospiraceae_NK4A136_group
ASV_3172	Bacteria	Bacteroidetes	Bacteroidia	Bacteroidales	Muribaculaceae	Unknown
ASV_3173	Bacteria	Bacteroidetes	Bacteroidia	Bacteroidales	Muribaculaceae	Unknown
ASV_3174	Bacteria	Bacteroidetes	Bacteroidia	Bacteroidales	Rikenellaceae	Alistipes
ASV_3175	Bacteria	Proteobacteria	Deltaproteobacteria	Desulfovibrionales	Desulfovibrionaceae	Bilophila
ASV_3176	Bacteria	Firmicutes	Clostridia	Clostridiales	Ruminococcaceae	Ruminococcaceae_UCG-014
ASV_3177	Bacteria	Firmicutes	Clostridia	Clostridiales	Unknown	
ASV_3178	Bacteria	Firmicutes	Clostridia	Clostridiales	Lachnospiraceae	Lachnospiraceae_UCG-006
ASV_3179	Bacteria	Bacteroidetes	Bacteroidia	Bacteroidales	Prevotellaceae	Prevotellaceae_UCG-001
ASV_3180	Bacteria	Firmicutes	Clostridia	Clostridiales	Ruminococcaceae	Ruminiclostridium_9
ASV_3181	Bacteria	Firmicutes	Clostridia	Clostridiales	Ruminococcaceae	Oscillibacter
ASV_3182	Bacteria	Tenericutes	Mollicutes	Mollicutes_RF39	Unknown	
ASV_3183	Bacteria	Firmicutes	Clostridia	Clostridiales	Lachnospiraceae	Unknown
ASV_3184	Bacteria	Proteobacteria	Gammaproteobacteria	Betaproteobacteriales	Burkholderiaceae	Parasutterella
ASV_3185	Bacteria	Firmicutes	Clostridia	Clostridiales	Clostridiales_vadinBB60_group	Unknown
ASV_3186	Bacteria	Bacteroidetes	Bacteroidia	Bacteroidales	Rikenellaceae	Alistipes
ASV_3187	Bacteria	Firmicutes	Clostridia	Clostridiales	Lachnospiraceae	Unknown
ASV_3188	Bacteria	Firmicutes	Clostridia	Clostridiales	Lachnospiraceae	Lachnospiraceae_NK4A136_group
ASV_3189	Bacteria	Bacteroidetes	Bacteroidia	Bacteroidales	Muribaculaceae	Unknown
ASV_3190	Bacteria	Firmicutes	Clostridia	Clostridiales	Ruminococcaceae	Ruminiclostridium_5
ASV_3191	Bacteria	Firmicutes	Clostridia	Clostridiales	Lachnospiraceae	Lachnospiraceae_UCG-001
ASV_3192	Bacteria	Firmicutes	Clostridia	Clostridiales	Ruminococcaceae	Unknown
ASV_3193	Bacteria	Bacteroidetes	Bacteroidia	Bacteroidales	Rikenellaceae	Alistipes
ASV_3194	Bacteria	Unknown				
ASV_3195	Bacteria	Bacteroidetes	Bacteroidia	Bacteroidales	Unknown	

ASV_3196	Bacteria	Tenericutes	Mollicutes	Mollicutes_RF39	Unknown	
ASV_3197	Bacteria	Firmicutes	Clostridia	Clostridiales	Ruminococcaceae	Ruminiclostridium_5
ASV_3198	Bacteria	Firmicutes	Clostridia	Clostridiales	Ruminococcaceae	Ruminiclostridium
ASV_3199	Bacteria	Unknown				
ASV_3200	Bacteria	Firmicutes	Clostridia	Clostridiales	Lachnospiraceae	ASF356
ASV_3201	Bacteria	Bacteroidetes	Bacteroidia	Bacteroidales	Muribaculaceae	Unknown
ASV_3202	Bacteria	Bacteroidetes	Bacteroidia	Bacteroidales	Muribaculaceae	Unknown
ASV_3203	Bacteria	Firmicutes	Clostridia	Clostridiales	Ruminococcaceae	Ruminiclostridium
ASV_3204	Bacteria	Bacteroidetes	Bacteroidia	Bacteroidales	Muribaculaceae	Unknown
ASV_3205	Bacteria	Firmicutes	Clostridia	Clostridiales	Ruminococcaceae	Unknown
ASV_3206	Bacteria	Bacteroidetes	Bacteroidia	Bacteroidales	Rikenellaceae	Alistipes
ASV_3207	Bacteria	Firmicutes	Clostridia	Clostridiales	Eubacteriaceae	Anaerofustis
ASV_3208	Bacteria	Firmicutes	Clostridia	Clostridiales	Ruminococcaceae	Unknown
ASV_3209	Bacteria	Bacteroidetes	Bacteroidia	Bacteroidales	Prevotellaceae	Prevotellaceae_NK3B31_group
ASV_3210	Bacteria	Firmicutes	Clostridia	Clostridiales	Unknown	
ASV_3211	Bacteria	Proteobacteria	Gammaproteobacteria	Xanthomonadales	Xanthomonadaceae	Stenotrophomonas
ASV_3212	Bacteria	Firmicutes	Clostridia	Clostridiales	Lachnospiraceae	Unknown
ASV_3213	Bacteria	Firmicutes	Erysipelotrichia	Erysipelotrichales	Erysipelotrichaceae	Faecalibaculum
ASV_3214	Bacteria	Firmicutes	Unknown			
ASV_3215	Bacteria	Bacteroidetes	Bacteroidia	Bacteroidales	Muribaculaceae	Unknown
ASV_3216	Bacteria	Tenericutes	Mollicutes	Mollicutes_RF39	Unknown	
ASV_3217	Bacteria	Firmicutes	Clostridia	Clostridiales	Lachnospiraceae	Unknown
ASV_3218	Bacteria	Bacteroidetes	Bacteroidia	Bacteroidales	Prevotellaceae	Prevotellaceae_UCG-001
ASV_3219	Bacteria	Bacteroidetes	Bacteroidia	Bacteroidales	Prevotellaceae	Prevotellaceae_UCG-001
ASV_3220	Bacteria	Firmicutes	Bacilli	Lactobacillales	Lactobacillaceae	Lactobacillus
ASV_3221	Bacteria	Firmicutes	Clostridia	Clostridiales	Unknown	
ASV_3222	Bacteria	Firmicutes	Clostridia	Clostridiales	Peptococcaceae	Dehalobacterium
ASV_3223	Bacteria	Firmicutes	Clostridia	Clostridiales	Ruminococcaceae	UBA1819
ASV_3224	Bacteria	Firmicutes	Unknown			
ASV_3225	Bacteria	Firmicutes	Clostridia	Clostridiales	Unknown	
ASV_3226	Bacteria	Firmicutes	Clostridia	Clostridiales	Lachnospiraceae	A2
ASV_3227	Bacteria	Bacteroidetes	Bacteroidia	Bacteroidales	Muribaculaceae	Unknown
ASV_3228	Bacteria	Firmicutes	Clostridia	Clostridiales	Ruminococcaceae	Ruminiclostridium
ASV_3229	Bacteria	Bacteroidetes	Bacteroidia	Bacteroidales	Muribaculaceae	Unknown
ASV_3230	Bacteria	Bacteroidetes	Bacteroidia	Bacteroidales	Muribaculaceae	Unknown
ASV_3231	Bacteria	Tenericutes	Mollicutes	Mollicutes_RF39	Unknown	
ASV_3232	Bacteria	Tenericutes	Mollicutes	Mollicutes_RF39	Unknown	

ASV_3233	Bacteria	Bacteroidetes	Bacteroidia	Bacteroidales	Muribaculaceae	Unknown
ASV_3234	Bacteria	Bacteroidetes	Bacteroidia	Bacteroidales	Muribaculaceae	Unknown
ASV_3235	Bacteria	Bacteroidetes	Bacteroidia	Bacteroidales	Rikenellaceae	Alistipes
ASV_3236	Bacteria	Actinobacteria	Coriobacteriia	Coriobacteriales	Eggerthellaceae	Adlercreutzia
ASV_3237	Bacteria	Firmicutes	Clostridia	Clostridiales	Ruminococcaceae	Ruminiclostridium_9
ASV_3238	Bacteria	Bacteroidetes	Bacteroidia	Flavobacteriales	Flavobacteriaceae	Capnocytophaga
ASV_3239	Bacteria	Bacteroidetes	Bacteroidia	Bacteroidales	Muribaculaceae	Muribaculum
ASV_3240	Bacteria	Firmicutes	Clostridia	Clostridiales	Lachnospiraceae	Unknown
ASV_3241	Bacteria	Bacteroidetes	Bacteroidia	Bacteroidales	Muribaculaceae	Unknown
ASV_3242	Bacteria			Unknown		
ASV_3243	Bacteria	Firmicutes	Clostridia	Clostridiales	Lachnospiraceae	Unknown
ASV_3244	Bacteria	Bacteroidetes	Bacteroidia	Bacteroidales	Rikenellaceae	Alistipes
ASV_3245	Bacteria	Bacteroidetes	Bacteroidia	Bacteroidales	Muribaculaceae	Unknown
ASV_3246	Bacteria	Bacteroidetes	Bacteroidia	Bacteroidales	Prevotellaceae	Prevotellaceae_NK3B31_group
ASV_3247	Bacteria	Firmicutes	Bacilli	Lactobacillales	Lactobacillaceae	Lactobacillus
ASV_3248	Bacteria	Firmicutes	Clostridia	Clostridiales	Lachnospiraceae	Unknown
ASV_3249	Bacteria	Firmicutes	Clostridia	Clostridiales	Lachnospiraceae	Unknown
ASV_3250	Bacteria	Firmicutes	Clostridia	Clostridiales	Lachnospiraceae	Unknown
ASV_3251	Bacteria	Bacteroidetes	Bacteroidia	Bacteroidales	Muribaculaceae	Unknown
ASV_3252	Bacteria	Bacteroidetes	Bacteroidia	Bacteroidales	Muribaculaceae	Unknown
ASV_3253	Bacteria	Bacteroidetes	Bacteroidia	Bacteroidales		Unknown
ASV_3254	Bacteria	Firmicutes	Clostridia	Clostridiales	Lachnospiraceae	Unknown
ASV_3255	Bacteria	Firmicutes	Clostridia	Clostridiales	Lachnospiraceae	Lachnospiraceae_UCG-006
ASV_3256	Bacteria	Firmicutes	Bacilli	Lactobacillales	Lactobacillaceae	Lactobacillus
ASV_3257	Bacteria	Firmicutes	Clostridia	Clostridiales	Lachnospiraceae	Lachnospiraceae_NK4A136_group
ASV_3258	Bacteria	Tenericutes	Mollicutes	Mollicutes_RF39	Unknown	
ASV_3259	Bacteria	Firmicutes	Bacilli	Lactobacillales	Lactobacillaceae	Lactobacillus
ASV_3260	Bacteria	Actinobacteria	Coriobacteriia	Coriobacteriales	Eggerthellaceae	Denitrobacterium
ASV_3261	Bacteria	Firmicutes	Clostridia	Clostridiales	Clostridiales_vadinBB60_group	Unknown
ASV_3262	Bacteria	Firmicutes	Clostridia	Clostridiales	Ruminococcaceae	Ruminococcus_1
ASV_3263	Bacteria	Firmicutes	Clostridia	Clostridiales	Lachnospiraceae	Lachnospiraceae_NK4A136_group
ASV_3264	Bacteria	Bacteroidetes	Bacteroidia	Bacteroidales	Rikenellaceae	Alistipes
ASV_3265	Bacteria	Bacteroidetes	Bacteroidia	Bacteroidales	Muribaculaceae	Unknown
ASV_3266	Bacteria	Firmicutes	Clostridia	Clostridiales	Lachnospiraceae	Lachnospiraceae_UCG-001
ASV_3267	Bacteria	Bacteroidetes	Bacteroidia	Bacteroidales	Rikenellaceae	Alistipes
ASV_3268	Bacteria	Firmicutes	Clostridia	Clostridiales	Ruminococcaceae	Unknown
ASV_3269	Bacteria	Bacteroidetes	Bacteroidia	Bacteroidales	Muribaculaceae	Unknown

ASV_3270	Bacteria	Bacteroidetes	Bacteroidia	Bacteroidales	Muribaculaceae	Unknown
ASV_3271	Bacteria	Firmicutes	Clostridia	Clostridiales	Lachnospiraceae	ASF356
ASV_3272	Bacteria	Firmicutes	Clostridia	Clostridiales	Lachnospiraceae	Lachnospiraceae_NK4A136_group
ASV_3273	Bacteria	Bacteroidetes	Bacteroidia	Bacteroidales	Rikenellaceae	Alistipes
ASV_3274	Bacteria	Bacteroidetes	Bacteroidia	Bacteroidales	Rikenellaceae	Alistipes
ASV_3275	Bacteria	Bacteroidetes	Bacteroidia	Bacteroidales	Muribaculaceae	Unknown
ASV_3276	Bacteria	Tenericutes	Mollicutes	Mollicutes_RF39	Unknown	
ASV_3277	Bacteria	Firmicutes	Clostridia	Clostridiales	Lachnospiraceae	Lachnospiraceae_NK4A136_group
ASV_3278	Bacteria	Bacteroidetes	Bacteroidia	Unknown		
ASV_3279	Bacteria	Firmicutes	Clostridia	Clostridiales	Ruminococcaceae	Ruminococcaceae_UCG-014
ASV_3280	Bacteria	Bacteroidetes	Bacteroidia	Bacteroidales	Muribaculaceae	Unknown
ASV_3281	Bacteria	Firmicutes	Clostridia	Clostridiales	Lachnospiraceae	ASF356
ASV_3282	Bacteria	Firmicutes	Clostridia	Clostridiales	Ruminococcaceae	Unknown
ASV_3283	Bacteria	Firmicutes	Clostridia	Clostridiales	Lachnospiraceae	Unknown
ASV_3284	Bacteria	Firmicutes	Bacilli	Lactobacillales	Unknown	
ASV_3285	Bacteria	Firmicutes	Clostridia	Clostridiales	Ruminococcaceae	Unknown
ASV_3286	Bacteria	Firmicutes	Clostridia	Clostridiales	Lachnospiraceae	Lachnospiraceae_NK4A136_group
ASV_3287	Bacteria	Cyanobacteria	Melainabacteria	Gastranaerophilales	Unknown	
ASV_3288	Bacteria	Firmicutes	Clostridia	Clostridiales	Peptococcaceae	Unknown
ASV_3289	Bacteria	Firmicutes	Clostridia	Clostridiales	Lachnospiraceae	Lachnospiraceae_NK4A136_group
ASV_3290	Bacteria	Firmicutes	Unknown			
ASV_3291	Bacteria	Firmicutes	Clostridia	Clostridiales	Lachnospiraceae	Unknown
ASV_3292	Bacteria	Bacteroidetes	Bacteroidia	Bacteroidales	Rikenellaceae	Alistipes
ASV_3293	Bacteria	Bacteroidetes	Bacteroidia	Bacteroidales	Muribaculaceae	Unknown
ASV_3294	Bacteria	Proteobacteria	Gammaproteobacteria	Enterobacteriales	Enterobacteriaceae	Unknown
ASV_3295	Bacteria	Bacteroidetes	Bacteroidia	Bacteroidales	Unknown	
ASV_3296	Bacteria	Bacteroidetes	Bacteroidia	Bacteroidales	Prevotellaceae	Prevotellaceae_NK3B31_group
ASV_3297	Bacteria	Firmicutes	Clostridia	Clostridiales	Lachnospiraceae	Unknown
ASV_3298	Bacteria	Firmicutes	Clostridia	Clostridiales	Lachnospiraceae	Lachnospiraceae_NK4A136_group
ASV_3299	Bacteria	Firmicutes	Clostridia	Clostridiales	Ruminococcaceae	Unknown
ASV_3300	Bacteria	Firmicutes	Clostridia	Clostridiales	Unknown	
ASV_3301	Bacteria	Bacteroidetes	Bacteroidia	Bacteroidales	Muribaculaceae	Unknown
ASV_3302	Bacteria	Firmicutes	Bacilli	Lactobacillales	Lactobacillaceae	Lactobacillus
ASV_3303	Bacteria	Firmicutes	Clostridia	Clostridiales	Ruminococcaceae	Ruminiclostridium_9
ASV_3304	Bacteria	Bacteroidetes	Bacteroidia	Bacteroidales	Muribaculaceae	Unknown
ASV_3305	Bacteria	Firmicutes	Clostridia	Clostridiales	Ruminococcaceae	Unknown
ASV_3306	Bacteria	Bacteroidetes	Bacteroidia	Bacteroidales	Bacteroidaceae	Bacteroides

ASV_3307	Bacteria	Bacteroidetes	Bacteroidia	Bacteroidales		Unknown
ASV_3308	Bacteria	Firmicutes	Clostridia	Clostridiales	Clostridiaceae_1	Unknown
ASV_3309	Bacteria	Firmicutes	Clostridia	Clostridiales	Lachnospiraceae	Lachnospiraceae_NK4A136_group
ASV_3310	Bacteria	Firmicutes	Clostridia	Clostridiales	Lachnospiraceae	Unknown
ASV_3311	Bacteria	Bacteroidetes	Bacteroidia	Bacteroidales	Muribaculaceae	Unknown
ASV_3312	Bacteria	Firmicutes	Clostridia	Clostridiales	Ruminococcaceae	Unknown
ASV_3313	Bacteria	Firmicutes	Clostridia	Clostridiales	Clostridiales_vadinBB60_group	Unknown
ASV_3314	Bacteria	Bacteroidetes	Bacteroidia		Unknown	
ASV_3315	Bacteria	Bacteroidetes	Bacteroidia	Bacteroidales	Muribaculaceae	Unknown
ASV_3316	Bacteria			Unknown		
ASV_3317	Bacteria	Firmicutes	Clostridia	Clostridiales	Ruminococcaceae	Ruminococcaceae_UCG-014
ASV_3318	Bacteria	Bacteroidetes	Bacteroidia	Bacteroidales	Prevotellaceae	Prevotellaceae_UCG-001
ASV_3319	Bacteria	Firmicutes	Clostridia	Clostridiales	Lachnospiraceae	Unknown
ASV_3320	Bacteria	Firmicutes	Clostridia	Clostridiales	Lachnospiraceae	Unknown
ASV_3321	Bacteria	Bacteroidetes	Bacteroidia	Bacteroidales	Muribaculaceae	Unknown
ASV_3322	Bacteria	Bacteroidetes	Bacteroidia	Bacteroidales	Muribaculaceae	Unknown
ASV_3323	Bacteria	Firmicutes	Clostridia	Clostridiales	Lachnospiraceae	Unknown
ASV_3324	Bacteria	Firmicutes	Clostridia	Clostridiales	Lachnospiraceae	Unknown
ASV_3325	Bacteria	Firmicutes	Clostridia	Clostridiales	Ruminococcaceae	Unknown
ASV_3326	Bacteria	Firmicutes	Clostridia	Clostridiales	Lachnospiraceae	Unknown
ASV_3327	Bacteria	Bacteroidetes	Bacteroidia	Bacteroidales	Muribaculaceae	Unknown
ASV_3328	Bacteria	Firmicutes	Clostridia	Clostridiales	Lachnospiraceae	Lachnospiraceae_NK4A136_group
ASV_3329	Bacteria	Firmicutes	Clostridia	Clostridiales	Lachnospiraceae	Unknown
ASV_3330	Bacteria	Firmicutes	Clostridia	Clostridiales	Lachnospiraceae	Lachnospiraceae_UCG-001
ASV_3331	Bacteria	Firmicutes	Clostridia	Clostridiales	Ruminococcaceae	Ruminiclostridium_9
ASV_3332	Bacteria	Firmicutes	Clostridia	Clostridiales	Lachnospiraceae	ASF356
ASV_3333	Bacteria	Bacteroidetes	Bacteroidia	Bacteroidales	Muribaculaceae	Muribaculum
ASV_3334	Bacteria	Firmicutes	Clostridia	Clostridiales	Ruminococcaceae	Unknown
ASV_3335	Bacteria	Firmicutes	Clostridia	Clostridiales	Ruminococcaceae	Ruminiclostridium_9
ASV_3336	Bacteria	Firmicutes	Clostridia	Clostridiales	Ruminococcaceae	Ruminiclostridium_9
ASV_3337	Bacteria	Bacteroidetes	Bacteroidia	Bacteroidales		Unknown
ASV_3338	Bacteria	Firmicutes	Bacilli	Lactobacillales	Unknown	
ASV_3339	Bacteria	Bacteroidetes	Bacteroidia	Bacteroidales		Unknown
ASV_3340	Bacteria	Firmicutes	Clostridia	Clostridiales		Unknown
ASV_3341	Bacteria	Firmicutes	Clostridia	Clostridiales	Ruminococcaceae	Unknown
ASV_3342	Bacteria	Firmicutes	Clostridia	Clostridiales	Lachnospiraceae	Unknown
ASV_3343	Bacteria	Bacteroidetes	Bacteroidia	Bacteroidales	Muribaculaceae	Unknown

ASV_3344	Bacteria	Bacteroidetes	Bacteroidia	Bacteroidales	Muribaculaceae	Unknown
ASV_3345	Bacteria			Unknown		
ASV_3346	Bacteria	Firmicutes	Clostridia	Clostridiales	Lachnospiraceae	Unknown
ASV_3347	Bacteria	Firmicutes	Clostridia	Clostridiales	Peptococcaceae	Unknown
ASV_3348	Bacteria	Firmicutes	Erysipelotrichia	Erysipelotrichales	Erysipelotrichaceae	Faecalibaculum
ASV_3349	Bacteria	Firmicutes	Erysipelotrichia	Erysipelotrichales	Erysipelotrichaceae	Faecalibaculum
ASV_3350	Bacteria	Firmicutes	Clostridia	Clostridiales	Ruminococcaceae	Ruminiclostridium_9
ASV_3351	Bacteria	Firmicutes	Clostridia	Clostridiales	Lachnospiraceae	Unknown
ASV_3352	Bacteria	Firmicutes	Clostridia	Clostridiales	Lachnospiraceae	Unknown
ASV_3353	Bacteria	Firmicutes	Clostridia	Clostridiales	Lachnospiraceae	Lachnospiraceae_NK4A136_group

Supplemental Table 2

Format: tab delmited text

SampleID ASV_1 ASV_2 ASV_3 ASV_4 ASV_5 ASV_6 ASV_7 ASV_8 ASV_9 ASV_10 ASV_11 ASV_12 ASV_13 ASV_14 ASV_15 ASV_16 ASV_17
ASV_18 ASV_19 ASV_20 ASV_21 ASV_22 ASV_23 ASV_24 ASV_25 ASV_26 ASV_27 ASV_28 ASV_29 ASV_30 ASV_31 ASV_32 ASV_33 ASV_34 ASV_35
ASV_36 ASV_37 ASV_38 ASV_39 ASV_40 ASV_41 ASV_42 ASV_43 ASV_44 ASV_45 ASV_46 ASV_47 ASV_48 ASV_49 ASV_50 ASV_51 ASV_52 ASV_53
ASV_54 ASV_55 ASV_56 ASV_57 ASV_58 ASV_59 ASV_60 ASV_61 ASV_62 ASV_63 ASV_64 ASV_65 ASV_66 ASV_67 ASV_68 ASV_69 ASV_70 ASV_71
ASV_72 ASV_73 ASV_74 ASV_75 ASV_76 ASV_77 ASV_78 ASV_79 ASV_80 ASV_81 ASV_82 ASV_83 ASV_84 ASV_85 ASV_86 ASV_87 ASV_88 ASV_89
ASV_90 ASV_91 ASV_92 ASV_93 ASV_94 ASV_95 ASV_96 ASV_97 ASV_98 ASV_99 ASV_100 ASV_101 ASV_102 ASV_103
ASV_104 ASV_105 ASV_106 ASV_107 ASV_108 ASV_109 ASV_110 ASV_111 ASV_112
ASV_113 ASV_114 ASV_115 ASV_116 ASV_117 ASV_118 ASV_119 ASV_120 ASV_121
ASV_122 ASV_123 ASV_124 ASV_125 ASV_126 ASV_127 ASV_128 ASV_129 ASV_130
ASV_131 ASV_132 ASV_133 ASV_134 ASV_135 ASV_136 ASV_137 ASV_138 ASV_139
ASV_140 ASV_141 ASV_142 ASV_143 ASV_144 ASV_145 ASV_146 ASV_147 ASV_148
ASV_149 ASV_150 ASV_151 ASV_152 ASV_153 ASV_154 ASV_155 ASV_156 ASV_157
ASV_158 ASV_159 ASV_160 ASV_161 ASV_162 ASV_163 ASV_164 ASV_165 ASV_166
ASV_167 ASV_168 ASV_169 ASV_170 ASV_171 ASV_172 ASV_173 ASV_174 ASV_175
ASV_176 ASV_177 ASV_178 ASV_179 ASV_180 ASV_181 ASV_182 ASV_183 ASV_184
ASV_185 ASV_186 ASV_187 ASV_188 ASV_189 ASV_190 ASV_191 ASV_192 ASV_193
ASV_194 ASV_195 ASV_196 ASV_197 ASV_198 ASV_199 ASV_200 ASV_201 ASV_202
ASV_203 ASV_204 ASV_205 ASV_206 ASV_207 ASV_208 ASV_209 ASV_210 ASV_211
ASV_212 ASV_213 ASV_214 ASV_215 ASV_216 ASV_217 ASV_218 ASV_219 ASV_220
ASV_221 ASV_222 ASV_223 ASV_224 ASV_225 ASV_226 ASV_227 ASV_228 ASV_229
ASV_230 ASV_231 ASV_232 ASV_233 ASV_234 ASV_235 ASV_236 ASV_237 ASV_238
ASV_239 ASV_240 ASV_241 ASV_242 ASV_243 ASV_244 ASV_245 ASV_246 ASV_247
ASV_248 ASV_249 ASV_250 ASV_251 ASV_252 ASV_253 ASV_254 ASV_255 ASV_256
ASV_257 ASV_258 ASV_259 ASV_260 ASV_261 ASV_262 ASV_263 ASV_264 ASV_265
ASV_266 ASV_267 ASV_268 ASV_269 ASV_270 ASV_271 ASV_272 ASV_273 ASV_274
ASV_275 ASV_276 ASV_277 ASV_278 ASV_279 ASV_280 ASV_281 ASV_282 ASV_283
ASV_284 ASV_285 ASV_286 ASV_287 ASV_288 ASV_289 ASV_290 ASV_291 ASV_292
ASV_293 ASV_294 ASV_295 ASV_296 ASV_297 ASV_298 ASV_299 ASV_300 ASV_301
ASV_302 ASV_303 ASV_304 ASV_305 ASV_306 ASV_307 ASV_308 ASV_309 ASV_310
ASV_311 ASV_312 ASV_313 ASV_314 ASV_315 ASV_316 ASV_317 ASV_318 ASV_319

ASV_320	ASV_321	ASV_322	ASV_323	ASV_324	ASV_325	ASV_326	ASV_327	ASV_328
ASV_329	ASV_330	ASV_331	ASV_332	ASV_333	ASV_334	ASV_335	ASV_336	ASV_337
ASV_338	ASV_339	ASV_340	ASV_341	ASV_342	ASV_343	ASV_344	ASV_345	ASV_346
ASV_347	ASV_348	ASV_349	ASV_350	ASV_351	ASV_352	ASV_353	ASV_354	ASV_355
ASV_356	ASV_357	ASV_358	ASV_359	ASV_360	ASV_361	ASV_362	ASV_363	ASV_364
ASV_365	ASV_366	ASV_367	ASV_368	ASV_369	ASV_370	ASV_371	ASV_372	ASV_373
ASV_374	ASV_375	ASV_376	ASV_377	ASV_378	ASV_379	ASV_380	ASV_381	ASV_382
ASV_383	ASV_384	ASV_385	ASV_386	ASV_387	ASV_388	ASV_389	ASV_390	ASV_391
ASV_392	ASV_393	ASV_394	ASV_395	ASV_396	ASV_397	ASV_398	ASV_399	ASV_400
ASV_401	ASV_402	ASV_403	ASV_404	ASV_405	ASV_406	ASV_407	ASV_408	ASV_409
ASV_410	ASV_411	ASV_412	ASV_413	ASV_414	ASV_415	ASV_416	ASV_417	ASV_418
ASV_419	ASV_420	ASV_421	ASV_422	ASV_423	ASV_424	ASV_425	ASV_426	ASV_427
ASV_428	ASV_429	ASV_430	ASV_431	ASV_432	ASV_433	ASV_434	ASV_435	ASV_436
ASV_437	ASV_438	ASV_439	ASV_440	ASV_441	ASV_442	ASV_443	ASV_444	ASV_445
ASV_446	ASV_447	ASV_448	ASV_449	ASV_450	ASV_451	ASV_452	ASV_453	ASV_454
ASV_455	ASV_456	ASV_457	ASV_458	ASV_459	ASV_460	ASV_461	ASV_462	ASV_463
ASV_464	ASV_465	ASV_466	ASV_467	ASV_468	ASV_469	ASV_470	ASV_471	ASV_472
ASV_473	ASV_474	ASV_475	ASV_476	ASV_477	ASV_478	ASV_479	ASV_480	ASV_481
ASV_482	ASV_483	ASV_484	ASV_485	ASV_486	ASV_487	ASV_488	ASV_489	ASV_490
ASV_491	ASV_492	ASV_493	ASV_494	ASV_495	ASV_496	ASV_497	ASV_498	ASV_499
ASV_500	ASV_501	ASV_502	ASV_503	ASV_504	ASV_505	ASV_506	ASV_507	ASV_508
ASV_509	ASV_510	ASV_511	ASV_512	ASV_513	ASV_514	ASV_515	ASV_516	ASV_517
ASV_518	ASV_519	ASV_520	ASV_521	ASV_522	ASV_523	ASV_524	ASV_525	ASV_526
ASV_527	ASV_528	ASV_529	ASV_530	ASV_531	ASV_532	ASV_533	ASV_534	ASV_535
ASV_536	ASV_537	ASV_538	ASV_539	ASV_540	ASV_541	ASV_542	ASV_543	ASV_544
ASV_545	ASV_546	ASV_547	ASV_548	ASV_549	ASV_550	ASV_551	ASV_552	ASV_553
ASV_554	ASV_555	ASV_556	ASV_557	ASV_558	ASV_559	ASV_560	ASV_561	ASV_562
ASV_563	ASV_564	ASV_565	ASV_566	ASV_567	ASV_568	ASV_569	ASV_570	ASV_571
ASV_572	ASV_573	ASV_574	ASV_575	ASV_576	ASV_577	ASV_578	ASV_579	ASV_580
ASV_581	ASV_582	ASV_583	ASV_584	ASV_585	ASV_586	ASV_587	ASV_588	ASV_589
ASV_590	ASV_591	ASV_592	ASV_593	ASV_594	ASV_595	ASV_596	ASV_597	ASV_598
ASV_599	ASV_600	ASV_601	ASV_602	ASV_603	ASV_604	ASV_605	ASV_606	ASV_607
ASV_608	ASV_609	ASV_610	ASV_611	ASV_612	ASV_613	ASV_614	ASV_615	ASV_616
ASV_617	ASV_618	ASV_619	ASV_620	ASV_621	ASV_622	ASV_623	ASV_624	ASV_625
ASV_626	ASV_627	ASV_628	ASV_629	ASV_630	ASV_631	ASV_632	ASV_633	ASV_634
ASV_635	ASV_636	ASV_637	ASV_638	ASV_639	ASV_640	ASV_641	ASV_642	ASV_643
ASV_644	ASV_645	ASV_646	ASV_647	ASV_648	ASV_649	ASV_650	ASV_651	ASV_652

ASV_653	ASV_654	ASV_655	ASV_656	ASV_657	ASV_658	ASV_659	ASV_660	ASV_661
ASV_662	ASV_663	ASV_664	ASV_665	ASV_666	ASV_667	ASV_668	ASV_669	ASV_670
ASV_671	ASV_672	ASV_673	ASV_674	ASV_675	ASV_676	ASV_677	ASV_678	ASV_679
ASV_680	ASV_681	ASV_682	ASV_683	ASV_684	ASV_685	ASV_686	ASV_687	ASV_688
ASV_689	ASV_690	ASV_691	ASV_692	ASV_693	ASV_694	ASV_695	ASV_696	ASV_697
ASV_698	ASV_699	ASV_700	ASV_701	ASV_702	ASV_703	ASV_704	ASV_705	ASV_706
ASV_707	ASV_708	ASV_709	ASV_710	ASV_711	ASV_712	ASV_713	ASV_714	ASV_715
ASV_716	ASV_717	ASV_718	ASV_719	ASV_720	ASV_721	ASV_722	ASV_723	ASV_724
ASV_725	ASV_726	ASV_727	ASV_728	ASV_729	ASV_730	ASV_731	ASV_732	ASV_733
ASV_734	ASV_735	ASV_736	ASV_737	ASV_738	ASV_739	ASV_740	ASV_741	ASV_742
ASV_743	ASV_744	ASV_745	ASV_746	ASV_747	ASV_748	ASV_749	ASV_750	ASV_751
ASV_752	ASV_753	ASV_754	ASV_755	ASV_756	ASV_757	ASV_758	ASV_759	ASV_760
ASV_761	ASV_762	ASV_763	ASV_764	ASV_765	ASV_766	ASV_767	ASV_768	ASV_769
ASV_770	ASV_771	ASV_772	ASV_773	ASV_774	ASV_775	ASV_776	ASV_777	ASV_778
ASV_779	ASV_780	ASV_781	ASV_782	ASV_783	ASV_784	ASV_785	ASV_786	ASV_787
ASV_788	ASV_789	ASV_790	ASV_791	ASV_792	ASV_793	ASV_794	ASV_795	ASV_796
ASV_797	ASV_798	ASV_799	ASV_800	ASV_801	ASV_802	ASV_803	ASV_804	ASV_805
ASV_806	ASV_807	ASV_808	ASV_809	ASV_810	ASV_811	ASV_812	ASV_813	ASV_814
ASV_815	ASV_816	ASV_817	ASV_818	ASV_819	ASV_820	ASV_821	ASV_822	ASV_823
ASV_824	ASV_825	ASV_826	ASV_827	ASV_828	ASV_829	ASV_830	ASV_831	ASV_832
ASV_833	ASV_834	ASV_835	ASV_836	ASV_837	ASV_838	ASV_839	ASV_840	ASV_841
ASV_842	ASV_843	ASV_844	ASV_845	ASV_846	ASV_847	ASV_848	ASV_849	ASV_850
ASV_851	ASV_852	ASV_853	ASV_854	ASV_855	ASV_856	ASV_857	ASV_858	ASV_859
ASV_860	ASV_861	ASV_862	ASV_863	ASV_864	ASV_865	ASV_866	ASV_867	ASV_868
ASV_869	ASV_870	ASV_871	ASV_872	ASV_873	ASV_874	ASV_875	ASV_876	ASV_877
ASV_878	ASV_879	ASV_880	ASV_881	ASV_882	ASV_883	ASV_884	ASV_885	ASV_886
ASV_887	ASV_888	ASV_889	ASV_890	ASV_891	ASV_892	ASV_893	ASV_894	ASV_895
ASV_896	ASV_897	ASV_898	ASV_899	ASV_900	ASV_901	ASV_902	ASV_903	ASV_904
ASV_905	ASV_906	ASV_907	ASV_908	ASV_909	ASV_910	ASV_911	ASV_912	ASV_913
ASV_914	ASV_915	ASV_916	ASV_917	ASV_918	ASV_919	ASV_920	ASV_921	ASV_922
ASV_923	ASV_924	ASV_925	ASV_926	ASV_927	ASV_928	ASV_929	ASV_930	ASV_931
ASV_932	ASV_933	ASV_934	ASV_935	ASV_936	ASV_937	ASV_938	ASV_939	ASV_940
ASV_941	ASV_942	ASV_943	ASV_944	ASV_945	ASV_946	ASV_947	ASV_948	ASV_949
ASV_950	ASV_951	ASV_952	ASV_953	ASV_954	ASV_955	ASV_956	ASV_957	ASV_958
ASV_959	ASV_960	ASV_961	ASV_962	ASV_963	ASV_964	ASV_965	ASV_966	ASV_967
ASV_968	ASV_969	ASV_970	ASV_971	ASV_972	ASV_973	ASV_974	ASV_975	ASV_976
ASV_977	ASV_978	ASV_979	ASV_980	ASV_981	ASV_982	ASV_983	ASV_984	ASV_985

ASV_986	ASV_987	ASV_988	ASV_989	ASV_990	ASV_991	ASV_992	ASV_993	ASV_994
ASV_995	ASV_996	ASV_997	ASV_998	ASV_999	ASV_1000	ASV_1001	ASV_1002	ASV_1003
ASV_1004	ASV_1005	ASV_1006	ASV_1007	ASV_1008	ASV_1009	ASV_1010	ASV_1011	ASV_1012
ASV_1013	ASV_1014	ASV_1015	ASV_1016	ASV_1017	ASV_1018	ASV_1019	ASV_1020	ASV_1021
ASV_1022	ASV_1023	ASV_1024	ASV_1025	ASV_1026	ASV_1027	ASV_1028	ASV_1029	ASV_1030
ASV_1031	ASV_1032	ASV_1033	ASV_1034	ASV_1035	ASV_1036	ASV_1037	ASV_1038	ASV_1039
ASV_1040	ASV_1041	ASV_1042	ASV_1043	ASV_1044	ASV_1045	ASV_1046	ASV_1047	ASV_1048
ASV_1049	ASV_1050	ASV_1051	ASV_1052	ASV_1053	ASV_1054	ASV_1055	ASV_1056	ASV_1057
ASV_1058	ASV_1059	ASV_1060	ASV_1061	ASV_1062	ASV_1063	ASV_1064	ASV_1065	ASV_1066
ASV_1067	ASV_1068	ASV_1069	ASV_1070	ASV_1071	ASV_1072	ASV_1073	ASV_1074	ASV_1075
ASV_1076	ASV_1077	ASV_1078	ASV_1079	ASV_1080	ASV_1081	ASV_1082	ASV_1083	ASV_1084
ASV_1085	ASV_1086	ASV_1087	ASV_1088	ASV_1089	ASV_1090	ASV_1091	ASV_1092	ASV_1093
ASV_1094	ASV_1095	ASV_1096	ASV_1097	ASV_1098	ASV_1099	ASV_1100	ASV_1101	ASV_1102
ASV_1103	ASV_1104	ASV_1105	ASV_1106	ASV_1107	ASV_1108	ASV_1109	ASV_1110	ASV_1111
ASV_1112	ASV_1113	ASV_1114	ASV_1115	ASV_1116	ASV_1117	ASV_1118	ASV_1119	ASV_1120
ASV_1121	ASV_1122	ASV_1123	ASV_1124	ASV_1125	ASV_1126	ASV_1127	ASV_1128	ASV_1129
ASV_1130	ASV_1131	ASV_1132	ASV_1133	ASV_1134	ASV_1135	ASV_1136	ASV_1137	ASV_1138
ASV_1139	ASV_1140	ASV_1141	ASV_1142	ASV_1143	ASV_1144	ASV_1145	ASV_1146	ASV_1147
ASV_1148	ASV_1149	ASV_1150	ASV_1151	ASV_1152	ASV_1153	ASV_1154	ASV_1155	ASV_1156
ASV_1157	ASV_1158	ASV_1159	ASV_1160	ASV_1161	ASV_1162	ASV_1163	ASV_1164	ASV_1165
ASV_1166	ASV_1167	ASV_1168	ASV_1169	ASV_1170	ASV_1171	ASV_1172	ASV_1173	ASV_1174
ASV_1175	ASV_1176	ASV_1177	ASV_1178	ASV_1179	ASV_1180	ASV_1181	ASV_1182	ASV_1183
ASV_1184	ASV_1185	ASV_1186	ASV_1187	ASV_1188	ASV_1189	ASV_1190	ASV_1191	ASV_1192
ASV_1193	ASV_1194	ASV_1195	ASV_1196	ASV_1197	ASV_1198	ASV_1199	ASV_1200	ASV_1201
ASV_1202	ASV_1203	ASV_1204	ASV_1205	ASV_1206	ASV_1207	ASV_1208	ASV_1209	ASV_1210
ASV_1211	ASV_1212	ASV_1213	ASV_1214	ASV_1215	ASV_1216	ASV_1217	ASV_1218	ASV_1219
ASV_1220	ASV_1221	ASV_1222	ASV_1223	ASV_1224	ASV_1225	ASV_1226	ASV_1227	ASV_1228
ASV_1229	ASV_1230	ASV_1231	ASV_1232	ASV_1233	ASV_1234	ASV_1235	ASV_1236	ASV_1237
ASV_1238	ASV_1239	ASV_1240	ASV_1241	ASV_1242	ASV_1243	ASV_1244	ASV_1245	ASV_1246
ASV_1247	ASV_1248	ASV_1249	ASV_1250	ASV_1251	ASV_1252	ASV_1253	ASV_1254	ASV_1255
ASV_1256	ASV_1257	ASV_1258	ASV_1259	ASV_1260	ASV_1261	ASV_1262	ASV_1263	ASV_1264
ASV_1265	ASV_1266	ASV_1267	ASV_1268	ASV_1269	ASV_1270	ASV_1271	ASV_1272	ASV_1273
ASV_1274	ASV_1275	ASV_1276	ASV_1277	ASV_1278	ASV_1279	ASV_1280	ASV_1281	ASV_1282
ASV_1283	ASV_1284	ASV_1285	ASV_1286	ASV_1287	ASV_1288	ASV_1289	ASV_1290	ASV_1291
ASV_1292	ASV_1293	ASV_1294	ASV_1295	ASV_1296	ASV_1297	ASV_1298	ASV_1299	ASV_1300
ASV_1301	ASV_1302	ASV_1303	ASV_1304	ASV_1305	ASV_1306	ASV_1307	ASV_1308	ASV_1309
ASV_1310	ASV_1311	ASV_1312	ASV_1313	ASV_1314	ASV_1315	ASV_1316	ASV_1317	ASV_1318

ASV_1319	ASV_1320	ASV_1321	ASV_1322	ASV_1323	ASV_1324	ASV_1325	ASV_1326	ASV_1327
ASV_1328	ASV_1329	ASV_1330	ASV_1331	ASV_1332	ASV_1333	ASV_1334	ASV_1335	ASV_1336
ASV_1337	ASV_1338	ASV_1339	ASV_1340	ASV_1341	ASV_1342	ASV_1343	ASV_1344	ASV_1345
ASV_1346	ASV_1347	ASV_1348	ASV_1349	ASV_1350	ASV_1351	ASV_1352	ASV_1353	ASV_1354
ASV_1355	ASV_1356	ASV_1357	ASV_1358	ASV_1359	ASV_1360	ASV_1361	ASV_1362	ASV_1363
ASV_1364	ASV_1365	ASV_1366	ASV_1367	ASV_1368	ASV_1369	ASV_1370	ASV_1371	ASV_1372
ASV_1373	ASV_1374	ASV_1375	ASV_1376	ASV_1377	ASV_1378	ASV_1379	ASV_1380	ASV_1381
ASV_1382	ASV_1383	ASV_1384	ASV_1385	ASV_1386	ASV_1387	ASV_1388	ASV_1389	ASV_1390
ASV_1391	ASV_1392	ASV_1393	ASV_1394	ASV_1395	ASV_1396	ASV_1397	ASV_1398	ASV_1399
ASV_1400	ASV_1401	ASV_1402	ASV_1403	ASV_1404	ASV_1405	ASV_1406	ASV_1407	ASV_1408
ASV_1409	ASV_1410	ASV_1411	ASV_1412	ASV_1413	ASV_1414	ASV_1415	ASV_1416	ASV_1417
ASV_1418	ASV_1419	ASV_1420	ASV_1421	ASV_1422	ASV_1423	ASV_1424	ASV_1425	ASV_1426
ASV_1427	ASV_1428	ASV_1429	ASV_1430	ASV_1431	ASV_1432	ASV_1433	ASV_1434	ASV_1435
ASV_1436	ASV_1437	ASV_1438	ASV_1439	ASV_1440	ASV_1441	ASV_1442	ASV_1443	ASV_1444
ASV_1445	ASV_1446	ASV_1447	ASV_1448	ASV_1449	ASV_1450	ASV_1451	ASV_1452	ASV_1453
ASV_1454	ASV_1455	ASV_1456	ASV_1457	ASV_1458	ASV_1459	ASV_1460	ASV_1461	ASV_1462
ASV_1463	ASV_1464	ASV_1465	ASV_1466	ASV_1467	ASV_1468	ASV_1469	ASV_1470	ASV_1471
ASV_1472	ASV_1473	ASV_1474	ASV_1475	ASV_1476	ASV_1477	ASV_1478	ASV_1479	ASV_1480
ASV_1481	ASV_1482	ASV_1483	ASV_1484	ASV_1485	ASV_1486	ASV_1487	ASV_1488	ASV_1489
ASV_1490	ASV_1491	ASV_1492	ASV_1493	ASV_1494	ASV_1495	ASV_1496	ASV_1497	ASV_1498
ASV_1499	ASV_1500	ASV_1501	ASV_1502	ASV_1503	ASV_1504	ASV_1505	ASV_1506	ASV_1507
ASV_1508	ASV_1509	ASV_1510	ASV_1511	ASV_1512	ASV_1513	ASV_1514	ASV_1515	ASV_1516
ASV_1517	ASV_1518	ASV_1519	ASV_1520	ASV_1521	ASV_1522	ASV_1523	ASV_1524	ASV_1525
ASV_1526	ASV_1527	ASV_1528	ASV_1529	ASV_1530	ASV_1531	ASV_1532	ASV_1533	ASV_1534
ASV_1535	ASV_1536	ASV_1537	ASV_1538	ASV_1539	ASV_1540	ASV_1541	ASV_1542	ASV_1543
ASV_1544	ASV_1545	ASV_1546	ASV_1547	ASV_1548	ASV_1549	ASV_1550	ASV_1551	ASV_1552
ASV_1553	ASV_1554	ASV_1555	ASV_1556	ASV_1557	ASV_1558	ASV_1559	ASV_1560	ASV_1561
ASV_1562	ASV_1563	ASV_1564	ASV_1565	ASV_1566	ASV_1567	ASV_1568	ASV_1569	ASV_1570
ASV_1571	ASV_1572	ASV_1573	ASV_1574	ASV_1575	ASV_1576	ASV_1577	ASV_1578	ASV_1579
ASV_1580	ASV_1581	ASV_1582	ASV_1583	ASV_1584	ASV_1585	ASV_1586	ASV_1587	ASV_1588
ASV_1589	ASV_1590	ASV_1591	ASV_1592	ASV_1593	ASV_1594	ASV_1595	ASV_1596	ASV_1597
ASV_1598	ASV_1599	ASV_1600	ASV_1601	ASV_1602	ASV_1603	ASV_1604	ASV_1605	ASV_1606
ASV_1607	ASV_1608	ASV_1609	ASV_1610	ASV_1611	ASV_1612	ASV_1613	ASV_1614	ASV_1615
ASV_1616	ASV_1617	ASV_1618	ASV_1619	ASV_1620	ASV_1621	ASV_1622	ASV_1623	ASV_1624
ASV_1625	ASV_1626	ASV_1627	ASV_1628	ASV_1629	ASV_1630	ASV_1631	ASV_1632	ASV_1633
ASV_1634	ASV_1635	ASV_1636	ASV_1637	ASV_1638	ASV_1639	ASV_1640	ASV_1641	ASV_1642
ASV_1643	ASV_1644	ASV_1645	ASV_1646	ASV_1647	ASV_1648	ASV_1649	ASV_1650	ASV_1651

ASV_1652	ASV_1653	ASV_1654	ASV_1655	ASV_1656	ASV_1657	ASV_1658	ASV_1659	ASV_1660
ASV_1661	ASV_1662	ASV_1663	ASV_1664	ASV_1665	ASV_1666	ASV_1667	ASV_1668	ASV_1669
ASV_1670	ASV_1671	ASV_1672	ASV_1673	ASV_1674	ASV_1675	ASV_1676	ASV_1677	ASV_1678
ASV_1679	ASV_1680	ASV_1681	ASV_1682	ASV_1683	ASV_1684	ASV_1685	ASV_1686	ASV_1687
ASV_1688	ASV_1689	ASV_1690	ASV_1691	ASV_1692	ASV_1693	ASV_1694	ASV_1695	ASV_1696
ASV_1697	ASV_1698	ASV_1699	ASV_1700	ASV_1701	ASV_1702	ASV_1703	ASV_1704	ASV_1705
ASV_1706	ASV_1707	ASV_1708	ASV_1709	ASV_1710	ASV_1711	ASV_1712	ASV_1713	ASV_1714
ASV_1715	ASV_1716	ASV_1717	ASV_1718	ASV_1719	ASV_1720	ASV_1721	ASV_1722	ASV_1723
ASV_1724	ASV_1725	ASV_1726	ASV_1727	ASV_1728	ASV_1729	ASV_1730	ASV_1731	ASV_1732
ASV_1733	ASV_1734	ASV_1735	ASV_1736	ASV_1737	ASV_1738	ASV_1739	ASV_1740	ASV_1741
ASV_1742	ASV_1743	ASV_1744	ASV_1745	ASV_1746	ASV_1747	ASV_1748	ASV_1749	ASV_1750
ASV_1751	ASV_1752	ASV_1753	ASV_1754	ASV_1755	ASV_1756	ASV_1757	ASV_1758	ASV_1759
ASV_1760	ASV_1761	ASV_1762	ASV_1763	ASV_1764	ASV_1765	ASV_1766	ASV_1767	ASV_1768
ASV_1769	ASV_1770	ASV_1771	ASV_1772	ASV_1773	ASV_1774	ASV_1775	ASV_1776	ASV_1777
ASV_1778	ASV_1779	ASV_1780	ASV_1781	ASV_1782	ASV_1783	ASV_1784	ASV_1785	ASV_1786
ASV_1787	ASV_1788	ASV_1789	ASV_1790	ASV_1791	ASV_1792	ASV_1793	ASV_1794	ASV_1795
ASV_1796	ASV_1797	ASV_1798	ASV_1799	ASV_1800	ASV_1801	ASV_1802	ASV_1803	ASV_1804
ASV_1805	ASV_1806	ASV_1807	ASV_1808	ASV_1809	ASV_1810	ASV_1811	ASV_1812	ASV_1813
ASV_1814	ASV_1815	ASV_1816	ASV_1817	ASV_1818	ASV_1819	ASV_1820	ASV_1821	ASV_1822
ASV_1823	ASV_1824	ASV_1825	ASV_1826	ASV_1827	ASV_1828	ASV_1829	ASV_1830	ASV_1831
ASV_1832	ASV_1833	ASV_1834	ASV_1835	ASV_1836	ASV_1837	ASV_1838	ASV_1839	ASV_1840
ASV_1841	ASV_1842	ASV_1843	ASV_1844	ASV_1845	ASV_1846	ASV_1847	ASV_1848	ASV_1849
ASV_1850	ASV_1851	ASV_1852	ASV_1853	ASV_1854	ASV_1855	ASV_1856	ASV_1857	ASV_1858
ASV_1859	ASV_1860	ASV_1861	ASV_1862	ASV_1863	ASV_1864	ASV_1865	ASV_1866	ASV_1867
ASV_1868	ASV_1869	ASV_1870	ASV_1871	ASV_1872	ASV_1873	ASV_1874	ASV_1875	ASV_1876
ASV_1877	ASV_1878	ASV_1879	ASV_1880	ASV_1881	ASV_1882	ASV_1883	ASV_1884	ASV_1885
ASV_1886	ASV_1887	ASV_1888	ASV_1889	ASV_1890	ASV_1891	ASV_1892	ASV_1893	ASV_1894
ASV_1895	ASV_1896	ASV_1897	ASV_1898	ASV_1899	ASV_1900	ASV_1901	ASV_1902	ASV_1903
ASV_1904	ASV_1905	ASV_1906	ASV_1907	ASV_1908	ASV_1909	ASV_1910	ASV_1911	ASV_1912
ASV_1913	ASV_1914	ASV_1915	ASV_1916	ASV_1917	ASV_1918	ASV_1919	ASV_1920	ASV_1921
ASV_1922	ASV_1923	ASV_1924	ASV_1925	ASV_1926	ASV_1927	ASV_1928	ASV_1929	ASV_1930
ASV_1931	ASV_1932	ASV_1933	ASV_1934	ASV_1935	ASV_1936	ASV_1937	ASV_1938	ASV_1939
ASV_1940	ASV_1941	ASV_1942	ASV_1943	ASV_1944	ASV_1945	ASV_1946	ASV_1947	ASV_1948
ASV_1949	ASV_1950	ASV_1951	ASV_1952	ASV_1953	ASV_1954	ASV_1955	ASV_1956	ASV_1957
ASV_1958	ASV_1959	ASV_1960	ASV_1961	ASV_1962	ASV_1963	ASV_1964	ASV_1965	ASV_1966
ASV_1967	ASV_1968	ASV_1969	ASV_1970	ASV_1971	ASV_1972	ASV_1973	ASV_1974	ASV_1975
ASV_1976	ASV_1977	ASV_1978	ASV_1979	ASV_1980	ASV_1981	ASV_1982	ASV_1983	ASV_1984

ASV_1985	ASV_1986	ASV_1987	ASV_1988	ASV_1989	ASV_1990	ASV_1991	ASV_1992	ASV_1993
ASV_1994	ASV_1995	ASV_1996	ASV_1997	ASV_1998	ASV_1999	ASV_2000	ASV_2001	ASV_2002
ASV_2003	ASV_2004	ASV_2005	ASV_2006	ASV_2007	ASV_2008	ASV_2009	ASV_2010	ASV_2011
ASV_2012	ASV_2013	ASV_2014	ASV_2015	ASV_2016	ASV_2017	ASV_2018	ASV_2019	ASV_2020
ASV_2021	ASV_2022	ASV_2023	ASV_2024	ASV_2025	ASV_2026	ASV_2027	ASV_2028	ASV_2029
ASV_2030	ASV_2031	ASV_2032	ASV_2033	ASV_2034	ASV_2035	ASV_2036	ASV_2037	ASV_2038
ASV_2039	ASV_2040	ASV_2041	ASV_2042	ASV_2043	ASV_2044	ASV_2045	ASV_2046	ASV_2047
ASV_2048	ASV_2049	ASV_2050	ASV_2051	ASV_2052	ASV_2053	ASV_2054	ASV_2055	ASV_2056
ASV_2057	ASV_2058	ASV_2059	ASV_2060	ASV_2061	ASV_2062	ASV_2063	ASV_2064	ASV_2065
ASV_2066	ASV_2067	ASV_2068	ASV_2069	ASV_2070	ASV_2071	ASV_2072	ASV_2073	ASV_2074
ASV_2075	ASV_2076	ASV_2077	ASV_2078	ASV_2079	ASV_2080	ASV_2081	ASV_2082	ASV_2083
ASV_2084	ASV_2085	ASV_2086	ASV_2087	ASV_2088	ASV_2089	ASV_2090	ASV_2091	ASV_2092
ASV_2093	ASV_2094	ASV_2095	ASV_2096	ASV_2097	ASV_2098	ASV_2099	ASV_2100	ASV_2101
ASV_2102	ASV_2103	ASV_2104	ASV_2105	ASV_2106	ASV_2107	ASV_2108	ASV_2109	ASV_2110
ASV_2111	ASV_2112	ASV_2113	ASV_2114	ASV_2115	ASV_2116	ASV_2117	ASV_2118	ASV_2119
ASV_2120	ASV_2121	ASV_2122	ASV_2123	ASV_2124	ASV_2125	ASV_2126	ASV_2127	ASV_2128
ASV_2129	ASV_2130	ASV_2131	ASV_2132	ASV_2133	ASV_2134	ASV_2135	ASV_2136	ASV_2137
ASV_2138	ASV_2139	ASV_2140	ASV_2141	ASV_2142	ASV_2143	ASV_2144	ASV_2145	ASV_2146
ASV_2147	ASV_2148	ASV_2149	ASV_2150	ASV_2151	ASV_2152	ASV_2153	ASV_2154	ASV_2155
ASV_2156	ASV_2157	ASV_2158	ASV_2159	ASV_2160	ASV_2161	ASV_2162	ASV_2163	ASV_2164
ASV_2165	ASV_2166	ASV_2167	ASV_2168	ASV_2169	ASV_2170	ASV_2171	ASV_2172	ASV_2173
ASV_2174	ASV_2175	ASV_2176	ASV_2177	ASV_2178	ASV_2179	ASV_2180	ASV_2181	ASV_2182
ASV_2183	ASV_2184	ASV_2185	ASV_2186	ASV_2187	ASV_2188	ASV_2189	ASV_2190	ASV_2191
ASV_2192	ASV_2193	ASV_2194	ASV_2195	ASV_2196	ASV_2197	ASV_2198	ASV_2199	ASV_2200
ASV_2201	ASV_2202	ASV_2203	ASV_2204	ASV_2205	ASV_2206	ASV_2207	ASV_2208	ASV_2209
ASV_2210	ASV_2211	ASV_2212	ASV_2213	ASV_2214	ASV_2215	ASV_2216	ASV_2217	ASV_2218
ASV_2219	ASV_2220	ASV_2221	ASV_2222	ASV_2223	ASV_2224	ASV_2225	ASV_2226	ASV_2227
ASV_2228	ASV_2229	ASV_2230	ASV_2231	ASV_2232	ASV_2233	ASV_2234	ASV_2235	ASV_2236
ASV_2237	ASV_2238	ASV_2239	ASV_2240	ASV_2241	ASV_2242	ASV_2243	ASV_2244	ASV_2245
ASV_2246	ASV_2247	ASV_2248	ASV_2249	ASV_2250	ASV_2251	ASV_2252	ASV_2253	ASV_2254
ASV_2255	ASV_2256	ASV_2257	ASV_2258	ASV_2259	ASV_2260	ASV_2261	ASV_2262	ASV_2263
ASV_2264	ASV_2265	ASV_2266	ASV_2267	ASV_2268	ASV_2269	ASV_2270	ASV_2271	ASV_2272
ASV_2273	ASV_2274	ASV_2275	ASV_2276	ASV_2277	ASV_2278	ASV_2279	ASV_2280	ASV_2281
ASV_2282	ASV_2283	ASV_2284	ASV_2285	ASV_2286	ASV_2287	ASV_2288	ASV_2289	ASV_2290
ASV_2291	ASV_2292	ASV_2293	ASV_2294	ASV_2295	ASV_2296	ASV_2297	ASV_2298	ASV_2299
ASV_2300	ASV_2301	ASV_2302	ASV_2303	ASV_2304	ASV_2305	ASV_2306	ASV_2307	ASV_2308
ASV_2309	ASV_2310	ASV_2311	ASV_2312	ASV_2313	ASV_2314	ASV_2315	ASV_2316	ASV_2317

ASV_2318	ASV_2319	ASV_2320	ASV_2321	ASV_2322	ASV_2323	ASV_2324	ASV_2325	ASV_2326
ASV_2327	ASV_2328	ASV_2329	ASV_2330	ASV_2331	ASV_2332	ASV_2333	ASV_2334	ASV_2335
ASV_2336	ASV_2337	ASV_2338	ASV_2339	ASV_2340	ASV_2341	ASV_2342	ASV_2343	ASV_2344
ASV_2345	ASV_2346	ASV_2347	ASV_2348	ASV_2349	ASV_2350	ASV_2351	ASV_2352	ASV_2353
ASV_2354	ASV_2355	ASV_2356	ASV_2357	ASV_2358	ASV_2359	ASV_2360	ASV_2361	ASV_2362
ASV_2363	ASV_2364	ASV_2365	ASV_2366	ASV_2367	ASV_2368	ASV_2369	ASV_2370	ASV_2371
ASV_2372	ASV_2373	ASV_2374	ASV_2375	ASV_2376	ASV_2377	ASV_2378	ASV_2379	ASV_2380
ASV_2381	ASV_2382	ASV_2383	ASV_2384	ASV_2385	ASV_2386	ASV_2387	ASV_2388	ASV_2389
ASV_2390	ASV_2391	ASV_2392	ASV_2393	ASV_2394	ASV_2395	ASV_2396	ASV_2397	ASV_2398
ASV_2399	ASV_2400	ASV_2401	ASV_2402	ASV_2403	ASV_2404	ASV_2405	ASV_2406	ASV_2407
ASV_2408	ASV_2409	ASV_2410	ASV_2411	ASV_2412	ASV_2413	ASV_2414	ASV_2415	ASV_2416
ASV_2417	ASV_2418	ASV_2419	ASV_2420	ASV_2421	ASV_2422	ASV_2423	ASV_2424	ASV_2425
ASV_2426	ASV_2427	ASV_2428	ASV_2429	ASV_2430	ASV_2431	ASV_2432	ASV_2433	ASV_2434
ASV_2435	ASV_2436	ASV_2437	ASV_2438	ASV_2439	ASV_2440	ASV_2441	ASV_2442	ASV_2443
ASV_2444	ASV_2445	ASV_2446	ASV_2447	ASV_2448	ASV_2449	ASV_2450	ASV_2451	ASV_2452
ASV_2453	ASV_2454	ASV_2455	ASV_2456	ASV_2457	ASV_2458	ASV_2459	ASV_2460	ASV_2461
ASV_2462	ASV_2463	ASV_2464	ASV_2465	ASV_2466	ASV_2467	ASV_2468	ASV_2469	ASV_2470
ASV_2471	ASV_2472	ASV_2473	ASV_2474	ASV_2475	ASV_2476	ASV_2477	ASV_2478	ASV_2479
ASV_2480	ASV_2481	ASV_2482	ASV_2483	ASV_2484	ASV_2485	ASV_2486	ASV_2487	ASV_2488
ASV_2489	ASV_2490	ASV_2491	ASV_2492	ASV_2493	ASV_2494	ASV_2495	ASV_2496	ASV_2497
ASV_2498	ASV_2499	ASV_2500	ASV_2501	ASV_2502	ASV_2503	ASV_2504	ASV_2505	ASV_2506
ASV_2507	ASV_2508	ASV_2509	ASV_2510	ASV_2511	ASV_2512	ASV_2513	ASV_2514	ASV_2515
ASV_2516	ASV_2517	ASV_2518	ASV_2519	ASV_2520	ASV_2521	ASV_2522	ASV_2523	ASV_2524
ASV_2525	ASV_2526	ASV_2527	ASV_2528	ASV_2529	ASV_2530	ASV_2531	ASV_2532	ASV_2533
ASV_2534	ASV_2535	ASV_2536	ASV_2537	ASV_2538	ASV_2539	ASV_2540	ASV_2541	ASV_2542
ASV_2543	ASV_2544	ASV_2545	ASV_2546	ASV_2547	ASV_2548	ASV_2549	ASV_2550	ASV_2551
ASV_2552	ASV_2553	ASV_2554	ASV_2555	ASV_2556	ASV_2557	ASV_2558	ASV_2559	ASV_2560
ASV_2561	ASV_2562	ASV_2563	ASV_2564	ASV_2565	ASV_2566	ASV_2567	ASV_2568	ASV_2569
ASV_2570	ASV_2571	ASV_2572	ASV_2573	ASV_2574	ASV_2575	ASV_2576	ASV_2577	ASV_2578
ASV_2579	ASV_2580	ASV_2581	ASV_2582	ASV_2583	ASV_2584	ASV_2585	ASV_2586	ASV_2587
ASV_2588	ASV_2589	ASV_2590	ASV_2591	ASV_2592	ASV_2593	ASV_2594	ASV_2595	ASV_2596
ASV_2597	ASV_2598	ASV_2599	ASV_2600	ASV_2601	ASV_2602	ASV_2603	ASV_2604	ASV_2605
ASV_2606	ASV_2607	ASV_2608	ASV_2609	ASV_2610	ASV_2611	ASV_2612	ASV_2613	ASV_2614
ASV_2615	ASV_2616	ASV_2617	ASV_2618	ASV_2619	ASV_2620	ASV_2621	ASV_2622	ASV_2623
ASV_2624	ASV_2625	ASV_2626	ASV_2627	ASV_2628	ASV_2629	ASV_2630	ASV_2631	ASV_2632
ASV_2633	ASV_2634	ASV_2635	ASV_2636	ASV_2637	ASV_2638	ASV_2639	ASV_2640	ASV_2641
ASV_2642	ASV_2643	ASV_2644	ASV_2645	ASV_2646	ASV_2647	ASV_2648	ASV_2649	ASV_2650

ASV_2651	ASV_2652	ASV_2653	ASV_2654	ASV_2655	ASV_2656	ASV_2657	ASV_2658	ASV_2659
ASV_2660	ASV_2661	ASV_2662	ASV_2663	ASV_2664	ASV_2665	ASV_2666	ASV_2667	ASV_2668
ASV_2669	ASV_2670	ASV_2671	ASV_2672	ASV_2673	ASV_2674	ASV_2675	ASV_2676	ASV_2677
ASV_2678	ASV_2679	ASV_2680	ASV_2681	ASV_2682	ASV_2683	ASV_2684	ASV_2685	ASV_2686
ASV_2687	ASV_2688	ASV_2689	ASV_2690	ASV_2691	ASV_2692	ASV_2693	ASV_2694	ASV_2695
ASV_2696	ASV_2697	ASV_2698	ASV_2699	ASV_2700	ASV_2701	ASV_2702	ASV_2703	ASV_2704
ASV_2705	ASV_2706	ASV_2707	ASV_2708	ASV_2709	ASV_2710	ASV_2711	ASV_2712	ASV_2713
ASV_2714	ASV_2715	ASV_2716	ASV_2717	ASV_2718	ASV_2719	ASV_2720	ASV_2721	ASV_2722
ASV_2723	ASV_2724	ASV_2725	ASV_2726	ASV_2727	ASV_2728	ASV_2729	ASV_2730	ASV_2731
ASV_2732	ASV_2733	ASV_2734	ASV_2735	ASV_2736	ASV_2737	ASV_2738	ASV_2739	ASV_2740
ASV_2741	ASV_2742	ASV_2743	ASV_2744	ASV_2745	ASV_2746	ASV_2747	ASV_2748	ASV_2749
ASV_2750	ASV_2751	ASV_2752	ASV_2753	ASV_2754	ASV_2755	ASV_2756	ASV_2757	ASV_2758
ASV_2759	ASV_2760	ASV_2761	ASV_2762	ASV_2763	ASV_2764	ASV_2765	ASV_2766	ASV_2767
ASV_2768	ASV_2769	ASV_2770	ASV_2771	ASV_2772	ASV_2773	ASV_2774	ASV_2775	ASV_2776
ASV_2777	ASV_2778	ASV_2779	ASV_2780	ASV_2781	ASV_2782	ASV_2783	ASV_2784	ASV_2785
ASV_2786	ASV_2787	ASV_2788	ASV_2789	ASV_2790	ASV_2791	ASV_2792	ASV_2793	ASV_2794
ASV_2795	ASV_2796	ASV_2797	ASV_2798	ASV_2799	ASV_2800	ASV_2801	ASV_2802	ASV_2803
ASV_2804	ASV_2805	ASV_2806	ASV_2807	ASV_2808	ASV_2809	ASV_2810	ASV_2811	ASV_2812
ASV_2813	ASV_2814	ASV_2815	ASV_2816	ASV_2817	ASV_2818	ASV_2819	ASV_2820	ASV_2821
ASV_2822	ASV_2823	ASV_2824	ASV_2825	ASV_2826	ASV_2827	ASV_2828	ASV_2829	ASV_2830
ASV_2831	ASV_2832	ASV_2833	ASV_2834	ASV_2835	ASV_2836	ASV_2837	ASV_2838	ASV_2839
ASV_2840	ASV_2841	ASV_2842	ASV_2843	ASV_2844	ASV_2845	ASV_2846	ASV_2847	ASV_2848
ASV_2849	ASV_2850	ASV_2851	ASV_2852	ASV_2853	ASV_2854	ASV_2855	ASV_2856	ASV_2857
ASV_2858	ASV_2859	ASV_2860	ASV_2861	ASV_2862	ASV_2863	ASV_2864	ASV_2865	ASV_2866
ASV_2867	ASV_2868	ASV_2869	ASV_2870	ASV_2871	ASV_2872	ASV_2873	ASV_2874	ASV_2875
ASV_2876	ASV_2877	ASV_2878	ASV_2879	ASV_2880	ASV_2881	ASV_2882	ASV_2883	ASV_2884
ASV_2885	ASV_2886	ASV_2887	ASV_2888	ASV_2889	ASV_2890	ASV_2891	ASV_2892	ASV_2893
ASV_2894	ASV_2895	ASV_2896	ASV_2897	ASV_2898	ASV_2899	ASV_2900	ASV_2901	ASV_2902
ASV_2903	ASV_2904	ASV_2905	ASV_2906	ASV_2907	ASV_2908	ASV_2909	ASV_2910	ASV_2911
ASV_2912	ASV_2913	ASV_2914	ASV_2915	ASV_2916	ASV_2917	ASV_2918	ASV_2919	ASV_2920
ASV_2921	ASV_2922	ASV_2923	ASV_2924	ASV_2925	ASV_2926	ASV_2927	ASV_2928	ASV_2929
ASV_2930	ASV_2931	ASV_2932	ASV_2933	ASV_2934	ASV_2935	ASV_2936	ASV_2937	ASV_2938
ASV_2939	ASV_2940	ASV_2941	ASV_2942	ASV_2943	ASV_2944	ASV_2945	ASV_2946	ASV_2947
ASV_2948	ASV_2949	ASV_2950	ASV_2951	ASV_2952	ASV_2953	ASV_2954	ASV_2955	ASV_2956
ASV_2957	ASV_2958	ASV_2959	ASV_2960	ASV_2961	ASV_2962	ASV_2963	ASV_2964	ASV_2965
ASV_2966	ASV_2967	ASV_2968	ASV_2969	ASV_2970	ASV_2971	ASV_2972	ASV_2973	ASV_2974
ASV_2975	ASV_2976	ASV_2977	ASV_2978	ASV_2979	ASV_2980	ASV_2981	ASV_2982	ASV_2983

ASV_2984	ASV_2985	ASV_2986	ASV_2987	ASV_2988	ASV_2989	ASV_2990	ASV_2991	ASV_2992
ASV_2993	ASV_2994	ASV_2995	ASV_2996	ASV_2997	ASV_2998	ASV_2999	ASV_3000	ASV_3001
ASV_3002	ASV_3003	ASV_3004	ASV_3005	ASV_3006	ASV_3007	ASV_3008	ASV_3009	ASV_3010
ASV_3011	ASV_3012	ASV_3013	ASV_3014	ASV_3015	ASV_3016	ASV_3017	ASV_3018	ASV_3019
ASV_3020	ASV_3021	ASV_3022	ASV_3023	ASV_3024	ASV_3025	ASV_3026	ASV_3027	ASV_3028
ASV_3029	ASV_3030	ASV_3031	ASV_3032	ASV_3033	ASV_3034	ASV_3035	ASV_3036	ASV_3037
ASV_3038	ASV_3039	ASV_3040	ASV_3041	ASV_3042	ASV_3043	ASV_3044	ASV_3045	ASV_3046
ASV_3047	ASV_3048	ASV_3049	ASV_3050	ASV_3051	ASV_3052	ASV_3053	ASV_3054	ASV_3055
ASV_3056	ASV_3057	ASV_3058	ASV_3059	ASV_3060	ASV_3061	ASV_3062	ASV_3063	ASV_3064
ASV_3065	ASV_3066	ASV_3067	ASV_3068	ASV_3069	ASV_3070	ASV_3071	ASV_3072	ASV_3073
ASV_3074	ASV_3075	ASV_3076	ASV_3077	ASV_3078	ASV_3079	ASV_3080	ASV_3081	ASV_3082
ASV_3083	ASV_3084	ASV_3085	ASV_3086	ASV_3087	ASV_3088	ASV_3089	ASV_3090	ASV_3091
ASV_3092	ASV_3093	ASV_3094	ASV_3095	ASV_3096	ASV_3097	ASV_3098	ASV_3099	ASV_3100
ASV_3101	ASV_3102	ASV_3103	ASV_3104	ASV_3105	ASV_3106	ASV_3107	ASV_3108	ASV_3109
ASV_3110	ASV_3111	ASV_3112	ASV_3113	ASV_3114	ASV_3115	ASV_3116	ASV_3117	ASV_3118
ASV_3119	ASV_3120	ASV_3121	ASV_3122	ASV_3123	ASV_3124	ASV_3125	ASV_3126	ASV_3127
ASV_3128	ASV_3129	ASV_3130	ASV_3131	ASV_3132	ASV_3133	ASV_3134	ASV_3135	ASV_3136
ASV_3137	ASV_3138	ASV_3139	ASV_3140	ASV_3141	ASV_3142	ASV_3143	ASV_3144	ASV_3145
ASV_3146	ASV_3147	ASV_3148	ASV_3149	ASV_3150	ASV_3151	ASV_3152	ASV_3153	ASV_3154
ASV_3155	ASV_3156	ASV_3157	ASV_3158	ASV_3159	ASV_3160	ASV_3161	ASV_3162	ASV_3163
ASV_3164	ASV_3165	ASV_3166	ASV_3167	ASV_3168	ASV_3169	ASV_3170	ASV_3171	ASV_3172
ASV_3173	ASV_3174	ASV_3175	ASV_3176	ASV_3177	ASV_3178	ASV_3179	ASV_3180	ASV_3181
ASV_3182	ASV_3183	ASV_3184	ASV_3185	ASV_3186	ASV_3187	ASV_3188	ASV_3189	ASV_3190
ASV_3191	ASV_3192	ASV_3193	ASV_3194	ASV_3195	ASV_3196	ASV_3197	ASV_3198	ASV_3199
ASV_3200	ASV_3201	ASV_3202	ASV_3203	ASV_3204	ASV_3205	ASV_3206	ASV_3207	ASV_3208
ASV_3209	ASV_3210	ASV_3211	ASV_3212	ASV_3213	ASV_3214	ASV_3215	ASV_3216	ASV_3217
ASV_3218	ASV_3219	ASV_3220	ASV_3221	ASV_3222	ASV_3223	ASV_3224	ASV_3225	ASV_3226
ASV_3227	ASV_3228	ASV_3229	ASV_3230	ASV_3231	ASV_3232	ASV_3233	ASV_3234	ASV_3235
ASV_3236	ASV_3237	ASV_3238	ASV_3239	ASV_3240	ASV_3241	ASV_3242	ASV_3243	ASV_3244
ASV_3245	ASV_3246	ASV_3247	ASV_3248	ASV_3249	ASV_3250	ASV_3251	ASV_3252	ASV_3253
ASV_3254	ASV_3255	ASV_3256	ASV_3257	ASV_3258	ASV_3259	ASV_3260	ASV_3261	ASV_3262
ASV_3263	ASV_3264	ASV_3265	ASV_3266	ASV_3267	ASV_3268	ASV_3269	ASV_3270	ASV_3271
ASV_3272	ASV_3273	ASV_3274	ASV_3275	ASV_3276	ASV_3277	ASV_3278	ASV_3279	ASV_3280
ASV_3281	ASV_3282	ASV_3283	ASV_3284	ASV_3285	ASV_3286	ASV_3287	ASV_3288	ASV_3289
ASV_3290	ASV_3291	ASV_3292	ASV_3293	ASV_3294	ASV_3295	ASV_3296	ASV_3297	ASV_3298
ASV_3299	ASV_3300	ASV_3301	ASV_3302	ASV_3303	ASV_3304	ASV_3305	ASV_3306	ASV_3307
ASV_3308	ASV_3309	ASV_3310	ASV_3311	ASV_3312	ASV_3313	ASV_3314	ASV_3315	ASV_3316

ASV_3317 ASV_3318 ASV_3319 ASV_3320 ASV_3321 ASV_3322 ASV_3323 ASV_3324 ASV_3325
ASV_3326 ASV_3327 ASV_3328 ASV_3329 ASV_3330 ASV_3331 ASV_3332 ASV_3333 ASV_3334
ASV_3335 ASV_3336 ASV_3337 ASV_3338 ASV_3339 ASV_3340 ASV_3341 ASV_3342 ASV_3343
ASV_3344 ASV_3345 ASV_3346 ASV_3347 ASV_3348 ASV_3349 ASV_3350 ASV_3351 ASV_3352
ASV_3353

Jan_DL001	1644	1885	23	425	1635	209	319	298	145	79	114	97	67	175	333	40	113	137
92	462	226	854	318	171	49	98	74	66	92	0	64	127	25	38	57	38	0
70	185	109	33	92	46	45	0	57	0	81	0	208	43	66	4	0	57	0
0	0	0	225	70	268	91	62	0	70	61	48	131	123	54	97	40	63	0
0	0	35	6	21	8	9	0	78	0	0	0	60	6	45	0	27	70	0
0	32	0	0	0	18	0	73	127	9	0	65	39	74	0	62	13	0	57
0	0	0	13	48	15	0	0	0	0	15	0	30	20	0	4	25	43	0
0	0	45	38	0	0	0	42	0	0	0	0	30	0	33	30	0	0	0
0	35	0	4	0	0	0	0	0	0	29	0	0	0	0	0	0	33	0
0	0	11	35	0	47	0	35	0	18	0	0	0	54	8	15	51	12	0
0	40	46	0	0	59	0	25	0	31	0	24	0	0	117	86	0	0	0
44	0	0	0	0	0	38	0	0	34	0	0	0	0	0	4	0	10	25
0	0	0	0	0	0	0	0	0	0	0	0	22	0	13	46	0	0	0
23	0	0	29	44	0	0	0	0	0	0	0	0	0	17	0	0	0	0
10	0	0	0	7	0	0	0	23	0	19	2	0	0	0	23	0	0	0
0	19	21	0	0	0	0	60	9	0	0	0	18	0	18	7	0	0	0
0	20	0	0	0	0	0	5	0	18	0	0	16	0	78	0	0	0	0
33	0	0	17	16	0	0	0	0	30	0	0	0	33	29	0	0	0	0
6	0	0	0	3	0	0	16	55	50	0	0	0	0	51	0	13	0	0
0	0	0	0	0	0	0	0	0	0	0	0	8	0	0	0	0	0	6
0	0	0	0	0	16	14	2	36	0	0	0	5	0	5	0	0	10	0
0	0	0	0	0	0	0	6	0	10	0	0	16	0	10	0	6	0	0
0	0	27	0	0	0	0	0	0	0	0	0	0	0	38	0	0	0	0
0	3	0	70	0	0	0	0	0	0	36	0	0	0	0	0	30	17	6
11	14	12	0	0	0	0	0	0	0	0	0	0	0	0	0	0	0	0
0	0	0	0	3	0	14	2	0	0	0	0	0	0	0	0	0	0	0
6	0	0	0	0	0	18	0	0	0	0	0	0	0	0	0	9	0	0
0	0	0	0	0	0	0	10	0	0	0	0	0	0	0	0	0	0	0
22	0	0	0	0	0	0	0	0	0	0	8	0	0	0	0	0	0	0
0	0	0	0	13	0	0	0	0	0	0	0	10	0	0	0	0	0	0
0	9	0	23	0	19	0	0	0	0	0	0	8	25	11	0	0	0	0
0	0	0	0	0	0	0	11	0	0	0	0	0	0	0	0	0	0	0

0	0	0	12	0	4	0	0	0	0	0	0	0	9	0	0	0	0	9
0	9	0	0	0	0	0	3	0	0	0	0	0	0	10	0	0	0	0
0	0	0	0	8	0	0	0	0	0	0	0	0	0	0	21	0	0	0
0	0	0	0	0	0	0	0	0	0	0	0	0	0	0	0	0	0	0
0	0	0	0	0	0	0	0	0	0	0	0	0	0	0	0	0	0	0
0	0	0	6	0	0	0	0	0	0	0	0	0	0	0	0	0	0	0
0	0	0	0	0	0	0	0	0	0	0	0	0	0	6	0	0	0	0
0	0	0	0	0	0	0	0	0	0	0	0	0	0	0	0	0	0	0
0	20	0	16	0	0	14	0	0	0	0	0	0	0	0	0	0	0	5
5	0	0	0	0	0	0	0	0	0	0	0	0	4	0	0	0	0	0
0	0	0	0	0	0	0	0	18	0	0	0	4	0	0	0	0	0	0
0	7	0	0	0	0	0	0	0	0	0	0	0	0	0	0	0	0	0
0	0	0	5	0	0	0	0	0	0	0	0	0	0	0	0	0	0	0
0	0	0	0	0	0	0	0	3	0	0	0	0	0	0	8	12	0	0
0	0	0	0	0	0	0	0	15	0	0	0	0	0	0	0	0	0	0
0	0	0	0	0	0	0	0	0	0	0	0	0	0	0	0	0	0	11
0	0	0	0	17	0	0	0	0	0	0	0	0	0	0	0	0	0	0
0	0	0	0	0	0	0	0	0	0	0	0	0	0	0	3	0	0	0
0	0	0	0	0	0	0	0	0	0	0	0	0	0	0	0	0	0	0
0	0	0	0	0	0	0	0	0	0	0	0	0	0	0	0	0	0	0
0	0	0	0	0	0	0	0	0	0	0	0	0	0	0	0	0	0	0
0	0	0	0	0	0	0	0	0	0	0	0	0	0	0	0	0	0	0
0	0	0	0	0	0	0	0	0	0	0	0	0	0	0	0	0	0	0
0	0	0	0	0	0	0	0	0	0	0	0	0	0	0	0	0	0	0
0	0	0	0	0	0	0	0	0	0	0	0	0	0	0	0	0	0	0
0	0	0	0	0	0	0	0	0	0	0	0	0	0	0	0	0	0	0
0	0	0	0	0	0	0	0	0	0	0	0	0	0	0	0	0	0	0
0	0	0	0	0	0	0	0	0	0	0	0	0	0	0	0	0	0	0
0	0	0	0	0	0	0	0	0	0	0	0	0	0	0	0	0	0	0
0	0	0	0	0	0	0	0	0	0	0	0	0	0	0	0	0	0	13
0	0	0	0	0	0	0	0	0	0	0	0	0	0	0	0	0	0	0
0	0	0	0	0	0	0	0	0	0	0	0	0	0	0	0	0	0	0
0	0	0	0	0	0	0	0	0	0	0	0	0	0	0	0	0	0	0
0	0	0	0	0	0	0	0	0	0	0	0	0	0	0	0	0	0	0
0	0	0	0	0	0	0	0	0	0	0	0	0	0	0	0	0	0	0
0	0	0	0	0	0	0	0	0	0	0	0	0	0	0	0	0	0	0
0	0	0	0	0	0	0	0	0	0	0	0	0	0	0	0	0	0	0

0	0	0	0	0	0	0	0	0	0	0	0	0	0	0	0	0	0	0
0	0	0	0	0	0	0	0	0	0	0	0	0	0	0	0	0	0	0
0	0	0	0	0	0	0	0	0	0	0	0	0	0	0	0	0	0	13
0	0	0	0	0	0	0	0	0	0	0	0	0	0	0	0	0	0	0
0	0	0	0	0	0	0	0	0	0	0	0	0	0	0	0	0	0	0
0	0	0	0	0	0	0	0	0	0	0	0	0	0	0	0	0	0	0
0	0	0	0	0	0	0	0	0	0	0	0	0	0	0	0	0	0	0
0	0	0	0	0	0	0	0	0	0	0	0	0	0	0	0	0	0	0
0	0	0	0	0	0	0	0	0	0	0	0	0	0	0	0	0	0	0
0	0	0	0	0	0	0	0	0	0	0	0	0	0	0	0	0	0	0
0	0	0	0	0	0	0	0	0	0	0	0	0	0	0	0	0	0	0
0	0	0	0	0	0	0	0	0	0	0	0	0	0	0	0	0	0	0
0	0	0	0	0	0	0	0	0	0	0	0	0	0	0	0	0	0	0
0	0	0	0	0	0	0	0	0	0	0	0	0	0	0	0	0	0	0
0	0	0	0	0	0	0	0	0	0	0	0	0	0	0	0	0	0	0
0	0	0	0	0	0	0	0	0	0	0	0	0	0	0	0	0	0	0
0	0	0	0	0	0	0	0	0	0	0	0	0	0	0	0	0	0	0
0	0	0	0	0	0	0	0	0	0	0	0	0	0	3	0	0	0	0
0	0	0	0	0	0	0	0	0	0	0	0	0	0	0	0	0	0	0
0	0	0	0	0	0	0	0	0	0	0	0	0	0	0	0	0	0	0
0	0	0	0	0	0	0	0	0	0	0	0	0	0	0	0	0	0	0
0	0	0	0	0	0	0	0	0	0	0	0	0	0	0	0	0	0	0
0	0	0	0	0	0	0	0	0	0	0	0	0	0	0	0	0	0	0
0	0	0	0	0	0	0	0	0	0	0	0	0	0	0	0	0	0	0
0	0	0	0	0	0	0	0	0	0	0	0	0	0	0	0	0	0	0
0	0	0	0	0	0	0	0	0	0	0	0	0	0	0	0	0	0	0
0	0	0	0	0	0	0	0	0	0	0	0	0	0	0	0	0	0	0
0	0	0	0	0	0	0	0	0	0	0	0	0	0	0	0	0	0	0
0	0	0	0	0	0	0	0	0	0	0	0	0	0	0	0	0	0	0
0	0	0	0	0	0	6	0	0	0	0	0	0	0	0	0	0	0	0
0	0	0	0	0	0	0	0	0	0	0	0	0	0	0	0	0	0	0
0	0	0	0	0	0	0	0	0	0	0	0	0	0	0	0	0	0	0
0	0	0	0	0	0	0	0	0	0	0	0	0	0	0	0	0	0	0
0	0	0	0	0	0	0	0	0	0	0	0	0	0	0	0	0	0	0
0	0	0	0	0	0	0	0	0	0	0	0	0	0	0	0	0	0	0
0	0	0	0	0	0	0	0	0	0	0	0	0	0	0	0	0	0	0
0	0	0	0	0	0	0	0	0	0	0	0	0	0	0	0	0	0	0

0	0	0	0	0	0	0	0	0	0	0	0	0	0	0	0	5	0	0
0	0	0	0	0	0	0	0	0	0	0	0	0	0	0	0	0	0	0
0	0	0	0	0	0	0	0	0	0	0	0	0	0	0	0	0	0	0
0	0	0	0	0	0	0	0	0	0	0	0	0	0	0	0	0	0	0
0	0	0	0	0	0	0	0	0	0	0	0	0	0	0	0	0	0	0
0	0	0	0	0	0	0	0	0	0	0	0	0	0	0	0	0	0	0
0	0	0	0	0	0	0	0	0	0	0	0	0	0	0	0	0	0	0
0	0	0	0	0	0	0	0	0	0	0	0	0	0	0	0	0	0	0
0	0	0	0	0	0	0	0	0	0	0	0	0	0	0	0	0	0	0
0	0	0	0	0	0	0	0	0	0	0	0	0	0	0	0	0	0	0
0	0	0	0	0	0	0	0	0	0	0	0	0	0	0	0	0	0	0
0	0	0	0	0	0	4	4	0	0	0	0	0	0	0	0	0	0	0
0	0	0	0	0	0	0	0	0	0	0	0	0	0	0	0	0	0	0
0	0	0	0	0	0	0	0	0	0	0	0	0	0	0	0	0	0	0
0	0	0	0	0	0	0	0	0	0	0	0	0	0	0	0	0	0	0
0	0	0	0	0	0	0	0	0	0	0	0	0	0	0	0	0	0	0
0	0	0	0	0	0	0	0	0	0	0	0	0	0	0	0	0	0	0
0	0	0	0	0	0	0	0	0	0	0	0	0	0	0	0	0	0	0
0	0	0	0	0	0	0	0	0	0	0	0	0	0	0	0	0	0	0
0	0	0	0	0	0	0	0	0	0	0	0	0	0	0	0	0	0	0
0	0	0	0	0	0	0	0	0	0	0	0	0	0	0	0	0	0	0
0	0	0	0	0	0	0	0	0	0	0	0	0	0	0	0	0	0	0
0	0	0	0	0	0	0	0	0	0	0	0	0	0	0	0	0	0	0
0	0	0	0	0	0	0	0	0	0	0	0	0	0	0	0	0	0	0
0	0	0	0	0	0	0	0	0	0	0	0	0	0	0	0	0	0	0
0	0	0	0	0	0	0	0	0	0	0	0	0	0	0	0	0	0	0
0	0	0	0	0	0	0	0	0	0	0	0	0	0	0	0	0	0	0
0	0	0	0	0	0	0	0	0	0	0	0	0	0	0	0	0	0	0
0	0	0	0	0	0	0	0	0	0	0	0	0	0	0	0	0	0	0
0	0	0	0	0	0	0	0	0	0	0	0	0	0	0	0	0	0	0
0	0	0	0	0	0	0	0	0	0	0	0	0	0	0	0	0	0	0
0	0	0	0	0	0	0	0	0	0	0	0	0	0	0	0	0	0	0
0	0	0	0	0	0	0	0	0	0	0	0	0	0	0	0	0	0	0
0	0	0	0	0	0	0	0	0	0	0	0	0	0	0	0	0	0	0
0	0	0	0	0	0	0	0	0	0	0	0	0	0	0	0	0	0	0
0	0	0	0	0	0	0	0	0	0	0	0	0	0	0	0	0	0	0
0	0	0	0	0	0	0	0	0	0	0	0	0	0	0	0	0	0	0

0	0	0	0	0	0	0	0	0	0	0	0	0	0	0	0	0	0	0
0	0	0	0	0	0	0	0	0	0	0	0	0	0	0	0	0	0	0
0	0	0	0	0	0	0	0	0	0	0	0	0	0	0	0	0	0	0
0	0	0	0	0	0	0	0	0	0	0	0	0	0	0	0	0	0	0
0	0	0	0	0	0	0	0	0	0	0	0	0	0	0	0	0	0	0
0	0	0	0	0	0	0	0	0	0	0	0	0	0	0	0	0	0	0
0	0	0	0	0	0	0	0	0	0	0	0	0	0	0	0	0	0	0
0	0	0	0	0	0	0	0	0	0	0	0	0	0	0	0	0	0	0
0	0	0	0	0	0	0	0	0	0	0	0	0	0	0	0	0	0	0
0	0	0	0	0	0	0	0	0	0	0	0	0	0	0	0	0	0	0
0	0	0	0	0	0	0	0	0	0	0	0	0	0	0	0	0	0	0
0	0	0	0	0	0	0	0	0	0	0	0	0	0	0	0	0	0	0
0	0	0	0	0	0	0	0	0	0	0	0	0	0	0	0	0	0	0
0	0	0	0	0	0	0	0	0	0	0	0	0	0	0	0	0	0	0
0	0	0	0	0	0	0	0	0	0	0	0	0	0	0	0	0	0	0
0	0	0	0	0	0	0	0	0	0	0	0	0	0	0	0	0	0	0
0	0	0	0	0	0	0	0	0	0	0	0	0	0	0	0	0	0	0
0	0	0	0	0	0	0	0	0	0	0	0	0	0	0	0	0	0	0
0	0	0	0	0	0	2	2	2	2	0	0	0	0	0	0	0	0	0
0	0	0	0	0	0	0	0	0	0	0	0	0	0	0	0	0	0	0
0	0	0	0	0	0	0	0	0	0	0	0	0	0	0	0	0	0	0
0	0	0	0	0	0	0	0	0	0	0	0	0	0	0	0	0	0	0
0	0	0	0	0	0	0	0	0	0	0	0	0	0	0	0	0	0	0
0	0	0	0	0	0	0	0	0	0	0	0	0	0	0	0	0	0	0
0	0	0	0	0	0	0	0	0	0	0	0	0	0	0	0	0	0	0
0	0	0	0	0	0	0	0	0	0	0	0	0	0	0	0	0	0	0
0	0	0	0	0	0	0	0	0	0	0	0	0	0	0	0	0	0	0
0	0	0	0	0	0	0	0	0	0	0	0	0	0	0	0	0	0	0
0	0	0	0	0	0	0	0	0	0	0	0	0	0	0	0	0	0	0
0	0	0	0	0	0	0	0	0	0	0	0	0	0	0	0	0	0	0
0	0	0	0	0	0	0	0	0	0	0	0	0	0	0	0	0	0	0
0	0	0	0	0	0	0	0	0	0	0	0	0	0	0	0	0	0	0
0	0	0	0	0	0	0	0	0	0	0	0	0	0	0	0	0	0	0
0	0	0	0	0	0	0	0	0	0									
Jan_DL010	2144	637	814	216	212	199	247	174	198	97	311	609	91	323	275	482	402	246
192	1347	63	0	100	58	135	244	163	58	173	0	133	188	42	169	199	0	0
0	151	246	20	94	132	77	128	225	195	135	73	239	99	47	62	19	116	0

0	66	56	81	112	25	0	56	118	8	56	0	121	0	28	0	13	44	7
27	0	18	31	0	40	60	25	66	26	0	0	20	0	0	0	30	30	63
69	15	0	0	0	0	17	0	0	0	0	0	0	25	28	0	0	11	23
0	0	0	10	32	35	0	0	0	0	0	5	11	0	40	0	0	16	0
0	0	64	0	0	0	0	0	0	0	0	0	7	0	0	0	0	0	0
0	0	0	11	0	0	0	87	8	89	12	0	0	0	0	0	15	0	14
0	0	8	69	0	0	108	0	0	0	0	21	0	0	0	0	10	0	0
0	0	0	0	94	0	0	0	32	0	0	0	0	0	7	85	0	0	0
0	57	13	0	0	0	15	0	0	0	0	0	0	0	0	29	0	21	0
0	0	0	30	0	0	0	79	12	0	0	0	0	11	15	0	0	0	0
0	0	0	10	0	0	0	0	0	0	0	0	11	0	0	0	24	8	0
0	0	0	0	8	0	0	0	0	9	0	0	0	0	6	4	0	0	0
0	0	0	0	0	0	0	0	0	0	0	17	51	0	0	0	0	0	0
0	0	0	0	0	0	0	0	0	0	0	0	9	12	0	7	0	0	0
0	0	11	0	0	0	0	5	0	34	0	0	0	0	0	0	8	0	0
16	0	0	0	7	7	0	16	8	3	0	0	18	0	24	0	0	0	0
0	0	7	0	0	0	0	0	0	0	0	0	0	0	0	0	0	0	0
0	0	0	0	4	3	18	0	0	0	0	19	0	0	0	0	0	0	0
0	0	0	0	0	0	0	0	0	0	0	0	0	8	0	0	2	24	0
0	0	0	0	0	0	0	0	2	0	0	0	0	0	0	0	0	0	0
0	0	0	0	0	0	0	0	0	0	0	0	0	0	0	29	0	0	0
0	0	0	0	0	0	0	24	0	0	0	0	0	0	0	15	0	0	0
0	0	0	13	0	0	0	0	0	0	0	0	0	11	0	0	0	0	0
0	0	10	0	0	0	0	0	0	0	0	0	0	0	0	0	0	0	0
0	0	0	0	0	4	0	0	0	0	0	0	0	0	0	0	0	0	0
0	0	0	0	0	0	0	0	0	0	0	0	0	0	0	0	0	0	0
0	0	0	0	0	0	0	0	0	0	3	0	0	0	0	0	17	0	0
0	0	0	0	0	0	0	0	0	0	0	0	0	0	0	0	0	0	0
0	0	0	0	0	0	0	0	0	0	0	0	0	5	0	0	0	0	0
0	0	0	0	0	0	0	0	0	0	0	0	0	0	0	0	0	0	0
0	0	0	0	0	0	0	0	0	0	0	0	0	0	0	0	0	0	0
0	0	0	0	0	0	0	0	0	0	0	0	0	0	0	0	0	0	0
0	0	0	0	0	0	0	0	0	0	0	0	0	0	0	0	0	0	0
0	0	0	0	0	0	0	0	0	0	0	0	0	0	0	0	0	0	0
0	0	0	0	0	0	0	0	4	0	0	0	0	0	0	0	0	0	0
0	0	0	0	0	0	0	0	0	0	0	0	0	19	0	0	0	0	0
0	0	0	0	0	0	0	0	0	0	0	0	0	0	0	0	0	0	0

0	0	0	0	0	0	0	0	0	0	0	0	0	0	0	0	0	0	0
0	0	0	0	0	0	0	3	0	0	0	0	0	0	0	0	0	0	0
0	0	0	0	0	0	0	0	0	0	0	0	0	0	0	7	0	0	0
0	0	0	0	0	0	0	0	0	0	0	0	0	0	0	0	0	0	0
0	0	0	0	0	0	0	0	0	0	0	0	0	0	0	0	0	0	0
0	0	0	0	9	0	0	0	0	0	0	0	0	0	0	0	0	0	0
0	0	0	0	0	0	0	0	0	0	0	0	0	0	0	0	0	0	0
0	0	0	0	0	0	0	0	0	0	0	0	0	0	0	0	0	0	0
0	0	0	0	0	0	0	0	0	0	0	0	0	0	0	0	0	0	0
0	0	0	0	0	0	0	0	0	0	0	0	0	0	0	0	0	0	0
0	0	0	0	0	0	0	0	0	0	0	0	0	0	0	0	0	0	0
0	0	0	0	0	0	0	0	0	0	0	0	0	0	0	0	0	0	0
0	0	0	0	0	0	0	0	0	0	0	0	0	0	0	0	0	0	0
0	0	0	0	0	0	0	0	0	0	0	0	0	0	0	0	0	0	0
0	0	0	0	0	0	0	0	0	0	0	0	0	0	0	0	0	0	0
0	0	0	0	0	0	0	11	0	0	0	0	0	0	0	0	0	0	0
0	0	0	0	0	0	0	0	0	0	6	0	4	0	0	0	0	0	0
0	0	0	2	0	0	0	0	0	0	0	0	0	0	0	0	0	0	0
0	0	0	0	0	0	0	0	0	0	0	0	0	0	0	0	0	0	0
0	0	0	0	0	0	0	0	0	0	0	0	0	0	0	0	0	0	0
0	0	0	0	0	0	0	0	0	0	0	0	0	0	0	0	0	0	0
0	0	0	0	0	0	0	0	0	0	0	0	0	0	0	0	0	0	0
0	0	0	0	0	0	0	0	0	0	0	0	0	0	0	0	0	0	0
0	0	0	0	0	0	0	0	0	0	0	0	0	0	0	0	0	0	0
0	0	0	0	0	0	0	0	0	0	0	0	0	0	0	0	0	0	0
0	0	0	0	0	0	0	0	0	0	0	0	0	0	0	0	0	0	0
0	0	0	0	0	0	0	0	0	0	0	0	0	0	0	0	0	0	0
0	0	0	0	0	0	0	0	0	0	0	0	0	0	0	0	0	0	0
0	0	0	0	0	0	0	0	0	0	0	0	0	0	0	0	0	0	0
0	0	0	0	0	0	0	0	0	0	0	0	0	0	0	0	0	0	0
0	0	0	0	0	0	0	0	0	0	0	0	0	0	0	0	0	0	0
0	0	0	0	0	0	0	0	0	0	0	0	0	0	0	0	0	0	0
0	0	0	0	0	0	0	0	0	0	0	0	0	0	0	0	7	0	0
0	0	0	0	0	0	0	0	0	0	0	0	0	0	0	0	0	0	0
0	0	0	0	0	0	0	0	0	0	0	0	0	0	0	0	0	0	0
0	0	0	0	0	0	0	0	0	0	0	0	0	0	0	0	0	0	0
0	0	0	0	0	0	0	0	0	0	0	0	0	0	0	0	0	0	0

0	0	0	0	0	0	0	0	0	0	0	0	0	0	0	0	0	0	0
0	0	0	0	0	0	0	0	0	0	0	0	0	0	0	0	0	0	0
0	0	0	0	0	0	0	0	0	0	0	0	0	0	0	0	0	0	0
0	9	0	0	0	0	0	0	0	0	0	0	0	0	0	0	0	0	0
0	0	0	0	0	0	0	0	0	0	0	0	0	0	0	0	0	0	0
0	0	0	0	0	0	0	0	0	0	0	0	0	0	0	0	0	0	0
0	0	0	0	0	0	0	0	0	0	0	0	0	0	0	0	0	0	0
0	0	0	0	0	0	0	0	0	0	0	0	0	0	0	0	0	0	0
0	0	0	0	0	0	0	0	0	0	0	0	0	0	0	0	0	0	0
0	0	0	0	0	0	0	0	0	0	0	0	0	0	0	0	0	0	0
0	0	0	0	0	0	0	0	0	0	0	0	0	0	0	0	0	0	0
0	0	0	0	0	0	0	0	0	0	0	0	0	0	0	0	0	0	0
0	0	0	0	0	0	0	0	0	7	0	0	0	0	0	0	0	0	0
0	0	0	0	0	0	0	0	0	0	0	0	0	0	0	0	0	0	0
0	0	0	0	0	0	0	0	0	0	0	0	0	0	0	0	0	0	0
0	0	0	0	0	0	0	0	0	0	0	0	0	0	0	0	0	0	0
0	0	0	0	0	0	0	0	0	0	0	0	0	0	0	0	0	0	0
0	0	0	0	0	0	0	0	0	0	0	0	0	0	0	0	0	0	0
0	0	0	0	0	0	0	0	0	0	0	0	0	0	0	0	0	0	0
0	0	0	0	0	0	0	0	0	0	0	0	0	0	0	0	0	0	0
0	0	0	0	0	0	0	0	0	0	0	0	0	0	0	0	0	0	0
0	0	0	0	0	0	0	0	0	0	0	0	0	0	0	0	0	0	0
0	0	0	0	0	0	0	0	0	0	0	0	0	0	0	0	0	0	0
0	0	0	0	0	0	0	0	0	0	0	0	0	0	0	0	0	0	0
0	0	0	0	0	0	0	0	0	0	0	0	0	0	0	0	0	0	0
0	0	0	0	0	0	0	0	0	0	0	0	0	0	0	0	0	0	0
0	0	0	0	0	0	0	0	0	0	0	0	0	0	0	0	0	0	0
0	0	0	0	0	0	0	0	0	0	0	0	0	0	0	0	0	0	0
0	0	0	0	0	0	0	0	0	0	0	0	0	0	0	0	0	0	0
0	0	0	0	0	0	0	0	0	0	0	0	0	0	0	0	0	0	0
0	0	0	0	0	0	0	0	0	0	0	0	0	0	0	0	0	0	0
0	0	0	0	0	0	0	0	0	0	0	0	0	0	0	0	0	0	0
0	0	0	0	0	0	0	0	0	0	0	0	0	0	0	0	0	0	0
0	0	0	0	0	0	0	0	0	0	0	0	0	0	0	0	0	0	0
0	0	0	0	0	0	0	0	0	0	0	0	0	0	0	0	0	0	0
0	0	0	0	0	0	0	0	0	0	0	0	0	0	0	0	0	0	0
0	0	0	0	0	0	0	0	0	0	0	0	0	0	0	0	0	0	0

0	0	0	0	0	0	0	0	0	0	0	0	0	0	0	0	0	0	0
0	0	0	0	0	0	0	0	0	0	0	0	0	0	0	0	0	0	0
0	0	0	0	0	0	0	0	0	0	0	0	0	0	0	0	0	0	0
0	0	0	0	0	0	0	0	0	0	0	0	0	0	0	0	0	0	0
0	0	0	0	0	0	0	0	0	0	0	0	0	0	0	0	0	0	0
0	0	0	0	0	0	0	0	0	0	0	0	0	0	0	0	0	0	0
0	0	0	0	0	0	0	0	0	0	0	0	0	0	0	0	0	0	0
0	0	0	0	0	0	0	0	0	0	0	0	0	0	0	0	0	0	0
0	0	0	0	0	0	0	0	0	0	0	0	0	0	0	0	0	0	0
0	0	0	0	0	0	0	0	0	0	0	0	0	0	0	0	0	0	0
0	0	0	0	0	0	0	0	0	0	0	0	0	0	0	0	0	0	0
0	0	0	0	0	0	0	0	0	0	0	0	0	0	0	0	0	0	0
0	0	0	0	0	0	0	0	0	0	0	0	0	0	0	0	0	0	0
0	0	0	0	0	0	0	0	0	0	0	0	0	0	0	0	0	0	0
0	0	0	0	0	0	0	0	0	0	0	0	0	0	0	0	0	0	0
0	0	0	0	0	0	0	0	0	0	0	0	0	0	0	0	0	0	0
0	0	0	0	0	0	0	0	0	0	0	0	0	0	0	0	0	0	0
0	0	0	0	0	0	0	0	0	0	0	0	0	0	3	3	0	0	0
0	0	0	0	0	0	0	0	0	0	0	0	0	0	0	0	0	0	0
0	0	0	0	0	0	0	0	0	0	0	0	0	0	0	0	0	0	0
0	0	0	0	0	0	0	0	0	0	0	0	0	0	0	0	0	0	0
0	0	0	0	0	0	0	0	0	0	0	0	0	0	0	0	0	0	0
0	0	0	0	0	0	0	0	0	0	0	0	0	0	0	0	0	0	0
0	0	0	0	0	0	0	0	0	0	0	0	0	0	0	0	0	0	0
0	0	0	0	0	0	0	0	0	0	0	0	0	0	0	0	0	0	0
0	0	0	0	0	0	0	0	0	0	0	0	0	0	0	0	0	0	0
0	0	0	0	0	0	0	0	0	0	0	0	0	0	0	0	0	0	0
0	0	0	0	0	0	0	0	0	0	0	0	0	0	0	0	0	0	0
0	0	0	0	0	0	0	0	0	0	0	0	0	0	0	0	0	0	0
0	0	0	0	0	0	0	0	0	0	0	0	0	0	0	0	0	0	0
0	0	0	0	0	0	0	0	0	0	0	0	0	0	0	0	0	0	0
0	0	0	0	0	0	0	0	0	0	0	0	0	0	0	0	0	0	0
0	0	0	0	0	0	0	0	0	0	0	0	0	0	0	0	0	0	0
0	0	0	0	0	0	0	0	0	0	0	0	0	0	0	0	0	0	0
0	0	0	0	0	0	0	0	0	0	0	0	0	0	0	0	0	0	0
0	0	0	0	0	0	0	0	0	0	0	0	0	0	0	0	0	0	0
0	0	0	0	0	0	0	0	0	0	0	0	0	0	0	0	0	0	0

0	0	0	0	0	0	0	0	0	0	0	0	0	0	0	0	0	0	0
0	0	0	0	0	0	0	0	0	0	0	0	0	0	0	0	0	0	0
0	0	0	0	0	0	0	0	0	0	0	0	0	0	0	0	0	0	0
0	0	0	0	0	0	0	0	0	0	0	0	0	0	0	0	0	0	0
0	0	0	0	0	0	0	0	0	0	0	0	0	0	0	0	0	0	0
0	0	0	0	0	0	0	0	0	0	0	0	0	0	0	0	0	0	0
0	0	0	0	0	0	0	0	0	0	0	0	0	0	0	0	0	0	0
0	0	0	0	0	0	2	2	0	0	0	0	0	0	0	0	0	0	0
0	0	0	0	0	0	0	0	0	0	0	0	0	0	0	0	0	0	0
0	0	0	0	0	0	0	0	0	0	0	0	0	0	0	0	0	0	0
0	0	0	0	0	0	0	0	0	0	0	0	0	0	0	0	0	0	0
0	0	0	0	0	0	0	0	0	0	0	0	0	0	0	0	0	0	0
0	0	0	0	0	0	0	0	0	0	0	0	0	0	0	0	0	0	0
0	0	0	0	0	0	0	0	0	0	0	0	0	0	0	0	0	0	0
0	0	0	0	0	0	0	0	0	0	0	0	0	0	0	0	0	0	0
0	0	0	0	0	0	0	0	0	0	0	0	0	0	0	0	0	0	0
0	0	0	0	0	0	0	0	0	0	0	0	0	0	0	0	0	0	0
0	0	0	0	0	0	0	0	0	0	0	0	0	0	0	0	0	0	0
0	0	0	0	0	0	0	0	0	0	0	0	0	0	0	0	0	0	0
0	0	0	0	0	0	0	0	0	0	0	0	0	0	0	0	0	0	0
0	0	0	0	0	0	0	0	0	0	0	0	0	0	0	0	0	0	0
0	0	0	0	0	0	0	0	0	0	0	0	0	0	0	0	0	0	0
0	0	0	0	0	0	0	0	0	0	0	0	0	0	0	0	0	0	0
0	0	0	0	0	0	0	0	0	0	0	0	0	0	0	0	0	0	0
0	0	0	0	0	0	0	0	0	0	0	0	0	0	0	0	0	0	0
0	0	0	0	0	0	0	0	0	0									
Jan_DL100	1251	682	201	564	41	224	247	234	691	375	226	502	295	245	128	223	210	212
236	0	94	152	165	70	183	100	209	123	140	159	164	108	144	117	93	90	144
97	89	196	150	97	125	73	37	109	69	120	52	27	134	219	58	277	106	87
92	54	42	22	89	20	77	0	30	39	34	73	0	67	16	13	7	35	0
19	48	89	22	0	27	66	98	46	0	0	0	27	14	0	115	0	0	0
38	63	0	0	16	0	41	0	46	11	0	0	0	0	29	0	0	34	0
116	20	7	3	0	40	0	0	0	0	0	19	0	11	17	0	0	0	0
0	0	62	0	19	0	0	0	0	2	16	0	5	0	0	7	0	0	0
0	6	0	14	0	0	84	0	18	0	12	0	17	0	0	0	0	0	24
19	57	0	0	0	0	0	0	0	12	0	0	0	12	0	0	0	0	13
0	0	0	0	0	13	60	0	23	0	0	25	0	0	0	0	0	14	0

0	0	30	0	0	0	13	0	0	0	0	7	0	17	0	8	0	0	24
82	0	0	0	0	32	0	0	18	0	0	0	0	6	0	0	9	31	0
0	0	0	0	0	0	0	0	0	14	0	0	0	0	0	0	0	18	0
0	0	0	0	0	3	0	0	0	9	18	10	0	0	0	0	0	67	0
0	0	8	0	0	0	0	0	20	0	0	0	0	0	0	0	0	0	0
0	0	0	0	0	0	0	0	0	0	0	0	8	0	0	0	0	0	4
0	27	0	0	0	43	0	0	0	0	0	0	0	0	0	0	0	0	0
0	6	0	0	0	0	0	17	0	0	0	0	0	0	0	0	0	0	0
0	0	0	0	10	0	0	0	0	0	0	0	0	0	21	0	0	0	6
0	0	25	0	0	0	0	0	0	0	0	0	0	0	0	0	0	7	0
0	0	0	0	0	0	0	0	0	0	0	0	0	0	0	0	0	0	0
0	0	0	0	0	0	0	0	20	0	0	0	0	0	0	0	0	0	0
0	3	0	0	0	0	0	0	0	0	0	0	0	0	0	0	0	0	0
0	0	0	0	0	0	0	0	0	0	0	0	0	0	0	0	0	0	0
0	0	0	0	0	0	0	0	0	0	0	0	0	0	0	12	0	0	0
0	0	0	0	0	0	0	0	0	0	0	0	0	0	0	0	0	0	21
0	0	0	0	0	0	0	0	0	0	0	0	0	0	0	0	0	4	0
0	0	0	0	0	0	0	0	0	0	0	0	0	0	0	0	0	0	0
0	0	0	0	0	0	0	0	0	0	0	0	0	0	0	0	0	0	0
6	0	6	0	0	0	0	0	0	0	0	0	0	0	0	0	0	0	0
0	0	0	0	0	0	0	0	0	0	0	0	0	0	0	0	0	0	0
0	0	0	0	0	0	0	0	0	0	0	0	0	0	0	0	0	0	0
0	0	0	0	0	0	0	0	0	0	0	0	0	0	0	0	0	0	0
54	0	0	0	3	0	0	0	0	8	0	0	0	0	0	0	0	0	0
0	0	0	0	0	0	0	0	0	0	0	0	0	0	0	0	0	0	0
0	0	0	0	0	0	0	0	0	0	0	0	0	0	0	0	0	0	0
0	0	0	0	0	0	0	0	0	0	0	0	0	0	0	0	0	0	0
0	0	0	0	0	0	0	10	0	0	0	0	0	0	0	5	0	18	0
6	0	0	0	0	0	0	0	0	0	0	0	0	0	0	0	0	0	0
0	0	0	0	0	0	0	0	0	0	0	0	0	0	0	0	0	0	0
0	0	0	0	0	0	0	2	0	0	0	0	0	0	0	0	0	0	0
0	0	0	0	0	0	0	0	0	0	0	0	0	0	0	0	0	0	0
0	0	0	0	0	0	0	0	0	0	3	0	0	0	0	0	0	38	0
0	0	0	0	0	0	0	0	0	0	0	0	0	0	0	0	0	0	0
0	0	0	0	0	0	0	0	0	0	2	0	0	0	0	0	0	0	0
0	0	5	0	0	0	0	0	0	0	0	0	0	0	0	0	0	0	0
0	0	0	0	0	38	0	0	0	0	0	0	0	0	0	0	0	0	0

0	0	0	0	0	0	23	10	0	0	0	0	0	0	0	0	0	0	0
0	0	0	0	0	0	0	0	0	0	0	0	0	0	0	0	0	0	0
0	0	0	0	0	0	0	0	0	0	0	0	0	0	0	0	0	0	0
0	0	0	0	0	0	0	0	0	0	0	0	0	0	0	0	0	0	0
0	0	0	4	0	0	0	0	0	0	0	0	0	0	0	0	0	0	0
0	0	0	0	0	0	0	0	0	0	0	0	0	0	0	0	0	0	0
0	0	0	0	0	0	0	0	0	0	0	0	0	0	0	0	0	0	0
0	16	0	0	0	0	0	0	0	0	0	0	0	0	0	0	0	0	0
0	0	0	0	0	0	0	0	0	0	0	0	4	0	0	0	0	0	0
0	0	0	0	0	0	0	0	0	0	0	0	0	0	0	0	0	0	0
0	0	0	0	0	0	0	0	0	0	0	0	0	0	0	0	0	0	0
0	0	0	0	0	0	0	0	0	0	0	0	0	0	0	0	0	0	0
0	0	0	0	0	0	0	0	0	0	0	0	4	0	0	0	0	0	0
0	0	0	0	0	0	0	0	0	17	0	0	0	0	0	0	0	0	0
0	0	0	0	0	0	0	0	0	0	0	0	0	0	0	0	0	0	0
0	0	0	0	8	0	0	0	0	0	0	0	0	0	0	0	0	0	15
15	0	0	0	0	0	0	0	0	0	0	0	0	0	0	0	0	0	0
0	0	0	0	0	0	0	0	0	0	0	0	0	0	0	0	0	0	0
0	0	0	0	0	0	0	0	0	0	0	0	0	0	0	0	0	0	0
0	0	0	0	13	0	0	0	0	0	0	0	0	0	0	0	0	0	0
0	0	0	0	0	0	0	0	0	0	0	0	0	0	0	0	0	0	0
0	0	0	0	0	0	0	12	0	0	0	0	6	0	0	0	0	0	0
0	0	0	0	0	0	0	0	0	0	0	0	0	0	0	0	0	0	0
0	0	8	0	0	0	0	0	0	0	0	0	0	0	0	0	0	0	0
0	0	0	0	0	0	0	0	0	0	0	0	0	0	0	0	0	0	0
0	0	0	0	0	0	0	0	0	0	0	0	0	0	0	0	0	0	0
0	0	0	0	0	0	0	0	0	0	0	0	0	0	0	0	0	0	0
0	0	0	0	0	0	0	0	0	0	0	0	0	0	0	0	0	0	0
0	0	0	0	0	0	0	0	0	0	9	9	9	0	0	0	0	0	0
0	0	0	0	0	0	0	0	0	0	0	0	0	0	0	0	0	0	0
0	0	0	0	0	0	0	0	0	0	0	0	0	0	0	0	0	0	0
0	0	0	0	0	0	0	0	0	0	0	0	0	0	0	0	0	0	0
0	0	0	0	0	0	0	0	0	8	0	0	0	0	0	0	0	0	0
0	0	0	0	0	0	0	0	0	0	0	0	0	0	0	0	0	0	0
0	0	0	0	0	0	0	0	0	0	0	0	0	0	0	0	0	0	0
0	0	0	0	0	0	0	0	0	0	0	0	0	0	0	0	0	0	0
0	0	0	0	0	0	0	0	0	0	0	0	0	0	0	0	0	0	0

0	0	0	0	0	0	0	7	0	0	0	0	0	0	0	0	0	0	0
0	0	0	0	0	0	0	0	0	0	0	0	0	0	0	0	0	0	0
0	0	0	0	0	0	0	0	0	0	0	0	0	0	0	0	0	0	0
0	0	0	0	0	0	0	0	0	0	0	0	0	0	0	0	0	0	0
0	0	0	0	0	0	0	0	0	0	0	0	0	0	0	0	0	0	0
0	0	0	0	0	0	0	0	0	0	0	0	0	0	0	0	0	0	0
0	0	0	0	0	0	0	0	0	0	0	0	0	6	0	0	0	0	0
0	0	0	0	0	0	0	0	0	0	0	0	0	0	0	0	0	0	0
0	0	0	0	0	0	0	0	0	0	0	0	0	0	0	0	0	0	0
0	0	0	0	0	0	0	0	0	0	0	0	0	0	0	0	0	0	0
0	0	0	0	0	0	0	0	0	0	0	0	0	0	0	0	0	0	0
0	0	0	0	0	0	0	0	0	0	0	0	0	0	0	0	0	0	0
0	0	0	0	0	0	0	0	0	0	0	0	0	0	0	0	0	0	0
0	0	0	0	3	0	0	0	0	0	0	0	0	5	5	0	0	0	0
0	0	0	0	0	0	0	0	0	0	0	0	0	0	0	0	0	0	0
0	0	0	0	0	0	0	0	0	0	0	0	0	0	0	0	0	0	0
0	0	0	0	0	0	0	0	0	0	0	0	0	0	0	0	0	0	0
0	0	0	0	0	0	0	0	0	0	0	0	0	0	0	0	0	0	0
0	0	0	0	0	0	0	0	0	0	0	0	0	0	0	0	0	0	0
0	0	0	0	0	0	0	0	0	0	0	0	0	0	0	0	0	0	0
0	0	0	0	0	0	0	0	0	0	0	0	0	0	0	0	0	0	0
0	0	0	0	0	0	0	0	0	0	0	0	0	0	0	0	0	0	0
0	0	0	0	0	0	0	0	0	0	0	0	0	0	0	0	0	0	0
0	0	0	0	0	0	0	0	0	0	0	0	0	0	0	0	0	0	0
0	0	0	0	0	0	0	0	0	0	0	0	0	0	0	0	4	4	0
0	0	0	0	0	0	0	0	0	0	0	0	0	0	0	0	0	0	0
0	0	0	0	0	0	0	0	0	0	0	0	0	0	0	0	0	0	0
0	0	0	0	0	0	0	0	0	0	0	0	0	0	0	0	0	0	0
0	0	0	0	0	0	0	0	0	0	0	0	0	0	0	0	0	0	0
0	0	0	0	0	0	0	0	0	0	0	0	0	0	0	0	0	0	0
0	0	0	0	0	0	0	0	0	0	0	0	0	0	0	0	0	0	0
0	0	0	0	0	0	0	0	0	0	0	0	0	0	0	0	0	0	0
0	0	0	0	0	0	0	0	0	0	0	0	0	0	0	0	0	0	0
0	0	0	0	0	0	0	0	0	0	0	0	0	0	0	0	0	0	0
0	0	0	0	0	0	0	0	0	0	0	0	0	0	0	0	0	0	0
0	0	0	0	0	0	0	0	0	0	0	0	0	0	0	0	0	0	0
0	0	0	0	0	0	0	0	0	0	0	0	0	0	0	0	0	0	0

0	0	0	0	0	0	0	0	0	0	0	0	0	0	0	0	0	0	0
0	0	0	0	0	0	0	0	0	0	0	0	0	0	0	0	0	0	0
0	0	0	0	0	0	0	0	0	0	0	0	0	0	0	0	0	0	0
3	3	3	3	3	3	0	0	0	0	0	0	0	0	0	0	0	0	0
0	0	0	0	0	0	0	0	0	0	0	0	0	0	0	0	0	0	0
0	0	0	0	0	0	0	0	0	0	0	0	0	0	0	0	0	0	0
0	0	0	0	0	0	0	0	0	0	0	0	0	0	0	0	0	0	0
0	0	0	0	0	0	0	0	0	0	0	0	0	0	0	0	0	0	0
0	0	0	0	0	0	0	0	0	0	0	0	0	0	0	0	0	0	0
0	0	0	0	0	0	0	0	0	0	0	0	0	0	0	0	0	0	0
0	0	0	0	0	0	0	0	0	0	0	0	0	0	0	0	0	0	0
0	0	0	0	0	0	0	0	0	0	0	0	0	0	0	0	0	0	0
0	0	0	0	0	0	0	0	0	0	0	0	0	0	0	0	0	0	0
0	0	0	0	0	0	0	0	0	0	0	0	0	0	0	0	0	0	0
0	0	0	0	0	0	0	0	0	0	0	0	0	0	0	0	0	0	0
0	0	0	0	0	0	0	0	0	0	0	0	0	0	0	0	0	0	0
0	0	0	0	0	0	0	0	0	0	0	0	0	0	0	0	0	0	0
0	0	0	0	0	0	0	0	0	0	0	0	0	0	0	0	0	0	0
0	0	0	0	0	0	0	0	0	0	0	0	0	0	0	0	0	0	0
0	0	0	0	0	0	0	0	0	0	0	0	0	0	0	0	0	0	0
0	0	0	0	0	0	0	0	0	0	0	0	0	0	0	0	0	0	0
0	0	0	0	0	0	0	0	0	0	0	0	0	0	0	0	0	0	0
0	0	0	0	0	0	0	0	0	0	0	0	0	0	0	0	0	0	0
0	0	0	0	0	0	0	0	0	0	0	0	0	0	0	0	0	0	0
0	0	0	0	0	0	0	0	0	0	0	0	0	0	0	0	0	0	0
0	0	0	0	0	0	0	0	0	0	0	0	0	0	0	0	0	0	0
0	0	0	0	0	0	0	0	0	0	0	0	0	0	0	0	0	0	0
0	0	0	0	0	0	0	0	0	0	0	0	0	0	0	0	0	0	0
0	0	0	0	0	0	0	0	0	0	0	0	0	0	0	0	0	0	0
0	0	0	0	0	0	0	0	0	0	0	0	0	0	0	0	2	2	2
2	0	0	0	0	0	0	0	0	0	0	0	0	0	0	0	0	0	0
0	0	0	0	0	0	0	0	0	0	0	0	0	0	0	0	0	0	0
0	0	0	0	0	0	0	0	0	0	0	0	0	0	0	0	0	0	0
0	0	0	0	0	0	0	0	0	0	0	0	0	0	0	0	0	0	0
0	0	0	0	0	0	0	0	0	0	0	0	0	0	0	0	0	0	0
0	0	0	0	0	0	0	0	0	0	0	0	0	0	0	0	0	0	0
0	0	0	0	0	0	0	0	0	0	0	0	0	0	0	0	0	0	0

0	0	0	0	0	0	0	0	0	0	0	0	0	0	0	0	0	0	0
0	0	0	0	0	0	0	0	0	0	0	0	0	0	0	0	0	0	0
0	0	0	0	0	0	0	0	0	0	0	0	0	0	0	0	0	0	0
0	0	0	0	0	0	0	0	0	0	0	0	0	0	0	0	0	0	0
0	0	0	0	0	0	0	0	0	0	0	0	0	0	0	0	0	0	0
0	0	0	0	0	0	0	0	0	0	0	0	0	0	0	0	0	0	0
0	0	0	0	0	0	0	0	0	0	0	0	0	0	0	0	0	0	0
0	0	0	0	0	0	0	0	0	0	0	0	0	0	0	0	0	0	0
0	0	0	0	0	0	0	0	0	0	0	0	0	0	0	0	0	0	0
0	0	0	0	0	0	0	0	0	0	0	0	0	0	0	0	0	0	0
0	0	0	0	0	0	0	0	0	0	0	0	0	0	0	0	0	0	0
0	0	0	0	0	0	0	0	0	0	0	0	0	0	0	0	0	0	0
0	0	0	0	0	0	0	0	0	0	0	0	0	0	0	0	0	0	0
0	0	0	0	0	0	0	0	0	0	0	0	0	0	0	0	0	0	0
0	0	0	0	0	0	0	0	0	0	0	0	0	0	0	0	0	0	0
0	0	0	0	0	0	0	0	0	0	0	0	0	0	0	0	0	0	0
0	0	0	0	0	0	0	0	0	0	0	0	1	0	0	0	0	0	0
0	0	0	0	0	0	0	0	0	0									
Jan_DL101	1276	1364	699	232	506	191	158	169	101	193	103	308	157	132	89	296	128	129
143	153	188	86	73	159	191	78	153	91	86	108	74	132	69	42	57	13	95
82	0	152	35	54	47	79	178	91	85	63	139	28	45	60	100	60	53	48
0	29	117	179	56	373	19	40	39	18	109	86	407	0	10	74	31	30	0
58	0	29	47	34	67	19	22	0	0	0	0	43	0	26	0	15	29	133
45	24	0	0	31	8	0	0	0	13	0	0	0	0	23	0	0	0	23
0	0	0	5	15	0	0	0	40	0	25	13	12	9	0	0	0	0	0
0	0	0	0	33	0	0	0	0	13	0	0	14	0	33	0	0	0	0
0	26	23	10	0	11	0	0	0	0	14	0	15	28	0	23	0	39	0
0	0	0	0	13	0	0	0	0	0	23	16	0	0	0	12	11	12	0
0	0	12	0	0	0	0	11	0	4	0	41	0	0	5	0	0	23	0
37	21	0	0	0	0	8	0	0	0	0	22	20	0	0	22	0	4	0
0	0	9	24	0	0	0	0	0	0	0	0	20	5	0	9	9	0	0
0	0	0	0	0	0	0	18	0	0	0	0	0	0	8	0	0	0	0
0	13	0	28	0	5	0	0	26	0	0	0	9	10	6	14	0	0	0
0	17	0	0	0	0	0	0	0	0	0	11	0	0	0	0	0	0	26
0	0	0	0	0	0	0	0	0	11	22	0	0	0	0	0	0	10	0
0	0	15	0	0	0	0	14	0	32	0	0	0	0	13	8	17	0	0
0	0	0	0	0	0	0	0	8	14	0	0	0	0	0	0	13	0	14

8	0	0	0	0	0	0	0	0	0	0	0	11	0	0	0	0	0	0
49	0	0	0	12	0	5	0	0	0	0	12	0	11	0	0	0	0	0
0	0	0	24	0	7	0	0	6	14	0	0	0	0	0	0	0	0	0
0	0	0	0	0	0	0	0	0	0	0	0	8	0	0	0	0	0	0
0	7	0	0	0	0	0	0	0	0	0	0	0	0	0	0	0	0	0
0	0	0	0	0	0	0	0	0	0	0	0	99	0	0	0	0	0	0
0	0	0	0	0	33	0	0	0	0	25	0	0	9	0	0	0	0	0
5	0	0	0	0	0	0	0	0	0	0	0	0	0	0	0	0	8	0
0	0	0	0	0	0	0	0	0	0	0	0	0	0	0	0	0	0	0
0	0	0	0	0	0	0	0	0	0	0	0	0	15	0	0	0	0	0
0	0	0	0	0	0	0	0	0	0	0	0	0	0	0	0	0	0	0
0	0	0	0	0	0	6	0	0	0	0	0	0	4	0	0	0	0	0
0	14	0	0	0	0	0	0	0	0	0	0	0	0	0	0	0	0	0
0	0	5	0	0	0	0	0	0	0	0	0	0	0	0	5	0	0	0
0	0	0	0	0	0	0	0	0	0	0	0	0	0	0	0	0	0	0
0	0	0	0	0	0	0	0	0	8	12	0	0	0	0	0	0	0	0
0	0	0	0	0	0	0	0	10	0	0	21	0	0	0	0	0	0	0
0	0	0	0	0	0	0	0	0	0	0	0	0	0	0	0	0	0	0
0	0	0	8	0	0	0	0	0	0	0	0	0	0	0	0	0	0	0
4	0	0	0	0	0	0	0	0	0	0	8	0	0	0	0	0	0	0
0	0	0	0	0	0	0	0	0	0	0	0	0	0	0	0	0	0	0
0	0	0	0	0	19	0	0	0	0	0	0	0	0	0	0	0	0	0
0	0	0	0	0	0	0	0	0	0	0	0	0	0	0	0	0	0	0
0	0	0	0	0	0	0	0	0	6	0	0	0	0	0	0	0	0	0
0	0	0	0	0	0	0	0	0	0	29	0	0	0	0	0	0	0	0
0	0	0	0	0	0	0	0	0	0	0	0	0	0	0	0	0	0	0
0	0	0	0	0	0	0	0	0	0	0	0	0	0	0	0	0	0	0
0	0	0	0	0	0	0	9	0	0	0	0	0	0	0	0	0	0	0
0	0	0	0	0	0	0	0	0	0	0	0	0	0	0	0	0	0	0
0	0	0	0	0	0	0	0	0	0	0	0	0	0	0	0	0	0	0
0	0	0	0	0	0	0	0	33	0	0	0	0	0	0	0	0	0	0
0	0	0	0	0	0	0	0	0	0	0	0	0	0	0	0	0	0	0
0	0	0	0	0	0	0	0	0	0	0	0	0	0	0	0	0	0	0
0	0	0	0	0	0	0	0	0	0	0	0	0	0	0	0	0	0	0
0	0	0	0	0	0	0	0	0	0	0	0	0	0	0	0	0	0	0
0	0	0	0	0	0	0	0	0	0	0	0	0	0	0	0	0	0	0
0	0	0	0	0	0	0	0	0	0	6	0	0	0	0	0	0	0	0

0	0	0	0	0	0	0	0	22	0	0	0	0	0	0	0	0	0	0
0	0	0	0	0	0	0	0	0	0	0	0	0	0	0	0	0	0	0
0	0	0	0	0	0	0	0	0	0	19	19	0	0	0	0	0	0	0
0	0	0	0	0	0	0	0	0	0	18	0	0	0	0	0	0	0	0
0	0	0	0	0	5	0	0	0	0	0	0	0	0	0	0	0	0	0
0	0	0	0	0	0	0	0	0	0	0	4	0	0	0	0	0	0	0
0	0	0	0	0	0	0	0	0	0	0	0	0	0	0	0	15	0	0
0	0	0	0	0	0	0	0	0	0	0	0	0	0	0	0	0	0	0
0	0	0	0	0	0	0	0	0	0	0	0	0	0	0	0	0	0	0
0	0	0	0	0	0	0	0	0	0	0	0	0	0	0	0	4	0	0
0	0	0	0	0	0	0	0	0	0	0	0	0	0	0	0	0	0	0
0	0	0	0	0	0	0	0	0	0	0	0	0	0	0	12	12	0	0
0	0	0	0	0	0	0	0	0	0	0	0	0	0	0	0	0	0	0
0	0	0	0	0	0	0	0	0	0	0	0	0	0	0	0	0	0	0
0	0	11	0	0	0	0	0	0	0	0	0	0	0	0	0	0	0	0
0	0	0	0	0	0	0	0	0	0	0	0	0	0	0	0	0	0	0
0	0	0	0	0	0	0	0	0	0	0	0	0	0	0	0	0	10	0
0	0	0	0	0	5	0	0	0	0	0	0	0	0	0	0	0	0	0
0	0	0	0	0	0	0	0	0	0	0	0	0	0	0	0	0	0	0
0	0	0	0	0	0	0	0	0	0	0	0	0	0	0	0	0	0	0
0	0	0	0	0	0	0	0	0	0	0	0	0	0	0	0	0	0	0
0	0	0	0	0	0	0	0	0	0	0	0	0	0	0	0	0	0	0
0	0	0	0	0	0	0	0	0	0	0	0	0	0	0	0	0	0	0
0	0	0	0	0	0	0	0	0	0	0	0	0	0	0	0	0	0	0
0	0	0	0	0	0	0	0	0	0	0	0	0	0	0	0	0	0	0
0	0	0	0	0	0	0	0	0	0	0	0	0	0	0	0	0	0	0
0	0	0	0	0	0	0	0	0	0	0	0	0	0	0	0	7	7	0
0	0	0	0	0	0	0	0	0	0	0	0	0	0	0	0	0	0	0
0	0	0	0	0	0	0	0	0	0	0	0	0	0	0	0	0	0	0
0	0	0	0	0	0	0	0	0	0	0	0	0	0	0	0	0	0	0
0	0	0	0	0	0	0	0	0	0	0	0	0	0	0	0	0	0	0
0	0	0	0	0	0	0	0	0	0	0	0	0	0	0	0	0	0	0
0	0	0	0	0	0	0	0	0	0	0	0	0	0	0	0	0	0	0
0	0	0	0	0	0	0	0	0	0	0	0	0	0	0	0	0	0	0
0	0	0	0	0	0	0	0	0	0	0	0	0	0	0	0	0	0	0
0	0	0	0	0	0	0	0	0	0	0	0	0	0	0	0	0	0	0
0	0	0	0	0	0	0	0	0	0	0	0	0	0	0	0	0	0	0

0	0	0	0	0	0	0	0	0	0	0	0	0	0	0	0	0	0	0
0	0	0	0	0	0	0	0	0	0	0	0	0	0	0	0	0	0	0
0	0	0	0	0	0	0	0	0	0	0	0	0	0	0	0	0	0	0
0	0	0	0	0	0	0	0	0	0	0	0	0	0	0	0	0	0	0
0	0	5	5	0	0	0	0	0	0	0	0	0	0	0	0	0	0	0
0	0	0	0	2	0	0	0	0	0	0	0	0	0	0	0	0	0	0
0	0	0	0	0	0	0	0	0	0	0	0	0	0	0	0	0	0	0
0	0	0	0	0	0	0	0	0	0	0	0	0	0	0	0	0	0	0
0	0	0	0	0	0	0	0	0	0	0	0	0	0	0	0	0	0	0
0	0	0	0	0	0	0	0	0	0	0	0	0	0	0	0	0	0	0
0	0	0	0	0	0	0	0	0	0	0	0	0	0	0	0	0	0	0
0	0	0	0	0	0	0	0	0	0	0	0	0	0	0	0	0	0	0
0	0	0	0	0	0	0	0	0	0	0	0	0	0	0	0	0	0	0
0	0	0	0	0	0	0	0	0	0	0	0	0	0	0	0	0	0	0
0	0	0	0	0	0	0	0	0	0	0	0	0	0	0	0	0	0	0
0	0	0	0	0	0	0	0	0	0	0	0	0	0	0	0	0	0	0
0	0	0	0	0	0	0	0	0	0	0	0	0	0	0	0	0	0	0
0	0	0	0	0	0	0	0	0	0	0	0	0	0	0	0	0	0	0
0	0	0	0	0	0	0	0	0	0	0	0	0	0	0	0	0	0	0
0	0	0	0	0	0	0	0	0	0	0	0	0	0	0	0	0	0	0
0	0	0	0	0	0	0	0	0	0	0	0	0	0	0	0	0	0	0
0	0	0	0	0	0	0	0	0	0	0	0	0	0	0	0	0	0	0
0	0	0	0	0	0	0	0	0	0	0	0	0	0	0	0	0	0	0
0	0	0	0	0	0	0	0	0	0	0	0	0	0	0	0	0	0	0
0	0	0	0	0	0	0	0	0	0	0	0	0	0	0	0	0	0	0
0	0	0	0	0	0	0	0	0	0	0	0	0	0	0	0	0	0	0
0	0	0	0	0	0	0	0	0	0	0	0	0	0	0	0	0	0	0
0	0	0	0	0	0	0	0	0	0	0	0	0	0	0	0	0	0	0
0	0	0	0	0	0	0	0	0	0	0	0	0	0	0	0	0	0	0
0	0	0	0	0	0	0	0	0	0	0	0	0	0	0	0	0	0	0
0	0	0	0	0	0	0	0	0	0	0	0	0	0	0	0	0	0	0
0	0	0	0	0	0	0	0	0	0	0	0	0	0	0	0	0	0	0
0	0	0	0	0	0	0	0	0	0	0	0	0	0	0	0	0	0	0
0	0	0	0	0	0	0	0	0	0	0	0	0	0	0	0	0	0	0
0	0	0	0	0	0	0	0	0	0	0	0	0	0	0	0	0	0	0
0	0	0	0	0	0	0	0	0	0	0	0	0	0	0	0	0	0	0
0	0	0	0	0	0	0	0	0	0	0	0	0	0	0	0	0	0	0

0	0	0	0	0	0	0	0	0	0	0	0	0	0	0	0	0	0	0
0	0	0	0	0	0	0	0	0	0	0	0	0	0	0	0	0	0	0
0	0	0	0	0	0	0	0	0	0	0	0	0	0	0	0	0	0	0
0	0	0	0	0	0	0	0	0	0	0	0	0	0	0	0	0	0	0
0	0	0	0	0	0	0	0	0	0	0	0	0	0	0	0	0	0	0
0	0	0	0	0	0	0	0	0	0	0	0	0	0	0	0	0	0	0
0	0	0	0	0	0	0	0	0	0	0	0	0	0	0	0	0	0	0
0	0	0	0	0	0	0	0	0	0	0	0	0	0	0	0	0	0	0
0	0	0	0	0	0	0	0	0	0	0	0	0	0	0	0	0	0	0
0	0	0	0	0	0	0	0	0	0	0	0	0	0	0	0	0	0	0
0	0	0	0	0	0	0	0	0	0	0	0	0	0	0	0	0	0	0
0	0	0	0	0	0	0	0	0	0	0	0	0	0	0	0	0	0	0
0	0	0	0	0	0	0	0	0	0	0	0	0	0	0	0	0	0	0
0	0	0	0	0	0	0	0	0	0	0	0	0	0	0	0	0	0	0
0	0	0	0	0	0	0	0	0	0	0	0	0	0	0	0	0	0	0
0	0	0	0	0	0	0	0	0	0	0	0	0	0	0	0	0	0	0
0	0	0	0	0	0	0	0	0	0	0	0	0	0	0	0	0	0	0
0	0	0	0	0	0	0	0	0	0	0	0	2	2	0	0	0	0	0
0	0	0	0	0	0	0	0	0	0	0	0	0	0	0	0	0	0	0
0	0	0	0	0	0	0	0	0	0	0	0	0	0	0	0	0	0	0
0	0	0	0	0	0	0	0	0	0	0	0	0	0	0	0	0	0	0
0	0	0	0	0	0	0	0	0	0	0	0	0	0	0	0	0	0	0
0	0	0	0	0	0	0	0	0	0	0	0	0	0	0	0	0	0	0
0	0	0	0	0	0	0	0	0	0	0	0	0	0	0	0	0	0	0
0	0	0	0	0	0	0	0	0	0	0	0	0	0	0	0	0	0	0
0	0	0	0	0	0	0	0	0	0	0	0	0	0	0	0	0	0	0
0	0	0	0	0	0	0	0	0	0	0	0	0	0	0	0	0	0	0
0	0	0	0	0	0	0	0	0	0	0	0	0	0	0	0	0	0	0
0	0	0	0	0	0	0	0	0	0	0	0	0	0	0	0	0	0	0
0	0	0	0	0	0	0	0	0	0	0	0	0	0	0	0	0	0	0
0	0	0	0	0	0	0	0	0	0	0	0	0	0	0	0	0	0	0
0	0	0	0	0	0	0	0	0	0	0	0	0	0	0	0	0	0	0
0	0	0	0	0	0	0	0	0	0	0	0	0	0	0	0	0	0	0
0	0	0	0	0	0	0	0	0	0	0	0	0	0	0	0	0	0	0
0	0	0	0	0	0	0	0	0	0	0	0	0	0	0	0	0	0	0
0	0	0	0	0	0	0	0	0	0	0	0	0	0	0	0	0	0	0
0	0	0	0	0	0	0	0	0	0	0	0	0	0	0	0	0	0	0

0	0	0	0	0	0	0	0	0	0	0	0	0	0	0	0	0	0	0
0	0	0	0	0	0	0	0	0	0	0	0	0	0	0	0	0	0	0
0	0	0	0	0	0	0	0	0	0	0	0	0	0	0	0	0	0	0
0	0	0	0	0	0	0	0	0	0	0	0	0	0	0	0	0	0	0
0	0	0	0	0	0	0	0	0	0	0	0	0	0	0	0	0	0	0
0	0	0	0	0	0	0	0	0	0	0	0	0	0	0	0	0	0	0
0	0	0	0	0	0	0	0	0	0	0	0	0	0	0	0	0	0	0
0	0	0	0	0	0	0	0	0	0	0	0	0	0	0	0	0	0	0
0	0	0	0	0	0	0	0	0	0	0	0	0	0	0	0	0	0	0
0	0	0	0	0	0	0	0	0	0									
Jan_DL102	397	658	682	388	32	0	39	34	93	208	381	209	177	31	180	221	25	41
30	206	102	12	61	76	188	139	262	160	54	0	78	193	90	187	95	564	0
126	64	0	34	81	143	0	407	0	74	0	112	30	16	0	109	73	0	0
0	136	131	12	0	0	0	72	120	102	29	0	23	0	17	0	21	38	0
41	85	0	0	0	39	38	0	56	0	0	58	16	69	17	53	23	0	0
0	40	0	0	71	0	60	0	0	30	0	0	0	0	15	0	0	83	0
0	0	0	20	4	0	0	0	0	0	0	10	0	0	32	0	0	0	0
0	0	0	0	0	66	0	0	0	8	0	0	0	0	0	14	0	0	0
52	0	0	0	0	0	13	53	0	44	0	0	0	0	0	0	0	0	6
31	0	0	0	0	0	55	0	0	0	0	0	0	15	0	0	7	0	0
0	0	0	0	51	0	0	0	0	0	0	0	0	0	4	64	0	0	0
0	0	0	0	0	0	0	0	0	0	0	0	0	0	0	0	0	0	18
0	0	0	0	0	0	0	44	0	10	0	0	0	10	0	0	0	42	0
0	0	0	0	0	0	5	0	0	13	0	59	0	0	0	0	0	0	0
10	0	0	0	0	0	21	0	0	0	0	0	0	0	0	0	0	0	0
0	0	7	0	0	0	0	0	6	0	24	0	0	0	0	0	0	0	0
0	4	11	0	0	0	0	0	0	0	0	0	0	6	0	6	0	0	6
0	0	0	0	0	0	0	0	0	0	0	0	0	0	0	4	0	0	0
0	0	0	0	0	0	0	0	0	0	0	6	10	0	0	0	0	0	0
0	0	0	0	13	0	0	0	0	0	0	0	0	0	0	11	0	0	0
0	0	6	0	0	0	0	0	0	0	12	0	0	0	0	0	0	0	0
0	0	0	0	0	0	15	2	0	0	0	0	0	0	15	17	0	0	0
0	0	0	0	0	0	0	0	0	0	0	0	0	0	0	0	0	0	0
0	6	0	0	0	0	0	0	0	0	0	0	0	0	0	23	0	0	0
0	0	0	0	0	0	0	0	0	0	0	21	0	0	0	0	0	0	0
0	0	0	0	3	0	0	0	0	0	0	0	0	0	0	11	0	0	0
0	0	0	0	0	0	0	0	0	0	0	0	0	0	0	0	0	0	0

0	0	0	0	0	0	0	0	0	0	0	45	0	0	0	0	0	0	0
0	0	0	0	0	4	0	0	0	0	0	0	0	0	12	0	0	0	0
0	0	0	0	0	0	0	0	0	0	0	0	0	0	0	0	0	0	0
0	0	0	0	0	0	0	0	0	0	0	0	10	0	0	0	0	0	0
0	0	0	0	0	0	0	0	0	0	0	0	0	0	0	0	0	0	0
0	0	0	0	0	0	0	0	0	0	0	0	8	0	0	0	0	0	0
0	0	0	0	0	0	0	0	0	0	0	0	0	0	0	0	0	0	0
0	0	0	0	0	16	0	0	0	0	0	0	0	0	0	0	0	0	0
0	0	0	0	0	0	0	0	0	0	0	0	0	0	0	0	0	0	0
0	0	0	0	0	0	0	0	0	0	6	0	0	0	0	0	0	0	0
0	0	0	0	0	0	22	0	0	0	0	0	0	0	0	0	0	0	0
0	44	0	0	0	0	0	0	0	0	0	0	0	0	0	0	0	0	0
0	0	0	0	0	0	0	0	0	0	0	0	0	0	0	0	0	0	0
0	0	0	0	0	0	0	0	0	0	0	0	0	0	0	0	0	0	0
0	0	0	0	0	0	0	0	0	0	0	0	0	0	0	0	0	9	0
0	0	0	0	0	0	0	10	0	0	0	0	0	0	0	0	0	0	0
0	0	0	0	0	0	0	0	0	0	0	0	0	0	0	0	0	0	0
0	0	0	0	0	0	0	0	0	0	0	0	0	0	0	0	0	0	0
0	0	0	0	0	0	0	0	0	33	0	0	0	0	0	0	0	0	0
3	0	0	0	0	31	0	0	0	0	0	0	0	0	0	30	0	0	0
0	0	0	0	0	0	0	0	0	0	0	0	0	0	0	0	0	0	0
0	0	0	0	0	0	0	0	0	0	0	0	0	0	0	0	0	0	0
0	0	0	0	0	0	0	0	0	0	0	0	0	0	0	0	0	0	0
24	0	0	0	0	0	0	0	0	0	0	0	0	0	0	0	0	0	0
0	0	0	0	0	0	0	0	0	0	0	0	0	0	0	0	0	0	0
0	0	0	0	0	0	0	0	0	0	0	0	0	0	0	0	0	0	0
20	0	0	0	0	0	0	0	0	0	0	0	0	0	0	0	0	0	0
0	0	0	0	0	0	0	0	0	0	0	0	0	0	0	0	0	0	0
0	0	0	0	0	0	0	0	6	0	0	0	0	0	0	0	0	0	0
0	0	0	0	0	0	0	0	0	0	0	0	0	0	0	0	0	0	0
16	0	0	0	0	0	0	0	0	0	0	0	0	0	0	0	0	0	0
0	0	0	0	0	0	0	0	0	0	0	0	0	0	0	0	0	0	0
0	0	0	0	0	0	0	0	0	0	0	0	0	0	0	0	0	0	0
0	0	0	0	0	0	0	0	0	0	0	0	0	0	0	0	0	0	0
0	0	0	0	0	0	0	0	0	0	0	0	0	0	0	0	0	0	0
0	0	0	0	0	0	0	0	0	0	0	0	0	0	0	0	0	0	0
0	0	0	0	0	0	0	0	0	0	0	0	0	0	0	0	0	0	0

0	0	0	0	0	0	0	0	0	0	0	0	0	0	0	0	0	0	0
0	0	0	0	10	10	0	0	0	0	0	0	0	0	0	0	0	0	0
0	0	0	0	0	0	0	0	0	0	0	0	0	0	0	0	0	0	0
0	0	0	0	0	0	0	0	0	0	0	0	0	0	0	0	0	0	0
0	0	0	0	0	0	0	3	0	0	0	0	0	0	0	0	0	0	0
0	0	0	0	0	0	0	0	0	0	0	0	0	0	0	0	0	0	0
0	0	0	0	0	0	0	0	0	0	0	0	0	0	0	0	0	0	0
0	0	0	0	0	0	0	0	0	0	0	0	0	0	0	0	0	0	0
0	0	0	0	0	0	0	0	0	0	0	0	0	0	0	0	0	0	0
0	0	0	0	0	0	0	0	0	0	0	0	0	0	0	0	0	0	0
0	0	0	0	7	0	0	0	0	0	0	0	0	0	0	0	0	0	0
0	0	0	0	0	0	0	0	0	0	0	0	0	0	0	0	0	0	0
0	0	0	0	0	0	0	0	0	0	0	0	0	0	0	0	0	0	0
0	0	0	0	0	0	0	0	0	0	0	0	0	0	0	0	0	0	0
0	0	0	0	0	0	0	0	0	0	0	0	0	0	0	0	0	0	0
0	0	0	0	0	0	0	0	0	0	0	0	0	0	0	0	0	0	0
0	0	0	0	0	0	0	0	0	0	0	0	0	0	0	0	0	0	0
0	0	0	0	0	0	0	0	0	0	0	0	0	0	0	0	0	0	0
0	0	0	0	0	0	0	0	0	0	0	0	0	0	0	0	0	0	0
0	0	0	0	0	0	0	0	0	0	0	0	0	0	0	0	0	0	0
0	0	0	0	0	0	0	0	0	0	0	0	0	0	0	0	0	0	0
0	0	0	0	0	0	0	0	0	0	5	0	0	0	0	0	0	0	0
0	0	0	0	0	0	0	0	0	0	0	0	0	0	0	0	0	0	0
0	0	0	0	0	0	0	0	0	0	0	0	0	0	0	0	0	0	0
0	0	0	0	0	0	0	0	0	0	0	0	0	0	0	0	0	0	0
0	0	0	0	0	0	0	0	0	0	0	0	0	0	0	0	0	0	0
0	0	0	0	0	0	0	0	0	0	0	0	0	0	0	0	0	0	0
0	0	0	0	0	0	0	0	0	0	0	0	0	0	0	0	0	0	0
0	0	0	0	0	0	0	0	0	0	0	0	0	0	0	0	0	0	0
0	0	0	0	0	0	0	0	0	0	0	0	0	0	0	0	0	4	4
4	0	0	0	0	0	0	0	0	0	0	0	0	0	0	0	0	0	0
0	0	0	0	0	0	0	0	0	0	0	0	0	0	0	0	0	0	0
0	0	0	0	0	0	0	0	0	0	0	0	0	0	0	0	0	0	0
0	0	0	0	0	0	0	0	0	0	0	0	0	0	0	0	0	0	0
0	0	0	0	0	0	0	0	0	0	0	0	0	0	0	0	0	0	0
0	0	0	0	0	0	0	0	0	0	0	0	0	0	0	0	0	0	0
0	0	0	0	0	0	0	0	0	0	0	0	0	0	0	0	0	0	0

0	0	0	0	0	0	0	0	0	0	0	0	0	0	0	0	0	0	0
0	0	0	0	0	0	0	0	0	0	0	0	0	0	0	0	0	0	0
0	0	0	0	0	0	0	0	0	0	0	0	0	0	0	0	0	0	0
0	0	0	0	0	0	0	0	0	0	0	0	0	0	0	0	0	0	0
0	0	0	0	0	0	0	0	0	0	0	0	0	0	0	0	0	0	0
0	0	0	0	0	0	0	0	0	0	0	0	0	0	0	0	0	0	0
0	0	0	0	0	0	0	3	3	3	0	0	0	0	0	0	0	0	0
0	0	0	0	0	0	0	0	0	0	0	0	0	0	0	0	0	0	0
0	0	0	0	0	0	0	0	0	0	0	0	0	0	0	0	0	0	0
0	0	0	0	0	0	0	0	0	0	0	0	0	0	0	0	0	0	0
0	0	0	0	0	0	0	0	0	0	0	0	0	0	0	0	0	0	0
0	0	0	0	0	0	0	0	0	0	0	0	0	0	0	0	0	0	0
0	0	0	0	0	0	0	0	0	0	0	0	0	0	0	0	0	0	0
0	0	0	0	0	0	0	0	0	0	0	0	0	0	0	0	0	0	0
0	0	0	0	0	0	0	0	0	0	0	0	0	0	0	0	0	0	0
0	0	0	0	0	0	0	0	0	0	0	0	0	0	0	0	0	0	0
0	0	0	0	0	0	0	0	0	0	0	0	0	0	0	0	0	0	0
0	0	0	0	0	0	0	0	0	0	0	0	0	0	0	0	0	0	0
0	0	0	0	0	0	0	0	0	0	0	0	0	0	0	0	0	0	0
0	0	0	0	0	0	0	0	0	0	0	0	0	0	0	0	0	0	0
0	0	0	0	0	0	0	0	0	0	0	0	0	0	0	0	0	0	0
0	0	0	0	0	0	0	0	0	0	0	0	0	0	0	0	0	0	0
0	0	0	0	0	0	0	0	0	0	0	0	0	0	0	0	0	0	0
0	0	0	0	0	0	0	0	0	0	0	0	0	0	0	0	0	0	0
0	0	0	0	0	0	0	0	0	0	0	0	0	0	0	0	0	0	0
0	0	0	0	0	0	0	0	0	0	0	0	0	0	0	0	0	0	0
0	0	0	0	0	0	0	0	0	0	0	0	0	0	0	0	0	0	0
0	0	0	0	0	0	0	0	0	0	0	0	0	0	0	0	0	0	0
0	0	0	2	2	2	2	2	2	2	0	0	0	0	0	0	0	0	0
0	0	0	0	0	0	0	0	0	0	0	0	0	0	0	0	0	0	0
0	0	0	0	0	0	0	0	0	0	0	0	0	0	0	0	0	0	0
0	0	0	0	0	0	0	0	0	0	0	0	0	0	0	0	0	0	0
0	0	0	0	0	0	0	0	0	0	0	0	0	0	0	0	0	0	0
0	0	0	0	0	0	0	0	0	0	0	0	0	0	0	0	0	0	0
0	0	0	0	0	0	0	0	0	0	0	0	0	0	0	0	0	0	0
0	0	0	0	0	0	0	0	0	0	0	0	0	0	0	0	0	0	0
0	0	0	0	0	0	0	0	0	0	0	0	0	0	0	0	0	0	0

0	0	0	0	0	0	0	0	0	0	0	0	0	0	0	0	0	0	0
0	0	0	0	0	0	0	0	0	0	0	0	0	0	0	0	0	0	0
0	0	0	0	0	0	0	0	0	0	0	0	0	0	0	0	0	0	0
0	0	0	0	0	0	0	0	0	0	0	0	0	0	0	0	0	0	0
0	0	0	0	0	0	0	0	0	0	0	0	0	0	0	0	0	0	0
0	0	0	0	0	0	0	0	0	0	0	0	0	0	0	0	0	0	0
0	0	0	0	0	0	0	0	0	0	0	0	0	0	0	0	0	0	0
0	0	0	0	0	0	0	0	0	0	0	0	0	0	0	0	0	0	0
0	0	0	0	0	0	0	0	0	0	0	0	0	0	0	0	0	0	0
0	0	0	0	0	0	0	0	0	0	0	0	0	0	0	0	0	0	0
0	0	0	0	0	0	0	0	0	0	0	0	0	0	0	0	0	0	0
0	0	0	0	0	0	0	0	0	0	0	0	0	0	0	0	0	0	0
0	0	0	0	0	0	0	0	0	0	0	0	0	0	0	0	0	0	0
0	0	0	0	0	0	0	0	0	0	0	0	0	0	0	0	0	0	0
0	0	0	0	0	0	0	0	0	0	0	0	0	0	0	0	0	0	0
0	0	0	0	0	0	0	0	0	0	0	0	0	0	0	0	0	0	0
0	0	0	0	0	0	0	0	0	0	0	0	0	0	0	0	0	0	0
0	0	0	0	0	0	0	0	0	0	0	0	0	0	0	0	0	0	0
0	0	0	0	0	0	0	0	0	0	0	0	0	0	0	0	0	0	0
0	0	0	0	0	0	0	0	0	0	0	0	0	0	0	0	0	0	0
0	0	0	0	0	0	0	0	0	0	0	0	0	0	0	0	0	0	0
0	0	0	0	0	0	0	0	0	0	0	0	0	0	0	0	0	0	0
0	0	0	0	0	0	0	0	0	0	0	0	0	0	0	0	0	0	0
0	0	0	0	0	0	0	0	0	0	0	0	0	0	0	0	0	0	0
0	0	0	0	0	0	0	0	0	0	0	0	0	0	0	0	0	0	0
0	0	0	0	0	0	0	0	0	0	0	0	0	0	0	0	0	0	0
0	0	0	0	0	0	0	0	0	0	0	0	0	0	0	0	0	0	0
0	0	0	0	0	0	0	0	0	0	0	0	0	0	0	0	0	0	0
0	0	0	0	0	0	0	0	0	0	0	0	0	0	0	0	0	0	0
0	0	0	0	0	0	0	0	0	0	0	0	0	0	0	0	0	0	0
0	0	0	0	0	0	0	0	0	0	0	0	0	0	0	0	0	0	0
0	0	0	0	0	0	0	0	0	0	0	0	0	0	0	0	0	0	0
0	0	0	0	0	0	0	0	0	0	0	0	0	0	0	0	0	0	0
0	0	0	0	0	0	0	0	0	0	0	0	0	0	0	0	0	0	0
0	0	0	0	0	0	0	0	0	0	0	0	0	0	0	0	0	0	0
0	0	0	0	0	0	0	0	0	0	0	0	0	0	0	0	0	0	0
0	0	0	0	0	0	0	0	0	0	0	0	0	0	0	0	0	0	0
0	0	0	0	0	0	0	0	0	0	0	0	0	0	0	0	0	0	0

0	0	0	0	0	0	0	0	0	0	0	0	0	0	0	0	0	0	0
0	0	0	0	0	0	0	0	0	0									
Jan_DL103	1399	1478	766	238	114	191	186	153	695	249	686	254	236	71	86	301	50	139
97	0	176	0	186	155	242	164	302	426	150	96	224	63	152	295	123	168	89
375	98	255	38	59	274	52	191	130	132	68	104	105	0	141	93	68	53	60
55	95	106	28	53	124	44	74	111	94	54	62	0	49	0	31	0	80	0
26	107	64	74	0	34	33	64	0	0	63	0	11	37	38	0	0	20	0
0	0	0	0	62	9	41	0	0	16	0	0	25	0	28	0	0	0	0
59	0	0	3	35	0	0	0	11	0	0	8	0	11	0	0	25	0	0
0	0	34	0	5	0	19	0	0	10	0	0	8	0	0	0	0	0	0
0	0	14	0	0	8	15	0	9	0	4	0	0	0	0	15	0	0	0
22	0	12	0	0	0	0	0	0	0	0	28	0	0	0	0	4	0	0
0	0	0	0	0	7	0	0	0	0	0	0	0	0	0	0	0	0	0
14	0	0	0	0	0	0	0	0	22	0	0	0	0	0	0	0	0	0
0	0	0	0	0	0	0	0	0	5	0	0	8	0	0	0	0	60	6
13	0	0	0	0	0	20	0	0	10	0	0	0	0	0	0	0	0	9
0	0	32	0	0	4	0	0	0	0	0	0	0	3	0	0	0	0	0
0	0	0	8	0	0	0	0	21	0	0	0	0	0	0	0	0	9	0
0	0	16	0	0	0	0	0	0	0	0	0	0	6	0	0	0	0	0
0	8	0	6	0	0	0	0	16	0	0	0	0	0	0	10	0	0	0
0	3	7	0	0	0	0	0	0	0	4	17	0	0	0	0	0	0	0
0	0	0	0	11	15	3	0	0	0	0	0	0	0	0	0	0	0	4
0	0	16	0	0	0	0	0	0	0	27	0	0	0	0	0	0	0	0
0	0	0	0	0	0	0	0	0	0	0	0	0	0	0	0	0	0	0
0	0	0	0	0	0	0	0	4	0	0	0	0	0	0	0	0	0	0
0	0	0	0	0	22	0	0	0	0	0	0	0	0	0	0	0	0	0
0	0	0	0	0	0	0	10	0	0	0	0	0	0	0	0	0	0	0
0	0	0	0	0	0	0	0	0	4	10	0	0	0	0	9	0	0	0
0	0	0	0	0	0	0	0	0	0	0	0	0	0	0	0	0	0	0
0	0	0	0	0	0	0	0	0	0	0	0	0	0	0	0	0	0	0
0	0	0	0	0	0	0	6	0	0	0	0	0	0	0	0	0	0	0
0	0	0	0	0	0	0	0	0	0	0	0	0	0	0	0	0	0	0
0	0	0	0	0	13	0	0	0	0	0	0	0	0	0	0	0	0	4
0	0	0	0	0	0	0	0	0	0	0	0	0	0	0	0	0	0	0
0	0	0	0	0	0	0	0	0	0	0	0	0	0	0	0	0	0	0
0	0	0	0	0	0	0	0	0	0	0	0	0	0	0	0	0	0	0
0	0	0	0	0	0	0	0	14	0	0	0	0	0	0	0	0	0	0

0	0	0	0	0	0	0	0	0	0	0	12	0	0	7	0	0	0	5
0	0	0	0	0	0	0	0	0	0	0	0	0	0	0	0	0	0	0
0	0	0	0	0	0	0	0	0	0	0	0	0	0	0	0	0	0	0
0	0	0	0	0	0	0	0	0	0	0	0	0	0	0	0	0	0	0
0	0	0	0	0	0	0	0	0	0	0	0	0	0	0	0	0	0	0
0	0	0	0	0	0	0	0	0	0	0	0	0	0	0	0	0	0	0
0	0	0	0	0	0	0	0	0	0	6	0	0	0	0	0	0	0	0
0	0	0	0	0	0	0	0	0	0	0	0	0	0	0	0	0	0	0
0	0	0	0	0	0	0	0	0	0	16	0	0	0	0	0	0	14	0
0	0	0	0	0	0	0	7	0	0	0	0	0	0	0	0	0	0	0
0	0	0	0	0	0	0	0	0	0	0	0	0	0	0	0	0	0	0
0	0	0	0	0	0	0	0	0	0	0	0	0	0	0	0	0	0	0
0	0	0	0	0	0	0	0	0	0	0	0	0	0	0	0	0	0	0
0	0	0	0	0	0	0	0	0	0	0	0	0	0	7	0	0	0	0
39	0	0	0	0	0	0	0	0	0	0	0	0	0	0	0	0	0	0
0	7	0	0	0	0	0	0	0	0	0	0	0	0	0	0	0	0	0
0	0	0	0	0	0	0	0	0	0	0	0	0	0	0	0	0	0	0
0	0	0	0	0	0	0	0	0	0	0	0	0	0	0	0	0	0	0
0	0	0	0	0	0	0	0	0	0	0	0	0	0	0	0	0	0	0
0	0	0	0	0	0	0	0	0	0	0	0	0	0	0	0	0	0	0
0	0	0	0	0	0	0	0	0	0	0	0	0	0	0	0	0	0	0
0	0	0	0	25	0	0	0	0	0	0	0	0	0	0	0	0	0	0
0	0	0	0	0	0	0	0	0	0	0	0	0	0	0	0	0	0	0
0	0	0	0	0	0	0	0	0	0	0	0	0	0	0	0	0	0	0
0	0	0	0	0	0	0	0	0	0	0	0	0	0	0	0	0	0	0
0	0	0	0	0	0	0	0	0	0	0	0	0	0	0	0	19	0	0
0	0	0	0	0	0	0	0	0	0	0	0	0	0	0	0	0	0	0
0	0	0	0	0	0	0	0	0	0	0	0	0	0	0	17	0	0	0
0	0	0	0	0	0	0	0	0	0	0	0	0	0	0	0	0	0	0
0	0	0	0	0	0	0	0	0	0	0	0	0	0	0	0	0	0	0
0	0	0	0	0	0	0	0	0	0	0	0	0	0	0	0	0	0	0
0	0	0	0	0	0	0	0	0	0	0	0	0	0	0	0	0	0	0
0	0	0	14	0	0	0	0	0	0	0	0	0	0	0	0	0	0	0
0	0	0	0	0	0	0	0	0	0	0	0	0	0	0	0	0	0	0
0	0	0	0	0	0	0	0	0	0	0	0	0	0	0	0	0	0	0
0	0	0	0	0	0	0	0	0	0	0	0	0	0	0	0	0	0	0
0	0	0	0	0	0	0	0	0	0	0	0	0	0	0	0	0	0	0

0	0	0	0	0	0	0	0	0	0	0	0	0	0	0	0	0	0	0
0	0	0	0	0	0	11	11	0	0	0	0	0	0	0	0	0	0	0
0	0	0	0	0	0	0	0	0	0	0	0	0	0	0	0	0	0	0
0	0	0	0	0	0	0	0	0	0	0	10	10	0	0	0	0	0	0
0	0	0	0	0	0	0	0	0	0	0	0	0	0	0	0	0	0	0
0	0	0	0	0	0	0	0	0	0	0	0	0	0	0	0	6	0	0
0	0	0	0	0	0	0	0	0	0	0	0	0	0	0	0	0	0	0
0	0	0	0	0	0	0	0	0	0	0	0	0	0	0	0	0	0	0
0	0	0	0	0	0	0	0	0	0	0	0	0	0	0	0	0	0	0
0	0	0	0	0	0	0	0	0	0	0	0	0	0	0	0	0	0	0
0	0	0	0	0	0	0	0	0	0	0	0	0	8	0	0	0	0	0
0	0	0	0	0	0	0	0	0	0	0	0	0	0	0	0	0	0	0
0	0	0	0	0	0	0	0	0	0	0	0	0	0	0	0	0	0	0
0	0	0	0	0	0	0	0	0	0	0	0	0	0	0	0	0	0	0
0	0	0	0	0	0	0	0	0	0	0	0	0	0	0	0	0	0	0
0	0	0	0	0	0	0	0	0	0	0	0	0	0	0	7	0	0	0
0	0	0	0	0	0	0	0	0	0	0	0	0	0	0	0	0	0	0
0	0	0	0	0	0	0	0	0	0	0	0	0	0	0	0	0	0	0
0	0	0	0	0	0	0	0	0	0	0	0	0	0	0	0	0	0	0
0	0	0	0	0	0	0	0	0	0	0	0	0	0	0	0	0	0	0
0	0	0	0	0	0	0	0	0	0	0	0	0	0	0	0	0	0	0
0	0	0	0	0	0	0	0	0	0	0	6	0	0	0	0	0	0	0
0	0	0	0	0	0	0	0	0	0	0	0	0	0	0	0	0	0	0
0	0	0	0	0	0	0	0	0	0	0	0	0	0	0	0	0	0	0
0	0	0	0	0	0	0	0	0	0	0	0	0	0	0	0	0	0	0
0	0	0	0	0	0	0	0	0	0	0	0	0	0	0	0	0	0	0
0	0	0	0	0	0	0	0	0	0	0	0	0	0	0	0	0	0	0
0	0	0	0	0	0	0	0	0	0	0	0	0	0	0	0	0	0	0
0	0	0	0	0	0	0	0	0	0	0	0	0	0	0	0	0	0	0
0	0	0	0	0	0	0	0	0	0	0	0	0	0	0	0	0	0	0
0	0	0	0	0	0	0	0	0	0	0	0	0	0	0	0	0	0	0
0	0	0	0	0	0	0	0	0	0	0	0	0	0	0	0	0	0	0
0	0	0	0	0	0	0	0	0	0	0	0	0	0	0	0	0	0	0
0	0	0	0	0	0	0	0	0	0	0	0	0	0	0	0	0	0	0
0	0	0	0	0	0	0	0	0	0	0	0	0	0	0	0	0	0	0
0	0	0	0	0	0	0	0	0	0	0	0	0	0	0	0	0	0	0
0	0	0	0	0	0	0	0	0	0	0	0	0	0	0	0	0	0	0

0	0	0	0	0	0	0	0	0	0	0	0	0	0	0	0	0	0	0
0	0	0	0	0	0	0	0	0	0	0	0	0	0	0	0	0	0	0
0	0	0	0	0	0	0	0	0	0	0	0	0	0	0	0	0	0	0
0	0	0	0	0	0	0	0	0	0	0	0	0	0	0	0	0	0	0
0	0	0	0	0	0	0	0	0	0	0	0	0	0	0	0	0	0	0
0	0	0	0	0	0	0	0	0	0	0	0	0	0	0	0	0	0	0
0	0	0	0	0	0	0	0	0	0	0	0	0	0	0	0	0	0	0
0	0	0	0	0	0	0	0	0	0	0	0	0	0	0	0	0	0	0
0	0	0	0	0	0	0	0	0	0	0	0	0	0	0	0	0	0	0
0	0	0	0	0	0	0	0	0	0	0	0	0	0	0	0	0	0	0
0	0	0	0	0	0	0	0	0	0	0	0	0	0	0	0	0	0	0
0	0	0	0	0	0	0	0	0	0	0	0	0	0	0	0	0	0	0
0	0	0	0	0	0	0	0	0	0	0	0	0	0	0	0	0	0	0
0	0	0	0	0	0	0	0	0	0	0	0	0	0	0	0	0	0	0
0	0	0	0	0	0	0	0	0	0	0	0	0	0	0	0	0	0	0
0	0	0	0	0	0	0	0	0	0	0	0	0	0	0	0	0	0	0
0	0	0	0	0	0	0	0	0	0	0	0	0	0	0	0	0	0	0
0	0	0	0	0	0	0	0	0	0	0	0	0	0	0	0	0	0	0
0	0	0	0	0	0	0	0	0	0	0	0	0	0	0	0	0	0	0
0	0	0	0	0	0	0	0	0	0	0	0	0	0	3	3	3	3	3
0	0	0	0	0	0	0	0	0	0	0	0	0	0	0	0	0	0	0
0	0	0	0	0	0	0	0	0	0	0	0	0	0	0	0	0	0	0
0	0	0	0	0	0	0	0	0	0	0	0	0	0	0	0	0	0	0
0	0	0	0	0	0	0	0	0	0	0	0	0	0	0	0	0	0	0
0	0	0	0	0	0	0	0	0	0	0	0	0	0	0	0	0	0	0
0	0	0	0	0	0	0	0	0	0	0	0	0	0	0	0	0	0	0
0	0	0	0	0	0	0	0	0	0	0	0	0	0	0	0	0	0	0
0	0	0	0	0	0	0	0	0	0	0	0	0	0	0	0	0	0	0
0	0	0	0	0	0	0	0	0	0	0	0	0	0	0	0	0	0	0
0	0	0	0	0	0	0	0	0	0	0	0	0	0	0	0	0	0	0
0	0	0	0	0	0	0	0	0	0	0	0	0	0	0	0	0	0	0
0	0	0	0	0	0	0	0	0	0	0	0	0	0	0	0	0	0	0
0	0	0	0	0	0	0	0	0	0	0	0	0	0	0	0	0	0	0
0	0	0	0	0	0	0	0	0	0	0	0	0	0	0	0	0	0	0
0	0	0	0	0	0	0	0	0	0	0	0	0	0	0	0	0	0	0
0	0	0	0	0	0	0	0	0	0	0	0	0	0	0	0	0	0	0
0	0	0	0	0	0	0	0	0	0	0	0	0	0	0	0	0	0	0

0	0	0	0	0	0	0	0	0	0	0	0	0	0	0	0	0	0	0
0	0	0	0	0	0	0	0	0	0	0	0	0	0	0	0	0	0	0
0	0	0	0	0	0	0	0	0	0	0	0	0	0	0	0	0	0	0
0	0	0	0	0	0	0	0	0	0	0	0	0	0	0	0	0	0	0
0	0	0	0	0	0	0	0	0	0	0	0	0	0	0	0	0	0	0
0	0	0	0	0	0	0	0	0	0	0	0	0	0	0	0	0	0	0
0	0	0	0	0	0	0	0	0	0	0	0	0	0	0	0	0	0	0
0	0	0	0	0	0	0	0	0	0	0	0	0	0	0	0	0	0	0
0	0	0	0	0	0	0	0	0	0	0	0	0	0	0	0	0	0	0
0	0	0	0	0	0	0	0	0	0	0	0	0	0	0	0	0	0	0
0	0	0	0	0	0	0	0	0	0	0	0	0	0	0	0	0	0	0
2	2	2	2	2	2	2	0	0	0	0	0	0	0	0	0	0	0	0
0	0	0	0	0	0	0	0	0	0	0	0	0	0	0	0	0	0	0
0	0	0	0	0	0	0	0	0	0	0	0	0	0	0	0	0	0	0
0	0	0	0	0	0	0	0	0	0	0	0	0	0	0	0	0	0	0
0	0	0	0	0	0	0	0	0	0	0	0	0	0	0	0	0	0	0
0	0	0	0	0	0	0	0	0	0	0	0	0	0	0	0	0	0	0
0	0	0	0	0	0	0	0	0	0	0	0	0	0	0	0	0	0	0
0	0	0	0	0	0	0	0	0	0	0	0	0	0	0	0	0	0	0
0	0	0	0	0	0	0	0	0	0	0	0	0	0	0	0	0	0	0
0	0	0	0	0	0	0	0	0	0	0	0	0	0	0	0	0	0	0
0	0	0	0	0	0	0	0	0	0	0	0	0	0	0	0	0	0	0
0	0	0	0	0	0	0	0	0	0	0	0	0	0	0	0	0	0	0
0	0	0	0	0	0	0	0	0	0	0	0	0	0	0	0	0	0	0
0	0	0	0	0	0	0	0	0	0	0	0	0	0	0	0	0	0	0
0	0	0	0	0	0	0	0	0	0	0	0	0	0	0	0	0	0	0
0	0	0	0	0	0	0	0	0	0	0	0	0	0	0	0	0	0	0
0	0	0	0	0	0	0	0	0	0	0	0	0	0	0	0	0	0	0
0	0	0	0	0	0	0	0	0	0	0	0	0	0	0	0	0	0	0
0	0	0	0	0	0	0	0	0	0	0	0	0	0	0	0	0	0	0
0	0	0	0	0	0	0	0	0	0									
Jan_DL104	2065	1690	109	474	55	406	411	328	225	205	154	156	134	290	175	200	256	150
152	703	245	0	327	215	68	110	81	64	157	0	139	69	86	72	87	13	0
50	204	96	305	46	64	83	0	81	61	152	0	295	56	0	0	132	123	0
0	6	22	35	145	21	100	42	43	44	96	112	56	138	44	60	17	164	0
0	0	0	19	15	16	26	0	51	0	0	78	26	0	70	85	61	22	0
0	0	0	108	0	7	13	0	59	29	0	0	0	20	0	48	11	0	24

0	10	0	6	7	26	0	0	0	110	0	0	0	0	0	0	0	0	59
85	57	91	0	8	0	0	0	0	10	40	0	13	0	0	75	0	0	0
0	30	0	0	78	0	18	0	13	0	6	0	17	5	0	0	10	0	18
0	0	15	24	0	7	0	0	0	17	0	0	0	33	0	0	27	0	24
0	0	0	0	0	0	0	0	0	0	0	7	28	0	14	0	0	0	0
0	0	0	0	0	0	0	0	0	31	55	0	0	0	0	0	0	0	0
0	0	0	0	0	0	0	0	0	0	0	0	27	15	12	0	0	13	0
0	0	0	9	0	0	0	0	0	0	0	0	0	0	11	0	0	0	0
18	0	0	0	0	0	36	0	0	0	0	10	0	0	0	0	0	0	0
0	0	0	0	0	0	0	10	0	0	0	28	9	0	10	0	0	0	0
0	0	0	0	0	56	0	0	0	0	22	0	0	0	4	0	0	0	0
0	0	10	0	0	0	0	0	5	0	0	0	0	0	0	0	0	0	0
5	0	42	0	0	18	0	0	13	0	0	13	13	0	0	8	14	0	0
0	0	0	0	0	0	0	11	0	0	30	0	0	0	0	0	0	0	0
0	0	0	0	0	0	0	0	5	0	0	0	0	0	0	0	0	14	0
0	0	0	0	0	4	0	0	0	0	0	6	3	4	0	15	8	0	0
0	0	0	0	0	0	8	0	0	0	0	0	53	0	0	0	0	0	0
0	0	0	10	0	0	17	0	0	0	0	0	0	0	0	0	0	0	0
0	0	5	0	0	0	0	0	0	0	0	0	12	0	0	0	0	0	0
0	0	0	0	0	0	0	9	0	0	0	0	0	0	0	0	0	0	6
4	0	0	0	0	0	0	0	0	0	0	0	0	0	0	0	5	0	0
0	0	0	0	0	0	0	0	0	0	0	0	0	0	0	3	0	0	0
0	0	0	0	0	0	0	7	0	0	0	0	0	0	0	0	0	0	0
0	4	0	0	0	0	0	0	0	0	0	0	0	0	0	0	0	0	0
0	0	0	0	0	0	0	0	0	0	0	0	0	0	0	0	0	0	0
0	0	0	0	0	0	0	0	0	0	0	0	0	0	0	0	0	0	0
0	0	0	0	0	0	0	0	0	0	0	0	0	0	0	0	0	0	0
0	0	0	0	0	0	10	0	0	0	0	0	0	0	6	9	0	0	0
0	0	0	0	0	0	0	0	0	0	0	0	0	0	0	0	0	0	0
0	0	0	0	0	0	0	0	0	0	0	0	0	0	0	0	0	0	0
0	0	0	0	0	0	0	0	0	0	0	0	0	0	0	0	19	0	0
0	0	0	0	0	0	0	0	0	0	0	0	0	0	0	0	0	0	0
0	0	0	0	0	0	0	0	0	0	0	9	0	0	0	0	0	0	0
0	0	0	4	0	0	0	0	0	0	0	0	0	0	0	0	0	0	0
0	0	0	0	0	0	0	0	0	0	10	0	0	0	0	0	0	0	0
0	0	0	0	0	0	0	0	0	0	0	6	0	0	0	0	0	0	0
0	0	0	0	0	0	0	0	0	0	0	0	0	0	0	0	0	0	0

0	0	0	0	0	0	0	0	0	0	4	0	21	0	0	0	0	0	0
0	0	0	0	0	0	0	0	0	0	0	0	0	0	0	0	0	0	0
0	0	0	0	0	0	0	0	0	0	0	0	0	0	0	0	0	10	0
0	0	0	0	0	0	0	0	0	0	0	0	0	0	0	0	0	0	0
0	0	0	0	0	0	0	0	0	0	0	0	0	0	0	0	0	0	0
0	0	0	0	0	0	0	0	0	0	0	0	0	0	0	0	0	0	0
0	0	0	0	0	0	0	0	0	0	0	0	0	0	0	0	0	0	0
0	0	0	0	0	0	5	0	0	0	0	19	0	0	0	0	0	0	0
0	0	0	0	0	0	0	0	0	0	0	0	0	0	0	0	0	0	0
0	0	0	0	0	0	0	0	0	0	0	0	0	0	0	0	0	0	0
0	0	0	6	0	0	0	0	0	0	0	0	0	0	0	0	23	0	0
0	0	0	0	0	0	0	0	0	0	0	0	0	0	0	0	0	0	0
0	0	0	0	0	0	0	0	0	0	0	0	0	0	0	0	0	0	0
0	0	0	0	0	0	0	0	0	0	0	25	0	0	0	0	0	0	0
0	0	0	0	0	0	0	0	0	0	0	0	0	0	0	0	0	0	0
0	0	0	2	0	0	0	0	0	0	0	0	0	0	0	0	0	0	0
0	0	0	0	0	0	0	0	0	0	0	0	0	2	0	0	0	0	0
0	0	0	0	0	0	0	0	0	0	0	0	0	0	0	0	0	0	0
0	0	0	0	0	0	0	0	0	0	0	0	0	0	0	0	18	18	0
0	0	0	0	0	0	0	0	0	0	0	0	0	0	0	0	0	0	0
0	0	0	0	0	0	0	0	0	0	0	0	0	0	0	0	0	0	0
0	0	0	0	0	0	0	0	0	0	0	0	0	0	0	0	0	0	0
0	0	0	0	0	0	0	0	0	0	0	0	0	0	0	0	0	0	0
0	0	0	0	0	0	0	0	0	0	0	0	0	0	0	0	0	0	0
0	0	0	0	0	0	0	0	0	0	0	0	0	0	0	0	0	0	0
0	0	0	0	0	0	0	0	0	0	0	0	0	0	0	0	0	0	0
0	0	0	0	0	0	13	0	0	0	0	0	0	0	0	0	0	0	0
0	0	0	0	0	0	0	0	0	0	0	0	0	0	0	0	0	0	0
0	0	0	0	0	0	0	12	0	0	0	0	0	0	0	0	0	0	0
0	0	0	0	0	0	0	0	0	0	0	0	0	0	0	0	0	0	0
0	0	0	0	0	0	0	0	0	0	0	11	0	0	0	0	0	0	0
0	0	0	0	0	0	0	0	0	0	0	0	0	0	0	0	0	0	0
0	0	0	0	0	0	0	0	0	0	0	0	0	0	0	0	0	0	0
0	10	10	0	0	0	0	0	0	0	0	0	0	0	0	0	0	0	0
0	0	0	0	0	0	0	0	0	0	0	0	0	0	0	0	0	0	0
0	0	0	0	0	0	0	0	0	0	0	0	0	0	0	0	0	0	0
0	0	0	0	0	0	0	0	0	0	9	0	0	0	0	0	0	0	0

0	0	0	0	0	0	0	0	0	0	0	0	0	0	0	0	0	0	0
0	0	0	0	0	0	0	0	0	0	0	0	0	0	0	0	0	0	0
0	0	0	0	0	0	0	0	0	0	0	0	0	0	0	0	0	0	0
0	0	0	0	0	0	0	0	0	0	0	0	0	0	0	0	0	0	0
0	0	0	0	0	0	0	0	0	0	0	0	0	0	0	0	0	0	0
0	0	0	0	0	0	0	0	0	0	0	0	0	0	0	0	0	0	0
0	0	0	0	0	0	0	0	0	0	0	0	0	0	0	0	0	0	0
0	0	0	0	0	0	0	0	0	0	0	0	0	0	0	0	0	0	0
0	0	0	7	7	0	0	0	0	0	0	0	0	0	0	0	0	0	0
0	0	0	0	0	0	0	0	0	0	0	0	0	0	0	0	0	0	0
0	0	0	0	0	0	0	0	0	0	0	0	0	0	0	0	0	0	0
0	0	0	0	0	0	0	0	0	0	0	0	0	0	0	0	0	0	0
0	0	0	0	0	0	0	0	0	0	0	0	0	0	0	0	0	0	0
0	0	0	0	0	0	0	0	0	0	0	0	0	0	0	0	0	0	0
0	0	0	0	0	0	0	0	6	6	0	0	0	0	0	0	0	0	0
0	0	0	0	0	0	0	0	0	0	0	0	0	0	0	0	0	0	0
0	0	0	0	0	0	0	0	0	0	0	0	0	0	0	0	0	0	0
0	0	0	0	0	0	0	0	0	0	0	0	0	0	0	0	0	0	0
0	0	0	0	0	0	0	0	0	0	0	0	0	0	0	0	0	0	0
0	0	0	0	0	0	0	0	0	0	0	0	0	0	0	0	0	0	0
0	0	0	0	0	0	0	0	0	0	0	0	0	0	0	0	0	0	0
0	0	0	0	0	0	0	0	0	0	0	0	0	0	0	0	0	0	0
0	0	0	0	0	0	0	0	0	0	0	0	0	0	0	0	0	0	0
0	0	0	0	0	0	0	0	0	0	0	0	0	0	0	0	0	0	0
0	0	0	0	0	0	0	0	0	0	0	0	0	0	0	0	0	0	0
0	0	0	0	0	0	0	0	0	0	0	0	0	0	0	0	0	0	0
0	0	0	0	0	0	0	0	0	0	0	0	0	0	0	0	0	0	0
0	0	0	0	0	0	0	0	0	0	0	0	0	0	0	0	0	0	0
0	0	0	0	0	0	0	0	0	0	0	0	0	0	0	0	0	0	0
0	0	0	0	0	0	0	0	0	0	0	0	0	0	0	0	0	0	0
0	0	0	0	0	0	0	0	0	0	0	0	0	0	0	0	0	0	0
0	0	0	0	0	0	0	0	0	0	0	0	0	0	0	0	0	0	0
0	0	0	0	0	0	0	0	0	0	0	0	0	0	0	0	0	4	0
0	0	0	0	0	0	0	0	0	0	0	0	0	0	0	0	0	0	0
0	0	0	0	0	0	0	0	0	0	0	0	0	0	0	0	0	0	0
0	0	0	0	0	0	0	0	0	0	0	0	0	0	0	0	0	0	0
0	0	0	0	0	0	0	0	0	0	0	0	0	0	0	0	0	0	0

0	0	0	0	0	0	0	0	0	0	0	0	0	0	0	0	0	0	0
0	0	0	0	0	0	0	0	0	0	0	0	0	0	0	0	0	0	0
0	0	0	0	0	0	0	0	0	0	0	0	0	0	0	0	0	0	0
0	0	0	0	0	0	0	0	0	0	0	0	0	0	0	0	0	0	0
0	0	0	0	0	0	0	0	0	0	0	0	0	0	0	0	0	0	0
0	0	0	0	0	0	0	0	0	0	0	0	0	0	0	0	0	0	0
0	0	0	0	0	0	0	0	0	0	0	0	0	0	0	0	0	0	0
0	0	0	0	0	0	0	0	0	0	0	0	0	0	0	0	0	0	0
0	0	0	0	0	0	0	0	0	0	0	0	0	0	0	0	0	0	0
0	0	0	0	0	0	0	0	0	0	0	0	0	0	0	0	0	0	0
0	0	0	0	0	0	0	0	0	0	0	0	0	0	0	0	0	0	0
0	0	0	0	0	0	0	0	0	0	0	0	0	0	0	0	0	0	0
0	0	0	0	0	0	0	0	0	0	0	0	0	0	3	0	0	0	0
0	0	0	0	0	0	0	0	0	0	0	0	0	0	0	0	0	0	0
0	0	0	0	0	0	0	0	0	0	0	0	0	0	0	0	0	0	0
0	0	0	0	0	0	0	0	0	0	0	0	0	0	0	0	0	0	0
0	0	0	0	0	0	0	0	0	0	0	0	0	0	0	0	0	0	0
0	0	0	0	0	0	0	0	0	0	0	0	0	0	0	0	0	0	0
0	0	0	0	0	0	0	0	0	0	0	0	0	0	0	0	0	0	0
0	0	0	0	0	0	0	0	0	0	0	0	0	0	0	0	0	0	0
0	0	0	0	0	0	0	0	0	0	0	0	0	0	0	0	0	0	0
0	0	0	0	0	0	0	0	0	0	0	0	0	0	0	0	0	0	0
0	0	0	0	0	0	0	0	0	0	0	0	0	0	0	0	0	0	0
0	0	0	0	0	0	0	0	0	0	0	0	0	0	0	0	0	0	0
0	0	0	0	0	0	0	0	0	0	0	0	0	0	0	0	0	0	0
0	0	0	0	0	0	0	0	0	0	0	0	0	0	0	0	0	0	0
0	0	0	0	0	0	0	0	0	0	0	0	0	0	0	0	0	0	0
0	0	0	0	0	0	0	0	0	0	0	0	0	0	0	0	0	0	0
0	0	0	0	0	0	0	0	0	0	0	0	0	0	0	0	0	0	0
0	0	0	0	0	0	0	0	0	0	0	0	0	0	0	0	0	0	0
0	0	0	0	0	0	0	0	0	0	0	0	0	0	0	0	0	0	0
0	0	0	0	0	0	0	0	0	0	0	0	0	0	0	0	0	0	0
0	0	0	0	0	0	0	0	0	0	0	0	0	0	0	0	0	0	0
0	0	0	0	0	0	0	0	0	0	0	0	0	0	0	0	0	0	0
0	0	0	0	0	0	0	0	0	0	0	0	0	0	0	0	0	0	0
0	0	0	0	0	0	0	0	0	0	0	0	0	0	0	0	0	0	0
0	0	0	0	0	0	0	0	0	0	0	0	0	0	0	0	0	0	0

0	0	0	0	0	0	0	0	0	0	0	0	0	0	0	0	0	0	0
0	0	0	0	0	0	0	0	0	0	0	0	0	0	0	0	0	0	0
0	0	0	0	0	0	0	0	0	0	0	0	0	0	0	0	0	0	0
0	0	0	0	0	0	0	0	2	2	0	0	0	0	0	0	0	0	0
0	0	0	0	0	0	0	0	0	0	0	0	0	0	0	0	0	0	0
0	0	0	0	0	0	0	0	0	0	0	0	0	0	0	0	0	0	0
0	0	0	0	0	0	0	0	0	0	0	0	0	0	0	0	0	0	0
0	0	0	0	0	0	0	0	0	0	0	0	0	0	0	0	0	0	0
0	0	0	0	0	0	0	0	0	0	0	0	0	0	0	0	0	0	0
0	0	0	0	0	0	0	0	0	0	0	0	0	0	0	0	0	0	0
0	0	0	0	0	0	0	0	0	0	0	0	0	0	0	0	0	0	0
0	0	0	0	0	0	0	0	0	0	0	0	0	0	0	0	0	0	0
0	0	0	0	0	0	0	0	0	0	0	0	0	0	0	0	0	0	0
0	0	0	0	0	0	0	0	0	0	0	0	0	0	0	0	0	0	0
0	0	0	0	0	0	0	0	0	0	0	0	0	0	0	0	0	0	0
0	0	0	0	0	0	0	0	0	0	0	0	0	0	0	0	0	0	0
0	0	0	0	0	0	0	0	0	0	0	0	0	0	0	0	0	0	0
0	0	0	0	0	0	0	0	0	0	0	0	0	0	0	0	0	0	0
0	0	0	0	0	0	0	0	0	0	0	0	0	0	0	0	0	0	0
0	0	0	0	0	0	0	0	0	0	0	0	0	0	0	0	0	0	0
0	0	0	0	0	0	0	0	0	0	0	0	0	0	0	0	0	0	0
0	0	0	0	0	0	0	0	0	0	0	0	0	0	0	0	0	0	0
0	0	0	0	0	0	0	0	0	0									
Jan_DL106	417	365	511	326	58	138	113	141	535	322	488	133	236	138	145	209	96	134
59	0	49	0	142	45	111	78	120	179	116	239	110	53	146	223	71	149	187
128	252	54	188	73	193	26	63	54	87	57	234	281	0	153	254	103	43	138
138	40	204	31	42	0	37	54	17	35	24	0	0	0	0	0	13	21	27
62	114	93	35	22	53	25	65	0	30	56	44	28	0	28	41	0	25	99
49	0	50	0	0	0	6	0	0	0	52	0	0	0	13	0	14	0	0
0	7	0	9	8	11	0	0	0	40	0	0	21	41	0	0	0	8	0
0	0	0	0	28	0	21	0	0	0	0	0	0	0	0	0	0	0	0
0	11	37	10	56	0	14	0	0	0	0	0	0	0	0	0	26	0	0
9	17	7	0	0	0	0	17	0	0	0	0	0	0	0	0	8	0	9
0	0	0	0	0	0	19	0	0	0	0	0	22	0	0	0	0	0	0
0	0	33	0	12	0	0	0	0	0	0	0	0	0	0	0	0	0	0
26	0	0	0	0	9	0	0	0	0	0	0	0	4	0	0	0	0	0
11	0	0	0	0	0	0	0	0	8	0	0	0	0	2	0	0	32	0

0	0	0	0	0	7	0	0	0	0	0	0	0	0	0	0	19	0	60
0	0	0	0	0	0	0	0	2	0	0	0	0	0	9	0	33	6	0
0	0	0	0	0	0	0	0	0	0	0	0	0	0	0	0	0	0	0
0	0	16	0	0	0	0	6	10	0	0	0	0	0	0	6	0	0	0
0	0	0	0	0	0	0	0	10	0	0	0	0	0	0	0	0	0	0
0	3	0	0	8	0	0	0	0	0	0	0	0	0	0	0	0	0	5
0	0	0	0	0	0	0	0	0	0	0	0	0	0	8	0	0	0	0
0	0	0	0	0	0	0	0	0	0	0	0	0	8	0	0	0	0	0
0	0	0	0	0	0	10	0	11	0	0	0	0	0	0	0	0	0	0
0	0	0	0	0	29	0	0	0	0	0	0	0	0	0	0	0	0	0
0	0	0	0	0	0	0	0	0	0	0	0	0	0	0	0	0	0	0
0	0	0	0	0	0	0	0	32	0	0	0	0	0	0	0	0	17	4
0	0	0	0	0	0	0	0	0	0	0	0	0	0	0	0	0	0	0
0	0	0	0	0	0	0	0	0	0	0	0	0	0	0	0	0	0	0
0	0	0	0	0	0	0	0	0	0	0	0	0	0	7	0	0	0	0
0	0	0	0	0	0	0	8	0	0	4	0	0	0	0	0	0	0	0
0	0	0	0	0	0	0	0	0	0	0	0	0	0	0	0	0	0	0
0	0	0	0	0	0	0	0	0	0	0	0	0	0	0	0	25	0	0
0	0	5	0	0	8	0	0	0	0	0	0	0	0	0	0	0	0	0
0	0	0	0	0	0	0	0	0	0	0	0	0	0	0	0	0	0	0
0	0	0	0	0	0	0	0	0	0	0	0	0	0	0	0	0	0	0
0	0	0	0	0	0	0	0	0	0	0	42	0	0	0	0	0	18	0
0	0	0	0	0	0	0	0	0	0	7	0	0	0	0	0	0	0	0
0	0	0	0	0	0	30	0	0	0	0	0	0	0	0	0	0	0	0
0	0	0	0	0	0	0	0	0	28	0	0	0	0	0	0	0	0	0
0	0	0	0	0	0	0	0	0	0	0	0	0	0	0	0	5	0	0
0	0	0	0	0	0	0	0	0	0	0	0	0	0	0	0	0	0	0
0	0	0	0	0	0	0	0	0	0	3	0	0	5	0	0	0	0	0
0	0	48	0	0	0	0	0	0	0	0	0	0	0	0	0	0	0	0
0	0	0	0	0	0	0	22	0	0	0	0	0	0	0	0	0	0	0
0	0	0	0	0	0	0	0	0	0	0	0	0	0	0	0	0	0	0
0	0	0	0	0	0	0	0	0	0	0	0	0	0	0	0	0	18	0
0	0	0	0	0	0	0	0	0	0	0	0	0	0	0	0	0	0	0
0	0	0	0	0	0	0	0	0	0	12	0	0	0	0	0	0	0	0
0	0	0	0	0	0	0	0	0	0	0	0	0	0	13	0	0	0	0
0	0	0	0	0	0	0	0	0	0	0	0	0	8	0	0	0	0	0
0	0	0	0	0	0	0	0	0	0	0	0	0	0	0	0	0	0	0

0	0	0	0	0	0	0	0	0	0	0	0	0	0	0	0	0	0	0
0	0	0	21	0	0	0	0	0	0	0	0	0	0	0	0	0	0	0
0	0	0	0	0	0	0	0	0	0	0	0	0	4	0	0	0	0	0
0	0	0	0	0	0	0	0	0	0	0	0	0	0	0	0	0	0	11
0	0	0	0	0	0	0	0	0	0	0	20	0	0	0	0	0	0	0
0	0	0	0	0	0	0	0	0	0	0	0	0	0	0	0	0	0	0
0	0	0	0	7	0	0	0	0	0	0	0	0	0	0	0	0	0	0
0	0	0	0	0	0	0	0	0	0	0	0	0	0	0	0	0	0	0
0	16	0	0	0	0	0	0	0	0	0	0	0	0	0	0	0	0	0
0	0	0	0	0	0	0	0	0	0	0	0	0	0	0	0	0	0	0
0	0	0	0	0	0	0	0	0	0	14	0	0	0	0	0	0	0	0
0	0	0	0	0	0	0	0	0	0	0	0	0	0	0	0	0	13	0
0	0	0	0	0	0	0	0	0	0	0	0	0	0	0	0	0	0	0
0	0	0	0	0	0	0	0	4	0	0	0	0	0	0	0	0	0	0
0	0	0	0	0	0	0	0	0	0	0	0	0	0	0	0	0	0	0
0	0	0	0	0	0	0	0	0	0	0	0	0	7	0	0	0	0	5
0	0	0	0	0	0	0	0	0	0	0	0	0	0	0	0	0	0	0
0	0	0	0	0	0	0	0	0	0	0	0	0	0	0	0	0	0	0
0	0	0	0	0	0	0	0	0	0	0	0	0	0	0	0	0	0	0
0	0	0	0	0	0	0	0	0	0	0	0	0	0	0	0	0	0	0
0	0	0	0	0	0	0	0	0	0	0	0	0	0	0	0	0	0	0
0	0	0	0	0	0	0	0	9	0	0	0	0	0	0	0	0	0	0
0	0	0	0	0	0	0	0	0	0	0	0	0	0	0	0	0	0	0
0	0	0	0	0	0	0	8	8	0	0	0	0	0	0	0	0	0	0
0	0	0	0	0	0	0	0	0	0	0	0	0	0	0	0	0	0	0
0	0	0	0	0	0	0	0	0	0	0	0	0	0	0	0	0	0	0
0	0	0	0	0	0	0	0	0	0	0	0	0	0	0	0	0	0	0
0	0	0	0	0	0	0	0	0	0	0	7	0	0	0	0	0	0	0
0	0	0	0	0	0	0	0	0	0	0	0	0	0	0	0	0	0	0
0	0	0	0	0	0	0	0	0	0	0	0	0	0	0	0	0	0	0
0	0	0	0	0	0	0	0	0	0	0	0	0	0	0	0	0	0	0
0	0	0	0	0	0	0	0	0	0	0	0	0	0	0	0	0	0	0
0	0	0	0	0	0	0	0	0	0	0	0	0	0	0	0	0	6	6
0	0	0	0	0	0	0	0	0	0	0	0	0	0	0	0	0	0	0
0	0	0	0	0	0	0	0	0	0	0	0	0	0	0	0	0	0	0
0	0	0	0	0	0	0	0	0	0	0	0	0	0	0	0	0	0	3
0	0	0	0	0	0	0	0	0	0	0	0	0	0	0	0	0	0	0

0	0	0	0	0	0	0	0	0	0	0	0	0	0	0	0	0	0	0
0	0	0	0	0	0	0	0	0	0	0	0	0	0	0	0	0	0	0
0	0	0	0	0	0	0	0	0	0	0	0	0	0	0	0	0	0	0
0	0	0	0	0	0	5	5	0	0	0	0	0	0	0	0	0	0	0
0	0	0	0	0	0	0	0	0	0	0	0	0	0	0	0	0	0	0
0	0	0	0	0	0	0	0	0	0	0	0	0	0	0	0	0	0	0
0	0	0	0	0	0	0	0	0	0	0	0	0	0	0	0	0	0	0
0	0	0	0	0	0	0	0	0	0	0	0	0	0	0	0	0	0	0
0	0	0	0	0	0	0	0	0	0	0	0	0	0	0	0	0	0	0
0	0	0	0	0	0	0	0	0	0	0	0	0	0	0	0	0	0	0
0	0	0	0	0	0	0	0	0	0	0	0	0	0	0	0	0	0	0
0	0	0	0	0	0	0	0	0	0	0	0	0	0	0	0	0	0	0
0	0	0	0	0	0	0	0	0	0	0	0	0	0	0	0	0	0	0
0	0	0	0	0	0	0	0	0	0	0	0	0	0	0	0	0	0	0
0	0	0	0	0	0	0	0	0	0	0	0	0	0	0	0	0	0	0
0	0	0	0	0	0	0	0	0	0	0	0	0	0	0	0	0	0	0
0	0	0	0	0	0	0	0	0	0	0	0	0	0	0	0	0	0	0
0	0	0	0	0	0	0	0	0	0	0	0	0	0	0	0	0	0	0
0	0	0	0	0	0	0	0	0	0	0	0	0	0	0	0	0	0	0
0	0	0	0	0	0	0	0	0	0	0	0	0	0	0	0	0	0	0
0	0	0	0	0	0	0	0	0	0	0	0	0	0	0	0	0	0	0
0	0	0	0	0	0	0	0	0	0	0	0	0	0	0	0	0	0	0
0	0	0	0	0	0	0	0	0	0	0	0	0	0	0	0	0	0	0
0	0	0	0	0	0	0	0	0	0	0	0	0	0	0	0	0	0	0
0	0	0	0	0	0	0	0	0	0	0	0	0	0	0	0	0	0	0
0	0	0	0	0	0	0	0	0	0	0	0	0	0	0	0	0	0	0
0	0	0	0	0	0	0	0	0	0	0	3	0	0	0	0	0	0	0
0	0	0	0	0	0	0	0	0	0	0	0	0	0	0	0	0	0	0
0	0	0	0	0	0	0	0	0	0	0	0	0	0	0	0	0	0	0
0	0	0	0	0	0	0	0	0	0	0	0	0	0	0	0	0	0	0
0	0	0	0	0	0	0	0	0	0	0	0	0	0	0	0	0	0	0
0	0	0	0	0	0	0	0	0	0	0	0	0	0	0	0	0	0	0
0	0	0	0	0	0	0	0	0	0	0	0	0	0	0	0	0	0	0
0	0	0	0	0	0	0	0	0	0	0	0	0	0	0	0	0	0	0
0	0	0	0	0	0	0	0	0	0	0	0	0	0	0	0	0	0	0
0	0	0	0	0	0	0	0	0	0	0	0	0	0	0	0	0	0	0
0	0	0	0	0	0	0	0	0	0	0	0	0	0	0	0	0	0	0

0	0	0	0	0	0	0	0	0	0	0	0	0	0	0	0	0	0	0
0	0	0	0	0	0	0	0	0	0	0	0	0	0	0	0	0	0	0
0	0	0	0	0	0	0	0	0	0	0	0	0	0	0	0	0	0	0
0	0	0	0	0	0	0	0	0	0	0	0	0	0	0	0	0	0	0
0	0	0	0	0	0	0	0	0	0	0	0	0	0	0	0	0	0	0
0	0	0	0	0	0	0	0	0	0	0	0	0	0	0	0	0	0	0
0	0	0	0	0	0	0	0	0	0	0	0	0	0	0	0	0	0	0
0	0	0	0	0	0	0	0	0	0	0	0	0	0	0	0	0	0	0
0	0	0	0	0	0	0	0	0	0	0	0	0	0	0	0	0	0	0
0	0	0	0	0	0	0	0	0	0	0	0	0	0	0	0	0	0	0
0	0	0	0	0	0	0	0	0	0	0	0	0	0	0	0	0	0	0
0	0	0	0	0	0	0	0	0	0	0	0	0	0	0	0	0	0	0
0	0	0	0	0	0	0	0	0	0	0	0	0	0	0	0	2	2	2
2	2	0	0	0	0	0	0	0	0	0	0	0	0	0	0	0	0	0
0	0	0	0	0	0	0	0	0	0	0	0	0	0	0	0	0	0	0
0	0	0	0	0	0	0	0	0	0	0	0	0	0	0	0	0	0	0
0	0	0	0	0	0	0	0	0	0	0	0	0	0	0	0	0	0	0
0	0	0	0	0	0	0	0	0	0	0	0	0	0	0	0	0	0	0
0	0	0	0	0	0	0	0	0	0	0	0	0	0	0	0	0	0	0
0	0	0	0	0	0	0	0	0	0	0	0	0	0	0	0	0	0	0
0	0	0	0	0	0	0	0	0	0	0	0	0	0	0	0	0	0	0
0	0	0	0	0	0	0	0	0	0	0	0	0	0	0	0	0	0	0
0	0	0	0	0	0	0	0	0	0	0	0	0	0	0	0	0	0	0
0	0	0	0	0	0	0	0	0	0	0	0	0	0	0	0	0	0	0
0	0	0	0	0	0	0	0	0	0	0	0	0	0	0	0	0	0	0
0	0	0	0	0	0	0	0	0	0	0	0	0	0	0	0	0	0	0
0	0	0	0	0	0	0	0	0	0	0	0	0	0	0	0	0	0	0
0	0	0	0	0	0	0	0	0	0	0	0	0	0	0	0	0	0	0
0	0	0	0	0	0	0	0	0	0	0	0	0	0	0	0	0	0	0
0	0	0	0	0	0	0	0	0	0	0	0	0	0	0	0	0	0	0
0	0	0	0	0	0	0	0	0	0	0	0	0	0	0	0	0	0	0
0	0	0	0	0	0	0	0	0	0	0	0	0	0	0	0	0	0	0
0	0	0	0	0	0	0	0	0	0	0	0	0	0	0	0	0	0	0
0	0	0	0	0	0	0	0	0	0	0	0	0	0	0	0	0	0	0
0	0	0	0	0	0	0	0	0	0	0	0	0	0	0	0	0	0	0
0	0	0	0	0	0	0	0	0	0	0	0	0	0	0	0	0	0	0
0	0	0	0	0	0	0	0	0	0	0	0	0	0	0	0	0	0	0

0	0	0	0	0	0	0	0	0	0	0	0	0	0	0	0	0	0	0
0	0	0	0	0	0	0	0	0	0	0	0	0	0	0	0	0	0	0
0	0	0	0	0	0	0	0	0	0	0	0	0	0	0	0	0	0	0
0	0	0	0	0	0	0	0	0	0	0	0	0	0	0	0	0	0	0
0	0	0	0	0	0	0	0	0	0	0	0	0	0	0	0	0	0	0
0	0	0	0	0	0	0	0	0	0	0	0	0	0	0	0	0	0	0
0	0	0	0	0	0	0	0	0	0	0	0	0	0	0	0	0	0	0
0	0	0	0	0	0	0	0	0	0	0	0	0	0	0	0	0	0	0
0	0	0	0	0	0	0	0	0	0	0	0	0	0	0	0	0	0	0
0	0	0	0	0	0	0	0	0	0	0	0	0	0	0	0	0	0	0
0	0	0	0	0	0	0	0	0	0	0	0	0	0	0	0	0	0	0
0	0	0	0	0	0	0	0	0	0	0	0	0	0	0	0	0	0	0
0	0	0	0	0	0	0	0	0	0	0	0	0	0	0	0	0	0	0
0	0	0	0	0	0	0	0	0	0	0	0	0	0	0	0	0	0	0
0	0	0	0	0	0	0	0	0	0									
Jan_DL107	1277	354	85	804	63	172	308	275	745	166	177	218	145	201	85	62	175	184
139	0	58	44	165	47	50	94	95	116	125	105	119	42	84	61	80	0	74
95	69	212	83	44	58	69	103	129	19	131	54	21	92	158	63	388	106	55
56	94	54	35	126	0	63	45	25	7	29	45	0	71	4	11	15	30	0
21	40	78	41	0	0	49	70	0	13	0	0	17	14	76	82	0	86	0
31	21	0	0	0	5	0	0	0	13	26	13	0	0	0	18	0	18	0
102	13	0	22	0	14	0	0	0	0	0	0	0	0	7	10	0	0	0
0	0	95	0	32	0	0	0	0	18	20	0	11	0	0	10	0	0	0
0	0	0	6	0	4	51	0	0	0	0	0	9	0	0	0	0	0	8
0	34	29	0	0	0	0	27	0	0	0	0	0	10	14	28	0	0	0
0	0	0	0	0	0	81	0	0	0	0	34	0	0	0	0	0	0	0
0	0	18	0	0	0	0	0	0	0	0	9	13	0	0	0	0	0	0
74	0	0	0	12	0	0	0	8	0	0	0	0	0	0	0	0	0	0
0	0	0	0	0	0	6	12	0	18	0	0	0	0	6	0	26	11	0
11	0	0	0	0	0	0	0	0	0	6	6	0	0	0	0	0	0	0
0	0	0	0	0	0	0	0	0	0	0	0	0	0	0	0	0	0	0
0	3	0	0	0	8	0	0	0	0	0	0	0	0	0	5	0	2	0
0	0	0	0	0	0	0	0	0	0	0	0	0	0	0	14	0	0	0
0	25	0	0	6	0	0	0	0	0	0	18	0	0	0	0	0	0	6
0	0	0	0	0	0	2	0	0	0	0	0	0	0	0	0	0	0	0
0	0	12	0	0	0	0	0	0	0	0	0	0	0	0	0	0	0	0
0	0	16	0	0	0	20	0	0	0	0	0	0	15	0	0	0	0	0

0	0	0	0	0	0	0	0	13	0	0	0	0	0	0	0	0	0	0
0	0	0	0	0	0	16	0	0	0	0	0	0	0	17	0	0	0	0
0	0	0	0	0	0	0	0	0	0	0	0	14	44	0	0	0	0	0
6	0	0	0	8	0	0	0	0	0	0	0	0	0	0	6	0	0	0
0	0	0	0	0	0	0	0	0	0	0	0	0	0	0	0	0	0	0
0	0	0	0	0	0	0	0	0	0	0	0	0	0	0	0	0	0	0
0	0	0	0	0	0	0	0	0	0	20	0	10	6	0	0	0	0	0
0	0	0	0	0	0	0	0	0	0	0	0	0	0	0	0	0	0	0
0	0	0	0	0	0	0	0	0	0	0	0	0	0	0	0	0	0	0
0	0	0	0	0	0	0	0	0	0	0	0	0	0	0	0	0	0	0
0	0	16	0	0	0	0	0	0	0	0	0	0	0	0	0	0	0	0
0	21	0	0	0	0	0	0	0	0	0	0	0	0	0	0	0	0	0
42	0	0	0	0	0	0	0	0	0	0	0	0	0	0	0	0	0	0
0	0	0	0	0	0	0	0	14	0	0	0	0	0	0	0	0	0	0
0	0	0	0	0	0	0	0	0	0	0	0	0	0	0	0	0	0	0
0	0	0	0	0	0	0	0	0	0	0	0	0	0	0	0	0	0	0
0	0	0	0	0	0	0	0	0	0	0	0	0	0	5	0	0	41	0
0	0	0	0	0	0	0	0	0	0	0	0	0	0	0	0	0	0	0
0	0	0	0	0	0	0	0	7	0	0	0	0	0	0	0	0	0	0
0	0	0	0	0	0	0	0	0	0	0	0	0	0	0	0	0	0	0
0	0	0	0	0	0	0	0	0	0	0	0	0	0	0	0	0	0	0
0	0	0	0	0	0	0	0	0	0	0	0	0	0	0	0	0	0	0
0	0	0	0	0	0	0	0	0	0	0	0	0	0	0	0	0	0	0
0	0	0	0	0	0	0	0	0	0	0	0	0	0	0	0	0	0	0
0	0	0	0	0	0	0	0	0	0	0	0	0	0	0	0	5	0	0
0	0	0	0	0	0	0	0	0	0	0	0	0	0	0	0	0	0	18
0	0	0	0	0	0	0	0	0	0	0	26	0	0	0	0	0	0	0
0	0	0	0	0	0	0	0	0	0	0	0	0	0	0	0	0	0	0
0	0	0	0	0	0	0	0	0	0	0	0	0	0	0	0	0	0	0
0	0	0	0	0	20	0	0	0	0	0	0	0	0	0	0	0	0	0
0	0	0	0	0	0	0	0	0	0	0	0	0	0	0	0	0	0	0
0	8	0	0	0	0	0	0	0	0	0	0	0	0	0	0	0	0	0
0	0	0	0	0	0	0	0	0	0	0	0	0	0	0	0	0	0	0
0	0	0	0	0	0	0	0	0	0	0	0	0	0	0	0	0	0	0
0	0	0	0	0	0	0	0	0	0	0	0	0	0	0	0	0	0	0
0	0	0	0	0	0	0	0	0	0	0	0	0	0	0	0	0	0	0
0	0	0	0	0	0	0	0	0	0	0	0	0	0	0	0	0	0	0

0	0	0	0	0	0	0	3	0	0	0	0	0	0	0	0	0	0	0
0	0	0	0	0	0	0	0	9	0	0	0	0	0	0	0	0	0	0
0	0	0	0	0	0	0	0	0	0	0	0	0	0	0	0	0	0	0
0	0	0	0	0	0	0	0	0	0	0	0	0	0	0	0	0	0	0
0	0	0	0	0	0	0	0	0	0	0	0	0	0	0	0	0	0	0
0	0	0	0	0	0	0	12	8	0	0	0	0	0	0	0	0	0	0
0	0	0	0	0	0	0	0	0	0	0	0	0	0	0	0	0	0	0
0	0	0	0	0	0	0	11	11	0	0	0	0	0	0	0	0	0	0
0	0	0	0	0	0	0	0	0	0	0	0	0	0	0	0	0	0	0
0	0	0	0	0	0	0	0	0	0	0	0	0	0	0	0	0	0	0
0	0	0	0	0	0	0	0	0	0	0	0	0	0	0	0	0	0	0
0	0	0	0	0	0	0	0	0	0	0	0	0	0	0	0	0	0	0
0	0	0	0	0	0	0	0	0	0	0	0	0	0	0	9	9	0	0
0	0	0	0	0	0	0	0	0	0	0	0	0	0	0	0	0	0	0
0	0	0	0	0	0	0	0	0	0	0	0	0	0	0	0	0	0	0
0	8	0	0	0	0	0	0	0	0	0	0	0	0	0	0	0	0	0
0	0	0	0	0	0	0	0	0	0	0	0	0	0	0	0	0	0	0
0	0	0	0	0	0	0	0	0	0	0	0	0	0	0	0	0	0	0
0	0	0	0	0	0	0	0	0	0	0	0	0	0	0	0	0	0	0
0	0	0	0	0	0	0	0	0	0	0	0	0	0	0	0	0	0	0
0	0	0	0	0	0	0	0	0	0	0	0	0	0	0	0	0	0	0
0	0	0	0	0	0	0	0	0	0	0	0	0	0	0	0	0	0	0
0	0	0	0	0	0	0	0	0	0	0	0	0	0	0	0	0	0	0
0	0	0	0	0	0	0	0	0	0	0	0	0	0	0	0	0	0	0
0	0	0	0	6	0	0	0	0	0	0	0	0	0	0	0	0	0	0
0	0	0	0	0	0	0	0	0	0	0	0	0	0	0	0	0	0	0
0	0	0	0	0	0	0	0	0	0	0	0	0	0	0	0	0	0	0
0	0	0	0	0	0	0	0	0	0	0	0	0	0	0	0	0	0	0
0	0	0	0	0	0	0	0	0	0	0	0	0	0	0	0	0	0	0
0	0	0	0	0	0	0	0	0	0	0	0	0	0	0	0	0	0	0
0	0	0	0	0	0	0	0	0	0	0	0	0	0	3	0	0	0	0
0	0	0	0	0	0	0	0	0	0	0	0	0	0	5	5	0	0	0
0	0	0	0	0	0	0	0	0	0	0	0	0	0	0	0	0	0	0
0	0	0	0	0	0	0	0	0	0	0	0	0	0	0	0	0	0	0
0	0	0	0	0	0	0	0	0	0	0	0	0	0	0	0	0	0	0
0	0	0	0	0	0	0	0	0	0	0	0	0	0	0	0	0	0	0
0	0	0	0	0	0	0	0	0	0	0	0	0	0	0	0	0	0	0

0	0	0	0	0	0	0	0	0	0	0	0	0	0	0	0	0	0	0
0	0	0	0	0	0	0	0	0	0	0	0	0	0	0	0	0	0	0
0	0	0	0	0	0	0	0	0	0	0	0	0	0	0	0	0	0	0
0	0	0	0	0	0	0	0	0	0	0	0	0	0	0	0	0	0	0
0	0	0	0	0	0	0	4	0	0	0	0	0	0	0	0	0	0	0
0	0	0	0	0	0	0	0	0	0	0	0	0	0	0	0	0	0	0
0	0	0	0	0	0	0	0	0	0	0	0	0	0	0	0	0	0	0
0	0	0	0	0	0	0	0	0	0	0	0	0	0	0	0	0	0	0
0	0	0	0	0	0	0	0	0	0	0	0	0	0	0	0	0	0	0
0	0	0	0	0	0	0	0	0	0	0	0	0	0	0	0	0	0	0
0	0	0	0	0	0	0	0	0	0	0	0	0	0	0	0	0	0	0
0	0	0	0	0	0	0	0	0	0	0	0	0	0	0	0	0	0	0
0	0	0	0	0	0	0	0	0	0	0	0	0	0	0	0	0	0	0
0	0	0	0	0	0	0	0	0	0	0	0	0	0	0	0	0	0	0
0	0	0	0	0	0	0	0	0	0	0	0	0	0	0	0	0	0	0
0	0	0	0	0	0	0	0	0	0	0	0	0	0	0	0	0	0	0
0	0	0	0	0	0	0	0	0	0	0	0	0	0	0	0	0	0	0
0	0	0	0	0	0	0	0	0	0	0	3	3	0	0	0	0	0	0
0	0	0	0	0	0	0	0	0	0	0	0	0	0	0	0	0	0	0
0	0	0	0	0	0	0	0	0	0	0	0	0	0	0	0	0	0	0
0	0	0	0	0	0	0	0	0	0	0	0	0	0	0	0	0	0	0
0	0	0	0	0	0	0	0	0	0	0	0	0	0	0	0	0	0	0
0	0	0	0	0	0	0	0	0	0	0	0	0	0	0	0	0	0	0
0	0	0	0	0	0	0	0	0	0	0	0	0	0	0	0	0	0	0
0	0	0	0	0	0	0	0	0	0	0	0	0	0	0	0	0	0	0
0	0	0	0	0	0	0	0	0	0	0	0	0	0	0	0	0	0	0
0	0	0	0	0	0	0	0	0	0	0	0	0	0	0	0	0	0	0
0	0	0	0	0	0	0	0	0	0	0	0	0	0	0	0	0	0	0
0	0	0	0	0	0	0	0	0	0	0	0	0	0	0	0	0	0	0
0	0	0	0	0	0	0	0	0	0	0	0	0	0	0	0	0	0	0
0	0	0	0	0	0	0	0	0	0	0	0	0	0	0	0	0	0	0
0	0	0	0	0	0	0	0	0	0	0	0	0	0	0	0	0	0	0
0	0	0	0	0	0	0	0	0	0	0	0	0	0	0	0	0	0	0
0	0	0	0	0	0	0	0	0	0	0	0	0	0	0	0	0	0	0
0	0	0	0	0	0	0	0	0	0	0	0	0	0	0	0	0	0	0
0	0	0	0	0	0	0	0	0	0	0	0	0	0	0	0	0	0	0
0	0	0	0	0	0	0	0	0	0	0	0	0	0	0	0	0	0	0

0	0	0	0	0	0	0	0	0	0	0	0	0	0	0	0	0	0	0
0	0	0	0	0	0	0	0	0	0	0	0	0	0	0	0	0	0	0
0	0	0	0	0	0	0	0	0	0	0	0	0	0	0	0	0	0	0
0	0	0	0	2	2	2	2	2	2	2	0	0	0	0	0	0	0	0
0	0	0	0	0	0	0	0	0	0	0	0	0	0	0	0	0	0	0
0	0	0	0	0	0	0	0	0	0	0	0	0	0	0	0	0	0	0
0	0	0	0	0	0	0	0	0	0	0	0	0	0	0	0	0	0	0
0	0	0	0	0	0	0	0	0	0	0	0	0	0	0	0	0	0	0
0	0	0	0	0	0	0	0	0	0	0	0	0	0	0	0	0	0	0
0	0	0	0	0	0	0	0	0	0	0	0	0	0	0	0	0	0	0
0	0	0	0	0	0	0	0	0	0	0	0	0	0	0	0	0	0	0
0	0	0	0	0	0	0	0	0	0	0	0	0	0	0	0	0	0	0
0	0	0	0	0	0	0	0	0	0	0	0	0	0	0	0	0	0	0
0	0	0	0	0	0	0	0	0	0	0	0	0	0	0	0	0	0	0
0	0	0	0	0	0	0	0	0	0	0	0	0	0	0	0	0	0	0
0	0	0	0	0	0	0	0	0	0	0	0	0	0	0	0	0	0	0
0	0	0	0	0	0	0	0	0	0	0	0	0	0	0	0	0	0	0
0	0	0	0	0	0	0	0	0	0	0	0	0	0	0	0	0	0	0
0	0	0	0	0	0	0	0	0	0	0	0	0	0	0	0	0	0	0
0	0	0	0	0	0	0	0	0	0	0	0	0	0	0	0	0	0	0
0	0	0	0	0	0	0	0	0	0	0	0	0	0	0	0	0	0	0
0	0	0	0	0	0	0	0	0	0	0	0	0	0	0	0	0	0	0
0	0	0	0	0	0	0	0	0	0	0	0	0	0	0	0	0	0	0
0	0	0	0	0	0	0	0	0	0	0	0	0	0	0	0	0	0	0
0	0	0	0	0	0	0	0	0	0	0	0	0	0	0	0	0	0	0
0	0	0	0	0	0	0	0	0	0	0	0	0	0	0	0	0	0	0
0	0	0	0	0	0	0	0	0	0	0	0	0	0	0	0	0	0	0
0	0	0	0	0	0	0	0	0	0	0	0	0	0	0	0	0	0	0
0	0	0	0	0	0	0	0	0	0	0	0	0	0	0	0	0	0	0
0	0	0	0	0	0	0	0	0	0	0	0	0	0	0	0	0	0	0
0	0	0	0	0	0	0	0	0	0	0	0	0	0	0	0	0	0	0
0	0	0	0	0	0	0	0	0	0	0	0	0	0	0	0	0	0	0
0	0	0	0	0	0	0	0	0	0	0	0	0	0	0	0	0	0	0
0	0	0	0	0	0	0	0	0	0	0	0	0	0	0	0	0	0	0
0	0	0	0	0	0	0	0	0	0	0	0	0	0	0	0	0	0	0
0	0	0	0	0	0	0	0	0	0	0	0	0	0	0	0	0	0	0
0	0	0	0	0	0	0	0	0	0	0	0	0	0	0	0	0	0	0

0	0	0	0	0	0	0	0	0	0	0	0	0	0	0	0	0	0	0
0	0	0	0	0	0	0	0	0	0	0	0	0	0	0	0	0	0	0
0	0	0	0	0	0	0	0	0	0	0	0	0	0	0	0	0	0	0
0	0	0	0	0	0	0	0	0	0	0	0	0	0	0	0	0	0	0
0	0	0	0	0	0	0	0	0	0	0	0	0	0	0	0	0	0	0
0	0	0	0	0	0	0	0	0	0	0	0	0	0	0	0	0	0	0
0	0	0	0	0	0	0	0	0	0									
Jan_DL108	1748	1277	1085	620	45	390	165	140	372	175	126	455	150	144	124	446	134	154
217	649	227	107	176	183	192	216	148	175	145	140	152	135	80	0	135	449	115
160	47	236	83	137	65	158	365	143	188	113	141	61	77	120	119	52	95	88
68	73	116	38	92	135	78	86	37	92	39	118	132	75	7	135	25	34	0
62	37	53	49	42	85	72	53	0	34	68	0	22	0	33	0	40	0	104
29	106	0	0	0	10	0	31	0	87	0	0	0	0	15	0	0	0	0
62	15	0	57	13	0	0	0	0	0	18	14	13	13	0	0	0	0	0
0	0	60	0	38	0	0	0	0	17	0	0	0	0	18	5	0	0	0
0	0	10	29	0	0	0	0	0	8	6	0	14	18	0	23	0	0	0
16	24	14	33	0	0	0	0	0	46	0	14	0	0	0	5	0	0	0
0	0	0	0	0	0	0	0	0	0	0	27	0	0	0	0	0	10	0
16	0	0	0	0	0	0	0	0	0	0	0	0	72	0	92	0	22	0
29	0	0	0	0	0	0	0	0	0	0	0	15	26	0	0	0	32	0
0	0	0	0	0	0	0	0	0	0	0	0	0	0	5	0	0	0	0
0	0	0	19	0	9	0	0	0	0	0	0	0	0	0	3	0	0	0
0	0	14	0	0	0	0	0	9	0	0	0	2	0	19	0	0	0	43
0	0	8	0	0	4	0	0	0	0	28	0	8	0	0	0	0	0	0
0	12	4	72	0	0	0	0	4	0	0	0	0	0	0	5	11	5	0
0	7	0	0	0	4	0	0	0	0	0	0	0	0	0	0	15	0	0
8	0	0	0	9	0	0	0	0	10	0	0	26	0	15	0	0	0	0
11	0	0	0	14	0	3	0	0	0	0	0	0	0	0	0	0	0	0
0	0	0	0	0	7	0	0	0	18	0	0	0	0	0	0	0	0	0
0	0	0	0	0	0	0	0	0	0	0	0	0	0	0	0	0	0	0
0	2	0	0	0	17	0	0	0	0	0	0	0	0	0	0	0	0	16
0	0	0	0	0	0	0	0	0	0	0	0	0	0	0	0	0	0	0
0	0	0	0	5	0	0	0	0	0	6	0	0	0	0	0	0	0	0
0	0	0	0	0	0	0	0	0	0	0	0	0	0	0	0	0	6	0
0	0	0	0	0	0	0	0	0	0	0	0	0	0	0	0	0	0	73
0	0	0	0	0	0	0	0	0	0	11	0	0	0	12	0	81	0	0
0	0	0	0	0	0	0	0	0	0	0	0	0	0	0	0	0	0	0

0	0	0	0	0	0	0	0	0	0	0	0	0	0	0	0	0	0	7
0	0	0	0	0	0	0	0	0	0	0	26	0	0	0	0	0	0	0
0	0	0	0	0	0	0	0	0	0	0	0	0	0	0	0	0	0	0
0	0	2	0	0	0	0	0	0	0	0	0	0	0	0	0	0	0	0
0	0	0	0	0	0	0	0	0	0	0	0	0	0	0	0	0	0	0
0	0	0	0	0	0	0	0	0	0	0	0	0	0	0	0	0	0	0
0	0	0	0	0	0	0	0	0	0	0	0	0	0	0	0	0	0	0
0	0	0	0	0	0	0	0	0	0	0	0	0	0	0	0	0	0	0
0	0	0	0	0	0	0	0	0	0	0	0	0	0	0	0	0	0	0
0	25	0	0	0	0	0	0	0	0	0	0	0	0	0	0	0	0	0
0	0	0	0	6	0	0	0	0	0	0	0	0	0	0	0	0	0	0
0	0	0	0	0	0	0	0	0	0	0	0	0	0	0	0	0	0	0
0	0	0	0	0	0	0	0	0	0	4	0	0	0	0	0	0	0	0
0	0	0	0	0	0	0	0	0	0	0	0	0	0	0	0	0	0	0
0	0	0	0	0	0	8	0	0	0	0	0	0	0	0	0	0	0	0
0	0	0	0	0	0	0	0	0	0	0	0	0	0	0	0	0	0	0
3	0	0	0	0	0	0	0	9	0	0	0	0	0	5	0	0	7	0
0	0	0	0	0	4	0	0	0	0	0	0	0	0	0	0	0	0	0
0	0	0	0	0	0	0	0	0	0	0	0	0	0	0	0	0	0	0
0	0	0	0	0	0	0	0	0	0	0	0	0	0	0	0	0	0	0
0	0	0	0	0	0	0	0	0	0	0	0	0	0	0	0	0	0	0
0	0	0	0	0	0	0	0	0	0	0	0	0	0	0	0	0	0	0
0	0	0	0	0	0	0	0	0	0	0	0	0	0	0	0	0	0	0
0	0	0	0	29	0	0	0	0	0	0	0	0	0	0	0	0	0	0
0	0	0	0	0	0	0	0	0	0	0	0	0	0	0	0	0	0	0
0	0	0	0	0	0	0	0	0	0	0	0	0	0	0	0	0	0	0
0	0	0	0	0	0	0	0	0	0	0	0	0	0	0	0	0	0	0
0	0	0	0	0	0	0	0	0	0	0	0	0	0	0	0	0	0	0
0	0	0	0	0	9	0	0	0	0	0	0	0	0	0	0	0	0	0
0	0	0	2	0	0	0	0	0	0	0	0	0	0	0	0	0	0	0
0	0	0	0	0	0	0	0	0	0	0	0	0	0	0	0	0	0	0
0	0	0	0	0	4	0	0	0	0	0	0	0	0	0	0	0	0	0
0	0	0	0	0	0	0	0	0	0	0	0	0	0	0	0	0	0	0
0	0	0	0	0	0	0	0	0	0	0	0	0	0	0	0	0	0	0
0	0	0	0	0	0	0	0	0	0	0	0	0	0	0	0	0	0	0
0	0	0	0	0	0	0	0	0	0	0	0	0	0	0	0	0	0	0
0	0	0	0	0	0	0	0	0	0	0	0	0	0	0	0	0	0	0

0	0	0	0	0	0	0	0	0	0	0	0	0	0	0	0	0	0	3
0	0	0	0	0	0	0	0	0	0	0	0	0	0	0	0	0	0	0
0	0	0	0	0	0	0	0	0	0	0	0	0	0	0	0	0	0	0
0	0	0	0	0	0	0	0	0	0	0	0	0	0	0	0	0	0	0
0	0	0	0	0	0	0	0	0	0	0	0	0	0	0	0	0	0	0
0	0	0	0	0	0	0	0	0	0	0	0	0	0	0	0	0	0	0
0	0	0	0	0	0	0	0	0	0	0	0	0	0	0	0	0	0	0
0	0	0	0	0	0	0	0	0	0	0	0	0	0	0	0	0	0	0
0	0	0	0	0	0	0	0	0	0	0	0	0	0	0	0	0	0	0
0	0	0	0	0	0	0	0	0	0	0	0	0	0	0	0	0	0	0
0	0	0	0	0	0	0	0	0	0	0	0	0	0	0	0	0	0	0
0	0	0	0	0	0	0	0	0	0	0	0	0	0	0	0	0	0	0
0	0	0	0	0	0	0	0	0	0	0	0	0	0	0	0	0	0	0
0	0	0	10	0	0	0	0	0	0	0	0	0	0	0	0	0	0	0
0	0	0	0	0	0	0	0	0	0	0	0	0	0	0	0	0	0	0
0	0	0	0	0	0	0	0	0	0	0	0	0	0	0	0	0	0	0
0	0	0	0	0	0	0	0	0	0	0	0	0	0	0	9	9	0	0
0	0	0	0	0	0	0	0	0	0	0	0	0	0	0	0	0	0	0
0	0	0	0	0	0	0	0	0	0	0	0	0	0	0	0	0	0	0
0	0	0	0	0	0	0	0	0	0	0	0	0	0	0	0	0	0	0
0	0	0	0	0	0	0	0	0	0	0	0	0	0	0	0	0	0	0
0	8	8	0	0	0	0	0	0	0	0	0	0	0	0	0	0	0	0
0	0	0	0	0	0	0	0	0	0	0	0	0	0	0	0	0	0	0
0	0	0	0	0	0	0	0	0	0	0	0	0	0	0	0	0	0	0
0	0	0	0	0	0	0	0	0	0	0	0	0	0	0	0	0	0	0
0	0	0	0	0	0	0	0	0	0	0	0	0	0	0	0	0	0	0
0	0	0	0	0	0	0	0	0	0	0	0	0	0	0	0	0	0	0
0	0	0	0	0	0	0	0	0	0	0	0	0	0	0	0	0	0	0
0	0	0	0	0	0	0	0	0	0	0	0	0	0	0	0	0	0	0
0	0	0	0	0	0	0	0	0	0	0	0	0	0	0	0	0	0	0
0	0	0	0	0	0	0	0	0	0	0	0	0	0	0	0	0	0	0
0	0	0	0	0	0	0	0	0	0	0	0	0	0	0	0	0	0	0
0	0	0	0	0	0	0	0	0	0	0	0	0	0	0	0	0	0	0
0	0	0	0	0	0	0	0	0	0	0	0	0	0	0	0	0	0	6
6	0	0	0	0	0	0	0	0	0	0	0	0	0	0	0	0	0	0
0	0	0	0	0	0	0	0	0	0	0	0	0	0	0	0	0	0	0
0	0	0	0	0	0	0	0	0	0	0	0	0	0	0	0	0	0	0

0	0	0	0	0	0	0	0	0	0	0	0	0	0	0	0	0	0	0
0	0	0	0	0	0	0	0	0	0	0	0	0	0	0	0	0	0	0
0	0	0	0	0	0	0	0	0	0	0	0	0	0	0	0	0	0	0
0	0	0	0	0	0	0	0	0	0	0	0	0	0	0	0	0	0	0
0	0	0	0	0	0	0	0	0	0	0	0	0	0	0	0	0	0	0
0	0	0	0	0	0	0	0	0	0	0	0	0	0	0	0	0	0	0
0	0	0	0	0	0	0	0	0	0	0	0	0	0	0	0	0	0	0
0	0	0	0	0	0	0	0	0	0	0	0	0	0	0	0	0	0	0
0	0	0	0	0	0	0	0	0	0	0	0	0	0	0	0	0	0	0
0	0	0	0	0	0	0	0	0	0	0	0	0	0	0	0	0	0	0
0	0	0	0	0	0	0	0	0	0	0	0	0	0	0	0	0	0	0
0	0	0	0	0	0	0	0	0	0	0	0	0	0	0	0	0	0	0
0	0	0	0	0	0	0	0	0	0	0	0	0	0	0	0	0	0	0
0	0	0	0	0	0	0	0	0	0	0	0	0	0	0	0	0	0	0
0	0	0	0	0	0	0	0	0	0	0	0	0	0	0	0	0	0	0
0	0	0	0	0	0	0	0	0	0	0	0	0	0	0	0	4	4	0
0	0	0	0	0	0	0	0	0	0	0	0	0	0	0	0	0	0	0
0	0	0	0	0	0	0	0	0	0	0	0	0	0	0	0	0	0	0
0	0	0	0	0	0	0	0	0	0	0	0	0	0	0	0	0	0	0
0	0	0	0	0	0	0	0	0	0	0	0	0	0	0	0	0	0	0
0	0	0	0	0	0	0	0	0	0	0	0	0	0	0	0	0	0	0
0	0	0	0	0	0	0	0	0	0	0	0	0	0	0	0	0	0	0
0	0	0	0	0	0	0	0	0	0	0	0	0	0	0	0	0	0	0
0	0	0	0	0	0	0	0	0	0	0	0	0	0	0	0	0	0	0
0	0	0	0	0	0	0	0	0	0	0	0	0	0	0	0	0	0	0
0	0	0	0	0	0	0	0	0	0	0	0	0	0	0	0	0	0	0
0	0	0	0	0	0	0	0	0	0	0	0	0	0	0	0	0	0	0
0	0	0	0	0	0	0	0	0	0	0	0	0	0	0	0	0	0	0
0	0	0	0	0	0	0	0	0	0	0	0	0	0	0	0	0	0	0
0	0	0	0	0	0	0	0	0	0	0	0	0	0	0	0	0	0	0
0	0	0	0	0	0	0	0	0	0	0	0	0	0	0	0	0	0	0
0	0	0	0	0	0	0	0	0	0	0	0	0	0	0	0	0	0	0
0	0	0	0	0	0	0	0	0	0	0	0	0	0	0	0	0	0	0
0	0	0	0	0	0	0	0	0	0	0	0	0	0	0	3	0	0	0
0	0	0	0	0	0	0	0	0	0	0	0	0	0	0	0	0	0	0
0	0	0	0	0	0	0	0	0	0	0	0	0	0	0	0	0	0	0
0	0	0	0	0	0	0	0	0	0	0	0	0	0	0	0	0	0	0

0	0	0	0	0	0	0	0	0	0	0	0	0	0	0	0	0	0	0
0	0	0	0	0	0	0	0	0	0	0	0	0	0	0	0	0	0	0
0	0	0	0	0	0	0	0	0	0	0	0	0	0	0	0	0	0	0
0	0	0	0	0	0	0	0	0	0	0	0	0	0	0	0	0	0	0
0	0	0	0	0	0	0	0	0	0	0	0	0	0	0	0	0	0	0
0	0	0	0	0	0	0	0	0	0	0	0	0	0	0	0	0	0	0
0	0	0	0	0	0	0	0	0	0	0	0	0	0	0	0	0	0	0
0	0	0	0	0	0	0	0	0	0	0	0	0	0	0	0	0	0	0
0	0	0	0	0	0	0	0	0	0	0	0	0	0	0	0	0	0	0
0	0	0	0	0	0	0	0	0	0	0	0	0	0	0	0	0	0	0
0	0	0	0	0	0	0	0	0	0	0	0	0	0	0	0	0	0	0
0	0	0	0	0	0	0	0	0	0	0	0	0	0	0	0	0	0	0
0	0	0	0	0	0	0	0	0	0	0	0	0	0	0	0	0	0	0
0	0	0	0	0	0	0	0	0	0	0	0	0	0	0	0	0	0	0
0	0	0	0	0	0	0	0	0	0	0	0	0	0	0	0	0	0	0
0	0	0	0	0	0	0	0	0	0	0	0	0	0	0	0	0	0	0
0	0	0	0	0	0	0	0	0	0	0	0	0	0	0	0	0	0	0
0	0	0	0	0	0	0	0	0	0	0	0	0	0	0	0	0	0	0
0	0	0	0	0	0	0	0	0	0	0	0	0	0	0	0	0	0	0
0	0	0	0	0	0	0	0	0	0	0	0	0	0	0	0	0	0	0
0	0	0	0	0	0	0	0	0	0	0	0	0	0	0	0	0	0	0
0	0	0	0	0	0	0	0	0	0	0	0	0	0	0	0	0	0	0
0	0	0	0	0	0	0	0	0	2	2	2	2	0	0	0	0	0	0
0	0	0	0	0	0	0	0	0	0	0	0	0	0	0	0	0	0	0
0	0	0	0	0	0	0	0	0	0	0	0	0	0	0	0	0	0	0
0	0	0	0	0	0	0	0	0	0	0	0	0	0	0	0	0	0	0
0	0	0	0	0	0	0	0	0	0	0	0	0	0	0	0	0	0	0
0	0	0	0	0	0	0	0	0	0	0	0	0	0	0	0	0	0	0
0	0	0	0	0	0	0	0	0	0	0	0	0	0	0	0	0	0	0
0	0	0	0	0	0	0	0	0	0	0	0	0	0	0	0	0	0	0
0	0	0	0	0	0	0	0	0	0	0	0	0	0	0	0	0	0	0
0	0	0	0	0	0	0	0	0	0	0	0	0	0	0	0	0	0	0
0	0	0	0	0	0	0	0	0	0	0	0	0	0	0	0	0	0	0
0	0	0	0	0	0	0	0	0	0	0	0	0	0	0	0	0	0	0
0	0	0	0	0	0	0	0	0	0	0	0	0	0	0	0	0	0	0
0	0	0	0	0	0	0	0	0	0									

Jan_DL114	813	1173	182	252	1061	199	171	135	72	128	212	49	84	96	443	51	70	97
64	31	123	0	111	158	32	81	49	51	54	0	16	76	54	106	54	35	0
0	357	0	10	75	76	0	16	16	18	0	0	323	42	22	27	0	0	0
0	29	0	94	0	54	0	76	40	152	0	70	0	0	10	0	17	19	0
26	0	0	15	0	0	10	24	110	7	0	0	57	0	19	0	34	0	0
24	0	0	0	0	0	7	74	71	6	0	0	110	0	0	46	41	114	64
0	17	20	7	17	26	0	0	0	0	0	0	25	0	0	0	0	103	0
0	0	0	93	15	0	0	0	0	9	0	0	11	0	0	0	0	0	0
0	0	25	7	0	0	0	31	8	35	15	0	0	0	0	0	0	0	0
0	0	0	0	0	0	36	0	0	38	20	0	0	22	0	9	36	0	0
0	93	0	15	59	31	0	0	0	8	0	0	27	0	27	58	0	0	0
8	0	0	0	15	0	0	0	0	0	0	0	0	0	0	0	0	6	0
11	0	0	0	0	0	0	0	0	33	0	0	6	0	0	0	0	3	0
10	0	0	12	0	0	5	0	0	0	0	0	0	0	21	13	0	0	27
0	0	0	0	0	3	0	0	0	0	21	0	0	5	0	6	0	0	0
0	0	14	0	0	0	0	0	4	0	46	0	0	0	0	0	0	0	0
0	5	0	0	0	0	0	0	0	0	0	0	0	0	0	0	0	0	0
0	0	0	6	0	0	0	0	0	0	0	0	0	10	0	0	0	0	0
0	0	6	0	10	0	0	0	0	0	0	11	8	0	0	0	0	0	0
0	0	0	0	0	0	0	8	0	0	0	0	0	0	0	0	0	0	0
0	0	0	0	0	0	0	15	0	0	0	0	0	6	0	0	16	0	0
0	0	0	0	0	0	0	0	0	0	0	0	0	0	0	0	2	0	0
0	0	0	0	0	0	0	0	0	0	13	0	0	0	0	0	0	0	0
0	0	0	0	0	0	0	27	0	0	0	0	0	0	0	0	0	8	0
0	11	0	0	0	0	0	0	0	0	0	0	0	0	0	0	0	0	0
0	0	0	0	0	0	0	0	0	0	0	0	33	0	11	0	0	0	0
0	0	11	0	0	0	0	0	0	0	0	14	0	0	0	0	0	0	0
0	0	0	0	0	0	0	0	1	0	0	0	0	0	0	0	0	0	0
0	0	0	0	0	0	0	0	0	0	0	0	0	0	0	0	0	0	0
19	0	0	0	0	0	0	0	0	0	8	0	0	0	0	0	0	6	0
0	0	11	0	0	0	0	0	0	0	0	0	0	0	0	0	0	0	0
0	0	0	0	0	0	0	0	0	0	0	0	0	0	0	0	0	0	0
0	0	0	0	0	0	0	0	0	19	0	0	5	0	0	0	0	0	0
0	0	61	0	0	0	0	0	0	0	0	0	0	0	0	0	0	0	0
0	0	0	0	0	0	0	16	0	0	0	0	0	0	0	0	0	0	0
0	0	0	0	0	0	0	0	0	0	0	0	0	0	0	0	0	0	0
0	0	0	0	0	0	0	0	0	0	0	0	0	0	0	0	0	0	0

0	0	0	0	0	0	0	0	0	0	0	0	0	0	0	0	14	0	0
0	0	0	0	0	0	0	0	0	0	0	0	0	0	0	0	0	0	0
0	0	0	0	0	0	0	0	0	0	0	0	0	0	0	0	0	0	0
0	0	0	0	0	0	0	0	0	0	0	0	0	0	0	0	0	0	0
0	0	0	0	0	0	0	0	0	0	0	0	0	0	0	0	0	0	0
0	0	0	0	0	0	0	8	0	0	0	0	0	0	0	0	0	0	0
0	0	0	0	0	0	0	0	0	0	0	0	0	0	0	0	0	0	0
0	0	0	0	0	0	0	0	0	0	0	0	0	0	0	0	0	0	0
0	0	0	0	0	0	0	0	0	0	0	0	0	3	0	0	0	0	0
0	0	0	0	0	0	0	0	0	0	11	0	0	0	0	0	0	0	3
0	0	0	0	0	0	0	0	0	0	0	0	0	0	0	0	0	0	0
0	0	0	0	0	0	0	0	0	0	0	0	0	0	11	0	0	0	0
0	0	0	0	0	0	0	0	0	0	0	0	0	0	0	0	0	0	0
0	0	0	0	0	0	0	0	0	0	0	0	0	0	0	24	24	0	0
0	0	0	0	0	0	0	0	9	23	0	0	0	0	6	0	0	0	0
0	0	0	0	0	0	0	0	0	0	0	0	0	0	0	0	0	0	0
0	0	0	0	0	0	0	0	0	0	0	0	0	0	0	0	0	0	0
0	0	0	0	0	0	0	0	0	0	0	0	0	0	0	0	0	0	4
0	0	0	0	0	0	18	0	0	0	0	0	0	0	0	0	0	0	0
0	0	0	0	0	0	17	17	0	0	0	0	0	0	0	0	0	0	0
0	0	0	0	0	0	0	0	0	0	0	0	0	0	0	0	0	0	0
0	0	0	0	0	0	0	0	0	0	0	0	0	0	0	0	0	0	0
0	0	0	0	0	0	0	0	0	0	0	0	0	0	0	0	0	0	0
0	0	0	0	0	0	0	0	0	0	0	0	0	13	13	0	0	0	0
0	0	0	0	0	0	0	0	0	0	0	0	0	0	0	0	0	0	0
0	0	0	0	0	0	0	0	0	0	0	0	0	0	0	0	0	0	0
0	0	0	0	0	0	0	0	0	0	0	0	0	0	0	0	0	0	0
0	0	0	0	0	0	0	0	0	0	0	0	0	0	0	0	0	0	0
0	0	0	0	0	0	0	0	0	0	0	0	0	0	0	0	0	0	0
0	0	10	0	0	0	0	0	0	0	0	0	0	0	0	0	0	0	0
0	0	0	0	0	0	0	0	0	0	0	0	0	0	0	0	0	0	0
0	0	0	0	0	0	0	6	9	9	0	0	0	0	0	0	0	0	0
0	0	0	0	0	0	0	0	0	0	0	0	0	0	0	0	0	0	0
0	0	0	0	0	0	0	0	0	0	0	0	0	0	0	0	0	0	0
0	0	0	0	0	0	0	0	0	0	0	0	0	0	0	0	0	0	0
0	0	3	0	0	0	8	8	0	0	0	0	0	0	0	0	0	0	0
0	0	0	0	0	0	0	0	0	0	0	0	0	0	0	0	0	0	0

0	0	0	0	0	0	0	0	0	0	0	0	0	0	0	0	7	7	0
0	0	0	0	0	0	0	0	0	0	0	0	0	0	0	0	0	0	0
0	0	0	0	0	0	0	0	0	0	0	0	0	0	0	0	0	0	0
0	0	0	0	0	0	0	0	0	0	0	0	0	0	0	0	0	0	0
0	0	0	0	0	0	0	0	0	0	0	0	0	0	0	0	0	0	0
0	0	0	0	0	0	0	0	0	0	0	0	0	6	0	0	0	0	0
0	0	0	0	0	0	0	0	0	0	0	0	0	0	0	0	0	0	0
0	0	0	0	0	0	0	0	0	0	0	0	0	0	0	0	0	0	0
0	0	0	0	0	0	0	0	0	0	0	0	0	0	0	0	0	0	0
0	0	0	0	0	0	0	0	0	0	0	0	0	0	0	0	0	0	0
0	0	0	0	0	0	0	0	0	0	0	0	0	0	0	0	0	0	0
0	0	0	0	0	0	0	0	0	0	0	0	0	0	0	0	0	0	0
0	0	5	5	0	0	0	0	0	0	0	0	0	0	0	0	0	0	0
0	0	0	0	0	0	0	0	0	0	0	0	0	0	0	0	0	0	0
0	0	0	0	0	0	0	0	0	0	0	0	0	0	0	0	0	0	0
0	0	0	0	0	0	0	0	0	0	0	0	0	0	0	0	0	0	0
0	0	0	0	0	0	0	0	0	0	0	0	0	0	0	0	0	0	0
0	0	0	0	0	0	0	0	0	0	0	0	0	0	0	0	0	0	0
0	0	0	0	0	0	0	0	0	0	0	0	0	0	0	0	0	0	0
0	0	0	0	0	0	0	0	0	0	0	0	0	0	0	0	0	0	0
0	0	0	0	0	0	0	0	0	0	0	0	0	0	0	0	0	0	0
0	0	0	0	0	0	0	0	0	4	4	0	0	0	0	0	0	0	0
0	0	0	0	0	0	0	0	0	0	0	0	0	0	0	0	0	0	0
0	0	0	0	0	0	0	0	0	0	0	0	0	0	0	0	0	0	0
0	0	0	0	0	0	0	0	0	0	0	0	0	0	0	0	0	0	0
0	0	0	0	0	0	0	0	0	0	0	0	0	0	0	0	0	0	0
0	0	0	0	0	0	0	0	0	0	0	0	0	0	0	0	0	0	0
0	0	0	0	0	0	0	0	0	0	0	0	0	0	0	0	0	0	0
0	0	0	0	0	0	0	0	0	0	0	0	0	0	0	0	0	0	0
0	0	0	0	0	0	0	0	0	0	0	0	0	0	0	0	0	0	0
0	0	0	0	0	0	0	0	0	0	0	0	0	0	0	0	0	0	0
0	0	0	0	0	0	0	0	0	0	0	0	0	0	0	0	0	0	0
0	0	0	0	0	0	0	0	0	0	0	0	0	0	0	0	0	0	0
0	0	0	0	0	0	0	0	0	0	0	0	0	0	0	0	0	0	0
0	0	0	0	0	0	0	0	0	0	0	0	3	3	3	0	0	0	0
0	0	0	0	0	0	0	0	0	0	0	0	0	0	0	0	0	0	0
0	0	0	0	0	0	0	0	0	0	0	0	0	0	0	0	0	0	0

0	0	0	0	0	0	0	0	0	0	0	0	0	0	0	0	0	0	0
0	0	0	0	0	0	0	0	0	0	0	0	0	0	0	0	0	0	0
0	0	0	0	0	0	0	0	0	0	0	0	0	0	0	0	0	0	0
0	0	0	0	0	0	0	0	0	0	0	0	0	0	0	0	0	0	0
0	0	0	0	0	0	0	0	0	0	0	0	0	0	0	0	0	0	0
0	0	0	0	0	0	0	0	0	0	0	0	0	0	0	0	0	0	0
0	0	0	0	0	0	0	0	0	0	0	0	0	0	0	0	0	0	0
0	0	0	0	0	0	0	0	0	0	0	0	0	0	0	0	0	0	0
0	0	0	0	0	0	0	0	0	0	0	0	0	0	0	0	0	0	0
0	0	0	0	0	0	0	0	0	0	0	0	0	0	0	0	0	0	0
0	0	0	0	0	0	0	0	0	0	0	0	0	0	0	0	0	0	0
0	0	0	0	0	0	0	0	0	0	0	0	0	0	0	0	0	0	0
0	0	0	0	0	0	0	0	0	0	0	0	0	0	0	0	0	0	0
0	0	0	0	0	0	0	0	0	0	0	0	0	0	0	0	0	0	0
0	0	0	0	0	0	0	0	0	0	0	0	0	0	0	0	0	0	0
0	0	0	0	0	0	0	0	0	0	0	0	0	0	0	0	0	0	0
0	0	0	0	0	0	0	0	0	0	0	0	0	0	0	0	0	0	0
0	0	0	0	0	0	0	0	0	0	0	0	0	0	0	0	0	0	0
0	0	0	0	0	0	0	0	0	0	0	0	0	0	0	0	0	0	0
0	0	0	0	0	0	0	0	0	0	0	0	2	2	2	2	2	2	2
0	0	0	0	0	0	0	0	0	0	0	0	0	0	0	0	0	0	0
0	0	0	0	0	0	0	0	0	0	0	0	0	0	0	0	0	0	0
0	0	0	0	0	0	0	0	0	0	0	0	0	0	0	0	0	0	0
0	0	0	0	0	0	0	0	0	0	0	0	0	0	0	0	0	0	0
0	0	0	0	0	0	0	0	0	0	0	0	0	0	0	0	0	0	0
0	0	0	0	0	0	0	0	0	0	0	0	0	0	0	0	0	0	0
0	0	0	0	0	0	0	0	0	0	0	0	0	0	0	0	0	0	0
0	0	0	0	0	0	0	0	0	0	0	0	0	0	0	0	0	0	0
0	0	0	0	0	0	0	0	0	0	0	0	0	0	0	0	0	0	0
0	0	0	0	0	0	0	0	0	0	0	0	0	0	0	0	0	0	0
0	0	0	0	0	0	0	0	0	0	0	0	0	0	0	0	0	0	0
0	0	0	0	0	0	0	0	0	0	0	0	0	0	0	0	0	0	0
0	0	0	0	0	0	0	0	0	0	0	0	0	0	0	0	0	0	0
0	0	0	0	0	0	0	0	0	0	0	0	0	0	0	0	0	0	0
0	0	0	0	0	0	0	0	0	0	0	0	0	0	0	0	0	0	0
0	0	0	0	0	0	0	0	0	0	0	0	0	0	0	0	0	0	0
0	0	0	0	0	0	0	0	0	0	0	0	0	0	0	0	0	0	0

0	0	0	0	0	0	0	0	0	0	0	0	0	0	0	0	0	0	0
0	0	0	0	0	0	0	0	0	0	0	0	0	0	0	0	0	0	0
0	0	0	0	0	0	0	0	0	0	0	0	0	0	0	0	0	0	0
0	0	0	0	0	0	0	0	0	0	0	0	0	0	0	0	0	0	0
0	0	0	0	0	0	0	0	0	0	0	0	0	0	0	0	0	0	0
0	0	0	0	0	0	0	0	0	0	0	0	0	0	0	0	0	0	0
0	0	0	0	0	0	0	0	0	0	0	0	0	0	0	0	0	0	0
0	0	0	0	0	0	0	0	0	0	0	0	0	0	0	0	0	0	0
0	0	0	0	0	0	0	0	0	0	0	0	0	0	0	0	0	0	0
0	0	0	0	0	0	0	0	0	0	0	0	0	0	0	0	0	0	0
0	0	0	0	0	0	0	0	0	0	0	0	0	0	0	0	0	0	0
0	0	0	0	0	0	0	0	0	0	0	0	0	0	0	0	0	0	0
0	0	0	0	0	0	0	0	0	0	0	0	0	0	0	0	0	0	0
0	0	0	0	0	0	0	0	0	0	0	0	0	0	0	0	0	0	0
0	0	0	0	0	0	0	0	0	0	0	0	0	0	0	0	0	0	0
0	0	0	0	0	0	0	0	0	0	0	0	0	0	0	0	0	0	0
0	0	0	0	0	0	0	0	0	0	0	0	0	0	0	0	0	0	0
0	0	0	0	0	0	0	0	0	0	0	0	0	0	0	0	0	0	0
0	0	0	0	0	0	0	0	0	0	0	0	0	0	0	0	0	0	0
0	0	0	0	0	0	0	0	0	0	0	0	0	0	0	0	0	0	0
0	0	0	0	0	0	0	0	0	0	0	0	0	0	0	0	0	0	0
0	0	0	0	0	0	0	0	0	0	0	0	0	0	0	0	0	0	0
0	0	0	0	0	0	0	0	0	0	0	0	0	0	0	0	0	0	0
0	0	0	0	0	0	0	0	0	0	0	0	0	0	0	0	0	0	0
0	0	0	0	0	0	0	0	0	0	0	0	0	0	0	0	0	0	0
0	0	0	0	0	0	0	0	0	0	0	0	0	0	0	0	0	0	0
0	0	0	0	0	0	0	0	0	0	0	0	0	0	0	0	0	0	0
0	0	0	0	0	0	0	0	0	0	0	0	0	0	0	0	0	0	0
0	0	0	0	0	0	0	0	0	0									
Jan_DL115	652	584	808	535	106	168	175	162	259	225	94	504	193	101	173	178	80	123
76	32	68	0	124	81	181	119	169	124	86	0	66	241	89	0	83	409	0
131	104	34	44	108	0	72	444	25	75	0	116	47	0	97	108	208	43	0
0	241	112	32	0	27	0	73	51	165	14	0	0	0	59	19	14	127	17
70	0	47	21	0	39	122	53	39	0	71	0	0	62	33	53	16	0	82
0	80	0	130	82	0	88	0	39	35	0	0	0	9	36	0	0	0	0
0	0	0	35	0	0	0	0	9	0	5	0	4	0	82	48	0	0	77
0	0	0	0	32	67	0	0	0	11	0	0	8	0	0	0	0	0	0

0	0	40	0	0	11	19	0	0	0	0	0	26	0	26	0	0	16	0
25	38	9	0	0	0	0	0	0	0	0	42	0	16	0	0	10	0	0
0	0	0	0	0	0	35	0	0	0	0	0	0	0	0	0	0	0	0
0	0	0	0	0	0	0	0	0	0	63	0	0	16	0	0	32	0	0
0	67	0	0	0	0	0	0	2	11	0	47	11	17	11	0	0	0	0
0	0	0	17	24	0	0	0	0	41	0	36	13	0	0	0	12	0	0
21	0	23	0	0	0	0	0	0	18	0	0	0	0	12	9	0	0	0
70	0	0	0	0	0	0	0	0	0	13	0	0	33	0	0	0	0	27
0	0	9	64	0	0	0	0	0	0	0	0	0	8	0	12	0	0	0
0	0	0	0	0	0	0	0	0	0	0	0	0	0	0	0	8	0	5
7	0	38	0	14	0	0	0	0	0	0	0	0	0	14	0	0	0	0
0	0	12	0	8	0	0	10	0	0	0	0	0	17	0	0	0	0	7
0	31	0	0	14	0	15	0	0	0	0	0	0	0	0	0	0	11	0
0	94	7	0	20	0	0	0	0	0	0	0	0	0	0	0	0	23	0
0	0	0	0	0	12	0	0	10	0	0	0	0	0	0	0	7	0	0
0	6	0	0	0	0	0	0	0	0	19	0	0	0	15	0	0	0	0
0	0	0	0	10	0	0	0	0	0	39	0	0	0	0	0	44	22	0
0	29	0	5	3	0	0	0	7	0	0	0	0	0	0	0	0	0	0
0	0	0	0	0	0	0	0	0	0	0	0	0	0	0	0	0	6	0
0	0	36	0	0	0	0	0	0	0	0	0	0	0	0	0	0	0	0
0	0	0	0	0	0	0	0	0	0	6	0	0	0	0	0	0	0	0
0	3	0	0	0	0	0	0	0	0	0	0	0	0	0	0	0	0	0
0	0	0	0	0	0	0	0	0	0	0	0	0	0	0	0	0	25	0
0	0	0	0	0	0	0	0	0	0	0	0	0	0	0	0	15	0	0
0	0	0	0	0	0	0	0	0	0	0	0	14	8	0	0	0	0	10
0	0	0	0	0	0	0	0	0	0	0	0	0	0	0	0	0	0	0
0	0	0	0	0	10	0	0	0	0	0	0	0	0	0	0	0	0	0
0	0	0	0	0	0	0	0	0	0	0	0	0	0	0	0	9	0	0
0	0	0	0	11	0	0	0	0	0	0	0	0	0	0	0	0	0	0
0	6	0	0	0	0	14	0	0	0	7	0	0	0	0	0	0	0	0
0	0	0	0	0	0	0	0	0	0	0	0	0	0	6	0	0	0	0
0	10	0	0	0	0	0	0	0	0	0	0	0	0	0	0	0	0	0
0	0	0	0	0	0	0	0	0	0	0	0	0	0	0	0	0	0	0
0	0	0	0	29	0	0	0	0	0	0	0	0	0	0	0	0	0	0
0	0	0	0	0	0	0	0	0	0	0	0	0	0	0	0	0	0	0
0	0	0	0	0	0	0	0	7	0	0	0	0	0	0	0	0	0	0
0	0	0	0	6	0	0	0	0	0	0	0	0	0	0	0	0	0	0

0	0	0	0	0	0	0	0	0	0	0	0	0	0	0	0	0	0	0
0	0	0	0	0	0	0	21	0	0	0	0	0	0	9	0	0	0	0
0	0	0	0	0	0	0	0	0	0	0	0	0	0	0	0	0	0	0
0	0	0	0	0	0	0	0	0	0	0	6	0	0	0	0	0	0	0
0	0	0	0	0	0	0	0	0	0	0	0	0	0	0	0	0	0	0
0	0	0	0	0	0	0	0	0	0	0	0	0	0	0	0	0	0	0
0	0	0	0	0	0	0	0	0	0	0	0	0	0	0	0	0	0	0
0	0	0	0	0	0	0	0	0	0	0	0	0	0	0	0	0	0	0
0	0	0	0	0	0	0	0	0	0	0	0	0	0	0	0	0	0	0
24	0	0	0	0	0	0	0	0	0	0	0	0	0	0	0	0	0	0
0	0	0	0	0	0	0	0	0	0	0	0	0	0	0	0	0	0	0
0	0	0	0	0	0	0	0	0	0	0	0	0	0	0	0	0	0	0
0	0	0	0	0	0	0	0	0	0	0	0	0	0	0	0	0	0	0
0	0	0	0	0	0	0	0	0	0	0	0	0	0	0	0	0	0	0
0	0	0	0	0	0	0	0	0	0	0	0	0	0	0	0	0	0	0
0	0	0	0	0	0	0	0	0	0	0	0	0	0	0	0	0	0	0
0	0	0	0	0	0	0	0	0	0	0	0	0	0	0	0	0	0	0
0	0	15	0	0	0	0	0	0	0	0	0	0	0	0	0	0	0	0
0	0	0	0	0	0	0	0	0	0	0	0	0	0	0	0	0	0	0
0	0	0	0	0	8	0	0	0	0	0	0	0	0	13	0	0	0	0
0	0	0	0	0	0	0	0	0	0	0	0	0	0	0	0	0	0	0
0	0	0	0	0	0	0	0	0	0	0	0	0	0	0	0	0	0	0
0	0	0	0	0	0	0	0	0	0	0	0	0	0	0	0	0	0	0
0	0	0	0	0	0	0	0	0	0	0	0	0	0	0	0	0	0	0
0	0	0	0	0	11	0	0	0	0	0	0	0	0	0	0	0	0	0
0	0	0	0	0	0	0	0	0	0	0	0	0	0	0	0	0	0	0
0	0	0	0	0	0	0	0	0	0	0	0	0	0	0	0	0	0	0
0	0	0	0	0	0	0	0	0	0	0	0	0	0	0	0	0	0	0
0	0	0	0	0	0	0	0	0	0	0	0	0	0	0	0	0	0	0
0	0	0	0	0	0	0	0	0	0	0	0	0	0	0	0	0	0	0
0	0	0	0	0	0	0	0	0	0	0	0	0	0	0	0	0	0	0
0	0	0	0	0	0	0	0	0	0	0	0	0	0	0	0	0	0	0
0	0	0	0	0	0	0	0	0	0	0	0	0	0	8	8	0	0	0
0	0	0	0	0	0	0	0	0	0	0	0	0	0	0	0	0	0	0
0	0	0	0	0	0	0	0	0	0	0	0	0	0	0	0	0	0	0
0	0	0	0	0	0	0	0	0	0	0	0	0	0	0	0	0	0	0
0	0	0	0	0	0	0	0	0	0	0	0	0	0	0	0	0	0	0

0	0	0	0	0	0	0	0	0	0	0	0	0	0	0	0	0	0	0
0	0	0	0	0	0	0	0	0	0	0	0	0	0	0	0	0	0	0
0	0	0	0	0	0	0	0	0	0	0	0	0	0	0	0	0	0	0
0	0	0	0	0	0	0	0	0	0	0	0	0	0	0	0	0	0	0
0	0	0	0	0	0	0	0	0	0	0	0	0	0	0	0	0	0	0
0	0	0	0	0	0	0	0	0	6	0	0	0	0	0	0	0	0	0
0	0	0	0	0	0	0	0	0	0	0	0	0	0	0	0	0	0	0
0	0	0	0	0	0	0	0	0	0	0	0	0	0	0	0	0	0	0
0	0	0	0	0	0	0	0	0	0	0	0	0	0	0	0	0	0	0
0	0	0	0	0	0	0	0	0	0	0	0	0	0	0	0	0	0	0
0	0	0	0	0	0	0	0	0	0	0	0	0	0	0	0	0	0	0
0	0	0	0	0	0	0	0	0	0	0	0	0	0	0	0	0	0	0
0	0	0	0	0	0	0	0	0	0	0	0	0	0	0	0	0	0	0
0	0	0	0	0	0	0	0	0	0	0	0	0	0	0	0	0	0	0
0	0	0	0	0	0	0	0	0	0	0	0	0	0	0	0	0	0	0
0	0	0	0	0	0	0	0	0	0	0	0	0	0	0	0	0	0	0
0	0	0	0	0	0	0	0	0	0	0	0	0	0	0	0	0	0	0
0	0	0	0	0	0	0	0	0	0	0	0	0	0	0	0	0	0	0
0	0	0	0	0	0	0	0	0	0	0	0	0	0	0	0	0	0	0
0	0	0	0	0	0	0	0	0	0	0	0	0	0	0	0	0	0	0
0	0	0	0	0	0	0	0	0	0	0	0	0	0	0	0	0	0	0
0	0	0	0	0	0	0	0	0	0	0	0	0	0	0	0	0	0	0
0	0	0	0	0	0	0	0	0	0	0	0	0	0	0	0	0	0	0
0	0	0	0	0	0	0	0	4	4	4	0	0	0	0	0	0	0	0
0	0	0	0	0	0	0	0	0	0	0	0	0	0	0	0	0	0	0
0	0	0	0	0	0	0	0	0	0	0	0	0	0	0	0	0	0	0
0	0	0	0	0	0	0	0	0	0	0	0	0	0	0	0	0	0	0
0	0	0	0	0	0	0	0	0	0	0	0	0	0	0	0	0	0	0
0	0	0	0	0	0	0	0	0	0	0	0	0	0	0	0	0	0	0
0	0	0	0	0	0	0	0	0	0	0	0	0	0	0	0	0	0	0
0	0	0	0	0	0	0	0	0	0	0	0	0	0	0	0	0	0	0
0	0	0	0	0	0	0	0	0	0	0	0	0	0	0	0	0	0	0
0	0	0	0	0	0	0	0	0	0	0	0	0	0	0	0	0	0	0
0	0	0	0	0	0	0	0	0	0	0	0	0	0	0	0	0	0	0
0	0	0	0	0	0	0	0	0	0	0	0	0	0	0	0	0	0	0
0	0	0	0	0	0	0	0	0	0	0	0	0	0	0	0	0	0	0
0	0	0	0	0	0	0	0	0	0	0	3	3	0	0	0	0	0	0

0	0	0	0	0	0	0	0	0	0	0	0	0	0	0	0	0	0	0
0	0	0	0	0	0	0	0	0	0	0	0	0	0	0	0	0	0	0
0	0	0	0	0	0	0	0	0	0	0	0	0	0	0	0	0	0	0
0	0	0	0	0	0	0	0	0	0	0	0	0	0	0	0	0	0	0
0	0	0	0	0	0	0	0	0	0	0	0	0	0	0	0	0	0	0
0	0	0	0	0	0	0	0	0	0	0	0	0	0	0	0	0	0	0
0	0	0	0	0	0	0	0	0	0	0	0	0	0	0	0	0	0	0
0	0	0	0	0	0	0	0	0	0	0	0	0	0	0	0	0	0	0
0	0	0	0	0	0	0	0	0	0	0	0	0	0	0	0	0	0	0
0	0	0	0	0	0	0	0	0	0	0	0	0	0	0	0	0	0	0
0	0	0	0	0	0	0	0	0	0	0	0	0	0	0	0	0	0	0
0	0	0	0	0	0	0	0	0	0	0	0	0	0	0	0	0	0	0
0	0	0	0	0	0	0	0	0	0	0	0	0	0	0	0	0	0	0
0	0	0	0	0	0	0	0	0	0	0	0	0	0	0	0	0	0	0
0	0	0	0	0	0	0	0	0	0	0	0	0	0	0	0	0	0	0
0	0	0	0	0	0	0	0	0	0	0	0	0	0	0	0	0	0	0
0	0	0	0	0	0	0	0	0	0	0	0	0	0	0	0	0	0	0
0	0	0	0	0	0	0	0	0	0	0	0	0	0	0	0	0	0	0
0	0	0	0	0	0	0	0	0	0	0	0	0	0	0	0	0	0	0
0	0	0	0	0	0	0	0	0	0	0	0	0	0	0	0	0	0	0
0	0	0	0	0	0	0	0	0	0	0	0	0	0	0	0	0	0	0
0	0	0	0	0	0	0	0	0	0	0	0	0	0	0	0	0	0	0
0	0	0	0	0	0	0	0	0	0	0	0	0	0	0	0	0	0	0
0	0	0	0	0	0	0	0	0	0	0	0	0	0	0	0	0	0	0
0	0	0	0	0	0	0	0	0	0	0	0	0	0	0	0	0	0	0
0	0	0	0	0	2	2	2	2	2	2	0	0	0	0	0	0	0	0
0	0	0	0	0	0	0	0	0	0	0	0	0	0	0	0	0	0	0
0	0	0	0	0	0	0	0	0	0	0	0	0	0	0	0	0	0	0
0	0	0	0	0	0	0	0	0	0	0	0	0	0	0	0	0	0	0
0	0	0	0	0	0	0	0	0	0	0	0	0	0	0	0	0	0	0
0	0	0	0	0	0	0	0	0	0	0	0	0	0	0	0	0	0	0
0	0	0	0	0	0	0	0	0	0	0	0	0	0	0	0	0	0	0
0	0	0	0	0	0	0	0	0	0	0	0	0	0	0	0	0	0	0
0	0	0	0	0	0	0	0	0	0	0	0	0	0	0	0	0	0	0
0	0	0	0	0	0	0	0	0	0	0	0	0	0	0	0	0	0	0
0	0	0	0	0	0	0	0	0	0	0	0	0	0	0	0	0	0	0
0	0	0	0	0	0	0	0	0	0	0	0	0	0	0	0	0	0	0

0	0	0	0	0	0	0	0	0	0	0	0	0	0	0	0	0	0	0
0	0	0	0	0	0	0	0	0	0	0	0	0	0	0	0	0	0	0
0	0	0	0	0	0	0	0	0	0	0	0	0	0	0	0	0	0	0
0	0	0	0	0	0	0	0	0	0	0	0	0	0	0	0	0	0	0
0	0	0	0	0	0	0	0	0	0	0	0	0	0	0	0	0	0	0
0	0	0	0	0	0	0	0	0	0	0	0	0	0	0	0	0	0	0
0	0	0	0	0	0	0	0	0	0	0	0	0	0	0	0	0	0	0
0	0	0	0	0	0	0	0	0	0	0	0	0	0	0	0	0	0	0
0	0	0	0	0	0	0	0	0	0	0	0	0	0	0	0	0	0	0
0	0	0	0	0	0	0	0	0	0	0	0	0	0	0	0	0	0	0
0	0	0	0	0	0	0	0	0	0	0	0	0	0	0	0	0	0	0
0	0	0	0	0	0	0	0	0	0	0	0	0	0	0	0	0	0	0
0	0	0	0	0	0	0	0	0	0	0	0	0	0	0	0	0	0	0
0	0	0	0	0	0	0	0	0	0	0	0	0	0	0	0	0	0	0
0	0	0	0	0	0	0	0	0	0	0	0	0	0	0	0	0	0	0
0	0	0	0	0	0	0	0	0	0	0	0	0	0	0	0	0	0	0
0	0	0	0	0	0	0	0	0	0	0	0	0	0	0	0	0	0	0
0	0	0	0	0	0	0	0	0	0	0	0	0	0	0	0	0	0	0
0	0	0	0	0	0	0	0	0	0	0	0	0	0	0	1	0	0	0
0	0	0	0	0	0	0	0	0	0	0	0	0	0	0	0	0	0	0
0	0	0	0	0	0	0	0	0	0									
Jan_DL116	1014	1163	765	657	95	186	193	183	270	173	186	67	117	173	172	56	80	193
191	705	124	283	140	125	131	90	97	79	95	0	105	62	82	95	95	33	0
59	134	0	50	70	80	98	0	37	12	53	100	166	64	116	91	0	56	0
0	270	74	63	62	403	55	58	36	58	111	0	0	0	28	147	0	96	0
0	0	51	32	8	22	12	50	0	0	49	0	9	87	0	0	52	0	104
34	39	54	0	16	11	0	28	27	29	0	0	0	0	17	19	20	59	0
0	8	0	6	5	18	0	0	15	0	9	0	0	0	0	46	0	0	0
0	0	0	0	18	0	0	0	0	12	0	0	5	0	24	18	0	0	0
6	0	15	0	0	10	0	0	0	0	6	0	0	17	0	0	0	0	10
13	0	11	18	0	90	0	0	0	0	0	0	0	16	0	0	22	0	32
33	0	0	0	0	0	0	0	55	0	0	48	0	0	16	0	0	0	0
0	0	19	0	0	0	8	0	0	34	0	0	0	15	0	0	0	3	0
0	37	0	0	0	0	0	0	0	0	0	21	0	12	10	11	0	0	13
0	0	0	2	0	0	0	0	0	0	0	0	0	0	5	4	26	12	0
0	0	0	0	0	0	0	0	0	0	0	0	0	9	3	6	0	0	0
0	0	0	0	0	0	0	0	0	0	0	16	8	0	0	0	0	0	0

0	0	5	0	0	8	0	0	0	0	0	15	8	8	0	0	0	0	9
4	0	0	0	0	0	0	10	0	0	0	0	0	0	0	0	19	0	18
0	0	0	11	0	0	0	0	0	0	0	0	0	0	12	0	0	0	0
0	0	7	0	0	0	4	0	0	0	0	0	0	0	0	0	0	0	0
0	0	0	0	0	0	17	0	0	0	0	0	5	0	0	7	0	0	0
0	0	0	0	0	0	10	6	0	0	0	5	0	0	0	0	0	30	0
0	0	0	0	0	0	0	0	0	0	0	0	0	0	10	0	0	0	0
0	8	0	0	0	0	0	0	0	0	0	0	0	0	0	0	0	0	0
0	0	0	0	0	0	0	11	0	0	0	0	0	0	0	0	0	0	0
46	0	0	0	4	0	0	0	0	0	0	0	0	0	0	0	0	0	0
0	0	5	0	0	0	0	0	0	0	0	0	0	0	0	0	6	0	0
0	0	0	0	0	0	0	0	0	0	0	0	0	0	0	0	0	0	0
10	0	0	0	0	0	0	0	0	0	0	0	0	0	0	0	0	0	0
0	0	0	0	0	0	0	0	0	0	0	0	0	0	0	0	0	0	0
0	0	0	2	0	0	0	0	0	0	0	0	0	0	0	0	0	0	0
0	0	0	0	0	0	0	0	0	0	0	0	0	0	0	0	21	0	0
0	0	0	0	0	0	0	0	0	0	0	0	0	0	0	0	0	0	0
0	0	24	0	0	0	0	7	0	0	0	0	0	0	0	0	0	0	0
0	0	0	0	0	0	0	0	0	0	0	0	0	0	0	0	0	0	0
0	0	0	0	0	0	0	0	0	0	0	9	0	0	0	0	0	0	0
0	0	0	0	0	0	0	0	0	0	0	0	0	0	0	0	0	0	0
0	0	0	0	0	0	0	0	6	0	0	0	0	0	0	0	0	0	0
0	0	0	0	0	0	0	0	0	0	0	0	0	0	0	0	0	0	0
0	6	0	0	0	0	0	0	0	0	0	0	0	0	0	0	5	0	0
0	0	0	0	0	0	0	0	0	4	0	0	0	0	0	0	0	0	0
0	0	0	0	0	0	0	0	0	0	0	0	0	0	0	0	0	0	0
0	0	0	0	0	0	0	0	0	0	0	0	0	0	0	0	0	0	0
0	0	0	0	0	0	0	0	0	0	0	0	0	0	0	0	0	0	0
6	0	0	0	0	33	0	0	0	0	0	0	0	0	0	0	0	0	0
0	0	0	0	0	0	0	0	0	0	0	0	0	0	0	0	0	0	0
0	0	0	0	0	0	0	0	0	0	0	0	0	0	0	0	0	0	0
0	0	0	0	0	0	0	0	0	0	0	0	0	0	0	0	0	0	0
0	0	0	0	0	0	0	0	0	0	6	0	0	0	0	0	0	0	0
0	0	0	0	0	0	0	0	0	0	0	0	0	0	6	0	0	0	0
0	0	0	0	0	0	0	0	0	0	0	0	0	0	0	0	0	0	0
0	0	15	0	0	0	0	6	0	0	0	0	0	0	0	0	0	0	0
0	0	0	0	0	0	0	0	0	0	0	0	5	0	0	0	0	0	0

0	0	0	0	0	0	0	0	0	0	0	0	0	0	0	24	0	0	0
0	0	0	0	0	0	0	0	0	0	0	0	0	0	0	0	0	0	0
0	8	0	0	0	0	0	0	0	0	0	0	0	0	0	0	0	0	0
0	0	0	0	0	0	0	0	0	0	0	0	0	0	0	0	0	0	0
0	0	0	0	0	0	0	0	0	0	0	0	0	0	0	0	0	0	0
0	0	0	0	0	0	0	0	0	0	0	0	0	0	0	0	0	0	0
0	0	0	0	0	0	0	0	0	0	0	0	0	0	0	0	0	0	0
0	0	0	0	0	0	0	0	0	0	0	0	0	0	0	0	0	0	0
0	0	0	0	0	0	0	0	0	0	0	0	0	0	0	0	0	0	0
0	0	0	7	0	0	0	0	0	0	0	0	0	0	0	0	0	0	0
0	0	0	0	0	0	0	0	0	0	0	0	0	0	0	0	0	0	0
0	0	0	0	0	0	0	0	0	0	0	0	0	0	0	0	0	0	0
0	0	0	0	0	0	0	0	0	0	0	0	0	0	0	0	0	0	0
0	0	0	0	0	0	0	0	0	0	0	0	0	0	0	0	0	12	0
0	0	0	0	0	0	0	0	0	0	0	0	0	0	0	0	0	0	0
0	0	0	0	0	0	0	0	0	0	0	0	0	0	0	0	0	0	0
0	11	0	0	0	0	0	0	0	0	0	0	0	0	0	0	0	0	0
0	0	0	0	0	0	0	0	0	0	0	0	0	0	0	0	0	0	0
0	0	0	0	0	0	0	0	10	10	0	0	0	0	0	0	0	0	0
0	0	0	0	0	0	0	0	0	0	0	0	0	0	0	0	0	0	0
0	0	0	0	0	0	0	0	0	0	0	0	0	0	0	0	0	0	0
0	0	0	0	0	0	0	0	0	0	0	0	0	0	0	0	0	0	0
0	0	0	0	0	0	0	0	0	0	0	0	0	0	0	0	0	0	0
0	0	0	0	0	0	0	0	0	0	0	0	0	0	0	0	0	0	0
0	0	0	0	0	0	0	0	0	0	0	0	0	0	0	0	0	0	0
0	0	0	0	0	0	0	0	0	0	0	0	0	0	0	0	0	0	0
0	0	0	0	0	0	0	0	0	0	0	0	0	0	0	0	0	0	0
0	0	0	0	0	0	0	0	0	0	0	0	0	0	0	0	0	0	0
0	0	0	0	0	0	0	7	7	0	0	0	0	0	0	0	0	0	0
0	0	0	0	0	0	0	0	0	0	0	0	0	0	0	0	0	0	0
0	0	0	0	0	0	0	0	0	0	0	0	0	0	0	0	0	0	0
0	0	0	0	0	0	0	0	0	0	0	0	0	0	0	0	0	0	0
0	0	0	0	0	0	0	0	0	0	0	0	0	0	0	0	0	0	0
0	0	0	0	0	0	0	0	0	0	0	0	0	0	0	0	0	0	0
0	0	0	0	0	0	0	0	0	0	0	0	0	0	0	0	0	0	0
0	0	0	0	0	0	0	0	0	0	0	0	0	0	0	0	0	0	0
0	0	0	0	0	0	0	0	0	0	0	0	0	0	0	0	0	0	0

0	0	0	0	0	0	0	0	0	0	0	0	0	0	0	0	0	0	0
0	0	0	0	0	0	0	0	0	0	0	0	0	0	0	0	0	0	0
0	0	0	0	0	0	0	0	0	0	0	0	0	0	0	0	0	0	0
0	0	0	0	0	0	0	0	0	0	0	0	0	0	0	0	0	0	0
0	0	0	0	0	0	0	0	0	0	0	5	0	0	0	0	0	0	0
0	0	0	0	0	0	0	0	0	0	0	0	0	0	0	0	0	0	0
0	0	0	0	0	0	0	0	0	0	0	0	0	0	0	0	0	0	0
0	0	0	0	0	0	0	0	0	0	0	0	0	0	0	0	0	0	0
0	0	0	0	0	0	0	0	0	0	0	0	0	0	0	0	0	0	0
0	0	0	0	0	0	0	0	0	0	0	0	0	0	0	0	0	0	0
0	0	0	0	0	0	0	0	0	0	0	0	0	0	0	0	0	0	0
0	0	0	0	0	0	0	0	0	0	0	0	0	0	0	0	0	0	0
0	0	0	0	0	0	0	0	0	0	0	0	0	0	0	0	0	0	0
0	0	0	0	0	0	0	0	0	0	0	0	0	0	0	0	0	0	0
0	0	0	0	0	0	0	0	0	0	0	0	0	0	0	0	0	0	0
0	0	4	0	0	0	0	0	0	0	0	0	0	0	0	0	0	0	0
0	0	0	0	0	0	0	0	0	0	0	0	0	0	0	0	0	0	0
0	0	0	0	0	0	0	0	0	0	0	0	0	0	0	0	0	0	0
0	0	0	0	0	0	0	0	0	0	0	0	0	0	0	0	0	0	0
0	0	0	0	0	0	0	0	0	0	0	0	0	0	0	0	0	0	0
0	0	0	0	0	0	0	0	0	0	0	0	0	0	0	0	0	0	0
0	0	0	0	0	0	0	0	0	0	0	0	0	0	0	0	0	0	0
0	0	0	0	0	0	0	0	0	0	0	0	0	0	0	0	0	0	0
0	0	0	0	0	0	0	0	0	0	0	0	0	0	0	0	0	0	0
0	0	0	0	0	0	0	0	0	0	0	0	0	0	0	0	0	0	0
0	0	0	0	0	0	0	0	0	0	0	0	0	0	0	0	0	0	0
0	0	0	0	0	0	0	0	0	0	0	0	0	0	0	0	0	0	0
0	0	0	0	0	0	0	0	0	0	0	0	0	0	0	3	3	3	3
3	0	0	0	0	0	0	0	0	0	0	0	0	0	0	0	0	0	0
0	0	0	0	0	0	0	0	0	0	0	0	0	0	0	0	0	0	0
0	0	0	0	0	0	0	0	0	0	0	0	0	0	0	0	0	0	0
0	0	0	0	0	0	0	0	0	0	0	0	0	0	0	0	0	0	0
0	0	0	0	0	0	0	0	0	0	0	0	0	0	0	0	0	0	0
0	0	0	0	0	0	0	0	0	0	0	0	0	0	0	0	0	0	0
0	0	0	0	0	0	0	0	0	0	0	0	0	0	0	0	0	0	0
0	0	0	0	0	0	0	0	0	0	0	0	0	0	0	0	0	0	0
0	0	0	0	0	0	0	0	0	0	0	0	0	0	0	0	0	0	0

0	0	0	0	0	0	0	0	0	0	0	0	0	0	0	0	0	0	0
0	0	0	0	0	0	0	0	0	0	0	0	0	0	0	0	0	0	0
0	0	0	0	0	0	0	0	0	0	0	0	0	0	0	0	0	0	0
0	0	0	0	0	0	0	0	0	0	0	0	0	0	0	0	0	0	0
0	0	0	0	0	0	0	0	0	0	0	0	0	0	0	0	0	0	0
0	0	0	0	0	0	0	0	0	0	0	0	0	0	0	0	0	0	0
0	0	0	0	0	0	0	0	0	0	0	0	0	0	0	0	0	0	0
0	0	0	0	0	0	0	0	0	0	0	0	0	0	0	0	0	0	0
0	0	0	0	0	0	0	0	0	0	0	0	0	0	0	0	0	0	0
0	0	0	0	0	0	0	0	0	0	0	0	0	0	0	0	0	0	0
0	0	0	0	0	0	0	0	0	0	0	0	0	0	0	0	0	0	0
0	0	0	0	0	0	0	0	0	0	0	0	0	0	0	0	0	0	0
0	0	0	0	0	0	0	0	0	0	0	0	0	0	0	0	0	0	0
0	0	0	0	0	0	0	0	0	0	0	0	0	0	0	0	0	0	0
0	0	0	0	0	0	0	0	0	0	0	0	0	0	0	0	0	0	0
0	0	0	0	0	0	0	0	0	0	0	0	0	0	0	0	0	0	0
0	0	0	0	0	0	0	0	0	0	0	0	0	0	0	0	0	2	2
2	2	2	2	2	0	0	0	0	0	0	0	0	0	0	0	0	0	0
0	0	0	0	0	0	0	0	0	0	0	0	0	0	0	0	0	0	0
0	0	0	0	0	0	0	0	0	0	0	0	0	0	0	0	0	0	0
0	0	0	0	0	0	0	0	0	0	0	0	0	0	0	0	0	0	0
0	0	0	0	0	0	0	0	0	0	0	0	0	0	0	0	0	0	0
0	0	0	0	0	0	0	0	0	0	0	0	0	0	0	0	0	0	0
0	0	0	0	0	0	0	0	0	0	0	0	0	0	0	0	0	0	0
0	0	0	0	0	0	0	0	0	0	0	0	0	0	0	0	0	0	0
0	0	0	0	0	0	0	0	0	0	0	0	0	0	0	0	0	0	0
0	0	0	0	0	0	0	0	0	0	0	0	0	0	0	0	0	0	0
0	0	0	0	0	0	0	0	0	0	0	0	0	0	0	0	0	0	0
0	0	0	0	0	0	0	0	0	0	0	0	0	0	0	0	0	0	0
0	0	0	0	0	0	0	0	0	0	0	0	0	0	0	0	0	0	0
0	0	0	0	0	0	0	0	0	0	0	0	0	0	0	0	0	0	0
0	0	0	0	0	0	0	0	0	0	0	0	0	0	0	0	0	0	0
0	0	0	0	0	0	0	0	0	0	0	0	0	0	0	0	0	0	0
0	0	0	0	0	0	0	0	0	0	0	0	0	0	0	0	0	0	0
0	0	0	0	0	0	0	0	0	0	0	0	0	0	0	0	0	0	0
0	0	0	0	0	0	0	0	0	0	0	0	0	0	0	0	0	0	0
0	0	0	0	0	0	0	0	0	0	0	0	0	0	0	0	0	0	0

0	0	0	0	0	0	0	0	0	0	0	0	0	0	0	0	0	0	0
0	0	0	0	0	0	0	0	0	0	0	0	0	0	0	0	0	0	0
0	0	0	0	0	0	0	0	0	0	0	0	0	0	0	0	0	0	0
0	0	0	0	0	0	0	0	0	0	0	0	0	0	0	0	0	0	0
0	0	0	0	0	0	0	0	0	0	0	0	0	0	0	0	0	0	0
0	0	0	0	0	0	0	0	0	0	0	0	0	0	0	0	0	0	0
0	0	0	0	0	0	0	0	0	0	0	0	0	0	0	0	0	0	0
0	0	0	0	0	0	0	0	0	0	0	0	0	0	0	0	0	0	0
0	0	0	0	0	0	0	0	0	0	0	0	0	0	0	0	0	0	0
0	0	0	0	0	0	0	0	0	0	0	0	0	0	0	0	0	0	0
0	0	0	0	0	0	0	0	0	0	0	0	0	0	0	0	0	0	0
0	0	0	0	0	0	0	0	0	0	0	0	0	0	0	0	0	0	0
0	0	0	0	0	0	0	0	0	0									
Jan_DL117	1885	754	1041	574	28	76	59	61	139	330	114	353	323	82	43	421	72	80
97	175	143	339	158	117	224	124	194	154	31	0	76	125	143	59	105	695	0
173	151	0	0	84	50	93	391	24	157	0	162	51	0	32	127	63	0	0
0	58	139	9	0	126	0	104	125	101	18	0	39	0	36	0	31	122	161
83	0	0	14	0	60	35	31	0	79	0	0	12	26	25	0	38	15	120
12	78	0	0	79	13	115	14	0	50	0	0	0	37	16	0	0	160	0
0	14	35	31	9	0	0	0	0	0	17	49	7	3	62	0	0	0	0
0	0	0	0	0	50	0	0	0	12	0	0	10	0	19	0	0	0	0
187	0	30	14	0	5	0	0	242	0	0	0	0	97	0	0	0	0	0
0	0	5	0	0	0	0	0	0	0	0	38	0	0	0	6	10	0	0
0	0	18	43	0	9	0	0	0	0	0	0	0	0	17	0	0	0	0
0	0	0	0	0	0	0	0	0	10	0	0	0	0	0	0	0	20	31
0	14	0	18	0	4	0	27	97	59	0	7	5	13	9	0	0	0	0
14	0	0	27	0	0	0	0	0	14	0	0	8	0	0	2	0	0	0
11	0	0	0	0	0	12	0	12	0	8	0	0	0	12	0	0	0	0
0	0	19	0	0	0	0	16	8	0	39	0	0	37	0	9	17	0	0
0	0	23	36	0	0	0	0	0	0	0	0	0	0	0	0	0	0	13
0	0	0	21	25	0	14	12	0	0	0	0	0	15	0	0	0	60	0
0	0	53	0	17	0	0	0	0	0	0	22	27	20	0	0	11	0	0
0	13	7	0	13	0	0	61	0	0	0	0	0	0	0	0	0	0	0
35	0	24	0	0	0	0	0	0	0	0	0	6	0	6	0	0	0	0
0	0	0	0	0	0	0	0	0	0	0	0	0	0	0	0	4	0	0
0	0	0	0	0	0	0	0	3	0	0	0	0	0	0	0	0	0	0
0	12	0	0	0	0	0	4	0	0	0	0	0	0	0	0	0	0	0

0	0	0	0	6	0	0	0	0	0	39	19	0	0	0	0	0	0	0
0	0	0	0	0	0	15	3	0	0	0	19	0	0	0	0	0	32	0
0	0	0	0	0	0	0	0	0	10	0	0	0	0	0	0	0	5	0
0	0	0	0	0	0	0	0	0	0	65	0	0	0	0	18	13	0	0
0	0	0	0	0	5	32	0	0	0	28	0	0	0	0	0	0	0	0
0	9	0	0	0	0	0	0	0	0	0	16	0	0	0	0	0	0	0
0	0	0	0	0	0	0	0	0	0	0	0	11	0	0	0	0	0	0
0	0	0	0	0	0	0	0	0	18	0	0	0	0	0	0	0	0	0
0	0	25	0	0	0	0	0	0	0	0	0	48	0	0	0	0	0	0
0	0	0	0	0	0	0	0	0	0	0	0	0	0	0	0	0	0	0
0	0	0	0	0	0	0	0	0	0	0	0	0	0	0	0	0	0	0
0	0	0	0	0	0	0	28	0	0	0	0	0	0	0	0	0	0	0
0	0	0	0	0	0	0	0	0	0	0	0	0	0	0	0	0	0	0
0	0	0	0	0	0	0	0	0	0	0	0	0	0	0	0	0	0	0
0	0	8	0	0	0	0	0	0	0	0	0	0	0	0	0	0	0	0
0	0	0	0	0	0	0	0	0	0	0	0	0	0	0	0	0	0	0
0	14	0	0	0	0	0	5	0	0	0	0	0	0	0	0	0	0	8
0	0	0	0	0	0	0	0	0	0	0	8	0	0	0	0	0	6	0
0	0	0	0	0	0	0	0	0	0	0	0	0	0	0	0	0	0	0
0	0	0	0	0	0	0	0	0	0	0	0	0	0	0	0	0	0	0
26	0	0	5	7	0	0	0	0	0	0	0	0	0	0	0	0	0	0
0	0	0	0	0	0	0	0	0	0	0	0	0	0	0	0	0	5	0
0	0	0	0	0	0	0	7	0	0	0	0	0	0	0	0	0	0	0
0	0	0	0	0	0	0	0	0	0	0	0	0	0	0	0	0	0	0
0	0	0	0	0	0	0	0	0	0	6	0	0	0	0	0	0	0	0
0	0	0	0	0	0	19	0	0	0	0	0	0	0	0	0	0	0	0
0	0	0	0	0	0	0	0	0	0	0	12	0	0	0	0	0	0	0
0	0	0	0	0	0	0	0	0	0	8	0	0	0	25	0	0	0	0
0	0	0	0	0	0	0	0	0	0	0	0	0	0	0	0	0	0	0
0	0	0	0	0	0	0	0	0	0	0	0	0	0	0	0	0	0	0
0	0	0	0	0	0	12	0	0	0	0	0	0	0	0	28	0	0	0
0	0	0	0	0	0	0	0	0	0	0	0	0	0	0	0	4	0	0
0	0	0	0	0	0	0	0	0	0	0	0	0	0	0	0	0	0	0
0	0	0	0	0	0	0	0	0	0	0	0	0	0	0	0	0	0	0
0	0	0	0	0	0	0	0	0	0	0	0	0	0	0	0	0	0	0
0	0	0	0	0	0	0	0	0	0	0	0	0	0	0	0	0	0	0
0	0	0	0	0	0	0	0	0	0	0	0	0	0	0	0	0	0	0

0	0	0	0	0	0	0	0	0	0	0	0	0	0	0	0	0	0	0
0	0	0	0	0	0	0	18	0	0	0	0	0	0	0	0	0	0	0
0	0	0	0	0	0	0	0	0	0	0	0	0	0	0	0	0	0	0
0	0	0	0	0	0	0	0	0	0	0	0	8	0	0	0	0	0	0
0	0	0	0	0	0	0	0	0	0	0	0	0	0	0	0	0	0	0
0	0	0	0	0	0	0	0	0	0	0	0	0	0	0	0	0	0	0
0	0	0	0	0	0	0	0	0	0	0	0	0	0	0	0	0	0	0
0	0	0	0	0	0	0	0	0	0	0	0	0	0	0	0	0	0	0
0	0	0	0	0	0	0	0	0	0	0	0	0	0	13	0	0	0	0
0	0	0	0	0	0	0	0	0	0	0	0	0	0	0	0	0	0	0
0	0	0	0	0	0	0	0	0	0	0	0	0	0	0	0	0	0	0
0	0	0	0	0	0	0	0	0	12	0	0	0	0	0	0	0	0	0
0	0	0	0	0	0	0	0	0	0	0	0	0	0	0	0	0	0	0
0	0	0	0	0	0	0	0	0	0	0	0	0	0	0	0	0	0	0
0	0	0	0	0	0	0	0	0	0	0	0	0	0	0	0	0	0	0
0	0	0	0	0	0	0	0	10	0	0	0	0	0	0	0	0	0	0
0	0	0	0	0	0	0	0	0	0	0	0	0	0	0	0	0	0	0
0	0	0	0	0	0	0	0	0	0	0	0	0	0	0	0	0	0	0
0	0	0	0	0	0	0	0	0	0	0	0	0	0	0	9	0	0	0
0	0	0	0	0	0	0	0	0	0	0	0	0	0	0	0	0	0	0
0	0	0	0	0	0	0	0	0	0	0	0	0	0	0	0	0	0	0
0	0	0	0	0	0	0	0	0	0	0	0	0	0	0	0	0	0	0
0	0	0	0	0	0	0	0	0	0	0	0	0	0	0	0	0	0	0
0	0	0	0	0	0	0	0	0	0	0	0	0	0	0	0	0	0	0
0	0	0	0	0	0	0	0	0	0	0	0	0	0	0	0	0	0	0
0	0	0	0	0	0	0	0	0	0	0	0	0	0	0	0	0	0	0
0	0	0	0	0	0	0	0	0	0	0	0	0	0	0	0	0	0	0
0	0	0	0	0	0	0	0	0	0	0	0	0	0	4	0	7	7	0
0	0	0	0	0	0	0	0	0	0	0	0	0	0	0	0	0	0	0
0	0	0	0	0	0	0	0	0	0	0	0	0	0	0	0	0	0	0
0	0	0	0	0	0	0	0	0	0	0	0	0	0	0	0	0	0	0
0	0	0	0	0	0	0	0	0	0	0	0	0	0	0	0	0	0	0
0	0	0	0	0	0	0	0	0	0	0	0	0	0	0	0	0	0	0
0	0	0	0	0	0	0	0	0	0	0	0	0	0	0	0	0	0	0
0	0	0	0	0	0	0	0	0	0	0	0	6	0	0	0	0	0	0
0	0	0	0	0	0	0	0	0	0	0	0	0	0	0	0	0	0	0
0	0	0	0	0	0	0	0	0	0	0	0	0	0	0	0	0	0	0

0	0	0	0	0	0	0	0	0	0	0	0	0	0	0	0	0	0	0
0	0	0	0	0	0	0	0	0	0	0	0	0	0	0	0	0	0	0
0	0	0	0	0	0	0	0	0	0	0	0	0	0	0	0	0	0	0
0	0	0	0	0	0	0	0	0	0	0	0	0	0	0	0	0	0	0
0	0	0	0	0	0	0	0	0	0	0	0	0	0	0	0	0	0	0
0	0	0	0	0	0	0	0	0	0	0	0	0	0	0	0	0	0	0
0	0	0	0	5	0	0	0	0	0	0	0	0	0	0	0	0	0	0
0	0	0	0	0	0	0	0	0	0	0	0	0	0	0	0	0	0	0
0	0	0	0	0	0	0	0	0	0	0	0	0	0	0	0	0	0	0
0	0	0	0	0	0	0	0	0	0	0	0	0	0	0	0	0	0	0
0	0	0	0	0	0	0	0	0	0	0	0	0	0	0	0	0	0	0
0	0	0	0	0	0	0	0	0	0	0	0	0	0	0	0	0	0	0
0	0	0	0	0	0	0	0	0	0	0	0	0	0	0	0	0	0	0
0	0	0	0	0	0	0	0	0	0	0	0	0	0	0	0	0	0	0
0	0	0	0	0	0	0	0	0	0	0	0	0	0	0	0	0	0	0
0	0	0	0	0	0	0	0	0	0	0	0	0	0	0	0	0	0	0
0	0	0	0	0	0	0	0	0	0	0	0	4	4	0	0	0	0	0
0	0	0	0	0	0	0	0	0	0	0	0	0	0	0	0	0	0	0
0	0	0	0	0	0	0	0	0	0	0	0	0	0	0	0	0	0	0
0	0	0	0	0	0	0	0	0	0	0	0	0	0	0	0	0	0	0
0	0	0	0	0	0	0	0	0	0	0	0	0	0	0	0	0	0	0
0	0	0	0	0	0	0	0	0	0	0	0	0	0	0	0	0	0	0
0	0	0	0	0	0	0	0	0	0	0	0	0	0	0	0	0	0	0
0	0	0	0	0	0	0	0	0	0	0	0	0	0	0	0	0	0	0
0	0	0	0	0	0	0	0	0	0	0	0	0	0	0	0	0	0	0
0	0	0	0	0	0	0	0	0	0	0	0	0	0	0	0	0	0	0
0	0	0	0	0	0	0	0	0	0	0	0	0	0	0	0	0	0	0
0	0	0	0	0	0	0	0	0	0	0	0	0	0	0	0	0	0	0
0	0	0	0	0	0	0	0	0	0	0	0	0	0	0	0	0	0	0
0	0	0	0	0	0	0	0	0	0	0	0	0	0	0	0	0	0	0
0	0	0	0	0	0	0	0	0	0	0	0	0	0	0	0	0	0	0
0	0	0	0	0	0	0	0	0	0	0	0	0	0	0	0	0	0	0
0	0	0	0	0	0	0	0	0	0	0	0	0	0	0	0	0	0	0
3	3	3	3	3	0	0	0	0	0	0	0	0	0	0	0	0	0	0
0	0	0	0	0	0	0	0	0	0	0	0	0	0	0	0	0	0	0
0	0	0	0	0	0	0	0	0	0	0	0	0	0	0	0	0	0	0
0	0	0	0	0	0	0	0	0	0	0	0	0	0	0	0	0	0	0

0	0	0	0	0	0	0	0	0	0	0	0	0	0	0	0	0	0	0
0	0	0	0	0	0	0	0	0	0	0	0	0	0	0	0	0	0	0
0	0	0	0	0	0	0	0	0	0	0	0	0	0	0	0	0	0	0
0	0	0	0	0	0	0	0	0	0	0	0	0	0	0	0	0	0	0
0	0	0	0	0	0	0	0	0	0	0	0	0	0	0	0	0	0	0
0	0	0	0	0	0	0	0	0	0	0	0	0	0	0	0	0	0	0
0	0	0	0	0	0	0	0	0	0	0	0	0	0	0	0	0	0	0
0	0	0	0	0	0	0	0	0	0	0	0	0	0	0	0	0	0	0
0	0	0	0	0	0	0	0	0	0	0	0	0	0	0	0	0	0	0
0	0	0	0	0	0	0	0	0	0	0	0	0	0	0	0	0	0	0
0	0	0	0	0	0	0	0	0	0	0	0	0	0	0	0	0	0	0
0	0	0	0	0	0	0	0	0	0	0	0	0	0	0	0	0	0	0
0	0	0	0	0	0	0	0	0	0	0	0	0	0	0	0	0	0	0
0	0	0	0	0	0	0	0	0	0	0	0	0	0	0	0	0	0	0
0	0	0	0	0	0	0	0	0	0	0	0	0	0	0	0	0	0	0
0	0	0	0	0	0	0	0	0	0	0	0	0	0	0	0	0	0	0
0	0	0	0	0	0	0	0	0	0	0	0	0	0	0	0	0	0	0
0	0	0	0	0	0	0	0	0	0	0	0	0	0	0	0	0	0	0
0	0	0	0	0	0	0	0	0	0	0	0	0	0	0	0	0	0	0
0	0	0	0	0	0	0	0	0	0	0	0	0	0	0	0	0	0	0
0	0	0	0	0	0	0	0	0	0	0	0	0	0	0	0	0	0	0
0	0	0	0	0	0	0	0	0	0	0	0	0	0	0	0	0	0	0
0	0	0	0	0	0	0	0	0	0	0	0	0	0	0	0	0	0	0
0	0	0	0	0	0	0	0	0	0	0	0	0	0	0	0	0	0	0
0	0	0	0	0	0	0	0	0	0	0	0	0	0	0	0	0	0	0
0	0	0	0	0	0	0	0	0	0	0	0	0	0	0	0	0	0	0
0	0	0	0	0	0	0	0	0	0	0	0	0	0	0	0	0	0	0
0	0	0	0	0	0	0	0	0	0	0	0	0	0	0	0	0	0	0
0	0	0	0	0	0	0	0	0	0	0	0	0	0	0	0	0	0	0
0	0	0	0	0	0	0	0	0	0	0	0	0	0	0	0	0	0	0
0	0	0	0	0	0	0	0	0	0	0	0	0	0	0	0	0	0	0
0	0	0	0	0	0	0	0	0	0	0	0	0	0	0	0	0	0	0
0	0	0	0	0	0	0	0	0	0	0	0	0	0	0	0	0	0	0
0	0	0	0	0	0	0	0	0	0	0	0	0	0	0	0	0	0	0
0	0	0	0	0	0	0	0	0	0	0	0	0	0	0	0	0	0	0
0	0	0	0	0	0	0	0	0	0	0	0	0	0	0	0	0	0	0
0	0	0	0	0	0	0	0	0	0	0	0	0	0	0	0	0	0	0

0	0	0	0	0	0	0	0	0	0	0	0	0	0	0	0	0	0	0
0	0	0	0	0	0	0	0	0	0	0	0	0	0	0	0	0	0	0
0	0	0	0	0	0	0	0	0	0	0	0	0	0	0	0	0	0	0
0	0	0	0	0	0	0	0	0	0	0	0	0	0	0	0	0	0	0
0	0	0	0	0	0	0	0	0	0									
Jan_DL118	1582	1334	16	153	404	170	276	247	369	145	68	93	122	119	118	39	116	118
108	165	172	300	106	118	47	62	38	0	56	0	62	75	56	0	30	12	0
0	58	107	118	63	0	40	6	74	19	51	0	50	67	95	19	0	83	0
0	0	0	40	32	151	37	15	15	18	64	74	79	50	49	190	17	77	17
42	0	62	40	81	51	16	61	0	20	56	0	19	23	0	0	21	43	0
0	17	0	0	0	0	0	79	0	9	0	0	0	0	0	0	0	0	58
0	0	0	4	33	11	0	0	5	0	24	29	22	0	0	36	0	0	38
0	0	0	9	27	0	0	0	0	15	57	0	14	0	20	26	0	0	0
0	0	0	17	0	30	0	0	0	0	11	0	12	64	0	7	14	0	0
10	0	13	0	0	0	0	0	0	0	12	0	0	0	0	0	0	9	0
0	0	0	0	0	0	0	0	37	8	0	45	0	57	15	0	0	32	0
23	0	0	0	0	0	14	0	0	31	0	5	0	36	0	0	0	14	0
0	0	0	0	0	0	0	0	4	0	0	0	6	7	0	15	0	0	0
0	0	0	0	0	0	0	0	0	0	0	0	0	0	10	7	20	0	16
0	0	0	0	19	0	0	0	0	0	0	0	0	0	0	8	0	0	0
0	0	0	0	0	9	0	0	4	0	0	17	0	0	0	0	0	0	0
0	0	0	0	0	0	0	0	0	14	0	7	5	0	8	0	0	0	5
0	0	0	0	33	0	0	6	0	0	0	0	0	0	21	17	0	0	0
6	0	0	0	0	0	0	0	0	0	0	6	0	0	0	0	0	0	0
0	0	0	0	0	0	0	0	0	17	0	0	9	0	0	0	0	0	0
0	0	19	0	0	0	0	0	0	0	0	0	0	0	0	0	0	0	0
0	0	0	0	0	9	0	5	0	9	0	0	0	8	0	0	0	0	0
0	0	0	0	0	0	0	0	0	0	0	0	0	0	0	0	0	0	0
0	0	0	0	0	0	0	0	0	0	0	0	0	0	0	0	9	0	0
0	0	0	0	0	0	0	0	0	0	0	0	0	0	0	0	0	0	0
0	0	0	0	7	0	0	0	0	8	0	0	0	0	0	0	20	0	0
0	0	0	0	0	0	0	0	0	0	0	0	0	0	0	0	0	0	0
0	0	0	0	0	0	0	0	0	0	0	0	0	0	0	0	0	0	0
0	0	0	0	0	0	0	0	0	0	0	0	0	0	0	0	0	0	0
0	0	0	0	0	0	0	0	0	0	0	0	0	0	0	0	0	0	0
0	0	0	0	0	0	0	0	13	0	0	0	0	3	0	0	0	0	0
0	13	0	0	0	28	0	0	0	0	0	0	0	0	0	3	0	0	0

0	0	0	0	0	0	0	0	0	0	0	0	0	0	0	0	0	0	0
0	0	0	0	0	0	0	7	0	0	0	0	0	0	11	0	33	0	0
0	0	0	10	0	0	0	0	0	0	0	0	0	0	0	0	0	0	0
0	0	0	0	0	0	0	0	0	0	0	0	0	0	0	0	0	0	0
0	0	0	0	0	0	0	0	0	0	0	0	0	0	0	0	0	0	0
0	2	0	0	0	0	0	0	0	0	0	0	0	0	0	0	0	0	0
0	0	0	0	0	0	0	0	0	0	0	0	0	0	0	0	0	0	0
0	0	0	0	0	0	8	0	0	0	0	0	7	0	0	0	0	0	0
0	0	0	0	0	0	0	0	0	0	0	0	0	0	0	0	0	0	0
0	0	0	0	0	0	0	0	0	0	0	0	0	0	0	0	0	0	0
0	0	0	0	0	0	0	0	0	0	0	0	0	0	0	0	0	0	0
0	0	0	0	0	0	0	0	0	0	0	0	0	0	0	0	0	0	0
0	14	0	0	0	0	0	0	0	0	0	0	0	0	0	0	0	0	0
0	0	0	0	0	0	0	0	0	0	0	0	0	0	0	0	0	0	0
0	0	0	0	0	0	0	0	0	0	11	0	0	0	0	0	0	0	0
4	20	31	0	0	0	0	0	0	0	0	0	0	0	0	0	0	0	0
0	0	0	0	0	0	0	0	0	0	0	0	0	0	0	0	0	0	0
0	0	0	0	0	0	0	0	0	0	0	0	0	0	0	0	0	0	0
0	0	0	0	0	0	0	0	0	0	0	0	0	0	0	0	0	0	0
0	0	0	0	0	0	0	0	0	0	0	0	0	0	0	0	0	0	0
0	0	0	0	0	0	0	0	0	0	0	9	0	0	0	0	0	0	0
0	0	0	0	0	0	0	0	0	0	0	0	0	0	0	0	0	0	0
0	0	0	0	0	0	0	0	0	0	0	0	0	0	0	0	0	0	0
0	0	0	0	0	0	0	0	0	0	0	0	0	0	0	0	0	0	0
0	15	0	0	0	0	0	0	0	0	0	0	0	0	0	0	0	0	0
0	0	0	0	0	0	0	0	0	0	0	0	16	0	0	0	12	0	0
0	0	0	0	0	0	0	0	0	0	0	0	0	0	0	0	0	0	0
0	0	0	0	0	0	0	0	0	0	0	0	0	0	0	0	0	0	0
0	0	0	0	0	0	0	0	0	0	0	0	0	0	0	0	0	0	0
0	0	0	0	0	0	0	0	0	0	0	0	0	0	0	0	0	0	0
0	0	0	0	0	0	0	0	0	0	0	0	0	0	0	0	0	0	0
5	0	0	0	0	0	0	0	0	0	0	0	0	0	0	0	0	0	0
0	0	0	0	0	0	0	0	0	0	0	0	0	0	0	0	0	0	0
0	0	0	0	0	0	0	0	0	0	0	0	0	0	0	0	0	0	0
0	0	0	0	0	0	0	0	0	0	0	0	0	0	0	0	0	0	0
0	0	0	0	0	0	0	0	0	0	0	0	0	0	0	0	0	0	0
0	0	0	0	0	0	0	0	0	0	0	0	0	0	0	0	0	0	0

0	0	0	0	0	0	0	0	0	0	0	0	0	0	0	0	0	0	0
0	0	0	0	0	0	0	0	0	0	0	0	0	0	0	0	0	0	0
0	0	0	0	0	0	0	0	0	0	0	0	0	0	0	0	0	0	0
0	0	0	0	0	0	0	0	0	0	0	0	0	0	0	0	0	0	0
0	0	0	0	0	0	0	0	0	0	0	0	0	0	0	0	0	0	0
0	0	0	0	0	0	0	0	0	0	0	0	0	0	0	0	0	0	0
0	0	0	0	0	0	0	0	0	0	0	0	0	0	0	0	0	0	0
0	0	0	0	0	0	0	0	0	0	0	0	0	0	0	0	0	0	0
0	0	0	0	0	0	0	0	0	0	0	0	0	0	0	0	0	0	0
0	0	0	0	0	0	0	0	0	0	0	0	0	0	0	0	0	0	0
0	0	0	0	0	0	0	0	0	0	0	0	0	0	0	0	0	0	0
0	0	0	0	0	0	0	0	0	0	0	0	0	0	0	0	0	0	0
0	0	0	0	0	0	0	0	0	0	0	0	0	0	0	0	0	0	0
0	0	0	0	0	0	0	0	0	0	0	0	0	0	0	0	0	0	0
0	0	0	0	0	0	0	0	0	0	0	0	0	0	0	0	0	0	0
0	0	0	0	0	0	0	0	0	0	0	0	0	0	0	0	0	0	0
0	0	0	0	0	0	0	0	0	0	0	0	0	0	0	0	0	0	0
0	0	0	0	0	0	0	0	0	0	0	0	0	0	0	0	0	0	0
0	0	5	0	0	0	0	0	0	0	0	0	0	0	0	0	0	0	0
0	0	0	0	0	0	0	0	0	0	0	0	0	0	0	0	0	0	0
0	0	0	0	0	0	0	0	0	0	0	0	0	0	0	0	0	0	0
0	0	0	0	0	0	0	0	0	0	0	0	0	0	0	0	0	0	0
0	0	0	0	0	0	0	0	0	0	0	0	0	0	0	0	0	0	0
0	0	0	0	0	0	0	0	0	0	0	0	0	0	0	0	0	0	0
0	0	0	0	0	0	0	0	0	0	0	0	0	0	0	0	0	0	0
0	0	0	0	0	0	0	0	0	0	0	0	0	0	0	0	0	0	0
0	0	0	0	0	0	0	0	0	0	0	0	0	0	0	0	0	0	0
0	0	0	0	0	0	4	0	0	0	0	0	0	0	0	0	0	0	0
0	0	0	0	0	0	0	0	0	0	0	0	0	0	0	0	0	0	0
0	0	0	0	0	0	0	0	0	0	0	0	0	0	0	0	0	0	0
0	0	0	0	0	0	0	0	0	0	0	0	0	0	0	0	0	0	0
0	0	0	0	0	0	0	0	0	0	0	0	0	0	0	0	0	0	0
0	0	0	0	0	0	0	0	0	0	0	0	0	0	0	0	0	0	0
0	0	0	0	0	0	0	0	0	0	0	0	0	0	0	0	0	0	0
0	0	0	0	0	0	0	0	0	0	0	0	0	0	0	0	0	0	0
0	0	0	0	0	0	0	0	0	0	0	0	0	0	0	0	0	0	0
0	0	0	0	0	0	0	0	0	0	0	0	0	0	0	0	0	0	0

0	0	0	0	0	0	0	0	0	0	0	0	0	0	0	0	0	0	0
0	0	0	0	0	0	0	0	0	0	0	0	0	0	0	0	0	0	0
0	0	0	0	0	0	0	0	0	0	0	0	0	0	0	0	0	0	0
0	0	0	0	0	0	0	0	0	0	3	3	3	3	3	3	0	0	0
0	0	0	0	0	0	0	0	0	0	0	0	0	0	0	0	0	0	0
0	0	0	0	0	0	0	0	0	0	0	0	0	0	0	0	0	0	0
0	0	0	0	0	0	0	0	0	0	0	0	0	0	0	0	0	0	0
0	0	0	0	0	0	0	0	0	0	0	0	0	0	0	0	0	0	0
0	0	0	0	0	0	0	0	0	0	0	0	0	0	0	0	0	0	0
0	0	0	0	0	0	0	0	0	0	0	0	0	0	0	0	0	0	0
0	0	0	0	0	0	0	0	0	0	0	0	0	0	0	0	0	0	0
0	0	0	0	0	0	0	0	0	0	0	0	0	0	0	0	0	0	0
0	0	0	0	0	0	0	0	0	0	0	0	0	0	0	0	0	0	0
0	0	0	0	0	0	0	0	0	0	0	0	0	0	0	0	0	0	0
0	0	0	0	0	0	0	0	0	0	0	0	0	0	0	0	0	0	0
0	0	0	0	0	0	0	0	0	0	0	0	0	0	0	0	0	0	0
0	0	0	0	0	0	0	0	0	0	0	0	0	0	0	0	0	0	0
0	0	0	0	0	0	0	0	0	0	0	0	0	0	0	0	0	0	0
0	0	0	0	0	0	0	0	0	0	0	0	0	0	0	0	0	0	0
0	0	0	0	0	0	0	0	0	0	0	0	0	0	0	0	0	0	0
0	0	0	0	0	0	0	0	0	0	0	0	0	0	0	0	0	0	0
0	0	0	0	0	0	0	0	0	0	0	0	0	0	0	0	0	0	0
0	0	0	0	0	0	0	0	0	0	0	0	0	0	0	0	0	0	0
0	0	0	0	0	0	0	0	0	0	0	0	0	0	0	0	0	0	0
0	0	0	0	0	0	0	0	0	0	0	0	0	0	0	0	0	0	0
0	0	0	0	0	0	0	0	0	0	0	0	0	0	0	0	0	0	0
0	0	0	0	0	2	2	2	2	2	0	0	0	0	0	0	0	0	0
0	0	0	0	0	0	0	0	0	0	0	0	0	0	0	0	0	0	0
0	0	0	0	0	0	0	0	0	0	0	0	0	0	0	0	0	0	0
0	0	0	0	0	0	0	0	0	0	0	0	0	0	0	0	0	0	0
0	0	0	0	0	0	0	0	0	0	0	0	0	0	0	0	0	0	0
0	0	0	0	0	0	0	0	0	0	0	0	0	0	0	0	0	0	0
0	0	0	0	0	0	0	0	0	0	0	0	0	0	0	0	0	0	0
0	0	0	0	0	0	0	0	0	0	0	0	0	0	0	0	0	0	0
0	0	0	0	0	0	0	0	0	0	0	0	0	0	0	0	0	0	0
0	0	0	0	0	0	0	0	0	0	0	0	0	0	0	0	0	0	0
0	0	0	0	0	0	0	0	0	0	0	0	0	0	0	0	0	0	0

0	0	0	0	0	0	0	0	0	0	0	0	0	0	0	0	0	0	0
0	0	0	0	0	0	0	0	0	0	0	0	0	0	0	0	0	0	0
0	0	0	0	0	0	0	0	0	0	0	0	0	0	0	0	0	0	0
0	0	0	0	0	0	0	0	0	0	0	0	0	0	0	0	0	0	0
0	0	0	0	0	0	0	0	0	0	0	0	0	0	0	0	0	0	0
0	0	0	0	0	0	0	0	0	0	0	0	0	0	0	0	0	0	0
0	0	0	0	0	0	0	0	0	0	0	0	0	0	0	0	0	0	0
0	0	0	0	0	0	0	0	0	0	0	0	0	0	0	0	0	0	0
0	0	0	0	0	0	0	0	0	0	0	0	0	0	0	0	0	0	0
0	0	0	0	0	0	0	0	0	0	0	0	0	0	0	0	0	0	0
0	0	0	0	0	0	0	0	0	0	0	0	0	0	0	0	0	0	0
0	0	0	0	0	0	0	0	0	0	0	0	0	0	0	0	0	0	0
0	0	0	0	0	0	0	0	0	0	0	0	0	0	0	0	0	0	0
0	0	0	0	0	0	0	0	0	0	0	0	0	0	0	0	0	0	0
0	0	0	0	0	0	0	0	0	0	0	0	0	0	0	0	0	0	0
0	0	0	0	0	0	0	0	0	0	0	0	0	0	0	0	0	0	0
0	0	0	0	0	0	0	0	0	0	0	0	0	0	0	0	0	0	0
0	0	0	0	0	0	0	0	0	0	0	0	0	0	0	0	0	0	0
0	0	0	0	0	0	0	0	0	0	0	0	0	0	0	0	0	0	0
0	0	0	0	0	0	0	0	0	0	0	0	0	0	0	0	0	0	0
0	0	0	0	0	0	0	0	0	0	0	0	0	0	0	0	0	0	0
0	0	0	0	0	0	0	0	0	0	0	0	0	0	0	0	0	0	0
0	0	0	0	0	0	0	0	0	0	0	0	0	0	0	0	0	0	0
0	0	0	0	0	0	0	0	0	0	0	0	0	0	0	0	0	0	0
0	0	0	0	0	0	0	0	0	0	0	0	0	0	0	0	0	0	0
0	0	0	0	0	0	0	0	0	0	0	0	0	0	0	0	0	0	0
0	0	0	0	0	0	0	0	0	0	0	0	0	0	0	0	0	0	0
0	0	0	0	0	0	0	0	0	0	0	0	0	0	0	0	0	0	0
0	0	0	0	0	0	0	0	0	0	0	0	0	0	0	0	0	0	0
0	0	0	0	0	0	0	0	0	0	0	0	0	0	0	0	0	0	0
0	0	0	0	0	0	0	0	0	0	0	0	0	0	0	0	0	0	0
0	0	0	0	0	0	0	0	0	0	0	0	0	0	0	0	0	0	0
0	0	0	0	0	0	0	0	0	0	0	0	0	0	0	0	0	0	0
0	0	0	0	0	0	0	0	0	0									
Jan_DL012	1392	1406	727	684	109	229	136	115	511	177	617	393	120	116	190	575	159	215
177	0	181	111	119	128	451	172	532	316	231	353	186	168	84	272	128	126	371
249	33	0	60	77	183	82	446	99	238	253	341	37	92	148	395	237	222	235

236	96	270	29	182	0	39	67	142	15	197	61	32	0	5	49	11	42	247
82	292	74	90	57	67	70	60	0	58	148	90	20	14	42	167	64	28	66
0	12	144	0	72	15	105	5	0	21	19	0	75	0	41	0	0	56	0
0	11	0	0	13	0	0	0	0	0	11	15	0	10	0	0	16	0	0
0	0	0	30	0	82	27	27	38	17	10	0	22	97	17	0	0	0	0
0	15	0	77	0	29	38	0	0	0	0	0	22	12	0	113	2	0	8
0	0	13	0	0	0	0	21	0	14	0	19	0	0	26	9	7	0	0
34	0	0	0	0	0	0	0	0	9	0	0	0	0	0	0	0	0	0
12	0	22	0	9	15	0	0	0	0	0	8	28	0	0	10	0	0	0
0	0	17	19	0	9	0	0	30	0	0	19	4	8	0	0	0	48	0
0	0	0	0	0	0	0	0	0	0	0	0	0	7	6	0	0	15	0
0	0	0	4	0	7	0	0	0	0	7	0	0	0	12	5	0	0	0
0	0	0	0	0	0	0	0	10	51	0	0	6	28	0	0	0	0	0
0	7	0	0	0	39	0	0	0	0	0	0	0	0	0	12	0	0	0
0	0	0	0	0	0	0	0	0	0	0	12	0	0	0	0	0	0	0
6	7	0	0	10	0	0	0	0	0	37	0	0	0	0	0	0	0	12
25	0	0	0	28	0	7	0	0	0	0	0	0	0	0	0	28	0	11
0	0	0	0	5	0	0	0	0	0	0	0	0	0	0	0	0	0	0
0	0	10	0	0	0	7	5	0	0	0	10	0	4	0	0	0	0	0
0	0	0	0	0	0	0	28	18	0	0	0	9	0	0	0	0	0	0
0	0	0	0	0	22	0	0	0	0	0	0	0	0	0	0	0	0	0
0	17	0	0	0	0	0	0	0	0	0	0	0	0	0	0	0	37	0
0	0	0	0	0	0	7	0	0	0	0	0	0	0	0	0	0	0	0
0	0	0	0	0	0	0	0	0	0	0	0	0	0	0	0	0	0	0
0	0	0	0	0	0	0	0	0	0	0	0	0	0	0	0	0	0	21
0	0	0	0	0	0	0	0	0	0	0	0	0	0	0	0	0	0	0
0	11	11	5	0	0	0	0	0	0	0	5	0	0	0	0	0	0	0
0	8	0	0	0	0	0	0	0	0	0	0	0	0	0	0	0	0	0
0	0	0	0	0	0	0	0	0	0	0	0	0	0	0	0	0	0	0
0	0	5	0	0	0	0	0	0	0	0	0	0	0	0	0	0	0	0
0	0	0	0	0	0	0	0	0	0	0	0	0	0	0	0	0	0	0
0	0	10	0	0	0	0	0	0	0	0	0	5	0	0	0	0	0	0
7	0	0	0	0	0	0	0	0	0	0	0	0	0	0	0	0	0	0
0	0	0	0	0	0	0	0	0	0	0	0	0	0	0	0	0	0	0
0	0	0	0	0	0	0	0	0	0	0	0	0	0	0	0	0	0	0
0	0	0	0	0	0	0	0	0	0	0	0	0	0	0	0	0	0	0
0	0	0	0	0	0	3	0	0	0	0	0	0	0	0	0	7	0	0

0	0	0	0	0	0	0	0	0	0	0	0	0	5	0	0	0	0	0
0	0	0	0	0	0	0	0	0	0	0	0	0	0	0	0	0	0	0
0	0	0	0	0	0	0	0	0	0	0	0	0	0	0	0	0	0	0
0	0	0	0	0	0	0	0	0	0	0	0	0	0	0	13	0	0	0
0	0	0	0	0	0	0	0	0	0	0	5	0	0	0	0	0	0	0
0	0	0	0	0	0	0	0	0	0	5	0	0	0	0	0	0	0	0
0	0	0	0	0	0	0	0	0	5	0	0	0	0	0	0	0	0	0
0	0	0	0	0	0	0	0	0	0	0	0	0	0	0	0	0	0	0
0	0	0	0	0	0	0	0	0	0	0	0	0	0	0	0	0	0	0
0	0	2	0	0	0	0	0	0	0	0	0	0	0	0	3	0	0	0
0	0	0	0	0	0	0	0	0	0	0	0	0	0	0	0	0	0	0
0	0	0	0	0	0	0	0	0	0	0	0	0	0	0	0	0	0	0
0	0	0	0	0	0	0	0	0	0	0	0	0	0	0	0	0	0	0
0	0	0	0	0	0	0	0	0	0	0	0	0	0	0	0	0	0	0
0	0	0	0	0	0	0	0	0	0	0	0	0	0	0	0	0	0	0
0	0	13	0	0	0	0	0	0	0	0	0	0	0	0	0	0	0	0
0	0	0	0	0	0	0	0	0	0	0	0	0	0	0	0	0	0	0
0	8	0	0	0	0	0	0	0	0	0	0	0	0	0	0	0	0	0
0	0	0	0	0	0	0	0	0	0	0	0	0	0	0	0	0	0	0
0	0	0	0	0	0	0	0	0	0	0	0	0	0	0	0	0	0	0
0	0	0	0	0	0	0	0	0	0	0	0	0	0	0	0	0	0	0
0	0	0	0	0	0	0	0	0	0	0	0	0	0	0	0	0	0	0
3	0	0	0	0	0	0	0	0	0	0	0	0	0	0	0	0	0	0
0	0	0	0	0	0	0	0	0	0	0	0	0	0	0	0	0	0	0
0	0	0	0	0	0	0	0	0	0	0	0	0	0	0	0	0	0	0
0	0	0	0	0	0	0	0	0	0	0	0	0	0	0	0	0	0	0
0	0	0	0	0	0	0	0	0	0	0	0	0	0	0	0	0	0	0
0	0	0	0	0	0	0	0	0	0	0	0	0	0	0	0	0	0	0
0	0	0	0	0	0	0	0	0	0	0	0	0	0	0	0	0	0	0
0	0	0	0	0	0	0	0	0	0	0	0	0	0	0	17	0	0	0
0	0	0	0	0	0	0	0	0	0	0	0	0	0	0	0	0	0	0
0	0	0	0	0	0	0	0	0	0	0	0	0	0	0	0	0	0	0
0	0	0	0	0	0	0	0	0	0	0	0	0	0	0	0	0	0	0
0	0	0	0	0	0	0	0	0	0	0	0	0	0	0	0	0	0	0
0	0	0	0	0	0	0	0	0	0	0	0	0	0	0	0	0	0	0
0	0	0	0	0	0	0	0	0	0	0	0	0	0	0	0	0	0	0
0	0	0	0	0	0	0	0	0	0	0	0	0	0	0	0	0	0	0

0	0	0	0	0	0	0	0	0	0	0	0	0	0	0	0	0	0	0
0	0	0	0	0	0	0	0	0	0	0	0	0	0	0	0	0	0	0
0	0	0	0	0	0	0	0	0	0	0	0	0	0	0	0	0	0	0
0	0	0	0	0	0	0	0	0	0	0	0	0	0	0	0	0	0	0
0	0	0	0	0	0	0	0	0	0	0	0	0	0	0	0	0	0	0
0	0	0	0	0	0	0	0	0	0	0	0	0	0	0	0	0	0	0
0	0	0	0	0	0	0	0	0	0	0	0	0	0	0	0	0	0	0
0	0	0	0	0	0	0	0	0	0	0	0	0	0	0	0	0	0	0
0	0	0	0	0	0	0	0	0	0	0	0	0	0	0	0	0	0	0
0	0	0	0	0	0	0	0	0	0	0	0	0	0	0	0	0	0	0
0	0	0	0	0	0	0	0	0	0	0	0	0	0	0	0	0	0	0
0	0	0	0	0	0	0	0	0	0	0	0	0	0	0	0	0	0	0
0	0	0	0	0	0	0	0	0	0	0	0	0	0	0	0	0	0	0
0	0	0	0	0	0	0	0	0	0	0	0	0	0	0	0	0	0	0
0	0	0	0	0	0	0	0	0	0	0	0	0	0	0	0	0	0	0
0	0	0	0	0	0	0	0	0	0	0	0	0	0	0	0	0	0	0
0	0	0	0	0	0	0	0	0	0	0	0	0	0	0	0	0	0	0
0	0	0	0	0	0	0	0	0	0	0	0	0	0	0	0	0	0	0
0	0	0	0	0	0	0	0	0	0	0	0	0	0	0	0	0	0	0
0	0	0	0	0	0	0	0	0	0	0	0	0	0	0	0	0	0	0
0	0	0	0	0	0	0	0	0	0	0	0	0	0	0	0	0	0	0
0	0	0	0	0	0	0	0	0	0	0	0	0	0	0	0	0	0	0
0	0	0	0	0	0	0	0	0	0	0	0	0	0	0	0	0	0	0
0	0	0	0	0	0	0	0	0	0	0	0	0	0	0	0	0	0	0
0	0	0	0	0	0	0	0	0	0	0	0	0	0	0	0	0	0	0
0	0	0	0	0	0	0	0	0	0	0	0	0	0	0	0	0	0	0
0	0	0	0	0	0	0	0	0	0	0	0	0	0	0	0	0	0	0
0	0	0	0	0	0	0	0	0	0	0	0	0	0	0	0	0	0	0
0	0	0	0	0	0	0	0	0	0	0	0	0	0	0	0	0	0	0
0	0	0	0	0	0	0	0	0	0	0	0	0	0	0	0	0	0	0
0	0	0	0	0	0	0	0	0	0	0	0	0	0	0	0	0	0	0
6	0	0	0	0	0	0	0	0	0	0	0	0	0	0	0	0	0	0
0	0	0	0	0	0	0	0	0	0	0	0	0	0	0	0	0	0	0
0	0	0	0	0	0	0	0	0	0	0	0	0	0	0	0	0	0	0
0	0	0	0	0	0	0	0	0	0	0	0	0	0	0	0	0	0	0
0	0	0	0	0	0	0	0	0	0	0	0	0	0	0	0	0	0	0
0	0	0	0	0	0	0	0	0	0	0	0	0	0	0	0	0	0	0

0	0	0	0	0	0	0	0	0	0	0	0	0	0	0	0	0	0	0
0	0	0	0	0	0	0	0	0	0	0	0	0	0	0	0	0	0	0
0	0	0	0	0	0	0	0	0	0	0	0	0	0	0	0	0	5	5
0	0	0	0	0	0	0	0	0	0	0	0	0	0	0	0	0	0	0
0	0	0	0	0	0	0	0	0	0	0	0	0	0	0	0	0	0	0
0	0	0	0	0	0	0	0	0	0	0	0	0	0	0	0	0	0	0
0	0	0	0	0	0	0	0	0	0	0	0	0	0	0	0	0	0	0
0	0	0	0	0	0	0	0	0	0	0	0	0	0	0	0	0	0	0
0	0	0	0	0	0	0	0	0	0	0	0	0	0	0	0	0	0	0
0	0	0	0	0	0	0	0	0	0	0	0	0	0	0	0	0	0	0
0	0	0	0	0	0	0	0	0	0	0	0	0	0	0	0	0	0	0
0	0	0	0	0	0	0	0	0	0	0	0	0	0	0	0	0	0	0
0	0	0	0	0	0	0	0	0	0	0	0	0	0	0	0	0	0	0
0	0	0	0	0	0	0	0	0	0	0	0	0	0	0	0	0	0	0
0	0	0	0	0	0	0	0	0	0	0	0	0	0	0	0	0	0	0
0	0	0	0	0	0	0	0	0	0	0	0	0	0	0	4	0	0	0
0	0	0	0	0	0	0	0	0	0	0	0	0	0	0	0	0	0	0
0	0	0	0	0	0	0	0	0	0	0	0	0	0	0	0	0	0	0
0	0	0	0	0	0	0	0	0	0	0	0	0	0	0	0	0	0	0
0	0	0	0	0	0	0	0	0	0	0	0	0	0	0	0	0	0	0
0	0	0	0	0	0	0	0	0	0	0	0	0	0	0	0	0	0	0
0	0	0	0	0	0	0	0	0	0	0	0	0	0	0	0	0	0	0
0	0	0	0	0	0	0	0	0	0	0	0	0	0	0	0	0	0	0
0	0	0	0	0	0	0	0	0	0	0	0	0	0	0	0	0	0	0
0	0	0	0	0	0	0	0	0	0	0	0	0	0	0	0	0	0	0
0	0	0	0	0	0	0	0	0	0	0	0	0	0	0	0	0	0	0
0	0	0	0	0	0	0	0	0	0	0	0	0	0	0	0	0	0	0
0	0	0	0	0	0	0	0	0	0	0	0	0	0	0	0	0	0	0
0	0	0	0	0	0	0	0	0	0	0	0	0	0	0	0	0	0	0
0	0	0	0	0	0	0	0	0	0	0	0	0	0	0	0	0	0	0
0	0	0	0	0	0	0	0	0	0	0	0	0	0	0	0	0	0	0
0	0	0	0	0	0	0	0	0	0	0	0	0	0	0	0	0	0	0
0	0	0	0	0	0	0	0	0	0	0	0	0	0	0	0	0	0	0
0	0	0	0	0	0	0	0	0	0	0	0	0	0	0	0	0	0	0
0	0	0	0	0	0	0	0	0	0	0	0	0	0	0	0	0	0	0
0	0	0	0	0	0	0	0	0	0	0	0	0	0	0	0	0	0	0
0	0	0	0	0	0	0	0	0	0	3	3	0	0	0	0	0	0	0

0	0	0	0	0	0	0	0	0	0	0	0	0	0	0	0	0	0	0
0	0	0	0	0	0	0	0	0	0	0	0	0	0	0	0	0	0	0
0	0	0	0	0	0	0	0	0	0	0	0	0	0	0	0	0	0	0
0	0	0	0	0	0	0	0	0	0	0	0	0	0	0	0	0	0	0
0	0	0	0	0	0	0	0	0	0	0	0	0	0	0	0	0	0	0
0	0	0	0	0	0	0	0	0	0	0	0	0	0	0	0	0	0	0
0	0	0	0	0	0	0	0	0	0	0	0	0	0	0	0	0	0	0
0	0	0	0	0	0	0	0	0	0	0	0	0	0	0	0	0	0	0
0	0	0	0	0	0	0	0	0	0	0	0	0	0	0	0	0	0	0
0	0	0	0	0	0	0	0	0	0	0	0	0	0	0	0	0	0	0
0	0	0	0	0	0	0	0	0	0	0	0	0	0	0	0	0	0	0
0	0	0	0	0	0	0	0	0	0	0	0	0	0	0	0	0	0	0
0	0	0	0	0	0	0	0	0	0	0	0	0	0	0	0	0	0	0
0	0	0	0	0	0	0	0	0	0	0	0	0	0	0	0	0	0	0
0	0	0	0	0	0	0	0	0	0	0	0	0	0	0	0	0	0	0
0	0	0	0	0	0	0	0	0	0	0	0	0	0	0	0	0	0	0
0	0	0	0	0	0	0	0	0	0	0	0	0	0	0	0	0	0	0
0	0	0	0	0	0	0	0	0	0	0	0	0	0	0	0	0	0	0
0	0	0	0	0	0	0	0	0	0	0	0	0	0	0	0	0	0	0
0	0	0	0	0	0	0	0	0	0	0	0	0	0	0	0	0	0	0
0	2	2	2	2	2	2	0	0	0	0	0	0	0	0	0	0	0	0
0	0	0	0	0	0	0	0	0	0	0	0	0	0	0	0	0	0	0
0	0	0	0	0	0	0	0	0	0	0	0	0	0	0	0	0	0	0
0	0	0	0	0	0	0	0	0	0	0	0	0	0	0	0	0	0	0
0	0	0	0	0	0	0	0	0	0	0	0	0	0	0	0	0	0	0
0	0	0	0	0	0	0	0	0	0									
Jan_DL120	1992	1125	408	884	56	376	432	422	411	317	188	85	254	192	149	32	151	266
272	524	131	883	273	116	83	87	84	107	90	0	126	177	130	112	85	262	0
89	48	41	88	181	66	90	0	32	15	113	49	56	167	161	41	0	73	0
0	166	0	49	66	46	118	33	17	188	9	0	0	101	35	129	28	48	0
76	0	73	22	14	72	23	87	45	49	82	43	52	0	32	0	116	41	54
14	74	83	44	0	16	24	87	26	59	0	7	58	55	13	33	0	0	51
0	25	10	25	10	0	0	0	0	0	0	14	30	11	40	14	0	0	0
0	0	0	22	17	0	0	0	0	20	0	0	11	0	21	0	0	0	0
0	0	20	0	0	4	0	13	0	10	35	0	32	66	0	0	0	0	13
15	0	17	0	233	117	0	18	0	84	23	0	0	18	0	55	6	324	0
0	0	13	0	0	0	0	0	0	0	0	0	0	0	24	0	0	33	0

0	26	9	0	0	0	0	0	0	67	0	10	0	0	0	0	89	23	0
0	0	0	0	0	0	0	0	7	0	0	17	37	16	0	0	0	0	0
0	0	0	21	22	0	0	0	0	11	0	0	23	0	5	6	0	5	0
26	0	0	7	0	5	0	0	7	23	0	0	0	0	0	13	0	0	0
8	0	26	0	0	0	0	0	2	0	0	0	0	0	10	23	0	0	0
0	0	20	0	0	0	0	7	0	0	0	13	14	10	14	9	0	0	11
0	15	0	0	0	0	0	6	0	0	0	0	0	0	0	0	0	0	0
8	0	0	15	9	0	0	16	0	0	0	0	0	0	13	0	16	0	11
0	0	0	0	0	0	5	31	0	0	17	0	0	30	0	0	0	0	9
0	0	0	0	0	0	0	0	0	0	0	0	0	0	0	0	0	0	0
0	0	0	0	0	0	0	0	0	0	0	0	0	0	0	0	11	0	0
0	0	0	13	0	0	0	0	0	0	46	0	0	0	29	0	9	0	0
0	0	0	0	0	12	0	0	0	0	0	0	0	0	0	0	0	0	14
0	0	0	0	7	0	0	8	0	0	0	0	0	0	0	0	0	0	0
0	0	0	18	5	0	14	13	0	0	0	0	0	0	0	0	0	0	5
0	0	0	0	0	0	0	0	0	0	0	0	0	0	0	0	0	3	0
0	0	0	0	0	0	0	0	0	0	0	0	0	0	0	0	52	0	0
15	0	0	0	0	0	0	0	0	0	0	0	0	0	0	0	0	0	0
0	0	0	0	0	0	0	0	0	0	0	7	0	0	0	0	0	0	0
0	0	0	0	0	0	0	0	0	0	0	0	0	0	0	0	0	0	0
0	0	0	0	0	0	0	0	0	0	0	0	0	0	0	0	0	0	0
0	0	0	8	0	0	0	0	0	0	0	0	0	0	0	0	0	0	0
0	0	0	0	0	0	0	0	0	0	0	0	0	0	0	7	0	0	0
0	0	0	0	0	0	0	0	0	0	0	0	0	0	0	0	0	0	0
0	0	16	0	0	0	0	0	0	0	0	0	0	0	0	0	0	0	0
0	0	0	0	0	0	0	0	0	0	0	0	0	0	0	0	0	0	0
0	0	0	0	0	0	0	0	0	0	0	0	0	0	0	0	0	0	0
0	0	0	0	0	0	0	0	0	0	0	0	0	0	0	0	0	0	0
0	0	0	0	0	0	0	0	0	0	0	0	0	0	0	0	0	0	0
0	0	0	0	0	0	0	0	0	0	0	0	0	0	0	0	13	0	0
0	0	0	0	0	0	0	2	0	0	0	0	0	0	0	0	0	0	0
0	0	0	0	0	0	0	0	0	0	16	0	0	0	0	0	0	0	0
0	0	0	0	0	0	0	0	0	0	0	0	0	0	0	0	0	0	0
0	0	0	0	0	0	0	0	0	0	0	0	0	0	0	0	0	0	0
0	0	0	0	0	0	0	0	0	0	0	0	0	24	0	0	0	0	0
0	0	0	0	0	0	0	0	0	0	0	0	0	0	0	0	0	0	0
0	0	0	0	0	0	0	0	0	0	0	0	0	0	0	0	0	0	0

0	0	0	0	0	0	0	0	0	0	0	0	0	0	0	0	0	0	0
0	0	0	0	0	0	0	0	0	0	0	0	0	0	0	0	0	0	0
0	0	0	0	0	0	0	0	0	0	0	0	0	0	0	0	0	0	0
0	0	0	0	0	0	0	0	6	0	0	0	0	0	0	0	0	0	0
6	0	0	0	0	0	0	0	12	0	36	0	0	0	0	0	35	0	0
0	0	0	0	0	0	0	0	0	0	0	0	0	0	0	0	0	0	0
0	0	0	0	0	0	7	0	0	0	0	0	0	0	0	0	0	0	0
0	0	0	0	0	0	0	0	0	0	0	0	0	0	0	0	0	0	0
0	0	0	0	0	0	0	0	0	0	0	0	0	0	0	0	28	0	0
0	0	0	0	0	0	0	10	0	0	0	0	0	0	0	0	0	0	0
0	0	0	0	0	0	0	0	0	0	0	0	0	0	0	0	0	0	0
0	0	0	0	0	0	0	0	0	0	0	0	0	0	0	0	0	0	0
0	23	0	12	0	0	0	0	0	0	0	0	0	0	0	0	0	0	0
0	0	0	0	0	0	0	0	0	0	0	0	0	0	0	0	0	0	0
0	0	0	0	0	0	0	0	0	0	0	8	0	0	0	0	0	0	0
0	0	0	0	0	0	0	0	0	0	0	0	0	0	0	0	0	0	0
0	0	0	0	0	0	0	0	0	0	0	0	0	0	0	0	0	0	0
0	0	0	0	0	0	0	0	0	0	0	0	0	0	17	0	0	0	0
0	0	0	0	5	0	0	0	0	0	0	0	0	0	2	0	0	0	0
0	0	0	0	0	0	0	0	0	0	0	0	0	0	0	0	0	0	0
0	0	0	0	0	0	0	0	0	0	0	3	0	0	0	0	0	0	0
0	0	0	0	15	0	0	0	0	0	0	0	0	0	0	0	0	0	0
0	0	0	0	0	0	0	0	0	0	0	0	0	0	14	0	0	0	0
0	0	4	0	0	0	0	0	0	0	0	0	0	0	0	0	0	0	0
0	0	0	0	0	0	0	0	0	0	0	0	0	0	0	0	0	0	0
0	0	0	0	0	0	0	0	0	0	0	0	0	0	0	0	0	0	0
0	0	0	0	0	0	0	0	0	0	0	0	0	0	0	0	0	0	0
0	0	0	0	0	0	0	0	0	0	0	0	0	0	0	0	0	0	0
0	0	0	0	0	0	0	0	0	0	0	0	0	0	0	0	0	0	0
0	0	0	0	11	0	0	0	0	0	0	0	0	0	0	0	0	0	0
0	0	0	0	0	0	0	0	0	0	0	0	0	0	0	0	0	0	0
0	0	0	0	0	0	0	0	0	0	0	0	0	0	0	0	0	0	0
0	0	0	0	0	0	0	0	0	0	0	0	0	0	0	0	0	0	0
0	0	0	0	0	0	0	0	0	0	0	0	0	0	0	0	0	0	0
0	0	0	0	0	0	0	0	0	0	0	0	0	0	0	0	0	0	0
0	0	0	0	0	0	0	0	0	0	0	0	0	0	0	0	0	0	0
0	9	0	0	0	0	0	0	0	0	0	0	0	0	0	0	0	0	0

0	0	0	0	0	0	0	0	0	0	0	0	0	0	0	0	0	0	0
0	0	0	0	0	0	0	0	0	0	0	0	0	0	0	0	0	0	0
0	0	0	0	0	0	0	0	0	0	0	0	0	0	0	0	0	0	0
0	0	0	0	0	0	8	0	0	0	0	0	0	0	0	0	0	0	0
0	0	0	0	0	0	0	0	0	0	0	0	0	0	0	0	0	0	0
0	0	0	0	0	0	0	0	0	0	0	0	0	0	0	0	0	0	0
0	0	0	0	0	0	0	0	0	0	0	0	0	0	0	0	0	0	0
0	0	0	0	0	0	0	0	0	0	0	0	0	0	0	0	0	0	0
0	0	0	0	0	7	0	0	0	0	0	0	0	0	0	0	0	0	0
0	0	0	0	0	0	0	0	0	0	0	0	0	0	0	0	0	0	0
0	0	0	0	0	0	0	0	0	0	0	0	0	0	0	0	0	0	0
0	0	0	0	0	0	0	0	0	0	0	0	0	0	0	0	0	0	0
0	0	0	0	0	0	0	0	0	0	0	0	0	0	0	0	0	0	0
0	0	0	0	0	0	0	0	0	0	0	0	0	0	0	0	0	0	0
0	0	0	0	0	0	0	0	0	0	0	0	0	0	0	0	0	0	0
0	0	0	0	0	0	0	0	0	0	0	0	0	0	0	0	0	0	0
0	0	0	0	0	0	0	0	0	0	0	0	0	0	0	0	0	0	0
0	0	0	0	0	0	0	0	0	0	0	0	0	0	0	0	0	0	0
0	0	0	0	0	0	0	0	0	0	0	0	0	0	0	0	0	0	0
0	0	0	0	0	0	0	0	0	0	0	0	0	0	0	0	0	0	0
0	0	0	0	0	0	0	0	0	0	0	0	0	0	0	0	0	0	0
0	0	0	0	0	0	0	0	0	0	0	0	0	0	0	0	0	0	0
0	0	0	0	0	0	0	0	0	0	0	0	0	0	0	0	0	0	0
0	0	0	0	0	0	0	0	0	0	0	0	0	0	0	0	0	0	0
0	0	0	0	5	0	0	0	0	0	0	0	0	0	0	0	0	0	0
0	0	0	0	0	0	0	0	0	0	0	0	0	0	0	0	0	0	0
0	0	0	0	0	0	0	0	0	0	0	0	0	0	0	0	0	0	0
0	0	0	0	0	0	0	0	0	0	0	0	0	0	0	0	0	0	0
0	0	0	0	0	0	0	0	0	0	0	0	0	0	0	0	0	0	0
0	0	0	0	0	0	0	0	0	0	0	0	0	0	0	0	0	0	0
0	0	0	0	0	0	0	0	0	0	0	0	0	0	0	0	0	0	0
0	0	0	0	0	0	0	0	0	0	0	0	0	0	0	0	0	0	0
0	0	0	0	0	0	0	0	0	0	0	0	0	0	0	0	0	0	0
0	0	0	0	0	0	0	0	0	0	0	0	0	0	0	0	0	0	0
0	0	0	0	0	0	0	0	0	0	0	0	0	0	0	0	0	0	4
0	0	0	0	0	0	0	0	0	0	0	0	0	0	0	0	0	0	0
0	0	0	0	0	0	0	0	0	0	0	0	0	0	0	0	0	0	0

0	0	0	0	0	0	0	0	0	0	0	0	0	0	0	0	0	0	0
0	0	0	0	0	0	0	0	0	0	0	0	0	0	0	0	0	0	0
0	0	0	0	0	0	0	0	0	0	0	0	0	0	0	0	0	0	0
0	0	0	0	0	0	0	0	0	0	0	0	0	0	0	0	0	0	0
0	0	0	0	0	0	0	0	0	0	0	0	0	0	0	0	0	0	0
0	0	0	0	0	0	0	0	0	0	0	0	0	0	0	0	0	0	0
0	0	0	0	0	0	0	0	0	0	0	0	0	0	0	0	0	0	0
0	0	0	0	0	0	0	0	0	0	0	0	0	0	0	0	0	0	0
0	0	0	0	0	0	0	0	0	0	0	0	0	0	0	0	0	0	0
0	0	0	0	0	0	0	0	0	0	0	0	0	0	0	0	0	0	0
0	0	0	0	0	0	0	0	0	0	0	0	0	0	0	0	0	0	0
0	0	0	0	0	0	0	0	0	0	0	0	0	0	0	0	0	0	0
0	0	0	0	0	0	0	0	0	0	0	0	0	0	0	0	0	0	0
0	0	0	0	0	0	0	0	0	0	0	0	0	0	0	0	0	0	0
0	0	0	0	0	0	0	0	0	0	0	0	0	0	0	0	0	0	0
0	0	0	0	0	0	0	0	0	0	0	0	0	0	0	0	0	0	0
0	0	3	3	0	0	0	0	0	0	0	0	0	0	0	0	0	0	0
0	0	0	0	0	0	0	0	0	0	0	0	0	0	0	0	0	0	0
0	0	0	0	0	0	0	0	0	0	0	0	0	0	0	0	0	0	0
0	0	0	0	0	0	0	0	0	0	0	0	0	0	0	0	0	0	0
0	0	0	0	0	0	0	0	0	0	0	0	0	0	0	0	0	0	0
0	0	0	0	0	0	0	0	0	0	0	0	0	0	0	0	0	0	0
0	0	0	0	0	0	0	0	0	0	0	0	0	0	0	0	0	0	0
0	0	0	0	0	0	0	0	0	0	0	0	0	0	0	0	0	0	0
0	0	0	0	0	0	0	0	0	0	0	0	0	0	0	0	0	0	0
0	0	0	0	0	0	0	0	0	0	0	0	0	0	0	0	0	0	0
0	0	0	0	0	0	0	0	0	0	0	0	0	0	0	0	0	0	0
0	0	0	0	0	0	0	0	0	0	0	0	0	0	0	0	0	0	0
0	0	0	0	0	0	0	0	0	0	0	0	0	0	0	0	0	0	0
0	0	0	0	0	0	0	0	0	0	0	0	0	0	0	0	0	0	0
0	0	0	0	0	0	0	0	0	0	0	0	0	0	0	0	0	0	0
0	0	0	0	0	0	0	0	0	0	0	0	0	0	0	0	0	0	0
0	0	0	0	0	0	0	0	0	0	0	0	0	0	0	0	0	0	0
0	0	0	0	0	0	0	0	0	0	0	0	0	0	0	0	0	0	0
0	0	0	0	0	0	0	0	0	0	0	0	0	0	0	0	0	0	0
0	0	0	0	0	0	0	0	0	0	0	0	0	0	0	0	0	0	0
0	0	0	0	0	0	0	0	0	0	0	0	0	0	0	0	0	0	0

0	0	0	0	0	0	0	0	0	0	0	0	0	0	0	0	0	0	0
0	0	0	0	0	0	0	0	0	0	0	0	0	0	0	0	0	0	0
0	0	0	0	0	0	0	0	0	0	0	0	0	0	0	0	0	0	0
0	0	0	0	0	0	0	0	0	0	0	0	0	0	0	0	0	0	0
0	0	0	0	0	0	0	0	0	0	0	0	0	2	2	2	2	2	2
2	2	2	0	0	0	0	0	0	0	0	0	0	0	0	0	0	0	0
0	0	0	0	0	0	0	0	0	0	0	0	0	0	0	0	0	0	0
0	0	0	0	0	0	0	0	0	0	0	0	0	0	0	0	0	0	0
0	0	0	0	0	0	0	0	0	0	0	0	0	0	0	0	0	0	0
0	0	0	0	0	0	0	0	0	0	0	0	0	0	0	0	0	0	0
0	0	0	0	0	0	0	0	0	0	0	0	0	0	0	0	0	0	0
0	0	0	0	0	0	0	0	0	0	0	0	0	0	0	0	0	0	0
0	0	0	0	0	0	0	0	0	0	0	0	0	0	0	0	0	0	0
0	0	0	0	0	0	0	0	0	0	0	0	0	0	0	0	0	0	0
0	0	0	0	0	0	0	0	0	0	0	0	0	0	0	0	0	0	0
0	0	0	0	0	0	0	0	0	0	0	0	0	0	0	0	0	0	0
0	0	0	0	0	0	0	0	0	0	0	0	0	0	0	0	0	0	0
0	0	0	0	0	0	0	0	0	0									
Jan_DL122	1558	1207	494	675	163	1356	615	621	288	484	135	275	427	416	284	204	408	352
378	373	84	0	119	85	129	277	170	127	120	0	229	91	182	73	202	169	0
129	45	0	264	143	39	144	31	54	88	135	48	30	227	54	63	0	169	0
0	0	58	41	122	0	78	68	42	125	15	168	46	53	72	19	16	22	0
26	18	31	32	0	0	41	29	86	67	0	67	97	62	23	0	65	0	25
0	55	125	158	49	22	0	0	0	11	0	0	0	56	49	20	0	0	57
0	86	0	24	0	0	0	0	0	88	8	58	11	0	0	0	0	0	80
80	0	0	11	0	0	0	0	49	34	0	0	7	0	0	0	0	0	0
0	13	0	0	44	9	0	0	0	0	15	0	20	0	0	0	10	0	14
43	0	13	0	119	0	0	12	0	28	0	0	0	0	0	37	22	176	0
0	0	0	22	0	0	0	0	0	0	0	0	29	0	0	0	0	28	0
0	0	12	0	20	43	0	0	0	0	67	25	29	0	0	0	0	13	0
0	0	0	0	0	0	0	0	0	0	0	9	0	18	0	0	0	0	0
14	0	0	8	20	0	40	19	0	0	0	0	20	0	6	0	0	0	0
0	0	0	30	0	0	13	0	19	0	0	6	0	0	0	3	0	0	31
0	0	0	0	0	0	0	0	0	0	0	0	0	0	0	0	0	9	0
0	0	0	0	0	0	0	0	0	0	13	0	0	0	0	16	0	0	17
0	0	0	0	0	0	0	25	0	0	0	0	0	0	0	6	0	0	0
0	0	36	0	16	0	0	0	0	8	0	11	0	0	0	0	0	0	0

0	0	0	0	0	0	6	4	0	0	0	0	0	0	0	0	0	0	6
0	0	79	0	10	0	0	0	0	0	0	0	12	16	0	0	0	0	0
0	0	0	0	0	0	0	0	0	0	0	6	0	9	0	0	10	0	0
0	5	0	0	0	0	12	0	0	0	0	0	0	0	12	0	10	0	0
0	0	0	0	0	0	0	0	0	0	0	0	0	0	0	0	0	0	11
0	0	21	0	0	0	0	0	0	0	0	0	0	0	0	0	0	0	0
0	0	0	0	0	0	0	0	0	0	0	0	0	0	0	0	0	0	15
2	0	0	0	0	0	18	0	0	0	0	0	0	0	0	0	0	7	0
0	0	11	0	0	5	0	0	0	40	0	0	0	0	0	0	0	0	0
0	0	0	0	0	0	0	0	0	0	0	0	0	0	0	0	0	0	0
0	0	0	0	0	0	0	0	0	0	0	0	0	0	0	0	0	0	0
0	0	0	0	0	0	0	0	0	0	0	0	0	0	0	0	0	0	0
7	0	0	0	0	0	0	0	0	0	0	0	0	0	0	0	0	0	0
0	0	0	0	0	0	0	0	0	0	0	0	0	0	0	0	0	0	0
0	0	0	0	0	0	0	0	0	0	0	0	0	0	0	0	0	0	21
0	0	0	0	0	0	0	0	0	0	0	0	0	0	0	0	0	0	0
0	0	0	0	0	0	0	0	0	0	0	0	0	0	0	0	0	0	0
0	0	0	0	0	0	0	0	0	0	0	0	0	0	0	0	0	0	0
0	0	0	6	0	0	0	0	0	0	0	0	0	0	0	0	0	0	0
0	0	0	0	0	0	0	0	0	57	0	0	0	0	0	0	16	0	0
6	0	0	0	0	0	0	0	0	0	0	0	0	0	0	0	0	0	0
0	0	0	0	0	0	0	0	0	0	0	0	0	0	0	0	0	0	0
0	0	0	0	0	0	0	0	0	0	0	0	0	0	0	0	0	38	6
0	0	0	0	0	0	0	32	0	0	0	0	0	0	0	0	0	0	0
0	0	0	0	0	0	0	0	9	0	0	0	0	0	0	0	0	0	0
0	42	0	0	0	0	0	0	0	0	0	0	0	0	65	65	0	0	0
58	0	0	34	0	0	0	0	0	0	0	0	0	0	0	0	0	0	0
0	0	0	0	0	0	0	0	0	0	0	0	0	0	0	0	0	0	13
0	0	0	0	0	0	0	0	55	0	0	0	13	0	0	0	0	0	0
0	0	0	0	0	0	0	0	0	0	0	0	0	0	0	0	0	0	0
0	0	0	0	0	0	0	0	0	0	0	0	0	0	0	0	0	0	0
0	0	0	0	0	0	0	0	0	0	0	6	0	0	0	0	0	0	0
0	0	0	0	0	0	0	0	0	0	0	0	6	0	0	0	0	4	0
0	0	0	0	0	0	0	0	0	0	0	0	0	0	0	0	0	0	0
0	0	0	0	0	0	0	0	0	0	0	0	0	0	0	0	7	0	0
0	0	0	0	0	35	0	0	0	0	0	0	0	0	0	0	0	0	0
0	0	0	0	0	0	0	0	0	0	0	0	0	0	0	0	0	0	0

0	0	0	0	0	0	0	0	0	0	0	0	0	31	0	0	0	0	0
0	0	0	0	0	0	0	0	0	0	0	0	0	0	0	0	0	0	0
0	0	0	0	0	0	0	0	0	0	0	0	0	0	0	0	0	0	0
0	0	0	0	0	0	0	0	7	0	0	0	0	0	0	8	0	25	0
0	0	0	0	0	0	0	0	0	0	0	0	0	0	0	0	0	0	0
0	0	0	0	23	0	0	0	0	0	0	0	0	0	0	0	0	0	0
0	0	0	0	0	0	0	0	0	0	0	0	0	0	0	0	0	0	0
0	0	0	0	0	0	0	0	0	0	0	0	0	0	0	0	0	0	0
0	0	20	0	0	0	0	0	0	0	0	0	0	0	0	0	0	0	0
0	0	0	0	0	0	0	0	0	0	0	0	0	0	0	0	0	0	0
0	0	0	0	0	0	0	0	0	0	0	0	0	0	0	0	0	0	0
0	0	0	0	0	0	0	0	0	0	0	0	0	0	0	0	0	0	0
0	0	0	0	0	0	0	0	0	0	0	0	0	0	0	0	0	0	4
0	0	0	0	0	0	0	0	0	0	16	0	0	0	0	0	0	0	0
0	0	0	0	0	0	0	0	0	0	0	0	0	0	0	0	0	0	0
0	0	0	0	0	0	0	0	0	0	0	0	0	0	0	0	0	0	0
0	0	0	0	0	0	0	0	0	0	0	0	0	0	0	0	0	0	0
0	0	0	0	0	0	0	0	0	0	0	0	0	0	0	0	0	0	0
0	0	0	0	0	0	0	0	0	13	0	0	0	0	0	0	0	0	0
0	0	0	0	0	0	0	0	0	0	0	0	0	0	0	0	0	0	0
0	0	0	0	0	0	0	0	0	0	0	0	0	0	0	0	0	0	0
0	0	0	0	0	0	0	0	0	0	0	0	0	0	0	0	0	0	0
0	0	0	0	0	0	0	0	0	0	0	0	0	0	0	0	0	0	0
0	0	0	0	0	0	0	0	0	0	0	0	0	0	11	0	0	0	0
0	0	0	0	0	0	0	0	0	0	0	0	0	0	0	0	0	0	0
0	0	0	0	0	0	0	0	0	0	0	0	0	0	0	0	0	0	0
0	0	0	0	0	0	0	0	0	0	0	0	0	0	0	0	0	0	0
0	0	0	0	0	0	0	0	0	0	0	0	0	0	0	0	0	0	0
0	0	0	0	0	0	0	0	0	0	0	0	0	0	0	0	0	0	0
0	0	0	0	0	0	0	0	0	0	0	0	0	0	0	0	0	0	0
0	0	0	0	0	0	0	0	0	0	0	0	0	0	0	0	0	0	0
9	0	0	0	0	0	0	0	0	0	0	0	0	0	0	0	0	0	0
0	0	0	0	0	0	0	0	0	0	0	0	0	0	0	0	0	0	0
0	0	0	0	0	0	0	0	0	0	0	0	0	0	0	0	0	0	0
0	0	0	0	0	0	0	0	0	0	0	0	0	0	0	0	0	0	0
0	0	0	0	0	0	0	0	0	0	0	0	0	0	0	0	0	0	0
0	8	0	0	0	0	0	0	0	0	0	0	0	0	0	0	0	0	0

0	0	0	0	0	0	0	0	0	0	0	0	0	0	0	0	0	0	0
0	0	0	0	0	0	0	0	0	0	0	0	0	0	0	0	0	0	0
0	0	0	0	0	0	0	0	0	0	0	0	0	0	0	0	0	0	0
0	0	0	0	0	0	0	0	0	0	0	0	0	0	0	0	0	0	0
0	0	0	0	0	0	0	0	0	0	0	0	0	0	7	0	0	0	0
0	0	0	0	0	0	0	0	0	0	0	0	0	0	0	0	0	0	0
0	0	0	0	0	0	0	0	0	0	0	0	0	0	0	0	0	0	0
0	0	0	0	0	0	0	0	0	0	0	0	0	0	0	0	0	0	0
0	0	0	0	0	0	0	0	0	0	0	0	0	0	0	0	0	0	0
0	0	0	0	0	0	0	0	0	0	0	0	0	0	0	0	0	0	0
0	0	0	0	0	0	0	0	0	0	0	0	0	0	0	0	0	0	0
0	0	0	0	0	0	0	0	0	0	6	0	0	0	0	0	0	0	0
0	0	0	0	0	0	0	0	0	0	0	0	0	0	0	0	0	0	0
0	0	0	0	0	0	0	0	0	0	0	0	0	0	0	0	0	0	0
0	0	0	0	0	0	0	0	0	0	0	0	0	0	0	0	0	0	0
0	0	0	0	0	0	0	0	0	0	0	0	0	0	0	0	0	0	0
0	0	0	0	0	0	0	0	0	0	0	0	0	0	0	0	0	0	0
0	0	0	0	0	0	0	0	0	0	0	0	0	0	0	0	0	0	0
0	0	0	0	0	0	0	0	0	0	0	0	0	0	0	0	0	0	0
0	0	0	0	0	0	0	0	0	0	0	0	5	5	5	0	0	0	0
0	0	0	0	0	0	0	0	0	0	0	0	0	0	0	0	0	0	0
0	0	0	0	0	0	0	0	0	0	0	0	0	0	0	0	0	0	0
0	0	0	0	0	0	2	0	0	0	0	0	0	0	0	0	0	0	0
0	0	0	0	0	0	0	0	0	0	0	0	0	0	0	0	0	0	0
0	0	0	0	0	0	0	0	0	0	0	0	0	0	0	0	0	0	0
0	0	0	0	0	0	0	0	0	0	0	0	0	0	0	0	0	0	0
0	0	0	0	0	0	0	0	0	0	0	0	0	0	0	0	0	0	0
0	0	0	0	0	0	0	0	0	0	0	0	0	0	0	0	0	0	0
0	0	0	0	0	0	0	0	0	0	0	0	0	0	0	0	0	0	0
0	0	0	0	0	0	0	0	0	0	0	0	0	0	0	0	0	0	0
0	0	0	0	0	0	0	0	0	0	0	0	0	0	0	0	0	0	0
0	0	0	0	0	0	0	0	0	0	0	0	0	0	0	0	0	0	0
0	0	0	0	0	0	0	0	0	0	0	0	0	0	0	0	0	0	0
0	0	4	4	0	0	0	0	0	0	0	0	0	0	0	0	0	0	0
0	0	0	0	0	0	0	0	0	0	0	0	0	0	0	0	0	0	0
0	0	0	0	0	0	0	0	0	0	0	0	0	0	0	0	0	0	0
0	0	0	0	0	0	0	0	0	0	0	0	0	0	0	0	0	0	0

0	0	0	0	0	0	0	0	0	0	0	0	0	0	0	0	0	0	0
0	0	0	0	0	0	0	0	0	0	0	0	0	0	0	0	0	0	0
0	0	0	0	0	0	0	0	0	0	0	0	0	0	0	0	0	0	0
0	0	0	0	0	0	0	0	0	0	0	0	0	0	0	0	0	0	0
0	0	0	0	0	0	0	0	0	0	0	0	0	0	0	0	0	0	0
0	0	0	0	0	0	0	0	0	0	0	0	0	0	0	0	0	0	0
0	0	0	0	0	0	0	0	0	0	0	0	0	0	0	0	0	0	0
0	0	0	0	0	0	0	0	0	0	0	0	0	0	0	0	0	0	0
0	0	0	0	0	0	0	0	0	0	0	0	0	0	0	0	0	0	0
0	0	0	0	0	0	0	0	0	0	0	0	0	0	0	0	0	0	0
0	0	0	0	0	0	0	0	0	0	0	0	0	0	0	0	0	0	0
0	0	0	0	0	0	0	0	0	0	0	0	0	0	0	0	0	0	0
0	0	0	0	0	0	0	0	0	0	0	0	0	0	0	0	0	0	3
3	3	3	3	3	0	0	0	0	0	0	0	0	0	0	0	0	0	0
0	0	0	0	0	0	0	0	0	0	0	0	0	0	0	0	0	0	0
0	0	0	0	0	0	0	0	0	0	0	0	0	0	0	0	0	0	0
0	0	0	0	0	0	0	0	0	0	0	0	0	0	0	0	0	0	0
0	0	0	0	0	0	0	0	0	0	0	0	0	0	0	0	0	0	0
0	0	0	0	0	0	0	0	0	0	0	0	0	0	0	0	0	0	0
0	0	0	0	0	0	0	0	0	0	0	0	0	0	0	0	0	0	0
0	0	0	0	0	0	0	0	0	0	0	0	0	0	0	0	0	0	0
0	0	0	0	0	0	0	0	0	0	0	0	0	0	0	0	0	0	0
0	0	0	0	0	0	0	0	0	0	0	0	0	0	0	0	0	0	0
0	0	0	0	0	0	0	0	0	0	0	0	0	0	0	0	0	0	0
0	0	0	0	0	0	0	0	0	0	0	0	0	0	0	0	0	0	0
0	0	0	0	0	0	0	0	0	0	0	0	0	0	0	0	0	0	0
0	0	0	0	0	0	0	0	0	0	0	0	0	0	0	0	0	0	0
0	0	0	0	0	0	0	0	0	0	0	0	0	0	0	0	0	0	0
0	0	0	0	0	0	0	0	0	0	0	0	0	0	0	0	0	0	0
0	0	0	0	0	0	0	0	0	0	0	0	0	0	0	0	0	0	0
0	0	0	0	0	0	0	0	0	0	0	0	0	0	0	0	0	0	0
0	0	0	0	0	0	0	0	0	0	0	0	0	0	0	0	0	0	0
0	0	0	0	0	0	0	0	0	0	0	0	0	0	0	0	0	0	0
0	0	0	0	0	0	0	0	0	0	0	0	0	0	0	0	0	0	0
0	0	0	0	0	0	0	0	0	0	0	0	0	0	0	0	0	0	0
0	0	0	0	0	0	0	0	0	0	0	0	0	0	0	0	0	0	0
0	0	0	0	0	0	0	0	0	0	0	0	0	0	0	0	0	0	0

0	0	0	0	0	0	0	0	0	0	0	0	0	0	0	0	0	0	0
0	0	0	0	0	0	0	0	0	0	0	0	0	0	0	0	0	0	0
0	0	0	0	0	0	0	2	2	2	2	2	2	2	2	2	2	0	0
0	0	0	0	0	0	0	0	0	0	0	0	0	0	0	0	0	0	0
0	0	0	0	0	0	0	0	0	0	0	0	0	0	0	0	0	0	0
0	0	0	0	0	0	0	0	0	0	0	0	0	0	0	0	0	0	0
0	0	0	0	0	0	0	0	0	0	0	0	0	0	0	0	0	0	0
0	0	0	0	0	0	0	0	0	0	0	0	0	0	0	0	0	0	0
0	0	0	0	0	0	0	0	0	0	0	0	0	0	0	0	0	0	0
0	0	0	0	0	0	0	0	1	0									
Jan_DL123	1324	1028	485	327	454	405	126	134	258	303	107	240	244	280	212	216	210	142
302	206	137	360	132	109	121	93	140	109	135	0	111	61	121	0	102	320	0
115	25	43	89	120	0	144	113	50	101	73	65	39	128	33	41	42	88	0
0	0	0	23	66	48	81	61	45	162	83	0	7	90	38	0	23	17	0
43	43	21	31	0	32	58	17	0	80	0	0	175	0	30	0	31	0	0
42	42	50	0	0	0	0	60	0	20	0	5	0	0	45	16	0	0	0
0	107	14	20	0	0	0	0	0	0	8	21	13	0	0	0	0	0	0
0	0	0	0	0	0	0	0	54	20	0	0	5	0	0	0	0	71	0
0	16	0	0	0	17	0	0	9	0	22	0	0	0	0	0	8	0	13
31	0	12	0	68	29	0	0	0	42	0	0	0	0	0	14	14	87	0
0	0	0	0	0	0	0	0	0	28	0	0	0	0	0	0	0	45	0
0	0	0	0	0	0	0	0	0	0	0	16	0	0	0	0	0	22	0
0	0	0	0	0	0	0	0	7	0	0	65	0	10	0	0	0	0	0
0	0	0	0	0	0	0	0	0	0	0	0	0	0	0	0	0	0	13
0	0	0	0	0	0	0	0	10	0	28	0	0	0	0	11	0	0	0
0	0	0	54	0	0	0	0	0	0	0	0	0	0	0	6	0	0	0
0	6	6	0	0	0	0	0	0	0	0	0	0	0	0	0	0	0	19
0	0	0	0	0	0	0	11	0	0	0	0	0	0	9	0	0	0	0
0	0	0	0	0	2	0	15	0	0	0	10	0	12	0	0	0	0	0
0	0	0	0	0	0	0	0	0	0	0	0	0	0	0	0	0	0	10
0	0	27	0	17	0	0	0	0	0	21	0	7	0	0	0	0	0	0
0	0	0	0	0	0	0	8	0	0	0	0	0	0	0	0	0	0	0
0	0	0	0	0	0	0	0	0	0	0	0	12	0	10	0	7	0	0
0	0	0	7	0	0	0	0	0	0	0	0	0	0	0	0	0	0	10
0	0	0	0	0	0	0	0	0	0	0	0	0	0	0	0	0	0	0
0	0	38	0	2	0	20	0	0	0	0	0	0	0	0	0	0	0	0
15	0	0	0	0	0	0	0	0	0	0	0	0	0	0	0	0	0	0

0	0	8	0	0	0	0	0	0	0	0	0	0	0	0	32	0	0	0
0	0	0	0	0	0	0	0	9	0	0	0	0	0	0	0	0	0	0
0	0	0	0	0	0	0	0	0	0	11	0	0	0	0	0	0	0	0
12	0	0	0	0	0	0	0	0	0	0	0	0	0	9	0	0	0	0
0	0	0	0	0	0	0	0	0	0	0	0	0	0	0	5	0	0	0
0	0	0	5	0	0	0	0	0	0	18	0	0	0	0	0	0	0	0
0	0	0	0	0	0	0	0	0	0	0	0	0	0	0	0	0	0	0
0	0	0	0	14	0	0	0	0	0	0	0	0	0	0	0	0	0	0
7	0	0	0	0	0	0	0	0	0	0	0	0	0	0	0	0	0	0
0	0	0	0	0	0	4	0	0	0	0	0	0	0	0	0	0	0	0
0	0	0	0	0	0	0	0	0	0	0	0	0	0	0	0	0	0	0
0	0	4	0	0	0	0	0	0	0	0	0	0	0	0	0	0	0	12
8	0	0	0	0	0	0	0	0	0	0	0	0	0	0	0	0	0	0
0	0	0	0	0	0	0	0	0	0	0	0	0	0	0	0	0	0	0
12	0	0	0	0	0	0	0	0	0	0	0	0	0	13	0	0	0	0
0	0	0	0	0	0	0	0	0	0	0	0	0	9	0	0	12	0	0
0	0	0	0	0	0	0	0	0	0	0	0	0	0	0	0	0	0	0
0	0	0	0	0	0	0	0	0	0	0	0	0	0	0	0	0	0	0
0	0	0	0	0	0	0	0	0	0	0	0	0	0	0	0	0	0	0
0	0	0	0	0	0	0	0	0	0	0	0	0	40	0	0	0	0	0
0	0	0	0	0	0	0	0	0	0	0	0	0	0	0	0	0	0	0
0	0	0	0	0	14	0	0	0	0	0	0	0	0	0	0	0	0	0
0	0	0	0	0	0	0	0	0	0	0	0	0	0	0	0	0	0	0
0	0	0	0	0	19	0	0	0	0	0	0	0	0	0	0	0	0	0
0	0	0	0	0	0	11	0	0	0	0	0	11	0	0	0	0	0	0
0	0	28	0	0	0	0	0	0	0	0	0	0	0	0	0	0	0	0
0	0	0	0	0	0	0	0	0	0	0	0	0	0	15	0	0	0	0
0	0	0	0	0	0	0	0	0	0	0	0	0	0	0	0	0	0	0
0	0	0	0	0	0	0	0	0	0	0	0	0	0	0	0	0	0	0
0	0	0	0	0	0	0	0	0	0	0	0	0	0	0	0	0	0	0
0	0	0	0	0	0	0	0	0	0	0	0	0	0	0	20	0	0	0
0	0	0	0	0	0	0	19	0	0	0	0	0	0	0	0	0	0	0
0	0	0	0	0	0	0	0	0	0	0	0	0	0	0	0	0	0	0
0	0	0	0	0	0	0	0	0	0	0	0	0	0	0	0	0	0	0
0	0	0	0	0	0	0	0	0	0	0	0	0	0	0	0	0	0	0
0	0	0	0	0	0	0	0	0	0	0	0	0	0	0	0	0	0	0
0	0	0	0	0	0	0	0	0	0	0	0	0	0	0	0	0	0	0

0	0	0	0	0	0	0	0	0	0	0	0	0	0	0	0	0	0	0
0	0	0	0	0	0	0	0	0	0	0	0	0	0	0	0	0	0	0
0	0	0	0	0	0	0	0	0	0	0	0	0	0	0	0	0	0	0
0	0	0	0	0	0	0	0	0	0	0	0	0	0	0	0	0	0	0
0	0	0	0	0	0	0	0	0	0	0	0	0	0	0	0	0	0	0
0	0	0	0	0	0	0	0	0	0	0	0	0	0	0	0	0	0	0
0	11	0	0	0	0	0	0	0	0	0	0	0	6	0	0	0	0	0
0	0	0	0	0	0	0	0	0	0	0	0	0	0	0	0	0	0	0
0	0	0	0	0	0	0	0	0	0	0	0	0	0	0	0	0	0	0
0	0	0	0	0	0	0	0	0	0	0	0	0	0	0	0	0	0	0
0	0	0	0	0	0	0	0	0	0	0	0	0	0	0	0	0	0	0
9	0	0	0	0	0	0	0	0	0	0	0	0	0	0	0	0	0	0
0	0	0	0	0	0	0	0	0	0	0	0	0	0	0	0	0	0	0
0	0	0	0	0	0	0	0	0	0	0	0	0	0	0	0	3	0	0
0	0	0	0	0	0	0	0	0	0	0	0	8	8	8	0	0	0	0
0	0	0	0	0	0	0	0	0	0	0	0	0	0	0	0	0	0	0
0	0	0	0	0	0	0	0	0	0	0	0	0	0	0	0	0	0	0
0	0	0	0	0	0	0	0	0	0	0	0	0	0	0	0	0	0	0
0	0	0	0	0	0	0	0	0	0	0	0	0	0	0	0	0	0	0
0	0	0	0	0	0	0	0	0	0	0	0	0	0	0	0	0	0	0
0	0	0	0	0	0	0	0	0	0	0	0	0	0	0	0	0	0	0
0	0	0	0	0	0	0	0	0	0	0	0	0	0	0	0	0	0	0
0	0	0	0	0	0	0	0	0	0	0	0	0	0	0	0	0	0	0
0	0	0	0	0	0	0	0	0	0	0	0	0	0	0	0	0	0	0
0	0	0	0	0	0	0	0	0	0	0	0	0	0	0	0	0	0	6
6	6	6	0	0	0	0	0	0	0	0	0	0	0	0	0	0	0	0
0	0	0	0	0	0	0	0	0	0	0	0	0	0	0	0	0	0	0
0	0	0	0	0	0	0	0	0	0	0	0	0	0	0	0	0	0	0
0	0	0	0	0	0	0	0	0	0	0	0	0	0	0	0	0	0	0
0	0	0	0	0	0	0	0	0	0	0	0	0	0	0	0	0	0	0
0	0	0	0	0	0	0	0	0	0	0	0	0	0	0	0	0	0	0
0	0	0	0	0	0	0	0	0	0	0	0	0	0	0	0	0	0	0
0	0	0	0	0	0	0	0	0	0	0	0	0	0	5	5	5	5	5
0	0	0	0	0	0	0	0	0	0	0	0	0	0	0	0	0	0	0
0	0	0	0	0	0	0	0	0	0	0	0	0	0	0	0	0	0	0
0	0	0	0	0	0	0	0	0	0	0	0	0	0	0	0	0	0	0
0	0	0	0	0	0	0	0	0	0	0	0	0	0	0	0	0	0	0

0	0	0	0	0	0	0	0	0	0	0	0	0	0	0	0	0	0	0
0	0	0	0	0	0	0	0	0	0	0	0	0	0	0	0	0	0	0
0	0	0	0	0	0	0	0	0	0	0	0	0	0	0	0	0	0	0
0	0	0	0	0	0	0	0	0	0	0	0	0	0	0	0	0	0	0
0	0	0	0	0	0	0	0	0	0	0	0	0	0	0	0	0	0	0
0	0	0	0	0	0	0	0	0	0	0	0	0	0	0	4	0	0	0
0	0	0	0	0	0	0	0	0	0	0	0	0	0	0	0	0	0	0
0	0	0	0	0	0	0	0	0	0	0	0	0	0	0	0	0	0	0
0	0	0	0	0	0	0	0	0	0	0	0	0	0	0	0	0	0	0
0	0	0	0	0	0	0	0	0	0	0	0	0	0	0	0	0	0	0
0	0	0	0	0	0	0	0	0	0	0	0	0	0	0	0	0	0	0
0	0	0	0	0	0	0	0	0	0	0	0	0	0	0	0	0	0	0
0	0	0	0	0	0	0	0	0	0	0	0	0	0	0	0	0	0	0
0	0	0	0	0	0	0	0	0	0	0	0	0	0	0	0	0	0	0
0	0	0	0	0	0	0	0	0	0	0	0	0	0	0	0	0	0	0
0	0	0	0	0	0	0	0	0	0	0	0	0	0	0	0	0	0	0
0	0	0	0	0	0	0	0	0	0	0	0	0	0	0	0	0	0	0
0	0	0	0	0	0	0	0	0	0	0	0	0	0	0	0	0	0	0
0	0	0	0	0	0	0	0	0	0	0	0	0	0	0	0	0	0	0
0	0	0	0	0	0	0	0	0	0	0	0	0	0	0	0	0	0	3
3	3	3	3	3	3	3	3	3	0	0	0	0	0	0	0	0	0	0
0	0	0	0	0	0	0	0	0	0	0	0	0	0	0	0	0	0	0
0	0	0	0	0	0	0	0	0	0	0	0	0	0	0	0	0	0	0
0	0	0	0	0	0	0	0	0	0	0	0	0	0	0	0	0	0	0
0	0	0	0	0	0	0	0	0	0	0	0	0	0	0	0	0	0	0
0	0	0	0	0	0	0	0	0	0	0	0	0	0	0	0	0	0	0
0	0	0	0	0	0	0	0	0	0	0	0	0	0	0	0	0	0	0
0	0	0	0	0	0	0	0	0	0	0	0	0	0	0	0	0	0	0
0	0	0	0	0	0	0	0	0	0	0	0	0	0	0	0	0	0	0
0	0	0	0	0	0	0	0	0	0	0	0	0	0	0	0	0	0	0
0	0	0	0	0	0	0	0	0	0	0	0	0	0	0	0	0	0	0
0	0	0	0	0	0	0	0	0	0	0	0	0	0	0	0	0	0	0
0	0	0	0	0	0	0	0	0	0	0	0	0	0	0	0	0	0	0
0	0	0	0	0	0	0	0	0	0	0	0	0	0	0	0	0	0	0
0	0	0	0	0	0	0	0	0	0	0	0	0	0	0	0	0	0	0
0	0	0	0	0	0	0	0	0	0	0	0	0	0	0	0	0	0	0
0	0	0	0	0	0	0	0	0	0	0	0	0	0	0	0	0	0	0

0	0	0	0	0	0	0	0	0	0	0	0	0	0	0	0	0	0	0
0	0	0	0	0	0	0	0	0	0	0	0	0	0	0	0	0	0	0
0	0	0	0	0	0	0	0	0	0	0	0	0	0	0	0	0	0	0
0	0	0	0	0	0	0	0	0	0	0	0	0	0	0	0	0	0	0
0	0	0	0	0	0	0	0	0	0	0	0	0	0	0	0	0	0	0
0	0	0	0	0	0	0	0	0	0	0	0	0	0	0	0	0	0	0
0	0	0	0	0	0	0	0	0	0	0	0	0	0	0	0	0	0	0
0	0	0	0	0	0	0	0	0	0	0	0	0	0	0	0	0	0	0
0	0	0	0	0	0	0	0	0	0	0	0	0	0	0	0	0	0	0
0	0	0	2	2	2	2	2	2	2	2	2	0	0	0	0	0	0	0
0	0	0	0	0	0	0	0	0	0	0	0	0	0	0	0	0	0	0
0	0	0	0	0	0	0	0	0	0	0	0	0	0	0	0	0	0	0
0	0	0	0	0	0	0	0	0	0	0	0	0	0	0	0	0	0	0
0	0	0	0	0	0	0	0	0	0	0	0	0	0	0	0	0	0	0
0	0	0	0	0	0	0	0	0	0	0	0	0	0	0	0	0	0	0
0	0	0	0	0	0	0	0	0	0	0	0	0	0	0	0	0	0	0
0	0	0	0	0	0	0	0	0	0	0	0	0	0	0	0	0	0	0
0	0	0	0	0	0	0	0	0	0	0	0	0	0	0	0	0	0	0
0	0	0	0	0	0	0	0	0	0	0	0	0	0	0	0	0	0	0
0	0	0	0	0	0	0	0	0	0	0	0	0	0	0	0	0	0	0
0	0	0	0	0	0	0	0	0	0	0	0	0	0	0	0	0	0	0
0	0	0	0	0	0	0	0	0	0	0	0	0	0	0	0	0	0	0
0	0	0	0	0	0	0	0	0	0	0	0	0	0	0	0	0	0	0
0	0	0	0	0	0	0	0	0	0	0	0	0	0	0	0	0	0	0
0	0	0	0	0	0	0	0	0	0	0	0	0	0	0	0	0	0	0
0	0	0	0	0	0	0	0	0	0	0	0	0	0	0	0	0	0	0
0	0	0	0	0	0	0	0	0	0	0	0	0	0	0	0	0	0	0
0	0	0	0	0	0	0	0	0	0	0	0	0	0	0	0	0	0	0
0	0	0	0	0	0	0	0	0	0	0	0	0	0	0	0	0	0	0
0	0	0	0	0	0	0	0	0	0	0	0	0	0	0	0	0	0	0
0	0	0	0	0	0	0	0	0	0	0	0	0	0	0	0	0	0	0
0	0	0	0	0	0	0	0	0	0	0	0	0	0	0	0	0	0	0
0	0	0	0	0	0	0	0	0	0	0	0	0	0	0	0	0	0	0
0	0	0	0	0	0	0	0	0	0	0	0	0	0	0	0	0	0	0
0	0	0	0	0	0	0	0	0	0	0	0	0	0	0	0	0	0	0
0	0	0	0	0	0	0	0	0	0	0	0	0	0	0	0	0	0	0
0	0	0	0	0	0	0	0	0	0	0	0	0	0	0	0	0	0	0
0	0	0	0	0	0	0	0	0	0	0	0	0	0	0	0	0	0	0

0	0	0	0	0	0	0	0	0	0	0	0	0	0	0	0	0	0	0
0	0	0	0	0	0	0	0	0	0									
Jan_DL124	1159	1572	1311	869	111	120	136	127	62	482	422	210	436	384	296	111	130	119
199	597	179	984	183	145	332	324	257	198	143	0	158	249	210	233	225	109	30
180	21	0	18	146	157	76	0	76	32	90	197	22	95	0	204	49	69	0
0	491	184	140	62	41	68	109	99	79	8	0	7	77	135	0	14	102	28
84	0	0	73	25	68	27	6	86	47	0	91	0	12	0	0	42	0	173
0	75	59	0	58	0	102	0	50	38	0	0	0	0	50	67	0	91	31
0	63	0	89	53	44	0	0	0	0	11	66	0	5	116	0	0	0	0
0	0	0	0	114	0	0	0	0	7	0	0	14	0	44	11	0	0	0
0	0	138	0	0	6	0	43	37	30	0	0	22	96	0	0	0	28	46
0	0	27	114	0	141	0	0	0	0	0	32	0	33	0	0	42	0	0
0	0	6	62	51	26	0	0	79	0	0	0	0	0	38	46	0	0	0
0	0	0	0	0	0	31	0	0	38	0	0	0	0	0	0	0	0	0
0	27	22	27	0	14	0	0	0	0	0	25	0	20	17	23	0	0	0
7	0	0	0	38	0	0	0	0	42	0	44	9	0	0	0	0	0	0
0	0	0	13	0	0	0	0	15	0	9	0	0	12	11	14	0	0	0
0	0	20	0	0	0	0	51	0	0	0	35	18	0	18	0	0	0	27
0	0	13	0	0	0	0	0	0	10	10	0	7	13	0	12	11	12	12
8	0	0	0	12	61	0	17	0	5	0	0	0	0	0	0	41	0	7
0	7	0	0	0	0	0	0	25	15	0	0	0	0	21	0	13	0	0
0	0	0	0	0	0	5	31	0	0	0	0	0	0	0	0	0	0	0
16	23	0	0	0	6	10	0	0	0	0	0	0	0	7	0	0	0	0
0	0	0	0	18	0	0	0	0	0	0	0	0	0	30	0	0	0	0
0	0	0	0	0	0	0	0	0	0	63	3	0	0	0	0	8	0	0
0	0	0	0	0	0	0	0	0	0	0	0	0	0	0	0	0	0	0
0	0	0	0	0	0	0	9	0	0	0	0	0	0	0	0	0	0	0
0	0	0	0	0	0	0	0	0	19	0	0	0	0	0	0	0	0	0
0	0	19	0	0	0	0	0	0	8	0	0	0	0	0	0	0	14	0
0	0	0	0	0	0	0	12	0	0	0	0	0	0	0	0	0	9	0
15	0	0	0	0	0	0	0	39	0	0	0	0	0	0	0	0	0	0
0	0	0	0	0	0	0	0	0	0	5	0	0	0	0	0	86	0	0
17	3	0	6	0	0	0	0	0	0	0	0	0	0	0	0	0	0	0
0	25	0	0	0	0	24	0	0	0	0	0	0	0	0	15	0	0	0
0	0	0	0	0	0	0	0	0	0	0	0	0	5	0	0	0	0	0
0	0	0	0	0	0	0	0	0	0	0	0	0	0	0	17	0	0	0
0	0	0	0	0	0	0	0	0	0	0	4	10	0	0	0	0	4	0

0	0	0	0	0	0	14	0	0	0	0	0	0	0	0	0	23	0	0
0	0	0	0	10	0	0	0	0	0	0	0	0	0	54	0	0	0	0
0	0	0	0	0	0	0	0	0	0	0	0	0	0	0	0	0	0	0
0	0	0	0	0	0	0	0	0	0	50	0	0	0	3	0	0	0	0
0	6	0	0	0	0	8	0	0	0	0	0	0	0	0	0	0	0	0
0	0	0	0	0	0	0	0	0	0	0	0	0	0	0	0	0	0	0
0	0	0	0	0	6	0	0	0	0	0	0	0	8	0	0	10	0	0
0	0	0	0	0	0	0	0	0	0	7	0	0	0	0	0	0	0	0
0	0	0	0	0	0	0	0	0	0	0	0	0	0	0	0	0	0	0
0	0	0	0	0	0	0	0	0	0	0	0	0	0	0	0	0	0	0
0	0	0	0	0	0	0	0	7	0	0	0	0	0	0	0	0	0	0
5	0	0	0	0	0	0	0	0	0	0	0	0	0	0	0	0	0	0
0	0	0	0	0	0	0	0	0	0	0	0	0	0	0	0	0	0	0
0	0	0	0	0	0	0	0	0	0	0	0	0	0	0	0	0	0	0
0	0	0	16	0	0	0	0	0	0	0	0	0	0	0	0	0	0	0
0	0	0	0	0	0	0	0	0	0	0	0	0	0	0	0	0	0	0
0	0	0	0	0	0	0	0	0	0	0	0	0	0	0	0	0	0	0
0	0	0	0	24	0	0	8	0	0	0	0	0	0	0	0	0	0	0
0	0	0	0	0	0	0	0	0	0	0	0	0	0	0	0	0	0	0
0	0	0	0	0	0	0	0	0	9	0	0	0	0	0	0	0	0	0
0	0	0	0	0	0	0	0	0	0	0	0	0	0	0	0	0	0	0
0	0	0	0	0	0	0	0	0	0	0	0	0	0	0	0	0	0	0
0	0	0	11	0	0	0	0	0	0	0	0	0	0	0	0	0	0	0
0	0	0	0	0	0	0	0	0	0	0	0	0	0	0	0	0	0	0
0	0	0	7	0	0	0	0	0	0	0	0	0	0	0	0	0	0	0
0	0	0	0	0	0	0	0	0	0	0	0	0	0	0	0	0	0	0
0	0	0	0	0	0	0	0	0	0	0	0	0	0	0	0	0	0	0
0	0	0	0	0	0	0	0	0	0	0	0	0	0	0	0	0	0	0
0	0	0	0	0	0	0	0	0	0	0	0	0	0	0	0	0	0	0
0	0	0	0	0	0	0	0	0	0	0	0	0	0	0	0	0	0	0
0	0	0	0	0	0	0	0	0	0	0	0	0	0	0	0	0	0	7
0	0	0	0	0	0	0	0	0	0	0	0	0	0	0	0	0	0	0
0	0	0	0	0	0	0	0	0	0	0	0	0	0	0	0	0	0	0
0	0	0	0	0	0	0	0	0	0	0	0	0	0	0	0	0	0	0
0	0	0	0	0	0	0	0	0	0	0	0	4	0	0	0	0	0	0
0	0	0	0	0	0	0	0	0	0	0	0	0	0	0	0	0	0	0
0	0	0	0	0	0	0	0	0	0	0	0	0	0	0	0	0	0	0

0	0	0	0	0	0	0	0	0	0	0	0	0	0	0	0	0	0	0
0	0	0	0	0	0	0	0	4	0	0	0	0	0	0	0	0	0	0
0	0	0	0	0	0	0	0	0	0	0	0	0	0	0	0	0	0	0
0	0	0	0	0	0	0	0	0	0	0	0	0	0	0	0	0	0	0
0	0	0	0	0	0	0	0	0	0	0	0	0	0	0	0	0	0	0
0	0	0	0	0	0	0	0	0	0	0	0	0	0	0	0	0	0	0
0	0	0	0	0	0	0	0	0	0	0	0	0	0	0	0	0	0	0
0	0	0	0	0	0	0	12	0	0	0	0	0	0	0	0	0	0	0
0	0	0	0	0	0	0	0	0	0	0	0	0	0	0	0	0	0	0
0	0	0	0	0	0	0	0	0	0	0	11	11	0	0	0	0	0	0
0	0	0	0	0	0	0	0	0	0	0	0	0	0	0	0	0	0	0
0	0	0	0	0	0	0	0	0	0	0	0	0	0	0	0	0	0	0
0	0	0	0	6	0	0	0	0	0	0	0	0	0	0	0	0	0	0
0	0	0	0	0	0	0	0	0	0	0	0	0	0	0	0	0	0	0
0	0	0	0	0	0	0	0	0	0	0	0	0	0	0	0	0	0	0
0	0	0	0	0	0	0	0	0	0	0	0	0	0	0	0	0	0	0
0	0	0	0	0	0	0	0	0	0	0	0	0	0	0	0	0	0	0
0	0	0	0	0	0	0	0	0	0	0	0	0	0	0	0	0	5	0
0	0	0	0	0	0	0	0	0	0	0	0	0	0	0	0	0	0	0
0	0	0	0	0	0	0	0	0	0	0	0	0	0	0	0	0	0	0
0	0	0	0	0	0	0	0	0	0	0	0	0	0	0	0	0	0	0
0	0	0	0	0	0	0	0	0	0	0	0	0	0	0	8	8	0	0
0	0	0	0	0	0	0	0	0	0	0	0	0	0	0	0	0	0	0
0	0	0	0	0	0	0	0	0	0	0	0	0	0	0	0	0	0	0
0	0	0	0	0	0	0	0	0	0	0	0	0	0	0	0	0	0	0
0	0	0	0	0	0	0	0	0	0	0	0	0	0	0	0	0	0	0
0	0	0	0	0	0	0	0	0	0	0	0	0	0	0	0	0	0	0
0	0	0	0	0	0	0	0	0	0	0	0	0	0	0	0	0	0	0
0	0	0	0	0	0	0	0	0	0	0	7	0	0	0	0	0	0	0
0	0	0	0	0	0	0	0	0	0	0	0	0	0	0	0	0	0	0
0	0	0	0	0	0	0	0	0	0	0	0	0	0	0	0	0	0	0
0	0	0	0	0	0	0	0	0	0	0	0	0	0	0	0	0	0	0
0	0	0	0	0	0	0	0	0	0	0	0	0	0	0	0	0	0	0
0	0	0	0	0	0	0	0	0	0	0	0	0	0	0	0	0	0	0
0	0	0	0	0	0	0	0	0	0	0	0	0	0	0	0	0	0	0
0	0	0	0	0	0	6	0	0	0	0	0	0	0	0	0	0	0	0
0	0	0	0	0	0	0	0	0	0	0	0	0	0	0	0	0	0	0

0	0	0	0	0	0	0	0	0	0	0	0	0	0	0	0	0	0	0
0	0	0	0	0	0	0	0	0	0	0	0	0	0	0	0	0	0	0
0	0	0	0	0	0	0	0	0	0	0	0	0	0	0	0	0	0	0
0	0	0	0	0	0	0	0	0	0	0	0	0	0	0	0	0	0	0
0	0	0	0	0	0	0	0	0	0	0	0	0	0	0	0	0	0	0
0	0	0	0	0	0	0	0	0	0	0	0	0	0	0	0	0	0	0
0	0	0	0	0	0	3	0	0	0	0	0	0	0	0	0	0	0	0
0	0	0	0	0	0	0	0	0	0	0	0	0	0	0	0	0	0	0
0	0	0	0	0	0	0	0	0	0	0	0	0	0	0	0	0	0	0
0	0	0	0	0	0	0	0	0	0	0	0	0	0	0	0	0	0	0
0	0	0	0	0	0	0	0	0	0	0	0	0	0	0	0	0	0	0
0	0	0	0	0	0	0	0	0	0	0	0	0	0	0	0	0	0	0
0	0	0	0	0	0	0	0	0	0	0	0	0	0	0	0	0	0	0
0	0	0	0	0	0	0	0	0	0	0	0	0	0	0	0	0	0	0
0	0	0	0	0	0	0	0	0	0	0	0	0	0	0	0	0	0	0
0	0	0	0	0	0	0	0	0	0	0	0	0	0	0	0	0	0	0
0	0	0	0	0	0	0	0	0	0	0	0	0	0	0	0	0	0	0
0	0	0	0	0	0	0	0	0	0	0	0	0	0	0	0	0	0	0
0	0	0	0	0	0	0	0	0	0	0	0	0	0	0	0	0	0	0
0	0	0	0	0	0	0	0	0	0	0	0	0	0	0	0	0	0	0
0	4	4	0	0	0	0	0	0	0	0	0	0	0	0	0	0	0	0
0	0	0	0	0	0	0	0	0	0	0	0	0	0	0	0	0	0	0
0	0	0	0	0	0	0	0	0	0	0	0	0	0	0	0	0	0	0
0	0	0	0	0	0	0	0	0	0	0	0	0	0	0	0	0	0	0
0	0	0	0	0	0	0	0	0	0	0	0	0	0	0	0	0	0	0
0	0	0	0	0	0	0	0	0	0	0	0	0	0	0	0	0	0	0
0	0	0	0	0	0	0	0	0	0	0	0	0	0	0	0	0	0	0
0	0	0	0	0	0	0	0	0	0	0	0	0	0	0	0	0	0	0
0	0	0	0	0	0	0	0	0	0	0	0	0	0	0	0	0	0	0
0	0	0	0	0	0	0	0	0	0	0	0	0	0	0	0	0	0	0
0	0	0	0	0	0	0	0	0	0	0	0	0	0	0	0	0	0	0
0	0	0	0	0	0	0	0	0	0	0	0	0	0	0	0	0	0	0
0	0	0	0	0	0	0	0	0	0	0	0	0	0	0	0	0	0	0
0	0	0	0	0	0	0	0	0	0	0	0	0	0	0	0	0	0	0
0	0	0	0	0	0	0	0	0	0	0	0	0	0	0	0	0	0	0
0	0	0	0	0	0	0	0	0	0	0	0	0	0	0	0	0	0	0
0	0	0	0	0	0	0	0	0	0	0	0	0	0	0	0	0	0	0

0	0	0	0	0	0	0	0	0	0	0	0	0	0	0	0	0	0	0
0	0	0	0	0	0	0	0	0	0	0	0	0	0	0	0	0	0	0
0	0	0	0	0	0	0	0	0	0	0	0	0	0	0	3	3	3	3
3	3	3	3	0	0	0	0	0	0	0	0	0	0	0	0	0	0	0
0	0	0	0	0	0	0	0	0	0	0	0	0	0	0	0	0	0	0
0	0	0	0	0	0	0	0	0	0	0	0	0	0	0	0	0	0	0
0	0	0	0	0	0	0	0	0	0	0	0	0	0	0	0	0	0	0
0	0	0	0	0	0	0	0	0	0	0	0	0	0	0	0	0	0	0
0	0	0	0	0	0	0	0	0	0	0	0	0	0	0	0	0	0	0
0	0	0	0	0	0	0	0	0	0	0	0	0	0	0	0	0	0	0
0	0	0	0	0	0	0	0	0	0	0	0	0	0	0	0	0	0	0
0	0	0	0	0	0	0	0	0	0	0	0	0	0	0	0	0	0	0
0	0	0	0	0	0	0	0	0	0	0	0	0	0	0	0	0	0	0
0	0	0	0	0	0	0	0	0	0	0	0	0	0	0	0	0	0	0
0	0	0	0	0	0	0	0	0	0	0	0	0	0	0	0	0	0	0
0	0	0	0	0	0	0	0	0	0	0	0	0	0	0	0	0	0	0
0	0	0	0	0	0	0	0	0	0	0	0	0	0	0	0	0	0	0
0	0	0	0	0	0	0	0	0	0	0	0	0	0	0	0	0	0	0
0	0	0	0	0	0	0	0	0	0	0	0	0	0	0	0	0	0	0
0	0	0	0	0	0	0	0	0	0	0	0	0	0	0	0	0	0	0
0	0	0	0	0	0	0	0	0	0	0	0	0	0	0	0	0	0	0
0	0	0	0	0	0	0	0	0	0	0	0	0	0	0	0	0	0	0
0	0	0	0	0	0	0	0	0	0	0	0	0	0	0	0	0	0	0
0	0	0	0	0	0	0	0	0	0	0	0	0	0	0	0	0	0	0
0	0	0	0	0	0	0	0	0	2	2	2	2	2	2	2	2	0	0
0	0	0	0	0	0	0	0	0	0	0	0	0	0	0	0	0	0	0
0	0	0	0	0	0	0	0	0	0	0	0	0	0	0	0	0	0	0
0	0	0	0	0	0	0	0	0	0	0	0	0	0	0	0	0	0	0
0	0	0	0	0	0	0	0	0	0	0	0	0	0	0	0	0	0	0
0	0	0	0	0	0	0	0	0	0	0	0	0	0	0	0	0	0	0
0	0	0	0	0	0	0	0	0	0									
Jan_DL125	1882	797	604	642	180	769	548	539	227	305	185	290	240	556	151	298	530	278
182	226	61	11	114	0	59	228	54	262	118	465	172	140	120	90	204	48	349
225	56	203	22	118	77	55	15	233	123	99	71	82	102	39	68	91	98	325
247	45	67	100	93	97	41	103	33	21	27	0	0	51	0	28	22	22	69
39	79	23	65	0	43	35	0	0	55	51	0	44	18	39	0	11	18	0
68	23	48	0	0	0	0	0	0	11	0	21	0	0	15	26	28	0	0

0	42	20	23	4	0	0	0	0	0	0	33	23	10	0	0	0	0	0
0	0	0	0	0	0	0	0	11	12	0	0	8	0	0	0	0	0	0
0	0	17	0	0	12	0	0	0	0	8	0	0	0	31	0	0	51	0
0	0	10	0	0	0	0	6	0	21	43	0	26	0	0	0	6	0	35
20	0	37	0	0	0	0	0	0	0	0	0	17	0	0	0	0	0	0
0	44	0	0	33	0	0	0	0	0	0	0	0	0	0	0	0	11	0
0	0	0	0	0	5	0	0	0	0	0	0	0	15	0	22	8	0	14
0	0	0	11	0	0	0	0	0	18	0	0	0	0	2	0	0	0	0
0	51	0	0	0	0	0	0	0	0	0	2	0	5	5	0	0	0	0
19	0	4	0	0	0	0	0	0	0	0	8	0	0	7	0	0	0	0
0	4	0	0	0	0	0	0	0	0	0	0	0	0	0	0	0	0	0
0	9	6	0	0	0	0	7	16	0	0	0	0	0	0	4	0	42	0
0	3	0	0	8	16	0	0	0	6	0	0	0	0	0	0	0	0	0
0	0	3	0	0	0	0	0	0	0	0	0	0	0	16	0	0	0	0
0	0	0	0	0	0	0	0	0	0	0	0	0	0	7	0	0	0	0
0	0	19	0	0	0	0	0	0	5	17	0	0	0	0	0	0	0	0
0	0	0	0	0	0	0	0	7	0	0	90	0	0	0	0	0	0	0
0	0	0	0	0	0	0	0	0	0	0	0	0	0	0	0	0	0	0
0	0	17	0	0	0	0	0	0	0	0	9	0	0	0	0	0	0	0
0	0	0	0	0	0	0	0	0	0	0	0	0	0	0	0	0	0	0
0	0	0	0	0	0	0	0	31	2	0	0	0	0	0	0	0	6	0
0	0	0	0	0	0	0	5	0	0	0	0	0	0	0	0	0	0	0
0	0	0	0	0	0	0	0	0	0	0	0	0	0	0	0	0	0	0
0	0	0	0	0	0	0	0	0	0	0	0	0	0	0	0	0	0	0
0	0	0	0	0	0	0	0	0	0	0	0	0	0	0	0	0	0	0
0	0	0	0	0	0	0	0	0	0	0	0	0	9	0	0	0	0	0
0	6	0	0	0	0	0	27	0	0	19	0	0	0	0	17	0	0	0
0	0	0	0	0	0	0	4	0	0	0	0	0	0	0	0	0	0	0
0	3	0	5	9	0	0	0	0	0	0	0	0	0	0	0	0	5	0
0	0	6	0	0	0	0	0	0	0	0	0	0	0	0	0	0	0	0
0	45	0	0	0	0	0	0	0	0	0	0	0	0	0	0	0	0	0
0	0	0	0	0	0	0	0	0	20	0	0	0	0	0	0	0	0	0
0	0	0	0	0	0	0	0	0	0	0	0	0	0	0	0	0	0	0
0	0	0	0	0	0	0	0	0	0	0	0	0	0	0	0	0	0	0
0	0	0	0	0	0	0	0	0	0	0	0	0	0	0	0	0	0	0
0	0	0	0	0	0	0	0	0	0	0	0	0	0	0	24	22	0	0
0	0	0	0	0	0	0	0	0	0	0	0	0	0	0	0	0	0	0

0	0	0	0	0	0	0	0	0	0	0	0	0	0	0	0	0	0	0
0	0	0	0	0	0	0	0	0	0	0	0	0	0	0	0	0	0	0
0	0	0	0	0	0	0	0	0	0	0	0	0	0	0	0	0	0	0
0	0	0	0	0	0	0	0	0	0	0	0	0	0	0	0	0	0	0
0	0	0	0	0	0	0	0	0	0	0	0	0	0	0	0	0	0	0
0	0	0	0	0	0	0	0	0	0	0	0	0	0	0	0	3	0	0
0	0	0	0	0	0	0	0	0	0	0	0	0	0	0	0	0	0	0
0	0	6	0	0	0	0	0	0	0	0	0	0	0	0	0	0	0	0
0	0	0	0	0	0	0	0	0	0	0	0	0	0	0	0	0	0	0
0	0	0	0	0	0	0	0	0	0	0	0	0	0	0	0	0	0	0
0	0	0	0	0	0	0	0	0	0	0	0	0	0	0	0	0	0	0
0	0	0	0	0	0	0	0	0	0	0	0	0	0	0	0	0	0	0
0	0	0	0	0	0	0	0	0	0	0	0	0	0	0	0	0	0	0
0	0	0	0	0	0	0	0	0	0	0	0	0	0	0	0	0	0	0
0	0	0	0	0	0	0	0	0	0	0	0	0	0	0	0	0	0	0
0	0	0	0	0	0	0	0	0	7	0	0	0	0	0	0	0	0	0
0	0	0	0	0	0	0	0	9	0	0	0	0	0	0	0	0	0	0
0	0	0	0	0	0	0	0	0	0	0	0	0	0	0	0	0	0	0
0	0	0	0	0	0	0	0	0	0	0	0	0	0	0	0	0	0	0
0	0	0	0	0	0	0	0	0	0	0	1	0	0	0	0	0	0	0
0	0	0	0	0	0	0	0	0	0	0	0	0	0	0	0	0	0	0
0	0	0	0	0	0	0	0	0	0	0	0	0	0	0	0	0	0	0
0	0	0	0	0	0	0	0	0	0	0	0	0	0	0	0	0	0	0
0	0	0	0	0	0	0	0	0	0	0	0	0	0	0	0	0	0	0
0	0	0	0	0	0	0	0	0	0	0	0	0	0	0	0	0	0	0
0	0	0	0	0	0	0	0	0	0	0	0	0	0	0	0	0	0	0
0	0	0	0	0	0	0	0	0	0	0	0	0	0	0	0	0	0	0
0	0	0	0	0	0	0	0	0	0	0	0	0	0	0	0	0	0	0
0	0	0	0	0	0	0	0	0	0	0	0	0	0	0	0	0	0	0
0	0	0	0	0	0	0	0	0	0	0	0	0	0	0	0	0	0	0
0	0	0	0	0	0	0	0	0	0	0	0	0	0	0	0	0	0	0
0	0	0	0	0	0	0	0	0	0	0	0	0	0	0	0	0	0	0
0	0	0	0	0	0	0	0	0	0	0	0	0	0	0	0	0	0	0
0	0	0	0	0	0	0	0	0	0	0	0	0	0	0	0	0	0	0
0	0	0	0	0	0	0	0	0	0	0	0	0	0	0	0	0	0	0
0	0	0	0	0	0	0	0	0	0	0	0	0	0	0	0	0	0	0
0	0	0	0	0	0	0	0	0	0	0	0	0	0	0	0	0	0	0

0	0	0	0	0	0	0	0	0	0	0	0	0	0	0	0	0	0	0
0	0	0	0	0	0	0	0	0	0	0	0	0	9	0	0	0	0	0
0	0	0	0	0	0	0	0	0	0	0	0	0	0	0	0	0	0	0
0	0	0	0	0	0	0	0	0	0	0	0	0	0	0	0	0	0	0
0	0	0	0	0	0	0	0	0	0	0	0	0	0	0	0	0	0	0
0	0	0	0	0	0	0	0	0	0	0	0	0	0	0	0	0	0	0
0	0	0	0	0	0	0	0	0	0	0	0	0	0	0	0	0	0	0
0	0	0	0	0	0	0	0	0	0	0	0	0	0	0	0	0	0	0
0	0	0	0	0	0	0	0	0	0	0	0	0	0	0	0	0	0	0
0	0	0	0	0	0	0	0	0	0	0	0	0	0	0	0	0	0	0
0	0	0	0	0	0	0	0	0	0	0	0	0	0	0	0	0	0	0
0	0	0	0	0	0	0	0	0	0	0	0	0	0	0	0	0	0	0
0	0	0	0	0	0	0	0	0	0	0	0	0	0	0	0	0	0	0
0	0	0	0	0	0	0	0	0	0	0	0	0	0	0	0	0	0	0
0	0	0	0	0	0	0	0	0	0	0	0	0	0	0	0	0	0	0
0	0	0	0	0	0	0	0	0	0	0	0	0	0	0	0	0	0	0
0	0	0	0	0	0	0	0	0	0	0	0	0	0	0	0	0	0	0
0	0	0	0	0	0	0	0	0	0	0	0	0	0	0	0	0	0	0
0	0	0	0	0	0	0	0	0	0	0	0	0	0	0	0	0	0	0
0	0	0	0	0	0	0	0	0	0	0	0	0	0	0	0	0	0	0
0	0	0	0	0	0	0	0	0	0	0	0	0	0	0	0	0	0	0
0	0	0	0	0	0	0	0	0	0	0	0	0	0	0	0	0	0	0
0	0	0	0	0	0	0	0	0	0	0	0	0	0	0	0	0	0	0
0	0	0	0	0	0	0	0	0	0	0	0	0	0	0	0	0	0	0
0	0	0	0	0	0	0	0	0	0	0	0	0	0	0	0	0	0	0
0	0	0	0	0	0	0	0	0	0	0	0	0	0	0	0	0	0	0
0	0	0	0	0	0	0	0	0	0	0	0	0	0	0	0	0	0	0
0	0	0	0	0	0	0	0	0	0	0	0	0	0	0	0	0	0	0
0	0	0	0	0	0	0	0	0	0	0	0	0	0	0	0	0	0	0
0	0	0	0	0	0	0	0	0	0	0	0	0	0	0	0	0	0	0
0	0	0	0	0	0	0	0	0	0	0	0	0	0	0	0	0	0	0
0	0	0	0	0	0	0	0	0	0	0	0	0	0	0	0	0	0	0
0	0	0	0	0	0	0	0	0	0	0	0	0	0	0	0	0	0	0
0	0	0	0	0	0	0	0	0	0	0	0	0	0	0	0	0	0	0
0	0	0	0	0	0	0	0	0	0	0	0	0	0	0	0	0	0	0
0	0	0	0	0	0	0	0	0	0	0	0	0	0	0	0	0	0	0
0	0	0	0	0	0	0	0	0	0	0	0	0	0	0	0	0	0	0

0	0	0	0	0	4	0	0	0	0	0	0	0	0	0	0	0	0	0
0	0	0	0	0	0	0	0	0	0	0	0	0	0	0	0	0	0	0
0	0	0	0	0	0	0	0	0	0	0	0	0	0	0	0	0	0	0
0	0	0	0	0	0	0	0	0	0	0	0	0	0	0	0	0	0	0
0	0	0	0	0	0	0	0	0	0	0	0	0	0	0	0	0	0	0
0	0	0	0	0	0	0	0	0	0	0	0	0	0	0	0	0	0	0
0	0	0	0	0	0	0	0	0	0	0	0	0	0	0	0	0	0	0
0	0	0	0	0	0	0	0	0	0	0	0	0	0	0	0	0	0	0
0	0	0	0	0	0	0	0	0	0	0	0	0	0	0	0	0	0	0
0	0	0	0	0	0	0	0	0	0	0	0	0	0	0	0	0	0	0
0	0	0	0	0	0	0	0	0	0	0	0	0	0	0	0	0	0	0
0	0	0	0	0	0	0	0	0	0	0	0	0	0	0	0	0	0	0
0	0	0	0	0	0	0	0	0	0	0	0	0	0	0	0	0	0	0
0	0	0	0	0	0	0	0	0	0	0	0	0	0	0	0	0	0	0
0	0	0	0	0	0	0	0	0	0	0	0	0	0	0	0	0	0	0
0	0	0	0	0	0	0	0	0	0	0	0	0	0	0	0	0	0	0
0	0	0	0	0	0	0	0	0	3	3	0	0	0	0	0	0	0	0
0	0	0	0	0	0	0	0	0	0	0	0	0	0	0	0	0	0	0
0	0	0	0	0	0	0	0	0	0	0	0	0	0	0	0	0	0	0
0	0	0	0	0	0	0	0	0	0	0	0	0	0	0	0	0	0	0
0	0	0	0	0	0	0	0	0	0	0	0	0	0	0	0	0	0	0
0	0	0	0	0	0	0	0	0	0	0	0	0	0	0	0	0	0	0
0	0	0	0	0	0	0	0	0	0	0	0	0	0	0	0	0	0	0
0	0	0	0	0	0	0	0	0	0	0	0	0	0	0	0	0	0	0
0	0	0	0	0	0	0	0	0	0	0	0	0	0	0	0	0	0	0
0	0	0	0	0	0	0	0	0	0	0	0	0	0	0	0	0	0	0
0	0	0	0	0	0	0	0	0	0	0	0	0	0	0	0	0	0	0
0	0	0	0	0	0	0	0	0	0	0	0	0	0	0	0	0	0	0
0	0	0	0	0	0	0	0	0	0	0	0	0	0	0	0	0	0	0
0	0	0	0	0	0	0	0	0	0	0	0	0	0	0	0	0	0	0
0	0	0	0	0	0	0	0	0	0	0	0	0	0	0	0	0	0	0
0	0	0	0	0	0	0	0	0	0	0	0	0	0	0	0	0	0	0
0	0	0	0	0	0	0	0	0	0	0	0	0	0	0	0	0	0	0
0	0	0	0	0	0	0	0	0	0	0	0	0	0	0	0	0	0	0
0	0	0	0	0	0	0	0	0	0	0	0	0	0	0	0	0	0	0
0	0	0	0	0	0	0	0	0	0	0	0	0	0	0	0	0	0	0
0	0	0	0	0	0	0	0	0	0	0	0	0	0	0	0	0	0	0

0	0	0	0	0	0	0	0	0	0	0	0	0	0	0	0	0	0	0
0	0	0	0	0	0	0	0	0	0	0	0	0	0	0	0	0	0	0
0	0	0	0	0	0	0	0	0	0	0	0	0	0	0	0	0	0	0
0	0	0	0	0	0	0	0	0	0	0	0	0	0	0	0	0	0	0
0	0	0	0	0	0	0	0	0	0	0	0	0	0	0	0	0	0	0
0	0	0	0	0	0	0	0	0	0	0	0	0	0	0	0	0	0	0
0	0	0	0	0	0	0	0	0	0	0	0	0	0	0	0	0	0	0
0	0	0	0	0	2	0	0	0	0	0	0	0	0	0	0	0	0	0
0	0	0	0	0	0	0	0	0	0	0	0	0	0	0	0	0	0	0
0	0	0	0	0	0	0	0	0	0	0	0	0	0	0	0	0	0	0
0	0	0	0	0	0	0	0	0	0	0	0	0	0	0	0	0	0	0
0	0	0	0	0	0	0	0	0	0	0	0	0	0	0	0	0	0	0
0	0	0	0	0	0	0	0	0	0	0	0	0	0	0	0	0	0	0
0	0	0	0	0	0	0	0	0	0	0	0	0	0	0	0	0	0	0
0	0	0	0	0	0	0	0	0	0	0	0	0	0	0	0	0	0	0
0	0	0	0	0	0	0	0	0	0	0	0	0	0	0	0	0	0	0
0	0	0	0	0	0	0	0	0	0	0	0	0	0	0	0	0	0	0
0	0	0	0	0	0	0	0	0	0	0	0	0	0	0	0	0	0	0
0	0	0	0	0	0	0	0	0	0	0	0	0	0	0	0	0	0	0
0	0	0	0	0	0	0	0	0	0	0	0	0	0	0	0	0	0	0
0	0	0	0	0	0	0	0	0	0	0	0	0	0	0	0	0	0	0
0	0	0	0	0	0	0	0	0	0	0	0	0	0	0	0	0	0	0
0	0	0	0	0	0	0	0	0	0									
Jan_DL126	1344	1748	360	376	907	1129	281	266	357	267	126	128	211	242	333	90	169	263
235	633	225	8	119	207	46	173	66	0	81	0	151	68	103	0	120	13	0
30	117	67	84	62	0	76	9	60	28	95	52	139	93	61	42	0	121	0
0	73	0	30	69	289	42	66	0	0	62	109	11	49	278	51	28	104	86
34	22	32	84	0	0	18	33	74	0	49	0	0	0	85	0	22	71	23
23	33	64	102	45	0	0	49	0	0	0	0	5	35	29	0	0	0	0
0	0	0	36	49	0	0	0	0	0	16	0	14	0	0	0	7	0	52
0	18	0	0	16	0	0	0	0	0	0	0	14	0	18	66	0	0	0
0	18	16	7	0	7	0	0	2	0	18	0	0	0	0	0	0	0	0
0	0	8	0	0	0	0	28	0	36	67	0	0	0	0	0	40	0	24
0	0	16	0	0	0	0	0	0	0	0	0	18	73	0	0	0	0	0
17	13	9	0	0	0	0	0	0	0	60	0	0	0	0	0	0	7	29
0	0	0	29	0	8	0	0	0	0	0	0	0	11	0	0	53	0	0
0	0	0	0	0	0	0	0	0	0	0	0	10	0	45	0	0	0	0

0	0	25	0	0	0	111	0	84	0	0	0	0	0	0	4	0	0	0
0	0	0	0	0	0	0	0	52	0	0	23	19	0	0	0	16	0	0
0	5	7	0	0	0	0	30	0	0	0	0	0	0	0	17	0	0	0
0	17	10	0	0	0	0	9	0	0	0	0	0	0	0	9	0	0	42
7	0	0	0	0	0	0	0	0	6	0	0	0	0	0	0	0	0	0
0	26	0	0	0	0	17	10	0	0	0	0	0	0	0	0	0	0	0
0	0	0	0	8	0	0	0	0	0	0	0	0	0	0	0	0	0	0
0	0	0	0	0	0	0	0	0	0	0	0	0	4	49	0	0	0	0
0	0	0	0	0	0	0	0	0	0	0	0	0	0	0	0	0	0	0
0	7	0	8	0	0	0	0	0	0	0	0	0	0	0	0	0	0	0
0	11	15	0	0	0	0	0	0	0	0	0	0	0	0	0	0	0	0
0	0	0	0	0	0	0	8	0	0	0	0	0	0	0	0	0	0	0
0	0	0	0	0	0	0	0	0	0	0	0	0	0	0	0	0	0	0
0	0	0	11	0	0	21	11	0	0	0	0	0	0	0	0	0	0	0
0	0	0	0	0	14	0	0	48	0	0	0	0	0	0	0	0	0	0
0	0	0	0	0	0	0	0	0	0	0	0	0	0	0	0	0	0	0
8	14	0	6	0	24	0	0	0	0	0	0	0	4	0	0	0	0	0
10	0	0	0	0	0	0	0	0	0	0	0	0	0	0	0	0	0	0
0	0	0	0	0	0	0	0	0	0	0	0	0	16	0	0	0	0	0
0	0	0	0	0	0	0	0	0	0	0	0	0	0	15	0	0	0	0
0	0	0	35	0	0	0	0	0	0	0	0	0	0	0	0	0	0	0
0	0	12	0	0	0	0	0	0	0	0	0	0	0	0	0	0	0	0
0	0	15	0	0	0	0	0	0	0	0	0	0	0	0	0	0	0	0
0	0	0	0	0	0	0	0	0	0	0	0	0	0	0	0	0	0	0
0	0	0	0	0	0	0	0	0	0	0	11	19	0	0	0	0	0	0
0	0	0	0	0	0	0	0	0	0	0	0	0	0	0	0	0	0	0
0	0	0	0	0	0	0	0	0	0	0	0	0	3	0	0	0	0	0
0	0	0	0	0	0	0	0	0	0	0	0	0	0	7	0	0	0	0
0	0	0	0	0	0	0	0	14	0	0	0	0	0	0	0	0	0	0
0	0	0	0	0	0	0	0	0	0	0	0	0	0	0	0	0	0	0
0	0	0	0	0	0	2	0	0	0	0	9	0	0	0	0	0	0	0
0	0	0	0	0	0	0	0	0	0	0	0	0	0	0	0	0	0	0
0	0	0	0	0	0	0	0	0	0	0	0	0	0	0	0	0	5	0
0	0	0	0	0	0	0	0	0	0	0	0	0	0	0	0	0	0	0
0	0	0	0	0	0	0	0	0	0	0	0	0	0	0	0	0	0	0
0	0	0	0	0	0	0	0	0	0	0	0	0	0	0	0	0	0	0
0	0	0	0	0	0	0	0	0	0	0	0	0	0	0	0	0	0	0

0	0	0	0	0	0	23	0	0	0	0	0	0	0	0	0	0	0	0
0	0	0	0	0	0	0	0	0	0	0	0	0	0	0	0	0	0	0
0	0	0	0	0	0	0	0	0	0	0	0	0	0	12	0	0	0	0
0	0	0	0	0	0	0	0	0	0	0	0	0	0	0	0	0	0	0
0	0	0	0	0	0	0	0	0	0	0	0	0	0	0	0	0	0	0
0	0	0	0	0	0	0	0	0	0	0	0	0	0	0	0	0	0	0
0	0	0	0	0	0	0	0	0	0	0	0	0	0	0	0	0	0	0
0	0	0	0	0	0	0	0	0	0	0	0	0	0	0	22	0	0	0
0	0	0	0	0	0	0	0	0	0	0	0	0	7	0	0	0	0	0
0	0	0	0	0	0	0	0	0	0	0	0	0	0	0	0	0	0	0
0	0	0	0	0	0	0	0	0	0	0	0	0	5	0	0	0	0	0
0	0	0	0	0	0	0	0	0	0	0	0	0	0	0	0	0	0	0
0	0	0	0	0	0	0	0	0	0	0	0	0	0	0	0	0	0	0
0	0	0	0	0	0	0	0	0	0	0	0	0	0	0	0	0	0	0
0	0	0	0	0	0	0	0	0	0	0	0	0	0	0	0	0	0	0
0	0	0	0	0	0	0	0	0	0	0	0	0	0	0	0	0	0	0
10	0	0	0	0	0	0	0	0	0	0	0	0	0	0	0	0	0	0
0	0	0	0	0	0	0	0	0	0	0	0	0	0	0	0	0	0	0
0	0	0	0	0	0	0	0	0	0	0	0	0	0	0	0	0	0	0
0	0	0	13	0	0	0	0	0	0	0	0	0	0	0	0	0	0	0
0	0	0	0	0	0	0	0	0	0	0	0	0	0	0	0	0	0	0
0	0	0	0	0	0	0	0	0	0	0	0	0	12	0	0	0	0	0
0	0	0	0	0	0	0	0	0	0	0	0	0	0	0	0	0	0	0
0	0	0	0	0	0	0	0	0	0	0	0	0	0	0	0	0	0	0
0	0	0	0	0	0	0	0	0	0	0	0	0	0	0	0	0	0	0
0	0	0	0	0	0	0	0	0	0	0	0	0	0	0	0	0	0	0
0	0	0	0	0	0	0	0	0	0	0	0	0	0	0	0	0	0	0
0	0	0	0	0	0	0	0	0	0	0	0	0	0	0	0	0	0	0
0	0	0	0	0	0	0	0	0	0	0	0	0	0	0	0	0	0	0
0	0	0	0	0	0	0	0	0	0	0	0	0	0	0	0	0	0	0
0	0	0	0	0	0	0	0	0	0	0	0	0	0	0	0	0	0	0
0	0	0	0	0	0	0	0	0	0	0	0	0	0	0	0	0	0	0
0	0	0	0	0	0	0	0	0	0	0	0	0	0	0	0	0	0	0
0	0	0	0	0	0	0	0	0	0	0	8	8	0	0	0	0	0	0
0	0	0	0	0	0	0	0	0	0	0	0	0	0	0	0	0	0	0
0	0	0	0	0	0	0	0	0	0	0	0	0	0	0	0	0	0	0
0	0	0	0	0	0	0	0	0	0	0	0	0	0	0	0	0	0	0

0	0	0	0	0	0	0	0	0	0	0	0	0	0	0	0	0	0	0
0	0	0	0	0	0	0	0	0	0	0	0	0	0	0	0	0	0	0
0	0	7	0	0	0	0	0	0	0	0	0	0	0	0	0	0	0	0
0	0	0	0	0	0	0	0	0	0	0	0	0	0	0	0	0	0	0
0	0	0	0	0	0	0	0	0	0	0	0	0	0	0	0	0	0	0
0	0	0	0	0	0	0	0	0	0	0	0	0	0	0	0	0	0	0
0	0	0	0	0	0	0	0	0	0	0	0	0	0	0	0	0	0	0
0	0	0	0	0	0	0	0	0	0	0	0	0	0	0	0	0	0	0
0	0	0	0	0	0	0	0	0	0	0	0	0	0	0	0	0	0	0
0	0	0	0	0	0	0	0	0	0	0	0	0	0	0	0	0	0	0
0	0	0	0	0	0	0	0	0	0	0	0	0	0	0	0	0	0	0
0	0	0	0	0	0	0	0	0	0	0	0	0	0	0	0	0	0	0
0	0	0	0	0	0	0	0	0	0	0	0	0	0	0	0	0	0	0
0	0	0	0	0	0	0	0	0	0	0	0	0	0	0	0	0	0	0
0	0	0	0	0	0	0	0	0	0	0	0	0	0	0	0	0	0	0
0	0	0	0	0	0	0	0	0	0	0	0	0	0	0	0	0	0	0
0	0	0	0	0	0	0	0	0	0	0	0	5	0	0	0	0	0	0
0	0	0	0	0	0	0	0	0	0	0	0	0	0	0	0	0	0	0
0	0	0	0	0	0	0	0	0	0	0	0	0	0	0	0	0	0	0
0	0	0	0	0	0	0	0	0	0	0	0	0	0	0	0	0	0	0
0	0	0	0	0	0	0	0	0	0	0	0	0	0	0	0	0	0	0
0	0	0	0	0	0	0	0	0	0	0	0	0	0	0	0	0	0	0
0	0	0	0	0	0	0	0	0	0	0	0	0	0	0	0	0	0	0
0	0	0	0	0	0	0	0	0	0	0	0	0	0	0	0	0	0	0
0	0	0	0	0	0	0	0	0	0	0	0	0	0	0	0	0	0	0
0	0	0	0	0	0	0	0	0	0	0	0	0	0	0	0	0	0	0
0	0	0	0	0	0	0	0	0	0	0	0	0	0	0	0	0	0	0
0	0	0	0	0	0	0	4	4	4	4	0	0	0	0	0	0	0	0
0	0	0	0	0	0	0	0	0	0	0	0	0	0	0	0	0	0	0
0	0	0	0	0	0	0	0	0	0	0	0	0	0	0	0	0	0	0
0	0	0	0	0	0	0	0	0	0	0	0	0	0	0	0	0	0	0
0	0	0	0	0	0	0	0	0	0	0	0	0	0	0	0	0	0	0
0	0	0	0	0	0	0	0	0	0	0	0	0	0	0	0	0	0	0
0	0	0	0	0	0	0	0	0	0	0	0	0	0	0	0	0	0	0
0	0	0	0	0	0	0	0	0	0	0	0	0	0	0	0	0	0	0
0	0	0	0	0	0	0	0	0	0	0	0	0	0	0	0	0	0	0
0	0	0	0	0	0	0	0	0	0	0	0	0	0	0	0	0	0	0

0	0	0	0	0	0	0	0	0	0	0	0	0	0	0	0	0	0	0
0	0	0	0	0	0	0	0	0	0	0	0	0	0	0	0	0	0	0
0	0	0	0	0	0	0	0	0	0	0	0	0	0	0	0	0	0	0
0	0	0	0	0	0	0	0	0	0	0	0	0	0	0	0	0	0	0
0	0	0	0	0	0	0	0	0	0	0	0	0	0	0	0	0	0	0
0	0	0	0	0	0	0	0	0	0	0	0	0	0	0	0	0	0	0
0	0	0	0	0	0	0	0	0	0	0	0	0	0	0	0	0	0	3
3	3	3	0	0	0	0	0	0	0	0	0	0	0	0	0	0	0	0
0	0	0	0	0	0	0	0	0	0	0	0	0	0	0	0	0	0	0
0	0	0	0	0	0	0	0	0	0	0	0	0	0	0	0	0	0	0
0	0	0	0	0	0	0	0	0	0	0	0	0	0	0	0	0	0	0
0	0	0	0	0	0	0	0	0	0	0	0	0	0	0	0	0	0	0
0	0	0	0	0	0	0	0	0	0	0	0	0	0	0	0	0	0	0
0	0	0	0	0	0	0	0	0	0	0	0	0	0	0	0	0	0	0
0	0	0	0	0	0	0	0	0	0	0	0	0	0	0	0	0	0	0
0	0	0	0	0	0	0	0	0	0	0	0	0	0	0	0	0	0	0
0	0	0	0	0	0	0	0	0	0	0	0	0	0	0	0	0	0	0
0	0	0	0	0	0	0	0	0	0	0	0	0	0	0	0	0	0	0
0	0	0	0	0	0	0	0	0	0	0	0	0	0	0	0	0	0	0
0	0	0	0	0	0	0	0	0	0	0	0	0	0	0	0	0	0	0
0	0	0	0	0	0	0	0	0	0	0	0	0	0	0	0	0	0	0
0	0	0	0	0	0	0	0	0	0	0	0	0	0	0	0	0	0	0
0	0	0	0	0	0	0	0	0	0	0	0	0	0	0	0	0	0	0
0	0	0	0	0	0	0	0	0	0	0	0	0	0	0	0	0	0	0
0	0	0	0	0	0	0	0	0	0	0	0	0	0	0	0	0	0	0
0	0	0	0	0	0	0	0	0	0	0	0	0	0	0	0	0	0	0
0	0	0	0	0	0	0	0	0	0	0	0	0	0	0	0	0	0	0
0	0	0	0	0	0	0	0	0	0	0	0	0	0	0	0	0	0	0
0	0	0	0	0	0	0	0	0	0	0	0	0	0	0	0	0	0	0
0	0	0	0	0	0	0	0	0	0	0	0	0	0	0	0	0	0	0
0	0	0	0	0	0	0	0	0	0	0	0	0	0	0	0	0	0	0
0	0	0	0	0	0	0	0	0	0	0	0	0	0	0	0	0	0	0
0	0	0	0	0	0	0	0	0	0	0	0	0	0	0	0	0	0	0
0	0	0	0	0	0	0	0	0	0	0	0	0	0	0	0	0	2	2
2	2	2	2	2	2	2	2	2	2	0	0	0	0	0	0	0	0	0
0	0	0	0	0	0	0	0	0	0	0	0	0	0	0	0	0	0	0
0	0	0	0	0	0	0	0	0	0	0	0	0	0	0	0	0	0	0

0	0	0	0	0	0	0	0	0	0	0	0	0	0	0	0	0	0	0
0	0	0	0	0	0	0	0	0	0	0	0	0	0	0	0	0	0	0
0	0	0	0	0	0	0	0	0	0	0	0	0	0	0	0	0	0	0
0	0	0	0	0	0	0	0	0	0	0	0	0	0	0	0	0	0	0
0	0	0	0	0	0	0	0	0	0	0	0	0	0	0	0	0	0	0
0	0	0	0	0	0	0	0	0	0	0	0	0	0	0	0	0	0	0
0	0	0	0	0	0	0	0	0	0	0	0	0	0	0	0	0	0	0
0	0	0	0	0	0	0	0	0	0	0	0	0	0	0	0	0	0	0
0	0	0	0	0	0	0	0	0	0	0	0	0	0	0	0	0	0	0
0	0	0	0	0	0	0	0	0	0	0	0	0	0	0	0	0	0	0
0	0	0	0	0	0	0	0	0	0	0	0	0	0	0	0	0	0	0
0	0	0	0	0	0	0	0	0	0	0	0	0	0	0	0	0	0	0
0	0	0	0	0	0	0	0	0	0	0	0	0	0	0	0	0	0	0
0	0	0	0	0	0	0	0	0	0	0	0	0	0	0	0	0	0	0
0	0	0	0	0	0	0	0	0	0									
Jan_DL127	2630	605	292	872	22	725	416	394	533	481	374	144	455	509	14	510	406	281
477	19	71	0	319	54	161	230	119	400	178	79	160	255	182	192	142	203	96
366	48	362	121	146	111	165	0	217	248	193	98	69	248	117	102	112	181	0
43	0	74	14	133	143	128	132	37	20	14	0	0	130	0	55	13	40	72
113	75	57	76	0	95	37	39	0	0	106	0	0	111	65	0	8	0	0
83	71	0	0	59	0	0	0	0	15	0	0	0	0	34	0	0	0	0
62	51	0	30	0	0	0	0	0	35	0	0	6	0	0	0	0	0	0
0	0	58	0	0	0	0	33	0	7	0	0	0	0	0	0	0	0	0
0	12	0	0	0	20	0	0	0	0	0	0	18	0	0	0	10	22	0
0	0	15	0	0	0	0	13	0	45	0	0	20	0	0	11	4	0	0
0	0	7	0	0	0	15	0	0	0	0	0	0	0	0	0	0	0	0
0	0	0	0	9	0	0	0	0	0	0	0	0	0	0	0	0	24	0
0	0	0	22	0	10	0	0	19	0	0	0	15	15	0	0	0	0	4
0	0	0	0	0	0	0	0	0	0	0	0	0	0	4	0	0	0	0
0	0	0	0	0	5	0	0	0	0	0	0	0	0	0	3	0	0	0
25	0	0	0	0	0	0	0	0	0	0	0	3	0	7	0	21	0	0
0	0	0	0	0	0	0	0	0	0	0	0	0	0	0	0	0	0	0
0	20	0	0	0	71	0	23	0	0	0	0	25	0	0	16	0	0	0
6	16	0	0	23	5	0	0	0	0	0	0	0	0	0	0	0	0	0
0	0	0	0	0	0	3	0	0	0	0	0	0	0	0	0	0	0	0
0	0	0	0	0	0	0	0	0	0	0	0	0	0	0	0	0	0	0
0	0	0	0	0	0	4	0	5	0	0	0	0	0	0	0	0	0	0

0	0	0	0	0	0	0	0	0	0	0	0	0	0	0	0	7	0	0
0	0	0	0	0	0	8	0	0	0	0	0	0	0	0	0	0	0	10
0	0	0	0	0	0	0	0	0	0	0	0	0	0	0	0	0	0	0
0	0	0	0	9	0	5	0	0	0	0	0	11	0	0	0	0	0	0
0	0	0	0	0	0	0	0	9	0	0	0	0	0	0	0	0	6	0
0	0	0	0	0	0	0	0	0	0	0	0	0	0	0	0	0	0	0
0	0	0	0	0	0	0	0	0	0	0	0	0	0	0	0	0	0	0
0	0	0	0	0	0	0	0	0	0	0	0	0	0	0	0	0	0	0
0	0	0	0	0	0	0	0	0	7	0	0	24	0	0	0	0	0	9
0	0	0	0	0	0	0	0	0	0	0	0	0	0	0	0	0	0	0
0	12	0	0	0	0	0	0	0	0	0	0	0	0	0	0	0	0	0
0	0	0	9	0	0	0	5	0	0	0	0	0	0	0	0	0	0	0
0	0	0	0	0	0	0	0	0	0	0	0	0	0	0	0	0	0	0
10	0	0	0	0	0	0	0	0	0	0	0	0	0	0	0	0	0	0
0	0	0	0	0	0	0	0	0	0	0	0	0	0	0	0	0	0	10
0	0	0	0	0	0	0	0	0	0	0	0	0	0	8	0	0	0	0
0	0	0	0	0	0	0	0	0	0	0	0	0	0	0	0	0	0	8
0	0	0	0	0	0	5	0	0	0	0	0	0	0	0	0	0	0	0
0	0	0	0	0	0	0	0	0	0	0	0	0	9	0	0	0	0	0
0	0	0	0	0	0	0	0	0	0	0	0	0	0	0	0	0	0	0
0	0	0	0	0	0	0	0	0	0	0	0	0	0	0	0	0	0	0
0	0	0	0	0	0	0	0	0	0	0	0	0	0	0	0	0	0	0
0	0	0	0	0	0	0	0	0	0	0	0	0	0	0	0	0	0	0
0	0	0	0	0	0	0	0	0	0	0	0	0	0	0	0	0	0	0
0	0	0	0	0	0	0	0	0	0	0	0	0	0	0	0	0	0	0
0	0	0	0	0	0	0	0	0	0	0	0	0	0	0	0	0	0	0
0	0	0	0	0	0	0	0	0	0	0	0	0	0	0	0	0	0	0
0	0	0	0	0	0	0	0	0	0	0	0	0	0	0	0	0	0	0
0	0	0	0	0	0	0	0	0	0	0	0	0	0	0	0	0	0	0
0	0	0	0	0	0	0	0	0	0	0	0	0	0	0	0	0	0	0
0	0	0	0	0	0	0	0	0	0	0	0	0	0	0	0	0	0	0
0	0	0	0	0	0	0	0	0	0	0	0	0	0	0	0	0	0	0
0	0	0	0	0	0	0	0	0	0	0	0	0	0	0	0	0	0	0
0	0	0	0	0	0	0	0	0	0	0	0	0	0	0	0	0	0	0
0	0	0	0	0	0	0	0	0	0	0	0	0	0	0	0	0	0	0
0	0	0	0	0	0	0	0	0	0	0	0	0	0	0	0	0	0	0
0	0	0	0	0	0	0	0	0	0	0	0	0	0	0	0	0	0	0

0	0	0	0	0	0	0	0	0	0	0	0	0	0	0	0	0	0	0
0	0	0	0	0	0	0	0	0	0	0	0	0	0	0	0	0	0	0
0	0	0	0	0	0	0	0	0	0	0	0	0	0	0	0	0	0	0
0	0	0	0	0	0	0	0	0	0	0	0	0	0	0	0	0	0	0
0	0	0	0	0	0	0	0	0	0	0	0	0	0	0	0	0	0	0
0	0	0	0	0	0	0	0	0	0	0	0	0	0	0	0	0	0	0
0	0	0	0	0	0	0	0	0	0	0	0	0	0	0	0	0	0	0
0	0	0	0	0	0	0	0	0	0	0	0	0	0	0	0	0	0	0
0	0	0	0	0	0	0	0	0	0	0	0	0	0	0	0	0	0	0
0	0	0	0	0	0	0	0	0	0	0	0	0	0	0	5	0	0	0
0	0	0	0	0	0	0	0	0	0	0	0	0	0	0	0	0	0	0
0	0	0	0	0	0	0	0	0	0	0	0	0	0	0	0	0	0	0
0	0	0	0	0	0	0	0	0	0	0	0	0	0	0	0	0	0	0
0	0	0	0	0	0	0	0	0	0	0	0	0	0	0	0	0	0	0
0	0	0	0	0	0	0	0	0	0	0	0	0	0	0	0	0	0	0
0	0	0	0	0	0	0	0	0	0	0	0	0	0	0	0	0	0	0
0	0	0	0	0	0	0	0	0	0	0	0	0	0	0	0	0	0	0
0	0	0	0	0	0	0	0	0	0	0	0	0	0	0	0	0	0	0
0	0	0	0	0	0	0	0	0	0	0	0	0	0	0	0	0	0	0
0	0	0	0	0	0	0	0	0	0	0	0	0	0	0	0	0	0	0
0	0	0	0	0	0	0	0	0	0	0	0	0	0	0	0	0	0	0
0	0	0	0	0	0	0	0	0	0	0	0	0	0	0	0	0	0	0
0	0	0	0	0	0	0	0	0	0	0	0	0	0	0	0	0	0	0
0	0	0	0	0	0	0	0	0	0	0	0	0	0	0	0	0	0	0
0	0	0	0	0	0	0	0	0	0	0	0	0	0	0	0	0	0	0
0	0	0	0	0	0	0	0	0	0	0	0	0	0	0	0	0	0	0
0	0	0	0	0	0	0	0	0	0	0	0	0	0	0	0	9	9	0
0	0	0	0	0	0	0	0	0	0	0	0	0	0	0	0	0	0	0
0	0	0	0	0	0	0	0	0	0	0	0	0	0	0	0	0	0	0
0	0	0	0	0	0	0	0	0	0	0	0	0	0	0	0	0	0	0
0	0	0	0	0	0	0	0	0	0	0	0	0	0	0	0	0	0	0
0	0	0	0	0	0	0	0	0	0	0	0	0	0	0	0	0	0	0
0	0	0	0	0	0	0	0	0	0	0	0	0	0	0	0	0	0	0
0	0	0	0	0	0	0	0	0	0	0	0	0	0	0	0	0	0	0
0	0	0	0	0	0	0	0	0	0	0	0	0	0	0	0	0	0	0
0	0	0	0	0	0	0	0	0	0	0	0	0	0	0	0	0	0	0
0	0	0	0	0	0	0	0	0	0	0	0	0	0	0	0	0	0	0

0	0	0	7	0	0	0	0	0	0	0	0	0	0	0	0	0	0	0
0	0	0	0	0	0	0	0	0	0	0	0	0	0	0	0	0	0	0
0	0	0	0	0	0	0	0	0	0	0	0	0	0	0	0	0	0	0
0	0	0	0	0	0	0	0	0	0	0	0	0	0	0	0	0	0	0
0	0	0	0	0	0	0	0	0	0	0	0	0	0	0	0	0	0	0
0	0	0	0	0	0	0	0	0	0	0	0	0	0	0	0	0	0	0
0	0	0	0	0	0	0	0	0	0	0	0	0	0	0	0	0	0	0
0	0	0	0	0	0	0	0	0	0	0	0	0	0	0	0	0	0	0
0	0	0	0	0	0	0	0	0	0	0	0	0	0	0	0	0	0	0
0	0	0	0	0	0	0	0	0	0	0	0	0	0	0	0	0	0	0
0	0	0	0	0	0	0	0	0	0	0	0	0	0	0	0	0	0	0
0	0	0	0	0	0	0	0	0	0	0	0	0	0	0	0	0	0	0
0	0	0	0	0	0	0	0	0	0	0	0	0	0	0	0	0	0	0
0	0	0	0	0	0	0	0	0	0	0	0	0	0	0	0	0	0	0
0	0	0	0	0	0	0	0	0	0	0	0	0	0	0	0	0	0	0
0	0	0	0	0	0	0	0	0	0	0	0	0	0	0	0	0	0	0
0	0	0	0	0	0	0	0	0	0	0	0	0	0	0	0	0	0	0
0	0	0	0	0	0	0	0	0	0	0	0	0	0	0	0	0	0	0
0	0	0	0	0	0	0	0	0	0	0	0	0	0	0	0	0	0	0
0	0	0	0	0	0	0	0	0	0	0	0	0	0	0	0	0	0	0
0	0	0	0	0	0	0	0	0	0	0	0	0	0	0	0	0	0	0
0	0	0	0	0	0	0	0	0	0	0	0	0	0	0	0	0	0	0
0	0	0	0	0	0	0	0	0	0	0	0	0	0	0	0	0	0	0
0	0	0	0	0	0	0	0	0	0	0	0	0	0	0	0	0	0	0
0	0	0	0	0	0	0	0	0	0	0	0	0	0	0	0	0	0	0
0	0	0	0	0	0	0	0	0	0	0	0	0	0	0	0	0	0	0
0	0	0	0	0	0	0	0	0	0	0	0	0	0	0	0	0	0	0
0	0	0	0	0	0	0	0	0	0	0	0	0	0	0	0	0	0	0
0	0	0	0	0	0	0	0	0	0	0	0	0	0	0	0	0	0	0
0	0	0	0	0	0	0	0	0	0	0	0	0	0	0	0	0	0	0
0	0	0	0	0	0	0	0	0	0	0	0	0	0	0	0	0	0	0
0	0	0	0	0	0	0	0	0	0	0	0	0	0	0	0	0	0	0
0	0	0	0	0	0	0	0	0	0	0	0	0	0	0	0	0	0	0
0	0	0	0	0	0	0	0	0	0	0	0	0	0	0	0	0	0	0
0	0	0	0	0	0	0	0	0	0	0	0	0	0	0	0	0	0	0
0	0	0	0	0	0	0	0	0	0	0	0	0	0	0	0	0	0	0
0	0	0	0	0	0	0	0	0	0	0	0	0	0	0	0	0	0	0

0	0	0	0	0	0	0	0	0	0	0	0	0	0	0	0	0	0	0
0	0	0	0	0	0	0	0	0	0	0	0	0	0	0	0	0	0	0
0	0	0	0	0	0	0	0	0	0	0	0	0	0	0	0	0	0	0
0	0	0	0	0	0	0	0	0	0	0	0	0	0	0	0	0	0	0
0	0	0	0	0	0	0	0	0	0	0	0	0	0	0	0	0	0	0
0	0	0	0	0	0	0	0	0	0	0	0	0	0	0	0	0	0	0
0	0	0	0	0	0	0	0	0	0	0	0	0	0	0	0	0	0	0
0	0	0	0	0	0	0	0	0	0	0	0	0	0	0	0	0	0	0
0	0	0	0	0	0	0	0	0	0	0	0	0	0	0	0	0	3	0
0	0	0	0	0	0	0	0	0	0	0	0	0	0	0	0	0	0	0
0	0	0	0	0	0	0	0	0	0	0	0	0	0	0	0	0	0	0
0	0	0	0	0	0	0	0	0	0	0	0	0	0	0	0	0	0	0
0	0	0	0	0	0	0	0	0	0	0	0	0	0	0	0	0	0	0
0	0	0	0	0	0	0	0	0	0	0	0	0	0	0	0	0	0	0
0	0	0	0	0	0	0	0	0	0	0	0	0	0	0	0	0	0	0
0	0	0	0	0	0	0	0	0	0	0	0	0	0	0	0	0	0	0
0	0	0	0	0	0	0	0	0	0	0	0	0	0	0	0	0	0	0
0	0	0	0	0	0	0	0	0	0	0	0	0	0	0	0	0	0	0
0	0	0	0	0	0	0	0	0	0	0	0	0	0	0	0	0	0	0
0	0	0	0	0	0	0	0	0	0	0	0	0	0	0	0	0	0	0
0	0	0	0	0	0	0	0	0	0	0	0	0	0	0	0	0	0	0
0	0	0	0	0	0	0	0	0	0	0	0	0	0	0	0	0	0	0
0	0	0	0	0	0	0	0	0	0	0	0	0	0	0	0	0	0	0
0	0	0	0	0	0	0	0	0	0	0	0	0	0	0	0	0	0	0
0	0	0	0	0	0	0	0	0	0	0	0	0	0	0	0	0	0	0
0	0	0	0	0	0	0	0	0	0	0	0	0	0	0	0	0	0	0
0	0	0	0	0	0	0	0	0	0	0	0	0	0	0	0	0	0	0
0	0	0	0	0	0	0	0	0	0	0	0	0	0	0	0	0	0	0
0	0	0	0	0	0	0	0	0	0	0	0	0	0	0	0	0	0	0
0	0	0	0	0	0	0	0	0	0	0	0	0	0	0	0	0	0	0
0	0	0	0	0	0	0	0	0	0	0	0	0	0	0	0	0	0	0
0	0	0	0	0	0	0	0	0	0	0	0	0	0	0	0	0	0	0
0	0	0	0	0	0	0	0	0	0	0	0	0	0	0	0	0	0	0
0	0	0	0	0	0	0	0	0	0	0	0	0	0	0	0	0	0	0
0	0	0	0	0	2	2	2	2	0	0	0	0	0	0	0	0	0	0
0	0	0	0	0	0	0	0	0	0	0	0	0	0	0	0	0	0	0
0	0	0	0	0	0	0	0	0	0	0	0	0	0	0	0	0	0	0

0	0	0	0	0	0	0	0	0	0	0	0	0	0	0	0	0	0	0
0	0	0	0	0	0	0	0	0	0	0	0	0	0	0	0	0	0	0
0	0	0	0	0	0	0	0	0	0	0	0	0	0	0	0	0	0	0
0	0	0	0	0	0	0	0	0	0	0	0	0	0	0	0	0	0	0
0	0	0	0	0	0	0	0	0	0	0	0	0	0	0	0	0	0	0
0	0	0	0	0	0	0	0	0	0	0	0	0	0	0	0	0	0	0
0	0	0	0	0	0	0	0	0	0									
Jan_DL128	792	1077	478	438	441	425	38	59	293	187	255	86	173	141	146	257	109	173
108	247	192	68	56	160	149	101	164	117	59	135	105	72	82	133	52	217	139
135	55	0	124	61	84	76	15	22	92	35	137	53	49	51	91	0	39	89
82	0	112	117	23	52	0	52	36	73	91	83	0	0	86	244	40	50	0
41	40	33	45	9	52	18	29	0	0	53	0	0	30	16	104	47	23	52
8	24	0	0	65	8	0	0	0	11	0	59	0	0	27	0	0	0	0
0	0	0	13	27	21	0	0	0	0	25	23	9	0	0	0	14	0	0
0	0	0	0	0	0	0	50	0	18	0	0	14	0	26	18	0	0	0
0	0	45	0	0	16	0	0	0	0	24	0	21	27	0	0	24	0	8
0	0	9	13	0	0	0	0	0	17	18	10	96	0	0	0	9	0	12
18	0	14	0	0	0	0	0	0	4	0	0	0	0	15	0	0	0	0
0	0	0	0	11	0	0	0	0	0	0	0	0	0	0	0	13	9	0
0	0	14	15	0	10	0	0	0	0	0	0	0	4	38	0	0	0	14
0	0	0	0	0	0	0	0	0	6	0	0	0	10	16	0	0	0	0
0	0	19	0	0	4	0	0	0	0	7	0	0	0	12	6	0	0	0
0	0	0	0	0	0	0	0	4	0	0	0	5	0	0	0	8	0	0
0	8	0	0	0	0	0	0	0	0	10	11	0	4	17	8	46	0	0
0	0	7	0	0	0	0	9	6	0	0	0	0	12	16	0	0	23	0
0	0	0	16	0	9	0	0	0	5	0	0	0	30	0	0	0	0	14
0	0	0	0	0	0	0	0	0	10	0	0	9	0	37	0	0	0	0
0	0	0	0	0	0	0	0	0	0	0	0	0	0	9	0	0	0	0
0	0	0	0	0	0	4	0	0	8	0	0	0	0	0	0	0	0	0
0	0	13	3	0	0	0	0	3	0	0	0	0	0	0	0	0	0	0
0	0	0	0	0	0	0	0	0	0	0	0	26	0	0	0	0	0	0
0	0	0	0	0	0	0	0	0	0	0	10	0	0	0	0	0	0	0
0	0	0	0	0	0	0	0	0	0	0	11	0	0	0	0	0	0	0
0	0	18	0	0	0	0	0	0	7	0	0	0	0	0	0	0	0	0
0	0	0	0	0	0	0	0	0	0	0	0	0	0	0	0	0	0	0
0	0	0	0	0	0	0	0	0	0	0	6	0	0	0	0	0	0	0
0	6	0	0	0	0	0	0	0	0	0	0	0	0	0	0	0	0	0

0	0	0	4	0	0	0	0	0	0	0	0	0	0	0	0	0	0	0
7	0	0	0	0	0	0	0	0	0	0	0	0	0	0	0	0	0	0
0	0	0	0	0	0	0	0	7	0	0	0	0	0	0	0	0	0	0
4	0	0	0	0	0	0	0	0	0	0	0	0	0	0	0	0	0	0
0	0	0	0	0	0	0	0	11	0	0	0	5	0	0	0	0	0	0
0	0	0	0	0	0	0	0	0	0	0	0	0	0	0	0	0	0	0
0	0	0	0	0	0	0	0	0	0	0	0	0	0	0	0	0	0	0
0	0	0	0	0	0	0	0	0	0	0	0	0	0	0	0	0	0	0
0	0	0	0	0	0	0	0	0	0	0	13	0	0	0	0	0	0	0
0	0	0	0	0	0	0	0	0	0	0	0	0	0	0	0	0	0	0
0	0	0	0	0	0	0	0	0	0	0	0	0	0	0	0	0	0	0
0	0	0	0	0	0	0	0	0	0	0	0	0	0	0	0	0	0	0
0	0	0	0	0	0	0	0	0	0	0	0	0	0	0	0	12	0	0
0	0	0	0	0	0	0	0	0	0	0	0	0	0	0	0	0	0	0
0	0	0	0	0	0	0	0	0	0	0	0	0	0	0	0	0	0	0
0	0	0	0	0	0	0	0	0	0	0	0	0	0	0	0	0	0	0
0	0	0	0	0	0	0	0	0	0	0	0	0	0	0	0	0	0	0
0	6	0	0	0	0	0	0	0	0	0	0	0	0	0	0	0	0	0
0	0	0	0	0	0	0	0	0	0	0	0	0	0	0	0	0	0	0
0	0	0	0	0	0	0	0	0	0	0	0	0	0	0	0	0	0	0
0	0	0	0	0	0	0	0	0	0	0	0	0	0	0	0	0	0	0
0	0	0	0	0	0	0	0	0	0	0	0	0	0	0	0	0	0	26
0	0	0	0	0	0	0	0	0	0	0	0	0	0	0	0	0	0	0
0	0	0	0	0	0	0	0	0	0	0	0	0	0	0	0	0	0	0
0	0	0	0	0	0	0	0	0	0	0	0	0	0	0	0	0	0	0
0	0	0	0	0	0	0	0	0	0	0	0	0	0	0	0	0	0	0
0	0	0	0	0	0	0	0	0	0	0	0	0	0	0	0	0	0	0
0	0	0	0	0	0	0	0	0	0	0	0	0	0	0	0	0	0	0
0	0	0	0	0	0	0	0	0	10	0	0	0	0	0	0	0	0	0
0	0	0	0	0	0	0	0	0	0	0	0	0	0	0	0	0	0	0
0	0	0	0	0	0	0	0	0	0	0	0	0	0	0	0	0	0	0
0	0	0	0	0	0	0	0	0	0	0	0	0	0	0	0	0	0	0
0	0	0	0	0	0	0	0	0	0	0	0	0	0	0	0	0	0	0
0	0	0	0	0	0	0	0	0	0	0	0	0	0	0	0	0	0	0
0	0	0	0	0	0	0	0	0	0	0	0	0	0	0	0	0	0	0
0	0	0	0	0	0	0	0	0	0	0	0	0	0	0	0	0	0	0
0	0	0	0	0	0	0	0	0	0	0	0	0	0	0	0	0	0	0

0	0	0	0	0	0	0	0	0	0	0	0	0	0	0	0	0	0	0
0	0	0	0	0	0	0	0	0	0	0	0	0	0	0	0	0	0	0
0	0	0	0	0	0	0	0	0	0	0	0	0	0	0	0	0	0	0
0	0	0	0	0	0	0	0	0	0	0	0	0	0	0	0	0	0	0
0	0	0	0	0	0	0	0	0	0	0	0	0	0	0	0	0	0	0
0	0	0	0	0	0	0	0	0	0	0	0	0	0	0	0	0	0	0
0	0	0	0	0	0	0	0	0	0	0	0	0	0	0	0	0	0	0
0	0	0	0	0	0	0	0	0	0	0	0	0	0	0	0	0	0	0
0	0	0	0	0	0	0	0	0	0	0	0	0	8	8	0	0	0	0
0	0	0	0	0	0	0	0	0	0	0	0	0	0	0	0	0	0	0
0	0	0	0	0	0	0	0	0	0	0	0	0	0	0	0	0	0	0
0	0	0	0	0	0	0	0	0	0	0	0	0	0	0	0	0	0	0
0	0	0	0	0	0	0	0	0	0	0	0	0	0	0	0	0	0	0
0	0	0	0	0	0	0	0	0	0	0	0	0	0	0	0	0	0	0
0	0	0	0	0	0	0	0	0	0	0	0	0	0	0	0	0	0	0
0	0	0	0	0	0	0	0	0	0	0	0	0	0	0	0	0	0	0
0	0	0	0	0	0	0	0	0	0	0	0	0	0	0	0	0	0	0
0	0	0	0	0	0	0	0	0	0	0	0	0	0	0	0	0	0	0
0	0	0	0	0	0	0	0	0	0	0	0	0	0	0	0	0	0	0
0	0	0	0	0	0	0	0	0	0	0	0	0	0	0	0	0	0	0
0	0	0	0	0	0	0	0	0	0	0	0	0	0	0	0	0	0	0
0	0	0	0	0	0	0	0	0	0	0	0	0	0	0	0	0	0	0
0	0	0	0	0	0	0	0	0	0	0	0	0	0	0	0	0	0	0
0	0	0	0	0	0	0	0	0	0	0	0	0	0	0	0	0	0	0
0	0	0	0	0	0	0	0	0	0	0	0	0	0	0	0	0	0	0
0	0	0	0	0	0	0	0	0	5	0	0	0	0	0	0	0	0	0
0	0	0	0	0	0	0	0	0	0	0	0	0	0	0	0	0	0	0
0	0	0	0	0	0	0	0	0	0	0	0	0	0	0	0	0	0	0
0	0	0	0	0	0	0	0	0	0	0	0	0	0	0	0	0	0	0
0	0	0	0	0	0	0	0	0	0	0	0	0	0	0	0	0	0	0
0	0	0	0	0	0	0	0	0	0	0	0	0	0	0	0	0	0	0
0	0	0	0	0	0	0	0	0	0	0	0	0	0	0	0	0	0	0
0	0	0	0	0	0	0	0	0	0	0	0	0	0	0	0	0	0	0
0	0	0	0	0	0	0	0	0	0	0	0	0	0	0	0	0	0	0
0	0	0	0	0	0	0	0	0	0	0	0	0	0	0	0	0	0	0
0	0	0	0	0	0	0	0	0	0	0	0	0	0	0	0	0	0	0
0	4	4	4	4	4	0	0	0	0	0	0	0	0	0	0	0	0	0

0	0	0	0	0	0	0	0	0	0	0	0	0	0	0	0	0	0	0
0	0	0	0	0	0	0	0	0	0	0	0	0	0	0	0	0	0	0
0	0	0	0	0	0	0	0	0	0	0	0	0	0	0	0	0	0	0
0	0	0	0	0	0	0	0	0	0	0	0	0	0	0	0	0	0	0
0	0	0	0	0	0	0	0	0	0	0	0	0	0	0	0	0	0	0
0	0	0	0	0	0	0	0	0	0	0	0	0	0	0	0	0	0	0
0	0	0	0	0	0	0	0	0	0	0	0	0	0	0	0	0	0	0
0	0	0	0	0	0	0	0	0	0	0	0	0	0	0	0	0	0	0
0	0	0	0	0	0	0	0	0	0	0	0	0	0	0	0	0	0	0
0	0	0	0	0	0	0	0	0	0	0	0	0	0	0	0	0	0	0
0	0	0	0	0	0	0	0	0	0	0	0	0	0	0	0	0	0	0
0	0	0	0	0	0	0	0	0	0	0	0	0	0	0	0	0	0	0
0	0	0	0	0	3	3	3	3	0	0	0	0	0	0	0	0	0	0
0	0	0	0	0	0	0	0	0	0	0	0	0	0	0	0	0	0	0
0	0	0	0	0	0	0	0	0	0	0	0	0	0	0	0	0	0	0
0	0	0	0	0	0	0	0	0	0	0	0	0	0	0	0	0	0	0
0	0	0	0	0	0	0	0	0	0	0	0	0	0	0	0	0	0	0
0	0	0	0	0	0	0	0	0	0	0	0	0	0	0	0	0	0	0
0	0	0	0	0	0	0	0	0	0	0	0	0	0	0	0	0	0	0
0	0	0	0	0	0	0	0	0	0	0	0	0	0	0	0	0	0	0
0	0	0	0	0	0	0	0	0	0	0	0	0	0	0	0	0	0	0
0	0	0	0	0	0	0	0	0	0	0	0	0	0	0	0	0	0	0
0	0	0	0	0	0	0	0	0	0	0	0	0	0	0	0	0	0	0
0	0	0	0	0	0	0	0	0	0	0	0	0	0	0	0	0	0	0
0	0	0	0	0	0	0	0	0	0	0	0	0	0	0	0	0	0	0
0	0	0	0	0	0	0	0	0	0	0	0	0	0	0	0	0	0	0
0	0	0	0	0	0	0	0	0	0	0	0	0	0	0	0	0	0	0
0	0	0	0	0	0	0	0	0	0	0	0	0	0	0	0	0	0	0
0	0	0	0	0	0	0	0	0	0	0	0	0	0	0	0	0	0	0
0	0	0	0	0	0	0	0	0	0	0	0	0	0	0	0	0	0	0
0	0	0	0	0	0	0	0	0	0	0	0	0	0	0	0	0	0	0
0	0	0	0	0	0	0	0	0	0	0	0	0	0	0	0	0	0	0
0	0	0	0	0	0	0	0	0	0	0	0	0	0	0	0	0	0	0
0	0	0	0	0	0	0	0	0	0	0	0	0	0	0	0	0	0	0
0	0	0	0	0	0	0	0	0	0	0	0	0	0	0	0	0	0	0
0	0	0	0	0	0	0	0	0	0	0	0	0	0	0	0	0	0	0
0	0	0	0	0	0	0	0	0	0	0	0	0	0	0	0	2	2	2

2	2	2	2	2	2	2	0	0	0	0	0	0	0	0	0	0	0	0
0	0	0	0	0	0	0	0	0	0	0	0	0	0	0	0	0	0	0
0	0	0	0	0	0	0	0	0	0	0	0	0	0	0	0	0	0	0
0	0	0	0	0	0	0	0	0	0	0	0	0	0	0	0	0	0	0
0	0	0	0	0	0	0	0	0	0	0	0	0	0	0	0	0	0	0
0	0	0	0	0	0	0	0	0	0	0	0	0	0	0	0	0	0	0
0	0	0	0	0	0	0	0	0	0	0	0	0	0	0	0	0	0	0
0	0	0	0	0	0	0	0	0	0	0	0	0	0	0	0	0	0	0
0	0	0	0	0	0	0	0	0	0	0	0	0	0	0	0	0	0	0
0	0	0	0	0	0	0	0	0	0	0	0	0	0	0	0	0	0	0
0	0	0	0	0	0	0	0	0	0	0	0	0	0	0	0	0	0	0
0	0	0	0	0	0	0	0	0	0	0	0	0	0	0	0	0	0	0
0	0	0	0	0	0	0	0	0	0	0	0	0	0	0	0	0	0	0
0	0	0	0	0	0	0	0	0	0	0	0	0	0	0	0	0	0	0
0	0	0	0	0	0	0	0	0	0	0	0	0	0	0	0	0	0	0
0	0	0	0	0	0	0	0	0	0	0	0	0	0	0	0	0	0	0
0	0	0	0	0	0	0	0	0	0	0	0	0	0	0	0	0	0	0
0	0	0	0	0	0	0	0	0	0	0	0	0	0	0	0	0	0	0
0	0	0	0	0	0	0	0	0	0	0	0	0	0	0	0	0	0	0
0	0	0	0	0	0	0	0	0	0	0	0	0	0	0	0	0	0	0
0	0	0	0	0	0	0	0	0	0	0	0	0	0	0	0	0	0	0
0	0	0	0	0	0	0	0	0	0	0	0	0	0	0	0	0	0	0
0	0	0	0	0	0	0	0	0	0	0	0	0	0	0	0	0	0	0
0	0	0	0	0	0	0	0	0	0	0	0	0	0	0	0	0	0	0
0	0	0	0	0	0	0	0	0	0	0	0	0	0	0	0	0	0	0
0	0	0	0	0	0	0	0	0	0	0	0	0	0	0	0	0	0	0
0	0	0	0	0	0	0	0	0	0	0	0	0	0	0	0	0	0	0
0	0	0	0	0	0	0	0	0	0	0	0	0	0	0	0	0	0	0
0	0	0	0	0	0	0	0	0	0	0	0	0	0	0	0	0	0	0
0	0	0	0	0	0	0	0	0	0	0	0	0	0	0	0	0	0	0
0	0	0	0	0	0	0	0	0	0	0	0	0	0	0	0	0	0	0
0	0	0	0	0	0	0	0	0	0	0	0	0	0	0	0	0	0	0
0	0	0	0	0	0	0	0	0	0	0	0	0	0	0	0	0	0	0
0	0	0	0	0	0	0	0	0	0	0	0	0	0	0	0	0	0	0
0	0	0	0	0	0	0	0	0	0	0	0	0	0	0	0	0	0	0
0	0	0	0	0	0	0	0	0	0									

Jan_DL	129	1253	1317	173	277	820	400	203	194	123	220	443	203	151	273	85	172	252	197
	143	59	173	0	112	209	41	111	33	117	161	320	158	96	82	218	73	0	295
	107	82	56	44	75	135	54	0	73	43	0	56	94	88	23	33	125	20	229
	195	28	45	131	0	65	44	109	27	0	31	0	54	0	0	29	0	50	32
	0	35	14	104	22	34	41	12	0	43	0	64	13	0	60	0	19	51	0
	29	19	0	0	0	0	0	0	0	0	0	61	0	35	10	34	29	0	0
	0	0	0	24	76	27	0	0	0	0	13	0	16	11	0	0	0	0	0
	0	0	0	0	0	0	0	0	0	36	0	0	5	45	0	0	0	0	0
	0	0	0	0	0	69	27	0	0	0	26	0	10	21	0	0	0	0	0
	0	23	21	0	0	0	0	8	0	0	40	0	134	0	0	0	21	0	56
	0	0	18	0	0	0	0	0	0	8	0	9	71	0	10	0	0	0	0
	17	0	0	0	0	0	79	0	0	0	0	0	0	0	0	0	0	0	0
	0	0	0	0	0	15	0	0	0	0	0	0	0	6	0	13	7	0	48
	0	0	0	0	0	0	0	0	0	31	0	0	6	16	10	45	0	0	0
	0	42	0	0	19	0	11	0	0	0	0	0	0	0	14	4	0	0	0
	0	41	0	0	0	0	0	0	0	0	0	0	4	0	7	0	0	34	0
	0	6	4	0	0	0	0	0	0	0	0	0	0	0	0	8	0	24	0
	0	0	11	0	0	0	0	0	58	0	0	0	0	0	0	14	0	0	0
	0	9	0	0	0	0	0	0	19	0	0	0	0	0	0	0	0	0	0
	0	72	5	0	0	0	4	0	0	17	0	0	12	0	0	0	0	0	0
	0	0	0	0	0	8	0	0	8	0	0	0	0	0	0	0	0	0	0
	0	0	30	0	0	0	0	0	0	9	0	0	0	0	0	0	0	0	0
	0	0	0	0	0	0	0	0	2	0	0	0	0	0	0	0	0	0	0
	0	13	0	0	0	0	0	0	0	0	0	0	0	0	0	0	13	0	0
	0	15	0	0	7	0	0	0	0	0	0	0	0	0	0	0	0	0	0
	0	0	9	0	0	0	0	0	0	9	0	0	5	0	0	0	0	0	0
	0	0	0	0	0	0	0	0	0	2	0	0	0	0	0	0	0	0	0
	0	0	0	0	0	11	0	0	0	0	0	0	0	0	0	0	0	0	0
	0	0	0	0	0	0	0	0	0	0	5	13	0	0	0	0	0	0	0
	0	0	0	0	0	0	0	0	0	0	0	0	8	0	0	0	0	0	0
	0	9	0	0	0	0	0	0	23	0	0	0	0	0	0	0	0	0	0
	0	0	0	0	0	0	0	0	0	0	0	0	0	0	0	0	0	0	0
	0	0	0	0	0	0	0	0	0	0	0	0	0	0	0	0	0	0	0
	0	0	0	0	0	0	0	0	0	0	0	0	0	0	0	0	0	0	0
	0	5	0	0	0	0	0	0	0	0	0	0	0	0	27	0	0	7	10
	0	0	6	0	0	0	0	0	0	0	0	0	0	0	0	0	0	0	0
	0	0	0	0	0	0	0	0	0	0	0	0	0	0	0	0	0	0	0

0	0	0	0	0	0	0	0	0	0	0	0	0	0	0	0	0	0	0
0	0	0	0	23	0	0	0	0	0	0	0	0	0	0	0	0	0	0
0	0	0	0	0	0	0	0	0	0	0	0	0	0	0	0	0	0	0
0	0	0	0	8	0	0	0	0	0	0	0	0	0	0	0	0	0	0
0	0	0	0	0	0	0	5	0	0	0	0	0	0	0	0	0	0	0
0	0	0	0	0	0	0	0	0	8	0	0	0	0	0	0	0	16	0
0	0	0	0	0	0	0	0	0	0	0	0	0	0	0	0	0	0	0
0	0	0	0	0	0	0	0	0	0	0	0	0	0	0	0	0	0	0
0	0	0	0	0	0	0	0	0	0	0	9	0	0	0	0	0	0	0
0	0	5	0	0	0	0	0	0	0	0	0	0	0	0	0	0	0	0
0	0	0	0	0	0	0	0	0	0	0	0	0	0	0	0	0	0	0
0	0	0	0	0	0	0	0	0	0	0	0	0	0	0	0	0	0	0
0	0	0	0	0	0	0	0	0	0	0	0	0	0	0	0	0	0	0
0	0	0	0	0	0	0	0	0	0	0	0	0	0	0	0	0	0	0
0	0	0	0	0	0	0	0	0	0	0	0	0	0	0	0	0	0	0
0	0	0	0	0	0	0	15	15	0	0	0	5	0	0	0	0	0	0
0	0	0	0	0	0	0	0	0	0	0	0	0	5	0	0	0	0	0
0	0	0	0	0	0	0	0	0	0	0	0	0	0	0	0	0	0	0
0	0	0	0	0	0	0	0	0	0	9	0	0	0	0	0	0	0	0
0	0	0	0	0	0	0	0	0	0	0	0	0	0	0	0	0	0	0
0	0	0	0	0	0	0	0	0	0	0	0	0	0	0	0	0	0	0
0	0	0	3	0	0	0	0	0	0	0	0	0	0	0	0	0	0	0
0	0	0	0	0	0	0	0	0	0	0	0	0	0	0	0	0	0	0
0	0	0	0	0	0	0	0	0	0	0	0	0	0	0	0	0	0	0
0	0	0	0	0	0	0	0	0	0	0	0	0	0	0	0	0	0	0
0	0	0	0	0	0	0	0	0	0	0	0	0	0	0	0	0	0	0
0	0	0	0	0	0	0	0	0	0	0	0	0	0	0	0	0	0	0
0	0	0	0	0	0	0	0	0	0	0	0	0	14	0	0	0	0	0
0	0	0	0	0	0	0	0	0	0	0	0	0	0	0	0	4	0	0
0	0	0	0	0	0	0	0	0	0	0	0	0	0	0	0	0	0	0
0	0	0	0	0	0	0	0	0	0	0	0	0	0	0	0	0	0	0
0	0	0	0	0	0	0	0	0	0	0	0	0	0	0	0	0	0	0
0	0	0	0	0	0	0	0	0	0	0	0	0	0	0	0	0	0	0
0	0	0	0	0	0	0	0	0	0	0	6	0	0	0	0	0	0	0
0	0	0	0	0	0	0	0	0	0	0	0	0	0	0	0	0	0	0
0	0	0	0	0	0	0	0	0	0	0	0	0	0	0	0	0	0	0
0	0	0	0	10	0	0	0	0	0	0	0	0	0	0	0	0	0	0

0	0	0	0	0	0	0	0	0	0	0	0	0	0	0	0	0	0	0
0	0	0	0	0	0	0	0	0	0	0	0	0	0	0	0	0	0	0
0	0	0	0	0	0	0	0	0	0	0	0	0	0	0	0	0	0	0
0	0	0	0	0	0	0	0	0	0	0	0	0	0	0	0	0	0	0
0	0	0	0	0	0	0	0	0	0	0	0	0	0	0	0	0	0	0
0	0	0	0	0	0	0	0	0	0	0	0	0	0	0	0	0	0	0
0	0	0	0	0	0	0	0	0	0	0	0	0	0	0	0	0	0	0
0	0	0	0	0	0	0	0	0	0	0	0	0	0	0	0	0	0	0
0	0	0	0	0	0	0	0	0	0	0	0	0	0	0	0	0	0	0
0	0	0	0	0	0	0	0	0	0	0	0	0	0	0	0	0	0	0
0	0	0	0	0	0	0	0	0	0	0	0	0	0	0	0	0	0	0
0	0	0	0	0	0	0	0	0	0	0	0	0	0	0	0	0	0	0
0	0	0	0	0	0	0	0	0	0	0	0	0	0	0	0	0	0	0
0	0	0	0	0	0	0	0	0	0	0	0	0	0	0	0	0	0	0
0	0	0	0	0	0	0	0	0	0	0	0	0	0	0	0	0	0	0
0	0	0	0	0	0	0	0	0	0	0	0	0	0	0	0	0	0	0
0	0	0	0	6	0	0	0	0	0	0	0	0	0	0	0	0	0	0
0	0	0	0	0	0	0	0	0	0	0	0	0	0	0	0	0	0	0
0	0	0	0	0	0	0	0	0	0	0	0	0	0	0	0	0	0	0
0	0	0	0	0	0	0	0	0	0	0	0	0	0	0	0	0	0	0
0	0	0	0	0	0	0	0	0	0	0	0	0	0	0	0	0	0	0
0	0	0	0	0	0	0	0	0	0	0	0	0	0	0	0	0	0	0
0	0	0	0	0	0	0	0	0	0	0	0	0	0	0	0	0	0	0
0	0	0	0	0	0	0	0	0	0	0	0	0	0	0	0	0	0	0
0	0	0	0	0	0	0	0	0	0	0	0	0	0	0	0	0	0	0
0	0	0	0	0	0	0	0	0	0	0	0	0	0	0	0	0	0	0
0	0	0	0	0	0	0	0	0	0	0	0	0	0	0	0	0	0	0
0	0	0	0	0	0	0	0	0	0	0	0	0	0	0	0	0	0	0
0	0	0	0	0	0	0	0	0	0	0	0	0	0	0	0	0	0	0
0	0	0	0	0	0	0	0	0	0	0	0	0	0	0	0	0	0	0
0	0	0	0	0	0	0	0	0	0	0	0	0	0	0	0	0	0	0
0	0	0	0	0	0	0	0	0	0	0	0	0	0	0	0	0	0	0
0	0	0	0	0	0	0	0	0	0	0	0	0	0	0	0	0	0	0
0	0	0	0	0	0	0	0	0	0	0	0	0	0	0	0	4	4	4
0	0	0	0	0	0	0	0	0	0	0	0	0	0	0	0	0	0	0
0	0	0	0	0	0	0	0	0	0	0	0	0	0	0	0	0	0	0
0	0	0	0	0	0	0	0	0	0	0	0	0	0	0	0	0	0	0

0	0	0	0	0	0	0	0	0	0	0	0	0	0	0	0	0	0	0
0	0	0	0	0	0	0	0	0	0	0	0	0	0	0	0	0	0	0
0	0	0	0	0	0	0	0	0	0	0	0	0	0	0	0	0	0	0
0	0	0	0	0	0	0	0	0	0	0	0	0	0	0	0	0	0	0
0	0	0	0	0	0	0	0	0	0	0	0	0	0	0	0	0	0	0
0	0	0	0	0	0	0	0	0	0	0	0	0	0	0	0	0	0	0
0	0	0	0	0	0	0	0	0	0	0	0	0	0	0	0	0	0	0
0	0	0	0	0	0	0	0	0	0	0	0	0	0	0	0	0	0	0
0	0	0	0	0	0	0	0	0	0	0	0	0	0	0	0	0	0	0
0	0	0	0	0	0	0	0	0	0	0	0	0	0	0	0	0	0	0
0	0	0	0	0	0	0	0	0	0	0	0	0	0	0	0	0	0	0
0	0	0	0	0	0	0	0	0	0	0	0	0	0	0	0	0	0	0
0	3	3	3	3	3	3	0	0	0	0	0	0	0	0	0	0	0	0
0	0	0	0	0	0	0	0	0	0	0	0	0	0	0	0	0	0	0
0	0	0	0	0	0	0	0	0	0	0	0	0	0	0	0	0	0	0
0	0	0	0	0	0	0	0	0	0	0	0	0	0	0	0	0	0	0
0	0	0	0	0	0	0	0	0	0	0	0	0	0	0	0	0	0	0
0	0	0	0	0	0	0	0	0	0	0	0	0	0	0	0	0	0	0
0	0	0	0	0	0	0	0	0	0	0	0	0	0	0	0	0	0	0
0	0	0	0	0	0	0	0	0	0	0	0	0	0	0	0	0	0	0
0	0	0	0	0	0	0	0	0	0	0	0	0	0	0	0	0	0	0
0	0	0	0	0	0	0	0	0	0	0	0	0	0	0	0	0	0	0
0	0	0	0	0	0	0	0	0	0	0	0	0	0	0	0	0	0	0
0	0	0	0	0	0	0	0	0	0	0	0	0	0	0	0	0	0	0
0	0	0	0	0	0	0	0	0	0	0	0	0	0	0	0	0	0	0
0	0	0	0	0	0	0	0	0	0	0	0	0	0	0	0	0	0	0
0	0	0	0	0	0	0	0	0	0	0	0	0	0	0	0	0	0	0
0	0	0	0	0	0	0	0	0	0	0	0	0	0	0	0	0	0	0
0	0	0	0	0	0	0	0	0	0	0	0	0	0	0	0	0	0	0
0	0	0	0	0	0	0	0	0	0	0	0	0	0	0	0	0	0	0
0	0	0	0	0	0	0	0	0	0	0	0	0	0	0	0	0	0	0
0	0	0	0	0	0	0	0	0	0	0	0	0	0	0	0	0	0	0
0	0	0	0	0	0	0	0	0	0	0	0	0	0	0	0	0	0	0
0	0	0	0	0	0	0	0	0	0	0	0	0	0	0	0	0	0	0
0	0	0	0	0	0	0	0	0	0	0	0	0	0	0	0	0	0	0
0	0	0	0	0	0	0	0	0	0	0	0	0	0	0	0	0	0	0
0	0	0	0	0	0	0	0	0	0	0	0	0	0	0	0	0	0	0

0	0	0	0	0	0	0	0	0	0	0	0	0	0	0	0	0	0	0
0	0	0	0	0	0	0	0	0	0	0	0	0	0	0	0	0	0	2
2	2	2	2	2	2	2	2	2	2	0	0	0	0	0	0	0	0	0
0	0	0	0	0	0	0	0	0	0	0	0	0	0	0	0	0	0	0
0	0	0	0	0	0	0	0	0	0	0	0	0	0	0	0	0	0	0
0	0	0	0	0	0	0	0	0	0	0	0	0	0	0	0	0	0	0
0	0	0	0	0	0	0	0	0	0	0	0	0	0	0	0	0	0	0
0	0	0	0	0	0	0	0	0	0	0	0	0	0	0	0	0	0	0
0	0	0	0	0	0	0	0	0	0	0	0	0	0	0	0	0	0	0
0	0	0	0	0	0	0	0	0	0	0	0	0	0	0	0	0	0	0
0	0	0	0	0	0	0	0	0	0	0	0	0	0	0	0	0	0	0
0	0	0	0	0	0	0	0	0	0	0	0	0	0	0	0	0	0	0
0	0	0	0	0	0	0	0	0	0	0	0	0	0	0	0	0	0	0
0	0	0	0	0	0	0	0	0	0	0	0	0	0	0	0	0	0	0
0	0	0	0	0	0	0	0	0	0	0	0	0	0	0	0	0	0	0
0	0	0	0	0	0	0	0	0	0	0	0	0	0	0	0	0	0	0
0	0	0	0	0	0	0	0	0	0	0	0	0	0	0	0	0	0	0
0	0	0	0	0	0	0	0	0	0	0	0	0	0	0	0	0	0	0
0	0	0	0	0	0	0	0	0	0	0	0	0	0	0	0	0	0	0
0	0	0	0	0	0	0	0	0	0	0	0	0	0	0	0	0	0	0
0	0	0	0	0	0	0	0	0	0	0	0	0	0	0	0	0	0	0
0	0	0	0	0	0	0	0	0	0	0	0	0	0	0	0	0	0	0
0	0	0	0	0	0	0	0	0	0	0	0	0	0	0	0	0	0	0
0	0	0	0	0	0	0	0	0	0	0	0	0	0	0	0	0	0	0
0	0	0	0	0	0	0	0	0	0	0	0	0	0	0	0	0	0	0
0	0	0	0	0	0	0	0	0	0	0	0	0	0	0	0	0	0	0
0	0	0	0	0	0	0	0	0	0	0	0	0	0	0	0	0	0	0
0	0	0	0	0	0	0	0	0	0	0	0	0	0	0	0	0	0	0
0	0	0	0	0	0	0	0	0	0									
Jan_DL013	1687	1567	831	655	69	99	117	103	368	550	240	146	453	58	163	517	48	142
144	0	138	222	163	118	168	165	181	135	103	230	128	132	194	94	95	26	186
164	76	72	47	67	71	56	711	0	234	27	252	23	81	128	231	0	57	121
142	166	233	42	0	0	47	69	69	45	10	0	19	50	0	0	37	47	0
30	204	52	53	96	28	30	36	0	80	162	64	15	0	64	33	33	19	0
9	29	0	0	91	17	0	0	0	81	21	0	0	0	38	12	62	0	0
85	0	0	41	8	0	0	0	0	0	7	0	18	22	0	0	0	35	0
0	0	0	17	0	58	23	0	0	32	0	0	8	0	48	0	0	16	0

0	5	0	23	0	13	0	0	0	0	21	0	19	74	70	81	0	0	0
33	0	17	20	0	0	0	0	0	22	10	8	10	0	46	0	4	0	0
42	0	8	0	0	0	0	12	0	6	34	0	0	60	0	0	0	0	10
16	0	36	0	0	52	16	0	0	0	0	0	13	0	0	0	0	5	0
0	0	20	0	0	2	0	0	4	6	0	8	0	9	0	10	0	30	0
7	0	0	0	0	0	0	0	0	0	0	0	13	8	11	0	0	17	0
14	0	0	0	24	8	0	0	0	0	0	0	0	0	7	10	7	0	0
0	0	0	0	0	0	0	0	8	0	0	0	0	0	12	8	0	0	0
0	0	5	0	0	51	0	0	0	0	9	0	0	8	6	19	0	0	2
13	12	0	0	0	0	0	0	0	0	0	9	0	0	0	0	0	0	5
0	0	0	0	6	10	0	0	6	0	0	0	4	0	0	0	0	0	4
17	0	10	0	0	0	6	0	0	0	27	0	0	0	0	0	0	0	5
10	0	0	0	10	0	0	0	0	0	0	0	0	0	0	0	0	0	0
0	0	0	14	4	7	4	0	0	0	0	0	0	0	0	0	0	18	0
0	0	12	0	0	0	0	0	0	0	0	0	0	0	0	0	0	0	0
0	6	0	0	0	0	0	0	0	0	0	0	0	0	0	0	0	13	0
0	0	0	0	0	0	0	0	0	0	0	0	0	0	0	0	0	0	0
0	0	0	0	14	0	6	0	0	0	0	0	0	10	0	0	0	0	0
0	0	7	0	0	0	0	0	0	0	0	0	0	0	0	0	0	3	0
0	0	0	0	0	0	0	0	24	4	0	0	0	0	0	0	0	0	0
0	0	0	0	0	0	0	0	0	0	0	0	0	0	0	0	0	0	0
0	0	7	0	0	0	0	0	0	0	0	0	0	0	0	0	0	0	0
0	0	0	0	0	0	0	0	0	0	0	0	25	0	0	0	0	0	0
0	0	0	0	0	0	0	0	0	0	0	0	20	0	0	0	0	0	0
0	0	0	0	0	0	0	24	0	0	0	0	0	0	0	0	0	0	0
0	0	0	0	0	0	6	0	0	0	0	0	0	0	0	0	0	0	0
0	6	0	0	0	13	0	0	0	0	0	0	0	0	0	0	0	0	0
0	0	0	0	0	0	0	0	0	0	0	0	0	62	0	0	0	0	0
0	0	0	0	0	0	11	0	0	0	0	0	0	0	0	0	0	0	0
0	0	0	0	0	0	0	0	0	0	0	0	0	0	0	0	0	0	0
0	0	0	0	0	0	0	0	0	0	0	0	0	0	0	0	0	0	12
0	0	0	0	0	0	4	0	0	0	0	0	0	0	0	0	0	0	0
0	0	0	0	0	0	4	0	0	0	0	0	0	0	0	0	0	0	0
0	0	0	0	0	0	0	0	16	0	0	0	0	0	0	0	0	0	0
0	0	0	0	0	0	0	0	0	0	0	0	0	0	0	0	9	0	0
0	0	0	0	0	0	0	0	0	0	0	11	0	0	0	0	0	0	0
0	0	0	0	0	0	0	0	0	0	0	0	0	0	0	0	0	0	0

0	0	0	0	0	0	0	0	0	0	0	0	0	0	0	0	0	0	9
0	0	5	0	0	0	0	0	0	0	0	0	0	0	0	0	0	0	0
0	0	0	0	0	0	0	5	0	0	0	0	0	0	0	0	0	0	0
0	0	0	0	0	0	0	0	0	0	0	0	0	0	0	0	0	0	0
0	0	0	0	0	0	0	0	0	0	0	0	0	0	0	0	0	0	0
0	0	0	0	0	0	0	0	0	0	0	0	0	0	0	0	0	0	0
0	0	0	0	0	0	0	0	0	0	0	0	0	0	0	0	0	0	0
0	0	0	0	0	0	0	0	0	0	0	0	0	0	0	0	0	0	0
0	0	0	0	0	0	0	0	0	0	0	0	0	0	0	0	0	0	0
0	0	0	0	0	0	0	0	0	0	0	0	0	0	0	0	0	0	0
0	0	0	0	0	0	0	0	0	0	0	0	0	0	0	0	0	0	0
0	0	0	0	0	0	0	0	0	0	0	0	0	0	0	0	0	0	0
0	0	0	0	0	0	0	0	0	0	0	0	0	0	0	0	0	0	0
0	0	0	0	0	0	0	0	0	0	0	0	0	0	0	0	0	0	0
0	0	0	0	0	0	0	0	0	0	0	0	0	0	0	0	0	0	0
0	0	0	0	0	0	0	0	0	0	0	0	0	0	0	0	0	0	0
0	0	0	0	0	0	0	0	0	0	0	0	0	0	0	0	0	0	0
0	0	0	0	0	0	0	0	0	0	0	0	0	19	0	0	0	0	0
0	0	0	0	0	0	0	0	0	0	0	0	0	0	0	0	0	0	0
0	0	0	0	0	0	0	0	0	0	0	0	0	0	0	0	0	0	0
0	0	0	0	0	0	7	0	0	0	0	0	0	0	0	0	0	0	0
0	0	0	0	0	0	0	0	0	0	0	0	0	0	0	0	0	0	0
0	0	0	0	0	0	0	0	0	0	0	0	0	0	0	0	0	0	0
0	0	0	0	0	0	0	0	0	0	0	0	0	0	0	0	0	0	0
0	0	0	0	0	0	0	0	0	0	0	0	0	0	0	0	0	0	0
0	0	0	0	0	0	0	0	0	0	0	0	0	0	0	0	0	0	0
0	0	0	0	0	0	0	0	0	0	0	0	0	0	0	0	0	0	0
0	0	0	0	0	0	0	0	0	0	0	0	0	0	0	0	0	0	0
0	0	0	0	0	0	0	0	0	0	0	0	0	0	0	0	0	0	0
0	0	0	0	0	0	0	0	0	0	0	0	0	0	0	0	0	0	0
0	0	0	0	0	0	0	0	0	0	0	0	0	0	0	0	0	0	0
0	0	0	0	0	0	0	0	0	0	0	0	0	0	0	0	0	0	0
0	0	0	0	0	0	0	0	0	0	0	0	0	0	0	0	0	0	0
0	0	0	0	0	0	0	0	0	0	0	0	0	0	0	0	0	0	0
0	0	10	0	0	0	0	0	0	0	0	0	0	0	0	0	0	0	0
5	0	0	0	0	0	0	0	0	0	0	0	0	0	0	0	0	0	0
0	0	0	0	0	0	0	0	0	0	0	0	0	0	0	0	0	0	0

0	0	9	9	0	0	0	0	0	0	0	0	0	0	0	0	0	0	0
0	0	0	0	0	0	0	0	0	0	0	0	0	0	0	0	0	0	0
0	0	0	0	0	0	0	0	0	0	0	0	0	0	0	0	0	0	0
0	0	0	0	0	0	0	0	0	0	0	0	0	0	0	0	0	0	0
0	0	0	0	0	0	0	0	0	0	0	0	0	0	5	0	0	0	0
0	0	0	0	0	0	0	0	0	0	0	0	0	0	0	0	0	0	0
0	0	0	0	0	0	0	0	0	0	0	0	0	0	0	0	0	0	0
0	0	0	0	0	0	0	0	0	0	0	0	0	0	0	0	0	0	0
0	0	0	0	0	0	0	0	0	0	0	0	0	0	0	0	0	0	0
0	0	0	0	0	0	0	0	0	0	0	0	0	0	0	0	0	0	0
0	0	0	0	7	0	0	0	0	0	0	0	0	0	0	0	0	0	0
0	0	0	0	0	0	0	0	0	0	0	0	0	0	0	0	0	0	0
0	0	0	0	0	0	0	0	0	0	0	0	0	0	0	0	0	0	0
0	0	0	0	0	0	0	0	0	0	0	0	0	0	0	0	0	0	0
0	0	0	0	0	0	0	0	0	0	0	0	0	0	0	0	0	0	0
0	0	0	0	0	0	0	0	0	0	0	0	0	0	0	0	0	0	0
0	0	0	0	0	0	0	0	0	0	0	0	0	0	0	0	0	0	0
0	0	0	0	0	0	0	0	0	0	0	0	0	0	0	0	0	0	0
0	0	0	0	0	0	0	0	0	0	0	0	0	0	0	0	0	0	0
0	0	0	0	0	0	0	0	0	0	0	0	0	0	0	0	0	0	0
0	0	0	0	0	0	0	0	0	0	0	0	0	0	0	0	0	0	0
0	0	0	0	0	0	0	0	0	0	0	0	0	0	0	0	0	0	0
0	0	0	0	0	0	0	0	0	0	0	0	0	0	0	0	0	0	0
0	0	0	0	0	0	0	0	0	0	0	0	0	0	0	0	0	0	0
0	0	0	0	0	0	0	0	0	0	0	0	0	0	0	0	0	0	0
0	0	0	0	0	0	0	0	0	0	0	0	0	0	0	0	0	0	0
0	0	0	0	0	0	0	0	0	0	0	0	0	0	0	0	0	0	0
0	0	0	0	0	0	0	0	0	0	0	0	0	0	0	0	0	0	0
0	0	0	0	0	0	0	0	0	0	0	0	0	0	0	0	0	0	0
0	0	0	0	0	0	0	0	0	0	0	0	0	0	0	0	0	0	0
0	0	0	0	0	0	0	0	0	0	0	0	0	0	0	0	0	0	0
0	0	0	0	0	0	0	0	0	0	0	0	0	0	0	0	0	0	0
0	0	0	0	0	0	0	0	0	0	0	0	0	0	0	0	0	0	0
0	0	0	0	0	0	0	0	0	0	0	0	0	0	0	0	0	0	0
0	0	0	0	0	0	0	0	0	0	0	0	0	0	0	0	0	0	0
0	0	0	0	0	0	0	0	0	4	4	0	0	0	0	0	0	0	0
0	0	0	0	0	0	0	0	0	0	0	0	0	0	0	0	0	0	0

0	0	0	0	0	0	0	0	0	0	0	0	0	0	0	0	0	0	0
0	0	0	0	0	0	0	0	0	0	0	0	0	0	0	0	0	0	0
0	0	0	0	0	0	0	0	0	0	0	0	0	0	0	0	0	0	0
0	0	0	0	0	0	0	0	0	0	0	0	0	0	0	0	0	0	0
0	0	0	0	0	0	0	0	0	0	0	0	0	0	0	0	0	0	0
0	0	0	0	0	0	0	0	0	0	0	0	0	0	0	0	0	0	0
0	0	0	0	0	0	0	0	0	0	0	0	0	0	0	0	0	0	0
0	0	0	0	0	0	0	0	0	0	0	0	0	0	0	0	0	0	0
0	0	0	0	0	0	0	0	0	0	0	0	0	0	0	0	0	0	0
0	0	0	0	0	0	0	0	0	0	0	0	0	0	0	0	0	0	0
0	0	0	0	0	0	0	0	0	0	0	0	0	0	0	0	0	0	0
0	0	0	0	0	0	0	0	0	0	0	0	0	0	0	0	0	0	0
0	0	0	0	0	0	0	0	0	0	0	0	0	0	0	0	0	0	0
0	0	0	0	0	0	0	0	0	0	0	0	0	0	0	0	0	0	0
0	0	0	0	0	0	0	0	0	0	0	0	0	3	3	3	3	0	0
0	0	0	0	0	0	0	0	0	0	0	0	0	0	0	0	0	0	0
0	0	0	0	0	0	0	0	0	0	0	0	0	0	0	0	0	0	0
0	0	0	0	0	0	0	0	0	0	0	0	0	0	0	0	0	0	0
0	0	0	0	0	0	0	0	0	0	0	0	0	0	0	0	0	0	0
0	0	0	0	0	0	0	0	0	0	0	0	0	0	0	0	0	0	0
0	0	0	0	0	0	0	0	0	0	0	0	0	0	0	0	0	0	0
0	0	0	0	0	0	0	0	0	0	0	0	0	0	0	0	0	0	0
0	0	0	0	0	0	0	0	0	0	0	0	0	0	0	0	0	0	0
0	0	0	0	0	0	0	0	0	0	0	0	0	0	0	0	0	0	0
0	0	0	0	0	0	0	0	0	0	0	0	0	0	0	0	0	0	0
0	0	0	0	0	0	0	0	0	0	0	0	0	0	0	0	0	0	0
0	0	0	0	0	0	0	0	0	0	0	0	0	0	0	0	0	0	0
0	0	0	0	0	0	0	0	0	0	0	0	0	0	0	0	0	0	0
0	0	0	0	0	0	0	0	0	0	0	0	0	0	0	0	0	0	0
0	0	0	0	0	0	0	0	0	0	0	0	0	0	0	0	0	0	0
0	0	0	0	0	0	0	0	0	0	0	0	0	0	0	0	0	0	0
0	0	0	0	0	0	0	0	0	0	0	0	0	0	0	0	0	0	0
0	0	0	0	0	0	0	0	0	0	0	0	0	0	0	0	0	0	0
0	0	0	0	0	0	0	0	0	0	0	0	0	0	0	0	0	0	0
0	0	0	0	0	0	0	0	0	0	0	0	0	0	0	0	0	0	0
0	0	0	0	0	0	0	0	0	0	0	0	0	0	0	0	0	0	0
0	0	0	0	0	0	0	0	0	0	0	0	0	0	0	0	0	0	0

0	0	0	0	0	0	0	0	0	0	0	0	0	0	0	0	0	0	0
0	0	0	0	0	0	0	0	0	0	0	0	0	0	0	0	0	0	0
0	0	0	0	0	0	0	0	0	0	0	0	0	0	0	0	0	0	0
0	0	0	0	0	0	0	0	0	0	0	0	0	0	0	0	0	0	0
0	0	0	0	0	0	0	0	0	0	0	0	0	0	0	0	0	0	0
0	0	0	0	0	0	0	0	0	0	0	0	2	2	2	2	2	2	2
0	0	0	0	0	0	0	0	0	0	0	0	0	0	0	0	0	0	0
0	0	0	0	0	0	0	0	0	0	0	0	0	0	0	0	0	0	0
0	0	0	0	0	0	0	0	0	0	0	0	0	0	0	0	0	0	0
0	0	0	0	0	0	0	0	0	0	0	0	0	0	0	0	0	0	0
0	0	0	0	0	0	0	0	0	0	0	0	0	0	0	0	0	0	0
0	0	0	0	0	0	0	0	0	0	0	0	0	0	0	0	0	0	0
0	0	0	0	0	0	0	0	0	0	0	0	0	0	0	0	0	0	0
0	0	0	0	0	0	0	0	0	0	0	0	0	0	0	0	0	0	0
0	0	0	0	0	0	0	0	0	0	0	0	0	0	0	0	0	0	0
0	0	0	0	0	0	0	0	0	0	0	0	0	0	0	0	0	0	0
0	0	0	0	0	0	0	0	0	0	0	0	0	0	0	0	0	0	0
0	0	0	0	0	0	0	0	0	0	0	0	0	0	0	0	0	0	0
0	0	0	0	0	0	0	0	0	0	0	0	0	0	0	0	0	0	0
0	0	0	0	0	0	0	0	0	0	0	0	0	0	0	0	0	0	0
0	0	0	0	0	0	0	0	0	0									
Jan_DL131	1050	1322	540	453	43	565	110	104	380	274	872	197	191	207	69	198	145	210
190	21	143	0	101	138	135	92	130	106	111	165	212	26	99	382	80	132	147
83	23	163	58	62	296	134	29	93	90	112	202	0	66	75	176	150	63	81
81	43	175	44	61	20	49	79	60	28	138	0	0	44	11	23	0	52	79
42	67	43	95	0	34	51	49	0	74	37	0	0	0	0	103	20	0	69
21	52	42	0	0	0	0	0	0	7	0	56	0	0	10	0	0	0	0
0	0	0	2	0	0	0	0	0	0	14	0	0	0	0	0	0	0	0
0	23	0	11	0	0	0	0	0	6	0	0	0	0	0	0	0	0	0
0	0	0	0	0	6	32	0	0	0	0	0	0	10	0	0	19	19	0
0	27	20	0	0	0	0	0	0	0	0	0	180	0	0	0	10	0	10
48	0	0	0	0	0	0	0	49	3	0	0	0	0	6	0	0	0	0
0	0	0	0	0	0	7	0	0	0	0	0	0	0	0	0	0	7	0
35	0	0	14	0	13	0	0	3	0	0	0	0	0	23	0	0	0	0
0	0	0	0	0	0	0	0	0	9	0	0	0	0	10	0	0	5	0
8	0	21	0	0	3	0	0	0	0	0	0	0	0	0	0	5	0	0
0	0	0	0	0	0	0	0	0	0	0	14	0	0	0	0	0	0	0

0	0	0	0	0	0	0	0	0	0	9	0	7	0	0	0	33	0	0
0	0	0	0	0	0	0	13	6	0	0	0	0	0	0	0	0	4	41
0	0	0	0	5	8	0	0	0	0	0	0	0	0	0	0	0	0	0
0	0	0	0	0	0	4	0	0	0	0	0	8	0	0	0	0	0	0
0	0	0	0	0	0	0	0	0	0	0	0	0	0	0	0	0	0	0
0	0	0	0	0	0	0	0	0	7	0	0	0	0	0	0	0	0	0
0	0	0	0	0	0	0	0	7	0	0	0	0	0	0	0	0	0	0
0	0	0	0	0	0	0	0	0	0	0	0	0	0	0	0	0	0	0
0	0	0	0	0	0	0	0	0	0	0	0	0	0	0	0	0	0	0
0	0	0	17	0	0	0	0	0	0	0	5	0	0	0	0	0	0	0
0	0	0	0	0	0	0	0	0	0	0	0	0	0	0	0	0	0	0
0	0	0	0	0	0	0	0	0	0	0	0	0	0	0	0	0	0	0
0	0	0	0	0	0	0	0	0	0	0	0	0	0	0	0	0	0	0
0	2	0	0	0	0	0	0	0	0	0	0	0	0	0	0	0	0	0
0	0	0	0	0	0	0	0	0	0	0	0	16	0	0	0	0	0	0
17	0	0	0	0	0	0	0	0	0	0	14	0	0	0	0	0	0	0
0	0	0	0	0	0	0	0	0	0	0	0	0	0	0	0	0	0	0
0	18	0	0	0	0	0	0	0	0	0	0	0	0	0	0	0	0	0
0	0	0	0	0	0	0	0	0	0	0	0	0	0	0	0	0	0	0
0	0	0	0	0	0	0	0	0	0	0	0	0	0	0	0	0	0	0
0	0	0	0	0	0	0	0	0	0	0	0	0	0	0	0	0	0	0
0	0	0	0	0	0	0	0	0	0	0	0	0	0	0	0	0	0	0
0	0	0	0	0	0	0	0	0	0	0	0	0	0	0	0	0	0	0
0	0	0	0	0	0	0	0	0	0	0	0	0	0	0	0	0	0	0
0	0	0	0	0	0	0	0	0	0	0	0	0	0	0	0	0	0	0
0	0	0	0	0	0	0	0	0	0	0	0	0	0	0	0	0	0	0
0	0	0	0	0	0	0	0	0	0	0	0	0	0	0	0	0	0	0
0	0	0	0	0	0	0	0	0	0	0	0	0	0	0	0	0	0	0
0	0	0	0	0	0	0	0	0	0	0	0	0	0	0	0	0	0	0
0	0	0	0	0	0	0	0	0	0	0	0	0	0	0	0	0	0	0
0	0	0	0	0	0	0	0	0	0	0	0	0	0	0	0	0	0	0
0	0	0	0	0	0	0	0	0	0	0	0	0	0	0	0	0	0	0
0	0	0	0	0	0	0	0	6	0	0	0	0	0	0	0	0	0	0
0	0	0	0	0	0	0	0	0	0	0	0	0	0	0	0	0	0	0
0	0	0	0	0	0	0	0	0	0	0	0	0	0	0	0	0	0	0
0	0	0	0	0	0	0	0	0	0	0	0	0	0	0	0	0	0	0
0	0	0	0	0	0	0	0	0	0	0	0	0	0	0	0	0	0	0

0	0	0	0	0	0	0	0	0	0	0	0	0	0	0	0	0	0	0
0	0	0	0	0	0	0	0	0	0	0	0	0	0	0	0	0	0	0
0	0	0	0	0	0	0	0	0	0	0	0	0	0	0	0	0	0	0
0	0	0	0	0	0	0	0	0	0	0	0	0	0	0	0	0	0	0
0	0	0	0	0	0	0	0	0	0	0	0	0	0	0	0	0	0	0
0	0	0	0	0	0	0	0	0	0	0	0	0	0	0	0	0	0	0
0	0	0	0	0	0	0	0	0	0	0	0	0	0	0	0	0	0	0
0	0	0	0	0	0	0	0	0	0	0	0	0	0	0	0	0	0	0
0	0	0	0	0	0	0	0	0	0	0	0	0	0	0	0	0	0	0
3	0	0	0	0	0	0	0	0	0	0	0	0	0	0	0	0	0	0
0	0	0	0	0	0	0	0	0	0	0	0	0	0	0	0	0	0	0
0	0	0	0	0	0	0	0	0	0	0	0	0	0	0	0	0	0	0
0	0	0	0	0	0	0	0	0	0	0	0	0	0	0	0	0	0	0
0	0	0	0	0	0	0	0	0	0	0	0	0	0	0	0	0	0	0
0	0	0	0	0	0	0	0	0	0	0	0	0	0	0	0	0	0	0
0	0	0	0	0	0	0	0	0	0	0	0	0	0	0	0	0	0	0
0	0	0	0	0	0	0	0	0	0	0	0	0	0	0	0	0	0	0
0	0	0	0	0	0	0	0	0	0	0	0	0	0	0	0	0	0	0
0	0	0	0	0	0	0	0	0	0	0	0	0	0	0	0	0	0	0
0	0	0	0	0	0	0	0	0	0	0	0	0	0	0	0	0	0	0
0	0	0	0	0	0	0	0	0	0	0	0	0	0	0	0	0	0	0
0	0	0	0	0	0	0	0	0	0	0	0	0	0	0	0	0	0	0
0	0	0	0	0	0	0	0	0	0	0	0	0	0	0	9	9	0	0
0	0	0	0	0	0	0	0	0	0	0	0	0	0	0	0	0	0	0
0	0	0	0	0	0	0	0	0	0	0	0	0	0	0	0	0	0	0
0	0	0	0	0	0	0	0	0	0	0	0	0	0	0	0	0	0	0
0	0	0	0	0	0	0	0	0	0	0	8	0	0	0	0	0	0	0
0	0	0	0	0	0	0	0	0	0	0	0	0	0	0	0	0	0	0
0	0	0	0	0	0	0	0	0	0	0	0	0	0	0	0	0	0	0
0	0	0	0	0	0	0	0	0	0	0	0	0	0	0	0	0	0	0
0	0	0	0	0	0	0	0	0	0	0	0	0	0	0	0	0	0	0
0	0	0	0	0	0	0	0	0	0	0	0	0	0	0	0	0	0	0
0	0	0	0	0	0	0	0	0	0	0	0	0	0	0	0	0	0	0
0	0	0	0	0	0	0	0	0	0	0	0	0	0	0	0	0	0	0
0	0	0	0	0	0	0	0	0	0	0	0	0	0	0	0	0	0	0
0	0	0	0	0	0	0	0	0	0	0	0	0	0	0	0	0	0	0
0	0	0	0	0	0	0	0	0	0	0	0	0	0	0	0	0	0	0

0	0	0	0	0	0	0	0	0	0	0	0	0	0	0	0	0	0	0
0	0	0	0	0	0	0	0	0	0	0	0	0	0	0	0	0	0	0
0	0	0	0	0	0	0	0	0	0	0	0	0	0	0	0	0	0	0
0	0	0	0	0	0	0	0	0	0	0	0	0	0	0	0	0	0	0
0	0	0	0	0	0	0	0	0	0	0	0	0	0	0	0	0	0	0
0	0	0	0	0	0	0	0	0	0	0	0	0	0	0	0	0	0	0
0	0	0	0	0	0	0	0	0	0	0	0	0	0	0	0	0	0	0
0	0	0	0	0	0	0	0	0	0	0	0	0	0	0	0	0	0	0
0	0	0	5	0	0	0	0	0	0	0	0	0	0	0	0	0	0	0
0	0	0	0	0	0	0	0	0	0	0	0	0	0	0	0	0	0	0
0	0	0	0	0	0	0	0	0	0	0	0	0	0	0	0	0	0	0
0	0	0	0	0	0	0	0	0	0	0	0	0	0	0	0	0	0	0
0	0	0	0	0	0	0	0	0	0	0	0	0	0	0	0	0	0	0
0	0	0	0	0	0	0	0	0	0	0	0	0	0	0	0	0	0	0
0	0	0	0	0	0	0	0	0	0	0	0	0	0	0	0	0	0	0
0	0	0	0	0	0	0	0	0	0	0	0	0	0	0	0	0	0	0
0	0	0	0	0	0	0	0	0	0	0	0	0	0	0	0	0	0	0
0	0	0	0	0	0	0	0	0	0	0	0	0	0	0	0	0	0	0
0	0	4	0	0	0	0	0	0	0	0	0	0	0	0	0	0	0	0
0	0	0	0	0	0	0	0	0	0	0	0	0	0	0	0	0	0	0
0	0	0	0	0	0	0	0	0	0	0	0	0	0	0	0	0	0	0
0	0	0	0	0	0	0	0	0	0	0	0	0	0	0	0	0	0	0
0	0	0	0	0	0	0	0	0	0	0	0	0	0	0	0	0	0	0
0	0	0	0	0	0	0	0	0	0	0	0	0	0	0	0	0	0	0
0	0	0	0	0	0	0	0	0	0	0	0	0	0	0	0	0	0	0
0	0	0	0	0	0	0	0	0	0	0	0	0	0	0	0	0	0	0
0	0	0	0	0	0	0	0	0	0	0	0	0	0	0	0	0	0	0
0	0	0	0	0	0	0	0	0	0	0	0	0	0	0	0	0	0	0
0	0	0	0	0	0	0	0	0	0	0	0	0	0	0	0	0	0	0
0	0	0	0	0	0	0	0	0	0	0	0	0	0	0	0	0	0	0
0	0	0	0	0	0	0	0	0	0	0	0	0	0	0	0	0	0	0
0	0	0	0	0	0	0	0	0	0	0	0	0	0	0	0	0	0	0
0	0	0	0	0	0	0	0	0	0	0	0	0	0	0	0	0	0	0
0	0	0	0	0	0	0	3	3	3	3	0	0	0	0	0	0	0	0
0	0	0	0	0	0	0	0	0	0	0	0	0	0	0	0	0	0	0
0	0	0	0	0	0	0	0	0	0	0	0	0	0	0	0	0	0	0
0	0	0	0	0	0	0	0	0	0	0	0	0	0	0	0	0	0	0

0	0	0	0	0	0	0	0	0	0	0	0	0	0	0	0	0	0	0
0	0	0	0	0	0	0	0	0	0	0	0	0	0	0	0	0	0	0
0	0	0	0	0	0	0	0	0	0	0	0	0	0	0	0	0	0	0
0	0	0	0	0	0	0	0	0	0	0	0	0	0	0	0	0	0	0
0	0	0	0	0	0	0	0	0	0	0	0	0	0	0	0	0	0	0
0	0	0	0	0	0	0	0	0	0	0	0	0	0	0	0	0	0	0
0	0	0	0	0	0	0	0	0	0	0	0	0	0	0	0	0	0	0
0	0	0	0	0	0	0	0	0	0	0	0	0	0	0	0	0	0	0
0	0	0	0	0	0	0	0	0	0	0	0	0	0	0	0	0	0	0
0	0	0	0	0	0	0	0	0	0	0	0	0	0	0	0	0	0	0
0	0	0	0	0	0	0	0	0	0	0	0	0	0	0	0	0	0	0
0	0	0	0	0	0	0	0	0	0	0	0	0	0	0	0	0	0	0
0	0	0	0	0	0	0	0	0	0	0	0	0	0	0	0	0	0	0
0	0	0	0	0	0	0	0	0	0	0	0	0	0	0	0	0	0	0
0	0	0	0	0	0	0	0	0	0	0	0	0	0	0	0	0	0	0
0	0	0	0	0	0	0	0	0	0	0	0	0	0	0	0	0	0	0
0	0	0	0	0	0	0	0	0	0	0	0	0	0	0	0	0	0	0
0	0	0	0	0	0	0	0	0	0	0	0	0	0	0	0	0	0	0
0	0	0	0	0	0	0	0	0	0	0	0	0	0	0	0	0	0	0
0	0	0	0	0	0	0	0	0	0	0	0	0	0	0	0	0	0	0
0	0	0	0	0	0	0	0	0	0	0	0	0	0	0	0	0	0	0
0	0	0	0	0	0	0	0	0	0	0	0	0	0	0	0	0	0	0
0	0	0	0	0	0	0	0	0	0	0	0	0	0	0	0	0	0	0
0	0	0	0	0	0	0	0	0	0	0	0	0	0	2	2	2	2	2
2	2	2	2	0	0	0	0	0	0	0	0	0	0	0	0	0	0	0
0	0	0	0	0	0	0	0	0	0	0	0	0	0	0	0	0	0	0
0	0	0	0	0	0	0	0	0	0	0	0	0	0	0	0	0	0	0
0	0	0	0	0	0	0	0	0	0	0	0	0	0	0	0	0	0	0
0	0	0	0	0	0	0	0	0	0	0	0	0	0	0	0	0	0	0
0	0	0	0	0	0	0	0	0	0	0	0	0	0	0	0	0	0	0
0	0	0	0	0	0	0	0	0	0	0	0	0	0	0	0	0	0	0
0	0	0	0	0	0	0	0	0	0	0	0	0	0	0	0	0	0	0
0	0	0	0	0	0	0	0	0	0	0	0	0	0	0	0	0	0	0
0	0	0	0	0	0	0	0	0	0	0	0	0	0	0	0	0	0	0
0	0	0	0	0	0	0	0	0	0	0	0	0	0	0	0	0	0	0
0	0	0	0	0	0	0	0	0	0	0	0	0	0	0	0	0	0	0
0	0	0	0	0	0	0	0	0	0	0	0	0	0	0	0	0	0	0

0	0	0	0	0	0	0	0	0	0	0	0	0	0	0	0	0	0	0
0	0	0	0	0	0	0	0	0	0	0	0	0	0	0	0	0	0	0
0	0	0	0	0	0	0	0	0	0	0	0	0	0	0	0	0	0	0
0	0	0	0	0	0	0	0	0	0	0	0	0	0	0	0	0	0	0
0	0	0	0	0	0	0	0	0	0	0	0	0	0	0	0	0	0	0
0	0	0	0	0	0	0	0	0	0	0	0	0	0	0	0	0	0	0
0	0	0	0	0	0	0	0	0	0	0	0	0	0	0	0	0	0	0
0	0	0	0	0	0	0	0	0	0	0	0	0	0	0	0	0	0	0
0	0	0	0	0	0	0	0	0	0	0	0	0	0	0	0	0	0	0
0	0	0	0	0	0	0	0	0	0	0	0	0	0	0	0	0	0	0
0	0	0	0	0	0	0	0	0	0	0	0	0	0	0	0	0	0	0
0	0	0	0	0	0	0	0	0	0	0	1	0	0	0	0	0	0	0
0	0	0	0	0	0	0	0	0	0									
Jan_DL135	1188	1227	375	511	492	328	262	262	42	136	156	79	289	226	201	22	171	184
188	131	137	0	288	108	51	158	49	277	135	178	144	80	105	74	85	9	189
230	308	136	46	92	58	96	0	116	0	145	57	84	70	0	33	0	141	115
130	32	48	161	102	93	133	64	15	65	99	0	0	106	21	0	45	13	38
0	0	0	59	65	0	7	0	0	39	0	0	49	39	40	61	14	11	36
0	62	93	0	0	57	0	0	0	15	0	0	0	0	0	77	9	0	0
0	0	0	5	19	0	0	0	0	0	9	0	24	42	0	0	21	0	0
0	0	0	0	36	0	0	37	0	39	0	0	34	0	0	0	0	0	0
0	11	0	0	0	59	0	0	0	0	0	0	17	0	0	0	20	40	0
0	0	12	0	0	0	0	21	0	28	0	0	47	0	0	0	7	0	67
21	31	0	0	0	0	0	45	0	3	0	0	59	0	0	0	0	0	0
13	67	0	0	0	0	19	0	0	26	0	0	0	0	0	0	0	17	0
0	0	0	0	0	0	0	0	32	0	0	0	15	8	0	0	70	0	30
18	0	0	3	25	0	0	0	0	0	0	0	0	6	13	0	0	0	17
12	0	0	0	0	0	10	0	0	0	0	0	0	11	6	0	0	0	0
0	19	0	0	0	0	0	0	0	0	0	0	8	0	10	13	11	0	0
0	0	9	0	0	0	0	0	0	8	0	0	15	10	0	7	0	0	0
0	0	0	51	0	0	0	0	0	0	0	0	0	21	0	0	0	0	0
0	9	0	0	5	4	0	0	0	0	0	0	0	0	0	0	0	0	0
0	0	0	0	0	0	9	3	0	0	0	0	0	0	0	0	0	0	2
0	0	0	4	0	0	0	0	0	0	0	0	12	0	0	0	0	0	0
0	0	0	0	0	0	0	0	0	0	0	0	0	2	0	0	0	0	0
0	0	23	0	0	0	0	0	0	0	0	0	0	0	0	0	0	0	0
0	0	0	0	0	0	17	17	0	0	0	0	0	0	0	0	0	0	0

0	10	12	0	0	0	0	0	0	0	0	0	0	0	0	0	0	0	0
0	0	0	6	0	0	12	0	0	0	0	0	16	0	0	0	0	0	0
0	0	0	0	0	0	0	0	0	15	0	0	0	0	0	0	10	0	0
0	0	0	0	0	0	0	0	0	0	0	0	0	0	0	0	0	0	0
0	0	0	0	0	0	0	0	0	0	0	0	0	0	0	0	0	0	0
0	8	0	0	0	0	0	0	0	0	0	0	0	0	0	0	0	14	0
0	0	0	6	0	0	0	0	0	13	0	0	0	0	0	0	19	0	3
0	0	0	0	0	0	0	0	0	17	0	0	0	0	0	0	0	0	0
0	0	0	0	0	0	0	0	0	0	0	0	0	0	0	0	0	0	0
0	0	0	0	0	0	0	0	0	0	0	0	0	0	0	0	0	0	0
0	0	0	0	0	0	0	0	0	0	0	0	0	0	0	0	0	0	0
0	0	0	0	0	0	0	0	0	0	0	0	0	0	0	0	0	0	0
0	0	0	0	0	0	8	0	0	0	0	0	0	0	0	0	0	0	0
0	3	0	0	0	0	0	0	0	0	0	0	0	0	0	0	0	0	0
0	0	0	0	0	0	0	0	0	0	0	0	0	0	0	12	0	0	0
0	0	28	0	0	0	0	0	0	0	0	0	0	0	0	0	0	0	0
0	0	0	0	0	0	0	0	0	0	0	0	0	0	0	19	0	0	0
0	0	0	0	0	0	0	0	0	0	4	0	0	0	0	0	0	0	0
0	0	0	0	0	0	0	0	0	0	0	0	0	0	0	0	0	0	0
0	0	0	0	0	0	0	0	0	0	0	0	0	0	0	0	0	0	0
0	0	0	0	0	0	0	0	0	0	0	0	0	0	0	0	0	0	0
0	0	0	0	0	0	0	0	0	0	0	0	0	0	0	0	0	0	0
0	0	0	0	0	0	0	0	0	0	0	0	0	0	0	0	0	0	0
0	0	0	0	0	0	0	0	0	0	0	0	0	0	0	0	0	0	0
0	0	0	0	0	0	0	0	0	0	0	0	0	0	0	0	0	0	0
0	0	0	0	0	0	0	0	0	0	0	0	0	0	11	0	0	0	0
0	0	0	0	0	0	0	0	0	0	0	0	0	0	0	0	0	0	0
0	0	0	0	0	0	0	0	0	0	0	0	0	0	0	0	0	0	0
0	0	0	0	0	0	0	0	0	0	0	0	0	0	0	0	0	0	0
0	0	0	0	0	0	18	0	0	0	0	0	0	0	0	0	0	0	0
0	0	0	0	0	0	0	0	0	0	0	0	0	0	0	0	0	0	0
0	0	0	0	9	0	0	0	0	0	0	0	0	4	0	0	0	0	0
0	0	0	0	0	0	0	0	0	0	0	0	0	0	0	0	0	0	0
0	0	0	0	0	0	0	0	0	0	0	0	0	0	0	0	0	0	0
0	0	0	0	0	0	0	0	0	0	0	0	0	0	0	0	0	0	0
0	0	0	0	0	0	0	0	0	0	0	0	0	0	0	0	0	0	0
0	0	0	0	0	0	0	0	0	0	0	0	0	0	0	0	0	0	0

0	0	0	0	0	0	0	0	0	0	0	0	0	0	0	0	0	0	0
0	0	0	0	0	0	0	0	0	0	0	0	0	0	0	0	0	0	0
0	0	0	0	0	0	0	0	0	0	0	0	0	0	0	0	0	0	0
0	0	0	0	0	0	0	0	0	0	0	0	0	0	0	0	0	0	0
0	0	0	0	0	0	0	0	0	0	0	0	0	0	0	0	0	0	0
0	0	0	0	0	0	0	0	0	0	0	0	0	0	0	0	0	0	0
0	0	0	0	0	0	0	0	0	0	0	0	0	0	0	0	0	0	0
0	0	0	0	0	0	0	0	0	0	0	0	0	0	0	0	0	0	0
0	0	0	0	0	0	0	0	0	0	0	0	0	0	0	0	0	0	0
0	0	0	0	0	0	0	0	0	0	0	0	0	0	0	0	0	0	0
0	0	0	0	0	0	0	0	0	0	0	0	0	0	0	0	0	0	0
0	0	0	0	0	0	0	0	0	0	0	6	0	0	0	0	0	0	0
0	0	0	0	0	0	0	0	0	0	0	0	0	0	0	0	0	0	0
0	0	0	0	0	0	0	0	0	0	0	0	0	0	0	0	0	0	0
0	0	0	0	0	0	9	0	0	0	0	0	0	0	0	0	0	0	0
0	0	0	0	0	0	0	0	0	0	0	0	0	0	0	7	0	0	0
0	0	0	0	0	0	0	0	0	0	0	0	0	0	0	0	0	0	0
0	0	0	0	0	0	0	0	0	0	0	0	0	0	0	0	0	0	0
0	0	0	0	0	0	8	0	0	0	0	0	0	0	0	0	0	0	0
0	0	0	0	0	0	0	0	0	0	0	0	0	0	0	0	0	0	0
0	0	0	0	0	0	0	0	0	0	0	0	0	0	0	0	0	0	0
0	0	0	0	0	0	0	0	0	0	0	0	0	0	0	0	0	0	0
0	0	0	0	0	0	0	0	0	0	0	0	0	0	0	0	0	0	0
0	0	0	0	0	0	0	0	0	0	0	0	0	0	0	0	0	0	0
0	0	0	0	0	0	0	0	0	0	0	0	0	0	0	0	0	0	0
0	0	0	0	0	0	0	0	0	0	0	0	0	0	0	0	0	0	0
0	0	0	0	0	0	0	0	0	0	0	0	0	0	0	0	0	0	0
0	0	0	0	0	0	0	0	0	0	0	0	0	0	0	0	0	0	0
0	0	0	0	0	0	0	0	0	0	0	0	0	6	0	0	0	0	0
0	0	0	0	0	0	0	0	0	0	0	0	0	0	0	0	0	0	0
0	0	0	0	0	0	0	0	0	0	0	0	0	0	0	0	0	0	0
0	0	0	0	0	0	0	0	0	0	0	0	0	0	0	0	0	0	0
0	0	0	0	0	0	0	0	0	0	0	0	0	0	0	0	0	0	0
0	0	0	0	0	0	0	0	0	0	0	0	0	0	0	0	0	0	0
0	0	0	0	0	0	0	0	0	0	0	0	0	0	0	0	0	0	0
0	0	0	0	0	0	0	0	0	0	0	0	0	0	0	0	0	0	0
0	0	0	0	0	0	0	0	0	5	0	0	0	0	0	0	0	0	0

0	0	0	0	0	0	0	0	0	0	0	0	0	0	0	0	0	0	0
0	0	0	0	0	0	0	0	0	0	0	0	0	0	0	0	0	0	0
0	0	0	0	0	0	0	0	0	0	0	0	0	0	0	0	0	0	0
0	0	0	0	0	0	0	0	0	0	0	0	0	0	0	0	0	0	0
0	0	0	0	0	0	0	0	0	0	0	0	0	0	0	0	0	0	0
0	0	0	0	0	0	0	0	0	0	0	0	0	0	0	0	0	0	0
0	0	0	0	0	0	0	0	0	0	0	0	0	0	0	0	0	0	0
0	0	0	0	0	0	0	0	0	0	0	0	0	0	0	0	0	0	0
0	0	0	0	0	0	0	0	0	0	0	0	0	0	0	0	0	0	0
0	0	4	4	4	4	0	0	0	0	0	0	0	0	0	0	0	0	0
0	0	0	0	0	0	0	0	0	0	0	0	0	0	0	0	0	0	0
0	0	0	0	0	0	0	0	0	0	0	0	0	0	0	0	0	0	0
0	0	0	0	0	0	0	0	0	0	0	0	0	0	0	0	0	0	0
0	0	0	0	0	0	0	0	0	0	0	0	0	0	0	0	0	0	0
0	0	0	0	0	0	0	0	0	0	0	0	0	0	0	0	0	0	0
0	0	0	0	0	0	0	0	0	0	0	0	0	0	0	0	0	0	0
0	0	0	0	0	0	0	0	0	0	0	0	0	0	0	0	0	0	0
0	0	0	0	0	0	0	0	0	0	0	0	0	0	0	0	0	0	0
0	0	0	0	0	0	0	0	0	0	0	0	0	0	0	0	0	0	0
0	0	0	0	0	0	0	0	0	0	0	0	0	0	0	0	0	0	0
0	0	0	0	0	0	0	0	0	0	0	0	0	0	0	0	0	0	0
0	0	0	0	0	0	0	0	0	0	0	0	0	0	0	0	0	0	0
0	0	0	0	0	0	0	0	0	0	0	0	0	0	0	0	0	0	0
0	0	0	0	0	0	0	0	0	0	0	0	0	0	0	0	0	0	0
0	0	0	0	0	0	0	0	3	3	3	3	3	0	0	0	0	0	0
0	0	0	0	0	0	0	0	0	0	0	0	0	0	0	0	0	0	0
0	0	0	0	0	0	0	0	0	0	0	0	0	0	0	0	0	0	0
0	0	0	0	0	0	0	0	0	0	0	0	0	0	0	0	0	0	0
0	0	0	0	0	0	0	0	0	0	0	0	0	0	0	0	0	0	0
0	0	0	0	0	0	0	0	0	0	0	0	0	0	0	0	0	0	0
0	0	0	0	0	0	0	0	0	0	0	0	0	0	0	0	0	0	0
0	0	0	0	0	0	0	0	0	0	0	0	0	0	0	0	0	0	0
0	0	0	0	0	0	0	0	0	0	0	0	0	0	0	0	0	0	0
0	0	0	0	0	0	0	0	0	0	0	0	0	0	0	0	0	0	0
0	0	0	0	0	0	0	0	0	0	0	0	0	0	0	0	0	0	0
0	0	0	0	0	0	0	0	0	0	0	0	0	0	0	0	0	0	0
0	0	0	0	0	0	0	0	0	0	0	0	0	0	0	0	0	0	0

0	0	0	0	0	0	0	0	0	0	0	0	0	0	0	0	0	0	0
0	0	0	0	0	0	0	0	0	0	0	0	0	0	0	0	0	0	0
0	0	0	0	0	0	0	0	0	0	0	0	0	0	0	0	0	0	0
0	0	0	0	0	0	0	0	0	0	0	0	0	0	0	0	0	0	0
0	0	0	0	0	0	0	0	0	0	0	0	0	0	0	0	0	0	0
0	0	0	0	0	0	0	0	0	0	0	0	0	0	0	0	0	0	0
0	0	0	0	0	0	0	0	0	0	0	0	0	0	0	0	0	0	0
0	0	0	0	0	0	0	0	0	0	0	0	0	0	0	0	0	0	0
0	0	0	0	0	0	0	0	0	0	0	0	0	0	0	0	0	0	0
0	0	0	0	0	0	0	0	0	0	0	0	0	0	0	0	0	0	0
0	0	0	0	0	0	0	0	0	0	0	0	0	0	0	0	0	0	0
0	0	0	0	0	0	0	0	0	0	0	0	0	0	0	0	0	0	0
0	0	0	0	0	0	0	0	0	0	0	0	0	0	0	0	0	0	0
0	0	0	0	0	0	0	0	0	0	0	2	2	2	2	0	0	0	0
0	0	0	0	0	0	0	0	0	0	0	0	0	0	0	0	0	0	0
0	0	0	0	0	0	0	0	0	0	0	0	0	0	0	0	0	0	0
0	0	0	0	0	0	0	0	0	0	0	0	0	0	0	0	0	0	0
0	0	0	0	0	0	0	0	0	0	0	0	0	0	0	0	0	0	0
0	0	0	0	0	0	0	0	0	0	0	0	0	0	0	0	0	0	0
0	0	0	0	0	0	0	0	0	0	0	0	0	0	0	0	0	0	0
0	0	0	0	0	0	0	0	0	0	0	0	0	0	0	0	0	0	0
0	0	0	0	0	0	0	0	0	0	0	0	0	0	0	0	0	0	0
0	0	0	0	0	0	0	0	0	0	0	0	0	0	0	0	0	0	0
0	0	0	0	0	0	0	0	0	0	0	0	0	0	0	0	0	0	0
0	0	0	0	0	0	0	0	0	0	0	0	0	0	0	0	0	0	0
0	0	0	0	0	0	0	0	0	0	0	0	0	0	0	0	0	0	0
0	0	0	0	0	0	0	0	0	0	0	0	0	0	0	0	0	0	0
0	0	0	0	0	0	0	0	0	0	0	0	0	0	0	0	0	0	0
0	0	0	0	0	0	0	0	0	0	0	0	0	0	0	0	0	0	0
0	0	0	0	0	0	0	0	0	0	0	0	0	0	0	0	0	0	0
0	0	0	0	0	0	0	0	0	0	0	0	0	0	0	0	0	0	0
0	0	0	0	0	0	0	0	0	0	0	0	0	0	0	0	0	0	0
0	0	0	0	0	0	0	0	0	0	0	0	0	0	0	0	0	0	0
0	0	0	0	0	0	0	0	0	0	0	0	0	0	0	0	0	0	0
0	0	0	0	0	0	0	0	0	0	0	0	0	0	0	0	0	0	0
0	0	0	0	0	0	0	0	0	0	0	0	0	0	0	0	0	0	0
0	0	0	0	0	0	0	0	0	0	0	0	0	0	0	0	0	0	0

0	0	0	0	0	0	0	0	0	0	0	0	0	0	0	0	0	0	0
0	0	0	0	0	0	0	0	0	0	0	0	0	0	0	0	0	0	0
0	0	0	0	0	0	0	0	0	0	0	0	0	0	0	0	0	0	0
0	0	0	0	0	0	0	0	0	0	1	0	0	0	0	0	0	0	0
0	0	0	0	0	0	0	0	0	0									
Jan_DL136	1500	1619	670	580	119	402	261	259	203	284	342	82	240	309	251	155	300	139
174	231	138	102	148	147	67	135	54	284	159	191	153	152	95	166	98	44	137
284	26	150	97	112	130	108	0	81	51	186	74	32	62	40	65	0	67	99
81	0	63	64	85	34	87	57	29	106	28	76	0	76	6	128	52	49	0
20	0	19	56	64	22	22	31	0	51	0	43	0	0	57	0	86	0	48
19	35	57	0	0	32	0	0	0	20	0	0	0	11	16	30	12	0	0
0	5	0	22	4	0	0	0	0	0	0	0	11	0	31	0	14	0	0
0	19	0	0	46	0	0	57	0	34	0	0	0	50	21	0	0	0	0
0	9	0	0	0	6	0	0	0	0	7	0	25	18	0	0	16	0	0
0	0	15	0	0	0	0	18	0	34	24	0	56	0	0	0	14	0	56
0	0	0	0	0	0	0	15	0	0	0	0	0	0	0	0	0	0	0
0	0	0	0	0	0	0	0	0	24	0	0	0	0	0	0	0	0	19
0	0	23	0	0	0	0	0	0	0	0	0	8	9	49	0	34	0	5
0	0	0	0	0	0	0	0	0	0	0	0	0	5	12	0	0	0	0
19	0	0	0	0	19	0	0	0	0	3	0	0	0	0	0	0	0	0
0	3	0	0	0	0	0	0	0	0	0	0	5	0	11	0	0	3	0
0	0	7	0	0	0	0	0	0	0	23	0	12	0	0	13	0	0	0
9	0	4	0	0	0	0	10	0	0	0	0	0	0	0	0	0	0	0
0	0	32	0	0	0	0	0	0	9	0	24	0	12	0	0	0	0	0
0	0	4	0	0	0	0	0	0	0	0	0	0	0	10	0	0	0	4
0	0	0	0	0	0	0	0	0	0	0	0	0	0	0	0	0	0	0
0	0	0	0	0	0	0	0	0	0	0	0	0	0	0	0	0	0	0
0	0	0	0	0	0	0	0	0	0	0	6	10	0	0	0	0	0	0
0	0	0	0	0	27	0	0	0	0	0	0	0	0	0	0	0	0	5
0	0	0	0	0	0	0	0	0	0	0	18	0	0	0	0	0	0	0
0	0	0	8	0	0	0	4	0	0	0	0	0	0	0	0	0	0	0
0	0	7	0	0	0	0	0	0	0	0	0	0	0	0	0	4	5	0
0	0	0	0	0	0	0	0	0	0	0	0	0	0	0	0	0	0	0
0	0	0	0	0	0	0	0	0	0	0	0	0	0	6	0	0	0	0
0	5	0	0	0	0	0	0	0	0	0	0	0	0	0	0	0	0	0
0	0	0	0	0	0	0	0	0	0	0	0	0	0	0	0	0	0	0
0	0	0	0	0	0	10	0	0	0	0	0	0	0	0	0	0	0	0

0	0	0	0	0	0	0	0	0	0	0	0	0	0	0	0	0	0	0
0	0	0	0	10	0	6	0	0	0	0	0	0	0	0	0	0	0	10
0	0	0	0	0	0	0	0	0	0	0	2	0	0	0	0	0	0	0
0	0	0	0	0	0	0	0	0	0	0	0	0	0	9	0	0	0	13
0	0	0	0	0	0	0	0	0	0	0	0	0	0	0	0	0	0	0
0	0	24	0	0	0	0	0	0	0	0	0	0	0	0	0	0	0	0
0	0	0	0	0	0	0	0	0	0	0	0	0	0	0	0	0	0	0
0	0	0	0	0	0	0	0	0	0	0	0	0	0	0	0	0	0	0
0	0	0	0	0	0	0	0	0	0	0	0	0	0	0	0	0	0	14
0	0	0	0	0	0	0	0	0	0	3	0	0	0	0	0	0	0	0
0	0	0	0	0	0	0	0	0	0	0	0	7	0	0	0	0	0	0
0	0	4	0	0	0	0	0	0	0	0	0	0	0	0	0	0	0	0
0	0	0	0	0	0	0	0	0	0	0	0	0	0	0	0	0	0	0
0	0	0	0	0	0	0	0	0	0	0	0	0	0	0	0	0	0	0
0	0	0	0	0	0	0	0	0	0	0	0	0	0	0	0	0	0	0
0	0	0	0	0	0	0	0	0	0	0	0	0	0	0	0	0	0	0
0	0	0	0	0	0	0	0	0	0	0	0	0	0	0	0	0	0	0
0	0	0	0	0	0	0	0	0	0	0	0	0	0	0	0	0	0	0
0	0	0	0	0	0	0	0	0	0	0	0	0	0	0	0	0	0	0
0	0	0	0	0	0	0	0	0	0	0	0	0	0	0	0	0	0	0
0	0	0	0	0	0	0	0	0	0	0	0	0	0	0	0	0	0	0
0	0	0	0	0	0	0	0	0	0	0	0	0	0	0	0	0	0	0
0	0	0	0	0	0	0	0	0	0	0	0	0	0	0	0	0	0	0
0	0	0	0	0	0	0	0	0	0	0	0	0	0	0	0	0	0	0
0	0	0	0	0	0	0	0	0	0	0	0	0	0	0	0	0	0	0
0	0	0	0	0	0	0	0	0	0	0	0	0	0	0	0	0	0	0
0	0	0	0	0	0	0	0	0	0	0	0	0	0	0	0	0	0	0
0	0	0	0	0	0	0	0	0	0	0	0	0	0	0	0	0	0	0
0	0	0	0	0	0	0	0	0	0	0	0	0	0	0	0	0	0	0
0	0	0	0	0	0	0	0	0	0	0	0	0	0	0	0	0	0	0
0	0	0	0	0	0	0	0	0	0	0	0	0	0	0	0	0	0	0
0	0	0	0	0	0	0	0	0	0	0	0	0	0	0	0	0	0	0
0	0	0	0	0	0	0	0	0	0	0	0	0	0	0	0	0	0	0
0	0	0	0	0	0	0	0	0	0	0	0	0	0	0	0	0	0	0
0	0	0	0	0	0	0	0	0	0	0	0	0	0	0	0	8	0	0
0	0	0	0	0	0	0	0	0	0	0	0	0	0	0	0	0	0	0
0	0	0	0	0	0	0	0	0	0	0	0	0	0	0	0	0	0	0

0	0	0	0	0	0	0	0	0	0	0	0	0	0	0	0	0	0	0
0	0	0	0	0	0	0	0	0	0	0	0	0	0	0	0	0	0	0
0	0	0	0	0	0	0	0	0	0	0	0	0	0	0	0	0	0	0
0	0	0	0	0	0	0	0	0	0	0	0	0	0	0	11	0	0	0
0	0	0	0	0	0	0	0	0	0	0	0	0	0	0	0	0	0	0
0	0	0	0	0	0	0	0	0	0	0	0	0	0	0	0	0	0	0
0	0	0	0	0	0	0	0	0	0	0	0	0	0	0	0	0	0	0
0	0	0	0	0	0	0	0	0	0	0	0	0	0	0	0	0	0	0
0	0	0	0	0	0	0	0	0	0	0	0	0	0	0	0	0	0	0
0	0	9	0	0	0	0	0	0	0	0	0	0	0	0	0	0	0	0
0	0	0	0	0	0	0	0	0	0	0	0	0	0	0	0	0	0	0
0	0	0	0	0	0	0	0	0	0	0	0	0	0	0	0	0	0	0
0	0	0	0	0	0	0	0	0	0	0	0	0	0	0	0	0	0	0
0	0	0	0	0	0	0	0	0	0	0	0	0	0	0	0	0	0	0
0	0	0	0	0	0	0	0	0	0	0	0	0	0	0	0	0	0	0
0	0	0	0	0	0	0	0	0	0	0	0	0	0	0	0	0	0	0
0	0	0	0	0	0	0	0	0	0	0	0	0	0	0	0	0	0	0
0	0	0	0	0	0	0	0	0	0	0	0	0	0	0	7	7	7	0
0	0	0	0	0	0	0	0	0	0	0	0	0	0	0	0	0	0	0
0	0	0	0	0	0	0	0	0	0	0	0	0	0	0	0	0	0	0
0	0	0	0	0	0	0	0	0	0	0	0	0	0	0	0	0	0	0
0	0	0	0	0	0	0	0	0	0	0	0	0	0	0	0	0	0	0
0	0	0	0	0	0	0	0	0	0	0	0	0	0	0	0	0	0	0
0	0	0	0	0	0	0	0	0	0	0	0	0	0	0	0	0	0	0
0	0	0	0	0	0	0	0	0	0	0	0	0	0	0	0	0	0	0
0	0	0	0	0	0	0	0	0	0	0	0	0	0	0	0	0	0	0
0	0	0	0	0	0	0	0	0	0	0	0	0	0	0	0	0	0	0
0	0	0	0	0	0	0	0	0	0	0	0	0	0	0	0	0	0	0
0	0	0	0	0	0	0	0	0	0	0	0	0	0	0	0	0	0	0
0	0	0	0	0	0	0	0	0	0	0	0	0	0	0	0	0	0	0
0	0	0	0	0	0	0	0	0	0	0	0	0	0	0	0	0	0	0
0	0	0	0	0	0	0	0	0	0	0	0	0	0	0	0	0	0	0
0	0	0	0	0	0	0	0	0	0	0	0	0	0	0	0	0	0	0
0	0	0	0	0	0	0	0	0	0	0	0	0	0	0	0	0	0	0
0	0	0	0	0	0	0	0	0	0	0	0	0	0	0	0	0	0	0
0	0	0	0	0	0	0	0	0	0	0	0	0	0	0	0	0	0	0
0	0	0	0	0	0	0	0	0	0	0	0	0	0	0	0	0	0	0

0	0	0	0	0	0	0	0	0	0	0	0	0	0	0	0	0	0	0
0	0	0	0	0	0	0	0	0	0	0	0	0	0	0	0	0	0	0
0	0	0	0	0	0	0	0	0	0	0	0	0	0	0	0	0	0	0
0	0	0	0	0	0	0	0	0	0	0	0	0	0	0	0	0	0	0
0	0	0	0	0	0	0	0	0	0	0	0	0	0	0	0	0	0	0
0	0	0	4	0	0	0	0	0	0	0	0	0	0	0	0	0	0	0
0	0	0	0	0	0	0	0	0	0	0	0	0	0	0	0	0	0	0
0	0	0	0	0	0	0	0	0	0	0	0	0	0	0	0	0	0	0
0	0	0	0	0	0	0	0	0	0	0	0	0	0	0	0	0	0	0
0	0	0	0	0	0	0	0	0	0	0	0	0	0	0	0	0	0	0
0	0	0	0	0	0	0	0	0	0	0	0	0	0	0	0	0	0	0
0	0	0	0	0	0	0	0	0	0	0	0	0	0	0	0	0	0	0
0	0	0	0	0	0	0	0	0	0	0	0	0	0	0	0	0	0	0
0	0	0	0	0	0	0	0	0	0	0	0	0	0	0	0	0	0	0
0	0	0	0	0	0	0	0	0	0	0	0	0	0	0	0	0	0	0
0	0	0	0	0	0	0	0	0	0	0	0	0	0	0	0	0	0	0
0	0	0	0	0	0	0	0	0	0	0	0	0	0	0	0	0	0	0
0	0	0	0	0	0	0	0	0	0	0	0	0	0	0	0	0	0	0
0	0	0	0	0	0	0	0	0	0	0	0	0	0	0	0	0	0	0
0	0	0	0	0	0	0	0	0	0	0	0	0	0	0	0	0	0	0
0	0	0	0	0	0	0	0	0	0	0	0	0	0	0	0	0	0	0
0	0	0	0	0	0	0	0	0	0	0	3	3	3	3	3	3	3	3
0	0	0	0	0	0	0	0	0	0	0	0	0	0	0	0	0	0	0
0	0	0	0	0	0	0	0	0	0	0	0	0	0	0	0	0	0	0
0	0	0	0	0	0	0	0	0	0	0	0	0	0	0	0	0	0	0
0	0	0	0	0	0	0	0	0	0	0	0	0	0	0	0	0	0	0
0	0	0	0	0	0	0	0	0	0	0	0	0	0	0	0	0	0	0
0	0	0	0	0	0	0	0	0	0	0	0	0	0	0	0	0	0	0
0	0	0	0	0	0	0	0	0	0	0	0	0	0	0	0	0	0	0
0	0	0	0	0	0	0	0	0	0	0	0	0	0	0	0	0	0	0
0	0	0	0	0	0	0	0	0	0	0	0	0	0	0	0	0	0	0
0	0	0	0	0	0	0	0	0	0	0	0	0	0	0	0	0	0	0
0	0	0	0	0	0	0	0	0	0	0	0	0	0	0	0	0	0	0
0	0	0	0	0	0	0	0	0	0	0	0	0	0	0	0	0	0	0
0	0	0	0	0	0	0	0	0	0	0	0	0	0	0	0	0	0	0
0	0	0	0	0	0	0	0	0	0	0	0	0	0	0	0	0	0	0
0	0	0	0	0	0	0	0	0	0	0	0	0	0	0	0	0	0	0

0	0	0	0	0	0	0	0	0	0	0	0	0	0	0	0	0	0	0
0	0	0	0	0	0	0	0	0	0	0	0	0	0	0	0	0	0	0
0	0	0	0	0	0	0	0	0	0	0	0	0	0	0	0	0	0	0
0	0	0	0	0	0	0	0	0	0	0	0	0	0	0	0	0	0	0
0	0	0	0	0	0	0	0	0	0	0	0	0	0	0	0	0	0	0
0	0	0	0	0	0	0	0	0	0	0	0	0	0	0	0	0	0	0
0	0	0	0	0	0	0	0	0	0	0	0	0	0	0	0	0	0	0
0	0	0	0	0	0	0	0	0	0	0	0	0	0	0	0	0	0	0
0	0	0	0	0	0	0	0	0	0	0	0	0	0	0	0	0	0	0
0	0	0	0	0	0	0	0	0	0	0	0	0	0	0	0	0	0	0
0	0	0	0	0	0	0	0	0	0	0	0	0	0	0	0	0	0	0
0	0	0	0	0	0	0	0	0	2	2	2	2	2	2	2	2	2	2
0	0	0	0	0	0	0	0	0	0	0	0	0	0	0	0	0	0	0
0	0	0	0	0	0	0	0	0	0	0	0	0	0	0	0	0	0	0
0	0	0	0	0	0	0	0	0	0	0	0	0	0	0	0	0	0	0
0	0	0	0	0	0	0	0	0	0	0	0	0	0	0	0	0	0	0
0	0	0	0	0	0	0	0	0	0	0	0	0	0	0	0	0	0	0
0	0	0	0	0	0	0	0	0	0	0	0	0	0	0	0	0	0	0
0	0	0	0	0	0	0	0	0	0	0	0	0	0	0	0	0	0	0
0	0	0	0	0	0	0	0	0	0	0	0	0	0	0	0	0	0	0
0	0	0	0	0	0	0	0	0	0	0	0	0	0	0	0	0	0	0
0	0	0	0	0	0	0	0	0	0	0	0	0	0	0	0	0	0	0
0	0	0	0	0	0	0	0	0	0	0	0	0	0	0	0	0	0	0
0	0	0	0	0	0	0	0	0	0	0	0	0	0	0	0	0	0	0
0	0	0	0	0	0	0	0	0	0	0	0	0	0	0	0	0	0	0
0	0	0	0	0	0	0	0	0	0	0	0	0	0	0	0	0	0	0
0	0	0	0	0	0	0	0	0	0	0	0	0	0	0	0	0	0	0
0	0	0	0	0	0	0	0	0	0	0	0	0	0	0	0	0	0	0
0	0	0	0	0	0	0	0	0	0	0	0	0	0	0	0	0	0	0
0	0	0	0	0	0	0	0	0	0	0	0	0	0	0	0	0	0	0
0	0	0	0	0	0	0	0	0	0	0	0	0	0	0	0	0	0	0
0	0	0	0	0	0	0	0	0	0	0	0	0	0	0	0	0	0	0
0	0	0	0	0	0	0	0	0	0	0	0	0	0	0	0	0	0	0
0	0	0	0	0	0	0	0	0	0	0	0	0	1	0	0	0	0	0
0	0	0	0	0	0	0	0	0	0									
Jan_DL137	1097	1514	172	368	689	287	334	377	429	78	377	90	161	463	0	23	458	144
151	26	237	0	229	208	32	132	32	112	199	597	109	56	63	173	94	0	526
88	176	157	210	38	132	70	0	100	0	126	36	75	67	76	28	83	102	324

337	13	0	56	92	0	111	35	13	10	45	0	0	80	0	0	19	0	58
35	0	48	123	47	0	21	50	24	0	116	66	13	0	49	0	0	133	24
23	13	97	0	19	12	0	0	0	4	25	0	0	0	0	29	0	0	0
0	0	20	5	20	0	0	0	0	57	0	0	18	47	0	0	0	0	0
59	27	0	0	0	0	26	0	69	39	0	0	8	0	32	0	0	0	0
0	68	0	0	33	0	0	0	0	0	0	0	8	0	0	0	16	0	27
0	0	13	0	0	0	0	0	0	0	16	0	57	0	0	0	0	0	37
16	0	0	0	0	0	0	0	0	9	0	0	0	0	0	0	0	0	0
31	0	0	0	0	0	0	0	0	0	0	0	0	0	0	0	0	14	0
0	0	0	11	0	0	0	0	0	0	0	0	0	0	0	0	0	0	8
13	0	0	0	0	0	0	0	0	0	0	0	0	14	8	0	0	0	0
0	0	0	0	0	0	0	0	0	0	0	0	0	0	0	0	48	0	0
0	22	0	0	0	0	0	0	0	0	0	0	0	0	0	22	0	0	0
0	16	0	0	0	0	0	0	0	7	0	4	0	0	0	0	0	0	0
0	0	18	0	0	0	0	10	35	14	0	0	0	0	0	14	0	0	5
8	18	0	0	0	5	0	0	0	13	0	0	0	0	0	0	0	0	0
21	0	0	0	0	0	7	0	0	0	0	0	0	0	0	0	0	0	0
0	0	0	0	0	11	0	0	0	0	0	0	0	0	0	15	0	0	0
0	0	0	0	0	0	0	0	0	0	0	0	0	0	0	0	0	0	0
0	0	0	0	0	0	0	0	0	0	0	0	0	0	0	0	0	0	0
0	0	0	0	0	0	47	0	0	0	0	0	0	0	0	0	7	0	0
2	0	0	0	0	0	0	0	0	0	0	0	0	0	0	0	0	0	0
0	0	0	0	0	0	0	7	0	0	0	0	0	0	0	0	0	0	2
0	0	0	0	0	0	0	0	0	0	0	0	0	0	0	0	0	0	49
0	0	0	0	0	0	0	0	0	0	0	0	0	0	0	0	0	0	0
0	0	0	0	0	0	0	0	0	0	18	0	10	0	13	0	0	0	0
13	0	5	0	0	0	0	0	0	0	0	0	0	0	0	0	0	0	0
0	0	0	0	0	0	0	0	28	0	0	0	0	0	0	0	0	0	0
0	0	0	0	0	0	0	15	0	0	26	0	0	0	0	0	0	0	0
0	0	0	0	13	0	16	0	0	0	0	0	0	0	0	0	0	0	16
0	0	0	0	0	0	0	0	0	0	0	0	0	0	9	0	0	0	0
0	0	0	0	0	0	0	0	0	0	0	0	0	0	0	0	0	5	0
0	0	0	0	0	0	0	0	0	0	0	0	0	0	0	0	0	0	0
0	0	0	0	0	0	0	0	0	0	0	0	0	0	0	0	0	0	19
0	0	0	0	0	0	0	0	0	0	0	0	0	0	0	0	0	0	0
5	0	0	0	0	0	0	0	0	0	0	0	0	0	0	0	0	0	0
0	0	0	0	0	0	0	0	0	0	0	0	0	0	0	0	0	0	0

0	0	0	0	0	0	0	0	0	0	0	0	0	0	0	0	0	0	0
0	0	0	0	0	0	0	0	0	0	0	0	0	0	0	0	0	0	0
0	0	0	0	0	0	0	0	0	7	0	0	0	0	0	0	0	0	7
0	0	0	0	0	13	0	0	0	0	0	0	0	0	0	0	0	0	0
0	0	0	0	0	0	0	0	0	0	0	0	0	0	0	0	0	0	0
0	0	0	0	0	0	0	0	0	0	0	0	0	0	0	0	0	0	0
0	0	0	0	0	0	0	0	0	0	0	0	0	0	0	0	10	0	0
0	0	0	0	0	0	0	0	0	0	0	0	0	0	0	0	0	0	0
0	0	0	0	0	0	0	0	0	0	0	0	0	0	0	0	0	0	0
0	0	0	0	0	0	0	0	0	0	0	0	0	0	9	0	0	8	0
0	0	0	0	0	0	0	0	0	0	0	0	0	0	0	0	0	0	0
0	0	0	0	0	0	0	0	0	0	0	0	0	0	0	0	0	0	0
0	0	0	0	0	0	0	0	0	0	0	0	0	0	0	0	0	0	0
0	0	0	0	0	0	0	0	0	0	0	0	0	0	0	0	0	0	0
0	0	0	0	0	0	0	0	0	0	0	0	0	0	0	0	0	0	0
0	0	0	0	16	0	0	0	0	0	0	0	0	0	0	0	0	0	0
25	0	0	0	0	0	0	0	0	0	0	0	2	0	0	0	0	0	0
0	0	0	0	0	0	0	0	0	0	0	0	0	0	0	0	0	0	0
0	0	0	0	0	0	4	0	0	0	0	0	0	0	0	0	0	0	0
0	0	0	0	0	0	0	0	0	0	0	0	0	0	0	0	0	0	0
0	0	0	0	0	0	0	0	0	0	0	0	0	0	0	0	0	0	0
0	0	0	0	0	0	0	0	0	0	0	0	0	0	0	0	0	0	0
3	0	0	0	0	0	0	0	0	0	0	0	0	0	0	0	0	0	0
0	0	0	0	0	0	0	0	0	0	0	0	0	0	0	0	0	0	0
0	0	0	0	0	0	0	0	0	0	0	0	0	0	0	0	0	0	0
0	0	0	0	0	0	0	0	0	0	0	0	0	0	0	0	0	0	0
0	0	0	0	0	0	0	0	0	0	0	0	0	0	0	0	0	0	0
0	0	0	0	0	0	0	0	0	0	0	0	0	0	0	0	0	0	0
0	0	0	0	0	0	0	0	0	0	0	0	0	0	0	0	0	0	0
0	0	0	0	0	0	0	0	0	0	0	0	0	0	0	0	0	0	0
0	0	0	0	0	0	0	0	0	0	0	0	0	0	0	0	0	0	0
0	0	0	0	0	0	0	0	0	0	0	0	0	0	0	0	0	0	0
0	0	0	0	0	0	0	0	0	0	0	0	0	0	0	0	0	0	0
0	0	0	0	0	0	0	0	0	0	0	0	0	0	0	0	0	0	0
0	0	0	0	0	0	0	0	0	0	0	0	0	0	0	0	0	0	0
0	0	0	0	0	0	0	0	0	0	0	0	0	0	0	0	0	0	0
0	0	0	0	0	0	0	0	0	0	0	0	0	0	0	0	0	0	0

0	0	0	0	0	0	0	0	0	0	0	0	0	0	0	0	0	0	0
0	0	0	0	0	0	0	0	0	0	0	0	0	0	0	0	0	0	0
0	0	0	0	0	0	0	0	0	0	0	0	0	0	0	0	0	0	0
0	0	0	0	0	0	0	0	0	0	0	0	0	0	0	0	0	0	0
0	0	0	0	0	0	0	0	0	0	0	0	0	0	8	0	0	0	0
0	0	0	0	0	0	0	0	0	0	0	0	0	0	0	0	0	0	0
0	0	0	0	0	0	0	0	0	0	0	0	0	0	0	0	0	0	0
0	0	0	0	0	0	0	0	0	0	0	0	0	0	0	0	0	0	0
0	0	0	0	0	0	0	0	0	0	0	0	0	0	0	0	0	0	0
0	0	0	0	0	0	0	0	0	0	0	0	0	0	0	0	0	0	0
0	0	0	0	7	0	0	0	0	0	0	0	0	0	0	0	0	0	0
0	0	0	0	0	0	0	0	0	0	0	0	0	0	0	0	0	0	0
0	0	0	0	0	0	0	0	0	0	0	0	0	0	0	0	0	0	0
0	0	0	0	0	0	0	0	0	0	0	0	0	0	0	0	0	0	0
0	0	0	0	0	0	0	0	0	0	0	0	0	0	0	0	0	0	0
0	0	0	0	0	0	0	0	0	0	0	0	0	0	0	0	0	0	0
6	0	0	0	0	0	0	0	0	0	0	0	0	0	0	0	0	0	0
0	0	0	0	0	0	0	0	0	0	0	0	0	0	0	0	0	0	0
0	0	0	0	0	0	0	0	0	0	0	0	0	0	0	0	0	0	0
0	0	0	0	0	0	0	0	0	0	0	0	0	0	0	0	0	0	0
0	0	0	0	0	0	0	0	0	0	0	0	0	0	0	0	0	0	0
0	0	0	0	0	0	0	0	0	0	0	0	0	0	0	0	0	0	0
0	0	0	0	0	0	0	0	0	0	0	0	0	0	0	0	0	0	0
0	0	0	0	0	0	0	0	0	0	0	0	0	0	0	0	0	0	0
0	0	0	0	0	0	0	0	0	0	0	0	0	0	0	0	0	0	0
0	0	0	0	5	5	0	0	0	0	0	0	0	0	0	0	0	0	0
0	0	0	0	0	0	0	0	0	0	0	0	0	0	0	0	0	0	0
0	0	0	0	0	0	0	0	0	0	0	0	0	0	0	0	0	0	0
0	0	0	0	0	0	0	0	0	0	0	0	0	0	0	0	0	0	0
0	0	0	0	0	0	0	0	0	0	0	0	0	0	0	0	0	0	0
0	0	0	0	0	0	0	0	0	0	0	0	0	0	0	0	0	0	0
0	0	0	0	0	0	0	0	0	0	0	0	0	0	0	0	0	0	0
0	0	0	0	0	0	0	0	0	0	0	0	0	0	0	0	0	0	0
0	0	0	0	0	0	0	0	0	0	0	0	0	0	0	0	0	0	0
0	0	0	0	0	0	4	4	0	0	0	0	0	0	0	0	0	0	0
0	0	0	0	0	0	0	0	0	0	0	0	0	0	0	0	0	0	0
0	0	0	0	0	0	0	0	0	0	0	0	0	0	0	0	0	0	0

0	0	0	0	0	0	0	0	0	0	0	0	0	0	0	0	0	0	0
0	0	0	0	0	0	0	0	0	0	0	0	0	0	0	0	0	0	0
0	0	0	0	0	0	0	0	0	0	0	0	0	0	0	0	0	0	0
0	0	0	0	0	0	0	0	0	0	0	0	0	0	0	0	0	0	0
0	0	0	0	0	0	0	0	0	0	0	0	0	0	0	0	0	0	0
0	0	0	0	0	0	0	0	0	0	0	0	0	0	0	0	0	0	0
0	0	0	0	0	0	0	0	0	0	0	0	0	0	0	0	0	0	0
0	0	0	0	0	0	0	0	0	0	0	0	0	0	0	0	0	0	0
0	0	0	0	0	0	0	0	0	0	0	0	0	0	0	0	0	0	0
0	0	0	0	0	0	0	0	0	0	0	0	0	0	0	0	0	0	0
0	0	0	0	0	0	0	0	0	0	0	0	0	0	0	0	0	0	0
0	0	0	0	0	0	0	0	0	0	0	0	0	0	0	0	0	0	0
0	0	0	0	0	0	0	0	0	0	0	0	0	0	0	0	0	0	0
0	0	0	0	0	0	0	0	0	0	0	0	0	0	0	0	0	0	0
0	0	0	3	3	3	0	0	0	0	0	0	0	0	0	0	0	0	0
0	0	0	0	0	0	0	0	0	0	0	0	0	0	0	0	0	0	0
0	0	0	0	0	0	0	0	0	0	0	0	0	0	0	0	0	0	0
0	0	0	0	0	0	0	0	0	0	0	0	0	0	0	0	0	0	0
0	0	0	0	0	0	0	0	0	0	0	0	0	0	0	0	0	0	0
0	0	0	0	0	0	0	0	0	0	0	0	0	0	0	0	0	0	0
0	0	0	0	0	0	0	0	0	0	0	0	0	0	0	0	0	0	0
0	0	0	0	0	0	0	0	0	0	0	0	0	0	0	0	0	0	0
0	0	0	0	0	0	0	0	0	0	0	0	0	0	0	0	0	0	0
0	0	0	0	0	0	0	0	0	0	0	0	0	0	0	0	0	0	0
0	0	0	0	0	0	0	0	0	0	0	0	0	0	0	0	0	0	0
0	0	0	0	0	0	0	0	0	0	0	0	0	0	0	0	0	0	0
0	0	0	0	0	0	0	0	0	0	0	0	0	0	0	0	0	0	0
0	0	0	0	0	0	0	0	0	0	0	0	0	0	0	0	0	0	0
0	0	0	0	0	0	0	0	0	0	0	0	0	0	0	0	0	0	0
0	0	0	0	0	0	0	0	0	0	0	0	0	0	0	0	0	0	0
0	0	0	0	0	0	0	0	0	0	0	0	0	0	0	0	0	0	0
0	0	0	0	0	0	0	0	0	0	0	0	0	0	0	0	0	0	0
0	0	0	0	0	0	0	0	0	0	0	0	0	0	0	0	0	0	0
0	0	0	0	0	0	0	0	0	0	0	0	0	0	0	0	0	0	0
0	0	0	0	0	0	0	0	0	0	0	0	0	0	0	0	0	0	0
0	0	0	0	0	0	0	0	0	0	0	0	0	0	0	0	0	0	0
0	0	0	0	0	0	0	0	0	0	0	0	0	0	0	0	0	0	0

0	0	0	0	0	0	0	0	0	0	0	0	0	0	0	0	0	0	0
0	0	0	0	0	0	0	0	0	0	0	0	0	0	0	0	0	0	0
0	0	0	0	0	0	0	0	0	0	0	0	0	0	0	0	0	0	0
0	0	0	0	0	0	0	0	0	0	0	0	0	0	0	0	0	0	0
0	0	0	0	0	0	0	0	0	0	0	2	2	2	2	0	0	0	0
0	0	0	0	0	0	0	0	0	0	0	0	0	0	0	0	0	0	0
0	0	0	0	0	0	0	0	0	0	0	0	0	0	0	0	0	0	0
0	0	0	0	0	0	0	0	0	0	0	0	0	0	0	0	0	0	0
0	0	0	0	0	0	0	0	0	0	0	0	0	0	0	0	0	0	0
0	0	0	0	0	0	0	0	0	0	0	0	0	0	0	0	0	0	0
0	0	0	0	0	0	0	0	0	0	0	0	0	0	0	0	0	0	0
0	0	0	0	0	0	0	0	0	0	0	0	0	0	0	0	0	0	0
0	0	0	0	0	0	0	0	0	0	0	0	0	0	0	0	0	0	0
0	0	0	0	0	0	0	0	0	0	0	0	0	0	0	0	0	0	0
0	0	0	0	0	0	0	0	0	0	0	0	0	0	0	0	0	0	0
0	0	0	0	0	0	0	0	0	0	0	0	0	0	0	0	0	0	0
0	0	0	0	0	0	0	0	0	0	0	0	0	0	0	0	0	0	0
0	0	0	0	0	0	0	0	0	0	0	0	0	0	0	0	0	0	0
0	0	0	0	0	0	0	0	0	0	0	0	0	0	0	0	0	0	0
0	0	0	0	0	0	0	0	0	0	0	0	0	0	0	0	0	0	0
0	0	0	0	0	0	0	0	0	0	0	0	0	0	0	0	0	0	0
0	0	0	0	0	0	0	0	0	0	0	0	0	0	0	0	0	0	0
0	0	0	0	0	0	0	0	0	0	0	0	0	0	0	0	0	0	0
0	0	0	0	0	0	0	0	0	0	0	0	0	0	0	0	0	0	0
0	0	0	0	0	0	0	0	0	0	0	0	0	0	0	0	1	0	0
0	0	0	0	0	0	0	0	0	0									
Jan_DL138	2708	2280	298	688	134	408	332	283	640	151	296	122	317	608	175	81	514	399
160	0	224	0	186	196	52	227	53	183	209	293	209	40	119	132	133	16	289
139	102	270	492	50	83	0	0	185	20	222	134	50	129	128	93	179	122	175
148	12	89	37	151	18	117	46	27	31	59	0	0	89	15	28	31	23	78
35	38	116	149	94	25	29	87	0	37	88	0	22	0	0	97	17	45	47
61	17	143	0	0	79	0	0	0	64	0	0	0	0	17	56	11	0	0
0	0	23	30	0	0	0	0	0	144	4	0	23	15	0	0	62	22	0
159	64	0	0	0	0	0	0	0	10	13	0	7	0	0	0	0	0	0
0	19	0	0	105	22	22	0	0	0	3	0	14	0	53	0	27	0	0
0	30	0	0	0	0	0	30	0	21	0	13	40	0	15	0	15	0	46
0	0	0	0	0	0	0	0	62	0	0	0	0	0	0	0	0	0	0

10	0	0	0	0	18	0	0	0	0	0	0	0	0	0	0	0	22	0
0	0	0	0	0	16	0	0	28	0	0	0	23	10	0	0	0	0	11
0	0	0	0	0	0	0	0	0	0	0	0	0	11	8	11	0	0	0
0	0	30	0	0	19	0	0	0	0	0	0	0	0	0	0	0	0	0
13	0	0	0	0	0	0	0	8	0	0	15	11	0	0	6	0	0	0
0	12	8	0	0	0	0	0	0	0	0	0	10	0	3	0	0	0	0
0	0	11	0	0	0	0	0	41	0	0	0	0	0	0	0	0	0	13
8	8	0	0	0	11	0	32	0	0	0	23	0	0	0	0	0	0	0
0	10	0	0	0	0	0	0	0	0	0	0	0	0	0	0	0	0	0
0	0	0	0	0	0	0	0	4	0	0	0	0	0	0	8	0	0	0
0	0	0	0	0	0	0	6	0	0	8	11	0	18	0	0	0	0	0
0	7	0	0	0	44	0	0	0	0	0	0	0	0	0	0	0	0	0
0	0	0	0	0	0	16	0	0	0	18	0	0	0	0	0	0	0	0
0	5	0	0	8	0	0	0	0	0	0	0	0	0	0	0	0	0	0
0	0	0	0	0	0	0	6	0	0	0	0	0	0	0	0	0	0	0
0	0	0	0	0	8	0	15	0	8	0	0	0	0	0	0	0	0	0
0	0	0	0	0	0	8	0	0	0	0	0	0	0	0	0	0	0	0
0	0	0	0	0	0	0	0	0	0	0	0	0	0	14	0	0	0	0
0	6	0	0	0	0	0	0	0	0	0	0	0	0	0	0	0	0	0
0	0	0	0	0	0	0	0	0	0	0	0	0	0	7	0	0	0	7
0	0	0	0	0	0	0	0	0	15	0	0	0	0	0	0	0	0	0
0	0	0	0	0	0	0	0	0	0	0	0	0	0	0	16	0	0	0
0	0	0	0	0	0	0	0	0	0	0	0	0	0	0	0	0	0	0
0	0	0	0	0	0	0	0	0	7	0	0	0	0	0	0	0	0	0
0	0	0	0	0	0	0	0	0	0	0	0	0	0	0	0	0	0	0
0	0	0	0	0	0	0	0	0	0	0	0	0	0	0	0	0	0	0
0	0	0	0	0	0	0	0	0	0	0	0	0	0	0	0	0	0	0
0	0	0	0	0	0	0	0	0	0	0	0	0	0	0	0	0	0	0
0	0	0	0	0	0	0	0	0	0	0	0	0	0	0	0	0	0	0
0	0	0	0	0	14	0	0	0	0	0	0	0	0	0	0	0	0	0
0	0	0	0	0	0	0	0	0	0	0	0	0	4	0	0	0	0	0
0	0	0	0	0	0	0	0	0	0	0	0	0	0	0	0	0	0	18
0	0	0	0	0	0	0	0	0	0	0	0	0	0	0	0	0	0	0
0	0	0	0	0	0	0	0	0	0	0	0	0	0	0	0	0	0	0
0	0	0	0	0	0	0	0	0	0	0	0	0	0	0	0	0	0	0
0	0	0	0	0	0	5	0	0	0	0	0	0	0	0	0	0	0	0
0	0	0	0	0	0	0	0	0	0	0	0	0	0	0	0	0	0	0

0	0	0	0	0	0	0	0	0	0	0	0	0	0	0	0	0	0	0
0	0	0	0	0	0	0	0	0	0	0	0	0	0	0	0	0	0	0
0	0	0	0	0	0	0	0	0	0	0	0	0	0	0	0	0	0	0
0	0	0	0	0	0	0	0	0	0	0	0	0	0	0	0	0	0	0
0	0	0	0	0	0	0	0	0	0	0	0	0	0	0	0	0	0	0
0	0	0	0	0	0	0	0	0	0	0	0	0	0	0	0	0	0	0
0	0	0	0	0	0	0	0	0	0	0	0	0	0	0	0	0	0	0
0	0	0	0	0	0	0	0	0	0	0	0	0	0	0	0	0	0	0
0	0	0	0	0	0	0	0	0	0	0	0	0	0	0	0	0	0	0
0	0	0	0	0	0	0	0	0	0	0	0	0	0	0	0	0	0	0
0	0	0	0	0	0	0	0	0	0	0	0	0	0	0	0	0	0	0
0	0	0	0	0	0	0	0	0	0	0	0	0	0	0	0	0	0	0
0	0	0	0	0	0	0	0	0	0	0	0	0	0	0	0	0	0	0
0	0	0	0	0	0	0	0	0	0	0	0	0	0	0	0	0	0	0
0	0	0	0	0	0	0	0	0	0	0	0	0	0	0	0	0	0	0
0	0	0	0	0	0	0	0	0	0	0	0	0	0	0	0	0	0	0
0	0	0	0	0	0	0	0	0	0	0	20	0	0	0	0	0	0	0
0	0	0	0	0	0	0	0	0	0	0	0	0	0	0	0	0	0	0
0	0	0	0	0	0	0	0	0	0	0	0	0	0	0	0	0	0	0
0	0	0	0	0	0	0	0	0	0	0	0	0	0	0	0	0	0	0
0	0	0	0	0	0	0	0	0	0	0	0	0	0	0	0	0	0	0
0	0	0	0	0	0	0	0	0	0	0	0	0	0	0	0	0	0	0
0	0	0	0	0	0	0	0	0	0	0	0	0	0	0	0	0	0	0
0	0	0	0	0	0	0	0	0	0	0	0	0	0	0	0	0	0	0
0	0	0	0	0	0	0	0	0	0	0	0	0	0	0	0	0	0	7
0	0	0	0	0	0	0	0	0	0	0	0	0	0	0	0	0	0	0
0	0	0	0	0	0	0	0	0	0	0	0	0	0	0	0	0	0	0
0	0	0	13	0	0	0	0	0	0	0	0	0	0	0	0	0	0	0
0	0	0	0	0	0	0	0	0	0	0	0	0	0	0	0	0	0	0
0	0	0	0	0	0	0	0	0	0	0	0	0	0	0	0	0	0	0
0	0	0	0	0	0	0	0	0	0	0	0	0	0	0	0	0	0	0
0	0	0	0	0	0	0	0	0	0	0	0	0	0	0	0	0	0	0
0	0	0	0	0	0	0	0	0	0	0	0	0	0	0	0	0	0	0
0	0	0	0	0	0	0	0	0	0	0	0	0	0	0	0	0	0	0
0	0	0	0	0	0	0	0	0	0	0	0	0	0	0	0	0	0	0
0	0	0	0	0	0	0	0	0	0	0	0	0	0	0	0	0	0	0
0	0	0	0	0	0	0	0	0	0	0	0	0	0	0	0	0	0	0

0	0	0	0	0	0	0	0	0	0	0	0	0	0	0	0	0	0	0
0	0	0	0	0	0	0	0	0	0	0	0	0	0	0	0	0	0	0
0	0	0	0	0	0	0	0	0	0	0	0	0	0	9	0	0	0	0
0	0	0	0	0	0	0	0	0	0	0	0	0	0	0	0	0	0	0
0	0	0	0	0	0	0	0	0	0	0	0	0	0	0	0	0	0	0
0	0	0	0	0	0	0	0	0	0	0	0	0	0	0	0	0	0	0
0	0	0	0	0	0	0	0	0	0	0	0	0	0	0	0	0	0	0
0	0	0	0	0	0	0	0	0	0	0	0	0	0	0	0	0	0	0
0	0	0	0	0	0	0	0	0	0	0	0	6	0	0	0	0	0	0
0	0	0	0	0	0	0	0	0	0	0	0	0	0	0	0	0	0	0
0	0	0	0	0	0	0	0	0	0	0	0	0	0	0	0	0	0	0
0	0	0	0	0	0	0	0	0	0	0	0	0	0	0	0	0	0	0
0	0	0	0	0	0	0	0	0	0	0	0	0	0	0	0	0	0	0
0	0	0	0	0	0	0	0	0	0	0	0	0	0	0	0	0	0	0
0	0	0	0	0	0	0	0	0	0	0	0	0	0	0	0	0	0	0
0	0	0	0	0	0	0	0	0	0	0	0	0	0	0	0	0	0	0
0	0	0	0	0	0	0	0	0	0	0	0	0	0	0	0	0	0	0
0	0	0	0	0	0	0	0	0	0	0	0	0	0	0	0	0	0	0
0	0	0	0	0	0	0	0	0	0	0	0	0	0	0	0	0	0	0
0	0	0	0	0	0	0	0	0	0	0	0	0	0	0	0	0	0	0
0	0	0	0	0	0	0	0	0	0	0	0	0	0	0	0	0	0	0
0	0	0	0	0	0	0	0	0	0	0	0	0	0	0	0	0	0	0
0	0	0	0	0	0	0	0	0	0	0	0	0	0	0	0	0	0	0
0	0	0	0	0	0	0	0	0	0	0	0	0	0	0	0	0	0	0
0	0	0	0	0	0	0	0	0	0	0	0	0	0	0	0	0	0	0
0	0	0	0	0	0	0	0	0	0	0	0	0	0	0	0	0	0	0
0	0	0	0	0	0	0	0	0	0	0	0	0	0	0	0	0	0	0
0	0	0	0	0	0	0	0	0	0	0	0	0	0	0	0	0	0	0
0	0	0	0	0	0	0	0	0	0	0	0	0	0	0	0	0	0	0
0	0	0	0	0	0	0	0	0	0	5	0	0	0	0	0	0	0	0
0	0	0	0	0	0	0	0	0	0	0	0	0	0	0	0	0	0	0
0	0	0	0	0	0	0	0	0	0	0	0	0	0	0	0	0	0	0
0	0	0	0	0	0	0	0	0	0	0	0	0	0	0	0	0	0	0
0	0	0	0	0	0	0	0	0	0	0	0	0	0	0	0	0	0	0
0	0	0	0	0	0	0	0	0	0	0	0	0	0	0	0	0	0	0
0	0	0	0	0	0	0	0	0	0	0	0	0	0	0	0	0	0	0
0	0	0	0	0	0	0	0	0	0	0	0	0	0	0	0	0	0	0

0	0	0	0	0	0	0	0	0	0	0	0	0	0	0	0	0	0	0
0	0	0	0	0	0	0	0	0	0	0	0	0	0	0	0	0	0	0
0	0	0	0	0	0	0	0	0	0	0	0	0	0	0	0	0	0	0
0	0	0	0	0	0	0	0	0	0	0	0	0	0	0	0	0	0	0
0	0	0	0	0	0	0	0	0	0	0	0	0	0	0	0	0	0	0
0	0	0	0	4	4	4	0	0	0	0	0	0	0	0	0	0	0	0
0	0	0	0	0	0	0	0	0	0	0	0	0	0	0	0	0	0	0
0	0	0	0	0	0	0	0	0	0	0	0	0	0	0	0	0	0	0
0	0	0	0	0	0	0	0	0	0	0	0	0	0	0	0	0	0	0
0	0	0	0	0	0	0	0	0	0	0	0	0	0	0	0	0	0	0
0	0	0	0	0	0	0	0	0	0	0	0	0	0	0	0	0	0	0
0	0	0	0	0	0	0	0	0	0	0	0	0	0	0	0	0	0	0
0	0	0	0	0	0	0	0	0	0	0	0	0	0	0	0	0	0	0
0	0	0	0	0	0	0	0	0	0	0	0	0	0	0	0	0	0	0
0	0	0	0	0	0	0	0	0	0	0	0	0	0	0	0	0	0	0
0	0	0	0	0	0	0	0	0	0	0	0	0	0	0	0	0	0	0
0	0	0	0	0	0	0	0	0	0	0	0	0	0	0	0	0	0	0
0	0	0	0	0	0	0	0	0	0	0	0	0	0	0	0	0	0	0
0	0	0	0	0	0	0	0	0	0	0	0	0	0	0	0	0	0	0
0	0	0	0	0	0	0	0	0	0	0	0	0	0	0	0	0	0	0
0	0	0	0	0	0	0	0	0	0	0	0	0	0	0	0	0	0	0
0	0	0	0	0	0	0	0	0	0	0	0	0	0	0	0	0	0	0
0	0	0	0	0	0	0	0	0	0	0	0	0	0	0	0	0	0	0
0	0	0	0	3	0	0	0	0	0	0	0	0	0	0	0	0	0	0
0	0	0	0	0	0	0	0	0	0	0	0	0	0	0	0	0	0	0
0	0	0	0	0	0	0	0	0	0	0	0	0	0	0	0	0	0	0
0	0	0	0	0	0	0	0	0	0	0	0	0	0	0	0	0	0	0
0	0	0	0	0	0	0	0	0	0	0	0	0	0	0	0	0	0	0
0	0	0	0	0	0	0	0	0	0	0	0	0	0	0	0	0	0	0
0	0	0	0	0	0	0	0	0	0	0	0	0	0	0	0	0	0	0
0	0	0	0	0	0	0	0	0	0	0	0	0	0	0	0	0	0	0
0	0	0	0	0	0	0	0	0	0	0	0	0	0	0	0	0	0	0
0	0	0	0	0	0	0	0	0	0	0	0	0	0	0	0	0	0	0
0	0	0	0	0	0	0	0	0	0	0	0	0	0	0	0	0	0	0
0	0	0	0	0	0	0	0	0	0	0	0	0	0	0	0	0	0	0
0	0	0	0	0	0	0	0	0	0	0	0	0	0	0	0	0	0	0
0	0	0	0	0	0	0	0	0	0	0	0	0	0	0	0	0	0	0

0	0	0	0	0	0	0	0	0	0	0	0	0	0	0	0	0	0	0
0	0	0	0	0	0	0	0	0	0	0	0	0	0	0	0	0	0	0
0	0	0	0	0	0	0	0	0	0	0	0	0	0	0	0	0	0	0
0	0	0	0	0	0	0	0	0	0	0	0	0	0	0	0	0	0	0
0	0	0	0	0	0	0	0	0	0	0	0	0	0	0	0	0	0	0
0	0	0	0	0	0	0	0	0	0	0	0	0	0	0	0	0	0	0
0	0	0	0	0	0	0	0	0	0	0	0	0	0	0	0	0	0	0
0	0	0	0	0	0	0	0	0	0	0	0	0	0	0	0	0	0	0
0	0	0	0	0	0	0	0	0	0	0	0	0	0	0	0	0	0	0
0	0	0	0	0	0	0	0	0	0	0	0	0	0	0	0	0	0	0
0	0	0	0	0	0	0	0	0	0	0	0	0	0	0	0	0	2	2
2	2	2	2	0	0	0	0	0	0	0	0	0	0	0	0	0	0	0
0	0	0	0	0	0	0	0	0	0	0	0	0	0	0	0	0	0	0
0	0	0	0	0	0	0	0	0	0	0	0	0	0	0	0	0	0	0
0	0	0	0	0	0	0	0	0	0	0	0	0	0	0	0	0	0	0
0	0	0	0	0	0	0	0	0	0	0	0	0	0	0	0	0	0	0
0	0	0	0	0	0	0	0	0	0	0	0	0	0	0	0	0	0	0
0	0	0	0	0	0	0	0	0	0									
Jan_DL139	1732	1024	703	329	102	99	148	143	65	260	199	192	208	89	153	288	110	127
100	58	185	442	153	165	231	175	188	179	69	0	87	207	121	0	104	89	0
160	150	0	27	111	47	108	376	27	121	45	179	47	0	19	113	79	0	0
0	125	140	93	0	80	0	51	82	72	36	0	59	0	47	0	43	149	112
106	0	0	17	0	55	38	0	0	62	0	0	53	16	0	0	43	11	159
0	39	0	0	65	8	65	5	32	32	0	0	0	52	27	11	0	0	0
0	20	26	38	30	27	0	0	0	0	7	84	9	5	0	0	0	0	0
0	18	0	0	0	49	0	0	0	31	0	0	15	0	20	0	0	0	0
76	0	50	0	0	0	0	26	265	30	8	0	0	23	0	0	0	0	0
0	0	12	22	0	0	0	0	0	0	0	33	0	0	0	0	34	0	0
0	0	0	14	33	19	0	0	0	5	0	12	0	0	14	0	0	0	0
0	0	0	0	0	0	0	0	0	0	0	0	0	0	0	0	0	7	84
0	14	12	18	0	0	0	36	18	100	0	24	0	12	83	12	0	0	0
0	0	0	33	69	0	0	0	0	0	0	0	10	17	0	0	0	0	18
0	0	0	0	0	0	0	0	38	0	8	0	0	0	7	8	0	0	0
0	0	0	0	0	0	0	17	5	0	56	11	0	21	0	8	0	0	0
0	0	12	68	0	0	0	0	0	0	0	0	0	45	0	0	0	0	10
0	0	0	0	0	0	0	15	0	0	0	0	0	0	0	0	8	54	0
0	0	0	0	0	0	0	0	0	8	0	0	22	0	0	0	7	0	0

0	0	0	0	0	0	0	8	0	16	0	0	0	0	0	0	0	0	4
0	0	0	0	5	0	15	0	0	0	15	0	0	0	12	0	0	0	0
0	0	0	0	17	0	0	6	0	0	0	9	0	0	0	0	6	0	0
0	0	0	0	0	27	0	0	0	0	0	0	0	0	0	0	0	0	0
0	9	0	0	0	0	0	13	0	0	0	0	0	0	0	0	0	0	15
0	6	0	0	10	0	0	0	0	0	14	15	0	0	0	0	0	0	0
0	0	0	0	0	0	0	0	0	0	0	0	10	11	0	0	0	20	0
12	0	11	0	0	0	0	0	0	0	0	0	0	0	0	0	0	8	0
0	0	28	0	0	0	0	0	0	0	72	0	0	0	0	8	46	14	0
0	0	0	0	0	0	0	0	0	0	0	39	0	0	0	0	0	0	0
0	6	0	0	0	0	0	0	0	0	9	11	0	0	0	12	0	0	0
0	0	7	4	0	0	0	0	0	0	0	0	23	0	0	0	0	27	0
0	0	0	0	0	0	0	0	0	0	0	0	0	0	0	0	0	0	0
0	0	0	0	0	0	0	0	0	0	0	0	0	0	0	0	0	0	0
0	0	0	0	0	0	0	0	11	70	15	0	0	0	0	0	0	0	0
0	0	0	0	0	4	0	0	0	0	0	5	0	0	0	0	0	0	0
0	0	0	0	0	0	0	24	0	0	8	0	0	0	0	0	30	0	0
0	0	0	0	0	0	0	0	0	0	7	0	0	0	0	0	0	0	0
0	0	7	0	0	9	0	0	0	0	0	0	0	0	0	0	0	0	0
0	0	0	0	0	0	0	0	0	0	0	0	0	0	0	0	0	0	0
0	0	0	0	0	0	0	0	0	0	0	6	0	0	0	0	0	0	0
0	0	0	0	0	0	0	0	0	0	0	0	0	0	0	0	0	0	5
0	0	0	0	0	0	0	0	0	0	0	21	0	0	0	0	0	0	5
0	0	0	0	0	0	0	0	0	0	0	0	0	0	0	0	0	0	0
0	0	0	0	0	0	0	0	0	0	0	0	0	0	0	0	0	0	0
0	0	0	3	15	0	0	0	0	0	0	0	0	0	0	0	0	0	0
0	0	0	0	0	0	0	12	0	0	0	0	0	0	0	0	8	0	0
0	0	0	0	0	0	0	0	0	10	0	0	6	0	4	0	0	0	0
0	0	0	0	0	0	0	0	0	0	0	0	0	0	0	0	0	0	0
0	0	0	0	0	0	0	0	0	0	0	0	0	0	0	0	0	0	38
0	8	0	0	0	0	0	0	0	0	0	0	10	0	0	0	0	0	0
0	0	0	0	0	0	0	0	0	0	0	0	0	0	0	0	0	0	0
0	0	0	0	0	0	0	0	0	0	12	0	12	0	0	0	0	0	0
0	0	0	0	0	0	0	0	0	0	0	0	0	0	0	0	0	0	0
0	0	6	0	0	0	0	0	0	0	0	0	0	0	0	0	0	0	0
0	0	0	0	0	0	0	0	0	0	0	0	0	0	0	0	0	0	0
0	0	0	0	0	0	0	0	0	0	0	0	0	0	0	0	0	0	0

0	0	0	0	0	0	0	0	0	0	0	0	0	0	0	0	0	0	0
0	0	0	4	0	0	0	0	0	0	0	0	0	0	0	0	0	0	0
0	0	0	0	0	0	0	0	0	0	0	0	0	0	0	0	0	0	0
0	0	0	0	0	0	0	0	0	0	0	0	0	0	0	0	0	0	0
0	0	0	0	0	0	0	0	0	0	0	0	0	0	0	0	0	0	0
0	0	0	0	0	0	0	0	0	0	0	0	0	0	0	0	0	0	0
0	0	0	0	0	0	0	0	0	0	0	0	0	0	0	0	0	0	0
0	0	0	0	0	0	0	0	16	0	0	0	0	0	0	0	0	0	0
0	0	0	0	0	0	0	0	0	0	0	0	0	0	0	0	0	0	0
0	0	0	0	0	0	0	0	0	0	0	0	0	0	0	0	0	0	0
0	0	0	0	0	0	0	0	0	0	0	0	0	0	0	0	0	0	0
0	0	0	0	0	0	0	0	0	0	0	0	0	0	0	0	0	0	0
0	0	13	0	0	0	0	0	0	0	0	7	0	0	0	0	0	0	0
0	0	0	0	0	0	0	0	0	0	0	0	0	0	0	0	0	0	0
0	0	0	0	0	0	0	0	0	0	0	0	0	0	0	0	0	0	0
0	0	0	0	0	0	0	0	0	0	0	0	0	0	0	0	0	0	0
0	0	0	0	0	0	0	0	0	0	0	0	0	0	0	0	0	0	0
0	0	0	0	0	0	0	0	0	0	0	0	0	0	0	0	0	0	0
0	0	0	0	0	0	0	0	0	0	0	0	0	0	10	0	0	0	0
0	0	0	0	0	0	0	0	0	0	0	0	0	0	0	0	0	0	0
0	0	0	0	0	0	0	0	0	0	0	0	0	0	0	0	0	0	0
0	0	0	0	0	0	0	0	0	0	0	0	0	0	0	0	0	0	0
0	0	0	0	9	0	0	0	0	0	0	0	0	0	0	0	0	0	0
0	0	0	0	0	0	0	0	0	0	0	0	0	0	0	0	0	0	0
0	0	0	0	0	0	0	0	0	0	0	0	0	0	0	0	0	0	0
0	0	0	0	0	0	0	0	0	0	0	0	0	0	0	0	0	0	0
0	0	0	0	0	0	0	0	0	0	0	0	0	0	0	0	0	0	0
0	0	0	0	0	0	0	0	0	0	0	0	0	0	0	0	0	0	0
0	0	0	0	0	0	0	0	0	0	0	0	0	0	0	3	0	0	0
3	0	0	0	0	0	0	0	0	0	0	0	0	0	0	0	0	0	0
0	0	0	0	0	0	0	0	0	0	0	0	0	0	0	0	0	0	0
0	0	0	0	0	0	0	0	0	0	0	0	0	0	0	0	0	0	0
0	0	0	0	0	0	0	0	0	0	0	0	0	0	2	0	0	0	0
0	0	0	0	0	0	0	0	0	0	0	0	0	0	0	0	0	0	0
0	0	0	0	0	0	0	0	0	0	0	0	0	0	0	0	0	0	0
0	0	0	0	0	0	0	0	0	0	0	0	0	0	0	0	0	0	0
0	0	0	0	0	0	0	0	0	0	0	0	0	0	0	0	0	0	0

0	0	0	0	0	0	0	0	0	0	0	0	0	0	0	0	0	0	0
0	0	0	0	0	0	0	0	0	0	0	0	0	0	0	0	0	0	0
0	0	0	0	0	0	0	0	0	0	0	0	0	0	0	0	0	0	0
0	0	0	0	0	0	0	0	0	0	0	0	0	0	0	0	0	0	0
0	0	0	0	0	0	0	0	0	0	0	0	0	0	0	0	0	0	0
0	0	0	0	0	0	0	0	0	0	0	0	0	0	0	0	0	0	0
0	0	0	0	0	0	0	0	0	0	0	0	0	0	0	0	0	0	0
0	0	0	0	0	0	0	0	0	0	0	0	0	0	0	0	0	0	0
0	0	0	0	0	0	0	0	0	0	0	0	0	0	0	0	0	0	0
0	0	0	0	0	0	0	0	0	0	0	0	0	0	0	0	0	0	0
0	0	0	0	0	0	0	0	0	0	0	0	0	0	0	0	0	0	0
0	0	0	0	0	0	0	0	0	0	0	0	0	0	0	0	0	0	0
0	0	0	0	0	0	0	0	0	0	0	0	0	0	0	0	0	0	0
0	0	0	0	0	0	0	0	0	0	0	0	0	0	0	0	0	0	0
0	0	0	0	0	0	0	0	0	0	0	0	0	0	0	0	0	0	0
0	0	0	0	0	0	0	0	0	0	0	0	0	0	0	0	0	0	0
0	0	0	0	0	0	0	0	0	0	0	0	0	0	0	0	0	0	0
0	0	0	0	0	0	0	0	0	0	0	0	0	0	0	0	0	0	0
0	0	0	0	0	0	0	0	0	0	0	0	0	0	0	0	0	0	0
0	0	0	0	0	0	0	0	0	0	0	0	0	0	0	0	0	0	0
0	0	0	0	0	0	0	0	0	0	0	0	0	0	0	0	0	0	0
0	0	0	0	0	0	0	0	0	0	0	0	0	0	0	0	0	0	0
0	0	0	0	0	0	0	0	0	0	0	0	0	0	0	0	0	0	0
0	0	0	0	0	0	0	0	0	0	0	0	0	0	0	0	0	0	0
0	0	0	0	0	0	0	0	0	0	0	0	0	0	0	0	0	0	0
0	0	0	0	0	0	0	0	0	0	0	0	0	0	0	0	0	0	0
0	0	0	0	0	0	0	0	0	0	0	0	0	0	0	0	0	0	0
0	0	0	0	0	0	0	0	0	0	0	0	0	0	0	0	0	0	0
0	0	0	0	0	0	0	0	0	0	0	0	0	0	0	0	0	0	0
0	0	0	0	0	0	0	0	0	0	0	0	0	0	0	0	0	0	0
0	0	0	0	0	0	0	0	0	0	0	0	0	0	0	0	0	0	0
0	0	0	0	0	0	0	0	0	0	0	0	0	0	0	0	0	0	0
0	0	0	0	0	0	0	0	0	0	0	0	0	0	0	0	0	0	0
0	0	0	0	0	0	0	0	0	0	0	0	0	0	0	0	0	0	0
0	0	0	0	0	0	0	0	0	0	0	0	0	0	0	0	0	0	0
3	0	0	0	0	0	0	0	0	0	0	0	0	0	0	0	0	0	0
0	0	0	0	0	0	0	0	0	0	0	0	0	0	0	0	0	0	0

0	0	0	0	0	0	0	0	0	0	0	0	0	0	0	0	0	0	0
0	0	0	0	0	0	0	0	0	0	0	0	0	0	0	0	0	0	0
0	0	0	0	0	0	0	0	0	0	0	0	0	0	0	0	0	0	0
0	0	0	0	0	0	0	0	0	0	0	0	0	0	0	0	0	0	0
0	0	0	0	0	0	0	0	0	0	0	0	0	0	0	0	0	0	0
0	0	0	0	0	0	0	0	0	0	0	0	0	0	0	0	0	0	0
0	0	0	0	0	0	0	0	0	0	0	0	0	0	0	0	0	0	0
0	0	0	0	0	0	0	0	0	0	0	0	0	0	0	0	0	0	0
0	0	0	0	0	0	0	0	0	0	0	0	0	0	0	0	0	0	0
0	0	0	0	0	0	0	0	0	0	0	0	0	0	0	0	0	0	0
0	0	0	0	0	0	0	0	0	0	0	0	0	0	0	0	0	0	0
0	0	0	0	0	0	0	0	0	0	0	0	0	0	0	0	0	0	0
0	0	0	0	0	0	0	0	0	0	0	0	0	0	0	0	0	0	0
0	0	0	0	0	0	0	0	0	0	0	0	0	0	0	0	0	0	0
0	0	0	0	0	0	0	0	0	0	0	0	0	0	0	0	0	0	0
0	0	0	0	0	0	0	0	0	0	0	0	0	0	0	0	0	0	0
0	0	0	0	0	0	0	0	0	0	0	0	0	0	0	0	0	0	0
0	0	0	0	0	0	0	0	0	0	0	0	0	0	0	0	0	0	0
0	0	0	0	0	0	0	0	0	0	0	0	0	0	0	0	0	0	0
0	0	0	0	0	0	0	0	0	0	0	0	0	0	0	0	0	0	0
0	0	0	0	0	0	0	0	0	0	0	0	0	0	0	0	0	0	0
0	0	0	0	0	0	0	0	0	0	0	0	0	0	0	0	0	0	0
0	0	0	0	0	0	0	0	0	0	0	0	0	0	0	0	0	0	0
0	0	0	0	0	0	0	0	0	0	0	0	0	0	0	0	0	0	0
0	0	0	0	0	0	0	0	0	0	0	0	0	0	0	0	0	0	0
2	2	2	2	2	2	2	2	0	0	0	0	0	0	0	0	0	0	0
0	0	0	0	0	0	0	0	0	0	0	0	0	0	0	0	0	0	0
0	0	0	0	0	0	0	0	0	0	0	0	0	0	0	0	0	0	0
0	0	0	0	0	0	0	0	0	0	0	0	0	0	0	0	0	0	0
0	0	0	0	0	0	0	0	0	0	0	0	0	0	0	0	0	0	0
0	0	0	0	0	0	0	0	0	0	0	0	0	0	0	0	0	0	0
0	0	0	0	0	0	0	0	0	0	0	0	0	0	0	0	0	0	0
0	0	0	0	0	0	0	0	0	0	0	0	0	0	0	0	0	0	0
0	0	0	0	0	0	0	0	0	0	0	0	0	0	0	0	0	0	0
0	0	0	0	0	0	0	0	0	0	0	0	0	0	0	0	0	0	0
0	0	0	0	0	0	0	0	0	0	0	0	0	0	0	0	0	0	0
0	0	0	0	0	0	0	0	0	0	0	0	0	0	0	0	0	0	0

0	0	0	0	0	0	0	0	0	0	0	0	0	0	0	0	0	0	0
0	0	0	0	0	0	0	0	0	0	0	0	0	0	0	0	0	0	0
0	0	0	0	0	0	0	0	0	0	0	0	0	0	0	0	0	0	0
0	0	0	0	0	0	0	0	0	0	0	0	0	0	0	0	0	0	0
0	0	0	0	0	0	0	0	0	0	0	0	0	0	0	0	0	0	0
0	0	0	0	0	0	0	0	0	0	0	0	0	0	0	0	0	0	0
0	0	0	0	0	0	0	0	0	0	0	0	0	0	0	0	0	0	0
0	0	0	0	0	0	0	0	0	0	0	0	0	0	0	0	0	0	0
0	0	0	0	0	0	0	0	0	0	0	0	0	0	1	1	0	0	0
0	0	0	0	0	0	0	0	0	0									
Jan_DL014	2910	1800	480	199	2345	185	353	331	20	339	264	263	260	1072	233	85	1420	375
292	0	125	0	130	111	135	267	143	51	241	1309	195	94	118	135	212	0	753
0	0	138	0	70	116	55	22	117	32	263	0	15	189	4	0	153	168	697
697	0	0	47	179	0	58	73	81	56	14	0	10	62	0	36	337	6	0
90	0	0	50	272	111	22	0	52	0	0	103	36	5	50	0	0	65	0
169	16	86	0	31	117	0	0	0	0	259	120	0	15	20	37	125	0	0
0	0	10	22	41	0	0	0	0	0	17	0	54	109	0	17	82	44	0
0	22	137	10	38	0	0	114	0	69	8	0	61	89	30	72	0	0	0
0	0	0	3	0	13	15	0	0	0	0	0	34	0	0	0	0	38	11
37	0	15	0	0	0	0	14	0	0	0	31	0	0	130	0	0	0	0
0	0	0	0	0	0	0	36	0	7	80	0	0	0	36	0	0	44	0
23	67	0	0	29	0	0	0	0	0	0	38	49	0	0	0	0	0	0
0	0	0	0	0	0	0	0	0	0	0	0	0	4	0	36	0	35	0
0	0	0	0	0	0	0	44	0	21	0	0	0	41	0	23	0	0	8
8	169	0	0	15	24	0	0	0	0	0	0	0	15	0	0	35	0	0
0	27	0	29	0	0	0	0	4	0	0	0	0	0	10	51	0	8	0
0	0	0	0	0	0	0	0	0	27	0	0	0	12	2	10	0	0	6
0	0	0	0	0	0	86	0	21	0	0	0	0	0	0	0	0	0	0
9	0	0	0	2	0	0	0	0	0	0	0	0	0	0	0	0	0	0
0	0	7	0	11	0	0	0	0	34	0	0	0	0	32	0	0	0	0
0	0	0	0	0	15	0	0	27	0	0	0	10	0	7	0	0	0	0
0	0	0	0	0	0	0	0	0	0	22	6	0	0	0	0	0	0	0
0	0	0	0	0	0	0	0	15	18	0	0	0	0	0	0	0	0	0
0	0	0	0	0	0	0	0	0	0	0	0	0	0	0	0	13	0	6
0	0	0	0	15	0	0	0	0	0	0	0	0	15	0	0	0	0	0
0	0	0	15	0	0	0	5	0	9	0	0	0	0	0	0	0	0	0
0	0	0	0	0	0	0	50	0	0	0	0	0	0	0	0	0	0	0

54	0	0	0	0	0	0	0	18	0	0	0	0	0	0	0	0	0	0
22	0	0	0	0	0	0	0	0	0	0	0	0	0	0	0	0	0	0
19	0	5	0	0	0	0	53	0	0	0	0	0	0	0	0	0	0	0
0	6	0	0	0	0	0	0	0	0	0	0	0	0	0	0	66	0	12
0	0	0	0	0	0	0	0	0	0	0	0	0	0	0	0	0	0	0
0	0	0	0	41	0	0	0	0	0	0	0	0	0	0	0	0	0	0
0	0	0	0	0	0	0	0	0	0	0	0	0	0	0	0	0	0	0
0	0	0	0	0	0	0	0	0	0	15	0	0	0	8	0	0	0	44
0	0	0	0	0	0	0	0	0	0	0	0	0	0	0	0	0	49	0
0	0	0	0	0	0	0	0	0	0	0	0	0	0	0	12	0	0	10
0	0	0	0	0	0	0	0	14	33	0	0	0	0	0	0	0	0	0
8	0	0	0	0	0	24	0	0	0	10	0	0	0	0	0	0	0	0
0	0	0	0	0	0	0	0	0	0	27	0	0	0	0	0	4	0	0
0	0	0	0	0	0	0	0	0	0	13	18	0	0	0	0	0	0	0
0	8	0	0	0	0	0	0	0	0	0	0	0	13	0	0	0	0	0
0	0	0	0	0	0	0	0	0	10	0	0	0	0	0	0	0	0	0
0	0	0	0	0	0	9	0	0	0	0	0	0	0	0	0	0	0	0
0	0	0	0	0	0	0	0	0	0	0	0	0	0	0	0	0	0	0
0	0	0	0	15	0	0	0	0	0	0	28	0	0	0	0	14	0	0
0	0	0	0	0	0	0	0	0	0	0	0	0	0	0	0	0	0	0
0	0	0	36	0	0	0	0	0	0	0	0	0	0	0	0	0	0	0
0	0	0	0	0	0	0	0	5	0	0	0	24	0	0	0	0	0	0
0	0	0	0	0	0	0	0	0	0	0	0	0	0	0	10	0	0	0
0	0	0	0	0	0	0	0	0	0	0	0	0	0	0	0	0	0	0
0	0	0	0	0	0	0	0	0	0	0	0	0	0	0	0	0	0	0
0	8	0	0	0	0	0	0	0	0	0	0	0	0	0	0	0	0	0
0	0	0	0	0	0	0	0	0	0	0	0	0	0	0	0	0	0	0
0	0	0	0	0	0	0	0	0	0	0	0	0	0	0	0	32	0	0
0	0	0	0	0	0	0	0	0	0	0	0	0	0	0	0	0	0	0
0	0	0	0	0	0	0	0	0	5	0	0	0	0	0	0	0	0	0
0	0	0	0	0	0	0	0	9	0	0	0	0	0	0	0	0	0	0
0	0	0	0	0	0	0	0	0	0	0	0	0	0	0	0	0	0	0
0	0	0	0	0	0	0	0	0	0	0	0	0	6	0	0	0	0	0
0	0	0	0	0	6	0	0	0	0	0	0	0	0	0	0	0	0	0
0	0	0	17	0	0	6	0	0	0	0	0	0	0	0	0	0	0	0
0	0	0	0	0	0	0	0	0	0	0	0	0	0	0	0	0	0	0
0	0	0	0	0	0	0	0	0	0	0	0	6	0	0	0	0	0	0

0	0	0	0	0	0	0	0	0	0	0	0	0	0	0	0	0	0	0
0	0	0	0	0	0	0	0	0	15	0	0	0	0	0	0	0	0	0
0	0	0	0	0	0	0	0	0	0	0	0	0	0	0	0	0	0	0
0	0	0	0	0	0	0	0	0	0	0	0	0	0	0	0	0	0	0
0	0	0	0	0	0	0	0	0	0	0	0	0	0	0	0	0	0	0
0	0	0	0	0	0	0	0	0	0	0	0	0	0	0	0	0	0	0
0	0	0	0	0	0	0	0	0	0	0	0	0	0	0	0	0	0	0
0	0	0	0	0	0	0	0	0	0	0	0	0	0	0	0	0	0	0
0	0	0	0	0	0	0	0	0	0	0	0	0	0	0	0	0	0	0
0	0	0	0	0	0	0	0	0	0	0	0	0	0	0	0	0	0	0
0	0	0	0	0	0	0	0	0	0	0	0	0	0	0	0	0	0	0
0	0	0	0	0	0	0	0	0	0	0	0	0	0	0	0	0	0	0
0	0	0	0	0	0	0	0	0	0	0	0	0	0	0	0	0	0	0
0	0	0	5	0	0	0	0	0	0	0	0	0	0	0	0	0	0	0
0	0	0	0	0	0	0	0	0	0	0	0	0	0	0	0	0	0	0
0	0	0	0	0	0	0	0	0	0	0	0	0	0	0	0	0	0	0
0	0	0	0	0	0	0	0	0	0	0	0	0	0	0	0	0	0	0
0	0	0	0	0	0	0	0	0	0	0	0	0	0	0	0	0	0	0
0	0	0	0	0	0	0	0	0	0	0	0	0	0	0	0	0	0	0
0	0	0	0	0	0	0	0	9	0	0	0	0	0	0	0	0	0	0
0	0	0	0	0	0	0	0	0	0	0	0	0	0	0	0	0	0	0
0	0	0	0	0	0	0	0	0	0	0	0	0	0	0	0	0	0	0
0	0	0	0	0	0	0	0	0	0	0	0	0	0	0	0	0	0	0
0	0	0	0	0	0	0	0	0	0	0	0	0	0	0	0	0	0	0
0	0	0	0	0	0	0	0	0	0	0	0	0	0	0	0	0	0	0
0	0	0	0	0	0	0	0	0	0	0	0	0	0	0	0	0	0	0
0	0	0	0	0	0	0	0	0	0	0	0	0	0	0	0	0	0	0
0	0	0	0	0	0	0	0	0	0	0	0	0	0	0	0	0	0	0
0	0	0	0	0	0	0	0	0	0	0	0	0	0	0	0	0	0	0
0	0	0	0	0	0	0	0	0	0	0	0	0	0	0	0	0	0	0
0	0	0	0	0	0	0	0	0	0	0	0	0	0	0	0	0	0	0
0	0	0	0	0	0	0	0	0	0	0	0	0	0	0	0	0	0	0
0	0	0	0	0	0	0	0	0	0	0	0	0	0	0	0	0	0	0
0	0	0	0	0	0	0	0	0	0	0	0	0	0	0	0	0	0	0
0	0	0	0	0	0	0	0	0	0	0	0	0	0	0	0	0	0	0
0	0	0	0	0	0	0	0	0	0	0	0	0	0	0	0	9	0	0
0	0	0	0	0	0	0	0	0	0	0	0	0	0	0	0	0	0	0

0	0	0	0	0	0	0	0	0	0	0	0	0	0	0	0	0	0	0
0	0	0	0	0	0	0	0	0	0	0	0	0	0	0	0	0	0	0
0	0	0	0	0	0	0	0	0	0	0	0	0	0	0	0	0	0	0
0	0	0	0	0	0	0	0	0	0	0	0	0	0	0	0	0	0	0
0	0	0	0	0	0	0	0	0	0	0	0	0	0	0	0	0	2	0
0	0	0	0	0	0	0	0	0	0	0	0	0	0	0	0	0	0	0
0	0	0	0	0	0	0	0	0	0	0	0	0	0	0	0	0	0	0
0	0	0	0	0	0	0	0	0	0	0	0	0	0	0	0	0	0	0
0	0	0	0	0	0	0	0	0	0	0	0	0	0	0	0	0	0	0
0	0	0	0	0	0	0	0	0	0	0	0	0	0	0	0	0	0	0
0	0	0	0	0	0	0	0	0	0	0	0	0	0	0	0	0	0	0
0	0	0	0	0	0	0	0	0	0	0	0	0	0	0	0	0	0	0
0	0	0	0	0	0	0	0	0	0	0	0	0	0	0	0	0	0	0
0	0	0	0	0	0	0	0	0	0	0	0	0	0	0	0	0	0	0
0	0	0	0	0	0	0	0	0	0	0	0	0	0	0	0	0	0	0
0	0	0	0	0	0	0	0	0	0	0	0	0	0	0	0	0	0	0
0	0	0	0	0	0	0	0	0	0	0	0	0	0	0	0	0	0	0
0	0	0	0	0	0	0	0	0	0	0	0	0	0	0	0	0	0	0
0	0	0	0	0	0	0	0	0	0	0	0	0	0	0	0	0	0	0
0	0	0	0	0	0	0	0	0	0	0	0	0	0	0	0	0	0	0
0	0	0	0	0	0	0	0	0	0	0	0	0	0	0	0	0	0	0
0	0	0	0	0	0	0	0	0	0	0	0	0	0	0	0	0	0	0
0	0	0	0	0	0	0	0	0	0	0	0	0	0	0	0	0	0	0
0	0	0	0	0	0	0	0	0	0	0	0	0	0	0	0	0	0	0
0	0	0	0	0	0	0	0	0	0	0	0	0	0	0	0	0	0	0
0	0	0	0	0	0	0	0	0	0	0	0	0	0	0	0	0	0	0
0	0	0	0	0	0	0	0	0	0	0	0	0	0	0	0	0	0	0
0	0	0	0	0	0	0	0	0	0	0	0	0	0	0	0	0	0	0
0	0	0	0	0	0	0	0	0	0	0	0	0	0	0	0	0	0	0
0	0	0	0	0	0	0	0	0	0	0	5	0	0	0	0	0	0	0
0	0	0	0	0	0	0	0	0	0	0	0	0	0	0	0	0	0	0
0	0	0	0	0	0	0	0	0	0	0	0	0	0	0	0	0	0	0
0	0	0	0	0	0	0	0	0	0	0	0	0	0	0	0	0	0	0
0	0	0	0	0	0	0	0	0	0	0	0	0	0	0	0	0	0	0
0	0	0	0	0	0	0	0	0	0	0	0	0	0	0	0	0	0	0
0	0	0	0	0	0	0	0	0	0	0	0	0	0	0	0	0	0	0
0	0	0	0	0	0	0	0	0	0	0	0	0	0	0	0	0	0	0

0	0	0	0	0	0	0	0	0	0	0	0	0	0	0	0	0	0	0
0	0	0	0	0	0	0	0	0	0	0	0	0	0	0	0	0	0	0
0	0	0	0	0	0	0	0	0	0	0	0	0	0	0	0	0	0	0
0	0	0	0	0	0	0	0	0	0	0	0	0	0	0	0	0	0	0
0	0	0	0	0	0	0	0	0	0	0	0	0	0	0	0	0	0	0
0	0	0	0	0	0	0	0	0	0	0	0	0	0	0	0	0	0	0
0	0	0	0	0	0	0	0	0	0	0	0	0	0	0	0	0	0	0
0	0	0	0	0	0	0	0	0	0	0	0	0	0	0	0	0	0	0
0	0	0	0	0	0	0	0	0	0	0	0	0	0	0	0	0	0	0
0	0	0	0	0	0	0	0	0	0	0	0	0	0	0	0	0	0	0
0	0	0	0	0	0	0	0	0	0	0	0	0	0	0	0	0	0	0
0	0	0	0	0	0	0	0	0	0	0	0	0	0	0	0	0	0	0
0	0	0	0	0	0	0	0	0	0	0	0	0	0	0	0	0	0	0
0	0	0	0	0	0	0	0	0	0	0	0	0	0	0	0	0	0	0
0	0	0	0	0	0	0	0	0	0	0	0	0	0	0	0	0	0	0
0	0	0	0	0	0	0	0	0	0	0	0	0	0	0	0	0	0	0
0	0	0	0	0	0	0	0	0	0	0	0	0	0	0	0	0	0	0
0	0	0	0	0	0	0	0	0	0	0	0	0	0	0	0	0	0	0
0	0	0	0	0	0	0	0	0	0	0	0	0	0	0	0	0	0	0
0	0	0	0	0	0	0	0	0	0	0	0	0	0	0	0	0	0	0
0	0	0	0	0	0	0	0	0	0	0	0	0	0	0	0	0	0	0
0	0	0	0	0	0	0	0	0	0	0	0	0	0	0	0	0	0	0
0	0	0	0	0	0	0	0	0	0	0	0	0	0	0	0	0	0	0
0	0	0	0	0	0	0	0	0	0	0	0	0	0	0	0	0	0	0
0	0	0	0	0	0	0	0	0	0	0	0	0	0	0	0	0	0	0
0	0	0	0	0	0	0	0	0	0	0	0	0	0	0	0	0	0	0
0	0	0	0	0	0	0	0	0	0	0	0	0	0	0	0	0	0	0
0	0	0	0	0	0	0	0	0	0	0	0	0	0	0	0	0	0	0
0	0	0	0	0	0	0	0	0	0	0	0	0	0	0	0	0	0	0
0	0	0	0	0	0	0	0	0	0	0	0	0	0	0	0	0	0	0
0	0	0	0	0	0	0	0	0	0	0	0	0	0	0	0	0	0	0
0	0	0	0	0	0	0	0	0	0	0	0	0	0	0	0	0	0	0
0	0	0	0	0	0	0	0	0	0	0	0	0	0	0	0	0	0	0
0	0	0	0	0	0	0	0	0	0	0	0	0	0	0	0	0	0	0
0	0	0	0	0	0	0	0	0	0	0	0	0	0	0	0	0	0	0
0	0	0	0	0	0	0	0	0	0	0	0	0	0	0	0	0	0	0
0	0	0	0	0	0	0	0	0	0	0	0	0	0	0	0	2	2	2

	2	0	0	0	0	0	0	0	0	0	0	0	0	0	0	0	0	0	0
	0	0	0	0	0	0	0	0	0	0									
Jan_DL1	40	1082	1389	181	495	1996	473	827	749	220	75	195	208	195	377	170	28	337	273
	190	32	135	0	234	157	41	194	44	237	216	131	98	89	83	77	141	56	106
	192	259	139	45	62	81	103	0	152	14	175	34	130	76	35	34	0	133	75
	77	12	0	210	141	81	109	78	11	64	144	72	0	96	33	55	81	0	130
	21	12	50	52	102	21	25	0	43	22	47	64	39	0	51	66	30	42	26
	101	21	121	0	0	165	0	0	0	5	50	77	28	12	8	40	37	0	0
	0	0	5	3	10	64	0	0	0	0	10	0	39	59	0	0	49	0	0
	0	9	0	0	22	0	0	0	84	25	0	0	24	0	49	0	0	0	0
	0	49	0	0	0	56	8	0	0	0	24	0	0	0	0	0	24	0	10
	10	0	13	0	0	0	0	76	0	0	0	6	41	0	0	64	5	0	0
	0	0	27	0	0	0	0	39	0	18	9	0	53	0	0	0	0	0	0
	0	46	0	0	36	0	0	0	0	0	0	0	0	0	0	0	0	0	24
	0	0	0	0	0	7	0	0	9	0	0	0	13	11	0	0	18	0	96
	19	0	0	0	0	0	0	0	0	0	0	0	0	0	14	0	0	0	0
	7	0	23	39	22	0	0	0	0	0	0	0	0	22	21	9	0	0	0
	0	15	0	0	0	0	0	0	0	0	0	0	7	0	27	0	0	25	0
	0	9	0	0	0	0	0	0	0	23	0	0	18	9	0	0	0	0	0
	0	0	0	0	0	0	3	7	5	0	0	0	0	0	32	9	0	0	0
	7	7	0	0	6	16	0	0	0	21	0	19	0	0	0	0	0	0	0
	0	0	0	0	0	0	0	0	0	0	0	0	0	0	0	0	0	0	6
	0	0	0	0	0	0	0	0	0	0	0	0	15	0	0	44	0	0	0
	0	0	0	0	0	0	0	3	0	0	27	10	0	0	0	0	0	0	0
	0	0	0	0	0	0	0	0	0	0	0	0	0	0	0	0	0	0	0
	0	4	0	7	0	0	14	0	0	0	0	0	0	0	0	0	0	7	14
	0	10	0	0	0	0	0	0	0	0	0	0	0	0	0	0	0	0	0
	0	0	0	0	0	0	0	0	0	0	0	0	50	0	0	0	0	0	0
	0	0	0	0	0	0	0	14	12	14	0	0	0	0	0	0	0	0	13
	0	0	0	0	0	8	0	0	0	0	0	0	0	0	0	0	0	0	0
	0	0	0	0	0	0	0	0	0	0	0	0	0	0	0	0	0	0	0
	0	0	0	0	0	0	0	0	0	0	0	0	0	0	0	0	0	0	0
	0	10	0	3	0	0	0	0	0	0	0	0	0	0	0	0	0	0	0
	0	0	0	0	0	0	0	0	8	13	0	0	10	0	0	0	0	0	0
	0	0	0	0	0	0	0	0	0	0	0	0	0	0	0	0	0	6	0
	0	11	0	0	0	0	9	0	0	0	0	0	0	0	0	0	0	0	0
	0	17	0	0	0	0	0	0	0	0	1	0	4	0	0	0	0	4	0

0	0	0	0	0	0	0	0	0	10	8	0	0	0	0	0	0	0	0
0	0	18	0	0	0	0	0	0	0	0	0	0	0	0	0	0	0	0
0	0	0	0	0	0	0	0	0	0	0	0	0	0	0	0	0	32	0
0	0	0	0	0	0	0	0	0	0	0	0	0	0	0	0	0	0	0
0	0	0	19	0	0	0	0	0	0	0	0	0	0	0	0	0	0	0
0	0	0	0	0	0	11	0	0	0	0	0	0	0	6	0	0	0	0
0	0	0	0	0	0	0	3	0	0	0	0	0	0	0	0	0	0	0
0	0	0	0	0	0	0	0	0	0	0	0	0	0	0	0	0	0	0
0	0	0	0	0	0	0	0	0	0	0	0	0	0	0	0	0	0	0
0	0	0	4	0	0	0	0	0	0	0	0	0	0	0	0	0	0	0
0	0	0	0	0	0	0	0	0	0	0	0	0	0	0	0	0	0	0
0	0	0	0	0	0	0	0	0	0	0	0	0	0	0	0	0	0	0
0	0	0	0	0	0	0	0	0	0	0	0	0	0	0	0	0	0	11
0	0	0	0	0	0	0	0	0	0	0	0	0	0	0	0	0	0	0
0	0	0	0	0	0	0	0	0	0	0	0	0	0	0	0	0	24	0
0	0	0	0	0	0	0	0	0	0	0	0	0	0	0	0	0	0	0
0	0	0	0	0	0	0	0	0	0	0	0	0	0	0	0	0	0	0
0	0	0	0	0	0	0	0	0	0	0	0	0	0	0	0	0	0	0
0	0	0	0	0	0	0	0	0	0	0	0	0	0	0	0	0	0	0
0	0	0	0	0	0	0	0	0	0	0	0	0	0	0	0	0	0	0
0	0	0	0	0	0	0	0	0	0	0	0	0	0	0	4	0	0	0
0	0	0	0	0	0	0	0	0	0	0	0	0	0	0	0	0	0	0
0	0	0	0	0	0	0	0	0	0	0	0	0	0	0	0	0	0	0
0	0	0	0	0	0	0	0	0	0	0	0	0	0	0	0	0	0	0
0	0	0	0	0	0	0	0	0	0	0	0	0	0	0	0	0	0	0
0	0	0	0	0	0	0	0	0	0	0	0	0	0	0	0	0	0	0
0	0	0	0	0	0	0	0	0	0	0	0	0	0	0	0	0	0	7
0	0	0	0	0	0	0	0	0	0	0	0	0	0	0	0	0	0	0
0	0	0	0	0	0	0	0	0	0	0	0	0	0	0	0	0	0	0
0	0	0	0	0	0	0	0	0	0	0	0	0	0	0	0	0	0	0
0	0	0	0	0	0	0	0	0	0	0	0	0	0	0	0	0	0	0
0	0	0	0	0	0	0	0	0	0	0	0	0	0	0	0	7	0	0
0	0	0	0	0	0	0	0	0	0	0	0	0	16	0	0	0	0	0
0	0	0	0	0	0	0	0	0	0	0	0	0	0	0	0	0	0	0
0	0	0	0	0	0	0	0	0	0	0	0	0	0	0	0	0	0	0
0	0	0	0	0	0	0	0	0	0	0	0	0	0	0	0	0	0	0
0	0	0	0	0	0	0	0	0	0	0	0	0	0	0	0	0	0	0

0	0	0	0	0	0	0	0	0	0	0	0	0	0	0	0	0	0	0
0	0	0	0	0	0	0	0	0	0	0	0	0	0	0	0	0	0	0
0	0	0	0	0	0	0	0	0	0	0	0	0	0	0	0	0	0	0
0	0	0	0	0	0	0	0	0	0	0	0	0	0	0	0	0	0	0
0	0	0	0	0	0	0	0	0	0	0	0	0	0	0	0	0	0	0
0	0	0	0	0	0	0	0	0	0	0	0	0	0	0	0	0	0	0
0	0	0	0	0	0	0	0	0	0	0	0	0	0	0	0	0	0	0
0	0	0	0	0	0	0	0	0	0	0	0	0	0	0	0	0	0	0
0	0	0	0	0	0	0	0	0	0	0	0	0	0	0	0	0	0	0
0	0	0	0	0	0	0	0	0	0	0	0	0	0	0	0	0	0	0
0	0	0	0	0	0	0	0	0	0	0	0	0	0	0	0	0	0	0
0	0	0	0	0	0	0	0	0	0	0	0	0	0	0	0	0	0	0
0	0	0	0	0	0	0	0	0	0	0	0	0	0	0	0	0	0	0
0	0	0	0	0	0	0	0	0	0	0	0	0	0	0	0	0	0	0
0	0	0	0	0	0	0	0	0	0	0	0	0	0	0	0	0	0	0
0	0	0	0	0	0	0	0	0	0	0	0	0	0	0	0	0	0	0
0	0	0	0	0	0	0	0	0	0	0	0	0	0	0	0	0	0	0
0	0	0	0	0	0	0	0	0	0	0	0	0	0	0	0	0	0	0
0	0	0	0	0	0	0	0	0	0	0	0	0	0	0	0	0	0	0
0	0	0	0	0	0	0	0	0	0	0	0	0	0	0	0	0	0	0
0	0	0	0	0	0	0	0	0	0	0	0	0	0	0	0	0	0	0
0	0	0	0	0	0	0	0	0	0	0	0	0	7	7	0	0	0	0
0	0	0	0	0	0	0	0	0	0	0	0	0	0	0	0	0	0	0
0	0	0	0	0	0	0	0	0	0	0	0	0	0	0	0	0	0	0
0	0	0	0	0	0	0	0	0	0	0	0	0	0	0	0	0	0	0
0	0	0	0	0	0	0	0	0	0	0	0	0	0	0	0	0	0	0
0	0	0	0	0	0	0	0	0	0	0	0	0	0	0	0	0	0	0
0	0	0	0	0	0	0	0	0	0	0	0	0	0	0	0	0	0	0
0	0	0	0	0	0	0	0	0	0	0	0	0	0	0	0	0	0	0
0	0	0	0	0	0	0	0	0	0	0	0	0	0	0	0	0	0	0
0	0	0	0	0	0	0	0	0	0	0	0	0	0	0	0	0	0	0
0	0	0	0	0	0	0	0	0	0	0	0	0	0	0	0	0	0	0
0	0	0	0	0	0	0	0	0	0	0	0	0	0	0	0	0	0	0
0	0	0	0	0	0	0	0	0	0	0	0	0	0	0	0	0	0	0
0	0	0	0	0	0	0	0	0	0	0	0	0	0	0	0	0	0	0
0	0	0	0	0	0	0	0	0	0	0	0	0	0	0	0	0	0	0
0	0	0	0	0	0	0	0	0	0	0	0	0	0	0	0	0	0	0

0	0	0	0	0	0	0	0	0	0	0	0	0	0	0	0	0	0	5
0	0	0	0	0	0	0	0	0	0	0	0	0	0	0	0	0	0	0
0	0	0	0	0	0	0	0	0	0	0	0	0	0	0	0	0	0	0
0	0	0	0	0	0	0	0	0	0	0	0	0	0	0	0	0	0	0
0	0	0	0	0	0	0	0	0	0	0	0	0	0	0	0	0	0	0
0	0	0	0	0	0	0	0	0	0	0	0	0	0	0	0	0	0	0
0	0	0	0	0	0	0	0	0	0	0	0	0	0	0	0	0	0	0
0	0	0	0	0	0	0	0	0	0	0	0	0	0	0	0	0	0	0
0	0	0	0	0	0	0	0	0	0	0	0	0	0	0	0	0	0	0
0	0	0	0	0	0	0	0	0	0	0	0	0	0	0	0	0	0	0
0	0	0	0	0	0	0	0	0	0	0	0	0	0	0	0	0	0	0
0	0	0	0	0	0	0	0	0	0	0	0	0	4	4	0	0	0	0
0	0	0	0	0	0	0	0	0	0	0	0	0	0	0	0	0	0	0
0	0	0	0	0	0	0	0	0	0	0	0	0	0	0	0	0	0	0
0	0	0	0	0	0	0	0	0	0	0	0	0	0	0	0	0	0	0
0	0	0	0	0	0	0	0	0	0	0	0	0	0	0	0	0	0	0
0	0	0	0	0	0	0	0	0	0	0	0	0	0	0	0	0	0	0
0	0	0	0	0	0	0	0	0	0	0	0	0	0	0	0	0	0	0
0	0	0	0	0	0	0	0	0	0	0	0	0	0	0	0	0	0	0
0	0	0	0	0	0	0	0	0	0	0	0	0	0	0	0	0	0	0
0	0	0	0	0	0	0	0	0	0	0	0	0	0	0	0	0	0	0
0	0	0	0	0	0	0	0	0	0	0	0	0	0	0	0	0	0	0
0	0	0	0	0	0	0	0	0	0	0	0	0	0	0	0	0	0	0
0	0	0	0	0	0	0	0	0	0	0	0	0	0	0	0	0	0	0
0	0	0	0	0	0	0	0	0	0	0	0	0	0	0	0	0	0	0
0	0	0	0	0	0	0	0	0	0	0	0	0	0	0	0	0	0	0
0	0	0	0	0	0	0	0	0	0	0	0	0	0	0	0	0	0	0
0	0	0	0	0	0	0	0	0	0	0	0	0	0	0	0	0	0	0
0	0	0	0	0	0	0	0	0	0	0	0	0	0	0	0	0	0	0
0	0	0	0	0	0	0	0	0	0	0	0	0	0	0	0	0	0	0
0	0	0	0	0	3	3	3	0	0	0	0	0	0	0	0	0	0	0
0	0	0	0	0	0	0	0	0	0	0	0	0	0	0	0	0	0	0
0	0	0	0	0	0	0	0	0	0	0	0	0	0	0	0	0	0	0
0	0	0	0	0	0	0	0	0	0	0	0	0	0	0	0	0	0	0
0	0	0	0	0	0	0	0	0	0	0	0	0	0	0	0	0	0	0
0	0	0	0	0	0	0	0	0	0	0	0	0	0	0	0	0	0	0
0	0	0	0	0	0	0	0	0	0	0	0	0	0	0	0	0	0	0

0	0	0	0	0	0	0	0	0	0	0	0	0	0	0	0	0	0	0
0	0	0	0	0	0	0	0	0	0	0	0	0	0	0	0	0	0	0
0	0	0	0	0	0	0	0	0	0	0	0	0	0	0	0	0	0	0
0	0	0	0	0	0	0	0	0	0	0	0	0	0	0	0	0	0	0
0	0	0	0	0	0	0	0	0	0	0	0	0	0	0	0	0	0	0
0	0	0	0	0	0	0	0	0	0	0	0	0	0	0	0	0	0	0
0	0	0	0	0	0	0	0	0	0	0	0	0	0	0	0	0	0	0
0	0	0	0	0	0	0	0	0	0	0	0	0	0	0	0	0	0	0
0	0	0	0	0	0	0	0	0	0	0	0	0	0	0	0	0	0	0
0	0	0	0	0	0	0	0	0	0	0	0	0	0	0	0	0	0	0
0	0	0	0	0	0	0	0	0	0	0	0	0	0	0	0	0	0	0
0	0	0	0	0	0	0	0	0	0	0	0	0	0	0	0	0	0	0
0	0	0	0	0	0	0	0	0	0	0	0	0	0	0	0	0	0	0
0	0	0	0	0	0	0	0	0	0	0	0	0	0	0	0	0	0	0
0	0	0	0	0	0	0	0	0	0	0	0	0	0	0	0	0	0	0
0	0	0	0	0	0	0	0	0	0	0	0	0	0	0	0	0	0	0
0	0	0	0	0	0	0	0	0	0	0	0	0	0	0	0	0	0	0
0	0	0	0	0	0	0	0	0	0	0	0	0	0	0	0	0	0	0
0	0	0	0	0	0	0	0	0	0	0	0	0	0	0	0	0	0	0
0	0	0	2	2	2	2	2	2	2	2	2	2	2	2	0	0	0	0
0	0	0	0	0	0	0	0	0	0	0	0	0	0	0	0	0	0	0
0	0	0	0	0	0	0	0	0	0	0	0	0	0	0	0	0	0	0
0	0	0	0	0	0	0	0	0	0	0	0	0	0	0	0	0	0	0
0	0	0	0	0	0	0	0	0	0	0	0	0	0	0	0	0	0	0
0	0	0	0	0	0	0	0	0	0	0	0	0	0	0	0	0	0	0
0	0	0	0	0	0	0	0	0	0	0	0	0	0	0	0	0	0	0
0	0	0	0	0	0	0	0	0	0	0	0	0	0	0	0	0	0	0
0	0	0	0	0	0	0	0	0	0	0	0	0	0	0	0	0	0	0
0	0	0	0	0	0	0	0	0	0	0	0	0	0	0	0	0	0	0
0	0	0	0	0	0	0	0	0	0	0	0	0	0	0	0	0	0	0
0	0	0	0	0	0	0	0	0	0									
Jan_DL141	1574	1326	2174	376	40	169	165	167	649	302	417	541	299	158	215	239	169	353
226	195	169	0	233	145	317	423	305	153	142	0	315	193	152	238	345	80	0
144	298	65	559	81	205	204	522	79	75	36	205	133	0	141	218	330	52	0
0	306	250	39	25	65	29	68	119	20	27	0	155	0	16	15	16	86	0
69	0	79	28	27	40	79	74	49	85	145	75	51	71	39	168	18	17	203
45	39	0	52	85	0	132	21	102	69	0	0	0	0	40	31	20	0	0

0	0	22	70	9	44	0	0	0	174	34	40	18	0	34	46	0	0	35
181	0	0	0	0	68	0	0	0	28	0	0	10	41	35	0	0	0	0
0	0	57	0	158	3	89	0	0	0	12	0	128	0	30	0	6	0	42
34	123	12	0	0	0	0	0	0	0	0	40	0	29	0	0	36	0	0
0	0	8	32	0	41	58	0	0	0	9	0	0	0	8	0	0	0	0
0	0	19	0	0	0	0	0	0	0	33	0	0	47	0	0	0	18	0
0	379	21	0	0	3	0	20	0	52	0	15	15	10	12	0	0	0	0
0	0	0	0	24	0	0	0	0	13	96	96	0	11	0	0	21	0	0
12	0	59	0	0	7	0	0	10	33	0	13	0	0	10	6	0	62	0
0	0	4	0	0	0	0	0	11	69	32	28	0	35	0	8	0	0	0
0	9	0	81	0	0	0	0	0	0	0	0	5	18	0	0	0	0	0
11	0	8	27	0	0	0	9	0	0	0	0	0	0	0	0	9	38	8
0	0	0	44	0	4	0	0	15	7	0	0	0	0	22	0	0	0	0
0	0	8	0	0	0	0	0	0	0	0	0	0	0	0	0	0	0	0
0	42	0	0	14	0	13	0	0	0	0	14	7	0	21	0	0	13	0
0	70	0	0	0	0	0	0	0	0	0	0	0	0	0	3	0	0	0
0	12	0	0	0	0	0	0	0	0	0	0	0	0	0	0	0	0	0
0	16	0	0	104	0	0	0	194	0	36	0	14	0	0	0	0	0	0
0	0	0	0	0	0	0	9	0	0	5	0	0	0	0	0	55	0	0
83	59	0	0	0	0	0	5	0	0	0	0	0	0	0	0	0	0	4
0	0	6	0	0	0	0	0	0	0	0	53	0	0	0	0	6	6	0
0	0	20	0	0	0	0	0	0	0	0	0	0	0	0	6	0	0	0
0	0	0	0	0	0	0	0	0	0	0	0	32	0	0	0	0	0	0
0	0	0	0	8	0	0	0	0	0	0	0	0	0	0	0	0	15	46
13	0	10	0	0	0	0	0	0	0	60	20	0	0	0	0	0	21	0
0	0	0	0	0	0	0	7	0	0	0	0	0	0	0	0	0	0	0
0	0	0	0	0	0	0	0	0	0	0	0	0	12	0	0	0	0	16
0	0	0	0	0	0	0	0	0	0	6	0	0	0	0	0	0	0	0
0	0	0	0	0	0	0	0	0	0	0	0	0	0	0	0	15	0	0
0	0	0	0	0	0	0	0	0	0	0	0	0	0	0	0	0	0	0
0	0	0	0	7	0	0	0	0	0	16	0	0	0	0	0	0	0	0
0	0	0	0	0	0	0	0	0	0	0	0	0	0	0	0	0	0	0
0	0	0	0	0	0	0	0	0	0	0	0	0	0	0	0	0	0	0
5	0	0	0	0	0	0	0	0	0	0	0	0	0	0	0	0	0	0
0	0	0	0	5	0	0	6	0	0	0	0	0	0	0	0	0	15	0
0	2	0	0	0	0	0	0	0	0	0	0	0	0	0	0	0	0	5
0	0	0	0	0	0	0	0	0	0	0	0	0	0	0	0	0	0	0

0	0	0	0	0	0	0	0	0	0	0	0	0	0	0	0	0	0	0
0	0	0	0	0	0	0	0	0	0	0	0	0	0	0	0	0	0	0
0	0	0	0	0	0	0	0	0	0	0	0	0	0	0	0	0	0	0
0	0	0	0	0	0	0	0	0	0	0	15	0	0	0	0	0	0	0
0	0	0	0	0	0	0	0	0	0	0	0	0	0	0	0	0	67	0
0	0	0	0	0	0	0	0	0	0	0	0	0	0	0	0	0	0	0
0	0	0	0	0	0	0	0	0	0	0	0	0	0	0	0	0	0	0
0	0	0	0	0	0	0	0	0	0	0	0	0	0	0	0	0	0	0
0	0	0	0	0	0	0	0	0	0	0	0	0	0	0	0	0	0	0
0	0	0	0	0	0	0	0	0	0	0	0	0	0	0	0	0	0	0
0	0	0	0	0	46	0	0	0	0	0	0	7	0	0	0	0	0	0
0	0	0	0	0	0	7	0	0	0	0	0	0	0	0	0	0	0	0
0	0	0	0	0	0	0	0	0	9	0	0	0	0	0	0	0	0	0
0	0	0	0	0	0	0	0	0	0	0	0	0	0	0	0	0	0	0
0	0	0	0	0	0	0	0	0	0	0	0	0	0	0	0	0	0	0
0	0	0	0	0	0	0	0	0	0	0	0	0	0	0	0	0	0	0
0	0	0	0	0	0	0	0	0	0	0	0	0	0	0	0	0	0	0
0	0	0	0	0	0	0	0	0	0	0	0	0	0	0	0	0	0	0
0	0	0	0	0	0	0	0	0	0	0	0	0	0	0	0	0	0	0
0	0	0	0	0	0	0	0	0	0	0	0	0	0	0	0	0	0	0
0	0	0	0	0	0	0	0	0	0	0	0	0	0	0	0	0	0	0
0	0	0	0	0	0	0	0	0	0	0	0	0	0	0	0	0	0	0
0	0	0	0	0	0	0	5	0	0	0	0	0	0	0	0	0	0	0
0	0	0	0	0	0	0	0	0	0	0	0	0	0	4	0	0	0	0
0	0	0	0	0	0	0	0	0	0	0	0	0	0	0	0	0	0	0
0	0	0	0	0	0	0	0	0	0	0	0	0	0	0	0	0	0	0
0	0	0	0	0	0	0	0	0	0	0	4	0	0	0	0	0	0	0
0	0	0	0	0	0	0	0	0	0	0	0	0	0	0	0	0	0	0
0	0	0	0	0	0	0	0	0	0	0	0	0	0	0	0	0	0	0
0	0	0	0	0	0	0	0	0	0	0	0	0	0	0	0	0	0	0
0	0	0	0	0	0	0	0	0	17	0	0	0	0	0	0	0	0	0
0	0	0	0	0	0	0	0	0	0	0	0	0	0	0	0	0	0	0
0	0	0	0	0	0	0	0	0	0	0	0	0	0	0	0	0	0	0
0	0	0	0	0	0	0	0	0	0	0	0	0	0	0	0	0	0	0
0	0	0	0	0	0	0	0	0	0	0	0	0	0	0	0	0	0	0
0	0	0	0	0	0	3	0	0	0	0	0	0	0	0	0	0	0	0
0	0	0	0	0	0	0	0	0	0	0	0	0	0	0	0	0	0	0

0	0	0	0	0	0	0	0	0	0	0	0	0	0	0	0	0	0	0
0	0	0	0	0	0	0	0	0	0	0	0	0	0	0	0	0	0	0
0	0	0	0	0	0	0	0	0	0	0	0	0	0	0	0	0	0	0
0	0	0	0	0	0	0	0	0	0	0	0	0	0	0	0	0	0	0
0	0	0	0	0	0	0	0	0	0	0	0	0	0	0	0	0	0	0
0	0	0	0	0	0	0	0	0	0	0	0	0	0	0	0	0	0	0
0	0	0	0	0	0	0	0	0	0	0	0	0	0	0	0	0	0	0
0	0	0	0	0	0	0	0	0	0	0	0	0	0	0	0	0	0	0
0	0	0	0	0	0	0	0	0	0	0	0	0	0	0	0	0	0	0
0	0	0	0	0	0	0	0	0	0	0	0	0	0	0	0	0	0	0
0	0	0	0	0	0	0	0	0	0	0	0	0	0	0	0	0	0	0
0	0	0	0	0	0	0	0	0	0	0	0	0	0	0	0	0	0	0
0	0	0	0	0	0	0	0	0	0	0	0	0	0	0	0	0	0	0
0	0	0	0	0	0	0	0	0	0	0	0	0	0	0	0	0	0	0
0	0	0	0	0	0	0	0	0	0	0	0	0	0	0	0	0	0	0
0	0	0	0	0	0	0	0	0	0	0	0	0	0	0	0	0	0	0
0	0	0	0	0	0	0	0	0	0	0	0	0	0	0	0	0	0	0
0	0	0	0	0	0	0	0	0	0	0	0	0	0	0	0	0	0	0
0	0	0	0	0	0	0	0	0	0	0	0	0	0	0	0	0	0	0
0	0	0	0	0	0	0	0	0	0	0	0	0	0	0	0	0	0	0
0	0	0	0	0	0	0	0	0	0	0	0	0	0	0	0	0	0	0
0	0	0	0	0	0	0	0	0	0	0	0	8	0	0	0	0	0	0
0	0	0	0	0	0	0	0	0	0	0	0	0	0	0	0	0	0	0
0	0	0	0	0	0	0	0	0	0	0	0	0	0	0	0	0	0	0
0	0	0	0	0	0	0	0	0	0	0	0	0	0	0	0	0	0	0
0	0	0	0	0	0	0	0	0	0	0	0	0	0	0	0	0	0	0
0	0	0	0	0	0	0	0	0	3	0	0	0	0	0	0	0	0	0
0	0	0	0	0	0	0	0	0	0	0	0	0	0	0	0	0	0	0
0	0	0	0	0	0	0	0	0	0	0	0	0	0	0	0	0	0	0
0	0	0	0	0	0	0	0	0	0	0	0	0	0	0	0	0	0	0
0	0	0	0	0	0	0	0	0	0	0	0	0	0	0	0	0	0	0
0	0	0	0	0	0	0	0	0	0	0	0	0	0	0	0	0	0	0
0	0	0	0	0	0	0	0	0	0	0	0	0	0	0	0	0	0	0
0	0	0	0	0	0	0	0	0	0	0	0	0	0	0	0	0	0	0
0	0	0	0	0	0	0	0	0	0	0	0	0	0	0	0	0	0	0
0	0	0	0	0	0	0	0	0	0	0	0	0	0	0	0	0	0	0
0	0	0	0	0	0	0	0	0	0	0	0	0	0	0	0	0	0	0

0	0	0	0	0	0	0	0	0	0	0	0	0	0	0	0	0	0	0
0	0	0	0	0	0	0	0	0	0	0	0	0	0	0	0	0	0	0
0	0	0	0	0	0	0	0	0	0	0	0	0	0	0	0	0	0	0
0	0	0	0	0	0	0	0	0	0	0	0	0	0	0	0	0	0	0
0	0	0	0	0	0	0	0	0	0	0	0	0	0	0	0	0	0	0
0	0	0	0	0	0	0	0	0	0	0	0	0	0	0	0	0	0	0
0	0	0	0	0	0	0	0	0	0	0	0	0	0	0	0	0	0	0
0	0	0	0	0	0	0	0	0	0	0	0	0	0	0	0	0	0	0
0	0	0	0	0	0	0	0	0	0	0	0	0	5	0	0	0	0	0
0	0	0	0	0	0	0	0	0	0	0	0	0	0	0	0	0	0	0
0	0	0	0	0	0	0	0	0	0	0	0	0	0	0	0	0	0	0
0	0	0	0	0	0	0	0	0	0	0	0	0	0	0	0	0	0	0
0	0	0	0	0	0	0	0	0	0	0	0	0	0	0	0	0	0	0
0	0	0	0	0	0	0	0	0	0	0	0	0	0	0	0	0	0	0
0	0	0	0	0	0	0	0	0	0	0	0	0	0	0	0	0	0	0
0	0	0	0	0	0	0	0	0	0	0	0	0	0	0	0	0	0	0
0	0	0	0	0	0	0	0	0	0	0	0	0	0	0	0	0	0	0
0	0	0	0	0	0	0	0	0	0	0	0	0	0	0	0	0	0	0
0	0	0	0	0	0	0	0	0	0	0	0	0	0	0	0	0	0	0
0	0	0	0	0	0	0	0	0	0	0	0	0	0	0	0	0	0	0
0	0	0	0	0	0	0	0	0	0	0	0	0	0	0	0	0	0	0
0	0	0	0	0	0	0	0	0	0	0	0	0	0	0	0	0	0	0
0	0	4	0	0	0	0	0	0	0	0	0	0	0	0	0	0	0	0
0	0	0	0	0	0	0	0	0	0	0	0	0	0	0	0	0	0	0
0	0	0	0	0	0	0	0	0	0	0	0	0	0	0	0	0	0	0
0	0	0	0	0	0	0	0	0	0	0	0	0	0	0	0	0	0	0
0	0	0	0	0	0	0	0	0	0	0	0	0	0	0	0	0	0	0
0	0	0	0	0	0	0	0	0	0	0	0	0	0	0	0	0	0	0
0	0	0	0	0	0	0	0	0	0	0	0	0	0	0	0	0	0	0
0	0	0	0	0	0	0	0	0	0	0	0	0	0	0	0	0	0	0
0	0	0	0	0	0	0	0	0	0	0	0	0	0	0	0	0	0	0
0	0	0	0	0	0	0	0	0	0	0	0	0	0	0	0	0	0	0
0	0	0	0	0	0	0	0	0	0	0	0	0	0	0	0	0	0	0
0	0	0	0	0	0	0	0	0	0	0	0	0	0	0	0	0	0	0
0	0	0	0	0	0	0	0	0	0	0	0	0	0	0	0	0	0	0
0	0	0	0	0	0	0	0	0	0	0	0	0	0	0	0	0	0	0
0	0	0	0	0	0	0	0	0	0	0	0	0	0	0	0	0	0	0

0	0	0	0	0	0	0	0	0	0	0	0	0	0	0	0	0	0	0
0	0	0	0	0	0	0	0	0	0	0	0	0	0	0	0	0	0	0
0	0	0	0	0	0	0	0	0	0	0	0	0	0	0	0	0	0	0
0	0	0	0	0	0	0	0	0	0	0	0	0	0	0	0	0	0	0
0	0	0	0	0	0	0	0	3	0	0	0	0	0	0	0	0	0	0
0	0	0	0	0	0	0	0	0	0	0	0	0	0	0	0	0	0	0
0	0	0	0	0	0	0	0	0	0	0	0	0	0	0	0	0	0	0
0	0	0	0	0	0	0	0	0	0	0	0	0	0	0	0	0	0	0
0	0	0	0	0	0	0	0	0	0	0	0	0	0	0	0	0	0	0
0	0	0	0	0	0	0	0	0	0	0	0	0	0	0	0	0	0	0
0	0	0	0	0	0	0	0	0	0	0	0	0	0	0	0	0	0	0
0	0	0	0	0	0	0	0	0	0	0	0	0	0	0	0	0	0	0
0	0	0	0	0	0	0	0	0	0	0	0	0	0	0	0	0	0	0
0	0	0	0	0	0	0	0	0	0	0	0	0	0	0	0	0	0	0
0	0	0	0	0	0	0	0	0	0	0	0	0	0	0	0	0	0	0
0	0	0	0	0	0	0	0	0	0	0	0	0	0	0	0	0	0	0
0	0	0	0	0	0	0	0	0	0	0	0	0	0	0	0	0	0	0
0	0	0	0	0	0	0	0	0	0	0	0	0	0	0	0	0	0	0
0	0	0	0	0	0	0	0	0	0	0	0	0	0	0	0	0	0	0
0	0	0	0	0	0	0	0	0	0	0	0	0	0	0	0	0	0	0
0	2	2	2	2	0	0	0	0	0	0	0	0	0	0	0	0	0	0
0	0	0	0	0	0	0	0	0	0	0	0	0	0	0	0	0	0	0
0	0	0	0	0	0	0	0	0	0									
Jan_DL142	1766	969	1300	993	146	23	34	42	165	720	162	191	614	31	57	207	0	111
97	345	107	163	139	83	359	290	274	249	42	0	69	135	317	64	226	334	0
268	95	30	20	100	62	86	996	46	98	29	290	32	0	29	263	67	20	0
0	206	257	26	0	138	0	99	202	79	24	0	22	0	22	0	16	261	81
111	59	0	29	0	84	46	27	0	113	0	0	17	10	28	0	22	11	220
0	88	0	0	79	11	118	0	7	37	0	0	0	15	54	23	0	232	11
0	5	0	9	29	0	0	0	0	0	0	31	0	0	45	0	0	0	0
0	138	0	0	0	79	0	0	0	22	0	0	12	0	0	16	0	0	0
151	0	23	45	0	4	0	0	131	0	6	0	0	97	13	0	0	0	0
35	17	0	0	0	0	0	0	0	0	0	62	0	0	0	14	29	0	0
0	0	0	5	0	0	0	0	0	0	0	0	0	0	45	0	0	0	0
0	0	0	0	0	13	0	0	0	0	0	0	0	0	0	0	0	0	8
0	41	0	0	0	0	0	20	85	57	0	26	0	13	42	0	0	0	0
0	0	0	0	0	0	0	0	0	29	0	78	0	0	0	0	0	0	0

11	0	0	0	0	4	0	0	0	0	0	0	0	0	0	0	0	0	0
0	0	8	0	0	0	0	64	0	0	16	16	0	33	0	7	0	0	0
0	0	9	61	0	0	0	0	0	0	7	0	7	0	0	5	0	0	10
0	14	0	32	0	0	0	8	0	0	0	0	0	0	0	0	0	0	0
8	11	0	0	14	0	0	26	0	11	0	0	20	24	0	0	13	0	0
0	0	0	0	34	0	0	52	0	0	0	49	0	0	0	0	0	0	5
54	0	0	0	0	0	8	0	0	0	19	0	0	0	7	0	0	0	0
0	0	72	0	0	0	0	0	0	0	0	0	0	0	0	0	0	0	0
0	0	0	0	0	0	0	0	4	0	0	0	0	0	0	0	4	0	0
0	7	0	0	0	18	0	0	0	0	0	0	16	0	0	0	0	0	0
0	0	0	0	0	0	0	0	0	0	176	16	0	0	0	0	0	0	0
0	0	0	0	2	0	16	0	28	11	0	8	0	0	0	0	0	14	0
0	0	11	0	0	0	0	0	0	17	0	0	0	0	0	0	0	0	0
0	0	0	0	0	0	0	0	0	0	18	0	0	0	0	0	0	11	0
0	0	0	0	0	0	0	0	0	0	19	13	0	0	0	0	0	0	0
0	0	0	0	0	0	0	0	0	0	0	0	0	0	0	0	0	22	0
0	0	0	0	0	0	0	0	0	0	0	0	0	0	0	0	0	0	0
0	0	15	0	0	0	0	0	0	0	0	0	0	0	0	0	0	0	0
0	9	0	0	0	0	0	0	0	0	0	0	0	0	0	0	0	0	0
0	0	0	0	0	0	0	0	0	0	0	0	0	0	0	0	0	0	0
0	0	0	0	0	0	0	0	0	0	0	0	0	0	0	0	0	0	0
0	0	0	0	0	0	0	0	0	0	0	0	0	0	0	0	22	0	0
0	0	0	0	0	0	0	0	0	0	0	0	0	0	0	0	0	0	0
0	0	0	0	0	0	0	0	8	0	0	0	0	0	0	0	0	0	0
0	0	0	0	0	0	0	0	0	0	0	0	0	0	0	0	0	0	0
0	0	0	0	0	0	0	0	0	0	0	0	0	0	0	0	0	0	0
0	0	0	0	0	0	0	0	0	6	0	0	0	0	0	0	0	0	0
0	0	68	0	0	0	0	0	0	0	0	0	0	0	0	0	0	0	4
0	0	0	0	0	0	0	0	0	0	0	0	0	0	0	0	0	0	0
0	0	0	0	0	0	0	0	9	0	0	0	0	0	0	0	0	0	0
0	0	0	0	11	0	0	0	0	0	0	0	0	0	0	0	0	0	0
0	0	0	0	11	0	0	0	0	0	3	0	0	0	0	3	0	0	0
0	0	0	0	0	0	0	0	0	0	0	0	0	0	0	0	0	0	0
0	0	0	0	0	0	0	0	0	0	0	0	0	0	0	0	0	0	0
0	0	0	0	0	0	0	0	0	0	0	0	0	0	0	0	0	0	0
0	0	0	0	0	0	0	0	0	0	0	0	0	0	0	0	0	0	0
0	0	0	0	0	0	0	0	0	0	0	0	0	0	0	0	0	0	0

0	0	0	0	0	0	0	0	0	0	0	0	0	0	0	0	0	0	0
0	0	0	0	0	0	0	0	0	0	0	0	0	0	0	0	0	0	0
0	0	0	0	0	0	0	0	0	0	0	0	0	0	0	0	0	0	0
0	0	0	0	0	0	0	0	0	0	0	0	0	0	0	0	0	0	0
0	0	0	0	0	0	0	0	0	0	0	0	0	0	0	0	0	0	0
0	0	0	0	0	0	0	0	0	0	0	0	0	0	0	0	0	0	0
0	0	0	0	0	0	0	0	0	12	0	0	0	0	0	0	0	0	0
0	0	0	0	0	0	0	0	0	0	0	0	0	0	0	0	0	0	0
0	0	0	0	0	0	0	0	0	0	0	0	0	0	0	0	0	0	0
0	0	0	0	0	0	0	0	0	0	0	0	0	0	0	0	0	0	0
0	0	0	0	0	0	0	0	0	0	0	0	0	0	0	0	0	0	0
0	0	0	0	0	0	0	0	0	4	0	0	0	0	0	0	0	0	0
0	0	0	0	0	0	0	0	0	0	0	0	0	0	0	0	0	0	0
0	0	0	0	0	0	0	0	0	0	0	0	0	0	0	0	0	0	0
0	0	0	0	0	0	0	0	0	0	0	0	0	0	0	0	0	0	0
0	0	0	0	0	0	0	0	0	0	0	0	0	0	0	0	0	0	0
0	0	0	0	0	0	0	0	0	0	0	0	0	0	0	0	0	0	0
0	0	0	0	0	0	0	0	0	0	0	0	0	0	0	0	0	0	0
0	0	0	0	0	0	0	0	0	0	0	0	0	0	0	0	0	0	0
0	0	0	0	0	0	0	0	0	0	0	0	0	0	0	0	0	0	0
0	0	0	0	0	0	0	0	0	0	0	0	0	0	0	0	0	0	0
0	0	0	0	0	0	0	0	0	0	0	0	0	0	0	0	0	0	0
0	0	0	0	0	0	0	0	0	0	0	0	0	0	0	0	0	0	0
0	0	0	0	0	0	0	0	0	0	0	0	0	0	0	0	0	0	0
0	0	0	0	0	0	0	0	0	0	0	0	0	0	0	0	0	0	0
0	0	0	0	0	0	0	0	0	0	0	0	0	0	0	0	0	0	0
0	0	0	0	0	0	0	0	0	0	0	0	0	0	0	0	0	0	0
0	0	0	0	0	0	0	0	0	0	0	0	0	0	0	0	0	0	0
0	0	0	0	0	0	0	0	0	0	0	0	0	0	0	0	0	0	0
0	0	0	0	0	0	0	0	0	0	0	0	0	0	0	0	0	0	0
0	0	0	0	0	0	0	0	0	0	0	0	0	0	0	0	0	0	0
0	0	0	0	0	0	0	0	0	0	0	0	0	0	0	0	0	0	0
0	0	0	0	0	0	0	0	0	0	0	0	0	0	0	0	0	0	0
0	0	0	0	4	0	0	0	0	0	0	0	0	0	0	2	0	0	0
0	0	0	0	0	0	0	0	0	0	0	0	0	0	0	0	0	0	0
0	0	0	0	0	0	0	0	0	0	0	0	0	0	0	0	0	0	0
0	0	0	0	0	0	0	0	0	0	0	0	0	0	0	0	0	0	0

0	0	0	0	0	0	0	0	0	0	0	0	0	0	0	0	0	0	0
0	0	0	0	0	0	0	0	0	0	0	0	0	0	0	0	0	0	0
0	0	0	0	0	0	0	0	0	0	0	0	8	8	0	0	0	0	0
0	0	0	0	0	0	0	0	0	0	0	0	0	0	0	0	0	0	0
0	0	0	0	0	0	0	0	0	0	0	0	0	0	0	0	0	0	0
0	0	0	0	0	0	0	0	0	0	0	0	0	0	0	0	0	0	0
0	0	0	0	0	0	0	0	0	0	0	0	0	0	0	0	0	0	0
0	0	0	0	0	0	0	0	0	0	0	0	0	0	0	0	0	0	0
0	0	0	0	0	0	0	0	0	0	0	0	0	0	0	0	0	0	0
0	0	0	0	0	0	0	0	0	0	0	0	0	0	0	0	0	0	0
0	0	0	0	0	0	0	0	0	0	0	0	0	0	0	0	0	0	0
0	0	0	0	0	0	0	0	0	0	0	0	0	0	0	0	0	0	0
0	0	0	0	0	0	0	0	0	0	0	0	0	0	0	0	0	0	0
0	0	0	0	0	0	0	0	0	0	0	0	0	0	0	0	0	0	0
0	0	0	0	0	0	0	0	0	0	0	0	0	0	0	0	0	0	0
0	0	0	0	0	0	0	0	0	0	0	0	0	0	0	0	0	0	0
0	0	0	0	0	0	0	0	0	0	0	0	0	0	0	0	0	0	0
0	0	0	0	0	0	0	0	0	0	0	0	0	0	0	0	0	0	0
0	0	0	0	0	0	0	0	0	0	0	0	0	0	0	0	0	0	0
0	0	0	0	0	0	0	0	0	0	0	0	0	0	0	0	0	0	0
0	0	0	0	0	0	0	0	0	0	0	0	0	0	0	0	0	0	0
0	0	0	0	0	0	0	0	0	0	0	0	0	0	0	0	0	0	0
0	0	0	0	0	0	0	0	0	0	0	0	0	0	0	0	0	0	0
0	0	0	0	5	0	0	0	0	0	0	0	0	0	0	0	0	0	0
0	0	0	0	0	0	0	0	0	0	0	0	0	0	0	0	0	0	0
0	0	0	0	0	0	0	0	0	0	0	0	0	0	0	0	0	0	0
0	0	0	0	0	0	0	0	0	0	0	0	0	0	0	0	0	0	0
0	0	0	0	0	0	0	0	0	0	0	0	0	0	0	0	0	0	0
0	0	0	0	0	0	0	0	0	0	0	0	0	0	0	0	0	0	0
0	0	0	0	0	0	0	0	0	0	0	0	0	0	0	0	0	0	0
0	0	0	0	0	0	0	0	0	0	0	0	0	0	0	0	0	0	0
0	0	0	0	0	0	0	0	0	0	0	0	0	0	0	0	0	0	0
0	0	0	0	0	0	0	0	0	0	0	0	0	0	0	0	0	0	0
0	0	0	0	0	0	0	0	0	0	0	0	0	0	0	0	0	0	0
0	0	0	0	0	0	0	0	0	0	0	0	0	0	0	0	0	0	0
0	0	0	0	0	0	0	0	0	0	0	0	0	0	0	0	0	0	0
0	0	0	0	0	0	0	0	0	0	4	4	0	0	0	0	0	0	0

0	0	0	0	0	0	0	0	0	0	0	0	0	0	0	0	0	0	0
0	0	0	0	0	0	0	0	0	0	0	0	0	0	0	0	0	0	0
0	0	0	0	0	0	0	0	0	0	0	0	0	0	0	0	0	0	0
0	0	0	0	0	0	0	0	0	0	0	0	0	0	0	0	0	0	0
0	0	0	0	0	0	0	0	0	0	0	0	0	0	0	0	0	0	0
0	0	0	0	0	0	0	0	0	0	0	0	0	0	0	0	0	0	0
0	0	0	0	0	0	0	0	0	0	0	0	0	0	0	0	0	0	0
0	0	0	0	0	0	0	0	0	0	0	0	0	0	0	0	0	0	0
0	0	0	0	0	0	0	0	0	0	0	0	0	0	0	0	0	0	0
0	0	0	0	0	0	0	0	0	0	0	0	0	0	0	0	0	0	0
0	0	0	0	0	0	0	0	0	0	0	0	0	0	0	0	0	0	0
0	0	0	0	0	0	0	0	0	0	0	0	0	0	0	0	0	0	0
0	0	0	0	0	0	0	0	0	0	0	0	0	0	0	0	0	0	0
0	0	0	0	0	0	0	0	0	0	0	0	0	0	0	0	0	0	0
0	0	0	0	0	0	0	0	0	0	0	0	0	0	0	0	0	0	0
0	0	0	0	0	0	0	0	0	0	0	0	0	0	0	0	0	0	0
0	0	0	0	0	0	0	0	0	0	0	0	0	0	0	0	0	0	3
3	3	3	3	3	3	0	0	0	0	0	0	0	0	0	0	0	0	0
0	0	0	0	0	0	0	0	0	0	0	0	0	0	0	0	0	0	0
0	0	0	0	0	0	0	0	0	0	0	0	0	0	0	0	0	0	0
0	0	0	0	0	0	0	0	0	0	0	0	0	0	0	0	0	0	0
0	0	0	0	0	0	0	0	0	0	0	0	0	0	0	0	0	0	0
0	0	0	0	0	0	0	0	0	0	0	0	0	0	0	0	0	0	0
0	0	0	0	0	0	0	0	0	0	0	0	0	0	0	0	0	0	0
0	0	0	0	0	0	0	0	0	0	0	0	0	0	0	0	0	0	0
0	0	0	0	0	0	0	0	0	0	0	0	0	0	0	0	0	0	0
0	0	0	0	0	0	0	0	0	0	0	0	0	0	0	0	0	0	0
0	0	0	0	0	0	0	0	0	0	0	0	0	0	0	0	0	0	0
0	0	0	0	0	0	0	0	0	0	0	0	0	0	0	0	0	0	0
0	0	0	0	0	0	0	0	0	0	0	0	0	0	0	0	0	0	0
0	0	0	0	0	0	0	0	0	0	0	0	0	0	0	0	0	0	0
0	0	0	0	0	0	0	0	0	0	0	0	0	0	0	0	0	0	0
0	0	0	0	0	0	0	0	0	0	0	0	0	0	0	0	0	0	0
0	0	0	0	0	0	0	0	0	0	0	0	0	0	0	0	0	0	0
0	0	0	0	0	0	0	0	0	0	0	0	0	0	0	0	0	0	0
0	0	0	0	0	0	0	0	0	0	0	0	0	0	0	0	0	0	0
0	0	0	0	0	0	0	0	0	0	0	0	0	0	0	0	0	0	0

0	0	0	0	0	0	0	0	0	0	0	0	0	0	0	0	0	0	0
0	0	0	0	0	0	0	0	0	0	0	0	0	0	0	0	0	0	0
0	0	0	0	0	0	0	0	0	0	0	0	0	0	0	0	0	0	0
0	0	0	0	0	0	0	0	0	0	0	0	0	0	0	0	0	0	0
0	0	0	0	0	0	0	0	0	0	0	0	0	0	0	0	0	0	0
0	0	0	0	0	0	0	0	0	2	2	2	2	2	2	2	0	0	0
0	0	0	0	0	0	0	0	0	0	0	0	0	0	0	0	0	0	0
0	0	0	0	0	0	0	0	0	0	0	0	0	0	0	0	0	0	0
0	0	0	0	0	0	0	0	0	0	0	0	0	0	0	0	0	0	0
0	0	0	0	0	0	0	0	0	0	0	0	0	0	0	0	0	0	0
0	0	0	0	0	0	0	0	0	0	0	0	0	0	0	0	0	0	0
0	0	0	0	0	0	0	0	0	0	0	0	0	0	0	0	0	0	0
0	0	0	0	0	0	0	0	0	0	0	0	0	0	0	0	0	0	0
0	0	0	0	0	0	0	0	0	0	0	0	0	0	0	0	0	0	0
0	0	0	0	0	0	1	1	0	0									
Jan_DL143	1196	1319	666	749	48	109	174	165	199	312	414	307	252	434	110	171	159	241
315	658	74	213	224	51	284	261	248	209	162	0	171	154	126	204	163	331	0
183	79	0	246	145	142	168	0	95	69	159	234	68	122	33	191	109	125	0
0	168	205	32	104	312	111	69	95	63	49	0	0	105	17	148	5	91	19
76	0	28	20	0	55	42	0	39	73	43	113	20	240	27	0	48	0	199
40	40	109	0	0	0	37	43	18	23	0	0	0	0	35	24	0	221	0
0	0	0	25	0	27	0	0	0	82	8	0	0	0	0	89	0	0	0
65	0	0	0	24	0	0	0	59	9	0	0	0	0	0	0	0	0	0
18	0	18	0	77	0	0	0	0	18	7	0	27	44	0	0	0	0	15
0	0	0	65	0	87	0	0	0	18	0	19	0	16	0	31	5	0	0
25	0	0	22	0	0	0	0	0	0	0	0	0	0	0	0	0	0	0
0	0	0	0	0	0	0	0	0	0	0	0	0	93	0	0	0	0	0
0	100	0	29	0	15	0	0	0	0	0	71	10	8	17	0	0	0	0
0	0	0	0	0	0	0	0	0	9	0	31	0	0	0	0	23	9	0
10	0	0	0	0	0	0	0	0	0	0	0	0	0	0	4	0	0	0
0	0	6	0	0	0	0	0	0	0	0	0	7	0	21	0	0	0	0
0	0	12	0	0	0	0	0	0	0	0	0	0	11	0	6	0	0	10
0	0	3	15	0	0	0	8	0	0	0	0	0	0	12	0	14	0	0
4	2	0	13	0	0	0	0	0	6	0	12	0	0	0	0	0	0	0
0	0	0	0	0	0	6	0	0	0	0	0	0	0	0	0	0	0	0
0	0	0	0	0	0	5	0	0	0	0	0	0	0	0	0	0	0	0
0	0	0	11	0	7	9	0	0	0	0	0	0	0	0	19	0	0	0

0	0	0	0	0	0	0	0	0	0	0	0	0	0	10	0	0	0	0
0	4	0	0	0	0	0	0	0	0	0	0	0	0	0	0	0	0	0
0	0	0	0	0	0	0	0	0	0	0	19	0	0	0	0	0	0	0
0	0	0	0	0	0	13	0	0	0	0	0	20	0	0	0	0	0	0
6	0	0	0	0	0	0	0	0	0	0	0	0	0	0	0	0	0	15
0	0	0	0	0	7	0	0	0	0	0	0	0	0	0	0	0	0	0
0	0	0	0	0	0	0	0	0	0	0	23	0	0	0	0	0	0	0
0	0	0	0	0	0	0	0	0	0	4	14	0	0	0	0	0	0	0
0	0	0	0	0	0	0	0	0	0	0	0	0	0	0	0	0	0	0
0	0	0	0	0	0	0	0	0	0	0	11	0	0	0	0	0	0	0
0	0	0	0	0	0	0	0	0	0	0	0	0	0	0	0	0	0	0
0	0	0	0	0	0	0	0	0	0	0	0	0	0	0	0	19	0	0
0	0	0	0	0	0	0	0	0	0	0	0	0	0	0	0	0	0	0
3	0	0	0	0	0	0	0	0	0	0	0	0	0	0	0	0	0	0
0	0	0	0	0	0	0	0	0	0	0	0	0	0	0	0	0	0	0
0	0	0	0	0	5	0	0	0	0	0	0	0	0	0	0	0	0	0
0	0	0	6	0	0	0	0	0	0	0	0	0	0	0	0	0	0	0
0	0	0	0	0	0	0	0	0	0	0	0	0	0	0	0	0	0	0
0	0	0	0	0	0	0	0	0	0	0	0	0	0	0	0	0	0	7
0	0	0	0	0	0	0	0	0	0	0	0	0	0	0	0	0	0	0
0	0	0	0	0	0	0	0	0	0	4	0	0	0	0	0	0	0	0
0	0	0	0	0	0	6	0	0	0	0	0	0	0	0	0	0	0	0
0	0	0	0	0	0	0	0	0	0	0	0	0	0	0	0	0	0	0
0	0	0	0	0	0	0	10	0	0	0	0	13	0	0	0	0	0	0
0	0	0	0	0	0	0	0	0	0	0	0	0	0	9	0	0	0	0
0	0	0	0	0	0	0	0	0	0	0	0	0	0	0	0	0	0	0
0	0	0	0	0	0	0	0	0	0	0	0	15	0	0	0	0	0	0
0	0	0	9	0	0	0	0	0	0	0	0	0	0	0	0	0	0	0
0	0	0	0	0	0	0	0	0	0	0	0	0	0	0	0	0	0	0
0	0	9	0	0	0	0	6	0	0	0	0	0	0	0	0	0	0	0
6	0	0	0	0	0	0	0	0	0	0	0	0	0	0	0	0	0	0
0	0	0	0	0	0	0	0	0	0	0	0	0	0	0	0	0	0	0
0	0	0	0	0	0	0	0	0	0	0	0	0	0	0	0	0	0	0
0	0	0	0	0	0	0	0	0	0	0	0	0	0	0	0	0	0	0
0	0	0	0	0	0	0	0	0	0	0	0	0	0	0	0	0	0	0
0	0	0	0	0	0	0	0	0	0	0	0	0	0	0	0	0	0	0
0	0	0	0	0	0	0	0	0	0	0	0	0	0	0	0	0	0	0

0	0	0	0	0	0	0	0	0	0	0	0	0	0	0	0	0	0	0
0	0	0	0	0	0	0	0	0	0	0	0	0	0	0	0	0	0	0
0	0	0	0	0	0	0	0	3	0	0	0	0	0	0	0	0	0	0
0	0	0	0	0	0	0	0	0	0	0	0	8	0	0	0	0	0	0
0	0	0	0	0	0	0	0	0	0	0	0	0	0	0	0	0	0	0
0	0	0	0	0	0	0	0	0	0	0	0	0	0	0	0	0	0	0
0	0	0	0	0	0	0	0	0	0	0	0	0	0	0	0	0	0	0
0	0	0	0	0	0	0	0	0	0	0	0	0	0	0	0	0	0	0
0	0	0	0	0	0	0	0	0	0	0	0	0	0	0	0	0	0	0
0	0	0	0	0	0	0	0	0	0	0	0	0	0	0	0	0	0	0
0	0	0	0	0	0	0	0	0	0	0	0	0	0	0	0	0	0	0
0	0	0	0	0	0	0	0	0	0	0	0	0	0	0	0	14	0	0
0	0	0	0	0	0	0	0	0	0	0	0	0	0	0	0	0	0	0
0	0	0	0	0	0	0	0	0	0	0	0	0	0	0	0	0	0	0
0	0	0	0	0	0	0	0	0	0	0	0	0	0	0	0	0	0	0
0	0	0	0	0	0	0	0	0	0	0	0	0	0	0	0	0	0	0
0	0	0	0	0	0	0	0	0	0	0	0	0	0	0	0	0	0	0
0	0	0	0	0	0	0	0	0	0	0	0	0	0	0	0	0	0	0
0	0	0	0	0	0	0	0	0	0	0	0	0	0	0	0	0	0	0
0	0	0	0	0	0	0	0	0	0	0	0	0	0	0	0	0	0	0
0	0	0	0	0	0	0	0	0	0	0	0	0	0	0	0	0	0	0
0	0	0	0	0	0	0	0	0	0	0	0	0	0	0	0	0	0	0
0	0	0	0	0	0	0	0	0	0	0	0	0	0	0	0	0	0	0
0	0	0	0	0	0	0	0	0	0	0	0	0	0	0	0	0	0	0
0	0	0	0	0	0	0	0	0	0	0	0	0	0	0	0	0	0	0
0	0	0	9	0	0	0	0	0	0	0	0	0	0	0	0	0	0	0
0	0	0	0	0	0	0	0	0	0	0	0	0	0	0	0	0	0	0
0	0	0	0	0	0	0	0	0	0	0	0	0	0	0	0	0	0	0
0	0	0	0	0	0	0	0	0	0	0	0	0	0	0	0	0	0	0
0	0	0	0	0	0	0	0	0	0	8	0	0	0	0	0	0	0	0
0	0	0	0	0	0	0	0	0	0	0	0	0	0	0	0	0	0	0
0	0	0	0	0	0	0	0	0	0	0	0	0	0	0	0	0	0	0
0	0	0	0	0	0	0	0	0	0	0	0	0	0	0	0	0	0	0
0	0	0	0	0	0	0	0	0	0	0	0	0	0	0	0	0	0	0
0	0	0	0	0	0	0	0	0	0	0	0	0	0	0	0	0	0	0
0	0	0	0	0	0	0	0	0	0	0	0	0	0	0	0	0	0	0
0	0	0	0	0	0	0	0	0	0	0	0	0	0	0	0	0	0	0

0	0	0	0	0	0	0	0	0	0	0	0	0	0	0	0	0	0	0
0	0	0	0	0	0	0	0	0	0	0	0	0	0	0	0	0	0	0
0	0	0	0	0	0	0	0	0	0	0	0	0	0	0	0	0	0	0
0	0	0	0	0	0	0	0	0	0	0	0	0	0	0	0	0	0	0
0	0	0	0	0	0	0	0	0	0	0	0	0	0	0	0	0	0	0
0	0	0	0	0	0	6	6	0	0	0	0	0	0	0	0	0	0	0
0	0	0	0	0	0	0	0	0	0	0	0	0	0	0	0	0	0	0
0	0	0	0	0	0	0	0	0	0	0	0	0	0	0	0	0	0	0
0	0	0	0	0	0	0	0	0	0	0	0	0	0	0	0	0	0	0
0	0	0	0	0	0	0	0	0	0	0	0	0	0	0	0	0	0	0
0	0	0	0	0	0	0	0	0	0	0	0	0	0	0	0	0	0	0
0	0	0	0	0	0	0	0	0	0	0	0	0	0	0	0	0	0	0
0	0	0	0	0	0	0	0	0	0	0	0	0	0	0	0	0	0	0
0	0	0	0	0	0	0	0	5	5	0	0	0	0	0	0	0	0	0
0	0	0	0	0	0	0	0	0	0	0	0	0	0	0	0	0	0	0
0	0	0	0	0	0	0	0	0	0	0	0	0	0	0	0	0	0	0
0	0	0	0	0	0	0	0	0	0	0	0	0	0	0	0	0	0	0
0	0	0	0	0	0	0	0	0	0	0	0	0	0	0	0	0	0	0
0	0	0	0	0	0	0	0	0	0	0	0	0	0	0	0	0	0	0
0	0	0	0	0	0	0	0	0	0	0	0	0	0	0	0	0	0	0
0	0	0	0	0	0	0	0	0	0	0	0	0	0	0	0	0	0	0
0	0	0	0	0	0	0	0	0	0	0	0	0	0	0	0	0	0	0
0	0	0	0	0	0	0	0	0	0	0	0	0	0	0	0	0	0	0
0	0	0	0	0	0	0	0	0	0	0	0	0	0	0	0	0	0	0
4	4	4	0	0	0	0	0	0	0	0	0	0	0	0	0	0	0	0
0	0	0	0	0	0	0	0	0	0	0	0	0	0	0	0	0	0	0
0	0	0	0	0	0	0	0	0	0	0	0	0	0	0	0	0	0	0
0	0	0	0	0	0	0	0	0	0	0	0	0	0	0	0	0	0	0
0	0	0	0	0	0	0	0	0	0	0	0	0	0	0	0	0	0	0
0	0	0	0	0	0	0	0	0	0	0	0	0	0	0	0	0	0	0
0	0	0	0	0	0	0	0	0	0	0	0	0	0	0	0	0	0	0
0	0	0	0	0	0	0	0	0	0	0	0	0	0	0	0	0	0	0
0	0	0	0	0	0	0	0	0	0	0	0	0	0	0	0	0	0	0
0	0	0	0	0	0	0	0	0	0	0	0	0	0	0	0	0	0	0
0	0	0	0	0	0	0	0	0	0	0	0	0	0	0	0	0	0	0
0	0	0	0	0	0	0	0	0	0	0	0	0	0	0	0	0	0	0
0	0	0	0	0	0	0	0	0	0	0	0	0	0	0	0	0	0	0

0	0	0	0	0	0	0	0	0	0	0	0	0	0	0	0	0	0	0
0	0	0	0	0	0	0	0	0	0	0	0	0	0	0	0	0	0	0
0	0	0	0	0	0	0	0	0	0	0	0	0	0	0	0	0	0	0
0	0	0	0	0	0	0	0	0	0	0	0	0	0	0	0	0	0	0
0	0	0	0	0	0	0	0	0	0	0	0	0	0	0	0	0	0	0
0	0	0	0	0	0	0	0	0	0	0	0	3	3	3	3	0	0	0
0	0	0	0	0	0	0	0	0	0	0	0	0	0	0	0	0	0	0
0	0	0	0	0	0	0	0	0	0	0	0	0	0	0	0	0	0	0
0	0	0	0	0	0	0	0	0	0	0	0	0	0	0	0	0	0	0
0	0	0	0	0	0	0	0	0	0	0	0	0	0	0	0	0	0	0
0	0	0	0	0	0	0	0	0	0	0	0	0	0	0	0	0	0	0
0	0	0	0	0	0	0	0	0	0	0	0	0	0	0	0	0	0	0
0	0	0	0	0	0	0	0	0	0	0	0	0	0	0	0	0	0	0
0	0	0	0	0	0	0	0	0	0	0	0	0	0	0	0	0	0	0
0	0	0	0	0	0	0	0	0	0	0	0	0	0	0	0	0	0	0
0	0	0	0	0	0	0	0	0	0	0	0	0	0	0	0	0	0	0
0	0	0	0	0	0	0	0	0	0	0	0	0	0	0	0	0	0	0
0	0	0	0	0	0	0	0	0	0	0	0	0	0	0	0	0	0	0
0	0	0	0	0	0	0	0	0	0	0	0	0	0	0	0	0	0	0
0	0	0	0	0	0	0	0	0	0	0	0	0	0	0	0	0	0	0
0	0	0	0	0	0	0	0	0	0	0	0	0	0	0	0	0	0	0
0	0	0	0	0	0	0	0	0	0	0	0	0	0	0	0	0	0	0
0	0	0	0	0	0	0	0	0	0	0	0	0	0	0	0	0	0	0
0	0	0	0	0	0	0	0	0	0	0	0	0	0	0	0	0	0	0
0	0	0	0	0	0	0	0	0	0	0	0	0	0	0	0	0	0	0
0	0	0	0	0	0	0	0	0	0	0	0	0	0	0	0	0	0	0
0	0	0	0	0	0	0	0	0	0	0	0	0	0	0	0	0	0	0
0	0	0	0	0	0	0	0	0	0	0	0	0	0	0	0	0	0	0
0	0	0	0	0	0	0	0	0	0	0	0	0	0	0	0	0	0	0
0	0	0	0	0	0	0	0	0	0	0	0	0	0	0	0	0	0	0
0	0	0	0	0	0	0	0	0	0	0	0	0	0	0	0	0	0	0
0	0	0	2	2	2	2	2	2	2	2	2	2	2	2	2	0	0	0
0	0	0	0	0	0	0	0	0	0	0	0	0	0	0	0	0	0	0
0	0	0	0	0	0	0	0	0	0	0	0	0	0	0	0	0	0	0
0	0	0	0	0	0	0	0	0	0	0	0	0	0	0	0	0	0	0
0	0	0	0	0	0	0	0	0	0	0	0	0	0	0	0	0	0	0
0	0	0	0	0	0	0	0	0	0	0	0	0	0	0	0	0	0	0

0	0	0	0	0	0	0	0	0	0	0	0	0	0	0	0	0	0	0
0	0	0	0	0	0	0	0	0	0	0	0	0	0	0	0	0	0	0
0	0	0	0	0	0	0	0	0	0	0	0	0	0	0	0	0	0	0
0	0	0	0	0	0	0	0	0	0	0	0	0	0	0	0	0	0	0
0	0	0	0	0	0	0	0	0	0	0	0	0	0	0	0	0	0	0
0	0	0	0	0	0	0	0	0	0	0	0	0	0	0	0	0	0	0
0	0	0	1	0	0	0	0	0	0									
Jan_DL144	894	1143	692	295	1823	91	68	45	100	202	148	255	216	58	283	367	54	105
106	160	128	0	130	91	244	143	199	151	52	0	104	139	132	63	103	40	0
154	320	0	30	85	42	114	381	12	165	33	224	142	0	15	196	68	46	0
0	148	178	245	38	25	0	66	89	75	79	0	90	0	314	16	19	68	53
0	0	0	12	0	28	41	0	92	54	0	0	0	168	46	0	19	0	197
0	22	0	122	49	0	63	0	34	20	0	0	0	56	23	30	0	0	18
0	19	70	7	16	16	0	0	0	0	34	0	11	0	77	71	0	0	61
0	0	0	23	95	69	0	0	0	0	0	0	15	0	0	0	0	0	0
20	0	18	0	0	17	0	0	43	0	5	0	0	15	32	0	0	0	0
32	0	0	0	0	0	0	0	0	0	0	0	0	28	0	0	65	0	6
0	0	0	25	0	10	0	0	0	17	0	0	0	0	0	0	0	0	0
9	0	0	0	23	0	0	0	0	0	0	0	0	35	0	0	0	3	11
0	73	37	0	0	0	0	10	27	22	0	15	0	10	0	22	5	0	0
6	0	0	17	0	0	0	0	0	24	0	34	30	0	7	47	42	0	0
0	0	0	13	0	0	14	0	0	0	0	0	0	12	6	8	0	0	0
0	0	0	0	0	0	0	0	0	0	37	0	0	23	0	7	0	0	0
0	0	0	171	0	3	0	0	0	0	0	0	11	8	0	0	0	0	7
0	0	14	15	33	0	0	0	0	0	0	0	0	0	0	0	0	0	7
0	0	0	53	8	0	0	0	9	0	0	0	0	0	0	0	0	0	0
0	0	0	0	11	0	0	22	0	8	0	0	0	0	0	0	0	0	4
0	62	0	0	8	0	54	0	0	0	12	0	0	0	7	0	0	0	0
0	0	0	0	0	0	0	0	0	0	0	8	0	0	0	0	4	12	0
0	0	0	0	0	14	0	0	0	0	0	3	0	0	0	0	0	0	0
0	5	0	24	0	0	0	14	0	0	0	0	0	0	32	0	0	0	0
17	0	0	0	12	0	0	0	0	0	0	0	0	0	0	0	0	0	0
0	0	0	0	0	0	0	0	0	0	0	17	4	0	0	0	0	0	4
0	0	0	0	0	0	0	8	0	0	0	0	0	0	0	0	0	12	0
0	0	38	0	0	0	0	0	0	0	34	0	0	0	0	0	0	0	0
0	0	0	0	0	0	0	0	0	0	0	0	0	0	0	0	0	0	0
0	0	0	0	0	0	0	0	0	0	0	0	0	0	0	0	0	0	0

0	0	0	2	0	0	0	0	0	0	0	0	0	0	0	0	0	57	0
0	0	0	0	0	0	0	0	0	0	0	0	0	0	0	0	0	0	0
0	0	0	0	0	0	0	0	0	0	0	0	0	0	0	0	0	0	0
0	0	0	0	0	0	0	0	0	0	3	0	0	0	0	0	0	0	0
0	0	0	0	0	18	0	0	0	0	15	0	8	9	0	0	0	0	0
0	0	0	0	0	0	0	0	0	0	0	0	0	0	0	0	0	0	0
0	0	0	0	0	0	0	0	0	0	9	0	0	0	0	0	0	0	0
0	0	15	0	0	0	0	0	0	0	0	0	0	0	0	0	0	0	0
0	0	0	0	0	0	0	0	0	0	0	0	0	0	0	0	0	0	0
0	0	0	0	0	0	0	0	0	0	0	0	0	0	0	0	0	0	0
0	0	0	0	0	0	0	0	0	0	0	0	0	0	0	0	0	0	0
0	0	0	0	33	0	0	0	0	0	0	0	0	0	0	0	0	0	0
0	0	0	0	0	0	0	0	0	6	0	0	0	0	0	0	0	0	0
0	0	0	0	0	0	0	29	0	0	0	0	0	0	0	0	0	0	0
0	0	0	0	0	0	0	0	0	0	0	0	0	0	0	0	0	0	0
0	0	0	0	0	0	0	0	0	0	0	0	0	0	0	0	0	0	0
0	0	0	0	0	0	0	0	0	0	0	0	0	0	0	0	0	0	0
0	0	0	0	0	0	0	0	0	0	0	0	0	0	0	0	0	0	0
0	0	0	0	0	10	0	0	0	0	9	0	0	0	0	0	0	0	0
0	0	0	0	0	0	0	0	0	0	0	0	0	0	0	0	0	0	0
0	0	0	0	0	0	0	0	0	0	0	0	0	0	0	0	0	0	0
0	0	0	0	0	0	0	0	0	0	0	0	0	0	0	0	0	0	0
0	0	0	0	0	0	0	0	0	0	0	0	0	0	0	0	0	0	0
0	0	15	0	0	0	0	0	0	0	0	0	0	0	0	0	0	0	0
0	0	0	0	0	0	0	0	0	0	0	0	0	0	0	0	0	0	0
0	0	0	0	0	0	0	0	0	0	0	0	0	0	0	0	0	0	0
0	0	0	0	0	0	0	0	0	0	0	0	0	0	0	0	0	0	0
0	0	0	0	0	0	0	0	0	0	0	0	0	0	0	0	0	0	0
0	0	0	0	0	0	0	0	0	0	0	0	0	0	0	0	0	0	0
0	0	0	6	0	0	0	0	0	0	0	0	0	0	0	0	0	0	0
0	0	0	0	0	0	0	0	0	0	0	0	0	0	0	0	0	0	0
0	0	0	0	0	0	0	0	0	0	0	14	8	0	0	0	0	0	0
0	0	0	0	0	0	0	0	0	8	0	0	0	0	0	0	0	0	0
0	0	0	0	0	0	0	0	0	0	0	0	0	0	0	0	0	0	0
0	0	0	0	0	0	0	0	0	0	0	0	0	0	0	0	0	0	0
0	0	0	0	0	0	0	0	0	0	0	0	0	0	0	0	0	0	0
0	0	0	0	0	0	0	0	0	0	0	0	0	0	0	0	0	0	0

0	6	0	0	0	0	0	0	0	0	0	0	0	0	0	0	0	0	0
0	0	0	0	0	0	0	0	0	0	0	0	0	0	0	0	0	0	0
0	0	0	0	0	0	0	0	0	0	0	0	0	0	0	0	0	0	0
0	0	0	0	0	0	0	0	0	0	0	0	0	0	0	0	0	0	0
0	0	0	0	0	0	0	0	0	0	0	0	0	0	0	0	0	0	0
0	0	0	0	0	0	0	0	0	0	0	0	0	0	0	0	0	0	0
0	0	0	0	0	0	0	0	0	0	0	0	0	0	0	0	0	0	0
0	0	0	0	0	0	0	0	0	0	0	0	0	0	0	0	0	0	0
0	0	0	0	0	0	0	0	0	0	0	0	0	0	0	0	0	5	0
0	0	0	0	0	0	0	0	0	0	0	0	0	0	0	0	0	0	0
0	0	0	0	0	0	0	0	0	0	0	0	0	0	0	0	0	0	0
0	0	0	0	0	0	0	0	0	0	0	0	0	0	0	0	0	0	0
0	0	0	0	0	0	0	0	0	0	0	0	0	0	0	0	0	0	0
0	0	0	0	0	0	0	0	0	0	0	0	0	0	0	0	0	0	0
0	0	0	0	0	0	0	0	0	0	0	0	0	0	0	0	0	0	0
0	0	0	0	0	0	0	0	0	0	0	0	0	0	0	0	0	0	0
0	0	0	0	0	0	0	0	0	0	0	0	0	0	0	0	0	0	0
0	0	0	0	0	0	0	0	0	0	0	0	0	0	0	0	0	0	0
0	0	0	0	0	0	0	0	0	0	0	0	0	0	0	0	0	0	0
0	0	0	0	0	0	0	0	0	0	0	0	0	0	0	0	0	0	0
0	0	0	0	0	0	0	0	0	0	0	0	0	0	0	0	0	0	0
0	0	0	0	0	0	0	0	0	0	0	0	0	0	0	0	0	0	0
0	0	0	0	0	0	0	0	0	0	0	0	0	0	0	0	0	0	0
0	0	0	0	0	0	0	0	0	0	0	0	0	0	0	0	0	0	0
0	0	0	0	0	0	0	0	0	0	0	0	0	0	0	0	0	0	0
0	0	0	0	0	0	0	0	0	0	0	0	0	0	0	0	0	0	0
0	0	0	0	0	0	0	0	0	0	0	0	0	0	0	0	0	0	0
0	0	0	0	0	0	0	0	0	0	0	0	0	0	0	0	0	0	0
0	0	0	0	0	0	0	0	0	0	0	0	0	0	0	0	0	0	0
0	0	0	0	0	0	0	0	0	0	0	0	0	0	0	0	0	0	0
0	0	0	0	0	0	0	0	0	0	0	0	0	0	0	0	0	0	0
0	0	0	0	0	0	0	0	0	0	0	0	0	0	0	0	0	0	0
0	0	0	0	0	0	0	0	0	0	0	0	0	0	0	0	0	0	0
0	0	0	0	0	0	0	0	0	0	0	0	0	0	0	0	0	0	0
0	0	0	0	0	0	0	0	0	0	0	0	0	0	0	0	0	0	0
0	0	0	0	0	0	0	0	0	0	0	0	0	0	0	0	0	0	0
0	0	0	0	0	0	0	0	0	0	0	0	0	0	0	0	0	0	0

0	0	0	0	0	5	0	0	0	0	0	0	0	0	0	0	0	0	0
0	0	0	0	0	0	0	0	0	0	0	0	0	0	0	0	0	0	0
0	0	0	0	0	0	0	0	0	0	0	0	0	0	0	0	0	0	0
0	0	0	0	0	0	0	0	0	0	0	0	0	0	0	0	0	0	0
0	0	0	0	0	0	0	0	0	0	0	0	0	0	0	0	0	0	0
0	0	0	0	0	0	0	0	0	0	0	0	0	0	0	0	0	0	0
0	0	0	0	0	0	0	0	0	0	0	0	0	0	0	0	0	0	0
0	0	0	0	0	0	0	0	0	0	0	0	0	0	0	0	0	0	0
0	0	0	0	0	0	0	0	0	0	0	0	0	0	0	0	0	0	0
0	0	0	0	0	0	0	0	0	0	0	0	0	0	0	0	0	0	0
0	0	0	0	0	0	0	0	0	0	0	0	0	0	0	0	0	0	0
0	0	0	0	0	0	0	0	0	0	0	0	0	0	0	0	0	0	0
0	0	0	0	0	0	0	0	0	0	0	0	0	0	0	0	0	0	0
0	0	0	0	0	0	0	0	0	0	0	0	0	0	0	0	0	0	0
0	0	0	0	0	0	0	0	0	0	0	0	0	0	0	0	0	0	0
0	0	0	0	0	0	0	0	0	0	0	0	0	0	0	0	0	0	0
0	0	0	0	0	0	0	0	0	0	0	0	0	0	0	0	0	0	0
0	0	0	0	0	0	0	0	0	0	0	0	0	0	0	0	0	0	0
0	0	0	0	0	0	0	0	0	0	0	0	0	0	0	0	0	0	0
0	0	0	0	0	0	0	0	0	0	0	0	0	0	0	0	0	0	0
0	0	0	0	0	0	0	0	0	0	0	0	0	0	0	0	0	0	0
0	0	0	0	0	0	0	0	0	0	0	0	0	0	0	0	0	0	0
0	0	0	0	0	0	0	0	0	0	0	0	0	0	0	0	0	0	0
0	0	0	0	0	0	0	0	0	0	0	0	0	0	0	0	0	0	0
0	0	0	0	0	0	0	0	0	0	0	0	0	0	0	0	0	0	0
0	0	0	0	0	0	0	0	0	0	0	0	0	0	0	0	0	0	0
0	0	0	0	0	0	0	0	0	0	0	0	0	0	0	0	0	0	0
0	0	0	0	0	3	3	3	3	3	0	0	0	0	0	0	0	0	0
0	0	0	0	0	0	0	0	0	0	0	0	0	0	0	0	0	0	0
0	0	0	0	0	0	0	0	0	0	0	0	0	0	0	0	0	0	0
0	0	0	0	0	0	0	0	0	0	0	0	0	0	0	0	0	0	0
0	0	0	0	0	0	0	0	0	0	0	0	0	0	0	0	0	0	0
0	0	0	0	0	0	0	0	0	0	0	0	0	0	0	0	0	0	0
0	0	0	0	0	0	0	0	0	0	0	0	0	0	0	0	0	0	0
0	0	0	0	0	0	0	0	0	0	0	0	0	0	0	0	0	0	0
0	0	0	0	0	0	0	0	0	0	0	0	0	0	0	0	0	0	0
0	0	0	0	0	0	0	0	0	0	0	0	0	0	0	0	0	0	0

0	0	0	0	0	0	0	0	0	0	0	0	0	0	0	0	0	0	0
0	0	0	0	0	0	0	0	0	0	0	0	0	0	0	0	0	0	0
0	0	0	0	0	0	0	0	0	0	0	0	0	0	0	0	0	0	0
0	0	0	0	0	0	0	0	0	0	0	0	0	0	0	0	0	0	0
0	0	0	0	0	0	0	0	0	0	0	0	0	0	0	0	0	0	0
0	0	0	0	0	0	0	0	0	0	0	0	0	0	0	0	0	0	0
0	0	0	0	0	0	0	0	0	0	0	0	0	0	0	0	0	0	0
0	0	0	0	0	0	0	0	0	0	0	0	0	0	0	0	0	0	0
0	0	0	0	0	0	0	0	0	0	0	0	0	0	0	0	0	0	0
0	0	0	0	0	0	0	0	0	0	0	0	0	0	0	0	0	0	0
0	0	0	0	0	0	0	0	0	0	0	0	0	0	0	0	0	0	0
0	0	0	0	0	0	0	0	0	0	0	0	0	0	0	0	0	0	0
0	0	0	0	0	0	0	0	0	0	0	0	0	0	0	0	0	0	0
0	0	0	0	0	0	0	0	0	0	0	0	0	0	0	0	0	0	0
0	0	0	0	0	0	0	0	0	0	0	0	0	0	0	0	0	0	0
0	0	0	0	0	0	0	0	0	0	0	0	0	0	0	0	0	0	0
0	0	0	0	0	0	0	0	0	0	0	0	0	0	0	2	2	2	2
2	2	2	2	2	2	0	0	0	0	0	0	0	0	0	0	0	0	0
0	0	0	0	0	0	0	0	0	0	0	0	0	0	0	0	0	0	0
0	0	0	0	0	0	0	0	0	0	0	0	0	0	0	0	0	0	0
0	0	0	0	0	0	0	0	0	0	0	0	0	0	0	0	0	0	0
0	0	0	0	0	0	0	0	0	0	0	0	0	0	0	0	0	0	0
0	0	0	0	0	0	0	0	0	0	0	0	0	0	0	0	0	0	0
0	0	0	0	0	0	0	0	0	0	0	0	0	0	0	0	0	0	0
0	0	0	0	0	0	0	0	0	0	0	0	0	0	0	0	0	0	0
0	0	0	0	0	0	0	0	0	0	0	0	0	0	0	0	0	0	0
0	0	0	0	0	0	0	0	0	0	0	0	0	0	0	0	0	0	0
0	0	0	0	0	0	0	0	0	0	0	0	0	0	0	0	0	0	0
0	0	0	0	0	0	0	0	0	0	0	0	0	0	0	0	0	0	0
0	0	0	0	0	0	0	0	0	0	0	0	0	0	0	0	0	0	0
0	0	0	0	0	0	0	0	0	0	0	0	0	0	0	0	0	0	0
0	0	0	0	0	0	0	0	0	0	0	0	0	0	0	0	0	0	0
0	0	0	0	0	0	0	0	0	0	0	0	0	0	0	0	0	0	0
0	0	0	0	0	0	0	0	0	0	0	0	0	0	0	0	0	0	0
0	0	0	0	0	0	0	0	0	0	0	0	0	0	0	0	0	0	0
1	1	0	0	0	0	0	0	0	0									

Jan_DL145	1351	1850	188	615	317	362	216	227	259	113	101	92	111	339	223	8	204	149
153	672	248	481	164	273	102	77	72	81	112	0	93	48	34	0	45	31	0
0	170	84	141	84	0	72	0	61	0	101	25	78	73	51	22	0	69	0
0	0	0	34	69	77	81	69	24	198	84	0	0	74	363	355	6	24	228
0	0	38	0	9	27	18	28	69	0	44	0	0	84	24	0	64	0	25
66	20	57	0	19	13	12	0	30	49	0	25	0	0	17	15	0	0	10
0	21	0	16	0	0	0	0	0	52	11	16	0	0	127	17	0	0	24
54	41	0	0	26	0	8	0	0	9	0	0	23	0	0	25	0	0	0
0	0	0	0	0	41	0	0	0	25	0	0	23	0	0	0	0	0	15
0	0	8	101	0	135	0	0	0	16	25	4	0	20	4	15	20	0	0
0	0	0	0	0	16	0	0	0	23	0	0	17	0	0	0	0	0	0
18	0	0	0	0	0	0	0	0	33	0	0	0	37	0	0	0	2	0
0	0	0	0	0	6	0	0	13	0	0	0	0	0	7	0	0	0	0
0	0	0	5	9	0	0	0	0	0	0	0	28	0	14	0	0	16	0
0	0	0	0	0	7	0	0	0	0	4	0	0	0	0	0	6	0	0
148	0	0	0	0	0	0	0	4	0	0	0	0	0	9	6	0	0	0
0	0	10	0	0	0	0	11	0	0	11	48	0	0	12	0	52	0	0
0	0	0	0	8	0	0	0	0	0	0	0	0	0	0	0	0	13	0
0	0	0	0	0	0	0	0	0	0	5	0	0	0	0	0	5	0	0
0	0	0	0	0	0	0	8	0	0	0	0	0	0	0	0	0	0	0
0	0	0	0	0	0	14	0	0	0	0	0	0	0	6	0	0	9	0
0	0	0	0	0	0	0	14	0	0	0	0	0	0	13	20	0	0	0
0	0	0	0	0	0	0	0	0	0	0	0	0	0	0	0	0	0	0
0	0	0	0	0	0	0	0	0	48	21	0	0	0	0	0	0	0	11
0	9	0	0	0	0	0	0	0	0	0	0	0	0	0	0	0	0	0
0	0	0	0	0	0	41	0	0	0	0	0	0	0	0	0	0	0	0
0	0	0	0	0	0	0	0	0	7	0	0	0	0	0	0	0	0	0
0	0	0	0	0	0	0	0	0	0	0	0	0	0	0	3	0	31	0
0	0	0	0	0	0	0	0	0	0	0	0	0	0	0	0	0	0	0
0	0	0	0	0	0	0	0	0	0	0	0	0	0	0	0	0	0	0
0	13	0	0	0	0	0	0	0	0	0	0	0	0	0	0	0	0	0
0	0	0	0	0	0	0	0	0	0	0	0	23	0	0	0	0	0	0
0	0	0	8	0	0	13	0	0	0	0	0	0	0	0	0	0	0	0
0	0	0	0	0	0	0	0	0	0	0	0	0	0	0	0	0	0	0
0	0	0	0	0	0	0	0	0	0	4	7	0	0	0	0	0	0	0
5	0	0	0	0	0	0	0	0	0	5	0	0	0	0	0	0	0	0
0	0	0	0	7	0	0	0	0	0	0	0	0	0	0	8	0	0	0

0	0	0	0	0	0	0	0	0	0	0	0	0	0	0	0	0	0	0
0	0	0	0	0	0	0	0	0	0	0	0	0	0	0	0	0	0	0
0	5	0	0	0	0	0	0	0	0	0	0	0	0	0	0	0	0	0
0	0	0	0	0	0	0	8	0	0	0	0	0	0	12	0	0	0	0
0	0	0	0	0	0	0	0	0	6	0	0	0	0	0	0	0	0	0
0	0	0	0	0	0	0	0	0	0	0	0	0	0	4	0	0	13	0
0	0	0	0	0	0	0	0	0	0	0	0	0	0	0	0	0	0	0
0	0	0	0	0	0	0	0	0	0	0	13	0	0	0	0	0	0	0
0	0	0	0	0	0	0	0	6	0	0	0	0	0	0	0	0	0	0
0	0	0	0	0	0	0	0	0	0	0	0	0	0	0	0	0	7	0
24	0	0	0	0	0	0	0	0	0	0	0	0	0	0	0	0	0	0
0	0	0	0	0	0	0	0	0	0	0	0	0	0	0	0	0	0	0
0	0	0	0	0	0	0	0	0	0	0	0	0	0	0	0	0	0	0
0	0	0	0	0	0	0	0	0	0	0	0	0	0	0	0	0	0	0
0	11	0	0	0	0	0	0	0	0	0	0	0	0	0	0	0	0	0
0	0	0	0	0	0	0	0	0	0	0	0	0	0	0	0	0	0	0
0	0	0	0	0	0	0	0	0	0	0	0	0	0	0	0	0	0	0
0	0	0	0	0	0	0	0	0	0	0	0	0	0	0	0	0	0	0
0	0	0	0	0	0	0	0	0	0	0	0	0	0	0	0	0	0	0
0	0	0	0	0	0	0	0	0	0	0	0	0	0	0	0	0	0	0
0	0	0	0	0	0	0	0	0	0	0	0	0	0	0	0	0	0	0
0	0	0	0	0	0	0	0	0	0	0	0	0	0	0	0	0	0	0
0	0	0	0	0	0	0	0	0	0	0	0	0	0	0	0	0	0	0
0	0	0	0	0	0	0	0	0	0	0	0	0	0	0	0	0	0	0
0	0	0	0	0	0	0	0	0	0	0	0	0	0	0	0	0	0	0
0	0	0	0	0	0	0	0	0	0	0	0	0	0	0	0	0	0	0
0	0	0	0	0	0	0	0	0	0	0	0	0	0	0	0	0	0	0
0	0	0	0	0	0	0	0	0	0	0	0	0	0	0	0	0	0	0
0	0	0	0	0	0	0	0	0	0	0	0	0	0	0	0	0	0	0
0	0	0	0	0	0	0	0	0	0	0	0	0	0	0	5	0	0	0
0	0	0	0	0	0	0	0	0	0	0	0	0	0	0	0	0	0	0
0	0	0	0	0	0	0	0	0	0	0	0	0	0	0	0	0	0	0
0	0	0	0	0	0	0	0	0	0	0	0	0	0	0	0	0	0	0
0	0	0	0	0	0	0	0	0	0	0	0	0	0	0	0	0	0	0
0	0	0	0	0	0	0	0	0	0	0	0	0	0	0	0	0	0	0
0	0	0	0	0	0	0	0	0	0	0	0	0	0	0	0	0	0	0
0	0	0	0	0	0	0	0	0	0	0	0	0	0	0	0	0	0	0

0	0	0	0	0	0	0	0	0	0	0	0	0	0	0	0	0	0	0
0	0	0	0	0	0	0	0	0	0	0	0	0	0	0	0	0	0	0
0	0	0	0	0	0	0	0	0	0	0	0	0	0	0	0	0	0	0
0	0	0	0	0	0	0	0	0	0	0	0	0	0	0	0	0	0	0
0	0	0	0	0	0	0	0	0	0	0	0	0	0	0	0	0	0	0
0	0	0	0	0	0	0	0	0	0	0	0	0	0	0	0	0	0	0
4	0	0	0	0	0	0	0	0	0	0	0	0	0	0	0	0	0	0
8	0	0	0	0	0	0	0	0	0	0	0	0	0	0	0	0	0	0
0	0	0	0	0	0	0	0	0	0	0	0	0	0	0	0	0	0	0
0	0	0	0	0	0	0	0	0	0	0	0	0	0	0	0	0	0	0
0	0	0	0	0	0	0	0	0	0	0	0	0	0	0	0	0	0	0
0	0	0	0	0	0	0	0	0	0	0	0	0	0	0	0	0	0	0
0	7	0	0	0	0	0	0	0	0	0	0	0	0	0	0	0	0	0
0	0	0	0	0	0	0	0	0	0	0	0	0	0	0	0	0	0	0
0	0	0	0	0	0	0	0	0	0	0	0	0	0	0	0	0	0	0
0	0	0	0	0	0	0	0	0	0	0	0	0	0	0	0	0	0	0
0	0	0	0	0	0	0	0	0	0	0	0	0	0	0	0	0	0	0
0	0	0	0	0	0	0	0	0	0	0	0	0	0	0	0	0	0	0
0	0	0	0	0	6	0	0	0	0	0	0	0	0	0	0	0	0	2
0	0	0	0	0	0	0	0	0	0	0	0	0	0	0	0	0	0	0
0	0	0	0	0	0	0	0	0	0	0	0	0	0	0	0	0	0	0
0	0	0	0	0	0	0	0	0	0	0	0	0	0	0	0	0	0	0
0	0	0	0	0	0	0	0	0	0	0	0	0	0	0	0	0	0	0
0	0	0	0	0	0	0	0	0	0	0	0	0	0	0	0	0	0	0
0	0	0	0	0	0	0	0	0	0	0	0	0	0	0	0	0	0	0
0	0	0	0	0	0	0	0	0	0	0	0	0	0	0	0	0	0	0
0	0	0	0	0	0	5	0	0	0	0	0	0	0	0	0	0	0	0
0	0	0	0	0	0	0	0	0	0	0	0	0	0	0	0	0	0	0
0	0	0	0	0	0	0	0	0	0	0	0	0	0	0	0	0	0	0
0	0	0	0	0	0	0	0	0	0	0	0	0	0	0	0	0	0	0
0	0	0	0	0	0	0	0	0	0	0	0	0	0	0	0	0	0	0
0	0	0	0	0	0	0	0	0	0	0	0	0	0	0	0	0	0	0
0	0	0	0	0	0	0	0	0	0	0	0	0	0	0	0	0	0	0
0	0	0	0	0	0	0	0	0	0	0	0	0	0	0	0	0	0	0
0	0	0	0	0	0	0	0	0	0	0	0	0	0	0	0	0	0	0
0	0	0	0	0	0	0	0	0	0	0	0	0	0	0	0	0	0	0
0	0	0	0	0	0	0	0	4	4	0	0	0	0	0	0	0	0	0

0	0	0	0	0	0	0	0	0	0	0	0	0	0	0	0	0	0	0
0	0	0	0	0	0	0	0	0	0	0	0	0	0	0	0	0	0	0
0	0	0	0	0	0	0	0	0	0	0	0	0	0	0	0	0	0	0
0	0	0	0	0	0	0	0	0	0	0	0	0	0	0	0	0	0	0
0	0	0	0	0	0	0	0	0	0	0	0	0	0	0	0	0	0	0
0	0	0	0	0	0	0	0	0	0	0	0	0	0	0	0	0	0	0
0	0	0	0	0	0	0	0	0	0	0	0	0	0	0	0	0	0	0
0	0	0	0	0	0	0	0	0	0	0	0	0	0	0	0	0	0	0
0	0	0	0	0	0	0	0	0	0	0	0	0	0	0	0	0	0	0
0	0	0	0	0	0	0	0	0	0	0	0	0	0	0	0	0	0	0
0	0	0	0	0	0	0	0	0	0	0	0	0	0	0	0	0	0	0
0	0	0	0	0	0	0	0	0	0	0	0	0	0	0	0	0	0	0
0	0	0	0	0	0	0	0	0	0	0	0	0	0	0	0	0	0	0
0	0	0	0	0	0	0	0	0	0	0	0	0	0	0	0	0	0	0
0	0	0	0	0	0	0	0	0	0	0	0	0	0	0	0	0	0	0
0	0	0	0	0	0	0	0	0	0	0	0	3	3	3	3	0	0	0
0	0	0	0	0	0	0	0	0	0	0	0	0	0	0	0	0	0	0
0	0	0	0	0	0	0	0	0	0	0	0	0	0	0	0	0	0	0
0	0	0	0	0	0	0	0	0	0	0	0	0	0	0	0	0	0	0
0	0	0	0	0	0	0	0	0	0	0	0	0	0	0	0	0	0	0
0	0	0	0	0	0	0	0	0	0	0	0	0	0	0	0	0	0	0
0	0	0	0	0	0	0	0	0	0	0	0	0	0	0	0	0	0	0
0	0	0	0	0	0	0	0	0	0	0	0	0	0	0	0	0	0	0
0	0	0	0	0	0	0	0	0	0	0	0	0	0	0	0	0	0	0
0	0	0	0	0	0	0	0	0	0	0	0	0	0	0	0	0	0	0
0	0	0	0	0	0	0	0	0	0	0	0	0	0	0	0	0	0	0
0	0	0	0	0	0	0	0	0	0	0	0	0	0	0	0	0	0	0
0	0	0	0	0	0	0	0	0	0	0	0	0	0	0	0	0	0	0
0	0	0	0	0	0	0	0	0	0	0	0	0	0	0	0	0	0	0
0	0	0	0	0	0	0	0	0	0	0	0	0	0	0	0	0	0	0
0	0	0	0	0	0	0	0	0	0	0	0	0	0	0	0	0	0	0
0	0	0	0	0	0	0	0	0	0	0	0	0	0	0	0	0	0	0
0	0	0	0	0	0	0	0	0	0	0	0	0	0	0	0	0	0	0
0	0	0	0	0	0	0	0	0	0	0	0	0	0	0	0	0	0	0
0	0	0	0	0	0	0	0	0	0	0	0	0	0	0	0	0	0	0
0	0	0	0	0	0	0	0	0	0	0	0	0	0	0	0	0	0	0
0	0	0	0	0	0	0	0	0	0	0	0	0	0	0	0	0	0	0

0	0	0	0	0	0	0	0	0	0	0	0	0	0	0	0	0	0	0
0	0	0	0	0	0	0	0	0	0	0	0	0	0	0	0	0	0	0
0	0	0	0	0	0	0	0	0	0	0	0	0	0	0	0	0	0	0
0	0	0	0	0	0	0	0	0	0	0	0	0	0	0	0	0	0	0
0	0	0	0	0	0	0	0	0	0	0	0	0	0	0	0	0	0	0
0	2	2	2	2	2	2	2	2	0	0	0	0	0	0	0	0	0	0
0	0	0	0	0	0	0	0	0	0	0	0	0	0	0	0	0	0	0
0	0	0	0	0	0	0	0	0	0	0	0	0	0	0	0	0	0	0
0	0	0	0	0	0	0	0	0	0	0	0	0	0	0	0	0	0	0
0	0	0	0	0	0	0	0	0	0	0	0	0	0	0	0	0	0	0
0	0	0	0	0	0	0	0	0	0	0	0	0	0	0	0	0	0	0
0	0	0	0	0	0	0	0	0	0	0	0	0	0	0	0	0	0	0
0	0	0	0	0	0	0	0	0	0	0	0	0	0	0	0	0	0	0
0	0	0	0	0	0	0	0	0	0	0	0	0	0	0	0	0	0	0
0	0	0	0	0	0	0	0	0	0	0	0	0	0	0	0	0	0	0
0	0	0	0	0	0	0	0	0	0	0	0	0	0	0	0	0	0	0
0	0	0	0	0	0	0	0	0	0	0	0	0	0	0	0	0	0	0
0	0	0	0	0	0	0	0	0	0	0	0	0	0	0	0	0	0	0
0	0	0	0	0	0	0	0	0	0	0	0	0	0	0	0	0	0	0
0	0	0	0	0	0	0	0	0	0	0	0	0	0	0	0	0	0	0
0	0	0	0	0	0	0	0	0	0	0	0	0	0	0	0	0	0	0
0	0	0	0	0	0	0	0	0	0	0	0	0	0	0	0	0	0	0
0	0	0	0	0	0	0	0	0	0	0	0	0	0	0	0	0	0	0
0	0	0	0	0	0	0	0	0	0	0	0	0	0	0	0	0	0	0
0	0	0	0	0	0	0	0	0	0	0	0	0	0	0	0	0	0	0
0	0	0	0	0	0	0	0	0	0	0	0	0	0	0	0	0	0	0
0	0	0	0	0	0	0	0	0	0	0	0	0	0	0	0	0	0	0
0	0	0	0	0	0	0	0	0	0	0	0	0	0	0	0	0	0	0
0	0	0	0	0	0	0	0	0	0									
Jan_DL146	1318	914	425	614	21	39	123	127	637	231	433	170	155	317	131	131	131	239
140	67	105	283	173	69	225	147	229	76	225	13	202	69	80	205	116	0	0
66	33	0	102	78	164	60	0	108	56	60	201	31	59	263	250	242	54	0
0	135	210	19	62	0	67	29	68	0	0	0	0	69	34	0	5	47	59
104	0	131	42	0	80	42	122	0	75	59	141	0	23	0	93	27	0	238
0	12	38	0	0	0	100	10	33	34	0	0	0	0	32	24	8	34	0
0	13	0	0	19	0	0	0	0	0	0	0	0	0	0	0	0	0	0
0	0	0	0	0	0	0	0	0	9	0	0	0	0	0	0	0	0	0

252	0	0	0	0	0	38	14	0	0	0	0	0	50	0	6	0	0	12
19	48	18	24	0	19	0	0	0	10	0	26	10	14	0	0	14	0	0
0	0	0	49	25	21	41	0	35	0	0	0	0	0	21	0	0	0	0
0	0	0	0	0	15	0	0	0	0	0	0	0	0	0	0	0	0	0
0	104	0	0	0	0	0	0	7	0	0	0	0	8	5	7	0	0	0
0	0	0	0	0	0	0	0	0	0	0	0	0	0	13	4	0	0	0
0	0	66	0	0	0	0	0	0	5	0	0	0	0	8	0	0	0	0
0	0	0	0	0	0	0	21	0	0	0	0	0	0	12	0	0	0	0
0	0	26	0	0	0	0	0	0	0	0	0	0	0	0	0	0	0	3
0	0	7	0	0	0	0	14	0	0	0	0	0	0	0	0	0	0	16
0	3	0	0	5	0	0	0	0	0	0	0	0	0	0	0	11	0	0
0	0	3	0	0	0	5	8	0	0	0	0	0	0	0	0	0	0	0
0	0	0	0	0	0	0	0	0	0	0	0	0	0	0	0	0	0	0
0	0	0	0	0	0	10	0	0	0	0	0	0	0	0	9	0	0	0
0	0	0	0	0	0	0	0	0	0	0	0	0	0	0	0	0	0	0
0	0	0	0	0	0	0	0	66	0	0	0	0	0	0	0	0	0	0
0	0	0	0	0	0	0	0	0	0	0	0	0	0	0	0	0	0	0
0	0	0	5	0	0	0	0	0	0	0	0	0	19	0	0	0	0	0
0	0	0	0	0	0	0	0	0	0	0	0	0	0	0	0	0	0	0
0	0	0	0	0	0	6	0	0	0	0	0	0	0	0	0	0	0	0
0	0	0	0	0	0	0	0	0	0	0	0	0	0	0	0	0	0	0
0	0	0	0	0	0	0	0	0	0	0	0	0	0	0	0	0	0	0
0	0	10	0	0	0	0	0	0	0	0	0	0	0	0	0	0	0	0
0	0	0	0	0	0	13	0	0	0	0	0	0	0	0	0	0	0	0
0	0	0	0	0	0	0	0	0	0	0	0	0	0	0	0	0	0	0
0	0	0	0	0	0	5	0	0	0	0	0	0	0	0	3	7	0	0
0	0	0	16	0	0	0	0	0	0	0	0	0	0	0	0	6	0	0
0	0	0	0	0	0	0	0	0	0	0	0	0	0	0	0	0	0	0
0	0	0	0	0	0	0	0	0	0	0	0	0	0	20	0	0	15	0
0	0	0	0	0	0	0	0	0	0	0	0	0	0	0	0	0	0	0
0	0	0	0	0	0	0	0	0	0	0	0	0	0	0	0	0	0	0
0	0	0	0	0	0	0	0	0	0	0	0	0	0	0	0	0	0	0
0	0	0	0	0	0	0	0	0	0	0	0	0	0	0	0	0	0	0
0	0	0	0	0	0	0	0	0	0	0	0	0	0	5	0	9	0	0
0	0	0	0	0	0	0	0	0	0	7	0	0	0	0	0	0	0	0
0	0	0	0	0	0	0	0	0	0	0	0	0	0	0	0	0	0	0
0	0	0	0	13	0	0	0	0	0	0	0	5	0	0	0	0	0	0

0	0	0	0	0	0	0	0	0	0	0	0	0	0	0	0	0	0	0
0	0	0	0	0	0	0	0	0	0	0	0	0	0	0	0	0	0	0
0	0	0	0	0	0	0	0	0	0	0	0	0	0	0	0	0	0	0
0	0	0	0	0	0	0	0	0	0	0	0	0	0	0	0	0	0	0
0	0	0	0	0	0	0	0	0	0	0	0	0	0	0	0	0	0	0
0	0	0	0	0	0	0	0	0	0	0	0	0	0	0	0	0	0	0
0	21	0	0	0	0	0	0	0	0	0	0	0	0	0	0	0	0	0
0	0	0	0	0	0	0	0	0	0	0	0	0	0	0	0	0	0	0
0	0	0	0	0	0	0	0	0	0	0	0	0	0	0	0	0	0	0
0	0	0	0	0	0	0	0	0	0	0	0	0	0	0	0	0	0	0
0	0	0	0	0	0	0	0	0	0	0	0	0	0	0	0	0	0	0
0	0	0	0	0	0	0	0	0	0	0	0	0	0	0	0	0	0	0
0	0	0	0	0	0	0	0	0	0	0	0	0	0	0	0	0	0	0
0	0	11	0	0	0	0	0	0	0	0	0	0	0	0	0	0	0	0
0	0	0	0	0	0	0	0	0	0	0	0	0	0	0	0	0	0	0
0	0	0	0	0	0	0	0	0	0	0	0	0	0	0	0	0	0	0
0	0	0	0	0	0	0	0	0	0	0	0	0	0	0	0	0	0	0
0	0	0	0	0	0	0	0	0	0	0	0	0	0	0	0	0	0	0
0	0	0	0	0	0	0	0	0	0	0	0	0	0	0	0	0	0	0
0	0	0	0	0	0	0	0	0	0	0	0	0	0	0	0	0	0	0
0	0	0	0	0	0	0	0	0	0	0	0	0	0	0	0	0	0	0
0	0	0	0	0	0	0	0	0	0	0	0	0	0	0	0	0	0	0
0	0	0	0	0	0	0	0	0	0	0	0	0	0	0	0	0	0	0
0	0	0	0	0	0	0	0	0	0	0	0	0	0	0	0	0	0	0
0	0	0	0	0	0	0	0	0	0	0	0	0	0	0	0	0	0	0
0	0	0	0	0	0	0	0	0	0	0	0	0	0	0	0	0	0	0
0	0	0	0	0	0	0	0	0	0	0	0	0	0	0	0	0	0	0
0	0	0	0	0	0	0	0	0	0	0	0	0	0	0	0	0	0	0
0	0	0	0	0	0	0	0	0	0	0	0	0	0	0	0	0	0	0
0	0	0	0	0	0	0	0	0	0	0	0	0	0	0	0	0	0	0
0	0	0	0	0	0	0	0	0	0	0	0	0	0	0	0	0	0	0
0	0	0	0	0	0	0	0	0	0	0	0	0	0	0	0	0	0	0
0	0	0	0	0	0	0	0	0	0	0	0	0	0	0	0	0	0	0
0	0	0	0	0	0	0	0	0	0	0	0	0	0	0	0	0	0	0
0	0	0	0	0	0	0	0	0	0	0	0	0	0	0	0	0	0	0
0	0	0	0	0	0	0	0	0	0	0	0	0	0	0	0	0	0	0
0	0	0	0	0	0	0	0	0	0	0	0	0	0	0	0	0	0	0

0	0	0	0	0	0	0	0	0	0	0	0	0	0	0	0	0	0	0
0	0	0	0	0	0	0	0	0	0	0	0	0	0	0	0	0	0	0
0	0	0	0	0	0	7	0	0	0	0	0	0	0	0	0	0	0	0
0	0	0	0	0	0	0	0	0	0	0	0	0	0	0	0	0	0	0
0	0	0	0	0	0	0	0	0	0	0	0	0	0	0	0	0	0	0
0	0	0	0	0	0	0	0	0	0	0	0	0	0	0	0	0	0	0
0	0	0	0	0	0	0	0	0	0	0	0	0	0	0	0	0	0	0
0	0	0	0	0	0	0	0	0	0	0	0	0	0	0	0	0	0	0
0	0	0	0	0	0	0	0	0	0	0	0	0	0	0	0	0	0	0
0	0	0	0	0	0	0	0	0	0	0	0	0	0	0	0	0	0	0
0	0	0	0	0	0	0	0	0	0	0	0	0	0	0	0	0	0	0
0	0	0	0	0	0	0	0	0	0	0	0	0	0	0	0	0	0	0
0	0	0	0	0	0	0	0	0	0	0	0	0	0	0	0	0	0	0
0	0	0	0	0	0	0	0	0	0	0	0	0	0	0	0	0	0	0
0	0	0	0	0	0	0	0	0	0	0	0	0	0	0	0	0	0	0
0	0	0	0	0	0	0	0	0	0	0	0	0	0	0	0	0	0	0
0	0	0	0	0	0	0	0	0	0	0	0	0	0	0	0	0	0	0
0	0	0	0	0	0	0	0	0	0	0	0	0	0	0	0	0	0	0
0	0	0	0	0	0	0	0	0	0	0	0	0	0	0	0	0	0	0
0	0	0	0	0	0	0	0	0	0	0	0	0	0	0	0	0	0	0
0	0	0	0	0	0	0	0	0	0	0	0	0	0	0	0	0	0	0
0	0	0	0	0	0	0	0	0	0	0	0	0	0	0	0	0	0	0
0	0	0	0	0	0	0	0	0	0	0	0	0	0	0	0	0	0	0
0	0	0	0	0	0	0	0	0	0	0	0	0	0	0	0	0	0	0
0	0	0	0	0	0	0	0	0	0	0	0	0	0	0	0	0	0	0
0	0	0	0	0	0	0	0	0	0	0	0	0	0	0	0	0	0	0
0	0	0	0	0	0	0	0	0	4	4	4	0	0	0	0	0	0	0
0	0	0	0	0	0	0	0	0	0	0	0	0	0	0	0	0	0	0
0	0	0	0	0	0	0	0	0	0	0	0	0	0	0	0	0	0	0
0	0	0	0	0	0	0	0	0	0	0	0	0	0	0	0	0	0	0
0	0	0	0	0	0	0	0	0	0	0	0	0	0	0	0	0	0	0
0	0	0	0	0	0	0	0	0	0	0	0	0	0	0	0	0	0	0
0	0	0	0	0	0	0	0	0	0	0	0	0	0	0	0	0	0	0
0	0	0	0	0	0	0	0	0	0	0	0	0	0	0	0	0	0	0
0	0	0	0	0	0	0	0	0	0	0	0	0	0	0	0	0	0	0
0	0	0	0	0	0	0	0	0	0	0	0	0	0	0	0	0	0	0
0	0	0	0	0	0	0	0	0	0	0	0	0	0	0	0	0	0	0

0	0	0	0	0	0	0	0	0	0	0	0	0	0	0	0	0	0	0
0	0	0	0	0	0	0	0	0	0	0	0	0	0	0	0	0	0	0
0	0	0	0	0	0	0	0	0	0	0	0	0	0	0	0	0	0	0
0	0	0	0	0	0	0	0	0	0	0	0	0	0	0	0	0	0	0
0	0	0	0	0	0	0	0	0	0	0	0	0	0	0	0	0	0	0
3	3	3	3	0	0	0	0	0	0	0	0	0	0	0	0	0	0	0
0	0	0	0	0	0	0	0	0	0	0	0	0	0	0	0	0	0	0
0	0	0	0	0	0	0	0	0	0	0	0	0	0	0	0	0	0	0
0	0	0	0	0	0	0	0	0	0	0	0	0	0	0	0	0	0	0
0	0	0	0	0	0	0	0	0	0	0	0	0	0	0	0	0	0	0
0	0	0	0	0	0	0	0	0	0	0	0	0	0	0	0	0	0	0
0	0	0	0	0	0	0	0	0	0	0	0	0	0	0	0	0	0	0
0	0	0	0	0	0	0	0	0	0	0	0	0	0	0	0	0	0	0
0	0	0	0	0	0	0	0	0	0	0	0	0	0	0	0	0	0	0
0	0	0	0	0	0	0	0	0	0	0	0	0	0	0	0	0	0	0
0	0	0	0	0	0	0	0	0	0	0	0	0	0	0	0	0	0	0
0	0	0	0	0	0	0	0	0	0	0	0	0	0	0	0	0	0	0
0	0	0	0	0	0	0	0	0	0	0	0	0	0	0	0	0	0	0
0	0	0	0	0	0	0	0	0	0	0	0	0	0	0	0	0	0	0
0	0	0	0	0	0	0	0	0	0	0	0	0	0	0	0	0	0	0
0	0	0	0	0	0	0	0	0	0	0	0	0	0	0	0	0	0	0
0	0	0	0	0	0	0	0	0	0	0	0	0	0	0	0	0	0	0
0	0	0	0	0	0	0	0	0	0	0	0	0	0	0	0	0	0	0
0	0	0	0	0	0	0	0	0	0	0	0	0	0	0	0	0	0	0
0	0	0	0	0	0	0	0	0	0	0	0	0	0	0	0	0	0	0
0	0	0	0	0	0	0	0	0	0	0	0	0	0	0	0	0	0	0
0	0	0	0	0	0	0	0	0	0	0	0	0	0	0	0	0	0	0
0	0	0	0	0	0	0	0	0	0	0	0	0	0	0	0	0	0	0
0	0	0	0	0	0	0	0	0	0	0	0	0	0	0	0	0	0	0
0	0	0	0	0	0	0	0	0	0	0	0	0	0	0	0	0	0	0
0	0	0	0	0	0	0	0	0	0	0	0	0	0	0	0	0	0	0
0	0	0	0	0	0	0	0	0	0	0	0	0	0	0	0	0	0	0
0	0	0	0	2	2	2	0	0	0	0	0	0	0	0	0	0	0	0
0	0	0	0	0	0	0	0	0	0	0	0	0	0	0	0	0	0	0
0	0	0	0	0	0	0	0	0	0	0	0	0	0	0	0	0	0	0
0	0	0	0	0	0	0	0	0	0	0	0	0	0	0	0	0	0	0
0	0	0	0	0	0	0	0	0	0	0	0	0	0	0	0	0	0	0

0	0	0	0	0	0	0	0	0	0	0	0	0	0	0	0	0	0	0
0	0	0	0	0	0	0	0	0	0	0	0	0	0	0	0	0	0	0
0	0	0	0	0	0	0	0	0	0	0	0	0	0	0	0	0	0	0
0	0	0	0	0	0	0	0	0	0	0	0	0	0	0	0	0	0	0
0	0	0	0	0	0	0	0	0	0	0	0	0	0	0	0	0	0	0
0	0	0	0	0	0	0	0	0	0	0	0	0	0	0	0	0	0	0
0	0	0	0	0	0	0	0	0	0	0	0	0	0	0	0	0	0	0
0	0	0	0	0	0	0	0	0	0	0	0	0	0	0	0	0	0	0
0	0	0	0	0	0	0	0	0	0	0	0	0	0	0	0	0	0	0
0	0	0	0	0	0	0	0	0	0	0	0	0	0	0	0	0	0	0
0	0	0	0	0	0	0	0	0	0	0	0	0	0	0	0	0	0	0
0	0	0	0	0	0	0	0	0	0	0	0	0	0	0	0	0	0	0
0	0	0	0	0	0	0	0	0	0	0	0	0	0	0	0	0	0	0
0	0	0	0	0	0	0	0	0	0	0	0	0	0	0	0	0	0	0
0	0	0	0	0	0	0	0	0	0	0	0	0	0	0	0	0	0	0
0	0	0	0	0	0	0	0	0	0	0	0	0	0	0	0	0	0	0
0	0	0	0	0	0	0	0	0	0	0	0	0	0	0	0	0	0	0
0	0	0	0	0	0	0	0	0	0	0	0	0	0	0	0	0	0	0
0	0	0	0	0	0	0	0	0	0	0	0	0	0	0	0	0	0	0
0	0	0	0	0	0	0	0	0	0	0	0	0	0	0	0	0	0	0
0	0	0	0	0	0	0	0	0	0									
Jan_DL147	1763	1134	385	391	46	943	248	230	641	276	34	519	238	185	140	257	164	230
273	426	125	0	148	80	50	180	77	52	115	0	96	118	133	0	168	411	0
52	62	89	155	122	0	62	65	63	111	98	56	89	155	221	52	137	122	0
0	0	0	5	82	23	74	56	0	95	24	84	8	56	449	0	18	32	0
0	10	124	22	0	0	119	111	33	38	98	0	37	0	23	46	46	39	21
33	63	78	126	0	0	0	54	0	0	0	0	0	33	22	0	20	0	0
0	0	0	47	0	0	0	0	0	0	10	0	12	0	0	0	0	0	104
0	5	0	8	0	0	0	0	62	22	0	0	0	0	0	18	0	0	0
0	0	12	0	0	0	22	0	0	0	10	0	0	0	0	0	0	0	0
34	55	0	0	23	0	0	11	0	43	12	0	0	0	0	0	15	40	8
0	0	17	0	0	0	0	0	0	5	0	0	0	0	0	0	0	22	0
0	7	8	0	5	0	0	0	0	8	44	42	32	0	0	540	17	17	14
0	0	0	0	0	0	0	0	0	0	0	0	0	36	0	0	0	0	0
0	0	0	5	0	0	0	18	0	6	0	0	0	0	7	0	0	0	0
10	0	28	0	0	0	43	0	13	0	0	2	0	0	0	0	0	0	0
0	0	0	48	0	0	0	0	0	0	0	0	7	0	0	0	0	0	0

0	0	14	0	0	0	0	0	0	0	54	0	0	0	0	12	0	0	10
11	0	0	0	0	0	0	10	0	0	0	0	0	0	0	0	0	0	0
8	0	0	0	7	0	0	0	7	0	0	9	0	0	0	0	0	0	0
0	0	0	0	0	0	0	2	0	13	0	0	0	0	0	0	0	0	0
0	0	0	0	17	0	0	0	0	0	17	0	10	0	0	0	0	0	0
0	0	0	0	0	0	0	7	0	0	0	0	0	24	0	0	17	0	0
0	0	0	0	0	0	10	0	6	0	0	60	13	0	9	0	6	0	0
0	0	0	0	0	0	0	0	0	0	0	0	0	0	0	0	0	0	11
0	0	0	0	0	0	0	0	0	0	0	0	0	0	0	0	0	0	0
0	0	24	0	0	0	0	0	0	0	0	0	0	0	0	0	0	0	13
0	0	0	0	0	0	0	0	0	0	0	0	0	0	0	0	0	0	0
0	0	12	0	0	0	0	0	0	0	0	0	0	0	0	14	0	0	0
0	0	0	0	0	0	0	0	13	0	9	0	0	0	0	0	0	0	0
0	0	0	0	0	0	0	0	0	0	10	0	8	0	0	0	0	0	0
0	0	0	0	0	0	0	0	0	0	3	0	0	0	4	0	0	0	0
0	0	0	0	0	0	0	0	0	0	0	0	0	0	0	0	0	0	0
0	0	0	0	0	0	0	0	0	0	0	0	45	0	0	0	0	0	0
0	0	0	0	0	0	0	0	0	0	0	0	0	0	0	0	0	0	0
0	0	0	0	0	0	0	0	0	0	0	0	0	0	0	0	0	0	0
0	0	0	0	0	0	0	0	0	0	0	0	0	0	0	0	0	0	0
0	0	0	0	0	0	0	0	0	0	0	0	0	0	0	0	0	0	0
0	0	0	0	0	0	0	0	0	0	0	0	0	0	0	0	0	0	0
0	0	0	0	0	0	0	0	0	0	0	0	10	0	0	0	0	0	0
7	0	0	0	0	0	0	0	0	0	0	0	0	0	0	0	7	0	0
0	0	0	0	0	0	0	0	0	0	0	0	0	0	0	0	0	0	0
0	0	0	0	0	0	0	0	0	0	4	0	0	0	0	0	0	0	0
0	0	0	0	0	0	0	0	0	0	0	0	0	0	0	0	0	0	0
0	0	0	0	0	0	0	0	7	0	0	0	0	0	0	0	0	0	0
0	0	0	0	0	0	0	0	0	0	0	0	0	0	0	0	0	0	0
0	0	0	0	0	0	0	0	0	0	0	0	0	0	0	0	0	0	0
0	0	0	0	11	0	13	0	0	0	0	0	0	0	0	0	0	0	0
0	0	0	0	0	0	0	0	0	0	0	0	0	0	0	0	0	0	0
0	0	0	0	0	0	0	0	0	0	0	0	0	0	0	0	0	0	0
0	0	0	0	0	0	0	0	0	0	0	0	0	0	0	0	0	0	0
0	0	0	0	0	2	0	0	0	0	0	0	3	0	0	0	0	0	0
0	0	0	0	0	0	0	0	0	0	0	0	0	0	0	0	0	0	0
0	0	0	0	0	0	0	0	0	0	0	0	0	0	0	0	0	0	0

0	0	0	0	0	0	0	0	0	0	0	0	0	0	0	0	0	0	0
0	0	0	0	0	0	0	0	0	0	0	0	0	0	0	0	0	0	0
0	0	0	0	0	0	0	0	0	0	0	0	0	0	0	0	0	0	0
0	0	0	0	0	0	0	0	0	0	0	0	0	0	7	0	0	0	0
0	0	0	0	0	0	0	0	0	0	0	0	0	0	0	0	0	0	0
0	0	0	0	0	0	0	0	0	0	0	0	0	0	0	0	0	0	0
0	0	0	0	0	0	0	0	0	0	0	0	0	0	0	0	0	0	0
0	0	0	0	0	0	0	0	0	0	0	0	0	0	0	0	0	0	0
0	0	0	0	0	0	0	0	0	0	0	0	0	0	0	0	0	0	0
0	0	0	0	0	0	0	0	0	0	0	0	0	0	0	0	0	0	0
0	0	0	0	0	0	0	0	0	0	0	0	0	0	0	0	0	0	0
0	0	0	0	0	0	0	0	0	0	0	0	0	0	0	0	0	0	0
0	0	0	0	0	0	0	0	0	0	0	0	0	0	0	0	0	0	0
0	0	0	0	0	0	0	0	0	0	0	0	0	0	0	0	0	0	0
0	0	0	0	0	0	0	0	0	0	0	0	0	0	0	0	0	0	0
0	0	0	0	0	0	0	0	0	0	0	0	0	0	0	0	0	0	0
0	0	0	0	0	0	0	0	0	0	0	0	0	0	0	0	0	0	0
0	0	0	0	0	0	0	0	0	0	0	0	0	0	0	0	0	0	0
0	0	0	0	0	0	0	0	0	0	0	0	0	0	0	0	0	0	0
0	0	0	0	0	0	0	0	0	0	0	0	0	0	0	0	0	0	0
0	0	0	0	0	0	0	0	0	0	0	0	0	0	0	0	0	0	0
0	0	0	0	0	0	0	0	0	0	0	0	0	0	0	0	0	0	0
0	0	0	0	0	0	0	0	0	0	0	0	0	0	0	0	0	0	0
0	0	0	0	0	0	0	0	0	0	0	0	0	0	0	0	0	0	0
0	10	10	0	0	0	0	0	0	0	0	0	0	0	0	0	0	0	0
0	0	0	0	0	0	0	0	0	0	0	0	0	0	0	0	0	0	0
0	0	0	0	0	0	0	0	0	0	0	0	0	0	0	0	0	0	0
0	0	0	0	0	0	0	0	0	0	0	0	0	0	0	0	0	0	0
0	0	0	0	0	0	0	0	0	0	0	0	0	0	0	0	0	0	0
0	0	0	0	0	0	0	0	0	0	0	0	0	0	0	0	0	0	0
0	0	0	0	0	0	0	0	0	0	0	0	0	0	0	0	0	0	0
0	0	0	0	0	0	0	0	0	0	0	0	0	0	0	0	0	0	0
0	0	0	0	0	0	0	0	0	0	0	0	0	0	0	0	0	0	0
0	0	0	0	0	0	0	0	0	0	0	0	0	0	0	0	0	0	0
0	0	0	0	0	0	0	0	0	0	0	0	0	0	0	0	0	0	0
0	0	0	0	0	0	0	0	0	0	0	0	0	0	0	0	0	0	0
0	0	0	0	0	0	0	0	0	0	0	0	0	0	0	0	0	0	0

0	0	0	7	0	0	0	0	0	0	0	0	0	0	0	0	0	0	0
0	0	0	0	0	0	0	0	0	0	0	0	0	0	0	0	0	0	0
0	0	0	0	0	0	0	0	0	0	0	0	0	0	0	0	0	0	0
0	0	0	0	0	0	0	0	0	0	0	0	0	0	0	0	0	0	0
0	0	0	0	0	0	0	0	0	0	0	0	0	0	0	0	0	0	0
0	0	0	0	0	0	0	0	0	0	0	0	0	0	0	0	0	0	0
0	0	0	0	0	0	0	0	0	0	0	0	0	0	0	0	0	0	0
0	0	0	0	0	0	0	0	0	0	0	0	0	0	0	0	0	0	0
0	0	0	0	0	0	0	0	0	0	0	0	0	0	0	0	0	0	0
0	0	0	0	0	0	0	0	0	0	0	0	0	0	0	0	0	0	0
0	0	0	0	0	0	0	0	0	0	0	0	0	0	0	0	0	0	0
0	0	0	0	0	0	0	0	0	0	0	0	0	0	0	0	0	0	0
0	0	0	0	0	0	0	0	0	0	0	0	0	0	0	0	0	0	0
0	0	0	0	0	0	0	0	0	0	0	0	0	0	0	0	0	0	0
0	0	0	0	0	0	0	0	0	0	0	0	0	5	5	5	0	0	0
0	0	0	0	0	0	0	0	0	0	0	0	0	0	0	0	0	0	0
0	0	0	0	0	0	0	0	0	0	0	0	0	0	0	0	0	0	0
0	0	0	0	0	0	0	0	0	0	0	0	0	0	0	0	0	0	0
0	0	0	0	0	0	0	0	0	0	0	0	0	0	0	0	0	0	0
0	0	0	0	0	0	0	0	0	0	0	0	0	0	0	0	0	0	0
0	0	0	0	0	0	0	0	0	0	0	0	0	0	0	0	0	0	0
0	0	0	0	0	0	0	0	0	0	0	0	0	0	0	0	0	0	0
0	0	0	0	0	0	0	0	0	0	0	0	0	0	0	0	0	0	0
0	0	0	0	0	0	0	0	0	0	0	0	0	0	0	0	0	0	0
0	0	0	0	0	0	0	0	0	0	0	0	0	0	0	0	0	0	0
0	0	0	0	0	0	0	0	0	0	0	4	0	0	0	0	0	0	0
0	0	0	0	0	0	0	0	0	0	0	0	0	0	0	0	0	0	0
0	0	0	0	0	0	0	0	0	0	0	0	0	0	0	0	0	0	0
0	0	0	0	0	0	0	0	0	0	0	0	0	0	0	0	0	0	0
0	0	0	0	0	0	0	0	0	0	0	0	0	0	0	0	0	0	0
0	0	0	0	0	0	0	0	0	0	0	0	0	0	0	0	0	0	0
0	0	0	0	0	0	0	0	0	0	0	0	0	0	0	0	0	0	0
0	0	0	0	0	0	0	0	0	0	0	0	0	0	0	0	0	0	0
0	0	0	0	0	0	0	0	0	0	0	0	0	0	0	0	0	0	0
0	0	0	0	0	0	0	0	0	0	0	0	0	0	0	0	0	0	0
0	0	0	0	0	0	0	0	0	0	0	0	0	0	0	0	0	0	0
0	0	0	0	0	0	0	0	0	0	0	0	0	0	0	0	0	0	0

0	0	0	0	0	0	0	0	0	0	0	0	0	0	0	0	0	0	0
0	0	0	0	0	0	0	0	0	0	0	0	0	0	0	0	0	0	0
0	0	0	0	0	0	0	0	0	0	0	0	0	0	0	0	0	0	0
0	0	0	0	0	0	0	0	0	0	0	0	0	0	0	0	0	0	0
0	0	0	0	0	0	0	0	0	0	0	0	0	0	0	0	0	0	0
0	0	0	3	3	0	0	0	0	0	0	0	0	0	0	0	0	0	0
0	0	0	0	0	0	0	0	0	0	0	0	0	0	0	0	0	0	0
0	0	0	0	0	0	0	0	0	0	0	0	0	0	0	0	0	0	0
0	0	0	0	0	0	0	0	0	0	0	0	0	0	0	0	0	0	0
0	0	0	0	0	0	0	0	0	0	0	0	0	0	0	0	0	0	0
0	0	0	0	0	0	0	0	0	0	0	0	0	0	0	0	0	0	0
0	0	0	0	0	0	0	0	0	0	0	0	0	0	0	0	0	0	0
0	0	0	0	0	0	0	0	0	0	0	0	0	0	0	0	0	0	0
0	0	0	0	0	0	0	0	0	0	0	0	0	0	0	0	0	0	0
0	0	0	0	0	0	0	0	0	0	0	0	0	0	0	0	0	0	0
0	0	0	0	0	0	0	0	0	0	0	0	0	0	0	0	0	0	0
0	0	0	0	0	0	0	0	0	0	0	0	0	0	0	0	0	0	0
0	0	0	0	0	0	0	0	0	0	0	0	0	0	0	0	0	0	0
0	0	0	0	0	0	0	0	0	0	0	0	0	0	0	0	0	0	0
0	0	0	0	0	0	0	0	0	0	0	0	0	0	0	0	0	0	0
0	0	0	0	0	0	0	0	0	0	0	0	0	0	0	0	0	0	0
0	0	0	0	0	0	0	0	0	0	0	0	0	0	0	0	0	0	0
0	0	0	0	0	0	0	0	0	0	0	0	0	0	0	0	0	0	0
0	0	0	0	0	0	0	0	0	0	0	0	0	0	0	0	0	0	0
0	0	0	0	0	0	0	0	0	0	0	0	0	0	0	0	0	0	0
0	0	0	0	0	0	0	0	0	0	0	0	0	0	0	0	0	0	0
0	0	0	0	0	0	0	0	0	0	0	0	0	0	0	0	0	0	0
0	0	0	0	0	0	0	0	0	0	0	0	0	0	0	0	0	0	0
0	0	0	0	0	0	0	0	0	0	0	0	0	0	0	0	0	0	0
0	0	0	0	0	0	0	0	0	0	0	0	0	0	0	0	0	0	0
0	0	0	0	0	0	0	0	0	0	0	0	0	0	0	0	0	0	0
0	0	0	0	0	0	0	0	0	0	0	0	0	0	0	0	0	0	0
0	0	0	0	0	0	0	0	0	0	2	2	2	2	2	2	2	2	0
0	0	0	0	0	0	0	0	0	0	0	0	0	0	0	0	0	0	0
0	0	0	0	0	0	0	0	0	0	0	0	0	0	0	0	0	0	0
0	0	0	0	0	0	0	0	0	0	0	0	0	0	0	0	0	0	0
0	0	0	0	0	0	0	0	0	0	0	0	0	0	0	0	0	0	0

0	0	0	0	0	0	0	0	0	0	0	0	0	0	0	0	0	0	0
0	0	0	0	0	0	0	0	0	0	0	0	0	0	0	0	0	0	0
0	0	0	0	0	0	0	0	0	0	0	0	0	0	0	0	0	0	0
0	0	0	0	0	0	0	0	0	0	0	0	0	0	0	0	0	0	0
0	0	0	0	0	0	0	0	0	0	0	0	0	0	0	0	0	0	0
0	0	0	0	0	0	0	0	0	0	0	0	0	0	0	0	0	0	0
0	0	0	0	0	0	0	0	0	0	0	0	0	0	0	0	0	0	0
0	0	0	0	0	0	0	0	0	0	0	0	0	0	0	0	0	0	0
0	0	0	0	0	0	0	0	0	0	0	0	0	0	0	0	0	0	0
0	0	0	0	0	0	0	0	0	0	0	0	0	0	0	0	0	0	0
0	0	0	0	0	0	0	0	0	0	0	0	0	0	0	0	0	0	0
0	0	0	0	0	0	0	0	0	0	0	0	0	0	0	0	0	0	0
0	0	0	0	0	0	0	0	0	0									
Jan_DL148	1380	357	807	677	70	460	291	272	254	254	381	425	239	131	77	72	67	296
384	1396	74	278	233	59	335	199	325	135	130	0	161	167	136	164	115	385	0
132	68	0	149	127	165	129	0	55	28	111	198	92	218	70	220	0	86	0
0	170	182	18	71	0	79	121	70	80	12	56	0	82	10	56	22	50	0
59	0	31	0	0	57	60	22	0	0	0	104	65	0	27	0	89	0	220
72	45	75	0	36	9	139	0	40	24	18	0	0	0	29	20	0	0	33
0	31	0	36	0	0	0	0	0	0	0	0	15	13	0	15	0	0	0
0	0	0	0	18	0	0	0	0	14	0	0	3	0	0	0	0	0	0
0	0	21	0	0	0	0	0	0	0	0	0	34	29	0	0	0	0	12
34	0	0	16	0	108	0	0	0	0	0	19	0	30	0	13	7	0	0
0	0	9	0	0	26	0	0	0	0	0	0	0	0	0	0	0	0	0
0	0	13	0	0	0	9	0	0	34	0	0	0	0	0	0	0	29	0
0	0	0	0	0	0	0	0	0	0	0	66	15	24	0	0	0	0	0
0	0	0	21	17	0	0	0	0	5	0	0	16	0	0	9	0	0	0
0	0	24	0	0	0	0	0	9	0	0	0	0	0	0	0	0	0	0
0	0	5	0	0	0	0	0	0	0	0	0	0	0	13	0	0	13	30
0	0	0	0	0	13	0	0	0	0	0	0	0	7	0	13	0	0	17
0	0	0	11	22	0	0	20	0	0	0	0	0	0	0	0	0	22	0
0	8	0	0	10	0	0	23	0	0	0	0	0	0	12	0	0	0	0
0	0	0	0	0	0	0	0	0	0	20	0	5	0	0	0	0	0	3
0	0	0	0	0	0	0	0	0	0	0	0	0	0	0	0	0	0	0
0	0	0	11	0	8	0	0	0	0	0	0	0	0	15	11	9	0	0
0	0	0	0	0	0	0	0	0	35	0	0	0	0	10	0	9	0	0
0	0	0	0	0	0	0	0	0	0	0	0	0	0	0	0	0	0	0

0	0	0	0	0	0	0	6	0	0	0	0	0	0	0	0	0	0	0
0	0	0	0	0	0	0	0	0	0	0	0	9	0	0	0	0	0	0
0	0	0	0	0	0	0	0	0	16	0	0	0	0	0	0	0	0	0
0	0	0	0	0	0	0	0	0	0	0	0	0	0	0	0	6	0	0
0	0	0	0	0	0	0	0	0	0	0	7	0	0	0	0	0	0	0
0	0	0	0	0	0	0	0	0	0	0	0	0	0	0	0	0	0	0
0	0	0	0	0	0	0	0	0	14	0	0	0	0	0	0	0	0	0
0	0	0	0	0	0	0	0	0	0	0	0	0	0	22	0	0	0	0
0	0	0	0	0	0	0	0	0	0	0	0	0	0	0	0	0	0	0
0	0	0	0	0	0	0	0	0	0	0	0	0	0	0	9	0	0	0
0	0	0	0	0	0	19	0	0	0	0	0	0	0	0	0	0	0	0
0	0	5	0	0	0	30	0	0	0	0	0	0	0	0	11	0	0	0
0	0	0	0	0	0	0	0	0	0	0	0	0	0	0	0	0	0	0
0	6	0	0	0	0	0	0	0	0	0	0	0	0	0	0	0	0	0
0	0	0	0	0	0	0	0	0	0	0	0	0	0	0	0	0	0	0
0	8	0	0	0	0	0	0	0	0	0	0	0	0	0	0	0	0	0
0	0	0	0	0	0	0	0	0	0	0	0	0	0	0	0	0	0	0
0	0	0	0	0	0	0	0	0	0	0	0	0	0	0	0	0	0	6
0	0	0	0	0	0	0	0	0	0	3	0	0	0	0	0	0	0	0
0	6	0	0	0	0	0	0	0	0	0	0	0	0	7	0	0	0	0
0	0	0	0	0	0	0	0	0	0	0	0	0	0	0	0	0	0	0
0	0	0	0	0	0	0	0	0	0	0	0	0	0	0	0	0	0	0
0	0	0	0	0	0	0	0	0	0	0	0	0	0	0	0	0	0	0
0	0	0	0	0	0	0	0	0	0	0	0	0	0	0	0	0	0	0
0	0	0	0	0	0	0	0	0	0	0	0	0	0	0	0	0	0	0
0	0	0	0	0	0	0	0	0	0	0	0	0	0	0	0	0	0	0
0	0	0	0	0	0	0	0	0	0	0	0	0	0	0	0	0	0	0
0	0	0	0	0	0	0	0	0	0	0	0	0	0	0	0	0	0	0
0	0	0	0	0	0	0	0	0	0	0	0	0	0	0	0	0	0	0
0	0	0	0	0	0	0	0	0	0	0	0	0	0	0	0	0	0	0
0	0	0	0	0	0	0	0	0	0	0	0	0	0	0	0	0	0	0
0	0	0	0	0	0	0	0	0	0	10	0	0	0	0	0	0	0	0
0	0	0	0	0	0	0	0	0	0	0	0	0	0	0	0	0	0	0
0	0	0	0	0	0	0	0	0	0	0	0	0	0	0	0	0	0	0
0	0	0	0	0	0	0	0	0	0	0	0	0	0	0	0	0	0	0
0	0	0	0	0	0	0	0	0	0	0	0	0	0	0	0	0	0	0
0	0	0	0	0	0	0	0	0	0	0	0	0	0	0	0	0	0	0

0	7	0	0	0	0	0	0	0	0	0	0	0	0	0	0	0	0	0
0	0	0	0	0	0	0	0	0	0	0	0	0	0	0	0	0	0	0
0	0	0	0	0	0	0	0	0	0	0	0	0	0	0	0	0	0	0
0	0	0	0	0	0	0	0	0	0	0	0	0	0	0	0	0	0	0
0	0	0	0	0	0	0	0	0	0	0	0	0	0	0	0	0	0	3
0	0	0	0	0	0	0	0	0	0	0	0	0	0	2	0	0	0	0
0	0	0	0	0	0	0	0	0	0	0	0	0	0	0	0	0	0	0
0	0	0	0	0	0	0	0	0	0	0	0	0	0	0	0	0	0	0
0	0	0	0	0	0	0	0	0	0	0	0	0	0	0	0	0	0	0
0	0	0	0	0	0	0	0	0	0	0	0	0	0	0	0	0	0	0
0	0	0	0	0	0	0	0	0	0	0	0	0	0	0	0	0	0	0
0	0	0	0	0	0	0	0	0	0	0	0	0	0	0	0	0	0	0
0	0	0	0	0	0	0	0	0	0	0	0	0	0	0	0	0	0	0
0	0	0	0	0	0	0	0	0	0	0	0	0	0	0	0	0	0	0
0	0	0	0	3	0	0	0	0	0	0	0	0	0	0	0	0	0	0
0	0	0	0	0	0	0	0	0	0	0	0	0	0	0	0	0	0	0
0	0	0	0	0	0	0	0	0	0	0	0	0	0	0	0	0	0	0
0	0	0	0	0	0	0	0	0	0	0	0	0	0	0	0	0	0	0
4	0	0	0	0	0	0	0	0	0	0	0	0	0	0	0	0	0	0
0	0	0	0	0	0	0	0	0	0	0	0	0	0	0	0	0	0	0
0	0	0	0	0	0	0	0	0	0	0	0	0	0	0	0	0	0	0
0	0	0	0	0	0	0	0	0	0	0	0	0	0	0	0	0	0	0
0	0	0	0	0	0	0	0	0	0	0	0	0	0	0	0	0	0	0
0	0	0	0	0	0	0	0	0	0	0	0	0	0	0	0	0	0	0
0	0	0	0	0	0	0	0	0	0	0	0	0	0	0	0	0	0	0
0	0	0	0	0	0	0	0	0	0	0	0	0	0	0	0	0	0	0
0	0	0	0	0	0	0	0	0	0	0	0	0	0	0	0	0	0	0
0	0	0	0	0	0	0	0	0	0	0	0	0	0	0	0	0	0	0
0	0	0	0	0	0	0	0	0	0	0	0	0	0	3	0	0	0	0
0	0	0	0	0	0	0	0	0	0	0	0	0	0	0	0	0	0	0
0	7	7	0	0	0	0	0	0	0	0	0	0	0	0	0	0	0	0
0	0	0	0	0	0	0	0	0	0	0	0	0	0	0	0	0	0	0
0	0	0	0	0	0	0	0	0	0	0	0	0	0	0	0	0	0	0
0	0	0	0	0	0	0	0	0	0	0	0	0	0	0	0	0	0	0
0	0	0	0	0	0	0	0	0	0	0	0	0	0	0	0	0	0	0
0	0	0	0	0	0	0	0	0	0	0	0	0	0	0	0	0	0	0
0	0	0	0	0	0	0	0	0	0	6	6	0	0	0	0	0	0	0

0	0	0	0	0	0	0	0	0	0	0	0	0	0	0	0	0	0	0
0	0	0	0	0	0	0	0	0	0	0	0	0	0	0	0	0	0	0
0	0	0	0	0	0	0	0	0	0	0	0	0	0	0	0	0	0	0
0	0	0	0	0	0	0	0	0	0	0	0	0	0	0	0	0	0	0
0	0	0	0	0	0	0	0	0	0	0	0	0	0	0	0	0	0	0
0	0	0	0	0	0	0	0	0	0	0	0	0	0	0	0	0	0	0
0	0	0	0	0	0	0	0	0	0	0	0	0	0	0	0	0	0	0
0	0	0	0	0	0	0	0	0	0	0	0	0	0	0	0	0	0	5
0	0	0	0	0	0	0	0	0	0	0	0	0	0	0	0	0	0	0
0	0	0	0	0	0	0	0	0	0	0	0	0	0	0	0	0	0	0
0	0	0	0	0	0	0	0	0	0	0	0	0	0	0	0	0	0	0
0	0	0	0	0	0	0	0	0	0	0	0	0	0	0	0	0	0	0
0	0	0	0	0	0	0	0	0	0	0	0	0	0	0	0	0	0	0
0	0	0	0	0	0	0	0	0	0	0	0	0	0	0	0	0	0	0
0	0	0	0	0	0	0	0	0	0	0	0	0	0	0	0	0	0	0
0	0	0	0	0	0	0	0	0	0	0	0	0	0	0	0	0	0	0
0	0	0	0	0	0	0	0	0	0	0	0	0	0	0	0	0	0	0
0	0	0	0	0	0	0	0	0	0	0	0	0	0	0	0	0	0	0
0	0	0	0	0	0	0	0	0	0	0	0	4	4	0	0	0	0	0
0	0	0	0	0	0	0	0	0	0	0	0	0	0	0	0	0	0	0
0	0	0	0	0	0	0	0	0	0	0	0	0	0	0	0	0	0	0
0	0	0	0	0	0	0	0	0	0	0	0	0	0	0	0	0	0	0
0	0	0	0	0	0	0	0	0	0	0	0	0	0	0	0	0	0	0
0	0	0	0	0	0	0	0	0	0	0	0	0	0	0	0	0	0	0
0	0	0	0	0	0	0	0	0	0	0	0	0	0	0	0	0	0	0
0	0	0	0	0	0	0	0	0	0	0	0	0	0	0	0	0	0	0
0	0	0	0	0	0	0	0	0	0	0	0	0	0	0	0	0	0	0
0	0	0	0	0	0	0	0	0	0	0	0	0	0	0	0	0	0	0
0	0	0	0	0	0	0	0	0	0	0	0	0	0	0	0	0	0	0
0	0	0	0	0	0	0	0	0	0	0	0	0	0	0	0	0	0	0
0	0	0	0	0	0	0	0	0	0	0	0	0	0	0	0	0	0	0
0	0	0	0	0	0	0	0	0	0	0	0	0	0	0	0	0	0	0
0	0	0	0	0	0	0	0	0	0	0	0	0	0	0	0	0	0	0
0	0	0	0	0	0	0	0	0	0	0	0	0	0	0	0	0	0	0
0	0	0	0	0	0	0	0	0	0	0	0	0	0	0	0	3	0	0
0	0	0	0	0	0	0	0	0	0	0	0	0	0	0	0	0	0	0
0	0	0	0	0	0	0	0	0	0	0	0	0	0	0	0	0	0	0

0	0	0	0	0	0	0	0	0	0	0	0	0	0	0	0	0	0	0
0	0	0	0	0	0	0	0	0	0	0	0	0	0	0	0	0	0	0
0	0	0	0	0	0	0	0	0	0	0	0	0	0	0	0	0	0	0
0	0	0	0	0	0	0	0	0	0	0	0	0	0	0	0	0	0	0
0	0	0	0	0	0	0	0	0	0	0	0	0	0	0	0	0	0	0
0	0	0	0	0	0	0	0	0	0	0	0	0	0	0	0	0	0	0
0	0	0	0	0	0	0	0	0	0	0	0	0	0	0	0	0	0	0
0	0	0	0	0	0	0	0	0	0	0	0	0	0	0	0	0	0	0
0	0	0	0	0	0	0	0	0	0	0	0	0	0	0	0	0	0	0
0	0	0	0	0	0	0	0	0	0	0	0	0	0	0	0	0	0	0
0	0	0	0	0	0	0	0	0	0	0	0	0	0	0	0	0	0	0
0	0	0	0	0	0	0	0	0	0	0	0	0	0	0	0	0	0	0
0	0	0	0	0	0	0	0	0	0	0	0	0	0	0	0	0	0	0
0	0	0	0	0	0	0	0	0	0	0	0	0	0	0	0	0	0	0
0	0	0	0	0	0	0	0	0	0	0	0	0	0	0	0	0	0	0
0	0	0	0	0	0	0	0	0	0	0	0	0	0	0	0	0	0	0
0	0	0	0	0	0	0	0	0	0	0	0	0	0	0	0	0	0	0
0	0	0	0	0	0	0	0	0	0	0	0	0	0	0	0	0	0	0
0	0	0	0	0	0	0	0	0	0	0	0	0	0	0	0	0	0	0
0	0	0	0	0	0	0	0	0	0	0	0	0	0	0	0	0	0	0
0	0	0	0	0	0	0	0	0	0	0	0	0	0	0	0	0	0	0
0	0	0	0	0	0	0	0	0	0	0	0	0	0	0	0	0	0	0
0	0	0	0	0	0	0	0	0	0	0	0	0	0	0	0	0	0	0
0	0	0	0	0	0	0	0	0	0	0	0	0	0	0	0	0	0	0
0	0	0	0	0	0	0	0	0	0	0	0	0	0	0	0	0	0	0
0	0	0	0	0	0	0	0	2	2	2	0	0	0	0	0	0	0	0
0	0	0	0	0	0	0	0	0	0	0	0	0	0	0	0	0	0	0
0	0	0	0	0	0	0	0	0	0	0	0	0	0	0	0	0	0	0
0	0	0	0	0	0	0	0	0	0	0	0	0	0	0	0	0	0	0
0	0	0	0	0	0	0	0	0	0	0	0	0	0	0	0	0	0	0
0	0	0	0	0	0	0	0	0	0	0	0	0	0	0	0	0	0	0
0	0	0	0	0	0	0	0	0	0	0	0	0	0	0	0	0	0	0
0	0	0	0	0	0	0	0	0	0	0	0	0	0	0	0	0	0	0
0	0	0	0	0	0	0	0	0	0	0	0	0	0	0	0	0	0	0
0	0	0	0	0	0	0	0	0	0	0	0	0	0	0	0	0	0	0
0	0	0	0	0	0	0	0	0	0	0	0	0	0	0	0	0	0	0
0	0	0	0	0	0	0	0	0	0	0	0	0	0	0	0	0	0	0

0	0	0	0	0	0	0	0	0	0	0	0	0	0	0	0	0	0	0
0	0	0	0	0	0	0	0	0	0	0	0	0	0	0	0	0	0	0
0	0	0	0	0	0	0	0	0	0	0	0	0	0	0	0	0	0	0
0	0	0	0	0	0	0	0	0	0	0	0	0	0	0	0	0	0	0
0	0	0	0	0	0	0	0	0	0									
Jan_DL149	2149	2469	344	538	84	458	943	999	867	223	280	415	214	551	81	0	555	170
323	728	339	109	293	261	59	172	59	69	216	0	192	233	113	127	145	18	0
0	60	82	334	246	129	129	0	113	0	114	0	78	141	240	0	0	148	0
0	0	0	20	133	41	221	61	75	12	69	188	0	149	26	0	11	21	8
17	0	162	0	0	0	38	127	0	0	103	145	0	83	75	0	94	36	0
95	0	97	0	22	10	0	0	0	81	0	0	0	0	29	37	27	0	0
92	0	0	25	11	11	0	0	0	117	4	0	28	0	0	15	22	0	0
106	22	0	0	22	0	0	0	0	23	28	0	10	0	0	0	0	0	0
0	12	0	0	83	0	0	16	0	0	4	0	48	26	0	0	0	0	25
36	0	19	37	0	0	0	0	0	31	54	10	0	0	0	10	8	0	0
102	0	0	0	0	0	0	0	0	0	0	43	0	84	0	0	0	0	0
3	0	0	0	36	0	0	0	0	0	0	0	0	0	0	0	0	33	0
0	0	0	0	0	0	0	0	32	0	0	0	0	0	0	8	0	0	0
0	0	0	0	0	0	0	0	0	0	0	0	0	0	0	3	48	10	23
10	0	31	0	0	0	0	0	0	0	0	12	0	7	7	6	0	0	0
0	0	0	0	0	0	0	0	5	0	7	14	21	0	11	0	0	0	0
0	0	12	0	0	0	0	0	0	0	0	10	0	0	0	0	0	0	3
0	16	7	17	0	0	0	0	0	18	0	0	0	5	22	0	0	0	6
17	6	17	0	3	8	0	0	0	0	0	3	0	0	0	0	0	0	0
0	0	3	0	7	0	0	0	0	0	0	0	0	0	0	0	0	0	0
0	0	0	0	0	0	0	0	0	0	0	0	0	0	0	0	0	10	0
0	0	0	6	0	0	0	3	0	0	0	0	0	0	0	0	0	0	0
0	0	0	0	0	0	0	0	0	0	0	0	0	0	0	0	6	0	0
0	8	0	0	0	0	0	0	0	0	0	0	0	0	36	0	0	0	0
0	0	0	0	0	0	0	0	0	0	0	0	0	0	0	0	0	34	0
0	0	0	13	0	0	0	0	0	0	0	0	0	19	0	0	0	0	0
14	0	0	0	0	0	40	0	0	0	0	0	0	0	0	0	0	0	0
0	0	0	0	0	0	0	0	0	0	0	0	0	0	0	0	0	0	0
0	0	0	0	0	0	0	15	17	14	0	0	0	0	12	0	0	0	0
0	0	6	0	0	0	0	0	0	0	0	23	0	0	0	8	0	0	11
0	0	0	0	0	0	0	0	0	0	0	0	25	0	0	0	0	0	0
0	0	0	0	0	0	0	0	0	0	0	0	0	0	0	16	0	0	0

0	0	0	0	0	0	0	0	0	0	0	0	0	0	0	0	0	0	0
12	0	0	26	0	0	4	0	0	0	0	0	0	0	0	5	0	0	0
0	0	0	0	0	0	0	0	0	0	0	0	0	0	0	0	0	0	0
18	0	0	0	0	0	0	0	0	0	0	0	0	0	0	0	0	0	0
0	0	0	0	0	0	0	0	0	0	0	0	9	0	0	0	0	0	0
0	0	0	0	0	0	0	0	0	0	0	0	0	0	0	0	0	0	0
0	0	0	0	0	0	0	0	0	0	0	0	0	0	0	0	0	0	0
10	6	0	0	0	0	0	0	0	5	0	0	0	0	0	0	0	0	0
0	0	0	0	0	0	0	3	0	0	0	0	0	0	0	0	0	0	11
0	0	0	0	0	0	0	4	0	0	0	0	0	0	7	0	0	0	0
0	0	0	0	0	0	0	0	0	0	0	0	0	0	0	0	0	0	0
0	0	0	0	11	0	0	0	0	0	0	0	0	0	0	0	0	0	0
0	0	0	5	0	0	0	0	0	0	0	0	0	0	0	0	0	0	0
0	0	0	0	0	0	0	0	0	0	0	0	0	0	0	0	0	0	0
0	0	0	0	0	0	0	0	0	0	0	0	0	0	0	0	0	0	0
0	0	0	0	0	0	0	0	0	0	17	0	0	0	0	0	0	0	0
0	0	0	0	0	0	0	0	0	0	0	0	0	0	0	0	0	0	0
0	0	0	0	0	0	0	0	0	0	0	0	0	0	0	0	0	0	0
0	0	0	0	0	0	0	0	0	0	0	0	0	0	0	0	0	0	0
0	0	5	0	0	0	0	0	0	0	0	0	0	0	0	0	0	0	0
0	0	0	0	0	0	0	0	0	0	0	0	0	0	0	0	0	0	0
0	0	0	0	0	0	0	0	0	0	0	0	0	0	0	0	0	0	0
0	0	0	0	0	0	0	0	0	0	0	0	0	0	0	0	0	3	0
0	0	0	0	0	0	0	0	0	0	0	0	0	0	0	0	0	0	0
0	0	0	0	0	0	0	0	0	0	0	0	0	0	0	0	0	0	0
0	0	0	0	0	0	4	0	0	0	0	7	0	0	0	0	0	0	0
0	0	0	0	0	0	0	0	0	0	0	0	0	0	0	0	0	0	0
0	0	0	0	0	0	0	0	0	0	0	0	0	0	0	0	0	0	0
0	0	0	0	0	0	0	0	0	0	0	0	0	0	0	0	0	0	0
0	0	0	0	0	0	0	0	0	0	0	0	0	0	0	0	0	0	0
0	0	0	0	0	0	0	0	0	0	0	0	3	0	23	0	0	0	0
0	0	4	0	0	0	0	0	0	0	0	0	0	0	0	0	0	0	0
0	0	0	0	0	0	0	0	0	0	0	0	0	0	0	0	0	0	0
0	0	0	0	0	0	0	0	0	0	0	0	0	0	0	0	0	0	0
0	0	0	0	0	0	0	0	0	0	0	0	0	0	0	0	0	0	0
0	0	0	0	0	0	0	0	0	0	0	0	0	0	0	0	0	0	0
0	0	0	0	0	0	0	0	0	0	0	0	0	0	0	0	0	0	0

0	0	0	0	0	0	0	0	0	0	0	0	0	0	0	0	0	0	0
0	0	0	0	0	0	0	0	0	0	0	0	0	0	0	0	0	0	0
0	0	0	0	0	0	0	0	0	0	0	0	0	0	0	0	0	0	0
0	0	0	0	0	0	0	0	0	0	0	0	0	0	0	0	0	0	0
0	0	0	0	0	0	0	0	0	0	0	0	0	0	0	0	0	0	0
0	0	0	0	0	0	0	0	0	0	0	0	0	0	0	0	0	0	0
0	0	0	0	0	0	0	0	0	0	0	0	0	0	0	0	0	0	0
0	0	0	0	0	0	0	0	0	0	0	0	0	0	0	0	0	0	0
0	0	0	0	0	0	0	0	0	0	0	0	0	0	0	0	0	0	0
0	0	0	0	0	0	0	0	0	0	0	0	0	0	0	0	0	0	0
0	0	0	0	0	0	0	0	0	0	0	0	0	0	0	0	0	0	0
0	0	0	0	0	0	0	0	0	0	0	0	0	0	0	0	0	0	0
0	0	0	0	0	0	0	0	0	0	0	0	0	0	0	0	0	0	0
0	0	0	0	0	0	0	0	0	0	0	0	0	0	0	0	0	0	0
0	0	0	0	0	0	0	0	0	0	0	0	0	0	0	0	0	0	0
0	0	0	0	0	0	0	0	0	0	0	0	0	0	0	0	0	0	0
0	0	0	0	0	0	0	0	0	0	0	0	0	0	0	0	0	0	0
0	0	0	0	0	0	0	0	0	0	0	0	0	0	0	0	0	0	0
0	0	0	0	0	0	0	0	0	0	0	0	0	0	0	0	0	0	0
0	0	0	0	0	0	0	0	0	0	0	0	0	0	0	0	0	0	0
0	0	0	0	0	0	0	0	0	0	0	0	0	0	0	0	0	0	0
0	0	0	0	0	0	9	0	0	0	0	0	0	0	0	0	0	0	0
0	0	0	0	0	0	0	0	0	0	0	0	0	0	0	0	0	0	0
0	0	0	0	0	0	0	0	0	0	0	0	0	0	0	0	0	0	0
0	0	0	0	0	0	0	0	0	0	0	0	0	0	0	0	0	0	0
0	0	0	0	0	0	0	0	0	0	0	0	0	0	0	0	0	0	0
0	0	0	0	0	0	0	0	0	0	0	4	0	0	0	0	0	0	0
0	0	0	0	0	0	0	0	0	0	0	0	0	0	0	0	0	0	0
0	0	0	0	0	0	0	0	0	0	0	0	0	0	0	0	0	0	0
0	0	0	0	0	0	0	0	0	0	0	0	0	0	0	0	0	0	0
0	0	0	0	0	0	0	0	0	0	0	0	0	0	0	0	0	0	0
0	0	0	0	0	0	0	0	0	0	0	0	0	0	0	0	0	0	0
0	0	0	7	0	0	0	0	0	0	0	0	0	0	0	0	0	0	0
0	0	0	0	0	0	0	0	0	0	0	0	0	0	0	0	0	0	0
0	0	0	0	0	0	0	0	0	0	0	0	0	0	0	0	0	0	0
0	0	0	0	0	0	0	0	0	0	0	0	0	0	0	0	0	0	0
0	0	0	0	0	0	0	0	0	0	0	0	0	0	0	0	0	0	0

0	0	0	0	0	0	0	0	0	0	0	0	0	0	0	0	0	0	0
0	0	0	0	0	0	0	0	0	0	0	0	0	0	0	0	0	0	0
0	0	0	0	0	0	0	0	0	0	0	0	0	0	0	0	0	0	0
0	0	0	0	0	0	0	0	0	0	0	0	0	0	0	0	0	0	0
0	0	0	0	0	0	0	0	0	0	0	0	0	0	0	0	0	0	0
0	0	0	0	0	0	0	0	0	0	0	0	0	0	0	0	0	0	0
0	0	0	0	0	0	0	0	0	0	0	0	0	0	0	0	0	0	0
0	0	0	0	0	0	0	0	0	0	0	0	0	0	0	0	0	0	0
0	0	0	0	0	0	0	0	0	0	0	0	0	0	0	0	0	0	0
0	0	0	0	0	0	0	0	0	0	0	0	0	0	0	0	0	0	0
0	0	0	0	0	0	0	0	0	0	0	0	0	0	0	0	0	0	0
0	0	0	0	0	0	0	0	0	0	0	0	0	0	0	0	0	0	0
0	0	0	0	0	0	0	0	0	0	0	0	0	0	0	0	0	0	0
0	0	0	0	0	0	0	0	0	0	0	0	0	0	0	0	0	0	0
0	0	0	0	0	0	0	0	0	0	0	0	0	0	0	0	0	0	0
0	0	0	0	0	0	0	0	0	0	0	0	0	0	0	0	0	0	0
0	0	0	0	0	0	0	0	0	0	0	0	0	0	0	0	0	0	0
0	0	0	0	0	0	0	0	0	0	0	0	0	0	0	0	0	0	0
0	0	0	0	0	0	0	0	0	0	0	0	0	0	0	0	0	0	0
0	0	0	0	0	0	0	0	0	0	0	0	0	0	0	0	0	0	0
0	0	0	0	0	0	0	0	0	0	0	0	0	0	0	0	0	0	0
0	0	0	0	0	0	0	0	0	0	0	0	0	0	0	0	0	0	0
0	0	0	0	0	0	0	0	0	0	0	0	0	0	0	0	0	0	0
0	0	0	0	0	0	0	0	0	0	0	0	0	0	0	0	0	0	0
0	0	0	4	0	0	0	0	0	0	0	0	0	0	0	0	0	0	0
0	0	0	0	0	0	0	0	0	0	0	0	0	0	0	0	0	0	0
0	0	0	0	0	0	0	0	0	0	0	0	0	0	0	0	0	0	0
0	0	0	0	0	0	0	0	0	0	0	0	0	0	0	0	0	0	0
0	0	0	0	0	0	0	0	0	0	0	0	0	0	0	0	0	0	0
0	0	0	0	0	0	0	0	0	0	0	0	0	0	0	0	0	0	0
0	0	0	0	0	0	0	0	0	0	0	0	0	0	0	0	0	0	0
0	0	0	0	0	0	0	0	0	0	0	0	0	0	0	0	0	0	0
0	0	0	0	0	0	0	0	0	0	0	0	0	0	0	0	0	0	0
0	0	0	0	0	0	0	0	0	0	0	0	0	0	0	0	0	0	0
0	0	0	0	0	0	0	0	0	0	0	0	0	0	0	0	0	0	0
0	0	0	0	0	0	0	0	0	0	0	0	0	0	0	0	0	0	0
0	0	0	0	0	0	0	0	0	0	0	0	0	0	0	0	0	0	0

0	0	0	0	0	0	0	0	0	0	0	0	0	0	0	0	0	0	0
0	0	0	0	0	0	0	0	0	0	0	0	0	0	0	0	0	0	0
0	0	0	0	0	0	0	0	0	0	0	0	0	0	0	0	0	0	0
0	0	0	0	0	0	0	0	0	0	0	0	0	0	0	0	0	0	0
0	0	0	0	0	0	0	0	0	0	0	0	0	0	0	0	0	0	0
0	0	0	0	0	0	0	0	0	0	0	0	0	0	0	0	0	0	0
0	0	0	0	0	0	0	0	0	0	0	0	0	0	0	0	0	0	0
0	0	0	0	0	0	0	0	0	0	0	0	3	0	0	0	0	0	0
0	0	0	0	0	0	0	0	0	0	0	0	0	0	0	0	0	0	0
0	0	0	0	0	0	0	0	0	0	0	0	0	0	0	0	0	0	0
0	0	0	0	0	0	0	0	0	0	0	0	0	0	0	0	0	0	0
0	0	0	0	0	0	0	0	0	0	0	0	0	0	0	0	0	0	0
0	0	0	0	0	0	0	0	0	0	0	0	0	0	0	0	0	0	0
0	0	0	0	0	0	0	0	0	0	0	0	0	0	0	0	0	0	0
0	0	0	0	0	0	0	0	0	0	0	0	0	0	0	0	0	0	0
0	0	0	0	0	0	0	0	0	0	0	0	0	0	0	0	0	0	0
0	0	0	0	0	0	0	0	0	0	0	0	0	0	0	0	0	0	0
0	0	0	0	0	0	0	0	0	0	0	0	0	0	0	0	0	0	0
0	0	0	0	0	0	0	0	0	0	0	0	0	0	0	0	0	0	0
0	0	0	0	0	0	0	0	0	0	0	0	0	0	0	0	0	0	0
0	0	0	0	0	0	0	0	0	0	0	0	0	0	0	0	0	0	0
0	0	0	0	0	0	0	0	0	0	0	0	0	0	0	0	0	0	0
0	0	0	0	0	0	0	0	0	0	0	0	0	0	0	0	0	0	0
0	0	0	0	0	0	0	0	0	0	0	0	0	0	0	0	0	0	0
0	0	0	0	0	0	0	0	0	0	0	0	0	0	0	0	0	0	0
0	0	0	0	0	0	0	0	0	0	0	0	0	0	0	0	0	0	0
0	0	0	0	0	0	0	0	0	0	0	0	0	0	0	0	0	0	0
0	0	0	0	0	0	0	0	0	0	0	0	0	0	0	0	0	0	0
0	0	0	0	0	0	0	2	2	2	2	2	2	0	0	0	0	0	0
0	0	0	0	0	0	0	0	0	0	0	0	0	0	0	0	0	0	0
0	0	0	0	0	0	0	0	0	0	0	0	0	0	0	0	0	0	0
0	0	0	0	0	0	0	0	0	0	0	0	0	0	0	0	0	0	0
0	0	0	0	0	0	0	0	0	0	0	0	0	0	0	0	0	0	0
0	0	0	0	0	0	0	0	0	0									
Jan_DL015	1770	1581	614	228	427	161	187	144	40	333	170	446	263	69	383	850	89	183
201	397	310	0	79	254	227	137	229	66	144	0	191	192	116	116	104	0	0
0	92	0	32	76	87	80	16	21	390	61	59	91	70	0	43	0	72	0

0	163	58	72	72	116	21	28	110	21	36	69	485	0	26	0	8	58	0
36	55	0	26	18	33	57	0	112	76	0	110	12	2	45	0	71	11	0
30	7	0	234	73	0	0	61	57	0	0	0	0	0	30	27	0	0	0
0	17	0	14	83	0	0	0	0	0	22	14	7	0	0	60	0	13	130
0	51	0	0	133	32	0	0	0	8	0	0	11	77	27	40	0	0	0
0	20	29	0	0	0	0	0	88	0	0	0	15	0	0	0	0	0	33
0	0	9	0	0	0	0	0	0	0	57	0	0	21	0	0	31	0	33
11	0	0	89	0	19	0	0	0	22	0	0	0	59	0	0	0	0	0
20	0	5	0	0	56	16	0	0	0	92	0	0	0	0	0	0	0	0
0	0	0	33	0	24	0	0	0	0	0	11	12	10	0	22	7	0	0
0	0	0	3	0	0	0	0	0	8	0	0	15	0	0	0	22	0	0
0	0	0	0	0	8	0	0	0	3	6	0	0	0	0	0	0	0	0
0	0	0	0	0	0	0	0	0	0	0	30	17	0	11	0	0	0	0
0	7	0	0	0	0	0	0	0	0	0	0	0	0	0	5	84	5	7
0	0	7	0	0	0	0	0	0	30	0	0	0	0	0	0	64	0	0
5	0	0	22	0	0	0	0	10	31	0	0	0	0	0	94	0	0	0
0	0	0	0	0	0	9	0	0	0	0	0	63	0	0	0	0	0	0
10	28	0	0	0	9	0	0	8	0	0	0	0	0	0	0	0	6	0
0	0	39	13	0	45	0	2	0	35	0	15	0	0	0	0	0	0	0
0	0	0	0	0	0	0	0	0	0	0	0	0	0	0	0	0	0	0
0	0	0	0	0	0	0	0	0	0	0	0	0	0	0	0	0	7	0
0	0	0	0	17	0	0	0	0	0	0	0	0	0	0	29	0	0	0
0	0	0	0	6	0	0	0	0	15	0	0	0	0	24	0	0	15	3
0	0	0	0	0	0	0	0	0	0	0	0	0	0	0	0	0	0	0
35	0	0	0	0	0	5	0	0	0	0	0	0	0	0	0	0	0	0
0	0	0	0	0	0	0	25	0	0	0	0	0	0	0	0	0	0	0
0	0	8	0	17	0	0	0	0	0	5	0	0	0	0	7	0	0	0
0	0	0	0	0	0	0	0	0	0	0	0	0	0	0	0	0	0	0
0	0	0	0	0	0	0	0	0	0	0	0	0	0	0	0	0	0	0
0	0	0	0	0	8	0	0	0	0	0	0	0	0	0	32	0	0	0
0	0	0	0	0	0	0	0	0	0	6	0	0	0	0	8	0	0	0
0	0	0	0	0	0	0	0	0	5	0	0	0	0	0	0	0	7	0
0	0	0	0	0	0	0	0	0	0	0	0	0	0	0	0	0	0	0
0	0	0	0	0	0	0	0	0	0	0	0	0	0	0	0	0	0	0
0	0	0	0	17	14	0	0	0	0	26	0	0	0	0	0	0	0	0
0	0	0	0	0	0	0	0	0	0	0	0	5	0	0	0	0	0	0
0	0	0	0	0	0	0	0	0	10	0	0	0	0	0	0	0	0	0

0	0	0	0	0	0	0	0	0	0	0	0	0	0	0	0	0	0	0
0	0	0	0	0	41	0	0	0	0	4	0	0	8	0	0	0	0	6
0	4	0	0	0	0	0	0	0	0	0	0	0	0	0	0	0	0	0
0	0	0	0	0	0	0	0	4	0	0	0	15	0	0	0	0	0	0
0	0	0	0	0	0	0	0	0	0	0	0	0	0	0	0	0	0	0
0	0	0	0	0	0	0	0	0	0	0	0	0	0	0	0	0	0	0
0	0	0	0	0	0	0	0	0	0	0	0	0	0	0	0	0	0	0
0	0	0	0	0	0	0	0	0	0	0	0	0	0	0	0	0	0	0
0	0	0	0	0	5	0	0	0	0	0	0	0	0	0	4	0	0	0
0	0	0	0	0	0	0	0	0	0	0	0	0	0	0	0	0	0	0
0	0	0	0	0	0	0	0	0	0	0	0	0	0	0	0	0	0	0
0	0	0	0	0	0	0	0	0	0	0	0	0	0	0	0	0	0	0
0	4	0	0	0	15	0	0	0	0	0	0	0	0	0	0	0	0	0
0	0	0	0	0	0	0	0	0	0	7	0	0	0	0	0	0	0	0
0	0	0	0	0	0	0	0	0	0	0	0	0	0	0	0	0	0	0
0	0	0	0	0	0	0	0	0	0	0	0	0	0	0	0	0	0	0
0	0	0	0	0	0	0	0	0	0	0	0	0	0	0	0	0	0	0
0	0	0	0	0	0	0	0	0	0	0	0	0	0	0	0	0	0	0
0	0	0	0	0	0	0	0	0	0	0	0	0	0	0	0	0	0	0
0	0	0	0	0	0	0	0	0	0	0	0	0	0	0	0	0	0	0
0	0	0	0	0	0	0	0	0	0	0	0	0	0	0	0	0	0	0
0	0	0	0	0	0	0	0	0	0	0	0	0	0	0	0	0	0	0
0	0	0	0	0	0	0	0	0	7	0	0	0	0	0	0	0	0	0
0	0	0	0	0	0	0	0	0	0	0	0	0	0	0	0	0	0	0
0	0	0	0	0	0	0	0	0	0	0	0	0	0	0	0	0	0	0
0	0	0	0	0	0	0	0	0	0	0	0	0	0	0	0	0	0	0
0	0	0	0	0	0	0	0	0	0	0	0	0	0	0	0	0	0	0
0	0	0	0	0	0	0	0	0	0	0	4	0	0	0	0	0	0	0
0	0	0	0	0	0	0	0	0	0	0	0	0	0	0	0	0	0	0
0	0	0	0	0	0	0	0	0	0	0	0	0	0	0	0	0	0	0
0	0	0	0	0	0	0	0	0	0	0	0	0	0	0	0	0	0	0
0	0	0	0	0	0	0	0	0	0	0	0	0	0	0	0	0	0	0
0	0	0	0	0	0	0	0	0	0	0	0	0	0	0	0	0	0	0
0	0	0	0	0	0	0	0	0	0	0	0	0	0	0	0	0	0	0
0	0	0	0	0	0	0	0	0	0	0	0	0	0	0	0	0	0	0
0	0	0	0	0	0	0	0	0	0	0	0	0	0	0	0	0	0	0
0	0	0	0	0	0	0	0	0	0	0	0	0	0	0	0	0	0	0

0	0	0	0	0	0	0	0	0	0	0	0	0	0	0	0	0	0	0
0	0	0	0	0	0	0	0	0	0	0	0	0	0	0	0	0	0	0
0	0	0	0	0	0	0	0	0	0	0	0	0	0	0	0	0	0	0
0	0	0	0	0	0	0	0	0	0	0	0	0	0	0	0	0	0	0
0	0	0	0	0	0	0	0	0	0	0	0	0	0	0	0	0	0	0
0	0	0	0	0	0	0	0	0	0	0	0	0	0	0	0	0	0	0
0	0	0	0	0	0	0	0	0	0	0	0	0	0	0	0	0	0	0
0	0	0	0	0	0	0	0	0	0	0	0	0	0	0	0	0	0	0
0	0	0	0	0	0	0	0	0	0	0	0	0	0	0	0	0	0	0
0	0	0	0	0	0	0	0	0	0	0	0	0	0	0	0	0	0	0
0	0	0	0	0	0	0	0	0	0	0	0	0	0	0	0	3	0	0
0	0	0	0	0	0	0	0	0	0	0	0	0	0	0	0	0	0	0
0	0	0	0	0	0	0	0	0	0	0	0	0	0	0	0	0	0	0
0	0	0	0	0	0	0	0	0	0	0	0	0	0	0	0	0	0	0
0	0	0	0	0	0	0	0	0	0	0	0	0	0	0	0	0	0	0
0	0	0	0	0	0	0	0	0	0	0	0	0	0	0	0	0	0	0
0	0	0	0	0	0	0	0	0	0	0	0	0	0	0	0	0	0	0
0	0	0	0	0	0	0	0	0	0	0	0	0	0	0	0	0	0	0
0	0	0	0	0	0	0	0	0	0	0	0	0	0	0	0	0	0	0
0	0	0	0	0	0	0	0	0	0	0	0	0	0	0	0	0	0	0
0	0	0	0	0	0	0	0	0	0	0	0	0	0	0	0	0	0	0
0	0	0	0	0	6	0	0	0	0	0	0	0	0	0	0	0	0	0
0	0	0	0	0	0	0	0	0	0	0	0	0	0	0	0	0	0	0
0	0	0	0	0	0	0	0	0	0	0	0	0	0	0	0	0	0	0
0	0	0	0	0	0	0	0	0	0	0	0	0	0	0	0	0	0	0
0	0	0	0	0	0	0	0	0	0	0	0	0	0	0	0	0	0	0
0	0	0	0	0	0	0	0	0	0	0	0	0	0	0	0	0	0	0
0	0	0	0	0	0	0	0	0	0	0	0	0	0	0	0	0	0	0
0	0	0	0	0	0	0	0	0	0	0	0	0	0	0	0	0	0	0
0	0	0	0	0	0	0	0	0	0	0	0	0	0	0	0	0	0	0
0	0	0	0	0	0	0	0	0	0	0	0	0	0	0	0	0	0	0
0	0	0	0	0	0	0	0	0	0	0	0	0	0	0	0	0	0	0
0	0	0	0	0	0	0	0	0	0	0	0	0	0	0	0	0	0	0
0	0	0	0	0	0	0	0	0	0	0	0	0	0	0	0	0	0	0
0	0	0	0	0	0	0	0	0	0	0	0	0	0	0	0	0	0	0
0	0	0	0	0	0	0	0	0	0	0	0	0	0	0	0	0	0	0
0	0	0	0	0	0	0	0	0	0	0	0	0	0	0	0	0	0	0

0	0	0	0	0	0	0	0	0	0	0	0	0	0	0	0	0	0	0
0	0	0	0	0	0	0	0	0	0	0	0	0	0	0	0	0	0	0
0	0	0	0	0	0	0	0	0	0	0	0	0	0	0	0	0	0	0
0	0	0	0	4	0	0	0	0	0	0	0	0	0	0	0	0	0	0
0	0	0	0	0	0	0	0	0	0	0	0	0	0	0	0	0	0	0
0	0	0	0	0	0	0	0	0	0	0	0	0	0	0	0	0	0	0
0	0	0	0	0	0	0	0	0	0	0	0	0	0	0	0	0	0	0
0	0	0	0	0	0	0	0	0	0	0	0	0	0	0	0	0	0	0
0	0	0	0	0	0	0	0	0	0	0	0	0	0	0	0	0	0	0
0	0	0	0	0	0	0	0	0	0	0	0	0	0	0	0	0	0	0
0	0	0	0	0	0	0	0	0	0	0	0	0	0	0	0	0	0	0
0	0	0	0	0	0	0	0	0	0	0	0	0	0	0	0	0	0	0
0	0	0	0	0	0	0	0	0	0	0	0	0	0	0	0	0	0	0
0	0	0	0	0	0	0	0	0	0	0	0	0	0	0	0	0	0	0
0	0	0	0	0	0	0	0	0	0	0	0	0	0	0	0	0	0	0
0	0	0	0	0	0	0	0	0	0	0	0	0	0	0	0	0	0	0
0	0	0	0	0	0	0	0	0	0	0	0	0	0	0	0	0	0	0
0	0	0	0	0	0	0	0	0	0	0	0	0	0	0	0	0	0	0
0	0	0	0	0	0	0	0	0	0	0	0	0	0	0	0	0	0	0
0	0	0	0	0	0	0	3	3	0	0	0	0	0	0	0	0	0	0
0	0	0	0	0	0	0	0	0	0	0	0	0	0	0	0	0	0	0
0	0	0	0	0	0	0	0	0	0	0	0	0	0	0	0	0	0	0
0	0	0	0	0	0	0	0	0	0	0	0	0	0	0	0	0	0	0
0	0	0	0	0	0	0	0	0	0	0	0	0	0	0	0	0	0	0
0	0	0	0	0	0	0	0	0	0	0	0	0	0	0	0	0	0	0
0	0	0	0	0	0	0	0	0	0	0	0	0	0	0	0	0	0	0
0	0	0	0	0	0	0	0	0	0	0	0	0	0	0	0	0	0	0
0	0	0	0	0	0	0	0	0	0	0	0	0	0	0	0	0	0	0
0	0	0	0	0	0	0	0	0	0	0	0	0	0	0	0	0	0	0
0	0	0	0	0	0	0	0	0	0	0	0	0	0	0	0	0	0	0
0	0	0	0	0	0	0	0	0	0	0	0	0	0	0	0	0	0	0
0	0	0	0	0	0	0	0	0	0	0	0	0	0	0	0	0	0	0
0	0	0	0	0	0	0	0	0	0	0	0	0	0	0	0	0	0	0
0	0	0	0	0	0	0	0	0	0	0	0	0	0	0	0	0	0	0
0	0	0	0	0	0	0	0	0	0	0	0	0	0	0	0	0	0	0
0	0	0	0	0	0	0	0	0	0	0	0	0	0	0	0	0	0	0
0	0	0	0	0	0	0	0	0	0	0	0	0	0	0	0	0	0	0

0	0	0	0	0	0	0	0	0	0	0	0	0	0	0	0	0	0	0
0	0	0	0	0	0	0	0	0	0	0	0	0	0	0	0	0	0	0
0	0	0	0	0	0	0	0	0	0	0	0	0	0	0	0	0	0	0
0	0	0	0	0	0	0	0	0	0	0	0	0	0	0	0	0	0	0
0	0	0	0	0	0	0	0	0	0	0	0	0	0	0	0	0	0	0
0	0	0	0	0	0	0	0	0	0	0	0	0	0	0	0	0	0	0
0	0	0	0	0	0	0	0	0	0	0	0	0	0	0	0	0	0	0
0	0	0	0	0	0	0	0	0	0	0	0	0	0	0	0	0	0	0
0	0	0	0	0	0	0	0	0	0	0	0	0	0	0	0	0	0	0
0	0	0	0	0	0	0	0	0	0	0	0	0	0	0	0	0	0	0
0	0	0	2	2	0	0	0	0	0	0	0	0	0	0	0	0	0	0
0	0	0	0	0	0	0	0	0	0	0	0	0	0	0	0	0	0	0
0	0	0	0	0	0	0	0	0	0	0	0	0	0	0	0	0	0	0
0	0	0	0	0	0	0	0	0	0	0	0	0	0	0	0	0	0	0
0	0	0	0	0	0	0	0	0	0	0	0	0	0	0	0	0	0	0
0	0	0	0	0	0	0	0	0	0	0	0	0	0	0	0	0	0	0
0	0	0	0	0	0	0	0	0	0	0	0	0	0	0	0	0	0	0
0	0	0	0	0	0	0	0	0	0	0	0	0	0	0	0	0	0	0
0	0	0	0	0	0	0	0	0	0	0	0	0	0	0	0	0	0	0
0	0	0	0	0	0	0	0	0	0	0	0	0	0	0	0	0	0	0
0	0	0	0	0	0	0	0	0	0	0	0	0	0	0	0	0	0	0
0	0	0	0	0	0	0	0	0	0	0	0	0	0	0	0	0	0	0
0	0	0	0	0	0	0	0	0	0	0	0	0	0	0	0	0	0	0
0	0	0	0	0	0	0	0	0	0	0	0	0	0	0	0	0	0	0
0	0	0	0	0	0	0	0	0	0	0	0	0	0	0	0	0	0	0
0	0	0	0	0	0	0	0	0	0									
Jan_DL150	841	492	462	263	510	542	68	65	478	199	40	177	182	141	204	187	84	127
147	349	51	82	65	55	55	73	77	0	21	0	86	50	90	0	63	155	0
63	29	19	227	62	0	57	40	18	55	0	67	33	83	143	42	43	26	0
0	40	61	33	26	95	32	28	13	16	0	64	3	0	109	0	8	31	12
0	34	93	21	0	36	26	77	0	31	57	0	70	0	27	0	84	43	0
0	82	0	128	0	0	0	60	0	43	0	0	0	11	22	0	0	0	0
0	76	0	39	0	0	0	0	0	62	0	18	13	0	32	0	0	0	94
60	65	0	0	0	0	0	0	0	8	0	0	0	0	0	0	0	0	0
0	0	28	5	61	0	0	0	0	0	6	0	18	0	0	0	0	0	0
0	0	8	0	0	0	0	14	0	0	23	16	0	0	0	0	12	0	16
0	0	10	0	0	0	0	0	0	0	0	0	0	0	0	0	0	0	0

14	6	16	0	0	0	0	0	0	0	53	0	0	0	0	0	0	4	0
0	0	0	0	0	0	0	0	0	0	0	8	0	12	0	0	15	0	0
0	0	0	0	39	0	0	0	0	0	0	0	0	0	0	0	0	10	0
10	0	31	10	0	0	11	0	19	0	0	0	0	0	0	0	0	0	0
0	0	0	0	0	0	0	0	0	0	0	0	15	0	0	8	0	0	14
0	0	11	0	0	0	0	8	0	0	18	0	0	5	0	11	0	0	5
0	0	0	0	0	0	0	0	6	0	0	0	0	0	0	8	0	0	9
0	21	44	0	0	0	0	0	8	3	0	7	0	0	0	0	0	0	6
0	11	0	0	0	0	0	0	0	10	0	0	7	0	0	0	0	0	0
0	0	0	0	0	5	0	0	3	0	0	0	0	0	0	0	0	0	0
0	0	0	0	0	0	0	3	0	0	0	0	0	19	0	0	8	0	0
0	0	0	0	0	0	0	0	5	0	0	7	0	0	0	0	0	0	0
0	6	0	0	0	0	0	0	0	0	0	0	0	0	0	0	0	0	0
0	0	0	0	0	0	0	0	0	0	0	0	0	0	0	0	0	0	0
0	0	0	0	0	0	0	0	0	0	0	0	0	0	0	0	0	0	24
0	0	0	0	0	0	0	0	0	0	0	0	0	0	0	0	0	0	0
0	0	17	0	0	0	0	18	0	0	0	0	0	0	0	0	10	0	0
0	0	0	0	0	0	0	13	12	0	0	0	0	0	7	0	0	0	0
0	0	0	0	0	0	0	0	0	0	0	0	0	0	0	0	0	0	0
0	0	0	0	0	0	0	0	0	0	0	0	0	0	0	0	0	0	0
20	0	0	0	0	0	0	0	0	0	0	0	0	0	0	7	0	0	0
0	0	0	0	0	0	0	0	0	0	0	0	0	0	0	0	0	0	0
6	0	0	0	0	0	0	0	0	0	0	0	0	0	0	12	0	0	0
0	0	0	0	0	0	0	0	0	0	0	5	0	0	0	10	0	0	0
6	0	11	0	0	0	0	0	0	0	0	0	0	0	0	0	0	0	0
0	0	0	0	0	0	0	0	0	0	0	0	0	0	0	0	0	0	0
0	0	0	0	0	0	0	0	0	6	0	0	0	0	0	0	0	0	0
0	0	0	0	0	0	0	0	0	0	0	0	24	0	0	0	0	0	0
0	0	0	0	0	0	0	0	0	0	0	0	0	0	0	0	0	0	0
0	0	0	0	0	0	0	0	0	0	0	0	0	0	0	0	0	4	0
0	0	0	0	0	0	0	0	0	0	0	0	0	0	3	0	0	0	0
0	0	0	0	0	0	0	0	0	0	0	0	0	0	0	0	0	0	0
0	0	0	0	0	0	0	0	0	0	0	0	0	0	0	0	0	0	0
0	0	0	0	0	0	0	0	0	0	0	0	0	0	0	0	0	0	0
0	0	0	0	0	0	0	0	0	0	0	0	0	0	0	4	0	0	0
0	0	0	0	0	0	0	0	0	0	0	0	0	0	0	0	0	0	0
0	0	0	0	0	0	0	0	0	0	0	0	0	0	0	0	0	0	0

0	0	0	0	0	0	0	0	0	0	0	0	0	0	0	0	0	0	0
0	0	0	0	0	0	0	0	0	0	0	0	0	0	0	0	0	0	0
0	0	0	0	0	0	0	0	0	0	0	0	0	21	0	0	0	0	0
0	0	0	0	0	0	0	0	0	0	0	0	0	0	0	0	0	0	0
0	0	0	0	0	0	0	0	0	0	0	0	0	0	0	0	0	0	0
0	0	0	0	0	0	3	0	0	0	0	0	0	0	0	0	0	0	0
0	0	0	0	0	0	0	0	0	0	0	0	0	0	0	0	0	0	0
0	0	0	0	0	0	0	0	0	0	0	0	0	0	0	0	0	0	0
0	0	0	0	0	0	0	0	0	0	0	0	0	0	0	0	0	0	0
0	0	0	0	0	7	0	0	0	0	0	0	0	0	0	0	0	0	0
0	0	0	0	0	0	0	0	0	0	0	0	0	0	0	0	0	0	0
0	0	0	0	0	0	0	0	0	0	0	0	0	0	0	0	0	0	0
0	0	0	0	0	0	0	0	0	0	0	0	0	0	0	0	0	0	0
0	0	0	0	0	0	0	0	0	0	0	0	0	0	0	0	0	0	0
0	0	0	0	0	0	0	0	0	0	0	0	0	0	0	0	0	0	0
0	0	0	0	0	0	0	0	0	0	0	0	0	0	0	0	0	0	0
0	0	0	0	0	0	0	0	0	0	0	0	0	0	0	0	0	0	0
0	0	0	0	0	0	0	0	0	0	0	0	0	0	0	0	0	0	0
0	0	0	0	0	0	0	0	0	0	0	0	0	0	0	0	0	0	0
0	0	0	0	0	0	0	0	0	0	0	0	0	0	0	0	0	0	0
0	0	0	0	0	0	0	0	0	0	0	0	0	0	0	0	0	0	4
0	0	0	0	0	0	0	0	0	0	0	0	0	0	0	0	0	0	0
0	0	0	0	0	0	0	0	0	0	0	0	0	0	0	0	0	0	0
0	0	0	0	0	0	0	0	0	0	0	0	0	0	0	0	0	0	0
0	0	0	0	0	0	0	0	0	0	0	0	0	0	0	0	0	0	0
0	0	0	0	0	0	0	0	0	0	0	0	0	0	0	0	0	0	0
5	0	0	0	0	0	0	0	0	0	0	0	0	0	0	0	0	0	0
0	0	0	0	0	0	0	0	0	0	0	0	0	0	0	0	0	0	0
0	0	0	0	0	0	0	0	0	0	0	0	0	0	0	0	0	0	0
0	0	0	0	0	0	0	0	0	0	0	0	0	0	0	0	0	0	0
0	0	0	0	0	0	0	0	0	0	0	0	0	0	0	0	0	0	0
0	0	0	0	0	0	0	0	0	6	0	0	0	0	0	0	0	0	0
0	0	0	0	0	0	0	0	0	0	0	0	0	0	0	0	0	0	0
0	0	0	0	0	0	0	0	0	0	0	0	0	0	0	0	0	0	0
0	0	0	0	0	0	0	0	0	0	0	0	0	0	0	0	0	0	0
0	0	0	0	0	0	0	0	0	0	0	0	0	0	0	0	0	0	0
0	0	0	0	0	0	0	0	0	0	0	0	0	0	0	0	0	0	0

0	0	0	0	0	0	0	0	0	0	0	0	0	0	5	0	0	0	0
0	0	0	0	0	0	0	0	0	0	0	0	0	0	0	0	0	0	0
0	0	0	0	0	0	0	0	0	0	0	0	0	0	0	0	0	0	0
0	0	0	0	0	0	0	0	0	0	0	0	0	0	0	0	0	0	0
0	0	0	0	0	0	0	0	0	0	0	0	0	0	0	0	0	0	0
0	0	0	0	0	0	0	0	0	0	0	0	0	0	0	0	0	0	0
0	0	0	0	0	0	0	0	0	0	0	0	0	0	0	0	0	0	0
0	0	0	0	0	0	0	0	0	0	0	0	0	0	0	0	0	0	0
0	0	0	0	0	0	0	0	0	0	0	0	0	0	0	0	0	0	0
0	0	0	0	4	4	4	4	0	0	0	0	0	0	0	0	0	0	0
0	0	0	0	0	0	0	0	0	0	0	0	0	0	0	0	0	0	0
0	0	0	0	0	0	0	0	0	0	0	0	0	0	0	0	0	0	0
0	0	0	0	0	0	0	0	0	0	0	0	0	0	0	0	0	0	0
0	0	0	0	0	0	0	0	0	0	0	0	0	0	0	0	0	0	0
0	0	0	0	0	0	0	0	0	0	0	0	0	0	0	0	0	0	0
0	0	0	0	0	0	0	0	0	0	0	0	0	0	0	0	0	0	0
0	0	0	0	0	0	0	0	0	0	0	0	0	0	0	0	0	0	0
0	0	0	0	0	0	0	0	0	0	0	0	0	0	0	0	0	0	0
0	0	0	0	0	0	0	0	0	0	0	0	0	0	0	0	0	0	0
0	0	0	0	0	0	0	0	0	0	0	0	0	0	0	0	0	0	0
0	0	0	0	0	0	0	0	0	0	0	0	0	0	0	0	0	0	0
0	0	0	0	0	0	0	0	0	0	0	0	0	0	0	0	0	0	0
0	0	0	0	0	0	0	0	0	0	0	3	3	3	0	0	0	0	0
0	0	0	0	0	0	0	0	0	0	0	0	0	0	0	0	0	0	0
0	0	0	0	0	0	0	0	0	0	0	0	0	0	0	0	0	0	0
0	0	0	0	0	0	0	0	0	0	0	0	0	0	0	0	0	0	0
0	0	0	0	0	0	0	0	0	0	0	0	0	0	0	0	0	0	0
0	0	0	0	0	0	0	0	0	0	0	0	0	0	0	0	0	0	0
0	0	0	0	0	0	0	0	0	0	0	0	0	0	0	0	0	0	0
0	0	0	0	0	0	0	0	0	0	0	0	0	0	0	0	0	0	0
0	0	0	0	0	0	0	0	0	0	0	0	0	0	0	0	0	0	0
0	0	0	0	0	0	0	0	0	0	0	0	0	0	0	0	0	0	0
0	0	0	0	0	0	0	0	0	0	0	0	0	0	0	0	0	0	0
0	0	0	0	0	0	0	0	0	0	0	0	0	0	0	0	0	0	0
0	0	0	0	0	0	0	0	0	0	0	0	0	0	0	0	0	0	0
0	0	0	0	0	0	0	0	0	0	0	0	0	0	0	0	0	0	0
0	0	0	0	0	0	0	0	0	0	0	0	0	0	0	0	0	0	0

0	0	0	0	0	0	0	0	0	0	0	0	0	0	0	0	0	0	0
0	0	0	0	0	0	0	0	0	0	0	0	0	0	0	0	0	0	0
0	0	0	0	0	0	0	0	0	0	0	0	0	0	0	0	0	0	0
0	0	0	0	0	0	0	0	0	0	0	0	0	0	0	0	0	0	0
0	0	0	0	0	0	0	0	0	0	0	0	0	0	0	0	0	0	0
0	0	0	0	0	0	0	0	0	0	0	0	0	0	0	0	0	0	0
0	0	0	0	0	0	0	0	0	0	0	0	0	0	0	0	0	0	0
0	0	0	0	0	0	0	0	0	0	2	2	2	0	0	0	0	0	0
0	0	0	0	0	0	0	0	0	0	0	0	0	0	0	0	0	0	0
0	0	0	0	0	0	0	0	0	0	0	0	0	0	0	0	0	0	0
0	0	0	0	0	0	0	0	0	0	0	0	0	0	0	0	0	0	0
0	0	0	0	0	0	0	0	0	0	0	0	0	0	0	0	0	0	0
0	0	0	0	0	0	0	0	0	0	0	0	0	0	0	0	0	0	0
0	0	0	0	0	0	0	0	0	0	0	0	0	0	0	0	0	0	0
0	0	0	0	0	0	0	0	0	0	0	0	0	0	0	0	0	0	0
0	0	0	0	0	0	0	0	0	0	0	0	0	0	0	0	0	0	0
0	0	0	0	0	0	0	0	0	0	0	0	0	0	0	0	0	0	0
0	0	0	0	0	0	0	0	0	0	0	0	0	0	0	0	0	0	0
0	0	0	0	0	0	0	0	0	0	0	0	0	0	0	0	0	0	0
0	0	0	0	0	0	0	0	0	0	0	0	0	0	0	0	0	0	0
0	0	0	0	0	0	0	0	0	0	0	0	0	0	0	0	0	0	0
0	0	0	0	0	0	0	0	0	0	0	0	0	0	0	0	0	0	0
0	0	0	0	0	0	0	0	0	0	0	0	0	0	0	0	0	0	0
0	0	0	0	0	0	0	0	0	0	0	0	0	0	0	0	0	0	0
0	0	0	0	0	0	0	0	0	0	0	0	0	0	0	0	0	0	0
0	0	0	0	0	0	0	0	0	0	0	0	0	0	0	0	0	0	0
0	0	0	0	0	0	0	0	0	0	0	0	0	0	0	0	0	0	0
0	0	0	0	0	0	0	0	0	0	0	0	0	0	0	0	0	0	0
0	0	0	0	0	0	0	0	0	0	0	0	0	0	0	0	0	0	0
0	0	0	0	0	0	0	0	0	0	0	0	0	0	0	0	0	0	0
0	0	0	0	0	0	0	0	0	0	0	0	0	0	0	0	0	0	0
0	0	0	0	0	0	0	0	0	0	0	0	0	0	0	0	0	0	0
0	0	0	0	0	0	0	0	0	0	0	0	0	0	0	0	0	0	0
0	0	0	0	0	0	0	0	0	0	0	0	0	0	0	0	0	0	0
0	0	0	0	0	0	0	0	0	0	0	0	0	0	0	0	0	0	0
0	0	0	0	0	0	0	0	0	0	0	0	0	0	0	0	0	0	0
0	0	0	0	0	0	0	0	0	0	0	0	0	0	0	0	0	0	0

	0	0	0	0	0	0	0	0	0	0	0	0	0	0	0	0	0	0	0
	0	0	0	0	0	0	0	0	0	0	0	0	0	0	0	0	0	0	0
	0	0	0	0	0	0	0	0	0	0	0	0	0	0	0	0	0	0	0
	0	0	0	0	0	0	0	0	0	0	0	0	0	0	0	0	0	0	0
	0	0	0	0	0	0	0	0	0	0	0	0	0	0	0	0	0	0	0
	0	0	0	0	0	0	0	0	0	0	0	0	0	0	0	0	0	0	0
	0	0	0	0	0	0	0	0	0	0	0	0	0	0	0	0	0	0	0
	0	0	0	0	0	0	0	0	0	0	0	0	0	0	0	0	0	0	0
	0	0	0	0	0	0	0	0	0	0	0	0	0	0	0	0	0	0	0
	0	0	0	0	0	0	0	0	0	0	0	0	0	0	0	0	0	0	0
	0	0	0	0	0	0	0	0	0	0	0	0	0	0	0	0	0	0	0
	0	0	0	0	0	0	0	0	0	0	0	0	0	0	0	0	0	1	0
	0	0	0	0	0	0	0	0	0	0	0	0	0	0	0	0	0	0	0
	0	0	0	0	0	0	0	0	0	0	0	0	0	0	0	0	0	0	0
	0	0	0	0	0	0	0	0	0	0	0	0	0	0	0	0	0	0	0
	0	0	0	0	0	0	0	0	0	0	0	0	0	0	0	0	0	0	0
	0	0	0	0	0	0	0	0	0	0	0	0	0	0	0	0	0	0	0
	0	0	0	0	0	0	0	0	0	0									
Jan_DL	151	1561	1113	347	528	170	554	401	458	207	203	122	118	158	270	167	84	253	203
	148	55	109	79	323	105	36	82	0	202	129	515	99	55	88	0	65	123	471
	168	93	234	265	76	45	0	0	205	18	76	26	105	105	77	19	0	100	344
	248	11	0	59	73	164	156	126	0	73	49	0	0	152	0	38	11	12	227
	41	32	29	82	0	0	24	23	55	16	43	0	39	45	31	0	8	16	0
	36	35	0	0	0	0	0	0	0	42	0	0	0	16	9	0	11	0	0
	0	44	38	8	14	11	0	0	0	79	0	0	8	9	0	0	0	0	0
	99	32	0	0	13	0	0	45	0	6	0	0	12	0	37	0	0	0	0
	0	0	0	0	72	45	0	0	0	0	11	0	27	0	34	0	34	39	0
	0	0	0	0	0	0	0	13	0	0	0	0	81	0	0	27	11	0	0
	0	0	0	0	0	0	0	12	0	0	8	0	32	0	0	0	0	0	0
	0	0	0	0	16	0	0	0	0	0	0	0	0	0	0	0	0	24	0
	0	0	0	0	0	16	0	0	13	0	0	0	0	8	0	16	23	0	0
	0	0	0	9	0	0	0	0	0	6	0	0	0	6	0	0	0	0	0
	0	60	0	17	19	13	10	0	0	0	9	0	0	9	0	5	14	0	0
	0	16	8	0	0	0	0	0	0	0	0	0	15	0	0	0	0	0	29
	0	9	22	0	0	24	0	0	0	0	0	5	0	0	0	0	0	0	5
	9	0	0	0	0	0	0	0	0	0	0	0	0	0	0	0	0	0	0
	3	15	0	0	0	7	0	0	16	7	0	0	0	18	0	0	11	0	0

0	32	0	0	0	0	3	0	0	10	0	0	0	0	0	0	0	0	0
0	0	0	0	0	0	0	0	12	0	0	0	0	0	0	0	0	0	0
0	0	0	7	0	16	4	0	0	0	0	9	0	0	0	0	0	25	0
0	0	0	0	0	0	0	0	0	44	0	0	0	0	0	0	0	0	0
0	0	0	0	0	17	0	0	0	0	0	0	0	0	55	0	7	7	9
0	0	0	0	9	0	0	0	0	0	0	0	0	0	0	0	0	0	0
0	0	0	12	0	0	0	0	0	0	0	0	0	0	0	0	0	19	0
0	0	0	0	0	13	0	0	0	0	0	0	0	0	0	0	0	0	0
0	0	0	0	0	13	0	5	0	0	0	0	0	0	0	0	0	0	0
0	0	0	0	0	0	0	0	0	9	0	15	0	0	0	0	0	0	0
8	3	0	0	0	0	0	0	0	0	0	0	0	0	0	0	0	0	0
0	0	0	0	0	0	0	0	0	0	0	0	0	0	0	0	0	0	0
0	0	0	0	0	0	0	0	12	0	0	0	0	0	0	6	0	0	0
0	16	0	0	0	0	0	0	0	8	0	0	0	0	0	0	0	0	0
0	0	0	0	0	0	0	0	0	0	21	0	0	0	0	0	0	0	0
0	0	0	0	0	11	0	0	0	0	0	0	0	4	0	0	0	0	12
3	0	0	0	0	0	0	0	0	0	0	0	0	0	0	0	0	0	0
0	0	0	0	0	0	0	0	0	0	0	0	0	0	0	0	0	0	0
0	11	0	0	0	0	0	0	14	18	0	0	0	0	0	0	0	0	0
0	0	0	0	0	0	0	0	0	0	0	0	0	0	0	0	0	0	14
0	0	0	0	0	0	0	23	0	0	0	0	0	0	0	0	6	0	0
0	0	0	0	0	0	0	0	0	0	0	0	0	0	0	0	0	0	0
0	0	0	0	0	0	0	0	0	0	0	0	0	0	0	0	0	0	0
0	0	0	0	0	0	0	0	0	5	0	0	0	0	0	0	0	0	0
0	0	0	0	0	0	0	0	0	0	0	0	0	0	0	11	0	0	0
0	0	0	4	0	0	0	0	0	0	0	0	0	0	0	0	0	0	0
0	0	0	0	0	0	0	0	0	0	0	0	0	0	0	0	0	0	0
0	0	0	0	0	0	0	0	0	0	0	0	0	0	0	0	0	0	0
0	0	0	0	0	0	0	0	0	0	0	0	0	0	20	0	0	0	0
0	0	0	0	0	0	0	0	0	0	0	0	0	0	0	3	1	0	0
0	0	0	0	0	0	0	0	0	0	0	0	0	0	0	0	0	0	0
0	0	0	0	0	0	0	0	0	0	0	0	0	0	0	0	0	0	0
0	0	0	0	0	0	0	0	0	0	0	0	0	0	0	0	0	0	0
0	0	0	0	0	0	0	0	0	0	0	0	0	0	0	0	0	0	17
0	0	0	0	0	0	0	0	0	0	0	0	0	0	0	0	0	0	0
0	0	0	0	0	0	0	0	0	0	0	7	0	0	0	0	0	0	0
0	0	0	0	0	0	0	0	0	0	0	0	0	0	0	0	0	0	0

0	0	0	0	0	0	0	0	0	0	0	0	0	0	0	0	0	0	0
0	0	0	0	0	0	0	0	0	0	0	0	0	0	0	0	0	0	0
0	0	0	0	0	0	0	0	0	0	0	0	0	0	0	0	0	0	0
0	0	0	0	0	0	0	0	0	0	0	0	0	0	0	0	0	0	0
0	0	0	0	0	0	0	0	0	0	0	0	0	0	0	0	0	0	0
0	0	0	0	0	0	0	0	0	0	0	0	0	0	0	0	0	0	0
0	0	0	0	0	0	0	0	0	0	0	0	0	0	0	0	0	0	0
0	0	0	0	0	0	0	0	0	0	0	0	0	0	0	0	0	0	0
0	0	0	0	0	0	0	0	0	0	0	0	0	0	0	0	0	0	0
0	0	0	0	0	0	0	0	0	0	0	0	0	0	0	0	0	0	0
0	0	0	0	0	0	0	0	0	0	0	0	0	0	0	0	0	0	0
0	0	0	0	0	0	0	0	0	0	0	0	0	0	0	0	0	0	0
0	0	0	0	0	0	0	0	0	0	0	0	0	0	0	0	0	0	0
0	0	0	0	0	0	0	0	0	0	0	0	0	0	0	0	0	0	0
0	0	0	0	0	0	0	0	0	0	0	0	0	0	0	0	0	0	0
0	0	0	0	0	0	0	0	0	0	0	0	0	0	0	0	0	0	0
0	0	0	0	0	0	0	0	0	0	0	0	0	0	0	0	0	0	0
0	0	0	0	0	0	0	0	0	0	0	0	0	0	0	0	0	0	0
0	0	0	0	0	0	0	0	0	0	0	0	0	0	0	0	0	0	0
0	0	0	0	0	0	0	0	0	0	0	0	0	0	0	0	0	0	0
0	0	0	0	0	0	0	0	0	0	0	0	0	0	0	0	0	0	0
0	0	0	0	0	0	0	0	0	0	0	0	0	0	0	0	0	0	0
0	0	0	0	0	0	0	0	0	0	0	0	0	0	0	0	0	0	0
0	0	0	0	0	0	0	0	0	0	0	0	0	0	0	0	0	0	0
0	0	0	0	0	0	0	0	0	0	0	0	0	0	0	0	0	0	0
0	0	0	0	0	0	0	0	0	0	0	0	0	0	0	0	0	0	0
0	0	0	0	0	0	0	0	0	0	0	0	0	0	0	0	0	0	0
0	0	0	0	0	0	0	0	0	0	0	0	0	0	0	0	0	0	0
0	0	0	0	0	0	0	0	0	0	0	0	0	0	0	0	0	0	0
0	0	0	0	0	0	0	0	0	0	0	0	0	0	0	0	0	0	0
0	0	0	0	0	0	0	0	0	0	0	0	0	0	0	0	0	0	0
0	0	0	0	0	0	0	0	0	0	0	0	0	0	0	0	0	0	0
0	0	0	0	0	0	0	0	0	0	0	0	0	0	0	0	0	0	0
0	0	0	0	0	0	0	0	0	0	0	0	0	0	0	0	0	0	0
0	0	0	0	0	0	0	0	0	0	0	0	0	0	0	0	0	0	0
0	0	0	0	0	0	0	0	0	0	0	0	0	0	0	0	0	0	0
0	0	0	0	0	0	0	0	0	0	0	0	0	0	0	0	0	0	0
0	0	0	0	0	0	0	0	0	0	0	0	0	0	0	0	0	0	0

0	0	0	0	0	0	0	0	0	0	0	0	0	0	0	0	0	0	0
0	0	0	0	0	0	0	0	0	0	0	0	0	0	0	0	0	0	0
0	0	0	0	0	0	0	0	0	0	0	0	0	0	0	0	0	0	0
0	0	0	0	0	0	0	0	0	0	0	0	0	0	0	0	0	0	0
0	0	0	0	0	0	0	0	0	0	0	0	0	0	0	0	0	0	0
0	0	0	0	0	0	0	0	0	0	0	0	0	0	0	0	0	0	0
0	0	0	0	0	0	0	0	0	0	0	0	0	0	0	0	0	0	0
0	0	0	0	0	0	0	0	0	0	0	0	0	0	0	0	0	0	0
0	0	0	0	0	0	0	0	0	0	0	0	0	0	0	0	0	0	0
0	0	0	0	0	0	0	0	0	0	0	0	0	0	0	0	0	0	0
0	0	0	0	0	0	0	0	0	0	0	0	0	0	0	0	0	0	0
0	0	0	0	0	0	0	0	0	0	0	0	0	0	0	0	0	0	0
0	0	0	0	0	0	0	0	0	0	0	0	0	0	0	0	0	0	0
0	0	0	0	0	0	0	0	0	0	0	0	0	0	0	0	0	0	0
0	0	0	0	0	0	0	0	0	0	0	0	0	0	0	0	0	0	0
0	0	0	0	0	0	0	0	0	0	0	0	0	0	0	0	0	0	0
0	0	0	0	0	0	0	0	0	0	0	0	0	0	0	0	0	0	0
0	0	0	0	0	0	0	0	0	0	0	0	0	0	0	0	0	0	0
0	0	0	0	0	0	0	0	0	0	0	0	0	0	0	0	0	0	0
0	0	0	4	0	0	0	0	0	0	0	0	0	0	0	0	0	0	0
0	0	0	0	0	0	0	0	0	0	0	0	0	0	0	0	0	0	0
0	0	0	0	0	0	0	0	0	0	0	0	0	0	0	0	0	0	0
0	0	0	0	0	0	0	0	0	0	0	0	0	0	0	0	0	0	0
0	0	0	0	0	0	0	0	0	0	0	0	0	0	0	0	0	0	0
0	0	0	0	0	0	0	0	0	0	0	0	0	0	0	0	0	0	0
0	0	0	0	0	0	0	0	0	0	0	0	0	0	0	0	0	0	0
0	0	0	0	0	0	0	0	0	0	0	0	0	0	0	0	0	0	0
0	0	0	0	0	0	0	0	0	0	0	0	0	0	0	0	0	0	0
0	0	0	0	0	0	0	0	0	0	0	0	0	0	0	0	0	0	0
0	0	0	0	0	0	0	0	0	0	0	0	0	0	0	0	0	0	0
0	0	0	0	0	0	0	0	0	0	0	0	0	0	0	0	0	0	0
0	0	0	0	0	0	0	0	0	0	0	0	0	0	0	0	0	0	0
0	0	0	0	0	0	0	0	0	0	0	0	0	0	0	0	0	0	0
0	0	0	0	0	0	0	0	0	0	0	0	0	0	0	0	0	0	0
0	0	0	0	0	0	0	0	0	3	0	0	0	0	0	0	0	0	0
0	0	0	0	0	0	0	0	0	0	0	0	0	0	0	0	0	0	0

0	0	0	0	0	0	0	0	0	0	0	0	0	0	0	0	0	0	0
0	0	0	0	0	0	0	0	0	0	0	0	0	0	0	0	0	0	0
0	0	0	0	0	0	0	0	0	0	0	0	0	0	0	0	0	0	0
0	0	0	0	0	0	0	0	0	0	0	0	0	0	0	0	0	0	0
0	0	0	0	0	0	0	0	0	0	0	0	0	0	0	0	0	0	0
0	0	0	0	0	0	0	0	0	0	0	0	0	0	0	0	0	0	0
0	0	0	0	0	0	0	0	0	0	0	0	0	0	0	0	0	0	0
0	0	0	0	0	0	0	0	0	0	0	0	0	0	0	0	0	0	0
0	0	0	0	0	0	0	0	0	0	0	0	0	0	0	0	0	0	0
0	0	0	0	0	0	0	0	0	0	0	0	0	0	0	0	0	0	0
0	0	0	0	0	0	0	0	0	0	0	0	0	0	0	0	0	0	0
0	0	0	0	0	0	0	0	0	0	0	0	0	0	0	0	0	0	0
0	0	0	0	0	0	0	0	0	0	0	0	0	0	0	0	0	0	0
0	0	0	0	0	0	0	0	0	0	0	0	0	0	0	0	0	0	0
0	0	0	0	0	0	0	0	0	0	0	0	0	0	0	0	0	0	0
0	0	0	0	0	0	0	0	0	0	0	0	0	0	0	0	0	0	0
0	0	0	0	0	0	0	0	0	0	0	0	0	0	0	0	0	0	0
0	0	0	0	0	0	0	0	0	0	0	0	0	0	0	0	0	0	0
0	0	0	0	0	0	0	0	0	0	0	0	0	0	0	0	0	0	0
0	0	0	0	0	0	0	0	0	0	0	0	0	0	0	0	0	0	0
0	0	0	0	0	0	0	0	0	0	0	0	0	0	0	0	0	0	0
0	0	0	0	0	0	0	0	0	0	0	0	0	0	0	0	0	0	0
0	0	0	0	0	0	0	0	0	0	0	0	0	0	0	0	0	0	0
0	0	0	0	0	0	0	0	0	0	0	0	0	0	0	0	0	0	0
0	0	0	0	0	0	0	0	0	0	0	0	0	0	0	0	0	0	0
0	0	0	0	0	0	0	0	0	0	0	0	0	0	0	0	0	0	0
0	0	0	0	0	0	2	2	0	0	0	0	0	0	0	0	0	0	0
0	0	0	0	0	0	0	0	0	0	0	0	0	0	0	0	0	0	0
0	0	0	0	0	0	0	0	0	0	0	0	0	0	0	0	0	0	0
0	0	0	0	0	0	0	0	0	0	0	0	0	0	0	0	0	0	0
0	0	0	0	0	0	0	0	0	0	0	0	0	0	0	0	0	0	0
0	0	0	0	0	0	0	0	0	0	0	0	0	0	0	0	0	0	0
0	0	0	0	0	0	0	0	0	0	0	0	0	0	0	0	0	0	0
0	0	0	0	0	0	0	0	0	0	0	0	0	0	0	0	0	0	0
0	0	0	0	0	0	0	0	0	0	0	0	0	0	0	0	0	0	0
0	0	0	0	0	0	0	0	0	0	0	0	0	0	0	0	0	0	0
0	0	0	0	0	0	0	0	0	0	0	0	0	0	0	0	0	0	0

0	0	0	0	0	0	0	0	0	0	0	0	0	0	0	0	0	0	0
0	0	0	0	0	0	0	0	0	0	0	0	0	0	0	0	0	0	0
0	0	0	0	0	0	0	0	0	0	0	0	0	0	0	0	0	0	0
0	0	0	0	0	0	0	0	0	0	0	0	0	0	0	0	0	0	0
0	0	0	0	0	0	0	0	0	0	0	0	0	0	0	0	0	0	0
0	0	0	0	0	0	0	0	0	0	0	0	0	0	0	0	0	0	0
0	0	0	0	0	0	0	0	0	0	0	0	0	0	0	0	0	0	0
0	0	0	0	0	0	0	0	0	0	0	0	0	0	0	0	0	0	0
0	0	0	0	0	0	0	0	0	0	0	0	0	0	0	0	0	0	0
0	0	0	0	0	0	0	0	0	0									
Jan_DL152	1051	762	391	498	14	405	302	299	353	106	261	280	93	276	252	73	260	275
205	138	89	56	131	75	155	123	134	85	158	96	90	70	77	111	97	78	113
66	42	74	97	73	92	103	0	81	26	126	116	32	93	82	112	175	72	0
48	37	107	40	87	42	41	26	24	31	20	0	0	0	16	71	12	31	14
40	35	41	29	22	30	63	38	0	28	70	85	16	13	34	118	10	15	82
49	16	65	0	0	0	44	7	17	9	0	0	0	0	11	14	0	24	0
0	27	0	14	7	0	0	0	0	0	0	0	7	0	0	17	0	0	0
0	0	0	0	0	0	0	0	19	3	0	0	0	0	0	0	0	0	0
22	0	17	0	0	7	28	0	0	19	0	0	0	31	18	0	0	0	0
0	55	0	45	0	0	0	0	0	0	0	0	0	0	0	0	9	0	0
0	0	0	0	0	0	0	0	0	0	0	0	0	0	11	0	0	0	0
0	0	24	0	0	23	0	0	0	0	0	0	0	14	0	0	0	10	0
0	0	0	0	0	19	0	0	0	0	0	10	0	3	0	0	0	0	10
0	0	0	0	0	0	0	0	0	0	0	0	0	0	6	0	0	6	0
15	0	0	0	0	0	0	0	11	0	0	0	0	0	4	0	0	0	0
0	0	0	0	0	0	0	0	0	0	0	0	0	0	0	0	0	0	0
0	0	0	0	0	0	0	0	0	0	0	0	0	8	10	0	0	0	7
0	0	0	0	0	0	0	5	0	0	0	0	0	0	8	13	0	0	0
8	0	0	0	0	0	0	0	0	4	0	0	0	9	0	0	0	0	5
0	0	0	0	0	0	0	0	0	0	0	0	0	0	4	0	0	0	0
0	0	0	0	0	0	0	0	0	0	0	0	0	0	0	0	0	0	0
0	0	0	0	0	0	0	0	0	0	0	0	0	0	0	0	0	0	0
0	0	0	0	0	0	0	0	11	0	44	0	0	0	0	0	0	0	0
0	0	0	0	0	0	0	0	0	0	0	0	0	0	0	0	0	0	0
0	0	0	0	0	0	0	0	0	0	0	0	0	0	0	0	0	0	0
0	0	0	0	0	0	0	0	0	0	0	0	0	0	0	0	0	0	0
0	0	0	0	0	0	0	0	0	0	0	0	0	0	0	0	0	0	0

0	22	0	0	0	0	0	0	0	0	0	0	0	0	0	0	14	0	0
0	0	0	0	0	0	0	0	0	0	0	0	0	0	0	0	0	0	0
0	0	0	0	0	0	0	0	0	0	0	0	0	0	0	0	0	0	0
0	4	6	0	0	0	0	0	0	0	0	0	0	0	0	0	0	0	0
0	0	0	0	0	0	0	0	27	0	0	0	0	0	0	0	0	0	0
0	0	0	0	0	0	0	0	0	0	0	0	0	0	0	0	0	0	0
0	0	0	0	0	0	5	0	0	0	0	0	0	0	0	0	0	0	0
0	0	0	0	0	6	0	0	0	0	0	0	0	0	0	0	0	0	0
0	0	0	0	0	0	0	0	0	0	0	0	0	0	12	0	0	0	0
0	0	0	0	0	0	0	0	0	0	0	0	0	0	0	0	0	0	0
0	2	0	0	0	0	0	0	0	0	0	0	0	0	0	0	0	0	0
0	0	0	0	0	0	0	0	0	0	0	0	0	0	0	0	0	0	0
0	0	0	0	0	0	0	0	0	0	0	0	0	0	0	0	0	0	0
0	0	0	0	0	0	0	5	0	0	0	0	0	0	0	0	0	0	0
0	0	0	0	0	0	0	0	0	0	0	0	0	0	0	0	0	0	0
0	0	0	0	0	0	0	0	0	0	0	0	0	0	0	0	0	0	0
0	0	0	0	0	0	0	0	0	0	0	0	0	0	0	0	0	0	0
0	0	0	0	0	0	0	0	0	0	0	0	0	0	0	0	0	0	0
0	0	0	0	0	0	0	0	0	0	0	0	0	0	0	0	0	0	0
0	0	0	0	0	0	0	0	0	0	0	0	0	0	0	0	0	0	0
0	0	0	0	0	0	0	0	0	0	0	0	0	0	0	0	0	0	0
0	0	0	0	0	0	0	0	0	0	0	0	0	0	0	0	0	0	0
0	0	0	0	0	20	0	0	0	0	0	0	0	0	0	0	0	0	0
0	0	0	0	0	0	0	0	0	0	0	0	15	0	0	0	0	0	0
0	0	0	0	0	0	0	0	0	0	0	0	0	0	0	0	0	0	0
0	0	0	0	0	0	0	0	0	0	0	0	0	0	0	0	0	0	0
0	4	0	0	0	0	0	0	0	0	0	0	0	7	0	0	0	0	0
0	0	0	0	0	0	0	0	0	0	0	0	0	0	0	0	0	0	0
0	0	0	0	0	0	0	0	0	0	0	0	0	0	0	0	0	0	0
0	0	0	0	0	0	0	0	0	0	0	0	0	0	0	0	0	0	14
0	0	0	0	0	0	0	0	0	0	0	0	0	0	0	0	0	0	0
0	0	0	0	0	0	0	0	0	0	0	0	0	0	0	0	0	0	0
0	0	0	0	0	0	0	0	0	0	0	0	0	0	0	0	0	0	0
0	0	0	0	0	0	0	0	0	0	0	0	0	0	0	0	0	0	0
0	0	0	0	0	0	0	0	0	0	0	0	0	0	0	0	0	0	0
0	0	0	0	0	0	0	0	0	0	0	0	0	0	0	0	0	0	0
0	0	0	0	0	0	0	0	0	0	0	0	0	0	0	0	0	0	0

0	0	0	0	0	0	0	0	0	0	0	0	0	0	0	0	0	0	0
2	0	0	0	0	0	0	0	0	0	0	0	0	0	0	0	0	0	0
0	0	0	0	0	0	0	0	0	0	0	0	0	0	0	0	0	0	0
0	0	0	0	0	0	0	0	0	0	0	0	0	0	0	0	0	0	0
0	0	0	0	0	0	0	0	0	0	0	0	0	0	0	0	0	0	0
0	0	0	0	0	0	0	0	0	0	0	0	0	0	0	0	0	0	0
0	0	0	0	0	0	0	0	0	0	0	0	0	0	0	0	0	0	0
0	0	0	0	0	0	0	0	0	0	0	0	0	0	0	0	0	0	0
0	0	0	0	0	0	0	0	0	0	0	0	0	0	0	0	0	0	0
0	0	0	0	0	0	0	0	0	0	0	0	0	0	0	0	0	0	0
0	0	0	0	0	0	0	0	0	0	0	0	0	0	0	0	0	0	0
0	0	0	0	0	0	0	0	0	0	0	0	0	0	0	0	0	0	0
0	0	0	0	0	0	0	0	0	0	0	0	0	0	0	0	0	0	0
0	0	0	0	0	0	0	0	0	0	0	0	0	0	0	0	0	0	0
0	0	0	0	0	0	0	0	0	0	0	0	0	0	0	0	0	0	0
0	0	0	0	0	0	0	0	0	0	0	0	0	0	0	0	0	0	0
0	0	0	0	0	0	0	0	0	0	0	0	0	0	0	0	0	0	0
0	0	0	0	0	0	0	0	0	0	0	0	0	0	0	0	0	0	0
0	0	0	0	0	0	0	0	0	0	0	0	0	0	0	0	0	0	0
0	0	0	0	0	0	0	0	0	0	0	0	6	0	0	0	0	0	0
0	0	0	0	0	0	0	0	0	0	0	0	0	0	0	0	0	0	0
0	0	0	0	0	0	0	0	0	0	0	0	0	0	0	0	0	0	0
0	0	0	0	0	0	0	0	0	0	0	0	0	0	0	0	0	0	0
0	0	0	0	0	0	0	0	0	0	0	0	0	0	0	0	0	0	0
0	0	0	0	0	0	0	0	0	0	0	0	0	0	0	0	0	0	0
0	0	0	0	0	0	0	0	0	0	0	0	0	0	0	0	0	0	0
0	0	0	0	0	0	0	0	0	0	0	0	0	0	0	0	0	0	0
0	0	0	5	5	0	0	0	0	0	0	0	0	0	0	0	0	0	0
0	0	0	0	0	0	0	0	0	0	0	0	0	0	0	0	0	0	0
0	0	0	0	0	0	0	0	0	0	0	0	0	0	0	0	0	0	0
0	0	0	0	0	0	0	0	0	0	0	0	0	0	0	0	0	0	0
0	0	0	0	0	0	0	0	0	0	0	0	0	0	0	0	0	0	0
0	0	0	0	0	0	0	0	0	0	0	0	0	0	0	0	0	0	0
0	0	0	0	0	0	0	0	0	0	0	0	0	0	0	0	0	0	0
0	0	0	0	0	0	0	0	0	0	0	0	0	0	0	0	0	0	0
0	0	0	0	0	0	0	0	0	0	0	0	0	0	0	0	0	0	0
0	0	0	0	0	0	0	0	0	0	0	0	0	4	4	0	0	0	0

0	0	0	0	0	0	0	0	0	0	0	0	0	0	0	0	0	0	0
0	0	0	0	0	0	0	0	0	0	0	0	0	0	0	0	0	0	0
0	0	0	0	0	0	0	0	0	0	0	0	0	0	0	0	0	0	0
0	0	0	0	0	0	0	0	0	0	0	0	0	0	0	0	0	0	0
0	0	0	0	0	0	0	0	0	0	0	0	0	0	0	0	0	0	0
0	0	0	0	0	0	0	0	0	0	0	0	0	0	0	0	0	0	0
0	0	0	0	0	0	0	0	0	0	0	0	0	0	0	0	0	0	0
0	0	0	0	0	0	0	0	0	0	0	0	0	0	0	0	0	0	0
0	0	0	0	0	0	0	0	0	0	0	0	0	0	0	0	0	0	0
0	0	0	0	0	0	0	0	0	0	0	0	0	0	0	0	0	0	0
0	0	0	0	0	0	0	0	0	0	0	0	0	0	0	0	0	0	0
0	0	0	0	0	0	0	0	0	0	0	0	0	0	0	0	0	0	0
0	0	0	0	0	0	0	0	0	0	0	0	0	0	0	0	0	0	0
0	0	0	0	3	3	3	3	0	0	0	0	0	0	0	0	0	0	0
0	0	0	0	0	0	0	0	0	0	0	0	0	0	0	0	0	0	0
0	0	0	0	0	0	0	0	0	0	0	0	0	0	0	0	0	0	0
0	0	0	0	0	0	0	0	0	0	0	0	0	0	0	0	0	0	0
0	0	0	0	0	0	0	0	0	0	0	0	0	0	0	0	0	0	0
0	0	0	0	0	0	0	0	0	0	0	0	0	0	0	0	0	0	0
0	0	0	0	0	0	0	0	0	0	0	0	0	0	0	0	0	0	0
0	0	0	0	0	0	0	0	0	0	0	0	0	0	0	0	0	0	0
0	0	0	0	0	0	0	0	0	0	0	0	0	0	0	0	0	0	0
0	0	0	0	0	0	0	0	0	0	0	0	0	0	0	0	0	0	0
0	0	0	0	0	0	0	0	0	0	0	0	0	0	0	0	0	0	0
0	0	0	0	0	0	0	0	0	0	0	0	0	0	0	0	0	0	0
0	0	0	0	0	0	0	0	0	0	0	0	0	0	0	0	0	0	0
0	0	0	0	0	0	0	0	0	0	0	0	0	0	0	0	0	0	0
0	0	0	0	0	0	0	0	0	0	0	0	0	0	0	0	0	0	0
0	0	0	0	0	0	0	0	0	0	0	0	0	0	0	0	0	0	0
0	0	0	0	0	0	0	0	0	0	0	0	0	0	0	0	0	0	0
0	0	0	0	0	0	0	0	0	0	0	0	0	0	0	0	0	0	0
0	0	0	0	0	0	0	0	0	0	0	0	0	0	0	0	0	0	0
0	0	0	0	0	0	0	0	0	0	0	0	0	0	0	0	0	0	0
0	0	0	0	0	0	0	0	0	0	0	0	0	0	0	0	0	0	0
0	0	0	0	0	0	0	0	0	0	0	0	0	0	0	0	0	0	0
0	0	0	0	0	0	0	0	0	0	0	0	0	0	0	0	0	0	2
2	2	2	2	2	2	0	0	0	0	0	0	0	0	0	0	0	0	0

0	0	0	0	0	0	0	0	0	0	0	0	0	0	0	0	0	0	0
0	0	0	0	0	0	0	0	0	0	0	0	0	0	0	0	0	0	0
0	0	0	0	0	0	0	0	0	0	0	0	0	0	0	0	0	0	0
0	0	0	0	0	0	0	0	0	0	0	0	0	0	0	0	0	0	0
0	0	0	0	0	0	0	0	0	0	0	0	0	0	0	0	0	0	0
0	0	0	0	0	0	0	0	0	0	0	0	0	0	0	0	0	0	0
0	0	0	0	0	0	0	0	0	0	0	0	0	0	0	0	0	0	0
0	0	0	0	0	0	0	0	0	0	0	0	0	0	0	0	0	0	0
0	0	0	0	0	0	0	0	0	0	0	0	0	0	0	0	0	0	0
0	0	0	0	0	0	0	0	0	0	0	0	0	0	0	0	0	0	0
0	0	0	0	0	0	0	0	0	0	0	0	0	0	0	0	0	0	0
0	0	0	0	0	0	0	0	0	0	0	0	0	0	0	0	0	0	0
0	0	0	0	0	0	0	0	0	0	0	0	0	0	0	0	0	0	0
0	0	0	0	0	0	0	0	0	0	0	0	0	0	0	0	0	0	0
0	0	0	0	0	0	0	0	0	0	0	0	0	0	0	0	0	0	0
0	0	0	0	0	0	0	0	0	0	0	0	0	0	0	0	0	0	0
0	0	0	0	0	0	0	0	0	0	0	0	0	0	0	0	0	0	0
0	0	0	0	0	0	0	0	0	0	0	0	0	0	0	0	0	0	0
0	0	0	0	0	0	0	0	0	0	0	0	0	0	0	0	0	0	0
0	0	0	0	0	0	0	0	0	0	0	0	0	0	0	0	0	0	0
0	0	0	0	0	0	0	0	0	0	0	0	0	0	0	0	0	0	0
0	0	0	0	0	0	0	0	0	0	0	0	0	0	0	0	0	0	0
0	0	0	0	0	0	0	0	0	0	0	0	0	0	0	0	0	0	0
0	0	0	0	0	0	0	0	0	0	0	0	0	0	0	0	0	0	0
0	0	0	0	0	0	0	0	0	0	0	0	0	0	0	0	0	0	0
0	0	0	0	0	0	0	0	0	0	0	0	0	0	0	0	0	0	0
0	0	0	0	0	0	0	0	0	0	0	0	0	0	0	0	0	0	0
0	0	0	0	0	0	0	0	0	0	0	0	0	0	0	0	0	0	0
0	0	0	0	0	0	0	0	0	0	0	0	0	0	0	0	0	0	0
0	0	0	0	0	0	0	0	0	0	0	0	0	0	0	0	0	0	0
0	0	0	0	0	0	0	0	0	0	0	0	0	0	0	0	0	0	0
0	0	0	0	0	0	0	0	0	0	0	0	0	0	0	0	0	0	0
0	0	0	0	0	0	0	0	0	0	0	0	0	0	0	0	0	0	0
0	0	0	0	0	0	0	0	0	0	0	0	0	0	0	0	0	0	0
0	0	0	0	0	0	0	0	0	0	0	0	0	0	0	0	0	0	0
0	0	0	0	0	0	0	0	0	0	0	0	0	0	0	0	0	0	0
0	0	0	0	0	0	0	0	0	0	0	0	0	0	0	0	0	0	0

0	0	0	0	0	0	0	0	0	0	0	0	0	0	0	0	0	0	0
0	0	0	0	0	0	0	0	0	0									
Jan_DL153	2162	1992	328	470	477	272	275	243	359	147	448	87	130	116	149	163	114	170
262	14	181	19	211	136	71	99	68	164	87	342	227	91	64	224	84	35	294
157	33	147	141	90	118	99	11	85	88	240	86	57	122	148	97	223	145	199
219	0	66	48	159	215	104	142	28	15	49	0	4	95	0	284	20	74	99
37	90	69	125	8	33	30	40	0	0	0	0	0	171	67	102	0	45	0
54	10	72	0	26	9	0	0	0	23	0	10	8	0	22	35	15	0	0
0	14	10	13	20	23	0	0	0	50	0	0	13	11	0	0	0	0	0
51	88	61	0	0	0	0	0	25	8	0	0	27	0	0	0	0	0	0
0	22	0	0	49	160	25	0	0	0	0	0	0	0	0	5	5	0	0
0	0	11	0	0	0	0	38	0	28	0	0	76	0	0	21	26	0	0
0	0	11	0	0	0	32	0	0	0	0	0	0	0	0	0	0	0	0
0	0	0	0	0	0	0	0	0	0	0	0	0	0	0	0	0	9	0
0	0	0	13	0	20	0	0	67	0	0	0	0	8	0	11	9	0	0
0	0	0	0	0	0	0	0	0	0	0	0	0	0	13	23	0	0	0
10	59	0	19	0	22	0	0	0	0	0	0	0	0	0	4	0	0	0
0	17	0	0	0	0	0	0	0	0	0	0	0	0	16	0	14	29	0
0	8	13	0	0	0	0	0	0	0	0	0	0	0	2	8	0	0	0
0	0	19	0	0	0	0	9	0	0	0	0	0	0	0	0	0	0	42
9	25	0	0	5	6	0	30	0	0	0	0	0	0	0	0	0	0	0
0	61	0	0	16	0	0	0	0	0	9	0	0	0	0	37	0	0	0
0	0	0	0	0	0	0	0	6	0	0	0	0	0	0	0	0	10	0
0	0	0	0	0	0	14	0	0	0	0	0	0	0	14	0	0	0	0
0	0	0	0	0	0	0	0	4	102	0	16	0	0	0	0	0	0	0
0	10	0	0	0	18	0	0	0	0	0	0	0	0	88	0	8	0	0
0	12	0	0	0	0	0	0	0	0	0	0	0	0	0	0	0	0	0
0	0	0	0	9	0	0	0	0	0	0	0	0	0	12	0	0	0	0
0	0	0	0	0	19	0	0	0	6	0	0	0	0	0	0	0	0	0
0	0	0	0	0	7	0	0	0	0	0	0	0	0	0	10	0	0	0
0	0	0	0	0	0	33	0	0	0	0	0	0	0	0	0	0	0	0
0	10	0	0	0	0	0	0	0	0	0	0	11	0	0	0	0	0	0
0	0	0	0	0	0	0	0	10	0	0	0	0	0	0	0	0	0	7
0	0	0	0	0	0	0	0	0	0	0	0	0	5	0	0	0	0	0
0	0	0	0	0	0	0	0	0	0	0	0	0	0	0	0	0	0	0
0	0	0	0	0	0	0	0	0	0	3	0	0	0	0	0	0	0	0
0	0	0	0	0	22	0	0	0	0	0	0	0	0	21	0	0	0	17

0	0	0	0	0	0	0	0	0	0	0	0	0	0	0	0	0	0	0
0	0	0	0	0	0	0	0	0	0	0	0	0	0	0	0	0	0	0
0	0	7	10	0	0	0	0	0	0	0	0	0	0	0	0	0	0	0
0	0	0	0	0	0	0	0	0	0	0	0	0	0	0	0	0	0	0
0	0	0	0	0	0	3	0	0	23	0	0	0	0	0	0	0	0	0
0	0	0	0	0	0	0	0	0	0	0	0	0	0	0	0	0	0	0
0	0	0	0	0	0	0	0	0	0	0	0	0	0	0	0	0	0	0
0	0	0	12	0	0	0	0	0	0	0	0	0	0	0	9	0	0	0
0	0	0	0	0	0	0	0	0	0	0	0	0	0	0	0	0	0	0
0	0	0	0	0	0	0	0	0	0	0	0	0	0	0	0	0	0	0
0	0	0	0	0	3	0	0	0	0	0	0	0	0	0	0	0	0	0
0	0	6	0	0	0	0	0	0	0	0	0	0	0	0	0	0	0	0
0	0	0	0	0	0	0	3	0	0	0	0	0	0	22	0	0	0	0
0	0	0	0	0	0	0	0	0	0	0	0	0	0	0	0	0	0	0
0	0	0	0	0	0	0	0	0	0	0	0	0	0	0	0	0	0	0
0	0	0	0	0	0	0	0	0	0	0	0	0	0	0	0	0	0	0
0	0	0	0	0	0	0	0	0	0	0	0	0	0	0	0	0	0	0
0	0	0	0	0	0	0	0	0	0	0	0	0	0	0	0	0	0	0
0	0	0	0	0	0	0	0	0	0	0	0	0	0	0	0	0	0	0
0	0	0	0	0	0	0	0	5	0	0	0	0	0	0	0	0	0	0
0	0	0	0	0	0	0	0	0	0	0	0	0	0	0	0	0	0	0
0	0	0	0	0	0	0	0	0	0	0	0	0	0	0	0	0	0	0
0	0	0	0	0	0	0	0	0	0	0	0	0	0	0	0	0	0	0
0	0	0	0	0	0	0	0	0	0	0	0	0	0	0	0	0	0	0
0	0	0	0	0	0	0	0	0	0	0	0	0	0	0	0	0	0	0
0	0	0	0	0	0	0	0	0	0	0	0	0	0	0	0	0	0	0
0	0	0	0	0	0	0	0	0	0	0	0	0	0	0	0	0	0	0
3	0	0	0	0	0	0	0	0	0	0	1	0	0	0	0	0	0	0
0	0	0	0	0	0	0	0	0	0	0	0	0	0	0	0	0	0	0
0	0	0	0	0	0	0	0	0	0	0	0	0	0	0	0	0	0	0
0	0	0	0	0	0	0	0	0	0	0	0	0	0	0	0	0	0	0
5	0	0	0	0	0	0	0	0	0	0	0	0	6	0	0	0	0	0
0	0	0	0	0	0	0	0	0	0	0	0	0	0	0	0	0	0	0
0	0	0	0	0	0	0	0	0	0	0	0	0	0	0	0	0	0	0
0	0	0	0	0	0	0	0	0	0	0	0	0	0	0	0	0	0	0
0	0	0	0	0	0	0	0	0	0	0	0	0	0	0	0	0	0	0
0	0	0	0	0	0	0	0	0	0	0	0	0	0	0	0	0	0	0

0	0	0	0	0	0	0	0	0	0	0	0	0	0	0	0	0	0	0
0	0	0	0	0	0	0	0	0	0	0	0	0	0	0	0	0	0	0
0	0	0	0	0	0	0	0	0	0	0	0	0	0	0	0	0	0	0
0	0	0	0	0	0	0	0	0	0	0	0	0	0	0	0	0	0	0
0	0	0	0	0	0	0	0	0	0	0	0	0	0	0	0	0	5	0
0	0	0	0	0	0	0	0	0	0	0	0	0	0	0	0	0	0	0
0	0	0	0	0	0	0	0	0	0	0	0	0	0	0	0	0	0	0
0	0	0	0	0	0	0	0	0	0	0	0	0	0	0	0	0	0	0
0	0	0	0	0	0	0	0	0	0	0	0	0	0	0	0	0	0	0
0	0	0	0	0	0	0	0	0	0	0	0	0	0	0	0	0	0	0
0	0	0	0	0	0	0	0	0	0	0	0	0	0	0	0	0	0	0
0	0	0	0	0	0	0	0	0	0	0	9	0	0	0	0	0	0	0
0	0	0	0	0	0	0	0	0	0	0	0	0	0	0	0	0	0	0
0	0	0	0	0	0	0	0	0	0	0	0	0	0	0	0	0	0	0
0	0	0	0	0	0	0	0	0	0	0	0	0	0	0	0	0	0	0
0	0	0	0	0	0	0	0	0	0	0	0	0	0	0	0	0	0	8
0	0	0	0	0	0	0	0	0	0	0	0	0	0	0	0	0	0	0
0	0	0	0	0	0	0	0	0	0	0	0	0	0	0	0	0	0	0
0	0	0	0	0	0	0	0	0	0	0	0	0	0	0	0	0	0	0
0	0	0	0	0	0	0	0	0	0	0	0	0	0	0	0	0	0	0
0	0	0	0	0	0	0	0	0	0	0	0	0	0	0	0	0	0	0
0	0	0	0	0	0	0	0	0	0	0	0	0	0	0	0	0	0	0
0	0	0	0	0	0	0	0	0	0	0	0	0	0	0	0	0	0	0
0	0	0	0	0	0	0	0	0	0	0	0	0	0	0	0	0	0	0
0	0	0	0	0	0	0	0	0	0	0	0	0	0	0	0	0	0	0
0	0	0	0	0	0	0	0	0	0	0	0	0	0	0	0	0	0	0
0	0	0	0	0	0	0	0	0	0	0	0	0	0	0	0	0	0	0
0	0	0	0	0	0	0	0	0	0	0	0	0	0	0	0	0	0	0
0	0	0	0	0	0	0	0	0	0	0	0	6	0	0	0	0	0	0
0	0	0	0	0	0	0	0	0	0	0	0	0	0	0	0	0	0	0
0	0	0	0	0	0	0	0	0	0	0	0	0	0	0	0	0	0	0
0	0	0	0	0	0	0	0	0	0	0	0	0	0	0	0	0	0	0
0	0	0	0	0	0	0	0	0	0	0	0	0	0	0	0	0	0	0
0	0	0	0	0	0	0	0	0	0	0	0	0	0	0	0	0	0	0
0	0	0	0	0	0	0	0	0	0	0	0	0	0	0	0	0	0	0
0	0	0	0	0	0	0	0	0	0	0	0	0	0	0	0	0	0	0
0	0	0	0	0	0	0	0	0	0	0	0	0	0	0	0	0	0	0

0	0	0	0	0	0	0	0	0	0	0	0	0	0	0	0	0	0	0
0	0	0	0	0	0	0	0	0	0	0	0	0	0	0	0	0	0	0
0	0	0	0	0	0	0	0	0	0	0	0	0	0	0	0	0	0	0
0	0	0	0	0	0	0	0	0	0	0	0	0	0	0	0	0	0	0
0	0	0	0	0	0	0	0	0	0	0	0	0	0	0	0	0	0	0
0	0	0	0	0	0	0	0	0	0	0	0	0	0	0	0	0	0	0
0	0	0	0	0	0	0	0	0	0	0	0	0	0	0	0	0	0	0
0	0	0	0	0	0	0	0	0	0	0	0	0	0	0	0	0	0	0
0	0	0	0	0	0	0	0	0	0	0	0	0	0	0	0	0	0	0
0	0	0	0	0	0	0	0	0	0	0	0	0	0	0	0	0	0	0
0	0	0	0	0	0	0	0	0	0	0	0	0	0	0	0	0	0	0
0	0	0	0	0	0	0	0	0	0	0	0	0	0	0	0	0	0	0
0	0	0	0	0	0	0	0	0	0	0	0	0	0	0	0	0	0	0
0	0	0	0	0	0	0	0	0	0	0	0	0	0	0	0	0	0	0
0	0	0	0	0	0	0	0	0	0	0	0	0	0	0	0	0	0	0
0	0	0	0	0	0	0	0	0	0	0	0	0	0	0	0	0	0	0
0	0	0	0	0	0	0	0	0	0	0	0	0	0	0	0	0	0	0
0	0	0	0	0	0	0	0	0	0	0	0	0	0	0	0	0	0	0
0	0	0	0	0	0	0	0	0	0	0	0	0	0	0	0	0	0	0
0	0	0	0	0	0	0	0	0	0	0	0	0	0	0	0	0	0	0
0	0	0	0	0	0	0	0	0	0	0	0	0	0	0	0	0	0	0
0	0	0	0	0	0	0	0	0	0	0	0	0	0	0	0	0	0	0
0	0	0	0	0	0	0	0	0	0	0	0	0	0	0	0	0	0	0
0	0	0	0	0	0	0	0	0	0	0	0	0	0	0	0	0	0	0
0	0	0	0	0	0	0	0	0	0	0	0	0	0	0	0	0	0	0
0	0	0	0	0	0	0	0	0	0	0	0	0	0	0	0	0	0	0
0	0	0	0	0	0	0	0	0	0	0	0	0	0	0	0	0	0	0
0	0	0	0	0	0	0	0	0	0	0	0	0	0	0	0	3	3	0
0	0	0	0	0	0	0	0	0	0	0	0	0	0	0	0	0	0	0
0	0	0	0	0	0	0	0	0	0	0	0	0	0	0	0	0	0	0
0	0	0	0	0	0	0	0	0	0	0	0	0	0	0	0	0	0	0
0	0	0	0	0	0	0	0	0	0	0	0	0	0	0	0	0	0	0
0	0	0	0	0	0	0	0	0	0	0	0	0	0	0	0	0	0	0
0	0	0	0	0	0	0	0	0	0	0	0	0	0	0	0	0	0	0
0	0	0	0	0	0	0	0	0	0	0	0	0	0	0	0	0	0	0
0	0	0	0	0	0	0	0	0	0	0	0	0	0	0	0	0	0	0
0	0	0	0	0	0	0	0	0	0	0	0	0	0	0	0	0	0	0

0	0	0	0	0	0	0	0	0	0	0	0	0	0	0	0	0	0	0
0	0	0	0	0	0	0	0	0	0	0	0	0	0	0	0	0	0	0
0	0	0	0	0	0	0	0	0	0	0	0	0	0	0	0	0	0	0
0	0	0	0	0	0	0	0	0	0	0	0	0	0	0	0	0	0	0
0	0	0	0	0	0	0	0	0	0	0	0	0	0	0	0	0	0	0
0	0	0	0	0	0	0	0	0	0	0	0	0	0	0	0	0	0	0
0	0	0	0	0	0	0	0	0	0	0	0	0	0	0	0	0	0	0
0	0	0	0	0	0	0	0	0	0	0	0	0	0	0	0	0	0	0
0	0	0	0	0	0	0	0	0	0	0	0	0	0	0	0	0	0	0
0	0	0	0	0	0	0	0	0	0	0	0	0	0	0	0	0	0	0
0	0	0	0	0	0	0	0	0	0	0	0	0	0	0	0	0	0	0
0	0	0	0	0	0	0	0	0	0	0	0	0	0	0	0	0	0	0
0	0	0	0	0	0	0	0	0	0	0	0	0	0	0	0	0	0	0
0	0	0	0	0	0	0	0	0	0	0	0	0	0	0	0	0	0	0
0	0	0	0	0	0	0	0	0	0	0	0	0	0	0	0	0	0	0
0	0	0	0	0	0	0	0	0	0	0	0	0	0	0	0	0	0	0
0	0	0	0	0	0	0	0	0	0	0	0	0	0	2	2	2	2	2
2	2	0	0	0	0	0	0	0	0	0	0	0	0	0	0	0	0	0
0	0	0	0	0	0	0	0	0	0	0	0	0	0	0	0	0	0	0
0	0	0	0	0	0	0	0	0	0	0	0	0	0	0	0	0	0	0
0	0	0	0	0	0	0	0	0	0	0	0	0	0	0	0	0	0	0
0	0	0	0	0	0	0	0	0	0	0	0	0	0	0	0	0	0	0
0	0	0	0	0	0	0	0	0	0	0	0	0	0	0	0	0	0	0
0	0	0	0	0	0	0	0	0	0	0	0	0	0	0	0	0	0	0
0	0	0	0	0	0	0	0	0	0	0	0	0	0	0	0	0	0	0
0	0	0	0	0	0	0	0	0	0	0	0	0	0	0	0	0	0	0
0	0	0	0	0	0	0	0	0	0	0	0	0	0	0	0	0	0	0
0	0	0	0	0	0	0	0	0	0	0	0	0	0	0	0	0	0	0
0	0	0	0	0	0	0	0	0	0	0	0	0	0	0	0	0	0	0
0	0	0	0	0	0	0	0	0	0	0	0	0	0	0	0	0	0	0
0	0	0	0	0	0	0	0	0	0									
Jan_DL154	1992	1679	488	555	172	462	301	314	360	416	571	354	345	329	246	271	255	188
280	402	144	0	119	95	183	180	151	343	251	220	185	199	169	250	117	25	181
269	50	46	59	133	173	94	0	23	136	282	54	66	160	113	0	311	139	136
96	47	0	68	161	21	42	156	24	5	45	0	20	0	0	66	11	39	0
46	23	48	102	45	45	81	48	61	67	106	128	8	0	45	104	42	0	0
95	35	185	0	0	6	0	0	0	19	0	68	0	45	14	0	18	0	0

0	0	0	34	45	38	0	0	0	0	7	0	18	0	0	0	0	0	0
0	30	0	0	0	0	0	0	0	45	0	0	0	121	45	0	0	0	0
0	0	11	0	0	22	65	0	0	0	0	0	10	29	27	0	0	0	0
0	46	11	0	0	0	0	0	0	39	14	0	124	0	9	0	10	0	0
0	0	22	0	0	0	68	0	0	0	0	0	0	0	0	0	0	0	0
0	0	11	0	0	0	16	0	0	0	0	0	0	0	0	0	0	2	0
0	0	0	0	0	21	0	0	0	0	0	0	0	0	0	0	19	0	39
0	0	0	0	0	0	0	0	0	0	98	0	0	0	8	14	0	0	0
25	0	0	0	0	0	5	0	0	0	0	3	0	4	0	0	2	72	0
19	0	0	0	0	0	0	0	0	93	0	0	6	0	0	0	0	0	0
0	0	0	0	0	0	0	0	0	0	0	0	0	0	0	6	0	7	0
0	13	9	0	0	0	0	13	17	0	0	0	0	0	0	11	0	0	20
5	0	0	0	0	0	0	22	0	0	0	0	0	0	0	0	0	0	0
0	58	0	0	0	0	3	0	0	0	0	0	0	0	0	0	39	0	0
0	0	0	0	0	0	0	0	0	0	0	0	0	0	0	0	0	0	0
0	0	0	0	0	0	0	0	0	0	0	0	0	15	0	0	0	0	0
0	0	12	0	0	0	0	0	14	0	0	4	0	0	0	0	0	0	0
0	9	0	0	0	0	0	0	0	0	0	0	0	0	0	0	0	0	0
0	0	0	0	0	0	0	0	0	0	0	0	0	0	0	0	0	0	0
0	0	0	0	0	0	0	0	0	0	0	0	0	0	0	0	0	0	0
14	0	17	0	0	0	0	0	0	6	0	0	0	0	0	0	0	7	0
0	0	0	0	0	6	0	0	0	0	0	0	0	0	0	0	0	0	0
0	0	0	0	0	0	0	0	0	0	0	0	0	0	0	0	0	0	0
0	6	0	0	16	0	0	0	0	0	0	0	0	0	0	0	0	14	0
0	0	0	0	0	0	0	0	0	0	0	0	0	0	0	0	0	0	0
0	0	0	0	0	0	0	0	0	0	0	0	0	0	0	0	0	0	0
0	0	0	0	0	0	0	0	0	0	0	0	0	0	0	0	0	0	0
5	0	0	0	0	0	0	0	0	0	0	0	0	0	0	0	0	0	0
0	0	0	15	0	0	0	0	9	0	7	0	0	0	20	0	0	3	0
0	0	0	0	0	0	0	0	0	0	0	0	0	0	0	0	0	0	0
0	0	0	0	0	0	0	0	0	0	0	0	0	0	0	0	0	0	0
0	2	0	0	0	0	0	0	0	0	0	0	0	0	0	0	0	0	0
0	0	0	0	25	0	0	0	0	0	0	0	0	0	0	0	0	0	0
0	0	0	0	0	0	0	0	0	0	0	0	0	0	0	0	0	0	0
0	0	0	0	0	0	0	0	0	0	0	0	0	0	0	0	0	0	0
0	0	0	0	0	0	0	0	0	0	0	0	0	0	0	0	0	0	0
0	0	0	0	0	0	0	0	0	0	0	0	0	0	0	0	0	0	0

0	0	0	0	0	0	0	0	0	0	0	0	0	0	5	0	0	0	0
0	0	0	0	0	0	0	0	0	0	0	10	0	0	0	0	0	0	0
0	0	0	0	0	0	0	0	0	0	0	0	0	0	0	0	0	0	0
0	0	0	0	0	0	0	0	0	0	0	0	0	0	0	0	0	0	0
0	0	0	0	0	0	0	0	0	0	0	0	0	0	0	0	0	0	0
0	0	0	0	0	0	0	0	0	0	0	0	0	0	0	0	0	0	0
0	0	0	0	0	0	0	0	0	0	0	0	0	0	0	0	0	0	0
0	0	0	0	0	0	0	0	0	0	0	0	0	0	0	0	0	0	0
0	0	0	0	0	0	0	0	0	0	0	0	0	0	0	0	0	0	0
0	0	0	0	0	0	0	0	0	0	0	0	0	0	0	0	0	0	0
0	0	0	0	0	0	0	0	0	0	0	0	0	0	0	0	0	0	0
0	0	0	0	0	0	0	0	3	0	0	0	0	0	0	0	0	0	0
0	0	0	0	0	0	0	0	0	0	0	0	0	0	0	0	0	0	0
0	0	0	0	0	0	0	0	0	0	0	0	0	0	0	0	0	0	0
0	0	0	0	0	0	0	0	0	0	0	0	0	0	0	0	0	0	0
0	0	0	0	0	0	0	0	0	0	0	0	0	0	0	0	0	0	0
0	0	0	0	0	0	0	0	0	0	0	0	0	0	0	0	0	0	0
0	0	0	0	0	0	0	0	0	0	0	0	0	0	0	12	0	0	0
0	0	0	0	0	0	0	0	0	0	0	0	0	0	0	0	0	0	0
0	0	0	0	0	0	0	0	0	0	0	0	0	0	0	0	0	0	0
0	0	0	0	0	0	0	0	0	0	0	0	0	0	0	0	0	0	0
0	0	0	0	0	0	0	0	0	0	0	0	0	0	0	0	0	0	0
0	0	0	0	0	0	0	0	0	0	0	0	0	0	0	0	0	0	0
0	0	0	0	0	0	0	0	0	0	0	0	0	0	0	0	0	0	0
0	0	0	0	0	0	0	17	0	0	0	0	0	0	0	0	0	0	0
0	0	0	0	0	0	0	0	0	0	0	0	0	0	0	0	0	0	0
0	0	0	0	0	0	0	0	0	0	0	0	0	0	0	0	0	0	0
0	0	0	0	0	0	0	0	0	0	0	0	0	0	0	0	0	0	0
0	0	0	0	0	0	0	0	0	0	0	0	0	0	0	0	0	0	0
0	0	0	0	0	0	0	0	0	0	0	0	0	0	0	0	0	0	0
0	0	0	0	0	0	0	0	0	0	0	0	0	0	0	0	0	0	0
0	0	0	0	0	0	0	0	0	0	0	0	0	0	0	0	0	0	0
0	0	0	0	0	0	0	0	0	0	0	0	0	0	0	0	0	0	0
0	0	0	0	0	0	0	0	0	0	0	0	0	0	0	0	0	0	0
0	0	0	0	0	0	0	0	0	0	0	0	0	0	0	0	0	0	0
0	0	0	0	0	0	0	0	0	0	0	0	0	0	0	0	0	0	0
0	0	0	0	0	0	0	0	0	0	0	0	0	0	0	0	0	0	0

0	0	0	0	0	0	0	0	0	0	0	0	0	0	0	0	0	0	0
0	0	0	0	0	0	0	0	0	0	0	0	0	0	0	10	0	0	0
0	0	0	0	0	0	0	0	0	0	0	0	0	0	0	0	0	0	0
0	0	0	0	0	0	0	0	0	0	0	0	0	0	0	0	0	0	0
0	0	0	0	0	0	0	0	0	0	0	0	0	0	0	0	0	0	0
0	0	0	0	0	0	0	0	0	0	0	0	0	0	0	0	0	0	0
0	0	0	0	0	0	0	0	0	0	0	0	0	0	0	0	0	0	0
0	0	0	0	0	0	0	0	0	0	0	0	0	0	0	0	0	0	0
0	0	0	0	0	0	0	0	0	0	0	0	0	0	0	0	0	0	0
0	0	0	0	0	0	0	0	0	0	0	0	0	0	0	0	0	0	0
0	0	0	0	0	0	0	0	0	0	0	0	0	0	0	0	0	0	0
0	0	0	0	0	0	0	0	0	0	0	0	0	0	0	0	0	0	0
0	0	0	0	0	0	0	0	0	0	0	0	0	0	0	0	0	0	0
0	0	0	0	0	0	0	0	0	0	0	0	0	0	0	0	0	0	0
0	0	0	0	0	0	0	0	0	0	0	0	0	0	0	0	0	0	0
0	0	0	0	0	0	0	0	0	0	0	0	0	0	0	0	0	0	0
0	0	0	0	0	0	0	0	0	0	0	7	0	0	0	0	0	0	0
0	0	0	0	0	0	0	0	0	0	0	0	0	0	0	0	0	0	0
0	0	0	0	0	0	0	0	0	0	0	0	0	0	0	0	0	0	0
0	0	0	0	0	0	0	0	0	0	0	0	0	0	0	0	0	0	0
0	0	0	0	0	0	0	0	0	0	0	0	0	0	0	0	0	0	0
0	0	0	0	0	0	0	0	0	0	0	0	0	0	0	0	0	0	0
0	0	0	0	0	0	0	0	0	0	0	0	0	0	0	0	0	0	0
0	0	0	0	0	0	0	0	0	0	0	0	0	0	0	0	0	0	0
0	6	6	0	0	0	0	0	0	0	0	0	0	0	0	0	0	0	0
0	0	0	0	0	0	0	0	0	0	0	0	0	0	0	0	0	0	0
0	0	0	0	0	0	0	0	0	0	0	0	0	0	0	0	0	0	0
0	0	0	0	0	0	0	0	0	0	0	0	0	0	0	0	0	0	0
0	0	0	0	0	0	0	0	0	0	0	0	0	0	0	0	0	0	0
0	0	0	0	0	0	0	0	0	0	0	0	0	0	0	0	0	0	0
0	0	0	0	0	0	0	0	0	0	0	0	0	0	0	0	0	0	0
0	0	0	0	0	0	0	0	5	5	5	0	0	0	0	0	0	0	0
0	0	0	0	0	0	0	0	0	0	0	0	0	0	0	0	0	0	0
0	0	0	0	0	0	0	0	0	0	0	0	0	0	0	0	0	0	0
0	0	0	0	0	0	0	0	0	0	0	0	0	0	0	0	0	0	0
0	0	0	0	0	0	0	0	0	0	0	0	0	0	0	0	0	0	0
0	0	0	0	0	0	0	0	0	0	0	0	0	0	0	0	0	0	0

0	0	0	0	0	0	0	0	0	0	0	0	0	0	0	0	0	0	0
0	0	0	0	0	0	0	0	0	0	0	0	0	0	0	0	0	0	0
0	0	0	0	0	0	0	0	0	0	0	0	0	0	0	0	0	0	0
0	0	0	0	0	0	0	0	0	0	0	0	0	0	0	0	0	0	0
0	0	0	0	0	0	0	0	0	0	0	0	0	0	0	0	0	0	0
0	0	0	0	0	0	0	0	0	0	0	0	0	0	0	0	0	0	0
0	0	0	0	0	0	0	0	0	0	0	0	0	0	0	0	0	0	0
0	0	0	0	0	0	0	0	0	0	0	0	0	0	0	4	0	0	0
0	0	0	0	0	0	0	0	0	0	0	0	0	0	0	0	0	0	0
0	0	0	0	0	0	0	0	0	0	0	0	0	0	0	0	0	0	0
0	0	0	0	0	0	0	0	0	0	0	0	0	0	0	0	0	0	0
0	0	0	0	0	0	0	0	0	0	0	0	0	0	0	0	0	0	0
0	0	0	0	0	0	0	0	0	0	0	0	0	0	0	0	0	0	0
0	0	0	0	0	0	0	0	0	0	0	0	0	0	0	0	0	0	0
0	0	0	0	0	0	0	0	0	0	0	0	0	0	0	0	0	0	0
0	0	0	0	0	0	0	0	0	0	0	0	0	0	0	0	0	0	0
0	0	0	0	0	0	0	0	0	0	0	0	0	0	0	0	0	0	0
0	0	0	0	0	0	0	0	0	0	0	0	0	0	0	0	0	0	0
0	0	0	0	0	0	0	0	0	0	0	0	0	0	0	0	0	0	0
0	0	0	0	0	0	0	0	0	0	0	0	0	0	0	0	0	0	0
0	0	0	0	0	0	0	0	0	0	0	0	0	0	0	0	0	0	0
0	0	0	0	0	0	0	0	0	0	0	0	0	0	0	0	0	0	0
0	0	0	0	0	0	0	0	0	0	0	0	0	0	0	0	0	0	0
0	0	0	0	0	0	0	0	0	0	0	0	0	0	0	0	0	0	0
0	0	0	0	0	0	0	0	0	0	0	0	0	0	0	0	0	0	0
0	0	0	0	0	0	3	3	3	3	0	0	0	0	0	0	0	0	0
0	0	0	0	0	0	0	0	0	0	0	0	0	0	0	0	0	0	0
0	0	0	0	0	0	0	0	0	0	0	0	0	0	0	0	0	0	0
0	0	0	0	0	0	0	0	0	0	0	0	0	0	0	0	0	0	0
0	0	0	0	0	0	0	0	0	0	0	0	0	0	0	0	0	0	0
0	0	0	0	0	0	0	0	0	0	0	0	0	0	0	0	0	0	0
0	0	0	0	0	0	0	0	0	0	0	0	0	0	0	0	0	0	0
0	0	0	0	0	0	0	0	0	0	0	0	0	0	0	0	0	0	0
0	0	0	0	0	0	0	0	0	0	0	0	0	0	0	0	0	0	0
0	0	0	0	0	0	0	0	0	0	0	0	0	0	0	0	0	0	0
0	0	0	0	0	0	0	0	0	0	0	0	0	0	0	0	0	0	0
0	0	0	0	0	0	0	0	0	0	0	0	0	0	0	0	0	0	0

0	0	0	0	0	0	0	0	0	0	0	0	0	0	0	0	0	0	0
0	0	0	0	0	0	0	0	0	0	0	0	0	0	0	0	0	0	0
0	0	0	0	0	0	0	0	0	0	0	0	0	0	0	0	0	0	0
0	0	0	0	0	0	0	0	0	0	0	0	0	0	0	0	0	0	0
0	0	0	0	0	0	0	0	0	0	0	0	0	0	0	0	0	0	0
0	0	0	0	0	0	0	0	0	0	0	0	0	0	0	0	0	0	0
0	0	0	0	0	0	0	0	0	0	0	0	0	0	0	0	0	0	0
0	0	0	0	0	0	0	0	0	0	0	0	0	0	0	0	0	0	0
0	0	0	0	0	0	0	0	0	0	0	0	0	0	0	0	0	0	0
0	0	0	0	0	0	0	0	0	0	0	0	0	0	0	0	0	0	0
0	0	0	0	0	0	0	0	0	0	0	0	0	0	0	0	0	0	0
0	0	0	0	0	0	0	0	0	0	0	0	0	0	0	0	0	0	0
0	0	0	0	0	0	0	0	0	0	0	0	0	0	0	0	0	0	0
0	0	0	0	0	0	0	0	0	0	0	0	0	0	0	0	0	0	0
0	0	0	0	2	2	2	2	2	2	2	2	2	2	0	0	0	0	0
0	0	0	0	0	0	0	0	0	0	0	0	0	0	0	0	0	0	0
0	0	0	0	0	0	0	0	0	0	0	0	0	0	0	0	0	0	0
0	0	0	0	0	0	0	0	0	0	0	0	0	0	0	0	0	0	0
0	0	0	0	0	0	0	0	0	0	0	0	0	0	0	0	0	0	0
0	0	0	0	0	0	0	0	0	0	0	0	0	0	0	0	0	0	0
0	0	0	0	0	0	0	0	0	0	0	0	0	0	0	0	0	0	0
0	0	0	0	0	0	0	0	0	0	0	0	0	0	0	0	0	0	0
0	0	0	0	0	0	0	0	0	0									
Jan_DL155	1268	1183	548	766	82	510	261	266	655	260	385	259	198	212	133	153	145	227
167	224	201	0	181	164	71	112	73	187	86	510	178	62	95	160	96	605	521
171	20	118	158	116	112	112	9	79	74	72	113	0	66	110	114	0	101	328
257	15	84	43	64	69	91	98	35	66	30	91	0	57	68	201	14	25	0
0	82	88	25	9	0	49	77	0	61	100	0	68	0	0	109	19	15	0
18	41	0	0	0	8	0	0	0	48	0	76	0	0	16	22	0	0	0
0	0	13	25	0	0	0	0	0	45	9	0	0	0	123	0	42	0	0
69	0	0	0	8	0	0	0	0	31	0	0	9	0	0	0	0	0	0
0	0	39	0	47	3	0	0	0	0	12	0	17	10	0	0	19	0	8
0	0	0	88	0	0	0	13	0	22	0	0	67	0	0	16	17	0	0
12	0	14	0	0	0	0	0	23	3	0	0	0	0	0	0	0	0	0
0	0	29	0	0	0	7	0	0	0	0	0	0	0	0	0	0	12	0
0	0	18	26	0	37	0	0	0	15	0	4	0	17	23	0	0	0	44
0	0	0	0	0	0	0	0	0	4	0	0	63	12	0	0	0	30	0

0	0	36	0	0	13	0	0	0	0	10	4	0	0	15	0	13	0	0
11	0	0	0	0	0	0	7	14	0	0	0	0	0	0	0	0	0	0
0	0	23	0	0	0	0	0	0	0	19	0	0	0	6	9	163	0	0
0	10	0	15	0	0	7	6	6	0	0	0	0	14	0	7	0	45	29
6	5	0	0	0	7	0	0	0	0	0	0	0	0	0	0	7	0	0
0	0	5	0	16	0	0	0	0	0	0	0	20	0	0	0	0	0	5
0	0	0	0	0	0	0	0	0	0	0	0	10	0	0	0	0	0	0
0	0	0	0	0	0	12	0	0	15	0	0	0	0	0	0	0	0	0
0	0	0	0	0	0	0	0	0	0	0	0	0	0	19	0	0	0	0
0	4	0	0	0	0	0	0	0	0	0	0	0	0	0	0	0	0	11
0	0	0	0	0	0	0	0	0	0	0	0	0	0	0	0	0	0	0
0	0	0	0	0	0	0	0	29	9	0	0	0	0	0	0	0	0	0
0	0	0	0	0	0	0	0	0	2	0	0	0	0	0	0	0	0	0
0	0	0	0	0	16	0	0	0	0	0	0	0	0	0	16	0	0	0
4	0	0	0	0	0	0	8	0	0	0	0	0	0	13	0	0	0	0
0	0	0	0	0	0	0	5	0	0	0	0	8	0	0	0	0	0	0
0	0	0	0	0	0	12	0	0	0	0	0	0	0	0	0	0	0	0
0	0	0	0	0	0	0	0	0	0	0	18	0	0	0	0	0	0	0
0	0	7	0	0	0	0	0	0	0	0	0	0	0	0	0	0	0	0
12	0	0	0	0	0	0	0	0	0	19	0	0	0	0	0	0	0	0
0	0	7	0	0	5	0	0	0	8	0	0	0	0	0	0	0	0	0
0	0	0	0	0	0	0	0	0	0	0	0	0	0	0	0	0	0	0
0	0	0	0	0	0	0	0	0	0	0	0	0	0	0	0	0	0	0
0	8	0	0	0	0	0	0	0	0	0	0	0	0	8	8	0	0	0
0	0	0	90	0	0	0	0	0	0	0	0	0	0	0	0	0	0	0
4	0	0	0	0	0	0	0	0	0	0	0	11	0	0	0	0	0	0
0	0	0	0	0	0	0	0	0	0	0	0	0	0	0	0	0	0	0
0	0	0	0	0	0	16	0	0	0	0	0	0	0	9	0	0	0	0
0	0	0	0	0	0	59	0	0	0	0	0	0	0	0	0	0	0	0
0	0	0	0	0	0	0	0	0	0	0	0	0	0	0	0	0	0	0
0	0	0	0	0	0	0	0	0	0	0	0	0	0	0	0	0	0	58
0	0	0	0	0	0	0	0	0	0	0	0	0	0	0	0	0	0	0
0	0	0	0	0	0	0	0	0	0	0	0	0	0	0	0	0	0	0
0	0	0	0	0	0	0	0	0	0	0	0	0	0	0	0	0	0	0
0	0	0	0	0	0	0	0	0	0	0	0	0	0	0	0	0	0	0
0	0	0	0	13	0	0	0	0	0	0	0	0	0	0	0	0	0	0
0	0	0	0	0	0	0	0	0	0	0	0	0	0	0	0	0	0	0

0	0	0	0	0	0	0	0	0	0	0	0	0	0	0	0	13	0	0
0	0	0	0	0	0	0	0	0	0	0	0	0	0	0	0	0	0	0
0	0	0	0	0	0	2	0	0	0	0	0	0	10	0	0	0	0	0
0	0	0	0	0	0	0	0	0	0	0	0	0	0	0	0	0	0	0
0	0	19	0	0	0	0	0	0	0	0	0	0	0	0	0	0	0	0
0	0	0	0	0	0	0	0	0	0	0	0	0	0	0	0	0	0	0
0	0	0	0	0	0	0	0	0	0	0	0	0	0	0	0	0	0	0
0	0	0	4	15	0	0	0	0	0	0	0	0	0	0	0	0	0	0
0	0	0	0	0	6	0	0	0	0	0	0	0	0	0	0	0	0	0
0	0	0	0	0	0	0	0	0	0	0	0	0	0	0	0	0	0	0
0	0	0	0	0	0	0	0	0	0	0	0	0	0	0	0	0	0	0
0	0	0	0	0	0	0	0	0	0	0	0	0	0	0	0	0	0	0
0	0	0	0	0	0	0	0	0	0	0	0	0	0	0	0	0	0	0
0	0	0	0	0	0	0	0	0	0	0	0	0	0	0	0	0	0	0
0	0	0	0	0	0	0	0	0	0	0	0	0	0	0	0	0	0	0
0	0	0	0	0	0	0	0	0	0	0	0	0	0	0	0	0	0	0
0	0	0	0	0	0	0	0	0	0	0	0	0	0	0	0	0	0	0
0	0	0	0	0	0	0	0	0	0	0	0	0	0	0	0	0	0	0
0	0	0	0	0	0	0	0	0	0	0	0	0	0	0	0	0	0	0
0	0	0	0	0	0	0	0	0	0	0	0	0	0	0	0	0	0	0
0	0	0	0	0	0	0	0	0	0	0	0	0	0	0	0	0	0	0
0	0	0	0	0	0	0	0	0	0	0	0	0	0	0	0	0	0	0
0	0	0	0	0	0	0	0	0	0	0	0	0	0	0	0	0	0	0
0	0	0	0	0	0	0	0	0	0	0	0	0	0	0	0	0	0	0
0	0	0	0	0	0	0	0	0	0	0	0	0	0	0	0	0	0	0
0	0	0	0	0	0	0	0	0	0	0	0	0	0	0	0	0	0	0
0	0	0	0	0	0	0	0	0	0	0	0	0	0	0	0	0	0	0
0	0	0	0	0	0	0	0	0	0	0	0	0	0	0	0	0	0	0
0	0	0	0	0	0	0	0	0	0	0	0	10	0	0	0	0	0	0
0	0	0	0	0	0	0	0	0	0	0	0	0	0	0	0	0	0	0
0	0	0	0	0	0	0	0	0	0	0	0	0	0	0	0	0	0	0
0	0	0	0	0	0	0	0	0	0	0	0	0	0	0	0	0	0	0
0	0	0	0	0	0	0	0	0	0	0	0	0	0	0	0	0	0	0
0	0	0	0	0	0	0	0	0	0	0	0	0	0	0	0	0	0	0
0	0	0	0	0	0	0	0	0	0	0	0	0	0	0	0	0	0	0
0	0	0	0	0	0	0	0	0	0	0	0	0	0	0	0	0	0	0
0	0	0	0	0	0	0	0	0	0	8	8	0	0	0	0	0	0	0

0	0	0	0	0	0	0	0	0	0	0	0	0	0	0	0	0	0	0
0	0	0	0	0	0	0	0	0	0	0	0	0	0	0	0	0	0	0
0	0	0	0	0	0	0	0	0	0	0	0	0	0	0	0	0	0	0
0	0	0	0	0	0	0	0	0	0	0	0	0	0	0	0	0	0	0
0	0	0	0	0	0	0	0	0	0	0	0	0	0	0	7	7	7	0
0	0	0	0	0	0	0	0	0	0	0	0	0	0	0	0	0	0	0
0	0	0	0	0	0	0	0	0	0	0	0	0	0	0	0	0	0	0
0	0	0	0	0	0	0	0	0	0	0	0	0	0	0	0	0	0	0
0	0	0	0	0	0	0	0	0	0	0	0	0	0	0	0	0	0	0
0	0	0	0	0	0	0	0	0	0	0	0	0	0	0	0	0	0	0
0	0	0	0	0	0	0	0	0	0	0	0	0	0	0	0	0	0	0
0	0	0	0	0	0	0	0	0	0	0	0	0	0	0	6	0	0	0
0	0	0	0	0	0	0	0	0	0	0	0	0	0	0	0	0	0	0
0	0	0	0	0	0	0	0	0	0	0	0	0	0	0	0	0	0	0
0	0	0	0	0	0	0	0	0	0	0	0	0	0	0	0	0	0	0
0	0	0	0	0	0	0	0	0	0	0	0	0	0	0	0	0	0	0
0	0	0	0	0	0	0	0	0	0	0	0	0	0	0	0	0	0	0
0	0	0	0	0	0	0	0	0	0	0	0	0	0	0	0	0	0	0
0	0	0	0	0	0	0	0	0	0	0	0	0	0	0	0	0	0	0
0	0	0	0	0	0	0	0	0	0	0	0	0	0	0	0	0	0	0
0	0	0	0	0	0	5	5	5	0	0	0	0	0	0	0	0	0	0
0	0	0	0	0	0	0	0	0	0	0	0	0	0	0	0	0	0	0
0	0	0	0	0	0	0	0	0	0	0	0	0	0	0	0	0	0	0
0	0	0	0	0	0	0	0	0	0	0	0	0	0	0	0	0	0	0
0	0	0	0	0	0	0	0	0	0	0	0	0	0	0	0	0	0	0
0	0	0	0	0	0	0	0	0	0	0	0	0	0	0	0	0	0	0
0	0	0	0	0	0	0	0	0	0	0	0	0	0	0	0	0	0	0
0	0	0	0	0	0	0	0	0	0	0	0	0	0	0	0	0	0	0
0	0	0	0	0	0	0	0	0	0	0	0	0	0	0	0	0	0	0
0	0	0	0	0	0	0	0	0	0	0	0	0	0	0	0	0	0	0
0	0	0	0	0	0	0	0	0	0	0	0	0	4	4	0	0	0	0
0	0	0	0	0	0	0	0	0	0	0	0	0	0	0	0	0	0	0
0	0	0	0	0	0	0	0	0	0	0	0	0	0	0	0	0	0	0
0	0	0	0	0	0	0	0	0	0	0	0	0	0	0	0	0	0	0
0	0	0	0	0	0	0	0	0	0	0	0	0	0	0	0	0	0	0
0	0	0	0	0	0	0	0	0	0	0	0	0	0	0	0	0	0	0
0	0	0	0	0	0	0	0	0	0	0	0	0	0	0	0	0	0	0

0	0	0	0	0	0	0	0	0	0	0	0	0	0	0	0	0	0	0
0	0	0	0	0	0	0	0	0	0	0	0	0	0	0	0	0	0	0
0	0	0	0	0	0	0	0	0	0	0	0	0	0	0	0	0	0	0
0	0	0	0	0	0	0	0	0	0	0	0	0	0	0	0	0	0	0
0	0	0	0	0	0	0	0	0	0	0	0	0	0	0	0	0	0	0
0	0	0	0	0	0	0	0	0	0	0	0	0	0	0	0	0	0	0
0	0	0	0	0	0	0	0	0	0	0	0	0	0	0	0	0	0	0
0	0	0	0	0	0	0	0	0	0	0	0	0	0	0	0	0	0	0
0	0	0	0	0	0	0	0	0	0	0	0	0	0	0	0	0	0	0
0	0	0	0	0	0	0	0	0	0	0	0	0	0	0	0	0	0	0
0	0	0	0	0	0	0	3	3	3	3	3	0	0	0	0	0	0	0
0	0	0	0	0	0	0	0	0	0	0	0	0	0	0	0	0	0	0
0	0	0	0	0	0	0	0	0	0	0	0	0	0	0	0	0	0	0
0	0	0	0	0	0	0	0	0	0	0	0	0	0	0	0	0	0	0
0	0	0	0	0	0	0	0	0	0	0	0	0	0	0	0	0	0	0
0	0	0	0	0	0	0	0	0	0	0	0	0	0	0	0	0	0	0
0	0	0	0	0	0	0	0	0	0	0	0	0	0	0	0	0	0	0
0	0	0	0	0	0	0	0	0	0	0	0	0	0	0	0	0	0	0
0	0	0	0	0	0	0	0	0	0	0	0	0	0	0	0	0	0	0
0	0	0	0	0	0	0	0	0	0	0	0	0	0	0	0	0	0	0
0	0	0	0	0	0	0	0	0	0	0	0	0	0	0	0	0	0	0
0	0	0	0	0	0	0	0	0	0	0	0	0	0	0	0	0	0	0
0	0	0	0	0	0	0	0	0	0	0	0	0	0	0	0	0	0	0
0	0	0	0	0	0	0	0	0	0	0	0	0	0	0	0	0	0	0
0	0	0	0	0	0	0	0	0	0	0	0	0	0	0	0	0	0	0
0	0	0	0	0	0	0	0	0	0	0	0	0	0	0	0	0	0	0
0	0	0	0	0	0	0	0	0	0	0	0	0	0	0	0	0	0	0
0	0	0	0	0	0	0	0	0	0	0	0	0	0	0	0	0	0	0
0	0	0	0	0	0	0	0	0	0	0	0	0	0	0	0	0	0	0
0	0	0	0	0	0	0	0	0	0	0	0	0	0	0	0	0	0	0
0	0	0	0	0	0	0	0	0	0	0	0	0	0	0	0	0	0	0
0	0	0	0	0	0	0	0	0	0	0	0	0	0	0	0	0	0	0
0	0	0	0	0	0	0	0	0	0	0	0	0	0	0	0	0	0	0
0	0	0	0	0	0	0	0	0	0	0	0	0	0	0	0	0	0	0
0	0	0	0	0	0	0	0	0	0	0	0	0	0	0	0	0	0	0
0	0	0	0	0	0	0	0	0	0	0	0	0	0	0	0	0	0	0
0	0	0	0	0	0	0	0	0	0	0	0	0	0	0	0	0	0	0

0	0	0	0	0	2	2	2	2	0	0	0	0	0	0	0	0	0	0
0	0	0	0	0	0	0	0	0	0	0	0	0	0	0	0	0	0	0
0	0	0	0	0	0	0	0	0	0	0	0	0	0	0	0	0	0	0
0	0	0	0	0	0	0	0	0	0	0	0	0	0	0	0	0	0	0
0	0	0	0	0	0	0	0	0	0	0	0	0	0	0	0	0	0	0
0	0	0	0	0	0	0	0	0	0	0	0	0	0	0	0	0	0	0
0	0	0	0	0	0	0	0	0	0	0	0	0	0	0	0	0	0	0
0	0	0	0	0	0	0	0	0	0	0	0	0	0	0	0	0	0	0
0	0	0	0	0	0	0	0	0	0	0	0	0	0	0	0	0	0	0
0	0	0	0	0	0	0	0	0	0	0	0	0	0	0	0	0	0	0
0	0	0	0	0	0	0	0	0	0	0	0	0	0	0	0	0	0	0
0	0	0	0	0	0	0	0	0	0	0	0	0	0	0	0	0	0	0
0	0	0	0	0	0	0	0	0	0	0	0	0	0	0	0	0	0	0
0	0	0	0	0	0	0	0	0	0	0	0	0	0	0	0	0	0	0
0	0	0	0	0	0	0	0	0	0									
Jan_DL156	1789	1659	453	426	266	479	442	440	871	113	247	310	305	136	76	98	97	110
341	174	190	0	180	158	80	232	77	155	199	96	185	91	112	108	192	56	95
118	128	135	326	95	77	190	0	135	25	150	66	49	115	143	41	0	115	0
50	26	49	31	128	75	80	38	27	20	22	0	0	64	5	14	50	21	55
29	0	110	68	47	32	65	106	0	0	148	67	0	35	47	0	17	18	49
77	15	105	0	56	35	0	0	0	6	0	32	0	8	12	7	0	0	0
70	15	21	10	0	23	0	0	0	72	0	0	19	14	0	0	31	0	0
79	9	0	0	0	0	0	0	0	7	0	0	7	0	13	0	0	0	0
0	10	0	0	56	10	0	0	0	0	0	0	23	142	21	0	19	0	0
0	0	20	19	0	0	0	15	0	0	0	6	0	0	0	17	0	0	0
0	0	0	0	0	0	0	0	0	0	0	0	0	0	3	0	0	0	0
5	0	0	0	0	0	12	0	0	0	0	0	0	0	0	0	0	32	0
0	0	0	0	0	20	0	0	24	0	0	0	20	27	0	16	6	0	19
12	0	0	0	0	0	0	0	0	0	0	0	0	0	0	0	0	0	0
13	0	45	0	0	0	5	0	0	0	0	0	0	0	0	0	0	0	0
0	0	0	0	0	0	0	0	0	0	0	12	5	0	0	0	15	0	0
0	0	0	0	0	7	0	0	0	0	14	0	0	0	0	7	0	0	0
8	0	7	0	0	0	0	3	0	0	0	0	0	0	0	0	0	0	0
0	0	0	0	6	8	0	0	0	0	0	0	0	0	0	0	0	0	0
0	0	0	0	0	0	2	0	0	0	0	0	0	0	0	0	0	0	0
0	0	0	0	0	0	0	0	0	0	0	0	0	0	0	0	0	0	0
0	0	0	0	0	0	0	0	0	0	0	0	0	0	0	0	0	0	0

0	0	0	0	0	0	0	0	2	61	0	0	0	0	0	0	5	0	0
0	0	0	0	0	9	0	0	0	0	0	0	0	0	0	0	0	0	0
0	0	0	0	0	0	0	0	0	0	0	0	0	0	0	0	0	0	0
0	0	0	14	0	0	0	0	0	2	0	0	0	0	0	0	0	0	0
0	0	0	0	0	9	0	17	0	11	0	0	0	0	0	0	0	0	11
0	0	0	0	0	0	0	0	0	0	0	0	0	0	0	0	0	0	0
0	0	0	0	0	0	0	0	0	0	0	0	0	0	0	0	0	0	0
0	0	0	0	0	0	0	3	0	0	0	0	0	0	0	0	0	0	0
0	0	0	0	0	0	0	0	0	0	0	0	0	0	0	0	0	0	0
0	9	0	0	0	0	0	0	0	8	0	0	0	0	0	0	0	0	0
0	0	0	0	0	0	0	0	0	0	0	0	0	0	0	0	0	0	0
0	0	0	0	0	0	0	10	0	0	0	0	0	0	0	0	0	0	0
0	0	0	0	0	0	0	0	0	0	0	0	0	0	0	0	0	0	0
0	0	0	0	0	0	0	0	0	0	0	0	0	0	0	0	0	0	0
0	0	0	0	0	0	0	0	0	0	0	0	0	0	0	0	0	0	0
0	0	0	0	0	0	0	0	0	0	0	0	0	0	0	0	0	0	0
0	0	0	0	0	0	0	0	0	0	0	0	0	0	0	0	0	0	0
0	0	0	0	0	0	0	0	0	0	0	0	0	0	0	0	0	0	0
0	0	0	0	0	0	0	0	0	0	0	0	0	0	0	0	0	0	0
0	0	0	0	0	0	0	0	0	0	0	0	0	0	0	0	0	0	0
0	0	0	0	0	0	0	0	0	0	0	0	0	0	0	0	0	0	0
0	0	0	0	0	0	0	0	0	0	0	0	0	0	0	0	0	0	0
0	0	0	0	0	0	0	0	0	0	0	0	0	0	0	0	0	0	0
0	0	0	0	0	0	0	0	0	0	0	0	0	0	0	0	0	0	6
0	0	0	0	0	0	0	0	0	0	0	0	0	0	0	0	0	0	0
0	0	0	0	0	0	0	0	0	0	0	0	0	0	0	0	0	0	0
0	0	0	0	0	0	0	0	0	0	0	0	0	0	0	0	0	0	0
0	0	0	0	0	0	0	0	0	0	0	0	0	0	0	0	0	0	0
0	0	0	0	0	0	0	0	0	0	0	0	0	0	0	0	0	0	0
0	0	0	0	0	0	0	0	0	0	0	0	0	0	0	0	0	0	0
0	0	0	0	0	0	0	0	0	0	0	0	0	0	0	0	0	0	0
0	0	0	0	0	0	0	0	0	0	0	0	0	0	0	0	0	0	0
0	0	0	0	0	0	0	0	0	0	0	0	0	0	0	0	0	0	0
0	0	0	0	0	0	0	0	0	0	0	0	0	0	0	0	0	0	0
0	0	0	0	0	0	0	0	0	0	0	0	0	0	0	0	0	0	0
0	0	0	0	0	0	0	0	0	0	0	0	0	0	0	0	0	0	0
0	0	0	0	0	0	0	0	0	0	0	0	0	0	0	0	0	0	0

0	0	0	0	0	0	0	0	0	0	0	21	0	0	0	0	0	0	0
0	0	0	0	0	3	0	0	0	0	0	0	0	0	0	0	0	0	0
0	0	0	0	0	0	0	0	0	0	0	0	0	0	0	0	0	0	0
0	0	0	0	0	0	0	0	0	0	0	0	0	0	0	0	0	0	0
0	0	0	0	0	0	0	0	0	0	0	0	0	0	0	0	0	0	0
0	0	0	0	0	0	0	0	0	0	0	0	0	0	0	0	0	0	0
0	0	0	0	0	0	0	0	0	0	0	0	0	0	0	0	0	0	0
0	0	0	0	0	0	0	0	0	0	0	0	0	0	0	0	0	0	0
0	0	0	0	0	0	0	0	0	0	0	0	0	0	0	0	0	0	0
0	0	0	0	0	0	0	0	0	0	0	0	0	0	0	0	0	0	0
0	0	0	0	0	0	0	0	0	0	0	0	0	0	0	0	0	0	0
0	0	0	0	0	0	0	0	0	0	0	0	0	0	0	0	0	0	0
0	0	0	0	0	0	0	0	0	0	0	0	0	0	0	0	0	0	0
0	0	0	0	0	0	0	0	0	0	0	0	0	0	0	0	0	0	0
0	0	0	0	0	0	0	0	0	0	0	0	0	0	0	0	11	0	0
0	0	0	0	0	0	0	0	0	0	0	0	0	0	0	0	0	0	0
0	0	0	0	0	0	0	0	0	0	0	0	0	0	0	0	0	0	0
0	0	0	0	0	0	0	0	0	0	0	0	0	0	0	0	0	0	0
0	0	0	0	0	0	0	0	0	0	0	0	0	0	0	0	0	0	0
0	0	0	0	0	0	0	0	0	0	0	0	0	0	0	0	0	0	0
0	0	0	0	0	0	0	0	0	0	0	0	0	0	0	0	0	0	0
0	0	0	0	9	9	9	0	0	0	0	0	0	0	0	0	0	0	0
0	0	0	0	0	0	0	0	0	0	0	0	0	0	0	0	0	0	0
0	0	0	0	0	0	0	0	0	0	0	0	0	0	0	0	0	0	0
0	0	0	0	0	0	0	0	0	0	0	0	0	0	0	0	0	0	0
0	0	0	0	0	0	0	8	0	0	0	0	0	0	0	0	0	0	0
0	0	0	0	0	0	0	0	0	0	0	0	0	0	0	0	0	0	0
0	0	0	0	0	0	0	0	0	0	0	0	0	0	0	0	0	0	0
0	0	0	0	0	0	0	0	0	0	0	0	0	0	0	0	0	0	0
0	0	0	0	0	0	0	0	0	0	0	0	0	0	0	0	0	0	0
0	0	0	0	0	0	0	0	0	0	0	0	0	0	0	0	0	0	0
0	0	0	0	0	0	0	0	0	0	0	0	0	0	0	0	0	0	0
0	0	0	0	0	0	0	0	0	0	0	0	0	0	0	0	0	0	0
0	0	0	0	0	0	0	0	0	0	0	0	0	0	0	0	0	0	0
0	0	0	0	0	0	0	0	0	0	0	0	0	0	0	0	0	0	0
0	0	0	0	0	0	0	0	0	0	0	0	0	0	0	0	0	0	0
0	0	0	0	0	0	0	0	0	0	0	3	0	0	0	0	0	0	0

6	0	0	0	0	0	0	0	0	0	0	0	0	0	0	0	0	0	0
0	0	0	0	0	0	0	0	0	0	0	0	0	0	0	0	0	0	0
0	0	0	0	0	0	0	0	0	0	0	0	0	0	0	0	0	0	0
0	0	0	0	0	0	0	0	0	0	0	0	0	0	0	0	0	0	0
0	0	0	0	0	0	0	0	0	0	0	0	0	0	0	0	0	0	0
0	0	0	0	0	0	0	0	0	0	0	0	0	0	0	0	0	0	0
0	0	0	0	0	0	0	0	0	0	0	0	0	0	0	0	0	0	0
0	0	0	0	0	0	0	0	0	0	0	0	0	0	0	0	0	0	0
0	0	0	0	0	0	0	0	0	0	0	0	0	0	0	0	0	0	0
0	0	0	0	0	0	0	0	0	0	0	0	0	0	0	0	0	0	0
0	0	0	0	0	0	0	0	0	0	0	0	0	0	0	0	0	0	0
0	0	0	0	0	0	0	0	0	0	0	0	0	0	0	0	0	0	0
0	0	0	0	0	0	0	0	0	0	0	0	0	0	0	0	0	0	0
0	0	0	0	0	0	0	0	0	0	0	0	0	0	0	0	0	0	0
0	0	0	0	0	0	0	0	0	0	0	0	0	0	0	0	0	0	0
0	0	0	0	0	0	0	0	0	0	0	0	0	0	0	0	0	0	0
0	0	0	0	0	0	0	0	0	0	0	0	0	0	0	0	0	0	0
0	0	0	0	0	0	0	0	0	0	0	0	0	0	0	0	0	0	0
0	0	0	0	0	0	0	0	0	0	0	0	0	0	0	0	0	0	4
0	4	4	0	0	0	0	0	0	0	0	0	0	0	0	0	0	0	0
0	0	0	0	0	0	0	0	0	0	0	0	0	0	0	0	0	0	0
0	0	0	0	0	0	0	0	0	0	0	0	0	0	0	0	0	0	0
0	0	0	0	0	0	0	0	0	0	0	0	0	0	0	0	0	0	0
0	0	0	0	0	0	0	0	0	0	0	0	0	0	0	0	0	0	0
0	0	0	0	0	0	0	0	0	0	0	0	0	0	0	0	0	0	0
0	0	0	0	0	0	0	0	0	0	0	0	0	0	0	0	0	0	0
0	0	0	0	0	0	0	0	0	0	0	0	0	0	0	0	0	0	0
0	0	0	0	0	0	0	0	0	0	0	0	0	0	0	0	0	0	0
0	0	0	0	0	0	0	0	0	0	0	0	0	0	0	0	0	0	0
0	0	0	0	0	0	0	0	0	0	0	0	0	0	0	0	0	0	0
0	0	0	0	0	0	0	0	0	0	0	0	0	0	0	0	0	0	0
0	0	0	0	0	0	0	0	0	0	0	0	0	0	0	0	0	0	0
0	0	0	0	0	0	0	0	0	0	0	0	0	0	0	0	0	0	0
0	0	0	0	0	0	0	0	0	0	0	0	0	0	0	0	0	0	0
0	0	0	0	0	0	0	0	0	0	0	0	0	0	0	0	0	0	0
0	0	0	0	0	0	0	0	0	0	0	0	0	0	0	0	0	3	0
0	0	0	0	0	0	0	0	0	0	0	0	0	0	0	0	0	0	0

0	0	0	0	0	0	0	0	0	0	0	0	0	0	0	0	0	0	0
0	0	0	0	0	0	0	0	0	0	0	0	0	0	0	0	0	0	0
0	0	0	0	0	0	0	0	0	0	0	0	0	0	0	0	0	0	0
0	0	0	0	0	0	0	0	0	0	0	0	0	0	0	0	0	0	0
0	0	0	0	0	0	0	0	0	0	0	0	0	0	0	0	0	0	0
0	0	0	0	0	0	0	0	0	0	0	0	0	0	0	0	0	0	0
0	0	0	0	0	0	0	0	0	0	0	0	0	0	0	0	0	0	0
0	0	0	0	0	0	0	0	0	0	0	0	0	0	0	0	0	0	0
0	0	0	0	0	0	0	0	0	0	0	0	0	0	0	0	0	0	0
0	0	0	0	0	0	0	0	0	0	0	0	0	0	0	0	0	0	0
0	0	0	0	0	0	0	0	0	0	0	0	0	0	0	0	0	0	0
0	0	0	0	0	0	0	0	0	0	0	0	0	0	0	0	0	0	0
0	0	0	0	0	0	0	0	0	0	0	0	0	0	0	0	0	0	0
0	0	0	0	0	0	0	0	0	0	0	0	0	0	0	0	0	0	0
0	0	0	0	0	0	0	0	0	0	0	0	0	0	0	0	0	0	0
0	0	0	0	0	0	0	0	0	0	0	0	0	0	0	0	0	0	0
0	0	0	0	0	0	0	0	0	0	0	0	0	0	0	0	0	0	0
0	0	0	0	0	0	0	0	0	0	0	0	0	0	0	0	0	0	0
0	0	0	0	0	0	0	0	0	0	0	0	0	0	0	0	0	0	0
0	0	0	0	0	0	0	0	0	0	0	0	0	0	0	0	0	0	0
0	0	0	0	0	0	0	0	0	0	0	0	0	0	0	0	0	0	0
0	0	0	0	0	0	0	0	0	0	0	0	0	0	0	0	0	0	0
0	0	0	0	0	0	0	0	0	0	0	0	0	0	0	0	0	0	0
0	0	0	0	0	0	0	0	0	0	0	0	0	0	0	0	0	0	0
0	0	0	0	0	0	0	0	0	0	0	0	0	0	0	0	0	0	0
0	0	0	0	0	0	0	0	0	2	2	2	2	2	2	2	2	0	0
0	0	0	0	0	0	0	0	0	0	0	0	0	0	0	0	0	0	0
0	0	0	0	0	0	0	0	0	0	0	0	0	0	0	0	0	0	0
0	0	0	0	0	0	0	0	0	0	0	0	0	0	0	0	0	0	0
0	0	0	0	0	0	0	0	0	0	0	0	0	0	0	0	0	0	0
0	0	0	0	0	0	0	0	0	0	0	0	0	0	0	0	0	0	0
0	0	0	0	0	0	0	0	0	0	0	0	0	0	0	0	0	0	0
0	0	0	0	0	0	0	0	0	0	0	0	0	0	0	0	0	0	0
0	0	0	0	0	0	0	0	0	0	0	0	0	0	0	0	0	0	0
0	0	0	0	0	0	0	0	0	0	0	0	0	0	0	0	0	0	0
0	0	0	0	0	0	0	0	0	0	0	0	0	0	0	0	0	0	0
0	0	0	0	0	0	0	0	0	0	0	0	0	0	0	0	0	0	0

0	0	0	0	0	0	0	0	0	0	0	0	0	0	0	0	0	0	0
0	0	0	0	0	0	0	0	0	0	0	0	0	0	0	0	0	0	0
0	0	0	0	0	0	0	0	0	0	0	0	0	0	0	0	0	0	0
0	0	0	0	0	0	0	0	0	0	0	0	0	0	0	0	0	0	0
0	0	0	0	0	0	0	0	0	0	0	0	0	0	0	0	0	0	0
0	0	0	0	0	0	0	0	0	0	0	0	0	0	0	0	0	0	0
0	0	0	0	0	0	0	0	0	0									
Jan_DL157	2021	1694	762	745	385	356	374	330	488	230	355	285	283	582	209	179	629	212
253	516	147	18	68	102	92	254	77	245	214	231	174	174	103	178	185	31	147
255	45	180	61	126	167	134	0	130	78	282	45	61	64	152	32	0	169	134
113	0	30	53	202	50	41	63	53	69	0	95	3	36	0	20	63	46	0
40	0	80	18	88	41	22	81	0	71	136	61	21	0	48	0	34	76	41
56	19	101	0	0	13	0	0	0	17	0	73	8	0	14	71	14	0	0
0	30	0	33	0	23	0	0	0	0	7	0	20	11	0	0	21	0	0
0	24	0	0	43	0	0	0	0	8	0	0	14	69	26	0	0	0	0
0	27	0	0	0	6	0	0	0	0	15	0	20	8	0	0	4	0	43
0	0	9	0	0	0	0	54	0	0	0	0	53	0	18	0	3	0	60
0	0	31	0	0	0	0	0	0	6	0	0	0	0	0	0	0	0	0
0	20	0	0	0	0	46	0	0	20	0	0	0	0	0	0	0	25	0
0	0	0	0	0	14	0	0	0	0	0	0	14	10	0	0	13	0	0
0	0	0	0	0	0	0	0	0	0	0	0	0	0	0	0	0	0	0
17	0	33	24	0	17	0	0	0	0	0	3	0	0	2	0	17	0	0
0	0	0	0	0	0	0	0	0	0	0	15	7	0	23	0	0	10	0
0	0	0	0	0	0	0	0	0	0	21	0	0	3	0	21	0	0	0
0	9	8	11	0	0	0	7	4	0	0	0	0	19	0	9	0	0	0
9	0	0	0	0	0	0	34	0	0	0	0	0	0	0	0	0	0	0
0	0	0	0	0	0	4	0	0	0	0	0	0	0	20	0	0	0	10
0	0	0	0	0	0	0	0	0	0	0	0	0	0	0	0	0	0	0
0	0	0	0	0	0	36	0	0	0	0	0	0	0	0	0	0	0	0
0	0	0	0	0	0	24	0	0	0	0	0	3	0	0	0	0	0	0
0	12	0	0	0	0	0	0	0	0	0	0	0	0	0	0	0	0	10
0	0	0	0	0	0	0	17	0	0	0	8	0	0	0	0	0	0	0
0	0	0	0	0	0	0	0	0	0	0	0	0	0	0	0	0	0	0
0	0	0	0	0	0	0	0	0	6	0	0	0	0	0	0	8	6	43
0	0	0	0	0	0	0	0	0	0	0	0	0	0	0	0	0	0	0
0	0	0	0	0	0	0	0	0	7	0	6	0	0	0	0	0	0	0
0	4	0	0	0	0	0	0	0	0	0	10	0	0	0	0	0	0	0

0	0	0	0	0	0	0	0	0	0	0	0	0	0	0	0	0	0	0
0	0	0	0	0	0	0	0	0	0	0	0	0	0	0	0	0	0	0
0	0	0	0	0	0	0	0	0	0	0	0	0	0	0	0	0	0	0
0	6	0	0	0	0	0	0	0	0	0	0	0	0	0	0	6	0	0
0	0	0	0	0	0	0	0	0	0	0	0	0	0	0	0	0	5	0
0	0	0	0	0	0	0	0	0	6	6	0	0	0	0	0	0	0	13
0	0	0	0	0	0	0	0	0	0	0	0	0	0	0	0	0	21	0
0	0	0	0	0	0	0	0	0	0	0	0	0	0	0	0	0	16	0
11	0	0	0	0	0	0	27	0	0	0	0	0	0	0	0	0	0	0
0	0	0	0	0	0	0	0	0	16	0	0	0	0	0	33	0	0	0
0	0	21	0	0	0	0	0	0	0	0	0	0	0	0	0	0	0	0
0	6	0	0	0	0	0	0	0	0	0	0	0	0	0	0	0	0	0
0	0	0	0	0	0	0	0	0	0	0	0	0	0	0	0	0	0	0
0	0	0	0	0	0	0	0	0	0	0	0	0	0	0	0	0	0	0
0	0	0	0	0	0	0	0	0	0	0	0	0	0	0	0	0	0	0
0	0	0	0	0	0	0	0	0	0	0	0	0	0	0	0	0	0	0
0	0	0	0	0	0	0	0	0	0	0	0	0	0	0	0	0	0	0
0	0	0	0	0	0	0	0	0	0	0	0	0	0	0	0	0	0	0
0	0	0	0	0	0	0	0	0	0	0	0	0	0	0	0	0	0	0
0	0	0	0	0	0	0	0	0	0	0	0	0	0	0	0	0	0	0
0	0	0	0	0	0	0	0	0	0	0	0	0	0	0	0	0	0	0
0	0	0	0	0	0	0	0	0	0	0	0	0	0	0	0	0	0	0
0	0	0	0	0	0	0	0	0	0	0	0	0	0	0	0	0	0	0
0	0	0	0	0	0	0	0	0	0	0	0	0	0	0	0	0	0	0
0	0	0	7	0	0	7	0	0	0	0	0	0	0	0	0	0	0	0
0	0	0	0	0	0	0	0	0	0	0	0	0	7	0	0	0	0	0
0	0	0	0	0	0	0	0	0	0	0	0	0	0	0	0	0	0	0
0	0	0	0	0	0	0	0	0	0	0	0	0	0	0	0	0	0	0
0	0	0	0	0	0	0	0	0	0	0	0	0	0	0	0	0	0	0
0	0	0	0	0	0	0	0	0	0	0	0	0	0	0	0	0	0	0
0	0	0	0	0	0	0	0	0	0	0	0	0	0	0	0	0	0	0
0	0	0	0	0	0	0	0	0	0	0	0	0	0	0	0	0	0	0
0	0	0	0	0	0	0	0	0	0	0	0	0	0	0	0	0	0	0
0	0	0	0	0	0	0	0	0	0	0	0	0	0	0	0	0	0	0
0	0	0	0	0	0	0	0	0	0	0	0	0	0	0	0	0	0	0
0	3	0	0	0	0	0	0	0	0	0	0	0	0	0	0	0	0	0
0	0	0	0	0	0	0	0	0	0	0	0	0	0	0	0	0	0	0

0	0	0	0	0	0	0	0	0	0	0	0	0	0	0	0	0	0	0
0	0	0	0	0	0	0	0	0	0	0	0	0	0	0	0	0	0	0
0	0	0	0	0	0	0	0	0	0	0	0	0	0	0	0	0	0	0
0	0	0	0	0	0	0	0	0	0	0	0	0	0	0	0	0	0	0
0	0	0	0	0	0	0	0	0	0	0	0	0	0	0	0	0	0	0
0	0	0	0	0	0	0	0	0	0	0	6	0	0	0	0	0	0	0
0	0	0	0	0	0	0	0	0	0	0	0	0	0	0	0	0	0	0
0	0	0	0	0	0	0	0	0	0	0	0	0	0	0	0	0	0	0
0	0	0	0	2	0	0	0	0	0	0	0	0	0	0	0	0	0	0
0	0	0	0	0	0	0	0	0	0	0	0	0	0	0	0	0	0	0
0	0	0	0	0	0	0	0	0	0	0	0	0	0	0	0	0	0	0
0	0	0	0	0	0	0	0	0	0	0	0	0	0	0	0	0	0	0
0	0	0	0	0	0	0	0	0	0	0	0	0	0	0	0	0	0	0
0	0	0	0	0	0	0	0	0	0	0	0	0	0	0	0	0	0	0
0	0	0	0	0	0	0	0	0	0	0	0	0	0	0	0	0	0	0
0	0	0	0	0	0	0	0	0	0	0	0	0	0	0	0	0	0	0
0	0	0	0	0	0	0	0	0	0	0	0	0	0	0	0	0	0	0
0	0	0	0	0	0	0	0	0	0	0	0	0	0	0	0	0	0	0
0	0	0	0	0	0	0	0	0	0	0	0	0	0	0	0	0	0	0
0	0	0	0	0	0	0	0	0	0	0	0	0	0	0	0	0	0	0
0	0	0	0	0	0	0	0	0	0	0	0	0	0	0	0	0	0	0
0	0	0	0	0	0	0	0	0	0	0	0	0	0	0	0	0	0	0
0	0	0	0	0	0	0	0	0	0	0	0	0	0	0	0	0	0	0
0	0	0	0	0	0	0	0	0	0	0	0	0	0	0	0	0	0	0
0	0	0	0	0	0	0	0	0	0	0	0	0	0	0	0	0	0	0
0	0	0	0	0	0	0	0	0	0	0	0	0	0	0	0	0	0	0
0	0	0	0	0	0	0	0	0	0	0	0	0	0	0	0	0	0	0
0	0	0	0	0	0	0	0	0	0	0	0	0	0	0	0	0	0	0
0	0	0	0	0	0	0	0	0	0	0	0	0	0	0	0	0	0	0
0	0	0	0	0	0	0	0	0	0	0	0	0	0	0	0	0	0	0
0	0	0	0	0	0	0	0	0	0	0	0	0	0	0	0	0	0	0
0	0	0	0	0	0	0	0	0	0	0	0	0	0	0	0	0	0	0
0	0	0	0	0	0	0	0	0	0	0	0	0	0	0	0	0	0	0
0	0	0	0	0	0	0	0	0	0	0	0	0	0	0	0	0	0	0
0	0	0	0	0	0	0	0	0	0	0	0	0	0	0	0	0	0	0
0	0	0	0	0	0	0	0	0	0	0	0	0	0	0	0	0	0	0
0	0	0	0	0	0	0	0	0	0	0	0	0	0	0	0	0	0	0

0	0	0	6	0	0	0	0	0	0	0	0	0	0	0	0	0	0	0
0	0	0	0	0	0	0	0	0	0	0	0	0	0	0	0	0	0	0
0	0	0	0	0	0	0	0	0	0	0	0	0	0	0	0	0	0	0
0	0	0	0	0	0	0	0	0	0	0	0	0	0	0	0	0	0	0
0	0	0	0	0	0	0	0	0	0	0	0	0	0	0	0	0	0	0
0	0	0	0	0	0	0	0	0	0	0	0	0	0	0	0	0	0	0
0	0	0	0	0	0	0	0	0	0	0	0	0	0	0	0	0	0	0
0	0	0	0	0	0	0	0	0	0	0	0	0	0	0	0	0	0	0
0	0	0	0	0	0	0	0	0	0	0	0	0	0	0	0	0	0	0
0	0	0	0	0	0	0	0	0	0	0	0	0	0	0	0	0	0	0
0	0	0	0	0	0	0	0	0	0	0	0	0	0	0	0	0	0	0
0	0	0	0	0	0	0	0	0	0	0	0	0	0	0	0	0	0	0
0	0	0	0	0	0	0	0	0	0	0	0	0	0	0	0	0	0	0
0	0	0	0	0	0	0	0	0	0	0	0	0	0	0	0	0	0	0
0	0	0	0	0	0	0	0	0	0	0	0	0	0	0	0	0	0	0
0	0	0	0	0	0	0	0	0	0	0	0	0	0	0	0	0	0	0
0	0	0	0	0	0	0	0	0	0	0	0	0	0	0	0	0	0	0
0	0	0	0	0	0	0	0	0	0	0	0	0	0	0	0	0	0	0
0	0	0	0	0	0	0	0	0	0	0	0	0	0	0	0	0	0	0
0	0	0	0	0	0	0	0	0	0	0	0	0	0	0	0	0	0	0
0	0	0	0	0	0	0	0	0	0	0	0	0	0	0	0	4	0	0
0	0	0	0	0	0	0	0	0	0	0	0	0	0	0	0	0	0	0
0	0	0	0	0	0	0	0	0	0	0	0	0	0	0	0	0	0	0
0	0	0	0	0	0	0	0	0	0	0	0	0	0	0	0	0	0	0
0	0	0	0	0	0	0	0	0	0	0	0	0	0	0	0	0	0	0
0	0	0	0	0	0	0	0	0	0	0	0	0	0	0	0	0	0	0
0	0	0	0	0	0	0	0	0	0	0	0	0	0	0	0	0	0	0
0	0	0	0	0	0	0	0	0	0	0	0	0	0	0	0	0	0	0
0	0	0	0	0	0	0	0	0	0	0	0	0	0	0	0	0	0	0
0	0	0	0	0	0	0	0	0	0	0	0	0	0	0	0	0	0	0
0	0	0	0	0	0	0	0	0	0	0	0	0	0	0	0	0	0	0
0	0	0	0	0	0	0	0	0	0	0	0	0	0	0	0	0	0	0
0	0	0	0	0	0	0	0	0	0	0	0	0	0	0	0	0	0	0
0	0	0	0	0	0	0	0	0	0	0	0	0	0	0	0	0	0	0
0	0	0	0	0	0	0	0	0	0	0	0	0	0	0	0	0	0	0
0	0	0	0	0	0	0	0	0	0	0	0	0	0	0	0	0	0	0
0	0	0	0	0	0	0	0	0	0	0	0	0	0	0	0	0	0	0

0	0	0	0	0	0	0	0	0	0	0	0	0	0	0	0	0	0	0
0	0	0	0	0	0	0	0	0	0	3	0	0	0	0	0	0	0	0
0	0	0	0	0	0	0	0	0	0	0	0	0	0	0	0	0	0	0
0	0	0	0	0	0	0	0	0	0	0	0	0	0	0	0	0	0	0
0	0	0	0	0	0	0	0	0	0	0	0	0	0	0	0	0	0	0
0	0	0	0	0	0	0	0	0	0	0	0	0	0	0	0	0	0	0
0	0	0	0	0	0	0	0	0	0	0	0	0	0	0	0	0	0	0
0	0	0	0	0	0	0	0	0	0	0	0	0	0	0	0	0	0	0
0	0	0	0	0	0	0	0	0	0	0	0	0	0	0	0	0	0	0
0	0	0	0	0	0	0	0	0	0	0	0	0	0	0	0	0	0	0
0	0	0	0	0	0	0	0	0	0	0	0	0	0	0	0	0	0	0
0	0	0	0	0	0	0	0	0	0	0	0	0	0	0	0	0	0	0
0	0	0	0	0	0	0	0	0	0	0	0	0	0	0	0	0	0	0
0	0	0	0	0	0	0	0	0	0	0	0	0	0	0	0	0	0	0
0	0	0	0	0	0	0	0	0	0	0	0	0	0	0	0	0	0	0
0	0	0	0	0	0	0	0	0	0	0	0	0	0	0	0	0	0	0
0	0	0	0	0	0	0	0	0	0	0	0	0	0	0	0	0	0	0
0	0	0	0	0	0	0	0	0	0	0	0	0	0	0	0	0	0	0
0	0	0	0	0	0	0	0	0	0	0	0	0	0	0	0	0	0	0
0	0	0	0	0	0	0	0	0	0	0	0	0	0	0	0	0	0	0
0	0	0	0	0	0	0	0	0	0	0	0	0	0	0	0	0	0	0
0	0	0	0	0	0	0	0	0	0	0	0	0	0	0	0	0	0	0
0	0	0	0	0	0	0	0	0	0	0	0	0	0	0	0	0	0	0
0	0	0	0	0	0	0	0	0	0	0	0	0	0	0	0	0	0	0
0	0	0	0	0	0	0	0	0	0	0	0	0	0	0	0	0	0	0
0	0	0	0	0	0	0	0	0	0	0	0	0	0	0	0	0	0	0
0	0	0	0	0	0	0	0	0	0	0	0	0	0	0	0	0	0	0
0	0	0	0	0	0	0	0	0	0	0	0	0	0	2	2	2	2	0
0	0	0	0	0	0	0	0	0	0	0	0	0	0	0	0	0	0	0
0	0	0	0	0	0	0	0	0	0	0	0	0	0	0	0	0	0	0
0	0	0	0	0	0	0	0	0	0	0	0	0	0	0	0	0	0	0
0	0	0	0	0	0	0	0	0	0	0	0	0	0	0	0	0	0	0
0	0	0	0	0	0	0	0	0	0	0	0	0	0	0	0	0	0	0
0	0	0	0	0	0	0	0	0	0	0	0	0	0	0	0	0	0	0
0	0	0	0	0	0	0	0	0	0	0	0	0	0	0	0	0	0	0
0	0	0	0	0	0	0	0	0	0									

Jan_DL158	1512	1031	522	475	85	441	219	250	533	364	677	76	261	248	50	214	178	212
210	0	87	0	121	124	154	105	96	176	127	137	142	80	168	327	83	49	93
131	33	162	84	101	202	118	24	116	102	0	137	0	0	121	117	90	0	82
67	38	143	19	0	16	68	108	46	22	29	0	0	70	0	30	22	40	27
33	52	83	89	0	41	28	59	0	0	0	0	0	34	0	38	20	0	76
29	30	0	0	39	15	0	0	0	36	0	41	0	0	14	26	0	0	0
0	0	0	18	25	0	0	0	0	0	13	0	0	5	0	0	0	0	0
0	10	0	0	0	0	0	0	0	20	0	0	3	0	25	0	0	0	0
0	0	0	5	0	8	0	0	0	0	0	0	33	0	0	0	0	0	0
0	0	14	0	0	0	0	0	0	0	0	5	74	0	0	13	13	0	0
30	13	0	0	0	0	20	0	62	0	0	0	0	0	0	0	0	0	0
0	0	0	0	0	11	0	0	0	26	0	0	0	0	0	0	0	6	0
0	0	0	0	0	0	0	0	22	0	0	0	0	11	9	0	0	0	17
0	0	0	0	0	0	0	0	0	14	0	0	3	7	0	0	0	0	0
32	0	43	0	0	6	0	0	12	0	0	0	0	6	0	4	0	0	0
5	0	10	0	0	0	0	0	0	0	0	0	0	0	0	0	0	10	0
0	0	9	0	0	0	0	0	0	0	12	0	5	0	0	0	42	0	0
0	0	0	0	0	0	0	20	0	0	0	0	0	0	0	25	0	0	47
0	9	0	0	9	0	0	0	0	0	0	9	0	0	0	0	0	0	0
0	0	0	0	0	0	0	0	0	5	0	0	0	0	0	0	0	0	0
0	0	0	0	0	0	0	0	0	0	0	13	8	0	0	0	0	0	0
0	0	0	0	0	0	0	0	0	0	0	0	0	0	11	0	0	0	0
0	0	0	0	0	0	0	0	0	0	0	0	0	0	0	0	7	0	0
0	0	0	0	0	0	10	0	0	0	0	0	0	0	0	0	0	0	0
0	0	0	0	0	0	0	0	0	0	0	0	0	0	0	0	0	0	0
0	0	7	0	0	0	0	3	0	0	0	0	0	0	0	0	0	0	5
0	0	3	0	0	0	0	6	0	0	0	0	0	0	0	0	0	0	0
0	0	0	0	0	9	0	0	0	0	0	0	0	0	0	0	0	0	0
0	0	0	0	0	0	0	0	0	5	0	0	0	0	0	0	0	0	0
0	0	0	0	0	0	0	0	0	0	5	0	0	0	0	0	0	0	0
0	0	0	0	0	0	0	0	0	0	0	0	0	0	0	0	0	0	10
10	0	0	0	0	0	16	0	0	0	0	49	0	0	0	6	0	0	0
0	0	0	0	0	0	0	16	0	0	0	0	0	0	0	0	0	0	0
0	0	0	0	0	0	0	0	0	0	0	0	0	0	0	0	0	0	0
0	0	0	0	0	0	0	0	0	0	0	0	0	0	0	0	0	0	0
0	0	0	0	0	0	0	0	0	0	0	0	0	0	0	0	0	0	0
0	0	0	0	0	0	0	0	0	0	0	0	0	0	0	0	0	0	0

0	0	0	0	0	0	0	0	0	0	0	0	0	0	0	0	0	0	0
0	0	0	0	0	0	0	0	0	0	0	0	0	0	0	0	0	0	0
0	0	0	0	0	0	0	0	0	0	0	0	0	0	0	14	0	0	0
0	0	0	0	0	0	0	0	0	0	0	0	0	0	0	0	0	0	0
0	0	0	0	0	0	0	0	0	0	0	0	0	0	0	0	0	0	0
0	0	0	0	0	0	0	0	0	0	0	0	8	0	0	0	0	0	0
0	0	0	0	0	0	0	0	0	0	0	0	0	3	0	0	0	0	0
0	0	0	0	0	0	0	0	0	0	0	0	0	0	0	0	0	0	0
0	0	0	0	0	0	0	0	0	0	0	0	0	0	0	0	0	0	0
0	0	0	0	0	0	0	0	0	0	0	0	0	0	0	0	0	0	0
0	0	0	0	0	0	0	3	0	0	0	0	0	0	0	0	0	0	0
0	0	0	0	0	0	0	0	0	0	0	0	0	0	0	0	0	0	0
0	0	0	1	0	0	0	0	0	0	0	0	0	0	0	0	0	0	0
0	0	0	0	0	0	0	0	0	0	0	0	0	0	0	0	0	0	0
0	0	0	0	0	0	0	0	0	0	0	0	0	0	0	0	0	0	0
0	0	0	0	0	0	0	0	0	0	0	0	0	0	0	0	0	0	0
0	0	8	0	0	0	0	0	0	0	0	0	0	0	0	0	0	0	0
0	0	0	0	0	0	0	0	0	0	0	0	0	0	0	0	0	0	0
0	0	0	0	0	0	0	0	0	0	0	0	0	0	0	0	0	0	0
0	0	0	0	0	0	0	0	0	0	0	0	0	0	0	0	0	0	0
0	0	0	0	0	0	0	0	0	0	0	0	0	0	0	0	0	0	0
0	0	0	0	0	0	0	0	0	0	0	0	0	0	0	0	0	0	0
0	0	0	0	0	0	0	0	0	0	0	0	18	0	0	4	0	0	0
0	0	0	0	0	0	0	0	0	0	0	0	0	0	0	0	0	0	0
0	0	0	0	0	0	0	0	0	0	0	0	0	0	0	0	0	0	0
0	0	0	0	0	0	0	0	0	0	0	0	0	0	0	0	0	0	0
0	0	0	0	0	0	0	0	0	0	0	0	0	0	0	0	0	0	0
0	0	0	0	0	0	0	0	0	0	0	0	0	0	0	0	0	0	0
0	0	0	0	0	0	0	0	0	0	0	0	0	0	0	0	0	0	0
0	0	0	0	0	0	0	0	0	0	0	0	0	0	0	0	0	0	0
0	0	0	0	0	0	0	0	0	0	0	0	0	0	0	0	0	7	0
0	0	0	0	0	0	0	0	0	0	0	0	0	0	0	8	0	0	0
0	0	0	0	0	0	0	0	0	0	0	0	0	0	0	0	0	0	0
0	0	0	0	0	0	0	0	0	0	0	0	0	0	0	0	0	0	0
0	0	0	0	0	0	0	0	0	0	0	0	0	0	0	0	0	0	0
0	0	0	0	0	0	0	0	0	0	0	0	0	0	0	0	0	0	0
0	0	0	0	0	0	0	0	0	0	0	0	0	0	0	0	0	0	0

0	0	0	0	0	0	0	0	0	0	0	0	0	0	0	0	0	0	0
0	0	0	0	0	0	0	0	0	0	0	0	0	0	0	0	0	0	0
0	0	0	0	0	0	0	0	0	0	0	0	0	0	0	0	0	0	0
0	0	0	0	0	0	0	0	0	0	0	0	0	0	0	0	0	0	0
0	0	0	0	0	0	0	0	0	0	0	0	0	0	0	0	0	0	0
0	0	0	0	0	0	0	0	0	0	0	0	0	0	0	0	0	0	0
0	0	0	0	0	0	0	0	0	0	0	0	0	0	0	0	0	0	0
0	0	0	0	0	0	0	0	0	0	0	0	0	0	0	0	0	0	0
0	0	0	0	0	0	0	0	0	0	0	0	0	0	0	0	0	0	0
0	0	0	7	0	0	0	0	0	0	0	0	0	0	0	0	0	0	0
0	0	0	0	0	0	0	0	0	0	0	0	0	0	0	0	0	0	0
0	0	0	0	0	0	0	0	0	0	0	0	0	0	0	0	0	0	0
0	0	0	0	0	0	0	0	0	0	0	0	0	0	0	0	0	0	0
0	0	0	0	0	0	0	0	0	0	0	0	0	0	0	0	0	0	0
0	0	0	0	0	0	0	0	0	0	0	0	0	0	0	0	0	0	0
0	0	0	0	0	0	0	0	0	0	6	6	6	0	0	0	0	0	0
0	0	0	0	0	0	0	0	0	0	0	0	0	0	0	0	0	0	0
0	0	0	0	0	0	0	0	0	0	0	0	0	0	0	0	0	0	0
0	0	0	0	0	0	0	0	0	0	0	0	0	0	0	0	0	0	0
0	0	0	0	0	0	0	0	0	0	0	0	0	0	0	0	0	0	0
0	0	0	0	0	0	0	0	0	0	0	0	0	0	0	0	0	0	0
0	0	0	0	0	0	0	0	0	0	0	0	0	0	0	0	0	0	0
0	0	0	0	0	0	0	0	0	0	0	0	0	0	0	0	0	0	0
0	0	0	0	0	0	0	0	5	0	0	0	0	0	0	0	0	0	0
0	0	0	0	0	0	0	0	0	0	0	0	0	0	0	0	0	0	0
0	0	0	0	0	0	0	0	0	0	0	0	0	0	0	0	0	0	0
0	0	0	0	0	0	0	0	0	0	0	0	0	0	0	0	0	0	0
0	0	0	0	0	0	0	0	0	0	0	0	0	0	0	0	0	0	0
0	0	0	0	0	0	0	0	0	0	0	0	0	0	0	0	0	0	0
0	0	0	0	0	0	0	0	0	0	0	0	0	0	0	0	0	0	0
0	0	0	0	0	0	0	0	0	0	0	0	0	0	0	0	0	0	0
0	0	0	0	0	0	0	0	0	0	0	0	0	0	0	0	0	0	0
0	0	0	0	0	0	0	0	0	0	0	0	0	0	0	0	0	0	0
0	4	0	0	0	0	0	0	0	0	0	0	0	0	0	0	0	0	0
0	0	0	0	0	0	0	0	0	0	0	0	0	0	0	0	0	0	0
0	0	0	0	0	0	0	0	0	0	0	0	0	0	0	0	0	0	0
0	0	0	0	0	0	0	0	0	0	0	0	0	0	0	0	0	0	0

0	0	0	0	0	0	0	0	0	0	0	0	0	0	0	0	0	0	0
0	0	0	0	0	0	0	0	0	0	0	0	0	0	0	0	0	0	0
0	0	0	0	0	0	0	0	0	0	0	0	0	0	0	0	0	0	0
0	0	0	0	0	0	0	0	0	0	0	0	0	0	0	0	0	0	0
0	0	0	0	0	0	0	0	0	0	0	0	0	0	0	0	0	0	0
0	0	0	0	0	0	0	0	0	0	0	0	0	0	0	0	0	0	0
0	0	0	0	0	0	0	0	0	0	0	0	0	0	0	0	0	0	0
0	0	0	0	0	0	0	0	0	0	0	0	0	0	0	0	0	0	0
0	0	0	0	0	0	0	0	0	0	0	0	0	0	0	0	0	0	0
0	0	0	0	0	0	0	0	0	0	0	0	0	0	0	0	0	0	0
0	0	0	0	0	0	0	0	0	0	0	0	0	0	0	0	0	0	0
0	0	0	0	3	3	3	3	0	0	0	0	0	0	0	0	0	0	0
0	0	0	0	0	0	0	0	0	0	0	0	0	0	0	0	0	0	0
0	0	0	0	0	0	0	0	0	0	0	0	0	0	0	0	0	0	0
0	0	0	0	0	0	0	0	0	0	0	0	0	0	0	0	0	0	0
0	0	0	0	0	0	0	0	0	0	0	0	0	0	0	0	0	0	0
0	0	0	0	0	0	0	0	0	0	0	0	0	0	0	0	0	0	0
0	0	0	0	0	0	0	0	0	0	0	0	0	0	0	0	0	0	0
0	0	0	0	0	0	0	0	0	0	0	0	0	0	0	0	0	0	0
0	0	0	0	0	0	0	0	0	0	0	0	0	0	0	0	0	0	0
0	0	0	0	0	0	0	0	0	0	0	0	0	0	0	0	0	0	0
0	0	0	0	0	0	0	0	0	0	0	0	0	0	0	0	0	0	0
0	0	0	0	0	0	0	0	0	0	0	0	0	0	0	0	0	0	0
0	0	0	0	0	0	0	0	0	0	0	0	0	0	0	0	0	0	0
0	0	0	0	0	0	0	0	0	0	0	0	0	0	0	0	0	0	0
0	0	0	0	0	0	0	0	0	0	0	0	0	0	0	0	0	0	0
0	0	0	0	0	0	0	0	0	0	0	0	0	0	0	0	0	0	0
0	0	0	0	0	0	0	0	0	0	0	0	0	0	0	0	0	0	0
0	0	0	0	0	0	0	0	0	0	0	0	0	0	0	0	0	0	0
0	0	0	0	0	0	0	0	0	0	0	0	0	0	0	0	0	0	0
0	0	0	0	0	0	0	0	0	0	0	0	0	0	0	0	0	0	0
0	0	0	0	0	0	0	0	0	0	0	0	0	0	0	0	0	0	0
0	0	0	0	0	0	0	0	0	0	0	0	0	0	0	0	0	0	0
0	0	0	0	0	0	0	0	0	0	0	0	0	0	0	0	0	0	0
0	0	0	0	0	0	0	0	0	0	0	0	0	0	0	0	0	0	0
0	0	0	0	0	0	0	0	0	0	0	0	0	0	0	0	0	0	0
0	0	0	0	0	0	0	0	0	0	0	0	0	0	0	0	0	0	0

0	0	0	0	0	2	2	2	2	2	2	0	0	0	0	0	0	0	0
0	0	0	0	0	0	0	0	0	0	0	0	0	0	0	0	0	0	0
0	0	0	0	0	0	0	0	0	0	0	0	0	0	0	0	0	0	0
0	0	0	0	0	0	0	0	0	0	0	0	0	0	0	0	0	0	0
0	0	0	0	0	0	0	0	0	0	0	0	0	0	0	0	0	0	0
0	0	0	0	0	0	0	0	0	0	0	0	0	0	0	0	0	0	0
0	0	0	0	0	0	0	0	0	0	0	0	0	0	0	0	0	0	0
0	0	0	0	0	0	0	0	0	0	0	0	0	0	0	0	0	0	0
0	0	0	0	0	0	0	0	0	0	0	0	0	0	0	0	0	0	0
0	0	0	0	0	0	0	0	0	0	0	0	0	0	0	0	0	0	0
0	0	0	0	0	0	0	0	0	0	0	0	0	0	0	0	0	0	0
0	0	0	0	0	0	0	0	0	0	0	0	0	0	0	0	0	0	0
0	0	0	0	0	0	0	0	0	0	0	0	0	0	0	0	0	0	0
0	0	0	0	0	0	0	0	0	0	0	0	0	0	0	0	0	0	0
0	0	0	0	0	0	0	0	0	0	0	0	0	0	0	0	0	0	0
0	0	0	0	0	0	0	0	0	0	0	0	0	0	0	0	0	0	0
0	0	0	0	0	0	0	0	0	0	0	0	0	0	0	0	0	0	0
0	0	0	0	0	0	0	0	0	0	0	0	0	0	0	0	0	0	0
0	0	0	0	0	0	0	0	0	0	0	0	0	0	0	0	0	0	0
0	0	0	0	0	0	0	0	0	0	0	0	0	0	0	0	0	0	0
0	0	0	0	0	0	0	0	0	0	0	0	0	0	0	0	0	0	0
0	0	0	0	0	0	0	0	0	0	0	0	0	0	0	0	0	0	0
0	0	0	0	0	0	0	0	0	0	0	0	0	0	0	0	0	0	0
0	0	0	0	0	0	0	0	0	0	0	0	0	0	0	0	0	0	0
0	0	0	0	0	0	0	0	0	0	0	0	0	0	0	0	0	0	0
0	0	0	0	0	0	0	0	0	0	0	0	0	0	0	0	0	0	0
0	0	0	0	0	0	0	0	0	0	0	0	0	0	0	0	0	0	0
0	0	0	0	0	0	0	0	0	0	0	0	0	0	0	0	0	0	0
0	0	0	0	0	0	0	0	0	0									
Jan_DL159	1179	1011	363	314	670	301	124	118	54	86	147	70	171	280	115	44	252	200
124	0	110	0	124	127	59	113	60	139	158	496	56	36	78	72	69	0	462
139	121	206	124	41	43	0	0	147	21	181	70	48	30	0	67	0	96	279
257	7	52	175	101	0	58	36	0	41	35	0	0	58	0	0	46	11	14
23	0	0	43	61	0	10	19	0	27	0	0	18	0	25	0	0	31	52
0	0	85	0	0	98	0	0	0	2	30	0	17	0	0	54	0	0	0
0	0	0	0	0	66	0	0	0	0	13	0	0	27	0	0	24	0	0
0	0	0	0	0	0	0	0	0	8	0	0	0	0	0	0	0	0	0

0	12	0	0	0	23	0	0	0	0	0	0	0	0	0	0	22	0	12
0	0	0	0	0	0	0	15	0	0	0	11	0	0	17	0	15	0	50
29	0	9	0	0	0	0	0	0	3	0	0	0	0	0	0	0	0	0
16	0	0	0	0	0	16	0	0	0	0	0	0	0	0	0	18	0	10
0	0	0	0	0	0	0	0	0	0	0	0	17	0	0	0	0	0	20
0	0	0	5	0	0	0	0	0	0	0	0	0	0	8	3	0	0	0
0	53	0	0	0	0	0	0	0	0	0	0	0	0	16	0	0	0	0
0	19	0	0	0	0	0	0	0	0	0	0	0	0	0	5	13	0	0
0	6	0	0	0	8	0	0	0	0	0	0	0	14	0	0	0	0	0
0	0	0	0	0	0	0	0	8	0	0	0	0	14	0	0	0	0	0
5	0	0	0	0	9	0	0	0	0	0	0	0	0	0	0	0	0	0
0	0	0	0	0	0	0	0	0	0	0	0	0	0	35	37	0	0	9
0	0	0	0	0	0	0	0	0	0	0	0	0	0	0	0	0	0	0
0	0	0	0	0	0	0	0	0	0	31	0	0	0	0	0	0	0	0
0	0	11	0	0	43	8	0	0	0	0	0	0	0	0	0	0	0	0
0	0	0	0	0	0	0	0	0	0	0	0	0	0	39	0	0	9	0
0	0	0	0	12	0	0	0	0	0	0	0	0	0	0	0	0	0	0
0	0	0	0	0	0	0	0	0	0	0	0	0	0	0	0	0	0	0
0	0	0	0	0	0	0	0	20	0	0	0	0	0	0	0	0	0	0
0	0	0	0	0	9	0	0	0	0	0	0	0	0	0	0	0	0	0
0	0	0	0	0	0	0	0	0	0	0	0	0	0	9	0	0	0	0
0	0	0	0	0	0	0	0	0	0	0	0	8	0	0	0	0	0	0
0	0	0	3	0	0	0	0	0	0	0	0	0	0	0	0	0	0	0
0	0	0	0	0	0	0	0	0	19	18	0	0	0	0	0	0	0	0
0	0	0	0	11	0	0	0	0	0	0	0	0	0	0	0	0	0	0
0	0	0	0	0	0	0	0	0	0	0	0	0	0	0	0	0	0	0
0	0	0	0	0	0	0	0	0	0	0	0	0	0	0	13	0	0	0
0	0	0	0	0	0	0	0	0	0	0	0	0	0	0	0	0	0	0
0	0	0	0	0	0	0	0	0	0	0	0	0	0	0	0	0	0	0
0	0	0	0	0	0	0	0	0	0	0	0	0	0	0	0	0	0	0
0	0	0	0	0	0	0	0	0	0	0	0	0	0	0	0	0	0	0
0	0	0	0	0	0	0	0	0	0	21	0	0	0	0	0	0	0	0
0	0	0	0	0	0	0	0	0	0	0	0	0	0	0	0	0	0	0
0	0	0	0	0	0	0	0	0	0	0	0	0	0	0	0	0	0	0
0	0	0	0	0	0	0	0	0	0	0	0	0	0	0	0	0	0	0
0	0	0	0	0	0	0	0	0	0	0	0	0	0	0	0	0	0	0
0	0	0	0	0	0	0	0	0	0	0	0	0	0	0	0	0	0	0

0	0	0	0	0	0	0	0	0	0	0	0	0	0	0	0	0	0	0
0	0	0	0	0	0	0	0	0	0	0	0	0	0	0	0	0	0	0
0	0	0	0	0	0	0	0	0	0	0	0	0	0	0	0	0	0	0
0	0	0	0	0	0	0	0	0	0	0	0	0	0	0	0	0	0	0
0	0	0	0	0	0	0	0	0	0	0	0	0	0	0	0	0	0	0
0	0	0	0	0	0	0	0	0	0	0	0	0	0	0	0	0	0	0
0	0	0	0	0	0	0	4	0	0	0	0	0	0	0	0	0	0	0
0	0	0	0	0	0	0	0	0	0	0	0	0	0	0	0	0	0	0
0	0	0	0	0	0	0	0	0	0	0	0	0	0	0	0	0	0	0
0	0	0	0	0	0	0	0	0	0	0	0	0	0	0	0	0	0	0
0	0	0	0	0	0	0	0	0	0	0	0	0	0	0	0	0	0	0
0	0	0	0	0	0	0	0	0	0	0	0	0	0	0	0	0	0	0
0	0	0	0	0	0	0	0	5	0	0	0	0	0	0	0	0	0	0
0	0	0	0	0	0	0	0	0	0	0	0	0	0	0	0	0	0	0
0	0	0	0	0	0	0	0	0	0	0	0	0	0	0	0	0	0	0
0	0	0	0	0	0	0	0	0	0	0	0	0	0	0	0	0	0	0
0	0	0	0	0	0	0	0	0	0	0	0	0	0	0	0	0	0	0
0	0	0	0	0	0	0	0	0	0	0	0	0	0	0	0	0	0	0
0	0	0	0	0	0	0	0	0	0	0	0	0	0	0	0	0	0	0
0	0	0	0	0	0	0	0	0	0	0	0	0	0	0	0	0	0	0
0	0	0	0	0	0	0	0	0	0	0	0	0	0	0	0	0	0	0
0	0	0	0	0	0	0	0	0	0	0	0	0	0	0	0	0	0	0
0	0	0	0	0	0	0	0	0	0	0	0	0	0	0	0	0	0	0
0	0	0	0	0	0	0	0	0	0	0	0	0	0	0	0	10	0	0
0	0	0	0	0	0	0	0	0	0	0	0	0	0	0	0	0	0	0
0	0	0	0	0	0	0	0	0	0	0	0	0	0	0	0	0	0	0
0	0	0	0	0	0	0	0	0	0	0	0	0	0	0	0	0	0	0
9	0	0	0	0	0	0	0	0	0	0	0	0	0	0	0	0	0	0
0	0	0	0	0	0	0	0	0	0	0	0	0	0	0	0	0	0	0
0	0	8	0	0	0	0	0	0	0	0	0	0	0	0	0	0	0	0
0	0	0	0	0	0	0	0	0	0	0	0	0	0	0	0	0	0	0
0	0	0	0	0	0	0	0	0	0	0	0	0	0	0	0	0	0	0
0	0	0	0	0	0	0	0	0	0	0	0	0	0	0	0	0	0	0
0	0	0	0	0	0	0	0	0	7	0	0	0	0	0	0	0	0	0
0	0	0	0	0	0	0	0	0	0	0	0	0	0	0	0	0	0	0
0	0	0	0	0	0	0	0	0	0	0	0	0	0	0	0	0	0	0
0	0	0	0	0	0	0	0	0	0	0	0	0	0	0	0	0	0	0

0	0	0	0	0	0	0	0	0	0	0	0	0	0	0	0	0	0	0
0	0	0	0	0	0	0	0	0	6	0	0	0	0	0	0	0	0	0
0	0	0	0	0	0	0	0	0	0	0	0	0	0	0	0	0	0	0
0	0	0	0	0	0	0	0	0	0	0	0	0	0	0	0	0	0	0
0	0	0	0	0	0	0	0	0	0	0	0	0	0	0	0	0	0	0
0	0	0	0	0	0	0	0	0	0	0	0	0	0	0	0	0	0	0
0	0	0	0	0	0	0	0	0	0	0	0	0	0	0	0	0	0	0
0	0	0	0	0	0	0	0	0	0	0	0	0	0	0	0	0	0	0
0	0	0	0	0	0	0	0	0	0	0	0	0	0	0	0	5	5	5
5	0	0	0	0	0	0	0	0	0	0	0	0	0	0	0	0	0	0
0	0	0	0	0	0	0	0	0	0	0	0	0	0	0	0	0	0	0
0	0	0	0	0	0	0	0	0	0	0	0	0	0	0	0	0	0	0
0	0	0	0	0	0	0	0	0	0	0	0	0	0	0	0	0	0	0
0	0	0	0	0	0	0	0	0	0	0	0	0	0	0	0	0	0	0
0	0	0	0	0	0	0	0	0	0	0	0	0	0	0	0	0	0	0
0	0	0	0	0	0	0	0	0	0	0	0	0	0	0	0	0	0	0
0	0	0	0	0	0	0	0	0	0	0	0	0	0	0	0	0	0	0
0	0	0	0	0	0	0	0	0	0	0	0	0	0	0	0	0	0	0
0	0	0	0	0	0	0	0	4	4	4	0	0	0	0	0	0	0	0
0	0	0	0	0	0	0	0	0	0	0	0	0	0	0	0	0	0	0
0	0	0	0	0	0	0	0	0	0	0	0	0	0	0	0	0	0	0
0	0	0	0	0	0	0	0	0	0	0	0	0	0	0	0	0	0	0
0	0	0	0	0	0	0	0	0	0	0	0	0	0	0	0	0	0	0
0	0	0	0	0	0	0	0	0	0	0	0	0	0	0	0	0	0	0
0	0	0	0	0	0	0	0	0	0	0	0	0	0	0	0	0	0	0
0	0	0	0	0	0	0	0	0	0	0	0	0	0	0	0	0	0	0
0	0	0	0	0	0	0	0	0	0	0	0	0	0	0	0	0	0	0
0	0	0	0	0	0	0	0	0	0	0	0	0	0	0	0	0	0	0
0	0	0	0	0	0	0	0	0	0	0	0	0	0	0	0	0	0	0
0	0	0	0	0	0	0	0	0	0	0	0	0	0	0	0	0	0	0
0	0	0	0	0	0	0	0	0	0	0	0	0	0	0	0	0	0	0
0	0	0	0	0	0	0	0	0	0	0	0	0	0	0	0	0	0	0
0	0	3	3	0	0	0	0	0	0	0	0	0	0	0	0	0	0	0
0	0	0	0	0	0	0	0	0	0	0	0	0	0	0	0	0	0	0
0	0	0	0	0	0	0	0	0	0	0	0	0	0	0	0	0	0	0
0	0	0	0	0	0	0	0	0	0	0	0	0	0	0	0	0	0	0
0	0	0	0	0	0	0	0	0	0	0	0	0	0	0	0	0	0	0

0	0	0	0	0	0	0	0	0	0	0	0	0	0	0	0	0	0	0
0	0	0	0	0	0	0	0	0	0	0	0	0	0	0	0	0	0	0
0	0	0	0	0	0	0	0	0	0	0	0	0	0	0	0	0	0	0
0	0	0	0	0	0	0	0	0	0	0	0	0	0	0	0	0	0	0
0	0	0	0	0	0	0	0	0	0	0	0	0	0	0	0	0	0	0
0	0	0	0	0	0	0	0	0	0	0	0	0	0	0	0	0	0	0
0	0	0	0	0	0	0	0	0	0	0	0	0	0	0	0	0	0	0
0	0	0	0	0	0	0	0	0	0	0	0	0	0	0	0	0	0	0
0	0	0	0	0	0	0	0	0	0	0	0	0	0	0	0	0	0	0
0	0	0	0	0	0	0	0	0	0	0	0	0	0	0	0	0	0	0
0	0	0	0	0	0	0	0	0	0	0	0	0	0	0	0	0	0	0
0	0	0	0	0	0	0	0	0	0	0	0	0	0	0	0	0	0	0
0	0	0	0	0	0	0	0	0	0	0	0	0	0	0	0	0	0	0
0	0	0	0	0	0	0	0	0	0	0	0	0	0	0	0	0	0	0
0	0	0	0	0	0	0	0	0	0	0	0	0	0	0	0	0	0	0
0	0	0	0	0	0	0	0	0	0	0	0	0	0	0	0	0	0	0
0	0	0	0	0	0	0	0	0	0	0	0	0	0	0	0	0	0	0
0	0	0	0	0	0	0	0	0	0	0	2	2	2	2	2	0	0	0
0	0	0	0	0	0	0	0	0	0	0	0	0	0	0	0	0	0	0
0	0	0	0	0	0	0	0	0	0	0	0	0	0	0	0	0	0	0
0	0	0	0	0	0	0	0	0	0	0	0	0	0	0	0	0	0	0
0	0	0	0	0	0	0	0	0	0	0	0	0	0	0	0	0	0	0
0	0	0	0	0	0	0	0	0	0	0	0	0	0	0	0	0	0	0
0	0	0	0	0	0	0	0	0	0	0	0	0	0	0	0	0	0	0
0	0	0	0	0	0	0	0	0	0	0	0	0	0	0	0	0	0	0
0	0	0	0	0	0	0	0	0	0	0	0	0	0	0	0	0	0	0
0	0	0	0	0	0	0	0	0	0	0	0	0	0	0	0	0	0	0
0	0	0	0	0	0	0	0	0	0	0	0	0	0	0	0	0	0	0
0	0	0	0	0	0	0	0	0	0	0	0	0	0	0	0	0	0	0
0	0	0	0	0	0	0	0	0	0	0	0	0	0	0	0	0	0	0
0	0	0	0	0	0	0	0	0	0	0	0	0	0	0	0	0	0	0
0	0	0	0	0	0	0	0	0	0	0	0	0	0	0	0	0	0	0
0	0	0	0	0	0	0	0	0	0	0	0	0	0	0	0	0	0	0
0	0	0	0	0	0	0	0	0	0	0	0	0	0	0	0	0	0	0
0	0	0	0	0	0	0	0	0	0	0	0	0	0	0	0	0	0	0
0	0	0	0	0	0	0	0	0	0	0	0	0	0	0	0	0	0	0
0	0	0	0	0	0	0	0	0	0	0	0	0	0	0	0	0	0	0

0	0	0	0	0	0	0	0	0	0	0	0	0	0	0	0	0	0	0
0	0	0	0	0	0	0	0	0	0	0	0	0	0	0	0	0	0	0
0	0	0	0	0	0	0	0	0	0	0	0	0	0	0	0	0	0	0
0	0	0	0	0	0	0	0	0	0	0	0	0	0	0	0	0	0	0
0	0	0	0	0	0	0	0	0	0	0	0	0	0	0	0	0	0	0
0	0	0	0	0	0	0	0	0	0	0	0	0	0	0	0	0	0	0
0	0	0	0	0	0	0	0	0	0	0	0	0	0	0	0	0	0	0
0	0	0	0	0	0	0	0	0	0	0	0	0	0	0	0	0	0	0
0	0	0	0	0	0	0	0	0	0	0	0	0	0	0	0	0	0	0
0	0	0	0	0	0	0	0	0	0	0	0	0	0	0	0	0	0	0
0	0	0	0	0	0	0	0	0	0	0	0	0	0	0	0	0	0	0
0	0	0	0	0	0	0	0	0	0	0	0	0	0	0	0	0	0	0
0	0	0	0	0	0	0	0	0	0	0	0	0	0	0	0	0	0	0
0	0	0	0	0	0	0	0	0	0	0	0	0	0	0	0	0	0	0
0	0	0	0	0	0	0	0	0	0	0	0	0	0	0	0	0	0	0
0	0	0	0	0	0	0	0	0	0	0	0	0	0	0	0	0	0	0
0	0	0	0	0	0	0	0	0	0	0	0	0	0	0	0	0	0	0
0	0	0	0	0	0	0	0	0	0	0	0	0	0	0	0	0	0	1
0	0	0	0	0	0	0	0	0	0	0	0	0	0	0	0	0	0	0
0	0	0	0	0	0	0	0	0	0	0	0	0	0	0	0	0	0	0
0	0	0	0	0	0	0	0	0	0									
Jan_DL160	1070	888	1068	472	39	20	50	72	140	378	116	272	359	91	142	248	89	67
90	104	68	54	212	60	244	115	211	206	0	0	117	149	143	53	84	349	0
174	206	0	43	41	43	103	377	16	93	48	235	84	0	30	237	135	52	0
0	181	202	43	29	57	0	112	80	44	0	0	43	0	36	0	0	90	32
40	0	38	7	0	32	52	0	0	59	33	0	0	149	0	68	8	0	164
0	29	0	37	52	0	94	7	17	11	0	0	11	71	17	9	0	0	0
0	7	0	44	0	22	0	0	0	0	8	0	0	0	0	0	0	0	0
0	0	0	0	0	62	0	0	0	0	0	0	0	0	25	0	0	0	0
504	0	0	0	0	0	0	0	25	15	0	0	12	10	24	0	0	0	0
19	32	14	0	0	0	0	0	0	0	0	53	0	12	0	0	27	0	0
0	0	0	17	0	0	0	0	0	0	0	0	0	0	0	0	0	0	0
0	0	0	0	0	0	0	0	0	0	0	0	0	0	0	0	0	3	25
0	124	20	0	0	0	0	18	0	23	0	10	0	6	0	0	0	0	0
0	0	0	0	0	0	0	0	0	16	0	41	0	10	0	5	0	0	0
0	0	0	0	0	0	0	0	2	0	0	0	0	0	2	0	0	0	0
0	0	0	0	0	0	0	0	5	0	17	0	0	29	0	0	0	0	0

0	0	0	97	0	0	0	0	0	0	0	0	0	9	0	8	0	0	2
0	0	0	12	0	75	0	0	0	0	0	0	0	0	3	0	0	0	0
0	0	0	19	9	0	0	0	8	0	0	0	0	0	0	0	0	0	0
0	0	0	0	0	0	0	0	0	7	0	0	0	0	0	0	0	0	0
23	0	0	0	11	0	0	0	0	0	0	0	0	0	0	0	0	0	0
0	0	0	0	0	0	0	0	0	0	0	0	0	0	0	0	0	0	0
0	0	0	0	0	8	0	0	2	0	0	0	0	0	0	0	0	0	0
0	10	0	0	0	0	0	0	71	0	0	0	0	0	0	0	0	0	0
0	0	0	0	0	0	0	0	0	0	16	0	0	0	0	0	0	0	0
0	0	0	7	0	0	0	0	28	0	0	0	0	0	0	0	0	0	0
0	0	0	0	0	0	0	0	0	0	0	0	0	0	0	0	0	4	0
0	0	19	0	0	0	0	0	0	0	13	0	0	0	0	0	0	0	0
0	0	0	0	0	0	0	0	0	0	0	5	0	0	0	0	0	0	0
0	0	0	0	0	0	0	0	0	0	0	11	0	0	0	0	0	11	0
0	0	0	0	0	0	0	0	0	0	0	0	0	0	0	0	0	20	0
11	0	0	0	0	0	0	0	0	6	0	0	0	0	0	0	0	0	0
0	0	0	0	0	0	0	0	0	0	0	0	0	0	0	0	0	0	0
3	0	0	0	0	0	0	0	10	0	0	0	0	0	0	0	0	0	0
0	0	0	0	0	0	0	0	0	0	7	0	0	0	0	0	0	0	0
0	0	0	0	0	0	0	0	0	0	0	0	0	0	0	0	0	0	0
0	0	0	0	0	0	0	0	0	0	0	0	0	0	0	0	0	0	0
0	0	0	0	0	0	0	0	0	0	0	0	0	0	0	0	0	0	0
0	0	0	0	0	0	0	0	0	0	0	0	0	0	0	0	0	0	0
0	0	0	0	0	0	0	0	0	0	0	0	0	0	0	0	0	0	0
0	0	0	0	0	0	0	0	0	0	0	0	0	0	0	0	0	0	0
0	0	10	0	0	0	0	0	0	0	0	0	0	0	0	0	0	0	0
0	0	0	0	0	0	0	0	0	0	0	0	0	0	0	0	0	0	0
0	0	0	0	0	0	0	0	0	0	0	0	0	0	0	0	0	0	0
0	0	0	0	0	0	0	0	0	0	0	0	0	0	0	0	0	0	0
0	0	0	0	0	0	0	0	0	0	0	0	0	0	0	0	0	0	0
0	0	0	0	0	0	0	0	0	0	0	0	0	0	0	0	0	0	0
0	0	0	0	0	0	0	0	0	0	0	0	0	0	0	0	0	0	0
0	0	0	0	0	0	0	0	0	0	0	0	0	0	0	0	0	0	0
0	10	0	0	0	0	0	0	0	0	0	0	0	0	0	0	0	0	0
0	0	0	0	0	0	14	0	0	0	0	0	0	0	0	0	0	0	0
0	0	0	0	0	0	0	0	0	0	0	0	0	0	0	0	0	0	0
0	0	0	0	0	0	0	0	0	0	0	0	0	0	0	0	0	0	0

0	0	0	0	0	0	0	0	0	0	0	0	0	0	0	0	0	0	0
0	0	0	0	0	0	0	0	0	0	0	0	0	0	0	0	0	0	0
0	0	0	0	0	0	0	0	0	0	0	0	0	0	0	0	0	0	0
0	0	0	0	0	0	0	0	0	0	7	0	0	0	0	0	0	0	0
0	0	0	0	0	0	0	0	0	0	0	0	0	0	0	0	0	0	0
0	0	0	0	0	0	0	0	0	0	0	0	0	0	0	0	0	0	0
0	0	0	0	0	0	0	0	0	0	0	0	0	0	0	0	0	0	0
0	0	0	0	0	0	0	0	0	0	0	0	0	0	0	0	0	0	0
0	0	0	0	0	0	0	0	0	0	0	0	0	0	0	0	0	0	0
0	0	0	0	0	0	0	0	0	0	0	0	0	0	0	0	0	0	0
0	0	0	0	0	0	0	0	0	0	0	0	0	0	0	0	0	0	0
0	0	0	0	0	0	0	0	0	0	0	0	0	0	0	0	0	0	0
0	0	0	0	0	0	0	0	0	0	0	0	0	0	0	0	0	0	0
0	0	0	0	0	0	0	0	0	0	0	0	0	0	0	0	0	0	0
0	0	0	0	0	0	0	0	0	12	0	0	0	0	0	0	0	0	0
0	0	0	0	0	0	0	0	0	0	0	0	0	0	0	0	0	0	0
0	0	0	0	0	0	0	0	0	0	0	0	0	0	0	0	0	0	0
0	0	0	0	0	0	0	0	0	0	0	0	0	0	0	0	0	0	0
0	0	0	0	0	0	0	0	0	0	0	0	0	0	0	0	0	0	0
0	0	0	0	0	0	0	0	0	0	0	0	0	0	0	0	0	0	0
0	0	0	0	0	0	0	0	0	0	0	0	0	0	0	0	0	0	0
0	0	0	0	0	0	0	0	0	0	0	0	0	9	0	0	0	0	0
0	0	0	0	0	0	0	0	0	0	0	0	0	0	0	0	0	0	0
0	0	0	0	0	0	0	0	0	0	0	0	0	0	0	0	0	0	0
0	0	0	0	0	0	0	0	0	0	0	0	0	0	0	0	0	0	0
0	0	0	0	0	0	0	0	0	0	0	0	0	0	0	0	0	0	0
0	0	0	0	0	0	0	0	0	0	0	0	0	0	0	0	0	0	0
0	0	0	0	0	0	0	0	0	0	0	0	0	0	0	0	0	0	0
0	0	0	0	0	0	0	0	0	0	0	0	0	0	0	0	0	0	0
0	0	0	0	0	0	0	0	0	0	7	7	0	0	0	0	0	0	0
0	0	0	0	0	0	0	0	0	0	0	0	0	0	0	0	0	0	0
0	0	0	0	0	0	0	0	0	0	0	0	0	0	0	0	0	0	0
0	0	0	0	0	0	0	0	0	0	0	0	0	0	0	0	0	0	0
0	0	0	0	0	0	0	0	0	0	0	0	0	0	0	0	0	0	0
0	0	0	0	0	0	0	0	0	0	0	0	0	0	0	0	0	0	0
0	0	0	0	0	0	0	0	0	0	0	0	6	0	0	0	0	0	0
0	0	0	0	0	0	0	0	0	0	0	0	0	0	0	0	0	0	0

0	0	0	0	0	0	0	0	0	0	0	0	0	0	0	0	0	0	0
0	0	0	0	0	0	0	0	0	0	0	0	0	0	0	0	0	0	0
0	0	0	0	0	0	0	0	0	0	0	0	0	0	0	0	0	0	0
0	0	0	0	0	0	0	0	0	0	0	0	0	0	0	0	0	0	0
0	0	0	0	0	0	0	0	0	0	0	0	0	0	0	0	0	0	0
0	0	0	0	0	0	0	0	0	0	0	0	0	0	0	0	0	0	0
0	0	0	0	5	5	0	0	0	0	0	0	0	0	0	0	0	0	0
0	0	0	0	0	0	0	0	0	0	0	0	0	0	0	0	0	0	0
0	0	0	0	0	0	0	0	0	0	0	0	0	0	0	0	0	0	0
0	0	0	0	0	0	0	0	0	0	0	0	0	0	0	0	0	0	0
0	0	0	0	0	0	0	0	0	0	0	0	0	0	0	0	0	0	0
0	0	0	0	0	0	0	0	0	0	0	0	0	0	0	0	0	0	0
0	0	0	0	0	0	0	0	0	0	0	0	0	0	0	0	0	0	0
0	0	0	0	0	0	0	0	0	0	0	0	0	0	0	0	0	0	0
0	0	0	0	0	0	0	0	0	0	0	0	0	0	0	0	0	0	0
0	0	0	0	0	0	0	0	0	0	0	0	0	0	0	0	0	0	0
0	0	0	0	0	4	4	4	4	0	0	0	0	0	0	0	0	0	0
0	0	0	0	0	0	0	0	0	0	0	0	0	0	0	0	0	0	0
0	0	0	0	0	0	0	0	0	0	0	0	0	0	0	0	0	0	0
0	0	0	0	0	0	0	0	0	0	0	0	0	0	0	0	0	0	0
0	0	0	0	0	0	0	0	0	0	0	0	0	0	0	0	0	0	0
0	0	0	0	0	0	0	0	0	0	0	0	0	0	0	0	0	0	0
0	0	0	0	0	0	0	0	0	0	0	0	0	0	0	0	0	0	0
0	0	0	0	0	0	0	0	0	0	0	0	0	0	0	0	0	0	0
0	0	0	0	0	0	0	0	0	0	0	0	0	0	0	0	0	0	0
0	0	0	0	0	0	0	0	0	0	0	0	0	0	0	0	0	0	0
0	0	0	0	0	0	0	0	0	0	0	0	0	0	0	0	0	0	0
0	0	0	0	0	0	0	0	0	0	0	0	0	0	0	0	0	0	0
0	0	0	0	0	0	0	0	0	0	0	0	0	0	0	0	0	0	0
0	0	0	0	0	0	0	0	0	0	0	0	0	0	0	0	0	0	0
0	0	0	0	3	3	3	3	3	0	0	0	0	0	0	0	0	0	0
0	0	0	0	0	0	0	0	0	0	0	0	0	0	0	0	0	0	0
0	0	0	0	0	0	0	0	0	0	0	0	0	0	0	0	0	0	0
0	0	0	0	0	0	0	0	0	0	0	0	0	0	0	0	0	0	0
0	0	0	0	0	0	0	0	0	0	0	0	0	0	0	0	0	0	0
0	0	0	0	0	0	0	0	0	0	0	0	0	0	0	0	0	0	0
0	0	0	0	0	0	0	0	0	0	0	0	0	0	0	0	0	0	0

0	0	0	0	0	0	0	0	0	0	0	0	0	0	0	0	0	0	0
0	0	0	0	0	0	0	0	0	0	0	0	0	0	0	0	0	0	0
0	0	0	0	0	0	0	0	0	0	0	0	0	0	0	0	0	0	0
0	0	0	0	0	0	0	0	0	0	0	0	0	0	0	0	0	0	0
0	0	0	0	0	0	0	0	0	0	0	0	0	0	0	0	0	0	0
0	0	0	0	0	0	0	0	0	0	0	0	0	0	0	0	0	0	0
0	0	0	0	0	0	0	0	0	0	0	0	0	0	0	0	0	0	0
0	0	0	0	0	0	0	0	0	0	0	0	0	0	0	0	0	0	0
0	0	0	0	0	0	0	0	0	0	0	0	0	0	0	0	0	0	0
0	0	0	0	0	0	0	0	0	0	0	0	0	0	0	0	0	0	0
0	0	0	0	0	0	0	0	0	0	0	0	0	0	0	0	0	0	0
0	0	0	0	0	0	0	0	0	0	0	0	0	0	0	0	0	0	0
0	0	0	0	0	0	0	0	0	0	0	0	0	0	0	0	0	0	0
0	0	0	0	0	0	0	0	0	0	0	0	0	0	0	0	0	0	0
0	0	0	0	0	0	0	0	0	0	0	0	0	0	0	0	0	0	0
0	0	0	0	0	0	0	0	0	0	0	0	0	0	0	0	0	0	0
0	0	0	0	0	0	0	0	0	0	0	0	0	0	0	0	0	0	0
0	0	0	0	0	0	0	0	0	0	0	0	0	0	0	0	0	0	0
0	0	0	0	0	0	0	0	0	0	0	0	0	0	0	0	0	0	0
0	2	2	2	2	2	2	0	0	0	0	0	0	0	0	0	0	0	0
0	0	0	0	0	0	0	0	0	0	0	0	0	0	0	0	0	0	0
0	0	0	0	0	0	0	0	0	0	0	0	0	0	0	0	0	0	0
0	0	0	0	0	0	0	0	0	0	0	0	0	0	0	0	0	0	0
0	0	0	0	0	0	0	0	0	0	0	0	0	0	0	0	0	0	0
0	0	0	0	0	0	0	0	0	0	0	0	0	0	0	0	0	0	0
0	0	0	0	0	0	0	0	0	0	0	0	0	0	0	0	0	0	0
0	0	0	0	0	0	0	0	0	0	0	0	0	0	0	0	0	0	0
0	0	0	0	0	0	0	0	0	0	0	0	0	0	0	0	0	0	0
0	0	0	0	0	0	0	0	0	0	0	0	0	0	0	0	0	0	0
0	0	0	0	0	0	0	0	0	0	0	0	0	0	0	0	0	0	0
0	0	0	0	0	0	0	0	0	0	0	0	0	0	0	0	0	0	0
0	0	0	0	0	0	0	0	0	0	0	0	0	0	0	0	0	0	0
0	0	0	0	0	0	0	0	0	0	0	0	0	0	0	0	0	0	0
0	0	0	0	0	0	0	0	0	0	0	0	0	0	0	0	0	0	0
0	0	0	0	0	0	0	0	0	0	0	0	0	0	0	0	0	0	0
0	0	0	0	0	0	0	0	0	0	0	0	0	0	0	0	0	0	0
0	0	0	0	0	0	0	0	0	0	0	0	0	0	0	0	0	0	0

0	0	0	0	0	0	0	0	0	0	0	0	0	0	0	0	0	0	0
0	0	0	0	0	0	0	0	0	0	0	0	0	0	0	0	0	0	0
0	0	0	0	0	0	0	0	0	0	0	0	0	0	0	0	0	0	0
0	0	0	0	0	0	0	0	0	0	0	0	0	0	0	0	0	0	0
0	0	0	0	0	0	0	0	0	0	0	0	0	0	0	0	0	0	0
0	0	0	0	0	0	0	0	0	0	0	0	0	0	0	0	0	0	0
0	0	0	0	0	0	0	0	0	0	0	0	0	0	0	0	0	0	0
0	0	0	0	0	0	0	0	0	0	0	0	0	0	0	0	0	0	0
0	0	0	0	0	0	0	0	0	0	0	0	0	0	0	0	0	0	0
0	0	0	0	0	0	0	0	0	0	0	0	0	0	0	0	0	0	0
0	0	0	0	0	0	0	0	0	0	0	0	0	0	0	0	0	0	0
0	0	0	0	0	0	0	0	0	0	0	0	0	0	0	0	0	0	0
0	0	0	0	0	0	0	0	0	0									
Jan_DL161	1152	785	278	508	41	378	405	400	319	127	258	115	258	455	70	85	464	165
215	46	67	0	159	68	57	146	45	189	138	205	132	85	93	153	105	49	197
130	94	167	111	83	114	106	0	96	30	135	64	33	58	100	44	60	90	140
120	4	52	33	116	14	66	26	19	67	20	0	7	61	10	0	43	8	96
34	0	42	88	70	59	17	41	0	38	78	0	28	0	33	40	0	23	55
44	79	108	0	0	0	0	0	0	16	0	0	0	0	21	10	0	0	0
0	17	15	6	0	0	0	0	0	0	0	0	11	22	0	0	6	0	0
0	11	0	0	0	0	0	0	44	34	0	0	8	0	14	0	0	0	0
0	17	0	0	0	9	0	0	0	0	0	0	0	0	0	0	16	0	0
0	31	0	24	0	0	0	25	0	14	0	8	25	0	0	34	0	0	0
37	0	0	0	0	0	0	0	0	0	0	0	12	0	0	0	0	0	0
0	0	0	0	0	0	0	0	0	0	0	0	0	0	0	0	4	12	0
0	0	0	20	0	0	0	0	0	0	0	5	0	11	0	0	0	0	14
8	0	0	0	0	0	0	0	0	0	0	0	0	0	0	0	0	0	0
10	0	0	0	0	4	0	0	0	0	0	6	0	0	0	0	14	0	0
0	0	0	0	0	0	0	0	0	0	0	0	11	0	7	0	0	0	0
0	0	0	0	0	0	0	0	0	0	0	0	11	0	0	8	0	0	0
3	0	0	3	0	0	0	3	6	0	0	0	0	0	0	0	0	0	0
0	11	0	0	7	0	0	0	0	0	0	0	0	0	0	0	0	0	0
0	0	0	0	0	0	0	5	0	0	0	0	0	0	0	0	0	0	0
0	0	0	0	4	0	0	0	0	0	0	0	0	0	0	0	0	0	0
0	0	0	0	0	0	0	0	0	0	0	9	0	0	0	0	0	0	0
0	0	0	0	0	0	0	0	0	0	0	0	0	0	0	0	6	0	0
0	0	0	0	0	0	0	0	0	0	0	0	0	0	0	0	0	0	0

0	0	0	0	0	0	0	6	0	0	0	0	0	0	0	0	0	0	0
0	0	0	0	0	0	0	0	0	0	0	0	0	5	0	0	0	0	0
0	0	0	0	0	0	0	0	0	18	0	0	0	0	0	0	0	0	11
0	0	0	0	0	0	0	0	0	4	0	0	0	0	0	0	0	0	0
0	0	0	0	0	0	0	0	0	0	0	0	0	0	0	0	0	0	0
0	0	7	0	0	0	0	0	0	0	0	0	0	0	0	0	0	0	0
0	0	0	0	0	0	0	0	0	0	0	0	0	0	0	0	0	0	0
0	0	0	0	0	0	0	0	0	0	5	0	0	0	0	0	0	0	0
0	0	0	0	0	0	0	0	0	0	0	0	0	0	0	0	0	0	0
0	0	0	0	0	0	0	0	0	0	0	0	0	0	0	0	0	0	0
0	0	0	0	0	0	0	0	0	0	0	0	0	0	0	0	0	0	0
0	0	0	0	0	0	0	0	0	0	0	0	0	0	0	0	0	0	0
0	0	0	0	0	0	0	0	0	0	0	0	0	0	0	0	0	0	0
0	0	0	0	0	0	0	0	0	0	0	0	0	0	0	0	0	0	0
0	0	0	0	6	0	0	0	0	0	0	0	0	0	0	0	0	0	0
0	0	0	0	0	0	0	0	0	0	0	0	0	0	0	0	0	0	0
0	0	0	0	0	0	7	0	0	0	0	0	0	0	0	38	0	0	0
0	0	0	0	0	0	0	0	0	0	6	0	0	0	0	0	0	0	0
0	0	0	0	0	0	0	0	0	0	0	0	11	0	0	0	0	0	0
0	0	0	0	0	0	0	0	0	0	0	0	0	0	0	0	0	0	0
0	0	0	0	0	0	0	0	0	0	0	0	0	0	0	0	0	0	0
0	0	0	0	0	0	0	0	0	0	0	0	0	0	0	0	0	0	0
0	0	0	0	0	0	0	0	0	0	0	0	0	0	0	0	0	0	0
0	0	0	0	0	0	0	0	0	0	0	0	0	0	0	0	0	0	0
0	0	0	0	0	0	0	0	0	0	0	0	0	0	0	0	0	0	0
0	0	0	0	0	0	0	0	0	0	0	0	0	0	0	0	0	0	0
0	0	0	0	0	0	0	0	0	0	0	0	0	0	11	0	0	0	0
0	0	0	0	0	0	0	0	0	0	0	0	0	0	0	0	0	0	0
0	0	0	0	0	0	0	0	0	0	0	0	0	0	0	0	0	0	0
0	0	0	0	0	0	0	0	0	0	0	0	0	0	0	0	0	0	0
0	0	0	0	0	0	0	0	0	0	0	0	0	0	0	0	0	0	0
0	0	0	0	0	0	0	0	0	0	0	0	0	0	0	0	0	0	0
0	0	0	0	0	0	0	0	0	0	0	0	0	0	0	0	0	0	0
0	0	0	0	0	0	0	0	0	0	0	0	0	0	0	0	0	0	0
0	0	0	0	0	0	0	0	0	0	0	0	0	0	0	0	0	0	0
0	0	0	0	0	0	0	0	0	0	0	0	0	0	0	0	0	0	0
0	0	0	0	0	0	0	0	0	0	0	0	0	0	0	0	0	0	0

0	0	0	0	0	0	0	0	0	0	0	0	0	0	0	0	0	0	0
0	0	0	0	0	0	0	0	0	0	0	0	0	0	0	0	0	0	0
0	0	0	0	0	0	0	0	0	0	0	0	0	0	0	0	0	0	0
0	0	0	0	0	0	0	0	0	0	0	0	0	0	0	0	0	0	0
0	0	0	0	0	0	0	0	0	0	0	0	0	0	0	0	0	0	0
0	0	0	0	0	0	0	0	0	0	0	0	0	0	0	0	0	0	0
0	0	0	0	0	0	0	0	0	0	0	0	0	0	0	0	0	0	0
0	0	0	0	0	0	0	0	0	0	0	0	0	0	0	0	0	0	0
0	0	0	0	0	0	0	0	0	0	0	0	0	0	0	0	0	0	0
0	0	0	0	0	0	0	0	0	0	0	0	0	0	0	0	0	0	0
0	0	0	0	0	0	0	0	0	0	0	0	0	0	0	0	0	0	0
0	0	0	0	0	0	0	0	0	0	0	0	0	0	0	0	0	0	0
0	9	0	0	0	0	0	0	0	0	0	0	0	0	0	0	0	0	0
0	0	0	0	0	0	0	0	0	0	0	0	0	0	0	0	0	0	0
0	0	0	0	0	0	0	0	0	0	0	0	0	0	0	0	0	0	0
0	0	0	0	0	0	0	0	0	0	0	0	0	0	0	0	0	0	0
0	0	0	0	0	0	0	0	0	0	0	0	0	0	0	0	0	0	0
0	0	0	0	0	0	0	0	0	0	0	0	0	0	0	0	0	0	0
0	0	0	0	0	0	0	0	0	0	0	0	0	0	0	0	0	0	0
0	0	0	0	0	0	0	0	0	0	0	0	0	0	0	0	0	0	0
0	0	0	0	0	0	0	0	0	0	0	0	0	0	0	0	0	0	0
0	0	0	0	0	0	0	0	0	0	0	0	0	0	0	0	0	0	0
0	0	0	0	0	0	0	0	0	0	0	0	0	0	0	0	0	0	0
0	0	0	0	0	0	0	0	0	0	0	0	0	0	0	0	0	0	0
0	0	0	0	0	0	0	0	0	0	0	0	0	0	0	0	0	0	0
0	0	0	0	0	0	0	0	0	0	0	0	0	0	0	0	0	0	0
0	0	0	0	0	0	0	0	0	0	0	0	0	0	0	0	0	0	0
0	0	0	0	0	0	0	0	0	0	0	0	0	0	0	0	0	0	0
0	0	0	0	0	0	0	0	0	0	0	0	0	0	0	0	0	0	0
0	0	0	0	0	0	0	0	0	0	0	0	0	0	0	0	0	0	0
0	0	0	0	0	0	0	0	0	0	0	0	0	0	0	0	0	0	0
0	0	0	0	0	0	0	0	0	0	5	5	0	0	0	0	0	0	0
0	0	0	0	0	0	0	0	0	0	0	0	0	0	0	0	0	0	0
0	0	0	0	0	0	0	0	0	0	0	0	0	0	0	0	0	0	0
0	0	0	0	0	0	0	0	0	0	0	0	0	0	0	0	0	0	0
0	0	0	0	0	0	0	0	0	0	0	0	0	0	0	0	0	0	0
0	0	0	0	0	0	0	0	0	0	0	0	0	0	0	0	0	0	0

0	0	0	0	0	0	0	0	0	0	0	0	0	0	0	0	0	0	0
0	0	0	0	0	0	0	0	0	0	0	0	0	0	0	0	0	0	0
0	0	0	0	0	0	0	0	0	0	0	0	0	0	0	0	0	0	0
0	0	0	0	0	0	0	0	0	0	0	0	0	0	0	0	0	0	0
0	0	0	0	0	0	0	0	0	0	0	0	0	0	0	0	0	0	0
0	0	0	0	0	0	0	4	4	4	0	0	0	0	0	0	0	0	0
0	0	0	0	0	0	0	0	0	0	0	0	0	0	0	0	0	0	0
0	0	0	0	0	0	0	0	0	0	0	0	0	0	0	0	0	0	0
0	0	0	0	0	0	0	0	0	0	0	0	0	0	0	0	0	0	0
0	0	0	0	0	0	0	0	0	0	0	0	0	0	0	0	0	0	0
0	0	0	0	0	0	0	0	0	0	0	0	0	0	0	0	0	0	0
0	0	0	0	0	0	0	0	0	0	0	0	0	0	0	0	0	0	0
0	0	0	0	0	0	0	0	0	0	0	0	0	0	0	0	0	0	0
0	0	0	0	0	0	0	0	0	0	0	0	0	0	0	0	0	0	0
0	0	0	0	0	0	0	0	0	0	0	0	0	0	0	0	0	0	0
0	0	0	0	0	0	0	0	0	0	0	0	0	0	0	0	0	0	0
0	0	0	0	0	0	0	0	0	0	0	0	0	0	0	0	0	0	0
0	0	0	0	0	0	0	0	0	0	0	0	0	0	0	0	0	0	0
0	0	0	0	0	0	0	0	0	3	3	3	0	0	0	0	0	0	0
0	0	0	0	0	0	0	0	0	0	0	0	0	0	0	0	0	0	0
0	0	0	0	0	0	0	0	0	0	0	0	0	0	0	0	0	0	0
0	0	0	0	0	0	0	0	0	0	0	0	0	0	0	0	0	0	0
0	0	0	0	0	0	0	0	0	0	0	0	0	0	0	0	0	0	0
0	0	0	0	0	0	0	0	0	0	0	0	0	0	0	0	0	0	0
0	0	0	0	0	0	0	0	0	0	0	0	0	0	0	0	0	0	0
0	0	0	0	0	0	0	0	0	0	0	0	0	0	0	0	0	0	0
0	0	0	0	0	0	0	0	0	0	0	0	0	0	0	0	0	0	0
0	0	0	0	0	0	0	0	0	0	0	0	0	0	0	0	0	0	0
0	0	0	0	0	0	0	0	0	0	0	0	0	0	0	0	0	0	0
0	0	0	0	0	0	0	0	0	0	0	0	0	0	0	0	0	0	0
0	0	0	0	0	0	0	0	0	0	0	0	0	0	0	0	0	0	0
0	0	0	0	0	0	0	0	0	0	0	0	0	0	0	0	0	0	0
0	0	0	0	0	0	0	0	0	0	0	0	0	0	0	0	0	0	0
0	0	0	0	0	0	0	0	0	0	0	0	0	0	0	0	0	0	0
0	0	0	0	0	0	0	0	0	0	0	0	0	0	0	0	0	0	0
0	0	0	0	0	0	0	0	0	0	0	0	0	0	0	0	0	0	0
0	0	0	0	0	0	0	0	0	0	0	0	0	0	0	0	0	0	0

0	0	0	0	0	0	0	0	0	0	0	0	0	0	0	0	0	0	0
0	0	0	0	0	0	0	0	0	0	0	0	0	0	0	0	0	0	0
0	0	0	0	0	0	0	0	0	0	0	0	0	0	0	0	0	0	0
0	0	0	0	0	0	0	0	0	0	0	0	0	0	0	0	0	0	0
0	0	0	0	0	0	0	0	0	0	0	0	0	0	0	0	0	0	0
0	0	0	0	0	0	0	0	0	0	0	0	0	0	0	0	0	0	0
0	0	0	0	0	0	0	2	2	2	2	2	0	0	0	0	0	0	0
0	0	0	0	0	0	0	0	0	0	0	0	0	0	0	0	0	0	0
0	0	0	0	0	0	0	0	0	0	0	0	0	0	0	0	0	0	0
0	0	0	0	0	0	0	0	0	0	0	0	0	0	0	0	0	0	0
0	0	0	0	0	0	0	0	0	0	0	0	0	0	0	0	0	0	0
0	0	0	0	0	0	0	0	0	0	0	0	0	0	0	0	0	0	0
0	0	0	0	0	0	0	0	0	0	0	0	0	0	0	0	0	0	0
0	0	0	0	0	0	0	0	0	0	0	0	0	0	0	0	0	0	0
0	0	0	0	0	0	0	0	0	0	0	0	0	0	0	0	0	0	0
0	0	0	0	0	0	0	0	0	0	0	0	0	0	0	0	0	0	0
0	0	0	0	0	0	0	0	0	0	0	0	0	0	0	0	0	0	0
0	0	0	0	0	0	0	0	0	0	0	0	0	0	0	0	0	0	0
0	0	0	0	0	0	0	0	0	0	0	0	0	0	0	0	0	0	0
0	0	0	0	0	0	0	0	0	0	0	0	0	0	0	0	0	0	0
0	0	0	0	0	0	0	0	0	0	0	0	0	0	0	0	0	0	0
0	0	0	0	0	0	0	0	0	0	0	0	0	0	0	0	0	0	0
0	0	0	0	0	0	0	0	0	0	0	0	0	0	0	0	0	0	0
0	0	0	0	0	0	0	0	0	0	0	0	0	0	0	0	0	0	0
0	0	0	0	0	0	0	0	0	0	0	0	0	0	0	0	0	0	0
0	0	0	0	0	0	0	0	0	0	0	0	0	0	0	0	0	0	0
0	0	0	0	0	0	0	0	0	0	0	0	0	0	0	0	0	0	0
0	0	0	0	0	0	0	0	0	0	0	0	0	0	0	0	0	0	0
0	0	0	0	0	0	0	0	0	0	0	0	0	0	0	0	0	0	0
0	0	0	0	0	0	0	0	0	0	0	0	0	0	0	0	0	0	0
0	0	0	0	0	0	0	0	0	0	0	0	0	0	0	0	0	0	0
0	0	0	0	0	0	0	0	0	0	0	0	0	0	0	0	0	0	0
0	0	0	0	0	0	0	0	0	0	0	0	0	0	0	0	0	0	0
0	0	0	0	0	0	0	0	0	0	0	0	0	0	0	0	0	0	0
0	0	0	0	0	0	0	0	0	0	0	0	0	0	0	0	0	0	0
0	0	0	0	0	0	0	0	0	0	0	0	0	0	0	0	0	0	0
0	0	0	0	0	0	0	0	0	0	0	0	0	0	0	0	0	0	0

0	0	0	0	0	0	0	0	0	0	0	0	0	0	0	0	0	0	0
0	0	0	0	0	0	0	0	0	0	0	0	0	0	0	0	0	0	0
0	0	0	0	0	0	0	0	0	0	0	0	0	0	0	0	0	0	0
0	0	0	0	0	0	0	0	0	0	0	0	0	0	0	0	0	0	0
0	0	0	0	0	0	0	0	0	0									
Jan_DL017	2098	1738	46	360	105	541	654	513	786	111	285	402	99	386	143	0	333	622
428	62	278	281	168	232	127	104	162	55	232	0	227	65	52	130	121	34	0
0	68	227	329	93	119	168	37	181	0	128	28	21	227	251	25	129	100	0
0	27	0	25	102	104	77	45	38	24	17	192	5	93	24	98	31	38	0
89	0	117	0	0	112	83	92	48	29	42	75	0	12	0	68	73	48	0
147	0	0	0	0	0	66	27	61	20	0	0	0	19	21	25	0	0	31
116	0	117	48	6	0	0	0	0	88	5	21	11	9	44	47	0	0	0
100	16	65	0	0	0	0	64	0	15	55	0	6	42	0	10	0	0	0
0	29	0	26	96	0	15	18	0	0	10	0	9	0	0	0	0	0	19
0	0	0	38	0	0	0	0	0	0	0	0	0	34	0	0	5	10	21
0	0	9	0	18	36	0	0	55	0	0	20	0	55	8	0	0	0	0
0	0	16	0	9	0	0	0	0	0	0	8	21	9	0	0	0	0	0
45	0	0	12	0	10	0	18	0	0	0	0	0	5	0	0	5	0	5
0	0	0	0	0	0	0	0	0	26	0	0	0	0	7	0	9	15	0
16	0	0	0	0	11	0	0	15	0	19	20	0	0	7	0	0	0	0
0	8	10	6	0	0	0	0	10	0	0	0	0	0	0	0	0	0	13
0	0	0	0	0	11	0	0	0	0	0	9	0	0	0	0	0	0	7
0	11	0	0	0	0	0	0	0	0	0	0	0	0	14	6	0	16	0
8	0	29	0	0	0	0	19	10	0	0	14	0	0	0	0	0	0	0
0	0	10	0	0	11	4	0	0	0	13	0	0	0	0	0	0	0	0
0	0	0	0	0	0	0	0	8	0	0	0	0	0	0	0	0	0	0
0	0	0	0	0	0	0	4	0	0	0	0	0	0	0	0	8	0	0
0	0	0	0	0	0	0	0	6	0	9	0	0	0	0	0	0	0	0
0	6	0	0	0	0	0	0	0	0	31	0	0	0	0	0	0	0	0
0	0	10	0	0	0	0	0	0	0	0	0	0	0	0	0	0	0	0
0	0	0	0	0	0	0	0	0	0	0	0	0	0	0	20	0	0	0
0	0	0	0	0	0	0	0	0	0	0	0	0	0	0	0	0	0	0
0	0	0	0	0	8	0	0	0	0	0	0	0	0	0	0	0	0	0
0	0	0	0	0	0	0	0	0	0	0	0	0	0	0	0	0	0	0
0	0	0	0	0	0	0	0	0	0	7	0	0	0	0	0	0	0	0
0	6	0	0	0	0	0	0	0	0	0	0	0	8	0	0	0	0	0
0	0	0	0	0	0	0	0	0	0	0	0	0	0	0	0	0	0	0

0	0	11	0	0	0	21	0	0	0	0	0	0	0	0	0	0	0	0
0	0	0	0	0	0	0	0	0	0	0	0	0	0	0	8	0	0	0
0	0	0	0	0	0	0	0	16	0	0	0	0	0	0	0	0	0	0
3	0	0	0	0	0	0	0	0	0	0	0	0	0	0	0	0	0	0
0	0	0	0	0	0	0	0	8	0	0	0	0	0	0	0	0	0	0
0	0	0	0	0	0	0	0	0	0	0	0	0	0	0	0	0	0	0
0	0	0	0	0	0	0	0	0	0	0	0	0	0	0	0	0	0	0
0	0	0	0	0	0	0	14	0	0	0	0	0	0	0	0	0	0	0
0	0	0	0	0	0	0	0	0	0	0	0	0	0	0	0	0	0	2
0	0	0	0	0	0	0	0	26	0	0	0	0	0	0	0	0	0	0
0	0	0	0	0	0	0	0	0	0	0	0	0	0	0	0	0	0	0
18	0	0	0	0	0	0	0	0	0	0	0	0	0	0	0	0	0	0
0	0	0	3	0	0	0	0	0	0	0	0	0	0	0	0	0	0	0
0	0	0	0	0	12	0	0	0	0	0	0	0	0	0	0	0	0	0
0	0	0	0	0	0	0	0	0	0	0	0	0	0	0	0	0	0	0
0	0	0	0	0	0	0	0	0	0	0	0	0	0	0	0	0	0	0
0	0	0	0	0	0	0	0	0	0	0	0	0	0	0	0	0	0	0
0	0	0	0	0	0	0	0	0	0	0	0	0	0	0	0	0	0	0
0	0	0	0	0	0	0	0	0	0	0	0	0	0	0	0	0	0	0
0	0	0	0	0	0	0	0	0	0	0	0	0	0	0	0	0	0	0
0	0	0	0	0	0	0	0	0	0	0	0	0	0	0	0	0	0	0
0	0	0	0	0	0	0	0	0	0	0	0	0	0	0	0	0	0	0
0	0	0	0	0	0	0	0	0	0	0	0	0	0	0	0	0	0	0
0	0	0	0	0	0	0	0	0	0	0	0	0	0	0	0	0	0	0
0	0	0	0	0	0	0	0	0	0	0	0	0	0	0	0	0	0	0
0	0	0	0	0	0	0	0	0	0	0	0	0	0	0	0	0	0	0
0	0	0	0	0	0	0	0	0	0	0	0	0	0	0	0	0	0	0
0	0	0	0	0	0	0	0	0	0	0	0	0	0	0	0	0	0	0
0	0	0	0	0	0	0	0	0	0	0	0	0	0	0	0	0	0	0
0	0	0	0	0	0	0	0	0	0	0	0	0	0	0	0	0	0	0
0	0	0	0	0	0	0	0	0	0	0	0	0	0	0	0	0	0	0
0	0	0	0	0	0	0	0	0	0	0	0	0	0	0	0	0	0	0
0	0	0	0	0	0	0	0	0	0	0	0	0	0	0	0	0	0	0
0	0	0	0	0	0	0	0	0	0	0	0	0	0	0	0	0	0	0
0	0	0	0	0	0	0	0	0	0	0	0	0	0	0	0	0	0	0
0	0	0	0	0	0	0	0	0	0	0	0	0	0	0	0	0	0	0
0	0	0	0	0	0	0	0	0	0	0	0	0	0	0	0	0	0	0

0	0	0	0	0	0	0	0	0	0	0	0	0	0	0	0	0	0	0
0	0	0	0	0	0	0	0	0	0	0	0	0	0	0	0	0	0	0
0	0	0	0	0	0	0	0	0	0	0	0	0	0	0	0	0	0	0
0	0	0	0	0	0	0	0	0	0	0	0	0	0	0	0	0	0	0
0	0	4	0	0	0	0	0	0	0	13	0	0	0	0	0	0	0	0
0	0	0	0	0	0	0	0	0	0	0	0	0	0	0	0	0	0	0
0	0	0	0	0	0	0	0	0	0	0	0	0	0	0	0	0	0	0
0	0	0	0	0	0	0	0	0	0	0	0	0	0	0	0	0	0	0
0	0	0	0	0	0	0	0	0	0	0	0	0	0	0	0	0	0	0
0	0	0	0	0	11	0	0	0	0	0	0	0	0	0	0	0	0	0
0	0	0	0	0	0	0	0	0	0	0	0	0	0	0	0	0	0	0
0	0	0	0	0	0	0	0	0	0	0	0	0	0	0	0	0	0	10
0	0	0	0	0	0	0	0	0	0	0	0	0	0	0	0	0	0	0
0	0	0	0	0	0	0	0	0	0	0	0	0	0	0	0	0	0	0
0	0	0	0	0	0	0	0	0	0	0	0	0	0	0	0	0	0	0
0	0	0	0	0	0	0	0	0	0	0	0	0	0	0	0	9	0	0
0	0	0	0	0	0	0	0	0	0	0	0	0	0	0	0	0	0	0
0	0	0	0	0	0	0	0	0	0	0	0	0	0	0	0	0	0	0
0	0	0	0	0	0	0	0	0	0	0	0	0	0	0	0	0	0	0
0	0	0	0	0	0	0	0	0	0	0	0	0	0	0	0	0	0	0
0	0	0	0	0	0	0	0	0	0	0	0	0	0	0	8	0	0	0
0	0	0	0	0	0	0	0	0	0	0	0	0	0	0	0	0	0	0
0	0	0	0	0	0	0	0	0	0	0	0	0	0	0	0	0	0	0
0	0	0	0	0	0	0	0	0	0	0	0	0	0	0	0	0	0	0
0	0	0	0	0	0	0	0	0	0	0	0	0	0	0	0	0	0	0
0	0	0	0	0	0	0	0	0	0	0	0	0	0	0	0	0	0	0
0	0	0	0	0	0	0	0	0	0	0	0	0	0	0	0	0	0	0
0	0	0	0	0	0	0	0	0	0	0	0	0	0	0	0	0	0	0
0	0	0	0	0	0	0	0	0	0	0	0	0	0	0	0	0	0	0
0	0	0	0	0	0	0	0	0	0	0	0	0	0	0	0	0	0	0
0	0	0	0	0	0	0	0	0	0	0	0	0	0	0	0	0	0	0
0	0	0	0	0	0	0	0	0	0	0	0	0	0	0	0	0	0	0
0	0	0	0	0	0	0	0	0	0	0	0	0	0	0	0	0	0	0
0	0	0	0	0	0	0	0	0	0	0	6	0	0	0	0	0	0	0
0	0	0	0	0	0	0	0	0	0	0	0	0	0	0	0	0	0	0
0	0	0	0	0	0	0	0	0	0	0	0	0	0	0	0	0	0	0
0	0	0	0	0	0	0	0	0	0	0	0	0	0	0	0	0	0	0

0	0	0	0	0	0	0	0	0	0	0	0	0	0	0	0	0	0	0
0	0	0	0	0	0	0	0	0	0	0	0	0	0	0	0	0	0	0
0	0	0	0	0	0	0	0	0	0	0	0	0	0	0	0	0	0	0
0	0	0	0	0	0	0	0	0	0	0	0	0	0	0	0	0	0	0
0	0	0	0	0	0	0	5	0	0	0	0	0	0	0	0	0	0	0
0	0	0	0	0	0	0	0	0	0	0	0	0	0	0	0	0	0	0
0	0	0	0	0	0	0	0	0	0	0	0	0	0	0	0	0	0	0
0	0	0	0	0	0	0	0	0	0	0	0	0	0	0	0	0	0	0
0	0	0	0	0	0	0	0	0	0	0	0	0	0	0	0	0	0	0
0	0	0	0	0	0	0	0	0	0	0	0	0	0	0	0	0	0	0
0	0	0	0	0	0	0	0	0	0	0	0	0	0	0	0	0	0	0
0	0	0	0	0	0	0	0	0	0	0	0	0	0	0	0	0	0	0
0	0	0	0	0	0	0	0	0	0	0	0	0	0	0	0	0	0	0
0	0	0	0	0	0	0	0	0	0	0	0	0	0	0	0	0	0	0
0	0	0	0	0	0	0	0	0	0	0	0	0	0	0	0	0	0	0
0	0	0	0	0	0	0	0	0	0	0	0	0	0	0	0	0	0	0
0	0	0	0	0	0	0	0	0	0	0	0	0	4	0	0	0	0	0
0	0	0	0	0	0	0	0	0	0	0	0	0	0	0	0	0	0	0
0	0	0	0	0	0	0	0	0	0	0	0	0	0	0	0	0	0	0
0	0	0	0	0	0	0	0	0	0	0	0	0	0	0	0	0	0	0
0	0	0	0	0	0	0	0	0	0	0	0	0	0	0	0	0	0	0
0	0	0	0	0	0	0	0	0	0	0	0	0	0	0	0	0	0	0
0	0	0	0	0	0	0	0	0	0	0	0	0	0	0	0	0	0	0
0	0	0	0	0	0	0	0	0	0	0	0	0	0	0	0	0	0	0
0	0	0	0	0	0	0	0	0	0	0	0	0	0	0	0	0	0	0
0	0	0	0	0	0	0	0	0	0	0	0	0	0	0	0	0	0	0
0	0	0	0	0	0	0	0	0	0	0	0	0	0	0	0	0	0	0
0	0	0	0	0	0	0	0	0	0	0	0	0	0	0	0	0	0	0
0	0	0	0	0	0	0	0	0	0	0	0	0	0	0	0	0	0	0
0	0	0	0	0	0	0	0	0	0	0	0	0	0	0	0	0	0	0
0	0	0	0	0	0	0	0	0	0	0	0	0	0	0	0	0	0	0
0	0	0	0	0	0	0	0	0	0	0	0	0	0	0	0	0	0	0
0	0	0	0	0	0	0	0	0	0	0	0	0	0	0	0	0	0	0
0	0	0	0	0	0	0	0	0	0	0	0	0	0	0	0	0	0	0
0	0	0	0	0	0	0	0	0	3	3	0	0	0	0	0	0	0	0
0	0	0	0	0	0	0	0	0	0	0	0	0	0	0	0	0	0	0
0	0	0	0	0	0	0	0	0	0	0	0	0	0	0	0	0	0	0

0	0	0	0	0	0	0	0	0	0	0	0	0	0	0	0	0	0	0
0	0	0	0	0	0	0	0	0	0	0	0	0	0	0	0	0	0	0
0	0	0	0	0	0	0	0	0	0	0	0	0	0	0	0	0	0	0
0	0	0	0	0	0	0	0	0	0	0	0	0	0	0	0	0	0	0
0	0	0	0	0	0	0	0	0	0	0	0	0	0	0	0	0	0	0
0	0	0	0	0	0	0	0	0	0	0	0	0	0	0	0	0	0	0
0	0	0	0	0	0	0	0	0	0	0	0	0	0	0	0	0	0	0
0	0	0	0	0	0	0	0	0	0	0	0	0	0	0	0	0	0	0
0	0	0	0	0	0	0	0	0	0	0	0	0	0	0	0	0	0	0
0	0	0	0	0	0	0	0	0	0	0	0	0	0	0	0	0	0	0
0	0	0	0	0	0	0	0	0	0	0	0	0	0	0	0	0	0	0
0	0	0	0	0	0	0	0	0	0	0	0	0	0	0	0	0	0	0
0	0	0	0	0	0	0	0	0	0	0	0	0	0	0	0	0	0	0
0	0	0	0	0	0	0	0	0	0	0	0	0	0	0	0	0	0	0
0	0	0	0	0	0	0	0	0	0	0	0	0	0	0	0	0	0	0
0	0	0	0	0	0	0	0	0	0	0	0	0	0	0	0	0	0	0
0	0	0	0	0	0	0	0	0	0	0	0	0	0	0	0	0	0	0
0	0	0	0	0	0	0	0	0	0	0	0	0	0	0	0	0	0	0
0	0	0	0	0	0	0	0	0	0	0	0	0	0	0	0	0	0	0
0	0	0	0	0	0	0	0	0	0	0	0	0	0	0	0	0	0	0
0	0	0	0	0	0	0	0	0	0	0	0	0	0	0	0	0	0	0
0	0	0	0	0	0	0	0	0	0	0	0	0	0	0	0	0	0	0
0	0	0	0	0	0	0	0	0	0	0	0	0	0	0	0	0	0	0
0	0	0	2	2	2	2	2	2	0	0	0	0	0	0	0	0	0	0
0	0	0	0	0	0	0	0	0	0	0	0	0	0	0	0	0	0	0
0	0	0	0	0	0	0	0	0	0	0	0	0	0	0	0	0	0	0
0	0	0	0	0	0	0	0	0	0	0	0	0	0	0	0	0	0	0
0	0	0	0	0	0	0	0	0	0	0	0	0	0	0	0	0	0	0
0	0	0	0	0	0	0	0	0	0	0	0	0	0	0	0	0	0	0
0	0	0	0	0	0	0	0	0	0	0	0	0	0	0	0	0	0	0
0	0	0	0	0	0	0	0	0	0	0	0	0	0	0	0	0	0	0
0	0	0	0	0	0	0	0	0	0	0	0	0	0	0	0	0	0	0
0	0	0	0	0	0	0	0	0	0	0	0	0	0	0	0	0	0	0
0	0	0	0	1	1	0	0	0	0									
Jan_DL018	1096	1961	0	327	3123	271	1246	1169	235	38	71	47	16	227	418	14	295	140
108	0	192	0	179	141	45	86	26	63	110	162	31	79	13	0	70	0	100
0	24	113	118	134	0	40	5	63	0	38	0	0	63	85	0	0	33	87

88	0	0	216	53	53	62	23	10	104	31	123	38	71	65	52	106	0	0
0	0	92	6	215	16	0	66	153	0	0	0	28	42	41	0	12	80	0
47	58	34	364	26	52	0	0	0	0	0	133	186	88	0	0	0	0	17
0	168	135	45	19	29	0	0	0	0	39	0	75	44	34	6	121	194	204
0	0	0	159	0	0	219	81	86	0	0	0	19	0	27	28	0	0	0
0	19	0	0	0	0	0	0	0	0	68	0	0	0	0	35	72	51	14
0	0	15	68	38	0	0	39	0	0	0	0	0	0	17	16	0	37	0
0	166	60	0	0	0	0	37	0	29	24	57	17	0	226	0	0	54	0
15	0	0	0	0	0	52	0	0	45	161	47	49	0	0	84	0	10	0
0	0	0	0	0	0	0	0	0	0	0	0	25	11	0	31	0	0	0
44	0	0	39	0	0	0	35	0	0	0	0	0	21	8	63	0	0	48
0	0	0	0	43	12	0	0	13	0	0	0	0	20	22	21	0	0	0
0	15	0	98	0	0	0	113	5	0	0	0	0	0	0	0	0	0	0
0	4	0	0	0	0	0	0	0	29	0	57	0	0	0	0	0	89	0
0	0	0	0	29	0	23	0	0	0	0	0	0	0	0	6	0	0	0
8	0	0	0	0	50	0	0	0	0	2	12	0	0	0	0	0	0	0
14	0	19	0	0	0	0	0	0	0	0	0	0	0	0	0	0	0	30
0	0	0	8	0	8	0	4	0	0	0	0	0	0	0	80	0	0	0
0	0	0	15	0	0	0	0	0	0	21	0	66	0	0	19	0	40	0
0	0	15	0	0	0	7	0	0	0	0	0	0	0	0	0	0	0	0
0	0	0	95	0	0	10	0	0	0	0	0	0	0	0	0	0	15	0
67	0	20	0	15	0	0	0	0	0	0	11	0	0	0	0	0	0	0
0	0	79	0	4	0	0	0	0	0	0	0	0	0	0	0	33	0	5
0	0	0	25	0	13	0	0	17	0	63	0	0	0	0	0	0	0	0
0	0	0	0	0	0	0	5	1	0	0	0	0	0	0	0	0	0	0
0	0	0	0	0	0	0	0	0	0	0	0	0	0	0	23	0	0	42
0	0	0	0	0	0	0	0	0	0	0	0	0	0	0	0	0	0	0
0	0	0	0	0	0	0	0	0	0	0	0	0	6	0	0	0	0	5
0	0	0	0	0	0	0	0	0	0	0	0	0	23	0	0	0	0	0
0	15	0	0	0	0	0	0	29	0	0	0	0	6	0	0	0	12	0
0	0	0	0	0	0	0	0	0	0	0	0	0	0	0	0	0	0	0
0	0	0	0	0	0	0	0	0	0	0	8	0	0	0	0	0	0	0
0	0	0	0	0	0	0	0	0	0	0	0	0	0	0	0	0	0	13
0	0	0	0	0	0	0	0	0	0	0	0	0	0	0	0	0	0	0
0	0	0	15	23	0	0	10	0	0	0	0	0	0	0	0	0	0	0
0	0	0	0	0	0	0	0	0	0	0	0	0	0	14	0	0	0	0
0	0	0	13	0	0	3	0	0	0	0	13	0	0	0	0	0	0	0

0	0	0	0	0	0	0	0	0	7	0	28	0	0	0	0	25	0	0
0	5	0	0	0	0	25	0	0	0	0	0	0	0	0	0	0	0	0
0	0	0	0	0	0	0	0	0	0	0	0	0	0	0	0	0	0	0
0	0	0	0	0	0	0	0	0	0	0	0	0	0	0	0	0	0	0
0	0	0	0	0	0	0	0	0	0	0	0	0	0	0	0	10	0	0
0	0	0	0	0	0	0	0	0	0	0	0	0	0	0	7	0	0	0
0	0	0	0	0	0	0	0	0	0	0	0	0	0	0	0	0	0	0
0	0	0	0	0	0	0	19	0	0	0	0	0	0	0	0	0	0	0
0	0	0	0	0	0	0	0	0	0	0	0	0	0	0	0	0	0	0
0	0	10	0	0	0	0	0	0	0	0	0	0	0	0	0	0	0	0
0	0	0	0	0	0	0	0	0	0	0	0	0	0	0	0	0	0	0
0	0	0	0	5	0	0	0	0	0	0	0	0	0	0	0	0	0	0
0	0	0	0	0	0	0	0	0	0	0	0	0	0	14	0	0	0	0
0	0	0	0	0	0	0	0	0	0	0	0	0	0	0	0	0	0	0
0	0	0	0	0	0	0	0	0	0	0	0	0	0	0	0	0	0	0
0	0	0	0	0	0	0	0	0	0	0	0	0	0	0	0	0	0	0
0	0	0	0	0	0	0	0	0	0	0	0	0	0	0	0	0	0	0
0	0	0	0	0	0	0	0	0	0	0	0	0	0	0	0	0	0	0
0	0	0	0	0	0	0	0	0	0	0	0	0	0	0	0	0	0	0
0	0	0	0	0	9	0	0	0	0	0	0	0	0	0	0	0	0	0
0	0	0	0	0	0	0	0	0	0	0	0	0	0	0	0	0	0	0
0	0	3	0	0	0	0	0	0	0	0	0	0	0	0	0	0	0	0
0	0	0	0	0	0	0	0	0	0	0	1	0	0	0	0	0	0	0
0	0	0	0	0	0	0	0	0	0	0	0	0	0	0	0	0	0	0
0	0	0	0	0	0	0	0	0	0	0	0	0	0	0	0	0	0	0
0	0	0	0	0	0	0	0	0	0	0	0	0	0	0	0	0	0	0
0	0	0	0	0	0	0	0	0	0	0	0	0	0	0	0	0	0	0
0	0	0	0	0	0	0	0	0	0	0	0	0	0	0	0	0	0	0
0	0	0	0	0	0	0	0	0	0	0	0	0	0	0	0	0	0	0
0	0	0	0	0	0	0	0	0	0	0	0	0	0	0	0	0	0	0
0	0	0	0	0	0	0	0	0	0	0	0	0	0	0	0	0	0	0
0	0	0	0	0	0	0	0	0	0	0	0	0	0	0	0	0	0	0
0	0	0	0	0	0	0	0	0	0	0	0	0	0	0	0	0	0	0
0	0	0	0	0	0	0	0	0	0	0	0	0	0	0	0	0	0	0
0	0	0	0	0	0	0	0	0	0	0	0	0	0	0	14	0	0	0
0	0	0	0	0	0	0	0	0	0	0	0	0	0	0	0	0	0	0
0	0	0	0	0	0	0	0	0	0	0	0	0	0	0	0	0	0	0

0	0	0	0	0	0	0	0	0	0	0	0	0	0	0	0	0	0	0
0	0	0	0	0	0	0	0	0	0	0	0	0	0	0	0	0	0	0
0	0	0	0	0	0	0	0	0	0	0	0	0	0	0	0	0	0	0
0	0	0	0	0	0	0	0	0	0	0	0	0	0	0	0	0	0	0
0	0	0	0	0	0	0	0	0	0	0	0	0	0	0	0	0	0	0
0	0	0	0	0	0	0	0	0	0	0	0	0	0	0	0	0	0	0
0	0	0	0	0	0	0	0	0	0	0	0	0	0	0	0	0	0	0
0	0	0	0	0	0	0	0	0	0	0	0	0	0	0	0	0	0	0
0	0	0	0	0	0	0	0	0	0	0	0	0	0	0	0	0	0	0
0	0	0	0	0	0	0	0	0	0	0	0	0	0	0	0	0	0	0
0	0	0	0	0	0	0	0	0	0	0	0	0	0	0	0	0	0	0
0	0	0	0	0	0	0	0	0	0	0	0	0	0	0	0	0	0	0
0	0	0	0	0	0	0	0	0	0	0	0	0	0	0	0	0	0	0
0	0	0	0	0	0	0	0	0	0	0	0	0	0	0	0	0	0	0
0	0	0	0	0	0	0	0	0	0	0	0	0	0	0	0	0	0	0
0	0	0	0	0	0	0	0	0	0	0	0	0	0	0	0	0	0	0
0	0	0	0	0	0	0	0	0	0	0	0	0	0	0	0	0	0	0
0	0	0	0	0	0	0	0	0	0	0	0	0	0	0	0	0	0	0
0	0	0	0	0	0	0	0	0	0	0	0	0	0	0	0	0	0	0
0	0	0	0	0	0	0	0	0	0	0	0	0	0	0	0	0	0	0
0	0	0	0	0	0	0	0	0	0	0	0	0	0	0	0	0	0	0
0	0	0	0	0	0	0	0	0	0	0	0	0	0	0	0	0	0	0
0	0	0	0	0	0	0	0	0	0	0	0	0	0	0	0	0	0	0
0	0	0	0	0	0	0	0	0	0	0	0	0	0	0	0	0	0	0
0	0	0	0	0	0	0	0	0	0	0	0	0	0	0	0	0	0	0
0	0	0	0	0	0	0	0	0	0	0	0	0	0	0	0	0	0	0
0	0	0	0	0	0	0	0	0	0	0	0	0	0	0	0	0	0	0
0	0	0	0	0	0	0	0	0	0	0	0	0	0	0	0	0	0	0
0	0	0	0	0	0	0	0	0	0	0	0	0	0	0	0	0	0	0
0	0	0	0	0	0	0	0	0	0	0	0	0	0	0	0	0	0	0
0	0	0	0	0	0	0	0	0	0	0	0	0	0	0	0	0	0	0
0	0	0	0	0	0	0	0	0	0	0	0	0	0	0	0	0	0	0
0	0	0	0	0	0	0	0	0	0	0	0	0	0	0	0	0	0	0
0	0	0	0	0	0	0	0	0	0	0	0	0	0	0	0	0	0	0
0	0	0	0	0	0	0	0	0	0	0	0	0	0	0	0	0	0	0
0	0	0	0	0	0	0	0	0	0	0	0	0	0	0	0	0	0	0
0	0	0	0	0	0	0	0	0	0	0	0	0	0	0	0	0	0	0

0	0	0	0	0	0	0	0	0	0	0	0	0	0	0	0	0	0	0
0	0	0	0	0	0	0	0	0	0	0	0	0	0	0	0	0	0	0
0	0	0	0	0	0	0	0	0	0	0	0	0	0	0	0	0	0	0
0	0	0	0	0	0	0	0	0	0	0	0	0	0	0	0	0	0	0
0	0	0	0	0	0	0	0	0	0	0	0	0	0	0	0	0	0	0
0	0	0	0	0	0	0	0	0	0	0	0	0	0	0	0	0	0	0
0	0	0	0	0	0	0	0	0	0	0	0	0	0	0	0	0	0	0
0	0	0	0	0	0	0	0	0	0	0	0	0	0	0	0	0	0	0
0	0	0	0	0	0	0	0	0	0	0	0	0	0	0	0	0	0	0
0	0	0	0	0	0	0	0	0	0	0	0	0	0	0	0	0	0	0
0	0	0	0	0	0	0	0	0	0	0	0	0	0	0	0	0	0	0
0	0	0	0	0	0	0	0	0	0	0	0	0	0	0	0	0	0	0
0	0	0	0	0	0	0	0	0	0	0	0	0	0	0	0	0	0	0
0	0	0	0	0	0	0	0	0	0	0	0	0	0	0	0	0	0	0
0	0	0	0	0	0	0	0	0	0	0	0	0	0	0	0	0	0	0
0	0	0	0	0	0	0	0	0	0	0	0	0	0	0	0	0	0	0
0	0	0	0	0	0	0	0	0	0	0	0	0	0	0	0	0	0	0
0	0	0	0	0	0	0	0	0	0	0	0	0	0	0	0	0	0	0
0	0	0	0	0	0	0	0	0	0	0	0	0	0	0	0	0	0	0
0	0	0	0	0	0	0	0	0	0	0	0	0	0	0	0	0	0	0
0	0	0	0	0	0	0	0	0	0	0	0	0	0	0	0	0	0	0
0	0	0	0	0	0	0	0	0	0	0	0	0	0	0	0	0	0	0
0	0	0	0	0	0	0	0	0	0	0	0	0	0	0	0	0	0	0
0	0	0	0	0	0	0	0	0	0	0	0	0	0	0	0	0	0	0
0	0	0	0	0	0	0	0	0	0	0	0	0	0	0	0	0	0	0
0	0	0	0	0	0	0	0	0	0	0	0	0	0	0	0	0	0	0
0	0	0	0	0	0	0	0	0	0	0	0	0	0	0	0	0	0	0
0	0	0	0	0	0	0	0	0	0	0	0	0	0	0	0	0	0	0
0	0	0	0	0	0	0	0	0	0	0	0	0	0	0	0	0	0	0
0	0	0	0	0	0	0	0	0	0	0	0	0	0	0	0	0	0	0
0	0	0	0	0	0	0	0	0	0	0	0	0	0	0	0	0	0	0
0	0	0	0	0	0	0	0	0	0	0	0	0	0	0	0	0	0	0
0	0	0	0	0	0	0	0	0	0	0	0	0	0	0	0	0	0	0
0	0	0	0	0	0	0	0	0	0	0	0	0	0	0	0	0	0	0
0	0	0	0	0	0	0	0	0	0	0	0	0	0	0	0	0	0	0
0	0	0	0	0	0	0	0	0	0	0	0	0	0	0	0	0	0	0
0	0	0	0	0	0	0	0	0	0	0	0	0	0	0	0	0	0	0

0	0	0	0	0	0	0	0	0	0	0	0	0	0	0	0	0	0	0
0	0	0	0	0	0	0	0	0	0	0	0	0	0	0	0	0	0	0
0	0	0	0	0	0	0	0	0	0	0	0	0	0	0	0	0	0	0
0	0	0	0	0	0	0	0	0	0	0	0	0	0	0	0	0	0	0
0	0	0	0	0	0	0	0	0	0	0	0	0	0	0	0	0	0	0
0	0	0	0	0	0	0	0	0	0	0	0	0	0	0	0	0	0	0
0	0	0	0	0	0	0	0	0	0	0	0	0	0	0	0	0	0	0
0	0	0	0	0	0	0	0	0	0	0	0	0	0	0	0	0	0	0
0	0	0	0	0	0	0	0	0	0	0	0	0	0	0	0	0	0	0
0	0	0	0	0	0	0	0	0	0	0	0	0	0	0	0	0	0	0
0	0	0	0	0	0	0	0	0	0	0	0	0	0	0	0	0	0	0
0	0	0	0	0	0	0	0	0	0	0	0	0	0	0	0	0	0	0
0	0	0	0	0	0	0	0	0	0	0	0	0	0	0	0	0	0	0
0	0	0	0	0	0	0	0	0	0	0	0	0	0	0	0	0	0	0
0	0	0	0	0	0	0	0	0	0	0	0	0	0	0	0	0	0	0
0	0	0	0	0	0	0	0	0	0	0	0	0	0	0	0	0	0	0
0	0	0	0	0	0	0	0	0	0	0	0	0	0	0	0	0	0	0
0	0	0	0	0	0	0	0	0	0	0	0	0	0	0	0	0	0	0
0	0	0	0	0	0	0	0	0	0	0	0	0	0	0	0	0	0	0
0	0	0	0	0	0	0	0	0	0	0	0	0	0	0	0	0	0	0
0	0	0	0	0	0	0	0	0	0	0	0	0	0	0	0	0	0	0
2	2	0	0	0	0	0	0	0	0	0	0	0	0	0	0	0	0	0
0	0	0	0	0	0	0	0	0	0	0	0	0	0	0	0	0	0	0
0	0	0	0	0	0	0	0	0	0	0	0	0	0	0	0	0	0	0
0	0	0	0	0	0	0	0	0	0	0	0	0	0	0	0	0	0	0
0	0	0	0	0	0	0	0	0	1									
Jan_DL002	1405	2443	8	193	1513	364	714	796	106	98	93	209	73	297	132	0	225	157
186	0	326	160	169	317	71	171	75	0	261	0	93	95	31	0	127	19	0
0	0	346	81	101	0	87	0	283	0	125	0	35	98	56	5	0	100	0
0	0	0	154	107	50	71	42	17	63	131	187	34	57	127	53	72	162	0
13	0	37	12	0	0	31	34	0	10	0	0	18	34	51	0	46	71	0
116	22	0	0	0	119	0	42	59	0	0	277	0	0	12	30	23	0	50
0	0	0	0	27	32	0	0	0	0	100	77	43	0	0	3	15	0	0
0	0	71	14	0	0	0	237	11	20	130	0	25	0	0	17	0	0	0
0	0	0	9	0	8	0	0	0	0	82	0	0	0	0	0	0	80	56
8	0	18	0	37	0	21	29	0	0	0	0	0	18	0	72	17	41	0
0	0	0	11	0	34	0	55	0	37	0	55	0	73	0	0	0	156	0

0	83	0	0	0	0	23	0	0	51	0	104	118	0	0	45	0	51	0
0	0	0	0	0	0	0	0	0	0	0	0	20	19	0	17	0	0	0
40	0	0	19	9	0	0	107	0	0	0	0	0	0	15	0	19	0	57
0	10	0	49	10	0	5	0	0	0	0	6	0	5	0	33	0	0	0
0	0	7	0	0	0	0	11	0	0	0	0	2	0	20	10	63	26	18
0	9	0	0	0	15	0	17	0	18	0	0	15	0	12	0	0	0	6
11	0	0	0	0	0	0	4	0	19	0	0	0	9	25	0	0	0	0
8	0	0	0	0	0	0	0	30	6	5	3	0	15	0	0	0	0	0
0	0	0	0	0	0	0	0	0	41	0	0	0	0	0	0	0	0	0
0	0	0	0	0	0	5	0	18	0	0	17	0	0	0	0	0	29	0
0	0	0	0	0	0	0	32	0	0	0	0	32	0	0	5	13	0	0
0	0	17	0	0	0	13	0	0	0	0	6	0	0	0	0	0	0	0
0	4	0	0	0	0	0	0	0	40	0	0	0	0	0	0	9	0	0
0	0	13	0	16	0	0	0	0	0	0	0	0	0	0	0	0	0	0
0	0	0	0	0	0	0	8	0	0	0	0	0	0	0	0	0	0	5
22	0	0	0	0	0	0	0	0	0	85	0	0	0	0	0	0	0	0
0	0	0	0	0	0	0	41	0	0	0	0	0	0	0	0	0	0	0
0	0	0	0	0	0	0	7	0	7	0	0	0	0	0	0	0	0	0
0	0	11	0	17	0	0	0	0	0	0	0	0	0	0	0	0	0	0
0	0	0	15	0	0	0	0	0	0	0	0	0	20	0	0	0	0	0
0	0	0	0	0	18	0	0	0	0	0	0	0	0	0	0	0	0	0
0	0	0	0	0	0	0	0	0	0	0	0	0	0	0	0	6	6	0
0	0	0	0	0	0	0	0	0	0	0	0	0	0	8	0	0	0	0
0	0	0	0	0	0	0	0	0	0	0	18	0	0	0	0	0	0	0
11	0	0	0	0	0	0	0	0	0	0	0	0	0	0	9	0	0	0
0	0	9	0	0	0	0	0	0	0	0	0	0	0	0	0	0	0	0
0	0	0	0	0	0	0	0	0	0	0	0	0	0	0	0	0	0	0
0	0	0	0	0	0	0	0	0	0	0	0	5	0	0	0	0	0	0
0	0	21	0	0	0	0	0	0	0	0	0	0	0	0	0	0	0	0
0	0	0	0	0	0	0	0	0	0	0	0	0	0	0	0	0	0	0
0	0	0	0	0	0	40	0	0	0	0	0	0	0	0	0	0	0	0
0	0	0	0	0	0	0	0	0	0	0	0	0	0	0	0	0	0	12
0	0	0	0	0	0	0	0	0	0	0	0	0	0	0	0	0	0	0
0	0	0	3	0	0	0	0	38	0	0	0	0	0	0	0	0	0	0
0	0	0	0	0	0	0	0	0	0	0	0	0	0	0	0	0	0	0
0	0	0	0	0	0	0	0	0	0	0	0	0	0	0	0	0	0	0
0	0	0	0	0	0	0	0	0	0	0	0	0	0	0	0	0	0	0

0	0	0	0	0	0	0	0	0	0	0	0	0	0	0	0	0	0	0
0	0	0	0	0	0	0	0	0	0	0	0	0	0	0	0	0	0	6
0	0	0	0	0	0	0	0	0	0	0	0	0	0	0	0	0	0	0
0	0	0	0	0	0	0	0	0	0	0	0	0	0	0	0	0	0	0
0	0	0	0	0	0	0	0	0	0	0	0	0	0	0	0	0	0	0
0	0	0	0	0	0	0	0	0	0	0	0	0	0	0	0	0	0	0
0	0	0	0	0	0	0	0	0	0	0	0	0	0	0	0	0	0	0
0	0	0	0	0	0	0	0	0	0	0	0	0	0	0	0	0	0	0
0	0	0	0	0	0	0	0	0	0	0	0	0	0	0	0	0	0	0
0	9	0	0	0	10	0	0	0	0	0	0	0	0	0	0	0	0	0
0	0	0	0	0	0	0	0	0	0	0	0	0	0	15	0	7	0	0
0	0	0	0	0	0	0	0	0	0	0	0	0	0	0	0	0	0	0
0	0	0	0	0	0	0	0	0	0	0	0	0	0	0	0	0	0	0
0	0	0	0	0	0	0	0	0	0	0	0	0	0	0	0	0	0	0
0	0	0	0	0	0	0	0	0	0	0	0	0	0	0	0	0	0	0
0	0	0	0	0	0	0	0	0	0	0	0	0	0	0	0	0	0	0
0	19	0	0	0	0	0	0	0	0	0	0	0	0	0	0	0	0	0
0	0	0	0	0	0	0	0	0	0	0	0	0	0	0	0	0	0	0
0	0	0	0	0	0	0	0	0	17	0	0	0	0	0	0	0	0	0
0	0	0	0	0	0	0	0	0	0	0	0	0	0	0	0	0	0	0
0	0	0	0	0	0	0	0	0	0	0	0	0	0	0	0	0	0	0
0	0	0	0	0	0	0	0	0	0	0	4	0	15	0	0	0	0	0
0	0	0	0	0	0	0	0	0	0	0	0	0	0	0	0	0	0	0
0	0	0	0	0	0	0	0	0	0	0	0	0	0	0	0	0	0	0
0	0	0	0	0	0	0	0	0	0	0	0	0	0	0	0	0	0	0
0	0	0	0	0	0	0	0	0	0	0	0	0	0	0	0	0	0	0
0	0	0	0	0	0	0	0	0	0	0	0	0	0	0	0	0	0	0
0	0	0	0	0	0	0	0	0	0	0	0	0	0	0	0	0	0	0
0	0	0	0	0	0	0	0	0	0	0	0	0	0	0	0	0	0	0
0	0	0	0	0	0	0	0	0	0	0	0	0	0	0	0	0	0	0
0	0	0	0	0	0	0	0	0	0	0	0	0	0	0	0	0	0	0
0	0	0	0	0	0	0	0	0	0	0	0	0	0	0	0	0	0	0
0	0	0	0	0	0	0	0	0	0	0	0	0	0	0	0	0	0	0
0	0	0	0	0	0	0	0	0	0	0	0	0	0	0	0	0	0	0
0	0	0	0	0	0	0	0	0	0	0	0	0	0	0	0	0	0	0
0	0	0	0	0	0	0	0	0	0	0	0	0	0	4	0	0	0	0
0	0	0	0	0	0	0	0	0	0	0	0	0	0	0	0	0	0	0

0	0	0	0	0	0	0	0	0	0	0	0	0	0	0	0	0	0	0
0	0	0	0	0	0	0	0	0	0	0	0	0	0	0	0	0	0	0
0	0	0	0	0	0	0	0	0	0	0	0	0	0	0	0	0	0	0
0	0	0	0	0	0	0	0	0	0	0	0	0	0	0	0	0	0	0
0	0	0	0	0	0	0	0	0	0	0	0	0	0	0	0	0	0	0
0	0	0	0	0	0	0	0	0	0	0	0	0	0	0	0	0	0	0
0	0	0	0	0	0	0	0	0	0	0	0	0	0	0	0	0	0	0
0	0	0	0	0	0	0	0	0	0	0	0	0	0	0	0	0	0	0
0	0	0	0	0	0	0	0	0	0	0	0	0	0	0	0	0	0	0
0	0	0	0	0	0	0	0	0	0	0	0	0	0	0	0	0	0	0
7	0	0	0	0	0	0	0	0	0	0	0	0	0	0	0	0	0	0
0	0	0	0	0	0	0	0	0	0	0	0	0	0	0	0	0	0	0
0	0	0	0	0	0	0	0	0	0	0	0	0	0	0	0	0	0	0
0	0	0	0	0	0	0	0	0	0	0	0	0	0	0	0	0	0	0
0	0	0	0	0	0	0	0	0	0	0	0	0	0	0	0	0	0	0
0	0	0	0	0	0	0	0	0	0	0	0	0	0	0	0	0	0	0
0	0	0	0	0	0	0	0	0	0	0	0	0	0	0	0	0	0	0
0	0	0	0	0	0	0	0	0	0	0	0	0	0	0	0	0	0	0
0	0	0	0	0	0	0	0	0	0	0	0	0	0	0	0	0	0	0
0	0	0	0	0	0	0	0	0	0	0	0	0	0	0	0	0	0	0
0	0	0	0	0	0	0	0	0	0	0	0	0	0	0	0	0	0	0
0	0	0	0	0	0	0	0	0	0	0	0	0	0	0	0	0	0	0
0	0	0	0	0	0	0	0	0	0	0	0	0	0	0	0	0	0	0
0	0	0	0	0	0	0	0	0	0	0	0	0	0	0	0	0	0	0
0	0	0	0	0	0	0	0	0	0	0	0	0	0	0	0	0	0	0
0	0	0	0	0	0	0	0	0	0	0	0	0	0	0	0	0	0	0
0	0	0	0	0	0	0	0	0	0	0	0	0	0	0	0	0	0	0
0	0	0	0	0	0	0	0	0	0	0	0	0	0	0	0	0	0	0
0	0	0	0	0	0	0	0	0	0	0	0	0	0	0	0	0	0	0
0	0	0	0	0	0	0	0	0	0	0	0	0	0	0	0	0	0	0
0	0	0	0	0	0	0	0	0	0	0	0	0	0	0	0	0	0	0
0	0	0	0	0	0	0	0	0	0	0	0	0	0	0	0	0	0	0
0	0	0	0	0	0	0	0	0	0	0	0	0	0	0	0	0	0	0
0	0	0	0	0	0	0	0	0	0	0	0	0	0	0	0	0	0	0
0	0	0	0	0	0	0	0	0	0	0	0	0	0	0	0	0	0	0
0	0	0	0	0	0	0	0	0	0	0	0	0	0	0	0	0	0	0
0	0	0	0	0	0	0	0	0	0	0	0	0	0	0	0	0	0	0

0	0	0	0	0	0	0	0	0	0	0	0	0	0	0	0	0	0	0
0	0	0	0	0	0	0	0	0	0	0	0	0	0	0	0	0	0	0
0	0	0	0	0	0	0	0	0	0	0	0	0	0	0	0	0	0	0
0	0	0	0	0	0	0	0	0	0	0	0	0	0	0	0	0	0	0
0	0	0	0	0	0	0	0	0	0	0	0	0	0	0	0	0	0	0
0	0	0	0	0	0	0	0	0	0	0	0	0	0	0	0	0	0	0
0	0	0	0	0	0	0	0	0	0	0	0	0	0	0	0	0	0	0
0	0	0	0	0	0	0	0	0	0	0	0	0	0	0	0	0	0	0
0	0	0	0	0	0	0	0	0	0	0	0	0	0	0	0	0	0	0
0	0	0	0	0	0	0	0	0	0	0	0	0	0	0	0	0	0	0
0	0	0	0	0	0	0	0	0	0	0	0	0	0	0	0	0	0	0
0	0	0	0	0	0	0	0	0	0	0	0	0	0	0	0	0	0	0
0	0	0	0	0	0	0	0	0	0	0	0	0	0	0	0	0	0	0
0	0	0	0	0	0	0	0	0	0	0	0	0	0	0	0	0	0	0
0	0	0	0	0	0	0	0	0	0	0	0	0	0	0	0	0	0	0
0	0	0	0	0	0	0	0	0	0	0	0	0	0	0	0	0	0	0
0	0	0	0	0	0	0	0	0	0	0	0	0	0	0	0	0	0	0
0	0	0	0	0	0	0	0	0	0	0	0	0	0	0	0	0	0	0
0	0	0	0	0	0	0	0	0	0	0	0	0	0	0	0	0	0	0
0	0	0	0	0	0	0	0	0	0	0	0	0	0	0	0	0	0	0
0	0	0	0	0	0	0	0	0	0	0	0	0	0	0	0	0	0	0
0	0	0	0	0	0	0	0	0	0	0	0	0	0	0	0	0	0	0
0	0	0	0	0	0	0	0	0	0	0	0	0	0	0	0	0	0	0
0	0	0	0	0	0	0	0	0	0	0	0	0	0	0	0	0	0	0
0	0	0	0	0	0	0	0	0	0	0	0	0	0	0	0	0	0	0
0	0	0	0	0	0	0	0	0	0	0	0	0	0	0	0	0	0	0
0	0	0	0	0	0	0	0	0	0	0	0	0	0	0	0	0	0	0
0	0	0	0	0	0	0	0	0	0	0	0	0	0	0	0	0	0	0
0	0	0	0	0	0	0	0	0	0	0	0	0	0	0	0	0	0	0
0	0	0	0	0	0	0	0	0	0	0	0	0	0	0	0	0	0	0
0	0	0	0	0	0	0	0	0	0	0	0	0	0	0	0	0	0	0
0	0	0	0	0	0	0	0	0	0	0	0	0	0	0	0	0	0	0
0	0	0	0	0	0	0	0	0	0	0	0	0	0	0	0	0	0	0
0	0	0	0	0	0	0	0	0	0	0	0	0	0	0	0	0	0	0
0	0	0	0	0	0	0	0	0	0	0	0	0	0	0	0	0	0	0
0	0	0	0	0	0	0	0	0	0	0	0	0	0	0	0	0	0	0
0	0	0	0	0	0	0	0	0	0	0	0	0	0	0	0	0	0	0

0	0	0	0	0	0	0	0	0	0	0	0	0	0	0	0	0	0	0
0	0	0	0	0	0	0	0	0	0	0	0	0	0	0	0	0	0	0
0	0	0	0	0	0	0	0	0	0	0	0	0	0	0	0	0	0	0
0	0	0	0	0	0	0	0	0	0	0	0	0	0	0	0	0	0	0
0	0	0	0	0	0	0	0	0	0	0	0	0	0	0	0	0	0	0
0	0	0	0	0	0	0	0	0	0	0	0	0	0	0	0	0	0	0
0	0	0	0	0	0	0	0	0	0	0	0	0	0	0	0	0	0	0
0	0	0	0	0	0	0	0	0	0	0	0	0	0	0	0	0	0	0
0	0	0	0	0	0	0	0	0	0	0	0	0	0	0	0	0	0	0
0	0	0	0	0	0	0	0	0	0	0	0	0	0	0	0	0	0	0
0	0	0	0	0	0	0	0	0	0	0	0	0	0	0	0	0	0	0
0	0	0	0	0	0	0	0	0	0	0	0	0	0	0	0	0	0	0
0	0	0	0	0	0	0	0	0	0	0	0	0	0	0	0	0	0	0
0	0	0	0	0	0	0	0	0	0	0	0	0	0	0	0	0	0	0
0	0	0	0	0	0	0	0	0	0	0	0	0	0	0	0	0	0	0
0	0	0	0	0	0	0	0	0	0	0	0	0	0	0	0	0	0	0
0	0	0	0	0	0	0	0	0	0	0	0	0	0	0	0	0	0	0
0	0	0	0	0	0	0	0	0	0									
Jan_DL020	1066	934	596	423	606	289	235	216	147	311	85	413	275	111	433	148	137	100
128	44	251	524	120	194	329	111	260	249	137	0	130	154	133	0	65	533	0
184	173	159	24	101	0	0	210	118	51	61	65	184	113	31	67	144	95	0
0	136	55	112	55	132	54	76	158	88	15	92	18	51	56	54	41	34	0
62	57	31	0	0	60	84	0	101	71	0	0	34	16	30	82	14	63	0
45	45	0	0	0	4	138	203	71	5	0	0	46	30	23	52	0	0	170
0	29	142	6	7	56	0	0	0	0	14	23	16	22	54	0	0	40	0
0	0	0	32	0	87	0	0	0	22	0	0	12	0	0	22	0	0	0
0	26	33	8	0	0	19	34	57	0	7	0	18	26	87	0	27	0	0
16	0	0	0	16	0	0	0	0	0	0	15	0	42	0	0	19	19	21
0	0	0	0	0	36	0	0	0	11	0	0	44	0	0	0	0	108	0
0	44	41	0	0	0	21	0	0	0	0	111	98	0	0	18	0	0	0
0	0	0	0	0	0	0	0	0	42	0	21	0	6	0	0	31	23	0
0	0	0	30	39	0	0	69	0	0	0	0	0	19	6	0	0	0	0
0	0	0	0	0	6	0	0	28	0	16	0	0	8	0	11	0	0	0
0	0	15	0	0	0	0	0	0	0	0	0	0	26	0	34	0	0	0
0	12	0	0	0	0	0	104	0	0	0	13	15	16	10	14	0	0	9
0	0	9	0	0	0	0	8	3	0	0	0	0	19	9	0	0	0	0
0	0	0	0	17	0	0	0	0	17	0	0	11	15	0	0	20	0	0

0	0	0	0	26	0	0	0	0	0	0	0	17	0	18	0	0	0	0
0	0	0	0	0	0	8	0	0	0	0	0	0	0	9	0	0	8	0
0	0	0	0	0	0	0	0	0	11	0	0	4	3	0	0	0	0	0
0	0	0	10	0	0	0	58	7	0	0	0	0	0	0	0	4	0	0
0	5	0	0	0	0	0	36	0	0	50	0	0	0	0	0	0	9	0
6	0	9	0	0	0	0	0	0	0	0	0	0	43	0	0	0	0	0
0	0	0	19	9	0	0	0	17	0	0	0	0	18	18	0	0	0	0
13	0	0	0	0	0	0	0	0	0	0	0	0	0	0	0	0	0	0
0	0	0	52	0	0	0	0	21	0	0	0	0	0	0	8	0	0	0
0	0	0	0	0	22	0	0	0	5	0	0	0	0	0	0	0	0	0
0	0	0	0	0	0	0	0	0	0	6	0	0	0	0	0	0	0	0
0	0	0	5	0	0	0	0	0	57	0	0	0	0	5	0	0	0	0
0	0	22	0	0	0	0	0	0	0	0	0	0	0	0	0	0	0	0
0	0	0	0	0	13	0	0	0	0	0	0	0	0	0	0	0	0	17
0	0	0	0	0	0	0	0	0	0	0	0	0	0	0	0	0	0	0
0	0	80	0	0	0	0	18	0	0	0	0	0	0	0	0	0	4	0
0	0	0	0	0	0	0	58	0	0	0	0	0	0	0	0	0	0	0
0	0	0	0	0	0	0	0	4	0	10	0	0	0	0	19	0	11	0
0	0	0	0	0	0	0	0	0	0	0	0	0	0	0	0	0	0	0
0	0	0	0	0	0	0	0	0	0	0	16	0	0	5	0	0	2	0
6	0	0	0	0	0	0	0	0	0	0	0	0	0	0	0	0	0	0
0	0	0	0	0	0	0	0	0	0	0	0	0	21	0	0	0	0	0
0	0	0	0	0	0	0	0	0	0	0	0	0	0	0	0	0	0	0
0	0	0	0	0	0	0	0	0	0	0	0	0	0	0	0	0	0	0
0	0	0	0	0	0	0	0	4	13	0	0	0	0	0	0	0	0	9
0	0	0	0	0	0	0	0	0	0	0	0	0	0	0	0	0	0	0
0	0	0	0	0	0	0	0	0	0	0	0	0	0	0	0	0	0	0
0	0	0	0	0	0	13	0	0	0	16	0	0	0	0	0	0	7	0
0	0	0	0	0	0	0	0	0	0	0	0	0	0	0	0	0	0	0
0	0	49	0	0	0	0	0	0	16	0	0	0	0	0	0	0	0	0
0	0	0	0	0	0	0	0	0	0	0	0	0	0	0	0	0	0	0
0	0	0	0	0	0	0	0	0	0	0	0	0	0	0	0	0	0	0
0	0	0	0	0	0	0	0	0	0	0	0	0	0	0	10	0	0	0
0	9	0	0	0	0	0	10	0	0	0	0	0	0	0	35	0	0	0
0	0	0	0	0	0	0	0	0	0	0	0	0	0	0	0	0	0	0
0	0	0	0	0	0	0	0	0	0	9	0	0	0	0	0	0	0	0
0	0	0	0	0	6	0	0	0	0	0	0	0	0	0	0	0	0	0

0	0	0	0	0	0	0	0	0	0	0	0	0	0	0	0	0	0	0
0	0	0	0	0	0	0	0	0	0	0	0	0	0	0	0	0	0	0
0	0	0	0	0	0	0	0	0	0	0	0	0	0	0	0	15	0	0
0	0	0	0	0	0	0	0	0	0	0	0	0	0	0	0	0	0	0
0	0	0	0	0	0	0	0	0	0	0	0	0	0	0	0	0	0	0
0	0	0	0	0	0	0	0	0	0	0	0	0	0	0	0	0	0	0
0	0	0	0	0	0	0	0	0	0	0	9	0	0	0	0	0	0	0
0	0	0	0	0	0	0	0	0	0	0	0	0	0	0	0	0	0	0
0	0	0	0	0	0	0	0	0	0	0	0	0	0	0	0	0	0	0
0	0	0	0	0	0	0	0	0	0	0	17	0	0	0	0	0	0	0
0	0	0	0	0	0	0	0	0	0	0	0	0	0	0	0	0	0	0
0	0	0	0	0	0	0	0	0	0	0	0	0	0	0	0	0	0	0
0	0	0	0	0	0	0	0	0	0	0	0	0	0	0	0	0	0	0
0	0	0	0	0	0	0	0	0	0	0	0	0	0	0	0	0	0	0
0	0	0	0	11	0	0	0	0	0	0	0	0	0	0	0	0	0	0
0	0	0	0	0	0	0	0	0	0	0	0	0	0	0	0	0	0	0
0	0	0	0	0	0	0	0	0	0	0	0	0	0	0	0	0	0	0
0	0	0	0	0	0	0	0	0	0	0	0	0	0	0	0	0	0	0
0	0	0	0	0	0	0	0	0	0	0	0	0	0	0	0	0	0	0
0	0	0	0	0	0	0	0	0	0	0	0	0	0	0	0	0	0	0
0	0	0	0	0	0	0	0	0	0	0	0	0	0	0	0	0	0	11
0	0	0	0	0	0	0	0	0	0	0	0	0	0	0	0	0	0	0
0	0	0	0	0	0	0	0	0	0	0	0	0	0	0	0	0	0	0
0	0	0	0	0	0	0	0	0	0	0	0	0	0	0	0	0	0	0
0	0	10	0	0	0	0	0	0	0	0	0	0	0	0	0	0	0	0
0	0	0	0	0	0	0	0	0	0	0	0	0	0	0	0	0	0	0
0	0	0	0	0	0	0	0	0	0	0	0	0	0	0	0	0	0	0
0	0	0	0	0	0	0	0	0	0	0	0	0	0	0	0	0	0	0
0	0	0	0	0	0	0	0	0	0	0	0	0	0	0	0	0	0	0
0	0	0	0	0	0	0	0	0	0	0	0	0	0	0	0	0	0	0
0	0	0	0	0	0	0	0	0	0	0	0	0	0	0	0	0	0	0
0	0	0	0	0	0	0	0	0	0	0	0	0	0	0	0	0	0	0
0	0	0	0	0	0	0	0	0	0	0	0	0	0	1	0	0	0	0
0	0	0	0	0	0	0	0	0	0	0	0	0	0	0	0	0	0	0
0	0	0	0	0	0	0	0	0	0	0	0	0	0	0	0	0	0	0
0	0	0	0	0	0	0	0	0	0	0	0	0	0	0	0	0	0	0
0	0	0	0	0	0	0	0	0	0	0	0	0	0	0	0	0	0	0

0	0	7	7	0	0	0	0	0	0	0	0	0	0	0	0	0	0	0
0	0	0	0	0	0	0	0	0	0	0	0	0	0	0	0	0	0	0
0	0	0	0	0	0	0	0	0	0	0	0	0	0	0	0	0	0	0
0	0	0	0	0	0	0	0	0	0	0	0	0	0	0	0	0	0	0
0	0	0	0	0	0	0	0	0	0	0	0	0	0	0	0	0	0	0
0	0	0	0	0	0	0	0	0	0	0	0	0	0	0	0	0	0	0
0	0	0	0	0	0	0	0	0	0	0	0	0	0	0	0	0	0	0
0	0	0	0	0	0	0	0	0	0	0	0	0	0	0	6	0	0	0
0	0	0	0	0	0	0	0	0	0	0	0	0	0	0	0	0	0	0
0	0	0	0	0	0	0	0	0	0	0	0	0	0	0	0	0	0	0
0	0	0	0	0	0	0	0	0	0	0	0	0	0	0	0	0	0	0
0	0	0	0	0	0	0	0	0	0	0	0	0	0	0	0	0	0	0
0	0	0	0	0	0	0	0	0	0	0	0	0	0	0	0	0	0	0
0	0	0	0	0	0	0	0	0	0	0	0	0	0	0	0	0	0	0
0	0	0	0	0	0	0	0	0	0	0	0	0	0	0	0	0	0	0
0	0	0	0	0	0	0	0	0	0	0	0	0	0	0	0	0	5	5
0	0	0	0	0	0	0	0	0	0	0	0	0	0	0	0	0	0	0
0	0	0	0	0	0	0	0	0	0	0	0	0	0	0	0	0	0	0
0	0	0	0	0	0	0	0	0	0	0	0	0	0	0	0	0	0	0
0	0	0	0	0	0	0	0	0	0	0	0	0	0	0	0	0	0	0
0	0	0	0	0	0	0	0	0	0	0	0	0	0	0	0	0	0	0
0	0	0	0	0	0	0	0	0	0	0	0	0	0	0	0	0	0	0
0	0	0	0	0	0	0	0	0	0	0	0	0	0	0	0	0	0	0
0	0	0	0	0	0	0	0	0	0	0	0	0	0	0	0	0	0	0
0	0	0	0	0	0	0	0	0	0	0	0	0	0	0	0	0	0	0
0	0	0	0	0	0	0	0	0	0	0	0	0	0	0	0	0	0	0
0	0	0	0	0	0	0	0	0	0	0	4	4	4	0	0	0	0	0
0	0	0	0	0	0	0	0	0	0	0	0	0	0	0	0	0	0	0
0	0	0	0	0	0	0	0	0	0	0	0	0	0	0	0	0	0	0
0	0	0	0	0	0	0	0	0	0	0	0	0	0	0	0	0	0	0
0	0	0	0	0	0	0	0	0	0	0	0	0	0	0	0	0	0	0
0	0	0	0	0	0	0	0	0	0	0	0	0	0	0	0	0	0	0
0	0	0	0	0	0	0	0	0	0	0	0	0	0	0	0	0	0	0
0	0	0	0	0	0	0	0	0	0	0	0	0	0	0	0	0	0	0
0	0	0	0	0	0	0	0	0	0	0	0	0	0	0	0	0	0	0
0	0	0	0	0	0	0	0	0	0	0	0	0	0	0	0	0	0	0
0	0	0	0	0	0	0	0	0	0	0	0	0	0	0	0	0	0	0

0	0	0	0	0	0	0	0	0	0	0	0	0	0	0	0	0	0	0
0	0	0	0	0	0	0	0	0	0	0	0	0	0	0	0	0	0	0
0	0	0	0	0	0	0	0	0	0	0	0	0	0	0	0	0	0	0
0	0	0	0	0	0	0	0	0	0	0	0	0	0	0	0	0	0	0
0	0	0	0	0	0	0	0	0	0	0	0	0	0	0	0	0	0	0
0	0	0	0	0	0	0	0	0	0	0	0	0	0	0	0	0	0	0
0	0	0	0	0	0	0	0	0	0	0	0	0	0	0	0	0	0	0
0	0	0	0	0	0	3	3	3	0	0	0	0	0	0	0	0	0	0
0	0	0	0	0	0	0	0	0	0	0	0	0	0	0	0	0	0	0
0	0	0	0	0	0	0	0	0	0	0	0	0	0	0	0	0	0	0
0	0	0	0	0	0	0	0	0	0	0	0	0	0	0	0	0	0	0
0	0	0	0	0	0	0	0	0	0	0	0	0	0	0	0	0	0	0
0	0	0	0	0	0	0	0	0	0	0	0	0	0	0	0	0	0	0
0	0	0	0	0	0	0	0	0	0	0	0	0	0	0	0	0	0	0
0	0	0	0	0	0	0	0	0	0	0	0	0	0	0	0	0	0	0
0	0	0	0	0	0	0	0	0	0	0	0	0	0	0	0	0	0	0
0	0	0	0	0	0	0	0	0	0	0	0	0	0	0	0	0	0	0
0	0	0	0	0	0	0	0	0	0	0	0	0	0	0	0	0	0	0
0	0	0	0	0	0	0	0	0	0	0	0	0	0	0	0	0	0	0
0	0	0	0	0	0	0	0	0	0	0	0	0	0	0	0	0	0	0
0	0	0	0	0	0	0	0	0	0	0	0	0	0	0	0	0	0	0
0	0	0	0	0	0	0	0	0	0	0	0	0	0	0	0	0	0	0
0	0	0	0	0	0	0	0	0	0	0	0	0	0	0	0	0	0	0
0	0	0	0	0	0	0	0	0	0	0	0	0	0	0	0	0	0	0
0	0	0	0	0	0	0	0	0	0	0	0	0	0	0	0	0	0	0
0	0	0	0	0	0	0	0	0	0	0	0	0	0	0	0	0	0	0
0	0	0	0	0	0	0	0	0	0	0	0	0	0	0	0	0	0	0
0	0	0	0	0	0	0	0	0	0	0	0	0	0	0	0	0	0	0
0	0	0	0	0	0	0	0	0	0	0	0	0	0	0	0	0	0	0
0	0	0	0	0	0	0	0	0	0	0	0	0	0	0	0	0	0	0
0	0	0	0	0	0	0	0	0	0	0	0	0	0	0	0	0	0	0
0	0	0	0	0	0	0	0	0	0	0	0	0	0	0	0	0	0	0
0	0	0	0	0	0	0	0	0	0	0	0	0	0	0	0	0	0	0
0	0	2	2	2	2	2	2	0	0	0	0	0	0	0	0	0	0	0
0	0	0	0	0	0	0	0	0	0	0	0	0	0	0	0	0	0	0
0	0	0	0	0	0	0	0	0	0	0	0	0	0	0	0	0	0	0
0	0	0	0	0	0	0	0	0	0	0	0	0	0	0	0	0	0	0

0	0	0	0	0	0	0	0	0	0	0	0	0	0	0	0	0	0	0
0	0	0	0	0	0	0	0	0	0	0	0	0	0	0	0	0	0	0
0	0	0	0	0	0	0	0	0	0	0	0	0	0	0	0	0	0	0
0	0	0	0	0	0	0	0	0	0	0	0	0	0	0	0	0	0	0
0	0	0	0	0	0	0	0	0	0	0	0	0	0	0	0	0	0	0
0	0	0	0	0	0	0	0	0	0	0	0	0	0	0	0	0	0	0
0	0	0	0	0	0	0	0	0	0	0	0	0	0	0	0	0	0	0
0	0	0	0	0	0	0	0	0	0	0	0	0	0	0	0	0	0	0
0	0	0	0	0	0	0	0	0	0	0	0	0	0	0	0	0	0	0
0	0	0	0	0	0	0	0	0	0									
Jan_DL021	1643	2553	444	280	403	137	25	20	277	230	96	268	166	271	486	757	247	129
200	0	178	0	134	130	220	91	207	71	182	106	146	119	74	0	46	0	135
0	0	124	33	93	34	0	241	81	307	89	55	0	85	70	45	381	0	0
50	0	43	74	53	0	42	62	105	133	157	0	88	0	0	145	158	82	0
108	56	23	38	574	104	51	33	142	37	0	52	29	59	0	174	27	63	0
0	30	0	0	0	125	0	0	0	0	52	48	65	0	0	21	100	0	0
0	49	0	9	60	15	0	0	0	0	50	0	56	14	0	0	15	62	0
0	0	49	53	0	0	30	0	0	75	21	0	32	54	0	54	0	0	0
0	24	0	53	0	21	88	0	0	0	14	0	33	0	0	0	24	0	0
0	57	40	39	17	0	0	15	0	18	24	51	0	0	66	0	0	18	0
0	43	0	0	0	0	105	22	82	14	29	0	42	75	19	0	0	0	0
16	0	0	0	15	0	0	0	0	0	0	0	0	0	0	0	0	0	0
0	0	0	0	0	0	0	0	0	0	0	0	0	5	0	30	0	38	0
12	0	0	0	0	0	0	5	0	0	88	0	0	11	0	0	0	0	29
0	0	0	0	0	3	0	0	0	0	0	0	0	18	20	5	49	81	0
0	0	0	0	0	0	0	11	15	107	0	0	11	0	23	0	26	0	0
0	0	0	0	0	0	0	0	0	0	0	0	0	7	0	29	0	3	3
22	0	0	0	0	0	13	0	3	0	0	0	38	0	0	0	0	0	10
0	0	0	0	0	11	0	0	0	12	0	0	0	0	0	0	0	0	0
0	5	16	0	0	0	11	0	0	0	0	0	0	0	0	28	68	0	10
0	0	0	0	0	10	9	9	16	0	0	0	20	0	0	0	0	7	0
0	0	0	0	0	0	9	0	0	0	49	17	0	48	0	0	10	0	0
0	0	0	0	0	0	40	0	12	0	0	0	16	0	0	0	0	0	0
0	15	0	0	0	0	23	0	0	0	0	0	0	0	0	0	0	7	22
0	0	0	0	0	0	0	0	0	0	0	0	0	0	0	0	0	0	0
0	0	0	0	9	82	0	7	0	0	0	0	0	15	0	0	0	0	0
0	0	0	0	0	0	0	0	36	0	0	0	0	0	0	0	0	7	0

0	0	0	0	0	0	0	0	1	8	0	0	0	0	0	5	0	0	0
0	0	0	0	0	0	0	0	0	0	0	0	0	0	0	0	0	0	0
14	0	0	0	0	0	0	0	0	0	0	0	0	0	0	0	0	0	0
28	12	0	0	0	0	0	0	0	0	0	0	0	0	7	0	0	0	0
0	0	0	0	0	0	0	0	0	0	0	0	0	0	0	0	0	0	0
0	0	0	0	0	0	36	0	0	0	0	0	0	0	0	0	0	0	0
0	0	0	0	0	0	10	0	0	0	0	0	0	0	0	0	0	0	0
0	0	0	0	0	0	0	0	0	0	0	0	0	0	0	0	0	0	0
0	0	0	0	0	0	0	0	0	0	0	0	0	0	0	0	0	0	0
0	0	0	0	0	29	5	0	0	0	0	0	0	0	0	0	0	0	0
0	0	0	0	0	0	0	0	0	0	0	0	0	0	0	0	0	0	12
0	0	0	0	0	0	0	0	0	0	0	0	0	0	0	0	0	0	13
0	0	0	9	0	0	10	0	0	0	0	0	0	0	0	0	0	0	0
0	0	0	11	0	0	0	0	0	0	0	0	0	0	0	0	0	0	0
0	10	0	0	0	6	0	24	0	0	0	0	0	0	0	0	0	0	0
0	0	0	11	0	0	0	0	0	0	0	0	0	0	0	0	14	0	13
0	0	0	0	0	0	0	0	0	0	0	0	0	9	27	0	0	0	0
0	0	0	0	0	0	0	0	0	0	0	0	0	0	0	0	0	0	0
0	0	0	0	0	0	12	0	0	0	0	0	15	0	0	0	0	0	0
6	0	0	0	0	0	0	0	0	0	0	0	0	0	0	0	0	0	0
0	0	0	0	0	0	0	0	0	0	0	0	0	43	0	0	0	0	0
0	0	0	0	0	0	0	0	0	0	0	0	0	0	0	0	13	0	0
0	0	0	0	0	0	0	11	0	0	0	0	0	0	0	0	0	0	0
0	0	0	0	0	0	0	0	0	0	0	0	0	0	0	0	0	0	0
0	0	0	0	0	0	0	0	0	0	0	0	0	0	0	0	0	0	0
0	0	0	0	0	0	0	0	0	0	0	0	0	0	0	0	0	0	0
0	0	0	0	0	0	0	0	0	0	0	0	0	0	0	0	0	0	0
0	0	0	0	0	0	0	0	0	0	0	0	0	0	0	0	0	0	0
0	0	0	0	0	0	0	0	0	0	0	0	0	0	0	0	0	0	0
0	0	0	0	0	0	0	0	0	0	0	0	0	0	0	0	0	0	0
0	0	0	0	0	0	0	0	0	0	0	0	0	0	0	0	0	0	27
0	0	0	0	0	0	0	0	0	0	0	0	0	0	0	0	0	0	0
0	0	0	0	0	0	0	0	0	0	0	0	0	0	0	0	0	0	0
0	0	0	0	0	0	0	0	0	0	0	0	0	0	0	0	0	0	0
0	0	0	0	0	0	0	0	0	0	0	0	0	0	0	0	0	0	0
0	0	0	0	0	0	0	0	0	0	0	0	0	0	0	0	0	0	0
0	0	0	0	0	0	0	0	0	0	0	0	0	0	0	0	0	0	0

0	0	0	0	0	0	0	0	0	0	0	0	0	0	0	0	0	0	0
15	0	0	0	0	0	0	0	0	0	0	0	0	0	0	0	0	0	0
0	0	0	0	0	0	0	0	0	0	0	0	0	0	0	0	0	0	0
0	0	0	0	0	0	0	0	0	0	0	0	0	0	0	0	0	0	0
0	0	0	0	0	0	0	0	0	0	0	0	0	0	0	0	0	0	0
0	0	0	0	0	0	0	0	0	0	0	0	4	0	0	0	0	0	0
0	0	0	0	0	0	0	0	0	0	0	0	0	0	0	0	0	0	0
0	0	0	0	0	0	0	0	0	0	0	0	0	0	0	0	0	0	0
0	0	0	0	0	0	0	0	0	0	0	0	0	0	0	0	0	0	0
0	0	0	0	0	0	0	0	0	0	0	0	0	0	0	0	0	0	0
0	0	0	0	0	0	0	0	0	0	0	0	0	0	0	0	0	0	0
0	0	0	0	0	0	0	0	0	0	0	0	0	0	0	0	0	0	0
4	0	0	0	0	0	0	0	0	0	0	0	0	0	0	0	0	0	0
0	0	0	0	0	0	0	0	0	0	0	0	0	0	0	0	0	0	0
0	0	0	0	0	0	0	0	0	0	0	0	0	0	0	0	0	0	0
0	0	0	0	0	0	0	0	0	0	0	0	0	0	0	0	0	0	0
0	0	0	0	0	0	0	0	0	0	0	0	0	0	0	0	0	0	0
0	0	0	0	0	0	0	0	0	0	10	0	0	0	0	0	0	0	0
0	0	0	0	0	0	0	0	0	0	0	0	0	0	0	0	0	0	0
0	0	0	0	0	0	0	0	0	0	0	0	0	0	0	0	0	0	0
0	0	0	0	0	0	0	0	0	0	0	0	0	0	0	0	0	0	0
0	0	0	0	0	0	0	0	0	0	0	0	0	0	0	0	0	0	0
0	0	0	0	0	0	0	0	0	0	3	0	0	0	0	0	0	0	0
0	0	0	0	0	0	0	0	0	0	0	0	0	0	0	0	0	0	0
0	0	0	0	0	0	0	0	0	0	0	0	0	0	0	0	0	0	0
0	0	0	0	0	0	0	0	0	0	0	0	0	0	0	0	0	0	0
0	0	0	0	0	0	0	0	0	0	0	0	0	0	0	0	0	0	0
0	0	0	0	0	0	0	0	0	0	0	0	0	0	0	0	0	0	0
0	0	0	0	0	0	0	0	0	0	0	0	0	0	0	0	0	0	0
0	0	0	0	0	0	0	0	0	0	0	0	0	0	0	0	0	0	0
0	0	0	0	0	0	0	0	0	0	0	0	0	0	0	0	0	0	0
0	0	0	0	0	0	0	0	0	0	0	0	0	0	0	0	0	0	7
0	0	0	0	0	0	0	0	0	0	0	0	0	0	0	0	0	0	0
0	0	0	0	0	0	0	0	0	0	0	0	0	0	0	0	0	0	0
0	0	0	0	0	0	0	0	0	0	0	0	0	0	0	0	0	0	0
0	0	0	0	0	0	0	0	0	0	0	0	0	0	0	0	0	0	0
0	0	0	0	0	0	0	0	0	0	0	0	0	0	0	0	0	0	0

0	0	0	0	0	0	0	0	0	0	0	0	0	0	0	0	0	0	0
0	0	0	0	0	0	0	0	0	0	0	0	0	0	0	0	0	0	0
0	0	0	0	0	0	0	0	0	0	0	0	0	0	6	6	0	0	0
0	0	0	0	0	0	0	0	0	0	0	0	0	0	0	0	0	0	0
0	0	0	0	0	0	0	0	0	0	0	0	0	0	0	0	0	0	0
0	0	0	0	0	0	0	0	0	0	0	0	0	0	0	0	0	0	0
0	0	0	0	0	0	0	0	0	0	0	0	0	0	0	0	0	0	0
0	0	0	0	0	0	0	0	0	0	0	0	0	0	0	0	0	0	0
0	0	0	0	0	0	0	0	0	0	0	0	0	0	0	0	0	0	0
0	0	0	0	0	0	0	0	0	0	0	0	0	0	0	0	0	0	0
0	0	5	0	0	0	0	0	0	0	0	0	0	0	0	0	0	0	0
0	0	0	0	0	0	0	0	0	0	0	0	0	0	0	0	0	0	0
0	0	0	0	0	0	0	0	0	0	0	0	0	0	0	0	0	0	0
0	0	0	0	0	0	0	0	0	0	0	0	0	0	0	0	0	0	0
0	0	0	0	0	0	0	0	0	0	0	0	0	0	0	0	0	0	0
0	0	0	0	0	0	0	0	0	0	0	0	0	0	0	0	0	0	0
0	0	0	0	0	0	0	0	0	0	0	0	0	0	0	0	0	0	0
0	0	0	0	0	0	0	0	0	0	0	0	0	0	0	0	0	0	0
0	0	0	0	0	0	0	0	0	0	0	0	0	0	0	0	0	0	0
0	0	0	0	0	0	0	0	0	0	0	0	0	0	0	0	0	0	0
0	0	0	0	0	0	0	0	0	0	0	0	0	0	0	0	0	0	0
0	0	0	0	0	0	0	0	0	0	0	0	0	0	0	0	0	0	0
0	0	0	0	0	0	0	0	0	0	0	0	0	0	0	0	0	0	0
0	0	0	0	4	4	0	0	0	0	0	0	0	0	0	0	0	0	0
0	0	0	0	0	0	0	0	0	0	0	0	0	0	0	0	0	0	0
0	0	0	0	0	0	0	0	0	0	0	0	0	0	0	0	0	0	0
0	0	0	0	0	0	0	0	0	0	0	0	0	0	0	0	0	0	0
0	0	0	0	0	0	0	0	0	0	0	0	0	0	0	0	0	0	0
0	0	0	0	0	0	0	0	0	0	0	0	0	0	0	0	0	0	0
0	0	0	0	0	0	0	0	0	0	0	0	0	0	0	0	0	0	0
0	0	0	0	0	0	0	0	0	0	0	0	0	0	0	0	0	0	0
0	0	0	0	0	0	0	0	0	0	0	0	0	0	0	0	0	0	0
0	0	0	0	0	0	0	0	0	0	0	0	0	0	0	0	0	0	0
0	0	0	0	0	0	0	0	0	0	0	0	0	0	0	0	0	0	0
0	0	0	0	0	0	0	0	0	0	0	0	0	0	0	0	0	0	0
0	0	0	0	0	0	0	0	0	0	0	0	0	0	0	0	0	0	0
0	0	0	0	0	0	0	0	0	0	0	0	0	0	0	0	0	0	0

0	0	0	0	0	0	0	0	0	0	0	0	0	0	0	0	0	0	0
0	0	0	0	0	0	0	0	0	0	0	0	0	0	0	0	0	0	0
0	0	0	0	0	0	0	0	0	0	0	0	0	0	0	0	0	0	0
0	0	0	0	0	0	0	0	0	0	0	0	3	3	3	0	0	0	0
0	0	0	0	0	0	0	0	0	0	0	0	0	0	0	0	0	0	0
0	0	0	0	0	0	0	0	0	0	0	0	0	0	0	0	0	0	0
0	0	0	0	0	0	0	0	0	0	0	0	0	0	0	0	0	0	0
0	0	0	0	0	0	0	0	0	0	0	0	0	0	0	0	0	0	0
0	0	0	0	0	0	0	0	0	0	0	0	0	0	0	0	0	0	0
0	0	0	0	0	0	0	0	0	0	0	0	0	0	0	0	0	0	0
0	0	0	0	0	0	0	0	0	0	0	0	0	0	0	0	0	0	0
0	0	0	0	0	0	0	0	0	0	0	0	0	0	0	0	0	0	0
0	0	0	0	0	0	0	0	0	0	0	0	0	0	0	0	0	0	0
0	0	0	0	0	0	0	0	0	0	0	0	0	0	0	0	0	0	0
0	0	0	0	0	0	0	0	0	0	0	0	0	0	0	0	0	0	0
0	0	0	0	0	0	0	0	0	0	0	0	0	0	0	0	0	0	0
0	0	0	0	0	0	0	0	0	0	0	0	0	0	0	0	0	0	0
0	0	0	0	0	0	0	0	0	0	0	0	0	0	0	0	0	0	0
0	0	0	0	0	0	0	0	0	0	0	0	0	0	0	0	0	0	0
0	0	0	0	0	0	0	0	0	0	0	0	0	0	0	0	0	0	0
0	0	0	0	0	0	0	0	0	0	0	0	0	0	0	0	0	0	0
0	0	0	0	0	0	0	0	0	0	0	0	0	0	0	0	0	0	0
0	0	0	0	0	0	0	0	0	0	0	0	0	0	0	0	0	0	0
0	0	0	0	0	0	0	0	0	0	0	0	0	0	0	0	0	0	0
0	0	0	0	0	0	0	0	0	0	0	0	0	0	0	0	0	0	0
0	0	0	0	0	0	0	0	0	0	0	0	0	0	0	0	0	0	0
0	0	0	0	0	0	0	0	0	0	0	0	0	0	0	0	0	0	0
0	0	0	0	0	0	0	0	0	0	0	0	0	0	0	0	0	0	0
0	0	0	0	0	0	0	0	0	0	0	0	2	2	2	2	2	2	2
0	0	0	0	0	0	0	0	0	0	0	0	0	0	0	0	0	0	0
0	0	0	0	0	0	0	0	0	0	0	0	0	0	0	0	0	0	0
0	0	0	0	0	0	0	0	0	0	0	0	0	0	0	0	0	0	0
0	0	0	0	0	0	0	0	0	0	0	0	0	0	0	0	0	0	0
0	0	0	0	0	0	0	0	0	0	0	0	0	0	0	0	0	0	0
0	0	0	0	0	0	0	0	0	0	0	0	0	0	0	0	0	0	0
0	0	0	0	0	0	0	0	0	0	0	0	0	0	0	0	0	0	0
0	0	0	0	0	0	0	0	0	0	0	0	0	0	0	0	0	0	0

0	0	0	0	0	0	0	0	0	0	0	0	0	0	0	0	0	0	0
0	0	0	0	0	0	0	0	0	0									
Jan_DL022	801	1468	1394	342	629	53	46	44	51	253	78	291	225	14	851	464	16	80
28	487	286	28	49	215	194	117	224	74	32	0	66	107	87	0	91	140	0
75	295	18	9	55	0	0	17	17	178	38	82	387	0	12	73	0	46	0
0	169	82	198	43	587	0	36	103	63	70	0	510	0	393	11	37	49	0
27	53	0	30	0	18	31	15	203	59	0	0	17	0	0	0	16	8	0
22	37	0	189	59	0	0	105	0	12	0	0	0	0	11	19	0	0	77
0	56	7	48	33	0	0	0	0	0	22	17	0	0	89	0	0	0	109
0	32	0	0	25	38	0	21	0	4	0	0	3	0	0	19	0	7	0
0	12	80	91	0	11	0	0	128	0	9	0	32	8	48	0	0	0	31
12	0	0	0	0	0	0	0	0	19	0	11	0	0	0	0	50	0	0
0	0	10	0	0	0	0	0	0	10	0	0	0	0	0	0	0	0	0
21	0	0	0	15	0	0	0	0	0	88	0	0	0	0	0	0	0	37
0	0	57	0	0	11	0	0	25	0	0	29	34	16	26	16	18	0	0
0	0	0	5	0	0	0	0	0	9	0	59	12	11	11	12	0	0	0
3	0	0	14	0	18	78	0	0	0	0	0	0	0	0	11	0	0	0
0	11	7	0	0	0	0	19	4	0	44	18	24	0	0	20	0	0	0
0	20	0	0	0	0	0	0	0	0	0	0	0	19	0	15	34	0	11
0	0	0	18	0	0	0	0	0	13	0	0	43	0	0	0	53	0	0
8	0	0	86	7	0	0	0	7	0	0	0	0	0	0	0	0	0	8
0	0	0	0	0	0	0	8	0	0	0	0	15	0	0	38	0	0	0
0	123	0	0	12	8	0	0	0	0	0	10	11	0	56	0	0	0	0
0	0	0	0	43	28	0	13	0	0	22	12	0	0	0	0	0	0	0
0	0	0	0	0	36	0	0	0	0	0	0	0	0	0	0	0	0	0
0	0	0	0	64	0	0	0	0	0	0	0	0	0	0	0	0	7	0
0	0	0	0	0	0	0	0	0	0	0	0	0	0	0	12	0	0	0
0	0	0	0	0	0	0	0	0	16	0	0	0	0	0	0	0	0	5
0	0	0	0	0	0	0	0	0	0	0	0	0	0	0	0	0	0	0
0	0	0	0	0	0	0	6	0	0	0	0	0	0	0	0	0	0	0
0	0	0	0	0	0	0	0	0	0	0	0	0	0	0	0	0	0	0
0	0	9	0	0	0	0	0	0	0	12	0	11	0	0	0	0	0	0
0	0	0	0	0	0	0	0	0	0	8	0	0	0	0	0	0	0	0
0	0	0	0	0	0	0	0	0	0	0	0	0	0	0	0	0	0	0
0	0	0	0	0	0	0	0	0	0	23	0	0	0	0	0	0	0	0
0	0	0	0	0	0	0	0	19	49	0	0	0	0	0	0	0	0	0
0	0	0	0	16	0	0	0	0	0	0	0	0	0	0	6	0	8	0

0	0	0	0	0	0	0	0	0	0	0	0	0	0	0	0	0	0	0
0	0	0	0	14	0	0	0	0	0	0	0	0	0	0	0	0	0	0
0	0	0	0	0	0	0	0	0	0	0	0	0	0	0	0	18	0	0
0	0	0	0	0	0	0	0	0	0	0	0	0	0	0	0	0	0	0
0	0	0	0	0	0	0	0	0	0	0	0	0	0	0	0	0	0	0
0	0	0	0	0	0	0	3	30	0	0	0	0	0	6	0	0	0	0
0	0	0	0	0	0	0	0	0	0	0	9	0	0	0	0	0	0	0
0	0	0	0	23	0	0	0	0	0	13	0	0	13	0	0	0	0	0
0	0	0	0	0	0	0	0	0	32	0	0	0	0	0	0	0	0	0
0	0	0	0	0	0	14	0	0	0	0	0	0	0	0	0	0	57	0
0	0	0	0	0	0	0	0	0	0	0	0	0	0	0	0	0	0	0
0	0	0	0	0	0	0	0	0	0	0	0	0	0	0	0	0	0	0
0	0	0	0	0	0	0	5	0	0	0	0	0	0	0	0	0	0	0
0	0	0	0	0	0	0	0	0	0	0	0	0	0	0	0	0	0	0
0	0	0	0	0	0	0	0	0	0	41	0	23	0	0	0	0	0	0
0	0	0	0	0	0	0	0	0	0	0	0	0	0	0	0	0	0	0
0	0	0	0	0	0	0	0	0	0	0	0	0	0	0	0	0	0	0
0	0	0	0	15	0	24	0	0	0	0	0	0	0	0	0	0	0	0
0	0	0	0	0	0	0	0	0	0	0	0	0	0	0	0	0	0	0
0	0	0	0	0	0	0	0	0	0	0	0	0	0	0	0	0	0	0
0	0	0	0	0	18	0	0	0	0	0	0	0	0	0	0	2	0	0
0	0	0	0	0	0	0	0	0	0	0	0	0	0	0	0	0	0	0
0	0	0	0	0	0	0	0	0	0	0	0	0	0	0	0	6	0	0
0	0	0	0	0	0	0	0	0	0	0	0	0	0	0	0	0	0	0
0	0	0	4	0	0	0	0	0	0	0	0	0	7	0	0	0	0	0
0	0	0	0	0	0	0	0	0	0	0	0	0	0	0	0	0	0	0
0	0	0	0	0	0	0	0	0	0	0	0	0	0	0	0	0	0	0
0	0	0	0	0	0	0	0	0	0	0	0	0	0	0	0	0	0	0
0	0	0	0	0	0	0	0	0	0	0	0	0	0	0	0	0	0	0
0	0	0	0	0	0	0	0	0	0	0	0	0	0	0	0	0	0	0
0	0	0	0	0	0	0	0	0	0	0	0	0	0	0	0	2	0	0
0	0	0	0	0	0	0	0	0	0	0	0	0	0	0	0	0	0	0
0	0	0	0	0	0	0	0	0	0	0	0	0	0	0	0	0	0	0
0	0	0	0	0	0	0	0	0	0	0	0	0	0	0	0	0	0	0
0	0	0	0	0	0	0	0	0	0	0	0	0	0	0	0	0	0	0
0	0	0	0	0	0	0	0	0	0	0	0	0	0	0	0	0	0	0
0	0	0	0	0	0	0	0	0	0	0	0	0	0	0	0	0	0	0

0	13	0	0	0	0	0	0	0	0	0	0	0	0	0	0	0	0	0
0	0	0	0	0	6	0	0	0	0	0	0	0	0	0	0	0	0	0
0	0	0	0	0	0	0	0	0	0	0	0	0	0	0	0	0	0	0
0	0	0	0	0	0	0	0	0	0	0	0	0	0	0	0	0	0	0
0	0	0	0	0	0	0	0	0	0	0	0	0	0	0	0	0	0	0
0	0	0	0	0	0	0	0	0	0	0	0	0	0	0	0	0	0	0
0	0	0	0	0	0	0	0	0	0	0	0	0	0	0	0	0	10	0
0	0	0	0	0	0	0	0	0	0	0	0	0	0	0	0	0	0	0
0	0	0	0	0	0	0	0	0	0	0	0	0	0	0	0	0	0	0
0	0	0	0	0	0	0	0	0	0	0	0	0	0	0	0	0	0	0
0	0	0	0	0	0	0	0	0	0	0	0	0	0	0	0	0	0	0
0	0	0	0	0	0	0	0	0	0	0	0	0	0	0	0	0	0	0
0	0	0	0	0	0	0	0	0	0	0	0	0	0	0	0	0	0	0
0	0	0	0	0	0	0	0	0	0	0	0	0	0	0	0	0	0	0
0	0	0	0	0	0	0	0	0	0	0	8	0	0	0	0	0	0	0
0	0	0	0	0	0	0	0	0	0	0	0	0	0	0	0	0	0	0
0	0	0	0	0	0	0	0	0	0	0	0	0	0	0	0	0	0	0
0	0	0	0	0	0	0	0	0	0	0	0	0	0	0	0	0	0	0
0	0	0	0	0	0	0	0	0	0	0	0	0	0	0	0	0	0	0
0	0	0	0	0	0	0	0	0	0	0	0	0	0	0	0	0	0	0
0	0	0	7	0	0	0	0	0	0	0	0	0	0	0	0	0	0	0
0	0	0	0	0	0	0	0	0	0	0	0	0	0	0	0	0	0	0
0	0	0	0	0	0	0	0	0	0	0	0	0	0	0	0	0	0	0
0	0	0	0	0	0	0	0	0	0	0	0	0	0	0	0	0	0	0
0	0	0	0	0	0	0	0	0	0	0	0	0	0	0	0	0	0	0
0	0	0	0	0	0	0	0	0	0	0	0	0	0	0	0	0	0	0
0	0	0	0	0	0	0	0	0	0	0	0	0	0	0	0	0	0	0
0	0	0	0	0	0	0	0	0	0	0	0	0	0	0	0	0	0	0
0	0	0	0	0	0	0	0	0	0	0	0	0	0	0	0	0	0	0
0	0	0	0	0	0	0	0	0	0	0	0	0	0	0	0	0	0	0
0	0	0	0	0	0	0	0	0	0	0	0	0	0	0	0	0	0	0
0	0	0	0	0	0	0	0	0	0	0	0	0	0	0	0	0	0	0
0	0	0	0	0	0	0	0	0	0	0	0	0	0	0	0	0	0	0
0	0	0	0	0	0	0	0	0	0	0	0	0	0	0	0	0	0	0
0	0	0	0	0	0	0	0	0	0	0	0	0	0	0	0	0	0	0
0	0	0	0	0	0	0	0	0	0	0	0	0	0	0	0	0	0	0
0	0	0	0	0	0	0	0	0	0	0	0	0	0	0	0	0	0	0

0	0	0	0	0	0	0	0	0	0	0	0	0	0	0	0	0	0	0
0	0	0	0	0	0	0	0	0	0	0	0	0	0	0	0	0	0	0
0	0	0	0	0	0	0	0	0	0	0	0	0	0	0	0	0	0	0
0	0	0	0	0	0	0	0	0	0	0	0	0	0	0	0	0	0	0
0	0	0	0	0	0	0	0	0	0	0	0	0	0	0	0	0	0	0
0	0	0	0	0	0	0	0	0	0	0	0	0	0	0	0	0	0	0
0	0	0	0	0	0	0	0	0	0	0	0	0	0	0	0	0	0	0
0	0	0	0	0	0	0	0	0	0	0	0	0	0	0	0	0	0	0
0	0	0	0	0	0	0	0	0	0	0	0	0	0	0	0	0	0	0
0	0	0	0	0	0	0	0	0	0	0	0	0	0	0	0	0	0	0
0	0	0	0	0	0	0	0	0	0	0	0	0	0	0	0	0	0	0
0	0	0	0	0	0	0	0	0	0	0	0	0	0	0	0	0	0	0
0	0	0	0	0	0	0	0	0	0	0	0	0	0	0	0	0	0	0
0	0	0	0	0	0	0	0	0	0	0	0	0	0	0	0	0	0	0
0	0	0	0	0	0	0	0	0	0	0	0	0	0	0	0	0	0	0
0	0	0	0	0	0	0	0	0	0	0	0	0	0	0	0	0	0	0
0	0	0	0	0	0	0	0	0	0	0	0	0	0	0	0	0	0	0
0	0	0	0	0	0	0	0	0	0	0	0	0	0	0	0	0	0	0
0	0	0	0	0	0	0	0	0	0	0	0	0	0	0	0	0	0	0
0	0	0	0	0	0	0	0	0	0	0	0	0	0	0	0	0	0	0
0	0	0	0	0	0	0	0	0	0	0	0	0	0	0	0	0	0	0
0	0	0	0	0	0	0	0	0	0	0	0	0	0	0	0	0	0	0
0	0	0	0	0	0	0	0	0	0	0	0	0	0	0	0	0	0	0
0	0	0	0	0	0	0	0	0	0	0	0	0	0	0	0	0	0	0
0	0	0	0	0	0	0	0	0	0	0	3	0	0	0	0	0	0	0
0	0	0	0	0	0	0	0	0	0	0	0	0	0	0	0	0	0	0
0	0	0	0	0	0	0	0	0	0	0	0	0	0	0	0	0	0	0
0	0	0	0	0	0	0	0	0	0	0	0	0	0	0	0	0	0	0
0	0	0	0	0	0	0	0	0	0	0	0	0	0	0	0	0	0	0
0	0	0	0	0	0	0	0	0	0	0	0	0	0	0	0	0	0	0
0	0	0	0	0	0	0	0	0	0	0	0	0	0	0	0	0	0	0
0	0	0	0	0	0	0	0	0	0	0	0	0	0	0	0	0	0	0
0	0	0	0	0	0	0	0	0	0	0	0	0	0	0	0	0	0	0
0	0	0	0	0	0	0	0	0	0	0	0	0	0	0	0	0	0	0
0	0	0	0	0	0	0	0	0	0	0	0	0	0	0	0	0	0	0
0	0	0	0	0	0	0	0	0	0	0	0	0	0	0	0	0	0	0
0	0	0	0	0	0	0	0	0	0	0	0	0	0	0	0	0	0	0

0	0	0	0	0	0	0	0	0	0	0	0	0	0	0	0	0	0	0
0	0	0	0	0	0	0	0	0	0	0	0	0	0	0	0	0	0	0
0	0	0	0	0	0	0	0	0	0	0	0	0	0	0	0	0	0	0
0	0	0	0	0	0	0	0	0	0	0	0	0	0	0	0	0	0	0
0	0	0	0	0	0	0	0	0	0	0	0	0	0	0	0	0	0	0
0	0	0	0	0	0	0	0	0	0	0	0	0	0	0	0	0	0	0
0	0	0	0	0	0	0	0	0	0	0	0	0	0	0	0	0	0	0
0	0	0	0	0	0	0	0	0	0	0	0	0	0	0	0	0	0	0
0	0	0	0	0	0	0	0	0	0	0	0	0	0	0	0	0	0	0
0	0	0	0	0	0	0	0	0	0	0	0	0	0	0	0	0	0	0
0	0	0	0	0	0	0	0	0	0	0	0	0	0	0	0	0	0	0
0	0	0	0	0	0	0	0	0	0	0	0	0	0	0	0	0	0	0
0	0	0	0	0	0	0	0	0	0	0	0	0	0	0	0	0	0	0
0	0	0	0	0	0	0	0	0	0	0	0	0	0	0	0	0	0	0
0	0	0	0	0	0	0	0	0	0	0	0	0	0	0	0	0	0	0
0	0	0	0	0	0	0	0	0	0	2	2	0	0	0	0	0	0	0
0	0	0	0	0	0	0	0	0	0	0	0	0	0	0	0	0	0	0
0	0	0	0	0	0	0	0	0	0	0	0	0	0	0	0	0	0	0
0	0	0	0	0	0	0	0	0	0	0	0	0	0	0	0	0	0	0
0	0	0	0	0	0	0	0	0	0	0	0	0	0	0	0	0	0	0
0	0	0	0	0	0	0	0	0	0	0	0	0	0	0	0	0	0	0
0	0	0	0	0	0	0	0	0	0	0	0	0	0	0	0	0	0	0
0	0	0	0	0	0	0	0	0	0	0	0	0	0	0	0	0	0	0
0	0	0	0	0	0	0	0	0	0	0	0	0	0	0	0	0	0	0
0	0	0	0	0	0	0	0	0	0	0	0	0	0	0	0	0	0	0
0	0	0	0	0	0	0	0	0	0	0	0	0	0	0	0	0	0	0
0	0	0	0	0	0	0	0	0	0	0	0	0	0	0	0	0	0	0
0	0	0	0	0	0	0	0	0	0	0	0	0	0	0	0	0	0	0
0	0	0	0	0	0	0	0	0	0	0	0	0	0	0	0	0	0	0
0	0	0	0	0	0	0	0	0	0	0	0	0	0	0	0	0	0	0
0	0	0	0	0	0	0	0	0	0									
Jan_DL023	2520	1587	1026	294	117	281	356	329	87	526	284	814	425	0	99	513	5	301
380	0	360	0	374	277	417	205	358	268	130	0	231	92	150	119	152	9	59
0	0	82	38	104	150	116	0	123	220	68	34	0	184	0	23	200	52	0
0	14	0	26	57	0	168	46	115	8	104	57	0	142	0	0	156	27	0
90	29	22	95	85	95	114	0	0	59	0	49	19	0	62	150	0	45	0
70	0	47	0	0	14	0	0	0	12	98	0	73	0	53	0	15	0	0

0	0	0	0	0	72	0	0	0	0	17	0	16	17	0	0	0	0	0
0	0	0	18	0	0	0	0	0	7	0	0	30	0	34	5	0	0	0
0	9	0	64	0	7	67	0	0	0	0	0	85	0	0	0	6	25	13
0	44	27	0	0	0	0	0	0	0	0	35	0	0	0	29	4	0	0
0	0	0	0	0	0	65	0	0	0	0	0	0	0	11	0	0	0	0
0	0	0	0	24	0	0	0	0	0	0	0	0	0	0	0	49	0	0
0	0	0	48	0	16	0	0	9	0	0	0	22	0	0	0	22	0	19
0	0	0	0	0	0	0	0	0	9	64	0	0	12	0	22	6	8	0
0	0	0	0	0	6	0	0	0	10	0	9	0	5	6	0	7	0	0
0	0	0	0	0	0	0	0	0	47	0	0	0	0	0	0	0	0	0
0	0	9	0	0	0	0	0	0	0	0	0	0	0	0	9	0	0	0
0	0	0	0	0	0	0	0	0	0	0	0	17	0	0	0	0	0	0
5	0	0	0	7	0	0	27	0	0	0	0	9	10	0	0	0	0	0
0	0	6	0	9	0	5	0	0	0	0	0	3	0	0	0	43	0	0
0	0	0	0	0	0	10	0	0	0	0	13	0	0	0	9	0	0	0
0	0	0	0	0	0	0	5	0	7	0	12	10	0	0	0	0	0	0
0	0	0	0	0	24	0	0	0	0	0	0	0	0	0	0	0	0	0
0	0	0	0	0	0	0	0	0	0	0	0	0	0	0	0	0	0	7
0	0	0	0	5	0	0	7	0	0	0	0	0	0	0	0	0	0	0
0	0	0	0	0	0	0	9	0	0	0	0	0	0	0	4	0	0	0
0	0	0	0	0	0	0	0	0	0	0	0	0	0	0	0	0	0	0
0	0	0	0	0	0	0	9	0	0	0	0	0	0	0	0	0	0	0
0	0	0	0	0	0	0	0	0	0	0	0	0	0	0	0	0	0	0
0	0	0	0	0	0	0	0	0	0	0	0	0	0	0	0	0	0	0
0	0	0	0	0	0	0	0	0	6	0	0	0	0	0	0	0	0	0
0	0	0	0	0	0	0	0	0	0	0	0	0	0	6	6	0	0	0
0	0	0	0	5	0	0	0	0	0	0	0	0	0	0	0	0	0	0
0	20	0	0	0	0	0	0	0	0	0	0	0	0	0	0	0	0	0
0	0	0	0	0	0	0	0	0	0	0	0	0	0	0	0	0	0	0
0	0	0	0	0	0	0	0	0	0	0	0	0	0	0	0	0	0	0
0	0	0	0	0	0	0	0	0	0	0	0	0	0	0	0	0	0	0
0	0	0	0	0	0	0	0	0	0	0	0	0	0	0	0	0	0	0
0	0	0	0	0	0	0	0	0	0	0	0	0	0	0	0	0	0	0
0	0	0	0	0	0	0	0	0	0	0	0	0	0	0	0	0	0	0
0	0	0	0	0	0	0	0	0	0	0	0	0	0	0	0	0	0	0
0	0	0	0	0	0	0	0	0	0	0	0	0	0	0	0	0	0	0
0	0	0	0	0	0	0	0	0	0	0	0	0	0	0	0	0	0	0

0	0	0	0	0	0	0	0	0	0	0	0	0	0	0	0	0	0	0
0	0	0	0	0	0	0	0	0	0	0	0	0	0	0	0	0	0	0
0	0	0	0	0	0	0	0	0	0	0	0	0	0	0	0	0	0	0
0	0	0	0	0	0	0	0	0	0	0	0	0	0	0	0	0	0	0
0	0	0	0	0	0	0	0	0	0	0	0	0	0	5	0	0	0	0
0	0	0	0	0	0	0	0	0	0	0	0	0	0	0	0	0	0	0
0	0	0	0	0	0	0	0	0	0	0	0	0	0	0	0	0	0	0
0	0	0	0	0	0	0	0	0	0	0	0	0	0	0	0	0	0	0
0	0	0	0	0	0	0	0	0	0	0	0	0	0	0	0	0	0	0
0	0	0	0	0	0	0	0	0	0	0	0	0	0	0	0	0	0	0
0	0	0	0	0	0	0	0	0	0	0	0	0	0	0	0	0	0	0
0	0	0	0	0	0	0	0	0	0	0	0	0	0	0	0	0	0	0
0	0	0	0	0	0	0	0	0	0	0	0	0	0	0	0	0	0	0
0	0	0	0	0	0	0	0	0	0	0	0	0	0	0	0	0	0	0
0	0	0	0	0	0	0	0	0	0	0	0	0	0	0	0	0	0	0
0	0	0	0	0	0	0	0	0	0	0	0	0	0	0	0	0	0	0
0	0	0	0	0	0	0	0	0	0	0	0	0	0	0	0	0	0	0
0	0	0	0	0	0	0	0	0	0	0	0	0	0	0	0	0	0	0
0	0	0	0	0	0	0	0	0	0	0	0	0	0	0	0	0	0	0
0	0	0	0	0	0	0	0	0	0	0	0	0	0	0	0	0	0	0
0	0	0	0	0	0	0	0	0	0	0	0	0	0	0	0	0	0	0
0	0	0	0	0	0	0	0	0	0	0	0	0	0	0	0	0	0	0
0	0	0	0	0	0	0	0	0	0	0	0	0	0	0	0	0	0	0
0	0	0	0	0	0	0	0	0	0	0	0	0	0	0	0	0	0	0
0	0	0	0	0	0	0	0	0	0	0	0	0	0	0	0	0	0	0
0	0	0	0	0	0	0	0	0	0	0	0	0	0	0	0	0	0	0
0	0	0	0	0	0	0	0	0	0	0	0	0	0	0	0	0	0	0
0	0	0	0	0	0	0	0	0	0	0	0	0	0	0	0	0	0	0
0	0	0	0	0	0	0	0	0	0	0	0	0	0	0	0	0	0	0
0	0	0	0	0	0	0	0	0	0	0	0	0	0	0	0	0	0	0
0	0	0	0	0	0	0	0	0	0	0	0	0	0	0	0	0	0	0
0	0	0	0	0	0	0	0	0	0	0	0	0	0	0	0	0	0	0
0	0	0	0	0	0	0	0	0	0	0	0	0	0	0	0	0	0	0
0	0	0	0	0	0	0	0	0	0	0	0	0	0	0	0	0	0	0
0	0	0	0	0	0	0	0	0	0	0	0	0	0	0	0	0	0	0
0	0	0	0	0	0	0	0	0	0	0	0	0	0	0	0	0	0	0
0	0	0	0	0	0	0	0	0	0	0	0	0	0	0	0	0	0	0

0	0	0	0	0	0	0	0	0	0	0	0	0	0	0	0	0	0	0
0	0	0	0	0	0	0	0	0	0	0	0	0	0	0	0	0	0	0
0	0	0	0	0	0	0	0	0	0	0	0	0	0	0	0	0	0	0
0	0	0	0	0	0	0	0	0	0	0	0	0	0	0	0	0	0	0
0	0	0	0	0	9	0	0	0	0	0	0	0	0	0	0	0	0	0
0	0	0	0	0	0	0	0	0	0	0	0	0	0	0	0	0	0	0
0	0	0	0	0	0	0	0	0	0	0	0	0	0	0	0	0	0	0
0	0	0	0	0	0	0	0	0	0	0	0	0	0	0	0	0	0	0
0	0	0	0	0	0	0	0	0	0	0	0	0	8	0	0	0	0	0
0	0	0	0	0	0	0	0	0	0	0	0	0	0	0	0	0	0	0
0	0	0	0	0	0	0	0	0	0	0	0	0	0	0	0	0	0	0
0	0	0	0	0	0	0	0	0	0	0	0	0	0	0	0	0	0	0
0	0	0	0	0	0	0	0	0	0	0	0	0	0	0	0	0	0	0
0	0	0	0	0	0	0	0	0	0	0	0	0	0	0	0	0	0	0
0	0	0	0	0	0	0	0	0	0	0	0	0	0	0	0	0	0	0
0	0	0	0	0	0	0	0	0	0	0	0	0	0	0	0	0	0	0
0	0	0	0	0	0	0	0	0	0	0	0	0	0	0	0	0	0	0
0	0	0	0	0	0	0	0	0	0	0	0	0	0	0	0	0	0	0
0	0	0	0	0	0	0	0	0	0	0	0	0	0	0	0	0	0	0
0	0	0	0	0	0	0	0	0	0	0	0	0	0	0	0	0	0	0
0	0	0	0	0	0	0	0	0	0	0	0	0	0	0	0	0	0	0
0	0	0	0	0	0	0	0	0	6	6	0	0	0	0	0	0	0	0
0	0	0	0	0	0	0	0	0	0	0	0	0	0	0	0	0	0	0
0	0	0	0	0	0	0	0	0	0	0	0	0	0	0	0	0	0	0
0	0	0	0	0	0	0	0	0	0	0	0	0	0	0	0	0	0	0
0	0	0	0	0	0	0	0	0	0	0	0	0	0	0	0	0	0	0
0	0	0	0	0	0	0	0	0	0	0	0	0	0	0	0	0	0	0
0	0	0	0	0	0	0	0	0	0	0	0	0	0	0	0	0	0	0
0	0	0	0	0	0	0	0	0	0	0	0	0	0	0	0	0	0	0
0	0	0	0	0	0	0	0	0	0	0	0	0	0	0	0	0	0	0
0	0	0	0	0	0	0	0	0	0	0	0	0	0	0	0	0	0	0
0	0	0	0	0	0	0	0	0	0	0	0	0	0	0	0	0	0	0
0	0	0	0	0	0	0	0	0	0	0	0	0	0	0	0	0	0	0
0	0	0	0	0	0	0	0	0	0	0	0	0	0	0	0	0	0	0
0	0	0	0	0	0	0	0	0	0	0	0	0	0	0	0	0	0	0
0	0	0	0	0	0	0	0	0	0	0	0	0	0	0	0	0	0	0
0	0	0	0	0	0	0	0	0	0	0	0	0	0	0	0	0	0	0

0	0	0	0	0	0	0	0	0	0	0	0	0	0	0	0	0	0	0
0	0	0	0	0	0	0	0	0	0	0	0	0	0	0	0	0	0	0
0	0	0	0	0	0	0	0	0	0	0	0	0	0	0	0	0	0	0
0	0	0	0	0	0	0	0	0	4	0	0	0	0	0	0	0	0	0
0	0	0	0	0	0	0	0	0	0	0	0	0	0	0	0	0	0	0
0	0	0	0	0	0	0	0	0	0	0	0	0	0	0	0	0	0	0
0	0	0	0	0	0	0	0	0	0	0	0	0	0	0	0	0	0	0
0	0	0	0	0	0	0	0	0	0	0	0	0	0	0	0	0	0	0
0	0	0	0	0	0	0	0	0	0	0	0	0	0	0	0	0	0	0
0	0	0	0	0	0	0	0	0	0	0	0	0	0	0	0	0	0	0
0	0	0	0	0	0	0	0	0	0	0	0	0	0	0	0	0	0	0
0	0	0	0	0	0	0	0	0	0	0	0	0	0	0	0	0	0	0
0	0	0	0	0	0	0	0	0	0	0	0	0	0	0	0	0	0	0
0	0	0	0	0	0	0	0	0	0	0	0	0	0	0	0	0	0	0
0	0	0	0	0	0	0	0	0	0	0	0	0	0	0	0	0	0	0
0	0	0	0	0	0	0	0	0	0	0	0	0	0	0	0	0	0	0
0	0	0	0	0	0	0	0	0	0	0	0	0	0	0	0	0	0	0
0	0	0	0	0	0	0	0	0	0	0	0	0	0	0	0	0	0	0
0	0	0	0	0	0	0	0	0	0	0	0	0	0	0	0	0	0	0
0	0	0	0	0	0	0	0	0	0	0	0	0	0	0	0	0	0	0
0	0	0	0	0	0	0	0	0	0	0	0	0	0	0	0	0	0	0
0	0	0	0	0	0	0	0	0	0	0	0	0	0	0	0	3	0	0
0	0	0	0	0	0	0	0	0	0	0	0	0	0	0	0	0	0	0
0	0	0	0	0	0	0	0	0	0	0	0	0	0	0	0	0	0	0
0	0	0	0	0	0	0	0	0	0	0	0	0	0	0	0	0	0	0
0	0	0	0	0	0	0	0	0	0	0	0	0	0	0	0	0	0	0
0	0	0	0	0	0	0	0	0	0	0	0	0	0	0	0	0	0	0
0	0	0	0	0	0	0	0	0	0	0	0	0	0	0	0	0	0	0
0	0	0	0	0	0	0	0	0	0	0	0	0	0	0	0	0	0	0
0	0	0	0	0	0	0	0	0	0	0	0	0	0	0	0	0	0	0
0	0	0	0	0	0	0	0	0	0	0	0	0	0	0	0	0	0	0
0	0	0	0	0	0	0	0	0	0	0	0	0	0	0	0	0	0	0
0	0	0	0	0	0	0	0	0	0	0	0	0	0	0	0	0	0	0
0	0	0	0	0	0	0	0	0	0	0	0	0	0	0	0	0	0	0
0	0	0	0	0	0	0	0	0	0	0	0	0	0	0	0	0	0	0
0	0	0	0	0	0	0	0	0	0	0	0	0	0	0	0	0	0	0
0	0	0	0	0	0	0	0	0	0	0	0	0	0	0	0	0	0	0

0	0	0	0	0	0	0	0	0	0	0	0	0	0	0	0	0	0	0
0	0	0	0	0	0	0	0	0	0	0	0	0	0	0	0	0	0	0
0	0	0	0	0	0	0	0	0	0	0	0	0	0	0	0	0	0	0
0	0	0	0	0	0	0	0	0	0	0	0	0	0	0	0	0	0	0
0	0	0	0	0	0	0	0	0	0	0	0	0	0	0	0	0	0	0
0	0	0	0	0	0	0	0	0	0	0	0	0	0	0	0	0	0	0
0	0	0	0	0	0	0	0	0	0	0	0	0	0	0	0	0	0	0
0	0	0	0	0	0	0	0	0	0	0	0	0	0	0	0	0	0	0
0	0	0	0	0	0	0	0	0	0	0	0	0	0	0	0	0	0	0
0	0	0	0	0	0	0	0	0	0	0	0	0	0	0	0	0	0	0
0	0	0	0	0	0	0	0	0	0	0	0	0	0	0	0	2	2	0
0	0	0	0	0	0	0	0	0	0	0	0	0	0	0	0	0	0	0
0	0	0	0	0	0	0	0	0	0	0	0	0	0	0	0	0	0	0
0	0	0	0	0	0	0	0	0	0	0	0	0	0	0	0	0	0	0
0	0	0	0	0	0	0	0	0	0	0	0	0	0	0	0	0	0	0
0	0	0	0	0	0	0	0	0	0	0	0	0	0	0	0	0	0	0
0	0	0	0	0	0	0	0	0	0	0	0	0	0	0	0	0	0	0
0	0	0	0	0	0	0	0	0	0	0	0	0	0	0	0	0	0	0
0	0	0	0	0	0	0	0	0	0	0	0	0	0	0	0	0	0	0
0	0	0	0	0	0	0	0	0	0	0	0	0	0	0	0	0	0	0
0	0	0	0	0	0	0	0	0	0	0	0	0	0	0	0	0	0	0
0	0	0	0	0	0	0	0	0	0	0	0	0	0	0	0	0	0	0
0	0	0	0	0	0	0	0	0	0									
Jan_DL025	1265	1541	0	327	2106	385	840	908	110	21	30	102	16	168	107	23	153	148
59	0	140	214	177	119	30	66	30	48	93	0	86	55	9	0	69	0	0
0	139	130	30	71	0	0	0	85	0	82	0	158	50	44	0	0	86	0
0	0	0	245	48	0	87	24	6	107	11	150	30	49	76	350	13	33	0
27	0	22	0	73	0	16	40	0	20	0	0	66	83	0	0	61	64	0
0	39	92	0	0	22	0	46	0	13	67	59	58	0	0	0	0	0	45
0	21	61	31	40	38	0	0	0	0	36	19	59	22	0	32	26	47	47
0	0	0	59	26	0	73	0	0	57	14	0	16	0	20	36	0	0	0
0	36	0	5	0	20	0	0	0	0	46	0	16	0	0	33	29	97	13
9	0	18	0	0	0	0	26	0	0	12	0	0	0	0	0	5	0	22
0	61	48	0	0	0	0	52	35	29	0	68	0	0	0	0	0	0	0
21	0	0	0	0	0	34	0	0	0	0	8	0	0	0	12	0	31	0
0	0	0	0	0	0	0	0	0	0	0	0	10	7	0	31	0	0	0
28	0	0	56	0	0	0	23	0	0	0	0	45	24	0	26	14	0	0

13	18	0	0	44	6	0	0	0	3	0	0	0	0	0	24	0	0	0
8	17	0	0	0	0	0	0	0	0	0	10	15	0	0	0	0	13	23
0	15	0	0	0	5	0	0	0	33	0	27	10	0	0	0	0	48	4
0	0	13	0	24	0	0	0	0	22	0	0	0	0	25	0	0	30	0
0	0	0	0	4	17	0	0	16	0	0	0	0	0	0	0	0	0	0
10	0	0	0	0	0	0	0	0	0	0	0	8	0	0	0	0	0	13
0	0	0	0	8	13	0	1	4	0	0	0	24	0	5	25	0	0	0
0	0	0	0	0	0	0	6	0	8	0	0	20	0	0	41	6	0	0
0	0	0	0	0	0	12	0	0	0	0	0	0	0	0	0	7	0	0
0	8	0	0	0	0	0	0	0	96	0	0	0	0	0	0	20	10	0
0	4	0	0	0	0	0	0	0	0	0	0	0	0	0	0	0	35	0
0	0	0	0	4	0	0	0	0	22	0	0	0	0	0	0	25	0	3
0	0	0	28	0	0	0	0	0	0	0	0	0	0	0	0	0	0	0
0	0	0	0	0	0	0	7	2	0	0	0	0	0	0	0	0	0	0
0	0	0	0	0	0	0	0	16	8	15	0	0	0	0	21	0	0	0
0	3	14	0	0	0	0	0	0	0	0	0	8	0	0	0	0	0	0
0	0	0	20	0	0	0	0	0	0	0	0	0	5	0	0	0	0	0
0	0	0	0	0	8	0	0	0	0	8	0	0	0	0	2	0	0	0
0	0	0	0	0	0	0	0	0	0	0	0	0	0	0	0	0	0	0
0	0	0	0	0	0	0	0	0	0	0	0	0	32	0	0	0	0	0
0	0	0	0	0	0	0	0	0	0	0	5	0	0	0	14	0	0	0
0	0	0	0	0	0	0	0	0	0	0	0	0	0	0	0	0	0	0
0	0	0	0	0	0	0	0	0	0	0	0	0	0	0	0	0	0	0
0	0	0	7	6	0	0	21	0	0	0	0	0	0	0	0	0	0	0
0	0	0	0	0	0	17	0	0	0	0	0	0	0	0	0	0	0	0
0	0	0	0	0	0	0	0	0	0	0	0	0	0	0	0	0	0	0
0	0	0	0	0	0	0	4	0	0	0	0	0	0	0	0	0	0	0
0	0	0	0	0	0	0	0	0	0	0	0	0	0	0	0	0	0	0
0	7	0	0	0	0	0	0	0	0	0	0	0	0	0	0	0	0	0
0	0	0	0	0	0	0	0	0	0	0	0	0	0	0	0	0	0	0
0	0	0	0	0	0	0	28	0	0	0	0	0	0	0	0	0	0	0
0	0	0	0	0	0	0	0	0	0	0	0	0	0	0	0	0	0	0
0	0	0	0	0	0	0	0	0	0	0	0	0	0	0	0	0	0	0
0	0	0	0	0	0	0	0	0	0	0	0	0	0	0	0	0	0	0
0	0	0	0	0	0	0	0	0	0	0	0	0	0	0	0	0	0	0
0	0	0	0	0	0	0	0	0	0	0	0	0	0	0	0	0	0	5
0	0	0	0	0	0	0	0	0	0	0	0	0	0	0	0	0	0	0

0	0	0	0	0	0	0	0	0	0	0	0	0	0	0	0	0	0	0
0	0	0	0	0	0	0	0	0	0	0	0	0	0	0	0	0	0	0
0	0	0	0	0	0	0	0	0	0	0	0	0	0	0	0	0	0	0
0	0	0	0	0	0	0	0	0	0	0	0	0	0	12	0	0	0	0
0	0	0	0	0	0	0	0	0	0	0	0	0	0	0	0	0	0	0
0	0	0	0	0	0	0	0	0	0	0	0	0	0	0	0	0	0	0
0	0	0	0	0	0	0	0	0	0	0	0	0	0	0	0	0	0	0
0	0	0	0	0	0	0	0	0	0	0	0	0	7	0	0	0	0	0
0	0	0	0	0	0	0	0	0	0	0	0	0	0	0	0	0	0	0
0	0	0	0	0	0	0	0	0	0	0	0	0	0	0	0	0	0	0
0	0	0	0	0	0	0	0	0	0	0	0	0	0	0	0	0	0	0
0	0	0	0	0	0	0	0	0	0	0	0	0	0	0	0	0	0	0
0	0	0	0	0	0	0	0	0	0	0	0	0	0	0	0	0	0	0
0	0	0	0	0	0	0	0	0	0	0	0	0	0	0	0	0	0	0
0	0	0	0	0	0	0	0	0	0	0	0	0	0	0	0	0	0	0
0	0	0	0	0	0	0	0	0	0	0	0	0	0	0	0	0	0	0
0	0	0	0	0	0	0	0	0	0	0	0	0	0	0	0	0	0	0
0	0	0	0	0	0	0	0	0	0	0	0	0	0	0	0	0	0	0
0	0	0	0	0	0	0	0	0	0	0	0	0	0	0	0	0	0	0
0	0	0	0	0	0	0	0	0	0	0	0	0	0	0	0	0	0	0
0	0	0	0	0	0	0	0	0	0	0	0	0	0	0	0	0	0	0
0	0	0	0	0	0	0	0	0	0	0	0	0	0	0	0	0	0	0
0	0	0	0	0	0	0	0	0	0	0	0	0	0	0	0	0	0	0
0	0	0	0	0	0	0	0	0	0	0	0	0	0	0	0	0	0	0
0	0	0	0	0	0	0	0	0	0	0	0	0	0	0	0	0	0	0
0	0	0	0	0	0	0	0	0	0	0	0	0	0	0	0	0	0	0
0	0	0	0	0	0	0	0	0	0	0	0	0	0	0	0	0	0	0
0	0	0	0	0	0	0	0	0	0	0	0	0	0	0	0	0	0	9
0	0	0	0	0	0	0	0	0	0	0	0	0	0	0	0	0	0	0
0	0	0	0	0	0	0	0	0	0	0	0	0	0	0	0	0	0	0
0	0	0	0	0	0	0	0	0	0	0	0	0	0	0	0	0	0	0
0	0	0	0	0	0	8	0	0	0	0	0	0	0	0	0	0	0	0
0	0	0	0	0	0	0	0	0	0	0	0	0	0	0	0	0	0	0
0	0	0	0	0	0	0	0	0	0	0	0	0	0	0	0	0	0	0
0	0	0	0	0	0	0	0	0	0	0	0	0	0	0	0	0	0	0
0	0	0	0	0	0	0	0	0	0	0	0	0	0	0	0	0	0	0
0	0	0	0	0	0	5	0	0	0	0	0	0	0	0	0	0	0	0

0	0	0	0	0	0	0	0	0	0	0	0	0	0	0	0	0	0	0
0	0	0	0	0	0	0	0	0	0	0	0	0	0	0	0	0	0	0
0	0	0	0	0	0	0	0	0	0	0	0	0	0	0	0	0	0	0
0	0	0	0	0	0	0	0	0	0	0	0	0	0	0	0	0	0	0
0	0	0	0	0	0	0	0	0	0	0	0	0	0	0	0	0	0	0
0	0	0	0	0	0	6	6	0	0	0	0	0	0	0	0	0	0	0
0	0	0	0	0	0	0	0	0	0	0	0	0	0	0	0	0	0	0
0	0	0	0	0	0	0	0	0	0	0	0	0	0	0	0	0	0	0
0	0	0	0	0	0	0	0	0	0	0	0	0	0	0	0	0	0	0
0	0	0	0	0	0	0	0	0	0	0	0	0	0	0	0	0	0	0
0	0	0	0	0	0	0	0	0	0	0	0	0	0	0	0	0	0	0
0	0	0	0	0	0	0	0	0	0	0	0	0	0	0	0	0	0	0
0	0	0	0	0	0	0	0	0	0	0	0	0	0	0	0	0	0	0
0	0	0	0	0	0	0	0	0	0	0	0	0	0	0	0	0	0	0
0	0	0	0	0	0	0	0	0	0	0	0	5	0	0	0	0	0	0
0	0	0	0	0	0	0	0	0	0	0	0	0	0	0	0	0	0	0
0	0	0	0	0	0	0	0	0	0	0	0	0	0	0	0	0	0	0
0	0	0	0	0	0	0	0	0	0	0	0	0	0	0	0	0	0	0
0	0	0	0	0	0	0	0	0	0	0	0	0	0	0	0	0	0	0
0	0	0	0	0	0	0	0	0	0	0	0	0	0	0	0	0	0	0
0	0	0	0	0	0	0	0	0	0	0	0	0	0	0	0	0	0	0
0	0	0	0	0	0	0	0	0	0	0	0	0	0	0	0	0	0	0
0	0	0	0	0	0	0	0	0	0	0	0	0	0	0	0	0	0	0
0	0	0	0	0	0	0	0	0	0	0	0	0	0	0	0	0	0	0
0	4	0	0	0	0	0	0	0	0	0	0	0	0	0	0	0	0	0
0	0	0	0	0	0	0	0	0	0	0	0	0	0	0	0	0	0	0
0	0	0	0	0	0	0	0	0	0	0	0	0	0	0	0	0	0	0
0	0	0	0	0	0	0	0	0	0	0	0	0	0	0	0	0	0	0
0	0	0	0	0	0	0	0	0	0	0	0	0	0	0	0	0	0	0
0	0	0	0	0	0	0	0	0	0	0	0	0	0	0	0	0	0	0
0	0	0	0	0	0	0	0	0	0	0	0	0	0	0	0	0	0	0
0	0	0	0	0	0	0	0	0	0	0	0	0	0	0	0	0	0	0
0	0	0	0	0	0	0	0	0	0	0	0	0	0	0	0	0	0	0
0	0	0	0	0	0	0	0	0	0	0	0	0	0	0	0	0	0	0
0	0	0	0	0	0	0	0	0	0	0	0	0	0	0	0	0	0	0
0	0	0	0	0	0	0	0	0	0	0	0	0	0	0	0	0	0	0
0	0	0	0	0	0	0	0	0	0	0	0	0	0	0	0	0	0	0

0	0	0	0	0	0	0	0	0	0	0	0	0	0	0	0	0	0	0
0	0	0	0	0	0	0	0	0	0	0	0	0	0	0	0	0	0	0
0	0	0	0	0	0	0	0	0	0	0	0	0	0	0	0	0	0	0
0	0	0	0	0	0	0	3	3	0	0	0	0	0	0	0	0	0	0
0	0	0	0	0	0	0	0	0	0	0	0	0	0	0	0	0	0	0
0	0	0	0	0	0	0	0	0	0	0	0	0	0	0	0	0	0	0
0	0	0	0	0	0	0	0	0	0	0	0	0	0	0	0	0	0	0
0	0	0	0	0	0	0	0	0	0	0	0	0	0	0	0	0	0	0
0	0	0	0	0	0	0	0	0	0	0	0	0	0	0	0	0	0	0
0	0	0	0	0	0	0	0	0	0	0	0	0	0	0	0	0	0	0
0	0	0	0	0	0	0	0	0	0	0	0	0	0	0	0	0	0	0
0	0	0	0	0	0	0	0	0	0	0	0	0	0	0	0	0	0	0
0	0	0	0	0	0	0	0	0	0	0	0	0	0	0	0	0	0	0
0	0	0	0	0	0	0	0	0	0	0	0	0	0	0	0	0	0	0
0	0	0	0	0	0	0	0	0	0	0	0	0	0	0	0	0	0	0
0	0	0	0	0	0	0	0	0	0	0	0	0	0	0	0	0	0	0
0	0	0	0	0	0	0	0	0	0	0	0	0	0	0	0	0	0	0
0	0	0	0	0	0	0	0	0	0	0	0	0	0	0	0	0	0	0
0	0	0	0	0	0	0	0	0	0	0	0	0	0	0	0	0	0	0
0	0	0	0	0	0	0	0	0	0	0	0	0	0	0	0	0	0	0
0	0	0	0	0	0	0	0	0	0	0	0	0	0	0	0	0	0	0
0	0	0	0	0	0	0	0	0	0	0	0	0	0	0	0	0	0	0
0	0	0	0	0	0	0	0	0	0	0	0	0	0	0	0	0	0	0
0	0	0	0	0	0	0	0	0	0	0	0	0	0	0	0	0	0	0
0	0	0	0	0	0	0	0	0	0	0	0	0	0	0	0	0	0	0
0	0	0	0	0	0	0	0	0	0	0	0	0	0	0	0	0	0	0
0	0	0	0	0	0	0	0	0	0	0	0	0	0	0	0	0	0	0
0	0	0	0	0	0	0	0	0	0	0	0	0	0	0	0	0	0	0
0	0	0	0	0	0	0	0	0	0	0	0	0	0	0	0	0	0	0
0	0	0	0	0	0	0	0	0	0	0	0	0	0	0	0	0	0	0
0	0	0	0	0	0	0	0	0	0	0	0	0	0	0	0	0	0	0
0	2	2	2	2	0	0	0	0	0	0	0	0	0	0	0	0	0	0
0	0	0	0	0	0	0	0	0	0	0	0	0	0	0	0	0	0	0
0	0	0	0	0	0	0	0	0	0	0	0	0	0	0	0	0	0	0
0	0	0	0	0	0	0	0	0	0	0	0	0	0	0	0	0	0	0
0	0	0	0	0	0	0	0	0	0	0	0	0	0	0	0	0	0	0
0	0	0	0	0	0	0	0	0	0	0	0	0	0	0	0	0	0	0

0	0	0	0	0	0	0	0	0	0	0	0	0	0	0	0	0	0	0
0	0	0	0	0	0	0	0	0	0	0	0	0	0	0	0	0	0	0
0	0	0	0	0	0	0	0	0	0	0	0	0	0	0	0	0	0	0
0	0	0	0	0	0	0	0	0	0	0	0	0	0	0	0	0	0	0
0	0	0	0	0	0	0	0	0	0	0	0	0	0	0	0	0	0	0
0	0	0	0	0	0	0	0	0	0	0	0	0	0	0	0	0	0	0
0	0	0	0	0	0	0	0	0	0	0	0	0	0	0	0	0	0	0
0	0	0	0	0	0	0	0	0	0	0	0	0	0	0	0	0	0	0
0	0	0	0	0	0	0	0	0	0	0	0	0	0	0	0	0	0	0
0	0	0	0	0	0	0	0	0	0	0	0	0	0	0	0	0	0	0
0	0	0	0	0	0	0	0	0	0	0	0	0	0	0	0	0	0	0
0	0	0	0	0	0	0	0	0	0	0	0	0	0	0	0	0	0	0
0	0	0	0	0	0	0	0	0	0	0	0	0	0	0	0	0	0	0
0	0	0	0	0	0	0	0	0	0	0	0	0	0	0	0	0	1	1
0	0	0	0	0	0	0	0	0	0									
Jan_DL026	1213	2075	223	545	190	429	491	510	48	203	210	351	177	500	603	149	479	144
179	0	261	480	279	201	113	153	196	111	125	0	135	155	84	133	139	130	0
102	213	92	58	158	137	87	12	63	54	138	34	221	65	21	40	0	129	0
0	73	0	68	98	129	0	87	74	103	51	155	9	99	46	16	82	58	0
41	29	0	28	0	39	41	17	142	0	0	44	196	0	42	0	94	23	0
74	36	110	0	23	39	54	96	315	42	55	11	57	42	31	51	53	0	110
0	53	90	6	34	0	0	0	0	0	16	41	30	16	51	222	10	72	0
0	0	0	46	0	0	35	0	0	0	41	0	11	0	0	23	0	549	0
0	0	26	11	0	5	0	0	130	0	24	0	16	0	0	16	0	0	42
0	0	12	0	0	27	0	0	0	19	0	9	0	110	0	0	7	0	0
0	51	42	0	0	168	0	35	0	11	0	0	0	0	16	0	0	0	519
18	0	0	0	0	0	45	0	0	0	0	0	0	34	0	0	0	34	34
0	0	21	21	0	0	0	6	0	86	0	0	9	7	0	22	0	40	0
20	0	0	37	0	0	0	0	0	0	0	0	0	0	7	0	17	0	0
10	0	0	0	20	21	0	0	21	107	79	0	0	6	22	0	0	0	0
0	0	20	0	0	0	0	0	7	0	36	0	0	0	11	19	9	0	0
0	3	10	0	0	51	0	35	0	0	0	6	15	0	27	8	0	21	8
0	0	0	0	0	0	17	0	0	7	0	0	0	28	0	0	0	0	0
0	0	0	0	7	0	0	0	0	0	0	0	17	12	0	0	15	0	3
0	0	23	0	0	70	0	5	0	0	0	0	0	0	0	0	0	0	0
0	0	0	0	0	0	0	0	0	0	0	0	0	0	0	0	111	0	0
0	0	0	0	16	16	0	4	0	0	0	9	13	0	0	0	13	0	0

0	0	12	0	0	0	0	21	0	0	0	0	9	0	0	0	2	0	0
0	0	0	0	0	10	0	0	0	0	0	0	0	0	0	0	0	0	0
0	0	0	0	0	0	0	0	0	0	0	0	0	42	0	0	0	0	33
0	0	0	0	0	0	0	0	0	0	0	0	0	0	0	5	0	0	3
16	0	16	0	0	0	0	0	0	0	0	0	0	0	0	0	14	0	0
0	17	0	16	0	0	0	0	0	50	0	0	0	0	0	15	0	0	0
0	0	0	0	0	0	0	0	0	0	0	0	0	0	0	0	0	0	0
15	0	0	0	0	0	0	0	0	0	0	11	0	0	0	28	0	0	0
0	0	15	0	0	0	14	0	0	0	0	0	0	0	20	0	0	0	2
0	0	0	0	0	0	17	0	0	0	0	0	16	0	0	0	0	0	0
0	0	0	0	0	0	0	0	0	12	0	0	0	0	0	0	0	0	0
0	0	0	0	0	0	0	0	0	0	0	0	0	0	0	0	0	0	0
0	0	0	0	13	0	0	0	0	0	0	0	0	0	0	0	0	0	0
8	0	0	0	0	0	0	0	0	0	0	0	0	0	51	13	0	0	0
0	0	0	0	0	0	0	0	27	0	0	0	32	0	0	0	0	0	0
0	0	0	0	0	30	0	0	15	0	0	0	0	0	0	0	13	0	0
0	0	0	0	0	0	0	0	0	0	0	0	0	0	0	0	0	0	12
0	0	0	0	0	0	3	8	0	0	0	0	0	0	0	0	0	0	0
0	0	0	0	0	0	0	0	0	13	0	0	0	0	0	0	0	0	0
0	0	0	0	0	0	0	0	0	0	0	0	0	0	0	0	0	0	0
0	0	0	0	0	0	0	0	0	0	0	0	0	0	0	0	0	0	0
0	0	0	0	0	0	0	0	0	0	0	0	0	0	0	12	0	0	0
0	0	0	10	0	0	11	0	0	0	0	0	12	0	0	0	0	0	0
0	0	0	0	0	0	0	0	0	0	0	0	0	0	0	0	0	0	0
0	0	0	19	0	0	0	0	0	0	0	0	0	0	0	0	0	8	0
0	0	0	0	0	0	0	0	0	0	0	0	0	0	0	0	0	0	0
0	0	0	0	0	0	0	0	0	0	0	0	0	0	0	0	0	0	0
0	0	0	0	0	0	26	0	0	0	0	0	0	0	0	0	0	0	0
0	0	0	0	0	0	0	0	0	0	0	0	0	0	0	0	0	0	0
0	0	0	0	4	0	0	0	0	0	0	0	0	0	0	0	0	0	0
0	0	0	0	0	0	13	0	0	0	0	0	0	0	0	0	0	0	0
0	0	0	0	6	0	0	0	0	0	0	0	0	0	0	0	0	0	0
0	0	0	0	0	0	0	0	0	0	0	0	0	0	0	0	0	0	0
0	0	0	0	0	0	0	0	0	0	0	0	0	0	0	0	0	0	0
0	0	0	0	0	0	0	0	0	0	0	0	0	0	0	24	0	0	0
0	0	0	0	0	0	0	0	0	0	0	0	0	0	0	0	0	0	0
0	0	0	0	0	0	0	0	0	0	0	0	0	19	0	0	0	0	0

0	0	0	0	16	0	0	0	0	0	0	0	0	0	0	0	0	0	0
0	0	0	0	0	0	0	0	0	0	0	0	0	0	0	0	0	0	0
0	0	0	0	0	0	0	0	0	0	0	0	0	0	0	0	0	0	0
0	0	0	0	0	0	0	0	0	0	0	0	0	0	0	0	0	0	0
13	0	0	0	0	3	0	0	0	0	0	0	0	0	0	8	0	0	0
0	0	0	0	0	0	0	0	0	0	0	0	0	0	0	0	0	0	0
0	0	0	0	0	0	0	0	0	8	0	0	0	0	0	0	0	0	0
0	0	0	0	0	0	0	0	0	0	0	0	0	0	0	0	0	0	0
0	0	0	0	0	0	0	0	0	0	0	0	0	0	0	0	0	0	0
0	0	0	0	0	0	0	0	0	0	0	0	0	0	0	0	0	0	0
0	0	0	0	0	0	0	0	0	0	0	0	0	0	0	0	0	0	0
0	0	0	0	0	0	0	0	0	0	0	0	0	0	0	0	0	0	0
0	0	0	0	0	0	0	0	0	0	0	0	0	0	0	0	0	0	0
0	0	5	0	0	0	0	0	0	0	0	0	0	0	0	0	0	0	0
0	0	0	0	0	0	0	0	0	0	0	0	0	0	0	0	0	0	0
0	0	0	0	0	0	0	0	0	0	0	0	0	0	0	0	0	0	0
0	0	0	0	0	0	0	0	0	0	0	0	0	0	0	0	0	0	0
0	0	0	0	0	0	0	0	0	0	0	0	0	0	0	0	0	0	0
0	0	0	0	0	1	0	0	0	0	0	0	0	0	0	0	0	0	0
0	0	0	0	0	0	0	0	7	0	0	0	0	0	0	0	0	0	0
0	0	0	0	0	0	0	0	0	0	0	0	0	0	0	0	0	0	0
0	0	0	0	0	0	0	0	0	0	0	0	0	0	0	0	0	0	0
0	0	0	0	0	0	0	0	0	0	0	0	0	0	0	0	0	0	0
0	0	0	0	0	0	0	0	0	0	0	0	0	0	0	0	0	0	0
0	0	0	0	0	0	0	0	0	0	0	0	0	0	0	0	0	0	0
0	0	0	0	0	0	0	0	0	0	0	0	0	0	0	0	0	0	0
0	0	0	0	0	0	0	0	0	0	0	0	0	0	0	0	0	0	0
0	0	0	0	0	0	0	0	0	0	0	0	0	0	0	0	0	0	0
0	0	0	0	0	0	0	0	0	0	0	0	0	0	0	0	0	0	0
0	0	0	0	0	0	0	0	0	0	0	0	0	0	0	0	0	0	0
0	0	0	0	0	0	0	0	0	0	0	0	0	0	0	0	0	4	0
0	0	0	0	0	0	0	0	0	0	0	0	0	0	0	0	0	0	0
0	0	0	0	0	0	0	0	0	0	0	0	0	0	0	0	0	0	0
0	0	0	0	0	0	0	0	0	0	0	0	0	0	0	0	0	0	0
0	0	0	0	0	0	0	0	0	0	0	0	0	0	0	0	0	0	0
0	0	0	0	0	0	0	0	0	0	0	0	0	0	0	0	0	0	0
0	0	0	0	0	0	0	0	0	0	0	0	0	4	0	0	0	0	0

0	0	0	0	0	0	0	0	0	0	0	0	0	0	0	0	0	0	0
0	0	0	0	0	0	0	0	0	0	0	0	0	0	0	0	0	0	0
0	0	0	0	0	0	0	0	0	0	0	0	0	0	0	0	0	0	0
0	0	0	0	0	0	0	0	0	0	0	0	0	0	0	0	0	0	0
0	0	0	0	0	0	0	0	0	0	0	0	0	0	0	0	0	0	0
0	0	0	0	0	0	0	0	0	0	0	0	0	0	0	0	0	0	0
0	0	0	0	0	0	0	0	0	0	0	0	0	0	0	0	0	0	0
0	0	0	0	0	0	0	0	0	0	0	0	0	0	0	0	0	0	0
0	0	0	0	0	0	0	0	0	0	0	0	0	0	0	0	0	0	0
0	0	0	0	0	0	0	0	0	0	0	0	0	0	0	0	0	0	0
0	0	0	0	0	0	0	0	0	0	0	0	0	0	0	0	0	0	0
0	0	0	0	0	0	0	0	0	0	0	0	0	0	0	0	0	0	0
0	0	0	0	0	0	0	0	0	0	0	0	0	0	0	0	0	0	0
0	0	0	0	0	0	0	0	0	0	0	0	0	0	0	0	0	0	0
0	0	0	0	0	0	0	0	0	0	0	0	0	0	0	0	0	0	0
0	0	0	0	0	0	0	0	0	0	0	0	0	0	0	0	0	0	0
0	0	0	0	0	0	0	0	0	0	0	0	0	0	0	0	0	0	0
0	0	0	0	0	0	0	0	0	0	0	0	0	0	0	0	0	0	0
0	0	0	0	0	0	0	0	0	0	0	0	0	0	0	0	0	0	0
0	0	0	0	0	0	0	0	0	0	0	0	0	0	0	0	0	0	0
0	0	0	0	0	0	0	0	0	0	0	0	0	0	0	0	0	0	0
0	0	0	0	0	0	0	0	0	0	0	0	0	0	0	0	0	0	0
0	0	0	0	0	0	0	0	0	0	0	0	0	0	0	0	0	0	0
0	0	0	0	0	0	0	0	0	0	0	0	0	0	0	0	0	0	0
0	0	0	0	0	0	0	0	0	0	0	0	0	0	0	0	0	0	0
0	0	0	0	0	0	0	0	0	0	0	0	0	0	0	0	0	0	0
0	0	0	0	0	0	0	0	0	0	0	0	0	0	0	0	0	0	0
0	0	0	0	0	0	0	0	0	0	0	0	0	0	0	0	0	0	0
0	0	0	0	0	0	0	0	0	0	0	0	0	0	0	0	0	0	0
0	0	0	0	0	0	0	0	0	0	0	0	0	0	0	0	0	0	0
0	0	0	0	0	0	0	0	0	0	0	0	0	0	0	0	0	0	0
0	0	0	0	0	0	0	0	0	0	0	0	0	0	0	0	0	0	0
0	0	0	0	0	0	0	0	0	0	0	0	0	0	0	0	0	0	0
0	0	0	0	0	0	0	0	0	0	0	0	0	0	0	0	0	0	0
0	0	0	0	0	0	0	0	0	0	0	0	0	0	0	0	0	0	0
0	0	0	0	0	0	0	0	0	0	0	0	0	0	0	0	0	0	0
0	0	0	0	0	0	0	0	0	0	0	0	0	0	0	0	0	0	0

0	0	0	0	0	0	0	0	0	0	0	0	0	0	0	0	0	0	0
0	0	0	0	0	0	0	0	0	0	0	0	0	0	0	0	0	0	0
0	0	0	0	0	0	0	0	0	0	0	0	0	0	0	0	0	0	0
0	0	0	0	0	0	0	0	0	0	0	0	0	0	0	0	0	0	0
0	0	0	0	0	0	0	0	0	0	0	0	0	0	0	0	0	0	0
0	0	0	0	0	0	0	0	0	0	0	0	0	0	0	0	0	0	0
0	0	0	0	0	0	0	0	0	0	0	0	0	0	0	0	0	0	0
0	0	0	0	0	0	0	0	0	0	0	0	0	0	0	0	0	0	0
0	0	0	0	0	0	0	0	0	0	0	0	0	0	0	0	0	0	0
0	0	0	0	0	0	0	0	0	0	0	0	0	0	0	0	0	0	0
0	0	0	0	0	0	0	0	0	0	0	0	0	0	0	0	0	0	0
0	0	0	0	0	0	0	0	0	0	0	0	0	0	0	0	0	0	0
0	0	0	0	0	0	0	0	0	0	0	0	0	0	0	0	0	0	0
0	0	0	0	0	0	0	0	0	0	0	0	0	0	0	0	0	0	0
0	0	0	0	0	0	0	0	0	0	0	0	0	0	0	0	0	0	0
0	0	0	0	0	0	0	0	0	0	0	0	0	0	0	0	0	0	0
0	0	0	0	0	0	3	0	0	0	0	0	0	0	0	0	0	0	0
0	0	0	0	0	0	0	0	0	0	0	0	0	0	0	0	0	0	0
0	0	0	0	0	0	0	0	0	0	0	0	0	0	0	0	0	0	0
0	0	0	0	0	0	0	0	0	0	0	0	0	0	0	0	0	0	0
0	0	0	0	0	0	0	0	0	0	0	0	0	0	0	0	0	0	0
0	0	0	0	0	0	0	0	0	0	0	0	0	0	0	0	0	0	0
0	0	0	0	0	0	0	0	0	0	0	0	0	0	0	0	0	0	0
0	0	0	0	0	0	0	0	0	0	0	0	0	0	0	0	0	0	0
0	0	0	0	0	0	0	0	0	0	0	0	0	0	0	0	0	0	0
0	0	0	0	0	0	0	0	0	0	0	0	0	0	0	0	0	0	0
0	0	0	0	0	0	0	0	0	0	0	0	0	0	0	0	0	0	0
0	0	0	0	0	0	0	0	0	0	0	0	0	0	0	0	0	0	0
0	0	0	0	0	0	0	0	0	0	0	0	0	0	0	0	0	0	0
0	0	0	0	0	0	0	0	0	0	0	0	0	0	0	0	0	0	0
0	0	0	0	0	0	0	0	0	0	0	0	0	0	0	0	0	0	0
0	0	0	0	0	0	0	0	0	0	0	0	0	0	0	0	0	0	0
0	0	0	0	0	0	0	0	0	0	0	0	0	0	0	0	0	0	0
0	0	0	0	0	0	0	0	0	0	0	0	0	0	0	0	0	0	0
0	0	0	0	0	0	0	0	0	0	0	0	0	0	0	0	0	0	0
0	0	0	0	0	0	0	0	0	0	0	0	0	0	0	0	0	0	0
0	0	0	0	0	0	0	0	0	0	0	0	0	0	0	0	0	0	0

0	0	0	0	0	0	0	0	0	0	0	0	0	0	0	0	0	0	2
2	0	0	0	0	0	0	0	0	0	0	0	0	0	0	0	0	0	0
0	0	0	0	0	0	0	0	0	0	0	0	0	0	0	0	0	0	0
0	0	0	0	0	0	0	0	0	0	0	0	0	0	0	0	0	0	0
0	0	0	0	0	0	0	0	0	0	0	0	0	0	0	0	0	0	0
0	0	0	0	0	0	0	0	0	0	0	0	0	0	0	0	0	0	0
0	0	0	0	0	0	0	0	0	0									
Jan_DL028	1707	1845	1125	770	177	107	62	64	146	461	154	247	378	59	182	466	43	220
217	0	159	597	135	168	291	127	321	255	72	0	126	294	229	79	104	239	0
229	229	23	20	104	57	107	162	37	198	46	115	230	85	22	91	95	85	0
0	208	88	32	0	79	47	34	163	263	150	0	118	68	387	21	15	215	39
57	38	32	0	0	36	58	0	49	105	0	0	30	0	0	0	54	11	36
0	35	0	69	75	7	173	72	13	30	0	0	0	0	27	0	23	0	65
0	42	0	0	14	0	0	0	0	0	13	0	19	0	0	0	0	0	0
0	0	0	16	0	87	0	0	73	19	0	0	8	0	40	0	0	148	0
0	0	12	14	0	16	0	0	10	0	0	0	16	0	78	0	0	35	22
0	0	26	0	229	0	0	0	0	31	0	20	0	0	0	14	0	0	0
45	0	9	0	0	0	0	0	41	6	0	0	0	0	0	0	0	0	123
0	0	0	0	0	46	15	0	0	0	0	17	26	0	0	0	21	0	0
0	0	0	20	0	0	0	0	0	0	0	11	8	2	18	0	33	0	0
0	0	0	8	0	0	0	15	0	0	0	0	0	0	10	6	0	0	0
9	0	0	0	8	0	0	0	0	0	6	0	0	0	0	4	0	0	0
0	0	0	0	0	0	0	0	11	0	26	28	16	38	10	0	0	0	0
6	0	11	0	0	0	0	0	0	0	12	0	0	0	0	16	0	0	0
0	0	0	0	0	0	0	0	0	0	0	296	0	0	0	0	0	0	0
0	0	0	22	9	0	0	0	0	0	0	0	7	0	20	0	0	0	0
0	9	0	0	0	0	6	8	0	9	0	0	0	0	0	0	0	0	10
0	0	0	0	9	8	6	32	0	0	0	8	0	0	14	0	0	0	0
0	0	0	10	0	0	9	19	0	0	0	0	0	0	0	16	6	0	0
0	0	0	0	0	0	7	0	0	0	6	0	16	0	0	0	0	0	0
0	9	0	0	0	0	0	0	0	0	0	0	0	0	0	0	0	5	0
0	0	0	0	0	0	0	0	0	0	0	0	0	0	0	6	0	0	0
0	0	0	0	0	0	0	0	0	0	0	0	0	0	0	0	0	0	4
6	0	0	0	0	0	0	0	0	0	0	0	0	0	0	0	0	7	0
22	0	0	0	0	0	0	0	0	0	0	0	0	0	0	0	7	23	0
0	0	0	0	0	0	0	0	12	0	0	0	0	0	0	0	0	0	0
0	9	0	0	0	0	0	0	0	0	0	0	0	0	0	0	0	0	0

0	4	0	0	0	0	0	0	0	0	0	0	0	0	0	0	14	0	0
5	0	9	0	0	0	0	0	0	0	0	0	0	17	0	0	0	0	0
0	3	0	0	0	0	0	0	0	0	25	0	0	12	0	0	0	0	0
0	0	0	0	0	0	0	0	0	0	0	0	0	0	0	0	0	0	0
0	0	0	0	0	0	0	0	0	0	0	0	0	0	0	0	5	5	0
0	0	0	0	0	0	0	0	0	0	5	0	0	0	0	0	0	0	0
0	0	0	0	0	0	0	0	0	0	0	0	0	0	0	0	0	0	0
0	0	0	0	0	0	0	0	0	0	0	0	0	0	0	0	0	0	0
0	0	0	0	0	0	0	0	0	0	0	0	0	0	0	0	0	0	0
0	0	0	0	0	0	0	0	0	0	0	0	0	0	0	0	0	0	0
0	0	0	0	0	0	0	0	0	0	0	0	0	0	0	0	0	9	0
0	0	0	0	0	0	0	0	0	0	0	0	0	0	0	0	0	0	0
0	0	0	0	0	0	0	0	0	0	0	0	0	0	0	0	0	0	0
0	0	0	0	0	0	11	0	0	0	0	0	0	0	0	0	0	0	0
0	0	0	0	0	0	0	0	0	0	0	0	7	0	0	0	0	0	0
0	0	0	0	0	0	0	0	0	0	0	0	0	0	0	0	0	0	0
6	0	0	20	0	0	0	0	0	0	0	0	0	0	0	0	0	0	0
0	0	0	0	6	0	0	0	0	0	0	0	0	0	0	0	0	0	0
0	0	0	14	0	0	0	0	0	0	0	0	0	0	0	0	0	0	0
0	0	0	0	0	0	0	0	0	0	0	0	0	0	0	0	0	0	0
0	0	0	0	0	0	0	0	0	0	0	0	0	0	0	0	0	0	0
0	0	0	0	0	0	0	0	0	0	0	0	0	0	0	0	0	0	0
0	0	0	0	0	0	0	0	0	0	0	0	0	0	0	0	0	0	0
0	0	0	0	0	0	0	0	0	0	0	0	0	0	0	0	0	0	0
0	0	0	0	0	0	0	0	0	0	0	0	0	0	0	0	0	0	0
0	0	0	0	0	0	0	0	0	0	0	0	0	0	0	0	0	0	0
0	0	0	0	0	0	0	0	0	0	0	0	0	0	0	0	0	0	0
0	0	0	0	0	0	0	0	0	0	0	0	0	0	0	0	0	0	0
0	0	0	0	0	0	0	0	0	0	0	0	0	0	0	0	0	0	0
0	0	0	0	0	0	0	0	0	0	0	0	0	0	0	0	0	0	0
0	0	0	0	0	0	0	0	0	0	0	0	0	0	0	0	0	0	0
0	0	0	0	0	0	0	0	0	0	0	0	0	0	0	0	0	0	0
0	0	0	0	0	0	0	0	0	0	0	0	0	0	0	0	0	0	0
0	0	0	0	0	0	0	0	0	0	0	0	0	0	0	0	0	0	0
0	0	0	0	0	0	0	0	0	0	0	0	0	0	0	0	0	0	0
0	0	0	0	0	0	0	0	0	0	0	0	0	0	0	0	0	0	0
0	0	0	0	0	0	0	0	0	0	0	0	0	0	0	3	0	0	0

0	0	0	0	0	0	0	0	0	0	0	0	0	0	0	0	0	0	0
0	0	0	0	0	0	0	0	0	0	0	0	0	0	0	0	0	0	0
0	0	0	0	0	0	0	0	0	0	0	0	0	0	0	0	0	0	0
0	0	0	0	0	0	0	0	0	0	0	0	0	0	0	0	0	0	0
0	0	0	0	0	0	0	0	0	0	0	0	0	0	0	0	0	0	0
0	0	0	0	0	0	0	0	0	0	0	0	0	0	0	0	0	0	0
0	0	0	0	0	0	0	0	0	0	0	0	0	0	0	0	0	0	0
0	0	0	0	0	0	0	0	0	0	0	0	0	0	0	0	0	0	0
0	0	0	0	0	0	0	0	0	0	0	0	0	0	0	0	0	0	0
0	0	0	0	0	0	12	0	0	0	0	0	0	0	0	0	0	0	0
0	0	0	0	0	0	0	0	0	0	0	0	0	0	0	0	0	0	0
0	0	0	0	0	0	0	0	0	0	0	0	0	0	0	0	0	0	0
0	0	0	0	0	0	0	0	0	0	0	0	0	0	0	0	0	0	0
0	0	0	0	0	0	0	7	0	0	0	0	0	0	0	0	0	0	0
0	0	0	0	0	0	0	0	0	0	0	0	0	0	0	0	0	0	0
0	0	0	0	0	0	0	0	0	0	0	0	0	0	0	0	0	0	0
0	0	0	0	0	0	0	0	0	0	0	0	0	0	0	0	0	0	0
0	0	0	0	0	0	0	0	0	0	0	0	0	0	0	0	0	0	0
0	0	0	0	0	0	0	0	0	0	0	0	0	0	0	0	0	0	0
0	0	0	0	0	0	0	0	0	0	0	0	0	0	0	0	0	0	0
0	0	0	0	0	0	0	0	0	0	0	0	0	0	0	0	0	0	0
0	0	0	0	0	0	0	0	0	0	0	0	0	0	0	0	0	0	0
0	0	0	0	0	0	0	0	0	0	0	0	0	0	0	0	0	0	0
0	0	0	0	0	0	0	0	0	0	0	0	0	0	0	0	0	0	0
0	0	0	0	0	0	0	0	0	0	0	0	0	0	0	0	0	0	0
0	0	0	0	0	0	0	0	0	0	0	0	0	0	0	0	0	0	0
0	0	0	0	0	0	0	0	0	0	0	0	0	0	0	0	0	0	0
0	0	0	0	0	0	0	0	0	0	0	0	0	0	0	0	0	0	0
0	0	0	0	0	0	0	0	0	0	0	0	0	0	0	0	0	0	0
0	0	0	0	0	0	0	0	0	0	0	0	0	0	0	0	0	0	0
0	0	0	0	0	0	0	0	0	0	0	0	0	0	0	0	0	0	0
0	0	0	0	0	0	0	0	0	0	0	0	0	0	0	0	0	0	0
0	0	0	0	0	0	0	0	0	0	0	0	0	0	0	0	0	0	0
0	0	0	0	0	0	0	0	0	0	0	0	0	0	0	0	0	0	0
0	0	0	0	0	0	0	0	0	0	0	0	0	0	0	0	0	0	0
0	0	0	0	0	0	0	0	0	0	0	0	0	0	0	0	0	0	0
0	0	0	0	0	0	0	0	0	0	0	0	0	0	0	0	0	0	0

0	0	0	0	0	0	0	0	0	0	0	0	0	0	0	0	0	0	0
0	0	0	0	0	0	0	0	0	0	0	0	0	0	0	0	0	0	0
0	0	0	0	0	0	0	0	0	0	0	0	0	0	0	0	0	0	0
0	0	0	0	0	0	0	0	0	0	0	0	0	0	0	0	0	0	0
0	0	0	0	0	0	0	0	0	0	0	0	0	0	0	0	0	0	0
0	0	0	0	0	0	0	0	0	0	0	0	0	0	0	0	0	0	0
0	0	0	0	0	0	0	0	0	0	0	0	0	0	0	0	0	0	0
0	0	0	0	0	0	0	0	0	0	0	0	0	0	0	0	0	0	0
0	0	0	0	0	0	0	0	0	0	0	0	0	0	0	0	0	0	0
0	0	0	0	0	0	0	0	0	0	0	0	0	0	0	0	0	0	0
0	0	0	0	0	0	0	0	0	0	0	0	0	0	0	0	0	0	0
0	0	0	0	0	0	0	0	0	0	0	0	0	0	0	0	0	0	0
0	0	0	0	0	0	0	0	0	0	0	0	0	0	0	0	0	0	0
0	0	0	0	0	0	0	0	0	0	0	0	0	0	0	0	0	0	0
0	0	0	0	0	0	0	0	0	0	0	0	0	0	0	0	0	0	0
0	0	0	0	0	0	0	0	0	0	0	0	0	0	0	0	0	0	0
0	0	0	0	0	0	0	0	0	0	0	0	0	0	0	0	0	0	0
0	0	0	0	0	0	0	0	0	0	0	0	0	0	0	0	0	0	0
0	0	0	0	0	0	0	0	0	0	0	0	0	0	0	0	0	0	0
0	0	0	0	0	0	0	0	0	0	0	0	0	0	0	0	0	0	0
0	0	0	0	0	0	0	0	0	0	0	0	4	4	4	0	0	0	0
0	0	0	0	0	0	0	0	0	0	0	0	0	0	0	0	0	0	0
0	0	0	0	0	0	0	0	0	0	0	0	0	0	0	0	0	0	0
0	0	0	0	0	0	0	0	0	0	0	0	0	0	0	0	0	0	0
0	0	0	0	0	0	0	0	0	0	0	0	0	0	0	0	0	0	0
0	0	0	0	0	0	0	0	0	0	0	0	0	0	0	0	0	0	0
0	0	0	0	0	0	0	0	0	0	0	0	0	0	0	0	0	0	0
0	0	0	0	0	0	0	0	0	0	0	0	0	0	0	0	0	0	0
0	0	0	0	0	0	0	0	0	0	0	0	0	0	0	0	0	0	0
0	0	0	0	0	0	0	0	0	0	0	0	0	0	0	0	0	0	0
0	0	0	0	0	0	0	0	0	0	0	0	0	0	0	0	0	0	0
0	0	0	0	0	0	0	0	0	0	0	0	0	0	0	0	0	0	0
0	0	0	0	0	0	0	0	0	0	0	0	0	0	0	0	0	0	0
0	0	0	0	0	0	0	0	0	0	0	0	0	0	0	0	0	0	0
0	0	0	0	0	0	0	0	0	0	0	0	0	0	0	0	0	0	0
0	0	0	0	0	0	0	0	0	0	0	0	0	0	0	0	0	0	0
0	0	0	0	0	0	0	0	0	0	0	0	0	0	0	0	0	0	0

0	0	0	0	0	0	0	0	0	0	0	0	0	0	0	0	0	0	0
0	0	0	0	0	0	0	0	0	0	0	0	0	0	0	0	0	0	0
0	0	0	0	0	0	0	0	0	0	0	0	0	0	0	0	0	0	0
0	0	0	0	0	0	0	0	0	0	0	0	0	0	0	0	0	0	0
0	0	0	0	0	0	0	0	0	0	0	0	0	0	0	0	0	0	0
0	0	0	0	0	0	0	0	0	0	0	0	0	0	0	0	0	0	0
0	0	0	0	0	0	0	0	0	0	0	0	0	0	0	0	0	0	0
0	0	0	0	0	0	0	0	0	0	0	0	0	0	0	0	0	0	0
0	0	0	0	0	0	0	0	0	0	0	0	0	0	0	0	0	0	0
0	0	0	0	0	0	0	0	0	0	0	0	0	0	0	0	0	0	0
0	0	0	0	0	0	0	0	0	0	0	0	0	0	0	0	0	0	0
0	0	0	0	0	0	0	0	0	0	0	0	0	0	0	0	0	0	0
0	0	0	0	0	0	0	0	0	0	0	0	0	0	0	0	0	0	0
0	0	0	0	0	0	0	0	0	0	0	0	0	0	0	0	0	0	0
0	0	0	0	0	0	0	0	0	0	0	0	0	0	0	0	0	0	0
0	0	0	0	0	0	0	0	0	0	0	0	0	0	0	0	0	0	0
0	0	0	0	0	0	0	0	0	0	0	0	0	0	0	0	0	0	0
0	0	0	0	0	0	0	0	0	0	0	0	0	0	0	0	0	0	0
0	0	0	0	0	0	0	0	0	0	0	0	0	0	0	0	0	0	0
0	0	0	0	0	0	0	0	0	0	0	0	0	0	0	0	0	0	0
0	0	0	0	0	0	0	0	0	0	0	0	0	0	0	0	0	0	0
0	0	0	0	0	0	0	0	0	0	0	0	0	0	0	0	0	0	0
0	0	0	0	0	0	0	0	0	0	0	0	0	0	0	0	0	0	0
0	0	0	0	0	0	0	0	0	0	0	0	0	0	0	0	0	0	0
0	0	0	0	0	0	0	0	0	0	0	0	0	0	0	0	0	0	0
0	0	0	0	0	0	0	0	0	0	0	0	0	0	0	0	0	0	0
0	0	0	0	0	0	0	0	0	0	0	0	0	0	0	0	2	2	2
2	2	2	2	0	0	0	0	0	0	0	0	0	0	0	0	0	0	0
0	0	0	0	0	0	0	0	0	0	0	0	0	0	0	0	0	0	0
0	0	0	0	0	0	0	0	0	0	0	0	0	0	0	0	0	0	0
0	0	0	0	0	0	0	0	0	0	0	0	0	0	0	0	0	0	0
0	0	0	0	0	0	0	0	0	0	0	0	0	0	0	0	0	0	0
0	0	0	0	0	0	0	0	0	0	0	0	0	0	0	0	0	0	0
0	0	0	0	0	0	0	0	0	0	0	0	0	0	0	0	0	0	0
0	0	0	0	0	0	0	0	0	0	0	0	0	0	0	0	0	0	0
0	0	0	0	0	0	0	0	0	0									

Jan_DL029	2263	975	516	1271	35	235	260	256	245	413	336	380	304	94	226	385	75	361
310	0	104	545	191	92	245	172	203	174	91	0	62	209	171	152	111	43	0
166	191	0	0	95	120	195	68	37	171	49	63	210	119	40	69	136	69	0
0	236	55	36	45	103	100	26	81	36	85	60	11	75	74	38	35	172	0
44	35	32	50	9	45	84	29	0	75	0	79	29	0	63	0	23	18	0
0	0	0	72	0	0	61	41	7	0	0	16	0	13	28	0	17	0	0
0	49	0	4	17	19	0	0	0	0	19	0	0	0	0	0	0	0	0
9	0	0	0	0	97	0	0	0	0	0	0	0	0	48	15	0	0	0
0	10	0	0	0	13	19	0	8	0	8	0	0	0	0	0	0	19	26
14	0	21	0	0	0	0	18	0	0	0	9	0	0	0	0	33	0	46
0	0	0	0	0	0	55	0	0	6	0	0	0	0	0	0	0	0	0
15	0	0	0	30	0	0	0	0	0	0	0	12	0	0	0	0	0	0
0	0	17	0	0	0	0	0	0	0	0	0	9	20	0	12	0	0	0
0	0	0	0	0	0	0	0	0	0	0	0	18	0	8	10	0	0	0
0	0	0	0	0	10	0	0	0	0	0	0	0	0	0	8	0	0	0
0	0	0	0	0	0	0	0	4	0	0	35	6	55	0	0	0	0	0
0	7	0	0	0	0	0	0	0	5	0	0	0	7	5	0	0	0	0
0	0	0	0	0	0	0	0	0	0	0	0	0	0	0	0	0	0	0
8	0	0	0	0	0	0	0	0	0	0	0	0	0	7	0	0	0	0
0	0	0	0	7	0	11	0	0	0	0	0	0	0	0	0	0	0	0
0	16	0	0	0	6	0	0	0	0	0	0	0	0	0	0	0	0	0
0	0	0	0	0	0	2	7	0	0	0	0	0	8	36	0	0	0	0
0	0	0	0	0	0	0	0	0	0	0	0	0	0	0	0	0	0	0
0	0	0	0	0	0	0	0	0	0	0	0	0	0	0	0	0	0	0
0	0	0	0	0	0	0	8	0	0	0	0	0	0	0	25	0	0	0
0	0	0	0	12	0	0	0	0	0	0	0	0	0	0	0	0	15	0
0	0	0	0	0	0	0	0	0	0	0	0	0	0	0	0	0	0	0
0	10	0	0	0	0	0	0	0	0	0	0	0	0	0	0	0	13	0
0	0	0	0	0	0	0	0	0	0	0	0	0	0	0	0	0	0	0
0	0	0	0	0	0	0	0	0	0	0	0	0	0	0	0	0	0	0
0	0	0	0	0	0	0	0	0	0	0	0	0	0	0	0	0	0	0
0	0	5	0	0	0	0	0	0	0	0	0	0	0	0	0	0	0	0
0	4	0	0	0	0	0	0	0	0	18	0	0	0	0	0	0	0	0
0	0	0	0	0	0	0	0	0	0	0	0	0	0	0	0	0	0	0
0	0	0	0	0	0	0	0	0	0	0	0	0	0	0	0	0	0	0
0	0	0	0	0	0	0	0	0	0	0	0	0	0	0	0	0	0	0
0	0	0	0	0	0	0	0	0	0	8	0	0	0	0	0	0	0	0

0	0	0	0	0	0	0	0	0	0	0	0	0	0	0	0	0	0	0
0	0	0	0	0	0	0	0	0	0	0	0	0	0	0	0	0	0	0
0	0	0	0	0	0	0	0	0	0	0	0	0	0	0	0	0	0	0
0	0	0	0	0	0	0	0	0	0	0	0	0	0	0	0	0	0	0
5	0	0	0	0	0	0	0	0	0	0	0	0	0	0	0	0	0	3
0	0	0	0	0	0	0	0	0	0	0	0	0	0	0	0	0	0	0
0	0	0	0	0	0	0	0	0	0	0	0	0	0	0	0	0	0	0
0	0	0	0	0	0	0	0	0	0	0	0	0	0	0	0	0	0	0
0	0	0	0	0	0	0	0	0	0	4	0	0	0	0	0	0	0	0
0	0	0	0	0	0	0	0	0	0	0	0	0	0	7	0	0	0	0
0	0	0	0	0	0	0	0	0	0	0	0	0	0	0	0	0	0	0
0	0	0	0	0	0	0	0	0	0	0	0	0	0	0	0	0	0	0
0	0	0	0	0	0	0	0	0	0	0	0	0	0	0	0	0	0	0
0	0	0	0	0	0	0	0	0	0	0	0	0	0	0	0	0	0	0
0	0	0	0	0	0	0	0	0	0	0	0	0	0	0	0	0	0	0
0	0	0	0	0	0	0	0	0	0	0	0	0	0	0	0	0	0	0
0	0	0	0	0	0	0	0	0	0	0	0	0	0	0	0	0	0	0
0	0	0	0	0	0	0	0	0	0	0	0	0	0	0	0	0	0	0
0	0	0	0	0	0	0	0	0	0	0	0	0	0	0	0	0	0	0
0	0	0	0	0	0	0	0	0	0	0	0	0	0	0	0	0	0	0
0	0	0	0	0	0	0	0	0	0	0	0	0	0	0	0	0	0	0
0	0	0	0	0	0	0	0	0	0	0	0	0	0	0	0	0	0	0
0	0	0	0	0	0	0	0	0	0	0	0	0	0	0	0	0	0	0
0	0	0	0	0	0	0	0	8	0	0	0	0	0	0	0	0	0	0
0	0	0	0	0	0	0	0	0	0	0	0	0	0	0	0	0	0	0
0	0	0	0	0	0	0	0	0	0	0	0	0	0	0	0	0	0	0
0	0	0	0	0	0	0	0	0	0	0	0	0	0	0	0	0	0	0
0	0	0	0	0	0	0	0	0	0	0	0	0	0	0	0	0	0	0
0	0	0	0	0	0	0	0	0	0	0	0	0	0	0	0	0	0	0
0	0	0	0	0	0	0	0	0	0	0	0	0	0	0	0	0	0	0
0	0	0	0	0	0	0	0	0	0	0	0	0	0	0	0	0	0	0
0	0	0	0	0	0	0	0	0	0	0	0	0	0	0	0	0	0	0
0	0	0	0	0	0	0	0	0	0	0	0	0	0	0	0	0	0	0
0	0	0	0	0	0	0	0	0	0	0	0	0	0	0	0	0	0	0
0	0	0	0	0	0	0	0	0	0	0	0	0	0	0	0	0	0	0
0	0	0	0	0	0	0	0	0	0	0	0	0	0	0	0	0	0	0
0	0	0	0	0	0	0	0	0	0	0	0	0	0	0	0	0	0	0

0	0	0	0	0	0	0	0	0	0	0	0	0	0	0	0	0	0	0
0	0	0	0	0	0	0	0	0	0	0	0	0	0	0	0	0	0	0
0	0	0	0	0	0	0	0	0	0	0	0	0	0	0	0	0	0	0
0	0	0	0	0	0	0	0	0	0	0	0	0	0	0	0	0	0	0
0	0	0	0	0	0	0	0	0	0	0	0	0	0	0	0	0	0	0
0	0	0	0	0	0	0	0	0	0	0	0	0	0	0	0	0	0	0
0	0	0	0	0	0	0	0	0	0	0	0	0	0	0	0	9	9	0
0	0	0	0	0	0	0	0	0	0	0	0	0	0	0	0	0	0	0
0	0	0	0	0	0	0	0	0	0	0	0	0	0	0	0	0	0	0
0	0	0	0	0	0	0	0	0	0	0	0	0	0	0	0	0	0	0
0	0	0	0	0	0	0	0	0	0	0	0	0	0	0	0	0	8	8
0	0	0	0	0	0	0	0	0	0	0	0	0	0	0	0	0	0	0
0	0	0	0	0	0	0	0	0	0	0	0	0	0	0	0	0	0	0
0	0	0	0	0	0	0	0	0	0	0	0	0	0	0	0	0	0	0
0	0	0	0	0	0	0	0	0	0	0	0	0	0	0	0	0	0	0
0	0	0	0	0	0	0	0	0	0	0	0	0	0	0	0	0	0	0
0	0	0	0	0	0	0	0	0	7	0	0	0	0	0	0	0	0	0
0	0	0	0	0	0	0	0	0	0	0	0	0	0	0	0	0	0	0
0	0	0	0	0	0	0	0	0	0	0	0	0	0	0	0	0	0	0
0	0	0	0	0	0	0	0	0	0	0	0	0	0	0	0	0	0	0
0	0	0	0	0	0	0	0	0	0	0	0	0	0	0	0	0	0	0
0	0	0	0	0	0	0	0	0	0	0	0	0	0	0	0	0	0	0
0	0	0	0	0	0	0	0	0	0	0	0	0	0	0	0	0	0	0
6	6	0	0	0	0	0	0	0	0	0	0	0	0	0	0	0	0	0
0	0	0	0	0	0	0	0	0	0	0	0	0	0	0	0	0	0	0
0	0	0	0	0	0	0	0	0	0	0	0	0	0	0	0	0	0	0
0	0	0	0	0	0	0	0	0	0	0	0	0	0	0	0	0	0	0
0	0	0	0	0	0	0	0	0	0	0	0	0	0	0	0	0	0	0
0	0	0	0	0	0	0	0	0	0	0	0	0	0	0	0	0	0	0
0	0	0	0	0	0	0	0	0	0	0	0	0	0	0	0	0	0	0
0	0	0	0	0	0	0	0	0	0	0	0	0	0	0	0	0	0	0
0	0	0	0	0	0	0	0	0	0	0	5	0	0	0	0	0	0	0
0	0	0	0	0	0	0	0	0	0	0	0	0	0	0	0	0	0	0
0	0	0	0	0	0	0	0	0	0	0	0	0	0	0	0	0	0	0
0	0	0	0	0	0	0	0	0	0	0	0	0	0	0	0	0	0	0
0	0	0	0	0	0	0	0	0	0	0	0	0	0	0	0	0	0	0
0	0	0	0	0	0	0	0	0	0	0	0	0	0	0	0	0	0	0

0	0	0	0	0	0	0	0	0	0	0	0	0	0	0	0	0	0	0
0	0	0	0	0	0	0	0	0	0	0	0	0	0	0	0	0	0	0
0	0	0	0	0	0	0	0	0	0	0	0	0	0	0	0	0	0	0
0	0	0	0	0	0	0	0	0	0	0	0	0	0	0	0	0	0	0
0	0	0	0	0	0	0	0	0	0	0	0	0	0	0	0	0	0	0
0	0	0	0	0	0	0	0	0	0	0	0	0	0	0	0	0	0	0
0	0	0	0	0	0	0	0	0	0	0	0	0	0	0	0	0	0	0
0	0	0	0	0	0	0	0	0	0	0	0	0	0	0	0	0	0	0
0	0	0	0	0	0	0	0	0	0	0	0	0	0	0	0	0	0	0
0	0	0	0	0	0	0	0	0	0	0	0	0	0	0	0	0	0	0
0	0	0	0	0	0	0	0	0	0	0	0	0	0	0	0	0	0	0
0	0	0	0	0	0	0	0	0	0	0	0	0	0	0	0	0	0	0
0	0	0	0	0	0	0	0	0	0	0	0	0	0	0	0	0	0	0
0	0	0	0	0	0	0	0	0	0	0	0	0	0	0	0	0	0	0
0	0	0	0	0	0	0	0	0	0	0	0	0	0	0	0	0	0	0
0	0	0	0	0	0	0	0	0	0	0	0	0	0	0	0	0	0	0
0	0	0	0	0	0	0	0	0	0	0	0	0	0	0	0	0	0	0
0	0	0	0	0	0	0	0	0	0	0	0	0	0	0	0	0	0	0
0	0	0	0	0	0	0	0	0	0	0	0	0	0	0	0	0	0	0
0	0	0	0	0	0	0	0	0	0	0	0	0	0	0	0	0	0	0
0	0	0	0	0	0	0	0	0	0	0	0	0	0	0	0	0	0	0
0	0	0	0	0	0	0	0	0	0	0	0	0	0	0	0	0	0	0
0	0	0	0	0	0	0	0	0	0	0	0	0	0	0	0	0	0	0
0	0	0	0	0	0	0	0	0	0	0	0	0	0	0	0	0	0	0
0	0	0	0	0	0	0	0	0	0	0	0	0	0	0	0	0	0	0
0	0	0	0	0	0	0	0	0	0	0	0	0	0	0	0	0	0	0
0	0	0	0	0	0	0	0	0	0	0	0	0	0	0	0	0	0	0
0	0	0	0	0	0	0	0	0	0	0	0	0	0	0	0	0	0	0
0	0	0	0	0	0	0	0	0	0	0	0	0	0	0	0	0	0	0
0	0	0	0	0	0	0	0	0	0	0	0	0	0	0	0	0	0	0
0	0	0	0	0	0	0	0	0	0	0	0	0	0	0	0	0	0	0
0	0	0	0	0	0	0	0	0	0	0	0	0	0	0	0	0	0	0
0	0	0	0	0	0	0	0	0	0	0	0	0	0	0	0	0	0	0
0	0	0	0	0	0	0	0	0	0	0	0	0	0	0	0	0	0	0
0	0	0	0	0	0	0	0	0	0	0	0	0	0	0	0	0	0	0
0	0	0	0	0	0	0	0	0	0	0	0	0	0	0	0	0	0	0
0	0	0	0	0	0	0	0	0	0	0	0	0	0	0	0	0	0	0
0	0	0	0	0	0	0	0	0	0	0	0	0	0	0	0	0	0	0

0	0	0	0	0	0	0	0	0	0	0	0	0	0	0	0	0	0	0
0	0	0	0	0	0	0	0	0	0	0	0	0	0	0	0	0	0	0
0	0	0	0	0	0	0	0	0	0	0	0	0	0	0	0	0	0	0
0	0	0	0	0	0	0	0	0	0	0	0	0	0	0	0	0	0	0
0	0	0	0	0	0	0	0	0	0	0	0	0	0	0	0	0	0	0
0	0	0	0	0	0	0	0	0	0	0	0	0	0	0	0	0	0	0
0	0	0	0	0	0	0	0	0	0	0	0	0	0	0	0	0	0	0
0	0	0	0	0	0	0	0	0	0	0	0	0	0	0	0	0	0	0
0	0	0	0	0	0	0	0	0	0	0	0	0	0	0	0	0	0	0
0	0	0	0	0	0	0	0	0	0	0	0	0	0	0	0	0	0	0
0	0	0	0	0	0	0	0	0	0	0	0	0	0	0	0	0	0	0
0	0	0	0	0	0	0	0	0	0	0	0	0	0	0	0	0	0	0
0	0	2	2	2	0	0	0	0	0	0	0	0	0	0	0	0	0	0
0	0	0	0	0	0	0	0	0	0	0	0	0	0	0	0	0	0	0
0	0	0	0	0	0	0	0	0	0	0	0	0	0	0	0	0	0	0
0	0	0	0	0	0	0	0	0	0	0	0	0	0	0	0	0	0	0
0	0	0	0	0	0	0	0	0	0	0	0	0	0	0	0	0	0	0
0	0	0	0	0	0	0	0	0	0	0	0	0	0	0	0	0	0	0
0	0	0	0	0	0	0	0	0	0	0	0	0	0	0	0	0	0	0
0	0	0	0	0	0	0	0	0	0	0	0	0	0	0	0	0	0	0
0	0	0	0	0	0	0	0	0	0	0	0	0	0	0	0	0	0	0
0	0	0	0	0	0	0	0	0	0	0	0	0	0	0	0	0	0	0
0	0	0	0	0	0	0	0	0	0	0	0	0	0	0	0	0	0	0
0	0	0	0	0	0	0	0	0	0	0	0	0	0	0	0	0	0	0
0	0	0	0	0	0	0	0	0	0	0	0	0	0	0	0	0	0	0
0	0	0	0	0	0	0	0	0	0	0	0	0	0	0	0	0	0	0
0	0	0	0	0	0	0	0	0	0	0	0	0	0	0	0	0	0	0
0	0	0	0	0	0	0	0	0	0	0	0	0	0	0	0	0	0	0
0	0	0	0	0	0	0	0	0	0									
Jan_DL030	1640	922	856	437	166	314	361	412	139	656	171	230	516	326	229	232	352	239
227	0	163	0	403	139	100	136	80	286	194	268	173	76	248	81	122	67	155
0	16	117	89	126	115	103	0	110	91	70	26	0	84	52	21	53	83	128
142	7	0	14	68	0	179	74	40	80	69	71	0	173	0	0	163	0	4
30	28	42	32	87	31	37	0	65	35	0	66	56	0	36	0	0	48	0
17	63	40	0	0	36	0	0	0	0	174	0	57	0	21	0	39	0	0
0	17	0	0	21	33	0	0	0	0	18	0	22	35	23	0	68	0	0
0	0	0	0	0	0	0	18	0	6	12	0	30	0	24	6	0	0	0

0	14	8	15	0	0	0	0	6	0	0	0	33	0	0	0	38	33	11
15	0	14	0	0	0	0	14	0	11	0	0	0	0	25	0	0	0	8
0	0	0	0	0	0	0	11	0	5	26	0	0	0	0	0	0	0	0
0	0	0	0	12	0	0	0	0	0	0	0	0	0	0	0	60	0	0
0	0	21	0	0	21	0	0	0	0	0	0	6	0	0	17	18	0	30
0	0	0	4	0	0	0	0	0	0	0	0	0	0	0	4	0	6	14
9	0	0	0	0	4	0	0	0	0	0	0	0	0	6	0	0	0	0
0	13	0	0	0	0	0	0	0	0	0	0	0	0	0	29	0	0	0
0	0	0	0	0	8	0	0	0	14	10	0	0	0	0	0	0	0	7
0	0	0	0	0	0	19	0	0	0	0	0	0	0	19	6	0	0	0
0	3	0	15	5	0	0	0	0	0	0	0	6	18	0	0	0	0	0
0	0	0	0	0	0	0	0	0	0	0	0	5	0	0	0	0	0	12
0	0	0	0	0	0	0	0	0	0	0	0	0	0	0	0	0	0	0
0	0	0	0	0	0	0	0	0	0	0	0	6	0	0	0	0	0	0
0	0	0	0	0	0	0	0	0	0	0	0	0	0	0	0	0	0	0
0	0	0	0	0	0	0	0	0	0	0	0	17	0	0	0	0	4	0
0	0	0	0	0	0	0	0	0	0	0	0	0	0	0	0	0	0	0
0	0	0	0	0	0	0	7	0	5	0	0	0	0	0	2	0	0	0
0	0	0	15	0	0	0	0	0	0	0	0	0	0	0	0	13	0	0
16	0	0	0	57	0	0	0	0	0	0	0	0	0	0	0	0	0	0
0	0	0	0	0	0	0	0	0	0	0	0	0	0	0	0	0	0	0
0	0	0	0	7	0	0	36	0	0	0	0	0	0	0	0	0	0	0
0	0	0	0	0	0	0	0	0	0	0	0	0	3	0	0	0	0	0
0	0	0	0	0	0	0	0	0	6	0	0	0	0	0	0	0	0	0
0	0	0	0	0	0	0	0	11	0	0	0	0	0	0	0	0	0	0
0	0	0	0	0	0	0	0	0	0	0	0	0	0	0	0	0	0	0
0	0	0	0	0	0	0	0	0	0	0	0	0	0	0	0	0	0	0
0	0	0	0	0	0	0	0	0	0	0	0	0	0	0	0	0	0	0
0	0	0	0	0	0	0	0	0	0	0	0	0	0	0	0	0	0	0
0	0	0	0	0	0	0	0	11	0	0	0	0	0	0	0	0	0	0
0	0	0	0	0	0	0	0	0	0	0	0	0	0	0	0	0	0	0
0	0	0	7	0	0	0	0	0	0	0	0	0	0	0	0	0	0	0
0	0	0	0	0	0	0	0	0	0	0	0	0	0	0	0	0	0	0
0	0	0	0	0	0	0	0	0	0	0	0	0	0	0	0	0	0	0
0	0	0	0	0	0	0	0	0	0	0	0	0	0	0	0	0	0	0
0	0	0	0	0	0	0	0	0	0	0	0	0	0	0	0	0	0	0
0	0	0	0	0	0	0	0	0	0	0	0	0	0	0	0	0	0	0

0	0	0	0	0	0	0	0	0	0	0	0	0	0	0	0	0	0	0
0	0	0	0	0	0	0	0	0	0	0	0	0	0	0	0	0	0	0
0	0	0	0	0	0	0	0	0	0	0	0	0	0	0	0	0	0	0
0	0	0	0	0	0	0	0	0	0	0	0	0	0	0	0	0	0	0
0	0	4	0	0	0	0	0	0	0	0	0	0	0	0	0	0	0	0
0	0	0	0	0	0	0	0	0	0	0	0	0	0	0	0	0	0	0
0	0	0	0	0	0	0	0	0	0	0	8	0	0	0	0	0	0	0
0	0	0	0	0	0	0	0	0	0	0	0	0	0	0	0	0	0	0
0	0	0	0	0	0	0	0	0	0	0	0	0	0	0	0	0	0	0
0	0	0	0	0	0	0	0	0	0	0	0	0	0	0	0	0	0	0
0	0	0	0	0	0	0	0	0	0	0	0	0	0	0	0	0	0	0
0	0	0	0	0	0	0	0	0	0	0	0	0	0	0	0	0	0	0
0	0	0	0	0	0	0	0	0	0	0	0	0	0	0	0	0	3	0
0	0	0	0	0	0	0	0	0	0	0	0	0	0	0	0	0	0	0
0	0	0	0	0	0	0	0	0	0	0	0	0	0	0	0	0	0	0
0	0	0	0	0	0	0	0	0	0	0	0	0	0	0	0	0	0	0
0	0	0	0	0	0	0	0	0	0	0	0	0	0	0	0	0	0	0
0	0	0	0	0	0	0	0	0	0	0	0	0	0	0	0	0	0	0
0	0	0	0	0	0	0	0	0	0	0	0	0	0	0	0	0	0	0
0	0	0	0	0	0	0	0	0	0	0	0	0	0	0	0	0	0	0
0	0	0	0	0	0	0	0	0	0	0	0	0	0	0	0	0	0	0
0	0	0	0	0	0	0	0	0	0	0	0	0	0	0	0	0	0	0
0	0	0	0	0	0	0	0	0	0	0	0	0	0	0	0	0	0	0
0	0	0	0	0	0	0	0	0	0	0	0	0	0	0	0	0	0	0
0	0	0	0	0	0	0	0	0	0	0	0	0	0	0	0	0	0	0
0	0	0	0	0	0	0	0	0	0	0	0	0	0	0	0	0	0	0
0	0	0	0	0	0	0	0	0	0	0	0	0	0	0	0	0	0	0
0	0	0	0	0	0	0	0	0	0	0	0	0	0	0	0	0	0	0
0	0	0	0	0	0	0	0	0	0	0	0	0	0	0	0	0	0	0
0	0	0	0	0	0	0	0	0	0	0	0	0	0	0	0	0	0	0
0	0	0	0	0	0	0	0	0	0	0	0	0	0	0	0	0	0	0
0	0	0	0	0	0	0	0	0	0	0	0	0	0	0	0	0	0	0
0	0	0	0	0	0	0	0	0	0	0	0	0	0	0	0	0	0	0
0	0	0	0	0	0	0	0	0	0	0	0	0	0	0	0	0	0	0
0	0	0	0	0	0	0	0	0	0	0	0	0	0	0	0	0	0	0
0	0	0	0	0	0	0	0	0	0	0	0	0	0	0	0	0	0	0
0	0	0	0	0	0	0	0	0	0	0	0	0	0	0	0	0	0	0

0	0	0	0	0	0	0	0	0	0	0	0	0	0	0	0	0	0	0
0	0	0	0	0	0	0	0	0	0	0	0	0	0	0	0	0	0	0
0	0	0	0	0	0	0	0	0	0	0	0	0	0	0	0	0	0	0
0	0	0	0	0	0	0	0	0	0	0	0	0	0	0	0	0	0	0
0	0	0	0	0	0	0	0	0	0	0	0	0	0	0	0	0	0	0
0	0	0	0	0	0	0	0	0	0	0	0	0	0	0	0	0	0	0
7	0	0	0	0	0	0	0	0	0	0	0	0	0	0	0	0	0	0
0	0	0	0	0	0	0	0	0	0	0	0	0	0	0	0	0	0	0
0	0	0	0	0	0	0	0	0	0	0	0	0	0	0	0	0	0	0
0	0	0	0	0	0	0	0	0	0	0	0	0	0	0	0	0	0	0
0	0	0	0	0	0	0	0	0	0	0	0	0	0	0	0	0	0	0
0	0	0	0	0	0	0	0	0	0	0	0	0	0	0	0	0	0	0
0	0	0	0	0	0	0	0	0	0	0	0	0	0	0	0	0	0	0
0	0	0	0	0	0	0	0	0	0	0	0	0	0	0	0	0	0	0
0	0	0	0	0	0	0	0	0	0	0	0	0	0	0	0	0	0	0
0	0	0	0	0	0	0	0	0	0	0	0	0	0	0	0	0	0	0
0	0	0	0	0	0	0	0	0	0	0	0	0	0	0	0	0	0	0
0	0	0	0	0	0	0	0	0	0	0	0	0	0	0	0	0	0	0
0	0	0	0	0	0	0	0	0	0	0	0	0	0	0	0	0	0	0
0	0	0	0	0	0	0	0	0	0	0	0	0	0	0	0	0	0	0
0	0	0	0	0	0	0	0	0	0	0	0	0	0	0	0	0	0	0
0	0	0	0	0	0	0	0	0	0	0	0	0	0	0	0	0	0	0
0	0	0	0	0	0	0	0	0	0	0	0	0	0	0	0	0	0	0
0	0	0	0	0	0	0	0	0	0	0	0	0	0	0	0	0	0	0
0	0	0	0	0	0	0	0	0	0	0	0	0	0	0	0	0	0	0
0	0	0	0	0	0	0	0	0	0	0	0	0	0	0	0	0	0	0
0	0	0	0	0	0	0	0	0	0	0	0	0	0	0	0	0	0	0
0	0	0	0	0	0	0	0	0	0	0	0	0	0	0	0	0	0	0
0	0	0	0	0	0	0	0	0	0	0	0	0	0	0	0	0	0	0
0	0	0	0	0	0	0	0	0	0	0	0	0	0	0	0	0	0	0
0	0	0	0	0	0	0	0	0	0	0	0	0	0	0	0	4	0	0
0	0	0	0	0	0	0	0	0	0	0	0	0	0	0	0	0	0	0
0	0	0	0	0	0	0	0	0	0	0	0	0	0	0	0	0	0	0
0	0	0	0	0	0	0	0	0	0	0	0	0	0	0	0	0	0	0
0	0	0	0	0	0	0	0	0	0	0	0	0	0	0	0	0	0	0
0	0	0	0	0	0	0	0	0	0	0	0	0	0	0	0	0	0	0
0	0	0	0	0	0	0	0	0	0	0	0	0	0	0	0	0	0	0

0	0	0	0	0	0	0	0	0	0	0	0	0	0	0	0	0	0	0
0	0	0	0	0	0	0	0	0	0	0	0	0	0	0	0	0	0	0
0	0	0	0	0	0	0	0	0	0	0	0	0	0	0	0	0	0	0
0	0	0	0	0	0	0	0	0	0	0	0	0	0	0	0	0	0	0
0	0	0	0	0	0	0	0	0	0	0	0	0	0	0	0	0	0	0
0	0	0	0	0	0	0	0	0	0	0	0	0	0	0	0	0	0	0
0	0	0	0	0	0	0	0	0	0	0	0	0	0	0	0	0	0	0
0	0	0	0	0	0	0	0	0	0	0	0	0	0	0	0	0	0	0
0	0	0	0	0	0	0	0	0	0	0	0	0	0	0	0	0	0	0
0	0	0	0	0	0	0	0	0	0	0	0	0	0	0	0	0	0	0
0	0	0	0	0	0	0	0	0	0	0	0	0	3	0	0	0	0	0
0	0	0	0	0	0	0	0	0	0	0	0	0	0	0	0	0	0	0
0	0	0	0	0	0	0	0	0	0	0	0	0	0	0	0	0	0	0
0	0	0	0	0	0	0	0	0	0	0	0	0	0	0	0	0	0	0
0	0	0	0	0	0	0	0	0	0	0	0	0	0	0	0	0	0	0
0	0	0	0	0	0	0	0	0	0	0	0	0	0	0	0	0	0	0
0	0	0	0	0	0	0	0	0	0	0	0	0	0	0	0	0	0	0
0	0	0	0	0	0	0	0	0	0	0	0	0	0	0	0	0	0	0
0	0	0	0	0	0	0	0	0	0	0	0	0	0	0	0	0	0	0
0	0	0	0	0	0	0	0	0	0	0	0	0	0	0	0	0	0	0
0	0	0	0	0	0	0	0	0	0	0	0	0	0	0	0	0	0	0
0	0	0	0	0	0	0	0	0	0	0	0	0	0	0	0	0	0	0
0	0	0	0	0	0	0	0	0	0	0	0	0	0	0	0	0	0	0
0	0	0	0	0	0	0	0	0	0	0	0	0	0	0	0	0	0	0
0	0	0	0	0	0	0	0	0	0	0	0	0	0	0	0	0	0	0
0	0	0	0	0	0	0	0	0	0	0	0	0	0	0	0	0	0	0
0	0	0	0	0	0	0	0	0	0	0	0	0	0	0	0	0	0	0
0	0	0	0	0	0	0	0	0	0	0	0	0	0	0	0	0	0	0
0	0	0	0	0	0	0	0	0	0	0	0	0	0	0	0	0	0	0
0	0	0	0	0	0	0	0	0	0	0	0	0	0	0	0	0	0	0
0	0	0	0	0	0	0	0	0	0	0	0	0	0	0	0	0	0	0
0	0	0	0	0	0	0	0	0	0	0	0	0	0	0	0	0	0	0
0	0	0	0	0	0	0	0	0	0	0	0	0	0	0	0	0	0	0
0	0	0	0	0	0	0	0	0	0	0	0	0	0	0	0	0	0	0
0	0	0	0	0	0	0	0	0	0	0	0	0	0	0	0	0	0	0
0	0	0	0	0	0	0	0	0	0	0	0	0	0	0	0	0	0	0
0	0	0	0	0	0	0	0	0	0	0	0	0	0	0	0	0	0	0

0	0	0	0	0	0	0	0	0	0	0	0	0	0	0	0	0	0	0
0	0	0	0	0	0	0	2	0	0	0	0	0	0	0	0	0	0	0
0	0	0	0	0	0	0	0	0	0	0	0	0	0	0	0	0	0	0
0	0	0	0	0	0	0	0	0	0	0	0	0	0	0	0	0	0	0
0	0	0	0	0	0	0	0	0	0	0	0	0	0	0	0	0	0	0
0	0	0	0	0	0	0	0	0	0	0	0	0	0	0	0	0	0	0
0	0	0	0	0	0	0	0	0	0	0	0	0	0	0	0	0	0	0
0	0	0	0	0	0	0	0	0	0	0	0	0	0	0	0	0	0	0
0	0	0	0	0	0	0	0	0	0	0	0	0	0	0	0	0	0	0
0	0	0	0	0	0	0	0	0	0	0	0	0	0	0	0	0	0	0
0	0	0	0	0	0	0	0	0	0	0	0	0	0	0	0	0	0	0
0	0	0	0	0	0	0	0	0	0	0	0	0	0	0	0	0	0	0
0	0	0	0	0	0	0	0	0	0	0	0	0	0	0	0	0	0	0
0	0	0	0	0	0	0	0	0	0	0	0	0	0	0	0	0	0	0
0	0	0	0	0	0	0	0	0	0	0	0	0	0	0	0	0	0	0
0	0	0	0	0	0	0	0	0	0	0	0	0	0	0	0	0	0	0
0	0	0	0	0	0	0	0	0	0	0	0	0	0	0	0	0	0	0
0	0	0	0	0	0	0	0	0	0	0	0	0	0	0	0	0	0	0
0	0	0	0	0	0	0	0	0	0	0	0	0	0	0	0	0	0	0
0	0	0	0	0	0	0	0	0	0	0	0	0	0	0	0	0	0	0
0	0	0	0	0	0	0	0	0	0	0	0	0	0	0	0	0	0	0
0	0	0	0	0	0	0	0	0	0									
Jan_DL031	302	1729	296	205	2522	242	341	272	46	67	66	63	48	95	469	29	112	121
88	339	369	0	79	300	27	85	36	25	137	0	10	49	31	0	63	0	0
0	183	0	89	52	46	54	0	76	11	0	0	274	0	0	0	0	33	0
0	11	0	88	66	27	24	29	20	69	20	50	137	0	95	55	78	12	35
0	0	0	33	11	0	11	0	133	0	0	84	55	99	31	0	63	44	0
10	31	0	170	0	83	0	222	0	4	46	0	44	66	0	92	0	0	199
0	87	0	0	0	27	0	0	0	0	77	53	40	12	0	209	0	0	94
0	0	0	17	30	0	24	50	0	15	222	0	10	0	20	0	0	0	0
0	0	79	11	0	17	0	0	0	21	33	0	0	0	41	0	7	57	31
0	0	10	0	36	14	0	6	0	0	0	0	0	0	0	77	25	22	0
0	0	0	61	0	6	0	0	0	92	0	0	0	0	0	0	0	37	0
20	0	0	0	0	0	25	0	0	0	93	23	0	50	0	0	0	13	14
0	0	41	8	0	0	0	0	0	4	0	0	0	0	0	15	45	0	0
17	0	0	8	0	0	0	16	0	25	0	0	0	15	8	15	17	0	19
0	0	0	0	0	0	0	0	0	0	0	0	0	35	5	8	26	0	0
0	13	8	53	0	0	0	0	12	0	0	0	0	0	12	0	0	0	0

0	5	0	0	0	0	0	0	0	28	0	0	0	26	0	0	0	6	0
27	0	13	0	0	0	10	0	8	17	0	0	0	22	0	0	0	0	0
0	0	0	0	0	0	0	25	11	0	0	10	0	0	92	0	7	0	0
0	0	6	0	0	0	0	0	0	17	0	0	0	0	0	0	0	0	0
0	0	0	0	0	7	0	0	0	0	0	0	9	0	6	0	0	18	0
0	0	0	0	0	0	0	23	0	0	0	0	0	0	0	0	0	0	0
0	0	0	0	0	0	21	0	0	0	0	0	0	0	0	0	0	0	0
0	0	0	0	0	0	9	26	0	0	0	0	0	0	0	0	8	11	0
0	7	0	0	16	0	0	36	0	0	0	0	0	0	0	0	0	0	36
0	0	37	0	0	0	0	0	0	8	0	0	0	0	0	0	0	24	0
6	0	21	0	0	0	0	0	0	0	0	0	0	0	0	0	15	7	17
0	8	0	0	0	0	0	0	0	0	0	0	0	0	0	0	21	0	0
0	0	0	0	0	0	0	0	0	0	0	0	0	0	0	0	0	0	0
0	0	0	0	0	0	0	0	65	0	0	0	0	0	0	0	0	0	0
0	0	0	0	0	0	0	0	0	0	0	0	0	6	0	0	0	0	0
0	0	0	0	0	0	0	0	17	0	0	0	0	0	0	0	0	0	0
0	0	0	0	9	15	0	0	0	0	0	0	0	12	0	0	0	0	0
0	0	0	0	10	16	0	0	0	0	0	0	0	0	0	0	0	0	0
0	0	0	0	0	0	0	22	0	0	0	0	0	0	0	0	0	0	0
0	44	0	0	0	69	0	0	0	0	11	0	0	0	0	0	0	0	0
0	0	0	0	0	0	0	0	0	0	0	0	0	0	0	0	0	0	0
0	0	0	0	0	0	0	0	17	0	0	0	0	0	0	0	0	0	0
0	0	0	0	0	0	0	0	0	0	0	0	4	0	0	0	0	0	0
0	0	0	10	0	0	0	0	0	0	7	14	0	0	0	0	0	0	0
0	0	0	0	0	0	20	0	0	0	0	0	0	0	0	0	0	13	0
0	0	0	0	0	0	0	0	0	0	0	0	0	0	0	0	0	0	0
0	8	0	0	12	0	0	0	0	7	0	0	0	0	0	0	0	0	0
0	0	0	0	0	0	0	0	0	0	0	0	0	0	0	0	0	0	0
0	0	0	0	0	0	0	0	0	0	0	0	0	0	0	0	0	0	0
0	0	0	0	13	0	4	0	0	0	0	0	0	0	0	0	0	0	0
0	0	0	0	0	0	0	0	0	0	0	0	0	0	0	0	0	0	0
0	0	0	0	0	0	0	0	0	0	0	0	0	0	0	0	0	0	0
0	13	0	0	0	0	0	0	0	0	0	0	0	0	0	0	0	0	0
0	0	0	0	0	0	0	0	0	0	0	0	0	0	0	0	0	0	0
0	0	0	0	0	0	0	0	22	7	0	0	0	0	0	0	0	0	0
0	0	0	0	0	0	0	0	0	0	0	0	0	0	0	0	0	0	0
0	0	0	0	0	0	0	0	0	0	0	0	0	0	0	0	0	0	0

0	0	0	0	0	0	0	0	0	0	0	0	0	0	0	0	0	0	0
0	0	0	0	0	0	0	0	0	0	0	0	0	0	15	0	0	0	0
0	0	0	0	0	0	0	0	0	0	0	0	0	0	0	0	0	0	0
0	0	0	0	0	0	0	0	0	0	0	0	0	0	0	0	0	0	0
0	0	0	0	0	0	0	0	0	0	0	0	0	0	0	0	0	0	0
0	6	0	0	0	0	0	0	0	0	0	0	0	0	0	0	0	0	0
0	0	0	0	0	0	0	0	0	0	0	0	0	0	0	0	0	0	0
0	0	0	0	0	0	0	0	0	0	0	0	0	0	0	0	0	0	0
0	0	0	0	0	0	0	0	2	0	0	0	0	0	0	0	0	0	0
0	0	0	0	0	0	0	0	0	0	0	0	0	0	0	0	0	0	0
0	0	0	0	0	0	0	0	0	0	0	0	0	0	0	0	0	0	0
0	0	0	0	0	0	0	0	0	0	0	0	0	0	0	0	0	0	0
0	3	0	0	0	0	0	0	0	0	0	0	0	0	0	0	0	0	0
0	0	0	0	0	0	0	0	0	0	0	0	0	0	0	0	0	0	0
0	0	0	0	0	0	0	0	0	0	0	0	0	0	0	0	0	0	0
0	0	0	0	0	0	0	0	0	0	0	0	0	0	0	0	0	0	0
0	0	0	0	0	0	0	0	0	0	0	0	0	0	0	0	0	0	0
0	0	0	0	0	0	0	0	0	0	0	0	0	0	0	0	0	0	0
0	0	0	0	0	0	0	0	0	0	0	0	0	0	0	0	0	0	0
0	0	0	0	0	0	0	0	0	0	0	0	0	0	0	0	0	0	0
0	0	0	11	0	0	0	0	0	0	0	0	0	0	0	0	0	0	0
3	0	0	0	0	0	0	0	0	0	0	0	0	0	0	0	0	0	0
0	0	0	0	0	0	0	0	0	0	0	0	0	0	0	0	0	0	0
0	0	0	0	0	0	0	0	0	0	0	0	0	0	0	0	0	0	0
0	0	0	0	0	0	0	0	0	0	0	0	0	0	0	0	0	0	0
0	0	0	0	0	0	0	0	0	0	9	0	0	0	0	0	0	0	0
0	0	0	0	0	0	0	0	2	0	0	0	0	0	0	0	0	0	0
0	0	0	0	0	0	0	0	0	0	0	0	0	0	0	0	0	0	0
0	0	0	0	0	0	0	0	0	0	0	0	0	0	0	0	0	0	0
8	0	0	0	0	0	0	0	0	0	0	0	0	0	0	0	0	0	0
0	0	0	0	0	0	0	0	0	0	0	0	0	0	0	0	0	0	0
0	0	0	0	0	0	0	0	0	0	0	0	0	0	0	0	0	0	0
0	0	0	0	0	0	0	0	0	0	0	0	0	0	0	0	0	0	0
0	0	0	0	0	0	0	0	0	0	0	0	0	0	0	0	0	0	0
0	0	0	0	0	7	0	0	0	0	0	0	0	0	0	0	0	0	0
0	0	0	0	0	0	0	0	0	0	0	0	0	0	0	0	0	0	0
0	0	0	0	0	0	0	0	0	0	0	0	0	0	0	0	0	0	0

0	0	0	0	0	0	0	0	0	0	0	0	0	0	0	0	0	0	0
0	0	0	0	0	0	0	0	0	0	0	0	0	0	0	0	0	0	0
0	0	0	0	0	0	0	0	0	0	0	0	0	0	0	0	0	0	0
0	0	0	0	0	0	0	0	0	0	0	0	0	0	0	0	0	0	0
0	0	0	0	0	0	0	0	0	0	0	0	0	0	0	0	0	0	0
0	0	0	0	0	0	0	0	0	0	0	0	0	0	0	0	0	0	0
0	0	0	0	0	0	0	0	0	0	0	0	0	0	0	0	0	0	0
0	0	0	0	0	0	0	0	0	0	0	0	0	0	0	0	0	0	0
0	0	0	0	0	0	0	0	0	0	0	0	0	0	0	0	0	0	0
0	0	0	0	0	0	0	0	0	0	0	0	0	0	0	0	0	0	0
0	0	0	0	0	0	0	0	0	0	0	0	0	0	0	0	0	0	0
0	0	0	0	0	0	0	0	0	0	0	0	0	0	0	0	0	0	0
0	0	0	0	0	0	5	5	0	0	0	0	0	0	0	0	0	0	0
0	0	0	0	0	0	0	0	0	0	0	0	0	0	0	0	0	0	0
0	0	0	0	0	0	0	0	0	0	0	0	0	0	0	0	0	0	0
0	0	0	0	0	0	0	0	0	0	0	0	0	0	0	0	0	0	0
0	0	0	0	0	0	0	0	0	0	0	0	0	0	0	0	0	0	0
0	0	0	0	0	0	0	0	0	0	0	0	0	0	0	0	0	0	0
0	0	0	0	0	0	0	0	0	0	0	0	0	0	0	0	0	0	0
0	0	0	0	0	0	0	0	0	0	0	0	0	0	0	0	0	0	0
0	0	0	0	0	0	0	0	0	0	0	0	0	0	0	0	0	0	0
0	0	0	0	0	0	0	0	0	0	0	0	0	4	4	0	0	0	0
0	0	0	0	0	0	0	0	0	0	0	0	0	0	0	0	0	0	0
0	0	0	0	0	0	0	0	0	0	0	0	0	0	0	0	0	0	0
0	0	0	0	0	0	0	0	0	0	0	0	0	0	0	0	0	0	0
0	0	0	0	0	0	0	0	0	0	0	0	0	0	0	0	0	0	0
0	0	0	0	0	0	0	0	0	0	0	0	0	0	0	0	0	0	0
0	0	0	0	0	0	0	0	0	0	0	0	0	0	0	0	0	0	0
0	0	0	0	0	0	0	0	0	0	0	0	0	0	0	0	0	0	0
0	0	0	0	0	0	0	0	0	0	0	0	0	0	0	0	0	0	0
0	0	0	0	0	0	0	0	0	0	0	0	0	0	0	0	0	0	0
0	0	0	0	0	0	0	0	0	0	0	0	0	0	0	0	0	0	0
0	0	0	0	0	0	0	0	0	0	0	0	0	0	0	0	0	0	0
0	0	0	0	0	0	0	0	0	0	0	0	0	0	0	0	0	0	0
0	0	0	0	0	0	0	0	0	0	0	0	0	0	0	0	0	0	0
0	0	0	0	0	0	0	0	0	0	0	0	0	0	0	0	0	0	0
0	0	0	0	0	0	0	0	0	0	0	0	0	0	0	0	0	0	0

0	0	0	0	0	0	0	0	0	0	0	0	0	0	0	0	0	0	0
0	0	0	0	0	0	3	0	0	0	0	0	0	0	0	0	0	0	0
0	0	0	0	0	0	0	0	0	0	0	0	0	0	0	0	0	0	0
0	0	0	0	0	0	0	0	0	0	0	0	0	0	0	0	0	0	0
0	0	0	0	0	0	0	0	0	0	0	0	0	0	0	0	0	0	0
0	0	0	0	0	0	0	0	0	0	0	0	0	0	0	0	0	0	0
0	0	0	0	0	0	0	0	0	0	0	0	0	0	0	0	0	0	0
0	0	0	0	0	0	0	0	0	0	0	0	0	0	0	0	0	0	0
0	0	0	0	0	0	0	0	0	0	0	0	0	0	0	0	0	0	0
0	0	0	0	0	0	0	0	0	0	0	0	0	0	0	0	0	0	0
0	0	0	0	0	0	0	0	0	0	0	0	0	0	0	0	0	0	0
0	0	0	0	0	0	0	0	0	0	0	0	0	0	0	0	0	0	0
0	0	0	0	0	0	0	0	0	0	0	0	0	0	0	0	0	0	0
0	0	0	0	0	0	0	0	0	0	0	0	0	0	0	0	0	0	0
0	0	0	0	0	0	0	0	0	0	0	0	0	0	0	0	0	0	0
0	0	0	0	0	0	0	0	0	0	0	0	0	0	0	0	0	0	0
0	0	0	0	0	0	0	0	0	0	0	0	0	0	0	0	0	0	0
0	0	0	0	0	0	0	0	0	0	0	0	0	0	0	0	0	0	0
0	0	0	0	0	0	0	0	0	0	0	0	0	0	0	0	0	0	0
0	0	0	0	0	0	0	0	0	0	0	0	0	0	0	0	0	0	0
0	0	0	0	0	0	0	0	0	0	0	0	0	0	0	0	0	0	0
0	0	0	0	0	0	0	0	0	0	0	0	0	0	0	0	0	0	0
0	0	0	0	0	0	0	0	0	0	0	0	0	0	0	0	0	0	0
0	0	0	0	0	0	0	0	0	0	0	0	0	0	0	0	0	0	0
0	0	0	0	0	0	0	0	0	0	0	0	0	0	0	0	0	0	0
0	0	0	0	0	0	0	0	0	0	0	0	0	0	0	0	0	0	0
0	0	0	0	0	0	0	0	0	0	0	0	0	0	0	0	0	0	0
0	0	0	0	0	0	0	0	0	0	0	0	0	0	0	0	0	0	0
0	0	0	0	0	0	0	0	0	0	0	0	0	0	0	0	0	2	2
2	0	0	0	0	0	0	0	0	0	0	0	0	0	0	0	0	0	0
0	0	0	0	0	0	0	0	0	0	0	0	0	0	0	0	0	0	0
0	0	0	0	0	0	0	0	0	0	0	0	0	0	0	0	0	0	0
0	0	0	0	0	0	0	0	0	0	0	0	0	0	0	0	0	0	0
0	0	0	0	0	0	0	0	0	0	0	0	0	0	0	0	0	0	0
0	0	0	0	0	0	0	0	0	0	0	0	0	0	0	0	0	0	0
0	0	0	0	0	0	0	0	0	0	0	0	0	0	0	0	0	0	0
0	0	0	0	0	0	0	0	0	0	0	0	0	0	0	0	0	0	0

0	0	0	0	0	0	0	0	0	0	0	0	0	0	0	0	0	0	0
0	0	0	0	0	0	0	0	0	0	0	0	0	0	0	0	0	0	0
0	0	0	0	0	0	0	0	0	0	0	0	0	0	0	0	0	0	0
0	0	0	0	0	0	0	0	0	0	0	0	0	0	0	0	0	0	0
0	0	0	0	0	0	0	0	0	0	0	0	0	0	0	0	0	0	0
0	0	0	0	0	0	0	0	0	0	0	0	0	0	0	0	0	0	0
0	0	0	0	0	0	0	0	0	0	0	0	0	0	0	0	0	0	0
0	0	0	0	0	0	0	0	0	0	0	0	0	0	0	0	0	0	0
0	0	0	0	0	0	0	0	0	0	0	0	0	0	0	0	0	0	0
0	0	0	0	0	0	0	0	0	0	0	0	0	0	0	0	0	0	0
0	0	0	0	0	0	0	0	0	0	0	0	0	0	0	0	0	0	0
0	0	0	0	0	0	0	0	0	0	0	0	0	0	0	0	0	0	0
0	0	0	0	0	0	0	0	0	0									
Jan_DL033	1004	629	1066	409	25	316	77	70	491	257	1088	188	212	17	225	323	13	56
146	0	74	32	249	58	300	89	446	181	143	0	169	126	103	517	73	311	0
164	0	0	92	116	449	70	111	0	118	32	103	26	49	66	136	133	0	0
0	109	120	7	0	28	0	85	105	18	0	105	4	108	0	0	0	37	0
108	107	40	20	0	90	27	28	50	54	0	202	107	0	15	92	21	18	0
31	33	0	0	0	0	0	0	50	23	0	0	0	0	49	0	18	123	17
0	0	0	0	4	0	0	0	0	0	0	0	0	0	0	0	0	0	0
0	0	0	0	0	0	0	0	0	8	0	0	5	0	0	0	0	24	0
242	0	0	5	0	2	29	52	7	36	0	0	0	0	0	7	0	0	0
0	68	7	0	0	0	0	0	0	24	0	0	0	11	0	0	8	0	0
0	0	14	12	38	0	0	0	0	0	0	0	0	0	0	0	0	0	18
0	0	0	0	0	0	0	0	0	0	0	0	0	0	0	0	0	0	0
79	0	0	36	0	0	0	0	0	34	0	13	13	4	0	0	0	21	0
0	0	0	0	18	0	0	0	0	0	0	0	0	0	0	0	0	0	0
0	0	0	0	0	7	0	0	0	0	5	0	0	0	0	3	0	0	0
20	0	0	0	0	0	0	0	0	0	26	0	0	0	0	0	0	0	0
0	0	0	0	0	2	0	0	0	0	0	0	0	0	6	10	0	3	3
0	27	0	0	0	0	0	0	0	0	0	0	0	0	0	0	0	0	0
8	0	0	0	0	0	0	0	0	0	0	0	6	0	0	0	0	0	0
0	0	0	0	10	16	9	4	0	0	0	0	0	0	0	0	0	0	0
0	0	0	0	11	0	0	0	0	0	0	0	0	0	3	0	16	0	0
0	0	0	0	18	0	0	0	0	0	0	0	0	11	0	0	0	0	0
0	0	0	0	0	0	0	0	0	0	0	0	0	0	0	0	0	0	0
0	0	0	0	15	0	0	0	0	0	0	0	0	0	0	0	0	0	0

0	0	0	0	0	0	0	0	0	0	0	0	0	0	0	0	0	0	0
0	0	0	6	0	0	0	0	0	0	0	0	0	0	0	18	0	0	0
0	0	0	0	0	0	0	0	0	0	0	0	0	0	0	0	4	0	0
0	0	0	0	0	0	0	0	0	0	0	7	0	0	0	0	0	0	0
0	0	0	0	0	0	0	0	0	0	0	0	0	0	12	0	0	0	0
0	0	0	0	0	0	0	7	0	0	0	0	0	0	0	0	0	0	0
0	0	0	0	0	0	0	0	0	20	0	22	0	0	0	0	0	0	0
0	0	0	0	0	0	7	0	0	0	0	0	0	0	0	4	0	0	0
0	0	0	0	0	4	0	0	0	0	0	0	0	0	0	0	7	0	0
3	0	0	0	0	12	0	0	0	0	0	0	0	0	0	0	0	0	0
0	0	0	0	0	0	25	0	0	0	0	0	0	0	0	0	0	0	0
0	0	0	0	0	0	0	0	0	0	0	0	0	0	0	25	0	0	0
0	0	0	0	0	0	0	0	0	0	0	0	0	0	0	0	0	0	0
0	0	0	0	0	0	0	0	0	0	0	0	0	0	0	0	0	0	0
0	0	0	0	0	0	0	0	0	0	0	0	0	0	0	0	0	0	0
0	0	0	0	0	0	0	0	0	0	0	0	0	0	0	0	0	0	0
0	0	0	0	0	0	0	0	0	0	0	0	0	0	0	0	0	0	0
0	0	0	0	0	0	0	0	0	0	0	0	0	0	0	0	0	0	0
0	0	0	0	0	0	0	0	0	4	0	0	0	0	0	0	0	0	0
0	0	0	0	0	0	0	0	4	0	0	0	0	0	0	0	0	0	0
0	0	0	0	0	0	0	0	0	0	0	0	0	0	0	0	0	0	0
0	0	0	0	0	0	0	0	0	0	0	0	0	0	0	0	0	0	0
0	0	0	0	0	0	0	0	0	0	0	0	0	0	0	0	0	5	0
0	0	0	0	0	0	0	0	0	0	0	0	0	0	0	0	0	0	0
0	0	0	0	0	0	0	0	0	0	0	0	0	0	0	0	0	0	0
0	0	0	0	0	0	0	0	0	0	0	0	0	0	0	0	0	0	0
0	0	0	0	0	0	0	0	0	0	0	0	0	0	0	0	0	0	0
0	0	0	0	0	0	0	0	0	0	0	0	0	0	0	0	0	0	0
0	0	0	0	0	0	0	0	0	0	0	0	0	0	0	0	0	0	0
0	0	0	0	0	0	0	0	0	0	0	0	0	0	0	0	0	0	0
0	0	0	0	0	0	0	0	0	0	0	0	0	0	0	0	0	0	0
0	0	0	0	0	0	0	0	0	0	0	0	0	0	0	0	0	0	0
0	0	0	0	0	0	0	0	0	0	0	0	0	0	0	0	0	15	0
0	0	0	0	0	0	0	0	0	0	0	0	0	0	0	0	0	0	0
0	0	0	0	5	0	0	0	0	0	0	0	0	0	0	0	0	0	0
0	0	0	0	0	0	0	0	0	0	0	0	0	0	0	0	0	0	0
0	0	0	0	0	0	0	0	0	0	0	0	0	0	0	0	0	0	0

0	0	0	0	0	0	0	0	0	0	0	0	0	0	0	0	0	0	0
0	0	0	0	0	0	0	0	0	0	0	0	0	0	0	0	0	0	0
0	0	0	0	0	0	0	0	0	0	0	0	0	0	0	0	0	0	0
0	0	0	0	0	0	0	0	0	0	0	0	0	0	0	0	0	0	0
0	0	0	0	0	0	0	0	0	0	0	0	0	0	0	0	0	0	0
0	0	0	0	0	0	0	0	0	0	0	0	0	0	3	0	0	0	0
0	0	0	0	0	0	0	0	0	0	0	0	0	0	0	0	0	0	0
0	0	0	0	0	0	0	0	0	0	0	0	0	0	0	0	0	0	0
0	0	0	0	0	0	0	0	0	0	0	0	0	0	0	0	0	0	0
0	0	0	0	0	0	0	0	0	0	0	0	0	0	0	0	0	0	0
0	0	0	0	0	0	0	0	0	0	0	0	0	0	0	0	0	0	0
0	0	0	0	0	0	0	0	0	0	0	0	0	0	0	0	0	0	0
0	0	0	0	0	0	0	0	0	0	0	0	0	0	0	0	0	0	0
0	0	0	0	0	0	0	0	0	0	0	0	0	0	0	0	0	0	0
0	0	0	0	0	0	0	0	0	0	0	0	0	0	0	0	0	0	0
0	0	0	0	0	0	0	0	0	0	0	0	0	0	0	0	0	0	0
0	0	0	0	0	0	0	0	0	0	0	0	0	0	0	0	0	0	0
0	0	0	0	0	0	0	0	0	0	0	0	0	0	0	0	0	0	0
0	0	0	0	0	0	0	0	0	0	0	0	0	0	0	0	0	0	0
0	0	0	0	0	0	0	0	0	0	0	0	0	0	0	0	0	0	0
0	0	0	0	0	0	0	0	0	0	0	0	0	0	0	0	0	0	0
0	0	0	0	0	0	0	0	0	0	0	0	0	0	0	0	0	0	0
0	0	0	0	0	0	0	0	0	0	0	0	0	0	0	0	0	0	0
0	0	0	0	0	0	0	0	0	0	0	0	0	0	0	0	0	0	0
0	0	0	0	0	0	0	0	0	0	0	0	0	0	0	0	0	0	0
0	0	0	0	0	0	0	0	0	0	0	0	0	0	0	0	0	0	0
0	0	0	0	0	0	0	0	0	0	0	0	0	0	0	0	0	0	0
0	0	0	0	0	0	0	0	0	0	0	0	0	0	0	0	0	0	0
0	0	0	0	0	0	0	0	0	0	0	0	0	0	0	0	0	0	0
0	0	0	0	0	0	0	0	0	0	0	0	0	0	0	0	0	0	0
0	0	0	0	0	0	0	0	0	0	0	0	0	0	0	0	0	0	0
0	0	0	0	0	0	0	0	0	0	0	0	0	0	0	0	0	0	0
0	0	0	0	0	0	0	0	0	0	0	0	0	0	0	0	0	0	0
0	0	0	0	0	0	0	0	0	0	0	0	0	0	0	0	0	0	0
0	0	0	0	0	0	0	0	0	0	0	0	0	0	0	0	0	0	0
0	0	0	0	0	0	0	0	0	0	0	0	0	0	0	0	0	0	0
0	0	0	0	0	0	0	0	0	0	0	0	0	0	0	0	0	0	0

0	0	0	0	0	0	0	0	5	0	0	0	0	0	0	0	0	0	0
0	0	0	0	0	0	0	0	0	0	0	0	0	0	0	0	0	0	0
0	0	0	0	0	0	0	0	0	0	0	0	0	0	0	0	0	0	0
0	0	0	0	0	0	0	0	0	0	0	0	0	0	0	0	0	0	0
0	0	0	0	0	0	0	0	0	0	0	0	0	0	0	0	0	0	0
0	0	0	0	0	0	0	0	0	0	0	0	0	0	0	0	0	0	0
0	0	0	0	0	0	0	0	0	0	0	0	0	0	0	0	0	0	0
0	0	0	0	0	0	0	0	0	0	0	0	0	0	0	0	0	0	0
0	0	0	0	0	0	0	0	0	0	0	0	0	0	0	0	0	0	0
0	0	0	0	0	0	0	0	0	0	0	0	0	0	0	0	0	0	0
0	0	0	0	0	0	0	0	0	0	0	0	0	0	0	0	0	0	0
0	0	0	0	0	4	4	0	0	0	0	0	0	0	0	0	0	0	0
0	0	0	0	0	0	0	0	0	0	0	0	0	0	0	0	0	0	0
0	0	0	0	0	0	0	0	0	0	0	0	0	0	0	0	0	0	0
0	0	0	0	0	0	0	0	0	0	0	0	0	0	0	0	0	0	0
0	0	0	0	0	0	0	0	0	0	0	0	0	0	0	0	0	0	0
0	0	0	0	0	0	0	0	0	0	0	0	0	0	0	0	0	0	0
0	0	0	0	0	0	0	0	0	0	0	0	0	0	0	0	0	0	0
0	0	0	0	0	0	0	0	0	0	0	0	0	0	0	0	0	0	0
0	0	0	0	0	0	0	0	0	0	0	0	0	0	0	0	0	0	0
0	0	0	0	0	0	0	0	0	0	0	0	0	0	0	0	0	0	0
0	0	0	0	0	0	0	0	0	0	0	0	0	0	0	0	0	0	0
0	0	0	0	0	0	0	0	0	0	0	0	0	0	0	0	0	0	0
0	0	0	0	0	0	0	0	0	0	0	0	0	0	0	0	0	0	0
0	0	0	0	0	0	0	0	0	0	0	0	0	0	0	0	0	0	0
0	0	0	0	0	0	0	0	0	0	0	0	0	0	0	0	0	0	0
0	0	0	0	0	0	0	0	0	0	0	0	0	0	0	0	0	3	3
0	0	0	0	0	0	0	0	0	0	0	0	0	0	0	0	0	0	0
0	0	0	0	0	0	0	0	0	0	0	0	0	0	0	0	0	0	0
0	0	0	0	0	0	0	0	0	0	0	0	0	0	0	0	0	0	0
0	0	0	0	0	0	0	0	0	0	0	0	0	0	0	0	0	0	0
0	0	0	0	0	0	0	0	0	0	0	0	0	0	0	0	0	0	0
0	0	0	0	0	0	0	0	0	0	0	0	0	0	0	0	0	0	0
0	0	0	0	0	0	0	0	0	0	0	0	0	0	0	0	0	0	0
0	0	0	0	0	0	0	0	0	0	0	0	0	0	0	0	0	0	0
0	0	0	0	0	0	0	0	0	0	0	0	0	0	0	0	0	0	0
0	0	0	0	0	0	0	0	0	0	0	0	0	0	0	0	0	0	0

0	0	0	0	0	0	0	0	0	0	0	0	0	0	0	0	0	0	0
0	0	0	0	0	0	0	0	0	0	0	0	0	0	0	0	0	0	0
0	0	0	0	0	0	0	0	0	0	0	0	0	0	0	0	0	0	0
0	0	0	0	0	0	0	0	0	0	0	0	0	0	0	0	0	0	0
0	0	0	0	0	0	0	0	0	0	0	0	0	0	0	0	0	0	0
0	0	0	0	0	0	0	0	0	0	0	0	0	0	0	0	0	0	0
0	0	0	0	0	0	0	0	0	0	0	0	0	0	0	0	0	0	0
0	0	0	0	0	0	0	0	0	0	0	0	0	0	0	0	0	0	0
0	0	0	0	0	0	0	0	0	0	0	0	0	0	0	0	0	0	0
0	0	0	0	0	0	0	0	0	0	0	0	0	0	0	0	0	0	0
0	0	0	0	0	0	0	0	0	0	0	0	0	0	0	0	0	0	0
0	0	0	0	0	0	0	0	0	0	0	0	0	0	0	0	0	0	0
0	0	0	0	0	0	0	0	0	0	0	0	0	0	0	0	0	0	0
0	0	0	0	0	0	0	0	0	0	0	0	0	0	0	0	0	0	0
0	0	0	0	0	0	0	0	0	0	0	0	0	0	0	0	0	0	0
0	0	0	0	0	0	0	0	0	0	0	0	0	0	0	0	0	0	0
0	0	0	0	0	0	0	0	0	0	0	2	2	2	2	0	0	0	0
0	0	0	0	0	0	0	0	0	0	0	0	0	0	0	0	0	0	0
0	0	0	0	0	0	0	0	0	0	0	0	0	0	0	0	0	0	0
0	0	0	0	0	0	0	0	0	0	0	0	0	0	0	0	0	0	0
0	0	0	0	0	0	0	0	0	0	0	0	0	0	0	0	0	0	0
0	0	0	0	0	0	0	0	0	0	0	0	0	0	0	0	0	0	0
0	0	0	0	0	0	0	0	0	0	0	0	0	0	0	0	0	0	0
0	0	0	0	0	0	0	0	0	0	0	0	0	0	0	0	0	0	0
0	0	0	0	0	0	0	0	0	0	0	0	0	0	0	0	0	0	0
0	0	0	0	0	0	0	0	0	0	0	0	0	0	0	0	0	0	0
0	0	0	0	0	0	0	0	0	0	0	0	0	0	0	0	0	0	0
0	0	0	0	0	0	0	0	0	0	0	0	0	0	0	0	0	0	0
0	0	0	0	0	0	0	0	0	0	0	0	0	0	0	0	0	0	0
0	0	0	0	0	0	0	0	0	0	0	0	0	0	0	0	0	0	0
0	0	0	0	0	0	0	0	0	0	0	0	0	0	0	0	0	0	0
0	0	0	0	0	0	0	0	0	0	0	0	0	0	0	0	0	0	0
0	0	0	0	0	0	0	0	0	0	0	0	0	0	0	0	0	0	0
0	0	0	0	0	0	0	0	0	0	0	0	0	0	0	0	0	0	0
0	0	0	0	0	0	0	0	0	0	0	0	0	0	0	0	0	0	0
0	0	0	0	0	0	0	0	0	0	0	0	0	0	0	0	0	0	0
0	0	0	0	0	0	0	0	0	0	0	0	0	0	0	0	0	0	0

0	0	0	0	0	0	0	0	0	0	0	0	0	0	0	0	0	0	0
0	0	0	0	0	0	0	0	0	0	0	0	0	0	0	0	0	0	0
0	0	0	0	0	0	0	0	0	0	0	0	0	0	0	0	0	0	0
0	0	0	0	0	0	0	0	0	0	0	0	0	0	0	0	0	0	0
0	0	0	0	0	0	0	0	0	0									
Jan_DL034	1914	1922	679	569	44	405	175	137	409	380	249	368	330	120	113	327	147	233
163	0	96	118	102	99	238	146	263	90	136	354	140	131	153	102	137	109	239
75	28	92	196	108	121	46	224	69	117	67	240	23	83	214	171	369	55	210
195	127	195	38	55	0	26	62	38	203	110	154	0	0	80	179	23	26	0
69	180	82	73	46	71	45	74	0	45	64	49	38	52	48	200	49	50	0
19	22	0	0	39	23	81	0	29	27	0	0	0	0	20	22	37	80	0
0	0	32	14	7	0	0	0	0	59	4	0	9	0	0	5	76	0	0
0	0	55	0	0	0	83	0	56	8	0	0	11	0	0	58	0	0	0
0	19	33	0	50	4	133	0	0	0	5	0	0	0	0	0	0	0	9
29	85	11	0	24	0	0	7	0	0	0	0	0	14	0	0	0	29	0
0	0	0	0	0	11	88	0	0	10	13	0	0	0	0	0	0	23	0
0	0	0	0	21	0	0	0	0	0	0	13	0	0	0	0	0	4	0
76	0	0	0	0	0	0	0	0	0	0	0	0	6	0	0	4	0	0
0	0	0	5	0	0	0	12	0	0	76	0	0	0	0	0	0	0	0
8	0	29	15	16	9	0	0	0	0	0	0	0	0	4	0	0	91	0
10	0	0	0	0	0	0	0	23	76	0	0	15	0	0	7	0	0	0
0	0	0	0	0	0	0	0	0	0	0	0	0	0	0	0	0	0	8
0	0	0	0	0	0	0	0	21	0	0	0	0	0	0	0	0	0	0
0	11	0	0	0	0	0	0	0	0	6	0	0	0	0	0	0	0	0
11	0	0	0	0	15	0	0	0	0	0	0	0	0	0	0	42	0	0
0	0	0	0	0	7	0	0	0	0	0	0	0	0	0	0	10	0	0
0	0	0	0	0	11	7	0	39	0	15	0	0	0	0	0	0	0	0
0	0	0	0	0	0	0	0	30	0	0	46	12	0	0	0	0	0	0
0	11	0	0	0	19	0	0	0	0	0	0	0	0	0	0	0	0	0
0	0	0	57	0	0	0	0	0	0	0	0	0	0	0	0	0	0	0
0	0	0	0	0	0	0	0	0	0	0	0	8	6	0	9	0	0	2
0	0	0	0	29	0	0	0	12	11	0	0	0	0	0	0	0	0	0
0	0	0	0	0	0	0	0	0	0	0	0	0	0	0	0	0	0	0
0	0	0	0	0	0	0	0	0	0	0	0	0	0	0	0	0	0	0
0	0	0	0	0	0	0	0	0	0	0	0	0	0	0	0	0	0	0
0	0	0	0	0	0	10	0	0	0	0	0	0	0	0	0	0	0	7
0	0	0	0	0	0	0	0	0	0	0	0	0	0	19	0	0	0	0

0	0	0	0	0	0	0	0	0	0	0	0	0	0	0	0	11	0	0
0	0	0	0	0	0	0	0	0	0	0	0	0	0	0	0	0	0	0
0	0	0	0	0	0	0	0	0	0	0	0	0	0	0	0	0	0	0
0	0	0	0	0	0	0	0	0	0	0	0	0	0	0	0	0	0	0
0	0	0	0	0	0	0	0	0	0	0	0	0	0	0	0	0	0	0
0	0	0	0	0	0	0	0	0	0	0	0	0	0	0	0	0	0	0
0	0	0	0	0	0	0	0	0	0	0	0	0	0	0	0	0	0	0
0	0	0	0	0	0	5	0	0	0	0	0	0	0	0	0	8	0	0
0	0	0	0	0	0	0	0	0	0	0	0	0	0	0	0	0	0	0
7	0	0	0	0	0	0	0	0	0	0	0	0	0	0	0	0	0	0
0	0	0	0	0	0	0	0	0	0	0	0	0	5	0	0	0	13	0
0	0	12	0	0	0	0	0	0	0	0	0	0	0	0	0	0	0	0
0	0	0	0	0	0	0	0	0	0	0	0	0	64	0	0	0	0	0
0	0	0	0	0	0	0	0	0	0	0	0	0	0	0	0	0	0	0
0	8	0	0	0	0	0	0	0	0	0	0	0	0	0	0	0	0	0
0	0	0	0	0	0	0	0	0	0	0	0	0	0	0	0	0	0	0
0	0	0	0	0	0	0	20	0	0	0	0	0	0	0	0	0	0	0
0	0	0	0	0	0	0	0	0	0	0	0	0	0	0	0	0	0	0
0	0	0	0	0	0	0	0	0	0	0	0	0	0	0	0	0	0	0
0	0	0	0	0	0	0	0	0	0	0	0	0	0	0	0	0	0	0
0	0	0	0	0	0	0	0	0	0	0	0	0	0	0	0	0	0	0
0	0	0	0	0	0	0	0	0	0	0	0	0	0	0	0	0	0	0
0	0	0	0	0	0	0	0	0	5	0	0	0	0	0	0	0	0	0
0	0	0	0	0	6	0	0	0	0	0	0	0	0	0	0	0	0	0
0	0	0	0	0	0	0	0	0	0	0	0	0	0	0	0	0	0	0
0	0	0	0	0	0	0	0	0	0	0	0	0	0	0	0	0	0	0
0	0	0	0	0	0	0	0	0	0	0	0	0	0	0	0	0	0	0
0	0	0	0	7	0	0	0	0	0	0	0	0	0	0	0	0	0	0
0	0	0	0	0	4	0	0	0	0	0	0	0	0	0	0	0	0	0
0	0	0	0	0	0	0	0	0	0	0	0	0	0	0	0	0	0	0
0	0	0	0	0	0	0	0	0	0	0	0	0	0	0	0	0	0	0
0	0	0	0	0	0	0	0	0	0	0	0	0	0	0	0	0	0	0
0	0	0	0	0	0	0	0	0	0	0	0	0	0	0	0	0	0	0
0	0	0	0	0	0	0	0	0	0	0	0	0	0	0	0	0	0	0
0	0	0	0	0	0	0	0	0	0	0	0	0	0	6	0	0	0	0
0	0	0	0	0	0	0	0	0	0	0	0	0	0	0	0	0	0	0
0	0	0	0	0	0	0	0	0	0	0	0	0	0	0	0	0	0	0

0	0	0	0	0	0	0	0	0	0	0	0	0	0	0	0	0	0	0
0	0	0	0	0	0	0	0	0	0	12	0	0	0	0	0	0	0	0
0	0	0	0	0	0	0	0	0	0	0	0	0	0	0	0	0	0	0
0	0	0	0	0	0	0	0	0	0	0	0	0	0	0	0	0	0	0
0	0	0	0	0	0	0	0	0	0	0	0	0	0	0	0	0	0	0
0	0	0	0	0	0	0	0	0	0	0	0	0	0	0	0	0	0	0
0	0	0	0	0	0	0	0	0	0	0	0	0	0	0	0	0	0	0
0	0	0	0	0	0	0	0	0	0	0	0	0	0	4	0	0	0	0
0	0	0	0	0	0	0	0	0	0	0	0	0	0	0	0	0	0	0
0	0	0	0	0	0	0	0	0	0	0	0	0	0	0	0	0	0	0
0	0	0	0	0	0	0	0	0	0	0	0	0	0	0	0	0	0	0
0	0	0	0	0	0	0	0	0	0	0	0	0	0	0	0	0	0	0
0	0	0	0	0	0	0	0	0	0	0	0	0	0	0	0	0	0	10
0	0	0	0	0	0	0	0	0	0	0	0	0	0	0	0	0	0	0
0	0	0	0	0	0	0	0	0	0	0	0	0	0	0	0	0	0	0
0	0	0	0	0	0	0	0	0	0	0	0	0	0	0	0	0	0	0
0	0	0	0	0	0	0	0	0	0	0	0	0	0	0	0	0	0	0
0	0	0	0	9	0	0	0	0	0	0	0	0	0	0	0	0	0	0
0	0	0	0	0	0	0	0	0	0	0	0	0	0	0	0	0	0	0
0	0	0	0	0	0	0	0	0	0	0	0	0	0	0	0	0	0	0
0	0	0	0	0	0	0	0	0	0	0	0	0	0	0	0	0	0	0
0	0	0	0	0	0	0	0	0	0	0	0	0	0	0	0	0	0	0
0	0	0	0	0	0	0	0	0	0	0	0	0	0	0	0	0	0	0
0	0	0	0	0	0	0	0	0	0	0	0	0	0	0	0	0	0	0
0	0	0	0	0	0	0	0	0	0	0	0	0	0	0	0	0	0	0
0	0	0	0	0	0	0	0	0	0	0	0	0	0	0	0	0	0	0
0	0	0	0	0	0	0	0	0	0	0	0	0	0	0	0	0	0	0
0	0	0	0	0	0	0	0	0	0	0	0	7	0	0	0	0	0	0
0	0	0	0	0	0	0	0	0	0	0	0	0	0	0	0	0	0	0
0	0	0	0	0	0	0	0	0	0	0	0	0	0	0	0	0	0	0
0	0	0	0	0	0	0	0	0	0	0	0	0	0	0	0	0	0	0
0	0	0	0	0	0	0	0	0	0	0	0	0	0	0	0	0	0	0
0	0	0	0	0	0	0	0	0	0	0	0	0	0	0	0	0	0	0
0	0	0	0	0	0	0	0	0	0	0	0	0	0	0	0	0	0	0
0	0	0	0	0	0	0	0	0	0	0	0	0	0	0	0	0	0	0
0	0	0	0	0	0	0	0	0	0	0	0	0	0	0	0	0	0	0
0	0	0	0	0	0	0	0	0	0	0	0	0	0	0	0	0	0	0

0	0	0	0	0	0	0	0	0	0	0	0	0	0	0	0	0	0	0
0	0	0	0	0	0	0	0	0	0	0	0	0	0	0	0	0	0	0
0	0	0	0	0	0	0	0	0	0	0	0	0	0	0	0	0	0	0
0	0	0	0	0	0	0	0	0	0	0	0	0	0	0	0	0	0	0
0	0	0	0	0	0	0	0	0	0	0	0	0	0	0	0	0	0	0
0	0	0	0	0	0	0	0	0	0	0	0	0	0	0	0	0	0	0
0	0	0	0	0	0	0	0	0	0	0	0	0	0	0	0	0	0	0
0	0	0	0	0	0	0	0	0	0	0	0	0	0	0	0	0	0	0
0	0	0	0	0	0	0	0	0	0	0	0	0	0	0	0	0	0	0
0	0	0	0	0	0	0	0	0	0	0	0	0	0	0	0	0	0	0
0	0	0	0	0	0	0	0	0	0	0	0	0	0	0	0	0	0	0
0	0	0	0	0	0	0	0	0	0	0	0	0	0	0	0	0	0	0
0	0	0	0	0	0	0	0	0	0	0	0	0	0	0	0	0	0	0
0	0	0	0	0	0	0	0	0	0	0	0	0	0	0	0	0	0	0
0	0	0	0	0	0	0	0	0	0	0	0	0	0	0	0	0	0	0
0	0	0	0	0	0	0	0	0	0	0	0	0	0	0	0	0	0	0
0	0	0	0	0	0	0	0	0	0	0	0	0	0	0	0	0	0	0
0	0	0	0	0	0	0	0	0	0	0	0	0	0	0	0	0	0	0
0	0	0	0	0	0	0	0	0	0	0	0	0	0	0	0	0	0	0
0	0	0	0	0	0	0	0	0	0	0	0	0	0	0	0	0	0	0
0	0	0	0	0	0	0	0	0	0	0	0	0	0	0	0	0	0	0
0	0	0	0	0	0	0	0	0	0	0	0	0	0	0	0	0	0	0
0	0	0	0	0	0	0	0	0	0	0	0	0	0	0	0	0	0	0
0	0	0	0	0	0	0	0	0	0	0	0	0	0	0	0	0	0	0
0	0	0	0	0	0	0	0	0	0	0	0	0	0	0	0	0	0	0
0	0	0	0	0	0	0	0	0	0	0	0	0	0	0	0	0	0	0
0	0	0	0	0	0	0	0	0	0	0	0	0	0	0	0	0	0	0
0	0	0	0	0	0	0	0	0	0	0	0	0	0	0	0	0	0	0
0	0	0	0	0	0	0	0	0	0	0	0	0	0	0	0	0	0	0
0	0	0	0	0	0	0	0	0	0	0	0	0	0	0	0	0	0	0
0	0	0	0	0	0	0	0	0	0	0	0	0	0	0	0	0	0	0
0	0	0	0	0	0	0	0	0	0	0	0	0	0	0	0	0	0	0
0	0	0	0	0	0	0	0	0	0	0	0	0	0	0	0	0	0	0
0	0	0	0	0	0	0	0	0	0	0	0	0	0	0	0	0	0	0
0	0	0	0	0	0	0	0	0	0	0	0	0	0	0	0	0	0	0
0	0	0	0	0	0	0	0	0	0	0	0	0	0	0	0	0	0	0
0	0	0	0	0	0	0	0	0	0	0	3	0	0	0	0	0	0	0

0	0	0	0	0	0	0	0	0	0	0	0	0	0	0	0	0	0	0
0	0	0	0	0	0	0	0	0	0	0	0	0	0	0	0	0	0	0
0	0	0	0	0	0	0	0	0	0	0	0	0	0	0	0	0	0	0
0	0	0	0	0	0	0	0	0	0	0	0	0	0	0	0	0	0	0
0	0	0	0	0	0	0	0	0	0	0	0	0	0	0	0	0	0	0
0	0	0	0	0	0	0	0	0	0	0	0	0	0	0	0	0	0	0
0	0	0	0	0	0	0	0	0	0	0	0	0	0	0	0	0	0	0
0	0	0	0	0	0	0	0	0	0	0	0	0	0	0	0	0	0	0
0	0	0	0	0	0	0	0	0	0	0	0	0	0	0	0	0	0	0
0	0	0	0	0	0	0	0	0	0	0	0	0	0	0	0	0	0	0
0	0	0	0	0	0	0	0	0	0	0	0	0	0	0	0	0	0	0
0	0	0	0	0	0	0	0	0	0	0	0	0	0	0	0	0	0	0
0	0	0	0	0	0	0	0	0	0	0	0	0	0	0	0	0	0	0
0	0	0	0	0	0	0	0	0	0	0	0	0	0	0	0	0	0	0
0	0	0	0	0	0	0	0	0	0	0	0	0	0	0	0	0	0	0
0	0	0	0	0	0	0	0	0	0	0	0	0	0	0	0	0	0	0
0	0	0	0	0	0	0	0	0	0	0	0	0	0	0	0	0	0	0
0	0	0	0	0	0	0	0	0	0	0	0	0	0	0	0	0	0	0
0	0	0	0	0	0	0	0	0	0	0	0	0	0	0	0	0	0	0
0	0	0	0	0	0	0	0	0	0	0	0	0	0	0	0	0	0	0
0	0	0	0	0	0	0	0	0	0	0	0	0	0	0	0	0	0	0
0	0	0	0	0	0	0	0	0	0	0	0	0	0	0	0	0	0	0
0	0	0	0	0	0	0	0	0	0	0	0	0	0	0	0	0	0	0
0	0	0	0	0	0	0	0	0	0	0	0	0	0	0	0	0	0	0
0	0	0	0	0	0	0	0	0	0	0	0	0	0	0	0	0	0	0
0	0	0	0	0	0	0	0	0	0	0	0	0	0	0	0	0	0	2
0	0	0	0	0	0	0	0	0	0	0	0	0	0	0	0	0	0	0
0	0	0	0	0	0	0	0	0	0	0	0	0	0	0	0	0	0	0
0	0	0	0	0	0	0	0	0	0	0	0	0	0	0	0	0	0	0
0	0	0	0	0	0	0	0	0	0	0	0	0	0	0	0	0	0	0
0	0	0	0	0	0	0	0	0	0	0	0	0	0	0	0	0	0	0
0	0	0	0	0	0	0	0	0	0	0	0	0	0	0	0	0	0	0
0	0	0	0	0	0	0	0	0	0	0	0	0	0	0	0	0	0	0
0	0	0	0	0	0	0	0	0	0									
Jan_DL036	734	1886	813	865	122	333	129	99	61	453	817	174	379	357	331	558	338	201
248	0	225	0	184	182	268	152	252	428	94	348	154	114	146	360	79	163	268
0	85	0	58	100	284	101	125	23	241	48	237	32	111	0	189	119	57	212

147	8	159	24	0	0	97	92	71	100	266	108	0	74	0	38	19	54	60
84	177	0	85	0	79	48	0	102	0	0	43	31	39	65	82	0	60	0
0	58	0	0	57	24	0	0	0	8	70	82	45	0	28	26	7	0	0
0	0	0	0	0	0	0	0	0	0	0	0	15	21	0	0	29	0	0
0	22	0	25	14	0	0	59	0	11	0	0	23	43	32	60	0	0	0
0	18	14	50	0	7	31	0	0	0	5	0	0	0	0	0	72	0	0
24	0	23	0	0	0	0	0	0	0	0	39	0	0	30	21	0	0	0
0	23	10	0	0	0	0	0	0	0	18	0	0	0	0	0	0	0	0
0	0	0	0	6	0	0	0	0	25	0	12	34	0	0	9	51	0	0
0	0	0	0	0	0	0	0	0	0	0	0	10	0	0	0	26	0	0
14	0	0	0	0	0	0	0	0	0	0	0	0	29	23	0	0	12	0
0	0	0	0	0	8	0	0	0	0	0	2	0	0	20	0	0	0	0
0	9	0	17	0	0	0	0	29	0	0	0	0	0	0	0	0	0	22
0	0	0	0	0	0	0	0	0	0	0	0	5	0	0	0	0	0	0
0	0	0	0	0	0	12	0	0	0	0	0	0	0	0	7	0	0	0
0	0	0	0	0	0	0	21	0	0	0	0	0	0	0	0	0	0	0
19	6	19	0	0	0	6	0	0	0	0	0	0	22	0	0	0	0	0
0	0	0	0	0	7	0	1	0	0	0	0	0	0	0	0	0	0	0
0	0	0	0	0	0	0	0	0	0	0	0	0	0	0	0	0	0	0
0	0	0	0	0	0	0	0	0	0	0	0	0	0	0	0	0	0	0
0	0	0	0	0	0	14	0	0	0	0	0	0	0	0	0	0	6	0
0	9	0	11	0	0	0	0	0	0	0	0	0	0	0	0	0	0	0
0	0	0	0	0	0	0	11	0	0	0	0	0	0	0	7	0	0	0
0	0	0	0	0	0	0	0	0	0	0	0	0	0	0	0	0	0	0
0	0	0	0	48	0	0	0	0	0	0	0	0	0	0	0	0	0	0
8	0	0	0	0	0	0	0	0	0	0	0	0	0	0	0	0	0	0
0	3	0	0	0	0	0	0	0	0	0	0	0	0	0	0	0	0	0
0	0	0	0	0	0	9	0	0	0	0	0	0	0	0	0	17	0	0
0	0	0	0	0	0	0	4	0	0	0	0	0	0	0	0	0	0	0
0	0	0	0	0	0	0	0	0	0	0	0	0	0	0	3	0	0	0
0	0	0	14	0	33	0	0	0	0	0	0	0	0	11	0	0	0	0
0	0	0	0	0	0	0	0	0	0	0	0	0	0	0	0	0	0	0
0	0	0	0	0	0	0	0	0	0	0	0	0	0	0	0	0	0	0
0	0	0	0	0	0	0	33	0	0	0	0	0	0	0	0	0	0	0
0	0	0	0	0	0	0	0	0	0	0	0	0	0	0	0	0	0	0
0	0	0	0	0	0	0	0	0	0	0	0	0	0	0	0	0	0	0
0	0	0	0	0	0	0	0	0	0	0	0	0	0	0	0	0	0	0

0	0	0	0	0	0	0	4	0	0	0	0	0	0	0	0	0	0	0
0	0	0	0	0	0	0	0	0	0	0	0	0	0	0	0	0	0	0
0	0	0	0	0	0	0	0	0	0	0	0	0	0	0	0	0	0	0
0	0	0	0	0	0	0	0	0	0	0	0	0	0	0	0	0	0	0
0	0	0	0	0	0	0	0	0	0	0	7	0	0	0	0	0	0	0
0	0	0	0	0	11	0	0	0	0	0	0	0	0	0	0	0	0	0
0	0	0	0	0	0	0	0	0	0	0	0	0	0	0	0	0	0	0
0	0	0	0	0	0	0	0	0	0	0	0	0	0	0	0	0	0	0
0	0	0	0	0	0	0	0	0	0	0	0	0	0	0	0	0	0	0
0	0	0	0	0	0	0	0	0	0	0	0	0	0	0	0	16	0	0
0	0	0	0	0	0	0	0	0	0	0	0	0	0	0	0	0	0	0
0	0	0	0	0	0	0	0	0	0	0	0	0	0	0	0	0	0	0
0	0	0	0	0	0	0	0	0	0	0	0	0	13	0	0	0	0	0
0	0	0	0	0	0	0	0	0	0	0	0	0	0	0	0	0	0	0
0	0	0	0	9	0	0	0	0	0	0	0	0	0	0	0	0	0	0
0	0	0	0	0	0	0	0	0	0	0	0	0	0	0	0	0	0	0
0	0	0	0	0	0	0	0	0	0	0	0	0	0	0	0	0	0	0
0	0	0	0	0	0	0	0	0	0	0	0	0	27	0	0	0	0	0
0	0	0	0	0	0	0	0	0	0	0	0	0	0	0	0	0	0	0
0	0	0	0	0	0	0	0	0	0	0	0	0	0	0	0	0	0	0
0	0	0	0	0	0	0	0	0	0	0	0	0	0	0	0	0	0	0
0	0	0	0	0	0	0	0	0	0	0	0	0	0	0	0	0	0	0
0	0	0	0	0	0	0	0	0	0	0	0	0	0	0	0	0	0	0
0	0	0	0	0	0	0	0	0	0	0	0	0	0	0	0	0	0	0
0	0	0	0	0	0	0	0	0	0	0	0	0	0	0	0	0	7	0
0	0	0	0	0	0	0	0	0	0	0	0	0	0	0	0	0	0	0
0	0	0	0	0	0	0	0	0	0	0	0	0	0	0	0	0	0	0
0	0	0	0	0	0	0	0	0	0	0	0	0	0	0	0	0	0	0
0	0	0	0	0	0	0	0	0	0	0	0	0	0	0	0	0	0	0
0	0	0	0	0	0	0	0	0	0	0	0	0	0	0	0	0	0	0
0	0	0	0	0	0	0	0	0	0	0	0	0	0	0	0	0	0	0
0	0	0	0	0	0	0	0	0	0	0	0	0	0	0	0	0	0	0
0	0	0	0	0	0	0	0	0	0	0	0	0	0	0	0	0	0	0
0	0	0	0	0	0	0	0	0	0	0	0	0	0	0	0	0	0	0
0	0	0	0	0	0	0	0	0	0	0	0	0	0	0	0	0	0	0
0	0	0	0	0	0	0	0	0	0	0	0	0	0	0	0	0	0	0
0	0	0	0	0	0	0	0	0	0	0	0	0	0	0	0	0	0	0

0	0	0	0	0	0	0	0	0	0	0	0	0	0	0	0	0	0	0
0	0	0	0	0	0	0	0	0	0	0	0	0	0	0	0	0	0	0
0	0	0	0	0	0	0	0	0	0	0	0	0	0	0	0	0	0	0
0	0	0	0	0	0	0	0	0	0	0	0	0	0	0	0	0	0	0
0	0	0	0	0	0	0	0	0	0	0	0	0	0	0	0	0	0	0
0	0	0	0	0	0	0	0	0	0	0	0	0	0	0	0	0	0	0
0	0	0	0	0	0	0	0	0	0	0	0	0	0	0	0	0	0	0
0	0	0	0	0	0	0	0	0	0	0	0	0	0	0	0	0	0	0
0	0	0	0	0	0	0	0	0	0	0	0	0	0	0	0	0	0	0
0	0	0	0	0	0	0	0	0	0	0	0	0	0	0	0	0	0	0
0	0	0	0	0	0	0	0	0	0	0	0	0	0	0	0	0	0	0
0	0	0	0	0	0	0	0	0	0	0	0	0	0	0	0	0	0	0
0	0	0	0	0	0	0	0	0	0	0	0	0	0	0	0	0	0	0
0	0	0	0	0	0	0	0	0	0	0	0	0	0	0	0	0	0	0
0	0	0	0	0	0	0	0	0	0	0	0	0	0	0	0	0	0	0
0	0	0	0	0	0	0	0	0	0	0	0	0	0	0	0	0	0	0
0	0	0	0	0	0	0	0	0	0	0	0	0	0	0	0	0	0	0
0	0	0	0	0	0	0	0	0	0	0	0	0	0	0	0	0	0	0
0	0	0	0	0	0	0	0	0	0	0	0	0	0	0	0	0	0	0
0	0	0	0	0	0	0	0	0	0	0	0	0	0	0	0	0	0	0
0	0	0	0	0	0	0	0	0	0	0	0	0	0	0	0	0	0	0
0	0	0	0	0	0	0	0	0	0	0	0	0	0	0	0	0	0	0
0	0	0	0	0	0	0	0	0	0	0	0	0	0	0	0	0	0	0
0	0	0	0	0	0	0	0	0	0	0	0	0	0	0	0	0	0	0
0	0	0	0	0	0	0	0	0	0	0	0	0	0	0	0	0	0	0
0	0	0	0	0	0	0	0	0	0	0	0	0	0	0	0	0	0	0
0	0	0	0	0	0	0	0	0	0	0	0	0	0	0	0	0	0	0
0	0	0	0	0	0	0	0	0	0	0	0	0	0	0	0	0	0	0
0	0	0	0	0	0	0	0	0	0	0	0	0	0	0	0	0	0	0
0	0	0	0	0	0	0	0	0	0	0	0	0	0	0	0	0	0	0
0	0	0	0	0	0	0	0	0	0	0	0	0	0	0	0	0	0	0
0	0	0	0	0	0	0	0	0	0	0	0	0	0	0	0	0	0	0
0	0	0	0	0	0	0	0	0	0	0	0	0	0	0	0	0	0	0
0	0	0	0	0	0	0	0	0	0	0	0	0	0	0	0	0	0	0
0	0	0	0	0	0	0	0	0	0	0	0	0	0	0	0	0	0	0
0	0	0	0	0	0	0	0	0	0	0	0	0	0	0	0	0	0	0
0	0	0	0	0	0	0	0	0	0	0	0	0	0	0	0	0	0	0

0	0	0	0	0	0	0	0	0	0	0	0	0	0	0	0	0	0	0
0	0	0	0	0	0	0	0	0	0	0	0	0	0	0	0	0	0	0
0	0	0	0	0	0	0	0	0	0	0	0	0	0	0	0	0	0	0
0	0	0	0	0	0	0	0	0	0	0	0	0	0	0	0	0	0	0
0	0	0	0	0	0	0	0	0	0	0	0	0	0	0	0	0	0	0
0	0	0	0	0	0	0	0	0	0	0	0	0	0	0	0	0	0	0
0	0	0	0	0	0	0	0	0	0	0	0	0	0	0	0	0	0	0
0	0	0	0	0	0	0	0	0	0	0	0	0	0	0	0	0	0	0
0	0	0	0	0	0	0	0	0	0	0	0	0	0	0	0	0	0	0
0	0	0	0	0	0	0	0	0	0	0	0	0	0	0	0	0	0	0
0	0	0	0	0	0	0	0	0	0	0	0	0	0	0	0	0	0	0
0	0	0	0	0	0	0	0	0	0	0	0	0	0	0	0	0	0	0
0	0	0	0	0	0	0	0	0	0	0	0	0	0	0	0	0	0	0
0	0	0	0	0	0	0	0	0	0	0	0	0	0	0	0	0	0	0
0	0	0	0	0	0	0	0	0	0	0	0	0	0	0	0	0	0	0
0	0	0	0	0	0	0	0	0	0	0	0	0	0	0	0	0	0	0
0	0	0	0	0	0	0	0	0	0	0	0	0	0	0	0	0	0	0
0	0	0	0	0	0	0	0	0	0	0	0	0	0	0	0	0	0	0
0	0	0	0	0	0	0	0	0	0	0	0	0	0	0	0	0	0	0
0	0	0	0	0	0	0	0	0	0	0	0	0	0	0	0	0	0	0
0	0	0	0	0	0	0	0	0	0	0	0	0	0	0	0	0	0	0
0	0	0	0	0	0	0	0	0	0	0	0	0	0	0	0	0	0	0
0	0	0	0	0	0	0	0	0	0	0	0	0	0	0	0	0	0	0
0	0	0	0	0	0	0	0	0	0	0	0	0	0	0	0	0	0	0
0	0	0	0	0	0	0	0	0	0	0	0	0	0	0	0	0	0	0
0	0	0	0	0	0	0	0	0	0	0	0	0	3	3	3	3	0	0
0	0	0	0	0	0	0	0	0	0	0	0	0	0	0	0	0	0	0
0	0	0	0	0	0	0	0	0	0	0	0	0	0	0	0	0	0	0
0	0	0	0	0	0	0	0	0	0	0	0	0	0	0	0	0	0	0
0	0	0	0	0	0	0	0	0	0	0	0	0	0	0	0	0	0	0
0	0	0	0	0	0	0	0	0	0	0	0	0	0	0	0	0	0	0
0	0	0	0	0	0	0	0	0	0	0	0	0	0	0	0	0	0	0
0	0	0	0	0	0	0	0	0	0	0	0	0	0	0	0	0	0	0
0	0	0	0	0	0	0	0	0	0	0	0	0	0	0	0	0	0	0
0	0	0	0	0	0	0	0	0	0	0	0	0	0	0	0	0	0	0
0	0	0	0	0	0	0	0	0	0	0	0	0	0	0	0	0	0	0
0	0	0	0	0	0	0	0	0	0	0	0	0	0	0	0	0	0	0

0	0	0	0	0	0	0	0	0	0	0	0	0	0	0	0	0	0	0
0	0	0	0	0	0	0	0	0	0	0	0	0	0	0	0	0	0	0
0	0	0	0	0	0	0	0	0	0	0	0	0	0	0	0	0	0	0
0	0	0	0	0	0	0	0	0	0	0	0	0	0	0	0	0	0	0
0	0	0	0	0	0	0	0	0	0	0	0	0	0	0	0	0	0	0
0	0	0	0	0	0	0	0	0	0	0	0	0	0	0	0	0	0	0
0	0	0	0	0	0	0	0	0	0	0	0	0	0	0	0	0	0	0
0	0	0	0	0	0	0	0	0	0	0	0	0	0	0	0	0	0	0
0	0	0	0	0	0	0	0	0	0	0	0	0	0	0	0	0	0	0
0	0	0	0	0	0	0	0	0	0	0	0	0	0	0	0	0	0	0
0	0	0	0	0	0	0	0	0	0	0	0	0	0	0	0	0	0	0
0	0	0	0	0	0	0	0	0	0	0	0	0	0	0	0	0	0	0
0	0	0	0	0	0	0	0	0	0	0	0	0	0	0	0	0	0	0
0	0	0	0	0	0	0	0	0	0	0	0	0	0	0	0	0	0	0
0	0	0	0	0	0	0	0	0	0	0	0	0	0	0	0	2	2	2
2	2	2	0	0	0	0	0	0	0	0	0	0	0	0	0	0	0	0
0	0	0	0	0	0	0	0	0	0	0	0	0	0	0	0	0	0	0
0	0	0	0	0	0	0	0	0	0	0	0	0	0	0	0	0	0	0
0	0	0	0	0	0	0	0	0	0	0	0	0	0	0	0	0	0	0
0	0	0	0	0	0	0	0	0	0	0	0	0	0	0	0	0	0	0
0	0	0	0	0	0	0	0	0	0	0	0	0	0	0	0	0	0	0
0	0	0	0	0	0	0	0	0	0	0	0	0	0	0	0	0	0	0
0	0	0	0	0	0	0	0	0	0	0	0	0	0	0	0	0	0	0
0	0	0	0	0	0	0	0	0	0	0	0	0	0	0	0	0	0	0
0	0	0	0	0	0	0	0	0	0	0	0	0	0	0	0	0	0	0
0	0	0	0	0	0	0	0	0	0									
Jan_DL037	1829	2019	693	1110	168	315	121	114	377	573	466	218	506	321	185	289	300	208
32	0	194	0	315	144	172	127	174	405	163	391	118	150	242	211	91	225	222
0	49	89	191	148	201	0	0	102	111	27	140	0	28	70	121	228	0	152
186	30	99	33	0	0	161	96	67	234	49	82	0	141	0	0	25	25	10
58	130	38	92	51	38	49	24	52	0	68	43	22	58	40	187	0	0	0
0	29	0	0	46	0	0	0	0	9	181	35	33	11	24	21	20	0	0
0	0	0	0	7	0	0	0	0	53	27	0	23	23	25	0	29	0	0
53	27	0	23	0	0	34	0	75	17	14	0	33	0	29	0	0	0	0
0	13	0	5	0	11	51	0	0	0	0	0	0	0	0	25	52	0	0
14	49	24	0	0	0	0	33	0	0	0	0	0	0	45	12	8	0	0
0	25	18	0	0	0	57	0	0	0	48	0	0	0	0	0	0	0	0

0	0	0	0	17	0	0	0	0	0	0	5	0	0	0	24	0	0	0
0	0	0	0	0	0	0	0	0	0	0	0	0	0	0	0	35	0	7
14	0	0	0	0	0	0	19	0	0	62	0	0	0	0	11	0	0	0
7	24	0	0	0	6	0	0	0	0	0	0	0	0	0	0	0	55	0
0	14	0	18	0	0	0	0	0	62	0	0	0	0	0	0	0	0	0
0	0	0	0	0	0	0	0	0	0	0	0	0	9	0	6	0	0	3
0	0	8	0	0	0	15	0	0	0	0	0	0	0	0	0	0	0	0
9	0	0	0	0	0	0	25	0	0	0	0	0	0	0	0	0	0	0
50	0	0	0	0	0	0	0	0	0	0	0	0	0	0	0	0	0	7
0	0	0	0	0	0	0	4	0	0	0	0	0	0	0	0	0	0	0
0	0	0	0	0	0	0	0	0	0	0	0	0	0	0	0	0	0	0
0	0	0	0	0	0	0	0	0	0	0	0	0	0	0	0	0	0	0
0	7	0	0	0	9	9	0	0	0	0	0	0	0	0	0	10	13	8
0	0	0	0	0	0	0	0	0	0	0	0	0	0	0	0	0	0	0
0	0	0	0	0	0	0	0	0	0	0	0	0	0	0	0	0	0	0
0	0	0	0	0	0	0	0	0	0	0	0	0	0	0	0	11	0	0
23	0	0	0	50	0	0	0	0	0	0	0	0	0	0	0	0	0	0
0	0	0	0	0	0	0	0	0	0	0	0	0	0	0	0	0	0	0
0	0	0	8	0	0	0	9	0	0	0	0	0	0	0	0	0	0	0
0	0	0	0	0	0	0	0	13	0	0	0	0	0	0	0	19	0	7
0	0	0	0	0	0	0	8	0	0	0	16	0	10	12	0	0	0	0
0	0	0	0	7	0	0	0	0	0	0	0	0	0	0	0	23	11	0
0	0	0	0	0	0	0	0	0	0	0	0	0	0	0	0	0	0	21
0	0	0	0	0	0	0	0	0	0	0	0	0	0	13	0	0	0	0
0	0	0	0	0	0	0	0	0	0	0	0	0	0	0	0	0	0	0
0	0	0	0	0	0	0	0	0	0	0	0	0	0	0	7	0	0	0
0	0	0	0	0	0	0	0	0	0	0	0	0	0	0	0	0	0	0
0	0	0	0	0	0	0	0	0	0	0	0	0	0	0	0	0	0	0
0	0	0	0	0	0	0	0	0	0	0	0	0	0	0	0	0	0	0
0	19	0	0	0	0	0	0	0	0	7	0	0	0	0	0	0	0	0
0	0	0	0	0	0	0	0	0	0	0	0	0	0	0	0	0	0	4
0	0	0	0	0	0	0	0	0	0	0	0	0	0	0	0	0	0	0
0	0	0	0	0	0	9	0	0	0	0	0	0	0	0	0	0	0	0
0	0	0	0	0	0	0	0	25	0	0	0	0	0	0	0	0	0	0
0	0	0	0	0	0	0	0	0	0	0	0	0	0	0	0	0	0	0
4	0	0	0	0	0	0	0	0	0	0	0	0	0	0	0	0	0	0
0	0	0	0	0	0	0	0	0	0	0	0	0	0	0	0	0	0	0

0	0	0	0	0	0	0	0	0	0	0	0	0	0	0	0	0	0	0
0	0	0	0	0	0	0	0	0	0	0	0	0	0	0	0	0	0	0
0	0	0	0	0	0	0	0	0	0	0	0	0	0	0	0	0	0	0
0	0	0	0	0	0	0	0	0	0	0	0	0	0	0	0	0	0	0
0	0	0	0	0	0	0	0	0	0	0	0	0	0	0	0	0	0	0
0	0	0	0	0	0	0	0	0	0	0	0	0	0	0	0	0	0	0
0	0	0	0	0	0	0	0	0	0	0	0	0	0	0	0	0	0	0
0	0	0	0	0	0	0	0	0	0	0	0	0	0	0	0	0	0	0
0	0	0	0	0	0	0	0	0	0	0	0	0	0	0	0	0	0	7
0	0	0	0	0	0	0	0	0	0	0	0	0	0	0	0	0	0	0
0	0	0	0	0	0	0	0	0	0	0	0	0	0	0	0	0	0	0
0	0	0	0	0	0	0	0	0	0	0	0	0	0	0	0	0	0	0
0	0	0	0	0	0	0	0	0	0	0	0	0	0	0	0	0	0	0
0	15	0	0	0	0	0	0	0	0	0	0	0	0	0	0	0	0	0
0	0	0	0	0	0	0	0	0	0	0	0	0	0	0	0	0	0	0
0	0	0	0	0	0	0	0	0	0	0	0	0	0	0	0	0	0	0
0	0	0	0	0	0	0	0	0	0	0	0	0	0	0	0	0	0	0
0	0	0	0	0	0	0	0	0	0	0	0	0	0	0	0	0	0	0
0	0	0	0	0	0	0	0	0	0	0	0	0	0	0	0	0	0	0
0	0	0	0	0	0	0	0	0	0	0	0	0	0	0	0	0	0	2
0	0	0	0	0	0	0	0	0	0	0	0	0	0	0	0	0	0	0
0	0	0	0	0	0	0	0	0	0	0	0	0	0	0	0	0	0	0
0	0	0	0	0	0	0	0	0	0	0	0	0	0	0	0	0	0	0
0	0	0	0	0	0	0	0	0	0	0	0	0	0	0	0	0	0	0
0	0	0	0	0	0	0	0	0	0	0	0	0	0	0	0	0	0	0
0	0	0	0	0	0	0	0	0	0	0	0	0	0	0	0	0	0	0
0	0	0	0	0	0	0	0	0	0	0	0	0	0	0	0	0	0	0
0	0	0	0	0	0	0	0	0	0	0	0	0	0	0	0	0	0	0
0	0	0	0	0	0	0	0	0	0	0	0	0	0	0	0	0	0	0
0	0	0	0	0	0	0	0	0	0	0	0	0	0	0	0	0	0	0
0	0	0	0	0	0	0	0	0	0	0	0	0	0	0	0	0	0	0
0	0	0	0	0	0	0	0	0	0	0	0	0	0	0	0	0	0	0
0	0	0	0	0	0	0	0	0	0	0	0	0	0	0	0	0	0	0
0	0	0	0	0	0	0	0	0	0	0	0	0	0	0	0	0	0	0
0	0	0	0	0	0	0	0	0	0	0	0	0	0	0	0	0	0	0
0	0	0	0	0	0	0	0	0	0	0	0	0	0	0	0	0	0	0
0	0	0	0	0	0	0	0	0	0	0	0	0	0	0	0	0	0	0

0	0	0	0	0	0	0	0	0	0	0	0	0	0	0	0	0	0	0
0	0	0	0	0	0	0	0	0	0	0	0	0	0	0	0	0	0	0
0	0	0	0	0	0	0	0	0	0	0	0	0	0	0	0	0	0	0
0	0	0	0	0	0	0	0	0	0	0	0	0	0	0	0	0	0	0
0	0	0	0	0	0	0	0	0	0	0	0	0	0	0	0	0	0	0
0	0	0	0	0	0	0	0	0	0	0	0	0	0	0	0	0	0	0
0	0	0	0	0	0	0	0	0	0	0	0	0	0	0	0	0	0	0
0	0	0	0	0	0	0	0	0	0	0	0	0	0	0	0	0	0	0
0	0	0	0	0	0	0	0	0	0	0	0	0	0	0	0	0	0	0
0	0	0	0	0	0	0	0	0	0	0	0	0	0	0	0	0	0	0
0	0	0	0	0	0	0	0	0	0	0	0	0	0	0	0	0	0	0
0	0	0	0	0	0	0	0	0	0	0	0	0	0	0	0	0	0	0
0	0	0	0	0	0	0	0	0	0	0	0	0	0	0	0	0	0	0
0	0	0	0	0	0	0	0	0	0	0	0	0	0	0	0	0	0	0
0	0	0	0	0	0	0	0	0	0	0	0	0	0	0	0	0	0	0
0	0	0	0	0	0	0	0	0	0	0	0	0	0	0	0	0	0	0
0	0	0	0	0	0	0	0	0	0	0	0	0	0	0	0	0	0	0
0	0	0	0	0	0	0	0	0	0	0	0	0	0	0	0	0	0	0
0	0	0	0	0	0	0	0	0	0	0	0	0	0	0	0	0	0	0
0	0	0	0	0	0	6	6	0	0	0	0	0	0	0	0	0	0	0
0	0	0	0	0	0	0	0	0	0	0	0	0	0	0	0	0	0	0
0	0	0	0	0	0	0	0	0	0	0	0	0	0	0	0	0	0	0
0	0	0	0	0	0	0	0	0	0	0	0	0	0	0	0	0	0	0
0	0	0	0	0	0	0	0	0	0	0	0	0	0	0	0	0	0	0
0	0	0	0	0	0	0	0	0	0	0	0	0	0	0	0	0	0	0
0	0	0	0	0	0	0	0	0	0	0	0	0	0	0	0	0	0	0
0	0	0	0	0	0	0	0	0	0	0	0	0	0	0	5	0	0	0
0	0	0	0	0	0	0	0	0	0	0	0	0	0	0	0	0	0	0
0	0	0	0	0	0	0	0	0	0	0	0	0	0	0	0	0	0	0
0	0	0	0	0	0	0	0	0	0	0	0	0	0	0	0	0	0	0
0	0	0	0	0	0	0	0	0	0	0	0	0	0	0	0	0	0	0
0	0	0	0	0	0	0	0	0	0	0	0	0	0	0	0	0	0	0
0	0	0	0	0	0	0	0	0	0	0	0	0	0	0	0	0	0	0
0	0	0	0	0	0	0	0	0	0	0	0	0	0	0	0	0	0	0
0	0	0	0	0	0	0	0	0	0	0	0	0	0	0	0	0	0	0
0	0	0	0	0	0	0	0	0	0	0	0	0	0	0	0	0	0	0
0	0	0	0	0	0	0	0	0	0	0	0	0	0	0	0	0	0	0

0	0	0	0	0	0	0	0	0	0	0	0	0	0	0	0	0	0	0
0	0	0	0	0	0	0	0	0	0	0	0	0	0	0	0	0	0	0
0	0	0	0	0	0	0	0	0	0	0	0	0	0	0	0	0	0	0
0	0	0	0	0	0	0	0	0	0	0	0	0	0	0	0	0	0	0
0	0	0	0	0	0	0	0	0	0	0	0	0	0	0	0	0	0	0
0	0	0	0	0	0	0	0	0	0	0	0	0	0	0	0	0	0	0
0	0	0	0	0	0	0	0	0	0	0	0	0	0	0	0	0	0	0
0	0	0	0	0	0	0	0	0	0	0	0	0	0	0	0	0	0	0
0	0	0	0	0	0	0	0	0	0	0	0	0	0	0	0	0	0	0
0	0	0	0	0	0	0	0	0	0	0	0	0	0	0	0	0	0	0
0	0	0	0	0	0	0	0	0	0	0	0	0	0	0	0	0	0	0
0	0	0	0	0	0	0	0	0	0	0	0	0	0	0	0	0	0	0
0	0	0	0	0	0	0	0	0	0	0	0	0	0	0	0	0	0	0
0	0	0	0	0	0	0	0	0	0	0	0	0	0	0	0	0	0	0
0	0	0	0	0	0	0	0	0	0	0	0	0	0	0	0	0	0	0
0	0	0	0	0	0	0	0	0	0	0	0	0	0	0	0	0	0	0
0	0	0	0	0	0	0	0	0	0	0	0	0	0	0	0	0	0	0
0	0	0	0	0	0	0	0	0	0	0	0	0	0	0	0	0	0	0
0	0	0	0	0	0	0	0	0	0	0	0	0	0	0	0	0	0	0
0	0	0	0	0	0	0	0	0	0	0	0	0	0	0	0	0	0	0
0	0	0	0	0	0	0	0	0	0	0	0	3	3	0	0	0	0	0
0	0	0	0	0	0	0	0	0	0	0	0	0	0	0	0	0	0	0
0	0	0	0	0	0	0	0	0	0	0	0	0	0	0	0	0	0	0
0	0	0	0	0	0	0	0	0	0	0	0	0	0	0	0	0	0	0
0	0	0	0	0	0	0	0	0	0	0	0	0	0	0	0	0	0	0
0	0	0	0	0	0	0	0	0	0	0	0	0	0	0	0	0	0	0
0	0	0	0	0	0	0	0	0	0	0	0	0	0	0	0	0	0	0
0	0	0	0	0	0	0	0	0	0	0	0	0	0	0	0	0	0	0
0	0	0	0	0	0	0	0	0	0	0	0	0	0	0	0	0	0	0
0	0	0	0	0	0	0	0	0	0	0	0	0	0	0	0	0	0	0
0	0	0	0	0	0	0	0	0	0	0	0	0	0	0	0	0	0	0
0	0	0	0	0	0	0	0	0	0	0	0	0	0	0	0	0	0	0
0	0	0	0	0	0	0	0	0	0	0	0	0	0	0	0	0	0	0
0	0	0	0	0	0	0	0	0	0	0	0	0	0	0	0	0	0	0
0	0	0	0	0	0	0	0	0	0	0	0	0	0	0	0	0	0	0
0	0	0	0	0	0	0	0	0	0	0	0	0	0	0	0	0	0	0
0	0	0	0	0	0	0	0	0	0	0	0	0	0	0	0	0	0	0

0	0	0	0	0	0	0	0	0	0	0	0	0	0	0	0	0	0	0
0	0	0	0	0	0	0	0	0	0	0	0	0	0	0	0	0	0	0
0	0	0	0	0	0	0	0	0	0	0	0	0	0	0	0	0	0	0
0	0	0	0	0	0	0	0	0	0	0	0	0	0	0	0	0	0	0
0	0	0	0	0	0	0	0	0	0	0	0	0	0	0	0	0	0	0
0	0	0	0	0	0	0	0	0	0	0	0	0	0	0	0	0	0	0
0	0	0	0	0	0	0	0	0	0	0	0	0	0	0	0	0	0	0
0	0	0	0	0	0	0	0	0	0	0	0	0	0	0	0	0	0	0
0	0	0	0	0	0	0	0	0	0	0	0	0	0	0	0	0	0	0
0	0	0	0	0	0	0	0	0	0	0	0	0	0	0	0	0	0	0
2	2	2	2	2	0	0	0	0	0	0	0	0	0	0	0	0	0	0
0	0	0	0	0	0	0	0	0	0	0	0	0	0	0	0	0	0	0
0	0	0	0	0	0	0	0	0	0	0	0	0	0	0	0	0	0	0
0	0	0	0	0	0	0	0	0	0	0	0	0	0	0	0	0	0	0
0	0	0	0	0	0	0	0	0	0	0	0	0	0	0	0	0	0	0
0	0	0	0	0	0	0	0	0	0	0	0	0	0	0	0	0	0	0
0	0	0	0	0	0	0	0	0	0	0	0	0	0	0	0	0	0	0
0	0	0	0	0	0	0	0	0	0									
Jan_DL038	1647	1225	1505	376	325	203	321	315	97	558	607	228	508	40	45	568	44	304
129	0	135	382	215	110	310	134	208	150	111	0	75	248	212	355	113	144	0
126	55	44	59	155	324	88	15	58	226	26	135	20	57	36	118	0	0	0
0	0	112	20	0	0	85	58	94	33	59	0	0	76	14	0	32	14	34
43	77	20	12	0	28	17	16	0	131	0	44	18	38	21	0	58	0	47
0	67	0	0	123	0	0	37	0	69	0	3	0	0	35	0	22	0	33
0	0	0	9	0	0	0	0	0	0	12	0	17	10	0	0	34	56	0
0	0	62	32	0	24	10	0	43	0	0	0	16	0	15	0	0	11	0
0	21	0	4	0	0	0	0	0	0	9	0	13	0	36	6	20	25	17
0	0	0	0	89	0	0	0	0	36	0	12	0	0	0	0	0	0	13
78	0	0	12	0	0	15	0	0	6	0	0	0	0	0	0	0	30	0
0	0	0	0	0	73	0	0	0	0	0	21	22	29	0	0	0	11	31
0	0	26	27	0	31	0	0	6	6	0	19	15	0	0	0	0	5	0
0	0	0	0	0	0	0	17	0	0	0	0	0	0	0	0	0	3	21
0	0	0	0	0	4	0	0	11	0	4	11	0	0	0	0	10	0	0
25	0	12	0	0	0	0	0	0	0	0	0	5	0	15	0	0	5	0
0	2	0	0	0	0	0	0	0	0	27	0	14	0	0	0	0	0	5
0	0	0	0	0	0	9	0	0	0	0	91	0	0	0	3	0	0	0
0	0	0	0	0	4	0	0	0	6	19	0	0	0	0	0	12	0	0

0	0	0	0	0	0	4	0	0	0	0	0	0	0	0	0	0	0	0
0	0	0	0	0	0	0	0	0	0	0	0	0	0	0	0	0	7	0
0	0	0	0	0	0	0	0	0	0	0	0	8	0	0	0	0	0	0
0	0	0	0	0	0	0	0	0	0	0	0	0	0	0	0	10	0	0
0	0	0	0	0	0	0	0	0	0	0	0	0	0	0	0	0	0	0
0	0	0	0	0	0	0	0	0	0	0	13	0	0	0	0	0	11	0
0	0	0	0	0	0	0	0	0	0	0	0	0	0	0	0	0	0	0
7	0	0	0	0	0	14	0	0	8	0	0	0	0	0	0	0	0	0
0	0	0	0	0	0	0	0	20	0	0	0	0	0	0	0	0	0	0
0	0	0	0	0	0	0	0	0	0	0	0	16	0	0	0	0	0	0
0	0	0	0	0	0	0	0	0	0	0	0	6	0	0	0	0	10	0
0	0	0	0	0	0	0	0	0	0	0	0	0	0	0	0	0	0	0
0	0	0	0	0	0	0	0	0	0	0	0	0	19	0	5	0	0	0
0	0	0	0	0	0	0	0	0	0	0	53	0	0	0	0	0	0	0
0	0	0	0	0	13	0	0	0	0	0	0	0	0	0	0	0	0	0
0	0	0	0	0	0	0	0	0	0	0	0	0	0	0	0	0	0	0
0	0	0	0	0	0	0	0	0	0	0	0	0	0	0	0	0	0	0
0	0	0	0	0	0	0	0	0	0	0	0	14	0	0	0	0	0	0
0	0	0	0	0	0	0	0	0	0	0	0	0	0	0	0	0	0	0
0	0	0	0	0	0	0	0	0	0	0	0	0	0	0	0	0	0	0
0	0	0	0	23	0	0	0	0	0	0	0	0	0	0	0	0	0	0
0	0	0	0	0	0	0	0	0	0	0	0	0	0	0	0	7	0	13
0	0	0	0	0	0	0	5	5	0	0	0	0	0	0	0	0	0	0
0	0	0	0	0	0	0	0	0	0	0	0	0	0	37	0	0	0	0
0	0	0	0	0	0	0	0	0	0	0	0	0	0	0	0	0	0	0
0	0	0	0	0	0	0	0	0	0	0	0	0	0	0	0	0	0	0
0	0	0	0	0	0	0	0	0	0	0	0	0	0	0	0	0	0	0
4	0	0	0	0	0	0	0	0	0	0	0	0	0	0	0	0	0	0
0	0	0	0	0	0	0	0	0	0	0	0	0	0	0	0	0	0	0
0	0	0	0	0	0	0	0	0	0	0	0	0	0	0	0	0	0	0
0	0	0	0	0	0	0	5	0	0	0	0	0	0	0	0	0	0	0
0	0	0	0	0	0	0	0	0	0	0	0	0	0	0	0	0	0	0
0	0	0	0	0	0	0	0	0	0	0	0	0	0	0	0	0	8	0
0	0	0	0	0	0	0	0	0	0	0	0	0	0	0	0	0	0	0
0	0	0	0	0	0	0	0	0	0	0	0	0	0	0	0	0	0	0
0	0	0	0	0	0	0	0	0	0	0	0	0	0	0	0	0	2	0
0	0	0	0	0	0	0	0	0	0	0	0	0	0	0	0	0	0	0

0	0	0	0	0	0	0	0	0	0	0	0	0	0	0	0	0	0	0
0	0	0	0	0	0	0	0	0	0	0	0	0	0	0	0	0	0	0
0	0	0	0	0	0	0	0	9	0	0	0	0	0	0	0	0	0	0
0	0	0	0	0	0	0	0	0	0	0	0	0	0	0	0	0	0	0
0	0	0	0	0	0	0	0	0	0	0	0	0	0	0	0	0	0	0
0	0	0	0	0	0	0	0	0	0	0	0	0	0	0	0	0	0	0
0	0	0	0	0	0	0	0	0	0	0	0	0	0	0	0	0	0	0
0	0	0	0	0	0	0	0	0	0	0	0	0	0	0	0	0	0	0
0	0	0	0	0	0	0	0	0	0	0	0	0	0	0	0	0	0	0
0	0	0	0	0	0	0	0	0	0	0	0	0	0	0	0	0	0	0
0	0	0	0	0	0	0	0	0	0	0	0	0	0	0	0	0	0	0
0	0	0	0	0	0	0	0	0	0	0	0	0	0	0	0	0	0	0
0	0	0	0	0	0	0	0	0	0	0	0	0	0	0	0	0	0	0
0	0	0	0	0	0	0	0	0	0	0	0	0	0	0	0	0	0	0
0	0	0	0	0	0	0	0	0	0	0	0	0	0	0	0	0	0	0
0	0	0	0	0	0	0	0	0	0	0	0	0	0	0	0	0	0	0
0	0	0	0	0	0	0	0	0	0	0	0	0	0	0	0	0	0	0
0	0	0	0	0	0	0	0	0	0	0	0	0	0	0	0	0	0	0
0	0	0	0	0	0	0	0	0	0	0	0	0	0	0	0	0	0	0
0	0	0	0	0	0	0	0	0	0	0	0	0	0	0	0	0	0	0
0	0	0	0	0	0	0	0	0	0	0	0	0	0	0	0	0	0	0
0	0	0	0	0	0	0	0	0	0	0	0	0	0	0	0	0	0	0
0	0	0	0	0	0	5	0	0	0	0	0	0	0	0	0	0	0	0
0	0	0	0	0	0	0	0	0	0	0	0	0	0	0	0	0	0	0
0	0	0	0	0	0	0	0	0	0	0	0	0	0	0	0	0	0	0
0	0	0	0	0	0	0	0	0	0	0	0	0	0	0	0	0	0	0
0	0	0	0	0	0	0	0	0	0	0	0	0	0	0	0	0	0	0
0	0	0	0	0	0	0	0	0	0	0	0	0	0	0	0	0	0	0
0	0	0	0	0	0	0	0	0	0	0	0	0	0	0	0	0	0	0
0	0	0	0	0	0	0	0	0	0	0	0	0	0	0	0	0	0	0
0	0	0	0	0	0	0	0	0	0	3	0	0	0	0	0	0	0	0
0	0	0	0	0	0	0	0	0	0	0	0	0	0	0	0	0	0	0
0	0	0	0	0	0	0	0	0	0	0	0	0	0	0	0	0	0	0
0	0	0	0	0	0	0	0	0	0	0	0	0	0	0	0	0	0	0
0	0	0	0	0	0	0	0	0	0	0	0	0	0	0	0	0	0	0
0	0	0	0	0	0	0	0	7	0	0	0	0	0	0	0	0	0	0
0	0	0	0	0	0	0	0	0	0	0	0	0	0	0	0	0	0	0

0	0	0	0	0	0	0	0	0	0	0	0	0	0	0	0	0	0	0
0	0	0	0	0	0	0	0	0	0	0	0	0	0	0	0	0	0	0
0	0	0	0	0	0	0	0	0	0	0	0	0	0	0	0	0	0	0
0	0	0	0	0	0	0	0	0	0	0	0	0	0	0	0	0	0	0
0	0	0	0	0	0	0	0	0	0	0	0	0	0	0	0	0	0	0
0	0	0	0	0	0	0	0	0	0	0	0	0	0	0	0	0	0	0
0	0	0	0	0	0	0	0	0	0	0	0	0	0	0	0	0	0	0
0	0	0	0	0	0	0	0	0	0	0	0	0	0	0	0	0	0	0
0	0	0	0	0	0	0	0	0	0	0	0	0	0	0	0	0	0	0
0	0	0	0	0	0	0	0	0	0	0	0	0	0	0	0	0	0	0
0	0	0	0	0	0	0	0	0	0	0	0	0	0	0	0	0	0	0
0	0	0	0	0	0	0	0	0	0	0	0	0	0	0	0	0	0	0
0	0	0	0	0	0	0	0	0	0	0	0	0	0	0	0	0	0	0
0	0	0	0	0	0	0	0	0	0	0	0	0	0	0	0	0	0	0
0	0	0	0	0	0	0	0	0	0	0	0	0	0	0	0	0	0	0
0	0	0	0	0	0	0	0	0	0	0	0	0	0	0	0	0	0	0
0	0	0	0	0	0	0	0	0	0	0	0	0	0	0	0	0	0	0
0	0	0	0	0	0	0	0	0	0	0	0	0	0	0	0	0	0	0
0	0	0	0	0	0	0	0	0	0	0	0	0	0	0	0	0	0	0
0	0	0	0	0	0	0	0	0	0	0	0	0	0	0	0	0	0	0
0	0	0	0	0	0	0	0	0	0	0	0	0	0	0	0	0	0	0
0	0	0	0	0	0	0	0	0	0	0	0	0	0	0	0	0	0	0
0	0	0	0	0	0	0	0	0	0	0	0	0	0	0	0	0	0	0
0	0	0	0	0	0	0	0	0	0	0	0	0	0	0	0	0	0	0
4	0	0	0	0	0	0	0	0	0	0	0	0	0	0	0	0	0	0
0	0	0	0	0	0	0	0	0	0	0	0	0	0	0	0	0	0	0
0	0	0	0	0	0	0	0	0	0	0	0	0	0	0	0	0	0	0
0	0	0	0	0	0	0	0	0	0	0	0	0	0	0	0	0	0	0
0	0	0	0	0	0	0	0	0	0	0	0	0	0	0	0	0	0	0
0	0	0	0	0	0	0	0	0	0	0	0	0	0	0	0	0	0	0
0	0	0	0	0	0	0	0	0	0	0	0	0	0	0	0	0	0	0
0	0	0	0	0	0	0	0	0	0	0	0	0	0	0	0	0	0	0
0	0	0	0	0	0	0	0	0	0	0	0	0	0	0	0	0	0	0
0	0	0	0	0	0	0	0	0	0	0	0	0	0	0	0	0	0	0
0	0	0	0	0	0	0	0	0	0	0	0	0	0	0	0	0	0	0
0	0	0	0	0	0	0	0	0	0	0	0	0	0	0	0	0	0	0
0	0	0	0	0	0	0	0	0	0	0	0	0	0	0	0	0	0	0

0	0	0	0	0	0	0	0	0	0	0	0	0	0	0	0	0	0	0
0	0	0	0	0	0	0	0	0	0	0	0	0	0	0	0	0	0	0
0	0	0	0	0	0	0	0	0	0	0	0	0	0	0	0	0	0	0
0	3	0	0	0	0	0	0	0	0	0	0	0	0	0	0	0	0	0
0	0	0	0	0	0	0	0	0	0	0	0	0	0	0	0	0	0	0
0	0	0	0	0	0	0	0	0	0	0	0	0	0	0	0	0	0	0
0	0	0	0	0	0	0	0	0	0	0	0	0	0	0	0	0	0	0
0	0	0	0	0	0	0	0	0	0	0	0	0	0	0	0	0	0	0
0	0	0	0	0	0	0	0	0	0	0	0	0	0	0	0	0	0	0
0	0	0	0	0	0	0	0	0	0	0	0	0	0	0	0	0	0	0
0	0	0	0	0	0	0	0	0	0	0	0	0	0	0	0	0	0	0
0	0	0	0	0	0	0	0	0	0	0	0	0	0	0	0	0	0	0
0	0	0	0	0	0	0	0	0	0	0	0	0	0	0	0	0	0	0
0	0	0	0	0	0	0	0	0	0	0	0	0	0	0	0	0	0	0
0	0	0	0	0	0	0	0	0	0	0	0	0	0	0	0	0	0	0
0	0	0	0	0	0	0	0	0	0	0	0	0	0	0	0	0	0	0
0	0	0	0	0	0	0	0	0	0	0	0	0	0	0	0	0	0	0
0	0	0	0	0	0	0	0	0	0	0	0	0	0	0	0	0	0	0
0	0	0	0	0	0	0	0	0	0	0	0	0	0	0	0	0	0	0
0	0	0	0	0	0	0	0	0	0	0	0	0	0	0	0	0	0	0
0	0	0	0	0	0	0	0	0	0	0	0	0	0	0	0	0	0	0
0	0	0	0	0	0	0	0	0	0	0	0	0	0	0	0	0	0	0
0	0	0	0	0	0	0	0	0	0	0	0	0	0	0	0	0	0	0
0	0	0	0	0	0	0	0	0	0	0	0	0	0	0	0	0	0	0
0	0	0	0	0	0	0	0	0	0	0	0	0	0	0	0	0	0	0
0	0	0	0	0	0	0	0	0	0	0	0	0	0	0	0	0	0	0
0	0	0	0	0	0	0	0	0	0	0	0	0	0	0	0	0	0	0
0	0	0	0	0	0	0	0	0	0	0	0	0	0	0	0	0	0	0
0	0	0	0	0	0	0	0	0	0	0	0	0	0	0	0	0	0	0
0	0	0	0	0	0	0	0	0	0	0	0	0	0	0	0	0	0	0
0	0	0	0	0	0	0	0	0	0	0	2	2	2	0	0	0	0	0
0	0	0	0	0	0	0	0	0	0	0	0	0	0	0	0	0	0	0
0	0	0	0	0	0	0	0	0	0	0	0	0	0	0	0	0	0	0
0	0	0	0	0	0	0	0	0	0	0	0	0	0	0	0	0	0	0
0	0	0	0	0	0	0	0	0	0	0	0	0	0	0	0	0	0	0
0	0	0	0	0	0	0	0	0	0	0	0	0	0	0	0	0	0	0
0	0	0	0	0	0	0	0	0	0	0	0	0	0	0	0	0	0	0

0	0	0	0	0	0	0	0	0	0	0	0	0	0	0	0	0	0	0
0	0	0	0	0	0	0	0	0	0	0	0	0	0	0	0	0	0	0
0	0	0	0	0	0	0	0	0	0	0	0	0	0	0	0	0	0	0
0	0	0	0	0	0	0	0	0	0	0	0	0	0	0	0	0	0	0
0	0	0	0	0	0	0	0	0	0	0	0	0	0	0	0	0	0	0
0	0	0	0	0	0	0	0	0	0	0	0	0	0	0	0	0	0	0
0	0	0	0	0	0	0	0	0	0	0	0	0	0	0	0	0	0	0
0	0	0	0	0	0	0	0	0	0	0	0	0	0	0	0	0	0	0
0	0	0	0	0	0	0	0	0	0	0	0	0	0	0	0	0	0	0
0	0	0	0	0	0	0	0	0	0									
Jan_DL039	1439	1665	763	996	111	421	397	345	172	344	148	220	324	102	172	116	127	149
122	35	340	551	332	277	65	97	44	105	92	0	128	120	172	62	74	149	0
84	105	0	39	170	81	149	7	54	28	43	28	36	0	83	21	0	38	0
0	11	0	29	55	114	130	0	41	194	13	108	11	120	37	5	94	6	18
31	0	67	15	24	27	21	33	44	60	56	67	54	0	32	0	141	24	0
9	0	0	0	0	0	0	0	0	124	0	0	0	0	8	37	0	0	59
0	0	26	52	3	21	0	0	0	0	0	76	10	21	0	0	0	85	0
0	0	0	30	0	0	149	0	64	0	53	0	41	0	20	20	0	0	0
0	20	0	0	0	0	0	0	0	0	12	0	0	0	36	13	20	0	18
13	0	0	0	12	56	22	9	0	50	28	0	0	0	0	0	0	13	0
0	0	25	0	0	0	0	30	0	0	11	0	18	0	0	0	0	28	0
10	0	0	0	16	0	0	0	0	0	0	18	23	0	0	0	0	0	0
0	0	18	0	0	52	0	0	17	20	0	0	7	9	13	12	0	0	0
24	0	0	30	0	0	0	24	0	0	0	0	0	12	0	0	0	14	0
45	0	0	0	0	9	0	0	0	0	5	7	0	9	11	6	25	0	0
8	0	22	13	0	0	0	0	0	0	0	0	12	0	34	22	0	0	0
0	5	7	0	0	0	0	0	0	0	0	0	13	0	21	0	0	111	7
24	0	0	8	0	34	0	0	0	0	0	0	17	0	0	0	0	0	0
0	5	0	0	0	4	0	0	9	0	0	0	7	10	0	0	16	0	0
0	0	12	0	0	0	0	0	0	0	0	0	0	0	0	0	0	0	12
11	0	0	0	0	0	0	0	0	0	0	0	6	0	12	0	0	46	0
0	0	0	0	0	0	0	0	86	0	0	0	0	0	0	0	0	0	0
0	0	10	0	0	0	0	0	0	0	0	0	0	0	4	0	28	0	0
0	0	0	0	0	0	0	0	0	0	0	0	0	0	0	0	0	6	0
0	0	0	63	6	0	0	0	0	0	0	0	0	0	0	0	0	0	0
0	0	0	0	0	0	0	3	0	7	0	0	0	0	0	3	0	0	0
7	0	0	0	0	6	0	0	0	0	0	0	0	0	0	0	0	0	0

0	0	0	0	0	0	0	0	31	6	0	0	0	0	0	0	0	0	0
0	0	0	0	0	0	0	0	0	0	13	0	0	0	0	0	0	0	0
0	0	0	0	0	0	0	0	0	0	0	0	0	0	0	0	0	0	0
0	0	0	0	0	0	33	74	0	0	0	0	7	0	21	0	0	0	0
0	0	0	0	0	0	0	0	0	0	0	0	0	0	9	0	0	0	0
0	0	17	0	0	4	0	0	0	0	0	25	0	0	0	29	0	0	0
0	0	0	0	0	0	14	0	0	0	0	0	0	0	8	0	0	0	0
0	0	0	0	0	0	0	0	21	0	0	0	0	0	0	0	0	0	0
3	0	0	0	0	0	0	0	0	0	0	0	0	0	0	0	0	0	0
0	0	7	0	15	0	0	0	0	0	0	0	0	0	0	0	0	0	0
0	0	0	0	0	0	0	0	0	0	0	0	0	0	0	0	0	0	0
0	0	0	0	0	0	0	0	0	0	0	0	0	0	0	0	0	0	0
0	0	0	0	0	0	0	0	0	0	0	0	0	0	0	0	0	0	0
71	18	19	0	0	0	0	0	0	0	0	0	0	0	0	0	0	0	0
9	0	0	0	0	0	0	0	6	0	0	0	0	0	0	0	0	0	4
0	0	0	0	0	0	0	0	0	0	0	0	0	0	4	0	0	0	0
0	0	0	0	0	0	0	0	0	0	0	0	0	0	0	0	0	0	0
0	0	0	0	0	0	0	0	0	0	0	0	0	0	0	0	0	0	0
0	0	0	0	0	0	0	0	0	0	0	0	0	0	15	0	0	0	0
0	0	0	0	0	0	9	0	0	0	0	0	0	0	0	0	0	0	0
0	0	0	0	0	0	0	0	0	0	0	0	0	0	0	0	0	0	0
0	0	0	0	0	0	0	0	0	0	0	0	0	0	0	0	0	0	0
0	0	0	0	0	0	0	0	0	0	0	0	0	0	0	0	0	0	0
0	0	0	0	0	0	0	0	0	0	0	0	0	0	0	0	0	0	0
0	0	0	0	0	0	0	0	0	0	0	0	0	0	0	0	0	0	0
0	8	0	0	0	0	0	0	0	0	0	0	0	0	0	0	0	0	0
0	0	0	0	0	0	0	0	0	0	0	0	0	0	0	0	0	0	0
0	0	0	0	0	0	0	0	0	0	0	0	0	0	0	0	0	0	0
0	0	0	9	0	0	0	0	0	0	0	0	0	0	0	0	0	0	0
0	0	0	0	0	0	0	0	0	0	0	0	0	0	0	0	0	0	0
0	0	0	0	0	5	0	0	0	0	0	0	0	0	0	0	0	0	0
0	0	0	0	0	0	0	0	0	0	0	0	0	0	0	0	0	0	0
0	0	0	0	0	0	0	0	0	0	0	0	0	0	0	0	0	0	0
14	0	0	0	0	0	0	0	0	0	0	0	0	0	0	0	0	0	0
0	0	0	0	0	4	0	0	0	0	0	0	0	0	0	0	0	0	0
0	0	0	0	0	0	0	0	0	0	0	0	0	0	0	0	0	0	0
0	0	0	0	0	0	0	0	0	0	0	0	0	0	0	0	0	17	0

0	0	0	0	0	0	0	0	0	0	0	0	0	0	0	0	0	0	0
0	0	0	0	0	0	0	0	0	0	0	0	0	0	0	0	0	0	0
0	0	0	0	0	0	0	0	0	0	0	0	0	0	0	0	0	0	0
0	0	0	0	0	0	0	0	0	0	0	0	0	0	0	0	0	0	0
0	0	0	0	0	0	0	0	0	0	0	0	0	0	0	0	0	0	0
0	0	0	0	0	0	0	0	0	0	0	0	0	0	0	0	6	0	0
0	0	0	0	0	0	0	0	0	0	0	0	0	0	0	0	0	0	0
0	0	0	0	0	0	0	0	0	0	0	0	0	0	0	0	0	0	0
0	0	0	0	0	0	0	0	0	0	0	0	0	0	0	0	0	0	0
0	0	0	0	0	0	0	0	0	0	0	0	0	0	0	0	0	0	0
0	0	0	0	0	11	0	0	0	0	0	0	0	0	0	0	0	0	0
0	0	0	0	0	0	0	0	0	0	0	0	0	0	0	0	0	0	0
0	0	0	0	0	0	0	0	0	0	0	0	0	0	0	0	0	0	0
0	0	0	0	0	2	0	0	0	0	10	0	0	0	0	0	0	0	0
0	0	0	0	0	0	0	0	0	0	0	0	0	0	0	0	0	0	0
0	0	0	0	0	0	0	0	0	0	0	0	0	0	0	0	0	0	0
0	0	0	0	0	0	0	0	0	0	0	0	0	0	0	0	0	0	0
0	0	0	0	0	0	0	0	0	0	0	0	0	0	0	0	0	0	0
0	0	0	0	0	0	0	0	0	0	0	0	0	0	0	0	0	0	0
0	0	0	0	0	0	0	0	0	0	0	0	0	0	0	0	0	0	0
0	0	0	0	0	0	0	0	0	0	0	0	0	0	8	0	0	0	0
0	0	0	0	0	0	0	0	0	0	0	0	0	0	0	0	0	0	0
0	0	0	0	0	0	0	0	0	0	0	0	0	0	0	0	0	0	0
0	0	0	0	0	0	0	0	0	0	0	0	0	0	0	0	0	0	0
0	0	0	0	0	0	0	0	0	0	0	0	0	0	0	0	0	0	0
0	0	0	0	0	0	0	0	0	0	0	0	0	0	0	0	0	0	0
0	0	0	0	0	0	0	0	0	0	0	0	0	0	0	0	0	0	0
0	0	0	0	0	0	0	0	0	0	0	0	0	0	0	0	0	0	0
0	0	0	0	0	0	0	0	0	0	0	0	0	0	0	0	0	0	0
0	0	0	0	0	0	0	0	0	0	0	0	0	0	0	0	0	0	0
0	0	0	0	0	0	0	0	0	0	0	0	0	0	0	0	0	0	0
0	0	0	0	0	0	0	0	0	0	0	0	0	0	0	0	0	0	0
0	0	0	0	0	0	0	0	0	0	0	0	0	0	0	0	0	0	0
0	0	0	0	0	0	0	0	0	0	0	0	0	0	0	0	0	0	0
0	0	0	0	0	0	0	0	0	0	0	0	0	0	0	0	0	0	0
0	0	0	0	0	0	0	0	0	0	0	0	0	0	0	0	0	0	0
0	0	0	0	0	0	0	0	0	0	0	0	0	0	0	0	0	0	0

0	0	0	0	0	0	0	0	0	0	0	0	0	0	0	0	0	0	0
0	0	0	0	0	0	0	0	0	0	0	0	0	0	0	0	0	0	0
0	0	0	0	0	0	0	0	0	0	0	0	0	0	0	0	0	0	0
0	0	0	0	0	0	0	0	0	0	0	0	0	0	0	0	0	0	0
0	0	0	0	0	0	0	0	0	0	0	0	0	0	0	0	0	0	0
0	0	0	0	0	0	0	0	0	0	0	0	0	0	0	0	0	0	0
0	0	0	0	0	0	0	0	0	0	0	0	0	0	0	0	0	0	0
0	0	0	0	0	0	0	0	0	0	0	0	0	0	0	0	0	0	0
0	0	0	0	0	0	0	0	0	0	0	0	0	0	0	0	0	0	0
0	0	0	0	0	0	0	0	0	0	0	0	0	0	0	0	0	0	0
0	0	0	0	0	0	0	0	0	0	0	0	0	0	0	0	0	0	0
0	0	0	0	0	0	0	0	0	0	0	0	0	0	0	0	0	0	0
0	0	0	0	0	0	0	0	0	0	0	0	0	0	0	0	0	0	0
0	0	0	0	0	0	0	0	0	0	0	0	0	0	0	0	0	0	0
0	0	0	0	0	0	0	0	0	0	0	0	0	0	0	0	0	0	4
0	0	0	0	0	0	0	0	0	0	0	0	0	0	0	0	0	0	0
0	0	0	0	0	0	0	0	0	0	0	0	0	0	0	0	0	0	0
0	0	0	0	0	0	0	0	0	0	0	0	0	0	0	0	0	0	0
0	0	0	0	0	0	0	0	0	0	0	0	0	0	0	0	0	0	0
0	0	0	0	0	0	0	0	0	0	0	0	0	0	0	0	0	0	0
0	0	0	0	0	0	0	0	0	0	0	0	0	0	0	0	0	0	0
0	0	0	0	0	0	0	0	0	0	0	0	0	0	0	0	0	0	0
0	0	0	0	0	0	0	0	0	0	0	0	0	0	0	0	0	0	0
0	0	0	0	0	0	0	0	0	0	0	0	0	0	0	0	0	0	0
0	0	0	0	0	0	0	0	0	0	0	0	0	0	0	0	0	0	0
0	0	0	0	0	0	0	0	0	0	0	0	0	0	0	0	0	0	0
0	0	0	0	0	0	0	0	0	0	0	0	0	0	0	0	0	0	0
0	0	0	0	0	0	0	0	0	0	0	0	0	0	0	0	0	0	0
0	0	0	0	0	0	0	0	0	0	0	0	0	0	0	0	0	0	0
0	0	0	0	0	0	0	0	0	0	0	0	0	0	0	0	0	0	0
0	0	0	0	0	0	0	0	0	0	0	0	0	0	0	0	0	0	0
0	0	0	0	0	0	0	0	0	0	3	0	0	0	0	0	0	0	0
0	0	0	0	0	0	0	0	0	0	0	0	0	0	0	0	0	0	0
0	0	0	0	0	0	0	0	0	0	0	0	0	0	0	0	0	0	0
0	0	0	0	0	0	0	0	0	0	0	0	0	0	0	0	0	0	0
0	0	0	0	0	0	0	0	0	0	0	0	0	0	0	0	0	0	0
0	0	0	0	0	0	0	0	0	0	0	0	0	0	0	0	0	0	0

0	0	0	0	0	0	0	0	0	0	0	0	0	0	0	0	0	0	0
0	0	0	0	0	0	0	0	0	0	0	0	0	0	0	0	0	0	0
0	0	0	0	0	0	0	0	0	0	0	0	0	0	0	0	0	0	0
0	0	0	0	0	0	0	0	0	0	0	0	0	0	0	0	0	0	0
0	0	0	0	0	0	0	0	0	0	0	0	0	0	0	0	0	0	0
0	0	0	0	0	0	0	0	0	0	0	0	0	0	0	0	0	0	0
0	0	0	0	0	0	0	0	0	0	0	0	0	0	0	0	0	0	0
0	0	0	0	0	0	0	0	0	0	0	0	0	0	0	0	0	0	0
0	0	0	0	0	0	0	0	0	0	0	0	0	0	0	0	0	0	0
0	0	0	0	0	0	0	0	0	0	0	0	0	0	0	0	0	0	0
0	0	0	0	0	0	0	0	0	0	0	0	0	0	0	0	0	0	0
0	0	0	0	0	0	0	0	0	0	0	0	0	0	0	0	0	0	0
0	0	0	0	0	0	0	0	0	0	0	0	0	0	0	0	0	0	0
0	0	0	0	0	0	0	0	0	0	0	0	0	0	0	0	0	0	0
0	0	0	0	0	0	0	0	0	0	0	0	0	0	0	0	0	0	0
0	0	0	0	0	0	0	0	0	0	0	0	0	0	0	0	0	0	0
0	0	0	0	0	0	0	0	0	0	0	0	0	0	0	0	0	0	0
0	0	0	0	0	0	0	0	0	0	0	0	0	0	0	0	0	0	0
0	0	0	0	0	0	0	0	0	0	0	0	0	0	0	0	0	0	0
0	0	0	0	0	0	0	0	0	0	0	0	0	0	0	0	0	0	0
0	0	0	0	0	0	0	0	0	0	0	0	0	0	0	0	0	0	0
0	0	0	0	0	0	0	0	0	0	0	0	0	0	0	0	0	0	0
0	2	0	0	0	0	0	0	0	0	0	0	0	0	0	0	0	0	0
0	0	0	0	0	0	0	0	0	0	0	0	0	0	0	0	0	0	0
0	0	0	0	0	0	0	0	0	0	0	0	0	0	0	0	0	0	0
0	0	0	0	0	0	0	0	0	0	0	0	0	0	0	0	0	0	0
0	0	0	0	0	0	0	0	0	0	0	0	0	0	0	0	0	0	0
0	0	0	0	0	0	0	0	0	0	0	0	0	0	0	0	0	0	0
0	0	0	0	0	0	0	0	0	0	0	0	0	0	0	0	0	0	0
0	0	0	0	0	0	0	0	0	0	0	0	0	0	0	0	0	0	0
0	0	0	0	0	0	0	0	0	0	0	0	0	0	0	0	0	0	0
0	0	0	0	0	0	0	0	0	0	0	0	0	0	0	0	0	0	0
0	0	0	0	0	0	0	0	0	0	0	0	0	0	0	0	0	0	0
0	0	0	0	0	0	0	0	0	0	0	0	0	0	0	0	0	0	0
0	0	0	0	0	0	0	0	0	0	0	0	0	0	0	0	0	0	0
0	0	0	0	0	0	0	0	0	0	0	0	0	0	0	0	0	0	0
0	0	0	0	0	0	0	0	0	0	0	0	0	0	0	0	0	0	0

0	0	0	0	0	0	0	0	0	0	0	0	0	0	0	0	0	0	0
0	0	0	0	0	0	0	0	0	0									
Jan_DL004	1865	2404	2044	711	79	42	86	64	235	1032	56	680	854	33	303	327	53	136
174	294	481	0	180	379	707	206	427	437	72	0	139	529	352	38	161	121	0
383	240	41	59	139	29	66	1082	31	126	40	148	92	74	71	147	303	67	0
0	47	149	21	27	71	56	129	409	27	45	0	141	60	0	0	5	86	11
180	89	49	124	0	165	117	0	68	141	56	0	56	58	0	176	64	29	62
25	56	0	0	125	0	219	0	0	44	0	11	0	41	61	0	0	33	7
0	0	0	14	15	15	0	0	0	0	18	11	20	0	0	0	0	0	0
0	34	0	0	9	206	0	0	0	0	8	0	31	0	0	8	0	0	0
0	0	45	3	0	4	39	110	45	113	12	0	0	34	0	22	0	0	13
55	55	17	10	0	0	106	0	0	20	0	24	0	0	0	0	7	0	0
0	0	0	27	142	0	87	0	63	0	0	0	0	0	0	110	0	0	0
0	0	0	0	0	0	0	0	0	0	0	0	0	0	0	0	123	14	0
0	0	0	0	0	0	0	0	10	22	0	27	0	0	69	0	0	0	0
0	0	0	0	0	0	0	0	0	11	57	35	0	0	0	0	0	0	0
0	0	0	0	0	0	63	0	19	0	0	8	0	0	6	0	0	77	0
0	0	26	0	0	0	0	0	5	89	0	49	0	102	0	0	12	0	0
0	5	9	0	0	0	0	5	0	0	0	0	0	0	0	18	100	0	0
0	0	8	5	0	0	0	0	0	0	0	0	0	0	0	17	0	0	0
33	0	0	0	8	0	0	17	0	0	0	0	41	0	17	65	19	0	0
0	0	0	0	13	0	12	0	0	0	0	102	4	69	0	15	38	0	0
0	0	0	52	12	0	0	0	0	0	24	0	0	0	11	0	0	0	0
0	0	0	0	0	0	0	0	0	10	0	0	0	0	0	0	0	0	0
0	90	0	0	0	0	0	0	9	0	0	0	26	0	49	0	0	0	0
0	0	0	0	0	0	0	0	0	0	0	0	0	0	0	0	0	0	18
5	0	0	0	5	0	0	0	0	0	0	0	0	0	0	0	0	0	0
111	0	0	0	13	0	0	24	60	0	0	0	0	0	0	14	0	0	0
0	0	18	0	0	0	0	0	0	0	0	0	0	0	0	0	0	0	0
0	0	0	0	0	0	0	3	0	0	0	0	0	0	0	0	0	0	194
0	0	0	0	0	56	0	24	0	0	0	0	6	0	0	0	185	0	0
0	0	0	0	0	0	0	0	0	0	0	0	0	0	0	0	0	0	0
13	4	0	0	0	0	0	36	0	0	103	0	0	0	0	0	0	0	0
8	0	63	0	0	0	0	0	0	0	0	0	0	0	0	6	0	0	0
0	0	0	0	0	0	0	0	0	0	0	0	0	0	0	0	0	0	0
0	0	0	0	0	0	0	0	24	0	0	0	0	0	0	0	0	0	0
0	0	6	0	20	0	0	0	0	0	0	0	6	0	0	0	0	0	0

0	0	0	0	0	0	0	0	0	0	0	0	0	0	0	0	7	0	0
0	0	0	0	0	0	0	0	0	0	0	0	0	0	0	0	0	0	0
0	14	0	0	0	0	0	0	0	0	0	0	0	0	16	0	0	0	0
0	0	0	0	0	0	0	0	0	0	0	0	0	0	0	0	0	0	0
0	0	0	0	0	0	0	0	0	0	0	0	0	0	0	0	0	0	0
0	0	0	0	16	0	0	0	0	0	0	0	0	0	0	0	18	0	14
3	0	0	0	0	0	0	0	0	0	0	0	0	0	0	0	0	0	0
0	0	0	0	0	0	16	0	0	0	0	0	0	0	0	0	0	0	0
0	0	0	0	0	0	0	0	14	0	0	0	0	0	0	0	0	0	18
0	0	0	0	0	0	0	0	0	0	0	0	0	0	0	0	0	0	0
0	0	0	0	0	0	5	0	0	0	0	0	0	0	0	0	0	0	0
0	0	0	0	0	0	0	0	0	8	0	40	0	0	0	0	0	0	0
0	0	0	0	0	0	0	0	0	0	0	75	0	0	0	0	0	0	0
0	0	0	0	0	0	0	0	0	0	0	0	0	0	0	0	0	0	0
0	16	0	0	0	0	0	0	0	0	0	0	0	0	0	0	0	0	0
0	0	10	0	0	0	0	0	0	0	0	0	0	0	0	0	0	23	0
0	0	0	0	0	0	0	0	0	0	0	0	0	0	0	0	0	0	0
9	0	0	0	0	0	0	0	0	0	0	0	0	0	0	0	0	0	0
0	0	0	0	0	0	0	0	0	0	0	0	22	0	0	0	0	0	0
0	0	0	0	0	0	0	0	0	11	27	0	0	0	0	0	0	0	0
0	0	0	0	0	0	0	0	0	0	0	0	0	0	0	0	0	0	0
0	0	0	0	0	0	0	0	0	0	0	0	0	0	0	0	0	0	0
0	0	0	0	0	0	0	0	0	0	0	0	0	0	0	0	0	0	0
0	0	0	0	0	0	0	0	0	0	0	0	11	0	0	0	0	0	0
0	0	0	0	0	0	0	0	0	0	0	0	0	0	0	0	0	0	0
0	0	0	0	0	0	0	0	0	0	0	0	0	0	0	0	0	0	0
0	0	0	0	0	0	0	0	2	0	0	0	0	0	0	0	0	0	0
0	0	0	0	0	0	0	0	30	0	0	0	0	0	0	0	0	0	0
0	0	0	0	0	0	0	0	0	0	0	0	0	0	0	0	0	0	0
0	0	0	0	0	0	0	0	0	0	0	0	0	0	0	0	0	0	0
0	0	0	0	0	0	0	0	0	0	0	0	0	0	0	0	0	0	0
0	0	0	0	0	0	0	0	0	0	0	0	0	0	0	0	0	0	0
0	0	0	0	0	0	0	0	0	0	0	0	0	0	0	0	0	0	0
0	0	0	0	0	0	0	0	0	0	0	0	0	0	0	0	0	0	0
0	0	0	0	0	0	0	0	0	0	0	0	0	0	0	0	0	0	0
0	0	0	0	0	0	0	0	0	0	0	0	0	0	0	0	0	0	0
0	0	0	0	0	0	0	0	0	0	0	0	0	0	0	0	0	0	0

0	0	0	0	0	0	0	0	0	0	0	0	0	0	0	0	0	0	0
0	0	0	0	0	0	0	0	0	0	0	0	0	0	0	0	0	0	0
0	0	0	0	0	0	0	0	0	0	0	0	0	0	0	0	0	0	0
0	0	0	0	0	0	0	0	0	0	0	0	0	0	0	0	0	0	0
0	0	0	0	0	0	0	0	0	0	0	0	0	0	0	0	0	0	0
0	0	0	0	0	0	0	0	0	0	0	0	0	0	0	0	0	0	0
0	0	0	0	0	0	0	0	0	0	0	0	0	0	0	0	0	0	0
0	0	0	0	0	0	0	0	0	0	0	0	0	0	0	0	0	0	0
0	0	0	0	0	0	0	0	0	0	0	0	0	0	0	0	0	0	0
0	0	0	0	0	0	0	0	0	0	0	0	0	0	0	0	0	0	0
0	0	0	0	0	0	0	0	0	0	0	0	0	0	0	0	0	0	0
0	0	0	0	0	0	0	0	0	0	0	0	0	0	0	0	0	0	0
0	0	0	0	0	0	0	0	0	0	0	0	0	0	0	0	0	0	0
0	0	0	0	0	0	0	0	0	0	0	0	0	0	0	0	0	0	0
0	0	0	0	0	0	0	0	0	0	0	0	0	0	0	0	0	0	0
0	0	0	0	0	0	0	0	0	0	0	0	0	0	0	0	0	0	0
0	0	0	0	0	0	0	0	0	0	0	0	0	0	0	0	0	0	0
0	0	0	0	0	0	0	0	0	0	0	0	0	0	0	0	0	0	0
0	0	0	0	0	0	0	0	0	0	0	0	0	0	0	0	0	0	0
0	0	0	0	0	0	0	0	0	0	0	0	0	0	0	0	0	0	11
0	0	0	0	0	0	0	0	0	0	0	0	0	0	0	0	0	0	0
0	0	0	0	0	0	0	0	0	0	0	0	0	0	0	0	0	0	0
0	0	0	0	0	0	0	0	0	0	0	0	0	0	0	0	0	0	0
0	0	0	0	0	0	0	0	0	0	0	0	0	0	0	0	0	0	0
0	0	0	0	0	0	0	0	0	0	0	0	0	0	0	0	0	0	0
0	0	0	0	0	0	0	0	0	0	0	0	0	0	0	0	0	0	0
0	0	0	0	0	0	0	0	0	0	0	0	0	0	0	0	0	0	0
0	0	0	0	0	0	0	0	0	0	0	0	0	0	0	0	0	0	0
0	0	0	0	0	0	0	0	0	0	0	0	0	0	0	0	0	0	0
0	0	0	0	0	0	0	0	0	0	0	0	0	0	0	0	0	0	0
0	0	0	0	0	0	0	0	0	0	0	0	0	0	0	0	0	0	0
0	0	0	0	0	0	0	0	0	0	0	0	0	0	0	0	0	0	0
0	0	0	0	0	0	0	0	0	0	0	0	0	0	0	0	0	0	0
0	0	0	0	0	0	0	0	0	0	0	0	0	0	0	0	0	6	0
0	0	0	0	0	0	0	0	0	0	0	0	0	0	0	0	0	0	0
0	0	0	0	0	0	0	0	0	0	0	0	0	0	0	0	0	0	0
0	0	0	0	0	0	0	0	0	0	0	0	0	0	0	0	0	0	0

0	0	0	0	0	0	0	0	0	0	0	0	0	0	0	0	0	0	0
0	0	0	0	0	0	0	0	0	0	0	0	0	0	0	0	0	0	0
0	0	0	0	0	0	0	0	0	0	0	7	7	0	0	0	0	0	0
0	0	0	0	0	0	0	0	0	0	0	0	0	0	0	0	0	0	0
0	0	0	0	0	0	0	0	0	0	0	0	0	0	0	0	0	0	0
0	0	0	0	0	0	0	0	0	0	0	0	0	0	0	0	0	0	0
0	0	0	0	0	0	0	0	0	0	0	0	0	0	0	0	0	0	0
0	0	0	0	0	0	0	0	0	0	0	0	0	0	0	0	0	0	0
0	0	0	0	0	0	0	0	0	0	0	0	0	0	0	0	0	0	0
0	0	0	0	0	0	0	0	0	0	0	0	0	0	0	0	0	0	0
0	0	0	0	0	0	0	0	0	0	0	0	0	0	0	0	0	0	0
0	0	0	0	0	0	0	0	0	0	0	0	0	0	0	0	0	0	0
0	0	0	0	0	0	0	0	0	0	0	0	0	0	0	0	0	0	0
0	0	0	0	0	0	0	0	0	0	0	0	0	0	0	0	0	0	0
0	0	0	0	0	0	0	0	0	0	0	0	0	0	0	0	0	0	0
0	0	0	0	0	0	0	0	0	0	0	0	0	0	0	0	0	0	0
0	0	0	0	0	0	0	0	0	0	0	0	0	0	0	0	0	0	0
0	0	0	0	0	0	0	0	0	0	0	0	0	0	0	0	0	0	0
0	0	0	0	0	0	0	0	0	0	0	0	0	0	0	0	0	0	0
0	0	0	0	0	0	0	0	0	0	0	0	0	0	0	0	0	0	0
0	0	0	0	0	0	0	0	0	0	0	0	0	0	0	0	0	0	0
0	0	0	0	0	0	0	0	0	0	0	0	0	0	0	0	0	0	0
0	0	0	0	0	0	0	0	0	0	0	0	0	0	0	0	0	0	0
0	0	0	0	0	0	0	0	0	0	0	0	0	0	0	0	0	0	0
0	0	0	0	0	0	0	0	0	0	0	0	0	0	0	0	0	0	0
0	0	0	0	0	0	0	0	0	0	0	0	0	0	0	0	0	0	0
0	0	0	0	0	0	0	0	0	0	0	0	0	0	0	0	0	0	0
0	0	0	0	0	0	0	0	0	0	0	0	0	0	0	0	0	0	0
0	0	0	0	0	0	0	0	0	0	0	0	0	0	0	0	0	0	0
0	0	0	0	0	0	0	0	0	0	0	0	0	0	0	0	0	0	0
0	0	0	0	0	0	0	0	0	0	0	0	0	0	0	0	0	0	0
0	0	0	0	0	0	0	0	0	0	0	0	0	0	0	0	0	0	0
0	0	0	0	0	0	0	0	0	0	0	0	0	0	0	0	0	0	0
0	0	0	0	0	0	0	0	0	0	0	0	0	0	0	0	0	0	0
0	0	0	0	0	0	0	0	0	0	0	0	0	0	0	0	0	0	0
0	0	0	0	0	0	0	0	0	0	0	0	0	0	0	0	0	0	0
0	0	0	0	0	0	0	4	4	4	0	0	0	0	0	0	0	0	0

0	0	0	0	0	0	0	0	0	0	0	0	0	0	0	0	0	0	0
0	0	0	0	0	0	0	0	0	0	0	0	0	0	0	0	0	0	0
0	0	0	0	0	0	0	0	0	0	0	0	0	0	0	0	0	0	0
0	0	0	0	0	0	0	0	0	0	0	0	0	0	0	0	0	0	0
0	0	0	0	0	0	0	0	0	0	0	0	0	0	0	0	0	0	0
0	0	0	0	0	0	0	0	0	0	0	0	0	0	0	0	0	0	0
0	0	0	0	0	0	0	0	0	0	0	0	0	0	0	0	0	0	0
0	0	0	0	0	0	0	0	0	0	0	0	0	0	0	0	0	0	0
0	0	0	0	0	0	0	0	0	0	0	0	0	0	0	0	0	0	0
0	0	0	0	0	0	0	0	0	0	0	0	0	0	0	0	0	0	0
0	0	0	0	0	0	0	0	0	0	0	0	0	0	0	0	0	0	0
0	0	0	0	0	0	0	0	0	0	0	0	0	0	0	0	0	0	0
0	0	0	0	0	0	0	0	0	0	0	0	0	0	0	0	0	0	0
0	0	0	0	0	0	0	0	0	0	0	0	0	0	0	0	0	0	0
0	0	0	0	0	0	0	0	0	0	0	0	0	0	0	0	0	0	0
0	0	0	0	0	0	0	0	0	0	0	0	0	0	0	0	0	0	0
0	0	0	0	0	0	0	0	0	0	0	0	0	3	0	0	0	0	0
0	0	0	0	0	0	0	0	0	0	0	0	0	0	0	0	0	0	0
0	0	0	0	0	0	0	0	0	0	0	0	0	0	0	0	0	0	0
0	0	0	0	0	0	0	0	0	0	0	0	0	0	0	0	0	0	0
0	0	0	0	0	0	0	0	0	0	0	0	0	0	0	0	0	0	0
0	0	0	0	0	0	0	0	0	0	0	0	0	0	0	0	0	0	0
0	0	0	0	0	0	0	0	0	0	0	0	0	0	0	0	0	0	0
0	0	0	0	0	0	0	0	0	0	0	0	0	0	0	0	0	0	0
0	0	0	0	0	0	0	0	0	0	0	0	0	0	0	0	0	0	0
0	0	0	0	0	0	0	0	0	0	0	0	0	0	0	0	0	0	0
0	0	0	0	0	0	0	0	0	0	0	0	0	0	0	0	0	0	0
0	0	0	0	0	0	0	0	0	0	0	0	0	0	0	0	0	0	0
0	0	0	0	0	0	0	0	0	0	0	0	0	0	0	0	0	0	0
0	0	0	0	0	0	0	2	2	0	0	0	0	0	0	0	0	0	0
0	0	0	0	0	0	0	0	0	0									
Jan_DL041	2851	1575	1152	651	102	313	367	403	93	520	625	301	464	467	307	491	432	303
296	4	137	0	253	111	360	260	355	362	126	575	197	204	262	289	259	291	470
316	88	137	845	171	226	77	111	75	190	69	196	101	215	32	175	230	83	315
297	143	182	37	53	65	63	102	102	123	41	103	0	87	35	53	18	33	0
73	111	24	69	180	82	62	20	82	76	0	48	79	85	0	119	27	66	44
47	80	0	0	0	24	81	0	63	40	0	0	44	0	45	22	0	22	0

0	21	32	21	27	0	0	0	0	290	18	36	10	20	0	0	0	0	0
299	0	0	24	31	11	41	0	0	40	7	0	13	0	0	0	0	0	0
0	0	58	0	255	0	40	0	0	0	9	0	0	0	0	47	11	0	0
37	40	0	0	0	0	0	0	0	0	0	25	0	28	0	0	9	0	0
0	0	0	0	0	15	0	0	35	0	0	0	29	0	0	0	0	0	0
23	0	0	0	13	29	0	0	0	25	0	0	0	0	0	0	0	9	0
0	0	0	0	0	0	0	0	0	18	0	0	7	19	0	0	0	0	28
0	0	0	9	25	0	0	0	0	0	0	0	0	21	0	0	0	0	0
0	0	0	0	10	19	0	0	18	2	7	0	0	0	9	0	0	0	0
7	0	15	0	0	0	0	0	0	0	0	0	0	0	0	11	0	0	0
0	5	18	0	0	0	0	0	0	9	0	0	0	14	0	0	0	0	5
0	0	0	9	0	42	0	0	10	0	0	0	0	22	0	0	0	12	39
0	7	42	0	0	0	0	0	0	10	0	15	0	0	0	0	21	0	0
9	0	7	0	10	78	2	0	0	0	0	0	0	0	0	21	0	0	8
0	0	0	0	0	0	0	0	0	0	17	0	9	0	0	0	101	0	0
0	0	0	0	19	31	0	0	0	0	0	0	0	0	0	0	0	0	0
0	0	0	0	0	0	0	0	0	0	0	0	0	0	0	0	4	0	0
0	0	0	0	0	23	0	0	0	0	0	0	0	0	0	0	0	0	7
0	0	0	0	0	0	0	10	0	0	0	5	0	0	0	0	0	0	0
0	0	0	0	0	0	0	0	0	0	0	0	0	0	0	2	0	0	0
0	0	0	0	0	0	17	0	0	0	0	0	0	0	0	0	11	3	0
0	0	0	0	0	9	0	0	0	35	0	0	0	0	0	0	0	0	0
0	0	0	0	0	0	0	0	0	0	0	0	0	0	0	0	0	0	0
0	0	0	0	0	0	0	0	0	0	4	0	6	0	0	0	0	0	0
0	0	0	0	0	0	16	0	0	0	0	0	0	0	0	0	0	0	3
0	0	0	0	0	0	0	0	0	0	11	0	0	0	10	0	0	0	0
0	0	0	0	0	0	0	0	0	0	0	0	0	0	0	0	0	0	0
0	0	0	0	0	0	0	0	0	0	0	0	0	0	0	0	0	0	0
0	0	0	0	0	0	0	18	11	0	0	4	0	0	0	0	0	0	0
0	0	0	0	0	0	0	0	0	0	0	0	0	0	0	0	0	0	0
0	0	0	0	0	0	0	0	0	0	0	0	0	0	0	4	0	0	0
0	0	0	0	0	0	0	0	0	0	0	0	0	0	0	0	0	0	0
0	0	0	0	0	0	5	0	0	0	0	0	0	41	0	0	0	0	0
7	0	0	0	0	0	0	0	0	0	0	0	0	0	0	0	0	0	0
0	0	0	0	0	0	0	0	0	0	0	0	0	0	0	0	0	0	0
0	0	0	0	0	0	0	0	0	0	0	0	0	0	0	0	0	0	0
0	0	0	0	0	0	0	0	0	0	0	0	0	0	0	0	0	0	0

0	0	16	0	0	10	0	0	0	0	0	0	0	0	0	0	92	0	0
0	0	0	0	0	0	0	0	0	0	0	0	0	0	0	0	0	0	0
0	0	0	0	0	0	0	0	0	0	3	0	0	0	0	0	0	0	0
0	0	7	0	0	0	0	0	0	0	0	0	0	0	0	0	0	0	0
0	0	0	0	0	0	0	0	0	0	0	0	0	0	0	0	0	0	0
0	0	0	0	0	0	0	0	0	0	0	0	0	0	0	0	0	0	0
0	0	0	0	0	0	0	0	0	0	0	0	0	0	0	0	0	0	0
0	0	0	0	0	0	0	0	0	0	0	0	0	0	0	0	0	0	0
0	0	0	0	0	0	0	0	0	0	0	0	6	0	0	0	0	0	0
0	0	0	0	0	0	0	0	7	0	0	0	0	0	0	0	0	0	0
0	5	0	0	0	0	0	0	0	0	0	0	0	0	0	0	0	0	0
0	0	0	0	0	0	0	0	0	0	0	12	0	0	0	0	0	0	0
0	0	0	0	0	0	0	0	0	0	0	0	0	0	0	0	0	0	0
0	0	0	0	0	0	0	0	0	14	7	0	0	0	0	11	0	0	0
0	0	0	0	0	0	0	0	0	0	0	0	0	0	0	0	0	0	0
0	27	0	0	0	0	0	0	0	0	0	0	0	0	0	0	0	0	0
0	0	0	0	0	0	0	5	0	0	0	0	0	0	0	0	0	0	0
0	0	0	0	0	0	0	0	0	0	0	7	0	0	0	0	0	0	0
0	0	0	14	0	0	0	0	0	0	0	0	0	0	0	0	0	0	0
0	0	0	0	0	0	0	0	0	0	0	0	0	0	0	0	0	0	0
0	0	0	0	0	0	0	0	0	0	0	0	0	0	0	0	0	0	0
0	0	0	0	0	0	0	0	0	0	0	0	0	0	0	0	0	0	0
0	0	0	0	0	0	0	0	0	0	0	0	0	0	0	0	0	0	0
0	0	0	0	0	0	0	0	0	0	0	0	0	0	0	0	0	0	0
0	0	0	0	0	0	0	0	0	0	0	0	0	0	0	0	0	0	0
0	0	0	0	0	0	0	0	0	0	0	0	0	0	0	0	0	0	0
0	0	0	0	0	0	0	0	0	0	0	0	0	0	0	0	0	0	0
0	0	0	0	0	0	0	0	0	0	0	0	0	0	0	0	0	0	0
0	0	0	0	0	0	0	0	0	0	0	0	0	0	0	0	0	0	0
0	0	0	0	0	0	0	0	0	0	0	0	0	0	0	0	0	0	0
0	0	0	0	0	0	0	0	0	0	0	0	0	0	0	0	0	0	0
0	0	0	0	0	0	0	0	0	0	0	0	0	0	0	0	0	0	0
0	0	0	0	0	0	0	0	0	0	0	0	0	0	0	0	0	0	0
0	0	0	0	0	0	0	0	0	0	0	0	0	0	0	0	0	0	0
0	0	0	0	0	0	0	0	0	0	0	0	0	0	0	0	0	0	0
0	0	0	0	0	0	0	0	0	0	0	0	0	0	0	0	0	0	0
0	0	0	0	0	0	0	0	0	0	0	0	0	0	0	0	0	0	0

0	0	0	0	0	0	0	0	0	0	0	0	0	0	0	0	0	0	0
0	0	0	0	0	0	0	0	0	0	0	0	0	0	0	0	0	0	0
0	0	0	0	0	0	0	0	0	0	0	0	0	0	0	0	0	0	0
0	0	0	0	0	0	0	0	0	0	0	0	0	0	0	0	0	0	0
0	0	0	0	0	0	0	0	0	0	0	0	0	0	0	1	0	0	0
0	0	0	0	0	0	0	0	0	0	0	0	0	0	0	0	0	0	0
0	0	0	0	0	0	0	0	0	0	0	0	0	0	0	0	0	0	0
0	0	0	0	0	0	0	0	0	0	0	0	0	0	0	0	0	0	0
0	0	0	0	0	0	0	0	0	0	0	0	0	0	0	0	0	0	0
0	0	0	0	0	0	0	0	0	0	0	0	0	0	0	0	0	0	0
0	0	0	0	0	0	0	0	0	0	0	0	0	0	0	0	0	0	0
0	0	0	0	0	0	0	0	0	0	0	0	0	0	0	0	0	0	0
0	0	0	0	0	0	0	0	0	0	0	0	0	0	0	0	0	0	0
0	0	0	0	0	0	0	0	0	0	0	0	0	0	0	0	0	0	0
0	0	0	0	0	0	0	0	0	0	0	0	0	0	0	0	0	0	0
0	0	0	0	0	0	0	0	0	0	0	0	0	0	0	0	0	0	0
0	0	0	0	0	0	0	0	0	0	0	0	0	0	0	0	0	0	0
0	0	0	0	0	0	0	0	0	0	0	0	0	0	0	0	0	0	0
0	0	0	0	0	0	0	0	0	0	0	0	0	0	0	0	0	0	0
0	0	0	0	0	0	0	0	0	0	0	0	0	0	0	0	0	0	0
0	0	0	0	0	0	0	0	0	0	0	0	0	0	0	0	0	0	0
0	0	0	0	0	0	0	0	0	0	0	0	0	0	0	0	0	0	0
0	0	0	0	0	0	0	0	0	0	0	0	0	0	0	0	0	0	0
0	0	0	0	0	0	0	0	0	0	0	0	0	0	0	0	0	0	0
0	0	0	0	0	0	0	0	0	0	0	8	0	0	0	0	0	0	0
0	0	0	0	0	0	0	0	0	0	0	0	0	0	0	0	0	0	0
0	0	0	0	0	0	0	0	0	0	0	0	0	0	0	0	0	0	0
0	0	0	0	0	0	0	0	0	0	0	0	0	0	0	0	0	0	0
0	0	0	0	0	0	0	0	0	0	0	0	0	0	0	0	0	0	0
0	0	0	0	0	0	0	0	0	0	0	0	0	0	0	0	0	0	0
0	0	0	0	0	0	0	0	0	0	0	0	7	0	0	0	0	0	0
0	0	0	0	0	0	0	0	0	0	0	0	0	0	0	0	0	0	0
0	0	0	0	0	0	0	0	0	0	0	0	0	0	0	0	0	0	0
0	0	0	0	0	0	0	0	0	0	0	0	0	0	0	0	0	0	0
0	0	0	0	0	0	0	0	0	0	0	0	0	0	0	0	0	0	0
0	0	0	0	0	0	0	0	0	0	0	0	0	0	0	0	0	0	0
0	0	0	0	0	0	0	0	0	0	0	0	0	0	0	0	0	0	0

0	0	0	0	0	0	0	0	0	0	0	0	0	0	0	0	0	0	0
0	0	0	0	0	0	0	0	0	0	0	0	0	0	0	0	0	0	0
0	0	0	0	0	0	0	0	0	0	0	0	0	0	0	0	0	0	0
0	0	0	0	0	0	0	0	0	0	0	0	0	0	0	0	0	0	0
0	0	0	0	0	0	0	0	0	0	0	0	0	0	0	0	0	0	0
0	0	0	0	0	0	0	0	0	0	0	0	0	0	0	0	0	0	0
0	0	0	0	0	0	0	0	0	0	0	0	0	0	0	0	0	0	0
0	0	0	0	0	0	0	0	0	0	0	0	0	0	0	0	0	0	0
0	0	0	0	0	0	0	0	0	0	0	0	0	0	0	0	0	0	0
0	0	0	0	0	0	0	0	0	0	0	0	0	0	0	0	0	0	0
0	0	0	0	0	0	0	0	0	0	0	0	0	0	0	0	0	0	0
0	0	0	0	0	0	0	0	0	0	0	0	0	0	0	0	0	0	0
5	0	0	0	0	0	0	0	0	0	0	0	0	0	0	0	0	0	0
0	0	0	0	0	0	0	0	0	0	0	0	0	0	0	0	0	0	0
0	0	0	0	0	0	0	0	0	0	0	0	0	0	0	0	0	0	0
0	0	0	0	0	0	0	0	0	0	0	0	0	0	0	0	0	0	0
0	0	0	0	0	0	0	0	0	0	0	0	0	0	0	0	0	0	0
0	0	0	0	0	0	0	0	0	0	0	0	0	0	0	0	0	0	0
0	0	0	0	0	0	0	0	0	0	0	0	0	0	0	0	0	0	0
0	0	0	0	0	0	0	0	0	0	0	0	0	0	0	0	0	0	0
0	0	0	0	0	0	0	0	0	0	0	0	0	0	0	0	0	0	0
0	0	0	0	0	0	0	0	0	0	0	0	0	0	0	0	0	0	0
0	0	0	0	0	0	0	0	0	0	0	0	0	0	0	0	0	0	0
0	0	0	0	0	0	0	0	0	0	0	0	0	0	0	0	0	0	0
0	0	0	0	0	0	0	0	0	0	0	0	0	0	0	0	0	0	0
0	0	0	0	0	0	0	0	0	0	0	0	0	0	0	0	0	0	0
0	0	0	0	0	4	4	0	0	0	0	0	0	0	0	0	0	0	0
0	0	0	0	0	0	0	0	0	0	0	0	0	0	0	0	0	0	0
0	0	0	0	0	0	0	0	0	0	0	0	0	0	0	0	0	0	0
0	0	0	0	0	0	0	0	0	0	0	0	0	0	0	0	0	0	0
0	0	0	0	0	0	0	0	0	0	0	0	0	0	0	0	0	0	0
0	0	0	0	0	0	0	0	0	0	0	0	0	0	0	0	0	0	0
0	0	0	0	0	0	0	0	0	0	0	0	0	0	0	0	0	0	0
0	0	0	0	0	0	0	0	0	0	0	0	0	0	0	0	0	0	0
0	0	0	0	0	0	0	0	0	0	0	0	0	0	0	0	0	0	0
0	0	0	0	0	0	0	0	0	0	0	0	0	0	0	0	0	0	0
0	0	0	0	0	0	0	0	0	0	0	0	0	0	0	0	0	0	0

0	0	0	0	0	0	0	0	0	0	0	0	0	0	0	0	0	0	0
0	0	0	0	0	0	0	0	0	0	0	0	0	0	0	0	0	0	0
0	0	0	0	0	0	0	0	0	0	0	0	0	0	0	0	0	0	0
0	0	0	0	0	0	0	0	0	0	0	0	0	0	0	0	0	0	0
0	0	0	0	0	0	0	0	0	0	0	0	0	0	0	0	0	0	0
0	0	0	0	0	0	0	0	0	0	0	0	0	0	0	0	0	0	0
0	0	0	0	0	0	0	0	0	0	0	0	0	0	0	0	0	0	0
0	0	0	0	0	0	0	0	0	0	0	0	0	0	0	0	0	0	0
3	3	3	3	0	0	0	0	0	0	0	0	0	0	0	0	0	0	0
0	0	0	0	0	0	0	0	0	0	0	0	0	0	0	0	0	0	0
0	0	0	0	0	0	0	0	0	0	0	0	0	0	0	0	0	0	0
0	0	0	0	0	0	0	0	0	0	0	0	0	0	0	0	0	0	0
0	0	0	0	0	0	0	0	0	0	0	0	0	0	0	0	0	0	0
0	0	0	0	0	0	0	0	0	0	0	0	0	0	0	0	0	0	0
0	0	0	0	0	0	0	0	0	0	0	0	0	0	0	0	0	0	0
0	0	0	0	0	0	0	0	0	0	0	0	0	0	0	0	0	0	0
0	0	0	0	0	0	0	0	0	0	0	0	0	0	0	0	0	0	0
0	0	0	0	0	0	0	0	0	0	0	0	0	0	0	0	0	0	0
0	0	0	0	0	0	0	0	0	0	0	0	0	0	0	0	0	0	0
0	0	0	0	0	0	0	0	0	0	0	0	0	0	0	0	0	0	0
0	0	0	0	0	2	2	2	2	2	2	2	2	2	0	0	0	0	0
0	0	0	0	0	0	0	0	0	0	0	0	0	0	0	0	0	0	0
0	0	0	0	0	0	0	0	0	0									
Jan_DL042	2262	2407	49	697	84	491	772	692	291	207	150	279	139	446	335	14	313	203
333	96	290	421	327	224	100	269	97	153	148	0	251	229	90	97	192	56	0
97	28	272	158	159	74	178	0	179	0	167	0	0	112	119	0	0	138	0
0	0	0	68	146	230	127	57	14	214	49	156	46	135	174	161	148	92	0
0	0	42	12	0	30	32	40	68	10	0	0	63	78	48	0	156	36	0
91	116	0	119	0	102	20	100	47	0	0	99	0	14	24	0	0	0	96
61	75	0	11	6	39	0	0	0	0	37	65	0	5	50	0	0	44	0
45	0	125	11	0	0	0	84	0	44	49	0	21	0	0	27	0	0	0
0	23	0	34	54	0	0	44	0	44	9	0	53	0	0	0	27	33	19
43	0	0	41	57	44	63	24	0	16	27	0	0	0	11	0	11	55	11
0	0	23	0	50	23	0	19	0	10	0	0	0	82	30	0	0	36	0
15	21	0	0	10	0	0	0	0	97	0	27	0	0	0	107	0	20	0
0	0	0	0	0	20	0	0	0	0	0	0	21	18	14	15	0	0	0
16	0	0	46	0	0	0	24	0	0	0	0	0	0	7	5	0	8	0

0	33	0	16	0	5	0	0	7	0	6	37	0	6	0	4	0	0	0
0	0	0	0	0	0	0	23	9	0	0	0	25	0	6	9	0	0	0
0	10	17	0	0	0	0	52	0	0	19	0	13	0	20	0	0	9	12
15	0	0	0	0	0	0	8	0	0	0	0	0	13	0	0	0	0	8
5	8	48	0	7	0	0	0	14	0	0	10	0	0	0	0	0	0	0
0	16	0	0	0	0	0	0	0	0	0	0	0	0	0	0	0	0	9
38	0	0	0	0	4	0	0	26	0	0	0	13	0	0	0	0	0	0
0	0	0	0	0	0	0	29	0	0	0	12	17	0	0	0	32	0	0
0	0	0	52	0	0	0	0	0	0	0	0	0	0	8	0	0	0	0
0	0	0	0	0	0	0	0	0	0	22	0	0	0	0	0	0	6	0
0	4	0	0	0	0	0	0	0	0	0	0	0	0	0	0	0	0	0
0	0	0	0	0	0	15	0	0	0	0	0	0	0	0	0	0	0	0
9	0	0	0	0	0	0	0	0	0	0	0	0	0	0	0	0	0	0
0	0	0	15	0	0	0	0	0	0	0	0	0	0	0	11	0	0	0
0	0	0	0	0	0	0	0	0	0	23	0	24	0	0	0	0	0	0
0	0	0	0	0	0	0	0	12	0	0	0	0	0	0	7	0	9	0
0	0	0	0	0	0	0	0	0	0	0	24	0	0	17	0	0	0	0
0	0	0	0	0	0	0	6	0	0	0	0	0	0	0	0	0	0	0
0	0	3	17	0	0	0	0	0	0	21	0	0	0	0	0	7	0	0
0	0	0	0	0	0	0	0	0	0	0	0	0	0	0	7	0	0	0
0	0	0	0	0	0	0	0	0	0	0	10	0	0	0	8	0	5	0
0	0	0	0	0	0	0	0	0	0	0	0	0	0	0	0	0	0	12
0	0	0	0	0	0	0	0	0	0	0	0	0	0	25	0	0	0	0
0	0	0	0	0	0	0	0	0	0	0	0	0	0	0	0	0	0	0
0	0	0	0	0	0	0	21	0	0	0	0	0	0	0	0	0	0	0
7	0	0	0	0	0	0	0	0	0	0	0	9	0	0	0	0	0	0
0	0	0	0	3	0	0	0	0	0	0	0	0	0	0	0	0	0	0
0	0	0	0	0	0	0	0	10	0	0	0	0	0	0	0	0	0	0
0	0	0	0	0	0	0	0	0	0	0	0	0	0	0	0	0	0	0
0	0	0	0	0	0	0	0	0	0	0	0	0	0	0	0	0	0	0
0	0	0	3	0	0	0	0	0	0	0	0	0	0	0	0	0	0	0
0	0	0	0	0	0	0	0	0	0	0	0	0	0	0	0	0	0	0
0	0	0	0	0	0	0	0	0	0	0	0	0	0	0	0	5	0	0
0	0	0	0	0	0	0	0	0	0	0	0	0	0	0	0	0	0	0
0	0	0	0	0	0	0	0	0	0	0	0	0	0	0	0	0	0	0
0	0	0	0	0	0	0	0	0	0	0	0	0	0	0	6	0	0	0
0	0	0	0	0	0	0	0	0	0	0	0	0	0	0	0	0	0	0

0	0	0	0	0	0	0	0	0	0	0	0	0	0	0	13	0	0	0
0	0	0	0	0	0	0	0	0	0	0	0	0	0	0	0	0	0	0
0	0	0	0	0	0	0	0	0	0	0	0	0	0	0	0	0	0	0
0	0	0	0	0	0	0	0	0	0	0	0	0	0	0	0	0	0	0
0	0	0	0	0	0	0	0	0	0	0	0	0	0	0	0	0	0	0
0	0	0	0	0	0	0	0	0	0	0	0	0	0	0	0	0	0	0
0	0	0	0	0	0	0	0	0	0	0	0	0	0	0	0	0	0	0
0	0	0	0	0	0	0	0	0	0	0	0	0	0	0	0	0	0	0
0	0	0	0	0	0	0	0	0	0	0	0	0	0	0	0	0	0	0
0	0	0	0	0	0	0	0	0	0	0	0	0	0	0	0	0	0	0
0	0	0	0	0	0	0	0	0	0	0	0	0	0	0	0	0	0	0
0	0	0	0	0	0	0	0	0	0	0	0	0	0	0	0	0	0	0
0	0	0	0	0	0	0	0	0	0	0	0	0	0	0	0	0	0	0
0	0	0	0	22	0	0	0	0	0	0	0	0	0	0	0	0	0	0
0	3	0	0	0	0	0	0	0	0	0	0	0	0	0	0	0	0	0
0	0	0	0	0	0	20	0	0	0	0	0	0	0	0	0	0	0	0
0	0	0	0	0	0	0	0	0	0	0	0	0	0	0	0	0	0	0
0	0	0	0	0	0	0	0	0	0	0	0	0	0	0	0	0	0	0
0	0	0	0	0	0	0	0	0	0	0	0	0	0	0	0	0	0	0
0	0	0	0	0	0	0	0	0	0	0	0	0	0	0	0	0	0	0
0	0	0	0	0	0	0	0	0	0	0	0	0	0	0	0	0	0	0
0	0	0	0	0	0	0	0	0	0	0	0	0	0	0	0	0	0	0
0	0	0	0	0	0	0	0	0	0	0	0	0	0	0	0	0	0	0
0	0	0	0	0	0	0	0	0	0	0	0	0	0	0	0	0	0	0
0	0	0	0	0	0	0	0	0	0	0	0	0	0	0	0	0	0	0
0	0	0	0	0	0	0	0	0	0	0	0	0	0	0	0	0	0	0
0	0	0	0	0	0	0	0	0	0	0	0	0	0	0	0	0	0	0
0	0	0	0	0	0	0	0	0	0	0	0	0	0	0	0	0	0	0
0	0	0	0	0	0	0	0	0	0	0	0	0	0	0	0	0	0	0
0	0	0	0	0	0	0	0	0	0	0	0	0	0	0	0	0	0	0
0	0	0	0	0	0	0	0	0	0	0	0	0	0	0	0	0	0	0
0	0	0	0	0	0	0	0	0	0	0	0	0	0	0	0	0	0	0
0	0	0	0	0	0	0	0	0	0	0	0	0	0	0	0	0	0	0
0	0	0	0	0	0	0	0	0	0	0	0	0	0	0	0	0	0	0
0	0	0	0	0	0	0	0	0	0	0	0	0	0	0	0	0	0	0
0	0	0	0	0	0	0	0	0	0	0	0	0	0	0	0	0	0	0
0	0	0	0	0	0	0	0	0	0	0	0	0	0	0	0	0	0	0

0	0	0	0	0	0	0	0	0	0	0	0	0	0	0	0	0	0	0
0	0	0	0	0	0	0	0	0	0	0	0	0	0	0	0	0	0	0
0	0	0	0	0	0	0	0	0	0	0	0	0	0	0	0	0	0	0
0	0	0	0	0	9	0	0	0	0	0	0	0	0	0	0	0	0	0
0	0	0	0	0	0	0	0	0	0	0	0	0	0	0	0	0	0	0
0	0	0	0	0	0	0	0	0	0	0	0	0	0	0	0	0	0	0
0	0	0	0	0	0	0	0	0	0	0	0	0	0	0	0	0	0	0
0	0	0	0	0	0	0	0	0	0	0	0	0	0	0	0	0	0	0
0	0	0	0	0	0	0	0	0	0	0	0	0	0	0	0	0	0	0
0	0	0	0	0	0	0	0	0	0	0	0	0	0	0	0	0	0	0
0	0	0	0	0	0	0	0	0	0	0	0	0	0	0	0	0	0	0
0	0	0	0	0	0	0	0	0	0	0	0	0	0	0	0	0	0	0
0	0	0	0	0	0	0	0	0	0	0	0	0	0	0	0	0	0	0
0	0	0	0	0	0	0	0	0	0	0	0	0	0	0	0	0	0	0
0	0	0	0	0	0	0	0	0	0	0	0	0	0	0	0	0	0	0
0	0	0	0	0	0	0	0	0	0	0	0	0	0	0	0	0	0	0
0	0	0	0	0	0	0	0	0	0	0	0	0	0	0	0	0	0	0
0	0	0	0	0	0	0	0	0	0	0	0	0	0	0	0	0	0	0
0	0	0	0	0	0	0	0	0	0	0	0	0	0	0	0	0	0	0
0	0	0	0	0	0	0	0	0	0	0	0	0	0	0	0	0	0	0
0	0	0	0	0	0	0	0	0	0	0	0	0	0	0	0	0	0	0
6	6	0	0	0	0	0	0	0	0	0	0	0	0	0	0	0	0	0
0	0	0	0	0	0	0	0	0	0	0	0	0	0	0	0	0	0	0
0	0	0	0	0	0	0	0	0	0	0	0	0	0	0	0	0	0	0
0	0	0	0	0	0	0	0	0	0	0	0	0	0	0	0	0	0	0
0	0	0	0	0	0	0	0	0	0	0	0	0	0	0	0	0	0	0
0	0	0	0	0	0	0	0	0	0	0	0	0	0	0	0	0	0	0
0	0	0	0	0	0	0	0	0	0	0	0	0	0	0	0	0	0	0
0	0	0	0	0	0	0	0	0	0	0	0	0	0	0	0	0	0	0
0	0	0	0	0	0	0	0	5	0	0	0	0	0	0	0	0	0	0
0	0	0	0	0	0	0	0	0	0	0	0	0	0	0	0	0	0	0
0	0	0	0	0	0	0	0	0	0	0	0	0	0	0	0	0	0	0
0	0	0	0	0	0	0	0	0	0	0	0	0	0	0	0	0	0	0
0	0	0	0	0	0	0	0	0	0	0	0	0	0	0	0	0	0	0
0	0	0	0	0	0	0	0	0	0	0	0	0	0	0	0	0	0	0
0	0	0	0	0	0	0	0	0	0	0	0	0	0	0	0	0	0	0
0	0	0	0	0	0	0	0	0	0	0	0	0	0	0	0	0	0	0

0	0	0	0	0	0	0	0	0	0	0	0	0	0	0	0	0	0	0
0	0	0	0	0	0	0	0	0	0	0	0	0	0	0	0	0	0	0
0	0	0	0	0	0	0	0	0	0	0	0	0	0	0	0	0	0	0
0	0	0	0	0	0	0	0	0	0	0	0	0	0	0	0	0	0	0
0	0	0	0	0	0	0	0	0	0	0	0	0	0	0	0	0	0	0
0	0	0	0	0	0	0	0	0	0	0	0	0	0	0	0	0	0	0
0	0	0	0	0	0	0	0	0	0	0	0	0	0	0	0	4	0	0
0	0	0	0	0	0	0	0	0	0	0	0	0	0	0	0	0	0	0
0	0	0	0	0	0	0	0	0	0	0	0	0	0	0	0	0	0	0
0	0	0	0	0	0	0	0	0	0	0	0	0	0	0	0	0	0	0
0	0	0	0	0	0	0	0	0	0	0	0	0	0	0	0	0	0	0
0	0	0	0	0	0	0	0	0	0	0	0	0	0	0	0	0	0	0
0	0	0	0	0	0	0	0	0	0	0	0	0	0	0	0	0	0	0
0	0	0	0	0	0	0	0	0	0	0	0	0	0	0	0	0	0	0
0	0	0	0	0	0	0	0	0	0	0	0	0	0	0	0	0	0	0
0	0	0	0	0	0	0	0	0	0	0	0	0	0	0	0	0	0	0
0	0	0	0	0	0	0	0	0	0	0	0	0	0	0	0	0	0	0
0	0	0	0	0	0	0	0	0	0	0	0	0	0	0	0	0	0	0
0	0	0	0	0	0	0	0	0	0	0	0	0	0	0	0	0	0	0
0	0	0	0	0	0	0	0	0	0	0	0	0	0	0	0	0	0	0
0	0	0	0	0	0	0	0	0	0	0	0	0	0	0	0	0	0	0
0	0	0	0	0	0	0	0	0	0	0	0	0	0	0	0	0	0	0
0	0	0	0	0	0	0	0	0	0	0	0	0	0	0	0	0	0	0
0	0	0	0	0	0	0	0	0	0	0	0	0	0	0	0	0	0	0
0	0	0	0	0	0	0	0	0	0	0	0	0	0	0	0	0	0	0
0	0	0	0	0	0	0	0	0	0	0	0	0	0	0	0	0	0	0
0	0	0	0	0	0	0	0	0	0	3	0	0	0	0	0	0	0	0
0	0	0	0	0	0	0	0	0	0	0	0	0	0	0	0	0	0	0
0	0	0	0	0	0	0	0	0	0	0	0	0	0	0	0	0	0	0
0	0	0	0	0	0	0	0	0	0	0	0	0	0	0	0	0	0	0
0	0	0	0	0	0	0	0	0	0	0	0	0	0	0	0	0	0	0
0	0	0	0	0	0	0	0	0	0	0	0	0	0	0	0	0	0	0
0	0	0	0	0	0	0	0	0	0	0	0	0	0	0	0	0	0	0
0	0	0	0	0	0	0	0	0	0	0	0	0	0	0	0	0	0	0
0	0	0	0	0	0	0	0	0	0	0	0	0	0	0	0	0	0	0
0	0	0	0	0	0	0	0	0	0	0	0	0	0	0	0	0	0	0
0	0	0	0	0	0	0	0	0	0	0	0	0	0	0	0	0	0	0

0	0	0	0	0	0	0	0	0	0	0	0	0	0	0	0	0	0	0
0	0	0	0	0	0	0	0	0	0	0	0	0	0	0	0	0	0	0
0	0	0	0	0	0	0	0	0	0	0	0	0	0	0	0	0	0	0
0	0	0	0	0	0	0	0	0	0	0	0	0	0	0	0	0	0	0
0	0	0	0	0	0	0	0	0	0	0	0	0	0	0	0	0	0	0
0	0	0	0	0	0	0	0	0	0	0	0	0	0	0	0	0	0	0
0	0	0	0	0	0	0	0	0	0	0	0	0	0	0	0	0	0	0
0	0	0	0	0	0	0	0	0	0	0	0	0	0	0	0	0	0	0
0	0	0	0	0	0	0	0	0	0	0	0	0	0	0	0	0	0	0
0	0	0	0	0	0	0	0	0	0	0	0	0	0	0	0	0	0	0
0	0	0	0	0	0	0	2	2	2	2	2	2	0	0	0	0	0	0
0	0	0	0	0	0	0	0	0	0	0	0	0	0	0	0	0	0	0
0	0	0	0	0	0	0	0	0	0	0	0	0	0	0	0	0	0	0
0	0	0	0	0	0	0	0	0	0	0	0	0	0	0	0	0	0	0
0	0	0	0	0	0	0	0	0	0									
Jan_DL044	1655	1597	431	720	32	322	176	131	59	352	456	57	279	91	160	211	97	263
83	25	222	366	149	236	166	114	159	102	100	0	131	148	137	237	76	81	0
72	106	44	0	67	145	54	0	115	123	0	51	101	27	0	0	0	0	0
0	52	0	6	27	100	86	47	75	109	88	91	24	81	39	80	26	224	139
0	0	0	56	0	0	3	0	42	77	0	57	34	19	47	0	73	0	0
0	24	0	0	0	0	0	64	0	16	0	0	64	33	21	19	0	0	0
0	36	12	17	0	0	0	0	0	0	4	27	0	0	0	8	0	0	0
28	0	0	21	0	0	0	11	0	0	9	0	5	0	18	0	0	0	0
0	0	0	4	0	9	0	0	0	0	7	0	0	0	73	0	0	0	0
0	0	18	0	50	0	0	0	0	0	0	0	0	0	0	5	0	65	0
0	0	0	0	0	0	0	0	0	0	0	0	0	62	0	0	0	0	0
0	0	0	0	11	0	0	0	0	0	0	3	20	0	0	0	0	0	30
0	0	0	0	0	0	0	0	92	15	0	10	8	0	71	0	13	12	0
0	0	0	0	0	0	0	0	0	0	0	0	0	0	0	0	0	0	0
0	0	0	0	0	0	0	0	0	0	0	3	0	0	0	0	0	0	0
0	0	7	0	0	0	0	0	9	0	0	14	0	0	0	0	11	0	0
0	7	0	0	0	0	0	0	0	0	0	0	0	3	16	0	0	0	0
0	0	0	0	0	0	0	0	0	0	0	0	0	0	0	0	0	0	0
0	0	0	0	0	0	0	0	0	0	108	12	0	0	0	0	0	0	0
0	7	0	0	10	0	12	0	0	0	0	0	0	0	0	0	0	0	0
0	0	0	0	0	0	0	0	0	0	5	0	0	0	0	0	0	0	0
0	4	0	0	0	0	0	0	0	0	0	0	0	0	0	0	0	0	0

0	0	0	5	0	0	0	0	0	0	0	0	0	0	0	0	0	0	0
0	0	0	0	0	0	0	0	0	0	0	0	0	0	0	0	0	0	0
0	0	0	0	8	0	0	0	0	0	0	0	0	0	0	0	0	11	0
0	0	0	0	2	0	0	0	0	4	0	0	0	0	0	0	0	0	0
0	0	0	0	0	0	0	0	0	0	0	0	0	0	0	0	0	4	10
0	14	0	0	0	0	0	0	0	0	0	0	0	0	0	0	0	12	0
0	0	0	0	0	0	0	0	0	0	0	0	0	0	0	0	0	0	0
0	5	0	0	0	0	0	0	0	0	0	0	0	0	0	0	0	0	0
0	0	0	0	0	0	0	0	0	0	0	0	0	0	0	0	0	0	0
0	0	0	0	0	0	0	0	0	0	0	0	0	0	0	0	0	0	0
0	0	0	0	0	0	0	0	5	0	0	0	0	0	0	0	0	0	8
0	0	0	0	0	0	0	0	0	0	0	0	0	0	0	0	0	0	0
0	0	0	0	0	0	0	0	0	0	0	0	0	0	0	0	0	3	0
0	0	0	0	0	0	30	0	0	0	0	0	0	0	0	0	0	0	0
16	51	0	0	0	0	0	0	0	0	0	0	0	0	0	0	0	0	0
0	0	0	0	0	0	0	0	0	0	0	0	0	0	0	0	0	0	0
2	0	0	0	0	0	0	0	0	0	0	0	0	0	0	0	0	0	0
0	0	0	0	0	0	0	0	0	0	0	0	10	0	0	0	0	0	0
0	0	0	0	0	0	0	0	0	0	0	0	0	0	0	0	0	0	0
0	0	0	0	0	0	0	0	0	0	0	0	0	0	0	0	0	0	0
0	0	0	0	0	0	0	0	0	0	0	0	0	0	0	0	0	0	0
0	0	0	0	0	0	0	0	0	0	0	0	0	0	0	0	0	0	0
0	0	0	0	0	0	0	0	0	0	0	0	0	0	0	0	0	0	0
0	14	0	0	0	0	0	0	0	0	0	0	0	0	0	0	0	0	0
0	0	0	0	0	0	0	0	0	0	0	0	0	0	0	0	0	0	0
0	0	0	0	0	0	0	0	0	0	0	0	0	0	0	0	0	0	0
0	0	0	0	0	0	0	0	0	0	0	0	0	10	0	0	0	0	0
0	0	0	0	0	10	0	0	0	0	0	0	0	0	0	0	0	0	0
0	0	0	0	0	0	0	0	0	0	0	0	0	0	0	0	0	0	0
0	0	0	0	0	0	0	0	0	0	0	0	0	0	0	0	0	0	0
0	0	0	0	0	0	0	0	0	0	0	0	0	0	0	0	0	0	0
0	0	0	0	0	0	0	0	0	0	0	0	0	0	0	0	0	0	0
0	0	0	0	0	0	0	0	0	0	0	0	0	0	0	0	0	0	0
0	0	0	0	0	0	0	0	0	0	0	0	0	0	0	0	0	0	0
0	0	0	0	0	0	0	0	0	0	0	0	0	0	0	0	0	0	0
0	0	0	0	0	0	0	0	0	0	0	0	0	0	0	0	0	0	0
0	0	0	0	0	0	0	0	0	0	0	0	0	0	0	0	0	0	0

0	0	0	0	0	0	18	0	0	0	0	0	0	0	0	0	0	0	0
0	0	0	0	0	0	0	0	0	0	0	0	0	0	0	0	0	0	0
0	0	0	0	0	0	0	0	0	0	0	0	0	0	0	0	0	0	0
0	0	0	0	0	0	0	0	0	0	0	0	0	0	0	0	0	0	0
0	0	0	0	0	0	0	0	0	0	0	0	0	0	0	0	0	0	0
0	0	0	0	0	0	0	0	0	0	0	0	0	0	0	0	0	0	0
0	0	0	0	0	0	0	8	0	0	0	0	0	0	0	0	0	0	0
0	0	0	0	0	0	0	0	0	0	0	0	0	0	0	0	0	0	0
0	0	0	0	0	0	0	0	0	0	0	0	0	0	0	0	0	5	0
0	0	0	0	0	0	0	0	0	0	0	0	5	0	0	0	0	0	0
0	0	0	0	0	0	0	0	0	0	0	0	0	0	0	0	0	0	0
0	0	0	0	0	0	0	0	0	0	0	0	0	0	0	0	0	0	0
0	0	0	0	0	0	0	0	0	0	0	0	0	0	0	0	0	0	0
0	0	0	0	0	0	0	0	0	0	0	0	0	0	0	0	0	0	0
0	0	0	0	0	0	0	0	0	0	0	0	0	0	0	0	0	0	0
0	0	0	0	0	0	0	0	0	0	0	0	0	0	0	0	0	0	9
0	0	0	0	0	0	0	0	0	0	0	0	0	0	0	0	0	0	0
0	0	0	0	0	0	0	0	0	0	0	0	0	0	0	0	0	0	0
0	0	0	0	0	0	0	0	0	0	0	0	0	0	0	0	0	0	0
0	0	0	0	0	0	0	0	0	0	0	0	0	0	0	0	0	0	0
0	0	0	0	0	0	0	0	0	0	0	0	0	0	0	0	0	0	0
0	0	0	0	0	0	0	0	0	0	0	0	0	0	0	0	0	0	0
0	0	0	0	0	0	0	0	0	0	0	0	0	0	0	0	0	0	0
0	0	0	0	0	0	0	0	0	0	0	0	0	0	0	7	0	0	0
0	0	0	0	0	0	0	0	0	0	0	0	0	0	0	0	0	0	0
0	0	0	0	0	0	0	0	0	0	0	0	0	0	0	0	0	0	0
0	0	0	0	0	0	0	0	0	0	0	0	0	0	0	0	0	0	0
0	0	0	0	0	0	0	0	0	0	0	0	0	0	0	0	0	0	0
0	0	0	0	0	0	0	0	0	0	0	0	0	0	0	0	0	0	0
0	0	0	0	0	0	0	0	0	0	0	0	0	0	0	6	0	0	0
0	0	0	0	0	0	0	0	0	0	0	0	0	0	0	0	0	0	0
0	0	0	0	0	0	0	0	0	0	0	0	0	0	0	0	0	0	0
0	0	0	0	0	0	0	0	0	0	0	0	0	0	0	0	0	0	0
0	0	0	0	0	0	0	0	0	0	0	0	0	0	0	0	0	0	0
0	0	0	0	0	0	0	0	0	0	0	0	0	0	0	0	0	0	0
0	0	0	0	0	0	0	0	0	0	0	0	0	0	0	0	0	0	0
0	0	0	0	0	0	0	0	0	0	0	0	0	0	0	0	0	0	0

0	0	0	0	0	0	0	0	0	0	0	0	0	5	0	0	0	0	0
0	0	0	0	0	0	0	0	0	0	0	0	0	0	0	0	0	0	0
0	0	0	0	0	0	0	0	0	0	0	0	0	0	0	0	0	0	0
0	0	0	0	0	0	0	0	0	0	0	0	0	0	0	0	0	0	0
0	0	0	0	0	0	0	0	0	0	0	0	0	0	0	0	0	0	0
0	0	0	0	0	0	0	0	0	0	0	0	0	0	0	0	0	0	0
0	0	0	0	0	0	0	0	0	0	0	0	0	0	0	0	0	0	0
0	0	0	0	0	0	0	0	0	0	0	0	0	0	0	0	0	0	0
0	0	0	0	0	0	0	0	0	0	0	0	0	0	0	0	0	0	0
0	0	0	0	0	0	0	0	0	0	0	0	0	0	0	0	0	0	0
0	0	0	0	0	0	0	0	0	0	0	0	0	0	0	0	0	0	0
0	0	0	0	0	0	0	0	0	0	0	0	0	0	0	0	0	0	0
0	0	0	0	0	0	0	0	0	0	0	0	0	0	0	0	0	0	0
0	0	0	0	0	0	0	0	0	0	0	0	0	0	0	0	0	0	0
0	0	0	0	0	0	0	0	0	0	0	0	0	0	0	0	0	0	0
0	0	0	0	0	0	0	0	0	0	0	0	0	0	0	0	0	0	0
0	0	0	0	0	0	0	0	0	0	0	0	0	0	0	0	0	0	0
0	0	0	0	0	0	0	0	0	0	0	0	0	0	0	0	0	0	0
0	0	0	0	0	0	0	0	0	0	0	0	0	0	0	0	0	0	0
0	0	0	0	0	0	0	0	0	0	0	0	0	0	0	0	0	0	0
0	0	0	0	0	0	0	0	0	0	0	0	0	0	0	0	0	0	0
0	0	0	0	0	0	0	0	0	0	0	0	0	0	0	0	0	0	0
0	0	0	0	0	0	0	0	0	0	0	0	0	0	0	0	0	0	0
0	0	0	0	0	0	0	0	0	0	0	0	0	0	0	0	0	0	0
0	0	0	0	0	0	0	0	0	0	0	0	0	0	0	3	3	3	0
0	0	0	0	0	0	0	0	0	0	0	0	0	0	0	0	0	0	0
0	0	0	0	0	0	0	0	0	0	0	0	0	0	0	0	0	0	0
0	0	0	0	0	0	0	0	0	0	0	0	0	0	0	0	0	0	0
0	0	0	0	0	0	0	0	0	0	0	0	0	0	0	0	0	0	0
0	0	0	0	0	0	0	0	0	0	0	0	0	0	0	0	0	0	0
0	0	0	0	0	0	0	0	0	0	0	0	0	0	0	0	0	0	0
0	0	0	0	0	0	0	0	0	0	0	0	0	0	0	0	0	0	0
0	0	0	0	0	0	0	0	0	0	0	0	0	0	0	0	0	0	0
0	0	0	0	0	0	0	0	0	0	0	0	0	0	0	0	0	0	0
0	0	0	0	0	0	0	0	0	0	0	0	0	0	0	0	0	0	0
0	0	0	0	0	0	0	0	0	0	0	0	0	0	0	0	0	0	0
0	0	0	0	0	0	0	0	0	0	0	0	0	0	0	0	0	0	0

0	0	0	0	0	0	0	0	0	0	0	0	0	0	0	0	0	0	0
0	0	0	0	0	0	0	0	0	0	0	0	0	0	0	0	0	0	0
0	0	0	0	0	0	0	0	0	0	0	0	0	0	0	0	0	0	0
0	0	0	0	0	0	0	0	0	0	0	0	0	0	0	0	0	0	0
0	0	0	0	0	0	0	0	0	0	0	0	0	0	0	0	0	0	0
0	0	0	0	0	0	0	0	0	0	0	0	0	0	0	0	0	0	0
0	0	0	0	0	0	0	0	0	0	0	0	0	0	0	0	0	0	0
0	0	0	0	0	0	0	0	0	0	0	0	0	0	0	0	0	0	0
0	0	0	0	0	0	0	0	0	0	0	0	0	0	0	0	0	0	0
0	0	0	0	0	0	0	0	0	0	0	0	0	0	0	0	0	0	0
0	0	0	0	0	0	0	0	0	0	0	0	0	0	0	0	0	0	0
0	0	0	0	0	0	0	0	0	0	0	0	0	0	0	0	0	0	0
0	0	0	0	0	0	0	0	0	0	0	0	0	0	0	0	0	0	0
0	0	0	0	0	0	0	0	0	0	0	0	0	2	2	2	2	2	2
2	2	2	0	0	0	0	0	0	0	0	0	0	0	0	0	0	0	0
0	0	0	0	0	0	0	0	0	0	0	0	0	0	0	0	0	0	0
0	0	0	0	0	0	0	0	0	0	0	0	0	0	0	0	0	0	0
0	0	0	0	0	0	0	0	0	0	0	0	0	0	0	0	0	0	0
0	0	0	0	0	0	0	0	0	0	0	0	0	0	0	0	0	0	0
0	0	0	0	0	0	0	0	0	0	0	0	0	0	0	0	0	0	0
0	0	0	0	0	0	0	0	0	0	0	0	0	0	0	0	0	0	0
0	0	0	0	0	0	0	0	0	0	0	0	0	0	0	0	0	0	0
0	0	0	0	0	0	0	0	0	0	0	0	0	0	0	0	0	0	0
0	0	0	0	0	0	0	0	0	0	0	0	0	0	0	0	0	0	0
0	0	0	0	0	0	0	0	0	0	0	0	0	0	0	0	0	0	0
0	0	0	0	0	0	0	0	0	0	0	0	0	0	0	0	0	0	0
0	0	0	0	0	0	0	0	0	0	0	0	0	0	0	0	0	0	0
0	0	0	0	0	0	0	0	0	0	0	0	0	0	0	0	0	0	0
0	0	0	0	0	0	0	0	0	0	0	0	0	0	0	0	0	0	0
0	0	0	0	0	0	0	0	0	0	0	0	0	0	0	0	0	0	0
0	0	0	0	0	0	0	0	0	0	0	0	0	0	0	0	0	0	0
0	0	0	0	0	0	0	0	0	0	0	0	0	0	0	0	0	0	0
0	0	0	0	0	0	0	0	0	0	0	0	0	0	0	0	0	0	0
0	0	0	0	0	0	0	0	0	0	0	0	0	0	0	0	0	0	0
0	0	0	0	0	0	0	0	0	0	0	0	0	0	0	0	0	0	0
0	0	0	0	0	0	0	0	0	0	0	0	0	0	0	0	0	0	0
0	0	0	0	0	0	0	0	0	0	0	0	0	0	0	0	0	0	0

0	0	0	0	0	0	0	0	0	0	0	0	0	0	0	0	0	0	0
0	0	0	0	0	0	0	0	0	0	0	0	0	0	0	0	0	0	0
0	0	0	0	0	0	0	0	0	0	0	0	0	0	0	0	0	0	0
0	0	0	0	0	0	0	0	0	0	0	0	0	0	0	0	0	0	0
0	0	0	0	0	0	0	0	0	0	0	0	0	0	0	0	0	0	0
0	0	0	0	0	0	0	0	0	0	0	0	0	0	0	0	0	0	0
0	0	0	0	0	0	0	0	0	0									
Jan_DL045	1265	1368	469	631	165	323	98	103	339	288	148	88	269	31	310	148	34	117
207	0	288	751	148	240	87	113	80	89	102	0	51	82	130	66	73	69	0
67	118	0	27	80	55	80	0	21	55	0	0	121	78	82	0	0	31	0
0	59	0	66	0	98	62	72	24	183	28	81	318	73	47	94	24	64	303
45	0	45	37	0	51	12	44	102	44	85	0	52	104	53	0	56	10	0
8	22	0	0	0	0	0	116	14	10	0	31	28	97	10	33	0	0	77
0	0	45	10	6	19	0	0	0	0	13	72	6	20	0	0	0	0	0
0	0	0	12	0	0	0	0	0	0	12	0	7	0	0	5	0	0	0
0	12	11	22	0	0	0	0	0	0	0	0	0	0	56	0	0	0	23
11	0	9	0	0	19	0	0	0	34	0	0	0	0	0	17	20	0	0
0	0	19	45	0	0	0	0	0	22	0	0	0	0	0	0	0	0	0
0	0	31	0	13	0	0	0	0	0	0	0	0	0	0	0	0	0	61
0	0	39	0	0	0	0	0	13	20	0	8	0	7	0	0	37	63	0
0	0	0	8	0	0	0	0	0	0	0	0	0	0	12	0	0	14	0
0	0	0	0	0	0	0	0	0	0	5	13	0	0	4	13	0	0	0
0	15	0	26	0	0	0	9	11	0	0	0	0	0	0	0	0	0	0
0	6	0	0	0	0	0	19	0	0	0	13	0	0	0	0	0	0	4
13	0	0	10	0	0	0	0	2	0	0	0	0	6	19	5	0	0	0
0	0	0	0	0	0	0	0	0	0	19	0	16	0	27	0	0	0	10
0	5	0	0	0	0	10	0	0	0	0	0	0	0	0	0	0	0	6
0	0	0	0	0	0	17	0	0	0	18	0	21	0	0	0	0	0	0
0	0	0	18	0	0	0	0	0	0	0	0	0	8	0	0	4	0	0
0	0	0	0	0	0	0	0	0	0	0	0	11	0	0	0	0	0	0
0	0	0	0	0	0	0	0	0	0	19	0	0	0	0	0	0	0	0
0	0	0	0	7	0	0	11	0	0	0	0	0	32	0	0	0	0	0
0	0	0	0	0	0	0	3	0	0	0	18	0	0	0	0	0	0	9
0	0	0	0	0	0	0	0	0	0	0	0	0	0	0	0	0	0	0
0	0	0	0	0	0	0	0	0	7	0	0	0	0	0	0	0	30	0
6	0	0	0	0	25	0	0	0	0	0	0	0	0	0	0	0	0	0
0	5	0	0	0	0	0	0	31	0	10	0	0	0	0	36	0	0	0

0	0	0	0	0	0	0	0	0	0	0	0	0	0	0	0	0	0	0
0	0	0	0	0	0	0	0	0	0	0	0	0	0	0	0	0	0	0
0	0	0	0	0	0	0	0	9	0	0	0	0	0	0	0	0	8	0
0	0	0	0	0	0	0	0	0	0	0	0	0	0	0	0	0	0	0
0	0	0	0	0	0	0	26	12	0	0	4	3	26	0	0	0	0	0
0	0	0	0	0	0	0	0	0	0	8	0	0	0	0	10	0	0	0
0	0	0	0	0	0	0	0	0	0	0	0	0	0	0	0	0	0	0
0	0	0	0	0	0	0	0	0	0	0	0	0	0	0	0	0	0	0
0	0	0	0	0	0	0	0	0	0	0	0	0	0	0	0	0	0	0
0	0	0	0	0	0	0	0	0	0	0	0	0	0	0	0	0	0	0
0	0	0	0	0	0	0	0	0	0	0	0	0	0	0	0	0	0	0
0	0	0	0	0	0	0	0	0	0	0	9	0	0	0	0	0	0	0
0	0	0	0	0	0	0	0	0	0	0	0	0	0	0	0	0	0	0
0	0	0	0	0	0	0	0	0	0	0	0	0	0	0	0	0	0	0
0	0	0	0	0	0	0	0	0	0	0	0	0	0	0	0	0	0	0
0	0	0	0	5	0	0	0	0	0	0	0	0	0	0	0	0	0	0
0	0	4	0	0	0	0	0	0	17	0	0	0	0	0	0	0	0	0
0	0	0	0	0	0	0	0	0	0	0	0	0	0	0	0	0	0	0
0	0	0	0	0	0	0	0	0	0	0	0	0	0	0	0	0	0	0
0	0	0	0	0	0	0	0	0	0	0	0	0	0	0	0	0	0	0
0	0	0	0	0	0	0	0	0	0	0	0	0	0	0	0	0	0	0
0	0	0	0	0	0	0	0	0	0	0	0	0	0	0	0	0	9	0
0	0	0	0	0	0	0	0	0	0	0	0	0	0	0	0	0	0	0
0	0	0	0	0	0	0	0	0	0	0	0	0	0	0	0	0	0	0
0	0	0	0	0	0	0	0	0	0	0	0	0	0	0	0	0	0	0
0	0	0	0	0	0	0	0	0	0	0	0	0	0	0	0	0	0	0
0	0	0	0	0	0	0	0	0	0	0	0	0	0	0	0	0	0	0
0	0	0	0	0	0	0	0	0	0	0	0	0	0	0	0	0	0	0
0	0	0	0	0	0	0	0	0	0	0	0	0	0	0	0	0	0	0
0	0	0	0	0	0	0	0	0	0	0	0	0	0	0	0	0	0	0
0	0	0	0	0	0	0	0	0	0	0	0	0	0	0	0	0	0	0
0	0	0	0	0	0	0	0	0	0	0	0	0	0	0	0	0	0	0
0	0	0	0	0	0	0	0	0	0	0	0	0	0	0	0	0	0	0
0	0	0	0	0	0	0	0	0	0	0	0	0	0	0	0	0	0	0
0	0	0	0	0	0	0	0	0	0	0	0	0	0	0	0	0	0	0
0	0	0	0	0	0	0	0	0	0	0	0	0	0	0	0	0	0	0
0	0	0	0	0	0	0	0	0	0	0	0	0	0	0	0	0	0	0

0	0	0	0	0	0	0	0	0	0	0	0	0	0	0	0	0	0	0
0	0	0	0	0	0	0	0	0	0	0	0	0	0	0	0	0	0	0
0	0	0	0	0	0	0	0	0	0	0	0	0	0	0	0	0	0	0
0	0	0	0	0	0	0	0	0	0	0	0	0	0	0	0	0	0	0
0	0	0	0	0	0	0	0	0	0	0	0	0	0	0	0	0	0	0
0	0	0	0	0	0	0	0	0	0	0	0	0	0	0	0	0	0	0
0	0	0	0	0	0	0	0	0	0	0	0	0	0	0	0	0	0	0
0	0	0	0	0	0	0	0	0	0	0	0	0	0	0	0	0	0	0
0	0	0	0	0	0	0	0	0	0	0	0	0	0	0	0	0	0	0
0	0	0	0	0	0	0	0	0	0	0	0	0	0	0	0	0	0	0
0	0	0	0	0	0	0	0	0	0	0	0	0	0	0	0	0	0	0
0	0	0	0	0	0	0	0	0	0	0	0	0	0	0	0	0	0	0
0	0	0	0	0	0	0	0	0	0	0	0	0	0	0	0	0	0	0
0	0	0	0	0	0	0	0	0	0	0	0	0	0	0	0	0	0	0
0	0	0	0	0	0	0	0	0	0	0	0	0	0	0	0	0	0	0
0	0	0	0	0	0	0	0	0	0	0	0	0	0	0	0	0	0	0
0	0	0	0	0	0	0	0	0	0	0	0	0	0	0	0	0	0	0
7	0	0	0	0	0	0	0	0	0	0	0	0	0	0	0	0	0	0
0	0	0	0	0	0	0	0	0	0	0	0	0	0	0	0	0	0	0
0	0	0	0	0	0	0	0	0	0	0	0	0	0	0	0	0	0	0
0	0	0	0	0	0	0	0	0	0	0	0	0	0	0	0	0	0	0
0	0	0	0	0	0	0	0	0	0	0	0	0	0	0	0	0	0	0
0	0	0	0	0	0	0	0	0	0	0	0	0	0	0	0	0	0	0
0	0	0	0	0	0	0	0	0	0	0	0	0	0	0	0	0	0	0
0	0	0	0	0	0	0	0	0	0	0	0	0	0	0	0	0	0	0
0	0	0	0	0	0	0	0	0	0	0	0	0	0	0	0	0	0	0
0	0	0	0	0	0	0	0	0	0	0	0	0	0	0	0	0	0	0
0	0	0	0	0	0	0	0	0	0	0	0	0	0	0	0	0	0	0
0	0	0	0	0	0	0	0	0	0	0	0	0	0	0	0	0	0	0
0	0	0	0	0	0	0	0	0	0	0	0	0	0	0	0	0	0	0
0	0	0	0	0	0	0	0	0	0	0	0	0	0	0	0	0	0	0
0	5	5	0	0	0	0	0	0	0	0	0	0	0	0	0	0	0	0
0	0	0	0	0	0	0	0	0	0	0	0	0	0	0	0	0	0	0
0	0	0	0	0	0	0	0	0	0	0	0	0	0	0	0	0	0	0
0	0	0	0	0	0	0	0	0	0	0	0	0	0	0	0	0	0	0
0	0	0	0	0	0	0	0	0	0	0	0	0	0	0	0	0	0	0
0	0	0	0	0	0	0	0	0	0	0	0	0	0	0	0	0	0	0

0	0	0	0	0	0	0	0	0	0	0	0	0	0	0	0	0	0	0
0	0	0	0	0	0	0	0	0	0	0	0	0	0	0	0	0	0	0
0	0	0	0	0	0	0	0	0	0	0	0	0	0	0	0	0	0	0
0	0	0	0	0	0	0	0	0	0	0	0	0	0	4	4	0	0	0
0	0	0	0	0	0	0	0	0	0	0	0	0	0	0	0	0	0	0
0	0	0	0	0	0	0	0	0	0	0	0	0	0	0	0	0	0	0
0	0	0	0	0	0	0	0	0	0	0	0	0	0	0	0	0	0	0
0	0	0	0	0	0	0	0	0	0	0	0	0	0	0	0	0	0	0
0	0	0	0	0	0	0	0	0	0	0	0	0	0	0	0	0	0	0
0	0	0	0	0	0	0	0	0	0	0	0	0	0	0	0	0	0	0
0	0	0	0	0	0	0	0	0	0	0	0	0	0	0	0	0	0	0
0	0	0	0	0	0	0	0	0	0	0	0	0	0	0	0	0	0	0
0	0	0	0	0	0	0	0	0	0	0	0	0	0	0	0	0	0	0
0	0	0	0	0	0	0	0	0	0	0	0	0	0	0	0	0	0	0
0	0	0	0	0	0	0	0	0	0	0	0	0	0	0	0	0	0	0
0	0	0	0	0	0	0	0	0	0	0	0	0	0	0	0	0	0	0
0	0	0	0	0	0	0	0	0	0	0	0	0	0	0	0	0	0	0
0	0	0	0	0	0	0	0	0	0	0	0	0	0	0	0	0	0	0
0	0	0	0	0	0	0	0	0	0	0	0	0	0	0	3	3	3	3
3	0	0	0	0	0	0	0	0	0	0	0	0	0	0	0	0	0	0
0	0	0	0	0	0	0	0	0	0	0	0	0	0	0	0	0	0	0
0	0	0	0	0	0	0	0	0	0	0	0	0	0	0	0	0	0	0
0	0	0	0	0	0	0	0	0	0	0	0	0	0	0	0	0	0	0
0	0	0	0	0	0	0	0	0	0	0	0	0	0	0	0	0	0	0
0	0	0	0	0	0	0	0	0	0	0	0	0	0	0	0	0	0	0
0	0	0	0	0	0	0	0	0	0	0	0	0	0	0	0	0	0	0
0	0	0	0	0	0	0	0	0	0	0	0	0	0	0	0	0	0	0
0	0	0	0	0	0	0	0	0	0	0	0	0	0	0	0	0	0	0
0	0	0	0	0	0	0	0	0	0	0	0	0	0	0	0	0	0	0
0	0	0	0	0	0	0	0	0	0	0	0	0	0	0	0	0	0	0
0	0	0	0	0	0	0	0	0	0	0	0	0	0	0	0	0	0	0
0	0	0	0	0	0	0	0	0	0	0	0	0	0	0	0	0	0	0
0	0	0	0	0	0	0	0	0	0	0	0	0	0	0	0	0	0	0
0	0	0	0	0	0	0	0	0	0	0	0	0	0	0	0	0	0	0
0	0	0	0	0	0	0	0	0	0	0	0	0	0	0	0	0	0	0
0	0	0	0	0	0	0	0	0	0	0	0	0	0	0	0	0	0	0
0	0	0	0	0	0	0	0	0	0	0	0	0	0	0	0	0	0	0

0	0	0	0	0	0	0	0	0	0	0	0	0	0	0	0	0	0	0
0	0	0	0	0	0	0	0	0	0	0	0	0	0	0	0	0	0	0
0	0	0	0	0	0	0	0	0	0	0	0	0	0	0	0	0	0	0
0	0	0	0	0	0	0	0	0	0	0	0	0	0	0	0	0	0	0
0	0	0	0	0	0	0	0	0	0	0	0	0	0	0	0	0	0	0
0	0	0	0	0	0	0	0	0	0	0	0	0	0	0	0	0	0	0
0	0	0	0	0	0	0	0	0	0	0	0	0	0	0	0	0	0	0
0	0	0	0	0	0	0	0	0	0	0	0	0	0	0	0	0	0	0
0	0	0	0	0	0	0	2	2	2	2	2	2	2	2	2	2	2	0
0	0	0	0	0	0	0	0	0	0	0	0	0	0	0	0	0	0	0
0	0	0	0	0	0	0	0	0	0	0	0	0	0	0	0	0	0	0
0	0	0	0	0	0	0	0	0	0	0	0	0	0	0	0	0	0	0
0	0	0	0	0	0	0	0	0	0	0	0	0	0	0	0	0	0	0
0	0	0	0	0	0	0	0	0	0	0	0	0	0	0	0	0	0	0
0	0	0	0	0	0	0	0	0	0	0	0	0	0	0	0	0	0	0
0	0	0	0	0	0	0	0	0	0	0	0	0	0	0	0	0	0	0
0	0	0	0	0	0	0	0	0	0	0	0	0	0	0	0	0	0	0
0	0	0	0	0	0	0	0	0	0	0	0	0	0	0	0	0	0	0
0	0	0	0	0	0	0	0	0	0	0	0	0	0	0	0	0	0	0
0	0	0	0	0	0	0	0	0	0	0	0	0	0	0	0	0	0	0
0	0	0	0	0	0	0	0	0	0	0	0	0	0	0	0	0	0	0
0	0	0	0	0	0	0	0	0	0	0	0	0	0	0	0	0	0	0
0	0	0	0	0	0	0	0	0	0	0	0	0	0	0	0	0	0	0
0	0	0	0	0	0	0	0	0	0	0	0	0	0	0	0	0	0	0
0	0	0	0	0	0	0	0	0	0	0	0	0	0	0	0	0	0	0
0	0	0	0	0	0	0	0	0	0	0	0	0	0	0	0	0	0	0
0	0	0	0	0	0	0	0	0	0	0	0	0	0	0	0	0	0	0
0	0	0	0	0	0	0	0	0	0	0	0	0	0	0	0	0	0	0
0	0	0	0	0	0	0	0	0	0	0	0	0	0	0	0	0	0	0
0	0	0	0	0	0	0	0	0	0	0	0	0	0	0	0	0	0	0
0	0	0	0	0	0	0	0	0	0	0	0	0	0	0	0	0	0	0
0	0	0	0	0	0	0	0	0	0	0	0	0	0	0	0	0	0	0
0	0	0	0	0	0	0	0	0	0	0	0	0	0	0	0	0	0	0
0	0	0	0	0	0	0	0	0	0	0	0	0	0	0	0	0	0	0
0	0	0	0	0	0	0	0	0	0	0	0	0	0	0	0	0	0	0
0	0	0	0	0	0	0	0	0	0									

Jan_DL046	1944	1288	1922	765	213	319	397	395	227	746	314	798	513	201	134	525	227	177
219	0	235	73	135	182	222	294	193	193	107	666	174	232	335	134	278	676	465
184	22	75	70	126	166	90	181	65	215	143	182	0	118	129	162	0	108	398
411	80	164	41	94	0	46	78	104	104	35	77	68	0	0	0	55	17	0
49	160	47	36	52	65	75	46	0	70	98	61	22	0	22	0	28	0	0
0	28	71	0	72	29	15	0	0	30	41	0	24	0	26	0	67	0	0
62	0	0	30	32	0	0	0	0	0	17	42	13	16	53	0	0	0	0
0	8	0	12	0	35	68	0	0	30	0	0	9	38	0	0	0	0	0
0	0	0	5	0	0	0	0	0	0	16	0	27	0	66	59	0	0	10
32	0	11	42	6	0	0	0	0	0	0	0	0	0	60	0	0	0	0
0	0	0	0	0	0	0	0	0	6	40	0	0	0	0	0	0	0	0
0	0	47	0	0	64	30	0	0	0	0	0	0	0	0	0	98	22	0
0	0	13	0	0	28	0	0	3	3	0	87	0	16	0	10	0	47	0
0	0	0	5	0	0	0	12	0	16	0	0	0	0	0	5	0	22	18
10	0	0	0	21	7	0	0	0	59	0	4	0	5	0	5	0	0	0
0	0	0	0	0	0	0	0	4	0	0	0	0	0	0	0	0	0	29
0	2	9	0	0	0	0	0	0	0	12	0	0	14	0	0	0	7	21
0	61	5	0	0	46	0	7	5	0	0	8	38	16	0	0	0	26	7
5	25	0	0	16	11	0	0	0	0	0	0	25	10	0	0	0	0	27
7	0	0	0	0	0	0	0	0	0	31	0	0	70	7	0	0	0	12
0	0	0	68	8	0	0	0	4	0	0	0	0	0	0	0	0	0	0
0	0	0	0	0	0	8	3	0	0	0	0	0	0	0	0	0	0	0
0	67	0	0	0	0	0	0	0	0	0	0	0	0	7	0	0	0	0
0	0	0	0	0	0	0	0	0	0	0	0	18	0	0	0	7	7	0
0	0	0	0	0	0	0	0	0	0	0	0	0	0	0	0	0	0	0
0	0	0	0	2	0	0	6	0	0	0	0	0	0	0	0	0	0	0
0	0	0	27	0	0	0	0	0	0	0	0	0	0	0	0	0	4	0
0	0	0	0	0	0	0	0	0	0	0	0	0	0	0	0	0	0	0
0	0	0	0	0	0	0	0	0	0	0	0	0	0	0	0	0	0	0
0	0	0	0	0	0	0	0	0	0	11	0	0	0	0	9	0	0	0
22	0	0	5	0	0	0	0	0	0	0	0	0	0	0	0	0	0	0
0	0	0	0	0	0	0	0	0	0	0	0	0	0	0	5	0	0	0
0	0	8	0	0	0	0	10	0	0	0	0	0	0	0	0	0	0	0
0	34	0	0	33	0	0	0	0	0	10	0	0	0	0	0	0	0	0
0	14	6	0	14	0	0	0	0	0	0	0	0	0	0	12	0	0	0
0	0	0	0	0	0	0	0	0	0	0	0	0	0	0	0	0	0	0
15	0	0	0	0	0	0	0	0	0	0	0	0	0	0	0	0	0	0

0	0	0	0	0	0	0	12	0	0	0	0	0	0	0	0	0	0	0
0	0	0	0	0	0	0	0	0	0	0	0	0	0	0	0	0	0	0
0	0	0	0	0	0	0	0	0	0	0	0	0	0	0	0	0	0	0
0	0	0	16	0	0	0	0	0	0	0	0	0	0	0	0	0	0	0
0	0	0	0	0	0	0	0	0	0	0	0	0	0	0	0	0	0	13
0	0	0	0	0	0	0	0	0	0	0	0	0	0	0	0	0	0	0
0	0	0	0	0	0	0	0	6	0	0	13	0	3	0	0	0	0	0
0	0	0	0	0	0	0	0	0	0	0	0	0	0	0	0	0	0	0
0	0	0	0	8	0	0	0	0	0	0	0	0	0	0	0	0	0	0
0	0	0	0	0	0	0	0	0	0	0	0	0	0	0	0	0	0	0
0	0	0	0	0	0	0	0	0	0	0	0	0	0	0	0	0	0	0
0	0	0	0	0	0	0	0	0	0	0	0	0	0	0	0	0	0	0
0	0	0	0	0	0	0	0	0	0	0	0	0	0	0	0	0	0	0
0	0	0	0	0	0	0	0	0	0	0	0	0	0	0	0	0	0	0
0	0	0	0	0	0	0	0	0	0	0	0	0	0	0	0	0	0	0
0	0	0	0	0	0	0	0	0	0	0	0	15	0	0	0	0	0	0
0	0	0	0	0	0	0	0	0	0	0	0	0	0	0	0	0	0	0
0	0	0	24	0	0	0	0	5	0	0	0	0	0	0	0	0	0	0
0	0	0	0	0	0	0	0	0	0	0	0	0	0	0	0	0	0	0
0	0	0	0	0	0	0	0	0	0	0	0	0	0	0	0	0	0	0
0	0	0	0	0	0	0	0	0	0	0	0	0	0	0	0	0	0	0
0	0	0	0	0	0	0	0	0	0	0	0	0	0	0	0	0	0	0
0	0	0	0	0	0	0	0	0	0	0	0	0	0	0	0	0	0	0
0	0	0	0	0	0	0	0	0	0	0	0	0	0	0	0	0	0	0
0	0	0	0	0	0	0	0	0	0	0	0	0	0	0	0	0	0	0
0	0	0	0	0	0	0	0	0	0	0	0	0	0	0	0	0	0	0
0	0	0	0	0	0	0	0	0	0	0	0	0	0	0	0	0	0	0
0	0	0	0	0	0	0	0	0	0	0	0	0	0	0	0	0	0	0
0	0	0	0	0	0	0	0	0	0	0	0	0	0	0	0	2	0	10
0	0	0	0	0	0	0	0	0	0	0	0	0	0	0	0	0	0	0
0	0	0	0	0	0	0	0	0	0	0	0	0	0	0	0	0	0	0
0	0	0	0	0	0	0	0	0	0	0	0	0	0	0	0	0	0	0
0	0	0	0	0	0	0	0	0	0	0	0	0	0	0	0	0	0	0
0	0	0	0	0	0	0	0	0	0	0	0	0	0	0	0	0	0	0
0	0	0	0	0	0	0	0	0	0	0	0	0	0	0	0	0	0	0
0	0	0	0	0	0	0	0	0	0	0	0	0	0	0	0	0	0	0
0	0	0	0	0	0	0	0	0	0	0	0	0	0	0	0	0	0	0

0	0	0	0	0	0	0	0	0	0	0	0	0	0	0	0	0	0	0
0	0	0	0	0	0	0	0	0	0	0	0	0	0	0	0	0	0	0
10	0	0	0	0	0	0	0	0	0	0	0	0	0	0	0	0	0	0
0	0	0	0	0	0	0	0	0	0	0	0	0	0	0	0	0	0	0
0	0	0	0	0	0	4	0	0	0	0	0	0	0	0	0	0	0	0
0	0	0	0	0	0	0	0	0	0	0	0	0	0	0	0	0	0	0
0	0	0	0	0	0	0	0	0	0	0	0	0	0	0	0	0	0	0
0	0	0	0	0	0	0	0	0	0	0	0	0	0	0	0	0	0	0
0	0	0	0	0	0	0	0	0	0	0	0	0	0	0	0	0	0	0
0	0	0	0	0	0	0	0	0	0	0	0	0	0	0	0	0	0	0
0	0	0	0	0	0	0	0	0	0	0	0	0	0	0	0	0	0	0
0	0	0	0	0	0	0	0	0	0	0	0	0	0	0	0	0	0	0
0	0	0	0	0	11	0	0	0	0	0	0	0	0	0	0	0	0	0
0	0	0	0	0	0	0	0	0	0	0	0	0	0	0	0	0	0	0
0	0	0	0	0	0	0	0	0	0	0	0	0	0	0	0	0	0	0
0	0	0	0	0	0	0	0	0	0	0	0	0	0	0	0	0	0	0
0	0	0	0	0	0	0	0	0	0	0	0	0	0	0	0	0	0	0
0	0	0	0	0	0	0	0	0	0	0	0	0	0	0	0	0	0	0
0	0	0	0	0	0	0	0	0	0	0	0	0	0	0	0	0	0	0
0	0	0	0	0	0	0	0	0	0	0	0	0	0	0	0	0	0	0
0	9	0	0	0	0	0	0	0	0	0	0	0	0	0	0	0	0	0
0	0	0	0	0	0	0	0	0	0	0	0	0	0	0	0	0	0	0
0	0	0	0	0	0	0	0	0	0	0	0	0	0	0	0	0	0	0
0	0	0	0	0	0	0	0	0	0	0	0	0	0	0	0	0	0	0
0	0	0	0	0	0	0	0	0	0	0	0	0	0	0	0	0	0	0
0	0	0	0	0	0	0	0	0	0	0	0	0	0	0	0	0	0	0
0	0	0	0	0	0	0	0	0	0	0	0	0	0	0	0	0	0	0
0	0	0	0	0	0	0	0	0	0	0	0	0	0	0	0	0	0	0
0	0	0	0	0	0	0	0	0	0	0	0	0	0	0	0	0	0	0
0	0	0	0	0	0	0	0	0	0	0	0	0	0	0	0	0	0	0
0	0	0	0	0	0	0	0	0	0	0	0	0	0	0	0	0	0	0
0	0	0	0	0	0	0	0	0	0	0	0	0	0	0	0	0	0	0
0	0	0	7	0	0	0	0	0	0	0	0	0	0	0	0	0	0	0
0	0	0	0	0	0	0	0	0	0	0	0	0	0	0	0	0	0	0
0	0	0	0	0	0	0	0	0	0	0	0	0	0	0	0	0	0	0
0	0	0	0	0	0	0	0	0	0	0	0	0	0	0	0	0	0	0
0	0	0	0	0	0	0	0	0	0	0	0	0	0	0	0	0	0	0

0	0	0	0	0	0	0	0	0	0	0	0	0	0	0	0	0	0	0
0	0	0	0	0	0	0	0	0	0	0	0	0	0	0	0	0	0	0
0	0	0	0	0	0	0	0	0	0	0	0	0	0	0	0	0	0	0
0	0	0	0	0	0	0	0	0	0	0	0	0	0	0	0	0	0	0
0	0	0	0	0	0	0	0	0	0	0	0	0	0	0	0	0	0	0
0	0	0	0	0	0	0	0	0	0	0	0	0	0	0	0	0	0	0
0	0	0	0	0	0	0	0	0	0	0	0	0	0	0	0	0	0	0
0	0	0	0	0	0	0	0	0	0	0	0	0	0	0	0	0	0	0
0	0	0	0	0	0	0	0	0	0	0	0	0	0	0	0	0	0	0
0	0	0	0	0	0	0	0	0	0	0	0	0	0	0	0	0	0	0
0	0	0	0	0	0	0	0	0	0	0	0	0	0	0	0	0	0	0
0	0	0	0	0	0	0	0	0	0	0	0	0	0	0	0	0	0	0
0	0	0	0	0	0	0	0	0	0	0	0	0	0	0	0	0	0	0
0	0	0	0	0	0	0	0	0	0	0	0	0	0	0	0	0	0	0
0	0	0	0	0	0	0	0	0	0	0	0	0	0	0	0	0	0	0
0	0	0	0	0	0	0	0	0	0	0	0	0	0	0	0	0	0	0
0	0	0	0	0	0	0	0	0	0	0	0	0	0	0	0	0	0	0
0	0	0	0	0	0	0	0	0	0	0	0	0	0	0	0	0	0	0
0	0	0	0	0	0	0	0	0	0	0	0	0	0	0	0	0	0	0
0	0	0	0	0	0	0	0	0	0	0	0	0	0	0	0	0	0	0
0	0	0	0	0	0	0	0	0	0	0	0	0	0	0	0	0	0	0
0	0	0	0	0	0	0	0	0	0	0	0	0	0	0	0	0	0	0
0	0	0	0	0	0	0	0	0	0	0	0	0	0	0	0	0	0	0
0	0	0	0	0	0	0	0	0	0	0	0	0	0	0	0	0	0	0
0	0	0	0	0	0	0	0	0	0	0	0	4	4	0	0	0	0	0
0	0	0	0	0	0	0	0	0	0	0	0	0	0	0	0	0	0	0
0	0	0	0	0	0	0	0	0	0	0	0	0	0	0	0	0	0	0
0	0	0	0	0	0	0	0	0	0	0	0	0	0	0	0	0	0	0
0	0	0	0	0	0	0	0	0	0	0	0	0	0	0	0	0	0	0
0	0	0	0	0	0	0	0	0	0	0	0	0	0	0	0	0	0	0
0	0	0	0	0	0	0	0	0	0	0	0	0	0	0	0	0	0	0
0	0	0	0	0	0	0	0	0	0	0	0	0	0	0	0	0	0	0
0	0	0	0	0	0	0	0	0	0	0	0	0	0	0	0	0	0	0
0	0	0	0	0	0	0	0	0	0	0	0	0	0	0	0	0	0	0
0	0	0	0	0	0	0	0	0	0	0	0	0	0	0	0	0	0	0
0	0	0	0	0	0	0	0	0	0	0	0	0	0	0	0	0	0	0
0	0	0	0	0	0	0	0	0	0	0	0	0	0	0	0	0	0	0

0	0	0	0	0	0	0	0	0	0	0	0	0	0	0	0	0	0	0
0	0	0	0	0	0	0	0	0	0	0	0	0	0	0	0	0	0	0
0	0	0	0	0	0	0	0	0	0	0	0	0	0	0	0	0	0	0
0	0	0	0	0	0	0	0	0	0	0	0	0	0	0	0	0	0	0
0	0	0	0	0	0	0	0	0	0	0	0	0	0	0	0	0	0	0
0	0	0	0	0	0	0	0	0	0	0	0	0	0	0	0	0	0	0
0	0	0	0	0	0	0	0	0	0	0	0	0	0	0	0	0	0	0
0	0	0	0	0	0	0	0	0	0	0	0	0	0	0	0	0	0	0
0	0	0	0	0	0	0	0	0	0	0	0	0	0	0	0	0	0	0
0	0	0	0	0	0	0	0	0	0	0	0	0	0	0	0	0	0	0
0	0	0	0	0	0	0	0	0	0	0	0	0	0	0	0	0	0	0
0	0	0	0	0	0	0	0	0	0	0	0	0	0	0	0	0	0	0
0	0	0	0	0	0	0	0	0	0	0	0	0	0	0	0	0	0	0
0	0	0	0	0	0	0	0	0	0	0	0	0	0	0	0	0	0	0
0	0	0	0	0	0	0	0	0	0	0	0	0	0	0	0	0	0	0
0	0	0	0	0	0	0	0	0	0	0	0	0	0	0	0	0	0	0
0	0	0	0	0	0	0	0	0	0	0	0	0	0	0	0	0	0	0
0	0	0	0	0	0	0	0	0	0	0	0	0	0	0	0	0	0	0
0	0	0	0	0	0	0	0	0	0	0	0	0	0	0	0	0	0	0
0	0	0	0	0	0	0	0	0	0	0	0	0	0	0	0	0	0	0
0	0	0	0	0	0	0	0	0	0	0	0	0	0	0	0	0	0	0
0	0	0	0	0	0	0	0	0	0	0	0	0	0	0	0	0	0	0
0	0	0	0	0	0	0	0	0	0	0	0	0	0	0	0	0	0	0
0	0	0	0	0	0	0	0	0	0	0	0	0	0	0	0	0	0	0
0	0	0	0	0	0	0	0	0	0	0	0	0	0	0	0	0	0	0
0	0	0	0	0	0	0	0	0	2	2	0	0	0	0	0	0	0	0
0	0	0	0	0	0	0	0	0	0	0	0	0	0	0	0	0	0	0
0	0	0	0	0	0	0	0	0	0	0	0	0	0	0	0	0	0	0
0	0	0	0	0	0	0	0	0	0									
Jan_DL047	2068	1824	446	218	754	186	169	182	50	212	101	316	144	207	256	432	226	128
174	0	145	27	74	124	139	220	134	0	76	555	123	74	71	61	172	0	354
0	44	40	39	46	52	71	33	60	159	43	89	16	71	0	80	11	28	288
341	10	91	69	40	0	22	40	30	31	44	48	152	0	4	0	60	0	0
48	88	0	36	243	44	39	0	67	43	0	0	40	17	35	0	16	75	0
0	19	0	0	0	334	0	0	0	19	79	36	28	0	21	0	78	0	0
0	0	8	22	47	69	0	0	0	0	104	0	16	54	0	7	0	0	0
0	0	0	25	0	7	57	0	8	13	12	0	62	39	26	60	0	0	0

0	0	0	46	0	0	0	0	0	0	34	0	10	0	13	108	11	23	0
24	0	11	0	0	0	0	0	0	0	0	32	0	0	99	0	0	0	0
0	50	0	0	0	0	0	25	0	25	97	0	0	50	0	0	0	28	0
23	0	0	0	0	0	30	0	0	0	0	8	0	0	0	0	58	10	6
0	0	25	0	0	0	0	0	0	0	0	8	5	4	0	12	0	0	0
0	0	0	0	0	0	0	13	0	0	0	0	0	17	0	4	0	0	17
0	0	0	0	0	9	5	0	0	0	0	0	0	11	13	6	26	0	0
0	11	0	20	0	0	0	14	10	0	0	0	4	0	0	0	31	0	0
0	0	0	0	0	0	0	0	0	17	0	15	0	8	0	0	0	0	0
8	0	0	0	0	0	0	0	25	0	0	6	24	0	0	0	0	0	0
3	3	0	0	0	8	0	0	0	0	0	0	0	0	0	0	0	0	33
24	0	27	0	0	0	0	0	0	0	18	0	5	0	0	22	0	0	0
0	0	0	0	0	7	7	25	0	0	0	30	0	0	7	26	0	0	0
0	0	0	0	0	0	0	0	0	0	0	19	12	0	0	0	0	0	0
0	0	0	0	0	0	0	0	0	0	0	0	9	0	0	0	0	0	0
0	0	0	0	0	0	0	0	0	0	0	0	12	0	0	0	0	0	0
9	0	0	0	0	0	0	0	0	0	0	0	0	0	0	0	0	0	0
0	0	0	0	7	0	0	0	0	0	0	9	0	0	0	0	0	0	0
0	0	0	0	0	0	0	0	0	0	0	0	0	0	0	0	0	0	0
0	0	0	0	0	4	0	0	0	0	0	0	0	0	0	0	0	0	0
0	0	0	0	0	0	0	0	0	10	0	0	0	0	0	0	0	0	0
0	4	0	0	0	0	0	0	0	19	0	0	0	0	0	0	0	0	0
0	0	0	0	0	0	0	0	0	0	0	0	0	0	0	0	0	0	0
0	0	0	0	0	0	0	0	0	0	0	0	0	0	0	0	0	0	0
0	0	0	0	5	12	0	0	0	0	0	0	0	0	0	0	0	0	0
0	0	0	0	0	0	0	0	0	0	0	0	0	0	9	0	0	0	0
0	10	0	0	3	0	0	0	0	0	0	0	0	0	0	0	0	0	0
0	0	0	0	0	0	0	0	0	0	0	0	0	0	12	0	0	0	7
0	0	0	0	0	0	30	0	0	0	0	0	0	0	0	6	0	0	0
0	0	0	0	0	0	0	0	0	0	0	0	0	0	0	0	0	0	0
0	0	0	0	0	0	0	0	26	0	0	0	0	0	3	0	0	0	0
0	0	0	0	0	0	0	10	0	0	0	7	0	0	16	0	0	0	0
0	0	0	0	0	0	0	0	0	0	0	0	0	0	0	0	0	0	0
0	0	0	0	0	0	0	0	0	0	7	0	0	0	11	0	0	0	0
0	0	0	0	0	0	0	0	0	0	0	0	0	11	0	0	0	0	0
0	0	8	0	0	0	0	0	0	0	0	0	0	0	0	0	0	0	0
0	0	0	4	0	0	0	0	0	0	0	0	0	0	0	0	0	0	0

0	0	0	0	0	0	0	0	0	0	0	0	0	0	0	0	0	0	0
0	0	0	0	0	0	0	0	0	0	0	0	0	0	0	0	0	0	0
0	0	0	0	0	0	0	0	0	0	0	0	0	0	0	0	0	0	0
0	0	0	0	0	0	0	0	0	0	0	0	0	0	0	0	0	0	0
0	0	0	0	0	0	0	0	0	0	0	0	0	0	0	0	0	0	0
0	0	0	0	0	0	0	0	5	0	0	0	0	0	0	0	0	0	0
12	0	0	0	6	0	0	0	0	0	0	0	0	0	0	0	0	0	0
0	0	0	0	0	0	0	0	0	0	0	0	0	0	0	0	0	0	0
0	0	0	0	0	0	0	0	0	0	0	0	0	0	0	0	0	23	0
0	0	0	0	0	0	0	0	0	0	0	0	0	0	0	0	0	0	0
0	0	0	0	0	0	0	0	0	0	0	0	0	0	0	0	0	0	0
0	0	0	0	0	0	0	0	0	0	0	0	0	0	0	0	0	0	0
0	0	0	0	0	0	0	0	0	0	0	0	0	0	0	0	0	0	0
0	0	0	0	0	0	0	0	0	0	0	0	0	0	0	0	0	0	0
0	0	0	0	0	0	0	0	0	0	0	0	0	0	0	0	0	0	0
0	0	0	0	0	0	0	0	0	0	0	0	0	0	0	0	0	0	0
0	0	0	0	0	0	0	0	0	0	0	0	0	0	0	0	0	0	0
0	0	0	0	0	0	0	0	0	0	0	0	0	0	0	0	0	0	0
0	0	0	0	0	0	0	0	0	0	0	0	0	0	0	0	0	0	0
0	0	0	0	0	0	0	0	0	0	0	0	0	0	0	0	0	0	0
0	0	0	0	0	0	0	0	0	0	0	0	0	0	0	0	0	0	0
0	0	0	0	0	0	0	0	0	0	0	0	0	0	0	0	0	0	0
0	0	0	0	0	0	0	0	0	0	0	0	0	0	0	0	0	0	0
0	0	0	0	0	0	0	0	0	0	0	0	0	0	0	0	0	0	0
0	0	0	0	0	0	0	0	0	0	0	0	0	0	0	0	0	0	0
0	0	0	0	0	0	0	0	0	0	0	0	0	0	0	0	0	0	0
0	0	0	0	0	0	0	0	0	0	0	0	0	0	0	0	0	0	0
0	0	0	0	0	0	0	0	0	0	0	0	0	0	0	0	0	0	0
0	0	0	0	0	0	0	0	0	0	0	0	0	0	0	0	0	0	0
0	0	0	0	0	0	0	0	0	0	0	0	0	0	0	0	0	0	0
0	0	0	0	0	0	0	0	0	0	0	0	0	0	0	0	0	0	0
0	0	0	0	0	0	0	0	0	0	0	0	0	0	0	0	0	0	0
0	0	0	0	0	0	0	0	0	0	0	0	0	0	0	0	0	0	0
0	0	0	0	0	0	0	0	0	0	0	0	0	0	0	0	0	0	0
0	0	0	0	0	0	0	0	0	0	0	0	0	0	0	0	0	0	0
0	0	0	0	0	0	0	0	0	0	0	0	0	0	0	0	0	0	0
0	0	0	0	0	0	0	0	0	0	0	0	0	0	0	0	0	0	0

0	0	0	0	0	0	0	0	0	0	0	0	0	0	0	0	0	0	0
0	0	0	0	0	0	0	0	0	0	0	0	0	0	0	0	0	0	0
0	0	0	0	0	0	0	0	0	0	0	0	0	0	0	0	0	0	0
0	0	0	0	0	0	0	0	0	0	0	0	0	0	0	0	0	0	0
0	0	0	0	0	0	0	0	0	0	0	0	0	0	0	0	0	0	0
0	0	0	0	0	0	0	0	0	0	0	0	0	0	0	0	0	0	0
0	0	0	0	0	0	0	0	0	0	0	0	0	0	0	0	0	0	0
0	0	0	0	0	0	0	0	0	0	0	0	0	0	0	0	0	0	0
0	0	0	0	0	0	0	0	0	0	0	0	0	0	0	0	0	0	0
0	0	0	0	0	0	0	0	0	0	0	0	0	0	0	0	0	0	0
0	0	0	0	0	0	0	0	0	0	0	0	0	0	0	0	0	0	0
0	0	0	0	0	0	0	0	0	0	0	0	0	0	0	0	0	0	0
0	0	0	0	0	0	0	0	0	0	0	0	0	0	0	0	0	0	0
0	0	0	0	0	0	0	0	0	0	0	0	0	0	0	0	0	0	0
0	0	0	0	0	0	0	6	6	6	0	0	0	0	0	0	0	0	0
0	0	0	0	0	0	0	0	0	0	0	0	0	0	0	0	0	0	0
0	0	0	0	0	0	0	0	0	0	0	0	0	0	0	0	0	0	0
0	0	0	0	0	0	0	0	0	0	0	0	0	0	0	0	0	0	0
0	0	0	0	0	0	0	0	0	0	0	0	0	0	0	0	0	0	0
0	0	0	0	0	0	0	0	0	0	0	0	0	0	0	0	0	0	0
0	0	0	0	0	0	0	0	0	0	0	0	0	0	0	0	0	0	0
0	0	0	0	0	0	0	0	0	0	0	0	0	0	0	0	0	0	0
0	0	0	0	0	0	0	0	0	0	0	0	0	0	0	0	0	0	0
5	0	0	0	0	0	0	0	0	0	0	0	0	0	0	0	0	0	0
0	0	0	0	0	0	0	0	0	0	0	0	0	0	0	0	0	0	0
0	0	0	0	0	0	0	0	0	0	0	0	0	0	0	0	0	0	0
0	0	0	0	0	0	0	0	0	0	0	0	0	0	0	0	0	0	0
0	0	0	0	0	0	0	0	0	0	0	0	0	0	0	0	0	0	0
0	0	0	0	0	0	0	0	0	0	0	0	0	0	0	0	0	0	0
0	0	0	0	0	0	0	0	0	0	0	0	0	0	0	0	0	0	0
0	0	0	0	0	0	0	0	0	0	0	0	0	0	0	0	0	0	0
0	0	0	0	0	0	0	0	0	0	0	0	0	0	0	0	0	0	0
0	0	0	0	0	0	0	0	0	0	0	0	0	0	0	0	0	0	0
0	0	0	0	0	0	0	0	0	0	0	0	4	0	0	0	0	0	0
0	0	0	0	0	0	0	0	0	0	0	0	0	0	0	0	0	0	0
0	0	0	0	0	0	0	0	0	0	0	0	0	0	0	0	0	0	0
0	0	0	0	0	0	0	0	0	0	0	0	0	0	0	0	0	0	0

0	0	0	0	0	0	0	0	0	0	0	0	0	0	0	0	0	0	0
0	0	0	0	0	0	0	0	0	0	0	0	0	0	0	0	0	0	0
0	0	0	0	0	0	0	0	0	0	0	0	0	0	0	0	0	0	0
0	0	0	0	0	0	0	0	0	0	0	0	0	0	0	0	0	0	0
0	0	0	0	0	0	0	0	0	0	0	0	0	0	0	0	0	0	0
0	0	0	0	0	0	0	0	0	0	0	0	0	0	0	0	0	0	0
0	0	0	0	0	0	0	0	0	0	0	0	0	0	0	0	0	0	0
0	0	0	0	0	0	0	0	0	0	0	0	0	0	0	0	0	0	0
0	0	0	0	0	0	0	0	0	0	0	0	0	0	0	0	0	0	0
0	0	0	0	0	0	0	0	0	0	0	0	0	0	0	0	0	0	0
0	0	0	0	0	0	0	0	0	0	0	0	0	0	0	0	0	0	0
0	0	0	0	0	0	0	0	0	0	0	0	0	0	0	0	0	0	0
0	0	0	0	0	0	0	0	0	0	0	0	0	0	0	0	0	0	0
0	0	0	0	0	0	0	0	0	0	0	0	0	0	0	0	0	0	0
0	0	0	0	0	0	0	0	0	0	0	0	0	0	0	0	0	0	0
0	0	0	0	0	0	0	0	0	0	0	0	0	0	0	0	0	0	0
0	0	0	0	0	0	0	0	0	0	0	0	0	0	0	0	0	0	0
0	0	0	0	0	0	0	0	0	0	0	0	0	0	0	0	0	0	0
0	0	0	0	0	0	0	0	0	0	0	0	0	0	0	0	0	0	0
0	0	0	0	0	0	0	0	0	0	0	0	0	0	0	0	0	0	0
0	0	0	0	0	0	0	0	0	0	0	0	0	0	0	0	0	0	0
0	0	0	0	0	0	0	0	0	0	0	0	0	0	0	0	0	0	0
0	0	0	0	0	0	0	0	0	0	0	0	0	0	0	0	0	0	0
0	0	0	0	0	0	0	0	0	0	0	0	0	0	0	0	0	0	0
0	0	0	0	0	0	0	0	0	0	0	0	0	0	0	0	0	0	0
0	0	0	0	0	0	0	0	0	0	0	0	0	0	0	0	0	0	0
0	0	0	0	0	0	0	0	0	0	0	0	0	0	0	0	0	0	0
0	0	0	0	0	0	0	0	0	0	0	0	0	0	0	0	0	0	0
0	0	0	0	0	0	0	0	0	0	0	0	0	0	0	0	0	0	0
0	0	0	0	0	0	0	0	0	0	0	0	0	0	0	0	0	0	0
0	0	0	0	0	0	0	0	0	0	0	0	0	0	0	0	0	0	0
0	0	0	0	0	0	0	0	0	0	0	0	0	0	0	0	0	0	0
0	0	0	0	0	0	0	0	0	0	0	0	0	0	0	0	0	0	0
0	0	0	0	0	0	0	0	0	0	0	0	0	0	0	0	0	0	0
0	0	0	0	0	0	0	0	0	0	0	0	0	0	0	0	0	0	0
0	0	0	0	0	0	0	0	0	0	0	0	0	0	0	0	0	0	0
0	0	0	0	0	0	0	0	0	0	0	0	0	0	0	0	0	0	0
0	0	0	0	0	0	0	0	0	0	0	0	0	0	0	0	0	0	0

0	0	0	0	0	0	0	0	0	0	0	0	0	0	0	0	0	0	0
0	0	0	0	0	0	0	0	0	0	0	0	0	0	0	0	0	0	0
0	0	0	0	0	0	0	0	0	0	0	0	0	0	0	0	0	0	0
0	0	0	0	0	0	0	0	0	0	0	0	0	0	0	0	0	0	2
2	0	0	0	0	0	0	0	0	0	0	0	0	0	0	0	0	0	0
0	0	0	0	0	0	0	0	0	0	0	0	0	0	0	0	0	0	0
0	0	0	0	0	0	0	0	0	0	0	0	0	0	0	0	0	0	0
0	0	0	0	0	0	0	0	0	0	0	0	0	0	0	0	0	0	0
0	0	0	0	0	0	0	0	0	0	0	0	0	0	0	0	0	0	0
0	0	0	0	0	0	0	0	0	0	0	0	0	0	0	0	0	0	0
0	0	0	0	0	0	0	0	0	0	0	0	0	0	0	0	0	0	0
0	0	0	0	0	0	0	0	0	0	0	0	0	0	0	0	0	0	0
0	0	0	0	0	0	0	0	0	0	0	0	0	0	0	0	0	0	0
0	0	0	0	0	0	0	0	0	0	0	0	0	0	0	0	0	0	0
0	0	0	0	0	0	0	0	0	0	0	0	0	0	0	0	0	0	0
0	0	0	0	0	0	0	0	0	0	0	0	0	0	0	0	0	0	0
0	0	0	0	0	0	0	0	0	0	0	0	0	0	0	0	0	0	0
0	0	0	0	0	0	0	0	0	0	0	0	0	0	0	0	0	0	0
0	0	0	0	0	0	0	0	0	0	0	0	0	0	0	0	0	0	0
0	0	0	0	0	0	0	0	0	0	0	0	0	0	0	0	0	0	0
0	0	0	0	0	0	0	0	0	0									
Jan_DL049	1446	1438	31	732	55	510	832	810	341	70	154	295	80	389	180	64	328	150
216	66	182	313	316	137	98	149	155	98	122	0	77	115	50	71	115	132	0
86	38	54	171	137	66	120	0	65	18	189	0	43	85	135	0	0	126	0
0	0	0	17	122	152	97	66	26	143	35	0	42	127	44	58	46	53	0
27	0	56	0	11	0	42	29	0	0	0	100	14	0	33	0	44	11	0
0	44	0	53	0	32	13	29	26	15	0	0	40	13	25	0	0	0	0
0	0	0	10	0	0	0	0	0	0	0	0	9	8	0	0	0	0	0
0	10	106	12	0	0	0	3	0	0	23	0	0	0	0	32	0	0	0
0	0	0	14	0	0	0	0	0	0	4	0	15	57	0	0	11	0	0
0	0	13	45	0	87	0	24	0	0	0	5	0	16	0	0	0	0	0
0	0	13	0	0	0	0	0	0	5	0	0	0	0	17	0	0	0	0
17	17	0	0	0	0	0	0	0	10	0	0	0	0	0	0	0	0	33
0	0	0	0	0	7	0	0	0	0	0	0	34	7	17	0	0	0	0
0	0	0	38	0	0	0	0	0	0	0	0	17	10	7	3	0	0	0
0	0	0	0	0	2	0	0	0	69	0	14	0	0	0	0	0	0	0
0	0	14	43	0	0	0	20	5	0	0	0	10	0	0	0	0	0	0

0	0	18	0	0	44	0	0	0	0	0	0	0	0	23	0	0	0	12
0	0	0	43	0	0	0	8	0	5	0	0	0	0	0	0	0	0	0
0	3	0	0	6	0	0	0	0	0	0	9	0	0	0	0	6	0	0
0	0	0	0	0	0	0	0	0	9	0	0	0	0	0	0	0	0	4
0	0	0	0	0	0	0	0	0	0	0	0	0	0	0	0	0	0	0
0	0	0	0	0	0	0	8	0	0	0	0	5	0	0	32	0	0	0
0	0	0	0	0	0	0	0	0	0	0	0	0	0	12	0	13	0	0
0	0	0	0	0	0	0	0	0	0	0	0	0	0	0	0	0	0	0
0	0	0	0	0	0	0	0	0	0	0	0	0	0	0	0	0	0	0
0	0	0	0	7	0	23	4	0	0	0	0	0	0	0	0	0	0	0
0	0	0	0	0	0	0	0	0	0	0	0	0	0	0	0	0	4	6
0	44	0	0	0	0	0	0	0	13	0	0	0	0	0	0	0	0	0
0	0	0	0	0	11	0	0	0	0	0	0	0	0	0	0	0	0	0
0	0	0	0	0	0	0	0	0	0	4	0	0	0	0	0	0	0	0
0	4	0	0	0	0	0	0	0	0	0	0	0	0	0	0	0	0	0
0	0	0	0	0	0	0	0	0	0	0	0	0	0	0	0	0	0	0
0	0	0	0	0	0	15	0	0	0	0	0	0	0	0	0	0	0	0
0	0	0	0	0	0	0	0	0	0	0	0	0	0	0	0	0	0	0
0	0	0	0	0	0	0	0	0	0	0	0	0	0	0	0	0	0	0
0	0	0	0	0	0	0	0	0	0	0	0	0	0	0	0	0	0	0
0	0	0	0	0	0	0	0	0	0	0	0	0	0	0	0	0	0	0
0	0	0	0	0	0	0	0	0	0	0	0	0	0	0	0	0	0	0
0	0	0	0	0	0	0	0	0	0	0	0	0	0	0	0	0	0	0
0	0	0	0	0	0	0	0	0	0	0	0	0	0	0	0	0	0	0
0	0	0	0	0	0	0	0	0	0	0	0	0	0	0	0	0	0	0
0	0	0	0	0	0	0	0	0	0	0	0	0	0	0	0	0	0	0
0	0	0	0	0	0	0	0	0	0	0	0	0	0	5	0	0	0	0
0	0	0	0	0	0	0	0	0	0	0	0	0	0	0	0	0	0	0
0	0	0	0	0	0	0	0	0	0	0	0	0	0	0	0	10	0	0
0	0	0	0	0	0	0	0	0	0	0	0	0	0	0	0	0	0	0
0	0	5	0	0	0	0	0	0	0	0	0	0	0	0	0	0	0	0
0	0	0	0	5	0	0	0	0	0	0	0	0	0	0	0	0	0	0
0	0	0	0	0	0	0	0	0	0	0	0	0	0	0	0	0	0	0
0	0	0	0	0	0	0	0	0	0	0	0	0	0	0	0	0	0	0
0	0	0	0	0	0	0	0	0	0	0	0	0	0	0	0	0	0	0
0	0	0	0	0	0	0	0	0	0	0	0	0	0	0	0	0	0	0
0	0	0	0	0	0	0	0	0	0	0	0	0	0	0	0	0	0	0

0	0	0	0	0	0	0	0	0	0	0	0	0	0	0	0	0	0	0
0	0	0	0	0	0	0	0	0	0	0	0	0	0	0	0	0	0	0
0	0	0	6	0	0	0	0	0	0	0	0	0	0	0	0	17	0	0
0	0	0	0	0	0	0	0	0	0	0	0	0	0	0	0	0	0	0
0	0	0	0	0	0	0	0	0	0	0	0	0	0	0	0	0	0	0
0	0	0	0	0	0	0	0	0	0	0	0	0	0	0	0	0	0	0
0	0	0	0	0	0	0	0	0	0	0	0	0	0	0	0	0	0	0
0	0	0	0	0	0	0	0	0	0	0	5	0	0	0	0	0	0	0
0	0	0	0	0	0	0	0	0	0	0	0	0	0	0	0	0	0	0
0	0	0	0	0	0	0	0	0	0	0	0	0	0	0	0	0	0	0
0	0	0	0	0	0	0	0	0	0	0	0	0	0	0	0	0	0	0
0	0	0	0	0	0	0	0	0	0	0	0	0	0	0	0	0	0	0
0	0	0	0	0	0	0	0	0	0	0	0	0	0	0	0	0	0	0
0	0	0	0	0	0	0	0	0	0	0	0	0	0	0	0	0	0	0
0	0	0	0	0	0	0	0	0	0	0	0	0	0	0	0	0	0	0
0	0	0	0	0	0	0	0	0	0	0	0	0	0	0	0	0	0	0
0	0	0	0	0	0	0	0	0	0	0	0	0	0	0	0	0	0	0
0	0	0	0	0	0	0	0	0	0	0	0	0	0	0	0	0	0	0
0	0	0	0	0	0	0	0	0	0	0	0	0	0	0	0	0	0	0
0	0	0	0	0	0	0	0	0	0	0	0	0	0	0	0	0	0	0
0	0	0	0	0	0	0	0	0	0	0	0	0	0	0	0	0	0	0
0	0	0	0	0	0	0	0	0	0	0	0	0	0	0	0	0	0	0
0	0	0	0	0	0	0	0	0	0	0	0	0	0	0	0	0	0	0
0	0	0	0	0	0	0	0	0	0	0	0	0	0	0	0	0	0	0
9	0	0	0	0	0	0	0	0	0	0	0	0	0	0	0	0	0	0
0	0	0	0	0	0	0	0	0	0	0	0	0	0	0	0	0	0	0
0	0	0	0	0	0	0	0	0	0	0	0	0	0	0	0	0	0	0
0	0	0	0	0	0	0	0	0	0	0	0	0	0	0	8	0	0	0
0	0	0	0	0	0	0	0	0	0	0	0	0	0	0	0	0	0	0
0	0	0	0	0	0	0	0	0	0	0	0	0	0	0	0	0	0	0
0	0	0	0	0	0	0	0	0	0	0	0	0	0	0	0	0	0	0
0	0	0	0	0	0	0	0	0	0	0	0	0	0	0	0	0	0	0
0	0	0	0	0	0	0	0	0	0	0	0	0	0	0	0	0	0	0
0	0	0	0	0	0	0	0	0	0	0	0	0	0	0	0	0	0	0
0	0	0	0	0	0	0	0	0	0	0	0	0	0	0	0	0	0	0
0	0	0	0	0	0	0	0	0	0	0	0	0	0	0	0	0	0	0
0	0	0	0	0	0	0	0	0	0	0	0	0	0	0	0	0	0	0

0	0	0	0	0	0	0	0	0	0	0	0	0	0	0	0	0	0	0
0	0	0	0	0	0	0	0	0	0	0	0	0	0	0	0	0	0	0
0	0	0	0	0	0	0	0	0	0	0	0	0	0	0	0	0	0	0
0	0	0	0	0	0	0	0	0	0	0	0	0	0	0	0	0	0	0
0	0	0	0	0	0	0	0	0	0	0	0	0	0	0	0	0	0	0
0	0	0	0	0	0	0	0	0	0	0	0	0	0	0	0	0	0	0
0	0	0	0	0	0	0	0	0	0	0	0	0	0	0	0	0	0	0
0	0	0	0	0	0	0	0	0	0	0	0	0	0	0	0	0	0	0
0	0	0	0	0	0	0	0	0	0	0	0	0	0	0	0	0	0	0
0	0	0	0	0	0	0	0	0	0	0	0	5	5	5	0	0	0	0
0	0	0	0	0	0	0	0	0	0	0	0	0	0	0	0	0	0	0
0	0	0	0	0	0	0	0	0	0	0	0	0	0	0	0	0	0	0
0	0	0	0	0	0	0	0	0	0	0	0	0	0	0	0	0	0	0
0	0	0	0	0	0	0	0	0	0	0	0	0	0	0	0	0	0	0
0	0	0	0	0	0	0	0	0	0	0	0	0	0	0	0	0	0	0
0	0	0	0	0	0	0	0	0	0	0	0	0	0	0	0	0	0	0
0	0	0	0	0	0	0	0	0	0	0	0	0	0	0	0	0	0	0
0	0	0	0	0	0	0	0	0	0	0	0	0	0	0	0	0	0	0
0	0	0	0	0	0	0	0	0	0	0	0	0	0	0	0	0	0	0
0	0	0	0	0	0	0	0	0	0	0	0	0	0	0	0	0	0	0
0	0	4	0	0	0	0	0	0	0	0	0	0	0	0	0	0	0	0
0	0	0	0	0	0	0	0	0	0	0	0	0	0	0	0	0	0	0
0	0	0	0	0	0	0	0	0	0	0	0	0	0	0	0	0	0	0
0	0	0	0	0	0	0	0	0	0	0	0	0	0	0	0	0	0	0
0	0	0	0	0	0	0	0	0	0	0	0	0	0	0	0	0	0	0
0	0	0	0	0	0	0	0	0	0	0	0	0	0	0	0	0	0	0
0	0	0	0	0	0	0	0	0	0	0	0	0	0	0	0	0	0	0
0	0	0	0	0	0	0	0	0	0	0	0	0	0	0	0	0	0	0
0	0	0	0	0	0	0	0	0	0	0	0	0	0	0	0	0	0	0
0	0	0	0	0	0	0	0	0	0	0	0	0	0	0	0	0	0	0
0	0	0	0	0	0	0	0	0	0	0	0	0	0	0	0	0	0	0
0	0	0	0	0	0	0	0	0	0	0	0	0	0	0	0	0	0	0
0	0	0	0	0	0	0	0	0	0	0	0	0	0	0	0	0	0	0
0	0	0	0	0	0	0	0	0	0	0	0	0	0	0	0	0	0	0
0	0	0	0	0	0	0	0	0	0	0	0	0	0	0	0	0	0	0
0	0	0	0	0	0	0	0	0	0	0	0	0	0	0	0	0	0	0
0	0	0	0	3	3	3	3	3	0	0	0	0	0	0	0	0	0	0

0	0	0	0	0	0	0	0	0	0	0	0	0	0	0	0	0	0	0
0	0	0	0	0	0	0	0	0	0	0	0	0	0	0	0	0	0	0
0	0	0	0	0	0	0	0	0	0	0	0	0	0	0	0	0	0	0
0	0	0	0	0	0	0	0	0	0	0	0	0	0	0	0	0	0	0
0	0	0	0	0	0	0	0	0	0	0	0	0	0	0	0	0	0	0
0	0	0	0	0	0	0	0	0	0	0	0	0	0	0	0	0	0	0
0	0	0	0	0	0	0	0	0	0	0	0	0	0	0	0	0	0	0
0	0	0	0	0	0	0	0	0	0	0	0	0	0	0	0	0	0	0
0	0	0	0	0	0	0	0	0	0	0	0	0	0	0	0	0	0	0
0	0	0	0	0	0	0	0	0	0	0	0	0	0	0	0	0	0	0
0	0	0	0	0	0	0	0	0	0	0	0	0	0	0	0	0	0	0
0	0	0	0	0	0	0	0	0	0	0	0	0	0	0	0	0	0	0
0	0	0	0	0	0	0	0	0	0	0	0	0	0	0	0	0	0	0
0	0	0	0	0	0	0	0	0	0	0	0	0	0	0	0	0	0	0
0	0	0	0	0	0	0	0	0	0	0	0	0	0	0	0	0	0	0
0	0	0	0	0	0	0	0	0	0	0	0	0	0	0	0	0	0	0
0	0	0	0	0	0	0	0	0	0	0	0	0	0	0	0	0	0	0
0	0	0	0	0	0	0	0	0	0	0	0	0	0	0	0	0	0	0
0	0	0	0	0	0	0	0	0	0	0	0	0	0	0	0	0	0	0
0	0	0	0	0	0	0	0	0	0	0	0	0	0	0	0	0	0	0
0	0	0	0	0	0	0	0	0	0	0	0	0	0	0	0	0	0	0
0	0	0	0	0	0	0	0	0	0	0	0	0	0	0	0	0	0	0
0	0	0	0	0	0	0	0	0	0	0	0	0	0	0	0	0	0	0
0	0	0	0	0	0	0	0	0	0	0	0	0	0	0	0	0	0	0
0	0	0	0	0	0	0	0	0	0	0	0	0	0	0	0	0	0	0
0	0	0	0	0	0	0	0	0	0	0	2	2	2	0	0	0	0	0
0	0	0	0	0	0	0	0	0	0	0	0	0	0	0	0	0	0	0
0	0	0	0	0	0	0	0	0	0	0	0	0	0	0	0	0	0	0
0	0	0	0	0	0	0	0	0	0	0	0	0	0	0	0	0	0	0
0	0	0	0	0	0	0	0	0	0	0	0	0	0	0	0	0	0	0
0	0	0	0	0	0	0	0	0	0	0	0	0	0	0	0	0	0	0
0	0	0	0	0	0	0	0	0	0	0	0	0	0	0	0	0	0	0
0	0	0	0	0	0	0	0	0	0	0	0	0	0	0	0	0	0	0
0	0	0	0	0	0	0	0	0	0	0	0	0	0	0	0	0	0	0
0	0	0	0	0	0	0	0	0	0	0	0	0	0	0	0	0	0	0
0	0	0	0	0	0	0	0	0	0	0	0	0	0	0	0	0	0	0
0	0	0	0	0	0	0	0	0	0	0	0	0	0	0	0	0	0	0

0	0	0	0	0	0	0	0	0	0	0	0	0	0	0	0	0	0	0
0	0	0	0	0	0	0	0	0	0	0	0	0	0	0	0	0	0	0
0	0	0	0	0	0	0	0	0	0	0	0	0	0	0	0	0	0	0
0	0	0	0	0	0	0	0	0	0	0	0	0	0	0	0	0	0	0
0	0	0	0	0	0	0	0	0	0	0	0	0	0	0	0	0	0	0
0	0	0	0	0	0	0	0	0	0	0	0	0	0	0	0	0	0	0
0	0	0	0	0	0	0	0	0	0	0	0	0	0	0	0	0	0	0
0	0	0	0	0	0	0	0	0	0	0	0	0	0	0	0	0	0	0
0	0	0	0	0	0	0	0	0	0	0	0	0	0	0	0	0	0	0
0	0	0	0	0	0	0	0	0	0	0	0	0	0	0	0	0	0	0
0	0	0	0	0	0	0	0	0	0	0	0	0	0	0	0	0	0	0
0	0	0	0	0	0	0	0	0	0	0	0	0	0	0	0	0	0	0
0	0	0	0	0	0	0	0	0	0									
Jan_DL005	1479	2884	794	476	160	119	63	62	329	685	618	165	490	53	361	503	59	178
131	32	425	638	142	292	450	161	273	80	76	0	163	333	223	325	118	0	0
67	304	0	48	211	286	93	427	38	211	24	217	144	0	184	249	105	36	0
0	25	234	72	0	115	51	62	265	49	117	0	0	69	30	10	36	117	228
145	173	94	25	0	129	28	80	89	188	65	62	71	146	58	0	120	61	85
13	48	0	0	155	11	46	149	0	80	0	18	64	0	23	36	30	0	124
0	0	0	27	9	28	0	0	0	0	45	0	0	6	0	0	132	68	0
0	0	0	72	0	100	0	0	0	0	0	0	96	0	0	13	0	674	0
0	31	0	265	0	4	0	67	0	91	26	0	16	0	130	31	24	31	0
0	0	39	0	117	0	73	0	0	0	18	34	0	0	0	0	0	52	78
214	44	21	46	68	0	0	19	0	0	0	35	0	103	0	65	0	35	0
16	0	49	0	15	0	0	0	0	60	0	0	20	55	0	0	0	36	65
0	0	87	18	0	0	0	0	23	6	0	8	0	24	74	0	0	23	0
0	0	0	33	93	0	0	0	0	12	0	0	533	36	12	0	0	32	0
12	0	0	0	0	8	0	0	9	0	5	23	0	0	3	25	19	0	0
0	0	46	7	0	0	0	0	11	0	0	22	21	67	0	0	0	0	0
0	22	14	0	0	0	0	54	0	0	15	0	38	0	32	12	0	0	0
38	0	7	0	19	0	0	0	0	0	0	105	0	0	0	0	0	0	0
9	0	41	34	19	7	0	27	15	0	0	0	34	0	41	15	37	0	9
0	0	3	0	8	0	0	0	0	0	0	60	11	10	0	0	0	0	0
0	29	0	0	0	0	27	5	57	0	0	28	10	0	12	0	0	18	0
0	0	0	36	0	27	0	0	6	0	0	10	0	8	0	0	0	29	0
0	0	0	0	0	0	0	0	0	0	0	0	54	0	8	0	0	0	0
0	0	0	0	0	0	35	0	0	0	0	0	0	0	0	0	0	0	0

0	0	13	0	13	0	0	0	0	0	0	0	0	0	0	0	0	0	42
0	0	0	14	10	0	0	7	0	0	0	0	0	0	0	12	0	0	0
0	0	9	0	0	0	0	0	0	0	0	0	0	0	0	0	7	0	0
0	0	0	25	0	14	0	0	0	0	0	0	0	0	0	16	0	0	0
0	0	0	0	0	0	0	0	0	0	0	0	11	0	0	0	0	0	0
0	0	13	0	0	0	0	0	39	0	0	0	0	0	0	0	0	0	0
0	11	0	0	0	0	7	0	0	0	0	0	0	10	0	0	0	0	0
0	0	5	0	0	2	0	0	0	0	0	0	0	7	0	0	0	0	0
0	0	0	0	0	0	0	0	0	0	0	28	0	0	0	0	0	8	0
7	0	0	0	0	0	14	0	29	0	0	0	0	0	0	0	0	0	0
0	0	0	0	0	0	0	0	0	0	0	0	0	0	0	0	10	0	0
0	0	0	0	0	0	18	0	0	0	0	0	0	0	0	0	0	26	0
0	0	60	0	0	0	0	0	0	0	0	0	19	0	0	0	0	0	0
0	3	0	8	0	0	0	0	0	0	0	0	0	0	0	0	0	0	0
0	0	0	0	0	0	0	0	0	0	0	0	2	0	0	0	0	0	0
9	0	0	0	0	109	8	0	0	0	0	0	0	0	0	0	0	0	0
0	0	0	0	0	0	0	0	0	4	0	0	0	0	0	0	0	0	0
0	0	0	0	0	0	0	0	0	0	0	0	0	0	0	0	0	0	0
0	0	0	0	0	0	0	0	0	0	0	0	12	0	0	0	0	0	0
0	0	0	0	0	0	0	0	0	0	0	0	0	6	0	10	0	0	18
0	0	0	0	0	0	0	0	0	0	0	0	16	0	0	0	0	0	0
0	0	0	0	0	0	8	12	0	0	0	0	0	0	0	0	0	0	0
5	0	0	3	0	0	0	0	0	0	0	10	0	0	3	0	15	0	0
0	0	0	0	0	0	0	0	0	0	17	0	0	0	0	0	0	0	0
0	0	0	0	0	0	0	0	0	0	12	0	0	16	0	0	0	0	0
0	16	0	0	0	0	0	0	0	0	0	0	0	0	0	7	0	0	0
0	0	0	0	0	0	0	0	0	0	0	0	2	0	0	0	0	0	0
0	0	0	0	0	0	0	9	0	0	0	0	0	0	0	0	0	0	0
0	0	0	0	0	0	0	0	0	0	0	0	0	0	0	0	0	0	0
0	0	0	0	0	0	0	0	50	0	0	0	0	0	0	0	0	0	4
0	0	0	0	0	0	0	0	0	0	0	0	0	0	0	0	0	0	0
0	0	0	0	0	0	0	0	0	0	0	0	0	0	0	0	0	0	0
0	0	0	0	0	0	0	0	0	0	0	0	0	0	0	0	0	0	0
0	0	0	0	0	0	0	0	0	0	0	0	0	0	0	0	0	0	0
0	0	17	0	0	0	0	0	0	0	0	0	0	0	0	0	0	0	0
0	0	0	0	0	0	0	0	0	0	0	0	0	0	0	0	0	0	0
0	0	0	0	0	0	0	0	0	0	0	0	0	0	0	0	0	0	0

0	0	0	0	0	0	0	0	0	0	0	0	0	5	0	0	0	0	0
0	0	0	0	0	0	0	0	0	0	0	0	0	0	0	0	0	0	0
0	0	0	0	0	0	0	0	0	15	0	8	0	0	0	0	0	0	0
0	0	0	0	0	0	0	0	0	0	0	0	0	0	0	0	0	0	0
0	0	0	0	0	0	0	0	0	0	0	0	6	0	0	0	0	0	0
0	0	0	0	0	0	0	0	0	0	0	13	0	0	0	0	0	0	0
0	0	0	0	0	0	0	0	0	0	0	0	0	0	0	0	0	0	2
0	0	0	0	0	0	0	0	0	0	0	0	0	0	0	0	0	0	0
0	0	0	0	0	0	0	0	0	0	0	0	0	0	0	0	0	0	0
0	0	0	0	0	0	0	0	0	0	0	0	0	0	0	0	0	0	0
0	0	0	0	0	0	0	0	0	0	0	0	0	0	0	0	0	0	0
0	0	0	0	0	0	0	0	0	0	0	0	0	0	0	0	0	0	0
0	0	0	0	0	0	0	0	0	0	0	0	0	0	0	0	0	0	0
0	0	0	0	0	0	0	0	0	0	0	0	0	0	0	0	0	0	0
0	0	0	0	0	0	0	0	0	0	0	0	0	0	0	0	0	0	0
0	0	0	0	0	0	0	0	0	0	0	0	0	0	0	3	0	0	0
0	0	0	0	0	1	0	0	0	0	0	0	0	0	0	0	0	0	0
0	0	0	0	0	0	0	0	0	0	0	0	0	0	0	0	0	0	0
0	0	0	0	0	0	0	0	0	0	0	0	0	0	0	0	0	0	0
0	0	0	0	0	0	0	5	0	0	0	0	0	0	0	0	0	0	0
0	0	0	0	0	0	0	0	0	0	0	0	0	0	0	0	0	0	0
0	0	0	0	0	0	0	0	0	0	0	0	0	0	0	0	0	0	0
0	0	4	0	0	0	0	0	0	0	0	0	0	0	0	0	0	0	0
0	0	0	0	0	0	0	0	0	0	0	0	0	13	0	0	0	0	0
0	0	0	0	0	0	0	0	0	0	0	0	0	0	0	0	0	0	0
0	0	0	0	0	0	0	0	0	0	0	0	0	0	0	0	0	0	0
0	0	0	0	0	0	0	0	0	0	0	0	12	0	0	0	0	0	0
0	0	0	0	0	0	0	0	0	0	0	0	0	0	0	0	0	0	0
0	0	0	0	0	0	0	0	0	0	0	0	0	0	0	0	0	0	0
0	0	0	0	0	0	0	0	0	0	0	0	0	0	0	0	0	0	0
0	0	0	0	0	0	0	0	0	0	0	0	0	0	0	0	0	0	0
0	0	0	0	0	0	0	0	0	0	0	0	0	0	0	0	0	0	0
0	0	0	0	0	0	0	0	0	0	0	0	0	0	0	0	0	0	0
0	0	0	0	0	0	0	0	0	0	0	0	0	0	0	0	0	0	0
0	0	0	0	0	0	0	0	0	0	0	0	0	0	0	0	0	0	0
0	0	0	0	0	0	0	0	0	0	0	0	0	0	0	0	0	0	0
0	0	0	0	0	0	0	0	0	0	0	0	0	0	0	0	0	0	0

0	0	0	0	0	0	0	0	0	0	0	0	0	0	0	0	0	0	0
0	0	0	0	0	0	0	0	0	0	0	0	0	0	0	0	0	0	0
0	0	0	0	0	0	0	0	0	0	0	0	0	0	0	0	0	0	0
0	0	0	0	0	0	0	0	0	0	0	0	0	0	0	0	0	0	0
0	0	0	0	0	0	0	0	0	0	0	0	0	0	0	0	0	0	0
0	0	0	0	0	0	0	0	0	0	0	0	0	0	0	0	0	0	0
0	0	0	0	0	0	0	0	0	0	0	0	0	0	0	0	0	0	0
0	0	0	0	0	0	0	0	0	0	0	0	0	0	0	0	0	0	0
0	0	0	0	0	0	0	0	0	0	0	0	0	0	0	0	0	0	0
0	0	0	0	0	0	0	0	0	0	0	0	0	0	0	0	0	0	0
0	0	0	0	0	0	0	0	0	0	0	0	0	0	0	0	0	0	0
0	0	0	0	0	0	0	0	0	0	0	0	0	0	0	0	0	0	0
0	0	0	0	0	0	0	0	0	0	0	0	0	0	0	0	0	0	0
0	0	0	0	0	0	0	0	0	0	0	0	0	0	0	0	0	0	0
0	0	0	0	0	0	0	0	0	0	0	0	0	0	0	0	0	0	0
0	0	0	0	0	0	0	0	0	0	0	0	0	0	0	0	0	0	0
0	0	0	0	0	0	0	0	0	0	0	0	0	0	0	0	0	0	0
0	0	0	0	0	0	0	0	0	0	0	0	0	0	0	0	0	0	0
0	0	0	0	0	0	0	0	0	0	0	0	0	0	0	0	0	0	0
0	0	0	0	0	0	0	0	0	0	0	0	0	0	0	0	0	0	0
0	0	0	0	0	0	0	0	0	0	0	0	0	0	0	6	0	0	0
0	0	0	0	0	0	0	0	0	0	0	0	0	0	0	0	0	0	0
0	0	0	0	0	0	0	0	0	0	0	0	0	0	0	0	0	0	0
0	0	0	0	0	0	0	0	0	0	0	0	0	0	0	0	0	0	0
0	0	0	0	0	0	0	0	0	0	0	0	0	0	0	0	0	0	0
0	0	0	0	0	0	0	0	0	0	0	0	0	0	0	0	0	0	0
0	0	0	0	0	0	0	0	0	0	0	0	0	0	0	0	0	0	0
0	0	0	0	0	0	0	0	0	0	0	0	0	0	0	0	0	0	0
0	0	0	0	0	0	0	0	0	0	0	0	0	0	0	0	0	0	0
0	0	0	0	0	0	0	0	0	0	0	0	0	0	0	0	0	0	0
0	0	0	0	0	0	0	0	0	0	0	0	0	0	0	0	0	0	0
0	0	0	0	0	0	0	0	0	0	0	0	0	0	0	0	0	0	0
0	0	0	0	0	0	0	0	0	0	0	0	0	0	0	0	0	0	0
0	0	0	0	0	0	0	0	0	0	0	0	0	0	0	0	0	0	0
0	0	0	0	0	0	0	0	0	0	0	0	0	0	0	0	0	0	0
0	0	0	0	0	0	0	0	0	0	0	0	0	0	0	0	0	0	0
0	0	0	0	0	0	0	0	0	0	0	0	0	0	0	0	0	0	0

0	0	0	0	0	0	0	0	0	0	0	0	0	0	0	0	0	0	0
0	0	0	0	0	0	0	0	0	0	0	0	0	0	0	0	0	0	0
0	0	0	0	0	0	0	0	0	0	0	0	0	0	0	0	0	0	0
0	0	0	0	0	0	0	0	0	0	0	0	0	0	0	0	0	0	0
0	0	0	0	0	0	0	0	0	0	0	0	0	0	0	0	0	0	0
0	0	0	0	0	0	0	0	0	0	0	0	0	0	0	0	0	0	0
0	0	0	0	0	0	0	0	0	0	0	0	0	0	0	0	0	0	0
0	0	0	0	0	0	0	0	0	0	0	0	0	0	0	0	0	0	0
0	0	0	0	0	0	0	0	0	0	0	0	0	0	0	0	0	0	0
0	0	0	0	0	0	0	0	0	0	0	4	4	4	0	0	0	0	0
0	0	0	0	0	0	0	0	0	0	0	0	0	0	0	0	0	0	0
0	0	0	0	0	0	0	0	0	0	0	0	0	0	0	0	0	0	0
0	0	0	0	0	0	0	0	0	0	0	0	0	0	0	0	0	0	0
0	0	0	0	0	0	0	0	0	0	0	0	0	0	0	0	0	0	0
0	0	0	0	0	0	0	0	0	0	0	0	0	0	0	0	0	0	0
0	0	0	0	0	0	0	0	0	0	0	0	0	0	0	0	0	0	0
0	0	0	0	0	0	0	0	0	0	0	0	0	0	0	0	0	0	0
0	0	0	0	0	0	0	0	0	0	0	0	0	0	0	0	0	0	0
0	0	0	0	0	0	0	0	0	0	0	0	0	0	0	0	0	0	0
0	0	0	0	0	0	0	0	0	0	0	0	0	0	0	0	0	0	0
0	0	0	0	0	0	0	0	0	0	0	0	0	0	0	0	0	0	0
0	0	0	0	0	0	0	0	0	0	0	0	0	0	0	0	0	0	0
0	0	0	0	0	0	0	0	0	0	0	0	0	0	0	0	0	0	0
0	0	0	0	0	0	0	0	0	0	0	0	0	0	0	0	0	0	0
0	0	0	0	0	0	0	0	0	0	0	0	0	0	0	0	0	0	0
0	0	0	0	0	0	0	0	0	0	0	0	0	0	0	0	0	0	0
0	0	0	0	0	0	0	0	0	0	0	0	0	0	0	0	0	0	0
0	0	0	0	0	0	0	0	0	3	3	3	3	0	0	0	0	0	0
0	0	0	0	0	0	0	0	0	0	0	0	0	0	0	0	0	0	0
0	0	0	0	0	0	0	0	0	0	0	0	0	0	0	0	0	0	0
0	0	0	0	0	0	0	0	0	0	0	0	0	0	0	0	0	0	0
0	0	0	0	0	0	0	0	0	0	0	0	0	0	0	0	0	0	0
0	0	0	0	0	0	0	0	0	0	0	0	0	0	0	0	0	0	0
0	0	0	0	0	0	0	0	0	0	0	0	0	0	0	0	0	0	0
0	0	0	0	0	0	0	0	0	0	0	0	0	0	0	0	0	0	0
0	0	0	0	0	0	0	0	0	0	0	0	0	0	0	0	0	0	0
0	0	0	0	0	0	0	0	0	0	0	0	0	0	0	0	0	0	0

0	0	0	0	0	0	0	0	0	0	0	0	0	0	0	0	0	0	0
0	0	0	0	0	0	0	0	0	0	0	0	0	0	0	0	0	0	0
0	0	0	0	0	0	0	0	0	0	0	0	0	0	0	0	0	0	0
0	2	2	2	2	2	0	0	0	0	0	0	0	0	0	0	0	0	0
0	0	0	0	0	0	0	0	0	0									
Jan_DL050	1397	1365	391	310	1299	164	558	534	34	132	196	197	126	165	715	229	207	186
161	1672	100	0	105	71	55	184	80	0	122	0	128	195	52	102	136	47	79
69	252	263	57	112	101	80	29	196	96	109	58	334	57	23	40	0	77	0
0	61	49	356	82	117	23	12	54	83	46	0	235	0	0	16	52	22	73
0	18	0	28	0	31	20	0	236	18	0	0	161	43	17	0	18	26	25
47	26	0	0	0	19	0	50	0	30	0	0	0	0	16	0	0	364	40
0	0	0	5	45	35	0	0	0	0	11	74	32	32	0	0	0	0	0
0	0	62	0	52	0	0	0	0	32	13	0	7	0	22	12	0	0	0
0	0	0	0	0	4	0	138	0	169	9	0	0	0	0	0	0	0	38
16	0	7	0	0	17	179	0	0	0	95	0	0	0	0	0	14	0	0
0	0	0	21	136	0	0	70	0	13	0	0	0	0	13	158	0	0	0
0	0	31	0	0	0	36	0	0	0	0	0	0	0	0	0	0	13	68
0	0	24	0	0	7	0	71	16	0	0	0	11	7	7	13	0	0	0
27	0	0	41	96	0	0	0	0	0	0	0	0	0	0	26	21	16	50
7	0	0	20	14	11	11	0	0	47	9	0	0	25	8	9	15	0	0
7	0	27	0	0	0	0	16	0	0	0	0	21	0	0	0	0	0	0
0	0	0	0	0	0	0	0	0	22	0	10	13	17	0	0	0	5	0
0	0	0	0	0	0	0	0	3	26	0	0	0	16	0	0	28	0	0
0	0	0	0	5	0	0	0	0	0	0	0	27	0	0	0	20	0	15
0	0	10	0	0	20	8	3	0	0	0	0	0	0	0	0	0	0	4
11	0	0	0	0	0	0	0	6	0	13	0	24	0	28	0	0	0	0
0	0	0	0	21	0	0	3	0	0	10	10	9	0	0	0	8	0	0
0	0	16	0	0	0	0	0	0	0	0	0	0	0	7	0	0	0	0
0	0	0	0	0	7	0	40	0	0	0	0	0	0	0	19	5	18	0
0	7	0	0	0	0	0	26	0	0	0	14	0	0	0	86	0	0	0
8	0	0	0	0	0	0	0	0	0	0	0	0	0	30	0	0	0	0
0	0	0	0	0	0	0	0	0	0	0	0	0	0	0	0	0	5	0
0	0	0	0	0	0	43	0	0	0	0	0	0	0	0	0	0	0	0
0	0	0	0	0	0	9	0	0	7	0	0	0	0	7	0	0	0	0
0	0	0	0	0	0	0	0	0	0	0	0	0	0	0	0	0	0	0
0	0	0	4	0	0	0	0	0	0	0	0	0	0	0	0	0	0	0
8	32	0	0	0	0	0	0	21	0	0	0	0	0	0	0	0	0	0

0	0	0	0	0	0	0	0	7	0	0	0	0	0	0	0	9	0	0
0	0	0	0	0	0	0	0	0	0	0	0	0	0	0	0	0	0	0
0	0	0	0	0	0	0	0	19	0	0	0	0	0	0	0	0	4	0
0	0	7	0	0	0	0	0	0	0	0	0	0	0	0	0	0	0	0
15	0	0	0	0	0	0	0	0	0	0	0	0	0	0	5	0	0	0
0	0	0	0	0	0	0	0	0	0	0	0	0	0	0	0	0	0	0
0	0	0	0	0	0	0	0	0	0	0	15	0	0	0	7	0	0	0
0	0	0	0	0	0	0	0	0	0	0	0	0	0	0	0	0	0	0
7	0	0	0	0	0	0	6	0	0	0	0	0	0	0	0	0	4	0
0	0	0	0	0	0	0	0	0	0	0	0	0	9	0	0	0	0	0
0	0	0	0	0	0	0	0	0	0	0	0	0	0	0	13	0	0	0
0	0	0	0	0	0	0	0	0	0	0	0	0	0	0	0	0	0	0
0	0	0	0	0	0	0	0	0	0	0	0	18	0	0	0	0	0	0
0	0	0	0	0	0	0	0	0	0	8	0	0	0	0	5	0	0	0
2	0	0	0	0	0	0	0	0	0	0	0	0	0	0	0	0	0	0
0	0	0	0	0	0	0	0	0	0	0	0	0	0	0	0	0	0	0
0	0	0	0	0	0	0	0	0	0	0	0	0	0	0	0	0	0	0
0	0	0	0	0	0	0	0	0	0	0	0	7	0	0	0	11	0	0
0	0	0	0	0	0	18	0	0	21	0	0	0	0	0	0	0	0	0
0	0	0	0	0	0	0	0	0	0	0	0	0	0	0	0	0	0	0
0	0	0	0	0	0	0	0	0	0	0	0	0	0	0	0	0	0	16
0	0	0	0	0	0	0	0	0	0	0	36	0	0	0	0	0	8	0
0	0	0	0	0	0	0	0	0	0	0	0	0	0	0	0	0	0	0
0	0	0	0	0	0	0	0	0	0	0	0	0	0	0	0	0	0	0
0	0	0	0	0	0	0	0	0	0	0	0	0	0	0	0	0	0	0
0	0	0	0	0	0	0	0	0	5	0	0	0	0	0	0	0	0	0
0	0	0	0	0	9	0	0	0	0	0	0	0	0	0	0	0	0	0
0	0	0	0	0	0	0	0	0	0	0	0	0	0	0	0	0	0	0
0	0	0	0	0	0	0	0	0	0	0	0	0	0	0	0	0	0	0
0	0	0	0	0	0	0	0	0	0	0	0	0	0	0	0	0	0	0
0	0	0	0	0	0	0	0	0	0	0	0	0	0	0	0	0	0	0
0	0	0	0	0	0	0	0	0	0	0	0	0	0	0	0	0	0	0
0	0	0	0	0	0	0	0	0	0	0	0	0	0	0	0	0	0	0
0	0	0	0	0	0	0	0	0	0	0	0	0	0	0	0	0	0	0
0	0	0	0	0	0	0	0	0	0	0	0	0	0	0	0	0	0	0
0	0	0	0	0	0	0	0	0	0	0	0	0	0	0	0	0	0	0
0	0	0	0	0	0	0	0	0	0	0	0	0	0	0	0	0	0	0

0	0	0	0	0	0	0	0	0	0	0	0	0	0	0	0	0	0	0
0	0	0	0	0	0	0	0	0	0	0	0	0	0	0	0	0	0	0
0	0	0	0	0	0	0	0	0	0	0	0	0	0	0	0	0	0	0
0	0	0	0	0	0	0	0	0	0	0	0	0	0	0	0	0	0	0
0	0	0	0	0	0	0	0	0	0	0	0	0	0	0	0	0	0	0
0	0	0	13	0	0	0	0	0	0	0	0	0	0	0	0	0	0	0
0	0	0	0	0	0	0	0	0	0	0	0	0	0	0	0	0	0	0
0	0	0	0	0	0	0	0	0	0	0	0	0	0	0	0	0	0	0
0	0	0	0	0	0	0	0	0	0	0	0	0	0	0	0	0	0	0
0	0	0	0	0	0	0	0	0	0	0	0	0	0	0	0	0	0	0
0	0	0	0	0	0	0	0	0	0	0	0	0	0	0	0	0	0	0
0	0	0	0	0	0	0	0	0	0	0	0	0	0	0	0	0	0	0
0	0	0	0	0	0	0	0	0	0	0	0	0	0	0	0	0	0	0
0	0	0	0	0	10	0	0	0	0	0	0	0	0	0	0	0	0	0
0	0	0	0	0	0	0	0	0	0	0	0	0	0	0	0	0	0	0
0	0	0	0	0	0	0	0	0	0	0	0	0	0	0	0	0	0	0
0	0	0	0	0	0	0	0	0	0	0	0	0	0	0	0	0	0	0
0	0	0	0	0	0	0	0	0	9	0	0	0	0	0	0	0	0	0
0	0	0	0	0	0	0	0	0	0	0	0	0	0	0	0	0	0	0
0	0	0	0	0	0	0	0	0	0	0	0	0	0	0	0	0	0	0
0	0	0	0	0	0	0	0	0	0	0	0	0	0	0	0	0	0	0
0	0	0	0	0	0	0	0	0	0	0	0	0	0	0	0	0	0	0
0	0	0	0	0	0	0	0	0	0	0	0	8	0	0	0	0	0	0
0	0	0	0	0	0	0	0	0	0	0	0	0	0	0	0	0	0	0
0	0	0	0	0	0	0	0	0	0	0	0	0	0	0	0	0	0	0
0	0	0	0	0	0	0	0	0	0	0	0	0	0	0	0	0	0	0
0	0	0	0	0	0	0	0	0	0	0	0	0	0	0	0	0	0	0
0	0	0	0	0	0	0	0	0	0	0	0	0	0	0	0	0	0	0
0	0	0	0	0	0	0	0	0	0	0	0	0	0	0	0	0	0	0
0	0	0	0	0	0	0	0	0	0	0	0	0	0	0	0	0	0	0
0	0	0	0	0	0	0	0	0	0	0	0	0	0	0	0	0	0	0
0	0	0	0	0	0	0	0	0	0	0	0	0	0	0	0	0	0	0
0	0	0	0	0	0	0	0	0	0	0	0	0	0	0	0	0	0	0
0	0	0	0	0	0	0	0	0	0	0	0	0	0	0	0	0	0	0
0	0	0	0	0	0	0	0	0	0	0	0	0	0	0	0	0	0	0
0	0	0	0	0	0	0	0	6	0	0	0	0	0	0	0	0	0	0
0	0	0	0	0	0	0	0	0	0	0	0	0	0	0	0	0	0	0

0	0	0	0	0	0	0	0	0	0	0	0	0	0	0	0	0	0	0
0	0	0	0	0	0	0	0	0	0	0	0	0	0	0	0	0	0	0
0	0	0	0	0	0	0	0	0	0	0	0	0	0	0	0	0	0	0
0	0	0	0	0	0	0	0	0	0	0	0	0	0	0	0	0	0	0
0	0	0	0	0	0	0	0	0	0	0	0	0	0	0	0	0	0	0
0	0	0	0	0	0	0	0	0	0	0	0	0	0	0	0	0	0	0
5	0	0	0	0	0	0	0	0	0	0	0	0	0	0	0	0	0	0
0	0	0	0	0	0	0	0	0	0	0	0	0	0	0	0	0	0	0
0	0	0	0	0	0	0	0	0	0	0	0	0	0	0	0	0	0	0
0	0	0	0	0	0	0	0	0	0	0	0	0	0	0	0	0	0	0
0	0	0	0	0	0	0	0	0	0	0	0	0	0	0	0	0	0	0
0	0	0	0	0	0	0	0	0	0	0	0	0	0	0	0	0	0	0
0	0	0	0	0	0	0	0	0	0	0	0	0	0	0	0	0	0	0
0	0	0	0	0	0	0	0	0	0	0	0	0	0	0	0	0	0	0
0	0	0	0	0	0	0	0	0	0	0	0	0	0	0	0	0	0	0
0	0	0	0	0	0	0	0	0	0	0	0	0	0	0	0	0	0	0
0	0	0	0	0	0	0	0	0	0	0	0	0	0	0	0	0	0	0
0	0	0	0	0	0	0	0	0	0	0	0	0	0	0	0	0	0	0
0	0	0	0	0	0	0	0	0	0	0	0	0	0	0	0	0	0	0
0	0	0	0	0	0	0	0	0	0	0	0	0	0	0	0	0	0	0
0	0	0	0	0	0	0	0	0	0	0	0	0	0	0	0	0	0	0
0	0	0	0	0	0	0	0	0	0	0	0	0	0	0	0	0	0	0
0	0	0	0	0	0	0	0	0	0	0	0	0	0	0	0	0	0	0
0	0	0	0	0	0	0	0	0	0	0	0	0	0	0	0	0	0	0
0	0	0	0	0	0	0	0	0	0	0	0	0	0	0	0	0	0	0
0	0	0	0	0	0	0	0	0	0	0	0	0	0	0	0	0	0	0
0	0	0	0	0	0	0	0	0	0	0	0	0	0	0	0	0	0	0
0	0	0	0	0	0	0	0	0	0	0	0	0	0	0	0	0	0	0
0	0	0	0	0	0	0	0	0	0	0	0	0	0	0	0	0	0	0
0	0	0	0	0	0	0	0	0	0	0	0	0	0	0	0	0	0	0
0	0	0	0	0	0	0	0	0	0	0	0	0	0	0	0	0	0	0
0	0	0	0	0	0	0	0	0	0	0	0	0	0	0	0	0	0	0
0	0	0	0	0	0	0	0	0	0	0	0	0	0	0	0	0	0	0
0	0	0	0	0	0	0	0	0	0	0	0	0	0	0	0	0	0	0
0	0	0	0	0	0	0	0	0	0	0	0	0	0	0	0	0	0	0
0	0	0	0	0	0	0	0	0	0	0	0	0	0	0	0	0	0	0
0	0	0	0	0	0	0	0	0	0	0	0	0	0	3	3	3	3	0

0	0	0	0	0	0	0	0	0	0	0	0	0	0	0	0	0	0	0
0	0	0	0	0	0	0	0	0	0	0	0	0	0	0	0	0	0	0
0	0	0	0	0	0	0	0	0	0	0	0	0	0	0	0	0	0	0
0	0	0	0	0	0	0	0	0	0	0	0	0	0	0	0	0	0	0
0	0	0	0	0	0	0	0	0	0	0	0	0	0	0	0	0	0	0
0	0	0	0	0	0	0	0	0	0	0	0	0	0	0	0	0	0	0
0	0	0	0	0	0	0	0	0	0	0	0	0	0	0	0	0	0	0
0	0	0	0	0	0	0	0	0	0	0	0	0	0	0	0	0	0	0
0	0	0	0	0	0	0	0	0	0	0	0	0	0	0	0	0	0	0
0	0	0	0	0	0	0	0	0	0	0	0	0	0	0	0	0	0	0
0	0	0	0	0	0	0	0	0	0	0	0	0	0	0	0	0	0	0
0	0	0	0	0	0	0	0	0	0	0	0	0	0	0	0	0	0	0
0	0	0	0	0	0	0	0	0	0	0	0	0	0	0	0	0	0	0
0	0	0	0	0	0	0	0	0	0	0	0	0	0	0	0	0	0	0
0	0	0	0	0	0	0	0	0	0	0	0	0	0	0	0	0	0	0
0	0	0	0	0	0	0	0	0	0	0	0	0	0	0	0	0	0	0
0	0	0	0	0	0	0	0	0	0	0	0	0	0	0	0	0	0	0
0	0	0	0	0	0	0	0	0	0	0	0	0	0	0	0	0	0	0
0	0	0	0	0	0	0	0	0	0	0	0	0	0	0	0	0	0	0
0	0	0	0	0	0	0	0	0	0	0	0	0	0	0	0	0	0	0
0	0	0	0	0	0	0	0	0	0	0	0	0	0	0	0	0	0	0
0	0	0	0	0	0	0	0	0	0	0	0	0	0	0	0	0	0	0
0	0	0	0	0	0	0	0	0	0	0	0	0	0	0	0	0	0	0
0	0	0	0	0	0	0	0	0	0	0	0	0	0	0	0	0	0	0
0	0	0	0	0	0	0	0	0	0	0	0	0	0	0	0	0	0	0
0	0	0	0	0	0	0	0	0	0	0	0	0	0	0	0	0	0	0
0	0	0	0	0	2	2	0	0	0	0	0	0	0	0	0	0	0	0
0	0	0	0	0	0	0	0	0	0	0	0	0	0	0	0	0	0	0
0	0	0	0	0	0	0	0	0	0	0	0	0	0	0	0	0	0	0
0	0	0	0	0	0	0	0	0	0	0	0	0	0	0	0	0	0	0
0	0	0	0	0	0	0	0	0	0	0	0	0	0	0	0	0	0	0
0	0	0	0	0	0	0	0	0	0	0	0	0	0	0	0	0	0	0
0	0	0	0	0	0	0	0	0	0	0	0	0	0	0	0	0	0	0
0	0	0	0	0	0	0	0	0	0									
Jan_DL052	1336	1664	1244	217	110	160	67	52	56	465	79	179	426	22	239	110	25	157
47	29	469	0	159	388	327	102	216	185	0	0	149	101	182	0	87	49	0
184	0	0	0	86	0	0	134	28	28	0	54	0	28	16	17	24	29	0

0	0	0	26	0	53	47	74	172	40	0	0	94	86	0	0	13	22	15
47	0	0	75	0	38	28	0	0	65	0	0	0	42	47	0	40	32	29
0	15	0	0	51	0	143	0	0	20	0	0	0	70	24	0	0	126	8
0	0	0	0	42	43	0	0	0	0	22	0	0	0	0	0	0	0	0
0	62	0	0	15	62	0	0	0	8	56	0	15	0	26	13	0	0	0
0	7	112	3	0	4	0	144	107	115	0	0	0	48	0	18	0	0	31
0	0	0	0	0	0	117	0	0	16	0	7	0	0	0	0	0	0	0
0	0	0	11	104	0	0	0	0	0	0	9	0	59	0	102	0	0	0
0	0	0	0	0	0	0	0	0	0	0	0	0	0	0	0	70	7	0
0	0	25	27	0	5	0	66	6	13	0	25	0	10	10	0	0	0	0
0	0	0	0	29	0	0	0	0	9	0	6	0	9	0	0	0	0	0
0	0	0	0	0	7	69	0	0	0	9	0	0	0	13	3	0	0	0
0	0	10	0	0	0	0	0	4	0	0	21	0	0	0	0	6	0	0
0	7	0	0	0	0	0	11	0	0	0	0	0	0	0	0	0	0	0
0	0	0	7	0	0	0	0	0	0	0	0	0	0	0	0	0	0	0
0	0	0	0	7	0	0	0	0	0	0	0	19	0	0	105	11	0	0
0	0	0	0	30	0	0	7	0	0	0	0	39	30	0	0	0	0	0
22	0	0	0	8	0	0	0	0	0	0	0	0	0	0	0	0	0	0
0	0	0	0	0	0	0	0	0	28	0	0	0	0	0	0	0	0	0
0	0	0	0	0	0	0	0	0	0	0	0	0	0	26	0	0	0	0
0	13	0	0	0	0	0	0	0	0	0	0	0	0	0	19	0	0	0
0	0	0	0	0	0	0	0	0	0	0	0	0	0	0	0	0	0	0
0	0	0	0	0	0	0	0	54	0	0	0	0	0	0	0	0	0	0
0	0	0	0	0	0	0	0	0	0	0	0	0	0	0	0	0	6	0
0	0	0	0	0	0	0	0	0	0	0	0	0	0	0	0	0	0	0
0	0	0	0	0	25	0	0	0	0	0	7	0	0	0	0	0	0	0
0	4	0	0	0	0	0	0	0	0	0	13	0	0	0	0	0	0	0
0	0	0	0	0	11	0	0	0	0	0	0	0	0	0	0	0	0	0
0	0	0	0	0	0	0	0	0	0	0	0	0	0	0	0	0	0	0
0	0	0	0	0	0	0	0	0	0	0	0	0	0	0	0	0	0	0
5	0	0	0	0	0	0	0	0	0	0	0	0	13	0	0	0	0	0
0	0	0	0	0	0	0	0	0	9	0	0	11	0	0	0	0	0	0
0	0	0	0	0	0	0	0	0	0	0	0	0	0	0	0	0	0	0
0	0	0	0	0	0	0	0	0	0	11	0	0	0	0	0	0	0	0
0	8	0	0	0	0	0	0	0	0	17	0	0	0	0	0	0	0	0
0	0	0	0	0	0	0	0	0	0	0	0	0	0	0	0	0	0	0
0	0	0	0	0	0	0	0	0	0	0	0	0	0	0	0	0	0	0

0	0	0	0	0	0	0	0	0	0	0	0	0	0	0	0	0	0	0
0	0	0	32	0	0	0	0	0	0	0	0	0	0	0	0	0	0	0
0	0	0	0	0	0	0	0	0	0	0	0	0	0	0	0	0	0	0
0	0	0	0	0	0	0	0	0	0	0	0	0	0	0	0	0	0	0
0	0	0	0	0	0	0	0	0	0	0	0	0	0	0	0	0	0	0
0	0	0	0	0	0	0	0	0	0	0	0	0	0	0	0	0	0	0
0	0	0	0	0	0	0	0	0	0	0	0	0	0	0	0	0	0	0
0	0	0	0	0	0	0	0	0	0	0	0	0	0	0	0	0	0	0
0	0	0	0	0	0	0	0	0	0	0	0	0	0	0	0	0	0	0
0	0	0	0	0	0	0	0	0	0	0	0	0	0	0	0	0	0	0
0	0	0	0	0	0	0	0	0	0	0	0	0	0	0	0	0	19	0
0	0	0	0	6	0	0	0	0	0	0	0	0	0	0	0	0	5	0
0	0	0	0	0	0	0	0	0	0	0	0	0	0	0	0	0	0	0
0	0	0	0	0	0	0	0	0	0	9	0	0	0	0	0	0	0	23
0	0	0	0	0	0	0	0	0	0	0	0	0	0	0	0	0	0	0
0	0	0	0	0	0	0	0	0	0	0	0	0	0	0	0	0	0	0
0	0	0	0	0	0	0	0	0	12	6	0	0	0	0	0	0	0	0
0	0	0	0	0	0	3	0	0	0	0	0	0	0	0	0	0	0	0
0	0	0	0	0	0	0	0	0	0	0	0	0	0	4	0	0	0	0
0	0	0	0	0	0	0	0	0	0	0	0	0	0	0	0	0	0	0
0	0	0	0	0	0	0	0	0	0	0	0	0	0	0	0	0	0	0
0	0	0	0	0	0	0	0	0	0	0	0	0	0	16	0	0	0	0
0	0	0	0	0	0	0	0	0	0	0	0	0	0	0	0	0	0	0
0	0	0	0	0	0	0	0	0	0	0	0	0	0	0	0	0	0	0
0	0	0	0	0	0	0	0	0	0	0	0	0	0	0	0	0	0	0
0	3	0	0	0	0	0	0	0	0	0	0	0	0	0	0	0	13	0
0	0	0	0	0	0	0	0	0	0	0	0	0	0	0	0	0	0	0
0	0	0	0	0	0	0	0	0	0	0	0	0	0	0	0	0	0	0
0	0	0	0	0	0	0	0	0	0	0	0	0	0	0	0	0	0	0
0	0	0	0	0	0	0	0	0	0	0	0	0	0	0	0	0	0	0
0	0	0	0	0	0	0	0	0	0	0	0	0	0	0	0	0	0	0
0	0	0	0	0	0	0	0	0	0	0	0	0	0	0	0	0	0	0
0	0	0	0	0	0	0	0	0	0	0	0	0	0	0	0	0	0	0
0	0	0	0	0	0	0	0	0	0	0	0	0	0	0	0	0	0	0
0	0	0	0	0	0	0	0	0	0	0	0	0	0	0	0	0	0	0
0	0	0	0	0	0	0	0	0	0	0	0	0	0	0	0	0	0	0
0	0	0	0	0	0	0	0	0	0	0	0	0	0	0	0	0	0	0

0	0	0	0	0	0	0	0	0	0	0	0	0	0	0	0	0	0	0
0	0	0	0	0	0	0	0	0	0	0	0	0	0	0	0	0	0	0
0	0	0	0	0	0	0	0	0	0	0	0	0	0	0	0	0	0	0
0	0	0	0	0	0	0	0	0	0	0	0	0	0	0	0	0	0	0
0	0	0	0	0	0	0	0	0	0	0	0	0	0	0	0	0	0	0
0	0	0	0	0	0	0	0	0	0	0	0	0	0	0	0	0	0	0
7	0	0	0	0	0	0	0	0	0	0	0	0	0	0	0	0	0	0
0	0	0	0	0	0	0	0	0	0	0	0	0	0	0	2	0	0	0
0	0	0	0	0	0	0	0	0	0	0	0	0	0	0	0	0	0	0
0	0	0	0	0	0	0	0	0	0	0	0	0	0	0	0	0	0	0
0	0	0	0	0	0	0	0	0	0	0	0	0	0	0	0	0	0	0
0	0	0	0	0	0	0	0	0	0	0	0	0	0	0	0	0	0	0
0	0	0	0	0	0	0	6	6	0	0	0	0	0	0	0	0	0	0
0	0	0	0	0	0	0	0	0	0	0	0	0	0	0	0	0	0	0
0	0	0	0	0	0	0	0	0	0	0	0	0	0	0	0	0	0	0
0	0	0	0	0	0	0	0	0	0	0	0	0	0	0	0	0	0	0
0	0	0	0	0	0	0	0	0	0	0	0	0	0	0	0	0	0	0
0	0	0	0	0	0	0	0	0	0	0	0	0	0	0	0	0	0	0
0	0	0	0	0	0	0	0	0	0	0	0	0	0	0	0	0	0	0
0	0	0	0	0	0	0	0	0	0	0	0	0	0	0	0	0	0	0
0	0	0	0	5	5	0	0	0	0	0	0	0	0	0	0	0	0	0
0	0	0	0	0	0	0	0	0	0	0	0	0	0	0	0	0	0	0
0	0	0	0	0	0	0	0	0	0	0	0	0	0	0	0	0	0	0
0	0	0	0	0	0	0	0	0	0	0	0	0	0	0	0	0	0	0
0	0	0	0	0	0	0	0	0	0	0	0	0	0	0	0	0	0	0
0	0	0	0	0	0	0	0	0	0	0	0	0	0	0	0	0	0	0
0	0	0	0	0	0	0	0	0	0	0	0	0	0	0	0	0	0	0
0	0	0	0	0	0	0	0	0	0	0	0	0	0	0	0	0	0	0
0	0	0	0	0	0	0	0	0	0	0	0	0	0	0	0	0	0	0
0	0	0	0	0	0	0	0	0	0	0	0	0	0	0	0	0	4	0
0	0	0	0	0	0	0	0	0	0	0	0	0	0	0	0	0	0	0
0	0	0	0	0	0	0	0	0	0	0	0	0	0	0	0	0	0	0
0	0	0	0	0	0	0	0	0	0	0	0	0	0	0	0	0	0	0
0	0	0	0	0	0	0	0	0	0	0	0	0	0	0	0	0	0	0
0	0	0	0	0	0	0	0	0	0	0	0	0	0	0	0	0	0	0
0	0	0	0	0	0	0	0	0	0	0	0	0	0	0	0	0	0	0
0	0	0	0	0	0	0	0	0	0	0	0	0	0	0	0	0	0	0

0	0	0	0	0	0	0	0	0	0	0	0	0	0	0	0	0	0	0
0	0	0	0	0	0	0	0	0	0	0	0	0	0	0	0	0	0	0
0	0	0	0	0	0	0	0	0	0	0	0	0	0	0	0	0	0	0
0	0	0	0	0	0	0	0	0	0	0	0	0	0	0	0	0	0	0
0	0	0	0	0	0	0	0	0	0	0	0	0	0	0	0	0	0	0
0	0	0	0	0	0	0	0	0	0	0	0	0	0	0	0	0	0	0
0	0	0	0	0	0	0	0	0	0	0	0	0	0	0	0	0	0	0
0	0	0	0	0	0	0	0	0	0	0	0	0	0	0	0	0	0	0
3	0	0	0	0	0	0	0	0	0	0	0	0	0	0	0	0	0	0
0	0	0	0	0	0	0	0	0	0	0	0	0	0	0	0	0	0	0
0	0	0	0	0	0	0	0	0	0	0	0	0	0	0	0	0	0	0
0	0	0	0	0	0	0	0	0	0	0	0	0	0	0	0	0	0	0
0	0	0	0	0	0	0	0	0	0	0	0	0	0	0	0	0	0	0
0	0	0	0	0	0	0	0	0	0	0	0	0	0	0	0	0	0	0
0	0	0	0	0	0	0	0	0	0	0	0	0	0	0	0	0	0	0
0	0	0	0	0	0	0	0	0	0	0	0	0	0	0	0	0	0	0
0	0	0	0	0	0	0	0	0	0	0	0	0	0	0	0	0	0	0
0	0	0	0	0	0	0	0	0	0	0	0	0	0	0	0	0	0	0
0	0	0	0	0	0	0	0	0	0	0	0	0	0	0	0	0	0	0
0	0	0	0	0	0	0	0	0	0	0	0	0	0	0	0	0	0	0
0	0	0	0	0	0	0	0	0	0	0	0	0	0	0	0	0	0	0
0	0	0	0	0	0	0	0	0	0	0	0	0	0	0	0	0	0	0
0	0	0	0	0	0	0	0	0	0	0	0	0	0	0	0	0	0	0
0	0	0	0	0	0	0	0	0	0	0	0	0	0	0	0	0	0	0
0	0	0	0	0	0	0	0	0	0	0	0	0	0	0	0	0	0	0
0	0	0	0	0	0	0	0	0	0	0	0	0	0	0	0	0	0	0
0	0	0	0	0	0	0	0	0	0	0	0	0	0	0	0	0	0	0
0	0	0	0	0	0	0	0	0	0	0	0	0	0	0	0	0	0	0
0	0	0	0	0	0	0	0	0	0	0	0	0	0	0	0	0	0	0
0	0	0	0	0	0	0	0	0	0	0	0	0	0	0	0	0	0	0
0	0	0	0	0	0	0	0	0	0	0	0	0	0	0	0	0	0	0
0	0	0	0	0	0	0	0	0	0	0	0	0	0	0	0	0	0	0
0	0	0	0	0	0	0	0	0	0	0	0	0	0	0	0	0	0	0
0	0	0	0	0	0	0	0	0	0	0	0	0	0	2	2	2	2	2
0	0	0	0	0	0	0	0	0	0	0	0	0	0	0	0	0	0	0
0	0	0	0	0	0	0	0	0	0	0	0	0	0	0	0	0	0	0
0	0	0	0	0	0	0	0	0	0	0	0	0	0	0	0	0	0	0

0	0	0	0	0	0	0	0	0	0	0	0	0	0	0	0	0	0	0
0	0	0	0	0	0	0	0	0	0	0	0	0	0	0	0	0	0	0
0	0	0	0	0	0	0	0	0	0	0	0	0	0	0	0	0	0	0
0	0	0	0	0	0	0	0	0	0	0	0	0	0	0	0	0	0	0
0	0	0	0	0	0	0	0	0	0	0	0	0	0	0	0	0	0	0
0	0	0	0	0	0	0	0	0	0	0	0	0	0	0	0	0	0	0
0	0	0	0	0	0	0	0	0	0	0	0	0	0	0	0	0	0	0
0	0	0	0	0	0	0	0	0	0	0	0	0	0	0	0	0	0	0
0	0	0	0	0	0	0	0	0	0	0	0	0	0	0	0	0	0	0
0	0	0	0	0	0	0	0	0	0	0	0	0	0	0	0	0	0	0
0	0	0	0	0	0	0	0	0	0	0	0	0	0	0	0	0	0	0
0	0	0	0	0	0	0	0	0	0	0	0	0	0	0	0	0	0	0
0	0	0	0	0	0	0	0	0	0	0	0	0	0	0	0	0	0	0
0	0	0	0	0	0	0	0	0	0	0	0	0	0	0	0	0	0	0
0	0	0	0	0	0	0	0	0	0	0	0	0	0	0	0	0	0	0
0	0	0	0	0	0	0	0	0	0	0	0	0	0	0	0	0	0	0
0	0	0	0	0	0	0	0	0	0	0	0	0	0	0	0	0	0	0
0	0	0	0	0	0	0	0	0	0	0	0	0	0	0	0	0	0	0
0	0	0	0	0	0	0	0	0	0	0	0	0	0	0	0	0	0	0
0	0	0	0	0	0	0	0	0	0	0	0	0	0	0	0	0	0	0
0	0	0	0	0	0	0	0	0	0	0	0	0	0	0	0	0	0	0
0	0	0	0	0	0	0	0	0	0	0	0	0	0	0	0	0	0	0
0	0	0	0	0	0	0	0	0	0	0	0	0	0	0	0	0	0	0
0	0	0	0	0	0	0	0	0	0	0	0	0	0	0	0	0	0	0
0	0	0	0	0	0	0	0	0	0	0	0	0	0	0	0	0	0	0
0	0	0	0	0	0	0	0	0	0									
Jan_DL053	1686	852	595	333	35	36	52	58	105	378	107	297	317	19	187	358	22	171
69	394	128	0	83	98	299	84	165	120	25	0	90	80	167	0	69	76	0
121	77	0	8	47	0	0	535	0	165	0	229	26	0	0	251	181	0	0
0	0	245	24	0	125	0	52	114	17	11	0	23	0	3	0	0	17	25
135	160	0	23	5	114	70	0	0	103	0	0	19	21	33	82	8	21	81
16	14	0	0	86	0	63	0	0	32	0	0	0	0	15	10	10	0	23
0	9	0	0	0	58	0	0	0	0	0	0	0	0	0	0	7	0	0
0	0	0	0	12	92	0	0	0	5	0	0	7	0	0	0	0	0	0
0	0	25	0	0	0	0	54	0	43	0	0	0	0	0	14	10	0	0
0	0	0	0	0	0	0	0	0	0	0	8	0	0	0	0	0	0	12
92	0	0	28	49	0	0	0	0	0	0	0	0	0	0	0	0	0	0

0	0	0	0	12	0	0	0	0	0	0	0	0	0	0	0	0	0	0
0	0	31	23	0	0	0	0	0	23	0	40	0	9	0	0	0	0	0
0	0	0	0	0	0	0	0	0	0	0	113	0	0	0	0	0	0	0
0	0	0	0	0	0	0	0	0	0	0	6	0	0	0	0	0	0	0
0	0	26	0	0	0	0	0	0	0	0	0	0	41	0	0	0	0	0
0	0	0	0	0	0	0	0	0	0	0	0	0	0	9	0	0	0	2
0	0	0	0	0	0	0	0	0	0	0	0	0	0	0	0	0	0	0
0	0	0	0	8	0	0	0	0	0	0	0	8	0	0	27	0	0	0
0	0	0	0	0	0	0	0	0	0	0	63	0	10	0	0	0	0	0
0	0	0	0	17	0	7	0	0	0	0	0	0	0	0	0	0	0	0
0	0	0	0	0	0	0	0	0	0	0	0	0	0	0	0	4	0	0
0	0	0	0	0	0	0	0	0	0	0	0	0	0	5	0	0	0	0
0	0	0	0	115	0	0	14	0	0	0	0	0	0	0	0	0	0	0
0	0	0	0	0	0	0	0	0	0	0	0	0	0	0	0	0	0	0
0	0	0	0	0	0	0	0	0	0	0	0	0	0	0	7	0	0	0
0	0	0	0	0	0	0	0	0	0	0	0	0	0	0	0	0	0	0
0	0	0	0	0	0	0	0	0	0	0	0	0	0	0	0	0	0	0
0	0	0	0	0	0	0	0	0	0	0	0	0	0	0	0	0	0	0
0	0	0	0	0	0	0	0	0	0	0	0	0	0	0	0	0	13	0
0	0	0	0	0	0	0	29	0	0	0	0	0	0	0	0	0	0	0
0	0	0	0	0	0	0	0	0	0	0	0	0	0	0	0	0	0	0
0	0	0	0	0	0	0	0	0	0	0	0	0	0	0	0	0	0	0
0	0	0	0	0	0	0	0	0	0	0	0	0	8	0	0	0	0	0
0	0	0	0	0	0	0	0	0	0	0	0	0	0	0	0	0	0	0
0	0	0	0	0	0	0	0	0	0	0	0	0	0	0	0	0	0	0
0	0	0	0	0	0	0	0	0	0	0	0	0	0	0	0	0	0	0
0	0	0	0	0	0	0	0	0	0	0	0	0	0	0	0	0	0	0
0	0	0	0	0	0	0	0	0	0	0	0	0	0	0	0	0	0	0
4	0	0	0	0	0	0	0	0	0	0	0	0	0	0	0	0	0	0
0	0	0	0	13	0	0	0	0	0	0	0	0	0	0	0	0	0	0
0	0	0	24	0	0	0	0	0	0	0	0	0	0	0	0	0	0	0
0	0	0	0	0	0	0	0	0	0	0	0	0	0	0	0	0	0	0
0	0	0	0	0	0	0	0	0	0	0	0	0	0	0	0	0	0	0
0	0	0	0	0	0	0	0	0	0	0	0	0	0	0	0	0	0	0
0	0	0	0	0	0	0	0	0	0	0	0	0	0	0	0	0	0	0
0	0	0	0	0	0	0	0	0	0	0	0	0	0	0	0	0	0	0
0	0	0	0	0	0	0	0	0	0	0	0	0	0	0	0	0	0	0

0	0	0	0	0	0	0	0	0	0	0	0	0	0	0	0	0	0	0
0	0	0	0	0	0	0	0	0	0	0	0	0	0	0	0	0	0	0
0	0	0	0	0	0	0	0	0	0	0	0	0	0	0	0	0	0	0
0	0	0	0	0	0	0	0	0	0	0	0	0	0	0	0	0	0	0
0	0	0	0	0	0	0	0	0	0	0	0	0	0	0	0	0	0	0
0	0	0	0	0	0	0	0	0	0	0	0	0	0	0	0	0	0	0
0	0	0	0	0	0	0	0	0	0	0	0	0	0	0	0	0	0	0
0	0	0	0	0	0	0	0	0	0	0	0	0	0	0	0	0	0	0
0	0	0	0	0	0	0	0	0	0	0	0	0	0	0	0	0	0	0
0	0	0	0	0	0	0	0	0	0	0	0	0	0	0	0	0	0	0
0	0	0	0	0	0	0	0	0	0	0	0	0	0	0	0	0	0	0
0	0	0	0	0	0	0	0	0	0	0	0	0	0	0	0	0	0	0
0	0	0	0	0	0	0	0	0	0	0	0	0	0	0	0	0	0	0
0	0	0	0	0	0	0	0	0	0	0	0	0	0	0	0	0	0	0
0	0	0	0	0	0	0	0	0	0	0	0	0	0	0	0	0	0	0
0	0	0	0	0	0	0	0	0	0	0	0	0	0	0	0	0	0	0
0	0	0	0	0	0	0	0	0	0	0	0	0	0	0	0	0	0	0
0	0	0	0	0	0	0	0	0	0	0	0	0	0	0	0	0	0	0
0	0	0	0	0	0	0	0	0	0	0	0	0	0	0	0	0	0	0
0	0	0	0	0	0	0	0	0	0	0	0	0	0	0	0	0	0	0
0	0	0	0	0	0	0	0	0	0	0	0	0	0	0	0	0	0	0
0	0	0	0	0	0	0	0	0	10	0	0	0	0	0	0	0	0	0
0	0	0	0	0	0	0	0	0	0	0	0	0	0	0	0	0	0	0
0	0	0	0	0	0	0	0	0	0	0	0	0	0	0	0	0	0	0
0	0	0	0	0	0	0	0	0	0	0	0	9	0	0	0	0	0	0
0	0	0	0	0	0	0	0	0	0	0	0	0	0	0	0	0	0	0
0	0	0	0	0	0	0	0	0	0	0	0	0	0	0	0	0	0	0
0	0	0	0	0	0	0	0	0	0	0	0	0	0	0	0	0	0	0
0	0	0	0	0	0	0	0	0	0	0	0	0	0	0	0	0	0	0
0	0	0	0	0	0	0	0	0	0	0	0	0	0	0	0	0	0	0
0	0	0	0	0	0	0	0	0	0	0	0	0	0	0	0	0	0	0
0	0	0	0	0	0	0	0	0	0	0	0	0	0	0	0	0	0	0
0	0	0	0	0	0	0	0	0	0	0	0	0	0	0	0	0	0	0
0	0	0	0	0	0	0	0	0	0	0	0	0	0	0	0	0	0	0
0	0	0	0	0	0	0	0	0	0	0	0	0	0	0	0	0	0	0
0	0	0	0	0	0	0	0	0	0	0	0	0	0	0	0	0	0	0
0	0	0	0	0	0	0	0	6	6	0	0	0	0	0	0	0	0	0

0	0	0	0	0	0	0	0	0	0	0	0	0	0	0	0	0	0	0
0	0	0	0	0	0	0	0	0	0	0	0	0	0	0	0	0	0	0
0	0	0	0	0	0	0	0	0	0	0	0	0	0	0	0	0	0	0
0	0	0	0	0	0	0	0	0	0	0	0	0	0	0	0	0	0	0
0	0	0	0	0	0	0	0	0	0	0	0	0	0	0	0	0	0	0
0	0	0	0	0	0	0	0	0	0	0	0	0	0	0	0	0	0	0
0	0	0	0	0	0	0	0	0	0	0	0	0	0	0	0	0	0	0
5	0	0	0	0	0	0	0	0	0	0	0	0	0	0	0	0	0	0
0	0	0	0	0	0	0	0	0	0	0	0	0	0	0	0	0	0	0
0	0	0	0	0	0	0	0	0	0	0	0	0	0	0	0	0	0	0
0	0	0	0	0	0	0	0	0	0	0	0	0	0	0	0	0	0	0
0	0	0	0	0	0	0	0	0	0	0	0	0	0	0	0	0	0	0
0	0	0	0	0	0	0	0	0	0	0	0	0	0	0	0	0	0	0
0	0	0	0	0	0	0	0	0	0	0	0	0	0	0	0	0	0	0
0	0	0	0	0	0	0	0	0	0	0	0	0	0	0	0	0	0	0
0	0	0	0	0	0	0	0	0	0	0	0	0	0	0	0	0	0	0
0	0	0	0	0	0	0	0	0	0	0	0	0	0	0	0	0	0	0
0	0	0	0	0	0	0	0	0	0	0	0	0	0	0	0	0	0	0
0	0	0	0	0	0	0	0	0	0	0	0	0	0	0	0	0	0	0
0	0	0	0	0	0	0	0	0	0	0	0	0	0	0	0	0	0	0
0	0	0	0	0	0	0	0	0	0	0	0	0	0	0	0	0	0	0
0	0	0	0	0	0	0	0	0	0	0	0	0	0	0	0	0	0	0
0	0	0	0	0	0	0	0	0	0	0	0	0	0	0	0	0	0	0
0	0	0	0	0	0	0	0	0	0	0	0	0	0	0	0	0	0	0
0	0	0	0	0	0	0	0	0	0	0	0	0	0	0	0	0	0	0
0	0	0	0	0	0	0	0	0	0	0	0	0	0	0	0	0	0	0
0	0	0	0	0	0	0	0	0	0	0	0	0	0	0	0	0	0	0
0	0	0	0	0	0	0	0	0	0	0	0	0	0	0	0	0	0	0
0	0	0	0	0	0	0	0	0	0	0	0	0	0	0	0	0	0	0
0	0	0	0	0	0	0	0	0	0	0	0	0	0	0	0	0	0	0
0	0	0	0	0	0	0	0	0	0	0	0	0	0	0	0	0	0	0
0	0	0	0	0	0	0	0	0	0	0	0	0	0	0	0	0	0	0
0	0	0	0	0	0	0	0	0	0	0	0	0	0	0	0	0	0	0
0	0	0	0	0	0	0	0	0	0	0	0	0	0	0	0	0	0	0
0	0	0	0	0	0	0	0	0	0	0	0	0	0	0	0	0	0	0
0	0	0	0	0	0	0	0	0	0	0	0	0	0	0	0	0	0	0
0	0	0	0	0	0	0	0	0	0	0	0	0	0	0	0	0	0	0

0	0	0	0	0	0	0	0	0	0	0	0	0	0	0	0	0	0	0
0	0	0	0	0	0	0	0	0	0	0	0	0	0	0	0	0	0	0
0	0	0	0	0	0	0	0	0	0	0	0	0	0	0	0	0	0	0
0	0	0	0	0	0	0	0	0	0	0	0	0	0	0	0	0	0	0
0	0	0	0	0	0	0	0	0	0	0	0	0	0	0	0	0	0	0
0	0	0	0	0	0	0	0	0	0	0	0	0	0	0	0	0	0	0
0	0	0	0	0	0	0	0	0	0	0	0	0	0	0	0	0	0	0
0	0	0	0	0	0	0	0	0	0	0	0	0	0	0	0	0	0	0
0	0	0	0	0	0	0	0	0	0	0	0	0	0	0	0	0	0	0
0	0	0	0	0	0	0	0	0	0	0	0	0	0	0	0	0	0	0
0	0	0	0	0	0	0	0	0	0	0	0	0	0	0	0	0	0	0
0	0	0	0	0	0	0	0	0	0	0	0	0	0	0	0	0	0	0
0	0	0	0	0	0	0	0	0	0	0	0	0	0	0	0	0	0	0
0	0	0	0	0	0	0	0	0	0	0	0	0	0	0	0	0	0	0
0	0	0	0	0	0	0	0	0	0	0	0	0	0	0	0	0	0	0
0	0	0	0	0	0	0	0	0	0	0	0	0	0	0	0	0	0	0
0	0	0	0	0	0	0	0	0	0	0	0	0	0	0	0	0	0	0
0	0	0	0	0	0	0	0	2	2	2	0	0	0	0	0	0	0	0
0	0	0	0	0	0	0	0	0	0	0	0	0	0	0	0	0	0	0
0	0	0	0	0	0	0	0	0	0	0	0	0	0	0	0	0	0	0
0	0	0	0	0	0	0	0	0	0	0	0	0	0	0	0	0	0	0
0	0	0	0	0	0	0	0	0	0	0	0	0	0	0	0	0	0	0
0	0	0	0	0	0	0	0	0	0	0	0	0	0	0	0	0	0	0
0	0	0	0	0	0	0	0	0	0	0	0	0	0	0	0	0	0	0
0	0	0	0	0	0	0	0	0	0	0	0	0	0	0	0	0	0	0
0	0	0	0	0	0	0	0	0	0	0	0	0	0	0	0	0	0	0
0	0	0	0	0	0	0	0	0	0	0	0	0	0	0	0	0	0	0
0	0	0	0	0	0	0	0	0	0	0	0	0	0	0	0	0	0	0
0	0	0	0	0	0	0	0	0	0	0	0	0	0	0	0	0	0	0
0	0	0	0	0	0	0	0	0	0	0	0	0	0	0	0	0	0	0
0	0	0	0	0	0	0	0	0	0	0	0	0	0	0	0	0	0	0
0	0	0	0	0	0	0	0	0	0	0	0	0	0	0	0	0	0	0
0	0	0	0	0	0	0	0	0	0	0	0	0	0	0	0	0	0	0
0	0	0	0	0	0	0	0	0	0	0	0	0	0	0	0	0	0	0
0	0	0	0	0	0	0	0	0	0	0	0	0	0	0	0	0	0	0
0	0	0	0	0	0	0	0	0	0	0	0	0	0	0	0	0	0	0
0	0	0	0	0	0	0	0	0	0	0	0	0	0	0	0	0	0	0

0	0	0	0	0	0	0	0	0	0	0	0	0	0	0	0	0	0	0
0	0	0	0	0	0	0	0	0	0	0	0	0	0	0	0	0	0	0
0	0	0	0	0	0	0	0	0	0	0	0	0	0	0	0	0	0	0
0	0	0	0	0	0	0	0	0	0	0	0	0	0	0	0	0	0	0
0	0	0	0	0	0	0	0	0	0	0	0	0	0	0	0	0	0	0
0	0	0	0	0	0	0	0	0	0	0	0	0	0	0	0	0	0	0
0	0	0	0	0	0	0	0	0	0	0	0	0	0	0	0	0	0	0
0	0	0	0	0	0	0	0	0	0	0	0	0	0	0	0	0	0	0
0	0	0	0	0	0	0	0	0	0	0	0	0	0	0	0	0	0	0
0	0	0	0	0	0	0	0	0	0	0	0	0	0	0	0	0	0	0
0	0	0	0	0	0	0	0	0	0	0	0	0	0	0	0	0	0	0
0	0	0	0	0	0	0	0	0	0	0	0	0	0	0	0	0	0	0
0	0	0	0	0	0	0	0	0	0	0	0	0	0	0	0	0	0	0
0	0	0	0	0	0	0	0	0	0	0	0	0	0	0	0	0	0	0
0	0	0	0	0	0	0	0	0	0	0	0	0	0	0	0	0	0	0
0	0	0	0	0	0	0	0	0	0	0	0	0	0	0	0	0	0	0
0	0	0	0	0	0	0	0	0	0	0	0	0	0	0	0	0	0	0
0	0	0	0	0	0	0	0	0	0									
Jan_DL054	1341	1337	971	468	2424	179	220	192	163	340	254	471	309	113	163	415	107	156
152	0	199	61	85	132	139	255	164	59	114	519	115	165	160	127	201	133	390
73	42	66	34	113	130	46	98	84	173	74	140	0	0	79	105	16	56	307
330	44	120	152	41	29	25	46	76	29	83	0	74	0	0	12	211	16	0
46	117	30	31	78	56	38	23	0	84	62	56	52	0	40	0	26	73	0
7	14	0	0	0	133	12	0	0	9	52	0	32	0	35	33	93	0	0
0	0	0	0	36	0	0	0	0	0	68	46	42	65	85	0	0	38	0
0	0	0	31	0	30	150	0	0	18	0	0	16	36	0	0	0	0	0
0	0	0	7	0	0	0	0	0	0	43	0	22	0	162	151	25	0	14
23	0	0	72	0	0	0	0	0	9	48	23	0	0	254	0	0	0	0
36	0	49	0	0	0	0	22	0	37	207	0	0	0	0	0	0	0	0
19	0	43	0	94	67	0	0	0	95	0	0	21	0	0	0	0	24	20
0	0	48	0	0	5	0	0	0	0	0	32	0	10	0	29	0	0	0
30	0	0	9	0	0	0	0	0	25	0	0	6	0	4	19	0	53	0
0	0	0	0	100	19	0	0	0	50	6	0	0	0	14	18	0	0	0
0	0	0	16	0	0	0	0	0	0	0	0	0	0	0	33	11	0	0
0	0	0	0	0	0	0	0	0	28	0	0	0	30	0	0	0	25	0
20	0	12	4	0	0	12	0	6	0	0	11	68	35	0	0	0	0	10
0	0	0	0	6	17	0	21	0	0	0	0	26	43	0	0	0	0	32

12	0	17	0	0	0	0	0	0	0	99	0	0	19	0	22	0	0	28
15	22	0	67	9	0	10	0	0	0	0	11	10	0	19	0	0	0	0
0	0	0	16	0	0	4	0	0	0	0	31	5	0	0	0	0	0	0
0	32	0	0	0	0	9	0	0	0	0	0	0	0	7	0	0	0	0
0	0	0	0	0	0	15	0	0	0	0	0	40	0	0	0	0	0	0
31	16	0	0	0	0	0	0	0	0	0	0	0	20	0	0	0	0	0
0	0	0	0	0	0	0	0	0	0	0	0	0	0	0	0	0	0	0
0	0	0	51	49	0	0	0	0	0	0	0	0	0	0	0	0	4	11
0	0	0	0	0	11	20	0	29	0	0	0	0	0	0	0	0	0	0
0	0	0	0	0	0	0	0	0	16	0	0	0	0	0	40	0	0	0
0	0	21	0	0	0	0	0	0	0	4	0	0	0	0	0	0	0	0
0	0	0	0	0	0	0	0	17	0	0	0	0	0	0	0	0	0	0
0	13	0	0	0	0	12	11	0	0	0	0	0	0	26	0	0	0	0
0	0	0	0	0	0	0	0	0	27	0	0	0	0	0	10	0	0	0
0	0	0	0	13	0	5	0	0	0	0	0	0	0	13	0	0	0	0
0	20	0	0	0	0	0	0	9	0	0	0	0	0	0	0	0	0	0
0	0	0	0	0	0	0	0	0	0	0	0	0	0	0	0	0	0	0
12	0	0	0	0	0	0	0	0	0	0	0	0	0	0	0	0	0	0
0	0	0	0	0	0	0	0	0	0	0	0	0	0	0	0	8	0	0
3	0	0	0	0	0	0	0	0	0	0	0	0	0	0	0	0	0	10
0	0	0	0	0	0	0	0	0	0	0	0	0	0	0	0	0	0	0
0	0	0	11	0	0	0	3	0	7	0	0	0	0	0	0	0	0	0
0	0	0	0	0	0	0	0	0	0	0	0	0	0	0	0	0	0	0
0	0	0	0	0	0	0	0	0	0	0	0	0	0	0	0	0	0	0
0	0	0	0	0	0	0	0	0	0	0	6	0	0	0	0	0	0	0
0	0	0	0	0	0	0	0	0	0	0	0	0	0	0	0	0	0	0
0	0	0	0	0	0	0	0	0	0	5	0	0	0	0	0	0	0	0
0	0	0	0	0	0	0	0	0	4	0	0	0	0	0	0	0	0	0
0	0	0	0	0	0	0	8	0	0	0	0	0	0	0	0	0	0	0
0	0	0	0	0	0	0	0	0	0	4	0	0	0	0	0	0	0	0
0	0	0	0	0	0	0	0	0	0	0	0	0	0	0	0	0	0	0
0	0	0	0	0	0	0	0	0	0	0	0	0	0	0	0	0	0	0
0	0	0	0	0	0	0	0	0	0	0	0	0	0	0	0	0	0	0
0	0	0	0	0	0	0	0	0	0	0	0	0	0	0	0	0	0	0
0	0	0	0	0	0	0	0	0	0	0	0	0	0	0	0	0	0	0
0	0	0	0	0	0	0	0	0	0	0	0	0	0	0	0	0	0	0
0	0	0	0	0	0	0	0	0	0	0	0	0	0	0	0	0	0	0

0	0	0	0	0	0	0	0	0	0	0	0	0	0	0	0	0	0	0
0	0	0	0	0	0	0	0	0	0	0	0	0	0	0	0	0	0	0
0	0	0	0	0	0	0	0	0	0	0	0	0	0	0	0	0	0	0
0	0	0	0	0	0	0	0	0	0	0	0	0	0	0	0	0	0	0
0	0	0	0	0	0	0	0	0	0	0	0	0	0	0	0	0	0	0
0	0	0	0	0	0	0	0	0	0	0	0	14	0	0	0	0	0	0
0	0	0	0	0	0	0	0	0	0	0	0	0	0	0	0	0	0	0
0	0	0	0	0	0	0	0	0	0	0	0	0	0	0	0	0	0	0
0	0	0	0	0	0	0	0	0	0	0	0	0	0	0	0	0	0	0
0	0	0	0	0	0	6	0	0	0	0	0	0	20	0	0	0	0	0
14	0	0	0	0	0	0	0	0	0	0	0	0	0	0	0	0	0	0
0	0	0	0	0	0	0	0	0	0	0	0	0	0	0	0	0	0	0
0	0	0	0	0	0	0	0	0	0	0	0	0	0	0	0	0	0	0
0	0	0	0	0	0	0	0	0	0	0	0	0	0	0	0	0	0	0
0	0	0	0	0	0	0	0	0	0	0	0	0	0	0	0	0	0	0
0	0	0	0	0	0	0	0	0	0	0	0	0	0	0	0	0	0	0
0	0	0	0	0	0	0	0	0	0	0	0	0	0	0	0	0	0	0
0	0	0	0	0	0	0	0	0	0	0	0	0	0	0	0	0	0	0
0	0	0	0	0	0	0	0	0	0	0	0	0	0	0	0	0	0	0
0	0	0	0	0	0	0	0	0	0	0	0	0	0	0	0	0	0	0
0	0	0	0	0	0	0	0	0	0	0	0	0	0	0	0	0	0	0
0	0	0	0	0	0	0	0	0	0	0	0	0	0	0	0	0	0	0
0	0	0	0	0	0	0	0	0	0	0	0	0	0	0	0	0	0	0
0	0	0	0	0	0	0	0	0	0	0	0	0	0	0	0	0	0	0
0	0	0	0	0	0	0	0	0	0	0	0	0	0	0	0	0	0	0
0	0	0	0	0	0	0	0	0	0	0	0	0	0	0	0	0	0	0
0	0	0	0	0	0	0	0	0	0	0	0	0	0	0	0	0	0	0
0	0	0	0	0	0	0	0	0	0	0	0	0	0	0	0	0	0	0
0	0	0	0	0	0	0	0	0	0	0	0	0	0	0	0	0	0	0
0	0	0	0	0	0	0	0	0	0	0	0	0	0	0	0	0	0	0
0	0	0	0	0	0	0	0	0	0	0	0	0	0	0	0	0	0	0
0	0	0	0	0	0	0	0	0	0	0	0	0	0	0	0	0	0	0
0	0	0	0	0	0	0	0	0	0	0	0	0	0	0	0	0	0	0
0	0	0	0	0	0	0	0	0	0	0	0	0	0	0	0	0	0	0
0	0	0	0	0	0	0	0	0	0	0	0	0	0	0	0	0	0	0
0	0	0	0	0	0	0	0	0	0	0	0	0	0	0	0	0	0	0
0	0	0	0	0	0	0	0	0	0	0	0	0	0	0	0	0	0	0

0	0	0	0	0	0	0	0	0	0	0	0	0	0	0	0	0	0	0
0	0	0	0	0	0	0	0	0	0	0	0	0	0	0	0	0	0	0
0	0	0	0	0	0	0	0	0	0	0	0	0	0	0	0	0	0	0
0	0	0	0	0	0	0	0	0	0	0	0	0	0	0	0	0	0	0
0	0	0	0	0	0	0	0	0	0	0	0	0	0	0	0	0	0	0
0	0	0	0	0	0	0	0	0	0	0	0	0	0	0	0	0	0	0
0	0	0	0	0	0	0	0	0	0	0	0	0	0	0	0	0	0	0
0	0	0	0	0	0	0	0	0	0	0	0	0	0	0	0	0	0	0
0	0	0	0	0	0	0	0	0	0	0	0	0	0	0	0	0	0	0
0	0	0	0	0	0	0	0	0	0	0	0	0	0	0	0	0	0	0
0	0	0	0	0	0	0	0	0	0	0	0	0	0	0	0	0	0	0
0	0	0	0	0	0	0	0	0	0	0	0	0	0	0	0	0	0	0
0	0	0	0	0	0	0	0	0	0	0	0	0	0	0	0	0	0	0
0	0	0	0	0	0	0	0	0	0	0	0	0	0	0	0	0	0	0
0	0	0	0	0	0	0	0	0	0	0	0	0	0	0	0	0	0	0
0	0	0	0	0	0	0	0	0	0	0	0	0	0	0	0	0	0	0
0	0	0	0	0	0	0	0	0	0	0	0	0	0	0	0	0	0	0
0	0	0	0	0	0	0	0	0	0	0	0	0	0	0	0	0	0	0
0	0	0	0	0	0	0	0	0	0	0	0	0	0	0	0	0	0	0
0	0	0	0	0	0	0	0	0	0	0	0	0	0	0	0	0	0	0
0	0	0	0	0	0	0	0	0	0	0	0	0	0	0	0	0	0	0
0	0	0	0	0	0	0	0	0	0	0	0	0	0	0	0	0	0	0
0	0	0	0	0	0	0	0	0	0	0	0	0	0	0	0	0	0	0
0	0	0	0	0	0	0	0	0	0	0	0	0	0	0	0	0	0	0
0	0	0	0	0	0	0	0	0	0	0	0	0	0	0	0	0	0	0
0	0	0	0	0	0	0	0	0	0	0	0	0	0	0	0	0	0	0
0	0	0	0	0	0	0	0	0	0	0	0	0	0	0	0	0	0	0
0	0	0	0	0	0	0	0	0	0	0	0	0	0	0	0	0	0	0
0	0	0	0	0	0	0	0	0	0	0	0	0	0	0	0	0	0	0
0	0	0	0	0	0	0	0	0	0	0	0	0	0	0	0	0	0	0
0	0	0	0	0	0	0	0	0	0	0	0	0	0	0	0	0	0	0
0	0	0	0	0	0	0	0	0	0	0	0	0	0	0	0	0	0	0
0	0	0	0	0	0	0	0	0	0	0	0	0	0	0	0	0	0	0
0	0	0	0	0	0	0	0	0	0	0	0	0	0	0	0	0	0	0
0	0	0	0	0	0	0	0	0	0	0	0	0	0	0	0	0	0	0
0	0	0	0	0	0	0	0	0	0	0	0	0	0	0	0	0	0	0
0	0	0	0	0	0	0	0	0	0	0	0	0	0	0	0	0	0	0

0	0	0	0	0	0	0	0	0	0	0	0	0	0	0	0	0	0	0
0	0	0	0	0	0	0	0	0	0	0	0	0	0	0	0	0	0	0
0	0	0	0	0	0	0	0	0	0	0	0	0	0	0	0	0	0	0
0	0	0	0	0	0	0	0	0	0	0	0	0	0	0	0	0	0	0
0	0	0	0	0	0	0	0	0	0	0	0	0	0	0	0	0	0	0
0	0	0	0	0	0	0	0	0	0	0	0	0	0	0	0	0	0	0
0	0	0	0	0	0	0	0	0	0	0	0	0	0	0	0	0	0	0
0	0	0	0	0	0	0	0	0	0	0	0	0	0	0	0	0	0	0
0	0	0	0	0	0	0	0	0	0	0	0	0	0	0	0	0	0	0
0	0	0	0	0	0	0	0	0	0	0	0	0	0	0	0	0	0	0
0	0	0	0	0	0	0	0	0	0	0	0	0	0	0	0	0	0	0
0	0	0	0	0	0	0	0	0	0	0	0	0	0	0	0	0	0	0
0	0	0	0	0	0	0	0	0	0	0	0	0	0	0	0	0	0	0
0	0	0	0	0	0	0	0	0	0	0	0	0	0	0	0	0	0	0
0	0	0	0	0	0	0	0	0	0	0	0	0	0	0	0	0	0	0
0	0	0	0	0	0	0	0	0	0	0	0	0	0	0	0	0	0	0
0	0	0	0	0	0	0	0	0	0	0	0	0	0	0	0	0	0	0
0	0	0	0	0	0	0	0	0	0	0	0	0	0	0	0	0	0	0
0	0	0	0	0	0	0	0	0	0	0	0	0	0	0	0	0	0	0
0	0	0	0	0	0	0	0	0	0	0	0	0	0	0	0	0	0	0
0	0	0	0	0	0	0	0	0	0	0	0	0	0	0	0	0	0	0
0	0	0	0	0	0	0	0	0	0	0	0	0	0	0	0	0	0	0
0	0	0	0	0	0	0	0	0	0	0	0	0	0	0	0	0	0	0
0	0	0	0	0	0	0	0	0	0	0	0	0	0	0	0	0	0	0
0	0	0	0	0	0	0	0	0	0	0	0	0	0	0	0	0	0	0
0	0	0	0	0	0	0	0	0	0	0	0	0	0	0	0	0	0	0
0	0	0	0	0	0	0	0	0	0	0	0	0	0	0	0	0	0	0
0	0	0	0	0	0	0	0	0	0	0	0	0	0	0	0	0	0	0
0	0	0	0	0	0	0	0	0	0	0	0	0	0	0	0	0	0	0
0	0	0	0	0	0	0	0	0	0	0	0	0	0	0	0	0	0	0
0	0	0	0	0	0	0	0	0	0	0	0	0	0	0	0	0	0	0
0	0	0	0	0	0	0	0	0	0	0	0	0	0	0	0	0	0	0
0	0	0	0	0	0	0	0	0	0	0	0	0	0	0	0	0	0	0
0	0	0	0	0	0	0	0	0	0	0	0	0	0	0	0	0	0	0
0	0	0	0	0	0	0	0	0	0	0	0	0	0	0	0	0	0	0
0	0	0	0	0	0	0	0	0	0	0	0	0	0	0	0	0	0	0
0	0	0	0	0	0	0	0	0	0	0	0	0	0	0	0	0	0	0

0	0	0	0	0	0	0	0	0	0	0	0	0	0	0	0	0	0	0
0	0	0	0	0	0	0	0	0	0	0	0	0	0	0	0	0	0	0
0	0	0	0	0	0	0	0	0	0	0	0	0	0	0	0	0	0	0
0	0	0	0	0	0	0	0	0	0	0	0	0	0	0	0	0	0	0
0	0	0	0	0	0	0	0	0	0	0	0	0	0	0	0	2	2	2
0	0	0	0	0	0	0	0	0	0	0	0	0	0	0	0	0	0	0
0	0	0	0	0	0	0	0	0	0	0	0	0	0	0	0	0	0	0
0	0	0	0	0	0	0	0	0	0	0	0	0	0	0	0	0	0	0
0	0	0	0	0	0	0	0	0	0	0	0	0	0	0	0	0	0	0
0	0	0	0	0	0	0	0	0	0									
Jan_DL055	2224	965	212	449	143	147	511	486	115	333	72	995	257	442	261	9	543	242
517	0	59	0	352	49	63	123	47	130	223	435	116	85	122	0	107	45	225
123	0	127	23	88	0	169	0	122	0	123	0	14	261	23	0	180	98	205
196	0	0	38	52	0	143	63	39	52	19	0	0	157	0	28	153	0	0
56	0	0	17	52	50	120	0	74	0	0	88	41	2	46	81	0	31	0
115	100	85	0	26	52	0	0	0	0	173	99	30	28	13	0	80	0	0
0	34	0	0	4	0	0	0	0	0	0	0	31	81	0	7	46	0	0
0	0	0	0	54	0	0	86	0	21	6	0	25	88	0	13	0	0	0
0	7	0	5	0	7	34	0	0	0	0	0	14	0	0	0	0	47	0
10	23	8	0	0	0	0	7	0	10	0	0	0	0	65	0	0	0	40
0	0	0	0	0	0	0	21	0	0	42	0	0	0	0	0	0	0	0
0	48	0	0	32	0	0	0	0	10	0	0	0	0	0	0	0	0	0
0	0	7	0	0	0	0	0	0	0	0	0	0	0	0	22	0	0	0
0	0	0	0	0	0	0	0	0	3	0	0	0	11	7	0	0	0	29
0	29	0	0	0	8	0	0	0	0	0	0	0	0	0	0	14	0	0
0	10	0	0	0	0	0	0	6	0	0	0	0	0	0	0	0	0	0
0	0	0	0	0	0	0	0	0	12	0	0	7	0	0	10	0	0	10
13	29	0	0	0	0	19	7	8	0	0	0	0	0	0	0	0	0	6
0	8	0	0	0	0	0	0	0	0	0	0	0	10	0	0	0	0	0
0	5	0	0	0	0	6	0	0	0	0	0	0	0	0	0	23	0	7
0	0	0	0	0	3	0	0	6	0	0	0	5	0	0	0	0	0	0
0	0	30	0	0	0	0	0	0	0	0	5	0	0	0	0	0	0	0
0	0	0	0	0	0	0	0	0	0	0	0	0	0	0	0	0	0	0
0	0	0	0	0	0	0	0	0	0	0	0	0	0	0	0	0	0	0
0	0	11	0	0	0	0	0	0	0	0	0	0	20	0	0	0	0	0
0	0	0	0	0	0	0	0	0	5	0	0	0	0	0	0	0	0	0
0	0	0	0	0	0	0	0	0	0	0	0	0	0	0	0	12	0	0

0	0	0	0	0	0	0	0	0	0	0	0	0	0	0	0	0	0	0
0	0	0	0	0	0	0	0	0	0	0	0	0	0	0	0	0	0	0
9	0	0	0	6	0	0	0	0	0	0	0	8	0	0	0	0	0	0
0	0	0	0	0	0	0	0	0	0	0	0	0	0	0	0	0	0	11
0	0	0	0	0	0	0	0	0	0	0	0	0	0	0	0	0	0	0
0	0	0	0	0	0	0	0	0	0	0	0	0	0	0	0	5	0	0
0	0	0	0	19	0	0	0	0	0	13	0	0	0	0	0	0	0	0
0	0	0	0	0	0	0	0	0	0	0	0	0	0	0	0	0	0	16
0	0	0	0	0	0	0	0	0	17	0	0	0	0	0	0	0	0	0
0	0	0	0	0	0	0	0	0	0	0	0	3	0	0	0	0	0	0
0	0	0	0	0	0	0	0	0	25	0	0	0	0	0	0	0	0	0
8	0	0	0	0	0	12	0	0	0	0	0	0	0	0	0	0	0	0
0	0	0	0	0	0	0	0	0	0	0	0	0	0	0	0	0	0	0
0	0	0	0	0	0	0	0	0	0	0	0	0	32	0	0	0	0	0
0	2	0	0	0	0	0	0	0	0	0	0	0	0	0	0	0	0	0
0	0	0	0	0	0	0	0	0	0	0	0	0	0	0	0	0	4	0
0	0	0	0	0	0	0	0	0	0	0	0	0	0	0	0	0	0	0
0	0	0	0	0	0	0	0	0	0	0	0	0	0	0	0	0	0	0
0	0	0	0	0	0	0	0	2	0	0	0	0	0	0	0	0	0	0
0	0	0	0	0	0	0	0	0	0	0	0	0	0	0	0	0	0	0
0	0	0	0	0	0	0	0	0	0	0	0	0	0	0	0	0	0	0
0	0	0	0	0	0	0	0	0	0	0	0	0	0	0	0	0	0	0
0	0	0	0	0	0	0	0	0	0	0	0	0	0	0	0	0	0	0
0	0	0	0	0	0	0	0	0	0	0	0	0	0	0	0	0	0	0
0	0	0	0	0	0	0	0	0	0	0	0	0	0	0	0	0	0	0
0	0	0	0	0	0	0	0	0	0	0	0	0	21	0	0	0	0	0
0	0	0	0	0	0	0	0	0	0	0	0	0	0	0	0	0	0	0
0	30	0	0	0	0	0	0	0	0	0	0	0	0	0	0	0	0	0
0	0	0	0	0	0	0	0	0	0	0	0	0	0	0	0	0	0	0
0	0	0	0	0	0	0	0	0	0	0	0	0	0	0	0	0	0	0
0	0	0	0	0	0	0	0	0	0	0	0	0	0	0	0	0	0	0
0	0	0	0	0	0	0	0	0	0	0	0	0	0	0	0	0	0	0
0	0	0	0	0	0	0	0	0	0	0	0	0	0	0	0	0	0	0
0	0	0	0	0	0	0	0	0	0	0	0	0	0	0	0	0	0	0
0	0	0	0	0	0	0	0	0	0	0	0	0	0	0	0	0	0	0
0	0	0	0	0	0	0	0	0	0	0	0	0	0	0	0	0	0	0
0	0	0	0	0	0	0	0	0	0	0	0	0	0	0	0	0	0	0

0	0	0	0	0	0	0	0	0	0	0	0	0	0	0	0	0	0	0
0	0	0	0	0	0	0	0	0	0	0	0	0	0	0	0	0	0	0
0	0	0	0	0	0	0	0	0	0	0	0	0	0	0	0	0	0	0
0	0	0	0	0	0	0	0	0	0	0	0	0	0	0	0	0	0	0
0	0	0	0	0	0	0	0	0	0	0	0	0	0	0	0	0	0	0
0	0	0	0	0	0	0	0	0	0	0	0	0	0	0	0	0	0	0
0	0	0	0	0	0	0	0	0	0	0	0	0	0	0	0	0	0	0
0	0	0	0	0	0	0	0	0	0	0	0	0	0	0	0	0	0	0
0	0	0	0	0	0	0	0	0	0	0	0	0	0	0	0	0	0	0
0	0	0	0	0	0	0	0	0	0	0	0	0	0	0	0	0	0	0
0	0	0	0	0	0	0	0	0	0	0	0	0	0	0	0	0	0	0
0	0	0	0	0	0	0	0	0	0	0	0	0	0	0	0	0	0	0
0	0	0	0	0	0	0	0	0	0	0	0	0	0	0	0	0	0	0
0	0	0	7	0	0	0	0	0	0	0	0	0	0	0	0	0	0	0
0	0	0	0	0	0	0	0	0	0	0	0	0	0	0	0	0	0	0
0	4	0	0	0	0	0	0	0	0	0	0	0	0	0	0	0	0	0
0	0	0	0	0	0	0	0	0	0	0	0	0	0	0	0	0	0	0
0	0	0	0	0	0	0	0	0	0	0	0	0	0	0	0	0	0	0
0	0	0	0	0	0	0	0	0	0	0	0	0	0	0	0	0	0	0
0	0	0	0	0	0	0	0	0	0	0	0	0	0	0	0	0	0	0
0	0	0	0	0	0	0	0	0	0	0	0	0	0	0	0	0	0	0
0	0	0	0	0	0	0	0	0	0	0	0	0	0	0	0	0	0	0
0	0	0	0	0	0	0	0	0	0	0	0	0	0	0	0	0	0	0
0	0	0	0	0	0	0	0	0	0	0	0	0	0	0	0	0	0	0
0	0	0	0	0	0	0	0	0	0	0	0	0	0	0	0	0	0	0
0	0	0	0	0	0	0	0	0	0	0	0	0	0	0	0	0	0	0
0	0	0	0	0	0	0	0	0	0	7	0	0	0	0	0	0	0	0
0	0	0	0	0	0	0	0	0	0	0	0	0	0	0	0	0	0	0
0	0	0	0	0	0	0	0	0	0	0	0	0	0	0	0	0	0	0
0	0	0	0	0	0	0	0	0	0	0	0	0	0	0	0	0	0	0
0	0	0	0	0	0	0	0	0	0	0	0	0	0	0	0	0	0	0
0	0	0	0	0	0	0	0	0	0	0	0	0	0	0	0	0	0	0
0	0	0	0	0	0	0	0	0	0	0	0	0	0	0	0	0	0	0
0	0	0	0	0	0	6	0	0	0	0	0	0	0	0	0	0	0	0
0	0	0	0	0	0	0	0	0	0	0	0	0	0	0	0	0	0	0
0	0	0	0	0	0	0	0	0	0	0	0	0	0	0	0	0	0	0
0	0	0	0	0	0	0	0	0	0	0	0	0	0	0	0	0	0	0

0	0	0	0	0	0	0	0	0	0	0	0	0	0	0	0	0	0	0
0	0	0	0	0	0	0	0	0	0	0	0	0	0	0	0	0	0	0
0	0	0	0	0	0	0	0	0	0	0	0	0	0	0	0	0	0	0
0	0	0	0	0	0	0	0	0	0	0	0	0	0	0	0	0	0	0
0	0	0	0	0	0	0	0	0	0	0	0	0	0	0	0	0	0	0
0	0	0	0	0	0	0	0	0	0	0	0	0	0	0	0	0	0	0
0	0	0	0	0	0	0	0	0	0	0	0	0	0	0	0	0	0	0
0	0	0	0	0	0	0	0	0	0	0	0	0	0	0	0	0	0	0
0	0	0	0	0	0	0	0	0	0	0	0	0	0	0	0	0	0	0
0	0	0	0	0	0	0	0	0	0	0	0	0	0	0	0	0	0	0
0	0	0	0	0	0	0	0	0	0	0	0	0	0	0	0	0	0	0
0	0	0	0	0	0	0	0	0	0	0	0	0	0	0	0	0	0	0
0	0	0	0	0	0	0	0	0	0	0	0	0	0	0	0	0	0	0
0	0	0	0	0	0	0	0	0	0	0	0	0	0	0	0	0	0	0
0	0	0	0	0	0	0	0	0	0	0	0	0	0	0	0	0	0	0
0	0	0	0	0	0	0	0	0	0	0	0	0	0	0	0	0	0	0
0	0	0	0	0	0	0	0	0	0	0	0	0	0	0	0	0	0	0
0	0	0	0	0	0	0	0	0	0	0	0	0	0	0	0	0	0	0
0	0	0	0	0	0	0	0	0	0	0	0	0	0	0	0	0	0	0
0	0	0	0	0	0	0	0	0	0	0	0	0	0	0	0	0	0	0
0	0	0	0	0	0	0	0	0	0	0	0	0	0	0	0	0	0	0
0	0	0	0	0	0	0	0	0	0	0	0	0	0	0	0	0	0	0
0	0	0	0	0	0	0	0	0	0	0	0	0	0	0	0	0	0	0
0	0	0	0	0	0	0	0	0	0	0	0	0	0	0	0	0	0	0
0	0	0	0	0	0	0	0	0	0	0	0	0	0	0	0	0	0	0
0	0	0	0	0	0	0	0	0	0	0	0	0	0	0	0	0	0	0
0	0	0	0	0	0	0	0	0	0	0	0	0	0	0	0	0	0	0
0	0	0	0	0	0	0	0	0	0	0	0	0	0	0	0	0	0	0
0	0	0	0	0	0	0	0	0	0	0	0	0	0	0	0	0	0	0
0	0	0	0	0	0	0	0	0	0	0	0	0	0	0	0	0	0	0
0	0	0	0	0	0	0	0	0	0	0	0	0	0	0	0	0	0	0
0	0	0	0	0	0	0	0	0	0	0	3	0	0	0	0	0	0	0
0	0	0	0	0	0	0	0	0	0	0	0	0	0	0	0	0	0	0
0	0	0	0	0	0	0	0	0	0	0	0	0	0	0	0	0	0	0
0	0	0	0	0	0	0	0	0	0	0	0	0	0	0	0	0	0	0
0	0	0	0	0	0	0	0	0	0	0	0	0	0	0	0	0	0	0
0	0	0	0	0	0	0	0	0	0	0	0	0	0	0	0	0	0	0

0	0	0	0	0	0	0	0	0	0	0	0	0	0	0	0	0	0	0
0	0	0	0	0	0	0	0	0	0	0	0	0	0	0	0	0	0	0
0	0	0	0	0	0	0	0	0	0	0	0	0	0	0	0	0	0	0
0	0	0	0	0	0	0	0	0	0	0	0	0	0	0	0	0	0	0
0	0	0	0	0	0	0	0	0	0	0	0	0	0	0	0	0	0	0
0	0	0	0	0	0	0	0	0	0	0	0	0	0	0	0	0	0	0
0	0	0	0	0	0	0	0	0	0	0	0	0	0	0	0	0	0	0
0	0	0	0	0	0	0	0	0	0	0	0	0	0	0	0	0	0	0
0	0	0	0	0	0	0	0	0	0	0	0	0	0	0	0	0	0	0
0	0	0	0	0	0	0	0	0	0	0	0	0	0	0	0	0	0	0
0	0	0	0	0	0	0	0	0	0	0	0	0	0	0	0	0	0	0
0	0	0	0	0	0	0	0	0	0	0	0	0	0	0	0	0	0	0
0	0	0	0	0	0	0	0	0	0	0	0	0	0	0	0	0	0	0
0	0	0	0	0	0	0	0	0	0	0	0	0	0	0	0	0	0	0
0	0	0	0	0	0	0	0	0	0	0	0	0	0	0	0	0	0	0
0	0	0	0	0	0	0	0	0	0	0	0	0	0	0	0	0	0	0
0	0	0	0	0	0	0	0	0	0	0	0	0	0	0	0	0	0	0
0	0	0	0	0	0	0	0	0	0	0	0	0	0	0	0	0	0	0
0	0	0	0	0	0	0	0	0	0	0	0	0	0	0	0	0	0	0
0	0	0	0	0	0	0	0	0	0	0	0	0	0	0	0	0	0	0
0	0	0	0	0	0	0	0	0	0	0	0	0	0	0	0	0	0	0
0	0	0	0	0	0	0	0	0	0	0	0	0	0	0	0	0	0	0
0	0	0	0	0	2	2	2	0	0	0	0	0	0	0	0	0	0	0
0	0	0	0	0	0	0	0	0	0	0	0	0	0	0	0	0	0	0
0	0	0	0	0	0	0	0	0	0	0	0	0	0	0	0	0	0	0
0	0	0	0	0	0	0	0	0	0	0	0	0	0	0	0	0	0	0
0	0	0	0	0	0	0	0	0	0	0	0	0	0	0	0	0	0	0
0	0	0	0	0	0	0	0	0	0	0	0	0	0	0	0	0	0	0
0	0	0	0	0	0	0	0	0	0	0	0	0	0	0	0	0	0	0
0	0	0	0	0	0	0	0	0	0	0	0	0	0	0	0	0	0	0
0	0	0	0	0	0	0	0	0	0	0	0	0	0	0	0	0	0	0
0	0	0	0	0	0	0	0	0	0	0	0	0	0	0	0	0	0	0
0	0	0	0	0	0	0	0	0	0	0	0	0	0	0	0	0	0	0
0	0	0	0	0	0	0	0	0	0	0	0	0	0	0	0	0	0	0
0	0	0	0	0	0	0	0	0	0	0	0	0	0	0	0	0	0	0
0	0	0	0	0	0	0	0	0	0	0	0	0	0	0	0	0	0	0
0	0	0	0	0	0	0	0	0	0	0	0	0	0	0	0	0	0	0

0	0	0	0	0	0	0	0	0	0	0	0	0	0	0	0	0	0	0
0	0	0	0	0	0	0	0	0	0									
Jan_DL057	1425	425	535	752	102	64	94	85	207	285	442	531	235	63	237	447	49	273
144	39	68	23	84	66	174	147	243	184	168	175	84	108	120	208	112	278	116
156	79	74	11	65	164	88	434	82	182	0	195	104	0	19	184	203	0	98
94	191	149	91	0	19	32	79	74	26	27	0	115	0	0	0	0	33	18
78	45	16	75	27	76	110	14	60	22	0	57	16	0	31	116	0	22	88
44	0	0	0	0	11	23	0	0	0	0	0	0	0	13	16	0	41	0
0	0	0	5	15	21	0	0	0	0	0	25	0	18	29	0	0	0	0
0	0	0	0	17	0	0	0	0	0	0	0	4	0	0	0	0	0	0
0	17	0	0	0	7	41	0	0	0	7	0	0	0	0	0	0	0	23
27	70	0	0	0	0	12	0	0	0	14	0	0	0	0	0	10	0	0
0	0	10	10	0	0	49	0	0	5	0	0	0	0	6	0	0	0	0
0	0	12	0	0	0	0	0	0	0	0	0	0	0	0	0	0	0	9
91	0	0	0	0	0	0	0	3	0	0	0	11	0	0	0	0	0	0
0	0	0	0	0	0	0	0	0	11	0	0	0	0	0	7	0	15	0
0	0	0	0	0	2	0	0	0	0	5	0	0	0	2	0	0	0	0
0	0	0	0	0	0	0	0	0	0	0	0	0	0	4	0	0	0	0
0	0	0	0	0	0	0	0	0	0	0	0	0	0	0	0	0	0	0
0	0	0	0	0	0	0	6	0	0	0	0	0	0	0	0	22	0	0
0	0	0	0	4	0	0	0	0	0	0	6	0	0	0	0	0	0	5
0	0	0	0	0	0	0	2	0	0	0	0	0	0	0	0	0	0	0
0	0	0	0	0	0	0	0	0	0	0	0	0	0	8	0	0	0	0
0	0	0	0	0	0	0	0	0	0	0	0	0	0	0	0	0	0	0
0	0	0	0	0	0	0	0	7	11	0	0	0	0	0	0	2	0	0
0	0	0	0	0	0	0	0	0	0	0	0	0	0	0	0	0	0	0
0	0	0	0	0	0	0	10	0	0	0	0	0	0	0	0	0	0	0
0	0	0	0	4	0	0	0	0	0	0	0	0	0	0	0	0	0	0
0	0	6	0	0	0	0	0	0	0	0	0	0	0	0	0	0	2	0
0	0	0	0	0	0	0	0	0	0	0	0	0	0	0	0	0	0	0
0	0	0	0	0	0	6	0	0	0	0	0	0	0	0	0	0	0	0
0	0	0	0	0	0	0	0	0	0	3	0	0	0	0	0	0	0	0
0	0	0	0	40	0	0	0	0	0	0	0	0	0	0	0	0	0	0
0	0	0	0	0	0	0	0	0	0	0	0	0	0	0	0	0	0	0
0	0	0	0	0	0	0	0	0	0	0	0	0	0	0	0	0	7	0
0	0	0	0	0	0	0	0	0	0	0	0	0	0	0	0	0	0	0
0	0	0	0	0	0	0	0	0	0	0	0	0	0	0	0	0	0	0

0	0	0	0	0	0	0	0	0	0	0	0	0	0	0	0	0	0	0
0	0	0	0	0	0	0	0	0	0	0	0	0	0	0	0	0	0	0
0	0	0	0	0	0	0	0	0	0	0	0	0	0	0	0	0	0	0
0	0	0	0	0	0	0	0	0	0	0	0	0	0	0	0	0	0	0
0	0	0	0	0	0	0	0	0	0	0	0	0	0	0	0	0	0	0
0	0	0	0	0	0	0	0	0	0	0	0	0	0	0	0	0	0	0
0	0	0	0	0	0	0	0	0	0	0	0	0	0	0	0	0	0	0
0	0	0	0	0	0	0	0	0	0	0	0	0	0	0	0	0	0	0
0	0	0	0	0	0	0	0	0	0	0	0	0	5	0	0	0	0	0
0	0	0	0	0	0	0	0	0	0	0	0	0	0	0	0	0	0	0
0	0	0	0	0	0	0	0	0	0	0	0	0	0	0	0	0	0	0
0	0	0	0	0	0	0	0	0	0	0	0	0	0	0	0	0	0	0
0	0	0	0	0	0	0	0	0	0	0	0	0	0	0	0	0	0	0
0	0	0	0	0	0	0	0	0	0	0	0	0	0	0	0	0	0	0
0	0	0	0	0	0	0	0	0	0	0	0	0	0	0	0	0	0	0
0	0	0	0	0	0	0	0	0	0	0	0	0	0	0	0	0	0	0
0	0	0	0	0	0	0	0	0	0	0	0	0	0	0	0	0	0	0
0	0	0	0	0	0	0	0	0	0	0	0	0	0	0	0	0	0	0
0	0	0	0	0	0	0	0	0	0	0	0	0	0	0	0	0	0	0
0	0	0	0	0	0	0	0	0	0	0	0	0	0	0	0	0	4	0
0	0	0	0	0	0	0	0	0	0	0	0	0	0	0	0	0	0	0
0	0	0	0	0	0	0	0	0	0	0	0	0	0	0	0	0	0	0
0	0	0	0	0	0	0	0	0	0	0	0	0	0	0	0	0	0	0
0	0	0	0	0	0	0	0	0	0	0	0	0	0	0	0	0	0	0
0	0	0	0	0	0	0	0	0	0	0	0	0	0	0	0	0	0	0
0	0	13	0	0	0	0	0	0	0	0	0	0	0	0	0	0	0	0
0	0	0	0	0	4	0	16	0	0	0	0	0	0	0	0	0	0	0
0	0	0	0	0	0	0	0	0	0	0	0	0	0	0	0	0	0	0
0	0	0	0	0	0	0	0	0	0	0	0	0	0	0	0	0	0	0
0	0	0	0	0	0	0	0	0	0	0	0	0	0	0	0	0	0	0
0	0	0	0	0	0	0	0	0	0	0	0	0	0	0	0	0	0	0
0	0	0	0	0	0	0	0	0	0	0	0	0	0	0	0	0	0	0
0	0	0	0	0	0	0	0	0	0	0	0	12	0	0	0	0	0	0
0	0	0	0	0	0	0	0	0	0	0	0	0	0	0	0	0	0	0
0	0	0	0	0	0	0	0	0	0	0	0	0	0	0	0	0	0	0
0	0	0	0	0	0	0	0	0	0	0	0	0	0	0	0	0	0	0
0	0	0	0	0	0	0	0	0	0	0	0	0	0	0	0	0	0	0

0	0	0	0	0	0	0	0	0	0	0	0	0	0	0	0	0	0	0
0	0	0	0	0	0	0	0	0	0	0	0	0	0	0	0	0	0	0
0	0	0	0	0	0	0	0	0	0	0	0	0	0	0	0	0	0	0
0	0	0	0	2	0	0	0	0	0	0	0	0	0	0	0	0	0	0
0	0	0	0	0	0	0	0	0	0	0	0	0	0	0	0	0	0	0
0	0	0	0	0	0	0	0	0	0	0	0	0	0	0	0	0	0	0
0	0	0	0	0	0	0	0	0	0	0	0	0	0	0	0	0	0	0
0	0	0	0	0	0	0	0	0	0	0	0	0	0	0	0	0	0	0
0	0	0	0	0	0	0	0	0	0	0	0	0	0	0	0	0	0	0
0	0	0	0	0	0	0	0	0	0	0	0	0	0	0	0	0	0	0
0	0	0	0	0	0	0	0	0	0	0	0	0	0	0	0	0	0	0
0	0	0	0	0	0	0	0	0	0	0	0	0	0	0	0	0	0	0
0	0	0	0	0	0	0	0	0	0	0	0	0	0	0	0	0	0	0
0	0	0	0	0	0	0	0	0	0	0	0	0	0	0	0	0	0	0
0	0	0	0	0	0	0	0	0	0	0	0	0	0	0	0	0	0	0
0	0	0	0	0	0	0	0	0	0	0	0	0	0	0	0	0	0	0
0	0	0	0	0	0	0	0	0	0	0	0	0	0	0	0	0	0	0
0	0	0	0	0	0	0	0	0	0	0	0	0	0	0	0	0	0	0
0	0	0	0	0	0	0	0	0	0	0	0	0	0	0	0	0	0	0
0	0	0	0	0	0	0	0	0	0	0	0	0	0	0	0	0	0	0
0	0	0	0	0	0	0	0	0	0	0	0	0	0	0	0	0	0	0
0	0	0	0	0	0	0	0	0	0	0	0	0	0	0	0	0	0	0
0	0	0	0	0	0	0	0	0	0	0	0	0	0	0	0	0	0	0
0	0	0	0	0	0	0	0	0	0	0	0	0	0	0	0	0	0	0
0	0	0	0	0	0	0	0	0	0	5	0	0	0	0	0	0	0	0
0	0	0	0	0	0	0	0	0	0	0	0	0	0	0	0	0	0	0
0	0	0	0	0	0	0	0	0	0	0	0	0	0	0	0	0	0	0
0	0	0	0	0	0	0	0	0	0	0	0	0	0	0	0	0	0	0
0	0	0	0	0	0	0	0	0	0	0	0	0	0	0	0	0	0	0
0	0	0	0	0	0	0	0	0	0	0	0	0	0	0	0	0	0	0
0	0	0	0	0	0	0	0	0	0	0	0	0	0	0	0	0	0	0
0	0	0	0	0	0	0	0	0	0	0	0	0	0	0	0	0	0	0
0	0	0	0	0	0	0	0	0	0	0	0	0	0	0	0	0	0	0
0	0	0	0	0	0	0	0	0	0	0	0	0	0	0	0	0	0	0
0	0	0	0	0	0	0	0	0	0	4	4	4	0	0	0	0	0	0
0	0	0	0	0	0	0	0	0	0	0	0	0	0	0	0	0	0	0
0	0	0	0	0	0	0	0	0	0	0	0	0	0	0	0	0	0	0

0	0	0	0	0	0	0	0	0	0	0	0	0	0	0	0	0	0	0
0	0	0	0	0	0	0	0	0	0	0	0	0	0	0	0	0	0	0
0	0	0	0	0	0	0	0	0	0	0	0	0	0	0	0	0	0	0
0	0	0	0	0	0	0	0	0	0	0	0	0	0	0	0	0	0	0
0	0	0	0	0	0	0	0	0	0	0	0	0	0	0	0	0	0	0
0	0	0	0	0	0	0	0	0	0	0	0	0	0	0	0	0	0	0
0	0	0	0	0	0	0	0	0	0	0	0	0	0	0	0	0	0	0
0	0	0	0	0	0	0	0	0	0	0	0	0	0	0	0	0	0	0
0	0	0	0	0	0	0	0	0	0	0	0	0	0	0	0	0	0	0
0	0	0	0	0	0	0	0	0	0	0	0	0	0	0	0	0	0	0
0	0	0	0	0	0	0	0	0	0	0	0	0	0	0	0	0	0	0
0	0	0	0	0	0	0	0	0	0	3	3	3	0	0	0	0	0	0
0	0	0	0	0	0	0	0	0	0	0	0	0	0	0	0	0	0	0
0	0	0	0	0	0	0	0	0	0	0	0	0	0	0	0	0	0	0
0	0	0	0	0	0	0	0	0	0	0	0	0	0	0	0	0	0	0
0	0	0	0	0	0	0	0	0	0	0	0	0	0	0	0	0	0	0
0	0	0	0	0	0	0	0	0	0	0	0	0	0	0	0	0	0	0
0	0	0	0	0	0	0	0	0	0	0	0	0	0	0	0	0	0	0
0	0	0	0	0	0	0	0	0	0	0	0	0	0	0	0	0	0	0
0	0	0	0	0	0	0	0	0	0	0	0	0	0	0	0	0	0	0
0	0	0	0	0	0	0	0	0	0	0	0	0	0	0	0	0	0	0
0	0	0	0	0	0	0	0	0	0	0	0	0	0	0	0	0	0	0
0	0	0	0	0	0	0	0	0	0	0	0	0	0	0	0	0	0	0
0	0	0	0	0	0	0	0	0	0	0	0	0	0	0	0	0	0	0
0	0	0	0	0	0	0	0	0	0	0	0	0	0	0	0	0	0	0
0	0	0	0	0	0	0	0	0	0	0	0	0	0	0	0	0	0	0
0	0	0	0	0	0	0	0	0	0	0	0	0	0	0	0	0	0	0
0	0	0	0	0	0	0	0	0	0	0	0	0	0	0	0	0	0	0
0	0	0	0	0	0	0	0	0	0	0	0	0	0	0	0	0	0	0
0	0	0	0	0	0	0	0	0	0	0	0	0	0	0	0	0	0	0
0	0	0	0	0	0	0	0	0	0	0	0	0	0	0	0	0	0	0
0	0	0	0	0	0	0	0	0	0	0	0	0	0	0	0	0	0	0
0	0	0	0	0	0	0	0	0	0	0	0	0	0	0	0	0	0	0
0	0	0	0	0	0	0	0	0	0	0	0	0	0	0	0	0	0	0
0	0	0	0	0	0	0	0	0	0	0	0	0	0	0	0	0	0	0
0	0	0	0	0	0	0	0	0	0	0	0	0	0	0	0	0	0	0
0	0	0	0	0	0	0	0	0	0	0	0	0	0	0	0	0	0	0
0	0	0	0	0	0	0	0	0	0	0	0	0	0	0	0	0	0	0

0	0	0	0	0	0	0	2	2	2	2	2	2	0	0	0	0	0	0
0	0	0	0	0	0	0	0	0	0	0	0	0	0	0	0	0	0	0
0	0	0	0	0	0	0	0	0	0	0	0	0	0	0	0	0	0	0
0	0	0	0	0	0	0	0	0	0	0	0	0	0	0	0	0	0	0
0	0	0	0	0	0	0	0	0	0	0	0	0	0	0	0	0	0	0
0	0	0	0	0	0	0	0	0	0	0	0	0	0	0	0	0	0	0
0	0	0	0	0	0	0	0	0	0	0	0	0	0	0	0	0	0	0
0	0	0	0	0	0	0	0	0	0	0	0	0	0	0	0	0	0	0
0	0	0	0	0	0	0	0	0	0	0	0	0	0	0	0	0	0	0
0	0	0	0	0	0	0	0	0	0	0	0	0	0	0	0	0	0	0
0	0	0	0	0	0	0	0	0	0	0	0	0	0	0	0	0	0	0
0	0	0	0	0	0	0	0	0	0	0	0	0	0	0	0	0	0	0
0	0	0	0	0	0	0	0	0	0	0	0	0	0	0	0	0	0	0
0	0	0	0	0	0	0	0	0	0	0	0	0	0	0	0	0	0	0
0	0	0	0	0	0	0	0	0	0	0	0	0	0	0	0	0	0	0
0	0	0	0	0	0	0	0	0	0	0	0	0	0	0	0	0	0	0
0	0	0	0	0	0	0	0	0	0	0	0	0	0	0	0	0	0	0
0	0	0	0	0	0	0	0	0	0	0	0	0	0	0	0	0	0	0
0	0	0	0	0	0	0	0	0	0	0	0	0	0	0	0	0	0	0
0	0	0	0	0	0	0	0	0	0	0	0	0	0	0	0	0	0	0
0	0	0	0	0	0	0	0	0	0	0	0	0	0	0	0	0	0	0
0	0	0	0	0	0	0	0	0	0	0	0	0	0	0	0	0	0	0
0	0	0	0	0	0	0	0	0	0	0	0	0	0	0	0	0	0	0
0	0	0	0	0	0	0	0	0	0	0	0	0	0	0	0	0	0	0
0	0	0	0	0	0	0	0	0	0	0	0	0	0	0	0	0	0	0
0	0	0	0	0	0	0	0	0	0	0	0	0	0	0	0	0	0	0
0	0	0	0	0	0	0	0	0	0	0	0	0	0	0	0	0	0	0
0	0	0	0	0	0	0	0	0	0	0	0	0	0	0	0	0	0	0
0	0	0	0	0	0	0	0	0	0	0	0	0	0	0	0	0	0	0
0	0	0	0	0	0	0	0	0	1	0	0	0	0	0	0	0	0	0
0	0	0	0	0	0	0	0	0	0									
Jan_DL058	1308	1537	33	604	149	472	392	335	537	140	464	968	124	646	208	23	621	317
812	10	182	187	243	186	240	218	392	61	231	0	254	203	57	236	145	52	0
0	52	283	172	107	175	318	143	196	11	390	0	0	401	204	25	184	224	0
0	0	0	47	324	42	110	45	123	51	21	152	6	91	46	17	104	80	0
123	0	101	0	0	132	137	80	61	36	0	49	43	0	36	116	41	9	0
43	17	133	0	0	0	0	53	120	17	0	52	0	0	26	40	0	0	0

86	75	0	32	0	0	0	0	0	50	0	26	12	11	80	15	0	0	0
0	0	127	0	0	0	0	49	0	13	13	0	0	0	20	0	0	0	0
0	11	0	0	0	0	47	24	0	0	18	0	9	0	0	0	0	0	29
0	0	8	61	0	0	58	0	0	0	0	6	0	31	12	13	0	0	0
0	0	13	30	37	46	0	0	0	5	0	0	0	0	19	0	0	0	0
0	36	14	0	0	0	0	0	0	0	0	0	0	0	0	0	0	4	0
81	0	0	19	0	0	0	19	0	0	0	0	0	3	0	11	6	0	0
11	0	0	0	0	0	0	0	0	0	0	0	0	0	0	0	0	10	0
0	0	0	0	0	0	0	0	12	29	22	3	0	0	7	0	0	0	0
0	0	0	0	0	0	0	0	0	0	0	0	0	0	3	0	0	0	14
0	12	11	0	0	0	0	0	0	18	9	0	16	0	5	0	0	5	6
0	0	0	0	0	0	0	0	2	0	0	0	0	0	0	7	0	0	0
11	9	0	0	0	0	0	0	0	0	0	0	0	11	0	0	0	0	0
0	10	0	0	0	14	6	0	0	0	0	0	0	0	0	0	0	0	0
0	0	0	0	0	0	0	0	0	0	0	0	0	0	0	0	0	4	0
0	0	0	0	0	6	0	6	0	0	0	0	0	4	0	17	33	0	0
0	0	0	0	0	0	0	0	5	0	0	0	0	0	0	0	0	0	0
0	0	0	17	0	0	0	0	0	0	26	0	0	0	0	0	0	0	7
0	0	0	0	0	0	0	0	0	0	0	0	0	0	0	0	0	0	0
28	0	0	0	0	0	0	5	0	0	0	0	0	17	0	30	0	0	0
0	0	0	0	0	0	0	0	0	0	0	0	0	0	0	0	0	0	0
0	0	0	0	0	0	0	0	0	0	0	0	0	0	0	0	0	0	0
0	0	0	0	0	0	0	0	0	0	0	0	0	0	0	0	0	0	0
12	0	0	35	7	0	0	0	0	0	7	0	0	0	0	0	4	0	0
0	0	0	0	0	0	0	0	0	0	33	0	0	0	3	0	0	0	0
0	0	0	0	0	0	0	0	0	7	0	0	0	0	0	0	0	0	0
0	0	0	0	0	0	0	0	0	0	0	0	0	0	0	0	0	0	0
0	0	0	0	0	0	0	0	0	0	0	0	0	0	0	0	0	0	0
0	0	0	0	0	0	0	0	0	0	0	0	3	0	0	0	5	0	0
0	0	0	0	0	0	0	0	0	0	0	0	0	0	0	0	0	0	0
0	0	0	0	0	44	0	0	0	0	0	0	0	0	0	0	0	0	0
0	0	0	0	0	0	0	0	0	0	0	0	0	0	0	0	0	0	0
0	0	0	0	0	0	0	0	0	0	0	0	0	0	0	0	0	0	0
0	0	0	0	0	0	0	20	0	0	0	0	0	0	0	0	0	0	0
0	0	0	0	0	0	0	0	0	0	0	0	0	0	0	0	0	0	0
0	0	0	0	0	0	0	2	0	0	0	0	0	0	0	0	0	0	0
0	0	0	0	0	0	0	0	7	0	0	0	0	0	0	0	0	0	0

0	0	0	0	13	0	0	0	0	0	0	0	0	0	0	0	0	0	0
0	0	0	0	0	0	0	0	0	0	0	0	5	0	0	0	0	0	0
0	0	0	0	0	0	0	0	0	0	0	0	0	0	0	0	0	0	0
0	0	0	0	0	0	0	0	0	0	0	0	0	0	0	0	0	0	0
0	0	0	0	0	0	0	0	0	0	0	0	0	0	0	0	0	0	0
0	0	0	0	0	0	0	14	0	0	0	0	0	7	0	0	0	0	0
0	0	0	0	0	0	0	0	0	0	0	0	0	0	0	0	0	0	0
0	0	0	0	0	0	0	0	0	0	0	0	0	0	0	0	0	0	0
0	0	7	0	0	0	0	0	0	0	0	0	0	0	0	0	0	0	0
0	0	0	0	0	0	0	0	0	0	0	0	0	0	0	0	0	0	0
0	0	0	0	0	0	0	0	0	0	0	0	0	0	0	0	0	0	0
0	0	0	0	0	0	0	0	0	0	0	0	0	0	0	0	0	0	0
0	0	0	0	0	0	0	0	0	0	0	0	0	0	0	0	0	0	0
0	0	0	0	0	0	0	0	0	0	0	0	0	0	0	0	0	0	0
0	0	0	0	0	0	0	0	0	0	0	0	0	0	0	0	0	0	0
0	0	0	0	0	0	0	0	0	0	0	0	0	0	0	0	0	0	0
0	0	0	0	0	0	0	0	0	0	0	0	0	0	0	0	0	0	0
0	0	0	0	0	0	0	0	0	0	0	0	0	0	0	0	0	0	0
0	0	0	0	0	0	0	0	0	0	0	0	0	0	0	0	0	0	0
0	0	0	0	0	0	0	0	0	0	0	0	0	0	0	0	0	0	0
0	0	0	0	0	0	0	0	0	0	0	0	0	0	0	0	0	0	0
0	0	0	0	0	0	0	0	0	0	0	0	0	0	0	0	0	0	0
0	0	0	0	0	0	0	0	0	0	0	0	0	0	0	0	0	0	0
0	0	0	0	0	0	0	0	0	0	0	0	0	0	0	0	0	0	0
0	0	0	0	0	0	0	0	0	0	0	0	0	0	0	0	0	0	0
0	0	0	0	0	0	0	0	0	0	0	0	0	0	0	0	0	0	0
0	0	0	0	0	0	0	0	0	0	0	0	0	0	0	0	0	0	0
0	0	0	0	0	0	0	0	0	0	0	0	0	0	0	0	0	0	0
0	0	0	0	0	0	0	0	0	0	0	0	0	0	0	0	0	0	0
0	0	0	0	0	0	0	0	0	0	0	0	0	0	0	0	0	0	0
0	0	0	0	0	0	0	0	0	0	0	0	0	0	0	0	0	0	0
0	0	0	0	0	0	0	0	0	0	0	0	0	0	0	0	0	0	0
0	0	0	0	0	0	0	0	0	0	0	0	0	0	0	0	0	0	0
0	0	0	0	0	0	0	0	0	0	0	0	0	0	0	0	0	0	0
0	0	0	0	0	0	0	0	0	0	0	0	0	0	0	0	0	0	0
0	0	0	0	0	0	0	0	0	0	0	0	0	0	0	0	0	0	0
0	0	0	0	0	0	0	0	0	0	0	0	0	0	0	0	0	0	0

0	0	0	0	0	0	0	0	0	0	0	0	0	0	0	0	0	0	0
0	0	0	0	0	0	0	0	0	0	0	0	0	0	0	0	0	0	0
0	0	0	0	0	0	0	0	0	0	0	0	0	0	0	0	0	0	0
0	0	0	0	0	0	0	0	0	0	0	0	0	0	0	0	0	0	0
0	0	0	0	0	0	0	0	0	0	0	0	0	0	0	0	0	0	0
0	0	0	0	0	0	0	0	0	0	0	0	0	0	0	0	0	0	0
0	0	0	0	0	0	0	0	0	0	0	0	0	0	0	0	0	0	0
0	0	0	0	0	0	0	0	0	0	0	0	0	0	0	0	0	0	0
0	0	0	0	0	0	0	0	0	0	0	0	0	0	0	0	0	0	0
0	0	0	0	0	0	0	0	0	0	0	0	0	0	0	0	0	0	0
0	0	0	0	0	0	0	0	0	0	0	0	0	0	0	0	0	0	0
0	0	0	0	0	0	0	0	0	0	0	0	0	0	0	0	0	0	0
0	0	0	0	0	0	0	0	0	0	0	0	0	2	0	0	0	0	0
0	0	0	0	0	0	0	0	0	0	0	0	0	0	0	0	0	0	0
0	0	0	0	0	0	0	0	0	0	0	0	0	0	0	0	0	0	0
0	0	0	0	0	0	0	0	0	0	0	0	0	0	0	0	0	0	0
0	0	0	0	0	0	0	0	0	0	0	0	0	0	0	0	0	0	0
0	0	0	0	0	0	0	0	0	0	0	0	0	0	0	0	0	0	0
0	0	0	0	0	0	0	7	0	0	0	0	0	0	0	0	0	0	0
0	0	0	0	0	0	0	0	0	0	0	0	0	0	0	0	0	0	0
0	0	0	0	0	0	0	0	0	0	0	0	0	0	0	0	0	0	0
0	0	0	0	0	0	0	0	0	0	0	0	0	0	0	0	0	0	0
0	0	0	0	0	0	0	0	0	0	0	0	0	0	0	0	0	0	0
0	0	0	0	0	0	0	0	0	0	0	0	0	0	0	0	0	0	0
0	0	0	0	0	0	0	0	0	0	0	0	0	0	0	0	0	0	0
0	0	0	0	0	0	0	0	0	0	0	0	0	0	0	0	0	0	0
6	6	0	0	0	0	0	0	0	0	0	0	0	0	0	0	0	0	0
0	0	0	0	0	0	0	0	0	0	0	0	0	0	0	0	0	0	0
0	0	0	0	0	0	0	0	0	0	0	0	0	0	0	0	0	0	0
0	0	0	0	0	0	0	0	0	0	0	0	0	0	0	0	0	0	0
0	0	0	0	0	0	0	0	0	0	0	0	0	0	0	0	0	0	0
0	0	0	0	0	0	0	0	0	0	0	0	0	0	0	0	0	0	0
0	0	0	0	0	0	0	0	0	0	0	0	0	0	0	0	0	0	0
0	0	0	0	0	0	0	0	0	0	0	0	0	0	0	0	0	0	0
0	0	0	0	0	0	0	0	0	0	0	0	0	0	0	0	0	0	0
0	0	0	0	0	0	0	0	0	0	0	0	0	0	0	0	0	0	0
0	0	0	0	0	0	0	0	0	0	0	0	0	0	0	0	0	0	0

0	0	0	0	0	0	0	0	0	0	0	0	0	0	0	0	0	0	0
0	0	0	0	0	0	0	0	0	0	0	0	0	0	0	0	0	0	0
0	0	0	0	0	0	0	0	0	0	0	0	0	0	0	0	0	0	0
0	0	0	0	0	0	0	0	0	0	0	0	0	0	0	0	0	0	0
0	0	0	0	0	0	0	0	0	0	0	0	0	0	0	0	0	0	0
0	0	0	0	0	0	0	0	0	0	0	0	0	0	0	0	0	0	0
0	0	0	0	0	0	0	0	0	0	0	0	0	0	0	0	0	0	0
0	0	0	0	0	0	0	0	0	0	0	0	0	0	0	0	0	0	0
0	0	0	0	0	0	0	0	0	0	0	0	0	0	0	0	0	0	0
0	0	0	0	0	0	0	0	0	0	0	0	0	0	0	0	0	0	0
0	0	0	0	0	0	0	4	4	0	0	0	0	0	0	0	0	0	0
0	0	0	0	0	0	0	0	0	0	0	0	0	0	0	0	0	0	0
0	0	0	0	0	0	0	0	0	0	0	0	0	0	0	0	0	0	0
0	0	0	0	0	0	0	0	0	0	0	0	0	0	0	0	0	0	0
0	0	0	0	0	0	0	0	0	0	0	0	0	0	0	0	0	0	0
0	0	0	0	0	0	0	0	0	0	0	0	0	0	0	0	0	0	0
0	0	0	0	0	0	0	0	0	0	0	0	0	0	0	0	0	0	0
0	0	0	0	0	0	0	0	0	0	0	0	0	0	0	0	0	0	0
0	0	0	0	0	0	0	0	0	0	0	0	0	0	0	0	0	0	0
0	0	0	0	0	0	0	0	0	0	0	0	0	0	0	0	0	0	0
0	0	0	0	0	0	0	0	0	0	0	0	0	0	0	0	0	0	0
0	0	0	0	0	0	0	0	0	0	0	0	0	0	0	0	0	0	0
0	0	0	0	0	0	0	0	0	0	0	0	0	0	0	0	0	0	0
0	0	0	0	0	0	0	0	0	0	0	0	0	0	0	0	0	0	0
0	0	0	0	0	0	0	0	0	0	0	0	0	0	0	0	0	0	0
0	0	0	0	0	0	0	0	0	0	0	0	0	0	0	0	0	0	0
0	0	0	0	0	0	0	0	0	0	0	0	0	0	0	0	0	0	0
0	0	0	0	0	0	0	0	0	0	0	0	0	0	0	0	0	0	0
0	0	0	0	0	3	3	0	0	0	0	0	0	0	0	0	0	0	0
0	0	0	0	0	0	0	0	0	0	0	0	0	0	0	0	0	0	0
0	0	0	0	0	0	0	0	0	0	0	0	0	0	0	0	0	0	0
0	0	0	0	0	0	0	0	0	0	0	0	0	0	0	0	0	0	0
0	0	0	0	0	0	0	0	0	0	0	0	0	0	0	0	0	0	0
0	0	0	0	0	0	0	0	0	0	0	0	0	0	0	0	0	0	0
0	0	0	0	0	0	0	0	0	0	0	0	0	0	0	0	0	0	0
0	0	0	0	0	0	0	0	0	0	0	0	0	0	0	0	0	0	0
0	0	0	0	0	0	0	0	0	0	0	0	0	0	0	0	0	0	0

0	0	0	0	0	0	0	0	0	0	0	0	0	0	0	0	0	0	0
0	0	0	0	0	0	0	0	0	0	0	0	0	0	0	0	0	0	0
0	0	0	0	0	0	0	0	0	0	0	0	0	0	0	0	0	0	0
0	0	0	0	0	0	0	0	0	0	0	0	0	0	0	0	0	0	0
0	0	0	0	0	0	0	0	0	0	0	0	0	0	0	0	0	0	0
0	0	0	0	0	0	0	0	0	0	0	0	0	0	0	0	0	0	0
0	0	0	0	0	0	0	0	0	0	0	0	0	0	0	0	0	0	0
0	0	0	0	0	0	0	0	0	0	0	0	0	0	0	0	0	0	0
0	0	0	0	0	0	0	0	0	0	0	0	0	0	0	0	0	0	0
0	0	0	0	0	0	0	0	0	0	0	0	0	0	0	0	0	0	0
0	0	0	0	0	0	0	0	0	0	0	0	0	0	0	0	0	0	0
0	0	0	0	0	0	0	0	0	0	0	0	0	0	0	0	0	0	0
0	0	0	0	0	0	0	0	0	0	0	0	0	0	0	0	0	0	0
0	0	0	0	0	0	0	0	0	0	0	0	0	0	0	0	0	0	0
0	0	0	0	0	0	0	0	0	0	0	0	0	0	0	0	0	0	0
0	0	0	0	0	0	0	0	0	0	0	0	0	0	0	0	0	0	0
0	0	0	0	2	2	0	0	0	0	0	0	0	0	0	0	0	0	0
0	0	0	0	0	0	0	0	0	0	0	0	0	0	0	0	0	0	0
0	0	0	0	0	0	0	0	0	0	0	0	0	0	0	0	0	0	0
0	0	0	0	0	0	0	0	0	0	0	0	0	0	0	0	0	0	0
0	0	0	0	0	0	0	0	0	0	0	0	0	0	0	0	0	0	0
0	0	0	0	0	0	0	0	0	0	0	0	0	0	0	0	0	0	0
0	0	0	0	0	0	0	0	0	0									
Jan_DL006	2339	2144	928	201	1087	340	320	286	93	471	193	433	367	268	230	544	347	297
187	0	172	49	104	138	184	245	216	56	167	690	111	151	182	100	205	14	404
0	23	126	76	103	101	68	43	108	189	88	120	0	96	83	85	88	72	345
370	51	97	52	69	0	44	54	42	11	14	64	432	44	14	0	104	10	0
28	103	25	33	85	31	46	26	0	0	35	50	31	11	0	44	27	81	0
32	14	0	0	53	35	0	0	0	19	83	0	72	35	15	32	58	0	0
0	4	0	21	68	36	0	0	0	0	71	17	15	59	27	9	0	0	0
0	0	0	40	0	33	102	58	0	0	28	0	10	60	0	22	0	0	0
0	34	0	0	0	8	0	0	0	0	35	0	0	0	58	111	18	0	0
11	20	0	0	0	0	0	0	0	0	17	41	0	0	143	0	3	0	0
0	30	17	0	0	0	0	30	38	34	107	0	0	0	16	0	0	36	0
26	0	16	0	36	0	0	0	0	0	0	33	27	0	0	0	82	19	13
0	0	19	0	0	0	0	0	0	0	0	9	8	6	0	10	0	30	0
13	0	0	0	0	0	0	22	0	9	0	0	0	19	4	0	8	24	0

0	0	0	0	26	17	0	0	0	0	0	0	0	26	7	9	0	0	0
0	0	0	0	0	0	0	0	0	0	0	0	0	0	17	0	0	0	0
0	15	0	0	0	34	0	0	0	10	12	0	13	15	0	0	0	0	11
9	0	21	0	0	0	0	0	14	0	0	0	56	0	0	0	0	0	0
12	0	0	0	3	0	0	0	6	19	0	0	0	40	0	0	0	0	60
7	0	14	0	0	0	0	0	0	0	65	0	0	56	34	15	0	0	9
0	0	0	0	0	7	9	2	0	0	0	62	0	0	5	0	0	0	0
0	0	0	0	0	0	0	5	0	0	0	18	13	0	0	0	5	0	0
0	40	10	0	0	0	0	0	0	0	0	0	0	0	0	0	0	0	0
0	0	0	0	0	27	0	0	0	0	0	0	21	0	0	0	0	8	12
9	0	0	0	9	0	0	0	0	0	0	0	0	0	0	0	0	0	0
0	0	0	0	0	0	0	9	0	0	0	7	0	0	13	0	0	0	0
0	0	0	0	0	0	17	0	0	9	0	0	0	0	0	0	0	4	0
0	0	0	0	0	0	18	0	0	0	0	0	0	0	0	0	0	0	0
0	0	0	0	0	0	0	0	10	10	0	0	0	0	0	19	0	0	0
0	0	0	0	17	0	0	0	0	0	6	0	0	0	0	0	0	0	0
0	0	0	0	0	0	0	0	0	0	0	0	0	0	0	0	0	0	0
0	0	0	0	0	0	0	0	0	0	0	0	0	0	0	0	0	0	0
0	0	0	0	0	17	0	0	0	0	0	0	0	0	0	0	0	0	0
0	0	0	0	0	0	11	0	0	0	0	0	0	0	0	0	0	0	0
0	30	8	0	0	0	0	0	0	0	0	0	0	0	5	0	0	0	0
0	0	8	0	0	0	0	0	0	0	0	0	0	0	0	0	0	0	0
0	0	0	0	0	0	44	0	0	0	0	0	0	0	0	5	0	0	0
0	0	0	0	0	0	0	7	0	0	0	0	0	0	0	0	0	0	0
0	0	0	0	0	0	0	0	0	0	0	0	0	0	0	0	0	0	0
0	0	0	14	0	0	0	0	0	0	18	0	0	0	0	0	0	0	0
0	0	0	0	0	0	0	0	0	0	12	0	0	0	0	0	0	0	0
0	0	0	0	0	0	0	0	0	0	0	0	0	0	0	0	0	0	0
0	0	0	0	0	0	0	0	0	0	0	0	0	5	0	0	0	0	0
0	0	0	0	0	0	0	0	0	0	0	13	0	0	0	0	0	0	0
0	0	0	0	0	0	0	0	0	0	0	0	0	0	0	0	0	0	0
0	0	0	0	0	0	0	0	0	0	0	0	0	0	0	0	0	0	0
0	0	0	0	0	0	0	0	0	0	0	0	0	0	0	0	0	0	0
0	0	0	0	0	0	0	0	0	0	0	0	0	0	0	0	0	0	0
0	0	0	0	0	0	0	0	0	0	0	0	0	0	0	0	0	0	0
0	0	0	0	0	0	0	0	0	0	0	0	0	0	0	0	0	0	0
0	0	0	0	0	0	0	0	0	0	0	0	0	0	0	0	0	0	0

18	0	0	0	7	0	0	0	0	0	0	0	0	0	0	0	0	0	0
0	0	0	0	0	0	0	0	0	0	0	0	0	0	0	0	0	0	0
0	0	0	0	0	0	0	0	0	0	0	0	0	0	0	0	0	0	0
0	0	0	9	0	0	0	39	0	0	0	0	0	0	0	0	0	0	0
0	0	0	0	0	0	0	0	0	0	0	0	0	0	0	10	0	0	0
0	0	0	0	0	0	0	0	0	0	0	0	0	0	0	0	0	0	0
0	0	0	0	0	0	0	0	0	0	0	0	0	0	0	0	0	0	0
0	0	0	0	0	0	0	0	0	0	0	0	0	0	0	0	0	0	0
0	0	0	0	0	0	0	7	0	0	0	0	0	0	0	0	0	0	0
0	0	0	0	0	6	0	0	0	0	0	0	0	0	0	0	0	0	0
0	0	0	0	0	0	5	0	0	0	0	0	0	0	0	25	0	0	0
0	0	0	0	0	0	0	0	0	0	0	0	0	0	0	0	0	0	0
0	0	0	0	0	0	0	0	0	0	0	0	0	0	0	0	0	0	0
0	0	0	0	0	0	0	0	0	0	0	0	0	0	0	0	0	0	0
0	0	0	0	0	0	0	0	0	0	0	0	0	0	0	0	0	0	0
0	0	0	0	0	0	0	0	0	0	0	0	0	0	0	0	0	0	0
0	0	0	0	0	0	0	0	0	0	0	0	0	0	0	0	0	0	0
0	0	0	0	0	0	0	0	0	0	0	0	0	0	0	0	0	0	0
0	0	0	0	0	0	0	0	0	0	0	0	0	0	0	0	0	0	0
0	0	0	0	0	0	0	0	0	0	0	0	0	0	0	0	0	0	0
0	0	0	0	0	0	0	0	0	0	0	0	0	0	0	0	0	0	0
0	0	0	0	0	0	0	0	0	0	0	0	0	0	0	0	0	0	0
0	0	0	0	0	0	0	0	5	0	0	0	0	0	0	0	0	0	0
0	0	0	0	0	0	0	0	0	0	0	0	0	0	0	0	0	0	0
0	0	0	0	0	0	0	0	0	0	0	0	0	0	0	0	0	0	0
0	0	0	0	0	0	0	0	0	0	0	0	0	0	0	0	0	0	0
0	0	0	0	0	0	0	0	0	0	0	6	0	0	0	0	0	0	0
0	0	0	0	0	0	0	0	0	0	0	0	0	0	0	0	0	0	0
0	0	0	0	0	0	0	0	0	0	0	0	0	0	0	0	0	0	0
0	0	0	0	0	0	0	0	0	0	0	0	0	0	0	0	0	0	0
0	0	0	0	0	0	0	0	0	0	0	0	0	0	0	0	0	0	0
0	0	0	0	0	0	0	0	0	0	0	0	0	0	0	0	0	0	0
0	0	0	0	0	0	0	0	0	0	0	0	0	0	0	0	0	0	0
0	0	0	0	0	0	0	0	0	0	0	0	0	0	0	0	0	0	0
0	0	0	0	0	0	0	0	0	0	0	0	0	0	0	0	0	0	0
0	0	0	0	0	0	0	0	0	0	0	0	0	0	0	0	0	0	0
0	0	0	0	0	0	0	0	0	0	0	0	0	0	0	0	0	0	0

0	0	0	0	0	0	0	0	0	0	0	0	0	0	0	0	0	0	0
0	0	0	0	0	0	0	0	0	0	0	0	0	0	0	0	0	0	0
0	0	0	0	0	0	0	0	0	0	0	0	0	0	0	0	0	0	0
0	0	0	0	0	0	0	0	0	0	0	0	0	0	0	0	0	0	0
0	0	0	0	0	0	0	0	0	0	0	0	0	0	0	0	0	0	0
0	0	0	0	0	0	0	0	0	0	0	0	0	0	0	0	0	0	0
0	0	0	0	0	0	0	0	0	0	0	0	0	0	0	0	0	0	0
0	0	0	0	0	0	0	0	0	0	0	0	0	4	0	0	0	0	0
0	0	0	0	0	0	0	0	0	0	0	0	0	0	0	0	0	0	0
0	0	0	0	0	0	0	0	0	0	0	0	0	0	0	0	0	0	0
0	0	0	0	0	0	0	0	0	0	0	0	0	0	0	0	0	0	0
0	0	0	0	0	0	0	0	0	0	0	0	0	0	0	0	0	0	0
0	0	0	0	0	0	0	0	0	0	0	0	0	0	0	0	0	0	0
0	0	0	0	0	0	0	0	0	0	0	0	0	0	0	0	0	0	0
0	0	0	0	0	0	0	0	0	0	0	0	0	0	0	0	0	0	0
0	0	0	0	0	0	0	0	0	0	0	0	0	0	0	0	0	0	0
0	0	0	0	0	0	0	0	0	0	0	0	0	0	0	0	0	0	0
0	0	0	0	0	0	0	0	0	0	0	0	0	0	0	0	0	0	0
0	0	0	0	0	0	0	0	0	0	0	0	0	0	0	0	0	0	0
0	0	0	0	0	0	0	0	0	0	0	0	0	0	0	0	0	0	0
0	0	0	0	0	0	0	0	0	0	0	0	0	0	0	0	0	0	0
0	0	0	0	0	0	0	0	0	0	0	0	0	0	0	0	0	0	0
0	0	0	0	0	0	0	0	0	0	0	0	0	0	0	0	0	0	0
0	0	0	0	0	0	0	0	0	0	0	0	0	0	0	0	0	0	0
0	0	0	0	0	0	0	0	0	0	0	0	0	0	0	0	0	0	0
0	0	0	0	0	0	0	0	0	0	0	0	0	0	0	0	0	0	0
0	0	0	0	0	0	0	0	0	0	0	0	0	0	0	0	0	0	0
0	0	0	0	0	0	0	0	0	0	0	0	0	0	0	0	0	0	0
0	0	0	0	0	0	0	0	0	0	0	0	0	0	0	0	0	0	0
0	0	0	0	0	0	0	0	0	0	0	0	0	0	5	0	0	0	0
0	0	0	0	0	0	0	0	0	0	0	0	0	0	0	0	0	0	0
0	0	0	0	0	0	0	0	0	0	0	0	0	0	0	0	0	0	0
0	0	0	0	0	0	0	0	0	0	0	0	0	0	0	0	0	0	0
0	0	0	0	0	0	0	0	0	0	0	0	0	0	0	0	0	0	0
0	0	0	0	0	0	0	0	0	0	0	0	0	0	0	0	0	0	0
0	0	0	0	0	0	0	0	0	0	0	0	0	0	0	0	0	0	0
0	0	0	0	0	0	0	0	0	0	0	0	0	0	0	0	0	0	0

0	0	0	0	0	0	0	0	0	0	0	0	0	0	0	0	0	0	0
0	0	0	0	0	0	0	0	0	0	0	0	0	0	0	0	0	0	0
0	0	0	0	0	0	0	0	0	0	0	0	0	0	0	0	0	0	0
0	0	0	0	0	0	0	0	0	0	0	0	0	0	0	0	0	0	0
0	0	0	0	0	0	0	0	0	0	0	0	0	0	0	0	0	0	0
0	0	0	0	0	0	0	0	0	0	0	0	0	0	0	0	0	0	0
0	0	0	0	0	0	0	0	0	0	0	0	0	0	0	0	0	0	0
0	0	0	0	0	0	0	0	0	0	0	0	0	0	0	0	0	0	0
0	0	0	0	0	0	0	0	0	0	0	0	0	0	0	0	0	0	0
0	0	0	0	0	0	0	0	0	0	0	0	0	0	0	0	0	0	0
0	0	0	0	0	0	0	0	0	0	0	0	0	0	0	0	0	0	0
0	0	0	0	0	0	0	0	0	0	0	0	0	0	0	0	0	0	0
0	0	0	0	0	0	0	0	0	0	0	0	0	0	0	0	0	0	0
0	0	0	0	0	0	0	0	0	0	0	0	0	0	0	0	0	0	0
0	0	0	0	0	0	0	0	0	0	0	0	0	0	0	0	0	0	0
0	0	0	0	0	0	0	0	0	0	0	0	0	0	0	0	0	0	0
0	0	0	0	0	0	0	0	0	0	0	0	0	0	0	0	0	0	0
0	0	0	0	0	0	0	0	0	0	0	0	0	0	0	0	0	0	0
0	0	0	0	0	0	0	0	0	0	0	0	0	0	0	0	0	0	0
0	0	0	0	0	0	0	0	0	0	0	0	0	0	0	0	0	0	0
0	0	0	0	0	0	0	0	0	0	0	0	0	0	0	0	0	0	0
0	0	0	0	0	0	0	0	0	0	0	0	0	0	0	0	0	0	0
0	0	0	0	0	0	0	0	0	0	0	0	0	0	0	0	0	0	0
0	0	0	0	0	0	0	0	0	0	0	0	0	0	0	0	0	0	0
0	0	0	0	0	0	0	0	0	0	0	0	0	0	0	0	0	0	0
0	0	0	0	0	0	0	0	0	0	0	0	0	0	0	0	0	0	0
0	0	0	0	0	0	0	0	0	0	0	0	0	0	0	0	0	0	0
0	3	0	0	0	0	0	0	0	0	0	0	0	0	0	0	0	0	0
0	0	0	0	0	0	0	0	0	0	0	0	0	0	0	0	0	0	0
0	0	0	0	0	0	0	0	0	0	0	0	0	0	0	0	0	0	0
0	0	0	0	0	0	0	0	0	0	0	0	0	0	0	0	0	0	0
0	0	0	0	0	0	0	0	0	0	0	0	0	0	0	0	0	0	0
0	0	0	0	0	0	0	0	0	0	0	0	0	0	0	0	0	0	0
0	0	0	0	0	0	0	0	0	0	0	0	0	0	0	0	0	0	0
0	0	0	0	0	0	0	0	0	0	0	0	0	0	0	0	0	0	0
0	0	0	0	0	0	0	0	0	0	0	0	0	0	0	0	0	0	0
0	0	0	0	0	0	0	0	0	0	0	0	0	0	0	0	0	0	0

0	0	0	0	0	0	0	0	0	0	0	0	0	0	0	0	0	0	0
0	0	0	0	0	0	0	0	0	0	0	0	0	0	0	0	0	0	0
0	0	0	0	0	0	0	0	0	0	0	0	0	0	0	0	0	0	0
0	0	0	0	0	0	0	0	0	0	0	0	0	0	0	0	0	0	0
0	0	0	0	0	0	0	0	0	0	0	0	0	0	0	0	0	0	0
0	0	0	0	0	0	0	0	0	0	0	0	0	0	0	0	0	0	0
0	0	0	0	0	0	0	0	0	0	0	0	0	0	0	0	0	0	0
0	0	0	0	0	0	0	0	0	0	0	0	0	0	0	0	0	0	0
0	0	0	0	0	0	0	0	0	0	0	0	0	0	0	0	0	0	0
0	0	0	0	0	0	0	0	0	0	0	0	0	0	0	0	0	0	0
0	0	0	0	0	0	0	0	0	0	0	0	0	0	0	0	0	2	2
0	0	0	0	0	0	0	0	0	0	0	0	0	0	0	0	0	0	0
0	0	0	0	0	0	0	0	0	0	0	0	0	0	0	0	0	0	0
0	0	0	0	0	0	0	0	0	0	0	0	0	0	0	0	0	0	0
0	0	0	0	0	0	0	0	0	0									
Jan_DL060	953	1567	595	391	174	74	65	55	122	340	527	172	309	12	214	300	0	113
137	0	157	0	129	191	165	121	161	217	81	280	131	87	122	215	84	46	225
211	26	0	26	77	134	54	208	19	146	64	167	0	52	38	171	97	93	159
142	106	184	29	32	0	61	67	86	46	162	0	32	61	0	0	34	32	14
0	148	0	99	99	0	49	17	0	50	43	0	15	33	0	54	0	0	0
16	11	0	0	0	12	16	0	0	7	15	12	36	22	16	0	0	17	0
0	15	0	0	26	0	0	0	0	0	24	9	8	19	0	0	0	34	0
0	0	0	25	0	55	44	0	0	6	0	0	17	0	0	0	0	0	0
0	0	0	80	0	67	0	0	0	0	0	0	8	0	10	77	18	0	0
0	0	14	0	9	0	0	0	0	17	0	0	0	0	49	0	0	0	0
0	21	0	0	0	0	0	0	0	7	26	0	24	63	0	0	0	15	0
0	0	12	0	0	0	0	0	0	0	0	0	0	0	0	0	0	0	0
0	0	0	23	0	0	0	0	0	0	0	0	5	6	0	0	0	38	0
0	0	0	0	0	0	0	0	0	0	0	0	0	0	0	0	0	9	0
0	0	0	0	0	0	0	0	0	3	5	0	0	0	0	0	0	0	0
0	0	0	0	0	0	0	0	10	0	0	0	4	0	12	0	0	0	0
0	0	0	0	0	0	0	0	0	0	0	0	0	0	7	4	0	0	0
0	0	0	0	0	0	0	0	0	0	0	15	0	11	0	0	0	0	0
0	0	0	0	11	0	0	0	0	0	0	0	0	0	0	0	0	0	0
17	7	0	0	9	0	8	0	0	0	0	0	0	0	0	0	0	0	8
0	12	0	0	0	0	0	2	0	0	0	0	0	0	9	0	0	0	0
0	0	0	0	0	0	0	6	0	0	0	13	0	0	0	0	0	0	0

0	0	0	0	0	0	0	0	0	0	0	0	3	0	0	0	0	0	0
0	3	0	0	0	0	0	0	0	0	0	0	0	0	0	0	0	0	0
0	0	0	0	0	0	0	0	0	0	0	0	0	0	0	0	0	0	0
0	0	0	0	0	0	0	11	0	0	0	0	0	0	0	0	0	0	0
0	0	0	0	0	0	0	0	0	0	0	0	0	0	0	0	10	0	0
0	0	0	0	0	0	0	0	3	0	0	0	0	0	0	0	0	16	0
0	0	0	0	0	0	0	0	0	0	0	0	0	0	0	0	0	0	0
0	7	0	0	0	0	0	0	0	0	0	0	0	0	0	0	0	0	0
0	0	0	0	0	0	0	0	0	0	0	0	0	0	0	0	0	0	0
0	0	0	0	0	0	0	0	0	0	0	0	0	0	0	0	0	0	0
0	0	0	0	0	0	9	0	0	0	0	0	0	0	0	0	0	0	0
0	0	0	0	0	0	0	0	23	0	0	0	0	0	0	0	0	0	0
0	0	0	0	0	0	0	0	0	0	0	0	0	0	0	0	0	0	0
0	0	0	0	0	0	0	0	0	0	0	0	0	0	0	0	0	0	0
0	0	0	0	0	0	0	0	0	0	0	0	0	0	0	0	0	0	0
0	0	0	0	0	0	0	0	0	0	0	0	0	0	0	0	0	0	0
3	0	0	0	0	0	0	0	0	0	11	0	0	0	0	0	0	0	0
0	0	0	0	0	0	0	0	0	0	0	0	0	0	0	0	0	0	0
0	0	0	0	0	0	0	0	0	0	0	0	0	0	0	0	0	0	0
0	0	0	0	0	0	0	0	0	0	0	0	0	0	0	0	0	0	0
0	0	0	0	0	0	0	0	0	0	0	0	0	0	0	0	0	0	0
0	0	0	0	0	0	0	0	0	14	0	0	0	0	0	0	0	0	0
0	0	0	0	0	0	0	0	0	0	0	0	0	0	0	0	0	0	0
0	0	0	0	0	0	0	0	0	0	2	0	0	0	0	0	0	0	0
0	0	0	0	0	0	0	0	0	0	0	0	0	0	0	0	0	0	0
0	0	0	0	0	0	0	0	0	0	0	0	0	0	0	0	0	0	0
0	0	0	0	0	0	0	0	0	0	0	0	0	0	0	0	0	0	0
0	0	0	0	0	0	0	0	0	0	0	0	0	0	0	0	0	0	0
0	0	0	0	0	0	0	0	0	0	0	0	0	0	0	0	0	0	0
0	0	0	0	0	0	0	0	0	0	0	0	0	0	0	0	0	0	0
0	0	0	0	0	0	0	0	0	0	0	0	0	0	0	0	0	0	0
0	0	0	0	0	0	0	0	0	0	0	0	0	0	0	0	0	0	0
0	0	0	0	0	0	0	0	5	0	0	0	0	0	0	0	0	0	0
0	0	0	0	0	0	0	0	0	0	0	0	0	0	0	0	0	0	0
0	0	0	0	0	0	0	0	0	0	0	0	0	0	0	0	0	0	0
0	0	0	0	0	0	0	0	0	0	0	0	0	0	0	0	0	0	0
0	0	0	0	0	0	0	0	0	0	0	0	0	0	0	0	0	0	0

0	0	0	0	0	0	0	0	0	0	0	0	0	0	0	0	0	0	0
0	0	0	0	0	0	0	0	0	0	0	0	0	0	0	0	0	0	0
0	0	0	0	0	0	0	0	0	0	0	0	0	0	0	0	0	0	0
0	0	0	0	0	0	0	0	0	0	0	0	0	0	0	0	0	0	0
0	0	0	0	0	0	0	0	0	0	0	0	0	0	0	0	0	0	0
0	0	0	0	0	0	0	0	0	0	0	0	0	0	0	0	0	0	0
0	0	0	0	0	0	0	0	0	0	0	0	0	0	0	0	0	0	0
0	0	0	0	0	0	0	0	0	0	0	0	0	0	0	0	0	0	0
0	0	0	0	0	0	0	0	0	0	0	0	0	0	0	0	0	0	0
0	0	0	0	0	0	0	0	0	0	0	0	0	0	0	0	0	0	0
0	0	0	0	0	0	0	0	0	0	0	0	0	0	0	0	0	0	0
0	0	0	0	0	0	0	0	0	0	0	0	0	0	0	0	0	0	0
0	0	0	0	0	0	0	0	0	0	0	0	0	0	0	0	0	0	0
0	0	0	0	0	0	0	0	0	0	0	0	0	0	0	0	0	0	0
0	0	0	0	0	0	0	0	0	0	0	0	0	0	0	0	0	0	0
0	0	0	0	0	0	0	0	0	0	0	0	0	0	0	0	0	0	0
0	0	0	0	0	0	0	0	0	0	0	0	0	0	0	0	0	0	0
0	0	0	0	0	0	0	0	0	0	0	0	0	0	0	0	0	0	0
0	0	0	0	0	0	0	0	0	0	0	0	0	0	0	0	0	0	0
0	0	0	0	0	0	0	0	0	0	0	0	0	0	0	0	0	0	0
0	0	0	0	0	0	0	0	0	0	0	0	0	0	0	0	0	0	0
0	0	0	0	0	0	0	0	0	0	0	0	0	0	0	0	0	0	0
0	0	0	0	0	0	0	0	0	0	0	0	0	0	0	0	0	0	0
0	0	0	0	0	0	0	0	0	0	0	0	0	0	0	0	0	0	0
0	0	0	0	0	0	0	0	0	0	0	0	0	0	0	0	0	0	0
0	0	0	0	0	0	0	0	0	0	0	0	0	0	0	0	0	0	0
0	0	0	0	0	0	0	0	0	0	0	0	0	0	0	0	0	0	0
0	0	0	0	0	0	0	0	0	0	0	0	0	0	0	0	0	0	0
0	0	0	0	0	0	0	0	0	0	0	0	0	6	0	0	0	0	0
0	0	0	0	0	0	0	0	0	0	0	0	0	0	0	0	0	0	0
0	0	0	0	0	0	0	0	0	0	0	0	0	0	0	0	0	0	0
0	0	0	0	0	0	0	0	0	0	0	0	0	0	0	0	0	0	0
0	0	0	0	0	0	0	0	0	0	0	0	0	0	0	0	0	0	0
0	0	0	0	0	0	0	0	0	0	0	0	0	0	0	0	0	0	0
0	0	0	0	0	0	0	0	0	0	0	0	0	0	0	0	0	0	0
0	0	0	0	0	0	0	0	0	0	0	0	0	0	0	0	0	0	0
0	0	0	0	0	0	0	0	5	0	0	0	0	0	0	0	0	0	0

0	0	0	0	0	0	0	0	0	0	0	0	0	0	0	0	0	0	0
0	0	0	0	0	0	0	0	0	0	0	0	0	0	0	0	0	0	0
0	0	0	0	0	0	0	0	0	0	0	0	0	0	0	0	0	0	0
0	0	0	0	0	0	0	0	0	0	0	0	0	0	0	0	0	0	0
0	0	0	0	0	0	0	0	0	0	0	0	0	0	0	0	0	0	0
0	0	0	0	0	0	0	0	0	0	0	0	0	0	0	0	0	0	0
0	0	0	0	0	0	0	0	0	0	0	0	0	0	0	0	0	0	0
0	0	0	0	0	0	0	0	0	0	0	0	0	0	0	0	0	0	0
0	0	0	0	0	0	0	0	0	0	0	0	0	0	0	0	0	0	0
0	0	0	0	0	0	0	0	0	0	0	0	0	0	4	4	4	0	0
0	0	0	0	0	0	0	0	0	0	0	0	0	0	0	0	0	0	0
0	0	0	0	0	0	0	0	0	0	0	0	0	0	0	0	0	0	0
0	0	0	0	0	0	0	0	0	0	0	0	0	0	0	0	0	0	0
0	0	0	0	0	0	0	0	0	0	0	0	0	0	0	0	0	0	0
0	0	0	0	0	0	0	0	0	0	0	0	0	0	0	0	0	0	0
0	0	0	0	0	0	0	0	0	0	0	0	0	0	0	0	0	0	0
0	0	0	0	0	0	0	0	0	0	0	0	0	0	0	0	0	0	0
0	0	0	0	0	0	0	0	0	0	0	0	0	0	0	0	0	0	0
0	0	0	0	0	0	0	0	0	0	0	0	0	0	0	0	0	0	0
0	0	0	0	0	0	0	0	0	0	0	0	0	0	0	0	0	0	0
0	0	0	0	0	0	0	0	0	0	0	0	0	0	0	0	0	0	0
0	0	0	0	0	0	0	0	0	0	0	0	0	0	0	0	0	0	0
0	0	0	0	0	0	0	0	0	0	0	0	0	0	0	0	3	0	0
0	0	0	0	0	0	0	0	0	0	0	0	0	0	0	0	0	0	0
0	0	0	0	0	0	0	0	0	0	0	0	0	0	0	0	0	0	0
0	0	0	0	0	0	0	0	0	0	0	0	0	0	0	0	0	0	0
0	0	0	0	0	0	0	0	0	0	0	0	0	0	0	0	0	0	0
0	0	0	0	0	0	0	0	0	0	0	0	0	0	0	0	0	0	0
0	0	0	0	0	0	0	0	0	0	0	0	0	0	0	0	0	0	0
0	0	0	0	0	0	0	0	0	0	0	0	0	0	0	0	0	0	0
0	0	0	0	0	0	0	0	0	0	0	0	0	0	0	0	0	0	0
0	0	0	0	0	0	0	0	0	0	0	0	0	0	0	0	0	0	0
0	0	0	0	0	0	0	0	0	0	0	0	0	0	0	0	0	0	0
0	0	0	0	0	0	0	0	0	0	0	0	0	0	0	0	0	0	0
0	0	0	0	0	0	0	0	0	0	0	0	0	0	0	0	0	0	0
0	0	0	0	0	0	0	0	0	0	0	0	0	0	0	0	0	0	0
0	0	0	0	0	0	0	0	0	0	0	0	0	0	0	0	0	0	0

0	0	0	0	0	0	0	0	0	0	0	0	0	0	0	0	0	0	0
0	0	0	0	0	0	0	0	0	0	0	0	0	0	0	0	0	0	0
0	0	0	0	0	0	0	0	0	0	0	0	0	0	0	0	0	0	0
0	0	0	0	0	0	0	0	0	0	0	0	0	0	0	0	0	0	0
0	0	0	0	0	0	0	0	0	0	0	0	0	0	0	0	0	0	0
0	0	0	0	0	0	0	0	0	0	0	0	0	0	0	0	0	0	0
0	0	0	0	0	0	0	0	0	0	0	0	0	0	0	0	0	0	0
0	0	0	0	0	0	0	0	0	0	0	0	0	0	0	0	0	0	0
0	0	0	0	0	0	0	0	0	0	0	0	0	0	0	0	0	0	0
0	0	0	0	0	0	0	0	0	0	0	0	0	0	0	0	0	0	0
0	0	0	0	0	0	0	0	0	0	0	0	0	0	0	0	0	0	0
0	0	0	0	0	0	0	0	0	0	0	0	0	0	2	2	2	2	2
2	2	2	2	0	0	0	0	0	0	0	0	0	0	0	0	0	0	0
0	0	0	0	0	0	0	0	0	0	0	0	0	0	0	0	0	0	0
0	0	0	0	0	0	0	0	0	0	0	0	0	0	0	0	0	0	0
0	0	0	0	0	0	0	0	0	0	0	0	0	0	0	0	0	0	0
0	0	0	0	0	0	0	0	0	0	0	0	0	0	0	0	0	0	0
0	0	0	0	0	0	0	0	0	0	0	0	0	0	0	0	0	0	0
0	0	0	0	0	0	0	0	0	0	0	0	0	0	0	0	0	0	0
0	0	0	0	0	0	0	0	0	0	0	0	0	0	0	0	0	0	0
0	0	0	0	0	0	0	0	0	0	0	0	0	0	0	0	0	0	0
0	0	0	0	0	0	0	0	0	0	0	0	0	0	0	0	0	0	0
0	0	0	0	0	0	0	0	0	0	0	0	0	0	0	0	0	0	0
0	0	0	0	0	0	0	0	0	0	0	0	0	0	0	0	0	0	0
0	0	0	0	0	0	0	0	0	0	0	0	0	0	0	0	0	0	0
0	0	0	0	0	0	0	0	0	0	0	0	0	0	0	0	0	0	0
0	0	0	0	0	0	0	0	0	0	0	0	0	0	0	0	0	0	0
0	0	0	0	0	0	0	0	0	0	0	0	0	0	0	0	0	0	0
0	0	0	0	0	0	0	0	0	0	0	0	0	0	0	0	0	0	0
0	0	0	0	0	0	0	0	0	0	0	0	0	0	0	0	0	0	0
0	0	0	0	0	0	0	0	0	0	0	0	0	0	0	0	0	0	0
0	0	0	0	0	0	0	0	0	0	0	0	0	0	0	0	0	0	0
0	0	0	0	0	0	0	0	0	0	0	0	0	0	0	0	0	0	0
0	0	0	0	0	0	0	0	0	0	0	0	0	0	0	0	0	0	0
0	0	0	0	0	0	0	0	0	0	0	0	0	0	0	0	0	0	0
0	0	0	0	0	0	0	0	0	0	0	0	0	0	0	0	0	0	0
0	0	0	0	0	0	0	0	0	0	0	0	0	0	0	0	0	0	0

0	0	0	0	0	0	0	0	0	0	0	0	0	0	0	0	0	0	0
0	0	0	0	0	0	0	0	0	0	0	0	0	0	0	0	0	0	0
0	0	0	0	0	0	0	0	0	0	0	0	0	0	0	0	0	0	0
0	0	0	0	0	0	0	0	0	0	0	0	0	0	0	0	0	0	0
0	0	0	0	0	0	0	0	0	0	0	0	0	0	0	0	0	0	0
0	0	0	0	0	0	1	0	0	0	0	0	0	0	0	0	0	0	0
0	0	0	0	0	0	0	0	0	0									
Jan_DL061	1267	854	504	422	31	202	137	118	253	242	283	187	204	48	213	320	45	138
129	0	63	0	136	87	177	116	192	206	0	170	131	123	104	112	94	115	130
192	36	0	137	75	80	58	193	46	108	52	196	0	49	86	224	112	59	95
95	72	185	14	49	62	31	42	71	36	25	85	0	0	0	0	20	15	0
37	195	48	28	14	0	42	35	0	0	111	0	14	0	32	0	0	14	0
0	18	0	0	95	0	0	0	0	20	0	0	33	0	22	11	21	0	0
0	0	0	5	15	0	0	0	0	0	4	0	7	13	0	0	0	0	0
0	17	0	13	0	0	26	0	0	5	0	0	7	0	0	18	0	0	0
0	3	0	26	0	9	0	0	0	0	6	0	12	0	21	48	4	0	0
0	12	11	9	0	0	0	0	0	8	0	0	0	0	20	0	0	0	0
34	0	0	0	0	0	0	0	0	0	0	0	0	0	0	0	0	0	0
12	0	15	0	0	0	11	0	0	0	0	0	9	0	0	0	0	3	0
0	0	0	18	0	0	0	0	0	0	0	16	0	0	0	0	0	22	0
0	0	0	2	0	0	0	0	0	4	0	0	0	0	9	0	0	11	0
0	0	0	8	0	0	0	0	5	0	0	0	0	0	0	4	14	0	0
0	0	0	11	0	0	0	0	9	0	0	0	0	0	0	0	0	0	0
0	0	0	0	0	0	0	0	0	0	0	0	0	0	0	7	0	0	5
0	0	0	0	0	0	0	5	0	0	0	10	0	0	0	0	0	0	0
0	8	0	0	0	0	0	0	0	0	0	0	0	0	0	0	0	0	0
24	0	0	0	0	0	0	0	0	0	0	0	0	0	0	0	0	0	0
0	0	0	0	13	0	0	0	0	0	0	0	0	0	0	0	0	0	0
0	0	0	0	0	0	0	0	0	0	0	6	0	0	0	0	0	0	0
0	0	0	0	0	0	0	0	0	0	0	0	0	0	0	0	0	0	0
0	0	0	0	0	0	0	0	0	0	0	0	0	0	0	0	0	0	0
0	0	0	0	0	0	0	0	0	0	0	13	0	0	0	0	0	0	0
0	0	0	0	8	0	0	0	0	0	0	0	0	0	0	0	0	0	0
0	0	0	0	0	0	0	0	0	4	0	0	0	0	0	0	0	0	0
0	0	0	0	0	0	0	0	0	0	0	0	0	0	0	0	0	0	0
0	0	0	0	0	0	0	0	0	0	0	0	0	0	0	0	0	0	0
0	4	0	0	0	0	0	0	0	0	0	13	0	0	0	0	0	0	0

0	0	0	0	0	0	0	0	0	0	0	0	0	0	0	0	0	0	0
0	0	0	0	0	0	0	2	0	0	0	0	0	0	0	0	0	0	0
0	0	0	0	0	0	0	0	0	0	0	0	0	0	0	0	0	0	7
0	0	0	0	0	16	0	0	0	0	0	0	0	0	0	0	0	0	0
0	0	9	0	0	0	0	0	0	0	0	0	0	0	0	0	0	0	0
0	0	0	0	0	0	0	0	0	0	0	0	0	0	0	0	0	0	0
0	0	0	0	5	0	0	0	0	0	0	0	0	0	0	0	0	0	0
0	0	0	0	0	0	0	0	0	0	0	0	0	0	0	0	0	0	0
0	0	0	0	0	0	0	0	0	0	0	0	0	0	0	7	0	0	0
0	0	0	0	0	0	0	0	0	5	0	0	0	0	0	0	0	0	0
0	0	0	0	0	0	0	4	0	0	0	0	0	0	0	0	0	0	0
0	0	0	0	0	0	0	0	0	0	0	0	0	0	0	0	0	0	0
0	0	0	0	0	0	0	0	0	0	0	0	0	0	0	0	0	0	0
0	0	0	0	0	0	0	0	0	0	0	0	0	0	0	0	0	0	0
0	0	0	0	0	0	0	0	0	0	0	0	0	0	0	0	0	0	0
0	0	0	0	0	0	0	0	0	0	0	0	0	0	0	0	0	0	0
0	0	0	0	0	0	0	0	0	4	0	0	0	0	0	0	0	0	0
0	0	0	0	0	0	0	0	0	0	0	0	0	0	0	0	0	0	0
0	0	0	0	0	0	0	0	0	0	0	0	0	0	0	0	0	0	0
0	0	0	0	0	0	0	0	0	0	0	0	0	0	0	0	0	0	0
0	0	0	0	0	0	0	0	0	0	0	0	0	0	0	0	0	0	0
0	0	0	0	0	0	0	0	0	0	0	0	0	0	0	0	0	0	0
0	0	0	0	0	0	0	0	0	0	0	0	0	0	0	0	0	0	0
0	0	0	0	0	0	0	0	0	0	0	0	0	0	0	0	0	0	0
0	0	0	0	0	0	0	0	0	0	0	0	0	0	0	0	0	0	0
0	0	0	0	0	0	0	0	0	0	0	0	0	0	0	0	0	0	0
0	0	0	0	0	0	0	0	0	0	0	0	3	0	0	0	0	0	0
0	0	0	0	0	0	0	0	0	0	0	0	0	0	0	0	0	0	0
0	0	0	0	0	0	0	0	0	0	0	0	0	0	0	0	0	0	0
0	0	0	0	0	0	0	0	0	0	0	0	0	0	0	0	0	0	0
0	0	0	0	0	0	0	0	0	0	0	0	0	0	0	0	0	0	0
0	0	0	0	0	0	0	0	0	0	0	0	0	0	0	0	0	0	0
0	0	0	0	0	0	0	0	0	0	0	0	0	0	0	0	0	0	0
0	0	0	0	0	0	0	0	0	0	0	0	0	0	0	0	0	0	0
0	0	0	0	0	0	0	0	0	0	0	0	0	0	0	0	0	0	0
0	0	0	0	0	0	0	0	0	0	0	0	0	0	0	0	0	0	0
0	0	0	0	0	0	0	0	0	0	0	0	0	0	0	0	0	0	0

0	0	0	0	0	0	0	0	0	0	0	0	0	0	0	0	0	0	0
0	0	0	0	0	0	0	0	0	0	0	0	0	0	0	0	0	0	0
0	0	0	0	0	0	0	0	0	0	0	0	0	0	0	0	0	0	0
0	0	0	0	0	0	0	0	0	0	0	0	0	0	0	0	0	0	0
0	0	0	0	0	0	0	0	0	0	0	0	0	0	0	0	0	0	0
0	0	0	0	0	0	0	0	0	0	0	0	0	0	0	0	0	0	0
0	0	0	0	0	0	0	0	0	0	0	0	0	0	0	0	0	0	0
0	0	0	0	0	0	0	0	0	0	0	0	0	0	0	0	0	0	0
0	0	0	0	0	0	0	0	0	0	0	0	0	0	0	0	0	0	0
0	0	0	0	0	0	0	0	0	0	0	0	0	0	0	0	0	0	0
0	0	0	0	0	0	0	0	0	0	0	0	0	0	0	0	0	0	0
0	0	0	0	0	0	0	0	0	0	0	0	0	0	0	0	0	0	0
0	0	0	0	0	0	0	0	0	0	0	0	0	0	0	0	0	0	0
0	0	0	0	0	0	0	0	0	0	0	0	0	0	0	0	0	0	0
0	0	0	0	0	0	0	0	0	0	0	0	0	0	0	0	0	0	0
0	0	0	0	0	0	0	0	0	0	0	0	0	0	0	0	0	0	0
0	0	0	0	0	0	0	0	0	0	0	0	0	0	0	0	0	0	0
0	0	0	0	0	0	0	0	0	0	0	0	0	0	0	0	0	0	0
0	0	0	0	0	0	0	0	0	0	0	0	0	0	0	0	0	0	0
0	0	0	0	0	0	0	0	0	0	0	0	0	0	0	0	0	0	0
0	0	0	0	0	0	0	0	0	0	0	0	0	0	0	0	0	0	0
0	0	0	0	0	0	0	0	0	0	0	0	0	0	0	0	0	0	0
0	0	0	0	0	0	0	0	0	0	0	0	0	0	0	0	0	0	5
0	0	0	0	0	0	0	0	0	0	0	0	0	0	0	0	0	0	0
0	0	0	0	0	0	0	0	0	0	0	0	0	0	0	0	0	0	0
0	0	0	0	0	0	0	0	0	0	0	0	0	0	0	0	0	0	3
0	0	0	0	0	0	0	0	0	0	0	0	0	0	0	0	0	0	0
0	0	0	0	0	0	0	0	0	0	0	0	0	0	0	0	0	0	0
0	0	0	0	0	0	0	0	0	0	0	0	0	0	0	0	0	0	0
0	0	0	0	0	0	0	0	0	0	0	0	0	0	0	0	0	0	0
0	0	0	0	0	0	0	0	0	0	0	0	0	0	0	0	0	0	0
0	0	0	0	0	0	0	0	0	0	0	0	4	0	0	0	0	0	0
0	0	0	0	0	0	0	0	0	0	0	0	0	0	0	0	0	0	0
0	0	0	0	0	0	0	0	0	0	0	0	0	0	0	0	0	0	0
0	0	0	0	0	0	0	0	0	0	0	0	0	0	0	0	0	0	0
0	0	0	0	0	0	0	0	0	0	0	0	0	0	0	0	0	0	0
0	0	0	0	0	0	0	0	0	0	0	0	0	0	0	0	0	0	0

0	0	0	0	0	0	0	0	0	0	0	0	0	0	0	0	0	0	0
0	0	0	0	0	0	0	0	0	0	0	0	0	0	0	0	0	0	0
0	0	0	0	0	0	0	0	0	0	0	0	0	0	0	0	0	0	0
0	0	0	0	0	0	0	0	0	0	0	0	0	0	0	0	0	0	0
0	0	0	0	0	0	0	0	0	0	0	0	0	0	0	0	0	0	0
0	0	0	0	0	0	0	0	0	0	0	0	0	0	0	0	0	0	0
0	0	0	0	0	0	0	0	0	0	0	0	0	0	0	0	0	0	0
0	0	0	0	0	0	0	0	0	0	0	0	0	0	0	0	0	3	3
0	0	0	0	0	0	0	0	0	0	0	0	0	0	0	0	0	0	0
0	0	0	0	0	0	0	0	0	0	0	0	0	0	0	0	0	0	0
0	0	0	0	0	0	0	0	0	0	0	0	0	0	0	0	0	0	0
0	0	0	0	0	0	0	0	0	0	0	0	0	0	0	0	0	0	0
0	0	0	0	0	0	0	0	0	0	0	0	0	0	0	0	0	0	0
0	0	0	0	0	0	0	0	0	0	0	0	0	0	0	0	0	0	0
0	0	0	0	0	0	0	0	0	0	0	0	0	0	0	0	0	0	0
0	0	0	0	0	0	0	0	0	0	0	0	0	0	0	0	0	0	0
0	0	0	0	0	0	0	0	0	0	0	0	0	0	0	0	0	0	0
0	0	0	0	0	0	0	0	0	0	0	0	0	0	0	0	0	0	0
0	0	0	0	0	0	0	0	0	0	0	0	0	0	0	0	0	0	0
0	0	0	0	0	0	0	0	0	0	0	0	0	0	0	0	0	0	0
0	0	0	0	0	0	0	0	0	0	0	0	0	0	0	0	0	0	0
0	0	0	0	0	0	0	0	0	0	0	0	0	0	0	0	0	0	0
0	0	0	0	0	0	0	0	0	0	0	0	0	0	0	0	0	0	0
0	0	0	0	0	0	0	0	0	0	0	0	0	0	0	0	0	0	0
0	0	0	0	0	0	0	0	0	0	0	0	0	0	0	0	0	0	0
0	0	0	0	0	0	0	0	0	0	0	0	0	0	0	0	0	0	0
0	0	0	0	0	0	0	0	0	0	0	0	0	0	0	0	0	0	0
0	0	0	0	0	0	0	0	0	0	0	0	0	0	0	0	0	0	0
0	0	0	0	0	0	0	0	0	0	0	0	0	0	0	0	0	0	0
0	0	0	0	0	0	0	0	0	0	0	0	0	0	0	0	0	0	0
0	2	2	2	2	0	0	0	0	0	0	0	0	0	0	0	0	0	0
0	0	0	0	0	0	0	0	0	0	0	0	0	0	0	0	0	0	0
0	0	0	0	0	0	0	0	0	0	0	0	0	0	0	0	0	0	0
0	0	0	0	0	0	0	0	0	0	0	0	0	0	0	0	0	0	0
0	0	0	0	0	0	0	0	0	0	0	0	0	0	0	0	0	0	0
0	0	0	0	0	0	0	0	0	0	0	0	0	0	0	0	0	0	0
0	0	0	0	0	0	0	0	0	0	0	0	0	0	0	0	0	0	0

0	0	0	0	0	0	0	0	0	0	0	0	0	0	0	0	0	0	0
0	0	0	0	0	0	0	0	0	0	0	0	0	0	0	0	0	0	0
0	0	0	0	0	0	0	0	0	0	0	0	0	0	0	0	0	0	0
0	0	0	0	0	0	0	0	0	0	0	0	0	0	0	0	0	0	0
0	0	0	0	0	0	0	0	0	0	0	0	0	0	0	0	0	0	0
0	0	0	0	0	0	0	0	0	0	0	0	0	0	0	0	0	0	0
0	0	0	0	0	0	0	0	0	0	0	0	0	0	0	0	0	0	0
0	0	0	0	0	0	0	0	0	0	0	0	0	0	0	0	0	0	0
0	0	0	0	0	0	0	0	0	0	0	0	0	0	0	0	0	0	0
0	0	0	0	0	0	0	0	0	0	0	0	0	0	0	0	0	0	0
0	0	0	0	0	0	0	0	0	0	0	0	0	0	0	0	0	0	0
0	0	0	0	0	0	0	0	0	0	0	0	0	0	0	0	0	0	0
0	0	0	0	0	0	0	0	0	0	0	0	0	0	0	0	0	0	0
0	0	0	0	0	0	0	0	0	0	0	0	0	0	0	0	0	0	0
0	0	0	0	0	0	0	0	0	0	0	0	0	0	0	0	0	0	0
0	0	0	0	0	0	0	0	0	0	0	0	0	0	0	0	0	0	0
0	0	0	0	0	0	0	0	0	0	0	0	0	0	0	0	0	0	0
0	0	0	0	0	0	0	0	0	0	0	0	0	0	0	0	0	0	0
0	0	0	0	0	0	0	0	0	0	0	0	0	0	0	0	0	0	0
0	0	0	0	0	0	0	0	0	0	0	0	0	0	0	0	0	0	0
0	0	0	0	0	0	0	0	0	0	0	0	0	0	0	0	0	0	0
0	0	0	0	0	0	0	0	0	0	0	0	0	0	0	0	0	0	0
0	0	0	0	0	0	0	0	0	0	0	0	0	0	0	0	0	0	0
0	0	0	0	0	0	0	0	0	0	0	0	0	0	0	0	0	0	0
0	0	0	0	0	0	0	0	0	0	0	0	0	0	0	0	0	0	0
0	0	0	0	0	0	0	0	0	0	0	0	0	0	0	0	0	0	0
0	0	0	0	0	0	0	0	0	0	0	0	0	0	0	0	0	0	0
0	0	0	0	0	0	0	0	0	0	0	0	0	0	0	0	0	0	0
0	0	0	0	0	0	0	0	0	0	0	0	0	0	0	0	0	0	0
0	0	0	0	0	0	0	0	0	0	0	0	0	0	0	0	0	0	0
0	0	0	0	0	0	0	0	0	0	0	0	0	0	0	0	0	0	0
0	0	0	0	0	0	0	0	0	0	0	0	0	0	0	0	0	0	0
0	0	0	0	0	0	0	0	0	0	0	0	0	0	0	0	0	0	0
0	0	0	0	0	0	0	0	0	0	0	0	0	0	0	0	0	0	0
0	0	0	0	0	0	0	0	0	0	0	0	0	0	0	0	0	0	0
0	0	0	0	0	0	0	0	0	0									

Jan_DL062	1985	843	411	144	2565	259	314	283	363	308	34	607	236	495	166	610	614	206
270	0	118	0	117	126	198	240	200	115	208	710	137	174	98	0	183	0	459
45	0	132	30	85	0	67	8	91	243	179	0	9	163	67	36	257	165	390
342	0	0	82	135	0	37	30	34	80	0	68	7	0	0	32	574	0	0
115	0	39	0	182	161	72	36	48	18	0	81	76	13	15	85	0	35	0
115	72	0	0	0	127	0	0	0	0	135	85	69	0	27	24	81	0	0
0	82	0	13	58	0	0	0	0	0	33	0	48	69	65	0	69	0	0
0	0	103	23	64	0	0	0	51	32	0	0	12	70	28	11	0	0	0
0	32	0	14	0	27	74	0	0	0	0	0	27	0	0	0	5	25	0
38	0	5	0	0	0	0	0	0	0	32	27	0	0	160	0	0	0	0
0	17	0	0	0	0	50	58	0	10	121	0	0	0	48	0	0	35	0
22	0	0	0	36	0	0	0	0	11	0	44	47	0	0	0	0	0	0
0	0	21	0	0	0	0	0	0	0	0	0	0	0	0	97	0	0	0
35	0	0	9	0	0	0	35	0	15	68	0	0	44	0	0	0	0	62
0	21	0	11	28	13	0	0	0	0	0	0	0	33	8	12	0	0	0
0	16	0	0	0	0	0	23	0	0	0	0	0	0	11	65	0	0	0
0	0	0	0	0	0	0	0	0	36	0	0	9	0	0	8	0	2	6
0	0	0	0	0	0	38	0	10	0	0	0	22	16	0	7	0	0	0
0	0	0	0	0	0	0	0	0	13	0	0	0	0	0	0	0	0	0
0	0	0	0	0	0	3	0	0	0	0	0	0	0	38	0	41	0	44
0	0	0	0	0	10	0	4	29	0	0	0	16	0	6	0	0	0	0
0	0	0	0	0	0	0	0	0	0	56	0	0	0	0	0	0	0	0
0	0	0	0	0	0	68	0	15	0	0	0	4	0	0	0	0	0	0
0	4	0	0	0	0	0	0	0	0	0	0	0	0	0	0	16	0	9
0	0	0	0	0	0	0	0	0	0	0	0	0	15	0	0	0	0	0
0	0	0	0	0	0	0	0	0	11	0	21	0	0	0	0	0	0	0
0	0	0	0	0	0	0	0	0	21	0	0	0	0	0	0	34	3	0
0	0	0	0	0	0	5	10	0	0	0	0	0	0	0	0	0	0	0
19	0	0	0	0	0	0	0	0	12	0	0	0	0	0	20	0	0	25
0	0	11	0	17	0	0	13	0	0	10	0	0	0	0	0	0	0	0
0	9	0	4	0	0	0	0	0	0	0	0	0	0	22	0	0	0	8
0	0	0	0	0	0	0	0	0	0	68	0	0	0	0	0	0	0	0
0	0	0	0	9	0	0	0	0	0	0	0	0	0	0	0	0	0	0
0	0	0	0	0	0	0	0	0	0	8	0	0	0	0	0	0	0	0
0	0	0	0	0	0	0	0	0	0	21	0	0	0	0	0	0	0	0
0	0	0	0	0	0	0	0	0	0	0	0	0	0	0	0	0	0	0
0	0	0	0	0	0	0	0	0	0	0	0	0	33	0	0	0	34	0

0	0	0	0	10	0	0	0	13	0	0	0	0	0	0	0	0	0	0
0	0	0	0	0	0	35	0	0	0	0	0	0	0	14	0	0	0	0
0	0	0	0	0	0	0	0	0	0	0	0	0	0	0	0	0	0	0
0	0	0	0	0	0	0	0	0	17	0	0	0	0	0	0	0	0	0
0	6	0	0	0	0	0	0	0	0	0	0	0	12	0	0	0	0	0
0	9	0	10	0	0	0	0	0	7	0	0	22	0	0	0	0	19	9
0	0	0	0	0	0	0	0	0	0	0	0	0	25	0	0	0	0	0
0	0	0	0	0	0	0	0	0	0	0	0	0	0	0	0	0	0	0
0	0	0	0	0	0	13	0	0	0	0	0	0	0	0	0	0	0	0
0	0	0	0	0	0	0	0	0	0	0	0	0	0	0	0	0	0	0
0	0	0	0	0	0	0	0	0	0	0	0	0	0	0	0	0	0	0
0	0	0	0	0	0	0	0	5	0	0	0	0	0	0	0	0	0	0
0	0	0	0	0	0	0	7	0	0	0	0	0	0	0	5	0	0	6
0	0	0	0	0	0	0	0	0	0	0	0	0	0	0	0	0	0	0
0	0	0	0	0	0	0	0	0	0	0	0	0	0	0	10	0	0	0
0	12	0	0	0	0	0	0	0	0	0	0	0	0	0	0	0	0	0
0	0	0	0	0	0	0	0	0	0	0	0	0	0	0	0	0	0	0
0	0	0	0	0	0	0	0	0	0	0	0	0	0	0	0	0	0	0
0	0	0	0	0	0	0	0	0	0	0	0	0	0	0	0	0	0	0
0	0	0	0	0	0	0	0	0	0	0	0	0	0	0	0	0	0	0
0	0	0	0	0	0	0	0	0	0	0	0	0	0	0	0	0	0	0
0	0	0	0	0	0	0	0	0	0	0	0	0	0	0	0	0	0	0
0	0	0	0	0	0	0	0	0	0	0	0	0	0	0	0	0	0	0
0	0	0	0	0	0	0	0	0	0	0	0	0	0	0	0	0	0	0
0	0	0	0	0	0	0	0	0	0	0	0	0	0	0	0	0	0	0
0	0	0	0	0	0	0	0	0	0	0	0	0	0	0	0	0	0	0
0	0	0	0	0	0	0	0	0	0	0	0	0	0	0	0	0	0	0
0	0	0	0	0	0	0	0	0	0	0	0	0	0	0	0	0	0	0
0	0	0	0	0	0	6	0	0	0	0	0	0	0	0	0	0	0	0
0	0	0	0	0	0	0	0	0	0	0	0	0	0	0	0	0	0	0
0	0	0	0	0	0	0	0	20	0	0	0	0	0	0	0	0	0	0
0	0	0	0	0	0	0	0	0	0	0	0	0	0	0	0	0	0	0
0	0	0	0	0	0	0	0	0	0	0	0	0	0	0	0	0	0	0
0	0	0	0	0	0	0	0	0	0	0	0	0	0	0	0	0	0	0
0	0	0	0	0	0	0	0	0	0	0	0	0	0	0	0	0	0	0
0	0	0	0	0	0	0	0	0	0	0	0	0	0	0	0	0	0	0
0	0	0	0	0	0	0	0	0	0	0	0	0	0	0	0	0	0	0

0	0	0	0	0	0	0	0	0	0	15	0	0	0	0	0	0	0	0
0	0	0	0	0	0	0	0	0	0	0	0	0	0	0	0	0	0	0
0	0	0	0	0	0	0	0	0	14	0	0	0	0	0	0	0	0	0
0	0	0	0	0	0	0	0	0	0	0	0	0	0	0	0	0	0	0
0	0	0	0	0	0	0	0	0	0	0	0	0	0	0	0	0	0	0
0	0	0	0	0	0	0	0	7	0	0	0	0	0	0	0	0	0	0
0	0	0	0	0	0	0	0	0	0	0	0	0	0	0	0	0	0	0
0	0	0	0	0	0	0	0	0	0	0	0	0	0	0	0	0	0	0
0	0	0	0	0	0	0	0	0	0	0	0	0	0	0	0	0	0	0
0	0	8	0	0	0	0	0	0	0	0	0	0	0	0	0	0	0	0
0	0	0	0	0	0	0	0	0	0	0	0	0	0	0	0	0	0	0
0	0	0	0	0	0	0	0	0	0	0	0	0	0	0	0	0	0	0
0	0	0	0	0	0	0	0	0	0	0	0	0	0	0	0	0	0	0
0	0	0	0	0	0	3	0	0	0	0	0	0	0	0	0	0	0	0
0	0	0	0	0	0	0	0	0	0	0	0	0	0	0	0	0	0	0
0	0	0	0	0	0	0	0	0	0	0	0	0	0	0	0	0	0	0
0	0	0	0	0	0	0	0	0	0	0	0	0	0	0	0	0	0	0
0	0	0	0	0	0	0	0	0	0	0	0	0	0	0	0	0	0	0
0	0	0	0	0	0	0	0	0	0	0	0	0	0	0	0	0	0	0
0	0	0	0	0	0	0	0	0	0	0	0	0	0	0	0	0	0	0
0	0	0	0	0	0	0	0	0	0	0	0	0	0	0	0	0	0	0
0	0	0	0	0	0	0	0	0	0	0	0	0	0	0	0	0	0	0
0	0	0	0	0	0	0	0	0	0	0	0	0	0	0	0	0	0	0
0	0	0	0	0	0	0	0	0	0	0	0	0	0	0	0	0	0	0
0	0	0	0	0	0	0	0	0	0	0	0	0	0	0	0	0	0	0
0	0	0	0	0	0	0	0	0	0	0	0	0	0	0	0	0	0	0
0	0	0	0	0	0	0	0	0	0	0	0	0	0	0	0	0	0	0
0	0	0	0	0	0	0	0	0	0	0	0	0	0	0	0	0	0	0
0	0	0	0	0	0	0	0	0	0	0	0	0	0	0	0	0	0	0
0	0	0	0	0	0	0	0	0	0	0	0	0	0	0	0	0	0	0
0	0	0	0	0	0	0	0	0	0	0	0	0	0	0	0	0	0	0
0	0	0	0	0	0	0	0	0	0	0	0	0	0	0	0	0	0	0
0	0	0	0	0	0	0	0	0	0	0	0	0	0	0	0	0	0	0
0	0	0	0	0	0	0	0	0	0	0	0	0	0	0	0	0	0	0
0	0	0	0	0	0	0	0	0	0	0	0	0	0	0	0	0	0	0
0	0	0	0	0	0	0	0	0	0	0	0	0	0	0	0	0	0	0
0	0	0	0	0	0	0	0	0	0	0	0	0	0	0	0	0	0	0

0	0	0	0	0	0	0	0	0	0	0	0	0	0	0	0	0	0	0
0	0	0	0	0	0	0	0	0	0	0	0	0	0	0	0	0	0	0
0	0	0	0	0	0	0	0	0	0	0	0	0	0	0	0	0	0	0
0	0	0	0	0	0	0	0	0	0	0	0	0	0	0	0	0	0	0
0	0	0	0	0	0	0	0	0	0	0	0	0	0	0	0	0	0	0
0	0	0	0	0	0	0	0	0	0	0	0	0	0	0	0	0	0	0
0	0	0	0	0	0	0	0	0	0	0	0	0	0	0	0	0	0	0
0	0	0	0	0	0	0	0	0	0	0	0	0	0	0	0	0	0	0
0	0	0	0	0	0	0	0	0	0	0	0	0	0	0	0	0	0	0
0	0	0	0	0	0	0	0	0	0	0	0	0	0	0	0	0	0	0
0	0	0	0	0	0	0	0	0	0	0	0	0	0	0	0	0	0	0
0	0	0	0	0	0	0	0	0	0	0	0	0	0	0	0	0	0	0
0	0	0	0	0	0	0	0	0	0	0	0	0	0	0	0	0	0	0
0	0	0	0	0	0	0	0	0	0	0	0	0	0	0	0	0	0	0
0	0	0	0	0	0	0	0	0	0	0	0	0	0	0	0	0	0	0
0	0	0	0	0	0	0	0	0	0	0	0	0	0	0	0	0	0	0
0	0	0	0	0	0	0	0	0	0	0	0	0	0	0	0	0	0	0
0	0	0	0	0	0	0	0	0	0	0	0	0	0	0	0	0	0	0
0	0	0	0	0	0	0	0	0	0	0	0	0	0	0	0	0	0	0
0	0	0	0	0	0	0	0	0	0	0	0	0	0	0	0	0	0	0
0	0	0	0	0	0	0	0	0	0	0	0	0	0	0	0	0	0	0
0	0	0	0	0	0	0	0	0	0	0	0	0	0	0	0	0	0	0
0	0	0	0	0	0	0	0	0	0	0	0	0	0	0	0	0	0	0
0	0	0	0	0	0	0	0	0	0	0	0	0	0	0	0	0	0	0
0	0	0	0	0	0	0	0	0	0	0	0	0	0	0	0	0	0	0
0	0	0	0	0	0	0	0	0	0	0	0	0	0	0	0	0	0	0
0	0	0	0	0	0	0	0	0	0	0	0	0	0	0	0	0	0	0
0	0	0	0	0	0	0	0	0	0	0	0	0	0	0	0	0	0	0
0	0	0	0	0	0	0	0	0	0	0	0	0	0	0	0	0	0	0
0	0	0	0	0	0	0	0	0	0	0	0	0	0	0	0	0	0	0
0	0	0	0	0	0	0	0	0	0	0	0	0	0	0	0	0	0	0
0	0	0	0	0	0	0	0	0	0	0	0	0	0	0	0	0	0	0
0	0	0	0	0	0	0	0	0	0	0	0	0	0	0	0	0	0	0
0	0	0	0	0	0	0	0	0	0	0	0	0	0	0	0	0	0	0
0	0	0	0	0	0	0	0	0	0	0	0	0	0	0	0	0	0	0
0	0	0	0	0	0	0	0	0	0	0	0	0	0	0	0	0	0	0
0	0	0	0	0	0	0	0	0	0	0	0	0	0	0	0	0	0	0

0	0	0	0	0	0	0	0	0	0	0	0	0	0	0	0	0	0	0
0	0	0	0	0	0	0	0	0	0	0	0	0	0	0	0	0	0	0
0	0	0	0	0	0	0	0	0	0	0	0	0	0	0	0	0	0	0
0	0	0	0	0	0	0	0	0	0	0	0	0	0	0	0	0	0	0
0	0	0	0	0	0	0	0	0	0	0	0	0	0	0	0	0	0	0
0	0	0	0	0	0	0	0	0	0	0	0	0	0	0	0	3	3	3
3	3	0	0	0	0	0	0	0	0	0	0	0	0	0	0	0	0	0
0	0	0	0	0	0	0	0	0	0	0	0	0	0	0	0	0	0	0
0	0	0	0	0	0	0	0	0	0	0	0	0	0	0	0	0	0	0
0	0	0	0	0	0	0	0	0	0	0	0	0	0	0	0	0	0	0
0	0	0	0	0	0	0	0	0	0	0	0	0	0	0	0	0	0	0
0	0	0	0	0	0	0	0	0	0	0	0	0	0	0	0	0	0	0
0	0	0	0	0	0	0	0	0	0	0	0	0	0	0	0	0	0	0
0	0	0	0	0	0	0	0	0	0	0	0	0	0	0	0	0	0	0
0	0	0	0	0	0	0	0	0	0	0	0	0	0	0	0	0	0	0
0	0	0	0	0	0	0	0	0	0	0	0	0	0	0	0	0	0	0
0	0	0	0	0	0	0	0	0	0	0	0	0	0	0	0	0	0	0
0	0	0	0	0	0	0	0	0	0	0	0	0	0	0	0	0	0	0
0	0	0	0	0	0	0	0	0	0	0	0	0	0	0	0	0	0	0
0	0	0	0	0	0	0	0	0	0	0	0	0	0	0	0	0	0	0
0	0	0	0	0	0	0	0	0	0	0	0	0	0	0	0	0	0	0
0	0	0	0	0	0	0	0	0	0	0	0	0	0	0	0	0	0	0
0	0	0	0	0	0	0	0	0	0	0	0	0	0	0	0	0	0	0
0	0	0	0	0	0	0	0	0	0	0	0	0	0	0	0	0	0	0
0	0	0	0	0	0	0	0	0	0	0	0	0	0	0	0	0	0	0
0	0	0	2	2	0	0	0	0	0	0	0	0	0	0	0	0	0	0
0	0	0	0	0	0	0	0	0	0	0	0	0	0	0	0	0	0	0
0	0	0	0	0	0	0	0	0	0	0	0	0	0	0	0	0	0	0
0	0	0	0	0	0	0	0	0	0									
Jan_DL063	1947	1822	456	244	626	717	526	421	133	235	122	776	162	185	132	34	216	176
330	1070	347	0	68	319	195	173	239	0	61	0	126	106	72	0	127	21	0
26	157	72	44	68	48	192	0	17	14	60	0	187	60	0	0	0	45	0
0	27	0	179	83	0	32	33	46	116	10	195	838	0	44	0	66	33	10
97	0	0	11	86	95	80	0	0	0	0	0	70	0	29	0	70	66	0
42	0	0	0	67	28	0	7	16	30	0	0	0	64	27	55	0	0	0
0	0	27	20	34	0	0	0	0	0	33	110	15	27	51	0	0	0	0
0	35	0	0	0	0	0	0	26	0	14	0	8	0	0	0	0	0	0

0	31	88	0	0	17	0	0	0	0	0	0	0	108	28	0	0	28	34
18	0	11	36	0	60	0	0	0	0	30	11	0	0	0	71	55	0	0
0	0	0	0	0	0	0	50	0	66	0	21	0	0	13	0	0	0	0
18	0	0	0	0	78	12	0	0	29	0	0	0	0	0	0	112	14	15
0	0	19	0	0	25	0	16	0	0	0	0	17	0	0	17	0	0	0
0	0	0	0	0	0	0	0	0	0	0	0	28	6	7	21	0	0	0
24	0	0	0	9	9	9	0	6	0	0	0	0	23	15	23	0	0	0
0	50	16	12	0	0	0	15	0	0	0	19	0	0	0	13	0	9	0
0	37	0	0	0	0	0	0	0	0	0	0	41	9	0	0	0	33	0
31	0	0	3	0	0	0	5	6	0	0	0	0	0	0	0	12	0	0
7	0	0	0	0	0	0	0	14	0	0	0	0	0	0	0	16	0	36
0	0	15	0	9	0	6	8	0	0	20	0	0	56	0	0	0	0	0
0	0	0	73	9	18	24	0	0	0	0	0	0	0	5	0	0	0	0
0	0	0	57	13	0	0	5	0	0	0	0	0	0	0	0	7	32	0
0	63	0	0	0	0	0	0	0	0	0	87	0	0	0	0	0	0	0
0	0	0	0	0	0	0	0	0	0	0	0	0	0	0	0	0	0	0
8	0	0	0	10	0	0	0	0	0	0	0	0	0	0	0	0	0	0
0	0	0	0	0	0	5	5	0	14	0	0	0	10	0	0	0	0	0
12	0	13	0	0	8	0	0	0	0	0	0	0	0	0	0	0	0	0
0	0	0	0	0	12	0	0	0	0	0	0	0	0	0	0	0	0	0
0	0	0	0	0	0	0	0	0	0	0	0	0	0	0	0	0	0	0
0	0	0	0	0	0	0	16	0	0	0	0	0	0	0	0	0	0	0
0	8	0	6	0	17	0	0	0	0	0	0	0	0	0	0	0	0	0
19	0	0	0	0	0	0	0	0	0	0	0	0	0	0	0	0	0	0
0	0	0	0	0	33	0	0	0	0	0	0	0	0	0	0	0	0	0
48	0	0	0	0	0	0	0	0	0	0	0	0	0	0	0	0	0	0
0	0	0	0	19	0	0	0	0	9	0	6	0	0	0	10	0	0	0
0	0	0	0	0	0	0	0	0	0	0	0	0	0	10	0	0	0	0
0	0	0	0	0	0	0	0	0	0	0	0	0	0	0	25	0	0	0
0	0	0	0	0	0	0	0	0	0	0	0	0	0	0	0	0	0	0
0	0	0	0	0	0	0	0	0	0	0	0	0	0	0	0	0	0	0
0	5	0	0	0	0	0	0	0	0	0	0	0	0	0	0	0	0	0
0	0	22	0	0	0	0	12	0	0	0	0	0	0	0	0	0	9	0
0	0	0	0	0	7	0	0	0	0	0	0	0	0	0	0	0	0	0
0	12	0	0	0	0	0	0	0	0	13	0	0	0	0	0	0	0	0
0	0	0	0	0	0	0	0	0	0	0	0	0	0	0	0	0	0	0
0	0	0	0	0	0	0	0	0	0	0	3	0	0	0	0	0	0	0

0	0	0	0	0	0	0	0	7	0	0	0	0	0	0	0	0	0	0
0	0	0	0	0	0	0	0	0	0	0	0	0	0	0	0	0	0	0
0	0	0	0	0	0	0	0	0	0	0	0	0	0	0	0	0	0	0
0	36	0	9	0	0	0	0	0	11	0	0	0	0	0	0	0	0	0
0	0	8	0	0	0	0	0	0	0	0	0	0	0	0	0	0	0	9
0	0	0	0	0	0	0	0	0	0	0	0	0	0	0	0	0	0	0
0	0	0	0	0	0	0	0	0	0	0	0	0	0	0	0	0	0	0
0	0	0	0	0	0	0	0	0	0	0	0	0	0	0	0	0	0	0
0	0	0	0	0	0	0	0	0	0	0	0	0	0	0	0	0	0	0
0	0	0	0	0	0	0	0	0	0	0	0	0	0	0	0	0	0	0
0	0	0	0	0	0	0	0	0	0	0	0	0	0	0	9	0	0	0
0	0	0	0	0	0	0	0	0	0	0	0	0	0	0	0	0	0	0
0	0	0	0	0	0	0	0	0	0	0	0	0	0	0	0	0	0	0
0	0	0	0	0	0	0	0	16	0	0	0	0	0	0	0	0	0	0
0	0	0	0	0	0	0	0	0	0	0	0	0	0	0	0	0	0	0
0	0	0	0	0	0	0	0	0	0	0	0	0	0	0	0	0	0	0
0	0	0	0	0	0	0	0	0	0	0	0	0	0	0	0	0	0	0
0	0	0	0	0	0	0	0	0	0	0	0	0	0	0	0	0	0	0
0	0	0	0	0	0	0	0	0	0	0	0	0	0	0	0	0	0	0
0	0	0	0	0	0	0	0	0	19	0	0	0	0	0	0	0	0	0
0	0	0	0	0	0	0	0	0	0	0	0	0	0	0	0	0	0	0
0	0	0	0	0	0	0	0	0	0	0	0	0	0	0	0	0	0	0
0	0	0	0	0	0	0	0	0	0	0	0	0	0	0	0	0	0	0
0	0	0	0	0	0	0	0	0	0	0	0	0	0	0	0	0	0	0
0	0	0	0	0	0	0	0	0	0	0	0	0	0	0	0	0	0	0
0	0	0	0	0	0	0	0	0	0	3	0	0	0	0	0	0	0	0
0	0	0	0	0	0	0	0	0	0	0	0	0	0	0	0	0	0	0
0	0	0	0	0	0	0	0	0	0	0	0	0	0	0	0	0	0	0
0	0	0	0	0	0	0	0	0	0	0	0	0	0	0	0	0	0	0
0	0	0	0	0	0	0	0	0	0	0	0	0	0	0	0	0	0	0
0	0	0	0	0	0	0	0	0	0	0	0	0	0	0	0	0	0	0
0	0	0	0	0	0	0	0	0	0	0	0	0	0	0	0	0	0	0
0	0	0	0	0	0	0	0	0	0	0	0	0	0	0	0	0	0	0
0	0	0	0	0	0	0	0	0	0	0	0	0	0	0	11	0	0	0
0	0	0	0	0	0	0	0	0	0	0	0	0	0	0	0	0	0	0
0	0	0	0	0	0	0	0	0	0	0	0	0	0	0	0	0	0	0
0	0	0	0	0	0	0	0	0	0	0	0	0	0	0	0	0	0	0

0	0	0	0	0	0	0	0	0	0	0	0	0	0	0	0	0	0	0
0	0	0	0	0	0	0	0	0	0	0	0	0	0	0	0	0	0	0
0	0	0	0	0	0	0	0	0	0	0	0	0	0	0	0	0	0	0
0	0	0	0	0	0	0	0	0	0	0	0	0	0	0	0	0	0	0
0	0	0	0	0	0	0	0	0	0	0	0	0	0	0	0	0	0	0
0	0	0	0	0	0	0	0	0	0	0	0	0	0	0	0	0	0	0
0	0	0	0	0	0	0	0	0	0	0	0	0	0	0	0	0	0	0
0	0	0	0	0	0	0	0	0	0	0	0	0	0	0	0	0	0	0
0	0	0	0	0	0	0	0	0	0	0	0	0	0	0	0	0	0	0
0	0	0	0	0	0	0	0	0	0	0	0	0	0	0	0	0	0	0
0	0	0	0	0	0	0	0	0	0	0	0	0	0	0	0	0	0	0
0	0	0	0	0	0	0	0	0	0	0	0	0	0	0	0	0	0	0
0	0	0	0	0	0	0	0	0	0	0	0	0	0	0	0	0	0	0
0	0	0	0	0	0	0	0	0	0	0	0	0	0	0	0	0	0	0
0	7	0	0	0	0	0	0	0	0	0	0	0	0	0	0	0	0	0
0	0	0	0	0	0	0	0	0	0	0	0	0	0	0	0	0	0	0
0	0	0	0	0	0	0	0	0	0	0	0	0	0	0	0	0	0	0
0	0	0	0	0	0	0	0	0	0	0	0	0	0	0	0	0	0	0
0	0	0	0	0	0	0	0	0	0	0	0	0	0	0	0	0	0	0
0	0	0	0	0	0	0	0	0	0	0	0	0	0	0	0	0	0	0
0	0	0	0	0	0	0	0	0	0	0	0	0	0	0	0	0	0	0
0	0	0	0	0	0	0	0	0	0	0	0	0	0	0	0	6	0	0
0	0	0	0	0	0	0	0	0	0	0	0	0	0	0	0	0	0	0
0	0	0	0	0	0	0	0	0	0	0	0	0	0	0	0	0	0	0
0	0	0	0	0	0	0	0	0	0	0	0	0	0	0	0	0	0	0
0	0	0	0	0	0	0	0	0	0	0	0	0	0	0	0	0	0	0
0	0	0	0	0	0	0	0	0	0	0	0	0	0	0	0	0	0	0
0	0	0	0	0	0	0	0	0	0	0	0	0	0	0	0	0	0	0
0	0	0	0	0	0	0	0	0	0	0	0	0	0	0	0	0	0	0
0	0	0	0	0	0	0	0	0	0	0	0	0	0	0	0	0	0	0
0	0	0	0	0	0	0	0	0	0	0	0	0	0	0	0	0	0	0
0	0	0	0	0	0	0	0	0	0	0	0	0	0	0	0	0	0	0
0	0	0	0	0	0	0	0	0	0	0	0	0	0	0	0	0	0	0
0	0	0	0	0	0	0	0	0	0	0	0	0	0	0	0	0	0	0
0	0	0	0	0	0	0	0	0	0	0	0	0	0	0	0	0	0	0
0	0	0	0	0	0	0	0	0	0	0	0	0	0	0	0	0	0	0
0	0	0	0	0	0	0	0	0	0	0	0	0	0	0	0	0	0	0

0	0	0	0	0	0	0	0	0	0	0	0	0	0	0	0	0	0	0
0	0	0	0	0	0	0	0	0	0	0	0	0	0	0	0	0	0	0
0	0	0	0	0	0	0	0	0	0	0	0	0	0	0	0	0	0	0
0	0	0	0	0	0	0	0	0	0	0	0	0	0	0	0	0	0	0
0	0	0	0	0	0	0	0	0	0	0	0	0	0	0	0	0	0	0
0	0	0	0	0	0	4	0	0	0	0	0	0	0	0	0	0	0	0
0	0	0	0	0	0	0	0	0	0	0	0	0	0	0	0	0	0	0
0	0	0	0	0	0	0	0	0	0	0	0	0	0	0	0	0	0	0
0	0	0	0	0	0	0	0	0	0	0	0	0	0	0	0	0	0	0
0	0	0	0	0	0	0	0	0	0	0	0	0	0	0	0	0	0	0
0	0	0	0	0	0	0	0	0	0	0	0	0	0	0	0	0	0	0
0	0	0	0	0	0	0	0	0	0	0	0	0	0	0	0	0	0	0
0	0	0	0	0	0	0	0	0	0	0	0	0	0	0	0	0	0	0
0	0	0	0	0	0	0	0	0	0	0	0	0	0	0	0	0	0	0
0	0	0	0	0	0	0	0	0	0	0	0	0	0	0	0	0	0	0
0	0	0	0	0	0	0	0	0	0	0	0	0	0	0	0	0	0	0
0	0	0	0	0	0	0	0	0	0	0	0	0	0	0	0	0	0	0
0	0	0	0	0	0	0	0	0	0	0	0	0	0	0	0	0	0	0
0	0	0	0	0	0	0	0	0	0	0	0	0	0	0	0	0	0	0
0	0	0	0	0	0	0	0	0	0	0	0	0	0	0	0	0	0	0
0	0	0	0	0	0	0	0	0	0	0	0	0	0	0	0	0	0	0
0	0	0	0	0	0	0	0	0	0	0	0	0	0	0	0	0	0	0
0	0	0	0	0	0	0	0	0	0	0	0	0	0	0	3	0	0	0
0	0	0	0	0	0	0	0	0	0	0	0	0	0	0	0	0	0	0
0	0	0	0	0	0	0	0	0	0	0	0	0	0	0	0	0	0	0
0	0	0	0	0	0	0	0	0	0	0	0	0	0	0	0	0	0	0
0	0	0	0	0	0	0	0	0	0	0	0	0	0	0	0	0	0	0
0	0	0	0	0	0	0	0	0	0	0	0	0	0	0	0	0	0	0
0	0	0	0	0	0	0	0	0	0	0	0	0	0	0	0	0	0	0
0	0	0	0	0	0	0	0	0	0	0	0	0	0	0	0	0	0	0
0	0	0	0	0	0	0	0	0	0	0	0	0	0	0	0	0	0	0
0	0	0	0	0	0	0	0	0	0	0	0	0	0	0	0	0	0	0
0	0	0	0	0	0	0	0	0	0	0	0	0	0	0	0	0	0	0
0	0	0	0	0	0	0	0	0	0	0	0	0	0	0	0	0	0	0
0	0	0	0	0	0	0	0	0	0	0	0	0	0	0	0	0	0	0
0	0	0	0	0	0	0	0	0	0	0	0	0	0	0	0	0	0	0
0	0	0	0	0	0	0	0	0	0	0	0	0	0	0	0	0	0	0

0	0	0	0	0	0	0	0	0	0	0	0	0	0	0	0	0	0	0
0	0	0	0	0	0	0	0	0	0	0	0	0	0	0	0	0	0	0
0	0	0	0	0	0	0	0	0	0	0	0	0	0	0	0	0	0	0
0	0	0	0	0	0	0	0	0	0	0	0	0	0	0	0	0	0	0
0	0	0	0	0	0	0	0	0	0	0	0	0	0	0	0	0	0	0
0	0	0	0	0	0	0	0	0	0	0	0	0	0	0	0	0	0	0
0	0	0	0	0	0	0	0	0	0	0	0	0	0	0	0	0	0	0
0	0	0	0	0	0	0	0	0	0	0	0	0	0	0	0	0	0	0
0	0	0	0	0	0	0	0	0	0	0	0	0	0	0	0	0	0	0
0	0	0	0	0	0	0	0	0	0	0	0	0	0	0	0	0	0	0
0	0	0	0	0	0	0	0	0	0	0	0	0	0	0	0	0	0	0
2	2	2	0	0	0	0	0	0	0	0	0	0	0	0	0	0	0	0
0	0	0	0	0	0	0	0	0	0	0	0	0	0	0	0	0	0	0
0	0	0	0	0	0	0	0	0	0	0	0	0	0	0	0	0	0	0
0	0	0	0	0	0	0	0	0	0	0	0	0	0	0	0	0	0	0
0	0	0	0	0	0	0	0	0	0	0	0	0	0	0	0	0	0	0
0	0	0	0	0	0	0	0	0	0	0	0	0	0	0	0	0	0	0
0	0	0	0	0	0	0	0	0	0	0	0	0	0	0	0	0	0	0
0	0	0	0	0	0	0	0	0	0	0	0	0	0	0	0	0	0	0
0	0	0	0	0	0	0	0	0	0	0	0	0	0	0	0	0	0	0
0	0	0	0	0	0	0	0	0	0									
Jan_DL065	1688	1041	40	359	35	226	458	432	492	119	322	342	86	357	158	24	290	290
332	121	244	386	163	191	107	80	160	53	151	0	94	97	57	151	66	84	0
0	78	272	210	133	122	125	20	142	0	129	27	35	186	251	46	130	123	0
0	40	0	24	122	152	78	38	67	36	0	75	0	57	14	87	18	33	0
63	49	96	0	0	54	68	93	48	0	0	0	28	15	0	41	11	26	0
0	0	0	0	0	7	64	43	60	19	0	84	0	0	24	18	16	0	50
109	21	115	16	0	20	0	0	0	72	0	0	0	14	51	59	0	0	0
88	0	93	0	0	0	0	0	0	8	20	0	4	0	0	0	0	0	0
0	15	0	6	61	3	17	17	0	27	24	0	0	0	0	0	0	0	25
0	0	0	72	9	0	0	0	0	15	0	0	0	25	6	23	0	0	0
19	0	0	17	0	0	18	15	22	0	0	12	0	0	0	0	0	24	0
0	0	0	0	0	0	0	0	0	0	0	6	0	22	0	6	0	7	26
32	0	0	0	0	8	0	16	0	0	0	0	0	6	0	11	0	0	0
0	0	0	22	0	0	0	0	0	7	0	0	0	0	0	7	0	9	14
10	0	0	0	0	15	0	0	13	0	25	0	0	7	13	0	0	0	0
0	4	0	0	0	0	0	0	0	0	0	0	0	0	8	9	0	0	0

0	0	16	0	0	32	0	6	0	0	0	7	0	0	0	0	0	0	11
12	13	0	0	0	0	4	0	0	0	0	0	0	0	0	0	0	0	0
0	0	0	0	4	0	0	0	0	0	0	15	0	0	0	0	11	0	0
0	0	0	0	0	19	4	0	0	0	0	0	0	0	0	0	0	0	0
0	0	0	0	0	0	3	0	0	0	0	0	0	0	0	0	0	0	0
0	0	24	0	0	0	0	0	0	0	0	0	0	0	0	0	7	41	0
0	0	0	0	0	0	0	26	0	0	0	0	0	0	8	0	0	0	0
0	0	0	0	0	0	0	0	0	0	0	0	0	0	0	13	7	0	0
0	0	0	0	0	0	0	0	0	0	0	7	0	0	0	0	0	22	0
0	0	0	0	0	0	16	0	0	0	0	0	0	0	0	5	0	0	0
0	0	0	0	0	0	0	0	0	0	0	0	0	0	0	0	7	0	0
0	0	0	0	0	0	0	0	0	0	0	0	0	0	0	0	0	0	0
0	0	0	0	0	0	0	0	0	0	0	0	0	0	0	0	0	0	0
24	0	6	13	0	0	0	0	0	0	6	0	0	0	0	0	0	0	0
0	0	0	0	21	0	0	0	0	0	0	0	0	4	0	0	0	0	0
0	0	0	0	0	0	0	0	0	0	0	0	0	0	0	0	0	0	0
0	0	0	0	0	0	0	0	0	0	0	0	0	0	0	0	0	0	0
0	0	0	0	0	14	0	0	0	0	0	0	0	0	0	9	0	0	0
0	0	0	0	13	0	0	0	0	0	0	0	0	0	0	0	0	0	0
0	0	0	0	0	0	0	0	0	0	0	0	0	0	0	0	0	0	0
8	0	0	0	0	0	0	0	22	0	0	0	0	0	0	0	0	0	0
0	0	0	0	0	0	0	0	0	0	0	0	0	0	0	0	0	0	0
0	0	0	0	0	0	0	0	0	0	0	0	0	17	0	0	0	0	0
0	0	0	0	0	0	0	0	0	0	0	0	0	0	0	0	0	0	0
0	0	0	0	0	0	0	0	0	0	0	0	0	0	0	0	0	0	0
0	0	0	0	0	0	0	0	0	0	0	0	0	0	0	0	0	0	0
0	0	0	0	0	0	0	0	7	0	0	0	0	0	0	0	0	0	0
0	0	0	0	0	0	0	0	0	0	0	0	0	0	0	0	0	0	0
0	0	0	0	0	0	0	0	0	0	0	0	0	0	0	0	0	0	0
0	0	0	0	0	0	0	0	0	0	0	0	0	0	0	0	0	0	0
6	0	0	0	0	0	0	0	0	0	0	0	0	0	0	0	0	0	0
0	0	0	0	0	0	0	0	0	0	0	0	0	0	0	0	0	0	0
0	0	0	0	0	0	0	0	0	0	0	0	0	0	0	0	0	0	0
0	0	0	0	0	0	0	0	0	0	0	0	0	0	0	0	0	0	0
0	0	0	0	0	0	0	0	0	0	0	0	0	0	0	0	0	0	0
0	0	0	0	0	0	0	0	0	0	0	0	0	0	0	0	0	0	0
0	0	0	0	0	0	0	0	0	0	0	0	0	0	0	0	0	0	0

0	0	0	0	0	0	3	0	0	0	0	0	0	0	0	0	0	0	0
0	0	0	0	0	0	0	0	0	0	0	0	0	0	0	0	0	0	0
0	0	0	0	0	0	0	0	0	0	0	0	0	0	0	0	0	0	0
0	0	0	0	0	0	0	0	0	0	0	0	4	0	0	0	0	0	0
0	0	0	0	0	0	0	0	0	0	0	0	0	0	0	0	0	0	0
0	0	0	0	0	0	0	0	0	0	0	0	0	0	0	0	0	0	0
0	0	0	0	3	0	0	0	0	0	0	0	0	0	0	0	0	0	0
0	0	0	0	0	0	0	0	0	0	0	0	0	0	0	0	0	0	0
0	0	0	0	0	0	0	0	0	0	0	0	0	0	0	0	0	0	0
0	0	0	0	0	0	0	0	0	0	0	0	0	0	0	0	0	0	0
0	0	0	0	0	0	0	0	0	0	0	0	0	0	0	10	0	0	0
0	0	0	0	0	0	0	0	0	0	0	0	0	0	0	0	0	0	0
0	0	0	0	0	0	0	0	0	0	0	0	0	0	0	0	0	0	0
0	0	0	0	0	0	0	0	0	0	0	0	0	0	0	0	0	0	0
0	0	0	0	0	0	0	0	0	0	0	0	0	0	0	0	0	0	0
0	0	0	0	0	0	0	0	0	0	0	0	0	0	0	0	0	0	0
0	0	0	0	0	0	0	0	0	0	0	0	0	0	0	0	0	0	0
0	0	0	0	0	0	0	0	0	0	0	0	0	0	0	0	0	0	0
0	0	0	0	0	0	0	0	0	0	0	0	0	0	0	0	0	0	0
0	0	0	0	0	0	0	0	0	0	0	0	0	0	0	0	0	0	0
0	0	0	0	0	0	0	0	0	0	0	0	0	0	0	0	0	0	0
0	0	0	0	0	0	0	0	0	0	0	0	0	0	0	0	0	0	0
0	0	0	0	0	0	0	0	0	0	0	0	0	0	0	0	0	0	0
0	0	0	0	0	0	0	0	0	0	0	0	0	0	0	0	0	0	0
0	0	0	0	0	0	0	0	0	0	0	0	0	0	0	0	0	0	0
0	0	0	0	0	0	0	0	0	0	0	0	0	0	0	0	0	0	0
0	0	0	0	0	0	0	0	0	0	0	0	0	0	0	0	0	0	0
0	0	0	0	0	0	0	0	0	0	0	0	0	0	0	0	0	0	0
0	0	0	0	0	0	0	0	0	0	0	0	0	0	0	0	0	0	0
0	0	0	0	0	0	0	0	0	0	0	0	0	0	0	0	0	0	0
0	0	0	0	0	0	0	0	0	0	0	0	0	0	0	0	0	0	0
0	0	0	0	0	0	0	0	0	0	0	0	0	0	0	0	0	0	0
0	0	0	0	0	0	0	0	0	0	0	0	0	0	0	0	0	0	0
0	0	0	0	0	0	0	0	0	0	0	0	7	7	0	0	0	0	0
0	0	0	0	0	0	0	0	0	0	0	0	0	0	0	0	0	0	0
0	0	0	0	0	0	0	0	0	0	0	0	0	0	0	0	0	0	0
0	0	0	0	0	0	0	0	0	0	0	0	0	0	0	0	0	0	0

0	0	0	0	0	0	0	0	0	0	0	0	0	0	0	0	0	0	0
0	0	0	0	0	0	0	0	0	0	0	0	0	0	0	0	0	0	0
0	0	0	0	0	0	0	0	0	0	0	0	0	0	6	0	0	0	0
0	0	0	0	0	0	0	0	0	0	0	0	0	0	0	0	0	0	0
0	0	0	0	0	0	0	0	0	0	0	0	0	0	0	0	0	0	0
0	0	0	0	0	0	0	0	0	0	0	0	0	0	0	0	0	0	0
0	0	0	0	0	0	0	0	0	0	0	0	0	0	0	0	0	0	0
0	0	0	0	0	0	0	0	0	0	0	0	0	0	0	0	0	0	0
0	0	0	0	0	0	0	0	0	0	0	0	0	0	0	0	0	0	0
0	0	0	0	0	0	0	0	0	0	0	0	0	0	0	0	0	0	0
0	0	0	0	0	0	0	0	0	0	0	0	0	0	0	0	0	0	0
0	0	0	0	0	0	0	0	0	0	0	0	0	0	0	0	0	0	0
0	0	0	0	0	0	0	0	0	0	0	0	0	0	0	0	0	0	0
0	0	0	0	0	0	0	0	0	0	0	0	0	0	0	0	0	0	0
0	0	0	0	0	0	0	0	0	0	0	0	0	0	0	0	0	0	0
0	0	0	0	0	0	0	0	0	0	0	0	0	0	0	0	0	0	0
0	0	0	0	0	0	0	0	0	0	0	0	0	0	0	0	0	0	0
0	0	0	0	0	0	0	0	0	0	0	0	0	0	0	0	0	0	0
0	0	0	0	0	0	0	0	0	0	0	0	0	0	0	0	0	0	0
0	0	0	0	0	0	0	0	0	0	0	0	0	0	0	0	0	0	0
0	0	0	0	0	0	0	0	0	0	0	0	0	0	0	0	0	0	0
0	4	0	0	0	0	0	0	0	0	0	0	0	0	0	0	0	0	0
0	0	0	0	0	0	0	0	0	0	0	0	0	0	0	0	0	0	0
0	0	0	0	0	0	0	0	0	0	0	0	0	0	0	0	0	0	0
0	0	0	0	0	0	0	0	0	0	0	0	0	0	0	0	0	0	0
0	0	0	0	0	0	0	0	0	0	0	0	0	0	0	0	0	0	0
0	0	0	0	0	0	0	0	0	0	0	0	0	0	0	0	0	0	0
0	0	0	0	0	0	0	0	0	0	0	0	0	0	0	0	0	0	0
0	0	0	0	0	0	0	0	0	0	0	0	0	0	0	0	0	0	0
0	0	0	0	0	0	0	0	0	0	0	0	0	0	0	0	0	0	0
0	0	0	0	0	0	0	0	0	0	0	0	0	0	0	0	0	0	0
0	0	0	0	0	0	0	0	0	0	0	0	0	0	0	0	0	0	0
0	0	0	0	0	0	0	0	0	0	0	0	0	0	0	0	0	0	0
0	0	0	0	0	0	0	0	0	0	0	0	0	0	0	0	0	0	0
0	0	0	0	0	0	0	0	0	0	0	0	0	0	0	0	0	0	0
0	0	0	0	0	0	0	0	0	0	0	0	0	0	0	0	0	0	0
0	0	0	0	0	0	0	0	0	0	0	0	0	0	6	0	0	0	0

0	0	0	0	0	0	0	0	0	3	0	0	0	0	0	0	0	0	0
0	0	0	0	0	0	0	0	0	0	0	0	0	0	0	0	0	0	0
0	0	0	0	0	0	0	0	0	0	0	0	0	0	0	0	0	0	0
0	0	0	0	0	0	0	0	0	0	0	0	0	0	0	0	0	0	0
0	0	0	0	0	0	0	0	0	0	0	0	0	0	0	0	0	0	0
0	0	0	0	0	0	0	0	0	0	0	0	0	0	0	0	0	0	0
0	0	0	0	0	0	0	0	0	0	0	0	0	0	0	0	0	0	0
0	0	0	0	0	0	0	0	0	0	0	0	0	0	0	0	0	0	0
0	0	0	0	0	0	0	0	0	0	0	0	0	0	0	0	0	0	0
0	0	0	0	0	0	0	0	0	0	0	0	0	0	0	0	0	0	0
0	0	0	0	0	0	0	0	0	0	0	0	0	0	0	0	0	0	0
0	0	0	0	0	0	0	0	0	0	0	0	0	0	0	0	0	0	0
0	0	0	0	0	0	0	0	0	0	0	0	0	0	0	0	0	0	0
0	0	0	0	0	0	0	0	0	0	0	0	0	0	0	0	0	0	0
0	0	0	0	0	0	0	0	0	0	0	0	0	0	0	0	0	0	0
0	0	0	0	0	0	0	0	0	0	0	0	0	0	0	0	0	0	0
0	0	0	0	0	0	0	0	0	0	0	0	0	0	0	0	0	0	0
0	0	0	0	0	0	0	0	0	0	0	0	0	0	0	0	0	0	0
0	0	0	0	0	0	0	0	0	0	0	0	0	0	0	0	0	0	0
0	0	0	0	0	0	0	0	0	0	0	0	0	0	0	0	0	0	0
0	0	0	0	0	0	0	0	0	0	0	0	0	0	0	0	0	0	0
0	0	0	0	0	0	0	0	0	0	0	0	0	0	0	0	0	0	0
0	0	0	0	0	0	0	0	0	0	0	0	0	0	0	0	0	0	0
0	0	0	0	0	0	0	0	0	0	0	0	0	0	0	0	0	0	0
0	0	0	0	0	0	0	0	0	0	0	0	0	0	0	0	0	0	0
0	0	0	0	0	0	0	0	0	0	0	0	0	0	0	0	0	0	0
0	0	0	0	0	0	0	0	0	0	0	0	0	0	0	0	0	0	0
0	0	2	2	2	2	2	2	2	0	0	0	0	0	0	0	0	0	0
0	0	0	0	0	0	0	0	0	0	0	0	0	0	0	0	0	0	0
0	0	0	0	0	0	0	0	0	0	0	0	0	0	0	0	0	0	0
0	0	0	0	0	0	0	0	0	0	0	0	0	0	0	0	0	0	0
0	0	0	0	0	0	0	0	0	0	0	0	0	0	0	0	0	0	0
0	0	0	0	0	0	0	0	0	0	0	0	0	0	0	0	0	0	0
0	0	0	0	0	0	0	0	0	0	0	0	0	0	0	0	0	0	0
0	0	0	0	0	0	0	0	0	0	0	0	0	0	0	0	0	0	0
0	0	0	0	0	0	0	0	0	0	0	0	0	0	0	0	0	0	0
0	0	0	0	0	0	0	0	0	0	0	0	0	0	0	0	0	0	0

0	0	0	0	0	0	0	0	0	0	0	0	0	0	0	0	0	0	0
0	0	0	0	0	0	0	0	0	0	0	0	0	0	0	0	0	0	0
0	0	0	0	0	0	0	0	0	0	0	0	0	0	0	0	0	0	0
0	0	0	0	0	0	0	0	0	0	0	0	0	0	0	0	0	0	0
0	0	0	0	0	0	0	0	0	0	0	0	0	0	0	0	0	0	0
0	0	0	0	0	0	0	0	0	0	0	0	0	0	0	0	0	0	0
0	0	0	0	0	0	0	0	0	0	0	0	0	0	0	0	0	0	0
0	0	0	0	0	0	0	0	0	0	0	0	0	0	0	0	0	0	0
0	0	0	0	0	0	0	0	0	0	0	0	0	0	0	0	0	0	0
0	0	0	0	0	0	0	0	0	0	0	0	0	0	0	0	0	0	0
0	0	0	0	0	0	0	0	0	0	0	0	0	0	0	0	0	0	0
0	0	0	0	0	0	0	0	0	0	0	0	0	0	0	0	0	0	0
0	0	0	0	0	0	0	0	0	0									
Jan_DL066	1562	1749	0	389	93	399	809	875	99	29	62	242	10	223	322	21	183	150
163	0	309	102	182	260	77	95	55	75	105	0	78	73	0	0	103	0	67
0	32	121	48	154	0	80	6	107	0	61	0	25	62	24	0	0	68	0
0	0	0	112	46	42	78	72	21	129	0	147	4	76	18	19	155	6	25
30	0	15	18	53	33	31	18	95	60	34	0	86	84	31	0	22	9	0
43	65	50	633	0	29	0	0	0	13	0	69	76	13	26	0	0	0	61
0	76	158	80	12	56	0	0	0	0	15	25	21	54	72	52	39	66	405
0	0	0	43	0	0	48	58	144	13	0	0	16	0	25	51	0	0	0
0	0	0	0	0	0	0	0	0	0	23	0	0	0	0	76	32	49	0
0	0	0	68	7	0	0	41	0	0	10	3	0	0	0	0	0	0	20
0	59	0	0	0	0	0	20	0	10	30	28	0	63	0	0	0	0	0
22	0	0	0	0	0	19	0	0	74	277	0	0	0	0	16	0	19	0
0	0	0	0	0	0	0	0	0	0	0	0	27	19	0	34	0	0	0
27	0	0	55	0	0	0	0	0	0	0	0	0	0	0	20	0	0	0
0	26	0	28	34	26	0	0	0	4	0	0	0	0	0	15	5	0	0
0	0	0	52	0	0	0	0	0	0	0	0	0	0	8	20	0	33	0
0	5	0	0	0	0	0	0	0	15	0	92	0	0	0	6	0	20	19
12	0	0	0	19	0	26	0	0	0	0	0	0	0	0	0	0	59	0
0	0	0	0	5	8	0	0	0	0	0	0	0	0	0	0	0	0	0
0	13	0	0	0	0	0	0	0	0	0	0	7	0	28	0	0	0	0
0	0	0	13	0	15	0	3	8	0	0	0	0	0	0	16	0	0	0
0	0	0	0	35	0	0	0	126	0	0	0	11	0	0	33	0	0	0
0	0	0	0	0	0	26	0	0	0	0	0	0	0	0	0	18	0	0
0	0	0	16	0	0	0	0	0	0	0	0	0	0	0	0	0	0	0

0	0	6	0	0	0	0	0	0	0	0	22	0	0	0	0	0	0	0
0	0	44	0	0	152	0	0	0	5	0	0	31	0	11	0	0	0	11
0	0	0	25	0	0	0	0	0	0	0	0	0	0	0	0	10	0	0
0	8	0	0	0	3	13	8	1	38	0	0	0	0	0	36	0	0	0
11	0	0	0	0	0	0	0	0	0	0	0	0	0	0	24	0	0	141
0	0	0	0	0	0	0	0	0	0	0	17	10	0	0	32	0	0	0
0	0	0	0	0	0	0	0	0	36	0	0	0	0	10	0	0	0	0
0	0	0	0	0	0	0	0	0	0	0	0	21	0	12	0	0	0	0
0	23	0	29	0	0	0	0	0	0	0	0	0	0	0	0	0	0	0
0	0	0	0	0	0	0	0	0	0	0	0	0	0	0	0	0	0	0
0	0	0	0	0	0	0	0	0	0	0	13	0	38	0	0	0	0	13
0	0	0	0	0	0	0	0	0	0	0	0	0	0	0	0	0	0	0
0	0	0	0	0	0	0	0	0	0	0	0	0	0	0	0	0	0	0
0	4	0	10	0	44	0	40	0	0	0	0	0	0	0	0	0	0	0
0	0	0	0	0	0	0	0	0	0	0	0	0	0	0	0	0	0	0
0	0	0	0	0	0	0	0	0	0	0	0	0	0	0	0	5	0	0
0	0	0	0	0	0	0	9	0	18	0	0	0	0	10	0	0	0	0
23	0	0	0	0	0	0	0	0	0	14	0	0	0	0	0	0	0	5
0	0	0	0	0	0	0	0	0	0	0	0	0	0	0	0	0	0	0
0	0	0	0	0	0	0	0	4	0	0	0	0	5	0	0	0	0	0
0	0	0	0	0	0	0	19	0	0	0	0	0	0	0	0	0	0	0
0	0	0	0	0	0	0	0	5	0	0	0	0	0	0	0	0	0	0
0	0	9	0	0	0	0	0	0	0	0	0	0	0	7	0	0	10	0
0	0	0	0	0	0	0	0	0	15	0	0	0	0	0	0	0	0	0
0	0	0	0	0	0	0	0	12	0	0	0	0	0	0	0	0	0	0
0	0	0	0	0	0	0	0	0	0	0	0	0	0	0	0	0	0	0
0	0	0	0	0	0	0	0	0	0	0	0	0	0	0	0	0	0	0
0	0	0	0	7	0	0	0	0	0	0	0	0	0	0	0	0	0	0
0	0	0	0	0	0	0	0	0	0	0	0	0	0	0	0	0	0	0
0	0	0	0	0	0	0	0	0	0	0	0	0	0	0	0	0	0	0
0	0	0	0	0	0	0	0	0	0	0	0	0	0	0	0	0	0	0
0	0	0	0	0	0	0	0	0	0	0	0	0	0	0	0	2	0	0
0	0	0	7	0	0	0	0	0	0	0	0	0	0	0	0	0	0	0
0	0	0	0	10	0	0	0	0	0	0	11	0	0	0	0	0	0	0
0	0	0	0	0	0	0	0	0	0	0	0	0	0	0	0	0	0	0
0	0	0	0	0	0	0	0	0	0	0	0	0	0	0	0	0	0	0
0	0	0	0	0	0	0	0	0	0	0	0	0	0	0	0	0	0	0

0	0	18	0	0	0	0	0	0	0	0	0	0	0	0	0	0	0	0
0	0	0	0	0	0	0	0	0	0	0	0	0	0	0	0	0	0	0
0	0	0	0	0	0	0	0	0	0	0	0	0	0	0	0	0	0	0
0	0	0	0	0	0	0	0	0	0	0	0	0	0	0	0	0	0	0
0	0	0	0	0	0	0	0	0	0	0	0	0	0	0	0	0	0	0
0	0	0	0	0	0	0	0	0	0	0	0	0	0	0	0	0	0	0
0	0	0	0	0	0	0	0	0	0	0	0	0	0	15	0	0	0	0
0	0	0	0	0	0	0	0	0	0	0	0	0	0	0	0	0	0	0
0	0	0	0	0	0	0	0	0	0	0	0	0	0	0	0	0	0	0
0	0	0	0	0	0	0	0	0	0	0	0	0	0	0	0	0	0	0
0	0	0	0	0	0	0	0	0	0	0	13	0	0	0	0	0	0	0
0	0	0	0	0	0	0	0	0	0	0	0	0	0	0	0	0	0	0
0	0	0	0	0	0	0	0	0	0	0	0	0	0	0	0	0	0	0
0	0	0	0	0	0	0	0	0	0	0	0	0	0	0	0	0	0	0
0	0	0	0	0	0	0	0	0	0	11	0	0	0	0	0	0	0	0
0	0	0	0	0	0	0	0	0	0	0	0	0	0	7	0	0	0	0
0	0	0	0	0	0	0	0	0	0	0	0	0	0	0	0	0	0	0
0	0	0	0	0	0	0	0	0	0	0	0	0	0	0	0	0	0	0
0	0	0	0	0	0	0	0	0	0	0	0	0	0	0	0	0	0	0
0	0	0	0	0	0	0	0	0	0	0	0	0	0	0	0	0	0	0
0	0	0	0	0	0	0	0	0	0	0	0	0	0	0	0	0	0	0
0	0	0	0	0	0	0	0	0	0	0	0	0	0	0	0	0	0	0
0	0	0	0	0	0	0	0	0	0	0	0	0	0	0	0	0	0	0
0	0	0	0	0	0	0	0	0	0	0	0	0	0	0	0	0	0	0
0	0	0	0	0	0	0	0	0	0	0	0	0	0	0	0	0	0	0
0	0	0	0	0	0	0	0	0	0	0	0	0	0	0	0	0	0	0
0	0	0	0	0	0	0	0	0	0	0	0	0	0	0	0	0	0	0
0	0	0	0	0	0	0	0	0	0	0	0	0	0	0	0	0	0	0
0	0	0	0	0	0	0	0	0	0	0	0	0	0	0	0	0	0	0
0	0	0	0	0	0	0	0	0	0	0	0	0	0	0	0	0	0	0
0	0	0	0	0	0	0	0	0	0	0	0	0	0	0	0	0	0	0
0	0	0	0	0	0	0	0	0	0	0	0	0	0	0	0	0	0	0
0	0	0	0	0	0	0	0	0	0	0	0	0	0	0	0	0	0	0
0	0	0	0	0	0	0	0	0	0	0	0	0	0	0	0	0	0	0
0	0	0	0	0	0	0	0	0	0	0	0	0	0	0	0	0	0	0
0	0	0	0	0	0	0	0	0	0	0	0	0	0	0	0	0	0	0
0	0	0	0	0	0	0	0	0	0	0	0	0	0	0	0	0	0	0

0	0	0	0	0	0	0	0	0	0	0	0	0	0	0	0	0	0	0
0	0	0	0	0	0	0	0	0	0	0	0	0	0	0	0	0	0	0
0	0	0	0	0	0	0	0	0	0	0	0	0	0	0	0	0	0	0
0	0	0	0	0	0	0	0	0	0	0	0	0	0	0	0	0	0	0
0	0	0	0	0	0	0	0	0	0	0	0	0	0	0	0	0	0	0
0	0	0	0	0	0	0	0	0	0	0	0	0	0	0	0	0	0	0
0	0	0	0	0	0	0	0	0	0	0	0	0	0	0	0	0	0	0
0	0	0	0	0	0	0	0	0	0	0	0	0	0	0	0	0	0	0
0	0	0	0	0	0	0	0	0	0	0	0	0	5	5	0	0	0	0
0	0	0	0	0	0	0	0	0	0	0	0	0	0	0	0	0	0	0
0	0	0	0	0	0	0	0	0	0	0	0	0	0	0	0	0	0	0
0	0	0	0	0	0	0	0	0	0	0	0	0	0	0	0	0	0	0
0	0	0	0	0	0	0	0	0	0	0	0	0	0	0	0	0	0	0
0	0	0	0	0	0	0	0	0	0	0	0	0	0	0	0	0	0	0
0	0	0	0	0	0	0	0	0	0	0	0	0	0	0	0	0	0	0
0	0	0	0	0	0	0	0	0	0	0	0	0	0	0	0	0	0	0
0	0	0	0	0	0	0	0	0	0	0	0	0	0	0	0	0	0	0
0	0	0	0	0	0	0	0	0	0	0	0	0	0	0	0	0	0	0
0	0	0	0	0	0	0	0	0	0	0	0	0	0	0	0	0	0	0
0	0	0	4	0	0	0	0	0	0	0	0	0	0	0	0	0	0	0
0	0	0	0	0	0	0	0	0	0	0	0	0	0	0	0	0	0	0
0	0	0	0	0	0	0	0	0	0	0	0	0	0	0	0	0	0	0
0	0	0	0	0	0	0	0	0	0	0	0	0	0	0	0	0	0	0
0	0	0	0	0	0	0	0	0	0	0	0	0	0	0	0	0	0	0
0	0	0	0	0	0	0	0	0	0	0	0	0	0	0	0	0	0	0
0	0	0	0	0	0	0	0	0	0	0	0	0	0	0	0	0	0	0
0	0	0	0	0	0	0	0	0	0	0	0	0	0	0	0	0	0	0
0	0	0	0	0	0	0	0	0	0	0	0	0	0	0	0	0	0	0
0	0	0	0	0	0	0	0	0	0	0	0	0	0	0	0	0	0	0
0	0	0	0	0	0	0	0	0	0	0	0	0	0	0	0	0	0	0
0	0	0	0	0	0	0	0	0	0	0	0	0	0	0	0	0	0	0
0	0	0	0	0	0	0	0	0	0	0	0	0	0	0	0	0	0	0
0	0	0	0	0	0	0	0	0	0	0	0	0	0	0	0	0	0	0
0	0	0	0	0	0	0	0	0	0	0	0	0	0	0	0	0	0	0
0	0	0	0	0	0	0	0	0	0	0	0	0	0	0	0	0	0	0
0	0	0	0	0	0	3	0	0	0	0	0	0	0	0	0	0	0	0
0	0	0	0	0	0	0	0	0	0	0	0	0	0	0	0	0	0	0

0	0	0	0	0	0	0	0	0	0	0	0	0	0	0	0	0	0	0
0	0	0	0	0	0	0	0	0	0	0	0	0	0	0	0	0	0	0
0	0	0	0	0	0	0	0	0	0	0	0	0	0	0	0	0	0	0
0	0	0	0	0	0	0	0	0	0	0	0	0	0	0	0	0	0	0
0	0	0	0	0	0	0	0	0	0	0	0	0	0	0	0	0	0	0
0	0	0	0	0	0	0	0	0	0	0	0	0	0	0	0	0	0	0
0	0	0	0	0	0	0	0	0	0	0	0	0	0	0	0	0	0	0
0	0	0	0	0	0	0	0	0	0	0	0	0	0	0	0	0	0	0
0	0	0	0	0	0	0	0	0	0	0	0	0	0	0	0	0	0	0
0	0	0	0	0	0	0	0	0	0	0	0	0	0	0	0	0	0	0
0	0	0	0	0	0	0	0	0	0	0	0	0	0	0	0	0	0	0
0	0	0	0	0	0	0	0	0	0	0	0	0	0	0	0	0	0	0
0	0	0	0	0	0	0	0	0	0	0	0	0	0	0	0	0	0	0
0	0	0	0	0	0	0	0	0	0	0	0	0	0	0	0	0	0	0
0	0	0	0	0	0	0	0	0	0	0	0	0	0	0	0	0	0	0
0	0	0	0	0	0	0	0	0	0	0	0	0	0	0	0	0	0	0
0	0	0	0	0	0	0	0	0	0	0	0	0	0	0	0	0	0	0
0	0	0	0	0	0	0	0	0	0	0	0	0	0	0	0	0	0	0
0	0	0	0	0	0	0	0	0	0	0	0	0	0	0	0	0	0	0
0	0	0	0	0	0	0	0	0	0	0	0	0	0	0	0	0	0	0
0	0	0	0	0	0	0	0	0	0	0	0	0	0	0	0	0	0	0
0	0	0	0	0	0	0	0	0	0	0	0	0	0	0	0	0	0	0
0	0	0	0	0	0	0	0	0	0	0	0	0	0	0	0	0	0	0
0	0	0	0	0	0	0	0	0	0	0	0	0	0	0	0	0	0	0
0	0	0	0	0	0	0	0	0	0	0	0	0	0	0	0	0	0	0
0	0	0	0	0	0	0	0	0	0	0	0	0	0	0	0	0	0	0
0	0	2	0	0	0	0	0	0	0	0	0	0	0	0	0	0	0	0
0	0	0	0	0	0	0	0	0	0	0	0	0	0	0	0	0	0	0
0	0	0	0	0	0	0	0	0	0	0	0	0	0	0	0	0	0	0
0	0	0	0	0	0	0	0	0	0	0	0	0	0	0	0	0	0	0
0	0	0	0	0	0	0	0	0	0	0	0	0	0	0	0	0	0	0
0	0	0	0	0	0	0	0	0	0	0	0	0	0	0	0	0	0	0
0	0	0	0	0	0	0	0	0	0	0	0	0	0	0	0	0	0	0
0	0	0	0	0	0	0	0	0	0	0	0	0	0	0	0	0	0	0
0	0	0	0	0	0	0	0	0	0	0	0	0	0	0	0	0	0	0
0	0	0	0	0	0	0	0	0	0	0	0	0	0	0	0	0	0	0
0	0	0	0	0	0	0	0	0	0	0	0	0	0	0	0	0	0	0

0	0	0	0	0	0	0	0	0	0	0	0	0	0	0	0	0	0	0
0	0	0	0	0	0	0	0	0	0	0	0	0	0	0	0	0	0	0
0	0	0	0	0	0	0	0	0	0	0	0	0	0	0	0	0	0	0
0	0	0	0	0	0	0	0	0	0	0	0	0	0	0	0	0	0	0
0	0	1	0	0	0	0	0	0	0									
Jan_DL068	285	1204	0	137	577	160	318	385	152	31	74	11	34	109	181	0	105	151
91	0	123	480	83	123	0	37	23	0	107	0	24	20	19	0	0	0	0
31	212	55	0	33	0	47	0	54	0	32	0	212	0	21	10	0	48	0
0	11	0	68	0	0	0	11	0	16	78	56	0	0	0	26	12	0	19
0	0	35	6	0	0	3	0	52	0	0	51	21	0	24	0	8	21	0
11	0	0	0	0	30	0	0	69	0	0	6	28	39	0	18	0	0	58
0	0	101	5	0	0	0	0	0	0	14	0	17	0	0	103	0	32	0
0	0	0	24	0	0	0	0	0	3	0	0	8	0	0	21	0	0	0
0	0	20	0	11	16	0	18	22	40	26	0	0	0	0	0	0	0	12
0	0	0	0	0	0	0	0	0	0	10	0	0	39	0	0	0	0	0
0	18	35	10	0	23	0	41	0	0	0	0	35	0	0	0	0	0	0
21	0	0	0	0	0	21	0	0	0	0	0	0	0	0	0	0	4	0
0	0	0	0	0	0	0	0	0	5	0	0	6	0	0	0	0	0	8
14	0	0	15	0	0	0	0	0	0	0	0	0	19	13	0	24	0	0
0	0	0	0	0	14	0	0	11	0	12	0	0	7	9	2	0	0	0
0	0	0	0	0	0	0	9	0	0	0	0	0	0	0	0	0	0	0
0	4	0	0	0	0	0	0	0	0	0	0	6	0	30	0	0	0	0
0	0	0	0	0	0	0	0	0	0	0	0	0	20	0	0	0	0	0
0	0	0	0	0	0	0	0	6	11	0	0	0	0	0	0	0	0	5
0	0	0	0	0	30	0	0	0	0	0	0	0	0	21	0	0	0	0
0	0	0	0	0	7	0	8	10	0	0	0	0	0	0	0	37	0	0
0	0	0	0	15	0	0	0	0	0	0	0	0	0	0	0	0	0	0
0	0	13	0	0	0	0	43	0	0	11	0	0	0	0	0	0	0	0
0	0	0	0	0	0	0	15	0	0	0	0	0	0	0	0	0	0	0
0	6	0	0	0	0	0	0	0	0	0	0	0	0	0	0	0	0	0
0	0	0	0	0	0	0	0	0	0	0	0	0	0	10	0	0	0	3
0	0	0	0	0	0	0	0	0	0	0	0	0	0	0	0	0	0	0
0	0	0	0	0	0	0	0	0	0	0	0	0	0	0	0	0	0	0
0	0	0	0	0	0	0	0	0	0	0	0	0	0	0	0	0	0	0
0	0	0	0	0	0	0	0	0	19	0	0	0	0	0	0	0	0	0
0	0	13	0	0	0	0	0	0	0	0	0	0	0	0	0	0	0	0
0	0	0	0	0	0	0	0	5	0	0	0	0	0	0	0	0	0	0

0	0	0	0	0	0	0	0	0	6	0	0	0	0	0	0	0	0	0
0	0	0	0	0	0	0	0	0	0	0	0	0	0	0	0	0	0	0
0	3	0	0	0	0	0	0	0	0	0	0	0	0	0	0	0	0	0
0	0	0	0	0	0	0	0	0	0	0	0	0	0	0	0	0	0	0
0	0	0	0	0	0	0	0	0	0	0	0	0	26	0	0	0	0	0
0	0	0	0	0	0	0	0	0	0	0	0	0	0	0	0	0	0	0
0	0	0	0	0	0	0	0	0	0	0	0	0	0	0	0	0	0	0
0	0	0	0	0	0	0	0	0	0	0	0	0	0	0	0	0	0	0
0	0	0	0	0	0	0	0	0	0	0	0	0	0	0	0	0	0	0
0	0	0	0	0	0	0	0	0	0	0	0	0	0	0	0	0	0	0
0	0	0	0	0	0	0	0	0	0	0	0	0	0	0	0	0	0	0
0	5	0	0	0	0	0	0	0	0	0	0	0	0	0	0	0	0	0
0	0	0	0	0	0	0	0	0	0	0	0	0	0	0	0	0	0	0
0	0	0	0	0	0	0	0	0	0	0	0	0	0	0	0	0	0	0
0	0	0	0	0	0	0	0	0	0	0	0	0	0	0	0	0	0	0
0	0	0	0	0	0	0	0	0	0	0	0	0	0	0	0	0	0	0
0	0	0	0	0	0	0	0	0	0	0	0	0	0	0	0	0	0	0
0	0	0	0	0	0	0	0	0	0	0	0	0	0	0	0	0	0	0
0	0	5	0	0	0	0	0	0	0	18	0	0	0	0	0	0	0	0
0	0	0	0	0	0	0	0	0	0	0	0	0	0	0	0	0	0	0
0	0	0	0	0	0	0	0	0	0	0	0	0	0	0	0	0	0	0
0	0	0	0	0	0	0	0	0	0	0	0	0	0	0	0	0	0	0
0	0	0	0	0	0	0	0	0	0	0	0	0	0	0	0	0	0	0
0	0	0	0	0	0	0	0	0	0	0	0	0	0	0	0	0	0	0
0	0	0	0	0	0	0	0	0	0	0	0	0	0	0	0	0	4	0
0	0	0	0	0	0	0	0	0	0	0	0	0	0	0	0	0	0	0
0	0	0	0	0	0	0	0	0	0	0	0	0	0	0	0	0	0	0
0	0	0	0	0	0	0	0	0	0	0	0	0	0	0	0	0	0	0
0	0	0	0	10	0	0	0	0	0	0	0	0	0	0	0	0	0	0
0	0	0	0	0	0	0	0	0	0	0	0	0	0	0	0	0	0	0
0	0	0	0	0	0	0	0	0	0	0	0	0	0	0	0	0	0	0
0	0	7	0	0	0	0	0	0	0	0	0	0	0	0	0	0	0	0
0	0	0	0	0	0	0	0	0	0	0	0	0	0	0	0	0	0	0
0	0	0	0	0	0	0	0	0	0	0	0	0	0	0	0	0	0	0
0	0	0	0	0	0	0	0	0	0	0	0	0	0	0	0	0	0	0
0	0	0	0	0	0	0	0	0	0	0	0	0	0	0	0	0	0	0
0	0	0	0	0	0	0	0	0	0	0	0	0	0	0	0	0	0	0

0	0	0	0	0	0	0	0	0	0	0	0	0	0	0	0	0	0	0
0	0	0	0	0	0	0	0	0	0	0	0	0	0	0	0	0	0	0
0	0	0	0	0	0	0	0	0	0	0	0	0	0	0	0	0	6	0
0	0	0	0	0	0	0	0	0	0	0	0	0	0	0	0	0	0	0
0	0	4	0	0	0	0	0	0	0	0	0	0	0	0	0	0	0	0
0	0	0	0	0	0	0	0	0	0	0	0	0	0	0	0	0	0	0
0	0	0	0	0	0	0	0	0	0	0	0	0	0	0	0	0	0	0
0	0	0	0	0	5	0	0	0	0	0	0	0	0	0	0	0	0	0
0	0	0	0	0	0	0	0	0	0	0	0	0	0	0	0	0	0	0
0	0	0	0	0	0	0	0	0	0	0	0	0	0	0	0	0	0	0
0	0	0	0	0	0	0	0	0	0	0	0	0	0	0	0	0	0	0
0	0	0	0	0	0	0	0	0	0	0	0	0	0	0	0	0	0	0
0	0	0	0	0	0	0	0	0	0	0	0	0	0	0	0	0	0	0
0	0	0	0	0	0	0	0	0	0	0	0	0	0	0	0	0	0	0
0	0	0	0	0	4	4	4	0	0	0	0	0	0	0	0	0	0	0
0	0	0	0	0	0	0	0	0	0	0	0	0	0	0	0	0	0	0
0	0	0	0	0	0	0	0	0	0	0	0	0	0	0	0	0	0	0
0	0	0	0	0	0	0	0	0	0	0	0	0	0	0	0	0	0	0
0	0	0	0	0	0	0	0	0	0	0	0	0	0	0	0	0	0	0
0	0	0	0	0	0	0	0	0	0	0	0	0	0	0	0	0	0	0
0	0	0	0	0	0	0	0	0	0	0	0	0	0	0	0	0	0	0
0	0	0	0	0	0	0	0	0	0	0	0	0	0	0	0	0	0	0
0	0	0	0	0	0	0	0	0	0	0	0	0	0	0	0	0	0	0
0	0	0	0	0	0	0	0	0	0	0	0	0	0	0	0	0	0	0
0	0	0	0	0	0	0	0	0	0	0	0	0	0	0	0	0	0	0
0	0	0	0	0	0	0	0	0	0	0	0	0	0	0	0	0	0	0
0	0	0	0	3	3	3	3	0	0	0	0	0	0	0	0	0	0	0
0	0	0	0	0	0	0	0	0	0	0	0	0	0	0	0	0	0	0
0	0	0	0	0	0	0	0	0	0	0	0	0	0	0	0	0	0	0
0	0	0	0	0	0	0	0	0	0	0	0	0	0	0	0	0	0	0
0	0	0	0	0	0	0	0	0	0	0	0	0	0	0	0	0	0	0
0	0	0	0	0	0	0	0	0	0	0	0	0	0	0	0	0	0	0
0	0	0	0	0	0	0	0	0	0	0	0	0	0	0	0	0	0	0
0	0	0	0	0	0	0	0	0	0	0	0	0	0	0	0	0	0	0
0	0	0	0	0	0	0	0	0	0	0	0	0	0	0	0	0	0	0
0	0	0	0	0	0	0	0	0	0	0	0	0	0	0	0	0	0	0
0	0	0	0	0	0	0	0	0	0	0	0	0	0	0	0	0	0	0

0	0	0	0	0	0	0	0	0	0	0	0	0	0	0	0	0	0	0
0	0	0	0	0	0	0	0	0	0	0	0	0	0	0	0	0	0	0
0	0	0	0	0	0	0	0	0	0	0	0	0	0	0	0	0	0	0
0	0	0	0	0	0	0	0	0	0	0	0	0	0	0	0	0	0	0
0	0	0	0	0	0	0	0	0	0	0	0	0	0	0	0	0	0	0
0	0	0	0	0	0	0	0	0	0	0	0	0	0	0	0	0	0	0
0	0	0	0	0	0	0	0	0	0	0	0	0	0	0	0	0	0	0
0	0	0	0	0	0	0	0	0	0	0	0	0	2	2	2	2	2	2
2	2	0	0	0	0	0	0	0	0	0	0	0	0	0	0	0	0	0
0	0	0	0	0	0	0	0	0	0	0	0	0	0	0	0	0	0	0
0	0	0	0	0	0	0	0	0	0	0	0	0	0	0	0	0	0	0
0	0	0	0	0	0	0	0	0	0	0	0	0	0	0	0	0	0	0
0	0	0	0	0	0	0	0	0	0	0	0	0	0	0	0	0	0	0
0	0	0	0	0	0	0	0	0	0	0	0	0	0	0	0	0	0	0
0	0	0	0	0	0	0	0	0	0	0	0	0	0	0	0	0	0	0
0	0	0	0	0	0	0	0	0	0	0	0	0	0	0	0	0	0	0
0	0	0	0	0	0	0	0	0	0	0	0	0	0	0	0	0	0	0
0	0	0	0	0	0	0	0	0	0	0	0	0	0	0	0	0	0	0
0	0	0	0	0	0	0	0	0	0	0	0	0	0	0	0	0	0	0
0	0	0	0	0	0	0	0	0	0	0	0	0	0	0	0	0	0	0
0	0	0	0	0	0	0	0	0	0	0	0	0	0	0	0	0	0	0
0	0	0	0	0	0	0	0	0	0	0	0	0	0	0	0	0	0	0
0	0	0	0	0	0	0	0	0	0	0	0	0	0	0	0	0	0	0
0	0	0	0	0	0	0	0	0	0	0	0	0	0	0	0	0	0	0
0	0	0	0	0	0	0	0	0	0	0	0	0	0	0	0	0	0	0
0	0	0	0	0	0	0	0	0	0	0	0	0	0	0	0	0	0	0
0	0	0	0	0	0	0	0	0	0	0	0	0	0	0	0	0	0	0
0	0	0	0	0	0	0	0	0	0	0	0	0	0	0	0	0	0	0
0	0	0	0	0	0	0	0	0	0	0	0	0	0	0	0	0	0	0
0	0	0	0	0	0	0	0	0	0	0	0	0	0	0	0	0	0	0
0	0	0	0	0	0	0	0	0	0	0	0	0	0	0	0	0	0	0
0	0	0	0	0	0	0	0	0	0	0	0	0	0	0	0	0	0	0
0	0	0	0	0	0	0	0	0	0	0	0	0	0	0	0	0	0	0
0	0	0	0	0	0	0	0	0	0	0	0	0	0	0	0	0	0	0
0	0	0	0	0	0	0	0	0	0	0	0	0	0	0	0	0	0	0
0	0	0	0	0	0	0	0	0	0	0	0	0	0	0	0	0	0	0
0	0	0	0	0	0	0	0	0	0	0	0	0	0	0	0	0	0	0

0	0	0	0	0	0	0	0	0	0	0	0	0	0	0	0	0	0	0
0	0	0	0	0	0	0	0	0	0	0	0	0	0	0	0	0	0	0
0	0	0	0	0	0	0	0	0	0	0	0	0	0	0	0	0	0	0
0	0	0	0	0	0	0	0	0	0	0	0	0	0	0	0	0	0	0
0	0	0	0	0	0	0	0	0	0	0	0	0	0	0	0	0	0	0
0	0	0	0	0	0	0	0	0	0	0	0	0	0	0	0	0	0	0
0	0	0	0	0	0	0	0	0	0	0	0	0	0	0	0	0	0	0
0	0	0	0	0	0	0	0	0	0	0	0	0	0	0	0	0	0	0
0	0	0	0	0	0	0	0	0	0	0	0	0	0	0	0	0	0	0
0	0	0	0	0	0	0	0	0	0	0	0	0	0	0	0	0	0	0
0	0	0	0	0	0	0	0	0	0	0	0	0	0	0	0	0	0	0
0	0	0	0	0	0	0	0	0	0	0	0	0	0	0	0	0	0	0
0	0	0	0	0	0	0	0	0	0	0	0	0	0	0	0	0	0	0
0	0	0	0	0	0	0	0	0	0	0	0	0	0	0	0	0	0	0
0	0	0	0	0	0	0	0	0	0	0	0	0	0	0	0	0	0	0
0	0	0	0	0	0	0	0	0	0	0	0	0	0	0	0	0	0	0
0	0	0	0	0	0	0	0	0	0	0	0	0	0	0	0	0	0	0
0	0	0	0	0	0	0	0	0	0	0	0	0	0	0	0	0	0	0
0	0	0	0	0	0	0	0	0	0	0	0	0	0	0	0	0	0	0
0	0	0	0	0	0	0	0	0	0	0	0	0	0	0	0	0	0	0
0	0	0	0	0	0	0	0	0	0	0	0	0	0	0	0	0	0	0
0	0	0	0	0	0	0	0	0	0	0	0	0	0	0	0	0	0	0
0	0	0	0	0	0	0	0	0	0	0	0	0	0	0	0	0	0	0
0	0	0	0	0	0	0	0	0	0	0	0	0	0	0	0	0	0	0
0	0	0	0	0	0	0	0	0	0	0	0	0	0	0	0	0	0	0
0	0	0	0	0	0	0	0	0	0	0	0	0	0	0	0	0	0	0
0	0	0	0	0	0	0	0	0	0	0	0	0	0	0	0	0	0	0
0	0	0	0	0	0	0	0	0	0	0	0	0	0	0	0	0	0	0
0	0	0	0	0	0	0	0	0	0	0	0	0	0	0	0	0	0	0
0	0	0	0	0	0	0	0	0	0	0	0	0	0	0	0	0	0	0
0	0	0	0	0	0	0	0	0	0	0	0	0	0	0	0	0	0	0
0	0	0	0	0	0	0	0	0	0	0	0	0	0	0	0	0	0	0
0	0	0	0	0	0	0	0	0	0	0	0	0	0	0	0	0	0	0
0	0	0	0	0	0	0	0	0	0									
Jan_DL069	655	751	360	282	11	187	39	39	114	176	599	61	208	35	139	89	34	123
49	11	145	0	71	96	110	113	120	94	94	93	137	54	105	286	74	9	88
0	17	122	11	28	167	45	25	85	44	0	113	0	0	14	102	148	0	57

47	11	94	14	0	139	35	17	105	11	22	0	55	0	0	63	9	10	0
64	77	0	38	14	59	9	31	0	9	0	0	13	0	15	50	0	0	0
0	0	0	0	0	0	38	0	0	0	0	0	10	0	23	0	7	25	0
0	0	0	5	0	0	0	0	0	0	0	0	10	0	0	0	0	0	0
0	0	25	0	19	0	0	0	11	0	0	0	2	0	0	0	0	0	0
0	0	21	12	0	11	21	0	0	0	0	0	0	0	0	128	0	0	15
0	0	0	19	0	0	0	0	0	0	0	0	0	0	0	0	3	0	0
0	0	0	0	0	0	21	0	0	0	0	0	0	0	15	0	0	0	0
0	0	25	0	0	0	0	0	0	0	0	0	0	0	0	0	0	0	0
0	0	0	17	0	0	0	0	0	0	0	0	3	3	0	0	0	0	0
0	0	0	0	0	0	0	0	0	0	0	0	0	0	0	0	0	12	0
0	0	0	0	0	0	0	0	0	0	6	0	0	0	0	0	0	0	0
0	0	0	7	0	0	0	0	0	0	0	0	0	0	0	0	20	0	0
0	0	0	0	0	0	0	0	0	0	0	0	0	0	17	0	0	0	5
0	0	0	0	0	0	0	0	0	0	0	0	0	0	0	0	0	0	0
0	0	4	0	0	0	0	0	0	0	0	0	0	0	0	0	5	0	0
0	0	0	0	0	0	0	0	0	7	0	0	20	0	0	0	0	0	0
0	0	0	0	0	0	0	0	0	0	0	0	0	0	4	0	0	0	0
0	0	0	0	0	0	5	0	0	31	0	0	0	3	0	0	0	0	0
0	0	0	0	0	0	0	0	2	0	0	0	0	0	0	0	0	0	0
0	0	0	0	0	0	0	0	0	0	0	0	0	0	0	0	0	0	0
0	0	0	0	0	0	0	0	0	0	0	0	0	9	0	0	0	0	0
0	0	0	0	0	0	0	0	0	0	0	0	0	0	4	0	0	0	0
0	0	0	0	33	0	0	0	0	0	0	0	0	0	0	0	6	0	0
0	0	0	0	0	0	3	0	0	0	0	0	0	0	0	0	0	0	0
0	0	0	0	0	2	0	0	0	0	0	0	0	0	0	0	0	0	0
0	0	0	0	0	0	0	0	0	0	0	0	0	0	0	0	0	0	0
0	0	0	0	0	6	0	0	0	0	0	0	0	0	0	0	0	0	0
0	0	0	0	0	0	5	0	0	0	0	0	0	0	0	0	0	0	0
0	0	0	0	0	0	0	0	0	0	0	0	0	0	0	0	14	0	0
0	0	0	0	0	0	0	0	0	0	0	0	0	0	0	0	0	0	0
0	0	0	0	0	0	0	0	0	0	0	0	0	0	0	0	0	0	0
0	0	0	0	0	0	0	0	0	0	0	0	0	0	0	0	0	0	0
0	0	0	0	8	0	0	0	0	0	0	0	0	0	0	0	0	0	0
0	0	0	0	0	0	0	0	0	0	0	0	0	0	0	0	0	0	0
0	0	0	0	0	0	0	0	0	0	0	0	0	0	0	0	0	0	0
0	0	0	0	10	0	0	0	0	0	0	0	0	0	0	0	0	0	0

0	0	0	0	0	0	0	0	0	0	0	0	0	0	0	0	0	0	0
0	0	0	0	0	0	0	0	0	0	0	0	0	0	0	0	0	0	0
0	0	0	0	0	0	0	0	0	0	0	0	0	0	0	0	0	0	0
0	0	0	0	0	0	0	0	0	0	0	0	0	0	0	0	0	0	0
0	0	0	0	0	0	0	0	0	0	0	0	0	0	0	0	0	0	0
0	0	0	0	0	0	0	0	0	0	0	0	0	0	0	0	0	0	0
0	0	0	0	0	0	0	0	0	0	0	0	0	0	0	0	0	0	0
0	0	0	0	0	0	0	0	0	0	0	0	0	0	0	0	0	0	0
0	0	0	0	0	0	0	0	0	0	0	0	0	0	0	0	0	0	0
0	0	0	0	0	0	0	0	0	0	0	0	0	7	0	0	0	0	0
0	0	0	0	0	0	0	0	0	0	0	0	0	0	0	0	0	0	0
0	0	0	0	0	0	0	0	0	0	0	0	0	0	0	0	0	0	0
0	0	0	0	0	0	0	0	0	0	0	0	0	0	0	0	0	0	0
0	0	0	0	0	0	0	0	0	0	0	0	0	0	0	0	0	0	0
0	0	0	0	0	0	0	0	0	0	0	0	0	0	0	0	0	0	0
0	0	0	0	0	0	0	0	0	0	0	0	0	0	0	0	0	0	0
0	0	0	0	0	0	0	0	0	0	0	0	0	0	0	0	0	0	0
0	0	0	0	0	0	0	0	0	0	0	0	0	0	0	0	0	0	0
0	0	0	0	0	0	0	0	0	0	0	0	0	0	0	0	0	0	0
0	0	0	0	0	0	0	0	0	0	0	0	0	0	0	0	0	0	0
0	0	0	0	0	0	0	0	0	0	0	0	0	0	0	0	0	0	0
0	0	0	0	0	0	0	0	0	0	0	0	0	0	0	0	0	0	0
0	0	0	0	0	0	0	0	0	0	0	0	0	0	0	0	0	0	0
0	0	0	0	0	0	0	0	0	0	0	0	0	0	0	0	0	0	0
0	0	0	0	0	0	0	0	0	0	0	0	0	0	0	0	0	0	0
0	0	0	0	0	0	0	0	0	0	0	0	0	0	0	0	0	0	0
0	0	0	0	0	0	0	0	0	0	0	0	0	0	0	0	0	0	0
0	0	0	0	0	0	0	0	0	0	0	0	0	0	0	0	0	0	0
0	0	0	0	0	0	0	0	0	0	0	0	0	0	0	0	0	0	0
7	0	0	0	0	0	0	0	0	0	0	0	0	0	0	0	0	0	0
0	0	0	0	0	0	0	0	0	0	0	0	0	0	0	0	0	0	0
0	0	0	0	0	0	0	0	0	0	0	0	0	0	0	0	0	0	0
0	0	0	0	0	0	0	0	0	0	0	0	0	0	0	0	0	0	0
0	0	0	0	0	0	0	0	0	0	0	0	0	0	0	0	0	0	0
0	0	0	0	0	0	0	0	0	0	0	0	0	0	0	0	0	0	0
0	0	0	0	0	0	0	0	0	0	0	0	0	0	0	0	0	0	0
0	0	0	0	0	0	0	0	0	0	0	0	0	0	0	0	0	0	0

0	0	0	0	0	0	0	0	0	0	0	0	0	0	0	0	0	0	0
0	0	0	0	0	0	0	0	0	0	0	0	0	0	0	0	0	0	0
0	0	0	0	0	0	0	0	0	0	0	0	0	0	0	0	0	0	0
0	0	0	0	0	0	0	0	0	0	0	0	0	0	0	0	0	0	0
0	0	0	0	0	0	0	0	0	0	0	0	0	0	0	0	0	0	0
0	0	0	0	0	0	0	0	0	0	0	0	0	0	0	0	0	0	0
0	0	0	0	0	0	0	0	0	0	0	0	0	0	0	0	0	0	0
0	0	0	0	0	0	0	0	0	0	0	0	0	0	0	0	0	0	0
0	0	0	0	0	0	0	0	0	0	0	4	4	4	0	0	0	0	0
0	0	0	0	0	0	0	0	0	0	0	0	0	0	0	0	0	0	0
0	0	0	0	0	0	0	0	0	0	0	0	0	0	0	0	0	0	0
0	0	0	0	0	0	0	0	0	0	0	0	0	0	0	0	0	0	0
0	0	0	0	0	0	0	0	0	0	0	0	0	0	0	0	0	0	0
0	0	0	0	0	0	0	0	0	0	0	0	0	0	0	0	0	0	0
0	0	0	0	0	0	0	0	0	0	0	0	0	0	0	0	0	0	0
0	0	0	0	0	0	0	0	0	0	0	0	0	0	0	0	0	0	0
0	0	0	0	0	0	0	0	0	0	0	0	0	0	0	0	0	0	0
0	0	0	0	0	0	0	0	0	0	0	0	0	0	0	0	0	0	0
0	0	0	0	0	0	0	0	0	0	0	0	0	0	0	0	0	0	0
0	0	0	0	0	0	0	0	0	0	0	0	0	0	0	0	0	0	0
0	0	0	0	0	0	0	0	0	0	0	0	3	3	0	0	0	0	0
0	0	0	0	0	0	0	0	0	0	0	0	0	0	0	0	0	0	0
0	0	0	0	0	0	0	0	0	0	0	0	0	0	0	0	0	0	0
0	0	0	0	0	0	0	0	0	0	0	0	0	0	0	0	0	0	0
0	0	0	0	0	0	0	0	0	0	0	0	0	0	0	0	0	0	0
0	0	0	0	0	0	0	0	0	0	0	0	0	0	0	0	0	0	0
0	0	0	0	0	0	0	0	0	0	0	0	0	0	0	0	0	0	0
0	0	0	0	0	0	0	0	0	0	0	0	0	0	0	0	0	0	0
0	0	0	0	0	0	0	0	0	0	0	0	0	0	0	0	0	0	0
0	0	0	0	0	0	0	0	0	0	0	0	0	0	0	0	0	0	0
0	0	0	0	0	0	0	0	0	0	0	0	0	0	0	0	0	0	0
0	0	0	0	0	0	0	0	0	0	0	0	0	0	0	0	0	0	0
0	0	0	0	0	0	0	0	0	0	0	0	0	0	0	0	0	0	0
0	0	0	0	0	0	0	0	0	0	0	0	0	0	0	0	0	0	0
0	0	0	0	0	0	0	0	0	0	0	0	0	0	0	0	0	0	0
0	0	0	0	0	0	0	0	0	0	0	0	0	0	0	0	0	0	0
0	0	0	0	0	0	0	0	0	0	0	0	0	0	0	0	0	0	0

0	0	0	0	0	0	0	0	0	0	0	0	0	0	0	0	0	0	0
0	0	0	0	0	0	0	0	0	0	0	0	0	0	0	0	0	0	0
0	0	0	0	0	0	0	0	0	0	0	0	0	0	2	2	2	0	0
0	0	0	0	0	0	0	0	0	0	0	0	0	0	0	0	0	0	0
0	0	0	0	0	0	0	0	0	0	0	0	0	0	0	0	0	0	0
0	0	0	0	0	0	0	0	0	0	0	0	0	0	0	0	0	0	0
0	0	0	0	0	0	0	0	0	0	0	0	0	0	0	0	0	0	0
0	0	0	0	0	0	0	0	0	0	0	0	0	0	0	0	0	0	0
0	0	0	0	0	0	0	0	0	0	0	0	0	0	0	0	0	0	0
0	0	0	0	0	0	0	0	0	0	0	0	0	0	0	0	0	0	0
0	0	0	0	0	0	0	0	0	0	0	0	0	0	0	0	0	0	0
0	0	0	0	0	0	0	0	0	0	0	0	0	0	0	0	0	0	0
0	0	0	0	0	0	0	0	0	0	0	0	0	0	0	0	0	0	0
0	0	0	0	0	0	0	0	0	0	0	0	0	0	0	0	0	0	0
0	0	0	0	0	0	0	0	0	0	0	0	0	0	0	0	0	0	0
0	0	0	0	0	0	0	0	0	0	0	0	0	0	0	0	0	0	0
0	0	0	0	0	0	0	0	0	0	0	0	0	0	0	0	0	0	0
0	0	0	0	0	0	0	0	0	0	0	0	0	0	0	0	0	0	0
0	0	0	0	0	0	0	0	0	0	0	0	0	0	0	0	0	0	0
0	0	0	0	0	0	0	0	0	0	0	0	0	0	0	0	0	0	0
0	0	0	0	0	0	0	0	0	0	0	0	0	0	0	0	0	0	0
0	0	0	0	0	0	0	0	0	0	0	0	0	0	0	0	0	0	0
0	0	0	0	0	0	0	0	0	0	0	0	0	0	0	0	0	0	0
0	0	0	0	0	0	0	0	0	0	0	0	0	0	0	0	0	0	0
0	0	0	0	0	0	0	0	0	0	0	0	0	0	0	0	0	0	0
0	0	0	0	0	0	0	0	0	0	0	0	0	0	0	0	0	0	0
0	0	0	0	0	0	0	0	0	0	0	0	0	0	0	0	0	0	0
0	0	0	0	0	0	0	0	0	0	0	0	0	0	0	0	0	0	0
0	0	0	0	0	0	0	0	0	0	0	0	0	0	0	0	0	0	0
0	0	0	0	0	0	0	0	0	0	0	0	0	0	0	0	0	0	0
0	0	0	0	0	0	0	0	0	0	0	0	0	0	0	0	0	0	0
0	0	0	0	0	0	0	0	0	0	0	0	0	0	0	0	0	0	0
0	0	0	0	0	0	0	0	0	0	0	0	0	0	0	0	0	0	0
0	0	0	0	0	0	0	0	0	0	0	0	0	0	0	0	0	0	0
0	0	0	0	0	0	0	0	0	0	0	0	0	0	0	0	0	0	0
0	0	0	0	0	0	0	0	0	0	0	0	0	0	0	0	0	0	0
0	0	0	0	0	0	0	0	0	0	0	0	0	0	0	0	0	0	0
0	0	0	0	0	0	0	0	0	0	0	0	0	0	0	0	0	0	0

0	0	0	0	0	0	0	0	0	0	0	0	0	0	0	0	0	0	0
0	0	0	0	0	0	0	0	0	0	0	0	0	0	0	0	0	0	0
0	0	0	0	0	0	0	0	0	0	0	0	0	0	0	0	0	0	0
0	0	0	0	0	0	0	0	0	0	0	0	0	0	0	0	0	0	0
0	0	0	0	0	0	0	0	0	0	0	0	0	0	0	0	0	0	0
0	0	0	0	0	0	0	0	0	0	0	0	0	0	0	0	0	0	0
0	0	0	0	0	0	0	0	0	0	0	0	0	0	0	0	0	0	0
0	0	0	0	0	0	0	0	0	0	0	0	0	0	0	0	0	0	0
0	0	0	0	0	0	0	0	0	0	0	0	0	0	0	0	0	0	0
0	0	0	0	0	0	0	0	0	0	0	0	0	0	0	0	0	0	0
0	0	0	0	0	0	0	0	0	0	0	0	0	0	0	0	0	0	0
0	0	0	0	0	0	0	0	0	0	0	0	0	0	0	0	0	0	0
0	0	0	0	0	0	0	0	0	0	0	0	0	0	0	0	0	0	0
0	0	0	0	0	0	0	0	0	0	0	0	0	0	0	0	0	0	0
0	0	0	0	0	0	0	0	0	0	0	0	0	0	0	0	0	0	0
0	0	0	0	0	0	0	0	0	0	0	0	0	0	0	0	0	0	0
0	0	0	0	0	0	0	0	0	0	0	0	0	0	0	0	0	0	0
0	0	0	0	0	0	0	0	0	0	0	0	0	0	0	0	0	0	0
0	0	0	0	0	0	0	0	0	0	0	0	0	0	0	0	0	0	0
0	0	0	0	0	0	0	0	0	0	0	0	0	0	0	0	0	0	0
0	0	0	0	0	0	0	0	0	0	0	0	0	0	0	0	0	0	0
0	0	0	0	0	0	0	0	0	0	0	0	0	0	0	0	0	0	0
0	0	0	0	0	0	0	0	0	0	0	0	0	0	0	0	0	0	0
0	0	0	0	0	0	0	0	0	0	0	0	0	0	0	0	0	0	0
0	0	0	0	0	0	0	0	0	0	0	0	0	0	0	0	0	0	0
0	0	0	0	0	0	0	0	0	0									
Jan_DL070	1238	1129	392	501	47	78	220	228	0	368	110	38	323	406	415	254	409	184
318	297	144	10	49	153	185	123	161	119	85	54	78	61	133	0	79	24	92
0	10	91	16	114	0	138	60	43	126	174	39	0	147	0	32	0	103	0
38	0	0	13	97	93	0	28	72	15	6	0	38	0	0	46	66	15	0
57	25	0	55	211	75	7	0	91	52	0	0	12	0	21	0	32	13	0
0	0	0	0	0	74	0	0	0	3	0	0	39	0	19	0	26	33	0
0	0	0	7	17	20	0	0	0	0	20	0	11	0	0	0	0	0	0
0	0	84	7	21	25	0	0	0	0	32	0	6	0	0	0	0	0	0
0	0	0	12	0	9	0	0	0	0	0	0	15	17	0	0	14	0	3
15	0	0	0	0	0	0	0	0	0	0	20	0	0	0	0	0	0	0
0	0	0	0	0	0	0	0	0	0	0	0	0	0	0	0	0	0	0

0	0	0	0	0	0	0	0	0	0	0	0	0	0	0	0	0	0	0
0	0	0	0	0	0	0	0	0	0	0	0	0	4	13	0	0	0	0
0	0	0	0	0	0	0	0	0	6	0	0	0	0	0	0	0	0	0
10	0	0	0	0	0	0	0	0	0	0	0	0	0	0	0	16	0	0
0	0	0	15	0	0	0	0	0	0	0	0	3	0	0	0	26	3	0
0	0	0	0	0	0	0	0	0	0	0	0	0	0	4	8	0	0	4
0	0	0	0	0	0	0	0	0	0	0	0	8	0	0	0	0	0	0
0	0	0	0	0	0	0	0	0	0	0	0	0	0	0	0	0	0	0
0	0	0	0	0	0	0	4	0	0	0	0	0	0	0	0	0	0	0
0	0	0	0	0	0	0	0	7	0	0	0	6	0	0	0	0	0	0
0	0	0	0	0	0	0	5	0	0	14	0	0	6	0	0	0	0	0
0	0	0	0	0	0	0	0	0	0	0	0	0	0	0	0	0	0	0
0	0	0	0	0	0	0	0	0	0	0	0	0	0	0	0	0	0	0
0	0	0	0	0	0	0	0	0	0	0	0	0	0	0	0	0	0	0
0	0	0	0	0	0	0	4	0	0	0	0	0	0	0	0	0	0	0
0	0	0	0	0	0	0	0	0	0	0	0	0	0	0	0	0	0	0
0	0	0	0	0	0	3	8	0	0	0	0	0	0	0	0	0	0	0
0	0	0	0	0	0	0	0	0	0	0	0	0	0	0	0	0	0	0
0	0	0	0	0	0	0	0	0	18	0	3	0	0	0	0	0	0	0
0	5	0	0	0	0	0	0	0	0	0	0	0	0	0	0	0	0	2
0	0	0	0	0	0	0	0	0	5	0	0	0	0	0	0	0	0	0
0	0	0	0	0	0	0	0	0	0	0	0	0	0	0	0	0	0	0
0	0	0	0	11	0	0	0	0	0	0	0	0	0	0	0	0	0	0
0	0	0	0	0	0	0	0	0	0	10	0	0	0	0	0	0	0	0
0	0	0	0	0	0	0	0	0	0	0	0	0	0	0	0	0	0	0
0	0	0	0	0	0	0	0	0	0	0	0	0	0	0	0	0	0	0
0	0	0	0	0	0	0	0	0	0	0	0	0	0	0	0	0	0	0
0	0	0	0	0	0	0	0	0	0	0	0	0	0	0	0	0	0	0
0	0	0	0	0	0	4	0	0	0	0	0	0	0	0	0	0	0	0
0	0	0	0	0	0	0	0	0	0	0	0	0	0	0	0	0	0	0
0	0	0	0	0	0	0	0	0	0	0	0	0	0	0	0	0	0	0
0	0	0	13	0	0	0	0	0	0	0	0	0	0	0	0	0	0	0
0	0	0	0	0	0	0	0	0	0	0	0	0	0	0	0	0	0	0
0	0	0	0	0	0	5	0	0	0	0	0	0	0	0	0	0	0	0
0	0	0	0	0	0	0	0	0	0	0	0	0	0	0	0	0	0	0
0	0	0	0	0	0	0	0	0	0	0	0	0	0	0	0	0	0	0
0	0	0	0	0	0	0	0	0	0	0	0	0	0	0	0	0	0	0

32	0	0	0	0	0	0	0	0	0	0	0	0	0	0	0	0	0	0
0	0	0	0	0	0	0	0	0	0	0	0	0	0	0	0	0	0	0
0	0	0	0	0	9	0	0	0	0	0	0	0	0	0	0	0	0	0
0	0	0	0	0	0	0	3	0	0	0	0	0	0	0	6	0	0	0
0	0	0	0	0	0	0	0	0	0	0	0	0	0	0	0	0	0	0
0	0	0	0	0	0	0	0	0	0	0	0	0	0	0	0	0	0	0
0	0	0	0	0	0	0	0	0	0	0	0	0	0	0	0	0	0	0
0	0	0	0	0	0	0	0	0	0	0	0	0	0	0	0	0	0	0
0	0	0	0	0	0	0	0	0	0	0	0	0	0	0	0	0	0	0
0	0	0	0	0	0	0	0	0	0	0	0	0	0	0	0	0	0	0
0	0	0	0	0	0	0	0	0	0	0	0	0	0	0	0	0	0	0
0	0	0	0	0	0	0	0	0	0	0	0	0	0	0	0	0	0	0
0	0	0	0	0	0	0	0	0	0	0	0	0	0	0	0	0	0	0
0	0	0	0	0	0	0	0	0	0	0	0	0	0	0	0	0	0	0
0	0	0	7	0	0	0	0	0	0	0	0	0	0	0	0	0	0	0
0	0	0	0	0	0	0	0	0	0	0	0	0	0	0	0	0	0	0
0	0	0	0	0	0	0	0	0	0	0	0	0	0	0	0	0	0	0
0	0	0	0	0	0	0	0	0	0	0	0	0	0	0	0	0	0	0
0	0	0	0	0	0	0	0	0	0	0	0	0	0	0	0	0	0	0
0	0	0	0	0	0	0	0	0	0	0	0	0	0	0	0	0	0	0
0	0	0	0	0	0	0	0	0	0	0	0	0	0	0	0	0	0	0
0	0	0	0	0	0	0	0	0	0	0	0	0	0	0	0	0	0	0
0	0	0	0	0	0	0	0	0	0	0	0	0	0	0	9	0	0	0
0	0	0	0	0	0	0	0	0	0	0	0	0	0	0	0	0	0	0
0	0	0	0	0	0	0	0	0	0	0	0	0	0	0	0	0	0	0
0	0	0	0	0	0	0	0	0	0	0	0	0	0	0	0	0	0	0
0	0	0	0	0	0	0	0	0	0	0	0	0	0	0	0	0	0	0
0	0	0	0	0	0	0	0	0	0	0	0	0	0	0	0	0	0	0
0	0	0	0	0	0	0	0	0	0	0	0	0	0	0	0	0	0	0
0	0	0	0	0	0	0	0	0	0	0	0	0	0	0	0	0	0	7
0	0	0	0	0	0	0	0	0	0	0	0	0	0	0	0	0	0	0
0	0	0	0	0	0	0	0	0	0	0	0	0	0	0	0	0	0	0
0	0	0	0	0	0	0	0	0	0	0	0	0	0	0	0	0	0	0
0	0	0	0	0	0	0	0	0	0	0	0	0	0	0	0	0	0	0
0	0	0	0	6	0	0	0	0	0	0	0	0	0	0	0	0	0	0
0	0	0	0	0	0	0	0	0	0	0	0	0	0	0	0	0	0	0
0	0	0	0	0	0	0	0	0	0	0	0	0	0	0	0	0	0	0

0	0	0	0	0	0	0	0	0	0	0	0	0	0	0	0	0	0	0
0	0	0	0	0	0	0	0	0	0	0	0	0	0	0	0	0	0	0
0	0	0	0	0	0	0	0	0	0	0	0	0	0	0	0	0	0	0
0	0	0	0	0	0	0	0	0	0	0	0	0	0	0	0	0	0	0
0	0	0	0	0	0	0	0	0	0	0	0	0	0	0	0	5	0	0
0	0	0	0	0	0	0	0	0	0	0	0	0	0	0	0	0	0	0
0	0	0	0	0	0	0	0	0	0	0	0	0	0	0	0	0	0	0
0	0	0	0	0	0	0	0	0	0	0	0	0	0	0	0	0	0	0
0	0	0	0	0	0	0	0	0	0	0	0	0	0	0	0	0	0	0
0	0	0	0	0	0	0	0	0	0	0	0	0	0	0	0	0	0	0
0	0	0	0	0	0	0	0	0	0	0	0	0	0	0	0	0	0	0
0	0	0	0	0	0	0	0	0	0	0	0	0	0	0	0	0	0	0
0	0	0	0	0	0	0	0	0	0	0	0	0	0	0	0	0	0	0
0	0	0	0	0	0	0	0	0	4	4	4	0	0	0	0	0	0	0
0	0	0	0	0	0	0	0	0	0	0	0	0	0	0	0	0	0	0
0	0	0	0	0	0	0	0	0	0	0	0	0	0	0	0	0	0	0
0	0	0	0	0	0	0	0	0	0	0	0	0	0	0	0	0	0	0
0	0	0	0	0	0	0	0	0	0	0	0	0	0	0	0	0	0	0
0	0	0	0	0	0	0	0	0	0	0	0	0	0	0	0	0	0	0
0	0	0	0	0	0	0	0	0	0	0	0	0	0	0	0	0	0	0
0	0	0	0	0	0	0	0	0	0	0	0	0	0	0	0	0	0	0
0	0	0	0	0	0	0	0	0	0	0	0	0	0	0	0	0	0	0
0	0	0	0	0	0	0	0	0	0	0	0	0	0	0	0	0	0	0
0	0	0	0	0	0	0	0	0	0	0	0	0	0	0	0	0	0	0
0	0	0	0	0	0	0	0	0	0	0	0	0	0	0	0	0	0	0
0	0	0	0	0	0	0	0	0	0	0	0	0	0	0	0	0	0	0
0	0	0	0	0	0	0	0	0	0	0	0	0	0	0	0	3	0	0
0	0	0	0	0	0	0	0	0	0	0	0	0	0	0	0	0	0	0
0	0	0	0	0	0	0	0	0	0	0	0	0	0	0	0	0	0	0
0	0	0	0	0	0	0	0	0	0	0	0	0	0	0	0	0	0	0
0	0	0	0	0	0	0	0	0	0	0	0	0	0	0	0	0	0	0
0	0	0	0	0	0	0	0	0	0	0	0	0	0	0	0	0	0	0
0	0	0	0	0	0	0	0	0	0	0	0	0	0	0	0	0	0	0
0	0	0	0	0	0	0	0	0	0	0	0	0	0	0	0	0	0	0
0	0	0	0	0	0	0	0	0	0	0	0	0	0	0	0	0	0	0
0	0	0	0	0	0	0	0	0	0	0	0	0	0	0	0	0	0	0
0	0	0	0	0	0	0	0	0	0	0	0	0	0	0	0	0	0	0

0	0	0	0	0	0	0	0	0	0	0	0	0	0	0	0	0	0	0
0	0	0	0	0	0	0	0	0	0	0	0	0	0	0	0	0	0	0
0	0	0	0	0	0	0	0	0	0	0	0	0	0	0	0	0	0	0
0	0	0	0	0	0	0	0	0	0	0	0	0	0	0	0	0	0	0
0	0	0	0	0	0	0	0	0	0	0	0	0	0	0	0	0	0	0
0	0	0	0	0	0	0	0	0	0	0	0	0	0	0	0	0	0	0
0	0	0	0	0	0	0	0	0	0	0	0	0	0	0	0	0	0	0
0	0	0	0	0	0	0	0	0	0	0	0	0	0	0	0	0	0	0
0	0	0	0	0	0	0	0	0	0	0	0	0	0	0	0	0	0	0
0	0	0	0	0	0	0	0	0	0	0	0	0	0	0	0	0	0	0
0	0	0	0	0	0	0	0	0	0	0	0	0	0	0	0	0	0	0
0	0	0	0	0	0	0	0	0	0	0	0	0	0	0	0	0	0	2
2	0	0	0	0	0	0	0	0	0	0	0	0	0	0	0	0	0	0
0	0	0	0	0	0	0	0	0	0	0	0	0	0	0	0	0	0	0
0	0	0	0	0	0	0	0	0	0	0	0	0	0	0	0	0	0	0
0	0	0	0	0	0	0	0	0	0	0	0	0	0	0	0	0	0	0
0	0	0	0	0	0	0	0	0	0	0	0	0	0	0	0	0	0	0
0	0	0	0	0	0	0	0	0	0	0	0	0	0	0	0	0	0	0
0	0	0	0	0	0	0	0	0	0	0	0	0	0	0	0	0	0	0
0	0	0	0	0	0	0	0	0	0	0	0	0	0	0	0	0	0	0
0	0	0	0	0	0	0	0	0	0	0	0	0	0	0	0	0	0	0
0	0	0	0	0	0	0	0	0	0	0	0	0	0	0	0	0	0	0
0	0	0	0	0	0	0	0	0	0	0	0	0	0	0	0	0	0	0
0	0	0	0	0	0	0	0	0	0	0	0	0	0	0	0	0	0	0
0	0	0	0	0	0	0	0	0	0	0	0	0	0	0	0	0	0	0
0	0	0	0	0	0	0	0	0	0	0	0	0	0	0	0	0	0	0
0	0	0	0	0	0	0	0	0	0	0	0	0	0	0	0	0	0	0
0	0	0	0	0	0	0	0	0	0	0	0	0	0	0	0	0	0	0
0	0	0	0	0	0	0	0	0	0	0	0	0	0	0	0	0	0	0
0	0	0	0	0	0	0	0	0	0	0	0	0	0	0	0	0	0	0
0	0	0	0	0	0	0	0	0	0	0	0	0	0	0	0	0	0	0
0	0	0	0	0	0	0	0	0	0	0	0	0	0	0	0	0	0	0
0	0	0	0	0	0	0	0	0	0	0	0	0	0	0	0	0	0	0
0	0	0	0	0	0	0	0	0	0	0	0	0	0	0	0	0	0	0
0	0	0	0	0	0	0	0	0	0	0	0	0	0	0	0	0	0	0
0	0	0	0	0	0	0	0	0	0	0	0	0	0	0	0	0	0	0
0	0	0	0	0	0	0	0	0	0	0	0	0	0	0	0	0	0	0

0	0	0	0	0	0	0	0	0	0	0	0	0	0	0	0	0	0	0
0	0	0	0	0	0	0	0	0	0	0	0	0	0	0	0	0	0	0
0	0	0	0	0	0	0	0	0	0	0	0	0	0	0	0	0	0	0
0	0	0	0	0	0	0	0	0	0	0	0	0	0	0	0	0	0	0
0	0	0	0	0	0	0	0	0	0	0	0	0	0	0	0	0	0	0
0	0	0	0	0	0	0	0	0	0	0	0	0	0	0	0	0	0	0
0	0	0	0	0	0	0	0	0	0	0	0	0	0	0	0	0	0	0
0	0	0	0	0	0	0	0	0	0	0	0	0	0	0	0	0	0	0
0	0	0	0	0	0	0	0	0	0	0	0	0	0	0	0	0	0	0
0	0	0	0	0	0	0	0	0	0	0	0	0	0	0	0	0	0	0
0	0	0	0	0	0	0	0	0	0	0	0	0	0	0	0	0	0	0
0	0	0	0	0	0	0	0	0	0	0	0	0	0	0	0	0	0	0
0	0	0	0	0	0	0	0	0	0	0	0	0	0	0	0	0	0	0
0	0	0	0	0	0	0	0	0	0	0	0	0	0	0	0	0	0	0
0	0	0	0	0	0	0	0	0	0	0	0	0	0	0	0	0	0	0
0	0	0	0	0	0	0	0	0	0	0	0	0	0	0	0	0	0	0
0	0	0	0	0	0	0	0	0	0	0	0	0	0	0	0	0	0	0
0	0	0	0	0	0	0	0	0	0									
Jan_DL071	1024	1017	695	591	10	0	15	11	361	164	120	136	160	47	234	139	49	59
72	256	90	51	141	104	469	159	413	92	0	0	60	84	100	0	73	71	0
95	44	0	0	48	0	0	60	21	58	40	259	59	33	63	266	176	76	0
0	529	199	10	0	51	39	47	156	30	66	0	149	0	87	0	0	51	0
43	293	32	59	0	43	25	19	55	124	0	59	0	0	26	0	8	0	0
0	0	0	72	95	0	111	8	0	6	0	8	0	0	29	0	0	0	0
0	0	0	0	3	0	0	0	0	0	0	0	0	0	0	0	0	0	65
14	0	0	0	9	101	0	0	0	6	0	0	0	0	0	26	0	12	0
0	4	12	21	0	0	22	0	55	0	0	0	21	0	0	0	0	0	10
0	0	15	0	0	0	0	0	0	0	0	19	0	0	0	0	10	0	0
0	0	0	0	0	0	32	0	0	4	0	0	0	0	0	0	0	0	16
0	0	0	0	10	0	0	0	0	0	0	0	0	0	0	0	0	0	14
0	0	0	0	0	0	0	0	0	0	0	12	15	4	39	0	8	0	0
0	0	0	0	0	0	0	0	0	0	0	53	0	0	0	0	0	0	0
0	0	0	0	0	0	0	0	0	0	0	0	0	0	0	0	0	0	0
0	0	0	0	0	0	0	0	5	0	32	12	28	53	0	0	0	0	0
0	0	0	0	0	0	0	0	0	0	0	0	0	0	0	0	0	0	0
0	0	0	10	0	0	0	0	0	0	0	0	0	0	0	0	37	0	0
0	0	0	0	4	0	0	0	11	0	0	0	0	0	0	0	0	0	0

0	0	0	0	0	0	4	0	0	0	0	72	0	0	0	19	0	0	0
0	0	0	0	12	0	0	0	0	0	0	0	5	0	0	0	0	0	0
0	0	0	0	0	0	0	7	0	0	0	7	0	7	0	0	3	0	0
0	0	0	0	0	6	0	0	0	0	0	0	0	0	0	0	0	0	0
0	0	0	0	0	0	0	0	0	0	0	0	0	0	0	0	0	0	0
0	0	0	0	0	0	0	0	0	0	0	0	0	0	0	10	0	0	0
0	0	0	6	8	0	0	0	0	0	0	0	0	0	0	0	0	13	0
0	0	0	0	0	0	0	0	0	0	0	0	0	0	0	0	0	2	0
0	0	0	0	0	0	0	0	0	0	0	0	0	0	0	0	0	8	0
0	0	0	0	0	5	0	10	0	0	0	0	0	0	0	0	0	0	0
0	0	0	0	0	0	0	0	0	0	0	0	0	0	0	0	0	0	0
0	0	0	0	0	0	0	0	0	0	0	0	0	0	0	0	0	0	0
0	0	0	0	0	0	0	4	0	0	0	0	0	0	0	0	0	0	0
0	0	0	0	0	0	0	0	0	0	0	0	0	0	0	0	0	0	0
0	0	0	0	0	0	0	0	0	0	0	0	0	0	0	0	0	0	0
0	0	0	0	0	0	0	0	0	0	0	0	0	0	0	0	0	5	0
0	0	0	0	0	0	0	0	0	0	0	0	78	0	0	0	0	0	0
0	0	0	0	0	0	0	0	0	0	7	0	0	0	9	0	0	0	0
0	0	0	0	0	0	0	0	0	0	0	0	0	0	0	0	0	0	0
0	0	0	0	0	0	0	0	0	0	0	0	0	0	0	0	0	0	0
0	0	0	0	0	0	0	0	0	0	0	0	0	0	0	0	0	0	0
0	0	0	0	0	0	0	0	0	0	0	0	0	0	0	0	0	0	0
0	0	0	0	0	0	0	0	0	0	0	0	0	0	0	0	0	0	0
0	0	0	0	0	0	0	0	0	0	0	0	0	0	0	0	0	0	0
0	0	0	0	0	0	0	0	0	0	0	0	0	0	0	0	0	0	0
0	0	0	0	0	0	0	0	0	0	0	0	0	0	0	0	0	0	0
0	0	0	0	0	0	0	0	0	0	0	0	0	0	0	0	0	0	0
0	0	0	0	0	0	0	0	0	0	0	0	0	0	0	0	0	0	0
0	0	0	0	0	0	0	0	0	0	0	0	0	0	0	0	0	0	0
0	0	0	0	0	0	0	0	0	0	0	0	0	0	0	0	0	0	0
0	0	0	0	0	0	0	0	0	0	0	0	0	0	0	0	0	0	0
0	0	0	0	0	0	0	0	0	0	0	0	0	0	0	0	0	0	0
0	0	0	0	0	0	0	0	0	0	0	0	0	0	0	0	0	0	0
13	0	0	0	0	0	0	0	0	0	0	0	0	0	0	0	0	0	0
0	0	0	0	0	0	0	0	0	0	0	0	5	0	0	0	0	0	0
0	0	0	0	0	0	0	0	0	0	0	0	0	0	0	0	0	0	0
0	0	0	0	0	0	0	0	0	0	0	0	0	0	0	0	0	0	0

0	0	0	0	0	0	0	0	0	0	0	0	0	0	4	0	0	0	0
0	0	0	2	0	0	0	0	0	0	0	0	0	0	0	0	0	0	0
0	0	0	0	0	0	0	0	0	0	0	0	0	0	0	0	0	0	0
0	0	0	0	0	0	0	0	0	0	0	0	0	0	0	0	0	0	0
0	0	0	0	0	0	0	15	0	0	0	0	0	0	0	0	0	0	0
0	0	0	0	0	0	0	0	0	0	0	0	0	0	0	0	0	0	0
0	0	0	0	0	0	0	0	0	0	0	0	0	0	0	0	0	0	0
0	0	0	0	0	0	0	0	0	0	0	0	0	0	0	0	0	0	0
0	0	0	0	0	0	0	0	0	0	0	0	0	0	0	0	0	0	0
0	0	0	0	0	0	0	0	0	0	0	0	0	0	0	0	2	0	0
0	0	0	0	0	0	0	0	0	0	0	0	0	0	0	0	0	0	0
0	0	0	0	0	0	0	0	0	0	0	0	0	0	0	0	0	0	0
0	0	0	0	0	0	0	0	0	0	0	0	0	0	0	0	0	0	0
0	0	0	0	0	0	0	0	0	0	0	0	0	0	0	0	0	0	0
0	0	0	0	0	0	0	0	0	0	0	0	0	0	0	0	0	0	0
0	0	0	0	0	0	0	0	0	0	0	0	0	0	0	0	0	0	0
0	0	0	0	0	0	0	0	0	0	0	0	0	0	0	0	0	0	0
0	0	0	0	0	0	0	0	0	0	0	0	0	0	0	0	0	0	0
0	0	0	0	0	0	0	0	0	0	0	0	0	0	0	0	0	0	0
0	0	0	0	0	0	0	0	0	0	0	0	0	0	0	0	0	0	0
0	0	0	0	0	0	0	0	0	0	0	0	0	0	0	0	0	0	0
0	0	0	0	0	0	0	0	0	0	0	0	0	0	0	0	0	0	0
0	0	0	0	0	0	0	0	0	0	0	0	0	0	0	0	7	0	0
0	0	0	0	0	0	0	0	0	0	0	0	0	0	0	0	0	0	0
0	0	0	0	0	0	0	0	0	0	0	0	0	0	0	0	0	0	0
0	0	0	0	0	0	0	0	0	0	0	0	0	0	0	0	0	0	0
0	0	0	0	0	0	0	0	0	0	0	0	0	0	0	0	0	0	0
0	0	0	0	0	0	0	0	0	0	0	0	0	0	0	0	0	0	0
0	0	6	0	0	0	0	0	0	0	0	0	0	0	0	0	0	0	0
0	0	0	0	0	0	0	0	0	0	0	0	0	0	0	0	0	0	0
0	0	0	0	0	0	0	0	0	0	0	0	0	0	0	0	0	0	0
0	0	0	0	0	0	0	0	0	0	0	0	0	0	0	0	0	0	0
0	0	0	0	0	0	0	0	0	0	0	0	0	0	0	0	0	0	0
0	0	0	0	0	0	0	0	0	0	0	0	0	0	0	0	0	0	0
0	0	0	0	0	0	0	0	0	0	0	0	0	0	0	0	0	0	0
0	0	0	0	0	0	0	0	0	5	5	0	0	0	0	0	0	0	0
0	0	0	0	0	0	0	0	0	0	0	0	0	0	0	0	0	0	0

0	0	0	0	0	0	0	0	0	0	0	0	0	0	0	0	0	0	0
0	0	0	0	0	0	0	0	0	0	0	0	0	0	0	0	0	0	0
0	0	0	0	0	0	0	0	0	0	0	0	0	0	0	0	0	0	0
0	0	0	0	0	0	0	0	0	0	0	0	0	0	0	0	0	0	0
0	0	0	0	0	0	0	0	0	0	0	0	0	0	0	0	0	0	0
0	0	0	0	0	0	0	0	0	0	0	0	0	0	0	0	0	0	0
0	0	0	0	0	0	0	0	0	0	0	0	0	0	0	0	0	0	0
0	0	0	0	0	0	0	0	0	0	0	0	0	0	0	0	0	0	0
0	0	0	0	0	4	0	0	0	0	0	0	0	0	0	0	0	0	0
0	0	0	0	0	0	0	0	0	0	0	0	0	0	0	0	0	0	0
0	0	0	0	0	0	0	0	0	0	0	0	0	0	0	0	0	0	0
0	0	0	0	0	0	0	0	0	0	0	0	0	0	0	0	0	0	0
0	0	0	0	0	0	0	0	0	0	0	0	0	0	0	0	0	0	0
0	0	0	0	0	0	0	0	0	0	0	0	0	0	0	0	0	0	0
0	0	0	0	0	0	0	0	0	0	0	0	0	0	0	0	0	0	0
0	0	0	0	0	0	0	0	0	0	0	0	0	0	0	0	0	0	0
0	0	0	0	0	0	0	0	0	0	0	0	0	0	0	0	0	0	0
0	0	0	0	0	0	0	0	0	0	0	0	0	0	0	0	0	0	0
0	0	0	0	0	0	0	0	0	0	0	0	0	0	0	0	0	0	0
0	0	0	0	0	0	0	0	0	0	0	0	0	0	0	0	0	0	0
0	0	0	0	0	0	0	0	0	0	0	0	0	0	0	0	0	0	0
0	0	0	0	0	0	0	0	0	0	0	0	0	0	3	3	3	0	0
0	0	0	0	0	0	0	0	0	0	0	0	0	0	0	0	0	0	0
0	0	0	0	0	0	0	0	0	0	0	0	0	0	0	0	0	0	0
0	0	0	0	0	0	0	0	0	0	0	0	0	0	0	0	0	0	0
0	0	0	0	0	0	0	0	0	0	0	0	0	0	0	0	0	0	0
0	0	0	0	0	0	0	0	0	0	0	0	0	0	0	0	0	0	0
0	0	0	0	0	0	0	0	0	0	0	0	0	0	0	0	0	0	0
0	0	0	0	0	0	0	0	0	0	0	0	0	0	0	0	0	0	0
0	0	0	0	0	0	0	0	0	0	0	0	0	0	0	0	0	0	0
0	0	0	0	0	0	0	0	0	0	0	0	0	0	0	0	0	0	0
0	0	0	0	0	0	0	0	0	0	0	0	0	0	0	0	0	0	0
0	0	0	0	0	0	0	0	0	0	0	0	0	0	0	0	0	0	0
0	0	0	0	0	0	0	0	0	0	0	0	0	0	0	0	0	0	0
0	0	0	0	0	0	0	0	0	0	0	0	0	0	0	0	0	0	0
0	0	0	0	0	0	0	0	0	0	0	0	0	0	0	0	0	0	0
0	0	0	0	0	0	0	0	0	0	0	0	0	0	0	0	0	0	0

0	0	0	0	0	0	0	0	0	0	0	0	0	0	0	0	0	0	0
0	0	0	0	0	0	0	0	0	0	0	0	0	0	0	0	0	0	0
0	0	0	0	0	0	0	0	0	0	0	0	0	0	0	0	0	0	0
0	0	0	0	0	0	0	0	0	0	0	0	0	0	0	0	0	0	0
0	0	0	0	0	0	0	0	0	0	0	0	0	0	0	0	0	0	0
0	0	0	0	0	0	0	0	0	0	0	0	0	0	0	0	0	0	0
0	0	0	0	0	0	0	0	0	0	0	0	0	0	0	0	0	0	0
0	0	0	0	0	0	0	0	0	0	0	0	0	0	0	0	0	0	0
0	0	0	0	2	2	2	2	2	2	2	2	0	0	0	0	0	0	0
0	0	0	0	0	0	0	0	0	0	0	0	0	0	0	0	0	0	0
0	0	0	0	0	0	0	0	0	0	0	0	0	0	0	0	0	0	0
0	0	0	0	0	0	0	0	0	0	0	0	0	0	0	0	0	0	0
0	0	0	0	0	0	0	0	0	0	0	0	0	0	0	0	0	0	0
0	0	0	0	0	0	0	0	0	0	0	0	0	0	0	0	0	0	0
0	0	0	0	0	0	0	0	0	0	0	0	0	0	0	0	0	0	0
0	0	0	0	0	0	0	0	0	0	0	0	0	0	0	0	0	0	0
0	0	0	0	0	0	0	0	0	0	0	0	0	0	0	0	0	0	0
0	0	0	0	0	0	0	0	0	0	0	0	0	0	0	0	0	0	0
0	0	0	0	0	0	0	0	0	0	0	0	0	0	0	0	0	0	0
0	0	0	0	0	0	0	0	0	0	0	0	0	0	0	0	0	0	0
0	0	0	0	0	0	0	0	0	0	0	0	0	0	0	0	0	0	0
0	0	0	0	0	0	0	0	0	0	0	0	0	0	0	0	0	0	0
0	0	0	0	0	0	0	0	0	0	0	0	0	0	0	0	0	0	0
0	0	0	0	0	0	0	0	0	0	0	0	0	0	0	0	0	0	0
0	0	0	0	0	0	0	0	0	0	0	0	0	0	0	0	0	0	0
0	0	0	0	0	0	0	0	0	0	0	0	0	0	0	0	0	0	0
0	0	0	0	0	0	0	0	0	0	0	0	0	0	0	0	0	0	0
0	0	0	0	0	0	0	0	0	0	0	0	0	0	0	0	0	0	0
0	0	0	0	0	0	0	0	0	0	0	0	0	0	0	0	0	0	0
0	0	0	0	0	0	0	0	0	0	0	0	0	0	0	0	0	0	0
0	0	0	0	0	0	0	0	0	0	0	0	0	0	0	0	0	0	0
0	0	0	0	0	0	0	0	0	0	0	0	0	0	0	0	0	0	0
0	0	0	0	0	0	0	0	0	0	0	0	0	0	0	0	0	0	0
0	0	0	0	0	0	0	0	0	0	0	0	0	0	0	0	0	0	0
0	0	0	0	0	0	0	0	0	0	0	0	0	0	0	0	0	0	0
0	0	0	0	0	0	0	0	0	0	0	0	0	0	0	0	0	0	0
0	0	0	0	0	0	0	0	0	0	0	0	0	0	0	0	0	0	0

0	0	0	0	0	0	0	0	0	0	0	0	0	0	0	0	0	0	0
0	0	0	0	0	0	0	0	0	0	0	0	0	0	0	0	0	0	0
0	0	0	0	0	0	0	0	0	0	0	0	0	0	0	0	0	0	0
0	0	0	0	0	0	0	0	0	0	0	0	0	0	0	0	0	0	0
0	0	0	0	0	0	0	0	0	0	0	0	0	0	0	0	0	0	0
0	0	0	0	0	0	0	0	0	0	0	0	0	0	0	0	0	0	0
0	0	0	0	0	0	0	0	0	0	0	0	0	0	0	0	0	0	0
1	0	0	0	0	0	0	0	0	0	0	0	0	0	0	0	0	0	0
0	0	0	0	0	0	0	0	0	0	0	0	0	0	0	0	0	0	0
0	0	0	0	0	0	0	0	0	0									
Jan_DL072	938	971	125	220	972	106	147	139	9	132	111	23	144	50	220	51	44	108
102	0	226	0	109	202	47	80	53	100	45	170	43	37	68	0	0	0	170
0	14	54	32	53	0	48	0	43	0	0	22	0	34	0	0	0	38	92
119	0	0	68	0	0	44	16	0	27	189	0	0	66	0	0	73	0	0
18	0	0	33	199	0	10	11	63	8	0	0	25	0	0	8	0	40	0
0	0	0	0	0	12	0	0	0	0	144	18	63	0	0	16	0	0	0
0	0	0	0	18	0	0	0	0	0	19	19	19	35	0	0	44	52	0
0	0	0	39	0	0	0	0	0	0	27	0	9	0	23	42	0	0	0
0	0	0	26	0	39	0	0	0	0	8	0	0	0	0	3	62	0	0
0	0	12	0	0	0	0	0	0	0	57	0	0	0	11	0	9	0	18
0	41	0	0	0	0	0	0	25	4	25	34	0	0	0	0	0	0	0
8	0	0	0	0	54	0	0	0	0	0	0	0	0	0	0	0	0	0
0	0	23	8	0	0	0	0	0	0	0	0	0	0	0	0	7	0	10
0	0	0	5	0	0	0	0	0	0	0	0	0	14	0	0	0	0	0
0	0	0	0	0	0	0	0	0	0	0	0	0	0	8	0	0	0	0
0	17	0	0	0	0	0	0	0	0	0	0	0	0	0	0	0	0	0
0	12	0	0	0	0	0	0	0	5	0	4	0	4	0	0	0	0	0
0	0	4	0	21	0	6	0	0	0	0	0	61	0	0	0	0	0	0
0	0	0	0	0	0	0	0	0	0	0	0	0	9	0	0	0	0	0
0	0	10	0	0	0	0	0	0	0	0	0	0	0	0	0	0	0	0
0	8	0	19	0	4	0	4	7	0	0	0	0	0	0	0	0	0	0
0	0	0	0	0	0	0	0	0	0	0	10	0	0	0	0	0	0	0
0	0	0	0	0	0	0	0	0	34	0	0	0	0	0	0	0	0	0
0	0	0	0	0	0	8	0	0	0	0	0	12	0	0	0	0	0	0
0	0	0	0	0	0	0	0	0	0	0	0	0	0	0	0	0	0	0
0	0	0	0	0	0	0	0	0	0	0	0	0	0	0	0	0	0	0
0	0	0	21	0	0	0	0	0	0	0	0	0	0	0	0	0	0	0

0	0	0	0	41	0	0	0	0	0	0	0	0	0	0	0	0	0	0
0	0	0	0	0	0	0	0	0	0	0	0	0	0	5	0	0	0	0
0	0	0	0	0	0	0	0	0	0	0	0	0	0	0	0	0	0	0
0	0	0	0	0	0	0	0	0	0	0	0	0	0	0	0	0	0	0
0	0	0	0	0	0	0	6	0	0	0	0	0	9	0	0	0	0	0
0	0	0	0	0	0	0	0	0	0	0	0	0	0	0	0	0	0	0
0	0	0	0	0	0	0	0	0	0	0	0	0	0	0	0	0	0	0
0	0	0	0	0	0	0	0	0	0	0	0	0	0	0	0	0	0	0
0	0	0	0	0	0	0	0	0	0	0	0	0	0	0	0	0	0	0
0	0	0	0	0	0	0	38	0	0	0	0	0	0	0	5	0	0	0
0	0	0	0	0	0	0	0	0	0	0	0	0	0	0	0	0	0	0
0	0	0	0	0	0	0	0	0	0	0	0	0	0	0	0	0	0	0
0	0	0	0	0	0	0	0	0	0	0	0	0	0	0	0	0	0	0
0	0	0	0	0	0	0	0	0	0	0	0	0	0	0	0	0	0	0
0	0	0	0	0	0	0	0	0	0	0	0	0	0	0	0	0	0	0
0	0	0	0	0	0	0	0	0	0	0	0	0	0	0	11	0	0	0
0	0	0	0	0	0	0	0	0	0	0	0	0	0	0	0	0	0	0
0	0	0	0	0	0	0	0	0	0	0	0	0	0	0	0	0	0	0
0	0	0	0	0	0	0	0	0	0	0	0	0	0	0	0	0	0	0
0	0	0	0	0	0	0	0	0	0	0	0	0	0	0	0	0	0	0
0	0	0	0	0	0	0	0	0	0	0	0	0	0	0	0	0	0	0
0	0	0	0	0	0	0	0	0	0	0	0	0	0	0	0	0	0	0
0	0	0	0	0	0	0	0	0	0	0	0	0	0	0	0	0	0	0
0	0	0	0	0	0	0	0	0	0	0	0	0	0	0	0	0	0	0
6	0	0	0	0	0	0	0	0	0	0	0	0	0	0	0	0	0	0
0	0	0	0	0	0	0	0	0	0	0	0	0	0	0	0	0	0	0
0	0	0	0	0	0	0	0	0	0	0	0	0	0	0	0	0	0	0
0	0	0	0	0	0	0	0	0	0	0	0	0	0	0	0	0	0	0
0	0	0	0	0	0	0	0	0	0	0	0	0	0	0	0	0	0	0
0	0	0	0	0	0	0	0	0	0	0	0	0	0	0	0	0	0	0
0	0	0	0	0	0	0	0	0	0	0	0	0	0	0	0	0	0	0
0	0	0	0	0	0	0	0	0	0	0	0	0	0	0	0	0	0	0
0	0	0	0	0	0	0	0	0	0	0	0	0	0	0	0	0	0	0
0	0	0	0	0	0	0	0	0	0	0	0	0	0	0	0	0	0	0
0	0	0	0	0	0	0	0	0	0	0	0	0	0	0	0	0	0	0
0	0	0	0	0	0	0	0	0	0	0	0	0	0	0	0	0	0	0
0	0	0	0	0	0	0	0	0	0	0	0	0	0	0	0	0	0	0

0	0	0	0	0	0	0	0	0	0	0	0	0	0	0	0	0	0	0
0	0	0	0	0	0	0	0	0	0	0	0	0	0	0	0	0	0	0
0	0	0	0	0	0	0	0	0	0	0	0	0	0	0	0	0	0	0
0	0	0	0	0	0	0	0	0	0	0	0	0	0	0	0	0	0	0
0	0	0	0	0	0	0	0	0	0	0	0	0	0	0	0	0	0	0
0	0	0	0	0	0	0	0	0	0	0	0	0	0	0	0	0	0	0
0	0	0	0	0	0	0	0	0	7	0	0	0	0	0	0	0	0	0
0	0	0	0	0	0	0	0	0	0	0	0	0	0	0	0	0	0	0
0	0	0	0	0	0	0	0	0	0	0	0	0	0	0	0	0	0	0
0	0	0	0	0	0	0	0	0	0	0	0	0	0	0	0	0	0	0
0	0	0	0	0	0	0	0	0	0	0	0	0	0	0	0	0	0	0
0	0	0	0	0	0	0	0	0	0	0	0	0	0	0	0	0	0	0
0	0	0	0	0	0	0	0	0	0	0	0	0	0	0	0	0	0	0
0	0	0	0	0	0	0	0	0	0	0	0	0	0	0	0	0	0	0
0	0	0	0	0	0	0	0	0	0	0	0	0	0	0	0	0	0	0
0	0	0	0	0	0	0	0	0	0	0	0	0	0	0	0	5	0	0
0	0	0	0	0	0	0	0	0	0	0	0	0	0	0	0	0	0	0
0	0	0	0	0	0	0	0	0	0	0	0	0	0	0	0	0	0	0
0	0	0	0	0	0	0	0	0	0	0	0	0	0	0	0	0	0	0
0	0	0	0	0	0	0	0	0	0	0	0	0	0	0	0	0	0	0
0	0	0	0	0	0	0	0	0	0	0	0	0	0	0	0	0	0	0
0	0	0	0	0	0	0	0	0	0	0	0	0	0	0	0	0	0	0
0	0	0	0	0	0	0	0	0	0	0	0	0	0	0	0	0	0	0
0	0	0	0	0	0	0	0	0	0	0	0	0	0	0	0	0	0	0
0	0	0	0	0	0	0	0	0	0	0	0	0	0	0	0	0	0	0
0	0	0	0	0	0	0	0	0	0	0	0	0	0	0	0	0	0	0
0	0	0	0	0	0	0	0	0	0	0	0	0	0	0	0	0	0	0
0	0	0	0	0	0	0	0	0	0	0	0	0	0	0	0	0	0	0
0	0	0	0	0	0	0	0	0	0	0	0	0	0	0	0	0	0	0
0	0	0	0	0	0	0	0	0	0	0	0	0	0	0	0	0	0	0
0	0	0	0	0	0	0	0	0	0	0	0	0	0	0	0	0	0	0
0	0	0	0	0	0	0	0	0	0	0	0	0	0	0	0	0	0	0
0	0	0	0	0	0	0	0	0	0	0	0	0	0	0	0	0	0	0
0	0	0	0	0	0	0	0	0	0	0	0	0	0	0	0	0	0	0
0	0	0	0	0	0	0	0	0	0	0	0	0	0	0	0	0	0	0
0	0	0	0	0	0	0	0	0	0	0	0	0	0	0	0	0	0	0
0	0	3	3	3	3	0	0	0	0	0	0	0	0	0	0	0	0	0

0	0	0	0	0	0	0	0	0	0	0	0	0	0	0	0	0	0	0
0	0	0	0	0	0	0	0	0	0	0	0	0	0	0	0	0	0	0
0	0	0	0	0	0	0	0	0	0	0	0	0	0	0	0	0	0	0
0	0	0	0	0	0	0	0	0	0	0	0	0	0	0	0	0	0	0
0	0	0	0	0	0	0	0	0	0	0	0	0	0	0	0	0	0	0
0	0	0	0	0	0	0	0	0	0	0	0	0	0	0	0	0	0	0
0	0	0	0	0	0	0	0	0	0	0	0	0	0	0	0	0	0	0
0	0	0	0	0	0	0	0	0	0	0	0	0	0	0	0	0	0	0
0	0	0	0	0	0	0	0	0	0	0	0	0	0	0	0	0	0	0
0	0	0	0	0	0	0	0	0	0	0	0	0	0	0	0	0	0	0
0	0	0	0	0	0	0	0	0	0	0	0	0	0	0	0	0	0	0
0	0	0	0	0	0	0	0	0	0	0	0	0	0	0	0	0	0	0
0	0	0	0	0	0	0	0	0	0	0	0	0	0	0	0	0	0	0
0	0	0	0	0	0	0	0	0	0	0	0	0	0	0	0	0	0	0
0	0	0	0	0	0	0	0	0	0	0	0	0	0	0	0	0	0	0
0	0	0	0	0	0	0	0	0	0	0	0	0	0	0	0	0	0	0
0	0	0	0	0	0	0	0	0	0	0	0	0	0	0	0	0	0	0
0	0	0	0	0	0	0	0	0	0	0	0	0	0	0	0	0	2	2
2	2	2	2	0	0	0	0	0	0	0	0	0	0	0	0	0	0	0
0	0	0	0	0	0	0	0	0	0	0	0	0	0	0	0	0	0	0
0	0	0	0	0	0	0	0	0	0	0	0	0	0	0	0	0	0	0
0	0	0	0	0	0	0	0	0	0	0	0	0	0	0	0	0	0	0
0	0	0	0	0	0	0	0	0	0	0	0	0	0	0	0	0	0	0
0	0	0	0	0	0	0	0	0	0	0	0	0	0	0	0	0	0	0
0	0	0	0	0	0	0	0	0	0	0	0	0	0	0	0	0	0	0
0	0	0	0	0	0	0	0	0	0	0	0	0	0	0	0	0	0	0
0	0	0	0	0	0	0	0	0	0	0	0	0	0	0	0	0	0	0
0	0	0	0	0	0	0	0	0	0	0	0	0	0	0	0	0	0	0
0	0	0	0	0	0	0	0	0	0	0	0	0	0	0	0	0	0	0
0	0	0	0	0	0	0	0	0	0	0	0	0	0	0	0	0	0	0
0	0	0	0	0	0	0	0	0	0	0	0	0	0	0	0	0	0	0
0	0	0	0	0	0	0	0	0	0	0	0	0	0	0	0	0	0	0
0	0	0	0	0	0	0	0	0	0	0	0	0	0	0	0	0	0	0
0	0	0	0	0	0	0	0	0	0	0	0	0	0	0	0	0	0	0
0	0	0	0	0	0	0	0	0	0	0	0	0	0	0	0	0	0	0
0	0	0	0	0	0	0	0	0	0	0	0	0	0	0	0	0	0	0
0	0	0	0	0	0	0	0	0	0	0	0	0	0	0	0	0	0	0

0	0	0	0	0	0	0	0	0	0	0	0	0	0	0	0	0	0	0
0	0	0	0	0	0	0	0	0	0	0	0	0	0	0	0	0	0	0
0	0	0	0	0	0	0	0	0	0	0	0	0	0	0	0	0	0	0
0	0	0	0	0	0	0	0	0	0	0	0	0	0	0	0	0	0	0
0	0	0	0	0	0	0	0	0	0	0	0	0	0	0	0	0	0	0
0	0	0	0	0	0	0	0	0	0	0	0	0	0	0	0	0	0	0
0	0	0	0	0	0	0	0	0	0	0	0	0	0	0	0	0	0	0
0	0	0	0	0	0	0	0	0	0	0	0	0	0	0	0	0	0	0
0	0	0	0	0	0	0	0	0	0	0	0	0	0	0	0	0	0	0
0	0	0	0	0	0	0	0	0	0	0	0	0	0	0	0	0	0	0
0	0	0	0	0	0	0	0	0	0	0	0	0	0	0	0	0	0	0
0	0	0	0	0	0	0	0	0	0	0	0	0	0	0	0	0	0	0
0	0	0	0	0	0	0	0	0	0	0	0	0	0	0	0	0	0	0
0	0	0	0	0	0	0	0	0	0	0	0	0	0	0	0	0	0	0
0	0	0	0	0	0	0	0	0	0	0	0	0	0	0	0	0	0	0
0	0	0	0	0	0	0	0	0	0	0	0	0	0	0	0	0	0	0
0	0	0	0	0	0	0	0	0	0	0	0	0	0	0	0	0	0	0
0	0	0	0	0	0	0	0	0	0	0	0	0	0	0	0	0	0	0
0	0	0	0	0	0	0	0	0	0	0	0	0	0	0	0	0	0	0
0	0	0	0	0	0	0	0	0	0	0	0	0	0	0	0	0	0	0
0	0	0	0	0	0	0	0	0	0	0	0	0	0	0	0	0	0	0
0	0	0	0	0	0	0	0	0	0	0	0	0	0	0	0	0	0	0
0	0	0	0	0	0	0	0	0	0	0	0	0	0	0	0	0	0	0
0	0	0	0	0	0	0	0	0	0	0	0	0	0	0	0	0	0	0
0	0	0	0	0	0	0	0	0	0	0	0	0	0	0	0	0	0	0
0	0	0	0	0	0	0	0	0	0	0	0	0	0	0	0	0	0	0
0	0	0	0	0	0	0	0	0	0	0	0	0	0	0	0	0	0	0
0	0	0	0	0	0	0	0	0	0	0	0	0	0	0	0	0	0	0
0	0	0	0	0	0	0	0	0	0	0	0	0	0	0	0	0	0	0
0	0	0	0	0	0	0	0	0	0	0	0	0	0	0	0	0	0	0
0	0	0	0	0	0	0	0	0	0	0	0	0	0	0	0	0	0	0
0	0	0	0	0	0	0	0	0	0	0	0	0	0	0	0	0	0	0
0	0	0	0	0	0	0	0	0	0	0	0	0	0	0	0	0	0	0
0	0	0	0	0	0	0	0	0	0	0	0	0	0	0	0	0	0	0
0	0	0	0	0	0	0	0	0	0	0	0	0	0	0	0	0	0	0
0	0	0	0	0	0	0	0	0	0	0	0	0	0	0	0	0	0	0
0	0	0	0	0	0	0	0	0	0	0	0	0	0	0	0	0	0	0
0	0	0	0	0	0	0	0	0	0	0	0	0	0	0	0	0	0	0

0	0	0	0	0	0	0	0	0	0	0	0	0	0	0	0	0	0	0
0	0	0	0	0	0	0	0	0	0									
Jan_DL077	782	697	275	330	133	61	22	31	33	136	0	19	107	0	108	198	9	62
30	0	112	265	59	108	96	81	94	49	68	0	44	49	61	0	57	36	0
0	134	0	0	30	0	0	0	0	57	0	22	144	31	0	28	6	0	0
0	0	0	26	0	23	18	10	30	52	113	0	113	0	300	0	7	46	0
0	0	17	0	0	12	8	0	0	0	0	0	17	0	15	0	13	0	0
0	0	0	97	28	0	51	0	13	3	0	0	0	0	15	0	18	0	49
0	49	0	0	0	28	0	0	0	0	6	0	0	0	0	0	0	0	0
0	0	0	0	0	57	0	0	20	5	0	0	16	0	31	0	0	0	0
0	13	0	0	0	7	0	0	0	0	0	0	7	0	33	0	0	37	17
0	0	0	0	9	0	0	24	0	7	0	0	0	0	0	20	0	0	0
33	16	0	0	0	0	0	0	0	6	0	0	0	0	0	0	0	0	0
13	0	0	0	0	0	7	0	0	0	0	32	38	0	0	0	0	0	0
0	0	0	0	0	0	0	0	0	0	0	0	0	2	0	0	0	0	0
0	0	0	0	35	0	0	11	0	0	0	0	3	9	0	0	0	0	0
0	0	0	0	12	0	7	0	0	0	6	0	0	10	0	5	0	0	0
0	0	0	0	0	0	0	0	0	0	0	0	0	0	0	0	0	0	0
0	0	0	0	0	0	0	3	0	7	0	0	0	0	0	0	0	0	0
0	0	0	0	0	0	0	0	0	0	0	7	0	0	0	0	0	0	0
0	0	0	28	0	0	0	0	0	0	0	0	0	0	6	0	0	0	0
0	0	0	0	0	0	0	0	0	0	0	0	0	0	0	0	0	0	0
0	0	0	0	0	0	0	0	0	0	0	0	0	0	0	0	0	0	0
0	0	0	0	0	0	0	7	0	5	0	0	0	0	0	15	0	0	0
0	0	0	0	0	0	0	0	0	0	0	0	0	0	0	0	0	0	0
0	0	0	0	0	0	0	0	0	0	0	0	0	0	0	0	0	0	0
0	0	0	0	0	0	0	8	0	0	0	0	0	0	0	33	0	0	0
0	0	0	0	0	0	0	0	0	13	0	0	0	0	0	0	0	0	0
0	0	0	0	0	0	0	0	0	0	0	0	0	0	0	0	0	0	0
0	0	0	0	0	0	0	0	0	0	0	0	0	0	0	0	0	0	0
0	0	0	0	0	0	2	0	0	0	0	0	0	0	0	0	0	0	0
0	6	0	0	0	0	0	0	0	0	0	0	0	0	0	0	3	0	0
0	0	0	0	0	0	0	0	0	0	0	0	0	0	0	0	0	0	0
0	0	0	0	0	0	0	0	0	0	0	0	0	0	0	0	0	0	0
0	0	0	0	0	0	0	0	0	0	0	0	0	0	0	0	0	0	0
0	0	0	0	0	0	0	0	0	0	0	0	0	0	0	0	0	0	0
0	0	0	0	0	0	0	0	0	0	0	0	0	0	0	0	0	6	0

0	0	0	0	0	0	0	0	0	0	0	0	0	0	0	0	0	0	0
0	0	0	0	0	0	0	0	0	0	0	0	0	0	0	0	0	0	0
0	0	0	0	0	0	0	0	0	0	0	0	0	0	0	0	0	0	0
0	0	0	0	0	0	0	0	0	0	0	0	0	0	0	0	0	0	0
0	0	0	0	0	0	0	0	0	0	0	0	0	0	0	0	0	0	0
0	0	0	0	0	0	0	0	0	0	0	0	0	0	0	0	0	0	0
0	0	0	0	0	0	0	0	0	0	0	0	0	0	0	0	0	0	0
0	0	0	0	0	0	0	0	8	0	0	0	0	0	0	0	0	0	0
0	0	0	0	13	0	0	0	0	0	0	0	0	0	0	0	0	0	8
0	0	0	0	0	0	0	0	0	0	0	0	0	0	0	0	0	0	0
0	0	0	0	0	0	0	0	0	0	0	0	0	0	0	0	0	0	14
0	0	0	0	0	0	0	0	0	0	0	0	0	0	0	0	0	0	0
0	0	0	0	0	0	0	0	0	0	0	0	0	0	0	0	0	0	0
0	0	0	0	0	0	0	0	0	0	0	0	0	0	0	0	0	0	0
0	0	0	0	0	0	0	0	0	0	0	0	0	0	0	0	0	0	0
0	0	0	0	0	0	0	15	0	0	0	0	0	0	0	0	0	0	0
0	0	0	0	0	0	0	0	0	0	0	0	0	0	0	0	0	0	0
0	0	0	0	0	0	0	0	0	0	0	0	0	0	0	0	0	0	0
0	0	0	0	0	0	0	0	0	0	0	0	0	0	0	0	0	0	0
0	0	0	0	0	0	0	0	0	0	0	0	0	0	0	0	0	0	0
0	0	0	0	0	0	0	0	0	0	0	0	11	0	0	0	0	0	0
0	0	0	0	0	0	0	0	0	0	0	0	0	0	0	0	0	0	0
0	0	0	0	0	0	0	0	0	0	0	0	0	0	0	0	0	0	0
0	0	0	0	0	0	0	0	0	0	0	0	0	0	0	0	0	0	0
0	0	0	0	0	0	0	0	0	0	0	0	0	0	0	0	0	0	0
0	0	0	0	0	0	0	0	0	0	0	0	0	0	0	0	0	0	0
0	0	0	0	0	0	0	0	0	0	0	0	0	0	0	0	0	0	0
0	0	0	0	0	0	0	0	0	0	0	0	0	0	0	0	0	0	0
0	0	0	0	0	0	7	7	0	0	0	0	0	0	0	0	0	0	0
0	0	0	0	0	0	0	0	0	0	0	0	0	0	0	0	0	0	0
0	0	0	0	0	0	0	0	0	0	0	0	0	0	0	0	0	0	0
0	0	0	0	0	0	0	0	0	0	0	0	0	0	0	0	0	0	0
0	0	0	0	0	0	0	0	0	0	0	0	0	0	0	0	0	0	0
0	0	0	0	0	0	0	0	0	0	0	0	0	0	0	0	0	0	0
0	0	0	0	0	0	0	0	0	0	0	0	0	0	0	0	0	0	0
0	0	0	0	0	0	0	0	0	0	0	0	0	0	0	0	0	0	0
0	0	0	0	0	0	0	0	0	0	0	0	0	0	0	0	0	0	0

0	0	0	0	0	0	0	0	0	0	0	0	0	0	0	0	0	0	0
0	0	0	0	0	0	0	0	0	0	0	0	0	0	0	0	0	0	0
0	0	0	0	0	0	0	0	0	0	0	0	0	0	0	0	0	0	0
0	0	0	0	0	0	0	0	0	0	0	0	0	0	0	0	0	0	0
0	0	0	0	0	0	0	0	0	0	0	0	0	0	0	0	0	0	0
0	0	0	0	0	0	0	0	0	0	0	0	4	4	0	0	0	0	0
0	0	0	0	0	0	0	0	0	0	0	0	0	0	0	0	0	0	0
0	0	0	0	0	0	0	0	0	0	0	0	0	0	0	0	0	0	0
0	0	0	0	0	0	0	0	0	0	0	0	0	0	0	0	0	0	0
0	0	0	0	0	0	0	0	0	0	0	0	0	0	0	0	0	0	0
0	0	0	0	0	0	0	0	0	0	0	0	0	0	0	0	0	0	0
0	0	0	0	0	0	0	0	0	0	0	0	0	0	0	0	0	0	0
0	0	0	0	0	0	0	0	0	0	0	0	0	0	0	0	0	0	0
0	0	0	0	0	0	0	0	0	0	0	0	0	0	0	0	0	0	0
0	0	0	0	0	0	0	0	0	0	0	0	0	0	0	0	0	0	0
0	0	0	0	0	0	0	0	0	0	0	0	0	0	0	0	0	0	0
0	0	0	0	0	0	0	0	0	0	0	0	0	0	0	0	0	0	0
0	0	0	0	0	0	0	0	0	0	0	0	0	0	0	0	0	0	0
0	0	0	0	0	0	0	0	0	0	0	0	0	0	0	0	0	0	0
0	0	0	0	0	0	0	0	0	0	0	0	0	0	0	0	0	0	0
0	0	0	0	0	0	0	0	0	0	0	0	0	0	0	0	0	0	0
0	0	0	0	0	0	0	0	0	0	0	0	0	0	0	0	0	0	0
0	0	0	0	0	0	0	0	0	0	0	0	0	0	0	0	0	0	0
0	0	0	0	0	0	0	0	0	0	0	0	0	0	0	0	0	0	0
0	0	0	0	0	0	0	0	0	0	0	0	0	0	0	0	0	0	0
0	0	0	0	0	0	0	0	0	0	0	0	0	0	0	0	0	0	0
0	0	0	0	0	0	0	0	0	0	0	0	0	0	0	0	0	0	0
0	0	0	0	0	0	0	0	0	0	0	0	0	0	0	0	0	0	0
0	0	0	0	0	0	0	0	0	0	0	0	0	0	0	0	0	0	0
0	0	0	0	0	0	0	0	0	0	0	0	0	0	0	0	0	0	0
0	0	0	0	0	0	0	0	0	0	0	0	0	0	0	0	0	0	0
0	0	0	0	0	0	0	0	0	0	0	0	0	0	0	0	0	0	0
0	0	0	0	0	0	0	0	0	0	0	0	0	0	0	0	0	0	0
0	0	0	2	2	2	2	2	0	0	0	0	0	0	0	0	0	0	0
0	0	0	0	0	0	0	0	0	0	0	0	0	0	0	0	0	0	0
0	0	0	0	0	0	0	0	0	0	0	0	0	0	0	0	0	0	0
0	0	0	0	0	0	0	0	0	0	0	0	0	0	0	0	0	0	0

0	0	0	0	0	0	0	0	0	0	0	0	0	0	0	0	0	0	0
0	0	0	0	0	0	0	0	0	0	0	0	0	0	0	0	0	0	0
0	0	0	0	0	0	0	0	0	0	0	0	0	0	0	0	0	0	0
0	0	0	0	0	0	0	0	0	0	0	0	0	0	0	0	0	0	0
0	0	0	0	0	0	0	0	0	0	0	0	0	0	0	0	0	0	0
0	0	0	0	0	0	0	0	0	0	0	0	0	0	0	0	0	0	0
0	0	0	0	0	0	0	0	0	0	0	0	0	0	0	0	0	0	0
0	0	0	0	0	0	0	0	0	0	0	0	0	0	0	0	0	0	0
0	0	0	0	0	0	0	0	0	0	0	0	0	0	0	0	0	0	0
0	0	0	0	0	0	0	0	0	0	0	0	0	0	0	0	0	0	0
0	0	0	0	0	0	0	0	0	0	0	0	0	0	0	0	0	0	0
0	0	0	0	0	0	0	0	0	0	0	0	0	0	0	0	0	0	0
0	0	0	0	0	0	0	0	0	0	0	0	0	0	0	0	0	0	0
0	0	0	0	0	0	0	0	0	0	0	0	0	0	0	0	0	0	0
0	0	0	0	0	0	0	0	0	0	0	0	0	0	0	0	0	0	0
0	0	0	0	0	0	0	0	0	0	0	0	0	0	0	0	0	0	0
0	0	0	0	0	0	0	0	0	0	0	0	0	0	0	0	0	0	0
0	0	0	0	0	0	0	0	0	0	0	0	0	0	0	0	0	0	0
0	0	0	0	0	0	0	0	0	0	0	0	0	0	0	0	0	0	0
0	0	0	0	0	0	0	0	0	0	0	0	0	0	0	0	0	0	0
0	0	0	0	0	0	0	0	0	0	0	0	0	0	0	0	0	0	0
0	0	0	0	0	0	0	0	0	0	0	0	0	0	0	0	0	0	0
0	0	0	0	0	0	0	0	0	0	0	0	0	0	0	0	0	0	0
0	0	0	0	0	0	0	0	0	0	0	0	0	0	0	0	0	0	0
0	0	0	0	0	0	0	0	0	0	0	0	0	0	0	0	0	0	0
0	0	0	0	0	0	0	0	0	0	0	0	0	0	0	0	0	0	0
0	0	0	0	0	0	0	0	0	0	0	0	0	0	0	0	0	0	0
0	0	0	0	0	0	0	0	0	0	0	0	0	0	0	0	0	0	0
0	0	0	0	0	0	0	0	0	0	0	0	0	0	0	0	0	0	0
0	0	0	0	0	0	0	0	0	0	0	0	0	0	0	0	0	0	0
0	0	0	0	0	0	0	0	0	0	0	0	0	0	0	0	0	0	0
0	0	0	0	0	0	0	0	0	0	0	0	0	0	0	0	0	0	0
0	0	0	0	0	0	0	0	0	0	0	0	0	0	0	0	0	0	0
0	0	0	0	0	0	0	0	0	0	0	0	0	0	0	0	0	0	0
0	0	0	0	0	0	0	0	0	0	0	0	0	0	0	0	0	0	0
0	0	0	0	0	0	0	0	0	0	0	0	0	0	0	0	0	0	0
0	0	0	0	0	0	0	0	0	0	0	0	0	0	0	0	0	0	0

0	0	0	0	0	0	0	0	0	0	0	0	0	0	0	0	0	0	0
0	0	0	0	0	0	0	0	0	0	0	0	0	0	0	0	0	0	0
0	0	0	0	0	0	0	0	0	0	0	0	0	0	0	0	0	0	0
0	0	0	0	0	0	0	0	0	0	0	0	0	0	0	0	0	0	0
0	0	0	0	0	0	0	0	0	0	0	0	0	0	0	0	0	0	0
0	0	0	0	0	0	0	0	0	0	0	0	0	0	0	0	0	0	0
0	0	0	0	0	0	0	0	0	0	0	0	0	0	0	0	0	0	0
0	0	0	0	0	0	0	0	0	0	0	0	0	0	0	0	0	0	0
0	0	0	0	0	0	0	0	0	0	0	0	0	0	0	0	0	0	0
0	0	0	0	0	0	0	0	0	0	0	0	0	0	0	0	0	0	0
0	0	0	0	0	0	0	0	0	0	0	0	0	0	0	0	0	0	0
0	0	0	0	0	0	0	0	0	0	0	0	0	0	0	0	0	0	0
0	0	0	0	0	0	0	0	0	0	0	0	0	0	0	0	0	0	0
0	0	0	0	0	0	0	0	0	0	0	0	0	0	0	0	0	0	0
0	0	0	0	0	0	0	0	0	0	0	0	0	0	0	0	0	0	0
0	0	0	0	0	0	0	0	0	0	0	0	0	0	0	0	0	0	0
0	0	0	0	0	0	0	0	0	0	0	0	0	0	0	0	0	0	0
0	0	0	0	0	0	0	0	0	0	0	0	0	0	0	0	0	0	0
0	0	0	0	0	0	0	0	0	0	0	0	0	0	0	0	0	0	0
0	0	0	0	0	0	0	0	0	0	0	0	0	0	0	0	0	0	0
0	0	0	0	0	0	0	0	0	0	0	0	0	0	0	0	0	0	0
0	0	0	0	0	0	0	0	0	0	0	0	0	0	0	0	0	0	0
0	0	0	0	0	0	0	0	0	0	0	0	0	0	0	0	0	0	0
0	0	0	0	0	0	0	0	0	0	0	0	0	0	0	0	0	0	0
0	0	0	0	0	0	0	0	0	0	0	0	0	0	0	0	0	0	0
0	0	0	0	0	0	0	0	0	0	0	0	0	0	0	0	0	0	0
0	0	0	0	0	0	0	0	0	0	0	0	0	0	0	0	0	0	0
0	0	0	0	0	0	0	0	0	0	0	0	0	0	0	0	0	0	0
0	0	0	0	0	0	0	0	0	0	0	0	0	0	0	0	0	0	0
0	0	0	0	0	0	0	0	0	0	0	0	0	0	0	0	0	0	0
0	0	0	0	0	0	0	0	0	0									
Jan_DL078	1515	1049	309	398	48	413	105	111	254	170	203	138	134	198	202	179	182	150
110	0	154	0	166	144	101	80	109	82	185	272	81	35	76	90	46	0	169
0	25	85	57	48	66	42	0	137	88	0	81	0	28	44	85	0	0	138
112	12	62	7	0	0	86	0	40	0	77	90	0	77	0	0	32	0	25
48	72	23	104	105	17	22	17	0	31	43	55	17	18	87	62	0	49	0
8	16	0	0	0	18	0	0	0	18	98	0	54	0	13	0	35	0	0

0	0	0	0	0	0	0	0	0	0	20	0	0	21	0	0	81	0	0
0	33	0	15	15	0	0	9	14	3	0	0	17	0	0	22	0	0	0
0	16	0	0	0	0	0	0	0	0	0	0	15	0	0	0	21	0	10
16	0	22	0	0	0	0	10	0	18	0	9	0	0	0	0	4	0	24
0	0	0	0	0	0	0	0	0	10	0	0	0	0	0	0	0	0	0
0	0	12	0	13	18	0	0	0	0	0	4	10	0	0	0	23	0	0
0	0	0	0	0	45	0	0	0	0	0	0	9	0	0	0	0	0	0
0	0	0	0	0	0	0	0	0	0	31	0	0	22	0	10	0	0	0
9	0	0	0	0	0	0	0	0	0	0	0	0	10	3	0	11	0	0
0	10	0	0	0	0	0	0	5	32	0	0	0	0	7	0	0	0	0
0	0	0	0	0	0	0	0	0	0	0	0	0	0	0	3	0	0	0
13	0	0	0	0	31	8	0	0	0	0	0	0	0	0	10	0	0	0
0	0	0	0	0	0	0	0	0	0	0	0	0	0	0	0	0	0	0
9	0	3	0	0	0	3	0	0	0	0	0	0	0	12	0	0	0	0
0	0	0	0	0	0	0	0	0	0	0	0	0	0	0	0	0	0	0
0	0	0	0	0	0	0	0	0	0	0	6	0	0	0	0	0	0	0
0	0	0	0	0	0	0	0	0	0	0	0	0	0	0	0	0	0	0
0	0	0	0	0	0	0	0	0	0	0	0	0	0	0	0	15	0	0
0	0	0	0	8	0	0	0	0	0	0	0	0	0	0	0	0	0	0
0	0	0	0	0	0	0	0	0	0	0	0	0	0	0	0	0	0	3
0	0	0	0	0	0	0	0	0	0	0	0	0	0	0	0	0	0	0
0	0	0	0	0	0	0	0	0	0	0	0	0	0	0	0	0	6	0
0	0	0	0	0	0	0	0	0	0	0	0	0	0	0	0	0	0	0
0	0	0	0	0	0	0	37	0	0	0	0	0	0	0	0	0	0	0
0	0	0	0	0	0	0	0	0	0	0	0	0	0	0	0	0	0	0
0	0	0	0	0	0	0	0	0	0	0	0	0	2	0	0	0	0	0
0	0	0	0	0	0	0	0	0	0	0	0	0	0	0	0	0	0	0
0	0	0	14	0	0	0	15	0	0	0	0	0	0	0	0	0	0	0
0	0	0	0	0	0	0	0	0	0	0	0	0	0	0	0	0	0	0
0	0	0	0	0	0	0	0	34	0	0	0	0	0	0	0	0	0	0
0	0	0	0	0	0	0	0	0	0	0	0	0	0	0	0	0	0	0
0	0	0	0	0	0	0	0	0	0	0	0	0	0	0	0	0	0	0
0	0	0	0	0	0	0	0	0	0	0	0	0	0	0	0	0	0	0
0	0	0	0	0	0	0	0	0	0	0	0	0	0	0	0	0	0	0
0	0	14	0	0	0	0	0	0	0	0	0	0	0	0	0	0	0	0
0	0	0	0	0	0	0	0	0	0	0	0	0	0	0	0	0	0	0
0	0	0	0	0	0	0	0	0	0	0	0	0	0	0	0	0	0	0

0	0	0	0	0	0	0	0	0	0	0	0	0	0	0	0	0	0	0
0	0	0	0	0	0	0	0	0	0	0	0	0	0	0	0	0	0	0
0	0	0	0	0	0	0	0	0	0	3	0	0	0	0	0	0	0	0
0	0	0	0	0	0	0	0	0	0	0	0	0	0	0	0	0	0	0
0	0	0	0	0	0	0	0	0	0	0	0	0	0	0	0	0	0	0
0	0	0	0	0	0	0	0	0	0	0	0	0	0	0	0	0	0	0
0	0	0	0	0	0	0	0	0	0	0	0	0	0	0	0	0	0	0
0	0	0	0	0	0	0	0	0	0	0	0	0	0	0	0	0	0	0
0	0	0	0	0	0	0	0	0	0	0	0	0	0	0	0	0	0	0
0	0	0	0	0	0	0	0	0	0	0	0	0	0	0	0	0	0	0
0	0	0	0	0	0	0	0	0	0	0	0	0	0	0	0	0	0	0
0	0	0	0	0	0	0	0	0	0	0	0	0	0	0	0	0	0	0
0	0	0	0	0	0	0	0	0	0	0	0	0	0	0	0	0	0	0
0	0	0	0	0	0	0	0	0	0	0	0	0	0	0	0	0	0	0
0	0	0	0	0	0	0	0	0	0	0	0	0	0	0	0	0	0	0
0	0	0	0	0	0	0	0	0	0	0	0	0	0	0	0	0	0	0
0	0	0	0	0	0	0	0	0	0	0	0	0	0	0	0	0	0	0
0	0	0	0	0	0	0	0	0	0	0	0	0	0	0	0	0	0	0
0	0	0	0	0	0	8	0	0	0	0	0	0	0	0	0	0	0	0
0	0	0	0	0	0	0	0	0	0	0	0	0	0	0	0	0	0	0
0	0	0	0	0	0	0	0	0	0	0	0	0	0	0	0	0	0	0
0	0	0	0	0	0	0	0	0	0	0	0	0	0	0	0	0	0	0
0	0	0	0	0	0	0	0	0	0	0	0	0	0	0	0	0	0	0
0	0	0	0	0	0	0	0	0	0	0	0	0	0	0	0	0	0	0
0	0	0	0	0	0	0	0	0	0	0	0	0	0	0	0	0	0	0
0	0	0	0	0	0	0	0	0	0	0	0	0	0	0	0	0	0	0
0	0	0	0	0	0	0	0	0	0	0	0	0	0	0	0	0	0	0
0	0	0	0	0	0	0	0	0	0	0	0	0	0	0	0	0	0	0
0	0	0	0	0	0	0	0	0	0	0	0	0	0	0	0	0	0	0
0	0	0	0	0	0	0	0	0	0	0	0	0	0	0	0	0	0	0
0	0	0	0	0	0	0	0	0	0	0	0	0	0	0	0	0	0	0
0	0	0	0	0	0	0	0	0	0	0	0	0	0	0	0	0	0	0
0	0	0	0	0	0	0	0	0	0	0	0	0	0	0	0	0	0	0
0	0	0	0	0	0	0	0	0	0	0	0	0	0	0	0	0	0	0
0	0	0	0	0	0	0	0	0	0	0	0	0	0	0	0	0	0	0
0	0	0	0	0	0	0	0	0	0	0	0	0	0	0	0	0	0	0
0	0	0	0	0	0	0	0	0	0	0	0	0	0	0	0	0	0	0

0	0	0	0	0	0	0	0	0	0	0	0	0	0	0	0	0	0	0
0	0	0	0	0	0	0	0	0	0	0	0	0	0	0	0	0	0	0
0	0	0	0	0	0	0	0	0	0	0	0	0	0	0	0	0	0	0
0	0	0	0	0	0	0	0	0	0	0	0	0	0	0	0	0	0	0
0	0	0	0	0	0	0	0	0	0	0	0	0	0	0	0	0	0	0
0	0	0	0	0	0	0	0	0	0	0	0	0	0	0	0	0	0	0
0	0	0	0	0	0	0	0	0	0	0	0	0	0	0	0	0	0	0
0	0	0	0	0	0	0	0	0	0	0	0	0	0	0	0	0	0	0
0	0	0	0	0	0	0	0	0	0	0	0	0	0	0	0	0	0	0
0	0	0	0	0	0	0	0	0	0	0	0	0	0	0	0	0	0	0
0	0	0	0	0	0	0	0	0	0	0	0	0	0	0	0	0	0	0
0	0	0	0	0	0	0	0	0	0	0	0	0	0	0	0	0	0	0
0	0	0	0	0	0	0	0	0	0	0	0	0	0	0	0	0	0	0
0	0	0	0	0	0	0	0	0	0	0	0	0	0	0	0	0	0	0
0	0	0	0	0	0	0	0	0	0	0	0	0	0	0	0	0	0	0
0	0	0	0	0	0	0	0	0	0	0	0	0	0	0	0	0	0	0
0	0	0	0	0	0	0	0	0	0	0	0	0	0	0	0	0	0	0
0	0	0	0	0	0	0	0	0	0	0	0	0	0	0	0	0	0	0
0	0	0	0	0	0	0	0	0	0	0	0	0	0	0	0	0	0	0
0	0	0	0	0	0	0	0	0	0	0	0	0	0	0	0	0	0	0
0	0	0	0	0	0	0	0	0	0	0	0	0	0	0	0	0	0	0
0	0	0	0	0	0	0	0	0	0	0	0	0	0	0	0	0	0	0
0	0	0	0	0	0	0	0	0	0	0	0	0	0	0	0	0	0	0
0	0	0	0	0	0	0	0	0	0	0	0	0	0	0	0	0	0	0
0	0	0	0	0	0	0	0	0	0	0	0	0	0	0	0	0	0	0
0	0	0	0	0	0	0	0	0	0	0	0	0	0	0	0	0	0	0
0	0	0	0	0	0	0	0	0	0	0	0	0	0	0	0	0	0	0
0	0	0	0	0	0	0	0	0	0	0	0	0	0	0	0	0	0	0
0	0	0	0	0	0	0	0	0	0	0	0	0	0	0	0	0	0	0
0	0	0	0	0	0	0	0	0	0	0	0	0	0	0	0	0	0	0
0	0	0	0	0	0	0	0	0	0	3	3	0	0	0	0	0	0	0
0	0	0	0	0	0	0	0	0	0	0	0	0	0	0	0	0	0	0
0	0	0	0	0	0	0	0	0	0	0	0	0	0	0	0	0	0	0
0	0	0	0	0	0	0	0	0	0	0	0	0	0	0	0	0	0	0
0	0	0	0	0	0	0	0	0	0	0	0	0	0	0	0	0	0	0
0	0	0	0	0	0	0	0	0	0	0	0	0	0	0	0	0	0	0
0	0	0	0	0	0	0	0	0	0	0	0	0	0	0	0	0	0	0

0	0	0	0	0	0	0	0	0	0	0	0	0	0	0	0	0	0	0
0	0	0	0	0	0	0	0	0	0	0	0	0	0	0	0	0	0	0
0	0	0	0	0	0	0	0	0	0	0	0	0	0	0	0	0	0	0
0	0	0	0	0	0	0	0	0	0	0	0	0	0	0	0	0	0	0
0	0	0	0	0	0	0	0	0	0	0	0	0	0	0	0	0	0	0
0	0	0	0	0	0	0	0	0	0	0	0	0	0	0	0	0	0	0
0	0	0	0	0	0	0	0	0	0	0	0	0	0	0	0	0	0	0
0	0	0	0	0	0	0	0	0	0	0	0	0	0	0	0	0	0	0
0	0	0	0	0	0	0	0	0	0	0	0	0	0	0	0	0	0	0
0	0	0	0	0	0	0	0	0	0	0	0	0	0	0	0	0	0	0
0	0	0	0	0	0	0	0	0	0	0	0	0	0	0	0	0	0	0
0	0	0	0	0	0	0	0	0	0	0	0	0	0	0	0	0	0	0
0	0	0	0	0	0	0	0	0	0	0	0	0	0	0	0	0	0	0
0	0	0	0	0	0	0	0	0	0	0	0	0	0	0	0	0	0	0
0	0	0	0	0	0	0	0	0	0	0	0	0	0	0	0	0	0	0
0	0	0	0	0	0	0	0	0	0	0	0	2	2	2	2	0	0	0
0	0	0	0	0	0	0	0	0	0	0	0	0	0	0	0	0	0	0
0	0	0	0	0	0	0	0	0	0	0	0	0	0	0	0	0	0	0
0	0	0	0	0	0	0	0	0	0	0	0	0	0	0	0	0	0	0
0	0	0	0	0	0	0	0	0	0	0	0	0	0	0	0	0	0	0
0	0	0	0	0	0	0	0	0	0	0	0	0	0	0	0	0	0	0
0	0	0	0	0	0	0	0	0	0	0	0	0	0	0	0	0	0	0
0	0	0	0	0	0	0	0	0	0	0	0	0	0	0	0	0	0	0
0	0	0	0	0	0	0	0	0	0	0	0	0	0	0	0	0	0	0
0	0	0	0	0	0	0	0	0	0	0	0	0	0	0	0	0	0	0
0	0	0	0	0	0	0	0	0	0	0	0	0	0	0	0	0	0	0
0	0	0	0	0	0	0	0	0	0	0	0	0	0	0	0	0	0	0
0	0	0	0	0	0	0	0	0	0	0	0	0	0	0	0	0	0	0
0	0	0	0	0	0	0	0	0	0	0	0	0	0	0	0	0	0	0
0	0	0	0	0	0	0	0	0	0	0	0	0	0	0	0	0	0	0
0	0	0	0	0	0	0	0	0	0	0	0	0	0	0	0	0	0	0
0	0	0	0	0	0	0	0	0	0	0	0	0	0	0	0	0	0	0
0	0	0	0	0	0	0	0	0	0	0	0	0	0	0	0	0	0	0
0	0	0	0	0	0	0	0	0	0	0	0	0	0	0	0	0	0	0
0	0	0	0	0	0	0	0	0	0	0	0	0	0	0	0	0	0	0
0	0	0	0	0	0	0	0	0	0	0	0	0	0	0	0	0	0	0
0	0	0	0	0	0	0	0	0	0	0	0	0	0	0	0	0	0	0

0	0	0	0	0	0	0	0	0	0	0	0	0	0	0	0	0	0	0
0	0	0	0	0	0	0	0	0	0	0	0	0	0	0	0	0	0	0
0	0	0	0	0	0	0	0	0	0	0	0	0	0	0	0	0	0	0
0	0	0	0	0	0	0	0	0	0	0	0	0	0	0	0	0	0	0
0	0	0	0	0	0	0	0	0	0	0	0	0	0	0	0	0	0	0
0	0	0	0	0	0	0	0	0	0	0	0	0	0	0	0	0	0	0
0	0	0	0	0	0	0	0	0	0	0	0	0	0	0	0	0	0	0
0	0	0	0	0	0	0	0	0	0	0	0	0	0	0	0	0	0	0
0	0	0	0	0	0	0	0	0	0	0	0	0	0	0	0	0	0	0
0	0	0	0	0	0	0	0	0	0	0	0	0	0	0	0	0	0	0
0	0	0	0	0	0	0	0	0	0	0	0	0	0	0	0	0	0	0
0	0	0	0	0	0	0	0	0	0	0	0	0	0	0	0	0	0	0
0	0	0	0	0	0	0	0	0	0	0	0	0	0	0	0	0	0	0
0	0	0	0	0	0	0	0	0	0	0	0	0	0	0	0	0	0	0
0	0	0	0	0	0	0	0	0	0	0	0	0	0	0	0	0	0	0
0	0	0	0	0	0	0	0	0	0	0	0	0	0	0	0	0	0	0
0	0	0	0	0	0	0	0	0	0	0	0	0	0	0	0	0	0	0
0	0	0	0	0	0	0	0	0	0	0	0	0	0	0	0	0	0	0
0	0	0	0	0	0	0	0	0	0	0	0	0	0	0	0	0	0	0
0	0	0	0	0	0	0	0	0	0	0	0	0	0	0	0	0	0	0
0	0	0	0	0	0	0	0	0	0	0	0	0	0	0	0	0	0	0
0	0	0	0	0	0	0	0	0	0	0	0	0	0	0	0	0	0	0
0	0	0	0	0	0	0	0	0	0									
Jan_DL079	1042	1397	64	580	53	1251	254	212	80	64	68	92	40	162	258	0	180	188
139	393	215	0	151	181	86	296	61	0	195	0	69	62	29	0	140	0	0
46	81	148	449	81	0	104	0	116	0	70	0	131	0	19	0	0	58	0
0	53	0	8	77	23	81	58	32	50	43	279	47	74	22	45	19	12	14
0	0	0	21	0	0	24	0	72	12	0	64	20	97	46	0	57	13	0
0	30	0	64	0	0	0	0	0	27	0	0	0	64	15	18	0	0	41
0	0	0	26	0	0	0	0	0	112	13	14	0	0	0	0	0	0	0
112	0	0	0	0	0	0	53	0	10	26	0	0	41	16	0	0	0	0
0	0	16	0	111	5	0	0	0	0	0	0	0	0	37	0	10	0	0
0	0	11	0	30	0	29	0	0	0	0	0	0	0	0	14	0	45	0
0	0	0	46	0	0	0	0	0	11	0	0	0	0	0	0	0	0	0
0	0	14	0	0	24	0	0	0	0	0	10	21	0	0	0	0	4	0
0	0	0	0	0	0	0	0	0	6	0	0	5	0	0	0	16	0	0
0	0	0	0	0	0	0	0	0	0	0	0	0	5	0	0	0	8	0

0	0	0	5	0	0	0	0	0	0	0	8	0	6	0	3	0	0	0
0	0	12	42	0	0	0	0	0	0	0	19	0	0	15	0	0	0	60
0	0	0	0	0	0	0	0	0	13	0	0	0	0	0	0	0	0	8
0	0	0	0	0	0	0	0	0	0	0	0	0	0	0	0	0	0	0
0	0	0	0	0	0	0	0	0	0	0	0	0	0	0	0	0	0	0
0	0	0	0	0	0	0	0	0	0	0	0	0	0	0	0	0	0	0
0	0	0	0	0	0	0	0	0	0	0	0	0	0	0	0	0	0	0
0	0	0	0	0	0	7	4	0	0	0	0	0	4	0	0	16	0	0
0	0	0	17	0	0	0	0	0	0	0	0	0	0	0	0	0	0	0
0	0	0	0	0	0	0	7	0	0	0	0	0	0	0	0	0	0	0
0	0	0	0	0	0	0	0	0	0	0	0	0	0	0	0	0	0	41
0	0	0	4	0	0	0	8	0	0	0	0	0	0	0	0	0	0	0
0	0	0	0	0	0	0	0	0	0	0	0	0	0	0	0	0	4	0
0	25	0	0	0	0	0	0	0	0	0	0	0	0	0	0	19	0	0
0	0	0	0	0	0	0	0	0	0	0	0	0	0	0	0	0	0	0
0	0	0	0	0	0	0	0	32	0	0	0	0	0	0	0	0	0	0
0	0	0	0	0	0	0	0	0	0	0	0	0	0	0	0	0	0	0
0	0	0	0	0	0	0	0	22	0	0	0	0	0	0	0	0	0	0
0	0	0	0	0	0	0	0	0	0	0	0	0	0	0	0	0	0	0
0	0	0	0	0	0	0	0	0	0	0	0	0	0	0	0	3	0	0
0	0	0	0	0	0	0	0	0	0	0	0	5	0	0	0	0	0	0
0	0	0	0	0	27	0	0	0	0	2	0	0	0	0	0	0	0	0
0	0	0	0	0	0	0	0	0	0	0	0	2	0	0	0	0	0	0
0	0	0	0	0	0	0	0	0	0	0	0	0	0	0	0	0	0	0
0	0	0	0	0	0	0	0	0	0	0	0	0	0	0	0	0	0	0
0	7	0	0	0	0	0	0	0	0	0	0	0	0	0	0	0	0	0
0	0	0	0	0	0	0	0	0	0	7	0	0	0	0	0	0	3	0
0	0	0	0	0	0	0	0	0	0	0	0	0	0	0	0	0	0	0
0	0	0	0	0	0	0	0	0	0	0	0	0	0	0	0	0	0	0
0	0	0	0	0	0	0	0	0	0	0	0	0	0	0	0	0	0	0
0	0	0	0	0	0	0	0	0	0	0	0	0	0	0	0	0	0	0
0	0	0	0	0	0	0	0	0	0	2	0	0	0	0	0	0	0	0
0	0	3	0	7	0	0	0	0	0	0	0	0	0	0	0	0	0	0
0	0	0	0	0	0	0	0	0	0	0	0	0	0	0	0	0	0	0
0	0	0	0	0	0	0	0	0	0	0	0	0	0	0	0	0	0	0
0	0	0	0	0	0	0	0	0	0	0	0	0	0	0	0	0	0	0
0	0	0	0	0	0	0	0	0	0	0	0	0	0	0	0	0	0	0

0	0	0	0	0	0	0	0	0	0	0	0	0	0	0	0	0	0	0
0	0	0	0	0	0	0	0	0	0	0	0	0	0	0	0	0	0	0
0	0	0	0	0	0	0	0	0	0	0	0	0	0	0	0	0	0	0
0	0	0	0	0	0	0	0	0	0	0	0	0	0	0	0	0	0	0
0	0	0	0	0	0	0	0	0	0	0	0	0	0	0	6	0	0	0
0	0	0	0	0	0	0	0	0	0	0	0	0	0	0	0	0	0	0
0	0	0	0	0	0	0	0	0	0	0	0	0	0	0	0	0	0	0
0	0	0	0	0	0	0	0	0	0	0	0	0	0	0	0	0	0	0
0	0	0	0	0	0	0	0	0	0	0	0	0	0	0	0	0	0	0
0	0	0	0	0	0	0	0	0	0	0	0	0	0	0	0	0	0	0
0	0	0	0	0	0	0	0	0	0	0	0	0	0	0	0	0	0	0
0	0	0	0	0	0	0	0	0	0	0	0	0	0	0	0	0	0	0
0	0	0	0	0	0	0	0	0	0	0	0	0	0	0	0	0	0	0
0	0	0	0	0	5	0	0	0	0	0	0	0	0	0	12	0	0	0
0	0	0	0	0	0	0	0	0	0	0	0	0	0	0	0	0	0	0
0	0	0	0	0	0	0	0	0	0	0	0	0	0	0	0	0	0	0
0	0	0	0	0	0	0	0	0	0	0	0	0	0	0	0	0	0	0
0	0	0	0	0	0	0	0	0	0	0	0	2	0	0	0	0	0	0
0	0	0	0	0	0	0	0	0	0	0	0	0	0	0	0	0	0	0
0	0	0	0	0	0	0	0	0	0	0	0	0	0	0	0	0	0	0
0	0	0	0	0	0	0	0	0	0	0	0	0	0	0	0	0	0	0
0	0	0	0	0	0	0	0	0	0	0	0	0	0	0	0	0	0	0
0	0	0	0	0	0	0	0	0	0	0	0	0	0	0	0	0	0	0
0	0	0	0	0	0	0	0	0	0	0	0	0	0	0	0	0	0	0
0	0	0	0	0	8	0	0	0	0	0	0	0	0	0	0	0	0	0
0	0	0	0	0	0	0	0	0	0	0	0	0	0	0	0	0	0	0
0	0	0	0	0	0	0	0	0	0	0	0	0	0	0	0	0	0	0
0	0	0	0	0	0	0	0	0	0	0	0	0	0	0	0	0	0	0
0	0	0	0	0	0	0	0	0	0	0	0	0	0	0	0	0	0	0
0	0	0	0	0	0	0	0	0	0	0	0	0	0	0	0	0	0	0
0	0	0	0	0	0	0	0	0	0	0	0	0	0	0	0	0	0	0
0	0	0	0	0	0	0	0	0	0	0	0	0	0	0	0	0	0	0
0	0	0	0	0	0	0	0	0	0	0	0	0	0	0	0	0	0	0
0	0	0	0	0	0	0	0	0	0	0	0	0	0	0	0	0	0	0
0	0	0	0	0	0	0	0	0	0	0	0	0	0	0	0	0	0	0
0	0	0	0	0	0	0	0	0	0	0	0	0	0	0	0	0	0	0
0	0	0	0	0	0	0	0	0	0	0	0	0	0	0	0	0	0	0

0	0	0	0	0	0	0	0	0	0	0	0	0	0	0	0	0	0	0
0	0	0	0	0	0	0	0	0	0	0	0	0	0	0	0	0	0	0
0	0	0	0	0	0	0	0	0	0	0	0	0	0	0	0	0	0	0
0	0	0	0	0	0	0	0	0	0	0	0	0	0	0	0	0	0	0
0	0	5	0	0	0	0	0	0	0	0	0	0	0	0	0	0	0	0
0	0	0	0	0	0	0	0	0	0	0	0	0	0	0	0	0	0	0
0	0	0	0	0	0	0	0	0	0	0	0	0	0	0	0	0	0	0
0	0	0	0	0	0	0	0	0	0	0	0	0	0	0	0	0	0	0
0	0	0	0	0	0	0	0	0	0	0	0	0	0	0	0	0	0	0
0	0	0	0	0	0	0	0	0	0	0	0	0	0	0	0	0	0	0
0	0	0	0	0	0	0	0	0	0	0	0	0	0	0	0	0	0	0
0	0	0	0	0	0	0	0	0	0	0	0	0	0	0	0	0	0	0
0	0	0	0	0	0	0	0	0	0	0	0	0	0	0	0	0	0	0
0	0	0	0	0	0	0	0	0	0	0	0	0	0	0	0	0	0	0
0	0	0	0	0	0	0	0	4	4	4	0	0	0	0	0	0	0	0
0	0	0	0	0	0	0	0	0	0	0	0	0	0	0	0	0	0	0
0	0	0	0	0	0	0	0	0	0	0	0	0	0	0	0	0	0	0
0	0	0	0	0	0	0	0	0	0	0	0	0	0	0	0	0	0	0
0	0	0	0	0	0	0	0	0	0	0	0	0	0	0	0	0	0	0
0	0	0	0	0	0	0	0	0	0	0	0	0	0	0	0	0	0	0
0	0	0	0	0	0	0	0	0	0	0	0	0	0	0	0	0	0	0
0	0	0	0	0	0	0	0	0	0	0	0	0	0	0	0	0	0	0
0	0	0	0	0	0	0	0	0	0	0	0	0	0	0	0	0	0	0
0	0	0	0	0	0	0	0	0	0	0	0	0	0	0	0	0	0	0
0	0	0	0	0	0	0	0	0	0	0	0	0	0	0	0	0	0	0
0	0	0	0	0	0	0	0	0	0	0	0	0	0	0	0	0	0	0
0	0	0	0	0	0	0	0	0	0	0	0	0	0	0	0	0	0	0
0	0	0	0	0	0	0	0	0	0	0	0	0	3	3	3	3	3	0
0	0	0	0	0	0	0	0	0	0	0	0	0	0	0	0	0	0	0
0	0	0	0	0	0	0	0	0	0	0	0	0	0	0	0	0	0	0
0	0	0	0	0	0	0	0	0	0	0	0	0	0	0	0	0	0	0
0	0	0	0	0	0	0	0	0	0	0	0	0	0	0	0	0	0	0
0	0	0	0	0	0	0	0	0	0	0	0	0	0	0	0	0	0	0
0	0	0	0	0	0	0	0	0	0	0	0	0	0	0	0	0	0	0
0	0	0	0	0	0	0	0	0	0	0	0	0	0	0	0	0	0	0
0	0	0	0	0	0	0	0	0	0	0	0	0	0	0	0	0	0	0
0	0	0	0	0	0	0	0	0	0	0	0	0	0	0	0	0	0	0

0	0	0	0	0	0	0	0	0	0	0	0	0	0	0	0	0	0	0
0	0	0	0	0	0	0	0	0	0	0	0	0	0	0	0	0	0	0
0	0	0	0	0	0	0	0	0	0	0	0	0	0	0	0	0	0	0
0	0	0	0	0	0	0	0	0	0	0	0	0	0	0	0	0	0	0
0	0	0	0	0	0	0	0	0	0	0	0	0	0	0	0	0	0	0
0	0	0	0	0	0	0	0	0	0	0	0	0	0	0	0	0	0	0
0	0	0	0	0	0	0	0	0	0	0	0	0	0	0	0	0	0	0
0	0	0	0	0	0	0	0	0	0	0	0	0	0	0	0	0	0	0
0	0	0	0	0	0	0	0	0	0	0	0	0	0	0	0	0	0	0
0	0	0	0	0	0	0	0	0	0	0	0	0	0	0	0	0	0	0
0	0	0	0	0	0	0	0	0	0	0	0	0	0	0	0	0	0	0
0	0	0	0	0	0	0	0	0	0	0	0	0	0	0	0	0	0	0
0	0	0	0	0	0	0	0	0	0	0	0	0	0	0	0	0	0	0
0	0	0	0	0	0	0	0	0	0	0	0	0	0	0	0	0	0	0
0	0	0	0	0	0	0	0	0	0	0	0	0	0	0	0	0	2	2
2	2	2	2	2	2	2	2	2	0	0	0	0	0	0	0	0	0	0
0	0	0	0	0	0	0	0	0	0	0	0	0	0	0	0	0	0	0
0	0	0	0	0	0	0	0	0	0	0	0	0	0	0	0	0	0	0
0	0	0	0	0	0	0	0	0	0	0	0	0	0	0	0	0	0	0
0	0	0	0	0	0	0	0	0	0	0	0	0	0	0	0	0	0	0
0	0	0	0	0	0	0	0	0	0	0	0	0	0	0	0	0	0	0
0	0	0	0	0	0	0	0	0	0	0	0	0	0	0	0	0	0	0
0	0	0	0	0	0	0	0	0	0	0	0	0	0	0	0	0	0	0
0	0	0	0	0	0	0	0	0	0	0	0	0	0	0	0	0	0	0
0	0	0	0	0	0	0	0	0	0	0	0	0	0	0	0	0	0	0
0	0	0	0	0	0	0	0	0	0	0	0	0	0	0	0	0	0	0
0	0	0	0	0	0	0	0	0	0	0	0	0	0	0	0	0	0	0
0	0	0	0	0	0	0	0	0	0	0	0	0	0	0	0	0	0	0
0	0	0	0	0	0	0	0	0	0	0	0	0	0	0	0	0	0	0
0	0	0	0	0	0	0	0	0	0	0	0	0	0	0	0	0	0	0
0	0	0	0	0	0	0	0	0	0	0	0	0	0	0	0	0	0	0
0	0	0	0	0	0	0	0	0	0	0	0	0	0	0	0	0	0	0
0	0	0	0	0	0	0	0	0	0	0	0	0	0	0	0	0	0	0
0	0	0	0	0	0	0	0	0	0	0	0	0	0	0	0	0	0	0
0	0	0	0	0	0	0	0	0	0	0	0	0	0	0	0	0	0	0
0	0	0	0	0	0	0	0	0	0	0	0	0	0	0	0	0	0	0
0	0	0	0	0	0	0	0	0	0	0	0	0	0	0	0	0	0	0

0	0	0	0	0	0	0	0	0	0	0	0	0	0	0	0	0	0	0
0	0	0	0	0	0	0	0	0	0	0	0	0	0	0	0	0	0	0
0	0	0	0	0	0	0	0	0	0	0	0	0	0	0	0	0	0	0
0	0	0	0	0	0	0	0	0	0	0	0	0	0	0	0	0	0	0
0	0	0	0	0	0	0	0	0	0	0	0	0	0	0	0	0	0	0
0	0	0	0	0	0	0	0	0	0	0	0	0	0	0	0	0	0	0
0	0	0	0	0	0	0	0	0	0	0	0	0	0	0	0	0	0	0
0	0	0	0	0	0	0	0	0	0	0	0	0	0	0	0	0	0	0
0	0	0	0	0	0	0	0	0	0	0	0	0	0	0	0	0	0	0
0	0	0	0	0	0	0	0	0	0	0	0	0	0	0	0	0	0	0
0	0	0	0	0	0	0	0	0	0	0	0	0	0	0	0	0	0	0
0	0	0	0	0	0	0	0	0	0	0	0	0	0	0	0	0	0	0
0	0	0	0	0	0	0	0	0	0	0	0	0	0	0	0	0	0	0
0	0	0	0	0	0	0	0	0	0	0	0	0	0	0	0	0	0	0
0	0	0	0	0	0	0	0	0	0									
Jan_DL081	617	1036	1051	381	192	22	35	28	55	262	767	71	238	21	308	388	17	57
109	101	68	0	117	77	171	115	261	143	0	0	106	88	94	379	49	128	0
155	41	0	0	71	284	59	131	0	176	0	126	19	41	13	86	47	0	0
0	125	95	62	0	16	0	52	135	17	23	0	12	0	0	0	26	32	6
54	72	0	29	0	33	9	0	89	0	0	108	16	0	16	0	15	0	0
0	18	0	0	74	44	44	0	24	10	0	0	0	0	22	0	0	200	23
0	0	0	0	30	15	0	0	0	0	0	0	0	0	36	0	0	0	0
0	0	0	7	0	33	0	0	0	0	0	0	7	0	32	37	0	0	0
39	0	23	4	0	0	0	62	0	58	0	0	0	0	0	71	0	0	17
23	0	0	0	0	0	57	0	0	0	0	26	0	0	0	0	10	0	0
0	0	12	57	0	0	0	0	0	0	0	0	0	0	3	0	0	0	0
0	0	0	0	0	0	10	0	0	0	0	0	0	0	0	0	0	0	0
0	0	16	0	0	0	0	131	0	12	0	0	10	0	0	0	0	41	0
0	0	0	0	0	0	0	0	0	0	0	0	0	0	0	0	0	0	0
0	0	0	0	0	3	7	0	9	0	0	0	0	0	0	4	0	0	0
0	0	0	0	0	0	0	0	0	0	33	0	0	0	0	0	0	0	0
0	0	0	0	0	0	0	0	0	0	0	0	0	8	0	0	0	0	3
0	0	0	0	0	0	0	0	0	0	0	0	0	0	0	0	10	0	0
0	0	0	0	9	0	0	0	0	0	0	0	8	0	19	0	0	0	0
0	0	0	0	0	0	3	0	0	0	0	0	0	0	0	0	0	0	0
11	0	0	0	0	3	0	0	0	0	0	0	0	0	17	0	0	0	0
0	0	0	0	31	0	0	0	0	6	0	0	0	0	0	0	0	0	0

0	0	0	0	0	32	0	0	0	0	0	0	0	0	0	0	0	0	0
0	0	0	0	0	0	0	0	0	0	0	0	0	0	0	54	0	0	0
0	0	0	0	0	0	0	0	0	0	0	24	0	18	0	0	0	5	0
0	0	0	0	0	0	0	0	0	0	0	0	0	0	0	0	0	0	0
0	0	0	0	0	0	0	0	0	4	0	0	0	0	0	0	0	4	0
0	0	0	0	0	0	0	0	0	0	0	44	0	0	0	0	0	0	0
0	0	0	0	0	0	0	0	0	0	0	0	0	0	3	0	0	0	0
0	3	0	0	0	0	0	0	0	0	0	0	0	0	0	0	0	4	0
0	3	0	0	0	0	0	0	0	0	0	0	0	0	0	46	0	0	0
0	0	5	0	0	0	0	0	0	0	0	0	0	0	0	0	0	0	0
0	0	0	0	0	0	0	0	0	0	0	0	0	0	0	0	0	0	0
0	0	0	0	0	0	0	0	0	0	10	0	0	0	0	0	0	0	0
0	0	0	0	0	0	0	0	0	0	0	0	0	0	0	0	0	0	0
0	0	0	0	0	0	0	0	0	0	0	0	0	0	0	0	0	0	0
0	0	0	0	12	0	0	0	0	0	0	0	0	0	0	0	0	0	0
0	0	0	0	0	0	0	0	0	0	0	0	0	0	0	0	0	0	0
0	0	0	0	0	0	0	0	0	0	0	0	0	4	0	0	0	0	0
0	0	0	0	0	0	0	0	0	0	0	0	0	0	0	0	0	0	0
0	0	0	0	0	0	0	0	0	0	0	0	0	0	0	0	0	0	0
0	0	0	0	0	0	0	0	0	0	0	0	0	0	0	0	0	0	0
0	0	0	0	0	0	0	0	0	0	0	0	0	0	0	0	0	0	0
0	0	0	0	0	0	0	0	0	0	0	0	16	0	0	0	0	0	0
0	0	0	0	0	0	0	0	0	0	0	0	0	0	0	0	7	0	0
0	0	0	0	0	0	0	0	0	0	2	0	0	0	0	0	0	0	0
0	0	0	0	0	0	0	0	0	0	0	0	0	0	0	0	0	0	0
0	0	0	0	0	0	0	0	0	0	0	0	0	0	0	0	0	0	0
0	0	0	0	0	0	0	0	0	0	0	0	0	0	0	0	0	0	0
0	0	0	0	0	0	0	0	0	0	0	0	0	0	0	0	0	0	0
0	0	0	0	0	0	0	0	0	0	0	0	0	0	0	0	0	0	0
0	0	0	0	0	0	0	2	0	0	10	0	0	0	0	0	0	0	0
0	0	0	0	0	0	0	0	0	0	0	0	0	0	0	0	0	0	0
0	0	0	0	0	0	0	0	0	0	0	0	0	0	0	0	0	0	0
0	0	0	0	0	0	0	0	0	0	0	0	0	0	0	0	0	0	0
0	0	0	0	0	0	0	0	13	0	0	0	0	0	0	0	0	0	0
0	0	19	0	0	0	0	0	0	0	0	0	0	0	0	0	0	0	0
0	0	0	0	0	0	0	0	0	0	0	0	0	0	0	0	0	0	0
0	0	0	0	0	0	0	0	0	0	0	0	0	0	0	0	0	0	0

0	0	0	0	0	0	0	0	0	0	0	0	0	0	0	0	0	0	0
0	0	0	0	0	0	0	0	0	0	0	0	0	0	0	0	0	0	0
0	0	0	0	0	0	0	0	0	0	0	0	0	0	0	0	0	0	0
0	0	0	0	0	0	0	0	0	0	0	0	0	0	0	0	0	0	0
0	0	0	0	0	0	0	0	0	0	0	0	0	0	0	0	0	0	0
0	0	0	0	0	0	0	0	0	0	0	0	0	0	0	0	0	0	0
0	0	0	0	0	0	0	0	0	0	0	0	0	0	0	0	0	0	0
0	0	0	0	0	0	0	0	0	0	0	0	0	0	0	0	0	0	0
0	0	0	0	0	0	0	0	0	0	0	0	0	0	0	0	0	0	0
0	0	0	0	0	0	0	0	0	0	0	0	0	0	0	0	0	0	0
0	0	0	0	0	0	0	0	0	0	0	0	0	0	0	0	0	0	0
0	0	0	0	0	0	0	0	0	0	0	2	0	0	0	0	0	0	0
0	0	0	0	0	0	0	0	0	0	0	0	0	0	0	0	0	0	0
0	0	0	0	0	0	0	0	0	0	0	0	0	0	0	0	0	0	0
0	0	0	0	0	0	0	0	0	0	0	0	0	0	0	0	0	0	0
0	0	0	0	0	0	0	0	0	0	0	0	0	0	0	0	0	0	0
0	0	0	0	0	0	0	0	0	0	0	0	0	0	0	0	0	0	0
0	0	0	0	0	0	0	0	0	0	0	0	0	0	0	0	0	0	0
0	0	0	0	0	0	0	0	0	0	0	0	0	0	0	0	0	0	0
0	0	0	0	0	0	0	0	0	0	0	0	0	0	0	0	0	0	0
0	0	0	0	0	0	0	0	0	0	0	0	0	0	0	0	0	0	0
0	0	0	0	0	0	0	0	0	0	0	0	0	0	0	0	0	0	0
0	0	0	0	0	0	0	0	0	0	0	0	0	0	0	0	0	0	0
0	0	0	0	0	0	0	0	0	0	0	0	0	0	6	0	0	0	0
0	0	0	0	0	0	0	0	0	0	0	0	0	0	0	0	0	0	0
0	0	0	0	0	0	0	0	0	0	0	0	0	0	0	0	0	0	0
0	0	0	0	0	0	0	0	0	0	0	0	0	0	0	0	0	0	0
0	0	0	0	0	0	0	0	0	0	0	0	0	0	0	0	0	0	0
0	0	0	0	0	0	0	0	0	0	0	0	0	0	0	0	0	0	0
0	0	0	0	0	0	0	0	0	0	0	0	0	0	0	0	0	0	0
0	0	0	0	0	0	0	0	0	0	0	0	0	0	0	0	0	0	0
0	0	0	0	0	5	0	0	0	0	0	0	0	0	0	0	0	0	0
0	0	0	0	0	0	0	0	0	0	0	0	0	0	0	0	0	0	0
0	0	0	0	0	0	0	0	0	0	0	0	0	0	0	0	0	0	0
0	0	0	0	0	0	0	0	0	0	0	0	0	0	0	0	0	0	0
0	0	0	0	0	0	0	0	0	0	0	0	0	0	0	0	0	0	0
0	0	0	0	0	0	0	0	0	0	0	0	0	0	0	0	0	0	0

0	0	0	0	0	0	0	0	0	0	0	0	0	0	0	0	0	0	0
0	0	0	0	0	0	0	0	0	0	0	0	0	0	0	0	0	0	0
0	0	0	0	0	0	0	0	0	0	0	0	0	0	0	0	0	0	0
0	0	4	4	4	0	0	0	0	0	0	0	0	0	0	0	0	0	0
0	0	0	0	0	0	0	0	0	0	0	0	0	0	0	0	0	0	0
0	0	0	0	0	0	0	0	0	0	0	0	0	0	0	0	0	0	0
0	0	0	0	0	0	0	0	0	0	0	0	0	0	0	0	0	0	0
0	0	0	0	0	0	0	0	0	0	0	0	0	0	0	0	0	0	0
0	0	0	0	0	0	0	0	0	0	0	0	0	0	0	0	0	0	0
0	0	0	0	0	0	0	0	0	0	0	0	0	0	0	0	0	0	0
0	0	0	0	0	0	0	0	0	0	0	0	0	0	0	0	0	0	0
0	0	0	0	0	0	0	0	0	0	0	0	0	0	0	0	0	0	0
0	0	0	0	0	0	0	0	0	0	0	0	0	0	0	0	0	0	0
0	0	0	0	0	0	0	0	0	0	0	0	0	0	0	0	0	0	0
0	0	0	0	0	0	0	0	0	0	0	0	0	0	0	0	0	0	0
0	0	0	0	0	0	0	0	0	0	0	0	0	0	0	0	0	0	0
0	0	0	0	0	0	0	3	3	3	3	3	0	0	0	0	0	0	0
0	0	0	0	0	0	0	0	0	0	0	0	0	0	0	0	0	0	0
0	0	0	0	0	0	0	0	0	0	0	0	0	0	0	0	0	0	0
0	0	0	0	0	0	0	0	0	0	0	0	0	0	0	0	0	0	0
0	0	0	0	0	0	0	0	0	0	0	0	0	0	0	0	0	0	0
0	0	0	0	0	0	0	0	0	0	0	0	0	0	0	0	0	0	0
0	0	0	0	0	0	0	0	0	0	0	0	0	0	0	0	0	0	0
0	0	0	0	0	0	0	0	0	0	0	0	0	0	0	0	0	0	0
0	0	0	0	0	0	0	0	0	0	0	0	0	0	0	0	0	0	0
0	0	0	0	0	0	0	0	0	0	0	0	0	0	0	0	0	0	0
0	0	0	0	0	0	0	0	0	0	0	0	0	0	0	0	0	0	0
0	0	0	0	0	0	0	0	0	0	0	0	0	0	0	0	0	0	0
0	0	0	0	0	0	0	0	0	0	0	0	0	0	0	0	0	0	0
0	0	0	0	0	0	0	0	0	0	0	0	0	0	0	0	0	0	0
0	0	0	0	0	0	0	0	0	0	0	0	0	0	0	0	0	0	0
0	0	0	0	0	0	0	0	0	0	0	0	0	0	0	0	0	0	0
0	0	0	0	0	0	0	0	0	0	0	0	0	0	0	0	0	0	0
0	0	0	0	0	0	0	0	0	0	0	0	0	0	0	0	0	0	0
0	0	0	0	0	0	0	0	0	0	0	0	0	0	0	0	0	0	0
0	0	0	0	0	0	0	0	0	0	0	0	0	0	0	0	0	0	0
0	0	0	0	0	0	0	0	0	0	0	0	0	0	0	0	0	0	0

0	0	0	0	0	0	0	0	0	0	0	0	0	0	0	0	0	0	0
0	0	0	0	0	0	0	0	0	0	0	2	2	2	2	2	2	2	2
0	0	0	0	0	0	0	0	0	0	0	0	0	0	0	0	0	0	0
0	0	0	0	0	0	0	0	0	0	0	0	0	0	0	0	0	0	0
0	0	0	0	0	0	0	0	0	0	0	0	0	0	0	0	0	0	0
0	0	0	0	0	0	0	0	0	0	0	0	0	0	0	0	0	0	0
0	0	0	0	0	0	0	0	0	0	0	0	0	0	0	0	0	0	0
0	0	0	0	0	0	0	0	0	0	0	0	0	0	0	0	0	0	0
0	0	0	0	0	0	0	0	0	0	0	0	0	0	0	0	0	0	0
0	0	0	0	0	0	0	0	0	0	0	0	0	0	0	0	0	0	0
0	0	0	0	0	0	0	0	0	0	0	0	0	0	0	0	0	0	0
0	0	0	0	0	0	0	0	0	0	0	0	0	0	0	0	0	0	0
0	0	0	0	0	0	0	0	0	0	0	0	0	0	0	0	0	0	0
0	0	0	0	0	0	0	0	0	0	0	0	0	0	0	0	0	0	0
0	0	0	0	0	0	0	0	0	0	0	0	0	0	0	0	0	0	0
0	0	0	0	0	0	0	0	0	0	0	0	0	0	0	0	0	0	0
0	0	0	0	0	0	0	0	0	0	0	0	0	0	0	0	0	0	0
0	0	0	0	0	0	0	0	0	0	0	0	0	0	0	0	0	0	0
0	0	0	0	0	0	0	0	0	0	0	0	0	0	0	0	0	0	0
0	0	0	0	0	0	0	0	0	0	0	0	0	0	0	0	0	0	0
0	0	0	0	0	0	0	0	0	0	0	0	0	0	0	0	0	0	0
0	0	0	0	0	0	0	0	0	0	0	0	0	0	0	0	0	0	0
0	0	0	0	0	0	0	0	0	0	0	0	0	0	0	0	0	0	0
0	0	0	0	0	0	0	0	0	0	0	0	0	0	0	0	0	0	0
0	0	0	0	0	0	0	0	0	0	0	0	0	0	0	0	0	0	0
0	0	0	0	0	0	0	0	0	0	0	0	0	0	0	0	0	0	0
0	0	0	0	0	0	0	0	0	0	0	0	0	0	0	0	0	0	0
0	0	0	0	0	0	0	0	0	0	0	0	0	0	0	0	0	0	0
0	0	0	0	0	0	0	0	0	0	0	0	0	0	0	0	0	0	0
0	0	0	0	0	0	0	0	0	0	0	0	0	0	0	0	0	0	0
0	0	0	0	0	0	0	0	0	0	0	0	0	0	0	0	0	0	0
0	0	0	0	0	0	0	0	0	0	0	0	0	0	0	0	0	0	0
0	0	0	0	0	0	0	0	0	0	0	0	0	0	0	0	0	0	0
0	0	0	0	0	0	0	0	0	0	0	0	0	0	0	0	0	0	0
0	0	0	0	0	0	0	0	0	0	0	0	0	0	0	0	0	0	0
0	0	0	0	0	0	0	0	0	0	0	0	0	0	0	0	0	0	0
0	0	0	0	0	0	0	0	0	0	0	0	0	0	0	0	0	0	0

0	0	0	0	0	0	0	0	0	0	0	0	0	0	0	0	0	0	0
0	0	0	0	0	0	0	0	0	0	0	0	0	0	0	0	0	0	0
0	0	0	0	0	0	0	0	0	0	0	0	0	0	0	0	0	0	0
0	0	0	0	0	0	0	0	0	0	0	0	0	0	0	0	0	0	0
0	0	0	0	0	0	0	0	0	0	0	0	0	0	0	0	0	0	0
0	0	0	0	0	0	0	0	0	0	0	0	0	0	0	0	0	0	0
0	0	0	0	0	0	0	0	0	0									
Jan_DL082	1430	1086	570	391	178	248	178	184	56	255	261	78	234	142	78	187	142	186
75	45	85	70	137	61	163	150	183	148	135	133	160	58	141	118	74	185	114
122	25	100	0	74	108	0	22	74	64	51	122	29	52	25	117	87	49	66
77	151	103	6	29	33	25	53	39	25	58	52	0	0	40	45	31	59	0
42	112	0	35	65	36	17	0	0	0	0	0	26	104	0	0	9	0	0
12	22	0	0	69	0	28	0	27	32	0	0	0	0	0	0	0	40	0
0	0	16	8	0	0	0	0	0	0	4	0	0	10	0	0	0	0	0
0	0	0	0	0	0	56	0	0	8	0	0	5	0	0	19	0	86	0
0	0	15	0	0	4	0	0	0	0	8	0	0	0	0	8	0	0	19
20	0	0	0	12	0	0	6	0	0	0	0	0	0	0	5	0	19	0
0	0	4	0	0	0	0	0	27	0	13	47	0	0	0	0	0	0	79
0	0	0	0	0	0	0	0	0	0	0	0	0	31	0	30	0	0	0
0	0	0	0	0	0	0	0	0	0	0	0	0	2	7	0	0	0	24
0	0	0	0	0	0	0	0	0	0	0	0	0	0	0	6	0	0	0
0	0	0	0	0	0	0	0	0	0	0	0	0	0	0	0	0	0	0
0	0	0	16	0	0	0	0	3	0	0	0	0	0	0	0	0	0	0
0	2	0	0	0	0	0	0	0	0	0	0	0	0	0	0	0	0	3
0	63	0	0	0	0	0	0	7	0	0	0	0	5	0	0	10	0	6
0	0	0	0	5	0	0	0	0	0	0	8	0	0	0	0	0	0	0
0	0	0	0	0	23	0	0	0	0	0	0	0	0	0	16	0	0	0
0	0	0	0	3	0	0	0	0	0	0	0	0	0	0	0	25	0	0
0	0	0	0	0	11	2	2	0	0	0	0	0	0	0	0	0	0	0
0	0	0	0	0	0	0	0	0	0	0	0	0	0	0	0	0	0	0
0	0	0	0	0	0	0	0	0	0	0	0	0	0	0	0	0	6	14
0	0	0	0	0	0	0	0	0	0	0	0	0	0	0	0	0	0	0
0	0	0	0	0	0	0	0	0	0	0	0	0	0	0	9	0	0	0
0	0	0	0	0	0	0	0	10	4	0	0	0	0	0	0	0	0	0
0	0	0	0	0	0	0	0	0	0	0	0	0	0	0	0	0	0	0
0	0	0	0	0	0	0	0	0	0	0	0	0	0	0	0	0	0	0
0	0	0	0	0	0	0	0	0	0	2	0	10	0	0	0	0	0	0

0	0	0	0	0	0	0	0	0	0	0	0	0	0	0	0	0	0	0
0	0	0	0	0	0	0	0	0	0	0	0	0	0	0	0	0	0	0
0	0	0	0	0	0	0	0	0	0	0	0	0	0	0	0	0	0	0
0	0	0	0	0	0	0	0	0	0	0	0	0	0	0	0	0	0	0
0	0	0	0	0	0	0	0	0	0	0	0	0	0	0	0	0	0	0
0	0	0	0	0	0	0	0	0	0	0	0	0	0	0	0	0	0	0
0	0	0	0	0	0	0	0	0	0	0	0	0	0	0	0	0	0	0
0	0	0	0	0	0	0	0	0	0	0	0	0	0	0	0	0	0	0
0	0	0	0	0	0	0	0	0	0	0	0	0	0	0	0	0	0	0
0	0	0	0	0	0	0	0	0	0	0	0	0	0	0	0	0	0	0
0	0	0	0	0	0	0	0	0	0	0	0	0	0	3	0	0	0	0
0	0	0	0	0	0	0	0	0	0	0	0	11	0	0	0	0	0	0
0	0	0	0	0	0	0	0	0	0	0	0	0	0	0	0	0	0	0
0	0	0	0	0	0	0	0	0	0	0	0	0	0	0	0	0	0	0
0	0	0	0	0	0	0	0	0	0	0	0	0	0	0	0	0	0	0
0	0	0	0	0	0	0	7	0	0	3	0	0	17	0	0	0	0	0
0	0	0	0	0	0	0	0	0	0	0	0	0	0	0	0	0	0	0
0	0	0	0	0	0	0	0	0	0	0	0	0	0	0	0	0	0	0
0	0	0	0	0	0	0	0	0	0	0	0	0	0	0	0	19	0	0
0	0	3	0	0	0	0	0	0	0	0	0	0	0	0	0	0	0	0
0	0	0	0	0	0	0	0	0	0	0	0	0	0	0	0	0	0	0
0	0	0	0	0	0	0	0	0	0	0	0	0	0	0	0	0	0	0
0	0	0	0	0	0	0	0	0	0	0	0	0	0	0	0	0	0	0
0	0	0	0	0	0	0	0	0	0	0	0	0	0	0	0	0	0	0
0	0	0	0	0	0	0	0	0	0	0	0	0	0	0	0	0	0	0
0	0	0	0	0	0	0	0	0	0	0	0	0	0	0	0	0	0	0
0	0	0	0	0	0	0	0	0	0	0	0	0	0	0	0	0	0	0
0	0	0	0	0	0	0	0	0	0	0	0	0	0	0	0	0	0	0
0	0	0	0	0	0	0	0	0	2	0	0	0	0	0	0	0	0	0
0	0	0	0	0	0	0	0	0	0	0	0	0	0	0	0	0	0	0
0	0	0	0	0	0	0	0	0	0	0	0	0	0	0	0	0	0	0
0	0	0	0	0	0	0	0	0	0	0	0	0	0	0	0	0	0	0
0	0	0	0	0	0	0	0	0	0	13	0	0	0	0	0	0	0	0
0	0	0	0	0	0	0	0	0	0	0	0	0	0	0	0	0	0	0
0	0	0	0	0	0	0	0	0	0	0	0	0	0	0	0	0	0	0
0	0	0	0	0	0	0	0	0	0	0	0	0	0	0	0	0	0	0
0	0	0	0	0	0	0	0	0	0	0	0	0	0	0	0	0	0	0

0	0	0	0	0	0	0	0	0	0	0	0	0	0	0	0	0	0	0
0	0	0	0	0	10	0	0	0	0	0	0	0	0	0	0	0	0	0
0	0	0	0	0	0	0	0	0	0	0	0	0	0	0	0	0	0	0
0	0	0	0	0	0	0	0	0	0	0	0	0	0	0	0	0	0	0
0	0	0	0	0	0	0	0	0	0	0	0	0	0	0	0	0	0	0
0	0	0	0	0	0	0	0	0	0	0	0	0	0	0	0	0	0	0
0	0	0	0	0	0	0	0	0	0	0	0	0	0	0	8	0	0	0
0	0	0	0	0	0	0	0	0	0	0	0	0	0	0	0	0	0	0
0	0	0	0	0	0	0	0	0	0	0	0	0	0	0	0	0	0	0
0	0	0	0	0	0	0	0	0	0	0	0	0	0	0	0	0	0	0
0	0	0	0	0	0	0	0	0	0	0	0	0	0	0	0	0	0	0
0	0	0	0	0	0	0	0	0	0	0	0	0	0	0	0	0	0	0
0	0	0	0	0	0	0	0	0	0	0	0	0	0	0	0	0	0	0
0	0	0	0	0	0	0	0	0	0	0	0	0	0	0	0	0	0	0
0	0	0	0	0	0	0	0	0	0	0	0	0	0	0	0	0	0	0
0	0	0	0	0	0	0	0	0	0	0	0	0	0	0	0	0	0	0
0	0	0	0	0	0	0	0	0	0	0	0	0	0	0	0	0	0	0
0	0	0	0	0	0	0	0	0	0	0	0	0	0	0	0	0	0	0
0	0	0	0	0	0	0	0	0	0	0	0	0	0	0	0	0	0	0
0	0	0	0	0	0	0	0	0	0	0	0	0	0	0	0	0	0	0
0	0	0	0	0	0	0	0	0	0	0	0	0	0	0	0	0	0	0
0	0	0	0	0	0	0	0	0	0	0	0	0	0	0	0	0	0	0
0	0	0	0	0	0	0	0	0	0	0	0	0	0	0	0	0	0	0
0	0	0	0	0	0	0	0	0	0	0	0	0	0	0	0	0	0	0
0	0	0	0	0	0	0	0	0	0	0	0	0	0	0	0	0	0	0
0	0	0	0	0	0	0	0	0	0	0	0	0	0	0	0	0	0	0
0	0	0	0	0	0	0	0	0	0	0	0	0	0	0	0	0	0	0
0	0	0	0	0	0	0	0	0	0	0	0	0	0	0	0	0	0	0
0	0	0	0	0	0	0	0	0	0	0	0	0	0	0	0	0	0	0
0	0	0	0	0	0	0	0	0	0	0	0	0	0	0	0	0	0	0
0	0	0	0	0	0	0	0	0	0	0	0	0	0	0	0	0	0	0
0	0	0	0	0	0	0	0	0	0	0	0	0	0	0	0	0	0	0
0	0	0	0	0	0	0	0	4	4	4	4	0	0	0	0	0	0	0
0	0	0	0	0	0	0	0	0	0	0	0	0	0	0	0	0	0	0
0	0	0	0	0	0	0	0	0	0	0	0	0	0	0	0	0	0	0
0	0	0	0	0	0	0	0	0	0	0	0	0	0	0	0	0	0	0
0	0	0	0	0	0	0	0	0	0	0	0	0	0	0	0	0	0	0

0	0	0	0	0	0	0	0	0	0	0	0	0	0	0	0	0	0	0
0	0	0	0	0	0	0	0	0	0	0	0	0	0	0	0	0	0	0
0	0	0	0	0	0	0	0	0	2	0	0	0	0	0	0	0	0	0
0	0	0	0	0	0	0	0	0	0	0	0	0	0	0	0	0	0	0
0	0	0	0	0	0	0	0	0	0	0	0	0	0	0	0	0	0	0
0	0	0	0	0	0	0	0	0	0	0	0	0	0	0	0	0	0	0
0	0	0	0	0	0	0	0	0	0	0	0	0	0	0	0	0	0	0
0	0	0	0	0	0	0	0	0	0	0	0	0	0	0	0	0	0	0
0	0	0	0	0	0	0	0	0	0	0	0	0	0	0	3	0	0	0
0	0	0	0	0	0	0	0	0	0	0	0	0	0	0	0	0	0	0
0	0	0	0	0	0	0	0	0	0	0	0	0	0	0	0	0	0	0
0	0	0	0	0	0	0	0	0	0	0	0	0	0	0	0	0	0	0
0	0	0	0	0	0	0	0	0	0	0	0	0	0	0	0	0	0	0
0	0	0	0	0	0	0	0	0	0	0	0	0	0	0	0	0	0	0
0	0	0	0	0	0	0	0	0	0	0	0	0	0	0	0	0	0	0
0	0	0	0	0	0	0	0	0	0	0	0	0	0	0	0	0	0	0
0	0	0	0	0	0	0	0	0	0	0	0	0	0	0	0	0	0	0
0	0	0	0	0	0	0	0	0	0	0	0	0	0	0	0	0	0	0
0	0	0	0	0	0	0	0	0	0	0	0	0	0	0	0	0	0	0
0	0	0	0	0	0	0	0	0	0	0	0	0	0	0	0	0	0	0
0	0	0	0	0	0	0	0	0	0	0	0	0	0	0	0	0	0	0
0	0	0	0	0	0	0	0	0	0	0	0	0	0	0	0	0	0	0
0	0	0	0	0	0	0	0	0	0	0	0	0	0	0	0	0	0	0
0	0	0	0	0	0	0	0	0	0	0	0	0	0	0	0	0	0	0
0	0	0	0	0	0	0	0	0	0	0	0	0	0	0	0	0	0	0
0	0	0	0	0	0	0	0	0	0	0	0	0	0	0	0	0	0	0
0	0	0	0	0	0	0	0	0	0	0	0	0	0	0	0	0	0	0
0	0	0	0	0	0	0	0	0	0	0	0	0	0	0	0	0	0	0
0	0	0	0	0	0	0	0	0	0	0	0	0	0	0	0	0	0	0
0	0	0	0	0	0	0	0	0	0	0	0	0	0	0	0	0	0	0
0	0	0	0	0	0	0	0	0	0	0	0	0	0	0	0	0	0	0
2	2	2	2	2	2	2	0	0	0	0	0	0	0	0	0	0	0	0
0	0	0	0	0	0	0	0	0	0	0	0	0	0	0	0	0	0	0
0	0	0	0	0	0	0	0	0	0	0	0	0	0	0	0	0	0	0
0	0	0	0	0	0	0	0	0	0	0	0	0	0	0	0	0	0	0
0	0	0	0	0	0	0	0	0	0	0	0	0	0	0	0	0	0	0
0	0	0	0	0	0	0	0	0	0	0	0	0	0	0	0	0	0	0

0	0	0	0	0	0	0	0	0	0	0	0	0	0	0	0	0	0	0
0	0	0	0	0	0	0	0	0	0	0	0	0	0	0	0	0	0	0
0	0	0	0	0	0	0	0	0	0	0	0	0	0	0	0	0	0	0
0	0	0	0	0	0	0	0	0	0	0	0	0	0	0	0	0	0	0
0	0	0	0	0	0	0	0	0	0	0	0	0	0	0	0	0	0	0
0	0	0	0	0	0	0	0	0	0	0	0	0	0	0	0	0	0	0
0	0	0	0	0	0	0	0	0	0	0	0	0	0	0	0	0	0	0
0	0	0	0	0	0	0	0	0	0	0	0	0	0	0	0	0	0	0
0	0	0	0	0	0	0	0	0	0	0	0	0	0	0	0	0	0	0
0	0	0	0	0	0	0	0	0	0	0	0	0	0	0	0	0	0	0
0	0	0	0	0	0	0	0	0	0	0	0	0	0	0	0	0	0	0
0	0	0	0	0	0	0	0	0	0	0	0	0	0	0	0	0	0	0
0	0	0	0	0	0	0	0	0	0	0	0	0	0	0	0	0	0	0
0	0	0	0	0	0	0	0	0	0	0	0	0	0	0	0	0	0	0
0	0	0	0	0	0	0	0	0	0	0	0	0	0	0	0	0	0	0
0	0	0	0	0	0	0	0	0	0	0	0	0	0	0	0	0	0	0
0	0	0	0	0	0	0	0	0	0	0	0	0	0	0	0	0	0	0
0	0	0	0	0	0	0	0	0	0	0	0	0	0	0	0	0	0	0
0	0	0	0	0	0	0	0	0	0	0	0	0	0	0	0	0	0	0
0	0	0	0	0	0	0	0	0	0	0	0	0	0	0	0	0	0	0
0	0	0	0	0	0	0	0	0	0	0	0	0	0	0	0	0	0	0
0	0	0	0	0	0	0	0	0	0	0	0	0	0	0	0	0	0	0
0	0	0	0	0	0	0	0	0	0	0	0	0	0	0	0	0	0	0
0	0	0	0	0	0	0	0	0	0	0	0	0	0	0	0	0	0	0
0	0	0	0	0	0	0	0	0	0	0	0	0	0	0	0	0	0	0
0	0	0	0	0	0	0	0	0	0	0	0	0	0	0	0	0	0	0
0	0	0	0	0	0	0	0	0	0	0	0	0	0	0	0	0	0	0
0	0	0	0	0	0	0	0	0	0	0	0	0	0	0	0	0	0	0
0	0	0	0	0	0	0	0	0	0	0	0	0	0	0	0	0	0	0
0	0	0	0	0	0	0	0	0	0	0	0	0	0	0	0	0	0	0
0	0	0	0	0	0	0	0	0	0	0	0	0	0	0	0	0	0	0
0	0	0	0	0	0	0	0	0	0	0	0	0	0	0	0	0	0	0
0	0	0	0	0	0	0	0	1	0	0	0	0	0	0	0	0	0	0
0	0	0	0	0	0	0	0	0	0	0	0	0	0	0	0	0	0	0
0	0	0	0	0	0	0	0	0	0	0	0	0	0	0	0	0	0	0
0	0	0	0	0	0	0	0	0	0									

Jan_DL083	885	1041	194	381	1004	214	336	374	124	174	120	37	173	98	55	67	109	191
145	0	56	91	112	39	67	59	83	56	111	40	54	66	84	0	0	40	66
46	0	131	59	62	0	0	30	87	16	55	68	43	85	15	40	105	63	0
0	40	0	92	37	22	27	54	24	12	59	72	0	0	21	110	10	0	77
21	31	0	22	25	0	10	36	0	0	0	0	33	24	17	55	3	11	0
18	7	0	0	11	12	0	0	10	0	0	5	57	99	0	19	15	14	0
0	0	0	0	13	0	0	0	0	0	0	0	16	8	0	0	48	0	0
0	0	0	26	0	0	30	0	29	7	0	0	7	0	0	0	0	0	0
0	11	20	0	0	0	0	0	0	0	10	0	0	0	0	12	0	0	0
0	0	10	0	0	0	0	0	0	0	0	0	0	16	0	0	0	0	8
0	17	0	0	0	0	0	0	0	0	12	0	0	0	0	0	0	0	0
0	0	0	0	0	19	0	0	0	0	0	0	0	0	0	0	0	0	0
0	0	0	14	0	0	0	0	0	0	0	0	0	0	0	0	0	0	0
0	0	0	0	0	0	0	0	0	0	0	0	0	0	5	0	0	0	0
0	0	0	21	25	0	0	0	0	0	0	0	0	0	0	6	4	0	0
0	0	0	0	0	0	0	0	0	0	0	0	0	0	0	0	0	0	0
0	6	0	0	0	0	0	0	0	6	0	0	0	4	0	0	0	0	0
0	0	0	0	0	0	0	0	0	0	0	0	0	0	0	0	0	0	0
0	0	0	0	0	0	0	0	0	8	0	0	0	0	0	0	0	0	0
0	0	0	0	0	0	0	0	0	0	0	0	0	0	0	0	0	0	0
0	0	0	0	0	0	0	9	0	0	0	15	0	0	0	0	0	0	0
0	0	0	0	0	0	0	0	0	0	35	0	0	0	0	0	0	0	0
0	0	0	4	0	0	0	0	0	0	0	0	0	0	0	0	0	0	0
0	0	0	0	0	0	0	0	0	0	0	0	0	0	0	0	0	0	7
0	0	0	0	0	0	0	0	0	0	0	0	0	0	0	0	0	0	0
0	0	0	0	0	0	0	0	0	0	0	0	0	0	0	5	0	0	0
0	0	0	0	0	0	0	0	35	0	0	0	0	0	0	0	8	0	0
0	0	0	0	0	0	0	0	0	0	0	0	0	0	0	0	0	0	0
0	0	0	0	0	0	0	0	0	0	0	0	0	0	0	0	0	0	0
0	0	0	0	0	0	0	0	0	0	0	0	0	0	0	0	0	0	0
0	0	0	0	0	0	0	0	0	0	0	0	0	0	0	0	0	0	0
0	0	0	0	0	0	0	0	0	0	0	0	0	0	0	0	0	0	0
0	0	0	0	0	0	0	0	0	0	0	0	0	0	0	0	0	8	0
0	0	0	0	0	0	0	0	0	0	0	0	0	0	0	0	0	0	0
0	0	0	0	0	0	0	0	0	0	0	0	0	0	0	0	0	0	0
0	0	0	0	0	0	0	0	0	0	0	0	0	0	0	0	0	0	0
0	0	0	0	0	0	0	0	0	0	0	0	0	0	0	0	0	0	0

0	0	0	0	0	0	0	0	0	0	0	0	0	0	0	0	0	0	0
0	0	0	0	0	0	0	0	0	0	0	0	0	0	0	0	0	0	0
0	0	0	0	0	0	0	0	0	0	0	0	0	0	0	0	5	0	0
0	0	0	0	0	0	0	0	0	0	0	0	0	0	0	0	0	0	0
0	0	0	0	0	0	0	0	0	0	0	0	0	0	0	0	0	0	0
0	0	0	0	0	0	0	0	0	0	0	0	0	0	0	0	0	0	0
0	0	0	0	0	0	0	0	0	0	0	0	0	0	0	0	0	0	0
0	0	0	0	0	0	0	0	0	0	0	0	0	0	0	0	0	0	0
0	0	0	0	0	0	0	0	0	0	0	0	0	0	0	0	0	0	0
0	0	0	0	0	0	0	0	0	0	0	0	0	0	0	0	0	0	0
0	0	0	0	0	0	0	0	0	0	0	0	0	0	0	0	11	0	0
0	0	0	0	0	0	0	0	0	0	0	0	0	0	0	0	0	0	0
0	0	0	0	0	0	0	5	0	0	0	0	0	0	0	0	0	0	0
0	0	0	0	0	0	0	0	0	0	0	0	0	0	0	0	0	0	0
0	0	0	0	0	0	0	0	0	0	0	0	0	0	0	0	0	0	0
0	0	0	0	0	0	0	0	0	0	0	0	0	0	0	0	0	0	0
0	0	0	0	0	0	0	0	0	0	0	0	0	0	0	0	0	0	0
0	0	0	0	0	0	0	0	0	0	0	0	0	0	0	0	0	0	0
0	0	0	0	0	0	0	0	0	0	0	0	0	0	0	0	0	0	0
0	0	0	0	0	0	0	0	0	0	0	0	0	0	0	0	0	0	0
0	0	0	0	0	0	0	0	0	0	0	0	0	0	0	0	0	0	0
0	0	0	0	0	0	0	0	0	0	0	0	0	0	0	0	0	0	0
0	0	0	0	0	0	0	0	0	0	0	0	0	0	0	0	0	0	0
0	0	0	0	0	0	0	0	0	0	0	0	0	0	0	0	0	0	0
0	0	0	0	0	0	0	0	0	0	0	0	0	0	0	0	0	0	0
0	0	0	0	0	0	0	0	0	0	0	0	0	0	0	0	0	0	0
0	0	0	0	0	0	0	0	0	0	0	0	0	0	0	0	0	0	0
0	0	0	0	0	0	0	0	0	0	0	0	0	0	0	0	0	0	0
0	0	0	0	0	0	0	0	0	0	0	0	0	0	0	0	0	0	0
0	0	0	0	0	0	0	0	0	0	0	0	0	0	0	0	0	0	0
0	0	0	0	0	0	0	0	0	0	0	0	0	0	0	0	0	0	0
0	0	0	0	0	0	0	0	0	0	0	0	0	0	0	0	0	0	0
0	0	0	0	0	0	0	0	0	0	0	0	0	0	0	0	0	0	0
0	0	0	0	0	0	0	0	0	0	0	0	0	0	0	0	0	0	0
0	0	0	0	0	0	0	0	0	0	0	0	0	0	0	0	0	0	0
0	0	0	0	0	0	0	0	0	0	0	0	0	0	0	0	0	0	0
0	0	0	0	0	0	0	0	0	0	0	0	0	0	0	0	0	0	0

0	0	0	0	0	0	0	0	0	0	0	0	0	0	0	0	0	0	0
0	0	0	0	0	0	0	0	0	0	0	0	0	0	0	0	0	0	6
0	0	0	0	0	0	0	0	0	0	0	0	0	0	0	0	0	0	0
0	0	0	0	0	0	0	0	0	0	0	0	0	0	0	0	0	0	0
0	0	0	0	0	0	0	0	0	0	0	0	0	0	0	0	0	0	0
0	0	0	0	0	0	0	0	0	0	0	0	0	0	0	0	0	0	0
0	0	0	0	0	0	0	0	0	0	0	0	0	0	0	0	0	0	0
0	0	0	0	0	0	0	0	0	5	0	0	0	0	0	0	0	0	0
0	0	0	0	0	0	0	0	0	0	0	0	0	0	0	0	0	0	0
0	0	0	0	0	0	0	0	0	0	0	0	0	0	0	0	0	0	0
0	0	0	0	0	0	0	0	0	0	0	0	0	0	0	0	0	0	0
0	0	0	0	0	0	0	0	0	0	0	0	0	0	0	0	0	0	0
0	0	0	0	0	0	0	0	0	0	0	0	0	0	0	0	0	0	0
0	0	0	0	0	0	0	0	0	0	0	0	0	0	0	0	0	0	0
0	0	0	0	0	0	0	0	0	0	0	0	0	0	0	0	0	0	0
0	0	0	0	0	0	0	0	0	0	0	0	0	0	0	0	0	0	0
0	0	0	0	0	0	0	0	4	0	0	0	0	0	0	0	0	0	0
0	0	0	0	0	0	0	0	0	0	0	0	0	0	0	0	0	0	0
0	0	0	0	0	0	0	0	0	0	0	0	0	0	0	0	0	0	0
0	0	0	0	0	0	0	0	0	0	0	0	0	0	0	0	0	0	0
0	0	0	0	0	0	0	0	0	0	0	0	0	0	0	0	0	0	0
0	0	0	0	0	0	0	0	0	0	0	0	0	0	0	0	0	0	0
0	0	0	0	0	0	0	0	0	0	0	0	0	0	0	0	0	0	0
0	0	0	0	0	0	0	0	0	0	0	0	0	0	0	0	0	0	0
0	0	0	0	0	0	0	0	0	0	0	0	0	0	0	0	0	0	0
0	0	0	0	0	0	0	0	0	0	0	0	0	0	0	0	0	0	0
0	0	0	0	0	0	0	0	0	0	0	0	0	0	0	0	0	0	0
0	0	0	0	0	0	0	0	0	0	0	0	0	0	0	0	0	0	0
0	0	0	0	0	0	0	0	0	0	0	0	0	0	0	0	0	0	0
0	0	0	0	0	0	0	0	0	0	3	3	3	3	0	0	0	0	0
0	0	0	0	0	0	0	0	0	0	0	0	0	0	0	0	0	0	0
0	0	0	0	0	0	0	0	0	0	0	0	0	0	0	0	0	0	0
0	0	0	0	0	0	0	0	0	0	0	0	0	0	0	0	0	0	0
0	0	0	0	0	0	0	0	0	0	0	0	0	0	0	0	0	0	0
0	0	0	0	0	0	0	0	0	0	0	0	0	0	0	0	0	0	0
0	0	0	0	0	0	0	0	0	0	0	0	0	0	0	0	0	0	0
0	0	0	0	0	0	0	0	0	0	0	0	0	0	0	0	0	0	0

0	0	0	0	0	0	0	0	0	0	0	0	0	0	0	0	0	0	0
0	0	0	0	0	0	0	0	0	0	0	0	0	0	0	0	0	0	0
0	0	0	0	0	0	0	0	0	0	0	0	0	0	0	0	0	0	0
0	0	0	0	0	0	0	0	0	0	0	0	0	0	0	0	0	0	0
0	0	0	0	0	0	0	0	0	0	0	0	0	0	0	0	0	0	0
0	0	0	0	0	0	0	0	0	0	0	0	0	0	0	0	0	0	0
0	0	0	0	0	0	0	0	0	0	0	0	0	0	0	0	0	0	0
0	0	0	0	0	0	0	0	0	0	0	0	0	0	0	0	0	0	0
0	0	0	0	0	0	0	0	0	0	0	0	0	0	0	0	0	0	0
0	0	0	0	0	0	0	0	0	0	0	0	0	0	0	0	0	0	0
0	0	0	0	0	0	0	0	0	0	0	0	0	0	0	0	0	0	0
0	0	0	0	0	0	0	0	0	0	0	0	0	0	0	0	0	0	0
0	0	0	0	0	0	0	0	0	0	0	0	2	2	2	2	2	2	2
0	0	0	0	0	0	0	0	0	0	0	0	0	0	0	0	0	0	0
0	0	0	0	0	0	0	0	0	0	0	0	0	0	0	0	0	0	0
0	0	0	0	0	0	0	0	0	0	0	0	0	0	0	0	0	0	0
0	0	0	0	0	0	0	0	0	0	0	0	0	0	0	0	0	0	0
0	0	0	0	0	0	0	0	0	0	0	0	0	0	0	0	0	0	0
0	0	0	0	0	0	0	0	0	0	0	0	0	0	0	0	0	0	0
0	0	0	0	0	0	0	0	0	0	0	0	0	0	0	0	0	0	0
0	0	0	0	0	0	0	0	0	0	0	0	0	0	0	0	0	0	0
0	0	0	0	0	0	0	0	0	0	0	0	0	0	0	0	0	0	0
0	0	0	0	0	0	0	0	0	0	0	0	0	0	0	0	0	0	0
0	0	0	0	0	0	0	0	0	0	0	0	0	0	0	0	0	0	0
0	0	0	0	0	0	0	0	0	0	0	0	0	0	0	0	0	0	0
0	0	0	0	0	0	0	0	0	0	0	0	0	0	0	0	0	0	0
0	0	0	0	0	0	0	0	0	0	0	0	0	0	0	0	0	0	0
0	0	0	0	0	0	0	0	0	0	0	0	0	0	0	0	0	0	0
0	0	0	0	0	0	0	0	0	0	0	0	0	0	0	0	0	0	0
0	0	0	0	0	0	0	0	0	0	0	0	0	0	0	0	0	0	0
0	0	0	0	0	0	0	0	0	0	0	0	0	0	0	0	0	0	0
0	0	0	0	0	0	0	0	0	0	0	0	0	0	0	0	0	0	0
0	0	0	0	0	0	0	0	0	0	0	0	0	0	0	0	0	0	0
0	0	0	0	0	0	0	0	0	0	0	0	0	0	0	0	0	0	0
0	0	0	0	0	0	0	0	0	0	0	0	0	0	0	0	0	0	0
0	0	0	0	0	0	0	0	0	0	0	0	0	0	0	0	0	0	0
0	0	0	0	0	0	0	0	0	0	0	0	0	0	0	0	0	0	0

0	0	0	0	0	0	0	0	0	0	0	0	0	0	0	0	0	0	0
0	0	0	0	0	0	0	0	0	0	0	0	0	0	0	0	0	0	0
0	0	0	0	0	0	0	0	0	0	0	0	0	0	0	0	0	0	0
0	0	0	0	0	0	0	0	0	0	0	0	0	0	0	0	0	0	0
0	0	0	0	0	0	0	0	0	0	0	0	0	0	0	0	0	0	0
0	0	0	0	0	0	0	0	0	0	0	0	0	0	0	0	0	0	0
0	0	0	0	0	0	0	0	0	0	0	0	0	0	0	0	0	0	0
0	0	0	0	0	0	0	0	0	0	0	0	0	0	0	0	0	0	0
0	0	0	0	0	0	0	0	0	0	0	0	0	0	0	0	0	0	0
0	0	0	0	0	0	0	0	0	0	0	0	0	0	0	0	0	0	0
0	0	0	0	0	0	0	0	0	0	0	0	0	0	0	0	0	0	0
0	0	0	0	0	0	0	0	0	0	0	0	0	0	0	0	0	0	0
0	0	0	0	0	0	0	0	0	0	0	0	0	0	0	0	0	0	0
0	0	0	0	0	0	0	0	0	0	0	0	0	0	0	0	0	0	0
0	0	0	0	0	0	0	0	0	0	0	0	0	0	0	0	0	0	0
0	0	0	0	0	0	0	0	0	0	0	0	0	0	0	0	0	0	0
0	0	0	0	0	0	0	0	0	0	0	0	0	0	0	0	0	0	0
0	0	0	0	0	0	0	0	0	0	0	0	0	0	0	0	0	0	0
0	0	0	0	0	0	0	0	0	0	0	0	0	0	0	0	0	0	0
0	0	0	0	0	0	0	0	0	0	0	0	0	0	0	0	0	0	0
0	0	0	0	0	0	0	0	0	0	0	0	0	0	0	0	0	0	0
0	0	0	0	0	0	0	0	0	0	0	0	0	0	0	0	0	0	0
0	0	0	0	0	0	0	0	0	0	0	0	0	0	0	0	0	0	0
0	0	0	0	0	0	0	0	0	0	0	0	0	0	0	0	0	0	0
0	0	0	0	0	0	0	0	0	0	0	0	0	0	0	0	0	0	0
0	0	0	0	0	0	0	0	0	0	0	0	0	0	0	0	0	0	0
0	0	0	0	0	0	0	0	0	0	0	0	0	0	0	0	0	0	0
0	0	0	0	0	0	0	0	0	0	0	0	0	0	0	0	0	0	0
0	0	0	0	0	0	0	0	0	0									
Jan_DL085	205	908	228	310	58	139	53	47	0	194	468	21	161	154	96	151	128	129
69	0	207	0	151	136	126	61	117	163	104	162	85	39	75	217	35	22	170
0	34	0	36	42	131	0	5	44	72	0	181	0	64	0	155	0	0	102
103	0	137	10	17	0	60	28	46	17	46	0	0	61	0	0	0	22	0
0	143	0	34	0	0	13	0	0	18	0	50	12	0	21	0	0	26	0
0	7	0	0	0	0	0	0	0	0	54	45	0	0	0	0	0	0	0
0	0	0	0	6	21	0	0	0	0	8	0	0	10	0	0	0	0	0
0	17	0	8	0	0	0	0	0	0	0	0	8	66	33	18	0	0	0

0	15	0	0	0	0	0	0	4	0	0	0	9	0	0	0	25	0	0
0	0	0	0	0	0	0	0	0	0	0	21	0	0	0	0	0	0	0
0	0	0	0	0	0	0	0	0	0	0	0	0	0	0	0	0	0	0
0	0	21	0	0	0	0	0	0	13	0	0	0	0	0	0	15	0	0
0	0	0	0	0	0	0	0	0	0	0	0	0	0	0	0	8	0	0
0	0	0	0	0	0	0	9	0	7	0	0	0	0	0	3	0	14	0
0	0	0	0	0	0	0	0	0	0	0	2	0	0	0	0	0	0	0
0	0	0	0	0	0	0	0	3	0	0	0	0	0	0	0	0	0	0
0	0	0	0	0	0	0	0	0	0	0	0	0	0	0	0	0	0	0
0	0	0	0	0	0	14	0	0	0	0	0	0	0	0	0	0	0	0
0	0	0	23	0	0	0	15	0	0	0	0	0	0	0	0	0	0	0
21	0	0	0	0	0	3	0	0	0	0	0	0	0	0	0	0	0	2
0	0	0	0	0	0	0	21	0	0	0	0	0	0	0	8	0	0	0
0	0	0	0	0	0	0	0	27	0	0	0	0	0	0	0	0	0	0
0	0	0	0	0	0	0	0	0	0	0	0	0	0	0	0	0	0	0
0	0	0	0	0	0	0	0	0	0	0	0	0	0	0	0	0	0	0
3	4	0	0	0	0	0	0	0	0	0	0	0	0	0	0	0	0	0
0	0	0	0	0	0	0	0	0	0	0	0	0	0	0	0	0	0	0
0	0	0	0	0	0	0	0	0	0	0	0	0	0	0	0	0	0	0
10	0	0	0	0	0	0	0	0	0	0	0	0	0	0	0	0	0	0
0	0	0	0	0	0	0	0	0	0	0	0	0	0	0	0	0	0	0
0	0	0	0	0	0	0	0	0	0	0	0	0	0	0	0	0	0	0
0	0	0	0	0	0	0	0	0	0	0	0	0	0	0	0	0	0	0
0	0	0	0	0	0	0	0	0	0	0	0	0	0	0	0	0	0	0
0	0	0	0	0	0	0	0	0	0	0	0	0	0	0	0	0	0	0
0	0	0	0	0	0	0	27	0	0	0	0	0	0	0	0	0	0	0
0	0	0	0	0	0	0	0	0	0	0	0	0	0	0	0	0	0	0
0	0	0	0	0	0	0	0	0	0	0	0	0	0	0	0	0	0	0
0	0	0	0	0	0	0	0	0	0	0	0	0	0	0	0	0	0	0
0	0	0	0	0	0	0	0	0	0	0	0	0	0	0	0	0	0	0
0	0	0	0	0	0	0	0	0	0	0	0	0	0	0	0	0	0	0
0	0	0	0	0	0	0	0	0	0	0	0	0	0	0	0	0	0	0
0	0	0	0	0	0	0	0	0	0	0	0	0	0	0	0	0	0	0
0	0	0	0	0	0	0	0	0	0	0	0	0	0	0	0	0	0	0
0	0	0	0	0	0	0	0	0	0	0	0	0	0	0	0	0	0	0
0	0	0	0	0	0	0	0	0	0	0	0	0	0	0	0	0	0	0
0	0	0	0	0	0	0	0	0	0	0	0	0	0	0	0	0	0	0

0	0	0	0	0	0	0	0	0	0	0	0	0	0	0	0	0	0	0
0	0	0	0	0	0	0	0	0	0	0	0	0	0	0	0	0	0	0
0	0	0	0	0	0	0	0	0	0	0	0	0	4	0	0	0	0	0
0	0	0	0	0	0	0	0	0	0	0	0	0	0	0	0	0	0	0
0	0	0	0	0	0	0	0	0	0	0	0	0	0	0	0	0	0	0
0	0	0	0	0	0	0	0	0	0	0	0	0	0	0	0	0	0	0
0	0	0	0	0	0	0	0	0	0	0	0	0	0	0	0	0	0	0
0	0	0	0	0	0	0	0	0	0	0	0	0	0	0	0	0	0	0
0	0	0	0	0	0	0	0	0	0	0	0	0	0	0	0	0	0	0
0	0	0	0	0	0	0	0	0	0	0	0	0	0	0	0	0	0	0
0	0	0	0	0	0	0	0	0	0	0	0	0	0	0	0	0	0	0
0	0	0	0	0	0	0	0	0	0	0	0	0	0	0	0	0	0	0
0	0	0	0	0	0	0	0	0	0	0	0	0	0	0	0	0	2	0
0	0	0	0	0	0	0	0	0	0	0	0	0	0	0	0	0	0	0
0	0	0	0	0	0	0	0	0	0	0	0	0	0	10	0	0	0	0
0	0	0	0	0	0	0	0	0	0	0	0	0	0	0	0	0	0	0
0	0	0	0	0	0	0	0	0	0	0	0	0	0	0	0	0	0	0
0	0	0	0	0	0	0	0	0	0	0	0	0	0	0	0	0	0	0
0	0	0	0	0	0	0	0	0	0	0	0	0	0	0	0	0	0	0
0	0	0	0	0	0	0	0	0	0	0	0	0	0	0	0	0	0	0
0	0	0	0	0	0	0	0	0	0	0	0	0	0	0	0	0	0	0
0	0	0	0	0	0	0	0	0	0	0	0	0	0	0	0	0	0	0
0	0	0	0	0	0	0	0	0	0	0	0	0	0	0	0	0	0	0
0	0	0	0	0	0	0	0	0	0	0	0	0	0	0	0	0	0	0
0	0	0	0	0	0	0	0	0	0	0	0	0	0	0	0	0	0	0
0	0	0	0	0	0	0	0	0	0	0	0	0	0	0	0	0	0	6
6	6	0	0	0	0	0	0	0	0	0	0	0	0	0	0	0	0	0
0	0	0	0	0	0	0	0	0	0	0	0	0	0	0	0	0	0	0
0	0	0	0	0	0	0	0	0	0	0	0	0	0	0	0	0	0	0
0	0	0	0	0	0	0	0	0	0	0	0	0	0	0	0	0	0	0
0	0	0	0	0	0	0	0	0	0	0	0	0	0	0	0	0	0	0
0	0	0	0	0	0	0	0	0	0	0	0	0	0	0	0	0	0	0
0	0	0	0	0	0	0	0	0	0	0	0	0	0	0	0	0	0	0
0	0	0	0	0	0	0	0	0	0	0	0	0	0	0	0	0	0	0
0	0	0	0	0	0	0	0	0	0	0	0	0	0	0	0	0	0	0
0	0	0	0	0	0	0	0	0	0	0	0	0	0	0	0	0	0	0
0	0	0	0	0	0	0	0	0	0	0	0	0	0	0	0	0	0	0

0	0	0	0	0	0	0	0	0	0	0	0	0	0	0	0	0	0	0
0	0	0	0	0	0	0	0	0	0	0	0	0	0	0	0	0	0	0
0	0	0	0	0	0	0	0	0	0	0	0	0	0	0	0	0	0	0
0	0	0	0	0	0	0	0	0	0	0	0	0	0	0	0	0	0	0
0	0	0	0	0	0	0	0	0	0	0	0	0	0	0	0	0	0	0
0	0	0	0	0	0	0	0	0	0	0	0	0	0	0	0	0	0	0
0	0	0	0	0	0	0	0	0	0	0	0	0	0	0	0	0	0	0
0	0	0	0	0	0	0	0	0	0	0	0	0	0	0	0	0	0	0
0	0	0	0	0	0	0	0	0	0	0	0	0	0	0	0	0	0	0
0	0	0	0	0	0	0	0	0	0	0	0	0	0	0	0	0	0	0
0	0	0	0	0	0	0	0	0	0	0	0	0	0	0	0	0	0	0
0	0	0	0	0	0	0	0	3	3	0	0	0	0	0	0	0	0	0
0	0	0	0	0	0	0	0	0	0	0	0	0	0	0	0	0	0	0
0	0	0	0	0	0	0	0	0	0	0	0	0	0	0	0	0	0	0
0	0	0	0	0	0	0	0	0	0	0	0	0	0	0	0	0	0	0
0	0	0	0	0	0	0	0	0	0	0	0	0	0	0	0	0	0	0
0	0	0	0	0	0	0	0	0	0	0	0	0	0	0	0	0	0	0
0	0	0	0	0	0	0	0	0	0	0	0	0	0	0	0	0	0	0
0	0	0	0	0	0	0	0	0	0	0	0	0	0	0	0	0	0	0
0	0	0	0	0	0	0	0	0	0	0	0	0	0	0	0	0	0	0
0	0	0	0	0	0	0	0	0	0	0	0	0	0	0	0	0	0	0
0	0	0	0	0	0	0	0	0	0	0	0	0	0	0	0	0	0	0
0	0	0	0	0	0	0	0	0	0	0	0	0	0	0	0	0	0	0
0	0	0	0	0	0	0	0	0	0	0	0	0	0	0	0	0	0	0
0	0	0	0	0	0	0	0	0	0	0	0	0	0	0	0	0	0	0
0	0	0	0	0	0	0	0	0	0	0	0	0	0	0	0	0	0	0
0	0	0	0	0	0	0	0	0	0	0	0	0	0	0	0	0	0	0
0	0	0	0	0	0	0	0	0	0	0	0	0	0	0	0	0	0	0
0	0	0	0	0	0	0	0	0	0	0	0	0	0	0	0	0	0	0
0	0	0	0	0	0	0	0	0	0	0	0	0	0	0	0	0	0	0
0	0	2	0	0	0	0	0	0	0	0	0	0	0	0	0	0	0	0
0	0	0	0	0	0	0	0	0	0	0	0	0	0	0	0	0	0	0
0	0	0	0	0	0	0	0	0	0	0	0	0	0	0	0	0	0	0
0	0	0	0	0	0	0	0	0	0	0	0	0	0	0	0	0	0	0
0	0	0	0	0	0	0	0	0	0	0	0	0	0	0	0	0	0	0
0	0	0	0	0	0	0	0	0	0	0	0	0	0	0	0	0	0	0
0	0	0	0	0	0	0	0	0	0	0	0	0	0	0	0	0	0	0

0	0	0	0	0	0	0	0	0	0	0	0	0	0	0	0	0	0	0
0	0	0	0	0	0	0	0	0	0	0	0	0	0	0	0	0	0	0
0	0	0	0	0	0	0	0	0	0	0	0	0	0	0	0	0	0	0
0	0	0	0	0	0	0	0	0	0	0	0	0	0	0	0	0	0	0
0	0	0	0	0	0	0	0	0	0	0	0	0	0	0	0	0	0	0
0	0	0	0	0	0	0	0	0	0	0	0	0	0	0	0	0	0	0
0	0	0	0	0	0	0	0	0	0	0	0	0	0	0	0	0	0	0
0	0	0	0	0	0	0	0	0	0	0	0	0	0	0	0	0	0	0
0	0	0	0	0	0	0	0	0	0	0	0	0	0	0	0	0	0	0
0	0	0	0	0	0	0	0	0	0	0	0	0	0	0	0	0	0	0
0	0	0	0	0	0	0	0	0	0	0	0	0	0	0	0	0	0	0
0	0	0	0	0	0	0	0	0	0	0	0	0	0	0	0	0	0	0
0	0	0	0	0	0	0	0	0	0	0	0	0	0	0	0	0	0	0
0	0	0	0	0	0	0	0	0	0	0	0	0	0	0	0	0	0	0
0	0	0	0	0	0	0	0	0	0	0	0	0	0	0	0	0	0	0
0	0	0	0	0	0	0	0	0	0	0	0	0	0	0	0	0	0	0
0	0	0	0	0	0	0	0	0	0	0	0	0	0	0	0	0	0	0
0	0	0	0	0	0	0	0	0	0	0	0	0	0	0	0	0	0	0
0	0	0	0	0	0	0	0	0	0	0	0	0	0	0	0	0	0	0
0	0	0	0	0	0	0	0	0	0	0	0	0	0	0	0	0	0	0
0	0	0	0	0	0	0	0	0	0	0	0	0	0	0	0	0	0	0
0	0	0	0	0	0	0	0	0	0	0	0	0	0	0	0	0	0	0
0	0	0	0	0	0	0	0	0	0	0	0	0	0	0	0	0	0	0
0	0	0	0	0	0	0	0	0	0	0	0	0	0	0	0	0	0	0
0	0	0	0	0	0	0	0	0	0	0	0	0	0	0	0	0	0	0
0	0	0	0	0	0	0	0	0	0	0	0	0	0	0	0	0	0	0
0	0	0	0	0	0	0	0	0	0	0	0	0	0	0	0	0	0	0
0	0	0	0	0	0	0	0	0	0	0	0	0	0	0	0	0	0	0
0	0	0	0	0	0	0	0	0	0	0	0	0	0	0	0	0	0	0
0	0	0	0	0	0	0	0	0	0	0	0	0	0	0	0	0	0	0
0	0	0	0	0	0	0	0	0	0	0	0	0	0	0	0	0	0	0
0	0	0	0	0	0	0	0	0	0	0	0	0	0	0	0	0	0	0
0	0	0	0	0	0	0	0	0	0	0	0	0	0	0	0	0	0	0
0	0	0	0	0	0	0	0	0	0	0	0	0	0	0	0	0	0	0
0	0	0	0	0	0	0	0	0	0	0	0	0	0	0	0	0	0	0
0	0	0	0	0	0	0	0	0	0	0	0	0	0	0	0	0	0	0
0	0	0	0	0	0	0	0	0	0	0	0	0	0	0	0	0	0	0

0	0	0	0	0	1	0	0	0	0	0	0	0	0	0	0	0	0	0
0	0	0	0	0	0	0	0	0	0	0	0	0	0	0	0	0	0	0
0	0	0	0	0	0	0	0	0	0	0	0	0	0	0	0	0	0	0
0	0	0	0	0	0	0	0	0	0	0	0	0	0	0	0	0	0	0
0	0	0	0	0	0	0	0	0	0	0	0	0	0	0	0	0	0	0
0	0	0	0	0	0	0	0	0	0	0	0	0	0	0	0	0	0	0
0	0	0	0	0	0	0	0	0	0	0	0	0	0	0	0	0	0	0
0	0	0	0	0	0	0	0	0	0	0	0	0	0	0	0	0	0	0
0	0	0	0	0	0	0	0	0	0	0	0	0	0	0	0	0	0	0
0	0	0	0	0	0	0	0	0	0	0	0	0	0	0	0	0	0	0
0	0	0	0	0	0	0	0	0	0	0	0	0	0	0	0	0	0	0
0	0	0	0	0	0	0	0	0	0	0	0	0	0	0	0	0	0	0
0	0	0	0	0	0	0	0	0	0	0	0	0	0	0	0	0	0	0
0	0	0	0	0	0	0	0	0	0	0	0	0	0	0	0	0	0	0
0	0	0	0	0	0	0	0	0	0	0	0	0	0	0	0	0	0	0
0	0	0	0	0	0	0	0	0	0	0	0	0	0	0	0	0	0	0
0	0	0	0	0	0	0	0	0	0	0	0	0	0	0	0	0	0	0
0	0	0	0	0	0	0	0	0	0	0	0	0	0	0	0	0	0	0
0	0	0	0	0	0	0	0	0	0	0	0	0	0	0	0	0	0	0
0	0	0	0	0	0	0	0	0	0	0	0	0	0	0	0	0	0	0
0	0	0	0	0	0	0	0	0	0									
Jan_DL086	1058	1344	985	304	41	793	144	115	355	299	78	56	238	38	209	240	44	137
247	238	220	181	124	188	186	155	163	75	70	0	83	128	134	0	85	30	0
50	0	43	72	93	0	106	0	67	70	55	0	40	98	45	0	0	74	0
0	104	0	5	49	54	44	58	13	29	37	148	103	0	30	21	9	30	0
28	0	34	35	0	29	10	46	53	19	0	0	34	90	26	0	19	0	0
0	18	0	66	0	0	51	58	0	18	0	38	0	31	24	15	0	0	0
51	0	0	7	5	18	0	0	0	0	10	0	6	0	0	0	0	0	0
0	0	0	0	0	0	0	41	0	0	17	0	0	0	0	0	0	0	0
0	4	0	0	0	0	0	0	0	0	5	0	0	0	33	0	0	0	0
27	0	0	0	0	47	0	0	0	0	0	15	0	0	0	0	21	0	0
0	0	0	42	0	0	0	0	0	12	0	0	0	0	0	0	0	0	0
0	0	11	0	0	0	0	0	0	0	0	0	0	0	0	0	0	0	0
0	0	0	0	0	8	0	0	7	12	0	11	5	3	0	0	12	0	0
0	0	0	3	17	0	0	0	0	0	0	0	0	0	0	0	0	10	0
9	0	0	22	0	0	0	0	0	0	0	21	0	0	5	0	0	0	0
0	0	0	0	0	0	0	0	0	0	0	12	0	0	10	0	0	0	0

0	3	0	0	0	14	0	0	0	0	0	11	0	0	0	0	0	0	0
0	0	0	6	4	0	0	0	0	0	0	0	0	0	0	0	0	0	0
6	0	0	0	0	0	0	0	0	0	0	0	3	0	0	0	0	0	13
0	0	0	0	0	0	0	5	0	0	0	0	0	0	0	0	0	0	0
0	0	0	0	0	4	0	0	0	0	0	0	0	0	0	0	0	0	0
0	0	0	0	0	0	0	0	0	0	0	0	0	0	0	14	0	0	0
0	0	0	0	0	0	0	0	0	0	0	0	6	0	0	0	0	0	0
0	0	0	0	0	0	0	0	0	0	0	0	0	0	0	0	0	0	0
2	0	0	0	0	0	0	0	0	0	0	0	0	0	0	0	0	6	0
0	0	0	6	0	0	0	0	0	0	0	0	0	0	0	0	0	0	4
4	0	0	0	0	0	0	0	0	0	0	0	0	0	0	0	0	5	0
0	0	0	0	0	0	0	0	0	0	0	0	0	0	0	0	0	0	0
0	0	0	0	0	0	0	0	0	0	0	0	11	0	0	0	0	0	0
0	0	0	0	0	0	0	0	0	23	0	0	0	0	0	0	0	0	0
0	0	0	0	0	0	0	0	0	0	0	0	0	3	0	0	0	0	0
8	0	0	0	0	0	0	0	0	0	0	0	0	0	0	0	0	0	0
0	0	4	0	0	0	0	0	0	0	0	0	0	0	0	0	0	0	0
0	0	0	0	0	0	0	0	0	0	0	0	0	0	0	0	0	0	0
0	0	0	0	0	0	0	0	0	0	0	0	3	0	0	0	0	0	0
0	0	0	0	0	0	0	0	0	0	0	0	0	0	0	0	0	0	0
13	0	0	0	0	0	0	0	28	0	0	0	0	0	0	0	0	0	0
23	0	0	0	0	0	0	0	0	0	0	0	0	0	7	0	0	0	0
0	0	0	0	0	0	0	0	0	0	0	8	0	0	0	0	0	0	0
0	0	0	0	0	0	0	0	0	0	0	0	0	0	0	0	0	0	0
0	0	0	0	0	0	0	0	0	0	0	0	0	0	0	0	0	0	0
0	0	0	0	0	0	0	0	0	0	0	0	27	0	0	0	0	0	0
0	0	0	0	0	0	0	0	0	0	0	0	0	0	0	0	0	0	0
0	0	0	0	0	0	0	0	0	0	0	0	0	0	6	0	0	0	0
0	0	0	0	0	0	0	0	0	0	0	0	0	0	0	0	0	0	0
0	0	0	0	0	0	0	0	0	0	0	0	0	0	0	0	0	0	0
0	0	0	0	0	0	0	0	0	0	0	0	0	0	0	0	0	0	0
0	0	0	0	0	0	0	0	0	0	0	0	0	0	0	0	0	0	0
0	0	0	0	0	0	0	0	0	0	0	0	0	0	0	0	0	0	0
0	0	0	0	0	0	0	0	0	0	0	0	0	0	0	0	0	0	0
0	0	0	0	0	0	0	0	0	0	0	0	0	0	0	0	0	0	0
0	0	0	0	0	0	0	0	0	0	0	0	0	0	0	0	0	0	0
0	0	0	0	0	0	0	0	0	0	0	0	0	0	0	0	0	0	0

0	0	0	0	0	0	0	0	0	0	0	0	0	0	0	0	0	0	0
0	0	0	0	0	0	0	0	0	0	0	0	0	0	0	0	0	0	0
0	0	0	0	0	0	0	0	0	0	0	0	0	0	0	0	0	0	0
0	0	0	0	0	0	0	0	0	0	0	0	0	0	0	0	0	0	0
0	0	0	0	0	0	0	0	0	0	0	0	0	0	0	0	0	0	0
0	0	0	3	0	0	0	0	0	0	0	0	0	0	0	0	0	0	0
0	0	0	0	0	0	0	0	0	0	0	0	0	0	0	0	0	0	0
0	0	0	0	0	0	0	0	0	0	0	0	0	0	0	0	0	0	0
0	0	0	0	0	0	0	0	0	0	0	0	0	0	0	0	0	0	0
0	0	0	0	0	0	0	0	0	0	0	0	0	0	0	0	0	0	0
0	0	0	0	0	0	0	0	0	0	0	0	0	0	0	0	0	0	0
0	0	0	0	0	0	12	0	0	0	0	0	0	0	0	0	0	0	0
0	0	0	0	0	0	0	0	0	0	0	0	0	0	0	0	0	0	0
0	0	0	0	0	0	0	0	0	0	0	0	0	0	0	5	0	0	0
0	0	0	0	0	0	0	0	0	0	0	0	0	0	0	0	0	0	0
0	0	0	0	0	0	0	0	0	0	0	0	0	0	0	0	0	0	0
0	0	0	0	0	0	0	0	0	0	0	0	0	0	0	0	0	0	0
0	0	0	0	0	0	0	0	0	0	0	0	0	0	0	0	0	0	0
0	0	0	0	0	0	0	0	0	0	0	0	0	0	0	0	0	0	0
0	0	0	0	0	0	0	0	0	0	0	0	0	0	0	0	0	0	0
0	0	0	0	0	0	0	0	0	0	0	0	0	0	0	0	0	0	8
0	0	0	0	0	0	0	0	0	0	0	0	0	0	0	0	0	0	0
0	0	0	0	0	0	0	0	0	0	0	0	0	0	0	0	0	0	0
0	0	0	0	0	0	0	0	0	0	0	0	0	0	0	0	0	4	0
0	0	0	0	0	0	0	0	0	0	0	0	0	0	0	0	0	0	0
0	0	0	0	0	0	0	0	0	0	0	0	0	0	0	0	0	0	0
0	0	0	0	0	0	0	0	0	0	0	0	0	0	0	0	0	0	0
0	0	0	0	0	0	0	0	0	0	0	0	0	0	0	0	0	0	0
0	0	0	0	0	0	0	0	0	0	0	0	0	0	0	0	0	0	0
0	0	0	0	0	0	0	0	0	0	0	0	0	0	0	0	0	0	0
0	0	0	0	0	0	0	0	0	0	0	0	0	0	0	0	0	0	0
0	0	0	0	0	0	0	0	0	0	0	0	0	0	0	0	0	0	0
0	0	0	0	0	0	0	0	0	0	0	0	0	0	0	0	0	0	0
0	0	0	0	0	0	0	0	0	0	0	0	0	0	0	0	0	0	0
0	0	0	0	0	0	0	0	0	0	0	0	0	0	0	0	0	0	0
0	0	0	0	0	0	0	0	0	0	0	0	0	0	0	0	0	0	0
0	0	0	0	0	0	0	0	0	0	0	0	0	0	0	0	0	0	0

0	0	0	0	0	0	0	0	0	0	0	5	0	0	0	0	0	0	0
0	0	0	0	0	0	0	0	0	0	0	0	0	0	0	0	0	0	0
0	0	0	0	0	0	0	0	0	0	0	0	0	0	0	0	0	0	0
0	0	0	0	0	0	0	0	0	0	0	0	0	0	0	0	0	0	0
0	0	0	0	0	0	0	0	0	0	0	0	0	0	0	0	0	0	0
0	0	0	0	0	0	0	0	0	0	0	0	0	0	0	0	0	0	0
0	0	0	0	0	0	0	0	0	0	0	0	0	0	0	0	0	0	0
0	0	0	0	0	0	0	0	0	0	0	0	0	0	0	0	0	0	0
0	0	0	0	0	0	0	0	0	0	0	0	0	0	0	0	0	0	0
0	0	0	0	0	0	0	0	0	0	0	0	0	0	0	0	0	0	0
0	0	0	0	0	0	0	0	0	0	0	0	0	0	0	0	0	0	0
0	0	0	0	0	0	0	0	0	0	0	0	0	0	0	0	0	0	0
0	0	0	0	0	0	0	0	0	0	0	0	0	0	0	0	0	0	0
0	0	0	0	0	0	0	0	0	0	0	0	0	0	0	0	0	0	0
0	0	0	0	0	0	0	0	0	0	0	0	0	0	0	0	0	0	0
0	0	0	0	0	0	0	0	0	0	0	0	0	0	0	0	0	0	0
0	0	0	0	0	0	0	0	0	0	0	0	0	0	0	0	0	0	0
0	0	0	0	0	0	0	0	0	0	0	0	0	0	0	0	0	0	0
0	0	0	0	0	0	0	0	0	0	0	0	0	0	0	0	0	0	0
0	0	0	0	0	0	0	0	0	0	0	0	0	0	0	0	0	0	0
0	0	0	0	0	0	0	0	0	0	0	0	0	0	0	0	0	0	0
0	0	0	0	0	0	0	0	0	0	0	0	0	0	0	0	0	0	0
0	0	0	0	0	0	0	0	0	0	0	0	0	0	0	0	0	0	0
0	0	0	0	0	0	0	0	0	3	0	0	0	0	0	0	0	0	0
0	0	0	0	0	0	0	0	0	0	0	0	0	0	0	0	0	0	0
0	0	0	0	0	0	0	0	0	0	0	0	0	0	0	0	0	0	0
0	0	0	0	0	0	0	0	0	0	0	0	0	0	0	0	0	0	0
0	0	0	0	0	0	0	0	0	0	0	0	0	0	0	0	0	0	0
0	0	0	0	0	0	0	0	0	0	0	0	0	0	0	0	0	0	0
0	0	0	0	0	0	0	0	0	0	0	0	0	0	0	0	0	0	0
0	0	0	0	0	0	0	0	0	0	0	0	0	0	0	0	0	0	0
0	0	0	0	0	0	0	0	0	0	0	0	0	0	0	0	0	0	0
0	0	0	0	0	0	0	0	0	0	0	0	0	0	0	0	0	0	0
0	0	0	0	0	0	0	0	0	0	0	0	0	0	0	0	0	0	0
0	0	0	0	0	0	0	0	0	0	0	0	0	0	0	0	0	0	0
0	0	0	0	0	0	0	0	0	0	0	0	0	0	0	0	0	0	0
0	0	0	0	0	0	0	0	0	0	0	0	0	0	0	0	0	0	0

0	0	0	0	0	0	0	0	0	0	0	0	0	0	0	0	0	0	0
0	0	0	0	0	0	0	0	0	0	0	0	0	0	0	0	0	0	0
0	0	0	0	0	0	0	0	0	0	0	0	0	0	0	0	0	0	0
0	0	0	0	0	0	0	0	0	0	0	0	0	0	0	0	0	0	0
0	0	0	0	0	0	0	0	0	0	0	0	0	0	0	0	0	0	0
0	0	0	0	0	0	0	0	0	0	0	0	0	0	0	0	0	0	0
0	0	0	0	0	0	0	0	0	0	0	0	0	0	0	0	0	0	0
0	0	0	0	0	0	0	0	0	0	0	0	0	0	0	0	0	0	0
0	0	0	0	0	0	0	0	0	0	0	0	0	0	0	2	2	2	2
2	2	2	2	0	0	0	0	0	0	0	0	0	0	0	0	0	0	0
0	0	0	0	0	0	0	0	0	0	0	0	0	0	0	0	0	0	0
0	0	0	0	0	0	0	0	0	0	0	0	0	0	0	0	0	0	0
0	0	0	0	0	0	0	0	0	0	0	0	0	0	0	0	0	0	0
0	0	0	0	0	0	0	0	0	0	0	0	0	0	0	0	0	0	0
0	0	0	0	0	0	0	0	0	0	0	0	0	0	0	0	0	0	0
0	0	0	0	0	0	0	0	0	0	0	0	0	0	0	0	0	0	0
0	0	0	0	0	0	0	0	0	0	0	0	0	0	0	0	0	0	0
0	0	0	0	0	0	0	0	0	0	0	0	0	0	0	0	0	0	0
0	0	0	0	0	0	0	0	0	0	0	0	0	0	0	0	0	0	0
0	0	0	0	0	0	0	0	0	0	0	0	0	0	0	0	0	0	0
0	0	0	0	0	0	0	0	0	0	0	0	0	0	0	0	0	0	0
0	0	0	0	0	0	0	0	0	0	0	0	0	0	0	0	0	0	0
0	0	0	0	0	0	0	0	0	0	0	0	0	0	0	0	0	0	0
0	0	0	0	0	0	0	0	0	0	0	0	0	0	0	0	0	0	0
0	0	0	0	0	0	0	0	0	0	0	0	0	0	0	0	0	0	0
0	0	0	0	0	0	0	0	0	0	0	0	0	0	0	0	0	0	0
0	0	0	0	0	0	0	0	0	0	0	0	0	0	0	0	0	0	0
0	0	0	0	0	0	0	0	0	0	0	0	0	0	0	0	0	0	0
0	0	0	0	0	0	0	0	0	0	0	0	0	0	0	0	0	0	0
0	0	0	0	0	0	0	0	0	0	0	0	0	0	0	0	0	0	0
0	0	0	0	0	0	0	0	0	0	0	0	0	0	0	0	0	0	0
0	0	0	0	0	0	0	0	0	0	0	0	0	0	0	0	0	0	0
0	0	0	0	0	0	0	0	0	0	0	0	0	0	0	0	0	0	0
0	0	0	0	0	0	0	0	0	0	0	0	0	0	0	0	0	0	0
0	0	0	0	0	0	0	0	0	0	0	0	0	0	0	0	0	0	0
0	0	0	0	0	0	0	0	0	0	0	0	0	0	0	0	0	0	0
0	0	0	0	0	0	0	0	0	0	0	0	0	0	0	0	0	0	0

0	0	0	0	0	0	0	0	0	0	0	0	0	0	0	0	0	0	0
0	0	0	0	0	0	0	0	0	0	0	0	0	0	0	0	0	0	0
0	0	0	0	0	0	0	0	0	0	0	0	0	0	0	0	0	0	0
0	0	0	0	0	0	0	0	0	0	0	0	0	0	0	0	0	0	0
0	0	0	0	0	0	0	0	0	0	0	0	0	0	0	0	0	0	0
0	0	0	0	0	0	0	0	0	0	0	0	0	0	0	0	0	0	0
0	0	0	0	0	0	0	0	0	0	0	0	0	0	0	0	0	0	0
0	0	0	0	0	0	0	0	0	0	0	0	0	0	0	0	0	0	0
0	0	0	0	0	0	0	0	0	0	0	0	0	0	0	0	0	0	0
0	0	0	0	0	0	0	0	0	0	0	0	0	0	0	0	0	0	0
0	0	0	0	0	0	0	0	0	0	0	0	0	0	0	0	0	0	0
0	0	0	0	0	0	0	0	0	0	0	0	0	0	0	0	0	0	0
0	0	0	0	0	0	0	0	0	0									
Jan_DL087	658	1366	67	390	1058	152	246	284	0	122	74	17	134	48	201	13	39	131
34	0	239	678	103	219	21	49	0	0	52	0	23	16	64	0	28	2	0
0	168	0	0	49	0	41	0	27	0	0	0	57	0	0	0	0	0	0
0	0	21	53	0	29	50	0	0	22	106	0	0	52	4	15	24	9	181
0	0	0	0	43	0	0	0	0	0	0	0	47	0	14	0	6	53	0
0	0	0	0	0	9	0	74	0	8	0	0	54	45	0	20	0	0	0
0	0	91	7	5	0	0	0	0	0	40	93	18	14	0	11	0	0	0
0	0	0	31	0	0	43	0	0	0	44	0	19	0	21	52	0	0	0
0	67	0	0	0	16	0	0	0	0	27	0	0	0	0	19	23	24	21
0	0	0	0	27	0	0	0	0	0	0	0	0	0	0	0	0	33	27
0	0	62	0	0	0	0	33	0	0	0	54	23	0	0	0	0	42	0
0	0	5	0	0	74	0	0	0	0	0	43	30	23	0	0	0	0	0
0	0	30	0	0	11	0	0	11	0	0	0	0	0	0	0	0	0	0
18	0	0	6	27	0	0	39	0	6	0	0	0	11	9	0	0	0	0
0	0	0	29	0	0	0	0	0	0	19	0	0	14	18	9	28	0	0
0	0	38	0	0	0	0	0	0	0	0	0	0	0	0	0	0	39	0
0	36	0	0	0	0	0	0	0	0	0	0	12	0	0	0	0	0	0
0	0	0	0	0	24	0	0	0	0	0	0	0	0	0	0	0	0	0
0	0	0	0	0	9	0	0	12	0	42	0	0	0	0	0	23	0	11
0	0	14	0	12	0	0	0	0	0	0	0	0	0	0	0	0	0	0
0	0	0	0	0	0	0	2	3	0	12	0	0	0	0	0	0	46	0
0	0	0	0	0	0	0	0	0	3	0	0	0	0	0	0	0	0	0
0	0	9	0	0	0	0	0	0	0	0	0	0	0	0	0	0	0	0
0	0	0	7	0	0	0	0	0	0	0	0	0	0	0	0	5	0	0

0	9	0	10	0	0	0	0	0	0	0	0	0	0	0	0	0	0	23
0	0	0	0	0	0	0	0	0	0	0	0	0	0	0	3	0	0	0
0	0	0	0	0	12	30	0	8	0	0	0	0	0	0	0	0	0	0
0	0	0	0	0	0	0	0	0	0	0	0	0	0	0	0	0	0	0
0	0	0	0	0	0	0	0	0	4	0	0	0	0	0	0	0	0	0
0	0	0	0	0	0	0	0	0	0	4	0	0	0	0	0	0	0	0
[truncated: 870,623 more chars]
